# High trophic niche overlap between sympatric peacock basses (Cichliformes: Cichlidae: *Cichla*): Concordant findings from three dietary analysis methods

**DOI:** 10.1111/jfb.70485

**Published:** 2026-05-12

**Authors:** Benton L. Fry, Hayden C. Roberts, Kirk O. Winemiller

**Affiliations:** ^1^ Ecology and Conservation Biology Texas A&M University College Station Texas USA; ^2^ Ecology and Evolutionary Biology Texas A&M University College Station Texas USA

**Keywords:** cryptic species, gut contents, metabarcoding, Neotropics, stable isotope, trophic ecology

## Abstract

Dietary analysis has contributed to our understanding of animal niches, interspecific interactions, community structure and the flow of matter and energy in food webs. We employed three methods of dietary analysis to estimate trophic niche overlap between two peacock bass species, *Cichla cataractae* and *Cichla ocellaris*, across sympatric (Essequibo River) and allopatric (Rupununi and Rewa Rivers) populations in Guyana – visual gut contents analysis, gut contents metabarcoding and stable isotope (δ^13^C, δ^15^N) analysis. During the dry season, both piscivorous species exhibited generalist feeding strategies and little evidence of trophic niche segregation with respect to prey taxonomic diversity. Metabarcoding identified 92 prey taxa spanning 28 genera, with Characiformes, Siluriformes and Cichliformes dominant in diets of all populations. Visual gut contents analyses corresponded with metabarcoding results but revealed *C. cataractae* consumed significantly larger prey than *C. ocellaris*, consistent with the larger mouth gape of *C. cataractae*. Stable isotope analysis indicated *C. cataractae* had higher nitrogen ratios than *C. ocellaris*, and the two species overlapped broadly with respect to carbon ratios. Overall, there was broad isotopic overlap between species and between allopatric and sympatric populations of *C. ocellaris* (≥ 70%). These findings suggest food resource partitioning with respect to taxonomic diversity is relatively unimportant for coexistence of these cryptic species, and prey size and habitat selection are the principal mechanisms of niche partitioning during the annual low‐water season.

## 1 INTRODUCTION

The simple question, *‘What do animals eat?’* has been the foundation of ecological research addressing food web structure, energy and nutrient dynamics, niche relationships and invasion ecology (McCann, 2007). Historically, diet studies relied on direct foraging observations or examination of gut contents. These methods are time‐consuming, require substantial taxonomic expertise and often produce relatively coarse dietary resolution (Horswill et al., 2018; Matthews et al., 2019). In aquatic systems, direct foraging observations are often impractical, and examination of gut contents often is the most feasible option. However, analysis of gut contents is only effective when material is examined prior to its degradation by digestion (Kytinou et al., 2020). In fishes, the frequent occurrence of empty stomachs, particularly among piscivores, requires collection and dissection of large numbers of individuals to obtain a robust sample (Winemiller, 1990). Given these limitations, alternative methods (e.g., non‐lethal procedures) should be considered.

Metabarcoding of gut contents provides an alternative approach for dietary analysis, with potential to improve taxonomic resolution compared to traditional techniques. Using this method, sequences of short hypervariable DNA regions (barcodes) from gut contents or faeces are compared to reference databases for taxonomic identification (Carreon‐Martinez et al., 2011). Because prey DNA can be detected even from small or highly digested samples, non‐lethal methods such as gastric lavage or faecal analysis can be employed (Deagle et al., 2019; Hoenig et al., 2020). This reduces the need for large samples and allows for the investigation of contents that may be morphologically unidentifiable. However, similar to visual gut contents analysis, metabarcoding provides only a snapshot of food ingested over a relatively brief time interval and generally yields occurrence data without information on the size, volume, or energetic/nutritional contribution of food items (Littleford‐Colquhoun et al., 2022).

Stable isotope analysis provides a complementary approach for diet analyses by estimating contributions of foods or basal production sources assimilated over longer time periods, from weeks to months depending on tissue type (O'Reilly et al., 2004). Comparison of isotopic ratios of carbon (δ^13^C) and nitrogen (δ^15^N) in consumer tissues and potential food items or basal resources are used to infer material and energy pathways in food webs (Boecklen et al., 2011; Layman et al., 2012). When potential dietary sources have sufficiently distinct isotopic ratios, their contributions to consumer biomass can be estimated reasonably (Hopkins III & Ferguson, 2012). However, such conditions do not occur in many systems, thereby limiting the precision of diet estimates using stable isotopes alone (Fry, 2007; Phillips et al., 2014). Moreover, diet estimation from stable isotope analysis often is affected by uncertainty regarding trophic discrimination factors (i.e., shifts in an element's isotopic ratio between source and consumer tissues) and variation in assimilation dynamics from different dietary sources (Stephens et al., 2023). Despite this, consistent enrichment of ^15^N has been shown to be a reliable indicator of the relative vertical trophic positions of consumers within food webs (Post, 2002; Stephens et al., 2023).

Given trade‐offs in temporal and taxonomic resolution along with logistical concerns across diet methods, studies conducting multiple approaches can provide more robust and comprehensive surveys. Nielsen et al. (2018) advocated for the use of multiple methods to reduce uncertainty of diet estimates. Additionally, metabarcoding has been shown to increase taxonomic richness of diet estimates compared to standalone visual examination of recovered gut contents (Barnett et al., 2010). Therefore, the use of both methods on gut contents improves taxonomic resolution, whereas stable isotope analysis may reveal long‐term trends in energy source assimilation.

Peacock basses (*Cichla*) are piscivorous cichlids well studied in both their native South American and introduced ranges. The Butterfly Peacock Bass (*Cichla ocellaris*), in particular, has been widely introduced for recreational fisheries and control of invasive species. Peacock bass have been shown to exert strong top‐down control on fish populations, sometimes with severe impact (Catelani et al., 2021; de Araújo et al., 2022; Espínola et al., 2015; Franco et al., 2021; Zaret & Paine, 1973). Within native habitats of South America, peacock bass are common large piscivores, with seasonal hydrology playing a key role in their feeding ecology. During the dry season, falling water levels and contraction of aquatic habitats increase fish densities and predator–prey encounters (Arrington & Winemiller, 2002; Winemiller & Jepsen, 1998), driving elevated feeding rates (Jepsen et al., 1997). As larger prey are depleted over the season, peacock bass gradually shift toward smaller prey (Jepsen et al., 1997; Winemiller & Jepsen, 2004). This seasonal increase in feeding coincides with improved body condition and gonadal development, underscoring the important role of the low‐water period for both energy acquisition and reproduction (Hoeinghaus et al., 2006; Lowe‐McConnell, 1964).

Many *Cichla* species co‐occur throughout their distributions in South American rivers, thus providing a suitable model for investigating mechanisms of coexistence. In Venezuelan rivers, Winemiller et al. (1997) and Jepsen et al. (1997) observed high interspecific dietary overlap among three *Cichla* species during the low‐water period. These studies further revealed that mouth gape width limits maximum prey size ingested, with the largest species, *Cichla temensis*, consuming larger prey and exploiting a broader prey size spectrum than two smaller species (*Cichla intermedia*, *Cichla orinocensis*). Despite these findings, it is unclear whether *Cichla* trophic niches are limited by interspecific competition or whether their dietary preferences are independent of interspecific interactions. In the Venezuelan study, all *Cichla* samples were collected in areas of sympatry, with the diet breadth of allopatric *Cichla* species undocumented.

Here, we examine diets of *Cichla cataractae* (Sabaj et al., 2020; locally known as Falls Lukunani) and *C. ocellaris* (Bloch & Schneider 1801; locally known as Pond Lukunani) across their distribution within the Essequibo River Basin, Guyana. *Cichla cataractae* was described in 2020 after more than 200 years of being unrecognized by ichthyologists as a distinct species from *C. ocellaris* (Sabaj et al., 2020). The two species are very similar morphologically (i.e., cryptic), which resulted in taxonomic confusion and consequently little understanding of ecological differences. Recent work investigating the spatial distribution and habitat use of *C. cataractae* and *C. ocellaris* revealed a high degree of interspecific habitat niche partitioning (Fry & Winemiller, manuscript in review) as in other *Cichla* populations (e.g., Jespen et al., 1997; Winemiler et al., 1997). Therefore, using specimens from Fry and Winemiller (in review) and additional sampling efforts, the present study uses a multifaceted approach to assess trophic mechanisms that may additionally reinforce sympatry of Guyanese populations. Specifically, we investigated visual gut contents analysis, diet metabarcoding and stable isotope analysis to determine if *C. cataractae* and *C. ocellaris* consume similar prey items with respect to their size and taxonomic identity, and whether *Cichla* spp. demonstrate differences in their trophic niche. Our findings may reveal additional dimensions reinforcing sympatry of *Cichla* species and complement existing research suggesting habitat partitioning and dietary (i.e., gape limitation) processes promote coexistence of these ecologically important fishes (Jespen et al., 1997; Winemiler et al., 1997; Fry & Winemiller, in review).

## 2 METHODS

### 2.1 Field sampling

**FIGURE 1:**
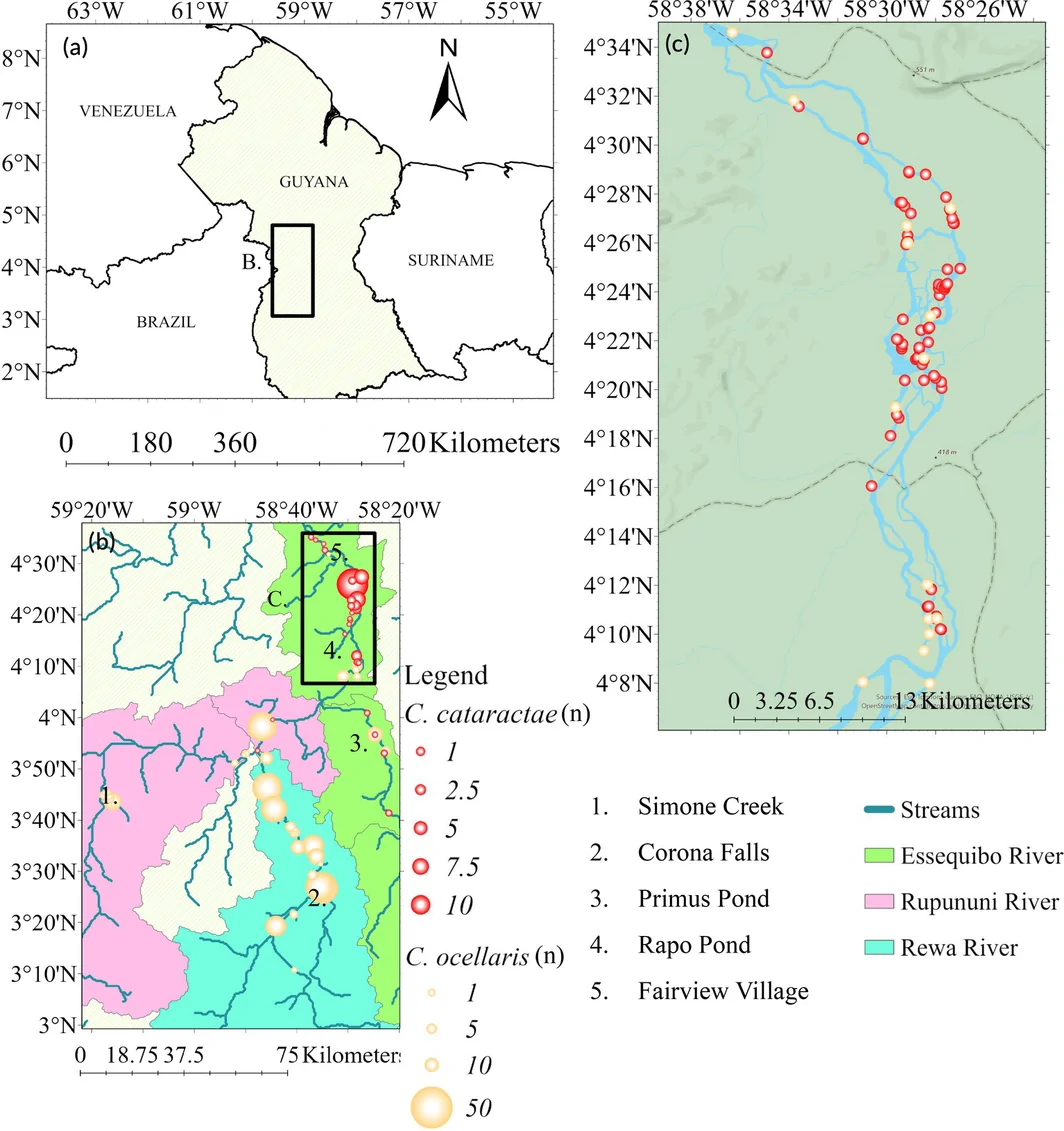
*Cichla* capture locations in the Essequibo River Basin, Guyana. Data are from the current study with four *C. cataractae* records from Sabaj et al. (2020). Panel A shows the extent of the Essequibo Basin surveyed. Panel B shows the relative abundance of the two species across the three river drainages. Panel C shows individual capture locations in an area of the Essequibo with high syntopy.

Sampling for this study was conducted in the Essequibo River Basin, Guyana. The Essequibo River lies within the Guiana Shield and has a drainage area of 156,828 km^2^ and an annual discharge ranging 154–178 km^3^ per year (Hughes et al., 2023). Sampling took place in the Essequibo, Rupununi and Rewa rivers, located between 3° 45′ N – 4° 34′ N and 59° 79′ – 58° 36′ W (Figure 1). These rivers contain a mosaic of habitats, including rapids, rocky shoals, sandbanks, side channels, floodplain lakes and creeks, with the relative proportion of each habitat type varying among rivers and among reaches within a given river (Fry & Winemiller, in review).

*Cichla* were sampled during the dry seasons in 2023 (January–March) and 2025 (March). Specimens analysed in this study captured during 2023 correspond to those analysed in Fry & Winemiller (in review). The 2023 collections included sites across the Rupununi, Rewa and Essequibo Rivers and represented a broad range of habitat types. The 2025 collections were focused on the Essequibo River, upstream from Fairview Village and downstream from the confluence of the Essequibo and Rupununi Rivers. *Cichla ocellaris* occurs widely across all three rivers and is sympatric with *C. cataractae* in the Essequibo River and lower reaches of the Rupununi River (Fry & Winemiller, manuscript in review). To account for population differences between allopatric and sympatric *C. ocellaris*, two populations were analysed separately: sympatric *C. ocellaris* (SCO) from the Essequibo River, and allopatric *C. ocellaris* (ACO) from the Rewa and upper Rupununi rivers. All *C. ocellaris* from the Rupununi were collected upstream from the highest upstream known location of *C. cataractae*, and these were assumed allopatric. Complete site descriptions are provided in Appendix A.

*Cichla* were collected using targeted angling and trolling (*n* = 1049) along with cast netting (*n* = 17). During the second campaign, targeted angling focused on habitats previously determined to be suitable for adult and sub‐adult size classes of *Cichla* (Appendix B). During both sampling campaigns, *Cichla* specimens were measured for total length (TL; cm) and mouth gape width (mm). Tissues for stable isotopes were removed from the distal dorsal portion of the caudal fin and stored in salt on a subset of fish from the first campaign (*n* = 121) and all fish from the second campaign (*n* = 126). In total, we collected fin tissue from 145 *C. cataractae*, 40 SCO, and 62 ACO for stable isotope analysis. During the first campaign, gut contents for diet analysis (*n* = 181; most fish had empty stomachs) were extracted using a modified gastric lavage technique described in Layman and Winemiller (2004). When sufficiently intact, prey items were measured for standard length (mm) and initially identified to the lowest taxonomic level before being preserved in 95% ethanol.

### 2.2 Lab preparations

#### 2.2.1 Stable isotopes

Stable isotope samples were prepared following established protocols (Arrington & Winemiller, 2002; Winemiller et al., 2011). Fin tissues were first soaked in distilled water for 48 h to remove residual salt. Samples were then dried in a laboratory oven at 60°C for 48 h. Once dried, each sample was ground to a fine, homogeneous powder using a mortar and pestle. Approximately 0.9–1.2 mg of powdered tissue was then individually placed into 4 × 6 mm tin capsules (*Costech Analytical*, Valencia, California). Samples were analysed at the Stable Isotope Laboratory at the University of California, Davis. Ratios of carbon and nitrogen isotopes were quantified using mass spectrometry and reported in standard per‐mil notation (δ^13^C and δ^15^N) reported as differences in isotopic values relative to international standards (Vienna Pee Dee Belemnite for carbon and atmospheric N₂ for nitrogen):

$$\mathrm{\delta X}=\left(\frac{R_{\text{sample}}}{R_{\text{standard}}}-1\right)\times 10^{3}$$

where R represents either ratios of ^13^C/^12^C or ^15^N/^14^N.

#### 2.2.2 Gut metabarcoding

DNA extractions from gut contents were performed using the Qiagen DNeasy Blood and Tissue Kit in a designated pre‐PCR laboratory space to minimize risk of contamination. The type of tissue selected for extraction varied depending on prey condition. To reduce the risk of obtaining DNA from gut contents of ingested prey, we attempted to obtain only tissue samples (usually muscle or bone) that were not in direct contact with the ingested prey's gastrointestinal tract. For intact prey, approximately 25 mg of dorsal muscle tissue (with or without bones and scales) was sampled. In cases where only bones or scales remained, all recovered material was used. For highly degraded material (i.e., chyme), approximately 0.5 mL was extracted. When multiple prey items were present in a single stomach, small portions of each taxon were pooled into a composite sample. Prior to extraction, all tissues were rinsed with 95% EtOH and air‐dried for 30 min. Extractions followed the manufacturer's protocol.

Prey DNA was amplified at a hypervariable region of the mitochondrial 12S rRNA gene using MiFish universal fish primers (MiFish‐F‐ GTCGGTAAAACTCGTGCCAGC and MiFish‐R‐CATAGTGGGGTATCTAATCCCAGTTT) developed by Miya et al. (2015). These primers provide broad taxonomic coverage (>95% of known fish species in reference databases) and generate short amplicons suitable for degraded DNA, such as those found in partially digested materials (Deng et al., 2024; Miya et al., 2015; Riaz et al., 2011). Amplification was carried out in a two‐step Polymerase Chain Reaction (PCR) process, with a positive control of Sockeye salmon (*Oncorhynchus nerka*) tissue and a negative control of nuclease‐free water included in each.

For the first PCR step, reactions contained 2 μL of template DNA, 5 μg/L MiFish primers (1.5 μL), 5X Green GoTaq Buffer (3 μL), 2 mM MgCl₂ (1.77 μL), 0.2 mM dNTPs (0.48 μL), 0.07 μL of GoTaq G2 Flexi DNA Polymerase and 8.3 μL of nuclease‐free water (final volume = 18.62 μL). Amplification followed an initial denaturation stage at 94°C for 3 min; 30 cycles of denaturation at 98°C for 5 s, annealing at 50°C for 10 s, extension at 72°C for 10 s; followed by a final extension stage at 72°C for 5 min. Amplification success was verified using agarose gel electrophoresis (1.5% agarose gel with GelGreen® stain in 1× TBE buffer, run at 100 V for 60 min). Amplicon bands were visualized under UV illumination with a Geldoc system, and products were compared against a 100 base pair (bp) ladder to confirm a target size of approximately 170 bp. Any runs where negative controls showed amplification were repeated until negative controls showed no evidence of amplification.

For our second PCR step, we used modified MiFish Primers with Nextera‐compatible primer tags added to the 5′ end of the sequences for Illumina Mi‐Seq, performed at the University of Minnesota Genomic Center (UMGC) using Modified MiFish‐F‐**TCGTCGGCAGCGTCAGATGTGTATAAGAGACAG**GTCGGTAAAACTCGTGCCAGC and Modified MiFish‐R‐**GTCTCGTGGGCTCGGAGATGTGTATAAGAGACAG**CATAGTGGGGTATCTAATCCCAGTTT primers (bolded nucleotides are Nextera‐compatible primers). For this PCR step, 2 μL of DNA template was amplified using 1.5 μL of 5 μg/L adapted primers, 5X Green GoTaq Buffer (3 μL), 2 mM MgCl₂ (1.77 μL), 0.2 mM dNTPs (0.48 μL), 0.07 μL of GoTaq G2 Flexi DNA Polymerase and 8.3 μL of nuclease‐free water (final volume = 18.62 μL). PCR amplification consisted of an initial denaturation stage at 94°C for 3 min; 30 cycles of denaturation at 98°C for 5 s, annealing at 65°C for 10 s, extension at 72°C for 10 s; followed by a final extension stage at 72°C for 5 min. After the second PCR step, samples were sequenced using Illumina Mi‐Seq at the UMGC. Processed raw reads at UMGC were demultiplexed to remove primer tags before each sample was deposited in its own FastQ file.

### 2.3 Statistical analyses

#### 2.3.1 Metabarcoding pipeline

Forward and reverse sequence data were first filtered using the ‘filterAndTrim’ function using default parameters in the *dada2* package (V 1.30.0) implemented with RStudio (Callahan et al., 2016; R Core Team, 2016) to remove any sequences with no data. Filtered sequences were then processed using the MiFish Pipeline (V 4.09) within the MitoFish framework under default parameters (read length 229 ± 25 bp; BLASTN identity ≥97%). This pipeline includes quality filtering, primer trimming, paired‐end merging, removal of chimeras and low‐quality reads and denoising to generate amplicon sequence variants (ASVs). Taxonomic assignments were made using the MitoFish database. To ensure conservative identifications, only genus‐level assignments were retained.

Because field conditions limited the ability to implement strict contamination controls, contamination was addressed bioinformatically. First, all reads present in the negative controls were subtracted from the corresponding samples. Second, sequencing depth was standardised by scaling reads to relative proportions within each sample. Finally, prey taxa representing <25% of a sample's reads were excluded from further analysis. Using these criteria, prey items were identified in 166 of 181 samples. Most stomachs contained a single prey taxon (*n* = 140). The remaining samples detected two taxa (*n* = 26). Raw sequences, read depths and reference sequences before bioinformatic reduction can be found in supplementary Files 1(S1) and 2 (S2).

#### 2.3.2 Morphology and diet relationships

To determine whether body size or gape variation was related to the diet of *Cichla*, we independently analysed group differences in total length, mouth gape width, number of prey items per specimen, prey item length and standardized prey item length (ratio of prey/predator length) using one‐way Analysis of Variance (ANOVA). Pairwise differences among *C. cataractae*, ACO and SCO were further assessed using Tukey's Honestly Significant Difference (HSD) tests for each analysis. Visual identification of gut contents was assessed between groups but not statistically analysed due to relatively coarse taxonomic resolution (i.e., family) and similarities to metabarcoding findings.

Using filtered metabarcoding data, prey compositional differences were assessed using multivariate statistics. For each *Cichla* specimen, genera assignments from barcoding were used to create a presence‐absence data matrix. Diet data were then transformed into a dissimilarity matrix using Jaccard distances for input in non‐metric multidimensional scaling (NMDS) modelling. Specimen scores from our dominant NMDS axes (i.e., NMDS 1, NMDS 2) were used as response variables for a Permuted Multivariate Analysis of Variance (PERMANOVA) with the predictor variable of *Cichla* group assignment (i.e., *C. cataractae*, ACO, SCO) using 1000 permutations to determine significant differences in diet diversity. Results were further visualized in two‐dimensional space after non‐metric multidimensional scaling (NMDS). Both NMDS and PERMANOVA analyses were conducted in the *vegan* package in R (V 2.6–10, Oksanen et al., 2022).

#### 2.3.3 Stable isotopes

Using estimated δ^13^C and δ^15^N values from each specimen, we used the *nicheROVER* package (Swanson et al., 2015), a probabilistic method for calculating niche region overlap that utilizes a Bayesian framework. For each comparison, we generated 1000 random draws from the posterior distributions of the mean (μ) and variance–covariance (Σ) parameters. The prior distribution was specified as π(μ, Σ) ∝ |Σ|^−n^ ^−1^, following the default *nicheROVER* implementation. The scalar vector of niche region sizes (alpha) was set at 0.95, corresponding to a 95% probability region. Pairwise ellipse overlaps were then calculated to determine the degree of isotopic niche overlap between groups. To confirm statistical significance, we conducted a Hotelling's two‐sample T2‐test in R using the *DescTools* package (Signorell, 2025, Version 00.99.54) on the raw isotope values. Additionally, to test for ontogenetic diet shifts, simple linear regression was used to assess the correlation between *Cichla* total length and δ^15^N (a proxy for trophic position) for each group.

## 3 RESULTS

### 3.1 Morphology and visual gut contents analysis

Our ANOVA and Tukey's HSD modelling revealed significant morphological and dietary differences among *Cichla* groups. We observed a significant difference in body size (*F*_2,951_ = 101.9, *p* < 0.001). On average, *Cichla cataractae* (mean ± SD = 46.8 ± 7.56 cm) was significantly larger (*p* < 0.001) than both SCO (34.5 ± 2.55 cm) and ACO (36.7 ± 4.76 cm). Body size did not differ between sympatric and allopatric *C. ocellaris* (*p* = 0.31). The proportion of individuals with food in their stomachs differed significantly among groups (*F*_2,876_ = 17.4, *p* < 0.001). A significantly greater proportion of *C. cataractae* (*p̂* = 0.41) contained prey compared to ACO (*p̂* = 0.24; *p* < 0.001) and SCO (*p̂* = 0.28; *p* < 0.001), whereas the two *C. ocellaris* groups did not differ (*p* = 0.61). Among individuals containing prey, the number of food items per stomach did not differ between groups (*F*_2,190_ = 0.51, *p* = 0.60).

Significant differences were also observed in mouth gape width (*F*_2,183_ = 48.0, *p* < 0.001) and prey length (*F*_2,75_ = 16.27, *p* < 0.001). *Cichla cataractae* had a significantly larger (*p* < 0.001) mouth gape (69.6 ± 12.7 mm) than ACO (54.6 ± 8.2 mm) and SCO (51.7 ± 4.6 mm), with no difference between *C. ocellaris* groups (*p* = 0.74). Mean prey length consumed by *C. cataractae* (117 ± 48 mm) was also significantly greater (*p* < 0.001) than that of ACO (57 ± 37 mm) and SCO (59 ± 24 mm), whereas prey length did not differ between sympatric and allopatric *C. ocellaris* (*p* = 0.98). Prey length relative to predator total length differed significantly among groups (*F*_2,75_ = 7.28, *p* = 0.002). *Cichla cataractae* consumed proportionally larger prey (0.25 ± 0.08) than ACO (0.15 ± 0.10; *p* < 0.001), but not SCO (0.18 ± 0.09; *p* = 0.114). The two *C. ocellaris* groups did not differ (*p* = 0.79). Visual prey identification was difficult due to advanced stages of digestion in most samples, but family identification was possible for 56 prey items removed from stomachs. Both species consumed fishes from several families and orders, including some of the same taxa (Table 1).

**TABLE 1:** *Cichla* prey visually identified from stomach contents (values are frequencies of occurrence).

Prey order	Prey family	*C. cataractae* (*n*)	*C. ocellaris* (*n*)
Characiformes	Acestrorhynchidae		1
	Characidae	2	23
Siluriformes	Doradidae	1	2
	Loricariidae	5	2
	Pimelodidae	2	6
Cichliformes	Cichlidae	1	5
Gymnotiformes	Apteronotidae		1
	Rhamphichthyidae	2	

#### 3.1.1 Gut contents DNA analysis

**FIGURE 2:**
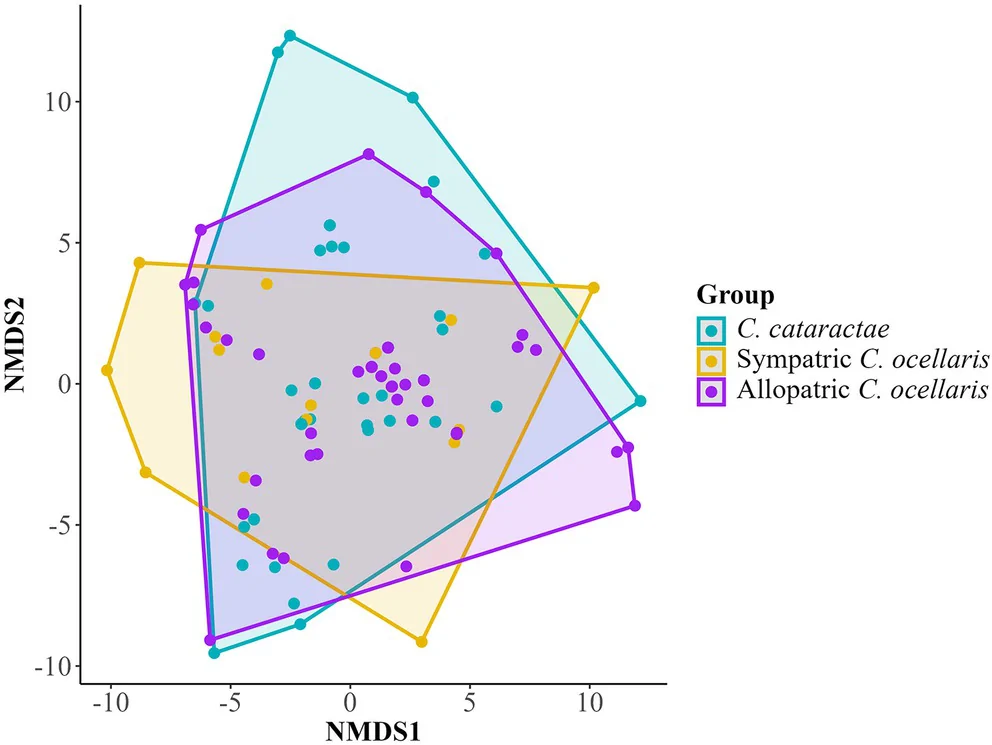
Ordination plot based on input Jaccard distances for non‐metric multidimensional scaling (NMDS) of *Cichla* diet composition using DNA analysis of gut contents (stress = 0.0011). Points represent prey community NMDS values, and shaded areas represent diet breadth minimum convex hulls for each group.

In total, 62, 28 and 92 unique food items across 25, 18 and 28 different genera were detected based on analysis of DNA from stomach contents of *C. cataractae*, SCO and ACO, respectively. Prey items identified from DNA analysis were taxonomically broad from multiple families and orders. For *C. cataractae*, the median prey count per genera was 2, with a maximum of 14 (mean ± SD; 2.5 ± 2.8). For SCO, the median prey count per genera was 1, with a maximum of 5 (1.6 ± 1.1). For ACO, the median prey count per genera was 2, with a maximum of 23 (3.3 ± 4.4). Prey items were from 12 families and 4 orders for *C. cataractae*, 10 families and 4 orders for SCO, and 13 families and 5 orders for ACO (Table 2). PERMANOVA of gut DNA identifications showed a significant difference in diet composition (*F* = 3.78, *p* = 0.001), though very weak, with group identity minimally explaining diet variation (*R* ^2^ = 0.05; Figure 2).

**TABLE 2:** *Cichla* diets determined by gut metabarcoding. Sympatric *C. ocellaris* were collected in the Essequibo River. Allopatric *C. ocellaris* were collected in the Rewa and upper Rupununi rivers.

Order	Family	Genus	*C. cataractae*	*C. ocellaris*	
				Sympatric	Allopatric
			Diet count (*n*)		
Characiformes	Acestrorhamphidae	*Hyphessobrycon*	2	1	8
		*Jupiaba*		1	9
		*Moenkhausia*	1		1
		*Poptella*	1	1	3
	Anostomidae	*Hypomasticus*	2		
		*Leporinus*	7		1
	Characidae	*Phenacogaster*			1
		*Roeboides*	1		3
		*Tetragonopterus*	1		1
	Chilodontidae	*Chilodus*		1	
	Curimatidae	*Cyphocharax*		2	
	Cynodontidae	*Cynodon*			2
	Erythrinidae	*Hoplias*	1		1
	Prochilodontidae	*Prochilodus*	1	2	3
	Serrasalmidae	*Metynnis*	1		
		*Serrasalmus*			1
Cichliformes	Cichlidae	*Acarichthys*			3
		*Geophagus*	1	3	4
		*Guianacara*	2	1	
		*Mesonauta*			1
		*Satanoperca*		1	
Clupeiformes	Engraulidae	*Anchoviella*		5	3
Gymnotiformes	Rhamphichthyidae	*Gymnorhamphichthys*	2		
	Sternopygidae	*Eigenmannia*			6
Siluriformes	Auchenipteridae	*Ageneiosus*		1	23
		*Auchenipterus*		3	1
	Callichthyidae	*Corydoras*			3
	Doradidae	*Amblydoras*		1	
		*Doras*	2		
		*Leptodoras*	6		
		*Tenellus*		1	
		*Trachydoras*	2		2
	Heptapteridae	*Pimelodella*	2		
	Loricariidae	*Aphanotorulus*		1	1
		*Cteniloricaria*	2	1	1
		*Hypoptopoma*	1		3
		*Hypostomus*	1		
		*Limatulichthys*			2
		*Loricaria*	2		
		*Loricariichthys*	1		
		*Peckoltia*	2		
		*Pseudancistrus*	14	1	3
	Pimelodidae	*Pimelodus*	4		1
		*Sorubim*		1	1

### 3.2 Stable isotope analysis

**FIGURE 3:**
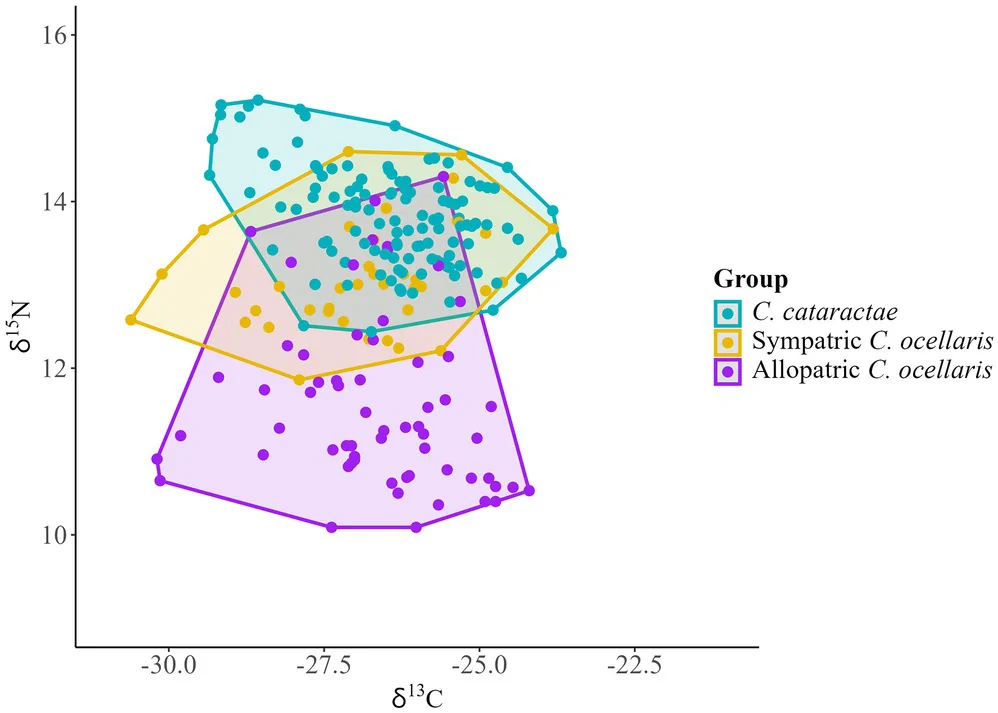
δ^13^C and δ^15^N isospace plot for three populations of *Cichla* from the Essequibo River Basin. Points represent individual permil (δ) differences in isotopic values relative to standards. Shaded areas represent isospaces illustrated with minimum convex hulls for each group.

**FIGURE 4:**
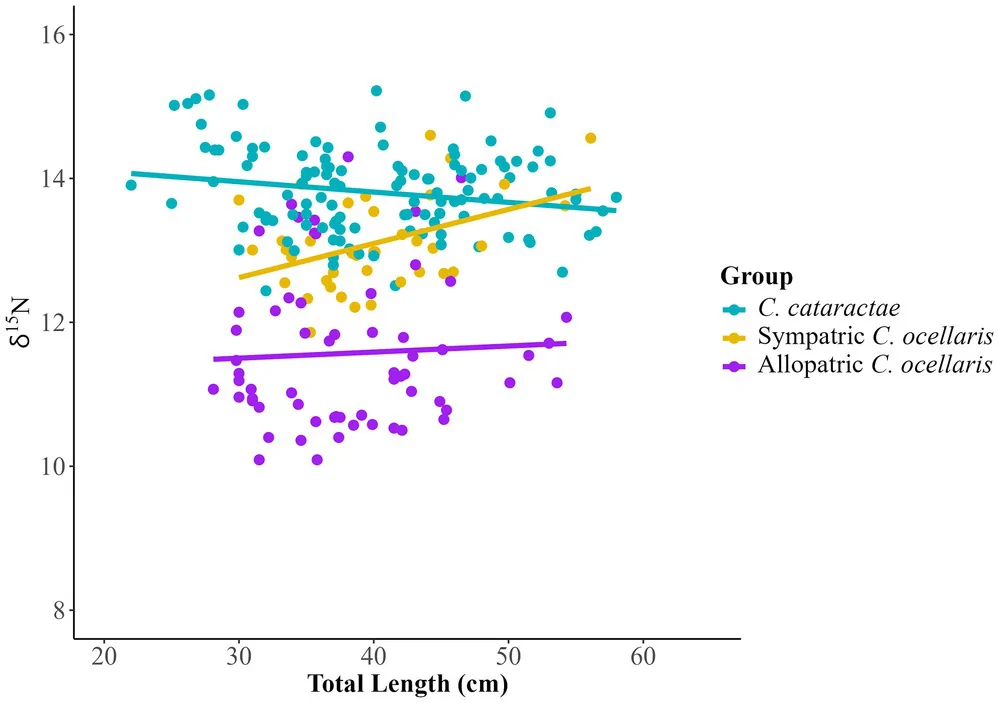
Relationship between per mil δ^15^N values and total length (cm) for *Cichla cataractae* (blue), sympatric *C. ocellaris* (gold) and allopatric *C. ocellaris* (purple). Each point represents an individual fish, with fitted linear regression lines shown for each group (solid lines).

Distributions of specimens within δ^13^C × δ^15^N isospaces differed interspecifically (*T* ^2^ = 51.4, df = 2, 168, *p* < 0.001), and intraspecifically among *Cichla* groups (*T* ^2^ = 70.2, df = 2, 99, *p* < 0.001; Figure 3). Isotopic niche overlap was asymmetric between *C. cataractae* and SCO. The probability that SCO individuals occurred within the isospace niche region of *C. cataractae* was moderate (median = 0.70, 95% CI: 0.56–0.83), and higher for the *C. cataractae*‐SCO comparison (median = 0.80, 95% CI: 0.66–0.92). SCO and ACO isotopic niche overlap was also asymmetric, with high SCO isospace overlap probability with ACO (median = 0.80, 95% CI: 0.62–0.93) and moderate ACO isospace overlap probability with SCO (median = 0.54, 95% CI: 0.26–0.59). *C. cataractae* had higher δ^15^N values than SCO (*t* = 6.54, df = 63.3, *p* < 0.001), and SCO had higher δ^15^N values than ACO (*t* = 9.22, df = 99.83, *p* < 0.001). Linear regressions for δ^15^N in relation to fish total length were inconsistent among groups (Figure 4). *Cichla cataractae* did not have any correlation between total length and δ^15^N values (*R* ^2^ = 0.012, *F* = 2.76, df = 1143, *p* = 0.10), SCO had a weak positive relationship (*R* ^2^ = 0.16, *F* = 8.58, df = 1.38, *p* = 0.006), and the relationship for ACO was nonsignificant (*R* ^2^ = 0.003, *F* = 0.17, df = 1.60, *p* = 0.68).

## 4 DISCUSSION

### 4.1 Trophic niche overlap

Our study found that both *C. cataractae* and *C. ocellaris* inhabiting the Essequibo River Basin are piscivores with broad diets. Findings from three different methods revealed an overall lack of dietary segregation by prey identity and moderate segregation by prey size during the annual low‐water period when prey abundance should be relatively low. These findings align with findings from a study that revealed high dietary overlap among detritivorous and herbivorous tilapiine cichlids during the low‐water period in south‐central Africa (Winemiller & Kelso‐Winemiller, 2003). In the Essequibo River, both *C. cataractae* and *C. ocellaris* consumed prey from multiple orders, families and genera, consistent with the broad diets documented for in *Cichla* species in rivers of Venezuela. In the Cinaruco River, *C. temensis*, *C. intermedia* and *C. orinocensis* consumed fishes from diverse taxa, with diet composition inferred to reflect prey size availability and local assemblage structure within habitats occupied more so than species‐specific preferences (Jepsen et al., 1997; Winemiller et al., 1997).

Prey size appears to be the principal factor influencing dietary differences between *C. cataractae* and *C. ocellaris*. Prior research has inferred that *Cichla* preferentially target prey nearest to the maximum size that can be consumed based on mouth gape size (Jepsen et al., 1997; Montaña et al., 2011). Our findings support this interpretation given that *C. cataractae* were significantly larger than *C. ocellaris* and had a larger relative mouth gape that would enable them to consume larger fish. This is supported by higher δ^15^N values of *C. cataractae*, which place them higher in the food web than *C. ocellaris* (Layman et al., 2007). Differences in body size distributions of the two species align with phylogenetic relationships within the genus (Willis et al., 2007), wherein Clade A species (e.g., *C. cataractae*) attain larger sizes than Clade B species (e.g., *C. ocellaris*). Both absolute prey size and relative prey size (standardized by predator size) were larger for *C. cataractae* compared to *C. ocellaris*, with the number of prey observed in stomachs varying minimally between species. This size‐based diet segregation likely facilitates coexistence between these syntopic species. *Cichla cataractae*, which preferentially consumes larger individuals within prey populations, may reduce competition by leaving smaller prey available for *C. ocellaris*. Conversely, *C. ocellaris* may be unable to exploit larger prey individuals, thereby leaving those resources available for *C. cataractae*.

Classical niche theory predicts that strong competition for limited resources may result in either competitive exclusion or coexistence via niche compression, resulting in niche partitioning. In the Essequibo River, two *Cichla* species are abundant in sympatry, indicating the absence of competitive exclusion. The nearly complete overlap in diet composition indicates a lack of trophic niche partitioning during the dry season. Even if trophic niche compression were occurring, SCO would be expected to exhibit a narrower diet breadth than ACO, yet their diet breadth based on prey identity, prey size and carbon stable isotope values were almost identical. Although ACO nitrogen values had a greater range, this is likely due to specimens being collected from both forested and savannah watersheds, which affect primary producer nitrogen values (Vander Zandan et al., 2005), rather than dietary differences. Several mechanisms may help explain the persistence of these cryptic species despite high dietary overlap. First, seasonal hydrology may yield intervals of relative food resource abundance. Piscivorous fishes inhabiting floodplain habitats may show higher levels of trophic niche segregation during the low‐water periods after prey populations have been reduced and competition therefore becomes more intense (e.g., Winemiller, 1989, 1991). In other cases, piscivorous fishes revealed broader trophic niches under low‐water conditions when prey become more concentrated in shrinking aquatic habitats (de Andrade et al., 2019; Ferreira et al., 2014; Hoeinghaus et al., 2006). Our study was conducted during the end of the low‐water period, when fish prey availability likely was low. The high degree of trophic niche overlap between sympatric populations suggests either that competition is weak or that these species partition niche space according to one or more alternative niche dimensions. Importantly, the two *Cichla* species strongly partitioned habitat during the low‐water period in the Essequibo River (Fry & Winemiller, manuscript in review), which likely reduces competition for prey by segregating foraging spaces even if prey spectra are similar among those spaces.

### 4.2 Methodological complementarity

The present study appears to be among the first to apply three different approaches to compare diets of closely related piscivorous species. By using different analysis methods, a more complete estimate of feeding ecology was obtained. Stable isotope analysis revealed that *C. cataractae* and sympatric *C. ocellaris* (SCO) assimilated isotopes from similar sources, and this assimilation is based on a longer time scale (several weeks) compared to evidence obtained from gut contents analysis. A major advantage of stable isotope analysis is the ability to estimate vertical trophic positions. The higher δ^15^N values observed for *C. cataractae* are consistent with the expectation that, among gape‐limited predators, larger individuals feed at higher trophic levels. However, stable isotope approaches generally are not effective for resolving diet composition at fine scales of resolution, especially when multiple basal resources are assimilated or isotopic signatures overlap broadly among sources (Phillips et al., 2014).

Visual and molecular analyses of gut contents helped overcome limitations of stable isotope analysis. Both methods indicated that the two generalist piscivores consumed diverse taxa. DNA metabarcoding proved especially valuable because it enabled identification of highly digested prey items that could not be visually resolved, expanding identifications to 44 genera. This level of resolution would have been impossible through visual inspection alone, given the rapid digestion rates of carnivorous tropical fishes (dos Santos et al., 2011). A limitation of DNA analysis of gut contents is that it does not provide information on prey size or volume. To compensate for this limitation, the visual analysis of gut contents allowed for several recovered prey items to be measured, which revealed size differences of prey consumed by the two species. Thus, the three methods were complementary. Visual identifications of a limited number of prey items partially confirmed findings from DNA analysis. Patterns obtained from NMDS ordinations of diet composition closely paralleled results from sympatric isospace plots. In addition, δ^15^N‐inferred vertical trophic positions were consistent with expectations based on prey size data, given that larger predatory fishes often have higher nitrogen isotopic ratios (e.g., Keppeler et al., 2020). Collectively, our findings highlight the value of integrating multiple dietary approaches to better understand fish trophic ecology. Gut contents analysis provides only a short‐term dietary estimate (‘snapshot’) and is sensitive to sample size. Stable isotope analysis provides a broader, time‐integrated view but lacks taxonomic resolution. DNA metabarcoding fills this gap by identifying food items at a finer scale of resolution, even for partially digested material. Taken together, these methods allow for a more comprehensive understanding of trophic ecology than can be obtained using any single approach.

### 4.3 Future directions

In addition to comparing trophic niches of sympatric peacock bass species in the Essequibo River, our study provides an initial description of the trophic ecology of the recently described *Cichla cataractae*. Additional research is needed for a more complete understanding of the autecology and roles of these species within aquatic communities of the Essequibo River Basin. We obtained evidence during the low‐water period of the annual flood cycle, and it is unknown whether observed patterns are consistent across phases of the hydrological cycle. Peacock bass species in the Cinaruco River, Venezuela, were found to shift diet composition and diet overlap across seasons (Jepsen et al., 1997). Considering this, further research should add a seasonal component to better understand temporal patterns in habitat use, diet and trophic patterns of Essequibo peacock bass. Regarding metabarcoding, the accuracy of our results is limited by the completeness of molecular reference libraries. As these resources expand, prey identification to the species level should be possible. Similarly, inclusion of isotopic ratios of additional elements, such as hydrogen or sulfur, could facilitate more accurate estimates of isotopic spaces occupied by populations. Stable isotope analysis of additional potential sources (prey) for input into mixing models might provide improved estimates of consumer diet. Incorporating these refinements in future research would further advance understanding of fish trophic ecology and coexistence in diverse tropical systems.

## AUTHOR CONTRIBUTIONS

Benton L. Fry and Kirk O. Winemiller conceptualized, planned and conducted all data collection and statistical analyses, secured funding and prepared the manuscript. Hayden C. Roberts aided in sample collection, lab processing and manuscript preparation. Benton L. Fry was the project lead. Kirk O. Winemiller was the project supervisor.

## FUNDING INFORMATION

Texas A&M HEEP Fellowship, Texas A&M Ecology and Evolution Biology Mini‐Grant (X2), Texas A&M Ecology and Conservation Biology Travel Grant, the eDNA Collaborative Microgrant, the US National Science Foundation IRES program grant no. OISE‐2153453, and the International Sportfish Fund via funds from the estate of George and Carolyn Kelso.

## Supporting information


**Table S1.** FASTA file with all *Cichla* diet reads.

**Data S1.** Diet DNA ASVs, reference sequences and read depths before bioinformatic manipulation.

## Data Availability

The data that supports the findings of this study are available in the supplementary material of this article.

## APPENDIX A SAMPLING LOCATIONS

Sampling was initiated in the Rupununi River on 9 January near the confluence of Simoni Creek (3° 45′ 34″ N, 59° 17′ 05″ W) and continued downstream until 21 January, with the last sampling site being 14.75 km below the confluence of the Rewa and Rupununi rivers (3° 59′ 39″ N, 58° 44′ 42″ W). This location was selected because it was the reach where *C. cataractae* were first collected in 2019 (Sabaj et al., 2020). Sampling in the Rewa River started on 23 January and ended on 8 March. Sampling in the Rewa progressed upstream until Corona Falls (3° 10′ 37″ N, 58° 40′ 22″ W), a natural barrier to upstream fish dispersal. Sampling in the Essequibo River occurred from 27 January to 6 February. and from 11 to 26 March. The first campaign sampled 31 km upstream from the confluence of the Essequibo and Rupununi rivers in a stretch of river locally referred to as ‘Primus' (3° 53′ 35″ N, 58° 22′ 39″ W). During the same campaign, we sampled downstream from the Essequibo‐Rupununi confluence for 25 km until Rapo Pond (4° 10′ 39″ N, 58° 26′ 13″ W). A second campaign and the 2025 sampling trip conducted surveys downstream from Rapo Pond to a location just upstream from the village of Fairview (4° 33′ 53″ N, 58° 34′ 49″ W).

## APPENDIX B CICHLA ANGLING METHODS

*Cichla* are popular sportfish and are highly susceptible to capture using artificial lures (Winemiller et al., 2021). In this study, we employed angling methods specifically designed to target fish in proximity to their habitat, using a range of lures tailored to local conditions. For trolling, we used both shallow‐diving (mean water depth <3 m) and deep‐diving crankbaits (mean water depth >3 m), such as Rapala X‐Raps and Rapala Floating Magnums.

In targeted angling, lure selection was guided by habitat characteristics, including water depth, structural complexity, and water quality. In deeper water (>3 m), we used lipless crankbaits (e.g., Bill Lewis Rat‐L‐Trap), deep‐diving crankbaits (e.g., Rapala Glass Shad Rap), and deep‐diving jerkbaits (e.g., Rapala Deep Shadow Rap), all with comparable size and sound profiles. In shallow water (<3 m), we primarily used shallow‐diving jerkbaits (e.g., Rapala Husky Jerk) and bucktail jigs (e.g., VMC Bucktail Jig). In structurally complex habitats or areas with low dissolved oxygen (<3.0 mg/L), we used weedless spoons (e.g., Johnson Silver Minnow) to minimize lure loss.

Trolling was conducted in each reach prior to targeted angling to identify areas warranting focused effort, particularly those lacking key features of *Cichla* habitat (e.g., pelagic zones in floodplain ponds). Given the limited knowledge of *C. cataractae* habitat use, we aimed to sample across all available habitat types. Trolling was restricted to sonar transect paths and accounted for less than 10% of the total angling effort.

Targeted angling was distributed throughout the most suitable *Cichla* habitats within each reach. In ponds and oxbow lakes, effort was typically spread by paddling along the perimeter. In creeks, drifting down the centreline usually provided full coverage. In river channels, a combination of controlled drifting in shallow mid‐channel areas and paddling along the margins was used to maximise coverage.

Cast nets (2 m bag with 10 mm mesh) were used to collect *Cichla* samples (*n* = 17) in hypoxic floodplains (dissolved oxygen <0.2 mg/L) when specimens were visually present in a habitat and not actively attacking lures. Cast net throws were targeted at only visible fish, and the capture location was recorded.
