# Supplementary material for: High trophic niche overlap between sympatric peacock basses (Cichliformes: Cichlidae: Cichla): Concordant findings from three dietary analysis methods
Source: J Fish Biol. 2026 May 12;109(2):1034–45. doi: 10.1111/jfb.70485 (PMC13521748; doi:10.1111/jfb.70485)
Supplement: Supplementary file 1 [file JFB-109-1034-s002.docx]

>4_Sample_ASV1

CACCGCGGTTATACGAGAGACCCTAGTTGATTAAAGCGGCGTAAAGAGTGGTTATGGAAC

TATTCTTTTAAAGCAGAAAACCTCTCAAACTGTTATACGCACCCAGAGGTCTGAATCCCT

CACACGAAAGTGACTTTATTTATGCCTACCAGAACCCACGAAAGCTGGGACA

>4_Sample_ASV2

CACCGCGGTTATACGAAAGACCCTAGTTTATAGGTACGGCGTAAAGGGTGGTTAGGGGAC

AAACAAAATAAAGCCAAAGACCTCCTAAGCCGTCATACGCTCACGGAAGCACGAAGCCCA

AACACGAAAGTAGCTTTACCGAACATGCCCGACTCCACGAAAGCTGGGGAA

>4_Sample_ASV3

CACCGCGGTTATACGAGAGGCCCAAGTTGACAGACACCGGCGTAAAGAGTGGCTAGGGAA

AAATTACTACTAAAGCCGAACACCTTCAGAACTGTCATACGTTCCCGAAGATAAGAAGCC

CCACCACGAAAGTGACTTTATATTACCTGACCCCACGAAAGCTAAGAAA

>4_Sample_ASV4

CACCGCGGTTATACGAAAGACCCTAGTTGATAGACACCGGCGTAAAGAGTGGCTAGGGAA

AAATTACTACTAAAGCCGAACACCTTCAGAACTGTCATACGTTCCCGAAGATAAGAAGCC

CCACCACGAAAGTGGCTTTATATTACCCGACCCCACGAAAGCTAAGAAA

>4_Sample_ASV5

CACCGCGGTTATACGAGAGGCCCAAGTTGACAGACACCGGCGTAAAGAGTGGCTAGGGAA

AAATTACTACTAAAGCCGAACACCTTCAGAACTGTCATACGTTCCCGAAGATAAGAAGCC

CCACCACGAAAGTGGCTTTATATTACCTGACCCCACGAAAGCTAAGAAA

>4_Sample_ASV6

CACCGCGGTTATACGAGAGGCCCAAGTTGACAGACACCGGCGTAAAGAGTGGCTAGGGAA

AAATTACTACTAAAGCCGAACACCTTCAGAACTGTCATACGTTCCCGAAGATAAGAAGCC

CCACCACGAAAGTGGCTTTATATTACCCGACCCCACGAAAGCTAAGAAA

>4_Sample_ASV7

CACCGCGGTTATACGAGAGGCCCAAGTTGACAGACACCGGCGTAAAGAGTGGCTAGGGAA

AAATTACTACTAAAGCCGAACACCTTCAGAACTGTCATACGTTCCCGAAGATAAGAAGCC

CCACCACGAAAGTGGCTTTATATTACCCGACCCCACGAAAGCTGTGAAA

>4_Sample_ASV8

CACCGCGGTTATACGAGAGGCCCAAGTTGACAGACACCGGCGTAAAGAGTGGCTAAGGAA

AAATTTCTACTAAAGCCGAACACCTTCAGAACTGTCATACGTTCCCGAAGAAAAGAAGCC

CCACCACGAAAGTGGCTTTATATTACCAGACCCCACGAAAGCTGTGAAA

>4_Sample_ASV9

CACCGCGGTTATACGAAAGACCCTAGTTTATAGAAACGGCGTAAAGGGTGGTTAAGGGAC

AAACAAAATAAGGCCAAAGACTTCCTAAGCCGTCATACGCTCCACGAAAGCACGAAGCCC

AAACACGAAAGTAGCCTTAATAACTTCACCTGACCCCACGAAAGCTGGGAGA

>4_Sample_ASV10

CACCGCGGTTATACGAGAGGCCCAAGTTGATAAGCCCGGCGTAAAGAGTGGTTATGGAGA

ATAAAACACTAAAGCTAAAGACCCCCTAGGCTGTTATACGCACCTGGCGGCTCGAACCAC

CTATACGAAAGTAGCTTTACCCCCCTTCCACCAGAATCCACGACAGCTGGGGCA

>4_Sample_ASV11

CACCGCGGTTATACGAGAGGCCCTAGTTGATAGCCACGGCGCAAAGAGTGGTTAAGGAGC

CCCACTAAATAAAGTCGAACACTTCCTAGGCCGTTATACGTTTTCTAGAAGCACGAAACC

CAATTACACGAAAGCAACTTTATACTAAACAACCTGACCCCACGAAAGCTAAGAAA

>4_Sample_ASV12

CACCGCGGTTATACGAGAGGCCCAAGTTGACAGACATCGGCGTAAAGAGTGGTTAAGGAA

ACCCCATTACTAAAGCCGAACGCCCTCAGAACTGTCATACGTTCCCGAAGGTAAGAAGCC

CCACTACGAAAGTGGCTTTAACCACACCCGACCCCACGAAAGCTGTGAAA

>4_Sample_ASV13

CACCGCGGTTATACGAGAGGCCCAAGTTGACAGACATCGGCGTAAAGAGTGGTTAAGGAA

ACCCCATTACTAAAGCCGAACGCCCTCAGAACTGTCATACGTTCCCGAAGGTAAGAAGCC

CCACTACGAAAGTGGCTTTAACCACACCCGACCCCACGAAAGCTAAGAAA

>4_Sample_ASV14

CACCGCGGTTATACGAGAGGCCCAAGTTGATAGATATCGGCGTAAAGAGTGGTTAAGGAA

ACCCCGTTACTAAAGCCGAACGCCCTCAGAACTGTCATACGTTCCCGAAGGTAAGAAGCC

CCACTACGAAAGTGGCTTTAACCACACCCGACCCCACGAAAGCTGTGAAA

>4_Sample_ASV15

CACCGCGGTTATACGAAAGACCCTAGTTGATAGATATCGGCGTAAAGAGTGGTTAAGGAA

ACCCCGTTACTAAAGCCGAACGCCCTCAGAACTGTCATACGTTCCCGAAGGTAAGAAGCC

CCACTACGAAAGTGGCTTTAACCACACCCGACCCCACGAAAGCTGTGAAA

>4_Sample_ASV16

CACCGCGGTTATACGAGAGGCCCAAGTTGATAGATATCGGCGTAAAGAGTGGTTAAGGAA

ACCCCGTTACTAAAGCCGAACGCCCTCAGAACTGTCATACGTTCCCGAAGGTAAGAAGCC

CCACTACGAAAGTGGCTTTAACCACACCCGACCCCACGAAAGCTAAGAAA

>4_Sample_ASV17

CACCGCGGTTATACGAGAGGCCCAAGTTGACAGACACCGGCGTAAAGAGTGGTTAAGGAA

ATTCATCAACTAAAGCCGAATGCCCTCAGGACTGTTATACGTTTGCGAAGGTAAGAAGCC

CTACTACGAAAGTGGCTTTATTCCACCTGATTCCACGAAAGCTAAGAAA

>4_Sample_ASV18

CACCGCGGTTATACGAGAGGCCCAAGTTGACAGACACCGGCGTAAAGAGTGGTTAAGGAA

ATTCATCAACTAAAGCCGAATGCCCTCAGGACTGTTATACGTTTGCGAAGGTAAGAAGCC

CTACTACGAAAGTGGCTTTATTCCACCTGATTCCACGAAAGCTGCGGAA

>4_Sample_ASV19

CACCGCGGTTATACGAGAGGCCCCAGTTGATATTAGCGGCGTAAAGGGTGGTTAAGGACT

TTAATAACTAAAGCTAAACACTTCCCCGGCTGTTGTACGTAGACCGGAAGCTTGAAGCCC

AGACACGAAAGTAGCTTTATTCCCCCTGACCCCACGAAAGCTGAGAAA

>4_Sample_ASV20

CACCGCGGTCACACGATTAACCCAAGTCAATAGAAGCCGGCGTAAAGAGTGTTTTAGATC

ACCCCCTCCCCAATAAAGCTAAAACTCACCTGAGTTGTAAAAAACTCCAGTTGACACAAA

ATAGACTACGAAAGTGGCTTTAACATATCTGAACACACAATAGCTAAGACC

>4_Sample_ASV21

CACCGCGGTTATACGAAAGACCCCAGTTTATAGACACGGCGTAAAGGGTGGTTATAGGAC

AAACTAACATAGAGCCAAAGACCCCCTAAGCCGTCATACGCTCACGAGGGCTCGAAGCCC

CTATACGAAAGTAGCTCTACCAAAAAATGCTCGACTCCACGAAAGCTGGGACA

>4_Sample_ASV22

CACCGCGGTTATACGAGAGGCCCCAGTTGATAAACACGGCGTAAAGGGTGGTTAAGGGAG

CATAAGAATAAAGCCGAAGGGCCCTTTGGCCGTTATACGCTTCTAGGTGTCCGAAGCCCA

AACACGAAAGTAGCTTTAGTTAAGCCCACCTGACTCCACGAAAACTGAGAAA

>4_Sample_ASV23

CACCGCGGTTATACGAGAGACCCTAGTTGATAAATACGGCGTAAAGAGTGGTTATGGGAA

AAACAAATAAAGTCAAACAACCTCTTAGCTGTTATACGCATTATGAGAGCATGAAGCCCC

CTCACGAAAGTAACTTTAATACCTCCTGACCCCACGAAAGCTAAGAAA

>4_Sample_ASV24

CACCGCGGTTATACGAGAGGCTCAAGTTGATGAACCCCGGCGTAAAGAGTGGTTAAGGGA

GATCAAAACTAAAGCCGAATGCTTTCAAAGCTGTTATACGCTTCCGAAAGTAAGAAGCTC

AATCACGAAAGTGGCTTTACTTTACCTGACCCCACGAAAGCTACGACA

>4_Sample_ASV25

CACCGCGGTTATACGAGAGACCCTAATTGATAGACACGGCGTAAAGAGTGGTTTAGGACA

ACCCAAAAATAAAGCTAAAGACCTTCTAAGCCGTCACACGCACGCCGAAGGCACGAAGCC

CTGACACGAAAGTAGCTTTACTATTACCCCCGACGCCACGAAAGCTAAGAAA

>4_Sample_ASV26

CACCGCGGTTATACGAAAGACCCTAGTTGATGGATCACGGCGTAAAGGGTGGTTAAGGGA

TATAAAAATAAAGCTAAAAATCCTCTTAGCTGTCATACGCTTTCCGAAGACATGAGGCCC

AATTACGAAAGTAGCTTTAAACCACCCTGACACCACGAAAGCCAAGAAA

>4_Sample_ASV27

CACCGCGGTTATACGAAAGACCCCAGTTTATAGATGCGGCGTAAAGGGTGGTTAAGAGAA

AATCAAAATAAGGCCAAAGATCCCCTAAGCAGTCATACGCTCCACGAGGACACGAAACCC

AAATACGAAAGTTGCCTTAATAAACTTCACCTGATCCCACGAAAGCTGAAAAA

>4_Sample_ASV28

CACCGCGGTTATACGAGAGGCTCAAGTTGATGAACCCCGGCGTAAAGAGTGGTTAAGGAA

AATTTAAAACTAAAGCCGAATGCCCCCAAAGCTGTTATACGCACCCGAGGATAAGAAGCC

CAATCACGAAAGTGGCTTTACACTACCTGAACCCACGAAAGCTATGAAA

>4_Sample_ASV29

CACCGCGGTTATACGAGAGGCCCAAGTTGACAAACCCCGGCGTAAAGAGTGGTTAAGGAA

AATTTAAAACTAAAGCCGAATGCCCCCAAAGCTGTTATACGCACCCGAGGATAAGAAGCC

CAATCACGAAAGTGGCTTTACACTACCTGAACCCACGAAAGCTATGAAA

>4_Sample_ASV30

CACCGCGGTTATACGAGAGGCCCAAGTTGACAAACCCCGGCGTAAAGAGTGGTTAAGGAA

AATTTAAAACTAAAGCCGAATGCCCCCAAAGCTGTTATACGCACCCGAGGGTAAGAAGCC

CAATCACGAAAGTGGCTTTACACTACCTGAACCCACGAAAGCTATGAAA

>4_Sample_ASV31

CACCGCGGTTATACGAGAGGCCCTAGTTGATAACTACCGGCGTAAAGAGTGGTTATGGAA

AAATATTTAATAAAGCCGAACACCCCCTCAGCCGTCATACGCACCTGGGAGCACGAAGAC

CTACTGCGAAAGCAGCTTTAATTATGCCTGACCCCACGACAGCTAAGAAA

>4_Sample_ASV32

CACCGCGGTTATACGAAAGACCCTAGTTTATAGACACGGCGTAAAGGGTGGTTCGGGACT

AAACAAAATAAAGCCAAAGACCTCCTAAGCCGTCATACGCTCACGGAAGCACGAAGCCCA

AGTACGAAAGTAGCTTTACAAAACATGCCTGACTCCACGAAAGCTAGGAAA

>4_Sample_ASV33

CACCGCGGTTATACGAAAGACCCTAGTTGATAGGCACGGCGTAAAGGGTGGTTAGGGATA

TATCAAAAATAAAGTTAAAGAGCCTCTAAGCTGTCGCACGCATCCCGAGAGCTCGAGACC

CAGACACGAAAGTAACTTTAAAATACCTCACCTGACCCCACGAAAGCTAAGAAA

>4_Sample_ASV34

CACCGCGGTTATACGAGAGACCCCAGTTGATAAACACGGCGTAAAGAGTGGTTAAGGGCA

CAAAACAAATAAAGTCAAACAACCTCTTAGCTGTTATACGCATTATGAGGATATGAAGCC

CCCCTACGAAAGTAACTTTACCAACCCCTGACCCCACGAAAGCTAAGAAA

>4_Sample_ASV35

CACCGCGGTTATACGAGAGACCCCAGTTGATAAACACGGCGTAAAGAGTGGTTAAGGGCA

CAAAACAAATAAAGTCAAACAACCTCTTAGCTGTTATACGCATTATGAGGATATGAAGCC

CCCCTACGAAAGTAACTTTACCAACCCCTGACCCCACGAAAGCTGGGAGA

>4_Sample_ASV36

CACCGCGGTTATACGAAAGACCCTAGTTTATAGGCACGGCGTAAAGGGTGGTTAGGGGAT

AAACAAAATAAAGCCAAAGACCTTCTAAGTCGTCATACACTCATGAAGGCACGAAGCCCA

AATACGAAAGTAACTTTACAACTTAGACCCGACTCCACGAAAGCTGGGGGA

>4_Sample_ASV37

CACCGCGGTTATACGAGAGGCCCAAGTTGACAAACTCCGGCGTAAAGCGTGGTTAAGGCA

CACTCAAAAACTAAAGCCGAATATCTTCAGAGCAGTCATACGCATCCGAAGACACGAAGC

CCCACCACGAAAGTGGCTTTATGACACCTGACCCCACGAAAGCTAGGACA

>35_Sample_ASV1

CACCGCGGTTATACGAAAGACCCTAGTTGATGGGTGCGGCGTAAAGGGTGGTTAGGGACT

ACAAAAAATAGAGCTAAAGATCCTCTAGGTCGTCATACACATCACGAGGAAACGAAGCCC

AAATACGAAAGTAGCTCTAAATATAATCCTGACGCCACGAAAGCCAAGAAA

>35_Sample_ASV2

CACCGCGGTTATACGAGAGACCCTAGTTGATTAAAGCGGCGTAAAGAGTGGTTATGGAAC

TATTCTTTTAAAGCAGAAAACCTCTCAAACTGTTATACGCACCCAGAGGTCTGAATCCCT

CACACGAAAGTGACTTTATTTATGCCTACCAGAACCCACGAAAGCTGGGACA

>35_Sample_ASV3

CACCGCGGTTATACGAAAGACCCTAGTTGATAGCCACGGCGTAAAGGGTGGTTAAGGAAC

CTAAAAAATAAAGCTAAAGACCCTCTAGGCCGTCATACGCATTACGAGGCAAACGAAACC

CCAACACGAAAGTAGCTTTAGACAAATATCCTGATGCCACGAAAGCCAAGAAA

>35_Sample_ASV4

CACCGCGGTTATACGAGAGGCCCAAGTTGACAGACACCGGCGTAAAGAGTGGCTAGGGAA

AAATTACTACTAAAGCCGAACACCTTCAGAACTGTCATACGTTCCCGAAGATAAGAAGCC

CCACCACGAAAGTGGCTTTATATTACCCGACCCCACGAAAGCTGAAAAA

>35_Sample_ASV5

CACCGCGGTTATACGAGAGGCCCAAGTTGACAGACACCGGCGTAAAGAGTGGCTAGGGAA

AAATTACTACTAAAGCCGAACACCTTCAGAACTGTCATACGTTCCCGAAGATAAGAAGCC

CCACCACGAAAGTGGCTTTATATTACCCGACCCCACGAAAGCTGTGAAA

>35_Sample_ASV6

CACCGCGGTTATACGAGAGGCCCAAGTTGACAGACACCGGCGTAAAGAGTGGCTAAGGAA

AAATTTCTACTAAAGCCGAACACCTTCAGAACTGTCATACGTTCCCGAAGAAAAGAAGCC

CCACCACGAAAGTGGCTTTATATTACCCGACCCCACGAAAGCTGTGAAA

>35_Sample_ASV7

CACCGCGGTTATACGAGAGACCCAAGTTGACAGACACCGGCGTAAAGAGTGGCTAAGGAA

AAATTTCTACTAAAGCCGAACACCTTCAGAACTGTCATACGTTCCCGAAGAAAATAAGCC

CCACCACGAAAGTGGCTTTATATTACCCGACCCCACGAAAGCTGTGAAA

>35_Sample_ASV8

CACCGCGGTTATACGAGAGGCCCAAGTTGATAAGCCCGGCGTAAAGAGTGGTTATGGAGA

ATAAAACACTAAAGCTAAAGACCCCCTAGGCTGTTATACGCACCTGGCGGCTCGAACCAC

CTATACGAAAGTAGCTTTACCCCCCTTCCACCAGAATCCACGAAAGCTGAAAAA

>35_Sample_ASV9

CACCGCGGTTATACGAGAGGCCCAAGTTGATAAGCCCGGCGTAAAGAGTGGTTATGGAGA

ATAAAACACTAAAGCTAAAGACCCCCTAGGCTGTTATACGCACCTGGCGGCTCGAACCAC

CTATACGAAAGTAGCTTTACCCCCCTTCCACCAGAATCCACGACAGCTGGGGCA

>35_Sample_ASV10

CACCGCGGTTATACGAGAGGCCCAAGTTGACAGACATCGGCGTAAAGAGTGGTTAAGGAA

ACCCCATTACTAAAGCCGAACGCCCTCAGAACTGTCATACGTTCCCGAAGGTAAGAAGCC

CCACTACGAAAGTGGCTTTAACCACACCCGACCCCACGAAAGCTGTGAAA

>35_Sample_ASV11

CACCGCGGTTATACGAGAGGCCCAAGTTGACAGACACCGGCGTAAAGAGTGGTTAAGGAA

ATTCATCAACTAAAGCCGAATGCCCTCAGGACTGTTATACGTTTGCGAAGGTAAGAAGCC

CTACTACGAAAGTGGCTTTATTCCACCTGATTCCACGAAAGCTGCGGAA

>35_Sample_ASV12

CACCGCGGTTATACGAGAGGCCCCAGTTGATATTAGCGGCGTAAAGGGTGGTTAAGGACT

TTAATAACTAAAGCTAAACACTTCCCCGGCTGTTGTACGTAGACCGGAAGCTTGAAGCCC

AGACACGAAAGTAGCTTTATTCCCCCTGACCCCACGAAAGCTGAGAAA

>35_Sample_ASV13

CACCGCGGTTATACGAGAGGCCCCAGTTGATATTAGCGGCGTAAAGGGTGGTTAAGGACT

TTAATAACTAAAGCTAAACACTTCCCCGGCTGTTGTACGTAGACCGGAAGCTTGAAGCCC

AGACACGAAAGTAGCTTTATTTCCCCTGACCCCACGAAAGCTGAGAAA

>35_Sample_ASV14

CACCGCGGTTATACGAGAGGCCCCAGTTGATATTAGCGGCGTAAAGGGTGGTTAAGGACT

TTAATAACTAAAGCCAAACACTTCCCCGGCTGTTGTACGTAGACCGGAAGCTTGAAGCCC

AGACACGAAAGTAGCTTTATTTCCCCTGACCCCACGAAAGCTGAGAAA

>35_Sample_ASV15

CACCGCGGTCACACGATTAACCCAAGTCAATAGAAGCCGGCGTAAAGAGTGTTTTAGATC

ACCCCCTCCCCAATAAAGCTAAAACTCACCTGAGTTGTAAAAAACTCCAGTTGACACAAA

ATAGACTACGAAAGTGGCTTTAACATATCTGAACACACAATAGCTAAGACC

>35_Sample_ASV16

CACCGCGGTTATACGAGAGACCCCAGTTGATAGCTACGGCGTAAAGAGTGGTTAAGGACC

CCATTTAATAAAGCCAAAGACCCCCCAAGCCGTCATACGCACACGAGGGCGCGAAGCTCG

CACACGAAAGTAGCTCTAATTTACCCGACGCCACGAAAGCTAAGGAA

>35_Sample_ASV17

CACCGCGGTTATACGAGAGACCCTAGTTGATAAATACGGCGTAAAGAGTGGTTATGGGAA

AAACAAATAAAGTCAAACAACCTCTTAGCTGTTATACGCATTATGAGAGCATGAAGCCCC

CTCACGAAAGTAACTTTAATACCTCCTGACCCCACGAAAGCTAAGAAA

>35_Sample_ASV18

CACCGCGGTTATACGAGAGACCCTAATTGATAGACACGGCGTAAAGAGTGGTTTGGGAAA

GCCCAAAAATAAAGCTAAAGACCTTCTAAGCCGTCACACGCACGCCGAAGGCACGAAGCC

CTGACACGAAAGTAGCTTTACTATTACCCCCGACGCCACGAAAGCTAAGAAA

>35_Sample_ASV19

CACCGCGGTTATACGAAAGACCCTAATTGATAGCTCACGGCGTAAAGGGTGGTTTGGGGA

AACCTCAAAATAAAGCTAAAGACCTTCTAAGCCGTTACACGCATACCGAAGGCACGAGGC

CCTAACACGAAAGTAGCTTTACTATTACCCCTGACGCCACGAAAGCTAAGAAA

>35_Sample_ASV20

AACCGCGGTTATACGAGAGACCCTAATTGATAGCTCACGGCGTAAAGGGTGGTTTGGGGA

AACCTCAAAATAAAGCTAAAGACCTTCTAAGCCGTTACACGCATACCGAAGGCACGAGGC

CCTAACACGAAAGTAGCTTTACTATTACCCCTGACGCCACGAAAGCTGAAAAA

>35_Sample_ASV21

AACCGCGGTTATACGAGAGACCCTAATTGATAGCTCACGGCGTAAAGGGTGGTTTGGGGA

AACCTCAAAATAAAGCTAAAGACCTTCTAAGCCGTTACACGCATACCGAAGGCACGAGGC

CCTAACACGAAAGTAGCTTTACTATTACCCCTGACGCCACGAAAGCTAAGAAA

>35_Sample_ASV22

CACCGCGGTTATACGAGAGACCCTAATTGATAGACACGGCGTAAAGAGTGGTTTAGGACA

ACCCAAAAATAAAGCTAAAGACCTTCTAAGCCGTCACACGCACGCCGAAGGCACGAAGCC

CTGACACGAAAGTAGCTTTACTATTACCCCCGACGCCACGAAAGCTAAGAAA

>35_Sample_ASV23

CACCGCGGTTATACGAAAGACCCTAGTTGATGGATCACGGCGTAAAGGGTGGTTAAGGGA

TATAAAAATAAAGCTAAAAATCCTCTTAGCTGTCATACGCTTTCCGAAGACATGAGGCCC

AATTACGAAAGTAGCTTTAAACCACCCTGACACCACGAAAGCCAAGAAA

>35_Sample_ASV24

CACCGCGGTTATACGAAAGACCCTAGTTTATAGATGCGGCGTAAAGGGTGGTTAAGGAAA

ACCTAAAATAATGCTAAAGACTCTCAAAGCTGTTATACGCTTCATGAGCACACGAAATCC

AAATACGAAAGTTGCCTTAATAAACTTTGCCTGACCCCACGAAAGCTGAAAAA

>35_Sample_ASV25

CACCGCGGTTATACGAAAGGCTCTAGTTTATAAATACGGCGTAAAGGGTGGTTAAGGAAA

AATTAAAATAATGCTAAAGACCCCCTAAGCAGTTATACGCTTCACGGAGGCACGAAACCC

AAATACGAAAGTTGCCTTAATAAACTTTACCTGACCCCACGAAAGCTGAGAAA

>35_Sample_ASV26

CACCGCGGTTATACGAAAGGCTCTAGTTTATAAATACGGCGTAAAGGGTGGTTAAGGAAA

AATTAAAATAATGCTAAAGACCCCCTAAGCAGTTATACGCTTCACGGAGGCACGAAACCC

AAATACGAAAGTTGCCTTAATAAACTTTACCTGACCCCACGAAAGCTGAAAAA

>35_Sample_ASV27

CACCGCGGTTATACGAGAGACCCTAGTTGATAGCTACGGCGTAAAGCGTGGTTAGGAGAA

CAAGATAAATAAAGTCAAACAATCTCTCGGCCGTTATACGTTATCTGAGAATATGAAGTC

CTACCACGAAAGTAACTTTAATTTTTCTGACCCCACGAAAACTGAGGAA

>35_Sample_ASV28

CACCGCGGTTAAACGAGAGGCCCTAGTTGATAGTACAACGGCGTAAAGGGTGGTTAAGGA

TAGTGAAATAATAAAGTCGAATGGCCCTTTGGCTGTCATACGCTTCTAGGAGTCCGAAGC

CCAATATACGAAAGTAACTTTAAGAGCGCCCACCTGACCCCACGAAAACTGAGAAA

>35_Sample_ASV29

CACCGCGGTTATACGAAAGACCCTAGTTGATAGGCACGGCGTAAAGGGTGGTTAGGGATA

TATTAAAAATAAAGTTAAAGAGCCTCTAAGCTGTCGCACGCATCCCGAGAGCTCGAGACC

CAGACACGAAAGTAACTTTAAAATACCTCACCTGACCCCACGAAAGCTAAGAAA

>35_Sample_ASV30

CACCGCGGTTATACGAGAGGCCCAAGTTGATACTCCACGGCGTAAAGAGTGGTTAGAAAA

GACCTACTACTAAAGCCGAACGCCTTCAAAGCTGTTATACGCATCCGAAGGTGAGAAGCC

CACCCACGAAAGTGGCTTTACAACCTTGACTCCACGAAAGCTGAAAAA

>35_Sample_ASV31

CACCGCGGTTATACGAGAGGCCCAAGTTGATACTCCACGGCGTAAAGAGTGGTTAGAAAA

GACCTACTACTAAAGCCGAACGCCTTCAAAGCTGTTATACGCATCCGAAGGTGAGAAGCC

CACCCACGAAAGTGGCTTTACAACCTTGACTCCACGAAAGCTATGACA

>35_Sample_ASV32

CACCGCGGTTATACGAGAGACCCCAGTTGATAAACACGGCGTAAAGAGTGGTTAAGGGCA

CAAAACAAATAAAGTCAAACAACCTCTTAGCTGTTATACGCATTATGAGGATATGAAGCC

CCCCTACGAAAGTAACTTTACCAACCCCTGACCCCACGAAAGCTAAGAAA

>35_Sample_ASV33

CACCGCGGTTATACGAGAGACCCTAGTTGATATATACGGCGTAAAGAGTGGTTTGGAATA

ACCAAGTAATATAGCCAAAGACCTCCCAAGCCGTCACACGCACCCCGGAGGCACGAAGCC

CAAACACGAAAGTAGCTTTATTAACTTACCGACGCCACGAAAGCTAAGAAA

>35_Sample_ASV34

CACCGCGGTTATACGAAAGACCCTAGTTTATAGGCACGGCGTAAAGGGTGGTTAGGGGAT

AAACAAAATAAAGCCAAAGACCTTCTAAGTCGTCATACACTCATGAAGGCACGAAGCCCA

AATACGAAAGTAACTTTACAACTTAGACCCGACTCCACGAAAGCTGGGGGA

>35_Sample_ASV35

CACCGCGGTTATACGAGAGGCCAAAGTTGACAGATATCGGCGTAAAGAGTGGTTAAGGAA

ATCTTAAAACTAAAGCCGAATGCCTTCAAGACTGTTATACGTTTCCGAAGGTAAGAAGCC

CCACTACGAAAGTGGCTTTACTCCACCTGACTCCACGAAAGCTGTGAAA

>35_Sample_ASV36

CACCGCGGTTATACGAGAGGCCCAAGTTGATAGACATCGGCGTAAAGAGTGGTTAAGGAA

GTCTCAAAACTAAAGCCGAATGCCTTCAAGACTGTTATACGTTTCCGAAGGTAAGAAGCC

CCACTACGAAAGTGGCTTTACTCCACCTGACTCCACGAAAGCTGTGAAA

>35_Sample_ASV37

CACCGCGGTTATACGAGAGGCCCAAGTTGATAGACATCGGCGTAAAGAGTGGTTAAGGAA

GTCTCAAAACTAAAGCCGAATGCCTTCAAGACTGTTATACGTTTCCGAAGGTAAGAAGCC

CCACTACGAAAGTGGCTTTATTCCACCTGACCCCACGAAAGCTGTGAAA

>35_Sample_ASV38

CACCGCGGTTATACGAGAGGCCCAAGTTGATAGACATCGGCGTAAAGAGTGGTTAAGGAA

GTCTCAAAACTAAAGCCGAATGCCTTCAAGACTGTTATACGTTTACGAAGGTAAGAAGCC

CCACTACGAAAGTGGCTTTATTCCACCTGACCCCACGAAAGCTGTGAAA

>35_Sample_ASV39

CACCGCGGTTATACGAGAGGCCCAAGTTGACAGATATCGGCGTAAAGAGTGGTTAAGGAA

ATCTTAAAACTAAAGCCGAATGCCTTCAAGACTGTTATACGTTTCCGAAGGTAAGAAGCC

CCACTACGAAAGTGGCTTTACTCCACCTGACTCCACGAAAGCTGTGAAA

>35_Sample_ASV40

CACCGCGGTTATACGAGAGGCCAAAGTTGACAGATATCGGCGTAAAGAGTGGTTAAGGAA

GTCTCAAAACTAAAGCCGAATGCCTTCAAGACTGTTATACGTTTCCGAAGGTAAGAAGCC

CCACTACGAAAGTGGCTTTACTCCACCTGACTCCACGAAAGCTGTGAAA

>35_Sample_ASV41

CACCGCGGTTATACGAGAGGCCAAAGTTGACAGATATCGGCGTAAAGAGTGGTTAAGGAA

ATCTCAAAACTAAAGCCGAATGCCTTCAAGACTGTTATACGTTTCCGAAGGTAAGAAGCC

CCACTACGAAAGTGGCTTTACTCCACCTGACTCCACGAAAGCTGTGAAA

>35_Sample_ASV42

CACCGCGGTTATACGAGAGACCCTAATTGATAGACTACGGCGTAAAGAGTGGTTTAGAAC

AAAAATTAATAAAGCCAAAGATCCTCCTGGCCGTCGCACGCATTTCGAGGATACGAAGCC

CCACTACGAAAGTAGCTTTACTACAACTTTTCTGACCCCACGAAAGCTAAGAAA

>35_Sample_ASV43

CACCGCGGTTATACGAAAGACCCTAGTTGATAGATCACGGCGTAAAGGGTGGTTAAGGAG

AACAAAAATAAAGCTAAAAATCCTCTAAGCTGTCATACGCTTTCCGAAGACATGAGACCC

AACCACGAAAGTAACTTTAAACTTTCCTGACACCACGAAAGCCAAGAAA

>36_Sample_ASV1

CACCGCGGTTATACGAAAGACCCTAGTTGATGGGTGCGGCGTAAAGGGTGGTTAGGGACT

ACAAAAAATAGAGCTAAAGATCCTCTAGGTCGTCATACACATCACGAGGAAACGAAGCCC

AAATACGAAAGTAGCTCTAAATATAATCCTGACGCCACGAAAGCCAAGAAA

>36_Sample_ASV2

CACCGCGGTTATACGAAAGACCCTAGTTGATAACTACGGCATAAAGGGTGGTTAGGGAGT

TTAAAAAATAAAGCCAAAGCTCTTCTAAGCTGTCATACGCTCTCTGAAAGAACGAGGCCC

AAACACGAAAGTAGCTTTAAATAAATAACCTGATACCACGAAAGCCAAGAAA

>36_Sample_ASV3

CACCGCGGTTATACGAGAGACCCTAGTTGATTAAAGCGGCGTAAAGAGTGGTTATGGAAC

TATTCTTTTAAAGCAGAAAACCTCTCAAACTGTTATACGCACCCAGAGGTCTGAATCCCT

CACACGAAAGTGACTTTATTTATGCCTACCAGAACCCACGAAAGCTGGGACA

>36_Sample_ASV4

CACCGCGGTTATACGAAAGACCCTAGTTGATAGCCACGGCGTAAAGGGTGGTTAAGGAAC

CTAAAAAATAAAGCTAAAGACCCTCTAGGCCGTCATACGCATTACGAGGCAAACGAAACC

CCAACACGAAAGTAGCTTTAGACAAATATCCTGATGCCACGAAAGCCAAGAAA

>36_Sample_ASV5

CACCGCGGTTATACGAGAGACCCTAATTAATAATTACGGCGTAAAGAGTGGTTAAAAGTT

AATATAAATAAAGCCAAAGATTTTCCAAGCTGTTATACGCACTCGAAAACATGAAGCCTT

ATAACGAAAGTAGCTTTACAAACCTTTTGACCCCACGAAAGCTATGATA

>36_Sample_ASV6

CACCGCGGTTATACGAGAGGCCCAAGTTGACAGACACCGGCGTAAAGAGTGGCTAGGGAA

AAATTACTACTAAAGCCGAACACCTTCAGAACTGTCATACGTTCCCGAAGATAAGAAGCC

CCACCACGAAAGTGACTTTATATTACCCGACCCCACGAAAGCTGTGAAA

>36_Sample_ASV7

CACCGCGGTTATACGAGAGGCCCAAGTTGACAGACACCGGCGTAAAGAGTGGCTAGGGAA

AAATTACTACTAAAGCCGAACACCCTCAGAACTGTCATACGTTCCCGAAGGTAAGAAGCC

CCACCACGAAAGTGGCTTTATAATACCCGACCCCACGAAAGCTGTGAAA

>36_Sample_ASV8

CACCGCGGTTATACGAGAGGCCCAAGTTGACAGACACCGGCGTAAAGAGTGGCTAGGGAA

AAATTACTACTAAAGCCGAACACCTTCAGAACTGTCATACGTTCCCGAAGATAAGAAGCC

CCACCACGAAAGTGGCTTTATATTACCCGACCCCACGAAAGCTAAGAAA

>36_Sample_ASV9

CACCGCGGTTATACGAGAGGCCCAAGTTGACAGACACCGGCGTAAAGAGTGGCTAGGGAA

AAATTACTACTAAAGCCGAACACCTTCAGAACTGTCATACGTTCCCGAAGATAAGAAGCC

CCACCACGAAAGTGGCTTTATATTACCCGACCCCACGAAAGCTGTGAAA

>36_Sample_ASV10

CACCGCGGTTATACGAGAGGCCCAAGTTGACAGACACCGGCGTAAAGAGTGGCTAAGGAA

AAATTTCTACTAAAGCCGAACACCTTCAGAACTGTCATACGTTTCCGAAGAAAAGAAGCC

CCACCACGAAAGTGGCTTTATATTACCCGACCCCACGAAAGCTGTGAAA

>36_Sample_ASV11

CACCGCGGTTATACGAGAGGCCCAAGTTGACAGACACCGGCGTAAAGAGTGGCTAAGGAA

AAATTTCTACTAAAGCCGAACACCTTCAGAACTGTCATACGTTCCCGAAGAAAAGAAGCC

CCACCACGAAAGTGGCTTTATATTACCCGACCCCACGAAAGCTGTGAAA

>36_Sample_ASV12

CACCGCGGTTATACGAGAGGCCCAAGTTGATAAGCCCGGCGTAAAGAGTGGTTATGGAGA

ATAAAACACTAAAGCTAAAGACCCCCTAGGCTGTTATACGCACCTGGCGGCTCGAACCAC

CTATACGAAAGTAGCTTTACCCCCCTTCCACCAGAATCCACGAAAGCTAAGAAA

>36_Sample_ASV13

CACCGCGGTTATACGAGAGACCCTAATTGATAGGCACGGCGTAAAGAGTGGTTATGGAGA

ATAAAACACTAAAGCTAAAGACCCCCTAGGCTGTTATACGCACCTGGCGGCTCGAACCAC

CTATACGAAAGTAGCTTTACCCCCCTTCCACCAGAATCCACGACAGCTGGGGCA

>36_Sample_ASV14

CACCGCGGTTATACGAGAGGCCCAAGTTGATAAGCCCGGCGTAAAGAGTGGTTATGGAGA

ATAAAACACTAAAGCTAAAGACCCCCTAGGCTGTTATACGCACCTGGCGGCTCGAACCAC

CTATACGAAAGTAGCTTTACCCCCCTTCCACCAGAATCCACGACAGCTGGGGCA

>36_Sample_ASV15

CACCGCGGTTATACGAGAGGCCCAAGTTGACAGACATCGGCGTAAAGAGTGGTTAAGGAA

ACCCCATTACTAAAGCCGAACGCCCTCAGAACTGTCATACGTTCCCGAAGGTAAGAAGCC

CCACTACGAAAGTGGCTTTAACCACACCCGACCCCACGAAAGCTGTGAAA

>36_Sample_ASV16

CACCGCGGTTATACGAGAGGCCCCAGTTGATATTAGCGGCGTAAAGGGTGGTTAAGGACT

TTAATAACTAAAGCTAAACACTTCCCCGGCTGTTGTACGTAGACCGGAAGCTTGAAGCCC

AGACACGAAAGTAGCTTTATTCCCCCTGACCCCACGAAAGCTGAGAAA

>36_Sample_ASV17

CACCGCGGTTATACGAGAGACCCCAGTTGATAGCTACGGCGTAAAGAGTGGTTAAGGACC

CCATTTAATAAAGCCAAAGACCCCCCAAGCCGTCATACGCACACGAGGGCGCGAAGCTCG

CACACGAAAGTAGCTCTAATTTACCCGACGCCACGAAAGCTAAGGAA

>36_Sample_ASV18

CACCGCGGTTATACGAGAAACCCTAGTTGACAAATACGGCGTAAAGAGTGGTTAAGGGGT

ATCAAAAATAAAGTCAAACATTTTCCTAGTTGTTATAAGCATTTCGAGAACATGAAGCCC

AACCACGAAAGTAACTTTACCTTCACCTGACCCCACGAAAGCTAAGAAA

>36_Sample_ASV19

CACCGCGGTTATACGAGAGACCCTAGTTGATAAATACGGCGTAAAGAGTGGTTATGGGAA

AAACAAATAAAGTCAAACAACCTCTTAGCTGTTATACGCATTATGAGAGCATGAAGCCCC

CTCACGAAAGTAACTTTAATACCTCCTGACCCCACGAAAGCTAAGAAA

>36_Sample_ASV20

CACCGCGGTTATACGAGAGACCCTAATTGATAGACACGGCGTAAAGAGTGGTTTGGGAAA

GCCCAAAAATAAAGCTAAAGACCTTCTAAGCCGTCACACGCACGCCGAAGGCACGAAGCC

CTGACACGAAAGTAGCTTTACTATTACCCCCGACGCCACGAAAGCTAAGAAA

>36_Sample_ASV21

CACCGCGGTTATACGAGAGACCCTAATTGATAGCTCACGGCGTAAAGGGTGGTTTGGGGA

AACCTCAAAATAAAGCTAAAGACCTTCTAAGCCGTTACACGCATACCGAAGGCACGAGGC

CCTAACACGAAAGTAGCTTTACTATTACCCCTGACGCCACGAAAGCTAAGAAA

>36_Sample_ASV22

AACCGCGGTTATACGAGAGACCCTAATTGATAGCTCACGGCGTAAAGGGTGGTTTGGGGA

AACCTCAAAATAAAGCTAAAGACCTTCTAAGCCGTTACACGCATACCGAAGGCACGAGGC

CCTAACACGAAAGTAGCTTTACTATTACCCCTGACGCCACGAAAGCTAAGAAA

>36_Sample_ASV23

CACCGCGGTTATACGAAAGACCCTAGTTTATAGATGCGGCGTAAAGGGTGGTTAAGGAAA

ACCTAAAATAATGCTAAAGACTCTCAAAGCTGTTATACGCTTCATGAGCACACGAAATCC

AAATACGAAAGTTGCCTTAATAAACTTTGCCTGACCCCACGAAAGCTGAAAAA

>36_Sample_ASV24

CACCGCGGTTATACGAAAGACCCTAGTTTATAGATGCGGCGTAAAGGGTGGTTAAGGAAA

ACCTAAAATAATGCTAAAGACTCTCAAAGCTGTTATACGCTTCATGAGCACACGAAATCC

AAATACGAAAGTTGCCTTAATAAACTTTGCCTGACCCCACGAAAGCTAAGAAA

>36_Sample_ASV25

CACCGCGGTTATACGAGAGACCCTAGTTGATAGCTACGGCGTAAAGCGTGGTTAGGAGAA

CAAGATAAATAAAGTCAAACAATCTCTCGGCCGTTATACGTTATCTGAGAATATGAAGTC

CTACCACGAAAGTAACTTTAATTTTTCTGACCCCACGAAAACTGAGGAA

>36_Sample_ASV26

CACCGCGGTTAAACGAGAGGCCCTAGTTGATAGTACAACGGCGTAAAGGGTGGTTAAGGA

TAGTGAAATAATAAAGTCGAATGGCCCTTTGGCTGTCATACGCTTCTAGGAGTCCGAAGC

CCAATATACGAAAGTAACTTTAAGAGCGCCCACCTGACCCCACGAAAACTGAGAAA

>36_Sample_ASV27

CACCGCGGTTATACGAAAGACCCTAGTTTATAGACACGGCGTAAAGGGTGGTTCGGGACT

AAACAAAATAAAGCCAAAGACCTCCTAAGCCGTCATACGCTCACGGAAGCACGAAGCCCA

AGTACGAAAGTAGCTTTACAAAACATGCCTGACTCCACGAAAGCTAGGAAA

>36_Sample_ASV28

CACCGCGGTTATACGAAAGACCCTAGTTGATAGGCACGGCGTAAAGGGTGGTTAGGGATA

TATTAAAAATAAAGTTAAAGAGCCTCTAAGCTGTCGCACGCATCCCGAGAGCTCGAGACC

CAGACACGAAAGTAACTTTAAAATACCTCACCTGACCCCACGAAAGCTAAGAAA

>36_Sample_ASV29

CACCGCGGTTATACGAGAGGCCCAAGTTGATACTCCACGGCGTAAAGAGTGGTTAGAAAA

GACCTACTACTAAAGCCGAACGCCTTCAAAGCTGTTATACGCATCCGAAGGTGAGAAGCC

CACCCACGAAAGTGGCTTTACAACCTTGACTCCACGAAAGCTATGACA

>36_Sample_ASV30

CACCGCGGTTATACGAGAGGCCCAAGTTGATACTCCACGGCGTAAAGAGTGGTTAGAAAA

GACCTACTACTAAAGCCGAACGCCTTCAAAGCTGTTATACGCATCCGAAGGTGAGAAGCC

CACCCACGAAAGTGGCTTTACAACCTTGACTCCACGAAAGCTAAGAAA

>36_Sample_ASV31

CACCGCGGTTATACGAGAGACCCCAGTTGATAAACACGGCGTAAAGAGTGGTTAAGGGCA

CAAAACAAATAAAGTCAAACAACCTCTTAGCTGTTATACGCATTATGAGGATATGAAGCC

CCCCTACGAAAGTAACTTTACCAACCCCTGACCCCACGAAAGCTAAGAAA

>36_Sample_ASV32

CACCGCGGTTATACGAGAGACCCTAGTTGATATATACGGCGTAAAGAGTGGTTTGGAATA

ACCAAGTAATATAGCCAAAGACCTCCCAAGCCGTCACACGCACCCCGGAGGCACGAAGCC

CAAACACGAAAGTAGCTTTATTAACTTACCGACGCCACGAAAGCTAAGAAA

>36_Sample_ASV33

CACCGCGGTTATACGAAAGACCCTAGTTTATAGGCACGGCGTAAAGGGTGGTTAGGGGAT

AAACAAAATAAAGCCAAAGACCTTCTAAGTCGTCATACACTCATGAAGGCACGAAGCCCA

AATACGAAAGTAACTTTACAACTTAGACCCGACTCCACGAAAGCTGGGGGA

>36_Sample_ASV34

CACCGCGGTTATACGAAAGACCCTAGTTGATAGACCACGGCGTAAAGGGTGGTTAAGGAA

CACAAAAATAAAGCTAAAGATCCTCTAAGCTGTCATACGCTTCCCGAAGACATGAAACCC

AATCACGAAAGTAGCTTTAAACTGTCCTGACACCACGAAAGCCAAGAAA

>36_Sample_ASV35

CACCGCGGTTATACGAGAGACCCTAATTGATAGACTACGGCGTAAAGAGTGGTTTAGAAC

AAAAATTAATAAAGCCAAAGATCCTCCTGGCCGTCGCACGCATTTCGAGGATACGAAGCC

CCACTACGAAAGTAGCTTTACTACAACTTTTCTGACCCCACGAAAGCTAAGAAA

>36_Sample_ASV36

CACCGCGGTTATACGAAAGACCCTAGTTGATAGATCACGGCGTAAAGGGTGGTTAAGGAG

AACAAAAATAAAGCTAAAAATCCTCTAAGCTGTCATACGCTTTCCGAAGACATGAGACCC

AACCACGAAAGTAACTTTAAACTTTCCTGACACCACGAAAGCCAAGAAA

>38_Sample_ASV1

CACCGCGGTTATACGAAAGACCCTAGTTGATGGGTGCGGCGTAAAGGGTGGTTAGGGACT

ACAAAAAATAGAGCTAAAGATCCTCTAGGTCGTCATACACATCACGAGGAAACGAAGCCC

AAATACGAAAGTAGCTCTAAATATAATCCTGACGCCACGAAAGCCAAGAAA

>38_Sample_ASV2

CACCGCGGTTATACGAAAGACCCTAGTTGATGGGTGCGGCGTAAAGGGTGGTTAGGGACT

ACAAAAAATAGAGCTAAAGATCCTCTAGGTCGTCATACACATCACGAGAAAACGAAGCCC

AAATACGAAAGTAGCTCTAAATATAATCCTGACGCCACGAAAGCCAAGAAA

>38_Sample_ASV3

CACCGCGGTTATACGAGAGACCCTAGTTGATTAAAGCGGCGTAAAGAGTGGTTATGGAAC

TATTCTTTTAAAGCAGAAAACCTCTCAAACTGTTATACGCACCCAGAGGTCTGAATCCCT

CACACGAAAGTGACTTTATTTATGCCTACCAGAACCCACGAGAGCTGGGACA

>38_Sample_ASV4

CACCGCGGTTATACGAGAGGCCCAAGTTGACAGACATCGGCGTAAAGAGTGGTTAAGGAA

AAATTACTGCTAAAGCCGAACACCCTCAGAACTGTCATACGTTCCCGAAGATAAGAAGCC

CCACCACGAAAGTGGCTTTATATTACCCGACCCCACGAAAGCTGTGAAA

>38_Sample_ASV5

CACCGCGGTTATACGAGAGGCCCAAGTTGACAGACACCGGCGTAAAGAGTGGCTAGGGAA

AAATTACTACTAAAGCCGAACACCTTCAGAACTGTCATACGTTCCCGAAGATAAGAAGCC

CCACCACGAAAGTGGCTTTATATTACCCGACCCCACGAAAGCTAAGAAA

>38_Sample_ASV6

CACCGCGGTTATACGAGAGGCCCAAGTTGACAGACACCGGCGTAAAGAGTGGCTAGGGAA

AAATTACTACTAAAGCCGAACACCTTCAGAACTGTCATACGTTCCCGAAGATAAGAAGCC

CCACCACGAAAGTGGCTTTATATTACCCGACCCCACGAAAGCTGTGAAA

>38_Sample_ASV7

CACCGCGGTTATACGAGAGGCCCAAGTTGACAGACACCGGCGTAAAGAGTGGCTAAGGAA

AAATTTCTACTAAAGCCGAACACCTTCAGAACTGTCATACGTTCCCGAAGAAAAGAAGCC

CCACCACGAAAGTGGCTTTATATTACCCGACCCCACGAAAGCTAAGAAA

>38_Sample_ASV8

CACCGCGGTTATACGAGAGGCCCAAGTTGACAGACACCGGCGTAAAGAGTGGCTAAGGAA

AAATTTCTACTAAAGCCGAACACCTTCAGAACTGTCATACGTTCCCGAAGAAAAGAAGCC

CCACCACGAAAGTGGCTTTATATTACCCGACCCCACGAAAGCTGTGAAA

>38_Sample_ASV9

CACCGCGGTTATACGAGAGGCCCAAGTTGATAAGCCCGGCGTAAAGAGTGGTTATGGAGA

ATAAAACACTAAAGCTAAAGACCCCCTAGGCTGTTATACGCACCTGGCGGCTCGAACCAC

CTATACGAAAGTAGCTTTACCCCCCTTCCACCAGAATCCACGAAAGCTAAGAAA

>38_Sample_ASV10

CACCGCGGTTATACGAGAGACCCCAGTTGATAGCTACGGCGTAAAGAGTGGTTATGGAGA

ATAAAACACTAAAGCTAAAGACCCCCTAGGCTGTTATACGCACCTGGCGGCTCGAACCAC

CTATACGAAAGTAGCTTTACCCCCCTTCCACCAGAATCCACGACAGCTGGGGCA

>38_Sample_ASV11

CACCGCGGTTATACGAGAGGCCCAAGTTGATAAGCCCGGCGTAAAGAGTGGTTATGGAGA

ATAAAACACTAAAGCTAAAGACCCCCTAGGCTGTTATACGCACCTGGCGGCTCGAACCAC

CTATACGAAAGTAGCTTTACCCCCCTTCCACCAGAATCCACGACAGCTGGGGCA

>38_Sample_ASV12

CACCGCGGTTATACGAGAGGCCCTAGTTGATAGCCACGGCGCAAAGAGTGGTTAAGGAGC

CCCACTAAATAAAGTCGAACACTTCCTAGGCCGTTATACGTTTTCTAGAAGCACGAAACC

CAATTACACGAAAGCAACTTTATACTAAACAACCTGACCCCACGAAAGCTAAGAAA

>38_Sample_ASV13

CACCGCGGTTATACGAGAGGCCCAAGTTGACAGACATCGGCGTAAAGAGTGGTTAAGGAA

ACCCCATTACTAAAGCCGAACGCCCTCAGAACTGTCATACGTTCCCGAAGGTAAGAAGCC

CCACTACGAAAGTGGCTTTAACCACACCCGACCCCACGAAAGCTAAGAAA

>38_Sample_ASV14

CACCGCGGTTATACGAGAGGCCCAAGTTGACAGACATCGGCGTAAAGAGTGGTTAAGGAA

ACCCCATTACTAAAGCCGAACGCCCTCAGAACTGTCATACGTTCCCGAAGGTAAGAAGCC

CCACTACGAAAGTGGCTTTAACCACACCCGACCCCACGAAAGCTGTGAAA

>38_Sample_ASV15

CACCGCGGTTATACGAGAGGCCCAAGTTGACAGACACCGGCGTAAAGAGTGGTTAAGGAA

ATTCATCAACTAAAGCCGAATGCCCTCAGGACTGTTATACGTTTGCGAAGGTAAGAAGCC

CTACTACGAAAGTGGCTTTATTCCACCTGATTCCACGAAAGCTGCGGAA

>38_Sample_ASV16

CACCGCGGTTATACGAGAGGCCCCAGTTGATATTAGCGGCGTAAAGGGTGGTTAAGGACT

TTAATAACTAAAGCTAAACACTTCCCCGGCTGTTGTACGTAGACCGGAAGCTTGAAGCCC

AGACACGAAAGTAGCTTTATTCCCCCTGACCCCACGAAAGCTGAGAAA

>38_Sample_ASV17

CACCGCGGTTATACGAGAAACCCTAGTTGACAAATACGGCGTAAAGAGTGGTTAAGGGGT

ATCAAAAATAAAGTCAAACATTTTCCTAGTTGTTATAAGCATTTCGAGAACATGAAGCCC

AACCACGAAAGTAACTTTACCTTCACCTGACCCCACGAAAGCTAAGAAA

>38_Sample_ASV18

CACCGCGGTTATACGAAAGACCCCAGTTTATAGACACGGCGTAAAGGGTGGTTATAGGAC

AAACTAACATAGAGCCAAAGACCCCCTAAGCCGTCATACGCTCACGAGGGCTCGAAGCCC

CTATACGAAAGTAGCTCTACCAAAAAATGCTCGACTCCACGAAAGCTGGGACA

>38_Sample_ASV19

CACCGCGGTTATACGAGAGACCCTAATTGATAGGTACGGCGTAAAGAGTGGTTTGGGATA

ACCCAAAAATAAAGCTAAAGACCTTCTAAGCCGTCACACGCATACCGAAGGCACGAGGCC

CCAACACGAAAGTAGCTTTACTATTACCCCGACGCCACGAAAGCTAAGAAA

>38_Sample_ASV20

CACCGCGGTTATACGAGAGACCCTAATTGATAGGCACGGCGTAAAGAGTGGTTTAGGGTA

ACCCATAAATAAAGCTAAAGACCTTCTAAGCCGTCACACGCACACCGAAGGCACGAGACC

CCAACACGAAAGTAGCTTTACTTCTACCCCTGACGCCACGAAAGCTAAGAAA

>38_Sample_ASV21

CACCGCGGTTATACGAAAGACCCTAGTTGATGGATCACGGCGTAAAGGGTGGTTAAGGGA

TATAAAAATAAAGCTAAAAATCCTCTTAGCTGTCATACGCTTTCCGAAGACATGAGGCCC

AATTACGAAAGTAGCTTTAAACCACCCTGACACCACGAAAGCCAAGAAA

>38_Sample_ASV22

CACCGCGGTCATACGATTAACCCAAGTTAATAGGCACACGGCGTAAAGCGTGTTTAAGCA

CTACGCCAAATAAAGTCAAACTCCAATTAAGCTGTAAAAAGCCATAATCAAGACGAAAAT

AAATAACGAAAGTAACTTTACAACCGCTGAAACACGATAGCTAAGATC

>38_Sample_ASV23

CACCGCGGTTATACGAGAGGCCCTAGTTGATAACTACCGGCGTAAAGAGTGGTTATGGAA

AAATATTTAATAAAGCCGAACACCCCCTCAGCCGTCATACGCACCTGGGAGCACGAAGAC

CTACTGCGAAAGCAGCTTTAATTATGCCTGACCCCACGACAGCTAAGAAA

>38_Sample_ASV24

CACCGCGGTTATACGAGAGGCCCAAGTTGATTATTACCGGCGTAAAGCGTGGTTAAGATT

TTTATAGGACTAAAGCCGAACCCCCTCAGAGCTGTTATACGCACCCGAAGGTTAGAAGTT

CAATCACGAAAGTGGCTTTACAACCCCGAACCCACGAAAGCTAAGATA

>38_Sample_ASV25

CACCGCGGTTATACGAGAGACCCTAATTGATAGGCGCGGCGTAAAGAGTGGTTTAGGACT

AAAACTAATAAAGCTAAAGATCCTCCCAGCCGTCGCACGCATTCTGAGGACACGAAACTC

AACCACGAAAGTAGCTTTAACATACTCTCCTGACCCCACGAAAGCTAAGAAA

>38_Sample_ASV26

CACCGCGGTTATACGAAAGACCCTAGTTGATAGGCACGGCGTAAAGGGTGGTTAGGGATA

TATCAAAAATAAAGTTAAAGAGCCTCTAAGCTGTCGCACGCATCCCGAGAGCTCGAGACC

CAGACACGAAAGTAACTTTAAAATACCTCACCTGACCCCACGAAAGCTAAGAAA

>38_Sample_ASV27

CACCGCGGTTATACGAAAGACCCTAGTTGATAACCACGGCGTAAAGGGTGGTTAAGGTAT

ACAACAAATAAAGCTAAATAGCCTCTAGGCCGTCGCACGCATTCCGAGAGCTCGAAACCC

AAAAACGAAAGTAGCTTTAAAATAAACAGACCTGACCCCACGAAAGCTAAGAAA

>38_Sample_ASV28

CACCGCGGTTATACGAGAGACCCCAGTTGATAGCCACGGCGTAAAGGGTGGTTAAGGTAT

ACAACAAATAAAGCTAAATAGCCTCTAGGCCGTCGCACGCATTCCGAGAGCTCGAAACCC

AAAAACGAAAGTAGCTTTAAAATAAACAGACCTGACCCCACGAAAGCTAAGAAA

>38_Sample_ASV29

CACCGCGGTTATACGAAAGACCCTAGTTGATAGCCACGGCGTAAAGGGTGGTTAAGGTAT

ACAACAAATAAAGCTAAATAGCCTCTAGGCCGTCGCACGCATTCCGAGAGCTCGAAACCC

AAAAACGAAAGTAGCTTTAAAATAAACAGACCTGACCCCACGAAAGCTAAGAAA

>38_Sample_ASV30

CACCGCGGTTATACGAGAGGCCCAAGTTGATACTCCACGGCGTAAAGAGTGGTTAGAAAA

GACCTACTACTAAAGCCGAACGCCTTCAAAGCTGTTATACGCATCCGAAGGTGAGAAGCC

CACCCACGAAAGTGGCTTTACAACCTTGACTCCACGAAAGCTATGACA

>38_Sample_ASV31

CACCGCGGTTATACGAGAGGCCCAAGTTGATACTCCACGGCGTAAAGAGTGGTTAGAAAA

GACCTACTACTAAAGCCGAACGCCTTCAAAGCTGTTATACGCATCCGAAGGTGAGAAGCC

CACCCACGAAAGTGGCTTTACAACCTTGACTCCACGAAAGCTAAGAAA

>38_Sample_ASV32

CACCGCGGTTATACGAGAGACCCTAGTTGATATATACGGCGTAAAGAGTGGTTTGGAATA

ACCAAGTAATATAGCCAAAGACCTCCCAAGCCGTCACACGCACCCCGGAGGCACGAAGCC

CAAACACGAAAGTAGCTTTATTAACTTACCGACGCCACGAAAGCTAAGAAA

>38_Sample_ASV33

CACCGCGGTTATACGAGAGACCCCAGTTGATATATACGGCGTAAAGAGTGGTTTGGAATA

ACCAAGTAATATAGCCAAAGACCTCCCAAGCCGTCACACGCACCCCGGAGGCACGAAGCC

CAAACACGAAAGTAGCTTTATTAACTTACCGACGCCACGAAAGCTAAGAAA

>38_Sample_ASV34

CACCGCGGTTATACGAGAGACCCCAGTTGATAGCTACGGCGTAAAGAGTGGTTTGGAATA

ACCAAGTAATATAGCCAAAGACCTCCCAAGCCGTCACACGCACCCCGGAGGCACGAAGCC

CAAACACGAAAGTAGCTTTATTAACTTACCGACGCCACGAAAGCTAAGAAA

>38_Sample_ASV35

CACCGCGGTTATACGAAAGACCCTAGTTTATAGGCACGGCGTAAAGGGTGGTTAGGGGAT

AAACAAAATAAAGCCAAAGACCTTCTAAGTCGTCATACACTCATGAAGGCACGAAGCCCA

AATACGAAAGTAACTTTACAACTTAGACCCGACTCCACGAAAGCTGGGGGA

>38_Sample_ASV36

CACCGCGGTTATACGAGAGACCCTAATTAATAGCTACGGCGTAAAGAGTGGTTTAGGACA

AAAATTAATAAAGCCGAAGTTTCTCCTAGCCGTTGTACGCACTCTGAGAACACGAAGCCC

CACTACGAAAGTAGCTTTACCTATTATTTCCTGACCCCACGAAAGCTAAGAAA

>38_Sample_ASV37

CACCGCGGTTATACGAGAGACCCTAATTAATAGATACGGCGTAAAGAGTGGTTTAGGACA

AAAATTAATAAAGCCGAAGTTTCTCCTAGCCGTTGTACGCACTCTGAGAACACGAAGCCC

CACTACGAAAGTAGCTTTACCTATTATTTCCTGACCCCACGAAAGCTAAGAAA

>38_Sample_ASV38

CACCGCGGTTATACGAGAGACCCTAATTAATAGCTACGGCGTAAAGAGTGGTTTAGGGTA

AAAATTAATAAAGCCGAAGTTTCTCCTAGCCGTTGTACGCACTCTGAGAACACGAAGCCC

CACTACGAAAGTAGCTTTACCTATTATTTCCTGACCCCACGAAAGCTAAGAAA

>38_Sample_ASV39

CACCGCGGTTATACGAAAGACCCTAGTTGATAGACCACGGCGTAAAGGGTGGTTAAGGAA

CACAAAAATAAAGCTAAAGATCCTCTAAGCTGTCATACGCTTCCCGAAGACATGAAACCC

AATCACGAAAGTAGCTTTAAACTGTCCTGACACCACGAAAGCCAAGAAA

>38_Sample_ASV40

CACCGCGGTTATACGAAAGACCCTAGTTGATAGATCACGGCGTAAAGGGTGGTTAAGGAA

AACAAAAATAAAGCTAAAAATCCTCTAAGCTGTCATACGCTTTCCGAAGACATGAGATCC

AACCACGAAAGTAGCTTTAAAATTTCCTGACACCACGAAAGCCAAGGAA

>38_Sample_ASV41

CACCGCGGTTATACGAAAGACCCTAGTTGATAGATCACGGCGTAAAGGGTGGTTAAGGAG

AACAAAAATAAAGCTAAAAATCTTCTAAGCTGTCATACGCTTTCCGAAGACATGAGACCC

AACCACGAAAGTAGCTTTAAACTTTCCTGACACCACGAAAGCTAAGAAA

>38_Sample_ASV42

CACCGCGGTTATACGAAAGACCCTAGTTGATAGATCACGGCGTAAAGGGTGGTTAAGGAG

AACAAAAATAAAGCTAAAAATCTTCTAAGCTGTCATACGCTTTCCGAAGACATGAGACCC

AACCACGAAAGTAGCTTTAAACTTTCCTGACACCACGAAAGCCAAGAAA

>40_Sample_ASV1

CACCGCGGTTATACGAGAGGCCCAAGTTGACAGACATCGGCGTAAAGAGTGGTTAAGGAA

AACCTTCAACTAAAGTCGAACGCTCTCAAGACTGTCGTACGTTTCCGAGAGTATGAAGCC

CCACCACGAAAGTGACTTTACCTCACCTGACTCCACGAAAGCTGCGAAA

>40_Sample_ASV2

CACCGCGGTTATACGAAAGACCCTAGTTGATGGGTGCGGCGTAAAGGGTGGTTAGGGACT

ACAAAAAATAGAGCTAAAGATCCTCTAGGTCGTCATACACATCACGAGGAAACGAAGCCC

AAGTACGAAAGTAGCTCTAAATATAATCCTGACGCCACGAAAGCCAAGAAA

>40_Sample_ASV3

CACCGCGGTTATACGAAAGACCCTAGTTGATGGGTGCGGCGTAAAGGGTGGTTAGGGACT

ACAAAAAATAGAGCTAAAGATCCTCTAGGTCGTCATACACATCACGAGGAAACGAAGCCC

AAATACGAAAGTAGCTCTAAATATAATCCTGACGCCACGAAAGCCAAGAAA

>40_Sample_ASV4

CACCGCGGTTATACGAAAGACCCTAGTTGATAACTACGGCATAAAGGGTGGTTAGGGAGT

TTAAAAAATAAAGCCAAAGCTCTTCTAAGCTGTCATACGCTCTCTGAAAGAACGAGGCCC

AAACACGAAAGTAGCTTTAAATAAATAACCTGATACCACGAAAGCCAAGAAA

>40_Sample_ASV5

CACCGCGGTTATACGAGAGACCCTAGTTGATTAAAGCGGCGTAAAGAGTGGTTATGGAAC

TATTCTTTTAAAGCAGAAAACCTCTCAAACTGTTATACGCACCCAGAGGTCTGAATCCCT

CACACGAAAGTGACTTTATTTATGCCTACCAGAACCCACGAAAGCTGGGACA

>40_Sample_ASV6

CACCGCGGTTATACGAAAGACCCTAGTTTATAGGTACGGCGTAAAGGGTGGTTAGGGGAC

AAACAAAATAAAGCCAAAGACCTCCTAAGCCGTCATACGCTCACGGAAGCACGAAGCCCA

AACACGAAAGTAGCTTTACCGAACATGCCCGACTCCACGAAAGCTGGGGAA

>40_Sample_ASV7

CACCGCGGTTATACGAGAGACCCTAATTAATAATTACGGCGTAAAGAGTGGTTAAAAGTT

AATATAAATAAAGCCAAAGATTTTCCAAGCTGTTATACGCACTCGAAAACATGAAGCCTT

ATAACGAAAGTAGCTTTACAAACCTTTTGACCCCACGAAAGCTATGATA

>40_Sample_ASV8

CACCGCGGTTATACGAGAGGCCCAAGTTGACAGACACCGGCGTAAAGAGTGGCTAGGGAA

AAATTACTACTAAAGCCGAACACCTTCAGAACTGTCATACGTTCCCGAAGATAAGAAGCC

CCACCACGAAAGTGACTTTATATTACCCGACCCCACGAAAGCTGTGAAA

>40_Sample_ASV9

CACCGCGGTTATACGAGAGGCCCAAGTTGACAGACACCGGCGTAAAGAGTGGCTAGGGAA

AAATTACTACTAAAGCCGAACACCTTCAGAACTGTCATACGTTCCCGAAGATAAGAAGCC

CCACCACGAAAGTGGCTTTATATTACCCGACCCCACGAAAGCTGTGAAA

>40_Sample_ASV10

CACCGCGGTTAAACGAGAGACCCTAGTTGATCATCACGGCGTAAAGAGTGGTTAGGGGAT

TATCATAAATAAAGCCAAAGACCTCCCAAGCTGTCGCACGCATTCCGGAGGCGCGAAGCC

CACACACGAAAGTAGCTTTAACTATTGACCCTGATGCCACGAAAGCTAAGAAA

>40_Sample_ASV11

CACCGCGGTTATACGAGAGGCCCAAGTTGATAAGCCCGGCGTAAAGAGTGGTTATGGAGA

ATAAAACACTAAAGCTAAAGACCCCCTAGGCTGTTATACGCACCTGGCGGCTCGAACCAC

CTATACGAAAGTAGCTTTACCCCCCTTCCACCAGAATCCACGACAGCTGGGGCA

>40_Sample_ASV12

CACCGCGGTTATACGAGAGGCCCAAGTTGACAGACATCGGCGTAAAGAGTGGTTAAGGAA

ACCCCATTACTAAAGCCGAACGCCCTCAGAACTGTCATACGTTCCCGAAGGTAAGAAGCC

CCACTACGAAAGTGGCTTTAACCACACCCGACCCCACGAAAGCTGTGAAA

>40_Sample_ASV13

CACCGCGGTTATACGAGAGGCCCAAGTTGATAGATATCGGCGTAAAGAGTGGTTAAGGAA

ACCCCGTTACTAAAGCCGAACGCCCTCAGAACTGTCATACGTTCCCGAAGGTAAGAAGCC

CCACTACGAAAGTGGCTTTAACCACACCCGACCCCACGAAAGCTGTGAAA

>40_Sample_ASV14

CACCGCGGTTATACGAGAGGCCCCAGTTGATATTAGCGGCGTAAAGGGTGGTTAAGGACT

TTAATAACTAAAGCTAAACACTTCCCCGGCTGTTGTACGTAGACCGGAAGCTTGAAGCCC

AGACACGAAAGTAGCTTTATTCCCCCTGACCCCACGAAAGCTGAGAAA

>40_Sample_ASV15

CACCGCGGTTATACGAGAGGCCCTAGTTGATAAATACGGCGTAAAGAGTGGTTAGAGGAC

TCCCATAATAAAGCCAAAGACCCCCCGGGCCGTCACACGCATACGAGGGCACGAAGTTCA

CACACGAAAGTAGCTTTAAATTACCCGACGCCACGAAAGCTAAGAAA

>40_Sample_ASV16

GACCGCGGTTATACGAGAGACTCTAGTTGATAGATACGGCGTAAAGAGTGGTTTGGGATA

AACTATAAATAAAGCCAAAGACCTTCCAAGCTGTTAAACGCATACCGAAGGCACGAAGCC

CCAACACGAAAGTAGCTTTACTACTACCCCCGATGCCACGAAAGCTAAGACA

>40_Sample_ASV17

CACCGCGGTCATACGATTAACCCAAGTTAATAGGCACACGGCGTAAAGCGTGTTTAAGCA

CTATGCCAAATAAAGTTAAACTCCAATTAAGCTGTAAAAAGCCATAATCAAGACGAAAAT

AAATAACGAAAGTAACTTTACAACCGCTGAAACACGATAGCTAAGATC

>40_Sample_ASV18

CACCGCGGTTATACGAAAGACCCTAGTTTATAGACACGGCGTAAAGGGTGGTTCGGGACT

AAACAAAATAAAGCCAAAGACCTCCTAAGCCGTCATACGCTCACGGAAGCACGAAGCCCA

AGTACGAAAGTAGCTTTACAAAACATGCCTGACTCCACGAAAGCTAGGAAA

>40_Sample_ASV19

CACCGCGGTTATACGAAAGACCCTAGTTGATAGGCACGGCGTAAAGGGTGGTTAGGGATA

TATCAAAAATAAAGTTAAAGAGCCTCTAAGCTGTCGCACGCATCCCGAGAGCTCGAGACC

CAGACACGAAAGTAACTTTAAAATACCTCACCTGACCCCACGAAAGCTAAGAAA

>40_Sample_ASV20

CACCGCGGTTATACGAAAGACCCTAGTTGATAGCCACGGCGTAAAGGGTGGTTAAGGTAT

ACAACAAATAAAGCTAAATAGCCTCTAGGCCGTCGCACGCATTCCGAGAGCTCGAAACCC

AAAAACGAAAGTAGCTTTAAAATAAACAGACCTGACCCCACGAAAGCTAAGAAA

>40_Sample_ASV21

CACCGCGGTTATACGAGAGGCCCAAGTTGATACTCCACGGCGTAAAGAGTGGTTAGAAAA

GACCTACTACTAAAGCCGAACGCCTTCAAAGCTGTTATACGCATCCGAAGGTGAGAAGCC

CACCCACGAAAGTGGCTTTACAACCTTGACTCCACGAAAGCTATGACA

>40_Sample_ASV22

CACCGCGGTTATACGAGAGGCCCAAGTTGATACTCCACGGCGTAAAGAGTGGTTAGAAAA

GACCTACTACTAAAGCCGAACGCCTTCAAAGCTGTTATACGCATCCGAAGGTGAGAAGCC

CACCCACGAAAGTGGCTTTACAACCTTGACTCCACGAAAGCTGGGACA

>40_Sample_ASV23

CACCGCGGTTATACGAGAGACCCCAGTTGATAAACACGGCGTAAAGAGTGGTTAAGGGCA

CAAAACAAATAAAGTCAAACAACCTCTTAGCTGTTATACGCATTATGAGGATATGAAGCC

CCCCTACGAAAGTAACTTTACCAACCCCTGACCCCACGAAAGCTAAGAAA

>40_Sample_ASV24

CACCGCGGTTATACGAAAGACCCTAGTTTATAGGCACGGCGTAAAGGGTGGTTAGGGGAT

AAACAAAATAAAGCCAAAGACCTTCTAAGTCGTCATACACTCATGAAGGCACGAAGCCCA

AATACGAAAGTAACTTTACAACTTAGACCCGACTCCACGAAAGCTGGGGGA

>40_Sample_ASV25

CACCGCGGTTATACGAAAGACCCTAGTTGATAGACCACGGCGTAAAGGGTGGTTAAGGAA

CACAAAAATAAAGCTAAAGATCCTCTAAGCTGTCATACGCTTCCCGAAGACATGAAACCC

AATCACGAAAGTAGCTTTAAACTGTCCTGACACCACGAAAGCCAAGAAA

>40_Sample_ASV26

CACCGCGGTTATACGAAAGACCCTAGTTGATAGATCACGGCGTAAAGGGTGGTTAAGGAG

AACAAAAATAAAGCTAAAAATCTTCTAAGCTGTCATACGCTTTCCGAAGACATGAGACCC

AACCACGAAAGTAGCTTTAAACTTTCCTGACACCACGAAAGCCAAGAAA

>41_Sample_ASV1

CACCGCGGTTATACGAAAGACCCTAGTTGATGGGTGCGGCGTAAAGGGTGGTTAGGGACT

ACAAAAAATAGAGCTAAAGATCCTCTAGGTCGTCATACACATCACGAGAAAACGAAGCCC

AAATACGAAAGTAGCTCTAAATATAATCCTGACGCCACGAAAGCCAAGAAA

>41_Sample_ASV2

CACCGCGGTTATACGAAAGACCCTAGTTGATGGGTGCGGCGTAAAGGGTGGTTAGGGACT

ACAAAAAATAGAGCTAAAGATCCTCTAGGTCGTCATACACATCACGAGGAAACGAAGCCC

AAATACGAAAGTAGCTCTAAATATAATCCTGACGCCACGAAAGCCAAGAAA

>41_Sample_ASV3

CACCGCGGTTATACGAGAGACCCTAGTTGATTAAAGCGGCGTAAAGAGTGGTTATGGAAC

TATTCTTTTAAAGCAGAAAACCTCTCAAACTGTTATACGCACCCAGAGGTCTGAATCCCT

CACACGAAAGTGACTTTATTTATGCCTACCAGAACCCACGAAAGCTGGGACA

>41_Sample_ASV4

CACCGCGGTTATACGAGAGGCCCAAGTTGACAGACACCGGCGTAAAGAGTGGCTAGGGAA

AAATTACTACTAAAGCCGAACACCTTCAGAACTGTCATACGTTCCCGAAGATAAGAAGCC

CCACCACGAAAGTGGCTTTATATTACCCGACCCCACGAAAGCTAAGAAA

>41_Sample_ASV5

CACCGCGGTTATACGAGAGGCCCAAGTTGACAGACACCGGCGTAAAGAGTGGCTAGGGAA

AAATTACTACTAAAGCCGAACACCTTCAGAACTGTCATACGTTCCCGAAGATAAGAAGCC

CCACCACGAAAGTGGCTTTATATTACCCGACCCCACGAAAGCTGTGAAA

>41_Sample_ASV6

CACCGCGGTTATACGAGAGGCCCAAGTTGACAGACACCGGCGTAAAGAGTGGCTAAGGAA

AAATTTCTACTAAAGCCGAACACCTTCAGAACTGTCATACGTTCCCGAAGAAAAGAAGCC

CCACCACGAAAGTGGCTTTATATTACCCGACCCCACGAAAGCTGTGAAA

>41_Sample_ASV7

CACCGCGGTTATACGAAAGACTCTAGTTGACAAACACGGCGTAAAGGGTGGTTAGGATAA

CAACAAAATAAAGCTAAAGACTTACCAAGCCGTCATACGCCCATGTAAGAGCGAAAACCA

CAAACGAAAGTAACTTTATTACACCATCCGAATCCACGAAAGCTAAGAAA

>41_Sample_ASV8

CACCGCGGTTATACGAAAGACCCTAGTTGATAGATCACGGCGTAAAGGGTGGTTAGGGAA

TACAAAAATAAAGCTAAAAATCCTCTAAGCTGTCATACGCTTCCCGAAGACATGAAACCC

AACCACGAAAGTAGCTTTAAACTCTCCTGACACCACGAAAGCCAAGGAA

>41_Sample_ASV9

CACCGCGGTTATACGAGAGGCCCAAGTTGATAAGCCCGGCGTAAAGAGTGGTTATGGAGA

ATAAAACACTAAAGCTAAAGACCCCCTAGGCTGTTATACGCACCTGGCGGCTCGAACCAC

CTATACGAAAGTAGCTTTACCCCCCTTCCACCAGAATCCACGACAGCTGGGGCA

>41_Sample_ASV10

CACCGCGGTTATACGAGAGACCCCAGTTGATACCCACGGCGTAAAGAGTGGTTATGGAGA

ATAAAACACTAAAGCTAAAGACCCCCTAGGCTGTTATACGCACCTGGCGGCTCGAACCAC

CTATACGAAAGTAGCTTTACCCCCCTTCCACCAGAATCCACGACAGCTGGGGCA

>41_Sample_ASV11

CACCGCGGTTATACGAGAGGCCCAAGTTGATAAGCCCGGCGTAAAGAGTGGTTATGGAGA

ATAAAACACTAAAGCTAAAGACCCCCTAGGCTGTTATACGCACCTGGCGGCTCGAACCAC

CTATACGAAAGTAGCTTTACCCCCCTTCCACCAGAATCCACGAAAGCTAAGAAA

>41_Sample_ASV12

CACCGCGGTTATACGAGAGGCCCTAGTTGATAGCCACGGCGCAAAGAGTGGTTAAGGAGC

CCCACTAAATAAAGTCGAACACTTCCTAGGCCGTTATACGTTTTCTAGAAGCACGAAACC

CAATTACACGAAAGCAACTTTATACTAAACAACCTGACCCCACGAAAGCTAAGAAA

>41_Sample_ASV13

CACCGCGGTTATACGAGAGGCCCAAGTTGACAGACATCGGCGTAAAGAGTGGTTAAGGAA

ACCCCATTACTAAAGCCGAACGCCCTCAGAACTGTCATACGTTCCCGAAGGTAAGAAGCC

CCACTACGAAAGTGGCTTTAACCACACCCGACCCCACGAAAGCTAAGAAA

>41_Sample_ASV14

CACCGCGGTTATACGAGAGGCCCAAGTTGACAGACATCGGCGTAAAGAGTGGTTAAGGAA

ACCCCATTACTAAAGCCGAACGCCCTCAGAACTGTCATACGTTCCCGAAGGTAAGAAGCC

CCACTACGAAAGTGGCTTTAACCACACCCGACCCCACGAAAGCTGTGAAA

>41_Sample_ASV15

CACCGCGGTTATACGAGAGGCCCCAGTTGATATTAGCGGCGTAAAGGGTGGTTAAGGACT

TTAATAACTAAAGCTAAACACTTCCCCGGCTGTTGTACGTAGACCGGAAGCTTGAAGCCC

AGACACGAAAGTAGCTTTATTCCCCCTGACCCCACGAAAGCTGAGAAA

>41_Sample_ASV16

CACCGCGGTTATACGAGAGGCCCTAGTTGATAAATACGGCGTAAAGAGTGGTTAGAGGAC

TCCCATAATAAAGCCAAAGACCCCCCGGGCCGTCACACGCATACGAGGGCACGAAGTTCA

CACACGAAAGTAGCTTTAAATTACCCGACGCCACGAAAGCTAAGAAA

>41_Sample_ASV17

CACCGCGGTTATACGAAAGACCCCAGTTTATAGACACGGCGTAAAGGGTGGTTATAGGAC

AAACTAACATAGAGCCAAAGACCCCCTAAGCCGTCATACGCTCACGAGGGCTCGAAGCCC

CTATACGAAAGTAGCTCTACCAAAAAATGCTCGACTCCACGAAAGCTGGGACA

>41_Sample_ASV18

CACCGCGGTTATACGAAAGACCCTAGTTGATGGATCACGGCGTAAAGGGTGGTTAAGGGA

TATAAAAATAAAGCTAAAAATCCTCTTAGCTGTCATACGCTTTCCGAAGACATGAGGCCC

AATTACGAAAGTAGCTTTAAACCACCCTGACACCACGAAAGCCAAGAAA

>41_Sample_ASV19

CACCGCGGTTATACGAAAGACCCCAGTTTATAGATGCGGCGTAAAGGGTGGTTAAGAGAA

AATCAAAATAAGGCCAAAGATCCCCTAAGCAGTCATACGCTCCACGAGGACACGAAACCC

AAATACGAAAGTTGCCTTAATAAACTTCACCTGATCCCACGAAAGCTGAAAAA

>41_Sample_ASV20

CACCGCGGTTATACGAGAGACTCTAGTTGATACCAACGGCGTAAAGAGTGGTTTGGGATG

CCCCTTAAAATAAAGCCAAAGACCTCCCAAGCCGTCAAACGCACCCCGGAGGCACGAAGC

CCCAACGCGAAAGCAACTTTACCTCCCTCCCGACGCCACGAAAGCTAAGAAA

>41_Sample_ASV21

CACCGCGGTTATACGAGAGGCCCTAGTTGATAACTACCGGCGTAAAGAGTGGTTATGGAA

AAATATTTAATAAAGCCGAACACCCCCTCAGCCGTCATACGCACCTGGGAGCACGAAGAC

CTACTGCGAAAGCAGCTTTAATTATGCCTGACCCCACGACAGCTAAGAAA

>41_Sample_ASV22

CACCGCGGTTATACGAAAGACCCTAGTTTATAGACACGGCGTAAAGGGTGGTTCGGGACT

AAACAAAATAAAGCCAAAGACCTCCTAAGCCGTCATACGCTCACGGAAGCACGAAGCCCA

AGTACGAAAGTAGCTTTACAAAACATGCCTGACTCCACGAAAGCTAGGAAA

>41_Sample_ASV23

CACCGCGGTTATACGAGAGGCCCAAGTTGATTATTACCGGCGTAAAGCGTGGTTAAGATT

TTTATAGGACTAAAGCCGAACCCCCTCAGAGCTGTTATACGCACCCGAAGGTTAGAAGTT

CAATCACGAAAGTGGCTTTACAACCCCGAACCCACGAAAGCTAAGATA

>41_Sample_ASV24

CACCGCGGTTATACGAGAGACCCTAATTGATAGGCGCGGCGTAAAGAGTGGTTTAGGACT

AAAACTAATAAAGCTAAAGATCCTCCCAGCCGTCGCACGCATTCTGAGGACACGAAACTC

AACCACGAAAGTAGCTTTAACATACTCTCCTGACCCCACGAAAGCTAAGAAA

>41_Sample_ASV25

CACCGCGGTTATACGAAAGACCCTAGTTGATAGGCACGGCGTAAAGGGTGGTTAGGGATA

TATCAAAAATAAAGTTAAAGAGCCTCTAAGCTGTCGCACGCATCCCGAGAGCTCGAGACC

CAGACACGAAAGTAACTTTAAAATACCTCACCTGACCCCACGAAAGCTAAGAAA

>41_Sample_ASV26

CACCGCGGTTATACGAAAGACCCTAGTTGATAGCCACGGCGTAAAGGGTGGTTAAGGTAT

ACAACAAATAAAGCTAAATAGCCTCTAGGCCGTCGCACGCATTCCGAGAGCTCGAAACCC

AAAAACGAAAGTAGCTTTAAAATAAACAGACCTGACCCCACGAAAGCTAAGAAA

>41_Sample_ASV27

CACCGCGGTTATACGAGAGGCCCAAGTTGATACTCCACGGCGTAAAGAGTGGTTAGAAAA

GACCTACTACTAAAGCCGAACGCCTTCAAAGCTGTTATACGCATCCGAAGGTGAGAAGCC

CACCCACGAAAGTGGCTTTACAACCTTGACTCCACGAAAGCTATGACA

>41_Sample_ASV28

CACCGCGGTTATACGAAAGACCCTAGTTTATAGGCACGGCGTAAAGGGTGGTTAGGGGAT

AAACAAAATAAAGCCAAAGACCTTCTAAGTCGTCATACACTCATGAAGGCACGAAGCCCA

AATACGAAAGTAACTTTACAACTTAGACCCGACTCCACGAAAGCTGGGGGA

>41_Sample_ASV29

CACCGCGGTTATACGAGAGACCCTAATTAATAGATACGGCGTAAAGAGTGGTTTAGGACA

AAAATTAATAAAGCCGAAGTTTCTCCTAGCCGTTGTACGCACTCTGAGAACACGAAGCCC

CACTACGAAAGTAGCTTTACCTATTATTTCCTGACCCCACGAAAGCTAAGAAA

>41_Sample_ASV30

CACCGCGGTTATACGAGAGACCCTAATTAATAGCTACGGCGTAAAGAGTGGTTTAGGGTA

AAAATTAATAAAGCCGAAGTTTCTCCTAGCCGTTGTACGCACTCTGAGAACACGAAGCCC

CACTACGAAAGTAGCTTTACCTATTATTTCCTGACCCCACGAAAGCTAAGAAA

>41_Sample_ASV31

CACCGCGGTTATACGAGAGACCCTAATTAATAGATACGGCGTAAAGAGTGGTTTAGGACA

AAAATTAATAAAGCCGAAGTTTCTCCTAGCCGTTGTACGCACTCTGAGAACACGAAGCCC

CACTACGAAAGTAGCTTTACCAATAATCTCCTGACCCCACGAAAGCTAAGAAA

>41_Sample_ASV32

CACCGCGGTTATACGAGAGACCCTAATTAATAGCTACGGCGTAAAGAGTGGTTTAGGACA

AAAATTAATAAAGCCGAAGTTTCTCCTAGCCGTTGTACGCACTCTGAGAACACGAAGCCC

CACTACGAAAGTAGCTTTACCTATTATTTCCTGACCCCACGAAAGCTAAGAAA

>41_Sample_ASV33

CACCGCGGTTATACGAAAGACCCTAGTTGATAGATCACGGCGTAAAGGGTGGTTAAGGAA

AACAAAAATAAAGCTAAAAATCCTCTAAGCTGTCATACGCTTTCCGAAGACATGAGATCC

AACCACGAAAGTAGCTTTAAAATTTCCTGACACCACGAAAGCCAAGGAA

>43_Sample_ASV1

CACCGCGGTTATACGAGAGGCCCAAGTTGACAGACATCGGCGTAAAGAGTGGTTAAGGAA

AACCTTCAACTAAAGTCGAACGCTCTCAAGACTGTCGTACGTTTCCGAGAGTATGAAGCC

CCACCACGAAAGTGACTTTACCTCACCTGACTCCACGAAAGCTGCGAAA

>43_Sample_ASV2

CACCACGGTTATACGAGAGGCCCAAGTTGAAAGACATCGGCGTAAAGAGTGGTTAAGGAA

AACCTTCTACTAAAGTCGAACGCTCTCAGGACTGTCGTACGTTTCCGAGAGTATGAAGCC

CCACCACGAAAGTGACTTTACCTCACCTGACTCCACGAAAGCTGCGAAA

>43_Sample_ASV3

CACCGCGGTTATACGAAAGACCCTAGTTGATGGGTGCGGCGTAAAGGGTGGTTAGGGACT

ACAAAAAATAGAGCTAAAGATCCTCTAGGTCGTCATACACATCACGAGAAAACGAAGCCC

AAATACGAAAGTAGCTCTAAATATAATCCTGACGCCACGAAAGCCAAGAAA

>43_Sample_ASV4

CACCGCGGTTATACGAAAGACCCTAGTTGATGGGTGCGGCGTAAAGGGTGGTTAGGGACT

ACAAAAAATAGAGCTAAAGATCCTCTAGGTCGTCATACACATCACGAGGAAACGAAGCCC

AAATACGAAAGTAGCTCTAAATATAATCCTGACGCCACGAAAGCCAAGAAA

>43_Sample_ASV5

CACCGCGGTTATACGAGAGACCCTAGTTGATTAAAGCGGCGTAAAGAGTGGTTATGGAAC

TATTCTTTTAAAGCAGAAAACCTCTCAAACTGTTATACGCACCCAGAGGTCTGAATCCCT

CACACGAAAGTGACTTTATTTATGCCTACCAGAACCCACGAAAGCTGGGACA

>43_Sample_ASV6

CACCGCGGTTATACGAGAGGCCCAAGTTGACAGACACCGGCGTAAAGAGTGGCTAGGGAA

AAATTACTACTAAAGCCGAACACCTTCAGAACTGTCATACGTTCCCGAAGATAAGAAGCC

CCACCACGAAAGTGGCTTTATATTACCCGACCCCACGAAAGCTGTGAAA

>43_Sample_ASV7

CACCGCGGTTATACGAGAGGCCCAAGTTGACAGACACCGGCGTAAAGAGTGGCTAAGGAA

AAATTTCTACTAAAGCCGAACACCTTCAGAACTGTCATACGTTCCCGAAGAAAAGAAGCC

CCACCACGAAAGTGGCTTTATATTACCCGACCCCACGAAAGCTGTGAAA

>43_Sample_ASV8

CACCGCGGTTATACGAAAGACTCTAGTTGACAAACACGGCGTAAAGGGTGGTTAGGATAA

CAACAAAATAAAGCTAAAGACTTACCAAGCCGTCATACGCCCATGTAAGAGCGAAAACCA

CAAACGAAAGTAACTTTATTACACCATCCGAATCCACGAAAGCTAAGAAA

>43_Sample_ASV9

CACCGCGGTTATACGAGAGGCCCAAGTTGATAAGCCCGGCGTAAAGAGTGGTTATGGAGA

ATAAAACACTAAAGCTAAAGACCCCCTAGGCTGTTATACGCACCTGGCGGCTCGAACCAC

CTATACGAAAGTAGCTTTACCCCCCTTCCACCAGAATCCACGACAGCTGGGGCA

>43_Sample_ASV10

CACCGCGGTTATACGAAAGACCCAAGTTGATAGTCACGGCGTAAAGAGTGGTTATGGAGA

ATAAAACACTAAAGCTAAAGACCCCCTAGGCTGTTATACGCACCTGGCGGCTCGAACCAC

CTATACGAAAGTAGCTTTACCCCCCTTCCACCAGAATCCACGACAGCTGGGGCA

>43_Sample_ASV11

CACCGCGGTTATACGAGAGGCCCTAGTTGATAGCCACGGCGCAAAGAGTGGTTAAGGAGC

CCCACTAAATAAAGTCGAACACTTCCTAGGCCGTTATACGTTTTCTAGAAGCACGAAACC

CAATTACACGAAAGCAACTTTATACTAAACAACCTGACCCCACGAAAGCTAAGAAA

>43_Sample_ASV12

CACCGCGGTTATACGAGAGGCCCAAGTTGACAGACATCGGCGTAAAGAGTGGTTAAGGAA

ACCCCATTACTAAAGCCGAACGCCCTCAGAACTGTCATACGTTCCCGAAGGTAAGAAGCC

CCACTACGAAAGTGGCTTTAACCACACCCGACCCCACGAAAGCTGTGAAA

>43_Sample_ASV13

CACCGCGGTTATACGAGAGGCCCTAGTTGATAACTACCGGCGTAAAGAGTGGTTAAGGAA

ACCCCATTACTAAAGCCGAACGCCCTCAGAACTGTCATACGTTCCCGAAGGTAAGAAGCC

CCACTACGAAAGTGGCTTTAACCACACCCGACCCCACGAAAGCTGTGAAA

>43_Sample_ASV14

CACCGCGGTTATACGAGAGGCCCCAGTTGATATTAGCGGCGTAAAGGGTGGTTAAGGACT

TTAATAACTAAAGCTAAACACTTCCCCGGCTGTTGTACGTAGACCGGAAGCTTGAAGCCC

AGACACGAAAGTAGCTTTATTCCCCCTGACCCCACGAAAGCTGAGAAA

>43_Sample_ASV15

CACCGCGGTTATACGAAAGACCCCAGTTTATAGACACGGCGTAAAGGGTGGTTATAGGAC

AAACTAACATAGAGCCAAAGACCCCCTAAGCCGTCATACGCTCACGAGGGCTCGAAGCCC

CTATACGAAAGTAGCTCTACCAAAAAATGCTCGACTCCACGAAAGCTGGGACA

>43_Sample_ASV16

AACCGCGGTTATACGAGAGACCCTAATTGATAGCTCACGGCGTAAAGGGTGGTTTGGGGA

AACCTCAAAATAAAGCTAAAGACCTTCTAAGCCGTTACACGCATACCGAAGGCACGAGGC

CCTAACACGAAAGTAGCTTTACTATTACCCCTGACGCCACGAAAGCTAAGAAA

>43_Sample_ASV17

CACCGCGGTTATACGAAAGACCCTAGTTGATGGATCACGGCGTAAAGGGTGGTTAAGGGA

TATAAAAATAAAGCTAAAAATCCTCTTAGCTGTCATACGCTTTCCGAAGACATGAGGCCC

AATTACGAAAGTAGCTTTAAACCACCCTGACACCACGAAAGCCAAGAAA

>43_Sample_ASV18

CACCGCGGTTATACGAGAGGCCCTAGTTGATAACTACCGGCGTAAAGAGTGGTTATGGAA

AAATATTTAATAAAGCCGAACACCCCCTCAGCCGTCATACGCACCTGGGAGCACGAAGAC

CTACTGCGAAAGCAGCTTTAATTATGCCTGACCCCACGACAGCTAAGAAA

>43_Sample_ASV19

CACCGCGGTTATACGAGAGGCCCAAGTTGATTATTACCGGCGTAAAGCGTGGTTAAGATT

TTTATAGGACTAAAGCCGAACCCCCTCAGAGCTGTTATACGCACCCGAAGGTTAGAAGTT

CAATCACGAAAGTGGCTTTACAACCCCGAACCCACGAAAGCTAAGATA

>43_Sample_ASV20

CACCGCGGTTATACGAGAGACCCTAGTTGATATATACGGCGTAAAGAGTGGTTTAGGACT

AAAACTAATAAAGCTAAAGATCCTCCCAGCCGTCGCACGCATTCTGAGGACACGAAACTC

AACCACGAAAGTAGCTTTAACATACTCTCCTGACCCCACGAAAGCTAAGAAA

>43_Sample_ASV21

CACCGCGGTTATACGAGAGACCCTAATTGATAGGCGCGGCGTAAAGAGTGGTTTAGGACT

AAAACTAATAAAGCTAAAGATCCTCCCAGCCGTCGCACGCATTCTGAGGACACGAAACTC

AACCACGAAAGTAGCTTTAACATACTCTCCTGACCCCACGAAAGCTAAGAAA

>43_Sample_ASV22

CACCGCGGTTATACGAAAGACCCTAGTTGATAGGCACGGCGTAAAGGGTGGTTAGGGATA

TATCAAAAATAAAGTTAAAGAGCCTCTAAGCTGTCGCACGCATCCCGAGAGCTCGAGACC

CAGACACGAAAGTAACTTTAAAATACCTCACCTGACCCCACGAAAGCTAAGAAA

>43_Sample_ASV23

CACCGCGGTTATACGAAAGACCCTAGTTGATAGCCACGGCGTAAAGGGTGGTTAAGGTAT

ACAACAAATAAAGCTAAATAGCCTCTAGGCCGTCGCACGCATTCCGAGAGCTCGAAACCC

AAAAACGAAAGTAGCTTTAAAATAAACAGACCTGACCCCACGAAAGCTAAGAAA

>43_Sample_ASV24

CACCGCGGTTATACGAGAGGCCCAAGTTGATACTCCACGGCGTAAAGAGTGGTTAGAAAA

GACCTACTACTAAAGCCGAACGCCTTCAAAGCTGTTATACGCATCCGAAGGTGAGAAGCC

CACCCACGAAAGTGGCTTTACAACCTTGACTCCACGAAAGCTATGACA

>43_Sample_ASV25

CACCGCGGTTATACGAGAGACCCTAGTTGATATATACGGCGTAAAGAGTGGTTTGGAATA

ACCAAGTAATATAGCCAAAGACCTCCCAAGCCGTCACACGCACCCCGGAGGCACGAAGCC

CAAACACGAAAGTAGCTTTATTAACTTACCGACGCCACGAAAGCTAAGAAA

>43_Sample_ASV26

CACCGCGGTTATACGAAAGACCCTAGTTTATAGGCACGGCGTAAAGGGTGGTTAGGGGAT

AAACAAAATAAAGCCAAAGACCTTCTAAGTCGTCATACACTCATGAAGGCACGAAGCCCA

AATACGAAAGTAACTTTACAACTTAGACCCGACTCCACGAAAGCTGGGGGA

>43_Sample_ASV27

CACCGCGGTTATACGAGAGACCCTAATTAATAGATACGGCGTAAAGAGTGGTTTAGGACA

AAAATTAATAAAGCCGAAGTTTCTCCTAGCCGTTGTACGCACTCTGAGAACACGAAGCCC

CACTACGAAAGTAGCTTTACCTATTATTTCCTGACCCCACGAAAGCTAAGAAA

>43_Sample_ASV28

CACCGCGGTTATACGAGAGACCCTAATTAATAGCTACGGCGTAAAGAGTGGTTTAGGGTA

AAAATTAATAAAGCCGAAGTTTCTCCTAGCCGTTGTACGCACTCTGAGAACACGAAGCCC

CACTACGAAAGTAGCTTTACCTATTATTTCCTGACCCCACGAAAGCTAAGAAA

>43_Sample_ASV29

CACCGCGGTTATACGAGAGACCCTAATTAATAGATACGGCGTAAAGAGTGGTTTAGGACA

AAAATTAATAAAGCCGAAGTTTCTCCTAGCCGTTGTACGCACTCTGAGAACACGAAGCCC

CACTACGAAAGTAGCTTTACCAATAATCTCCTGACCCCACGAAAGCTAAGAAA

>43_Sample_ASV30

CACCGCGGTTATACGAGAGACCCTAATTAATAGCTACGGCGTAAAGAGTGGTTTAGGACA

AAAATTAATAAAGCCGAAGTTTCTCCTAGCCGTTGTACGCACTCTGAGAACACGAAGCCC

CACTACGAAAGTAGCTTTACCTATTATTTCCTGACCCCACGAAAGCTAAGAAA

>43_Sample_ASV31

CACCGCGGTTATACGAAAGACCCTAGTTGATAGATCACGGCGTAAAGGGTGGTTAAGGAG

AACAAAAATAAAGCTAAAAATCTTCTAAGCTGTCATACGCTTTCCGAAGACATGAGACCC

AACCACGAAAGTAGCTTTAAACTTTCCTGACACCACGAAAGCCAAGAAA

>43_Sample_ASV32

CACCGCGGTTATACGAAAGACCCTAGTTGATAGATCACGGCGTAAAGGGTGGTTAAGGAA

AACAAAAATAAAGCTAAAAATCCTCTAAGCTGTCATACGCTTTCCGAAGACATGAGATCC

AACCACGAAAGTAGCTTTAAAATTTCCTGACACCACGAAAGCCAAGGAA

>44_Sample_ASV1

CACCGCGGTTATACGAGAGGCCCAAGTTGACAGACATCGGCGTAAAGAGTGGTTAAGGAA

AACCTTCAACTAAAGTCGAACGCTCTCAAGACTGTCGTACGTTTCCGAGAGTATGAAGCC

CCACCACGAAAGTGACTTTACCTCACCTGACTCCACGAAAGCTGCGAAA

>44_Sample_ASV2

CACCGCGGTTATACGAAAGACCCTAGTTGATGGGTGCGGCGTAAAGGGTGGTTAGGGACT

ACAAAAAATAGAGCTAAAGATCCTCTAGGTCGTCATACACATCACGAGGAAACGAAGCCC

AAATACGAAAGTAGCTCTAAATATAATCCTGACGCCACGAAAGCCAAGAAA

>44_Sample_ASV3

CACCGCGGTTATACGAGAGACCCTAGTTGATTAAAGCGGCGTAAAGAGTGGTTATGGAAC

TATTCTTTTAAAGCAGAAAACCTCTCAAACTGTTATACGCACCCAGAGGTCTGAATCCCT

CACACGAAAGTGACTTTATTTATGCCTACCAGAACCCACGAAAGCTATGACA

>44_Sample_ASV4

CACCGCGGTTATACGAGAGACCCTAGTTGATTAAAGCGGCGTAAAGAGTGGTTATGGAAC

TATTCTTTTAAAGCAGAAAACCTCTCAAACTGTTATACGCACCCAGAGGTCTGAATCCCT

CACACGAAAGTGACTTTATTTATGCCTACCAGAACCCACGAAAGCTGGGACA

>44_Sample_ASV5

CACCGCGGTTATACGAAAGACCCTAGTTTATAGGTACGGCGTAAAGGGTGGTTAGGGGAC

AAACAAAATAAAGCCAAAGACCTCCTAAGCCGTCATACGCTCACGGAAGCACGAAGCCCA

AACACGAAAGTAGCTTTACCGAACATGCCCGACTCCACGAAAGCTGGGGAA

>44_Sample_ASV6

CATGGCGATTATACGAGAGGCCCAAGTTGACAGACACTGGCGTAAAGAGTGGCTAAGGAA

AAATTACTACTAAAGCCGAACACCTTCAGAACTGTCATACGTTCCCGAAGATAAGAAGCC

CCACCACGAAAGTGGCTTTATATTACCCGACCCCACGAAAGCTGTGAAA

>44_Sample_ASV7

CACCGCGGTTAAACGAGAGACCCTAGTTGACAGACACCGGCGTAAAGAGTGGCTAGGGAA

AAATTACTACTAAAGCCGAACACCTTCAGAACTGTCATACGTTCCCGAAGATAAGAAGCC

CCACCACGAAAGTGGCTTTATATTACCCGACCCCACGAAAGCTAAGAAA

>44_Sample_ASV8

CACCGCGGTTATACGAGAGGCCCAAGTTGACAGACACCGGCGTAAAGAGTGGCTAGGGAA

AAATTACTACTAAAGCCGAACACCTTCAGAACTGTCATACGTTCCCGAAGATAAGAAGCC

CCACCACGAAAGTGGCTTTATATTACCCGACCCCACGAAAGCTAAGAAA

>44_Sample_ASV9

CACCGCGGTTATACGAGAGGCCCAAGTTGACAGACACCGGCGTAAAGAGTGGCTAGGGAA

AAATTACTACTAAAGCCGAACACCTTCAGAACTGTCATACGTTCCCGAAGATAAGAAGCC

CCACCACGAAAGTGGCTTTATATTACCCGACCCCACGAAAGCTGTGAAA

>44_Sample_ASV10

CATGGCGATTATACGAGAGGCCCAAGTTGACAGACACTGGCGTAAAGAGTGGCTAAGGAA

AAACTACTACTAAAGCCGAACACCTTCAGAACTGTCATACGTTCCCGAAGATAAGAAGCC

CCACCACGAAAGTGGCTTTATATTACCCGACCCCACGAAAGCTGTGAAA

>44_Sample_ASV11

CACCGCGGTTAAACGAGAGACCCTAGTTGATCATCACGGCGTAAAGAGTGGTTAGGGGAT

TATCATAAATAAAGCCAAAGACCTCCCAAGCTGTCGCACGCATTCCGGAGGCGCGAAGCC

CACACACGAAAGTAGCTTTAACTATTGACCCTGATGCCACGAAAGCTAAGAAA

>44_Sample_ASV12

CACCGCGGTTATACGAAAGACCCTAGTTGATAGATCACGGCGTAAAGGGTGGTTAGGGAA

TACAAAAATAAAGCTAAAAATCCTCTAAGCTGTCATACGCTTCCCGAAGACATGAAACCC

AACCACGAAAGTAGCTTTAAACTCTCCTGACACCACGAAAGCCAAGGAA

>44_Sample_ASV13

CACCGCGGTTATACGAAAGACCCTAGTTTATAGGCACGGCGTAAAGGGTGGTTAGGGAAT

ACAAAAATAAAGCTAAAAATCCTCTAAGCTGTCATACGCTTCCCGAAGACATGAAACCCA

ACCACGAAAGTAGCTTTAAACTCTCCTGACACCACGAAAGCCAAGGAA

>44_Sample_ASV14

CACCGCGGTTATACGAGAGGCCCAAGTTGATAAGCCCGGCGTAAAGAGTGGTTATGGAGA

ATAAAACACTAAAGCTAAAGACCCCCTAGGCTGTTATACGCACCTGGCGGCTCGAACCAC

CTATACGAAAGTAGCTTTACCCCCCTTCCACCAGAATCCACGACAGCTGGGGCA

>44_Sample_ASV15

CACCGCGGTTATACGAGAGGCCCAAGTTGACAGACATCGGCGTAAAGAGTGGTTAAGGAA

ACCCCATTACTAAAGCCGAACGCCCTCAGAACTGTCATACGTTCCCGAAGGTAAGAAGCC

CCACTACGAAAGTGGCTTTAACCACACCCGACCCCACGAAAGCTGTGAAA

>44_Sample_ASV16

CACCGCGGTTATACGAGAGGCCCAAGTTGATAGATATCGGCGTAAAGAGTGGTTAAGGAA

ACCCCGTTACTAAAGCCGAACGCCCTCAGAACTGTCATACGTTCCCGAAGGTAAGAAGCC

CCACTACGAAAGTGGCTTTAACCACACCCGACCCCACGAAAGCTGTGAAA

>44_Sample_ASV17

CACCGCGGTTATACGAGAGGCCCCAGTTGATATTAGCGGCGTAAAGGGTGGTTAAGGACT

TTAATAACTAAAGCTAAACACTTCCCCGGCTGTTGTACGTAGACCGGAAGCTTGAAGCCC

AGACACGAAAGTAGCTTTATTCCCCCTGACCCCACGAAAGCTGAGAAA

>44_Sample_ASV18

CACCGCGGTTATACGAGAGGCCCTAGTTGATAAATACGGCGTAAAGAGTGGTTAGAGGAC

TCCCATAATAAAGCCAAAGACCCCCCGGGCCGTCACACGCATACGAGGGCACGAAGTTCA

CACACGAAAGTAGCTTTAAATTACCCGACGCCACGAAAGCTAAGAAA

>44_Sample_ASV19

GACCGCGGTTATACGAGAGACTCTAGTTGATAGATACGGCGTAAAGAGTGGTTTGGGATA

AACTATAAATAAAGCCAAAGACCTTCCAAGCTGTTAAACGCATACCGAAGGCACGAAGCC

CCAACACGAAAGTAGCTTTACTACTACCCCCGATGCCACGAAAGCTAAGACA

>44_Sample_ASV20

CACCGCGGTTATACGAGAGACCCTAGTTGATAAATACGGCGTAAAGAGTGGTTATGGGAA

AAACAAATAAAGTCAAACAACCTCTTAGCTGTTATACGCATTATGAGAGCATGAAGCCCC

CTCACGAAAGTAACTTTAATACCTCCTGACCCCACGAAAGCTAAGAAA

>44_Sample_ASV21

CACCGCGGTTATACGAGAGACCCTAATTGATAGACACGGCGTAAAGAGTGGTTTGGGAAA

GCCCAAAAATAAAGCTAAAGACCTTCTAAGCCGTCACACGCACGCCGAAGGCACGAAGCC

CTGACACGAAAGTAGCTTTACTATTACCCCCGACGCCACGAAAGCTAAGAAA

>44_Sample_ASV22

AACCGCGGTTATACGAGAGACCCTAATTGATAGCTCACGGCGTAAAGGGTGGTTTGGGGA

AACCTCAAAATAAAGCTAAAGACCTTCTAAGCCGTTACACGCATACCGAAGGCACGAGGC

CCTAACACGAAAGTAGCTTTACTATTACCCCTGACGCCACGAAAGCTAAGAAA

>44_Sample_ASV23

CACCGCGGTCATACGATTAACCCAAGTTAATAGGCACACGGCGTAAAGCGTGTTTAAGCA

CTACGCCAAATAAAGTTAAACTCCAATTAAGCTGTAAAAAGCCATAATCAAGACGAAAAT

AAATAACGAAAGTAACTTTACAACCGCTGAAACACGATAGCTAAGATC

>44_Sample_ASV24

CACCGCGGTCATACGATTAACCCAAGTTAATAGGCACACGGCGTAAAGCGTGTTTAAGCA

CTATGCCAAATAAAGTTAAACTCCAATTAAGCTGTAAAAAGCCATAATCAAGACGAAAAT

AAATAACGAAAGTAACTTTACAACCGCTGAAACACGATAGCTAAGATC

>44_Sample_ASV25

CACCGCGGTTATACGAAAGACCCTAGTTTATAGACACGGCGTAAAGGGTGGTTCGGGACT

AAACAAAATAAAGCCAAAGACCTCCTAAGCCGTCATACGCTCACGGAAGCACGAAGCCCA

AGTACGAAAGTAGCTTTACAAAACATGCCTGACTCCACGAAAGCTAGGAAA

>44_Sample_ASV26

CACCGCGGTTATACGAAAGACCCTAGTTGATAGGCACGGCGTAAAGGGTGGTTAGGGATA

TATTAAAAATAAAGTTAAAGAGCCTCTAAGCTGTCGCACGCATCCCGAGAGCTCGAGACC

CAGACACGAAAGTAACTTTAAAATACCTCACCTGACCCCACGAAAGCTAAGAAA

>44_Sample_ASV27

CACCGCGGTTATACGAAAGACCCTAGTTGATAGCCACGGCGTAAAGGGTGGTTAAGGTAT

ACAACAAATAAAGCTAAATAGCCTCTAGGCCGTCGCACGCATTCCGAGAGCTCGAAACCC

AAAAACGAAAGTAGCTTTAAAATAAACAGACCTGACCCCACGAAAGCTAAGAAA

>44_Sample_ASV28

CACCGCGGTTATACGAGAGGCCCAAGTTGATACTCCACGGCGTAAAGAGTGGTTAGAAAA

GACCTACTACTAAAGCCGAACGCCTTCAAAGCTGTTATACGCATCCGAAGGTGAGAAGCC

CACCCACGAAAGTGGCTTTACAACCTTGACTCCACGAAAGCTATGACA

>44_Sample_ASV29

CACCGCGGTTATACGAGAGACCCCAGTTGATAAACACGGCGTAAAGAGTGGTTAAGGGCA

CAAAACAAATAAAGTCAAACAACCTCTTAGCTGTTATACGCATTATGAGGATATGAAGCC

CCCCTACGAAAGTAACTTTACCAACCCCTGACCCCACGAAAGCTAAGAAA

>44_Sample_ASV30

CACCGCGGTTATACGAAAGACCCTAGTTTATAGGCACGGCGTAAAGGGTGGTTAGGGGAT

AAACAAAATAAAGCCAAAGACCTTCTAAGTCGTCATACACTCATGAAGGCACGAAGCCCA

AATACGAAAGTAACTTTACAACTTAGACCCGACTCCACGAAAGCTGGGGGA

>44_Sample_ASV31

CACCGCGGTTATACGAAAGACCCTAGTTTATAGGCACGGCGTAAAGGGTGGTTAGGGGAT

AAACAAAATAAAGCCAAAGACCTTCTAAGTCGTCATACACTCATGAAGGCACGAAGCCCA

AATACGAAAGTAACTTTACAACTTAGACCCGACTCCACGAAAGCCAAGGAA

>44_Sample_ASV32

CACCGCGGTTATACGAGAGGCCCAAGTTGATAGACATCGGCGTAAAGAGTGGTTAAGGAA

GTCTCAAAACTAAAGCCGAATGCCTTCAAGACTGTTATACGTTTCCGAAGGTAAGAAGCC

CCACTACGAAAGTGGCTTTACTCCACCTGACTCCACGAAAGCTGTGAAA

>44_Sample_ASV33

CACCGCGGTTATACGAAAGACCCTAGTTGATAGATCACGGCGTAAAGGGTGGTTAAGGAG

AACAAAAATAAAGCTAAAAATCTTCTAAGCTGTCATACGCTTTCCGAAGACATGAGACCC

AACCACGAAAGTAGCTTTAAACTTTCCTGACACCACGAAAGCCAAGAAA

>45_Sample_ASV1

CACCGCGGTTATACGAAAGACCCTAGTTGATGGGTGCGGCGTAAAGGGTGGTTAGGGACT

ACAAAAAATAGAGCTAAAGATCCTCTAGGTCGTCATACACATCACGAGAAAACGAAGCCC

AAATACGAAAGTAGCTCTAAATATAATCCTGACGCCACGAAAGCCAAGAAA

>45_Sample_ASV2

CACCGCGGTTATACGAAAGACCCTAGTTGATGGGTGCGGCGTAAAGGGTGGTTAGGGACT

ACAAAAAATAGAGCTAAAGATCCTCTAGGTCGTCATACACATCACGAGGAAACGAAGCCC

AAATACGAAAGTAGCTCTAAATATAATCCTGACGCCACGAAAGCCAAGAAA

>45_Sample_ASV3

CACCGCGGTTATACGAGAGACCCTAGTTGATTAAAGCGGCGTAAAGAGTGGTTATGGAAC

TATTCTTTTAAAGCAGAAAACCTCTCAAACTGTTATACGCACCCAGAGGTCTGAATCCCT

CACACGAAAGTGACTTTATTTATGCCTACCAGAACCCACGAAAGCTGGGACA

>45_Sample_ASV4

CACCGCGGTTATACGAGAGGCCCAAGTTGACAGACACCGGCGTAAAGAGTGGCTAGGGAA

AAATTACTACTAAAGCCGAACACCTTCAGAACTGTCATACGTTCCCGAAGATAAGAAGCC

CCACCACGAAAGTGGCTTTATATTACCCGACCCCACGAAAGCTAAGAAA

>45_Sample_ASV5

CACCGCGGTTATACGAGAGGCCCAAGTTGACAGACACCGGCGTAAAGAGTGGCTAGGGAA

AAATTACTACTAAAGCCGAACACCTTCAGAACTGTCATACGTTCCCGAAGATAAGAAGCC

CCACCACGAAAGTGGCTTTATATTACCCGACCCCACGAAAGCTGTGAAA

>45_Sample_ASV6

CACCGCGGTTATACGAGAGGCCCAAGTTGACAGACACCGGCGTAAAGAGTGGCTAAGGAA

AAATTTCTACTAAAGCCGAACACCTTCAGAACTGTCATACGTTCCCGAAGAAAAGAAGCC

CCACCACGAAAGTGGCTTTATATTACCCGACCCCACGAAAGCTGTGAAA

>45_Sample_ASV7

CACCGCGGTTAAACGAGAGACCCTAGTTGATCATCACGGCGTAAAGAGTGGTTAGGGGAT

TATCATAAATAAAGCCAAAGACCTCCCAAGCTGTCGCACGCATTCCGGAGGCGCGAAGCC

CACACACGAAAGTAGCTTTAACTATTGACCCTGATGCCACGAAAGCTAAGAAA

>45_Sample_ASV8

CACCGCGGTTATACGAGAGACCCTAGTTGATAACTACGGCGTAAAGAGTGGTTATGGAGA

ATAAAACACTAAAGCTAAAGACCCCCTAGGCTGTTATACGCACCTGGCGGCTCGAACCAC

CTATACGAAAGTAGCTTTACCCCCCTTCCACCAGAATCCACGACAGCTGGGGCA

>45_Sample_ASV9

CACCGCGGTTATACGAGAGGCCCAAGTTGATAAGCCCGGCGTAAAGAGTGGTTATGGAGA

ATAAAACACTAAAGCTAAAGACCCCCTAGGCTGTTATACGCACCTGGCGGCTCGAACCAC

CTATACGAAAGTAGCTTTACCCCCCTTCCACCAGAATCCACGACAGCTGGGGCA

>45_Sample_ASV10

CACCGCGGTTATACGAGAGGCCCTAGTTGATAGCCACGGCGCAAAGAGTGGTTAAGGAGC

CCCACTAAATAAAGTCGAACACTTCCTAGGCCGTTATACGTTTTCTAGAAGCACGAAACC

CAATTACACGAAAGCAACTTTATACTAAACAACCTGACCCCACGAAAGCTAAGAAA

>45_Sample_ASV11

CACCGCGGTTATACGAGAGGCCCAAGTTGACAGACATCGGCGTAAAGAGTGGTTAAGGAA

ACCCCATTACTAAAGCCGAACGCCCTCAGAACTGTCATACGTTCCCGAAGGTAAGAAGCC

CCACTACGAAAGTGGCTTTAACCACACCCGACCCCACGAAAGCTGTGAAA

>45_Sample_ASV12

CACCGCGGTTATACGAGAGGCCCAAGTTGACAGACACCGGCGTAAAGAGTGGTTAAGGAA

ATTCATCAACTAAAGCCGAATGCCCTCAGGACTGTTATACGTTTGCGAAGGTAAGAAGCC

CTACTACGAAAGTGGCTTTATTCCACCTGATTCCACGAAAGCTGCGGAA

>45_Sample_ASV13

CACCGCGGTTATACGAGAGGCCCCAGTTGATATTAGCGGCGTAAAGGGTGGTTAAGGACT

TTAATAACTAAAGCTAAACACTTCCCCGGCTGTTGTACGTAGACCGGAAGCTTGAAGCCC

AGACACGAAAGTAGCTTTATTCCCCCTGACCCCACGAAAGCTGAGAAA

>45_Sample_ASV14

CACCGCGGTTATACGAGAGGCCCCAGTTGATATTAGCGGCGTAAAGGGTGGTTAAGGACT

TTAATAACTAAAGCTAAACACTTCCCCGGCTGTTGTACGTAGACCGGAAGCTTGAAGCCC

AGACACGAAAGTAGCTTTATTTCCCCTGACCCCACGAAAGCTGAGAAA

>45_Sample_ASV15

CACCGCGGTTATACGAGAGGCCCCAGTTGATATTAGCGGCGTAAAGGGTGGTTAAGGACT

TTAATAACTAAAGCCAAACACTTCCCCGGCTGTTGTACGTAGACCGGAAGCTTGAAGCCC

AGACACGAAAGTAGCTTTATTTCCCCTGACCCCACGAAAGCTGAGAAA

>45_Sample_ASV16

CACCGCGGTTATACGAAAGACCCCAGTTTATAGACACGGCGTAAAGGGTGGTTATAGGAC

AAACTAACATAGAGCCAAAGACCCCCTAAGCCGTCATACGCTCACGAGGGCTCGAAGCCC

CTATACGAAAGTAGCTCTACCAAAAAATGCTCGACTCCACGAAAGCTGGGACA

>45_Sample_ASV17

CACCGCGGTTATACGAGAGACCCTAATTGATAGACACGGCGTAAAGAGTGGTTTGGGAAA

GCCCAAAAATAAAGCTAAAGACCTTCTAAGCCGTCACACGCACGCCGAAGGCACGAAGCC

CTGACACGAAAGTAGCTTTACTACTGCCCCTGACGCCACGAAAGCTAAGAAA

>45_Sample_ASV18

CACCGCGGTTATACGAGAGACCCTAATTGATAGACACGGCGTAAAGAGTGGTTTGGGAAA

GCCCAAAAATAAAGCTAAAGACCTTCTAAGCCGTCACACGCACGCCGAAGGCACGAAGCC

CTGACACGAAAGTAGCTTTACTATTACCCCCGACGCCACGAAAGCTAAGAAA

>45_Sample_ASV19

AACCGCGGTTATACGAGAGACCCTAATTGATAGCTCACGGCGTAAAGGGTGGTTTGGGGA

AACCTCAAAATAAAGCTAAAGACCTTCTAAGCCGTTACACGCATACCGAAGGCACGAGGC

CCTAACACGAAAGTAGCTTTACTATTACCCCTGACGCCACGAAAGCTAAGAAA

>45_Sample_ASV20

CACCGCGGTTATACGAGAGACCCTAATTGATAGACACGGCGTAAAGAGTGGTTTAGGACA

ACCCAAAAATAAAGCTAAAGACCTTCTAAGCCGTCACACGCACGCCGAAGGCACGAAGCC

CTGACACGAAAGTAGCTTTACTATTACCCCCGACGCCACGAAAGCTAAGAAA

>45_Sample_ASV21

CACCGCGGTTATACGAAAGACCCTAGTTGATGGATCACGGCGTAAAGGGTGGTTAAGGGA

TATAAAAATAAAGCTAAAAATCCTCTTAGCTGTCATACGCTTTCCGAAGACATGAGGCCC

AATTACGAAAGTAGCTTTAAACCACCCTGACACCACGAAAGCCAAGAAA

>45_Sample_ASV22

CACCGCGGTTATACGAAAGACCCTAGTTTATAGATGCGGCGTAAAGGGTGGTTAAGGAAA

ACCTAAAATAATGCTAAAGACTCTCAAAGCTGTTATACGCTTCATGAGCACACGAAATCC

AAATACGAAAGTTGCCTTAATAAACTTTGCCTGACCCCACGAAAGCTGAAAAA

>45_Sample_ASV23

CACCGCGGTTATACGAAAGGCTCTAGTTTATAAATACGGCGTAAAGGGTGGTTAAGGAAA

AATTAAAATAATGCTAAAGACCCCCTAAGCAGTTATACGCTTCACGGAGGCACGAAACCC

AAATACGAAAGTTGCCTTAATAAACTTTACCTGACCCCACGAAAGCTGAGAAA

>45_Sample_ASV24

CACCGCGGTTATACGAAAGGCTCTAGTTTATAAATACGGCGTAAAGGGTGGTTAAGGAAA

AATTAAAATAATGCTAAAGACCCCCTAAGCAGTTATACGCTTCACGGAGGCACGAAACCC

AAATACGAAAGTTGCCTTAATAAACTTTACCTGACCCCACGAAAGCTGAAAAA

>45_Sample_ASV25

CACCGCGGTTATACGAGAGGCCCTAGTTGATAACTACCGGCGTAAAGAGTGGTTATGGAA

AAATATTTAATAAAGCCGAACACCCCCTCAGCCGTCATACGCACCTGGGAGCACGAAGAC

CTACTGCGAAAGCAGCTTTAATTATGCCTGACCCCACGACAGCTAAGAAA

>45_Sample_ASV26

CACCGCGGTTATACGAGAGGCCCAAGTTGATTATTACCGGCGTAAAGCGTGGTTAAGATT

TTTATAGGACTAAAGCCGAACCCCCTCAGAGCTGTTATACGCACCCGAAGGTTAGAAGTT

CAATCACGAAAGTGGCTTTACAACCCCGAACCCACGAAAGCTAAGATA

>45_Sample_ASV27

CACCGCGGTTATACGAGAGACCCTAATTGATAGGCGCGGCGTAAAGAGTGGTTTAGGACT

AAAACTAATAAAGCTAAAGATCCTCCCAGCCGTCGCACGCATTCTGAGGACACGAAACTC

AACCACGAAAGTAGCTTTAACATACTCTCCTGACCCCACGAAAGCTAAGAAA

>45_Sample_ASV28

CACCGCGGTTATACGAAAGACCCTAGTTGATAGGCACGGCGTAAAGGGTGGTTAGGGATA

TATCAAAAATAAAGTTAAAGAGCCTCTAAGCTGTCGCACGCATCCCGAGAGCTCGAGACC

CAGACACGAAAGTAACTTTAAAATACCTCACCTGACCCCACGAAAGCTAAGAAA

>45_Sample_ASV29

CACCGCGGTTATACGAGAGGCCCAAGTTGATACTCCACGGCGTAAAGAGTGGTTAGAAAA

GACCTACTACTAAAGCCGAACGCCTTCAAAGCTGTTATACGCATCCGAAGGTGAGAAGCC

CACCCACGAAAGTGGCTTTACAACCTTGACTCCACGAAAGCTATGACA

>45_Sample_ASV30

CACCGCGGTTATACGAGAGACCCCAGTTGATAAACACGGCGTAAAGAGTGGTTAAGGGCA

CAAAACAAATAAAGTCAAACAACCTCTTAGCTGTTATACGCATTATGAGGATATGAAGCC

CCCCTACGAAAGTAACTTTACCAACCCCTGACCCCACGAAAGCTAAGAAA

>45_Sample_ASV31

CACCGCGGTTATACGAGAGACCCTAGTTGATATATACGGCGTAAAGAGTGGTTTGGAATA

ACCAAGTAATATAGCCAAAGACCTCCCAAGCCGTCACACGCACCCCGGAGGCACGAAGCC

CAAACACGAAAGTAGCTTTATTAACTTACCGACGCCACGAAAGCTAAGAAA

>45_Sample_ASV32

CACCGCGGTTATACGAAAGACCCTAGTTTATAGGCACGGCGTAAAGGGTGGTTAGGGGAT

AAACAAAATAAAGCCAAAGACCTTCTAAGTCGTCATACACTCATGAAGGCACGAAGCCCA

AATACGAAAGTAACTTTACAACTTAGACCCGACTCCACGAAAGCTGGGGGA

>45_Sample_ASV33

CACCGCGGTTATACGAGAGACCCTAATTAATAGATACGGCGTAAAGAGTGGTTTAGGACA

AAAATTAATAAAGCCGAAGTTTCTCCTAGCCGTTGTACGCACTCTGAGAACACGAAGCCC

CACTACGAAAGTAGCTTTACCTATTATTTCCTGACCCCACGAAAGCTAAGAAA

>45_Sample_ASV34

CACCGCGGTTATACGAGAGACCCTAATTAATAGCTACGGCGTAAAGAGTGGTTTAGGGTA

AAAATTAATAAAGCCGAAGTTTCTCCTAGCCGTTGTACGCACTCTGAGAACACGAAGCCC

CACTACGAAAGTAGCTTTACCTATTATTTCCTGACCCCACGAAAGCTAAGAAA

>45_Sample_ASV35

CACCGCGGTTATACGAGAGACCCTAATTAATAGATACGGCGTAAAGAGTGGTTTAGGGTA

AAAATTAATAAAGCCGAAGTTTCTCCTAGCCGTTGTACGCACTCTGAGAACACGAAGCCC

CACTACGAAAGTAGCTTTACCTATTATTTCCTGACCCCACGAAAGCTAAGAAA

>45_Sample_ASV36

CACCGCGGTTATACGAGAGGCCCAAGTTGATAGACATCGGCGTAAAGAGTGGTTAAGGAA

GTCTCAAAACTAAAGCCGAATGCCTTCAAGACTGTTATACGTTTCCGAAGGTAAGAAGCC

CCACTACGAAAGTGGCTTTACTCCACCTGACTCCACGAAAGCTGTGAAA

>45_Sample_ASV37

CACCGCGGTTATACGAGAGGCCCAAGTTGACAGATATCGGCGTAAAGAGTGGTTAAGGAA

ATCTTAAAACTAAAGCCGAATGCCTTCAAGACTGTTATACGTTTACGAAGGTAAGAAGCC

CCACTACGAAAGTGGCTTTACTCCACCTGACTCCACGAAAGCTGTGAAA

>45_Sample_ASV38

CACCGCGGTTATACGAGAGGCCCAAGTTGATAGACATCGGCGTAAAGAGTGGTTAAGGAA

GTCTCAAAACTAAAGCCGAATGCCTTCAAGACTGTTATACGTTTCCGAAGGTAAGAAGCC

CCACTACGAAAGTGGCTTTATTCCACCTGACCCCACGAAAGCTGTGAAA

>45_Sample_ASV39

CACCGCGGTTATACGAGAGGCCCAAGTTGATAGACATCGGCGTAAAGAGTGGTTAAGGAA

GTCTCAAAACTAAAGCCGAATGCCTTCAAGACTGTTATACGTTTACGAAGGTAAGAAGCC

CCACTACGAAAGTGGCTTTATTCCACCTGACCCCACGAAAGCTGTGAAA

>45_Sample_ASV40

CACCGCGGTTATACGAGAGGCCAAAGTTGACAGATATCGGCGTAAAGAGTGGTTAAGGAA

GTCTCAAAACTAAAGCCGAATGCCTTCAAGACTGTTATACGTTTCCGAAGGTAAGAAGCC

CCACTACGAAAGTGGCTTTACTCCACCTGACTCCACGAAAGCTGTGAAA

>45_Sample_ASV41

CACCGCGGTTATACGAGAGGCCCAAGTTGACAGATATCGGCGTAAAGAGTGGTTAAGGAA

ATCTTAAAACTAAAGCCGAATGCCTTCAAGACTGTTATACGTTTCCGAAGGTAAGAAGCC

CCACTACGAAAGTGGCTTTACTCCACCTGACTCCACGAAAGCTGTGAAA

>45_Sample_ASV42

CACCGCGGTTATACGAGAGGCCAAAGTTGACAGATATCGGCGTAAAGAGTGGTTAAGGAA

ATCTTAAAACTAAAGCCGAATGCCTTCAAGACTGTTATACGTTTCCGAAGGTAAGAAGCC

CCACTACGAAAGTGGCTTTACTCCACCTGACTCCACGAAAGCTGTGAAA

>45_Sample_ASV43

CACCGCGGTTATACGAGAGACCCTAATTGATAGACTACGGCGTAAAGAGTGGTTTAGAAC

AAAAATTAATAAAGCCAAAGATCCTCCTGGCCGTCGCACGCATTTCGAGGATACGAAGCC

CCACTACGAAAGTAGCTTTACTACAACTTTTCTGACCCCACGAAAGCTAAGAAA

>45_Sample_ASV44

CACCGCGGTTATACGAAAGACCCTAGTTGATAGATCACGGCGTAAAGGGTGGTTAAGGAA

AACAAAAATAAAGCTAAAAATCCTCTAAGCTGTCATACGCTTTCCGAAGACATGAGATCC

AACCACGAAAGTAGCTTTAAAATTTCCTGACACCACGAAAGCCAAGGAA

>46_Sample_ASV1

CACCGCGGTTATACGAGAGGCCCAAGTTGACAGACATCGGCGTAAAGAGTGGTTAAGGAA

AACCTTCAACTAAAGTCGAACGCTCTCAAGACTGTCGTACGTTTCCGAGAGTATGAAGCC

CCACCACGAAAGTGACTTTACCTCACCTGACTCCACGAAAGCTGCGAAA

>46_Sample_ASV2

CACCACGGTTATACGAGAGGCCCAAGTTGAAAGACATCGGCGTAAAGAGTGGTTAAGGAA

AACCTTCTACTAAAGTCGAACGCTCTCAGGACTGTCGTACGTTTCCGAGAGTATGAAGCC

CCACCACGAAAGTGACTTTACCTCACCTGACTCCACGAAAGCTGCGAAA

>46_2_Sample_ASV1

CACCGCGGTTATACGAAAGACCCTAGTTGATGGGTGCGGCGTAAAGGGTGGTTAGGGACT

ACAAAAAATAGAGCTAAAGATCCTCTAGGTCGTCATACACATCACGAGGAAACGAAGCCC

AAATACGAAAGTAGCTCTAAATATAATCCTGACGCCACGAAAGCCAAGAAA

>46_Sample_ASV3

CACCGCGGTTATACGAAAGACCCTAGTTGATGGGTGCGGCGTAAAGGGTGGTTAGGGACT

ACAAAAAATAGAGCTAAAGATCCTCTAGGTCGTCATACACATCACGAGGAAACGAAGCCC

AAATACGAAAGTAGCTCTAAATATAATCCTGACGCCACGAAAGCCAAGAAA

>46_2_Sample_ASV2

CACCGCGGTTATACGAGAGACCCTAGTTGATTAAAGCGGCGTAAAGAGTGGTTATGGAAC

TATTCTTTTAAAGCAGAAAACCTCTCAAACTGTTATACGCACCCAGAGGTCTGAATCCCT

CACACGAAAGTGACTTTATTTATGCCTACCAGAACCCACGAAAGCTGGGACA

>46_Sample_ASV4

CACCGCGGTTATACGAGAGACCCTAGTTGATTAAAGCGGCGTAAAGAGTGGTTATGGAAC

TATTCTTTTAAAGCAGAAAACCTCTCAAACTGTTATACGCACCCAGAGGTCTGAATCCCT

CACACGAAAGTGACTTTATTTATGCCTACCAGAACCCACGAAAGCTGGGACA

>46_Sample_ASV5

CACCGCGGTTATACGAGAGACCCTAGTTGATTAAAGCGGCGTAAAGAGTGGTTATGGAAC

TATTCTTTTAAAGCAGAAAACCTCTCAAACTGTTATACGCACCCAGAGGTCTGAATCCCT

CACACGAAAGTGACTTTATTTATGCCTACCAGAACCCACGAAAGCTATGACA

>46_Sample_ASV6

CACCGCGGTTATACGAAAGACCCTAGTTTATAGGTACGGCGTAAAGGGTGGTTAGGGGAC

AAACAAAATAAAGCCAAAGACCTCCTAAGCCGTCATACGCTCACGGAAGCACGAAGCCCA

AACACGAAAGTAGCTTTACCGAACATGCCCGACTCCACGAAAGCTGGGGAA

>46_2_Sample_ASV3

CACCGCGGTTATACGAGAGACCCTAGTTGATATACACGGCGTAAAGAGTGGTTTGGGGTA

TATAATAAATAAAGCCAAAGACCTTCTAGGCCGTTGAACGCACCACGAAGGAGCGAAGTC

CCAATACGAAAGTAGCTTTATAAAACCTGACGCCACGAAAGCTAAGAAA

>46_2_Sample_ASV4

CACCGCGGTTATACGAGAGGCCCAAGTTGACAGACACCGGCGTAAAGAGTGGCTAGGGAA

AAATTACTACTAAAGCCGAACACCTTCAGAACTGTCATACGTTCCCGAAGATAAGAAGCC

CCACCACGAAAGTGACTTTAACACTCCCGACCCCACGAAAGCTAAGAAA

>46_2_Sample_ASV5

CACCGCGGTTATACGAGAGGCCCAAGTTGACAGACACCGGCGTAAAGAGTGGCTAGGGAA

AAATTACTACTAAAGCCGAACACCTTCAGAACTGTCATACGTTCCCGAAGATAAGAAGCC

CCACCACGAAAGTGACTTTATATTACCCGACCCCACGAAAGCTGTGAAA

>46_Sample_ASV7

CACCGCGGTTATACGAGAGGCCCAAGTTGACAGACACCGGCGTAAAGAGTGGCTAGGGAA

AAATTACTACTAAAGCCGAACACCTTCAGAACTGTCATACGTTCCCGAAGATAAGAAGCC

CCACCACGAAAGTGACTTTATATTACCCGACCCCACGAAAGCTGTGAAA

>46_2_Sample_ASV6

CACCGCGGTTATACGAGAGGCCCAAGTTGACAGACACCGGCGTAAAGAGTGGCTAGGGAA

AAATTACTACTAAAGCCGAACACCTTCAGAACTGTCATACGTTCCCGAAGATAAGAAGCC

CCACCACGAAAGTGGCTTTATATTACCCGACCCCACGAAAGCTGTGAAA

>46_Sample_ASV8

CACCGCGGTTATACGAGAGGCCCAAGTTGACAGACACCGGCGTAAAGAGTGGCTAGGGAA

AAATTACTACTAAAGCCGAACACCTTCAGAACTGTCATACGTTCCCGAAGATAAGAAGCC

CCACCACGAAAGTGGCTTTATATTACCCGACCCCACGAAAGCTGTGAAA

>46_Sample_ASV9

CACCGCGGTTAAACGAGAGACCCTAGTTGATCATCACGGCGTAAAGAGTGGTTAGGGGAT

TATCATAAATAAAGCCAAAGACCTCCCAAGCTGTCGCACGCATTCCGGAGGCGCGAAGCC

CACACACGAAAGTAGCTTTAACTATTGACCCTGATGCCACGAAAGCTAAGAAA

>46_Sample_ASV10

CACCGCGGTTATACGAGAGACCCTAGTTGATATATACGGCGTAAAGAGTGGTTTGGGGTT

ATGCCTCCAAAATAAAGCCGAAGATCTCCCAAGCCGTCACACGCACCCCGGAGACACGAA

GCCCTAATACGAAGGTAGCTTTATTAATTCCCCGACGCCACGAAAGCTAAGAAA

>46_Sample_ASV11

CACCGCGGTTATACGAGAGACCCTAGTTGATATATACGGCGTAAAGAGTGGTTTGGGGTT

ATGCCTCCAAAATAAAGTCGAAGATCTCCCAAGCCGTCACACGCACCCCGGAGACACGAA

GCCCTAATACGAAGGTAGCTTTATTAATTCCCCGACGCCACGAAAGCTAAGAAA

>46_Sample_ASV12

CACCGCGGTTATACGAGAGACCCTAGTTGATATATACGGCGTAAAGAGTGGTTTGGGATA

TTTCTCCAAAATAAAGCCGAAGATCTCCCAAGCCGTCACACGCATCCCGGAGACACGAAG

CCCTAATACGAAGGTAGCTTTATTAGTACCCCGACGCCACGAAAGCTAAGAAA

>46_Sample_ASV13

CACCGCGGTTATACGAGAGACCCTAGTTGATATATACGGCGTAAAGAGTGGTTTGGGGTT

ATGCCTCCAAAATAAAGCCGAAGATCTCCCAAGCCGTCACACGCATCCCGGAGACACGAA

GCCCTAATACGAAGGTAGCTTTATTAATTCCCCGACGCCACGAAAGCTAAGAAA

>46_Sample_ASV14

CACCGCGGTTATACGAGAGACCCTAGTTGATATATACGGCGTAAAGAGTGGTTTGGGGTT

ATGCCTCCAAAATAAAGCCGAAGATCTCCCAAGCCGTCACACGCATCCCGGAGACACGAA

GCCCTAATACGAAGGTAGCTTTATTAGTACCCCGACGCCACGAAAGCTAAGAAA

>46_Sample_ASV15

CACCGCGGTTATACGAGAGGCCCAAGTTGATAAGCCCGGCGTAAAGAGTGGTTATGGAGA

ATAAAACACTAAAGCTAAAGACCCCCTAGGCTGTTATACGCACCTGGCGGCTCGAACCAC

CTATACGAAAGTAGCTTTACCCCCCTTCCACCAGAATCCACGACAGCTGGGGCA

>46_2_Sample_ASV7

CACCGCGGTTATACGAGAGGCCCAAGTTGATAAGCCCGGCGTAAAGAGTGGTTATGGAGA

ATAAAACACTAAAGCTAAAGACCCCCTAGGCTGTTATACGCACCTGGCGGCTCGAACCAC

CTATACGAAAGTAGCTTTACCCCCCTTCCACCAGAATCCACGACAGCTGGGGCA

>46_Sample_ASV16

CACCGCGGTTATACGAGAGGCCCAAGTTGACAGACATCGGCGTAAAGAGTGGTTAAGGAA

ACCCCATTACTAAAGCCGAACGCCCTCAGAACTGTCATACGTTCCCGAAGGTAAGAAGCC

CCACTACGAAAGTGGCTTTAACCACACCCGACCCCACGAAAGCTGTGAAA

>46_2_Sample_ASV8

CACCGCGGTTATACGAGAGGCCCAAGTTGACAGACATCGGCGTAAAGAGTGGTTAAGGAA

ACCCCATTACTAAAGCCGAACGCCCTCAGAACTGTCATACGTTCCCGAAGGTAAGAAGCC

CCACTACGAAAGTGGCTTTAACCACACCCGACCCCACGAAAGCTGTGAAA

>46_2_Sample_ASV9

CACCGCGGTTATACGAGAGGCCCAAGTTGATAGATATCGGCGTAAAGAGTGGTTAAGGAA

ACCCCATTACTAAAGCCGAACGCCCTCAGAACTGTCATACGTTCCCGAAGGTAAGAAGCC

CCACTACGAAAGTGGCTTTAACCACACCCGACCCCACGAAAGCTGTGAAA

>46_Sample_ASV17

CACCGCGGTTATACGAGAGGCCCAAGTTGATAGATATCGGCGTAAAGAGTGGTTAAGGAA

ACCCCGTTACTAAAGCCGAACGCCCTCAGAACTGTCATACGTTCCCGAAGGTAAGAAGCC

CCACTACGAAAGTGGCTTTAACCACACCCGACCCCACGAAAGCTGTGAAA

>46_2_Sample_ASV10

CACCGCGGTTATACGAGAGGCCCAAGTTGACAGACACCGGCGTAAAGAGTGGTTAAGGAA

ATTCATCAACTAAAGCCGAATGCCCTCAGGACTGTTATACGTTTGCGAAGGTAAGAAGCC

CTACTACGAAAGTGGCTTTATTCCACCTGATTCCACGAAAGCTGCGGAA

>46_2_Sample_ASV11

CACCGCGGTTATACGAGAGGCCCCAGTTGATATTAGCGGCGTAAAGGGTGGTTAAGGACT

TTAATAACTAAAGCTAAACACTTCCCCGGCTGTTGTACGTAGACCGGAAGCTTGAAGCCC

AGACACGAAAGTAGCTTTATTCCCCCTGACCCCACGAAAGCTGAGAAA

>46_Sample_ASV18

CACCGCGGTTATACGAGAGGCCCCAGTTGATATTAGCGGCGTAAAGGGTGGTTAAGGACT

TTAATAACTAAAGCTAAACACTTCCCCGGCTGTTGTACGTAGACCGGAAGCTTGAAGCCC

AGACACGAAAGTAGCTTTATTCCCCCTGACCCCACGAAAGCTGAGAAA

>46_Sample_ASV19

CACCGCGGTTATACGAGAGGCCCTAGTTGATAAATACGGCGTAAAGAGTGGTTAGAGGAC

TCCCATAATAAAGCCAAAGACCCCCCGGGCCGTCACACGCATACGAGGGCACGAAGTTCA

CACACGAAAGTAGCTTTAAATTACCCGACGCCACGAAAGCTAAGAAA

>46_2_Sample_ASV12

CACCGCGGTTATACGAGAAACCCTAGTTGACAAATACGGCGTAAAGAGTGGTTAAGGGGT

ATCAAAAATAAAGTCAAACATTTTCCTAGTTGTTATAAGCATTTCGAGAACATGAAGCCC

AACCACGAAAGTAACTTTACCTTCACCTGACCCCACGAAAGCTAAGAAA

>46_Sample_ASV20

GACCGCGGTTATACGAGAGACTCTAGTTGATAGATACGGCGTAAAGAGTGGTTTGGGATA

AACTATAAATAAAGCCAAAGACCTTCCAAGCTGTTAAACGCATACCGAAGGCACGAAGCC

CCAACACGAAAGTAGCTTTACTACTACCCCCGATGCCACGAAAGCTAAGACA

>46_2_Sample_ASV13

CACCGCGGTTATACGAGAGACCCTAGTTGATAAATACGGCGTAAAGAGTGGTTATGGGAA

AAACAAATAAAGTCAAACAACCTCTTAGCTGTTATACGCATTATGAGAGCATGAAGCCCC

CTCACGAAAGTAACTTTAATACCTCCTGACCCCACGAAAGCTAAGAAA

>46_Sample_ASV21

CACCGCGGTTATACGAGAGACCCTAGTTGATAAATACGGCGTAAAGAGTGGTTATGGGAA

AAACAAATAAAGTCAAACAACCTCTTAGCTGTTATACGCATTATGAGAGCATGAAGCCCC

CTCACGAAAGTAACTTTAATACCTCCTGACCCCACGAAAGCTAAGAAA

>46_2_Sample_ASV14

AACCGCGGTTATACGAGAGACCCTAATTGATAGCTCACGGCGTAAAGGGTGGTTTGGGGA

AACCTCAAAATAAAGCTAAAGACCTTCTAAGCCGTTACACGCATACCGAAGGCACGAGGC

CCTAACACGAAAGTAGCTTTACTATTACCCCTGACGCCACGAAAGCTAAGAAA

>46_2_Sample_ASV15

CACCGCGGTTATACGAGAGACCCTAATTGATAGGTACGGCGTAAAGAGTGGTTTGGGATA

ACCCAAAAATAAAGCTAAAGACCTTCTAAGCCGTCACACGCATACCGAAGGCACGAGGCC

CCAACACGAAAGTAGCTTTACTATTACCCCGACGCCACGAAAGCTAAGAAA

>46_2_Sample_ASV16

CACCGCGGTTATACGAGAGACCCTAATTGATAGGCACGGCGTAAAGAGTGGTTTAGGGTA

ACCCATAAATAAAGCTAAAGACCTTCTAAGCCGTCACACGCACACCGAAGGCACGAGACC

CCAACACGAAAGTAGCTTTACTTCTACCCCTGACGCCACGAAAGCTAAGAAA

>46_2_Sample_ASV17

CACCGCGGTTATACGAAAGACCCTAGTTGATGGATCACGGCGTAAAGGGTGGTTAAGGGA

TATAAAAATAAAGCTAAAAATCCTCTTAGCTGTCATACGCTTTCCGAAGACATGAGGCCC

AATTACGAAAGTAGCTTTAAACCACCCTGACACCACGAAAGCCAAGAAA

>46_Sample_ASV22

CACCGCGGTTATACGAAAGACCCCAGTTTATAGATGCGGCGTAAAGGGTGGTTAAGAGAA

AATCAAAATAAGGCCAAAGATCCCCTAAGCAGTCATACGCTCCACGAGGACACGAAACCC

AAATACGAAAGTTGCCTTAATAAACTTCACCTGATCCCACGAAAGCTGAAAAA

>46_Sample_ASV23

CACCGCGGTCATACGATTAACCCAAGTTAATAGGCACACGGCGTAAAGCGTGTTTAAGCA

CTATGCCAAATAAAGTTAAACTCCAATTAAGCTGTAAAAAGCCATAATCAAGACGAAAAT

AAATAACGAAAGTAACTTTACAACCGCTGAAACACGATAGCTAAGATC

>46_Sample_ASV24

CACCGCGGTTATACGAAAGACCCTAGTTTATAGACACGGCGTAAAGGGTGGTTCGGGACT

AAACAAAATAAAGCCAAAGACCTCCTAAGCCGTCATACGCTCACGGAAGCACGAAGCCCA

AGTACGAAAGTAGCTTTACAAAACATGCCTGACTCCACGAAAGCTAGGAAA

>46_Sample_ASV25

CACCGCGGTTATACGAAAGACCCTAGTTGATAGGCACGGCGTAAAGGGTGGTTAGGGATA

TATCAAAAATAAAGTTAAAGAGCCTCTAAGCTGTCGCACGCATCCCGAGAGCTCGAGACC

CAGACACGAAAGTAACTTTAAAATACCTCACCTGACCCCACGAAAGCTAAGAAA

>46_Sample_ASV26

CACCGCGGTTATACGAAAGACCCTAGTTGATAGCCACGGCGTAAAGGGTGGTTAAGGTAT

ACAACAAATAAAGCTAAATAGCCTCTAGGCCGTCGCACGCATTCCGAGAGCTCGAAACCC

AAAAACGAAAGTAGCTTTAAAATAAACAGACCTGACCCCACGAAAGCTAAGAAA

>46_2_Sample_ASV18

CACCGCGGTTATACGAGAGGCCCAAGTTGATACTCCACGGCGTAAAGAGTGGTTAGAAAA

GACCTACTACTAAAGCCGAACGCCTTCAAAGCTGTTATACGCATCCGAAGGTGAGAAGCC

CACCCACGAAAGTGGCTTTACAACCTTGACTCCACGAAAGCTATGACA

>46_Sample_ASV27

CACCGCGGTTATACGAGAGGCCCAAGTTGATACTCCACGGCGTAAAGAGTGGTTAGAAAA

GACCTACTACTAAAGCCGAACGCCTTCAAAGCTGTTATACGCATCCGAAGGTGAGAAGCC

CACCCACGAAAGTGGCTTTACAACCTTGACTCCACGAAAGCTATGACA

>46_Sample_ASV28

CACCGCGGTTATACGAGAGACCCCAGTTGATAAACACGGCGTAAAGAGTGGTTAAGGGCA

CAAAACAAATAAAGTCAAACAACCTCTTAGCTGTTATACGCATTATGAGGATATGAAGCC

CCCCTACGAAAGTAACTTTACCAACCCCTGACCCCACGAAAGCTAAGAAA

>46_2_Sample_ASV19

CACCGCGGTTATACGAGAGACCCCAGTTGATAAACACGGCGTAAAGAGTGGTTAAGGGCA

CAAAACAAATAAAGTCAAACAACCTCTTAGCTGTTATACGCATTATGAGGATATGAAGCC

CCCCTACGAAAGTAACTTTACCAACCCCTGACCCCACGAAAGCTAAGAAA

>46_Sample_ASV29

CACCGCGGTTATACGAAAGACCCTAGTTTATAGGCACGGCGTAAAGGGTGGTTAGGGGAT

AAACAAAATAAAGCCAAAGACCTTCTAAGTCGTCATACACTCATGAAGGCACGAAGCCCA

AATACGAAAGTAACTTTACAACTTAGACCCGACTCCACGAAAGCTGGGGGA

>46_2_Sample_ASV20

CACCGCGGTTATACGAAAGACCCTAGTTTATAGGCACGGCGTAAAGGGTGGTTAGGGGAT

AAACAAAATAAAGCCAAAGACCTTCTAAGTCGTCATACACTCATGAAGGCACGAAGCCCA

AATACGAAAGTAACTTTACAACTTAGACCCGACTCCACGAAAGCTGGGGGA

>46_2_Sample_ASV21

CACCGCGGTTATACGAGAGACCCTAATTAATAGATACGGCGTAAAGAGTGGTTTAGGACA

AAAATTAATAAAGCCGAAGTTTCTCCTAGCCGTTGTACGCACTCTGAGAACACGAAGCCC

CACTACGAAAGTAGCTTTACCTATTATTTCCTGACCCCACGAAAGCTAAGAAA

>46_2_Sample_ASV22

CACCGCGGTTATACGAGAGGCCCAAGTTGACAGATATCGGCGTAAAGAGTGGTTAAGGAA

GTCTCAAAACTAAAGCCGAATGCCTTCAAGACTGTTATACGTTTCCGAAGGTAAGAAGCC

CCACTACGAAAGTGGCTTTACTCCACCTGACTCCACGAAAGCTGTGAAA

>46_2_Sample_ASV23

CACCGCGGTTATACGAGAGGCCCAAGTTGATAGACATCGGCGTAAAGAGTGGTTAAGGAA

GTCTCAAAACTAAAGCCGAATGCCTTCAAGACTGTTATACGTTTACGAAGGTAAGAAGCC

CCACTACGAAAGTGGCTTTATTCCACCTGACCCCACGAAAGCTGTGAAA

>46_2_Sample_ASV24

CACCGCGGTTATACGAGAGGCCCAAGTTGATAGACATCGGCGTAAAGAGTGGTTAAGGAA

GTCTCAAAACTAAAGCCGAATGCCTTCAAGACTGTTATACGTTTCCGAAGGTAAGAAGCC

CCACTACGAAAGTGGCTTTACTCCACCTGACTCCACGAAAGCTGTGAAA

>46_Sample_ASV30

CACCGCGGTTATACGAGAGACCCTAATTGATAGACTACGGCGTAAAGAGTGGTTTAGAAC

AAAAATTAATAAAGCCAAAGATCCTCCTGGCCGTCGCACGCATTTCGAGGATACGAAGCC

CCACTACGAAAGTAGCTTTACTACAACTTTTCTGACCCCACGAAAGCTAAGAAA

>46_Sample_ASV31

CACCGCGGTTATACGAAAGACCCTAGTTGATAGATCACGGCGTAAAGGGTGGTTAAGGAG

AACAAAAATAAAGCTAAAAATCTTCTAAGCTGTCATACGCTTTCCGAAGACATGAGACCC

AACCACGAAAGTAGCTTTAAACTTTCCTGACACCACGAAAGCCAAGAAA

>111_Sample_ASV1

CACCGCGGTTATACGAGAGGCCCAAGTTGACAGACATCGGCGTAAAGAGTGGTTAAGGAA

AACCTTCAACTAAAGTCGAACGCTCTCAAGACTGTCGTACGTTTCCGAGAGTATGAAGCC

CCACCACGAAAGTGACTTTACCTCACCTGACTCCACGAAAGCTGCGAAA

>111_Sample_ASV2

CACCGCGGTTATACGAAAGACCCTAGTTGATGGGTGCGGCGTAAAGGGTGGTTAGGGACT

ACAAAAAATAGAGCTAAAGATCCTCTAGGTCGTCATACACATCACGAGGAAACGAAGCCC

AAATACGAAAGTAGCTCTAAATATAATCCTGACGCCACGAAAGCCAAGAAA

>111_Sample_ASV3

CACCGCGGTTAAACGAGAGGCCCTAGTTGATGGTGCAACGGCGTAAAGGGTGGTTAAGGA

TAGCGAAATAATAAAGTCAAATGGCCCTCTGGCTGTCATACGCTTCTAGGAGTCCGAAGC

CCAGTATACGAAAGTAGCTTTAAGAAAGTCCACCTGACCCCACGAAAACTGAGGAA

>111_Sample_ASV4

CACCGCGGTTATACGAAAGACCCTAGTTGCTAGCCACGGCGTAAAGGGTGGTTAAGGACA

ACAACAAATAAAGCTAAAGATCCCCTAAGCCGTCATACGCATTCCGGGGGCACGAAGCCC

TAACACGAAAGTAGCTTTAAAAAATATACCTGACCCCACGAAAGCTAAGAAA

>111_Sample_ASV5

CACCGCGGTTATACGAGAGACCCTAGTTGATTAAAGCGGCGTAAAGAGTGGTTATGGAAC

TATTCTTTTAAAGCAGAAAACCTCTCAAACTGTTATACGCACCCAGAGGTCTGAATCCCT

CACACGAAAGTGACTTTATTTATGCCTACCAGAACCCACGAAAGCTGGGACA

>111_Sample_ASV6

CACCGCGGTTATACGAGAGACCCTAGTTGATTAAAGCGGCGTAAAGAGTGGTTATGGAAC

TATTCTTTTAAAGCAGAAAACCTCTCAAACTGTTATACGCACCCAGAGGTCTGAATCCCT

CACACGAAAGTGACTTTATTTATGCCTACCAGAACCCACGAAAGCTATGACA

>111_Sample_ASV7

CACCGCGGTTATACGAAAGACCCTAGTTTATAGGTACGGCGTAAAGGGTGGTTAGGGGAC

AAACAAAATAAAGCCAAAGACCTCCTAAGCCGTCATACGCTCACGGAAGCACGAAGCCCA

AACACGAAAGTAGCTTTACCGAACATGCCCGACTCCACGAAAGCTGGGGAA

>111_Sample_ASV8

CACCGCGGTTATACGAGAGGCCCAAGTTGACAGACACCGGCGTAAAGAGTGGCTAGGGAA

AAATTACTACTAAAGCCGAACACCTTCAGAACTGTCATACGTTCCCGAAGATAAGAAGCC

CCACCACGAAAGTGGCTTTATATTACCCGACCCCACGAAAGCTAAGACA

>111_Sample_ASV9

CACCGCGGTTATACGAGAGGCCCAAGTTGACAGACACCGGCGTAAAGAGTGGCTAGGGAA

AAATTACTACTAAAGCCGAACACCTTCAGAACTGTCATACGTTCCCGAAGATAAGAAGCC

CCACCACGAAAGTGGCTTTATATTACCCGACCCCACGAAAGCTGTGAAA

>111_Sample_ASV10

CACCGCGATTATACGAGAGGCCCAAGTTGACAGACATCGGCGTAAAGAGTGGTTAAGGAA

AAATTACTACTAAAGCCGAACACCTTCAGAACTGTCATACGTTCCCGAAGATAAGAAGCC

CCACCACGAAAGTGGCTTTATATTACCCGACCCCACGAAAGCTGTGAAA

>111_Sample_ASV11

CACCGCGGTTATACGAAAGACCCTAGTTTATAGAAACGGCGTAAAGGGTGGTTAAGGGAC

AAACAAAATAAGGCCAAAGACTTCCTAAGCCGTCATACGCTCCACGAAAGCACGAAGCCC

AAACACGAAAGTAGCCTTAATAACTTCACCTGACCCCACGAAAGCTGGGAGA

>111_Sample_ASV12

CACCGCGGTTAAACGAGAGACCCTAGTTGATCATCACGGCGTAAAGAGTGGTTAGGGGAT

TATCATAAATAAAGCCAAAGACCTCCCAAGCTGTCGCACGCATTCCGGAGGCGCGAAGCC

CACACACGAAAGTAGCTTTAACTATTGACCCTGATGCCACGAAAGCTAAGAAA

>111_Sample_ASV13

CACCGCGGTTATACGAGAGGCCCAAGTTGATAAGCCCGGCGTAAAGAGTGGTTATGGAGA

ATAAAACACTAAAGCTAAAGACCCCCTAGGCTGTTATACGCACCTGGCGGCTCGAACCAC

CTATACGAAAGTAGCTTTACCCCCCTTCCACCAGAATCCACGACAGCTGGGGCA

>111_Sample_ASV14

CACCGCGGTTATACGAGAGGCCCAAGTTGACAGACATCGGCGTAAAGAGTGGTTAAGGAA

ACCCCATTACTAAAGCCGAACGCCCTCAGAACTGTCATACGTTCCCGAAGGTAAGAAGCC

CCACTACGAAAGTGGCTTTAACCACACCCGACCCCACGAAAGCTGTGAAA

>111_Sample_ASV15

CACCGCGGTTATACGAGAGGCCCAAGTTGATAGATATCGGCGTAAAGAGTGGTTAAGGAA

ACCCCGTTACTAAAGCCGAACGCCCTCAGAACTGTCATACGTTCCCGAAGGTAAGAAGCC

CCACTACGAAAGTGGCTTTAACCACACCCGACCCCACGAAAGCTGTGAAA

>111_Sample_ASV16

CACCGCGGTTAAACGAGAGGCCCTAGTTGATATTACAACGGCGTAAAGGGTGGTTAAGGA

AAGCAAGCAATAAAGCCGAATGGCCCTTTGGCTGTCATACGCTTCTAGGTGTCCGAAGCC

CAATATACGAAAGTAGCTTTAGAAAAGCCCACCTGACACCACGAAAGCTGAGGAA

>111_Sample_ASV17

CACCGCGGTTATACGAGAGGCCCTAGTTGATAAATACGGCGTAAAGAGTGGTTAGAGGAC

TCCCATAATAAAGCCAAAGACCCCCCGGGCCGTCACACGCATACGAGGGCACGAAGTTCA

CACACGAAAGTAGCTTTAAATTACCCGACGCCACGAAAGCTAAGAAA

>111_Sample_ASV18

GACCGCGGTTATACGAGAGACTCTAGTTGATAGATACGGCGTAAAGAGTGGTTTGGGATA

AACTATAAATAAAGCCAAAGACCTTCCAAGCTGTTAAACGCATACCGAAGGCACGAAGCC

CCAACACGAAAGTAGCTTTACTACTACCCCCGATGCCACGAAAGCTAAGACA

>111_Sample_ASV19

CACCGCGGTTATACGAAAGACCCCAGTTTATAGACACGGCGTAAAGGGTGGTTATAGGAC

AAACTAACATAGAGCCAAAGACCCCCTAAGCCGTCATACGCTCACGAGGGCTCGAAGCCC

CTATACGAAAGTAGCTCTACCAAAAAATGCTCGACTCCACGAAAGCTGGGACA

>111_Sample_ASV20

CACCGCGGTTATACGAGAGACCCTAGTTGATAAATACGGCGTAAAGAGTGGTTATGGGAA

AAACAAATAAAGTCAAACAACCTCTTAGCTGTTATACGCATTATGAGAGCATGAAGCCCC

CTCACGAAAGTAACTTTAATACCTCCTGACCCCACGAAAGCTAAGAAA

>111_Sample_ASV21

CACCGCGGTTATACGAGAGGCTCAAGTTGATAAACCCCGGCGTAAAGAGTGGTTAAGGAA

AAATAGAAACTAAAGCCGAATGCTTTCAAGACTGTTATACGTTCCCGAGAGTAAGAAGAC

CAATTACGAAAGTGGCTTTACTTTACCTGACCCCACGAAAGCTACGACA

>111_Sample_ASV22

CACCGCGGTTATACGAGAGGCTCAAGTTGATAAACCCCGGCGTAAAGAGTGGTTAAGGAA

AAATAGAAACTAAAGCCGAATGCTTTCAAGACTGTTATACGTTCCCGAGAGTAAGAAGAC

CAATTACGAAAGTGGCTTTACTTTACCTGACCCCACGAAAGCTAAGACA

>111_Sample_ASV23

CACCGCGGTCATACGATTAACCCAAGTTAATAGGCACACGGCGTAAAGCGTGTTTAAGCA

CTATGCCAAATAAAGTTAAACTCCAATTAAGCTGTAAAAAGCCATAATCAAGACGAAAAT

AAATAACGAAAGTAACTTTACAACCGCTGAAACACGATAGCTAAGATC

>111_Sample_ASV24

CACCGCGGTTATACGAAAGACCCTAGTTTATAGACACGGCGTAAAGGGTGGTTCGGGACT

AAACAAAATAAAGCCAAAGACCTCCTAAGCCGTCATACGCTCACGGAAGCACGAAGCCCA

AGTACGAAAGTAGCTTTACAAAACATGCCTGACTCCACGAAAGCTAGGAAA

>111_Sample_ASV25

CACCGCGGTTATACGAAAGACCCTAGTTGATAGGCACGGCGTAAAGGGTGGTTAGGGATA

TATCAAAAATAAAGTTAAAGAGCCTCTAAGCTGTCGCACGCATCCCGAGAGCTCGAGACC

CAGACACGAAAGTAACTTTAAAATACCTCACCTGACCCCACGAAAGCTAAGAAA

>111_Sample_ASV26

CACCGCGGTTATACGAAAGACCCTAGTTGATAGCCACGGCGTAAAGGGTGGTTAAGGTAT

ACAACAAATAAAGCTAAATAGCCTCTAGGCCGTCGCACGCATTCCGAGAGCTCGAAACCC

AAAAACGAAAGTAGCTTTAAAATAAACAGACCTGACCCCACGAAAGCTAAGAAA

>111_Sample_ASV27

CACCGCGGTTATACGAGAGGCCCAAGTTGATACTCCACGGCGTAAAGAGTGGTTAGAAAA

GACCTACTACTAAAGCCGAACGCCTTCAAAGCTGTTATACGCATCCGAAGGTGAGAAGCC

CACCCACGAAAGTGGCTTTACAACCTTGACTCCACGAAAGCTGGGACA

>111_Sample_ASV28

CACCGCGGTTATACGAGAGGCCCAAGTTGATACTCCACGGCGTAAAGAGTGGTTAGAAAA

GACCTACTACTAAAGCCGAACGCCTTCAAAGCTGTTATACGCATCCGAAGGTGAGAAGCC

CACCCACGAAAGTGGCTTTACAACCTTGACTCCACGAAAGCTATGACA

>111_Sample_ASV29

CACCGCGGTTATACGAGAGACCCCAGTTGATAAACACGGCGTAAAGAGTGGTTAAGGGCA

CAAAACAAATAAAGTCAAACAACCTCTTAGCTGTTATACGCATTATGAGGATATGAAGCC

CCCCTACGAAAGTAACTTTACCAACCCCTGACCCCACGAAAGCTAAGAAA

>111_Sample_ASV30

CACCGCGGTTATACGAAAGACCCTAGTTTATAGGCACGGCGTAAAGGGTGGTTAGGGGAT

AAACAAAATAAAGCCAAAGACCTTCTAAGTCGTCATACACTCATGAAGGCACGAAGCCCA

AATACGAAAGTAACTTTACAACTTAGACCCGACTCCACGAAAGCTGGGGGA

>111_Sample_ASV31

CACCGCGGTTATACGAGAGGCCCAAGTTGACAAACTCCGGCGTAAAGCGTGGTTAAGGCA

CACTCAAAAACTAAAGCCGAATATCTTCAGAGCAGTCATACGCATCCGAAGACACGAAGC

CCCACCACGAAAGTGGCTTTATGACACCTGACCCCACGAAAGCTAGGACA

>111_Sample_ASV32

CACCGCGGTTATACGAGAGGCCCAAGTTGACAAACTCCGGCGTAAAGCGTGGTTAAGGCA

CACTCAAAAACTAAAGCCGAATATCTTCAGAGCAGTCATACGCATCCGAAGACACGAAGC

CCCACCACGAAAGTGGCTTTATGACACCTGACCCCACGAAAGCTAAGACA

>111_Sample_ASV33

CACCGCGGTTATACGAAAGACCCTAGTTGATAGATCACGGCGTAAAGGGTGGTTAAGGAG

AACAAAAATAAAGCTAAAAATCTTCTAAGCTGTCATACGCTTTCCGAAGACATGAGACCC

AACCACGAAAGTAGCTTTAAACTTTCCTGACACCACGAAAGCCAAGAAA

>111_Sample_ASV34

CACCGCGGTTATACGAAAGACCCTAGTTGATAGATCACGGCGTAAAGGGTGGTTAAGGAA

AACAAAAATAAAGCTAAAAATCCTCTAAGCTGTCATACGCTTTCCGAAGACATGAGATCC

AACCACGAAAGTAGCTTTAAAATTTCCTGACACCACGAAAGCCAAGGAA

>113_Sample_ASV1

CACCGCGGTTATACGAAAGACCCTAGTTGATGGGTGCGGCGTAAAGGGTGGTTAGGGACT

ACAAAAAATAGAGCTAAAGATCCTCTAGGTCGTCATACACATCACGAGGAAACGAAGCCC

AAATACGAAAGTAGCTCTAAATATAATCCTGACGCCACGAAAGCCAAGAAA

>113_Sample_ASV2

CACCGCGGTTATACGAGAGACCCTAGTTGATTAAAGCGGCGTAAAGAGTGGTTATGGAAC

TATTCTTTTAAAGCAGAAAACCTCTCAAACTGTTATACGCACCCAGAGGTCTGAATCCCT

CACACGAAAGTGACTTTATTTATGCCTACCAGAACCCACGAAAGCTGGGACA

>113_Sample_ASV3

CACCGCGGTTATACGAAAGACCCTAGTTTATAGGTACGGCGTAAAGGGTGGTTAGGGGAC

AAACAAAATAAAGCCAAAGACCTCCTAAGCCGTCATACGCTCACGGAAGCACGAAGCCCA

AACACGAAAGTAGCTTTACCGAACATGCCCGACTCCACGAAAGCTGGGGAA

>113_Sample_ASV4

CACCGCGGTTATACGAGAGGCCCAAGTTGACAGACACCGGCGTAAAGAGTGGCTAAGGAA

AAATTACTACTAAAGCCGAACACCTTCAGAACTGTCATACGTTCCCGAAGATAAGAAGCC

CCACCACGAAAGTGGCTTTATATTACCCGACCCCACGAAAGCTGTGAAA

>113_Sample_ASV5

CACCGCGGTTATACGAGAGGCCCAAGTTGACAGACACCGGCGTAAAGAGTGGCTAGGGAA

AAATTACTACTAAAGCCGAACACCTTCAGAACTGTCATGCGTTCCCGAAGATAAGAAGCC

CCACCACGAAAGTGGCTTTATATTACCCGACCCCACGAAAGCTGTGAAA

>113_Sample_ASV6

CACCGCGGTTATACGAGAGGCCCAAGTTGACAGACACCGGCGTAAAGAGTGGCTAGGGAA

AAATTACTACTAAAGCCGAACACCTTCAGAACTGTCATACGTTCCCGAAGATAAGAAGCC

CCACCACGAAAGTGGCTTTATATTACCCGACCCCACGAAAGCTGTGAAA

>113_Sample_ASV7

CACCGCGGTTATACGAGAGGCCCAAGTTGACAGACACCGGCGTAAAGAGTGGCTAAGGAA

AAATTTCTACTAAAGCCGAACACCTTCAGAACTGTCATACGTTCCCGAAGAAAAGAAGCC

CCACCACGAAAGTGGCTTTATATTACCCGACCCCACGAAAGCTGTGAAA

>113_Sample_ASV8

CACCGCGGTTAAACGAGAGACCCTAGTTGATCATCACGGCGTAAAGAGTGGTTAGGGGAT

TATCATAAATAAAGCCAAAGACCTCCCAAGCTGTCGCACGCATTCCGGAGGCGCGAAGCC

CACACACGAAAGTAGCTTTAACTATTGACCCTGATGCCACGAAAGCTAAGAAA

>113_Sample_ASV9

CACCGCGGTTATACGAGAGACCCTAGTTGATATATACGGCGTAAAGAGTGGTTTGGGATA

TTTCTCCAAAATAAAGTCGAAGATCTCCCAAGCCGTCACACGCATCCCGGAGACACGAAG

CCCTGATACGAAGGTAGCTTTATTAACACCCCGACGCCACGAAAGCTAAGAAA

>113_Sample_ASV10

CACCGCGGTTATACGAGAGACCCTAGTTGATATATACGGCGTAAAGAGTGGTTTGGGATA

TTTCTCCAAAATAAAGCCGAAGATCTCCCAAGCCGTCACACGCATCCCGGAGACACGAAG

CCCTGATACGAAGGTAGCTTTATTAACACCCCGACGCCACGAAAGCTAAGAAA

>113_Sample_ASV11

CACCGCGGTTATACGAGAGACCCTAGTTGATATATACGGCGTAAAGAGTGGTTTGGGATA

TATCCAGAATAAAGCCGAAGATCTCCCAAGCCGTCACACGCATCCCGGAGACACGAAGCC

CTGATACGAAGGTAGCTTTATTAACACCCCGACGCCACGAAAGCTAAGAAA

>113_Sample_ASV12

CGCCGCGGTTAAACGAGAGGCCCTAGTTGATGGAACAACGGCGTAAAGGGTGGTTAAGGA

TAGTGAGACAATAAAGTCGAATGGCCCTTTGGCTGTCATACGCTTCTGGGAGCCTGAAGC

CCACTATACGAAAGTAGCTTTAACAATGTCCACCTGACCCCACGAAAACTGAGAAA

>113_Sample_ASV13

CACCGCGGTTATACGAAAGACCCTAGTTGATAGATCACGGCGTAAAGGGTGGTTAGGGAA

TACAAAAATAAAGCTAAAAATCCTCTAAGCTGTCATACGCTTCCCGAAGACATGAAACCC

AACCACGAAAGTAGCTTTAAACTCTCCTGACACCACGAAAGCCAAGGAA

>113_Sample_ASV14

CACCGCGGTTATACGAGAGGCCCAAGTTGATAAGCCCGGCGTAAAGAGTGGTTATGGAGA

ATAAAACACTAAAGCTAAAGACCCCCTAGGCTGTTATACGCACCTGGCGGCTCGAACCAC

CTATACGAAAGTAGCTTTACCCCCCTTCCACCAGAATCCACGACAGCTGGGGCA

>113_Sample_ASV15

CACCGCGGTTATACGAGAGGCCCAAGTTGACAGACATCGGCGTAAAGAGTGGTTAAGGAA

ACCCCATTACTAAAGCCGAACGCCCTCAGAACTGTCATACGTTCCCGAAGGTAAGAAGCC

CCACTACGAAAGTGGCTTTAACCACACCCGACCCCACGAAAGCTGTGAAA

>113_Sample_ASV16

CACCGCGGTTATACGAGAGGCCCAAGTTGATAGATATCGGCGTAAAGAGTGGTTAAGGAA

ACCCCGTTACTAAAGCCGAACGCCCTCAGAACTGTCATACGTTCCCGAAGGTAAGAAGCC

CCACTACGAAAGTGGCTTTAACCACACCCGACCCCACGAAAGCTGTGAAA

>113_Sample_ASV17

CACCGCGGTCACACGATTAACCCAAGTCAATAGAAGCCGGCGTAAAGAGTGTTTTAGATC

ACCCCCTCCCCAATAAAGCTAAAACTCACCTGAGTTGTAAAAAACTCCAGTTGACACAAA

ATAGACTACGAAAGTGGCTTTAACATATCTGAACACACAATAGCTAAGACC

>113_Sample_ASV18

CACCGCGGTTATACGAGAGGCCCTAGTTGATAAATACGGCGTAAAGAGTGGTTAGAGGAC

TCCCATAATAAAGCCAAAGACCCTCCGGGCCGTCACACGCATACGAGGGCACGAAGTTCA

CACACGAAAGTAGCTTTAAATTACCCGACGCCACGAAAGCTAAGAAA

>113_Sample_ASV19

CACCGCGGTTATACGAGAAACCCTAGTTGACAAATACGGCGTAAAGAGTGGTTAAGGGGT

ATCAAAAATAAAGTCAAACATTTTCCTAGTTGTTATAAGCATTTCGAGAACATGAAGCCC

AACCACGAAAGTAACTTTACCTTCACCTGACCCCACGAAAGCTAAGAAA

>113_Sample_ASV20

CACCGCGGTTATACGAAAGACCCTAGTTTATAAGTACGGCGTAAAGGGTGGTTAGGGGAC

AAACAAAATAAAGCCAAAGACCTCCTAAGCCGTCATACGCTTACGGAGGCACGAAGCCCA

AACACGAAAGTAGCTTTACCGAACATGCCCGACTCCACGAAAGCTGGGAAA

>113_Sample_ASV21

CACCGCGGTTATACGAGAGACCCTAGTTGATAAATACGGCGTAAAGAGTGGTTATGGGAA

AAACAAATAAAGTCAAACAACCTCTTAGCTGTTATACGCATTATGAGAGCATGAAGCCCC

CTCACGAAAGTAACTTTAATACCTCCTGACCCCACGAAAGCTAAGAAA

>113_Sample_ASV22

CACCGCGGTTATACGAGAGGCTCAAGTTGATAAACCCCGGCGTAAAGAGTGGTTAAGGAA

AAATAGAAACTAAAGCCGAATGCTTTCAAGACTGTTATACGTTCCCGAGAGTAAGAAGAC

CAATTACGAAAGTGGCTTTACTTTACCTGACCCCACGAAAGCTACGACA

>113_Sample_ASV23

CACCGCGGTTATACGAGAGGCTCGAGTTGACAAACCCCGGCGTAAAGAGTGGTTAAGGAA

ACCTAAAGACTAAAGCCGAATGCCTTCAAAGCTGTTATACGCTCCCGAAGGTGAGAAGTT

CGATCACGAAAGTGGCTTTATAACCCCTGACCCCACGAAAGCTATGATA

>113_Sample_ASV24

AACCGCGGTTATACGAGAGACCCTAATTGATAGCTCACGGCGTAAAGGGTGGTTTGGGGA

AACCTCAAAATAAAGCTAAAGACCTTCTAAGCCGTTACACGCATACCGAAGGCACGAGGC

CCTAACACGAAAGTAGCTTTACTATTACCCCTGACGCCACGAAAGCTAAGAAA

>113_Sample_ASV25

CACCGCGGTTAAACGAGAGGCCCTAGTTGATAGTACAACGGCGTAAAGGGTGGTTAAGGA

TAGTGAAATAATAAAGTCGAATGGCCCTTTGGCTGTCATACGCTTCTAGGAGTCCGAAGC

CCAATATACGAAAGTAACTTTAAGAGCGCCCACCTGACCCCACGAAAACTGAGAAA

>113_Sample_ASV26

CACCGCGGTCATACGATTAACCCAAATTAATAGGCACACGGCGTAAAGCGTGTTTAAGCA

CTATGCCAAATAAAGTTAAACTCCAATTAAGCTGTAAAAAGCCATAATCAAGACGAAAAT

AAATAACGAAAGTAACTTTACAACCGCTAAAACACGATAGCTAAGATC

>113_Sample_ASV27

CACCGCGGTCATACGATTAACCCAAGTTAATAGGCACACGGCGTAAAGCGTGTTTAAGCA

CTACGCCAAATAAAGTTAAACTCCAATTAAGCTGTAAAAAGCCATAATCAAGACGAAAAT

AAATAACGAAAGTAACTTTACAACCGCTGAAACACGATAGCTAAGATC

>113_Sample_ASV28

CACCGCGGTTATACGAGAGGCCCAAGTTGATACTCCACGGCGTAAAGAGTGGTTAGAAAA

GACCTACTACTAAAGCCGAACGCCTTCAAAGCTGTTATACGCATCCGAAGGTGAGAAGCC

CACCCACGAAAGTGGCTTTACAACCTTGACTCCACGAAAGCTATGACA

>113_Sample_ASV29

CACCGCGGTTATACGAGAGACCCCAGTTGATAAACACGGCGTAAAGAGTGGTTAAGGGCA

CAAAACAAATAAAGTCAAACAACCTCTTAGCTGTTATACGCATTATGAGGATATGAAGCC

CCCCTACGAAAGTAACTTTACCAACCCCTGACCCCACGAAAGCTAAGAAA

>113_Sample_ASV30

CACCGCGGTTATACGAGAGACCCTAGTTGATATATACGGCGTAAAGAGTGGTTTGGAATA

ACCAAGTAATATAGCCAAAGACCTCCCAAGCCGTCACACGCACCCCGGAGGCACGAAGCC

CAAACACGAAAGTAGCTTTATTAACTTACCGACGCCACGAAAGCTAAGAAA

>113_Sample_ASV31

CACCGCGGTTATACGAAAGACCCTAGTTTATAGGCACGGCGTAAAGGGTGGTTAGGGGAT

AAACAAAATAAAGCCAAAGACCTTCTAAGTCGTCATACACTCATGAAGGCACGAAGCCCA

AATACGAAAGTAACTTTACAACTTAGACCCGACTCCACGAAAGCTGGGGGA

>113_Sample_ASV32

CACCGCGGTTATACGAGAGACCCTAATTAATAGATACGGCGTAAAGAGTGGTTTAGGACA

AAAATTAATAAAGCCGAAGTTTCTCCTAGCCGTTGTACGCACTCTGAGAACACGAAGCCC

CACTACGAAAGTAGCTTTACCTATTATTTCCTGACCCCACGAAAGCTAAGAAA

>113_Sample_ASV33

CACCGCGGTTATACGAGAGGCCCAAGTTGATAGACATCGGCGTAAAGAGTGGTTAAGGAA

GTCTCAAAACTAAAGCCGAATGCCTTCAAGACTGTTATACGTTTCCGAAGGTAAGAAGCC

CCACTACGAAAGTGGCTTTACTCCACCTGACTCCACGAAAGCTGTGAAA

>113_Sample_ASV34

CACCGCGGTTATACGAAAGACCCTAGTTGATAGCTACGGCGTAAAGGGTGGTTAAGGTAT

ATAACAAATAAAGCTAAAGAGCCTCCAAGCCGTCGTACGCATTCTGAGAGCTCGAAACCC

TAACACGAAAGTAGCTTTAAAAAATAACACCTGACCCCACGAAAGCTAAGAAA

>113_Sample_ASV35

CACCGCGGTCATACGATTAACCCAAATTAATAGATCCACGGCGTAAAGAGTGTTTAAGaa

aaaaaaCCACAATAGAGTTAAATTATAACTAAGCTGTAAAAAGCCCTAGTTAAAATAAAA

TAACCCACGAAAGTGACTCTAATAATCCTGACACACGATAGCTAGGACC

>113_Sample_ASV36

CACCGCGGTTATACGAAAGACCCTAGTTGATAGACCACGGCGTAAAGGGTGGTTAAGGAA

CACAAAAATAAAGCTAAAGATCCTCTAAGCTGTCATACGCTTCCCGAAGACATGAAACCC

AATCACGAAAGTAGCTTTAAACTGTCCTGACACCACGAAAGCCAAGAAA

>114_Sample_ASV1

CACCGCGGTTATACGAAAGACCCTAGTTGATGGGTGCGGCGTAAAGGGTGGTTAGGGACT

ACAAAAAATAGAGCTAAAGATCCTCTAGGTCGTCATACACATCACGAGGAAACGAAGCCC

AAATACGAAAGTAGCTCTAAATATAATCCTGACGCCACGAAAGCCAAGAAA

>114_Sample_ASV2

CACCGCGGTTATACGAGAGACCCTAGTTGATTAAAGCGGCGTAAAGAGTGGTTATGGAAC

TATTCTTTTAAAGCAGAAAACCTCTCAAACTGTTATACGCACCCAGAGGTCTGAATCCCT

CACACGAAAGTGACTTTATTTATGCCTACCAGAACCCACGAAAGCTGGGACA

>114_Sample_ASV3

CACCGCGGTTATACGAGAGGCCCAAGTTGACAGACACCGGCGTAAAGAGTGGCTAGGGAA

AAATTACTACTAAAGCCGAACACCTTCAGAACTGTCATACGTTCCCGAAGATAAGAAGCC

CCACCACGAAAGTGGCTTTATATTACCCGACCCCACGAAAGCTGTGAAA

>114_Sample_ASV4

CACCGCGGTTATACGAGAGGCCCAAGTTGACAGACACCGGCGTAAAGAGTGGCTAGGGAA

AAATTACTACTAAAGCCGAACACCTTCAGAACTGTCATACGTTCCCGAAGATAAGAAGCC

CCACCACGAAAGTGGCTTTATATTACCCGACCCCACGAAAGCTAAGAAA

>114_Sample_ASV5

CACCGCGGTTATACGAGAGGCCCAAGTTGACAGACACCGGCGTAAAGAGTGGCTAAGGAA

AAATTTCTACTAAAGCCGAACACCTTCAGAACTGTCATACGTTCCCGAAGAAAAGAAGCC

CCACCACGAAAGTGGCTTTATATTACCCGACCCCACGAAAGCTGTGAAA

>114_Sample_ASV6

CACCGCGGTTAAACGAGAGACCCTAGTTGATCATCACGGCGTAAAGAGTGGTTAGGGGAT

TATCATAAATAAAGCCAAAGACCTCCCAAGCTGTCGCACGCATTCCGGAGGCGCGAAGCC

CACACACGAAAGTAGCTTTAACTATTGACCCTGATGCCACGAAAGCTAAGAAA

>114_Sample_ASV7

CGCCGCGGTTAAACGAGAGGCCCTAGTTGATAGAACAACGGCGTAAAGGGTGGTTAAGGA

TAGCGAAATAATAAAGTCGAATGGCCCTTTGGCTGTCATACGCTTCTGGGAGTCTGAAGC

CCAATATACGAAAGTAGCTTTAATACCGTCCACCTGACCCCACGAAAACTGAGAAA

>114_Sample_ASV8

CGCCGCGGTTAAACGAGAGGCCCTAGTTGATGGAACAACGGCGTAAAGGGTGGTTAAGGA

TAGTGAGACAATAAAGTCGAATGGCCCTTTGGCTGTCATACGCTTCTGGGAGCCTGAAGC

CCACTATACGAAAGTAGCTTTAACAATGTCCACCTGACCCCACGAAAACTGAGAAA

>114_Sample_ASV9

CACCGCGGTTATACGAAAGACCCTAGTTGATAGATCACGGCGTAAAGGGTGGTTAGGGAA

TACAAAAATAAAGCTAAAAATCCTCTAAGCTGTCATACGCTTCCCGAAGACATGAAACCC

AACCACGAAAGTAGCTTTAAACTCTCCTGACACCACGAAAGCCAAGGAA

>114_Sample_ASV10

CACCGCGGTTATACGAGAGGCCCAAGTTGATAAGCCCGGCGTAAAGAGTGGTTATGGAGA

ATAAAACACTAAAGCTAAAGACCCCCTAGGCTGTTATACGCACCTGGCGGCTCGAACCAC

CTATACGAAAGTAGCTTTACCCCCCTTCCACCAGAATCCACGACAGCTGGGGCA

>114_Sample_ASV11

CACCGCGGTTATACGAGAGGCCCAAGTTGACAGACATCGGCGTAAAGAGTGGTTAAGGAA

ACCCCATTACTAAAGCCGAACGCCCTCAGAACTGTCATACGTTCCCGAAGGTAAGAAGCC

CCACTACGAAAGTGGCTTTAACCACACCCGACCCCACGAAAGCTGTGAAA

>114_Sample_ASV12

CACCGCGGTTATACGAGAGGCCCAAGTTGACAGACATCGGCGTAAAGAGTGGTTAAGGAA

ACCCCATTACTAAAGCCGAACGCCCTCAGAACTGTCATACGTTCCCGAAGGTAAGAAGCC

CCACTACGAAAGTGGCTTTAACCACACCCGACCCCACGAAAGCTAAGAAA

>114_Sample_ASV13

CACCGCGGTTATACGAGAGACCCTAGTTGATATATACGGCGTAAAGAGTGGTTAAGGAAA

CCCCATTACTAAAGCCGAACGCCCTCAGAACTGTCATACGTTCCCGAAGGTAAGAAGCCC

CACTACGAAAGTGGCTTTAACCACACCCGACCCCACGAAAGCTGTGAAA

>114_Sample_ASV14

CACCGCGGTCACACGATTAACCCAAGTCAATAGAAGCCGGCGTAAAGAGTGTTTTAGATC

ACCCCCTCCCCAATAAAGCTAAAACTCACCTGAGTTGTAAAAAACTCCAGTTGACACAAA

ATAGACTACGAAAGTGGCTTTAACATATCTGAACACACAATAGCTAAGACC

>114_Sample_ASV15

CACCGCGGTTATACGAGAGGCCCTAGTTGATAAATACGGCGTAAAGAGTGGTTAGAGGAC

TCCCATAATAAAGCCAAAGACCCTCCGGGCCGTCACACGCATACGAGGGCACGAAGTTCA

CACACGAAAGTAGCTTTAAATTACCCGACGCCACGAAAGCTAAGAAA

>114_Sample_ASV16

CACCGCGGTTATACGAAAGACCCTAGTTTATAAGTACGGCGTAAAGGGTGGTTAGGGGAC

AAACAAAATAAAGCCAAAGACCTCCTAAGCCGTCATACGCTTACGGAGGCACGAAGCCCA

AACACGAAAGTAGCTTTACCGAACATGCCCGACTCCACGAAAGCTGGGAAA

>114_Sample_ASV17

CACCGCGGTTATACGAGAGACCCTAGTTGATAAATACGGCGTAAAGAGTGGTTATGGGAA

AAACAAATAAAGTCAAACAACCTCTTAGCTGTTATACGCATTATGAGAGCATGAAGCCCC

CTCACGAAAGTAACTTTAATACCTCCTGACCCCACGAAAGCTAAGAAA

>114_Sample_ASV18

CACCGCGGTTATACGAGAGGCTCAAGTTGATAAACCCCGGCGTAAAGAGTGGTTAAGGAA

AAATAGAAACTAAAGCCGAATGCTTTCAAGACTGTTATACGTTCCCGAGAGTAAGAAGAC

CAATTACGAAAGTGGCTTTACTTTACCTGACCCCACGAAAGCTACGACA

>114_Sample_ASV19

AACCGCGGTTATACGAGAGACCCTAATTGATAGCTCACGGCGTAAAGGGTGGTTTGGGGA

AACCTCAAAATAAAGCTAAAGACCTTCTAAGCCGTTACACGCATACCGAAGGCACGAGGC

CCTAACACGAAAGTAGCTTTACTATTACCCCTGACGCCACGAAAGCTAAGAAA

>114_Sample_ASV20

CACCGCGGTTAAACGAGAGGCCCTAGTTGATAGTACAACGGCGTAAAGGGTGGTTAAGGA

TAGTGAAATAATAAAGTCGAATGGCCCTTTGGCTGTCATACGCTTCTAGGAGTCCGAAGC

CCAATATACGAAAGTAACTTTAAGAGCGCCCACCTGACCCCACGAAAACTGAGAAA

>114_Sample_ASV21

CACCGCGGTCATACGATTAACCCAAGTTAATAGGCACACGGCGTAAAGCGTGTTTAAGCA

CTACGCCAAATAAAGTTAAACTCCAATTAAGCTGTAAAAAGCCATAATCAAGACGAAAAT

AAATAACGAAAGTAACTTTACAACCGCTGAAACACGATAGCTAAGATC

>114_Sample_ASV22

CACCGCGGTTATACGAGAGGCCCAAGTTGATACTCCACGGCGTAAAGAGTGGTTAGAAAA

GACCTACTACTAAAGCCGAACGCCTTCAAAGCTGTTATACGCATCCGAAGGTGAGAAGCC

CACCCACGAAAGTGGCTTTACAACCTTGACTCCACGAAAGCTATGACA

>114_Sample_ASV23

CACCGCGGTTATACGAGAGACCCCAGTTGATAAACACGGCGTAAAGAGTGGTTAAGGGCA

CAAAACAAATAAAGTCAAACAACCTCTTAGCTGTTATACGCATTATGAGGATATGAAGCC

CCCCTACGAAAGTAACTTTACCAACCCCTGACCCCACGAAAGCTAAGAAA

>114_Sample_ASV24

CACCGCGGTTATACGAGAGACCCTAGTTGATATATACGGCGTAAAGAGTGGTTTGGAATA

ACCAAGTAATATAGCCAAAGACCTCCCAAGCCGTCACACGCACCCCGGAGGCACGAAGCC

CAAACACGAAAGTAGCTTTATTAACTTACCGACGCCACGAAAGCTAAGAAA

>114_Sample_ASV25

CACCGCGGTTATACGAAAGACCCTAGTTTATAGGCACGGCGTAAAGGGTGGTTAGGGGAT

AAACAAAATAAAGCCAAAGACCTTCTAAGTCGTCATACACTCATGAAGGCACGAAGCCCA

AATACGAAAGTAACTTTACAACTTAGACCCGACTCCACGAAAGCTGGGGGA

>114_Sample_ASV26

CACCGCGGTTATACGAAAGACCCTAGTTTATAGGCACGGCGTAAAGGGTGGTTAGGGGAT

AAACAAAATAAAGCCAAAGACCTTCTAAGTCGTCATACACTCATGAAGGCACGAAGCCCA

AATACGAAAGTAACTTTACAACTTAGACCCGACTCCACGAAAGCTAAGAAA

>114_Sample_ASV27

CACCGCGGTTATACGAGAGGCCCAAGTTGATAGACATCGGCGTAAAGAGTGGTTAAGGAA

GTCTCAAAACTAAAGCCGAATGCCTTCAAGACTGTTATACGTTTCCGAAGGTAAGAAGCC

CCACTACGAAAGTGGCTTTACTCCACCTGACTCCACGAAAGCTGTGAAA

>114_Sample_ASV28

CACCGCGGTTATACGAGAGGCCCAAGTTGACAAACTCCGGCGTAAAGCGTGGTTAAGGCA

CACTCAAAAACTAAAGCCGAATATCTTCAGAGCAGTCATACGCATCCGAAGACACGAAGC

CCCACCACGAAAGTGGCTTTATGACACCTGACCCCACGAAAGCTAGGACA

>114_Sample_ASV29

CACCGCGGTTATACGAAAGACCCTAGTTGATAGACCACGGCGTAAAGGGTGGTTAAGGAA

CACAAAAATAAAGCTAAAGATCCTCTAAGCTGTCATACGCTTCCCGAAGACATGAAACCC

AATCACGAAAGTAGCTTTAAACTGTCCTGACACCACGAAAGCCAAGAAA

>115_Sample_ASV1

CACCGCGGTTATACGAAAGACCCTAGTTGATGGGTGCGGCGTAAAGGGTGGTTAGGGACT

ACAAAAAATAGAGCTAAAGATCCTCTAGGTCGTCATACACATCACGAGAAAACGAAGCCC

AAATACGAAAGTAGCTCTAAATATAATCCTGACGCCACGAAAGCCAAGAAA

>115_Sample_ASV2

CACCGCGGTTATACGAAAGACCCTAGTTGATGGGTGCGGCGTAAAGGGTGGTTAGGGACT

ACAAAAAATAGAGCTAAAGATCCTCTAGGTCGTCATACACATCACGAGGAAACGAAGCCC

AAATACGAAAGTAGCTCTAAATATAATCCTGACGCCACGAAAGCCAAGAAA

>115_Sample_ASV3

CACCGCGGTTATACGAGAGACCCTAGTTGATTAAAGCGGCGTAAAGAGTGGTTATGGAAC

TATTCTTTTAAAGCAGAAAACCTCTCAAACTGTTATACGCACCCAGAGGTCTGAATCCCT

CACACGAAAGTGACTTTATTTATGCCTACCAGAACCCACGAAAGCTGGGACA

>115_Sample_ASV4

CACCGCGATTATACGAGAGGCCCAAGTTGACAGACATCGGCGTAAAGAGTGGTTAAGGAA

AAATTACTACTAAAGCCGAACACCTTCAGAACTGTCATACGTTCCCGAAGATAAGAAGCC

CCACCACGAAAGTGGCTTTATATTACCCGACCCCACGAAAGCTGTGAAA

>115_Sample_ASV5

CACCGCGGTTATACGAGAGGCCCAAGTTGACAGACACCGGCGTAAAGAGTGGCTAGGGAA

AAATTACTACTAAAGCCGAACACCTTCAGAACTGTCATACGTTCCCGAAGATAAGAAGCC

CCACCACGAAAGTGGCTTTATATTACCCGACCCCACGAAAGCTGTGAAA

>115_Sample_ASV6

CACCGCGGTTATACGAGAGGCCCAAGTTGACAGACACCGGCGTAAAGAGTGGCTAAGGAA

AAATTTCTACTAAAGCCGAACACCTTCAGAACTGTCATACGTTCCCGAAGAAAAGAAGCC

CCACCACGAAAGTGGCTTTATATTACCCGACCCCACGAAAGCTGTGAAA

>115_Sample_ASV7

CACCGCGGTTATACGAAAGACCCTAGTTGATAGATCACGGCGTAAAGGGTGGTTAGGGAA

TACAAAAATAAAGCTAAAAATCCTCTAAGCTGTCATACGCTTTCCGAAGACATGAGATCC

AACCACGAAAGTAGCTTTAAAATTTCCTGACACCACGAAAGCCAAGGAA

>115_Sample_ASV8

CACCGCGGTTATACGAGAGGCCCAAGTTGATAAGCCCGGCGTAAAGAGTGGTTATGGAGA

ATAAAACACTAAAGCTAAAGACCCCCTAGGCTGTTATACGCACCTGGCGGCTCGAACCAC

CTATACGAAAGTAGCTTTACCCCCCTTCCACCAGAATCCACGACAGCTGGGGCA

>115_Sample_ASV9

CACCGCGGTTATACGAGAGGCCCTAGTTGATAGCCACGGCGCAAAGAGTGGTTAAGGAGC

CCCACTAAATAAAGTCGAACACTTCCTAGGCCGTTATACGTTTTCTAGAAGCACGAAACC

CAATTACACGAAAGCAACTTTATACTAAACAACCTGACCCCACGAAAGCTAAGAAA

>115_Sample_ASV10

CACCGCGGTTATACGAGAGGCCCAAGTTGACAGACATCGGCGTAAAGAGTGGTTAAGGAA

ACCCCATTACTAAAGCCGAACGCCCTCAGAACTGTCATACGTTCCCGAAGGTAAGAAGCC

CCACTACGAAAGTGGCTTTAACCACACCCGACCCCACGAAAGCTAAGAAA

>115_Sample_ASV11

CACCGCGGTTATACGAGAGGCCCAAGTTGACAGACATCGGCGTAAAGAGTGGTTAAGGAA

ACCCCATTACTAAAGCCGAACGCCCTCAGAACTGTCATACGTTCCCGAAGGTAAGAAGCC

CCACTACGAAAGTGGCTTTAACCACACCCGACCCCACGAAAGCTGTGAAA

>115_Sample_ASV12

CACCGCGGTTATACGAGAAACCCTAGTTGACAAATACGGCGTAAAGAGTGGTTAAGGGGT

ATCAAAAATAAAGTCAAACATTTTCCTAGTTGTTATAAGCATTTCGAGAACATGAAGCCC

AACCACGAAAGTAACTTTACCTTCACCTGACCCCACGAAAGCTAAGAAA

>115_Sample_ASV13

CACCGCGGTTATACGAAAGACCCCAGTTTATAGACACGGCGTAAAGGGTGGTTATAGGAC

AAACTAACATAGAGCCAAAGACCCCCTAAGCCGTCATACGCTCACGAGGGCTCGAAGCCC

CTATACGAAAGTAGCTCTACCAAAAAATGCTCGACTCCACGAAAGCTGGGACA

>115_Sample_ASV14

CACCGCGGTTATACGAGAGACCCTAGTTGATAAATACGGCGTAAAGAGTGGTTATGGGAA

AAACAAATAAAGTCAAACAACCTCTTAGCTGTTATACGCATTATGAGAGCATGAAGCCCC

CTCACGAAAGTAACTTTAATACCTCCTGACCCCACGAAAGCTAAGAAA

>115_Sample_ASV15

AACCGCGGTTATACGAGAGACCCTAATTGATAGCTCACGGCGTAAAGGGTGGTTTGGGGA

AACCTCAAAATAAAGCTAAAGACCTTCTAAGCCGTTACACGCATACCGAAGGCACGAGGC

CCTAACACGAAAGTAGCTTTACTATTACCCCTGACGCCACGAAAGCTAAGAAA

>115_Sample_ASV16

CACCGCGGTTATACGAAAGACCCTAGTTGATGGATCACGGCGTAAAGGGTGGTTAAGGGA

TATAAAAATAAAGCTAAAAATCCTCTTAGCTGTCATACGCTTTCCGAAGACATGAGGCCC

AATTACGAAAGTAGCTTTAAACCACCCTGACACCACGAAAGCCAAGAAA

>115_Sample_ASV17

CACCGCGGTTATACGAGAGGCCCTAGTTGATAACTACCGGCGTAAAGAGTGGTTATGGAA

AAATATTTAATAAAGCCGAACACCCCCTCAGCCGTCATACGCACCTGGGAGCACGAAGAC

CTACTGCGAAAGCAGCTTTAATTATGCCTGACCCCACGACAGCTAAGAAA

>115_Sample_ASV18

CACCGCGGTTATACGAGAGGCCCAAGTTGATTATTACCGGCGTAAAGCGTGGTTAAGATT

TTTATAGGACTAAAGCCGAACCCCCTCAGAGCTGTTATACGCACCCGAAGGTTAGAAGTT

CAATCACGAAAGTGGCTTTACAACCCCGAACCCACGAAAGCTAAGATA

>115_Sample_ASV19

CACCGCGGTTATACGAGAGACCCTAATTGATAGGCGCGGCGTAAAGAGTGGTTTAGGACT

AAAACTAATAAAGCTAAAGATCCTCCCAGCCGTCGCACGCATTCTGAGGACACGAAACTC

AACCACGAAAGTAGCTTTAACATACTCTCCTGACCCCACGAAAGCTAAGAAA

>115_Sample_ASV20

CACCGCGGTTATACGAGAGACCCTAGTTGATATATACGGCGTAAAGAGTGGTTTAGGACT

AAAACTAATAAAGCTAAAGATCCTCCCAGCCGTCGCACGCATTCTGAGGACACGAAACTC

AACCACGAAAGTAGCTTTAACATACTCTCCTGACCCCACGAAAGCTAAGAAA

>115_Sample_ASV21

CACCGCGGTTATACGAAAGACCCTAGTTGATAGGCACGGCGTAAAGGGTGGTTAGGGATA

TATCAAAAATAAAGTTAAAGAGCCTCTAAGCTGTCGCACGCATCCCGAGAGCTCGAGACC

CAGACACGAAAGTAACTTTAAAATACCTCACCTGACCCCACGAAAGCTAAGAAA

>115_Sample_ASV22

CACCGCGGTTATACGAGAGGCCCAAGTTGATACTCCACGGCGTAAAGAGTGGTTAGAAAA

GACCTACTACTAAAGCCGAACGCCTTCAAAGCTGTTATACGCATCCGAAGGTGAGAAGCC

CACCCACGAAAGTGGCTTTACAACCTTGACTCCACGAAAGCTATGACA

>115_Sample_ASV23

CACCGCGGTTATACGAGAGACCCCAGTTGATAAACACGGCGTAAAGAGTGGTTAAGGGCA

CAAAACAAATAAAGTCAAACAACCTCTTAGCTGTTATACGCATTATGAGGATATGAAGCC

CCCCTACGAAAGTAACTTTACCAACCCCTGACCCCACGAAAGCTAAGAAA

>115_Sample_ASV24

CACCGCGGTTATACGAAAGACCCTAGTTTATAGGCACGGCGTAAAGGGTGGTTAGGGGAT

AAACAAAATAAAGCCAAAGACCTTCTAAGTCGTCATACACTCATGAAGGCACGAAGCCCA

AATACGAAAGTAACTTTACAACTTAGACCCGACTCCACGAAAGCTGGGGGA

>115_Sample_ASV25

CACCGCGGTTATACGAAAGACCCTAGTTTATAGGCACGGCGTAAAGGGTGGTTAGGGGAT

AAACAAAATAAAGCCAAAGACCTTCTAAGTCGTCATACACTCATGAAGGCACGAAGCCCA

AATACGAAAGTAACTTTACAACTTAGACCCGACTCCACGAAAGCTAAGAAA

>115_Sample_ASV26

CACCGCGGTTATACGAGAGACCCTAATTAATAGCTACGGCGTAAAGAGTGGTTTAGGGTA

AAAATTAATAAAGCCGAAGTTTCTCCTAGCCGTTGTACGCACTCTGAGAACACGAAGCCC

CACTACGAAAGTAGCTTTACCTATTATTTCCTGACCCCACGAAAGCTAAGAAA

>115_Sample_ASV27

CACCGCGGTTATACGAGAGACCCTAATTAATAGATACGGCGTAAAGAGTGGTTTAGGACA

AAAATTAATAAAGCCGAAGTTTCTCCTAGCCGTTGTACGCACTCTGAGAACACGAAGCCC

CACTACGAAAGTAGCTTTACCTATTATTTCCTGACCCCACGAAAGCTAAGAAA

>115_Sample_ASV28

CACCGCGGTTATACGAGAGACCCTAATTAATAGATACGGCGTAAAGAGTGGTTTAGGACA

AAAATTAATAAAGCCGAAGTTTCTCCTAGCCGTTGTACGCACTCTGAGAACACGAAGCCC

CACTACGAAAGTAGCTTTACCAATAATCTCCTGACCCCACGAAAGCTAAGAAA

>115_Sample_ASV29

CACCGCGGTTATACGAAAGACCCTAGTTGATAGATCACGGCGTAAAGGGTGGTTAAGGAA

AACAAAAATAAAGCTAAAAATCCTCTAAGCTGTCATACGCTTTCCGAAGACATGAGATCC

AACCACGAAAGTAGCTTTAAAATTTCCTGACACCACGAAAGCCAAGGAA

>127_Sample_ASV1

CACCGCGGTTATACGAAAGACCCTAGTTGATGGGTGCGGCGTAAAGGGTGGTTAGGGACT

ACAAAAAATAGAGCTAAAGATCCTCTAGGTCGTCATACACATCACGAGGAAACGAAGCCC

AAATACGAAAGTAGCTCTAAATATAATCCTGACGCCACGAAAGCCAAGAAA

>127_Sample_ASV2

CACCGCGGTTATACGAAAGACCCTAGTTGCTAGCCACGGCGTAAAGGGTGGTTAAGGACA

ACAACAAATAAAGCTAAAGATCCCCTAAGCCGTCATACGCATTCCGGGGGCACGAAGCCC

TAACACGAAAGTAGCTTTAAAAAATATACCTGACCCCACGAAAGCTAAGAAA

>127_Sample_ASV3

CACCGCGGTTATACGAGAGACCCTAGTTGATTAAAGCGGCGTAAAGAGTGGTTATGGAAC

TATTCTTTTAAAGCAGAAAACCTCTCAAACTGTTATACGCACCCAGAGGTCTGAATCCCT

CACACGAAAGTGACTTTATTTATGCCTACCAGAACCCACGAAAGCTGGGACA

>127_Sample_ASV4

CACCGCGGTTATACGAAAGACCCTAGTTTATAGGTACGGCGTAAAGGGTGGTTAGGGGAC

AAACAAAATAAAGCCAAAGACCTCCTAAGCCGTCATACGCTCACGGAAGCACGAAGCCCA

AACACGAAAGTAGCTTTACCGAACATGCCCGACTCCACGAAAGCTGGGGAA

>127_Sample_ASV5

CACCGCGGTTATACGAGAGGCCCAAGTTGACAGACACCGGCGTAAAGAGTGGCTAGGGAA

AAATTACTACTAAAGCCGAACACCTTCAGAACTGTCATACGTTCCCGAAGATAAGAAGCC

CCACCACGAAAGTGGCTTTATATTACCCGACCCCACGAAAGCTGTGAAA

>127_Sample_ASV6

CACCGCGGTTATACGAGAGGCCCAAGTTGACAGACACCGGCGTAAAGAGTGGCTAAGGAA

AAATTTCTACTAAAGCCGAACACCTTCAGAACTGTCATACGTTCCCGAAGAAAAGAAGCC

CCACCACGAAAGTGGCTTTATATTACCCGACCCCACGAAAGCTGTGAAA

>127_Sample_ASV7

CACCGCGGTTATACGAGAGGCTCAAGTTGACAGACATCGGCGTAAAGAGTGGTTAGGAAG

TTTTTGAAACTAAAGCCGAACGCCCTCAGAACTGTTATACGTACCCGAGGGTAAGAAGCC

CCACTACGAAAGTGGCTTTATATCTCCGACCCCACGAAAGCTGCGAAA

>127_Sample_ASV8

CACCGCGGTTAAACGAGAGACCCTAGTTGATCATCACGGCGTAAAGAGTGGTTAGGGGAT

TATCATAAATAAAGCCAAAGACCTCCCAAGCTGTCGCACGCATTCCGGAGGCGCGAAGCC

CACACACGAAAGTAGCTTTAACTATTGACCCTGATGCCACGAAAGCTAAGAAA

>127_Sample_ASV9

CGCCGCGGTTAAACGAGAGGCCCTAGTTGATGGAACAACGGCGTAAAGGGTGGTTAAGGA

TAGTGAGACAATAAAGTCGAATGGCCCTTTGGCTGTCATACGCTTCTGGGAGCCTGAAGC

CCACTATACGAAAGTAGCTTTAACAATGTCCACCTGACCCCACGAAAACTGAGAAA

>127_Sample_ASV10

CACCGCGGTTATACGAAAGACCCTAGTTGATAGATCACGGCGTAAAGGGTGGTTAGGGAA

TACAAAAATAAAGCTAAAAATCCTCTAAGCTGTCATACGCTTCCCGAAGACATGAAACCC

AACCACGAAAGTAGCTTTAAACTCTCCTGACACCACGAAAGCCAAGGAA

>127_Sample_ASV11

CACCGCGGTTATACGAGAGGCCCAAGTTGATAAGCCCGGCGTAAAGAGTGGTTATGGAGA

ATAAAACACTAAAGCTAAAGACCCCCTAGGCTGTTATACGCACCTGGCGGCTCGAACCAC

CTATACGAAAGTAGCTTTACCCCCCTTCCACCAGAATCCACGACAGCTGGGGCA

>127_Sample_ASV12

CACCGCGGTTATACGAGAGGCCCTAGTTGATAGCCACGGCGCAAAGAGTGGTTAAGGAGC

CCCACTAAATAAAGTCGAACACTTCCTAGGCCGTTATACGTTTTCTAGAAGCACGAAACC

CAATTACACGAAAGCAACTTTATACTAAACAACCTGACCCCACGAAAGCTAAGAAA

>127_Sample_ASV13

CACCGCGGTTATACGAGAGGCCCAAGTTGACAGACATCGGCGTAAAGAGTGGTTAAGGAA

ACCCCATTACTAAAGCCGAACGCCCTCAGAACTGTCATACGTTCCCGAAGGTAAGAAGCC

CCACTACGAAAGTGGCTTTAACCACACCCGACCCCACGAAAGCTGTGAAA

>127_Sample_ASV14

CACCGCGGTTATACGAGAGGCCCAAGTTGATAGATATCGGCGTAAAGAGTGGTTAAGGAA

ACCCCGTTACTAAAGCCGAACGCCCTCAGAACTGTCATACGTTCCCGAAGGTAAGAAGCC

CCACTACGAAAGTGGCTTTAACCACACCCGACCCCACGAAAGCTGTGAAA

>127_Sample_ASV15

CACCGCGGTTAAACGAGAGGCCCTAGTTGATATTACAACGGCGTAAAGGGTGGTTAAGGA

AAGCAAGCAATAAAGCCGAATGGCCCTTTGGCTGTCATACGCTTCTAGGTGTCCGAAGCC

CAATATACGAAAGTAGCTTTAGAAAAGCCCACCTGACACCACGAAAGCTGAGGAA

>127_Sample_ASV16

CACCGCGGTCACACGATTAACCCAAGTCAATAGAAGCCGGCGTAAAGAGTGTTTTAGATC

ACCCCCTCCCCAATAAAGCTAAAACTCACCTGAGTTGTAAAAAACTCCAGTTGACACAAA

ATAGACTACGAAAGTGGCTTTAACATATCTGAACACACAATAGCTAAGACC

>127_Sample_ASV17

CACCGCGGTTATACGAGAGGCCCTAGTTGATAAATACGGCGTAAAGAGTGGTTAGAGGAC

TCCCATAATAAAGCCAAAGACCCTCCGGGCCGTCACACGCATACGAGGGCACGAAGTTCA

CACACGAAAGTAGCTTTAAATTACCCGACGCCACGAAAGCTAAGAAA

>127_Sample_ASV18

CACCGCGGTTATACGAAAGACCCCAGTTTATAGACACGGCGTAAAGGGTGGTTATAGGAC

AAACTAACATAGAGCCAAAGACCCCCTAAGCCGTCATACGCTCACGAGGGCTCGAAGCCC

CTATACGAAAGTAGCTCTACCAAAAAATGCTCGACTCCACGAAAGCTGGGACA

>127_Sample_ASV19

CACCGCGGTTATACGAAAGACCCTAGTTTATAAGTACGGCGTAAAGGGTGGTTAGGGGAC

AAACAAAATAAAGCCAAAGACCTCCTAAGCCGTCATACGCTTACGGAGGCACGAAGCCCA

AACACGAAAGTAGCTTTACCGAACATGCCCGACTCCACGAAAGCTGGGAAA

>127_Sample_ASV20

CACCGCGGTTATACGAGAGACCCTAGTTGATAAATACGGCGTAAAGAGTGGTTATGGGAA

AAACAAATAAAGTCAAACAACCTCTTAGCTGTTATACGCATTATGAGAGCATGAAGCCCC

CTCACGAAAGTAACTTTAATACCTCCTGACCCCACGAAAGCTAAGAAA

>127_Sample_ASV21

CACCGCGGTTATACGAGAGGCTCAAGTTGATAAACCCCGGCGTAAAGAGTGGTTAAGGAA

AAATAGAAACTAAAGCCGAATGCTTTCAAGACTGTTATACGTTCCCGAGAGTAAGAAGAC

CAATTACGAAAGTGGCTTTACTTTACCTGACCCCACGAAAGCTACGACA

>127_Sample_ASV22

AACCGCGGTTATACGAGAGACCCTAATTGATAGCTCACGGCGTAAAGGGTGGTTTGGGGA

AACCTCAAAATAAAGCTAAAGACCTTCTAAGCCGTTACACGCATACCGAAGGCACGAGGC

CCTAACACGAAAGTAGCTTTACTATTACCCCTGACGCCACGAAAGCTAAGAAA

>127_Sample_ASV23

CACCGCGGTTATACGAAAGACCCTAGTTGATGGATCACGGCGTAAAGGGTGGTTAAGGGA

TATAAAAATAAAGCTAAAAATCCTCTTAGCTGTCATACGCTTTCCGAAGACATGAGGCCC

AATTACGAAAGTAGCTTTAAACCACCCTGACACCACGAAAGCCAAGAAA

>127_Sample_ASV24

CACCGCGGTTATACGAGAGACCCTAGTTGATAGCTACGGCGTAAAGCGTGGTTAGGAGAA

CAAGATAAATAAAGTCAAACAATCTCTCGGCCGTTATACGTTATCTGAGAATATGAAGTC

CTACCACGAAAGTAACTTTAATTTTTCTGACCCCACGAAAACTGAGGAA

>127_Sample_ASV25

CACCGCGGTTAAACGAGAGGCCCTAGTTGATAGTACAACGGCGTAAAGGGTGGTTAAGGA

TAGTGAAATAATAAAGTCGAATGGCCCTTTGGCTGTCATACGCTTCTAGGAGTCCGAAGC

CCAATATACGAAAGTAACTTTAAGAGCGCCCACCTGACCCCACGAAAACTGAGAAA

>127_Sample_ASV26

CACCGCGGTTATACGAGAGGCCCAAGTTGATACTCCACGGCGTAAAGAGTGGTTAGAAAA

GACCTACTACTAAAGCCGAACGCCTTCAAAGCTGTTATACGCATCCGAAGGTGAGAAGCC

CACCCACGAAAGTGGCTTTACAACCTTGACTCCACGAAAGCTATGACA

>127_Sample_ASV27

CACCGCGGTTATACGAGAGACCCCAGTTGATAAACACGGCGTAAAGAGTGGTTAAGGGCA

CAAAACAAATAAAGTCAAACAACCTCTTAGCTGTTATACGCATTATGAGGATATGAAGCC

CCCCTACGAAAGTAACTTTACCAACCCCTGACCCCACGAAAGCTAAGAAA

>127_Sample_ASV28

CACCGCGGTTATACGAGAGACCCTAGTTGATATATACGGCGTAAAGAGTGGTTTGGAATA

ACCAAGTAATATAGCCAAAGACCTCCCAAGCCGTCACACGCACCCCGGAGGCACGAAGCC

CAAACACGAAAGTAGCTTTATTAACTTACCGACGCCACGAAAGCTAAGAAA

>127_Sample_ASV29

CACCGCGGTTATACGAAAGACCCTAGTTTATAGGCACGGCGTAAAGGGTGGTTAGGGGAT

AAACAAAATAAAGCCAAAGACCTTCTAAGTCGTCATACACTCATGAAGGCACGAAGCCCA

AATACGAAAGTAACTTTACAACTTAGACCCGACTCCACGAAAGCTGGGGGA

>127_Sample_ASV30

CACCGCGGTTATACGAGAGGCCCAAGTTGATAGACATCGGCGTAAAGAGTGGTTAAGGAA

GTCTCAAAACTAAAGCCGAATGCCTTCAAGACTGTTATACGTTTCCGAAGGTAAGAAGCC

CCACTACGAAAGTGGCTTTACTCCACCTGACTCCACGAAAGCTGTGAAA

>127_Sample_ASV31

CACCGCGGTTATACGAGAGGCCCAAGTTGACAAACTCCGGCGTAAAGCGTGGTTAAGGCA

CACTCAAAAACTAAAGCCGAATATCTTCAGAGCAGTCATACGCATCCGAAGACACGAAGC

CCCACCACGAAAGTGGCTTTATGACACCTGACCCCACGAAAGCTAGGACA

>127_Sample_ASV32

CACCGCGGTTATACGAGAGACTCCAGTTGATAGCTACGGCGTAAAGAGTGGTTTGGGGCC

CGCCCAAAATAAAGCCAAAGACCTCCCAAGCCGTCAAACGCACCCCGGAGGCACGAAGTC

CTAACGCGAAAGCAACTTTACCTTCCCCGACGCCACGAAAGCTAAGAAA

>127_Sample_ASV33

CACCGCGGTTATACGAAAGACCCTAGTTGATAGCTACGGCGTAAAGGGTGGTTAAGGTAT

ATAACAAATAAAGCTAAAGAGCCTCCAAGCCGTCGTACGCATTCTGAGAGCTCGAAACCC

TAACACGAAAGTAGCTTTAAAAAATAACACCTGACCCCACGAAAGCTAAGAAA

>127_Sample_ASV34

CACCGCGGTTATACGAAAGACCCTAGTTGATAGACCACGGCGTAAAGGGTGGTTAAGGAA

CACAAAAATAAAGCTAAAGATCCTCTAAGCTGTCATACGCTTCCCGAAGACATGAAACCC

AATCACGAAAGTAGCTTTAAACTGTCCTGACACCACGAAAGCCAAGAAA

>127_Sample_ASV35

CACCGCGGTTATACGAAAGACCCTAGTTGATAGATCACGGCGTAAAGGGTGGTTAAGGAG

AACAAAAATAAAGCTAAAAATCCTCTAAGCTGTCATACGCTTTCCGAAGACATGAGACCC

AACCACGAAAGTAACTTTAAACTTTCCTGACACCACGAAAGCCAAGAAA

>131_Sample_ASV1

CACCGCGGTTATACGAAAGACCCTAGTTGATGGGTGCGGCGTAAAGGGTGGTTAGGGACT

ACAAAAAATAGAGCTAAAGATCCTCTAGGTCGTCATACACATCACGAGGAAACGAAGCCC

AAATACGAAAGTAGCTCTAAATATAATCCTGACGCCACGAAAGCCAAGAAA

>131_Sample_ASV2

CACCGCGGTTAAACGAGAGGCCCTAGTTGATGGTGCAACGGCGTAAAGGGTGGTTAAGGA

TAGCGAAATAATAAAGTCAAATGGCCCTCTGGCTGTCATACGCTTCTAGGAGTCCGAAGC

CCAGTATACGAAAGTAGCTTTAGAAAAGCCCACCTGACACCACGAAAGCTGAGGAA

>131_Sample_ASV3

CACCGCGGTTATACGAAAGACCCTAGTTGCTAGCCACGGCGTAAAGGGTGGTTAAGGACA

ACAACAAATAAAGCTAAAGATCCCCTAAGCCGTCATACGCATTCCGGGGGCACGAAGCCC

TAACACGAAAGTAGCTTTAAAAAATATACCTGACCCCACGAAAGCTAAGAAA

>131_Sample_ASV4

CACCGCGGTTATACGAGAGACCCTAGTTGATTAAAGCGGCGTAAAGAGTGGTTATGGAAC

TATTCTTTTAAAGCAGAAAACCTCTCAAACTGTTATACGCACCCAGAGGTCTGAATCCCT

CACACGAAAGTGACTTTATTTATGCCTACCAGAACCCACGAAAGCTGTGAAA

>131_Sample_ASV5

CACCGCGGTTATACGAGAGACCCTAGTTGATTAAAGCGGCGTAAAGAGTGGTTATGGAAC

TATTCTTTTAAAGCAGAAAACCTCTCAAACTGTTATACGCACCCAGAGGTCTGAATCCCT

CACACGAAAGTGACTTTATTTATGCCTACCAGAACCCACGAAAGCTGGGACA

>131_Sample_ASV6

CACCGCGGTTATACGAAAGACCCTAGTTTATAGGTACGGCGTAAAGGGTGGTTAGGGGAC

AAACAAAATAAAGCCAAAGACCTCCTAAGCCGTCATACGCTCACGGAAGCACGAAGCCCA

AACACGAAAGTAGCTTTACCGAACATGCCCGACTCCACGAAAGCTGGGGAA

>131_Sample_ASV7

CACCGCGGTTATACGAAAGACCCTAGTTTATAGGTACGGCGTAAAGGGTGGTTAGGGGAC

AAACAAAATAAAGCCAAAGACCTCCTAAGCCGTCATACGCTCACGGAAGCACGAAGCCCA

AACACGAAAGTAGCTTTACCGAACATGCCCGACTCCACGAAAGCTGTGAAA

>131_Sample_ASV8

CACCGCGGTTATACGAAAGACCCTAGTTGATAGCCACGGCGTAAAGGGTGGTTAAGGAAC

CTAAAAAATAAAGCTAAAGACCCTCTAGGCCGTCATACGCATTACGAGGCAAACGAAACC

CCAACACGAAAGTAGCTTTAGACAAATATCCTGATGCCACGAAAGCCAAGAAA

>131_Sample_ASV9

CACCGCGGTTATACGAGAGGCCCAAGTTGACAGACACCGGCGTAAAGAGTGGCTAGGGAA

AAATTACTACTAAAGCCGAACACCTTCAGAACTGTCATACGTTCCCGAAGATAAGAAGCC

CCACCACGAAAGTGACTTTATATTACCCGACCCCACGAAAGCTGTGAAA

>131_Sample_ASV10

CACCGCGGTTATACGAGAGGCCCAAGTTGACAGACACCGGCGTAAAGAGTGGCTAGGGAA

AAATTACTACTAAAGCCGAACACCTTCAGAACTGTCATACGTTCCCGAAGATAAGAAGCC

CCACCACGAAAGTGGCTTTATATTACCCGACCCCACGAAAGCTAAGAAA

>131_Sample_ASV11

CACCGCGGTTATACGAGAGGCCCAAGTTGACAGACACCGGCGTAAAGAGTGGCTAGGGAA

AAATTACTACTAAAGCCGAACACCTTCAGAACTGTCATACGTTCCCGAAGATAAGAAGCC

CCACCACGAAAGTGGCTTTATATTACCCGACCCCACGAAAGCTGTGAAA

>131_Sample_ASV12

CACCGCGGTTATACGAGAGGCCCAAGTTGACAGACATCGGCGTAAAGAGTGGTTAAGGAA

ACCCCATTACTAAAGCCGAACACCTTCAGAACTGTCATACGTTCCCGAAGATAAGAAGCC

CCACCACGAAAGTGGCTTTATATTACCCGACCCCACGAAAGCTGTGAAA

>131_Sample_ASV13

CACCGCGGTTATACGAGAGGCTCAAGTTGACAGACATCGGCGTAAAGAGTGGTTAGGAAG

TTTTTGAAACTAAAGCCGAACGCCCTCAGAACTGTTATACGTACCCGAGGGTAAGAAGCC

CCACTACGAAAGTGGCTTTATATCTCCGACCCCACGAAAGCTGCGAAA

>131_Sample_ASV14

CACCGCGGTTAAACGAGAGACCCTAGTTGATCATCACGGCGTAAAGAGTGGTTAGGGGAT

TATCATAAATAAAGCCAAAGACCTCCCAAGCTGTCGCACGCATTCCGGAGGCGCGAAGCC

CACACACGAAAGTAGCTTTAACTATTGACCCTGATGCCACGAAAGCTAAGAAA

>131_Sample_ASV15

CACCGCGGTTATACGAGAGACCCTAGTTGATATATACGGCGTAAAGAGTGGTTTGGGGTT

ATGCCTCCAAAATAAAGCCGAAGATCTCCCAAGCCGTCACACGCATCCCGGAGACACGAA

GCCCTAATACGAAGGTAGCTTTATTAGTACCCCGACGCCACGAAAGCTAAGAAA

>131_Sample_ASV16

CACCGCGGTTATACGAGAGACCCTAGTTGATATATACGGCGTAAAGAGTGGTTTGGGGTT

ATGCCTCCAAAATAAAGCCGAAGATCTCCCAAGCCGTCACACGCATCCCGGAGACACGAA

GCCCTAATACGAAGGTAGCTTTATTAATTCCCCGACGCCACGAAAGCTAAGAAA

>131_Sample_ASV17

CACCGCGGTTATACGAGAGGCCCAAGTTGATAAGCCCGGCGTAAAGAGTGGTTATGGAGA

ATAAAACACTAAAGCTAAAGACCCCCTAGGCTGTTATACGCACCTGGCGGCTCGAACCAC

CTATACGAAAGTAGCTTTACCCCCCTTCCACCAGAATCCACGACAGCTGGGGCA

>131_Sample_ASV18

CACCGCGGTTATACGAGAGGCCCTAGTTGATAGCCACGGCGCAAAGAGTGGTTAAGGAGC

CCCACTAAATAAAGTCGAACACTTCCTAGGCCGTTATACGTTTTCTAGAAGCACGAAACC

CAATTACACGAAAGCAACTTTATACTAAACAACCTGACCCCACGAAAGCTAAGAAA

>131_Sample_ASV19

CACCGCGGTTATACGAGAGGCTCAAGTTGATAAAATACGGCGTAAAGCGTGGTTAAAAGC

CCCCACTAAACTAAGACTAAACCTTTCCAAAGCTGTTATACGCACCCGGAAATATGAAAC

TCAACTACGAAAGTGGCCTTAATTTCCCCTTGACCCCACGAAAGCTGCGAAA

>131_Sample_ASV20

CACCGCGGTTATACGAGAGGCCCAAGTTGATAAAATACGGCGTAAAGCGTGGTTAAAAGC

CCCCACTAAACTAAGACTAAACCTTTCCAAAGCTGTTATACGCACCCGGAAATACGAAAC

TCAACTACGAAAGTGGCCTTAATTTCCCCTTGACCCCACGAAAGCTGCGAAA

>131_Sample_ASV21

CACCGCGGTTATACGAGAGGCCCAAGTTGATAAAATACGGCGTAAAGCGTGGTTAAAAGC

CCCCACTAAACTAAGACTAAACCTTTCCAAAGCTGTTATACGCACCCGGAAATATGAAAC

TCAACTACGAAAGTGGCCTTAATTTCCCCTTGACCCCACGAAAGCTGCGAAA

>131_Sample_ASV22

CACCGCGGTTATACGAGAGGCCCAAGTTGACAGACATCGGCGTAAAGAGTGGTTAAGGAA

ACCCCATTACTAAAGCCGAACGCCCTCAGAACTGTCATACGTTCCCGAAGGTAAGAAGCC

CCACTACGAAAGTGGCTTTAACCACACCCGACCCCACGAAAGCTGTGAAA

>131_Sample_ASV23

CACCGCGGTTATACGAGAGGCCCAAGTTGACAGACATCGGCGTAAAGAGTGGTTAAGGAA

ACCCCATTACTAAAGCCGAACGCCCTCAGAACTGTCATACGTTCCCGAAGGTAAGAAGCC

CCACTACGAAAGTGGCTTTATGACACCTGACCCCACGAAAGCTAGGACA

>131_Sample_ASV24

CACCGCGGTTATACGAGAGGCTCAAGTTGATAAACCCCGGCGTAAAGAGTGGTTAAGGAA

ACCCCATTACTAAAGCCGAACGCCCTCAGAACTGTCATACGTTCCCGAAGGTAAGAAGCC

CCACTACGAAAGTGGCTTTAACCACACCCGACCCCACGAAAGCTGTGAAA

>131_Sample_ASV25

CACCGCGGTTATACGAGAGGCCCAAGTTGATAGATATCGGCGTAAAGAGTGGTTAAGGAA

ACCCCGTTACTAAAGCCGAACGCCCTCAGAACTGTCATACGTTCCCGAAGGTAAGAAGCC

CCACTACGAAAGTGGCTTTAACCACACCCGACCCCACGAAAGCTGTGAAA

>131_Sample_ASV26

CACCGCGGTTATACGAGAGGCCCAAGTTGATACTCCACGGCGTAAAGAGTGGTTAAGGAA

ACCCCATTACTAAAGCCGAACGCCCTCAGAACTGTCATACGTTCCCGAAGGTAAGAAGCC

CCACTACGAAAGTGGCTTTAACCACACCCGACCCCACGAAAGCTAAGAAA

>131_Sample_ASV27

CACCGCGGTTATACGAGAGGCTCAAGTTGATAAACCCCGGCGTAAAGAGTGGTTAAGGAA

ACCCCATTACTAAAGCCGAACGCCCTCAGAACTGTCATACGTTCCCGAGGGTAAGAAGCC

CCACTACGAAAGTGGCTTTAACCACACCCGACCCCACGAAAGCTGTGAAA

>131_Sample_ASV28

CACCGCGGTTAAACGAGAGGCCCTAGTTGATATTACAACGGCGTAAAGGGTGGTTAAGGA

AAGCAAGCAATAAAGCCGAATGGCCCTTTGGCTGTCATACGCTTCTAGGTGTCCGAAGCC

CAATATACGAAAGTAGCTTTAGAAAAGCCCACCTGACACCACGAAAGCTGAGGAA

>131_Sample_ASV29

CACCGCGGTTAAACGAGAGGCCCTAGTTGATATTACAACGGCGTAAAGGGTGGTTAAGGA

AAGCAAGCAATAAAGCCGAATGGCCCTTTGGCTGTCATACGCTTCTAGGTGTCCGAAGCC

CAATATACGAAAGTAGCTTTAGAAAAGCCCACCTGACACCACGAAAGCTGTGAAA

>131_Sample_ASV30

CACCGCGGTTAAACGAGAGGCCCTAGTTGATATTACAACGGCGTAAAGGGTGGTTAAGGA

AAGCAAGCAATAAAGCCGAATGGCCCTTTGGCTGTCATACGCTTCTAGGTGTCCGAAGCT

CAATATACGAAAGTAGCTTTAGAAAAGCCCACCTGACACCACGAAAGCTGAGGAA

>131_Sample_ASV31

CACCGCGGTTATACGAGAGGCCCAAGTTGACAGACACCGGCGTAAAGAGTGGTTAAGGAA

ATTCATCAACTAAAGCCGAATGCCCTCAGGACTGTTATACGTTTGCGAAGGTAAGAAGCC

CTACTACGAAAGTGGCTTTATTCCACCTGATTCCACGAAAGCTGCGGAA

>131_Sample_ASV32

CACCGCGGTCACACGATTAACCCAAGTCAATAGAAGCCGGCGTAAAGAGTGTTTTAGATC

ACCCCCTCCCCAATAAAGCTAAAACTCACCTGAGTTGTAAAAAACTCCAGTTGACACAAA

ATAGACTACGAAAGTGGCTTTAACATATCTGAACACACAATAGCTAAGACC

>131_Sample_ASV33

CACCGCGGTTATACGAGAGGCCCCAGTTGATAAACACGGCGTAAAGGGTGGTTAAGGGAG

CATAAGAATAAAGCCGAAGGGCCCTTTGGCCGTTATACGCTTCTAGGTGTCCGAAGCCCA

AACACGAAAGTAGCTTTAGTTAAGCCCACCTGACTCCACGAAAACTGAGAAA

>131_Sample_ASV34

CACCGCGGTTATACGAGAGACCCTAGTTGATAAATACGGCGTAAAGAGTGGTTATGGGAA

AAACAAATAAAGTCAAACAACCTCTTAGCTGTTATACGCATTATGAGAGCATGAAGCCCC

CTCACGAAAGTAACTTTAACATTTCCTGACCCCACGAAAGCTGTGAAA

>131_Sample_ASV35

CACCGCGGTTATACGAGAGACCCTAGTTGATAAATACGGCGTAAAGAGTGGTTATGGGAA

AAACAAATAAAGTCAAACAACCTCTTAGCTGTTATACGCATTATGAGAGCATGAAGCCCC

CTCACGAAAGTAACTTTACCCCCTCCTGACCCCACGAAAGCTAAGAAA

>131_Sample_ASV36

CACCGCGGTTATACGAGAGACCCTAGTTGATAAATACGGCGTAAAGAGTGGTTATGGGAA

AAACAAATAAAGTCAAACAACCTCTTAGCTGTTATACGCATTATGAGAGCATGAAGCCCC

CTCACGAAAGTAACTTTAATACCTCCTGACCCCACGAAAGCTAAGAAA

>131_Sample_ASV37

CACCGCGGTTATACGAGAGGCTCAAGTTGATGAACCCCGGCGTAAAGAGTGGTTAAGAGA

GATCAAAACTAAAGCCGAATGCTTTCAAAGCTGTTATACGCTTCCGAAAGTAAGAAGCCC

AATCACGAAAGTGGCTTTACTTTACCTGACCCCACGAAAGCTACGACA

>131_Sample_ASV38

CACCGCGGTTATACGAGAGGCTCAAGTTGATAAACCCCGGCGTAAAGAGTGGTTAAGGAA

AAATAGAAACTAAAGCCGAATGCTTTCAAGACTGTTATACGTTCCCGAGAGTAAGAAGAC

CAATTACGAAAGTGGCTTTACTTTACCTGACCCCACGAAAGCTACGACA

>131_Sample_ASV39

CACCGCGGTTATACGAGAGGCTCAAGTTGATGAACCCCGGCGTAAAGAGTGGTTAAGAGA

GATCAAAACTAAAGCCGAATGCTTTCAAAGCTGTTATACGCTTCCGAAAGTAAGGAGCCC

AATCACGAAAGTGGCTTTACTTTACCTGACCCCACGAAAGCTACGACA

>131_Sample_ASV40

CACCGCGGTTATACGAGAGGCTCAAGTTGATAAACCCCGGCGTAAAGAGTGGTTAAGGAA

AAATAGAAACTAAAGCCGAATGCTTTCAAGACTGTTATACGTTCCCGAGAGTAAGAAGAC

CAATTACGAAAGTGGCTTTACTTTACCTGACCCCACGAAAGCTGTGAAA

>131_Sample_ASV41

CACCGCGGTTATACGAGAGGCCCAAGTTGACAAACCCCGGCGTAAAGAGTGGTTAAGGGA

GATCAAAACTAAAGCCGAATGCTTTCAAAGCTGTTATACGCTTCCGAAAGTAAGAAGCTC

AATCACGAAAGTGGCTTTACTTTACCTGACCCCACGAAAGCTACGACA

>131_Sample_ASV42

CACCGCGGTTATACGAGAGGCTCAAGTTGATGAACCCCGGCGTAAAGAGTGGTTAAGGGA

GATCAAAACTAAAGCCGAATGCTTTCAAAGCTGTTATACGCTTCCGAAAGTAAGAAGCTC

AATCACGAAAGTGGCTTTACTTTACCTGACCCCACGAAAGCTACGACA

>131_Sample_ASV43

CACCGCGGTTATACGAAAGACCCTAGTTGATGGATCACGGCGTAAAGGGTGGTTAAGGGA

TATAAAAATAAAGCTAAAAATCCTCTTAGCTGTCATACGCTTTCCGAAGACATGAGGCCC

AATTACGAAAGTAGCTTTAAACCACCCTGACACCACGAAAGCCAAGAAA

>131_Sample_ASV44

CACCGCGGTTATACGAAAGACCCCAGTTTATAGATGCGGCGTAAAGGGTGGTTAAGAGAA

AATCAAAATAAGGCCAAAGATCCCCTAAGCAGTCATACGCTCCACGAGGACACGAAACCC

AAATACGAAAGTTGCCTTAATAAACTTCACCTGATCCCACGAAAGCTGAAAAA

>131_Sample_ASV45

CACCGCGGTTATACGAGAGGCCCAAGTTGACAGGTACCGGCGTAAAGAGTGGTTAAGAAA

AAATATTAAACTAAAGCCGAACACCTTCAGAGCTGTCATACGTTTCCGAAGGCATGAAGC

CCCACCACGAAAGTGGCTTTATTTTGCCCGACTCCACGAAAGCTGTGAAA

>131_Sample_ASV46

CACCGCGGTTATACGAGAGGCCCAAGTTGACAAACCCCGGCGTAAAGAGTGGTTAAGAAA

AATTTAAAACTAAAGCCGAATGCCCTCAAAGCTGTTATACGCACCCGAAGGTAAGAAGCC

CAATCACGAAAGTGGCTTTACATTTCCCGAACGCACGAAAGCTGAGGAA

>131_Sample_ASV47

CACCGCGGTTATACGAGAGGCCCAAGTTGACAAACCCCGGCGTAAAGAGTGGTTAAGGAA

AATTTAAAACTAAAGCCGAATGCCCCCAAAGCTGTTATACGCACCCGAGGATAAGAAGCC

CAATCACGAAAGTGGCTTTACACTACCTGAACCCACGAAAGCTATGAAA

>131_Sample_ASV48

CACCGCGGTTATACGAGAGGCCCAAGTTGACAAACCCCGGCGTAAAGAGTGGTTAAGGAA

AATTTAAAACTAAAGCCGAATGCCCCCAAAGCTGTTATACGCACCCGAGGGTAAGAAGCC

CAATCACGAAAGTGGCTTTACACTACCTGAACCCACGAAAGCTATGAAA

>131_Sample_ASV49

CACCGCGGTTATACGAGAGGCCCTAGTTGATAACTACCGGCGTAAAGAGTGGTTATGGAA

AAATATTTAATAAAGCCGAACACCCCCTCAGCCGTCATACGCACCTGGGAGCACGAAGAC

CTACTGCGAAAGCAGCTTTAATTATGCCTGACCCCACGACAGCTAAGAAA

>131_Sample_ASV50

CACCGCGGTTATACGAAAGACCCTAGTTTATAGACACGGCGTAAAGGGTGGTTCGGGACT

AAACAAAATAAAGCCAAAGACCTCCTAAGCCGTCATACGCTCACGGAAGCACGAAGCCCA

AGTACGAAAGTAGCTTTACAAAACATGCCTGACTCCACGAAAGCTAGGAAA

>131_Sample_ASV51

CACCGCGGTTATACGAGAGGCCCAAGTTGATACTCCACGGCGTAAAGAGTGGTTAGAAAA

GACCTACTACTAAAGCCGAACGCCTTCAAAGCTGTTATACGCATCCGAAGGTGAGAAGCC

CACCCACGAAAGTGGCTTTACAACCTTGACTCCACGAAAGCTATGACA

>131_Sample_ASV52

CACCGCGGTTATACGAGAGGCCCAAGTTGATACTCCACGGCGTAAAGAGTGGTTAGAAAA

GACCTACTACTAAAGCCGAACGCCTTCAAAGCTGTTATACGCATCCGAAGGTGAGAAGCC

CACCCACGAAAGTGGCTTTACAACCTTGACTCCACGAAAGCTGTGAAA

>131_Sample_ASV53

CACCGCGGTTATACGAGAGGCCCAAGTTGATAGTCTTCGGCGTAAAGAGTGGTTAAAGAA

GCAATTAGACTAAGGCTAAACTCTTCCAAGGCTGTCATACGCACCCGGAAGCATGAAACC

CAACCACGAAAGTGGCCTTATTTACCTTGACCCCACGAAAGCTGTGAAA

>131_Sample_ASV54

TACCGCGGTTATACGAGAGGCCCAAGTTGATAGTCCTCGGCGTAAAGAGTGGTTAGAGAA

TCTATTAAACTAAGGCTAAACCCTTCCAAAGCCGTCATACGCATCCGGAAGCATGAAACC

CACCCACGAAAGTAGCCTTATTTACCTTGACCCCACGAAAGCTGTGAAA

>131_Sample_ASV55

CACCGCGGTTATACGAGAGGCCCAAGTTGATAGTCCTCGGCGTAAAGAGTGGTTAGAGAA

TCTATTAAACTAAGGCTAAACCCTTCCAAAGCCGTCATACGCATCCGGAAGCATGAAACC

CACCCACGAAAGTGACCTTATTTACCTTGACCCCACGAAAGCTGCGAAA

>131_Sample_ASV56

CACCGCGGTTATACGAGAGACCCCAGTTGATAAACACGGCGTAAAGAGTGGTTAAGGGCA

CAAAACAAATAAAGTCAAACAACCTCTTAGCTGTTATACGCATTATGAGGATATGAAGCC

CCCCTACGAAAGTAACTTTACCAACCCCTGACCCCACGAAAGCTAAGAAA

>131_Sample_ASV57

CACCGCGGTTATACGAGAGACCCCAGTTGATAAACACGGCGTAAAGAGTGGTTAAGGGCA

CAAAACAAATAAAGTCAAACAACCTCTTAGCTGTTATACGCATTATGAGGATATGAAGCC

CCCCTACGAAAGTAACTTTACCCCCTCCTGACCCCACGAAAGCTAAGAAA

>131_Sample_ASV58

CACCGCGGTTATACGAAAGACCCTAGTTTATAGGCACGGCGTAAAGGGTGGTTAGGGGAT

AAACAAAATAAAGCCAAAGACCTTCTAAGTCGTCATACACTCATGAAGGCACGAAGCCCA

AATACGAAAGTAACTTTACAACTTAGACCCGACTCCACGAAAGCTGGGGGA

>131_Sample_ASV59

CACCGCGGTTATACGAGAGGCCCAAGTTGACAAACTCCGGCGTAAAGCGTGGTTAAGGCA

CACTCAAAAACTAAAGCCGAATATCTTCAGAGCAGTCATACGCATCCGAAGACACGAAGC

CCCACCACGAAAGTGGCTTTAACCACACCCGACCCCACGAAAGCTGTGAAA

>131_Sample_ASV60

CACCGCGGTTATACGAGAGGCCCAAGTTGACAAACTCCGGCGTAAAGCGTGGTTAAGGCA

CACTCAAAAACTAAAGCCGAATATCTTCAGAGCAGTCATACGCATCCGAAGACACGAAGC

CCCACCACGAAAGTGGCTTTATGACACCTGACCCCACGAAAGCTAGGACA

>131_Sample_ASV61

CACCGCGGTCATACGATTAACCCAAATTAATAGATCCACGGCGTAAAGAGTGTTTAAGaa

aaaaaaaCCACAATAGAGTTAAATTATAACTAAGCTGTAAAAAGCCCTAGTTAAAATAAA

ATAACCCACGAAAGTGACTCTAATAATCCTGACACACGATAGCTAGGACC

>137_Sample_ASV1

CACCGCGGTTATACGAAAGACCCTAGTTGATGGGTGCGGCGTAAAGGGTGGTTAGGGACT

ACAAAAAATAGAGCTAAAGATCCTCTAGGTCGTCATACACATCACGAGAAAACGAAGCCC

AAATACGAAAGTAGCTCTAAATATAATCCTGACGCCACGAAAGCCAAGAAA

>137_Sample_ASV2

CACCGCGGTTATACGAAAGACCCTAGTTGATGGGTGCGGCGTAAAGGGTGGTTAGGGACT

ACAAAAAATAGAGCTAAAGATCCTCTAGGTCGTCATACACATCACGAGGAAACGAAGCCC

AAATACGAAAGTAGCTCTAAATATAATCCTGACGCCACGAAAGCCAAGAAA

>137_Sample_ASV3

CACCGCGGTTATACGAGAGACCCTAGTTGATTAAAGCGGCGTAAAGAGTGGTTATGGAAC

TATTCTTTTAAAGCAGAAAACCTCTCAAACTGTTATACGCACCCAGAGGTCTGAATCCCT

CACACGAAAGTGACTTTATTTATGCCTACCAGAACCCACGAAAGCTGGGACA

>137_Sample_ASV4

CACCGCGGTTATACGAAAGACCCTAGTTTATAGGTACGGCGTAAAGGGTGGTTAGGGGAC

AAACAAAATAAAGCCAAAGACCTCCTAAGCCGTCATACGCTCACGGAAGCACGAAGCCCA

AACACGAAAGTAGCTTTACCGAACATGCCCGACTCCACGAAAGCTGGGGAA

>137_Sample_ASV5

CATGGCGATTATACGAGAGGCCCAAGTTGACAGACACTGGCGTAAAGAGTGGCTAAGGAA

AAACTACTACTAAAGCCGAACACCTTCAGAACTGTCATACGTTCCCGAAGATAAGAAGCC

CCACCACGAAAGTGGCTTTATATTACCCGACCCCACGAAAGCTGTGAAA

>137_Sample_ASV6

CATGGCGATTATACGAGAGGCCCAAGTTGACAGACACTGGCGTAAAGAGTGGCTAAGGAA

AAATTACTACTAAAGCCGAACACCTTCAGAACTGTCATACGTTCCCGAAGATAAGAAGCC

CCACCACGAAAGTGGCTTTATATTACCCGACCCCACGAAAGCTGTGAAA

>137_Sample_ASV7

CACCGCGGTTATACGAGAGGCCCAAGTTGACAGACACCGGCGTAAAGAGTGGCTAGGGAA

AAATTACTACTAAAGCCGAACACCCTCAGAACTGTCATACGTTCCAGAAGGCAAGAAGCT

CCACCACGAAAGTGGCTTTATATTACCCGACCCCACGAAAGCTGTGAAA

>137_Sample_ASV8

CACCGCGGTTATACGAGAGGCCCAAGTTGACAGACACCGGCGTAAAGAGTGGCTAGGGAA

AAATTACTACTAAAGCCGAACACCTTCAGAACTGTCATACGTTCCCGAAGATAAGAAGCC

CCACCACGAAAGTGGCTTTACAATGCCTGACCCCACGAAAGCTGTGAAA

>137_Sample_ASV9

CATGGCGATTATACGAGAGGCCCAAGTTGACAGACACTGGCGTAAAGAGTGGCTAGGGAA

AAATTACTACTAAAGCCGAACACCTTCAGAACTGTCATACGTTCCCGAAGATAAGAAGCC

CCACCACGAAAGTGGCTTTATATTACCCGACCCCACGAAAGCTGTGAAA

>137_Sample_ASV10

CACCGCGATTATACGAGAGGCCCAAGTTGACAGACATCGGCGTAAAGAGTGGTTAAGGAA

AAATTACTACTAAAGCCGAACACCTTCAGAACTGTCATACGTTCCCGAAGATAAGAAGCC

CCACCACGAAAGTGGCTTTATATTACCCGACCCCACGAAAGCTGTGAAA

>137_Sample_ASV11

CACCGCGGTTATACGAGAGGCCCAAGTTGACAGACACCGGCGTAAAGAGTGGCTAGGGAA

AAATTACTACTAAAGCCGAACACCTTCAGAACTGTCATACGTTCCCGAAGATAAGAAGCC

CCACCACGAAAGTGGCTTTATATTACCCGACCCCACGAAAGCTGTGAAA

>137_Sample_ASV12

CACCGCGGTTATACGAGAGACCCTAGTTGATATATACGGCGTAAAGAGTGGTTTGGGATA

TTTCTCCAAAATAAAGCCGAAGATCTCCCAAGCCGTCACACGCATCCCGGAGACACGAAG

CCCTAATACGAAGGTAGCTTTATTAATTCCCCGACGCCACGAAAGCTAAGAAA

>137_Sample_ASV13

CACCGCGGTTATACGAGAGACCCTAGTTGATATATACGGCGTAAAGAGTGGTTTGGGGTT

ATGCCTCCAAAATAAAGCCGAAGATCTCCCAAGCCGTCACACGCATCCCGGAGACACGAA

GCCCTAATACGAAGGTAGCTTTATTAGTACCCCGACGCCACGAAAGCTAAGAAA

>137_Sample_ASV14

CACCGCGGTTATACGAGAGACCCTAGTTGATATATACGGCGTAAAGAGTGGTTTGGGGTT

ATGCCTCCAAAATAAAGCCGAAGATCTCCCAAGCCGTCACACGCATCCCGGAGACACGAA

GCCCTAATACGAAGGTAGCTTTATTAATTCCCCGACGCCACGAAAGCTAAGAAA

>137_Sample_ASV15

CACCGCGGTTATACGAGAGGCCCAAGTTGATAAGCCCGGCGTAAAGAGTGGTTATGGAGA

ATAAAACACTAAAGCTAAAGACCCCCTAGGCTGTTATACGCACCTGGCGGCTCGAACCAC

CTATACGAAAGTAGCTTTACCCCCCTTCCACCAGAATCCACGACAGCTGGGGCA

>137_Sample_ASV16

CACCGCGGTTATACGAGAGGCCCTAGTTGATAGCCACGGCGCAAAGAGTGGTTAAGGAGC

CCCACTAAATAAAGTCGAACACTTCCTAGGCCGTTATACGTTTTCTAGAAGCACGAAACC

CAATTACACGAAAGCAACTTTATACTAAACAACCTGACCCCACGAAAGCTAAGAAA

>137_Sample_ASV17

CACCGCGGTTATACGAGAGGCCCAAGTTGACAGACATCGGCGTAAAGAGTGGTTAAGGAA

ACCCCATTACTAAAGCCGAACGCCCTCAGAACTGTCATACGTTCCCGAAGGTAAGAAGCC

CCACTACGAAAGTGGCTTTAACCACACCCGACCCCACGAAAGCTAAGGAA

>137_Sample_ASV18

CACCGCGGTTATACGAGAGGCCCAAGTTGACAGACATCGGCGTAAAGAGTGGTTAAGGAA

ACCCCATTACTAAAGCCGAACGCCCTCAGAACTGTCATACGTTCCCGAAGGTAAGAAGCC

CCACTACGAAAGTGGCTTTAACCACACCCGACCCCACGAAAGCTGTGAAA

>137_Sample_ASV19

CACCGCGGTTATACGAGAGGCCCAAGTTGACAGACATCGGCGTAAAGAGTGGTTAAGGAA

ACCCCATTACTAAAGCCGAACGCCCTCAGAACTGTCATACGTTCCCGAAGGTAAGAAGCC

CCACTACGAAAGTGGCTTTAACCACACCCGACCCCACGAAAGCTGGGACA

>137_Sample_ASV20

CACCGCGGTTATACGAGAGGCCCAAGTTGATAGATATCGGCGTAAAGAGTGGTTAAGGAA

ACCCCGTTACTAAAGCCGAACGCCCTCAGAACTGTCATACGTTCCCGAAGGTAAGAAGCC

CCACTACGAAAGTGGCTTTAACCACACCCGACCCCACGAAAGCTGTGAAA

>137_Sample_ASV21

CACCGCGGTTATACGAGAGACCCTAGTTGATAGAGACGGCGTAAAGAGTGGTTAAGGAAA

CCCCATTACTAAAGCCGAACGCCCTCAGAACTGTCATACGTTCCCGAAGGTAAGAAGCCC

CACTACGAAAGTGGCTTTAACCACACCCGACCCCACGAAAGCTGTGAAA

>137_Sample_ASV22

CACCGCGGTTATACGAGAGACCCTAGTTGATAGAGACGGCGTAAAGAGTGGTTAAGGAAA

CCCCATTACTAAAGCCGAACGCCCTCAGAACTGTCATACGTTCCCGAAGGTAAGAAGCCC

CACTACGAAAGTGGCTTTAACCACACCCGACCCCACGAAAGCTGGGACA

>137_Sample_ASV23

CACCGCGGTTATACGAGAGGCCCAAGTTGACAGACATCGGCGTAAAGAGTGGTTAAGGAA

ACCCCATTACTAAAGCCGAACGCCCTCAGAACTGTCATACGTTCCCGAAGGTAAGAAGCC

CCACTACGAAAGTGACTTTACCACCTCCTGACCCCACGAAAGCTGTGAAA

>137_Sample_ASV24

CACCGCGGTTATACGAGAGGCCCAAGTTGACAGACACCGGCGTAAAGAGTGGTTAAGGAA

ATTCATCAACTAAAGCCGAATGCCCTCAGGACTGTTATACGTTTGCGAAGGTAAGAAGCC

CTACTACGAAAGTGGCTTTATTCCACCTGATTCCACGAAAGCTGCGGAA

>137_Sample_ASV25

CACCGCGGTTATACGAGAGGCCCCAGTTGATAAACACGGCGTAAAGGGTGGTTAAGGGAG

CATAAGAATAAAGCCGAAGGGCCCTTTGGCCGTTATACGCTTCTAGGTGTCCGAAGCCCA

AACACGAAAGTAGCTTTAGTTAAGCCCACCTGACTCCACGAAAACTGAGAAA

>137_Sample_ASV26

CACCGCGGTTATACGAGAGACCCTAGTTGATAAATACGGCGTAAAGAGTGGTTATGGGAA

AAACAAATAAAGTCAAACAACCTCTTAGCTGTTATACGCATTATGAGAGCATGAAGCCCC

CTCACGAAAGTAACTTTAATACCTCCTGACCCCACGAAAGCTAAGAAA

>137_Sample_ASV27

CACCGCGGTTATACGAGAGGCTCAAGTTGATGAACCCCGGCGTAAAGAGTGGTTAAGGGA

GATCAAAACTAAAGCCGAATGCTTTCAAAGCTGTTATACGCTTCCGAAAGTAAGAAGCTC

AATCACGAAAGTGGCTTTACTTTACCTGACCCCACGAAAGCTACGACA

>137_Sample_ASV28

CACCGCGGTTATACGAGAGACCCTAATTGATAGACACGGCGTAAAGAGTGGTTTGGGAAA

GCCCAAAAATAAAGCTAAAGACCTTCTAAGCCGTCACACGCACGCCGAAGGCACGAAGCC

CTGACACGAAAGTAGCTTTACTATTACCCCCGACGCCACGAAAGCTAAGAAA

>137_Sample_ASV29

CACCGCGGTTATACGAAAGACCCTAGTTGATGGATCACGGCGTAAAGGGTGGTTAAGGGA

TATAAAAATAAAGCTAAAAATCCTCTTAGCTGTCATACGCTTTCCGAAGACATGAGGCCC

AATTACGAAAGTAGCTTTAAACCACCCTGACACCACGAAAGCCAAGAAA

>137_Sample_ASV30

CACCGCGGTTATACGAAAGACCCCAGTTTATAGATGCGGCGTAAAGGGTGGTTAAGAGAA

AATCAAAATAAGGCCAAAGATCCCCTAAGCAGTCATACGCTCCACGAGGACACGAAACCC

AAATACGAAAGTTGCCTTAATAAACTTCACCTGATCCCACGAAAGCTGAAAAA

>137_Sample_ASV31

CACCGCGGTTATACGAGAGGCCCAAGTTGACAAACCCCGGCGTAAAGAGTGGTTAAGGAA

AATTTAAAACTAAAGCCGAATGCCCCCAAAGCTGTTATACGCACCCGAGGGTAAGAAGCC

CAATCACGAAAGTGGCTTTACACTACCTGAACCCACGAAAGCTATGAAA

>137_Sample_ASV32

CACCGCGGTTATACGAGAGGCCCAAGTTGACAAACCCCGGCGTAAAGAGTGGTTAAGGAA

AATTTAAAACTAAAGCCGAATGCCCCCAAAGCTGTTATACGCACCCGAGGATAAGAAGCC

CAATCACGAAAGTGGCTTTACACTACCTGAACCCACGAAAGCTATGAAA

>137_Sample_ASV33

CACCGCGGTTATACGAGAGGCCCTAGTTGATAACTACCGGCGTAAAGAGTGGTTATGGAA

AAATATTTAATAAAGCCGAACACCCCCTCAGCCGTCATACGCACCTGGGAGCACGAAGAC

CTACTGCGAAAGCAGCTTTAATTATGCCTGACCCCACGACAGCTAAGAAA

>137_Sample_ASV34

CACCGCGGTTATACGAAAGACCCTAGTTTATAGACACGGCGTAAAGGGTGGTTCGGGACT

AAACAAAATAAAGCCAAAGACCTCCTAAGCCGTCATACGCTCACGGAAGCACGAAGCCCA

AGTACGAAAGTAGCTTTACAAAACATGCCTGACTCCACGAAAGCTAGGAAA

>137_Sample_ASV35

CACCGCGGTTATACGAGAGGCCCAAGTTGATACTCCACGGCGTAAAGAGTGGTTAGAAAA

GACCTACTACTAAAGCCGAACGCCTTCAAAGCTGTTATACGCATCCGAAGGTGAGAAGCC

CACCCACGAAAGTGGCTTTACAACCTTGACTCCACGAAAGCTATGACA

>137_Sample_ASV36

CACCGCGGTTATACGAGAGACCCCAGTTGATAAACACGGCGTAAAGAGTGGTTAAGGGCA

CAAAACAAATAAAGTCAAACAACCTCTTAGCTGTTATACGCATTATGAGGATATGAAGCC

CCCCTACGAAAGTAACTTTACCAACCCCTGACCCCACGAAAGCTAAGAAA

>137_Sample_ASV37

CACCGCGGTTATACGAAAGACCCTAGTTTATAGGCACGGCGTAAAGGGTGGTTAGGGGAT

AAACAAAATAAAGCCAAAGACCTTCTAAGTCGTCATACACTCATGAAGGCACGAAGCCCA

AATACGAAAGTAACTTTACAACTTAGACCCGACTCCACGAAAGCTGGGGGA

>137_Sample_ASV38

CACCGCGGTTATACGAAAGACCCTAGTTGATAGCTACGGCGTAAAGGGTGGTTAAGGTAT

ATAACAAATAAAGCTAAAGAGCCTCCAAGCCGTCGTACGCATTCTGAGAGCTCGAAACCC

TAACACGAAAGTAGCTTTAAAAAATAACACCTGACCCCACGAAAGCTAAGAAA

>138_Sample_ASV1

CACCGCGGTTATACGAGAGGCCCAAGTTGACAGACATCGGCGTAAAGAGTGGTTAAGGAA

AACCTTCAACTAAAGTCGAACGCTCTCAAGACTGTCGTACGTTTCCGAGAGTATGAAGCC

CCACCACGAAAGTGACTTTACCTCACCTGACTCCACGAAAGCTGCGAAA

>138_Sample_ASV2

CACCGCGGTTATACGAAAGACCCTAGTTGATGGGTGCGGCGTAAAGGGTGGTTAGGGACT

ACAAAAAATAGAGCTAAAGATCCTCTAGGTCGTCATACACATCACGAGGAAACGAAGCCC

AAATACGAAAGTAGCTCTAAATATAATCCTGACGCCACGAAAGCCAAGAAA

>138_Sample_ASV3

CACCGCGGTTATACGAGAGACCCTAGTTGATTAAAGCGGCGTAAAGAGTGGTTATGGAAC

TATTCTTTTAAAGCAGAAAACCTCTCAAACTGTTATACGCACCCAGAGGTCTGAATCCCT

CACACGAAAGTGACTTTATTTATGCCTACCAGAACCCACGAAAGCTATGACA

>138_Sample_ASV4

CACCGCGGTTATACGAGAGACCCTAGTTGATTAAAGCGGCGTAAAGAGTGGTTATGGAAC

TATTCTTTTAAAGCAGAAAACCTCTCAAACTGTTATACGCACCCAGAGGTCTGAATCCCT

CACACGAAAGTGACTTTATTTATGCCTACCAGAACCCACGAAAGCTGGGACA

>138_Sample_ASV5

CACCGCGGTTATACGAAAGACCCTAGTTTATAGGTACGGCGTAAAGGGTGGTTAGGGGAC

AAACAAAATAAAGCCAAAGACCTCCTAAGCCGTCATACGCTCACGGAAGCACGAAGCCCA

AACACGAAAGTAGCTTTACCGAACATGCCCGACTCCACGAAAGCTGGGGAA

>138_Sample_ASV6

CACCGCGGTTATACGAGAGGCCCAAGTTGACAGACACCGGCGTAAAGAGTGGCTAGGGAA

AAATTACTACTAAAGCCGAACACCTTCAGAACTGTCATACGTTCCCGAAGATAAGAAGCC

CCACCACGAAAGTGGCTTTATATTACCCGATTCCACGAAAGCTAAGAAA

>138_Sample_ASV7

CACCGCGGTTATACGAGAGGCCCAAGTTGACAGACACCGGCGTAAAGAGTGGCTAGGGAA

AAATTACTACTAAAGCCGAACACCTTCAGAACTGTCATACGTTCCCGAAGATAAGAAGCC

CCACCACGAAAGTGGCTTTATATTACCCGACCCCACGAAAGCTAAGAAA

>138_Sample_ASV8

CACCGCGGTTATACGAGAGGCCCAAGTTGACAGACACCGGCGTAAAGAGTGGCTAGGGAA

AAATTACTACTAAAGCCGAACACCTTCAGAACTGTCATACGTTCCCGAAGATAAGAAGCC

CCACCACGAAAGTGGCTTTATATTACCCGACCCCACGAAAGCTGTGAAA

>138_Sample_ASV9

CACCGCGGTTATACGAGAGGCCCAAGTTGACAGACACCGGCGTAAAGAGTGGCTAAGGAA

AAATTTCTACTAAAGCCGAACACCTTCAGAACTGTCATACGTTCCCGAAGAAAAGAAGCC

CCACCACGAAAGTGGCTTTATATTACCCGACCCCACGAAAGCTGTGAAA

>138_Sample_ASV10

CACCGCGGTTATACGAGAGGCCCAAGTTGACAGACACCGGCGTAAAGAGTGGCTAAGGAA

AAATTTCTACTAAAGCCGAACACCTTCAGAACTGTCATACGTTCCCGAAGAAAAGAAGCC

CCACCACGAAAGTGGCTTTATATTACCCGACCCCACGAAAGCTAAGAAA

>138_Sample_ASV11

CACCGCGGTTAAACGAGAGACCCTAGTTGATCATCACGGCGTAAAGAGTGGTTAGGGGAT

TATCATAAATAAAGCCAAAGACCTCCCAAGCTGTCGCACGCATTCCGGAGGCGCGAAGCC

CACACACGAAAGTAGCTTTAACTATTGACCCTGATGCCACGAAAGCTAAGAAA

>138_Sample_ASV12

CACCGCGGTTATACGAGAGGCCCAAGTTGATAAGCCCGGCGTAAAGAGTGGTTATGGAGA

ATAAAACACTAAAGCTAAAGACCCCCTAGGCTGTTATACGCACCTGGCGGCTCGAACCAC

CTATACGAAAGTAGCTTTACCCCCCTTCCACCAGAATCCACGACAGCTGGGGCA

>138_Sample_ASV13

CACCGCGGTTATACGAGAGGCCCAAGTTGACAGACATCGGCGTAAAGAGTGGTTAAGGAA

ACCCCATTACTAAAGCCGAACGCCCTCAGAACTGTCATACGTTCCCGAAGGTAAGAAGCC

CCACTACGAAAGTGGCTTTAACCACACCCGACCCCACGAAAGCTGTGAAA

>138_Sample_ASV14

CACCGCGGTTATACGAGAGGCCCAAGTTGATAGATATCGGCGTAAAGAGTGGTTAAGGAA

ACCCCGTTACTAAAGCCGAACGCCCTCAGAACTGTCATACGTTCCCGAAGGTAAGAAGCC

CCACTACGAAAGTAGCTTTAACCACACCCGACCCCACGAAAGCTGTGAAA

>138_Sample_ASV15

CACCGCGGTCACACGATTAACCCAAGTCAATAGAAGCCGGCGTAAAGAGTGTTTTAGATC

ACCCCCTCCCCAATAAAGCTAAAACTCACCTGAGTTGTAAAAAACTCCAGTTGACACAAA

ATAGACTACGAAAGTGGCTTTAACATATCTGAACACACAATAGCTAAGACC

>138_Sample_ASV16

CACCGCGGTTATACGAGAGGCCCTAGTTGATAAATACGGCGTAAAGAGTGGTTAGAGGAC

TCCCATAATAAAGCCAAAGACCCCCCGGGCCGTCACACGCATACGAGGGCACGAAGTTCA

CACACGAAAGTAGCTTTAAATTACCCGACGCCACGAAAGCTAAGAAA

>138_Sample_ASV17

GACCGCGGTTATACGAGAGACTCTAGTTGATAGATACGGCGTAAAGAGTGGTTTGGGATA

AACTATAAATAAAGCCAAAGACCTTCCAAGCTGTTAAACGCATACCGAAGGCACGAAGCC

CCAACACGAAAGTAGCTTTACTACTACCCCCGATGCCACGAAAGCTAAGACA

>138_Sample_ASV18

CACCGCGGTTATACGAGAGACCCTAGTTGATAAATACGGCGTAAAGAGTGGTTATGGGAA

AAACAAATAAAGTCAAACAACCTCTTAGCTGTTATACGCATTATGAGAGCATGAAGCCCC

CTCACGAAAGTAACTTTAATACCTCCTGACCCCACGAAAGCTAAGAAA

>138_Sample_ASV19

CACCGCGGTCATACGATTAACCCAAGTTAATAGGCACACGGCGTAAAGCGTGTTTAAGCA

CTACGCCAAATAAAGTTAAACTCCAATTAAGCTGTAAAAAGCCATAATCAAGACGAAAAT

AAATAACGAAAGTAACTTTACAACCGCTGAAACACGATAGCTAAGATC

>138_Sample_ASV20

CACCGCGGTCATACGATTAACCCAAGTTAATAGGCACACGGCGTAAAGCGTGTTTAAGCA

CTATGCCAAATAAAGTTAAACTCCAATTAAGCTGTAAAAAGCCATAATCAAGACGAAAAT

AAATAACGAAAGTAACTTTACAACCGCTGAAACACGATAGCTAAGATC

>138_Sample_ASV21

CACCGCGGTTATACGAAAGACCCTAGTTTATAGACACGGCGTAAAGGGTGGTTCGGGACT

AAACAAAATAAAGCCAAAGACCTCCTAAGCCGTCATACGCTCACGGAAGCACGAAGCCCA

AGTACGAAAGTAGCTTTACAAAACATGCCTGACTCCACGAAAGCTAGGAAA

>138_Sample_ASV22

CACCGCGGTTATACGAAAGACCCTAGTTGATAGGCACGGCGTAAAGGGTGGTTAGGGATA

TATCAAAAATAAAGTTAAAGAGCCTCTAAGCTGTCGCACGCATCCCGAGAGCTCGAGACC

CAGACACGAAAGTAACTTTAAAATACCTCACCTGACCCCACGAAAGCTAAGAAA

>138_Sample_ASV23

CACCGCGGTTATACGAAAGACCCTAGTTGATAGCCACGGCGTAAAGGGTGGTTAAGGTAT

ACAACAAATAAAGCTAAATAGCCTCTAGGCCGTCGCACGCATTCCGAGAGCTCGAAACCC

AAAAACGAAAGTAGCTTTAAAATAAACAGACCTGACCCCACGAAAGCTAAGAAA

>138_Sample_ASV24

CACCGCGGTTATACGAGAGGCCCAAGTTGATACTCCACGGCGTAAAGAGTGGTTAGAAAA

GACCTACTACTAAAGCCGAACGCCTTCAAAGCTGTTATACGCATCCGAAGGTGAGAAGCC

CACCCACGAAAGTGGCTTTACAACCTTGACTCCACGAAAGCTATGACA

>138_Sample_ASV25

CACCGCGGTTATACGAGAGACCCCAGTTGATAAACACGGCGTAAAGAGTGGTTAAGGGCA

CAAAACAAATAAAGTCAAACAACCTCTTAGCTGTTATACGCATTATGAGGATATGAAGCC

CCCCTACGAAAGTAACTTTACCAACCCCTGACCCCACGAAAGCTAAGAAA

>138_Sample_ASV26

CACCGCGGTTATACGAAAGACCCTAGTTTATAGGCACGGCGTAAAGGGTGGTTAGGGGAT

AAACAAAATAAAGCCAAAGACCTTCTAAGTCGTCATACACTCATGAAGGCACGAAGCCCA

AATACGAAAGTAACTTTACAACTTAGACCCGACCCCACGAAAGCTGTGAAA

>138_Sample_ASV27

CACCGCGGTTATACGAAAGACCCTAGTTTATAGGCACGGCGTAAAGGGTGGTTAGGGGAT

AAACAAAATAAAGCCAAAGACCTTCTAAGTCGTCATACACTCATGAAGGCACGAAGCCCA

AATACGAAAGTAACTTTACAACTTAGACCCGACTCCACGAAAGCTGGGGGA

>138_Sample_ASV28

CACCGCGGTTATACGAGAGGCCCAAGTTGATAGACATCGGCGTAAAGAGTGGTTAAGGAA

GTCTCAAAACTAAAGCCGAATGCCTTCAAGACTGTTATACGTTTCCGAAGGTAAGAAGCC

CCACTACGAAAGTGGCTTTACTCCACCTGACTCCACGAAAGCTAAGAAA

>138_Sample_ASV29

CACCGCGGTTATACGAGAGGCCCAAGTTGATAGACATCGGCGTAAAGAGTGGTTAAGGAA

GTCTCAAAACTAAAGCCGAATGCCTTCAAGACTGTTATACGTTTCCGAAGGTAAGAAGCC

CCACTACGAAAGTGGCTTTACTCCACCTGACTCCACGAAAGCTGTGAAA

>138_Sample_ASV30

CACCGCGGTTATACGAAAGACCCTAGTTGATAGATCACGGCGTAAAGGGTGGTTAAGGAG

AACAAAAATAAAGCTAAAAATCTTCTAAGCTGTCATACGCTTTCCGAAGACATGAGACCC

AACCACGAAAGTAGCTTTAAACTTTCCTGACACCACGAAAGCCAAGAAA

>142_Sample_ASV1

CACCGCGGTTATACGAGAGGCCCAAGTTGACAGACATCGGCGTAAAGAGTGGTTAAGGAA

AACCTTCAACTAAAGTCGAACGCTCTCAAGACTGTCGTACGTTTCCGAGAGTATGAAGCC

CCACCACGAAAGTGACTTTACCTCACCTGACTCCACGAAAGCTGCGAAA

>142_Sample_ASV2

CACCGCGGTTATACGAAAGACCCTAGTTGATGGGTGCGGCGTAAAGGGTGGTTAGGGACT

ACAAAAAATAGAGCTAAAGATCCTCTAGGTCGTCATACACATCACGAGGAAACGAAGCCC

AAATACGAAAGTAGCTCTAAATATAATCCTGACGCCACGAAAGCCAAGAAA

>142_Sample_ASV3

CACCGCGGTTATACGAAAGACCCTAGTTGATAACTACGGCATAAAGGGTGGTTAGGGAGT

TTAAAAAATAAAGCCAAAGCTCTTCTAAGCTGTCATACGCTCTCTGAAAGAACGAGGCCC

AAACACGAAAGTAGCTTTAAATAAATAACCTGATACCACGAAAGCCAAGAAA

>142_Sample_ASV4

CACCGCGGTTATACGAGAGACCCTAGTTGATTAAAGCGGCGTAAAGAGTGGTTATGGAAC

TATTCTTTTAAAGCAGAAAACCTCTCAAACTGTTATACGCACCCAGAGGTCTGAATCCCT

CACACGAAAGTGACTTTATTTATGCCTACCAGAACCCACGAAAGCTAAGAAA

>142_Sample_ASV5

CACCGCGGTTATACGAGAGACCCTAGTTGATTAAAGCGGCGTAAAGAGTGGTTATGGAAC

TATTCTTTTAAAGCAGAAAACCTCTCAAACTGTTATACGCACCCAGAGGTCTGAATCCCT

CACACGAAAGTGACTTTATTTATGCCTACCAGAACCCACGAAAGCTGGGACA

>142_Sample_ASV6

CACCGCGGTTATACGAGAGACTCCAGTTGACAGACACCGGCGTAAAGAGTGGCTAGGGAA

AAATTACTACTAAAGCCGAACACCTTCAGAACTGTCATACGTTCCCGAAGATAAGAAGCC

CCACCACGAAAGTGGCTTTATATTACCCGACCCCACGAAAGCTAAGAAA

>142_Sample_ASV7

CACCGCGATTATACGAGAGGCCCAAGTTGACAGACATCGGCGTAAAGAGTGGTTAAGGAA

AAATTACTACTAAAGCCGAACACCTTCAGAACTGTCATACGTTCCCGAAGATAAGAAGCC

CCACCACGAAAGTGGCTTTATATTACCCGACCCCACGAAAGCTGTGAAA

>142_Sample_ASV8

CACCGCGGTTATACGAGAGACTCCAGTTGATAGACACCGGCGTAAAGAGTGGCTAGGGAA

AAATTACTACTAAAGCCGAACACCTTCAGAACTGTCATACGTTCCCGAAGATAAGAAGCC

CCACCACGAAAGTGGCTTTATATTACCCGACCCCACGAAAGCTGTGAAA

>142_Sample_ASV9

CACCGCGGTTATACGAGAGACTCCAGTTGACAGACACCGGCGTAAAGAGTGGCTAGGGAA

AAATTACTACTAAAGCCGAACACCTTCAGAACTGTCATACGTTCCCGAAGATAAGAAGCC

CCACCACGAAAGTGGCTTTATATTACCCGACCCCACGAAAGCTGTGAAA

>142_Sample_ASV10

CACCGCGGTTATACGAGAGGCCCAAGTTGACAGACACCGGCGTAAAGAGTGGCTAGGGAA

AAATTACTACTAAAGCCGAACACCTTCAGAACTGTCATACGTTCCCGAAGATAAGAAGCC

CCACCACGAAAGTGGCTTTATATTACCCGACCCCACGAAAGCTAAGAAA

>142_Sample_ASV11

CACCGCGGTTATACGAGAGGCCCAAGTTGACAGACACCGGCGTAAAGAGTGGCTAGGGAA

AAATTACTACTAAAGCCGAACACCTTCAGAACTGTCATACGTTCCCGAAGATAAGAAGCC

CCACCACGAAAGTGGCTTTATATTACCCGACCCCACGAAAGCTGTGAAA

>142_Sample_ASV12

CACCGCGGTTATACGAGAGGCCCAAGTTGACAGACACCGGCGTAAAGAGTGGCTAGGGAA

AAATTACTACTAAAGCCGAACACCTTCAGAACTGTCATACGTTCCCGAAGATAAGAAGCC

CCACCACGAAAGTGGCTTTACCTTCCCCGACGCCACGAAAGCTAAGAAA

>142_Sample_ASV13

CACCGCGGTTATACGAGAGGCCCAAGTTGACAGACACCGGCGTAAAGAGTGGCTAAGGAA

AAATTTCTACTAAAGCCGAACACCTTCAGAACTGTCATACGTTCCCGAAGAAAAGAAGCC

CCACCACGAAAGTGGCTTTATATTACCCGACCCCACGAAAGCTAAGAAA

>142_Sample_ASV14

CACCGCGGTTATACGAAAGACCCTAGTTTATAGAAACGGCGTAAAGGGTGGTTAAGGGAC

AAACAAAATAAGGCCAAAGACTTCCTAAGCCGTCATACGCTCCACGAAAGCACGAAGCCC

AAACACGAAAGTAGCCTTAATAACTTCACCTGACCCCACGAAAGCTGGGAGA

>142_Sample_ASV15

CACCGCGGTTATACGAGAGACCCTAGTTGATATATACGGCGTAAAGAGTGGTTTGGGATA

TTTCTCCAAAATAAAGTCGAAGATCTCCCAAGCCGTCACACGCATCCCGGAGACACGAAG

CCCTGATACGAAGGTAGCTTTATTAACACCCCGACGCCACGAAAGCTAAGAAA

>142_Sample_ASV16

CACCGCGGTTATACGAGAGGCCCAAGTTGATAAGCCCGGCGTAAAGAGTGGTTATGGAGA

ATAAAACACTAAAGCTAAAGACCCCCTAGGCTGTTATACGCACCTGGCGGCTCGAACCAC

CTATACGAAAGTAGCTTTACCCCCCTTCCACCAGAATCCACGACAGCTGGGGCA

>142_Sample_ASV17

CACCGCGGTTATACGAGAGGCCCAAGTTGACAGACATCGGCGTAAAGAGTGGTTAAGGAA

ACCCCATTACTAAAGCCGAACGCCCTCAGAACTGTCATACGTTCCCGAAGGTAAGAAGCC

CCACTACGAAAGTGGCTTTAACCACACCCGACCCCACGAAAGCTGTGAAA

>142_Sample_ASV18

CACCGCGGTTATACGAGAGGCCCAAGTTGATAGATATCGGCGTAAAGAGTGGTTAAGGAA

ACCCCGTTACTAAAGCCGAACGCCCTCAGAACTGTCATACGTTCCCGAAGGTAAGAAGCC

CCACTACGAAAGTGGCTTTAACCACACCCGACCCCACGAAAGCTGTGAAA

>142_Sample_ASV19

CACCGCGGTTATACGAGAGGCCCAAGTTGACAGACACCGGCGTAAAGAGTGGTTAAGGAA

ATTCATCAACTAAAGCCGAATGCCCTCAGGACTGTTATACGTTTGCGAAGGTAAGAAGCC

CTACTACGAAAGTGGCTTTATTCCACCTGATTCCACGAAAGCTGCGGAA

>142_Sample_ASV20

CACCGCGGTTATACGAGAGGCCCCAGTTGATATTAGCGGCGTAAAGGGTGGTTAAGGACT

TTAATAACTAAAGCTAAACACTTCCCCGGCTGTTGTACGTAGACCGGAAGCTTGAAGCCC

AGACACGAAAGTAGCTTTATTCCCCCTGACCCCACGAAAGCTGAGAAA

>142_Sample_ASV21

CACCGCGGTCACACGATTAACCCAAGTCAATAGAAGCCGGCGTAAAGAGTGTTTTAGATC

ACCCCCTCCCCAATAAAGCTAAAACTCACCTGAGTTGTAAAAAACTCCAGTTGACACAAA

ATAGACTACGAAAGTGGCTTTAACATATCTGAACACACAATAGCTAAGACC

>142_Sample_ASV22

CACCGCGGTTATACGAGAGGCCCTAGTTGATAAATACGGCGTAAAGAGTGGTTAGAGGAC

TCCCATAATAAAGCCAAAGACCCTCCGGGCCGTCACACGCATACGAGGGCACGAAGTTCA

CACACGAAAGTAGCTTTAAATTACCCGACGCCACGAAAGCTAAGAAA

>142_Sample_ASV23

CACCGCGGTTATACGAGAAACCCTAGTTGACAAATACGGCGTAAAGAGTGGTTAAGGGGT

ATCAAAAATAAAGTCAAACATTTTCCTAGTTGTTATAAGCATTTCGAGAACATGAAGCCC

AACCACGAAAGTAACTTTACCTTCACCTGACCCCACGAAAGCTAAGAAA

>142_Sample_ASV24

CACCGCGGTTATACGAGAGACTCCAGTTTATAGACACGGCGTAAAGGGTGGTTATAGGAC

AAACTAACATAGAGCCAAAGACCCCCTAAGCCGTCATACGCTCACGAGGGCTCGAAGCCC

CTATACGAAAGTAGCTCTACCAAAAAATGCTCGACTCCACGAAAGCTAAGAAA

>142_Sample_ASV25

CACCGCGGTTATACGAAAGACCCCAGTTTATAGACACGGCGTAAAGGGTGGTTATAGGAC

AAACTAACATAGAGCCAAAGACCCCCTAAGCCGTCATACGCTCACGAGGGCTCGAAGCCC

CTATACGAAAGTAGCTCTACCAAAAAATGCTCGACTCCACGAAAGCTAAGAAA

>142_Sample_ASV26

CACCGCGGTTATACGAAAGACCCCAGTTTATAGACACGGCGTAAAGGGTGGTTATAGGAC

AAACTAACATAGAGCCAAAGACCCCCTAAGCCGTCATACGCTCACGAGGGCTCGAAGCCC

CTATACGAAAGTAGCTCTACCAAAAAATGCTCGACTCCACGAAAGCTGGGACA

>142_Sample_ASV27

CACCGCGGTTATACGAGAGACCCTAATTGATAGGTACGGCGTAAAGAGTGGTTTGGGATA

ACCCAAAAATAAAGCTAAAGACCTTCTAAGCCGTCACACGCATACCGAAGGCACGAGGCC

CCAACACGAAAGTAGCTTTACTATTACCCCGACGCCACGAAAGCTAAGAAA

>142_Sample_ASV28

CACCGCGGTTATACGAAAGACCCTAGTTGATGGATCACGGCGTAAAGGGTGGTTAAGGGA

TATAAAAATAAAGCTAAAAATCCTCTTAGCTGTCATACGCTTTCCGAAGACATGAGGCCC

AATTACGAAAGTAGCTTTAAACCACCCTGACACCACGAAAGCCAAGAAA

>142_Sample_ASV29

CACCGCGGTTATACGAGAGACCCTAGTTGATAGCTACGGCGTAAAGCGTGGTTAGGAGAA

CAAGATAAATAAAGTCAAACAATCTCTCGGCCGTTATACGTTATCTGAGAATATGAAGTC

CTACCACGAAAGTAACTTTAATTTTTCTGACCCCACGAAAACTGAGGAA

>142_Sample_ASV30

CACCGCGGTTATACGAGAGGCCCTAGTTGATAACTACCGGCGTAAAGAGTGGTTATGGAA

AAATATTTAATAAAGCCGAACACCCCCTCAGCCGTCATACGCACCTGGGAGCACGAAGAC

CTACTGCGAAAGCAGCTTTAATTATGCCTGACCCCACGACAGCTAAGAAA

>142_Sample_ASV31

CACCGCGGTTATACGAGAGGCCCAAGTTGATACTCCACGGCGTAAAGAGTGGTTAGAAAA

GACCTACTACTAAAGCCGAACGCCTTCAAAGCTGTTATACGCATCCGAAGGTGAGAAGCC

CACCCACGAAAGTGGCTTTACAACCTTGACTCCACGAAAGCTAAGAAA

>142_Sample_ASV32

CACCGCGGTTATACGAGAGGCCCAAGTTGATACTCCACGGCGTAAAGAGTGGTTAGAAAA

GACCTACTACTAAAGCCGAACGCCTTCAAAGCTGTTATACGCATCCGAAGGTGAGAAGCC

CACCCACGAAAGTGGCTTTACAACCTTGACTCCACGAAAGCTATGACA

>142_Sample_ASV33

CACCGCGGTTATACGAGAGACCCCAGTTGATAAACACGGCGTAAAGAGTGGTTAAGGGCA

CAAAACAAATAAAGTCAAACAACCTCTTAGCTGTTATACGCATTATGAGGATATGAAGCC

CCCCTACGAAAGTAACTTTACCAACCCCTGACCCCACGAAAGCTAAGAAA

>142_Sample_ASV34

CACCGCGGTTATACGAGAGACCCTAATTAATAGATACGGCGTAAAGAGTGGTTTAGGACA

AAAATTAATAAAGCCGAAGTTTCTCCTAGCCGTTGTACGCACTCTGAGAACACGAAGCCC

CACTACGAAAGTAGCTTTACCTATTATTTCCTGACCCCACGAAAGCTAAGAAA

>142_Sample_ASV35

CACCGCGGTTATACGAGAGGCCCAAGTTGATAGACATCGGCGTAAAGAGTGGTTAAGGAA

GTCTCAAAACTAAAGCCGAATGCCTTCAAGACTGTTATACGTTTCCGAAGGTAAGAAGCC

CCACTACGAAAGTGGCTTTACTCCACCTGACTCCACGAAAGCTGTGAAA

>142_Sample_ASV36

CACCGCGGTTATACGAGAGGCCCAAGTTGATAGACATCGGCGTAAAGAGTGGTTAAGGAA

GTCTCAAAACTAAAGCCGAATGCCTTCAAGACTGTTATACGTTTCCGAAGGTAAGAAGCC

CCACTACGAAAGTGGCTTTACTCCACCTGACTCCACGAAAGCTAAGAAA

>142_Sample_ASV37

CACCGCGGTTATACGAGAGACTCCAGTTGATAGCTACGGCGTAAAGAGTGGTTTGGGGCC

CGCCCAAAATAAAGCCAAAGACCTCCCAAGCCGTCAAACGCACCCCGGAGGCACGAAGTC

CTAACGCGAAAGCAACTTTACCTTCCCCGACGCCACGAAAGCTAAGAAA

>142_Sample_ASV38

CACCGCGGTTATACGAAAGACCCTAGTTGATAGATCACGGCGTAAAGGGTGGTTAAGGAA

AACAAAAATAAAGCTAAAAATCCTCTAAGCTGTCATACGCTTTCCGAAGACATGAGATCC

AACCACGAAAGTAGCTTTAAAATTTCCTGACACCACGAAAGCCAAGGAA

>142_Sample_ASV39

CACCGCGGTTATACGAAAGACCCTAGTTGATAGATCACGGCGTAAAGGGTGGTTAAGGAG

AACAAAAATAAAGCTAAAAATCCTCTAAGCTGTCATACGCTTTCCGAAGACATGAGACCC

AACCACGAAAGTAACTTTAAACTTTCCTGACACCACGAAAGCCAAGAAA

>145_Sample_ASV1

CACCGCGGTTATACGAAAGACCCTAGTTGATGGGTGCGGCGTAAAGGGTGGTTAGGGACT

ACAAAAAATAGAGCTAAAGATCCTCTAGGTCGTCATACACATCACGAGGAAACGAAGCCC

AAATACGAAAGTAGCTCTAAATATAATCCTGACGCCACGAAAGCCAAGAAA

>145_Sample_ASV2

CACCGCGGTTATACGAAAGACCCTAGTTGATGGGTGCGGCGTAAAGGGTGGTTAGGGACT

ACAAAAAATAGAGCTAAAGATCCTCTAGGTCGTCATACACATCACGAGAAAACGAAGCCC

AAATACGAAAGTAGCTCTAAATATAATCCTGACGCCACGAAAGCCAAGAAA

>145_Sample_ASV3

CACCGCGGTTAAACGAGAGGCCCTAGTTGATGGTGCAACGGCGTAAAGGGTGGTTAAGGA

TAGCGAAATAATAAAGTCAAATGGCCCTCTGGCTGTCATACGCTTCTAGGAGTCCGAAGC

CCAGTATACGAAAGTAGCTTTAAGAAAGTCCACCTGACCCCACGAAAACTGAGGAA

>145_Sample_ASV4

CACCGCGGTTATACGAAAGACCCTAGTTGCTAGCCACGGCGTAAAGGGTGGTTAAGGACA

ACAACAAATAAAGCTAAAGATCCCCTAAGCCGTCATACGCATTCCGGGGGCACGAAGCCC

TAACACGAAAGTAGCTTTAAAAAATATACCTGACCCCACGAAAGCTAAGAAA

>145_Sample_ASV5

CACCGCGGTTATACGAGAGACCCTAGTTGATTAAAGCGGCGTAAAGAGTGGTTATGGAAC

TATTCTTTTAAAGCAGAAAACCTCTCAAACTGTTATACGCACCCAGAGGTCTGAATCCCT

CACACGAAAGTGACTTTATTTATGCCTACCAGAACCCACGAAAGCTGGGACA

>145_Sample_ASV6

CACCGCGGTTATACGAGAGGCCCAAGTTGACAGACACCGGCGTAAAGAGTGGCTAGGGAA

AAATTACTACTAAAGCCGAACACCTTCAGAACTGTCATACGTTCCCGAAGATAAGAAGCC

CCACCACGAAAGTGGCTTTATATTACCCGACCCCACGAAAGCTGTGAAA

>145_Sample_ASV7

CACCGCGGTTATACGAGAGGCCCAAGTTGACAGACACCGGCGTAAAGAGTGGCTAGGGAA

AAATTACTACTAAAGCCGAACACCTTCAGAACTGTCATACGTTCCCGAAGATAAGAAGCC

CCACCACGAAAGTGGCTTTATATTACCCGACCCCACGAAAGCTAAGAAA

>145_Sample_ASV8

CATGGCGATTATACGAGAGGCCCAAGTTGACAGACACTGGCGTAAAGAGTGGCTAAGGAA

AAACTACTACTAAAGCCGAACACCTTCAGAACTGTCATACGTTCCCGAAGATAAGAAGCC

CCACCACGAAAGTGGCTTTATATTACCCGACCCCACGAAAGCTGTGAAA

>145_Sample_ASV9

CATGGCGATTATACGAGAGGCCCAAGTTGACAGACACTGGCGTAAAGAGTGGCTAAGGAA

AAATTACTACTAAAGCCGAACACCTTCAGAACTGTCATACGTTCCCGAAGATAAGAAGCC

CCACCACGAAAGTGGCTTTATATTACCCGACCCCACGAAAGCTGTGAAA

>145_Sample_ASV10

CACCGCGATTATACGAGAGGCCCAAGTTGACAGACATCGGCGTAAAGAGTGGTTAAGGAA

AAATTACTGCTAAAGCCGAACACCTTCAGAACTGTCATACGTTCCCGAAGATAAGAAGCC

CCACCACGAAAGTGGCTTTATATTACCCGACCCCACGAAAGCTGTGAAA

>145_Sample_ASV11

CACCGCGATTATACGAGAGGCCCAAGTTGACAGACATCGGCGTAAAGAGTGGTTAAGGAA

AAATTACTACTAAAGCCGAACACCTTCAGAACTGTCATACGTTCCCGAAGATAAGAAGCC

CCACCACGAAAGTGGCTTTATATTACCCGACCCCACGAAAGCTGTGAAA

>145_Sample_ASV12

CACCGCGGTTAAACGAGAGACCCTAGTTGATCATCACGGCGTAAAGAGTGGTTAGGGGAT

TATCATAAATAAAGCCAAAGACCTCCCAAGCTGTCGCACGCATTCCGGAGGCGCGAAGCC

CACACACGAAAGTAGCTTTAACTATTGACCCTGATGCCACGAAAGCTAAGAAA

>145_Sample_ASV13

CGCCGCGGTTAAACGAGAGGCCCTAGTTGATGGAACAACGGCGTAAAGGGTGGTTAAGGA

TAGTGAGACAATAAAGTCGAATGGCCCTTTGGCTGTCATACGCTTCTGGGAGCCTGAAGC

CCACTATACGAAAGTAGCTTTAACAATGTCCACCTGACCCCACGAAAACTGAGAAA

>145_Sample_ASV14

CGCCGCGGTTAAACGAGAGGCCCTAGTTGATGGAACAACGGCGTAAAGGGTGGTTAAGGA

TAGTGAGACAATAAAGTCGAATGGCCCTTTGGCTGTCATACGCTTCTGGGAGCCTGAAGC

CCACTATACGAAAGTAGCTTTAACAACGTCCACCTGACCCCACGAAAACTGAGAAA

>145_Sample_ASV15

CACCGCGGTTATACGAAAGACCCTAGTTGATAGATCACGGCGTAAAGGGTGGTTAGGGAA

TACAAAAATAAAGCTAAAAATCCTCTAAGCTGTCATACGCTTCCCGAAGACATGAAACCC

AACCACGAAAGTAGCTTTAAACTCTCCTGACACCACGAAAGCCAAGGAA

>145_Sample_ASV16

CACCGCGGTTATACGAGAGGCCCAAGTTGATAAGCCCGGCGTAAAGAGTGGTTATGGAGA

ATAAAACACTAAAGCTAAAGACCCCCTAGGCTGTTATACGCACCTGGCGGCTCGAACCAC

CTATACGAAAGTAGCTTTACCCCCCTTCCACCAGAATCCACGACAGCTGGGGCA

>145_Sample_ASV17

CACCGCGGTTATACGAGAGGCCCAAGTTGACAGACATCGGCGTAAAGAGTGGTTAAGGAA

ACCCCATTACTAAAGCCGAACGCCCTCAGAACTGTCATACGTTCCCGAAGGTAAGAAGCC

CCACTACGAAAGTGGCTTTAACCACACCCGACCCCACGAAAGCTAAGAAA

>145_Sample_ASV18

CACCGCGGTTATACGAGAGGCCCAAGTTGACAGACATCGGCGTAAAGAGTGGTTAAGGAA

ACCCCATTACTAAAGCCGAACGCCCTCAGAACTGTCATACGTTCCCGAAGGTAAGAAGCC

CCACTACGAAAGTGGCTTTAACCACACCCGACCCCACGAAAGCTGGGACA

>145_Sample_ASV19

CACCGCGGTTATACGAGAGGCCCAAGTTGACAGACATCGGCGTAAAGAGTGGTTAAGGAA

ACCCCATTACTAAAGCCGAACGCCCTCAGAACTGTCATACGTTCCCGAAGGTAAGAAGCC

CCACTACGAAAGTGGCTTTAACCACACCCGACCCCACGAAAGCTGTGAAA

>145_Sample_ASV20

CACCGCGGTTAAACGAGAGGCCCTAGTTGATATTACAACGGCGTAAAGGGTGGTTAAGGA

AAGCAAGCAATAAAGCCGAATGGCCCTTTGGCTGTCATACGCTTCTAGGTGTCCGAAGCC

CAATATACGAAAGTAGCTTTAGAAAAGCCCACCTGACACCACGAAAGCTGAGGAA

>145_Sample_ASV21

CACCGCGGTTATACGAGAGGCCCTAGTTGATAAATACGGCGTAAAGAGTGGTTAGAGGAC

TCCCATAATAAAGCCAAAGACCCTCCGGGCCGTCACACGCATACGAGGGCACGAAGTTCA

CACACGAAAGTAGCTTTAAATTACCCGACGCCACGAAAGCTAAGAAA

>145_Sample_ASV22

CACCGCGGTTATACGAAAGACCCTAGTTTATAAGTACGGCGTAAAGGGTGGTTAGGGGAC

AAACAAAATAAAGCCAAAGACCTCCTAAGCCGTCATACGCTTACGGAGGCACGAAGCCCA

AACACGAAAGTAGCTTTACCGAACATGCCCGACTCCACGAAAGCTGGGAAA

>145_Sample_ASV23

CACCGCGGTTATACGAGAGACCCTAGTTGATAAATACGGCGTAAAGAGTGGTTATGGGAA

AAACAAATAAAGTCAAACAACCTCTTAGCTGTTATACGCATTATGAGAGCATGAAGCCCC

CTCACGAAAGTAACTTTAATACCTCCTGACCCCACGAAAGCTGTGAAA

>145_Sample_ASV24

CACCGCGGTTATACGAGAGACCCTAGTTGATAAATACGGCGTAAAGAGTGGTTATGGGAA

AAACAAATAAAGTCAAACAACCTCTTAGCTGTTATACGCATTATGAGAGCATGAAGCCCC

CTCACGAAAGTAACTTTAATACCTCCTGACCCCACGAAAGCTAAGAAA

>145_Sample_ASV25

CACCGCGGTTATACGAGAGGCTCAAGTTGATAAACCCCGGCGTAAAGAGTGGTTAAGGAA

AAATAGAAACTAAAGCCGAATGCTTTCAAGACTGTTATACGTTCCCGAGAGTAAGAAGAC

CAATTACGAAAGTGGCTTTACTTTACCTGACCCCACGAAAGCTACGACA

>145_Sample_ASV26

AACCGCGGTTATACGAGAGACCCTAATTGATAGCTCACGGCGTAAAGGGTGGTTTGGGGA

AACCTCAAAATAAAGCTAAAGACCTTCTAAGCCGTTACACGCATACCGAAGGCACGAGGC

CCTAACACGAAAGTAGCTTTACTATTACCCCTGACGCCACGAAAGCTAAGAAA

>145_Sample_ASV27

CACCGCGGTTATACGAAAGACCCTAGTTGATGGATCACGGCGTAAAGGGTGGTTAAGGGA

TATAAAAATAAAGCTAAAAATCCTCTTAGCTGTCATACGCTTTCCGAAGACATGAGGCCC

AATTACGAAAGTAGCTTTAAACCACCCTGACACCACGAAAGCCAAGAAA

>145_Sample_ASV28

CACCGCGGTTAAACGAGAGGCCCTAGTTGATAGTACAACGGCGTAAAGGGTGGTTAAGGA

TAGTGAAATAATAAAGTCGAATGGCCCTTTGGCTGTCATACGCTTCTAGGAGTCCGAAGC

CCAATATACGAAAGTAACTTTAAGAGCGCCCACCTGACCCCACGAAAACTGAGAAA

>145_Sample_ASV29

CACCGCGGTTATACGAGAGGCCCAAGTTGATACTCCACGGCGTAAAGAGTGGTTAGAAAA

GACCTACTACTAAAGCCGAACGCCTTCAAAGCTGTTATACGCATCCGAAGGTGAGAAGCC

CACCCACGAAAGTGGCTTTACAACCTTGACTCCACGAAAGCTATGACA

>145_Sample_ASV30

CACCGCGGTTATACGAGAGACCCCAGTTGATAAACACGGCGTAAAGAGTGGTTAAGGGCA

CAAAACAAATAAAGTCAAACAACCTCTTAGCTGTTATACGCATTATGAGGATATGAAGCC

CCCCTACGAAAGTAACTTTACCAACCCCTGACCCCACGAAAGCTAAGAAA

>145_Sample_ASV31

CACCGCGGTTATACGAGAGACCCTAGTTGATATATACGGCGTAAAGAGTGGTTTGGAATA

ACCAAGTAATATAGCCAAAGACCTCCCAAGCCGTCACACGCACCCCGGAGGCACGAAGCC

CAAACACGAAAGTAGCTTTATTAACTTACCGACGCCACGAAAGCTAAGAAA

>145_Sample_ASV32

CACCGCGGTTATACGAAAGACCCTAGTTTATAGGCACGGCGTAAAGGGTGGTTAGGGGAT

AAACAAAATAAAGCCAAAGACCTTCTAAGTCGTCATACACTCATGAAGGCACGAAGCCCA

AATACGAAAGTAACTTTACAACTTAGACCCGACTCCACGAAAGCTGGGGGA

>145_Sample_ASV33

CACCGCGGTTATACGAGAGGCCCAAGTTGATAGACATCGGCGTAAAGAGTGGTTAAGGAA

GTCTCAAAACTAAAGCCGAATGCCTTCAAGACTGTTATACGTTTCCGAAGGTAAGAAGCC

CCACTACGAAAGTGGCTTTACTCCACCTGACTCCACGAAAGCTGTGAAA

>145_Sample_ASV34

CACCGCGGTTATACGAGAGGCCCAAGTTGACAAACTCCGGCGTAAAGCGTGGTTAAGGCA

CACTCAAAAACTAAAGCCGAATATCTTCAGAGCAGTCATACGCATCCGAAGACACGAAGC

CCCACCACGAAAGTGGCTTTATGACACCTGACCCCACGAAAGCTAGGACA

>145_Sample_ASV35

CACCGCGGTTATACGAAAGACCCTAGTTGATAGCTACGGCGTAAAGGGTGGTTAAGGTAT

ATAACAAATAAAGCTAAAGAGCCTCCAAGCCGTCGTACGCATTCTGAGAGCTCGAAACCC

TAACACGAAAGTAGCTTTAAAAAATAACACCTGACCCCACGAAAGCTAAGAAA

>145_Sample_ASV36

CACCGCGGTTATACGAAAGACCCTAGTTGATAGACCACGGCGTAAAGGGTGGTTAAGGAA

CACAAAAATAAAGCTAAAGATCCTCTAAGCTGTCATACGCTTCCCGAAGACATGAAACCC

AATCACGAAAGTAGCTTTAAACTGTCCTGACACCACGAAAGCCAAGAAA

>146_Sample_ASV1

CACCGCGGTTATACGAAAGACCCTAGTTGATGGGTGCGGCGTAAAGGGTGGTTAGGGACT

ACAAAAAATAGAGCTAAAGATCCTCTAGGTCGTCATACACATCACGAGGAAACGAAGCCC

AAATACGAAAGTAGCTCTAAATATAATCCTGACGCCACGAAAGCCAAGAAA

>146_Sample_ASV2

CACCGCGGTTATACGAAAGACCCTAGTTGATGGGTGCGGCGTAAAGGGTGGTTAGGGACT

ACAAAAAATAGAGCTAAAGATCCTCTAGGTCGTCATACACATCACGAGAAAACGAAGCCC

AAATACGAAAGTAGCTCTAAATATAATCCTGACGCCACGAAAGCCAAGAAA

>146_Sample_ASV3

CACCGCGGTTAAACGAGAGGCCCTAGTTGATGGTGCAACGGCGTAAAGGGTGGTTAAGGA

TAGCGAAATAATAAAGTCAAATGGCCCTCTGGCTGTCATACGCTTCTAGGAGTCCGAAGC

CCAGTATACGAAAGTAGCTTTAAGAAAGTCCACCTGACCCCACGAAAACTGAGGAA

>146_Sample_ASV4

CACCGCGGTTATACGAAAGACCCTAGTTGCTAGCCACGGCGTAAAGGGTGGTTAAGGACA

ACAACAAATAAAGCTAAAGATCCCCTAAGCCGTCATACGCATTCCGGGGGCACGAAGCCC

TAACACGAAAGTAGCTTTAAAAAATATACCTGACCCCACGAAAGCTAAGAAA

>146_Sample_ASV5

CACCGCGGTTATACGAGAGACCCTAGTTGATTAAAGCGGCGTAAAGAGTGGTTATGGAAC

TATTCTTTTAAAGCAGAAAACCTCTCAAACTGTTATACGCACCCAGAGGTCTGAATCCCT

CACACGAAAGTGACTTTATTTATGCCTACCAGAACCCACGAAAGCTGGGACA

>146_Sample_ASV6

CACCGCGGTTATACGAAAGACCCTAGTTTATAGGTACGGCGTAAAGGGTGGTTAGGGGAC

AAACAAAATAAAGCCAAAGACCTCCTAAGCCGTCATACGCTCACGGAAGCACGAAGCCCA

AACACGAAAGTAGCTTTACCGAACATGCCCGACTCCACGAAAGCTGGGGAA

>146_Sample_ASV7

CACCGCGGTTATACGAGAGGCCCAAGTTGACAGACACCGGCGTAAAGAGTGGCTAGGGAA

AAATTACTACTAAAGCCGAACACCTTCAGAACTGTCATACGTTCCCGAAGATAAGAAGCC

CCACCACGAAAGTGGCTTTATATTACCCGACCCCACGAAAGCTAAGAAA

>146_Sample_ASV8

CACCGCGGTTATACGAGAGGCCCAAGTTGACAGACACCGGCGTAAAGAGTGGCTAGGGAA

AAATTACTACTAAAGCCGAACACCTTCAGAACTGTCATACGTTCCCGAAGATAAGAAGCC

CCACCACGAAAGTGGCTTTATATTACCCGACCCCACGAAAGCTGTGAAA

>146_Sample_ASV9

CACCGCGGTTATACGAGAGGCTCAAGTTGACAGACATCGGCGTAAAGAGTGGTTAGGAAG

TTTTTGAAACTAAAGCCGAACGCCCTCAGAACTGTTATACGTACCCGAGGGTAAGAAGCC

CCACTACGAAAGTGGCTTTATATCTCCGACCCCACGAAAGCTGCGAAA

>146_Sample_ASV10

CACCGCGGTTAAACGAGAGACCCTAGTTGATCATCACGGCGTAAAGAGTGGTTAGGGGAT

TATCATAAATAAAGCCAAAGACCTCCCAAGCTGTCGCACGCATTCCGGAGGCGCGAAGCC

CACACACGAAAGTAGCTTTAACTATTGACCCTGATGCCACGAAAGCTAAGAAA

>146_Sample_ASV11

CACCGCGGTTATACGAGAGGCCCAAGTTGATAAGCCCGGCGTAAAGAGTGGTTATGGAGA

ATAAAACACTAAAGCTAAAGACCCCCTAGGCTGTTATACGCACCTGGCGGCTCGAACCAC

CTATACGAAAGTAGCTTTACCCCCCTTCCACCAGAATCCACGACAGCTGGGGCA

>146_Sample_ASV12

CACCGCGGTTATACGAGAGGCCCTAGTTGATAGCCACGGCGCAAAGAGTGGTTAAGGAGC

CCCACTAAATAAAGTCGAACACTTCCTAGGCCGTTATACGTTTTCTAGAAGCACGAAACC

CAATTACACGAAAGCAACTTTATACTAAACAACCTGACCCCACGAAAGCTAAGAAA

>146_Sample_ASV13

CACCGCGGTTATACGAGAGGCCCAAGTTGACAGACATCGGCGTAAAGAGTGGTTAAGGAA

ACCCCATTACTAAAGCCGAACGCCCTCAGAACTGTCATACGTTCCCGAAGGTAAGAAGCC

CCACTACGAAAGTGGCTTTAACCACACCCGACCCCACGAAAGCTAAGAAA

>146_Sample_ASV14

CACCGCGGTTATACGAGAGGCCCAAGTTGACAGACATCGGCGTAAAGAGTGGTTAAGGAA

ACCCCATTACTAAAGCCGAACGCCCTCAGAACTGTCATACGTTCCCGAAGGTAAGAAGCC

CCACTACGAAAGTGGCTTTAACCACACCCGACCCCACGAAAGCTGTGAAA

>146_Sample_ASV15

CACCGCGGTTATACGAGAGGCCCAAGTTGATAGATATCGGCGTAAAGAGTGGTTAAGGAA

ACCCCGTTACTAAAGCCGAACGCCCTCAGAACTGTCATACGTTCCCGAAGGTAAGAAGCC

CCACTACGAAAGTGGCTTTAACCACACCCGACCCCACGAAAGCTGTGAAA

>146_Sample_ASV16

CACCGCGGTCACACGATTAACCCAAGTCAATAGAAGCCGGCGTAAAGAGTGTTTTAGATC

ACCCCCTCCCCAATAAAGCTAAAACTCACCTGAGTTGTAAAAAACTCCAGTTGACACAAA

ATAGACTACGAAAGTGGCTTTAACATATCTGAACACACAATAGCTAAGACC

>146_Sample_ASV17

CACCGCGGTTATACGAGAAACCCTAGTTGACAAATACGGCGTAAAGAGTGGTTAAGGGGT

ATCAAAAATAAAGTCAAACATTTTCCTAGTTGTTATAAGCATTTCGAGAACATGAAGCCC

AACCACGAAAGTAACTTTACCTTCACCTGACCCCACGAAAGCTAAGAAA

>146_Sample_ASV18

CACCGCGGTTATACGAAAGACCCCAGTTTATAGACACGGCGTAAAGGGTGGTTATAGGAC

AAACTAACATAGAGCCAAAGACCCCCTAAGCCGTCATACGCTCACGAGGGCTCGAAGCCC

CTATACGAAAGTAGCTCTACCAAAAAATGCTCGACTCCACGAAAGCTGGGACA

>146_Sample_ASV19

CACCGCAGTTATACGAAAGACCCCAGTTTATAGACACGGCGTAAAGGGTGGTTATAGGAC

AAACTAACATAGAGCCAAAGACCCCCTAAGCCGTCATACGCTCACGAGGGCTCGAAGCCC

CTATACGAAAGTAGCTCTACCAAAAAATGCTCGACTCCACGAAAGCTGGGACA

>146_Sample_ASV20

CACCGCGGTTATACGAGAGACCCTAGTTGATAAATACGGCGTAAAGAGTGGTTATGGGAA

AAACAAATAAAGTCAAACAACCTCTTAGCTGTTATACGCATTATGAGAGCATGAAGCCCC

CTCACGAAAGTAACTTTAATACTTCCTGACCCCACGAAAGCTAAGAAA

>146_Sample_ASV21

CACCGCGGTTATACGAGAGACCCTAGTTGATAAATACGGCGTAAAGAGTGGTTATGGGAA

AAACAAATAAAGTCAAACAACCTCTTAGCTGTTATACGCATTATGAGAGCATGAAGCCCC

CTCACGAAAGTAACTTTAATACCTCCTGACCCCACGAAAGCTAAGAAA

>146_Sample_ASV22

CACCGCGGTTATACGAAAGACCCTAGTTGATGGATCACGGCGTAAAGGGTGGTTAAGGGA

TATAAAAATAAAGCTAAAAATCCTCTTAGCTGTCATACGCTTTCCGAAGACATGAGGCCC

AATTACGAAAGTAGCTTTAAACCACCCTGACACCACGAAAGCCAAGAAA

>146_Sample_ASV23

CACCGCGGTTATACGAGAGGCCCTAGTTGATAACTACCGGCGTAAAGAGTGGTTATGGAA

AAATATTTAATAAAGCCGAACACCCCCTCAGCCGTCATACGCACCTGGGAGCACGAAGAC

CTACTGCGAAAGCAGCTTTAATTATGCCTGACCCCACGACAGCTAAGAAA

>146_Sample_ASV24

CACCGCGGTTATACGAGAGGCCCAAGTTGATTATTACCGGCGTAAAGCGTGGTTAAGATT

TTTATAGGACTAAAGCCGAACCCCCTCAGAGCTGTTATACGCACCCGAAGGTTAGAAGTT

CAATCACGAAAGTGGCTTTACAACCCCGAACCCACGAAAGCTAAGATA

>146_Sample_ASV25

CACCGCGGTTATACGAGAGACCCTAATTGATAGGCGCGGCGTAAAGAGTGGTTTAGGACT

AAAACTAATAAAGCTAAAGATCCTCCCAGCCGTCGCACGCATTCTGAGGACACGAAACTC

AACCACGAAAGTAGCTTTAACATACTCTCCTGACCCCACGAAAGCTAAGAAA

>146_Sample_ASV26

CACCGCGGTTATACGAAAGACCCTAGTTGATAGGCACGGCGTAAAGGGTGGTTAGGGATA

TATCAAAAATAAAGTTAAAGAGCCTCTAAGCTGTCGCACGCATCCCGAGAGCTCGAGACC

CAGACACGAAAGTAACTTTAAAATACCTCACCTGACCCCACGAAAGCTAAGAAA

>146_Sample_ASV27

CACCGCGGTTATACGAGAGGCCCAAGTTGATACTCCACGGCGTAAAGAGTGGTTAGAAAA

GACCTACTACTAAAGCCGAACGCCTTCAAAGCTGTTATACGCATCCGAAGGTGAGAAGCC

CACCCACGAAAGTGGCTTTACAACCTTGACTCCACGAAAGCTATGACA

>146_Sample_ASV28

CACCGCGGTTATACGAGAGACCCCAGTTGATAAACACGGCGTAAAGAGTGGTTAAGGGCA

CAAAACAAATAAAGTCAAACAACCTCTTAGCTGTTATACGCATTATGAGGATATGAAGCC

CCCCTACGAAAGTAACTTTACCAACCCCTGACCCCACGAAAGCTAAGAAA

>146_Sample_ASV29

CACCGCGGTTATACGAAAGACCCTAGTTTATAGGCACGGCGTAAAGGGTGGTTAGGGGAT

AAACAAAATAAAGCCAAAGACCTTCTAAGTCGTCATACACTCATGAAGGCACGAAGCCCA

AATACGAAAGTAACTTTACAACTTAGACCCGACTCCACGAAAGCTGGGGGA

>146_Sample_ASV30

CACCGCGGTTATACGAGAGACCCTAATTAATAGCTACGGCGTAAAGAGTGGTTTAGGGTA

AAAATTAATAAAGCCGAAGTTTCTCCTAGCCGTTGTACGCACTCTGAGAACACGAAGCCC

CACTACGAAAGTAGCTTTACCTATTATTTCCTGACCCCACGAAAGCTAAGAAA

>146_Sample_ASV31

CACCGCGGTTATACGAGAGACCCTAATTAATAGCTACGGCGTAAAGAGTGGTTTAGGACA

AAAATTAATAAAGCCGAAGTTTCTCCTAGCCGTTGTACGCACTCTGAGAACACGAAGCCC

CACTACGAAAGTAGCTTTACCTATTATTTCCTGACCCCACGAAAGCTAAGAAA

>146_Sample_ASV32

CACCGCGGTTATACGAGAGACCCTAATTAATAGATACGGCGTAAAGAGTGGTTTAGGACA

AAAATTAATAAAGCCGAAGTTTCTCCTAGCCGTTGTACGCACTCTGAGAACACGAAGCCC

CACTACGAAAGTAGCTTTACCTATTATTTCCTGACCCCACGAAAGCTAAGAAA

>146_Sample_ASV33

CACCGCGGTTATACGAAAGACCCTAGTTGATAGATCACGGCGTAAAGGGTGGTTAAGGAA

AACAAAAATAAAGCTAAAAATCCTCTAAGCTGTCATACGCTTTCCGAAGACATGAGATCC

AACCACGAAAGTAGCTTTAAAATTTCCTGACACCACGAAAGCCAAGGAA

>148_Sample_ASV1

CACCGCGGTTATACGAAAGACCCTAGTTGATGGGTGCGGCGTAAAGGGTGGTTAGGGACT

ACAAAAAATAGAGCTAAAGATCCTCTAGGTCGTCATACACATCACGAGGAAACGAAGCCC

AAATACGAAAGTAGCTCTAAATATAATCCTGACGCCACGAAAGCCAAGAAA

>148_Sample_ASV2

CACCGCGGTTATACGAAAGACCCTAGTTGATGGGTGCGGCGTAAAGGGTGGTTAGGGACT

ACAAAAAATAGAGCTAAAGATCCTCTAGGTCGTCATACACATCACGAGAAAACGAAGCCC

AAATACGAAAGTAGCTCTAAATATAATCCTGACGCCACGAAAGCCAAGAAA

>148_Sample_ASV3

CACCGCGGTTATACGAGAGACCCTAGTTGATTAAAGCGGCGTAAAGAGTGGTTATGGAAC

TATTCTTTTAAAGCAGAAAACCTCTCAAACTGTTATACGCACCCAGAGGTCTGAATCCCT

CACACGAAAGTGACTTTATTTATGCCTACCAGAACCCACGAAAGCTGGGACA

>148_Sample_ASV4

CACCGCGGTTATACGAGAGACCCTAGTTGATATACACGGCGTAAAGAGTGGTTTGGGGTA

TATAATAAATAAAGCCAAAGACCTTCTAGGCCGTTGAACGCACCACGAAGGAGCGAAGTC

CCAATACGAAAGTAGCTTTATAAAACCTGACGCCACGAAAGCTAAGAAA

>148_Sample_ASV5

CACCGCGGTTATACGAGAGGCCCAAGTTGACAGACACCGGCGTAAAGAGTGGCTAGGGAA

AAATTACTACTAAAGCCGAACACCTTCAGAACTGTCATACGTTCCCGAAGATAAGAAGCC

CCACCACGAAAGTGGCTTTACAATGCCTGACCCCACGAAAGCTGTGAAA

>148_Sample_ASV6

CACCGCGGTTATACGAGAGACCCCAGTTGACAGACACCGGCGTAAAGAGTGGCTAGGGAA

AAATTACTACTAAAGCCGAACACCTTCAGAACTGTCATACGTTCCCGAAGATAAGAAGCC

CCACCACGAAAGTGGCTTTATATTACCCGACCCCACGAAAGCTAAGAAA

>148_Sample_ASV7

CACCGCGGTTATACGAGAGGCCCAAGTTGACAGACACCGGCGTAAAGAGTGGCTAGGGAA

AAATTACTACTAAAGCCGAACACCTTCAGAACTGTCATACGTTCCCGAAGATAAGAAGCC

CCACCACGAAAGTGGCTTTATATTACCCGACCCCACGAAAGCTAAGAAA

>148_Sample_ASV8

CACCGCGGTTATACGAGAGGCCCAAGTTGACAGACACCGGCGTAAAGAGTGGCTAGGGAA

AAATTACTACTAAAGCCGAACACCTTCAGAACTGTCATACGTTCCCGAAGATAAGAAGCC

CCACCACGAAAGTGGCTTTATATTACCCGACCCCACGAAAGCTGTGAAA

>148_Sample_ASV9

CACCGCGGTTATACGAGAGGCCCAAGTTGATAAGCCCGGCGTAAAGAGTGGTTATGGAGA

ATAAAACACTAAAGCTAAAGACCCCCTAGGCTGTTATACGCACCTGGCGGCTCGAACCAC

CTATACGAAAGTAGCTTTACCCCCCTTCCACCAGAATCCACGACAGCTGGGGCA

>148_Sample_ASV10

CACCGCGGTTATACGAGAGGCCCAAGTTGACAGACATCGGCGTAAAGAGTGGTTAAGGAA

ACCCCATTACTAAAGCCGAACGCCCTCAGAACTGTCATACGTTCCCGAAGGTAAGAAGCC

CCACTACGAAAGTGGCTTTAACCACACCCGACCCCACGAAAGCTGGGACA

>148_Sample_ASV11

CACCGCGGTTATACGAGAGGCCCAAGTTGACAGACATCGGCGTAAAGAGTGGTTAAGGAA

ACCCCATTACTAAAGCCGAACGCCCTCAGAACTGTCATACGTTCCCGAAGGTAAGAAGCC

CCACTACGAAAGTGGCTTTAACCACACCCGACCCCACGAAAGCTGTGAAA

>148_Sample_ASV12

CACCGCGGTTATACGAGAGGCCCAAGTTGATAGATATCGGCGTAAAGAGTGGTTAAGGAA

ACCCCGTTACTAAAGCCGAACGCCCTCAGAACTGTCATACGTTCCCGAAGGTAAGAAGCC

CCACTACGAAAGTGGCTTTAACCACACCCGACCCCACGAAAGCTGTGAAA

>148_Sample_ASV13

CACCGCGGTTATACGAGAAACCCTAGTTGACAAATACGGCGTAAAGAGTGGTTAAGGGGT

ATCAAAAATAAAGTCAAACATTTTCCTAGTTGTTATAAGCATTTCGAGAACATGAAGCCC

AACCACGAAAGTAACTTTACCTTCACCTGACCCCACGAAAGCTAAGAAA

>148_Sample_ASV14

CACCGCGGTTATACGAGAGACCCTAGTTGATAAATACGGCGTAAAGAGTGGTTATGGGAA

AAACAAATAAAGTCAAACAACCTCTTAGCTGTTATACGCATTATGAGAGCATGAAGCCCC

CTCACGAAAGTAACTTTAATACCTCCTGACCCCACGAAAGCTAAGAAA

>148_Sample_ASV15

CACCGCGGTTATACGAGAGGCTCAAGTTGATAAACCCCGGCGTAAAGAGTGGTTAAGGAA

AAATAGAAACTAAAGCCGAATGCTTTCAAGACTGTTATACGTTCCCGAGAGTAAGAAGAC

CAATTACGAAAGTGGCTTTACTTTACCTGACCCCACGAAAGCTACGACA

>148_Sample_ASV16

AACCGCGGTTATACGAGAGACCCTAATTGATAGCTCACGGCGTAAAGGGTGGTTTGGGGA

AACCTCAAAATAAAGCTAAAGACCTTCTAAGCCGTTACACGCATACCGAAGGCACGAGGC

CCTAACACGAAAGTAGCTTTACTATTACCCCTGACGCCACGAAAGCTAAGAAA

>148_Sample_ASV17

CACCGCGGTTATACGAAAGACCCTAGTTGATGGATCACGGCGTAAAGGGTGGTTAAGGGA

TATAAAAATAAAGCTAAAAATCCTCTTAGCTGTCATACGCTTTCCGAAGACATGAGGCCC

AATTACGAAAGTAGCTTTAAACCACCCTGACACCACGAAAGCCAAGAAA

>148_Sample_ASV18

CACCGCGGTTATACGAGAGGCCCAAGTTGATACTCCACGGCGTAAAGAGTGGTTAGAAAA

GACCTACTACTAAAGCCGAACGCCTTCAAAGCTGTTATACGCATCCGAAGGTGAGAAGCC

CACCCACGAAAGTGGCTTTACAACCTTGACTCCACGAAAGCTATGACA

>148_Sample_ASV19

CACCGCGGTTATACGAGAGACCCCAGTTGATAAACACGGCGTAAAGAGTGGTTAAGGGCA

CAAAACAAATAAAGTCAAACAACCTCTTAGCTGTTATACGCATTATGAGGATATGAAGCC

CCCCTACGAAAGTAACTTTACCAACCCCTGACCCCACGAAAGCTAAGAAA

>148_Sample_ASV20

CACCGCGGTTATACGAAAGACCCTAGTTTATAGGCACGGCGTAAAGGGTGGTTAGGGGAT

AAACAAAATAAAGCCAAAGACCTTCTAAGTCGTCATACACTCATGAAGGCACGAAGCCCA

AATACGAAAGTAACTTTACAACTTAGACCCGACTCCACGAAAGCTGGGGGA

>148_Sample_ASV21

CACCGCGGTTATACGAGAGACCCTAATTAATAGATACGGCGTAAAGAGTGGTTTAGGACA

AAAATTAATAAAGCCGAAGTTTCTCCTAGCCGTTGTACGCACTCTGAGAACACGAAGCCC

CACTACGAAAGTAGCTTTACCTATTATTTCCTGACCCCACGAAAGCTAAGAAA

>148_Sample_ASV22

CACCGCGGTTATACGAGAGGCCCAAGTTGACAGATATCGGCGTAAAGAGTGGTTAAGGAA

GTCTCAAAACTAAAGCCGAATGCCTTCAAGACTGTTATACGTTTCCGAAGGTAAGAAGCC

CCACTACGAAAGTGGCTTTACTCCACCTGACTCCACGAAAGCTGTGAAA

>148_Sample_ASV23

CACCGCGGTTATACGAGAGGCCCAAGTTGATAGACATCGGCGTAAAGAGTGGTTAAGGAA

GTCTCAAAACTAAAGCCGAATGCCTTCAAGACTGTTATACGTTTCCGAAGGTAAGAAGCC

CCACTACGAAAGTGGCTTTACTCCACCTGACTCCACGAAAGCTGTGAAA

>148_Sample_ASV24

CACCGCGGTTATACGAGAGGCCCAAGTTGACAAACTCCGGCGTAAAGCGTGGTTAAGGCA

CACTCAAAAACTAAAGCCGAATATCTTCAGAGCAGTCATACGCATCCGAAGACACGAAGC

CCCACCACGAAAGTGGCTTTATGACACCTGACCCCACGAAAGCTAGGACA

>148_Sample_ASV25

CACCGCGGTTATACGAAAGACCCTAGTTGATAGCTACGGCGTAAAGGGTGGTTAAGGTAT

ATAACAAATAAAGCTAAAGAGCCTCCAAGCCGTCGTACGCATTCTGAGAGCTCGAAACCC

TAACACGAAAGTAGCTTTAAAAAATAACACCTGACCCCACGAAAGCTAAGAAA

>152_Sample_ASV1

CACCGCGGTTATACGAGAGGCCCAAGTTGACAGACATCGGCGTAAAGAGTGGTTAAGGAA

AACCTTCAACTAAAGTCGAACGCTCTCAAGACTGTCGTACGTTTCCGAGAGTATGAAGCC

CCACCACGAAAGTGACTTTACCTCACCTGACTCCACGAAAGCTGCGAAA

>152_Sample_ASV2

CACCGCGGTTATACGAAAGACCCTAGTTGATGGGTGCGGCGTAAAGGGTGGTTAGGGACT

ACAAAAAATAGAGCTAAAGATCCTCTAGGTCGTCATACACATCACGAGGAAACGAAGCCC

AAGTACGAAAGTAGCTCTAAATATAATCCTGACGCCACGAAAGCCAAGAAA

>152_Sample_ASV3

CACCGCGGTTATACGAAAGACCCTAGTTGATGGGTGCGGCGTAAAGGGTGGTTAGGGACT

ACAAAAAATAGAGCTAAAGATCCTCTAGGTCGTCATACACATCACGAGGAAACGAAGCCC

AAATACGAAAGTAGCTCTAAATATAATCCTGACGCCACGAAAGCCAAGAAA

>152_Sample_ASV4

CACCGCGGTTATACGAGAGACCCTAGTTGATTAAAGCGGCGTAAAGAGTGGTTATGGAAC

TATTCTTTTAAAGCAGAAAACCTCTCAAACTGTTATACGCACCCAGAGGTCTGAATCCCT

CACACGAAAGTGACTTTATTTATGCCTACCAGAACCCACGAAAGCTGGGACA

>152_Sample_ASV5

CACCGCGGTTATACGAGAGACCCTAGTTGATTAAAGCGGCGTAAAGAGTGGTTATGGAAC

TATTCTTTTAAAGCAGAAAACCTCTCAAACTGTTATACGCACCCAGAGGTCTGAATCCCT

CACACGAAAGTGACTTTATTTATGCCTACCAGAACCCACGAAAGCTATGACA

>152_Sample_ASV6

CACCGCGGTTATACGAGAGACCCTAGTTGATTAAAGCGGCGTAAAGAGTGGTTATGGAAC

TATTCTTTTAAAGCAGAAAACCTCTCAAACTGTTATACGCACCCAGAGGTCTGAATCCCT

CACACGAAAGTGACTTTATTTATGCCTACCAGAACCCACGAAAGCTGGGGAA

>152_Sample_ASV7

CACCGCGGTTATACGAAAGACCCTAGTTTATAGGTACGGCGTAAAGGGTGGTTAGGGGAC

AAACAAAATAAAGCCAAAGACCTCCTAAGCCGTCATACGCTCACGGAAGCACGAAGCCCA

AACACGAAAGTAGCTTTACCGAACATGCCCGACTCCACGAAAGCTGGGGAA

>152_Sample_ASV8

CACCGCGGTTATACGAGAGGCCCAAGTTGACAGACACCGGCGTAAAGAGTGGCTAGGGAA

AAATTACTACTAAAGCCGAACACCTTCAGAACTGTCATACGTTCCCGAAGATAAGAAGCC

CCACCACGAAAGTGGCTTTATATTACCCGACTCCACGAAAGCTGGGGAA

>152_Sample_ASV9

CACCGCGGTTATACGAGAGGCCCAAGTTGACAGACACCGGCGTAAAGAGTGGCTAGGGAA

AAATTACTACTAAAGCCGAACACCTTCAGAACTGTCATACGTTCCCGAAGATAAGAAGCC

CCACCACGAAAGTGGCTTTATATTACCCGACCCCACGAAAGCTGGGGAA

>152_Sample_ASV10

CACCGCGGTTATACGAGAGGCCCAAGTTGACAGACACCGGCGTAAAGAGTGGCTAGGGAA

AAATTACTACTAAAGCCGAACACCTTCAGAACTGTCATACGTTCCCGAAGATAAGAAGCC

CCACCACGAAAGTGGCTTTATATTACCCGACCCCACGAAAGCTGTGAAA

>152_Sample_ASV11

CACCGCGGTTATACGAGAGGCCCAAGTTGACAGACACCGGCGTAAAGAGTGGCTAAGGAA

AAATTTCTACTAAAGCCGAACACCTTCAGAACTGTCATACGTTCCCGAAGAAAAGAAGCC

CCACCACGAAAGTGGCTTTATATTACCCGACCCCACGAAAGCTGTGAAA

>152_Sample_ASV12

CACCGCGGTTAAACGAGAGACCCTAGTTGATCATCACGGCGTAAAGAGTGGTTAGGGGAT

TATCATAAATAAAGCCAAAGACCTCCCAAGCTGTCGCACGCATTCCGGAGGCGCGAAGCC

CACACACGAAAGTAGCTTTAACTATTGACCCTGATGCCACGAAAGCTAAGAAA

>152_Sample_ASV13

CACCGCGGTTATACGAGAGGCCCAAGTTGATAAGCCCGGCGTAAAGAGTGGTTATGGAGA

ATAAAACACTAAAGCTAAAGACCCCCTAGGCTGTTATACGCACCTGGCGGCTCGAACCAC

CTATACGAAAGTAGCTTTACCCCCCTTCCACCAGAATCCACGACAGCTGGGGCA

>152_Sample_ASV14

CACCGCGGTTATACGAGAGGCCCAAGTTGACAGACATCGGCGTAAAGAGTGGTTAAGGAA

ACCCCATTACTAAAGCCGAACGCCCTCAGAACTGTCATACGTTCCCGAAGGTAAGAAGCC

CCACTACGAAAGTGGCTTTAACCACACCCGACCCCACGAAAGCTGTGAAA

>152_Sample_ASV15

CACCGCGGTTATACGAGAGGCCCTAGTTGATAAATACGGCGTAAAGAGTGGTTAGAGGAC

TCCCATAATAAAGCCAAAGACCCCCCGGGCCGTCACACGCATACGAGGGCACGAAGTTCA

CACACGAAAGTAGCTTTAAATTACCCGACGCCACGAAAGCTAAGAAA

>152_Sample_ASV16

GACCGCGGTTATACGAGAGACTCTAGTTGATAGATACGGCGTAAAGAGTGGTTTGGGATA

AACTATAAATAAAGCCAAAGACCTTCCAAGCTGTTAAACGCATACCGAAGGCACGAAGCC

CCAACACGAAAGTAGCTTTACTACTACCCCCGATGCCACGAAAGCTAAGACA

>152_Sample_ASV17

CACCGCGGTCATACGATTAACCCAAGTTAATAGGCACACGGCGTAAAGCGTGTTTAAGCA

CTATGCCAAATAAAGTTAAACTCCAATTAAGCTGTAAAAAGCCATAATCAAGACGAAAAT

AAATAACGAAAGTAACTTTACAACCGCTGAAACACGATAGCTAAGATC

>152_Sample_ASV18

CACCGCGGTTATACGAGAGGCCCTAGTTGATAACTACCGGCGTAAAGAGTGGTTATGGAA

AAATATTTAATAAAGCCGAACACCCCCTCAGCCGTCATACGCACCTGGGAGCACGAAGAC

CTACTGCGAAAGCAGCTTTAATTATGCCTGACCCCACGACAGCTAAGAAA

>152_Sample_ASV19

CACCGCGGTTATACGAAAGACCCTAGTTTATAGACACGGCGTAAAGGGTGGTTCGGGACT

AAACAAAATAAAGCCAAAGACCTCCTAAGCCGTCATACGCTCACGGAAGCACGAAGCCCA

AGTACGAAAGTAGCTTTACAAAACATGCCTGACTCCACGAAAGCTAGGAAA

>152_Sample_ASV20

CACCGCGGTTATACGAAAGACCCTAGTTGATAGGCACGGCGTAAAGGGTGGTTAGGGATA

TATCAAAAATAAAGTTAAAGAGCCTCTAAGCTGTCGCACGCATCCCGAGAGCTCGAGACC

CAGACACGAAAGTAACTTTAAAATACCTCACCTGACCCCACGAAAGCTAAGAAA

>152_Sample_ASV21

CACCGCGGTTATACGAAAGACCCTAGTTGATAGCCACGGCGTAAAGGGTGGTTAAGGTAT

ACAACAAATAAAGCTAAATAGCCTCTAGGCCGTCGCACGCATTCCGAGAGCTCGAAACCC

AAAAACGAAAGTAGCTTTAAAATAAACAGACCTGACCCCACGAAAGCTAAGAAA

>152_Sample_ASV22

CACCGCGGTTATACGAGAGGCCCAAGTTGATACTCCACGGCGTAAAGAGTGGTTAGAAAA

GACCTACTACTAAAGCCGAACGCCTTCAAAGCTGTTATACGCATCCGAAGGTGAGAAGCC

CACCCACGAAAGTGGCTTTACAACCTTGACTCCACGAAAGCTATGACA

>152_Sample_ASV23

CACCGCGGTTATACGAGAGACCCCAGTTGATAAACACGGCGTAAAGAGTGGTTAAGGGCA

CAAAACAAATAAAGTCAAACAACCTCTTAGCTGTTATACGCATTATGAGGATATGAAGCC

CCCCTACGAAAGTAACTTTACCAACCCCTGACCCCACGAAAGCTAAGAAA

>152_Sample_ASV24

CACCGCGGTTATACGAAAGACCCTAGTTTATAGGCACGGCGTAAAGGGTGGTTAGGGGAT

AAACAAAATAAAGCCAAAGACCTTCTAAGTCGTCATACACTCATGAAGGCACGAAGCCCA

AATACGAAAGTAACTTTACAACTTAGACCCGACTCCACGAAAGCTGGGGGA

>152_Sample_ASV25

CACCGCGGTTATACGAGAGGCCCAAGTTGATAGACATCGGCGTAAAGAGTGGTTAAGGAA

GTCTCAAAACTAAAGCCGAATGCCTTCAAGACTGTTATACGTTTCCGAAGGTAAGAAGCC

CCACTACGAAAGTGGCTTTACTCCACCTGACTCCACGAAAGCTGTGAAA

>152_Sample_ASV26

CACCGCGGTTATACGAAAGACCCTAGTTGATAGATCACGGCGTAAAGGGTGGTTAAGGAG

AACAAAAATAAAGCTAAAAATCTTCTAAGCTGTCATACGCTTTCCGAAGACATGAGACCC

AACCACGAAAGTAGCTTTAAACTTTCCTGACACCACGAAAGCCAAGAAA

>162_2_Sample_ASV1

CACCGCGGTTATACGAGAGGCCCAAGTTGACAGACATCGGCGTAAAGAGTGGTTAAGGAA

AACCTTCAACTAAAGTCGAACGCTCTCAAGACTGTCGTACGTTTCCGAGAGTATGAAGCC

CCACCACGAAAGTGACTTTACCTCACCTGACTCCACGAAAGCTGCGAAA

>162_Sample_ASV1

CACCGCGGTTATACGAAAGACCCTAGTTGATGGGTGCGGCGTAAAGGGTGGTTAGGGACT

ACAAAAAATAGAGCTAAAGATCCTCTAGGTCGTCATACACATCACGAGGAAACGAAGCCC

AAATACGAAAGTAGCTCTAAATATAATCCTGACGCCACGAAAGCCAAGAAA

>162_2_Sample_ASV2

CACCGCGGTTATACGAAAGACCCTAGTTGATGGGTGCGGCGTAAAGGGTGGTTAGGGACT

ACAAAAAATAGAGCTAAAGATCCTCTAGGTCGTCATACACATCACGAGGAAACGAAGCCC

AAATACGAAAGTAGCTCTAAATATAATCCTGACGCCACGAAAGCCAAGAAA

>162_2_Sample_ASV3

CACCGCGGTTATACGAAAGACCCTAGTTGATAACTACGGCATAAAGGGTGGTTAGGGAGT

TTAAAAAATAAAGCCAAAGCTCTTCTAAGCTGTCATACGCTCTCTGAAAGAACGAGGCCC

AAACACGAAAGTAGCTTTAAATAAATAACCTGATACCACGAAAGCCAAGAAA

>162_2_Sample_ASV4

CACCGCGGTTATACGAGAGACCCTAGTTGATTAAAGCGGCGTAAAGAGTGGTTATGGAAC

TATTCTTTTAAAGCAGAAAACCTCTCAAACTGTTATACGCACCCAGAGGTCTGAATCCCT

CACACGAAAGTGACTTTATTTATGCCTACCAGAACCCACGAAAGCTGGGACA

>162_2_Sample_ASV5

CACCGCGGTTATACGAGAGACCCTAGTTGATTAAAGCGGCGTAAAGAGTGGTTATGGAAC

TATTCTTTTAAAGCAGAAAACCTCTCAAACTGTTATACGCACCCAGAGGTCTGAATCCCT

CACACGAAAGTGACTTTATTTATGCCTACCAGAACCCACGAAAGCCAAGAAA

>162_Sample_ASV2

CACCGCGGTTATACGAGAGACCCTAGTTGATTAAAGCGGCGTAAAGAGTGGTTATGGAAC

TATTCTTTTAAAGCAGAAAACCTCTCAAACTGTTATACGCACCCAGAGGTCTGAATCCCT

CACACGAAAGTGACTTTATTTATGCCTACCAGAACCCACGAAAGCTGGGACA

>162_2_Sample_ASV6

CACCGCGGTTATACGAAAGACCCTAGTTTATAGGTACGGCGTAAAGGGTGGTTAGGGGAC

AAACAAAATAAAGCCAAAGACCTCCTAAGCCGTCATACGCTCACGGAAGCACGAAGCCCA

AACACGAAAGTAGCTTTACCGAACATGCCCGACTCCACGAAAGCTGGGGAA

>162_2_Sample_ASV7

CACCGCGGTTATACGAGAGACCCTAGTTGATATACACGGCGTAAAGAGTGGTTTGGGGTA

TATAATAAATAAAGCCAAAGACCTTCTAGGCCGTTGAACGCACCACGAAGGAGCGAAGTC

CCAATACGAAAGTAGCTTTATAAAACCTGACGCCACGAAAGCTAAGAAA

>162_2_Sample_ASV8

CACCGCGGTTATACGAGAGGCCCAAGTTGACAGACACCGGCGTAAAGAGTGGCTAGGGAA

AAATTACTACTAAAGCCGAACACCTTCAGAACTGTCATACGTTCCCGAAGATAAGAAGCC

CCACCACGAAAGCGGCTTTATATTACCCGACCCCACGAAAGCTGTGAAA

>162_2_Sample_ASV9

CACCGCGGTTATACTAGAGTCCCAAGTTGACAGACACCGGCGTAAAGAGTGGCTAGGGAA

AAATTACTACTAAAGCCGAACACCTTCAGAACTGTCATACGTTCCCGAAGATAAGAAGCC

CCACCACGAAAGTGGCTTTATATTACCCGACCCCACGAAAGCTGTGAAA

>162_2_Sample_ASV10

CACCGCGGTTATACGAGAGGCCCAAGTTGACAGACACCGGCGTAAAGAGTGGCTAGGGAA

AAATTACTACTAAAGCCGAACACCTTCAGAACTGTCATACGTTCCCGAAGATAAGAAGCC

CCACCACGAAAGTGGCTTTATATTACCCGACCCCACGAAAGCTGTGAAA

>162_Sample_ASV3

CACCGCGGTTATACGAGAGGCCCAAGTTGACAGACACCGGCGTAAAGAGTGGCTAGGGAA

AAATTACTACTAAAGCCGAACACCTTCAGAACTGTCATACGTTCCCGAAGATAAGAAGCC

CCACCACGAAAGTGGCTTTATATTACCCGACCCCACGAAAGCTGTGAAA

>162_Sample_ASV4

CACCGCGATTATACGAGAGGCCCAAGTTGACAGACATCGGCGTAAAGAGTGGTTAAGGAA

AAATTACTACTAAAGCCGAACACCTTCAGAACTGTCATACGTTCCCGAAGATAAGAAGCC

CCACCACGAAAGTGGCTTTATATTACCCGACCCCACGAAAGCTGTGAAA

>162_Sample_ASV5

CACCGCGGTTATACGAGAGGCCCAAGTTGACAGACACCGGCGTAAAGAGTGGCTAAGGAA

AAATTTCTACTAAAGCCGAACACCTTCAGAACTGTCATACGTTCCCGAAGAAAAGAAGCC

CCACCACGAAAGTGGCTTTATATTACCCGACCCCACGAAAGCTGTGAAA

>162_2_Sample_ASV11

CACCGCGGTTATACGAGAGGCCCAAGTTGACAGACACCGGCGTAAAGAGTGGCTAAGGAA

AAATTTCTACTAAAGCCGAACACCTTCAGAACTGTCATACGTTCCCGAAGAAAAGAAGCC

CCACCACGAAAGTGGCTTTATATTACCCGACCCCACGAAAGCTGTGAAA

>162_2_Sample_ASV12

CACCGCGGTTATACGAAAGACCCTAGTTTATAGAAACGGCGTAAAGGGTGGTTAAGGGAC

AAACAAAATAAGGCCAAAGACTTCCTAAGCCGTCATACGCTCCACGAAAGCACGAAGCCC

AAACACGAAAGTAGCCTTAATAACTTCACCTGACCCCACGAAAGCTGGGAGA

>162_2_Sample_ASV13

CACCGCGGTTAAACGAGAGACCCTAGTTGATCATCACGGCGTAAAGAGTGGTTAGGGGAT

TATCATAAATAAAGCCAAAGACCTCCCAAGCTGTCGCACGCATTCCGGAGGCGCGAAGCC

CACACACGAAAGTAGCTTTAACTATTGACCCTGATGCCACGAAAGCTAAGAAA

>162_Sample_ASV6

CACCGCGGTTATACGAGAGGCCCAAGTTGATAAGCCCGGCGTAAAGAGTGGTTATGGAGA

ATAAAACACTAAAGCTAAAGACCCCCTAGGCTGTTATACGCACCTGGCGGCTCGAACCAC

CTATACGAAAGTAGCTTTACCCCCCTTCCACCAGAATCCACGACAGCTGGGGCA

>162_2_Sample_ASV14

CACCGCGGTTATACGAGAGGCCCAAGTTGATAAGCCCGGCGTAAAGAGTGGTTATGGAGA

ATAAAACACTAAAGCTAAAGACCCCCTAGGCTGTTATACGCACCTGGCGGCTCGAACCAC

CTATACGAAAGTAGCTTTACCCCCCTTCCACCAGAATCCACGACAGCTGGGGCA

>162_Sample_ASV7

CACCGCGGTTATACGAGAGGCCCTAGTTGATAGCCACGGCGCAAAGAGTGGTTAAGGAGC

CCCACTAAATAAAGTCGAACACTTCCTAGGCCGTTATACGTTTTCTAGAAGCACGAAACC

CAATTACACGAAAGCAACTTTATACTAAACAACCTGACCCCACGAAAGCTAAGAAA

>162_Sample_ASV8

CACCGCGGTTATACGAGAGGCCCAAGTTGACAGACATCGGCGTAAAGAGTGGTTAAGGAA

ACCCCATTACTAAAGCCGAACGCCCTCAGAACTGTCATACGTTCCCGAAGGTAAGAAGCC

CCACTACGAAAGTGGCTTTAACCACACCCGACCCCACGAAAGCTGTGAAA

>162_Sample_ASV9

CACCGCGGTTATACGAGAGGCCCAAGTTGACAGACATCGGCGTAAAGAGTGGTTAAGGAA

ACCCCATTACTAAAGCCGAACGCCCTCAGAACTGTCATACGTTCCCGAAGGTAAGAAGCC

CCACTACGAAAGTGGCTTTAACCACACCCGACCCCACGAAAGCTAAGAAA

>162_2_Sample_ASV15

CACCGCGGTTATACGAGAGGCCCAAGTTGACAGACATCGGCGTAAAGAGTGGTTAAGGAA

ACCCCATTACTAAAGCCGAACGCCCTCAGAACTGTCATACGTTCCCGAAGGTAAGAAGCC

CCACTACGAAAGTGGCTTTAACCACACCCGACCCCACGAAAGCTGTGAAA

>162_2_Sample_ASV16

CACCGCGGTTATACGAGAGGCCCAAGTTGACAGACACCGGCGTAAAGAGTGGTTAAGGAA

ATTCATCAACTAAAGCCGAATGCCCTCAGGACTGTTATACGTTTGCGAAGGTAAGAAGCC

CTACTACGAAAGTGGCTTTATTCCACCTGATTCCACGAAAGCTGCGGAA

>162_2_Sample_ASV17

CACCGCGGTTATACGAGAGGCCCCAGTTGATATTAGCGGCGTAAAGGGTGGTTAAGGACT

TTAATAACTAAAGCTAAACACTTCCCCGGCTGTTGTACGTAGACCGGAAGCTTGAAGCCC

AGACACGAAAGTAGCTTTATTCCCCCTGACCCCACGAAAGCTGAGAAA

>162_2_Sample_ASV18

CACCGCGGTCACACGATTAACCCAAGTCAATAGAAGCCGGCGTAAAGAGTGTTTTAGATC

ACCCCCTCCCCAATAAAGCTAAAACTCACCTGAGTTGTAAAAAACTCCAGTTGACACAAA

ATAGACTACGAAAGTGGCTTTAACATATCTGAACACACAATAGCTAAGACC

>162_Sample_ASV10

CACCGCGGTCACACGATTAACCCAAGTCAATAGAAGCCGGCGTAAAGAGTGTTTTAGATC

ACCCCCTCCCCAATAAAGCTAAAACTCACCTGAGTTGTAAAAAACTCCAGTTGACACAAA

ATAGACTACGAAAGTGGCTTTAACATATCTGAACACACAATAGCTAAGACC

>162_2_Sample_ASV19

CACCGCGGTTATACGAGAAACCCTAGTTGACAAATACGGCGTAAAGAGTGGTTAAGGGGT

ATCAAAAATAAAGTCAAACATTTTCCTAGTTGTTATAAGCATTTCGAGAACATGAAGCCC

AACCACGAAAGTAACTTTACCTTCACCTGACCCCACGAAAGCTAAGAAA

>162_2_Sample_ASV20

CACCGCGGTTATACGAGAAACCCTAGTTGACAAATACGGCGTAAAGAGTGGTTAAGGGGT

ATCAAAAATAAAGTCAAACATTTTCCTAGTTGTTATAAGCATTTCGAGAACATGAAGCCC

AACCACGAAAGTAACTTTACCTCCACCTGACCCCACGAAAGCTAAGAAA

>162_Sample_ASV11

CACCGCGGTTATACGAAAGACCCCAGTTTATAGACACGGCGTAAAGGGTGGTTATAGGAC

AAACTAACATAGAGCCAAAGACCCCCTAAGCCGTCATACGCTCACGAGGGCTCGAAGCCC

CTATACGAAAGTAGCTCTACCAAAAAATGCTCGACTCCACGAAAGCTGGGACA

>162_2_Sample_ASV21

CACCGCGGTTATACGAGAGACCCTAGTTGATAAATACGGCGTAAAGAGTGGTTATGGGAA

AAACAAATAAAGTCAAACAACCTCTTAGCTGTTATACGCATTATGAGAGCATGAAGCCCC

CTCACGAAAGTAACTTTAATACCTCCTGACCCCACGAAAGCTAAGAAA

>162_Sample_ASV12

CACCGCGGTTATACGAGAGACCCTAGTTGATAAATACGGCGTAAAGAGTGGTTATGGGAA

AAACAAATAAAGTCAAACAACCTCTTAGCTGTTATACGCATTATGAGAGCATGAAGCCCC

CTCACGAAAGTAACTTTAATACCTCCTGACCCCACGAAAGCTAAGAAA

>162_2_Sample_ASV22

CACCGCGGTTATACGAGAGACCCCAGTTGATAAACACGGCGTAAAGAGTGGTTAAGGGCA

CAAAACAAATAAAGTCAAACAACCTCTTAGCTGTTATACGCATTATGAGAGCATGAAGCC

CCCTCACGAAAGTAACTTTAATACCTCCTGACCCCACGAAAGCTAAGAAA

>162_2_Sample_ASV23

CACCGCGGTTATACGAGAGACCCTAATTGATAGGTACGGCGTAAAGAGTGGTTTGGGATA

ACCCAAAAATAAAGCTAAAGACCTTCTAAGCCGTCACACGCATACCGAAGGCACGAGGCC

CCAACACGAAAGTAGCTTTACTATTACCCCGACGCCACGAAAGCTAAGAAA

>162_Sample_ASV13

CACCGCGGTTATACGAAAGACCCTAGTTGATGGATCACGGCGTAAAGGGTGGTTAAGGGA

TATAAAAATAAAGCTAAAAATCCTCTTAGCTGTCATACGCTTTCCGAAGACATGAGGCCC

AATTACGAAAGTAGCTTTAAACCACCCTGACACCACGAAAGCCAAGAAA

>162_2_Sample_ASV24

CACCGCGGTTATACGAGAGACCCTAGTTGATAGCTACGGCGTAAAGCGTGGTTAGGAGAA

CAAGATAAATAAAGTCAAACAATCTCTCGGCCGTTATACGTTATCTGAGAATATGAAGTC

CTACCACGAAAGTAACTTTAATTTTTCTGACCCCACGAAAACTGAGGAA

>162_2_Sample_ASV25

CACCGCGGTCATACGATTAACCCAAGTTAATAGGCACACGGCGTAAAGCGTGTTTAAGCA

CTACGCCAAATAAAGTTAAACTCCAATTAAGCTGTAAAAAGCCATAATCAAGACGAAAAT

AAATAACGAAAGTAACTTTACAACCGCTGAAACACGATAGCTAAGATC

>162_2_Sample_ASV26

CACCGCGGTTATACGAGAGGCCCTAGTTGATAACTACCGGCGTAAAGAGTGGTTATGGAA

AAATATTTAATAAAGCCGAACACCCCCTCAGCCGTCATACGCACCTGGGAGCACGAAGAC

CTACTGCGAAAGCAGCTTTAATTATGCCTGACCCCACGACAGCTAAGAAA

>162_Sample_ASV14

CACCGCGGTTATACGAGAGGCCCTAGTTGATAACTACCGGCGTAAAGAGTGGTTATGGAA

AAATATTTAATAAAGCCGAACACCCCCTCAGCCGTCATACGCACCTGGGAGCACGAAGAC

CTACTGCGAAAGCAGCTTTAATTATGCCTGACCCCACGACAGCTAAGAAA

>162_Sample_ASV15

CACCGCGGTTATACGAGAGGCCCAAGTTGATTATTACCGGCGTAAAGCGTGGTTAAGATT

TTTATAGGACTAAAGCCGAACCCCCTCAGAGCTGTTATACGCACCCGAAGGTTAGAAGTT

CAATCACGAAAGTGGCTTTACAACCCCGAACCCACGAAAGCTAAGATA

>162_Sample_ASV16

CACCGCGGTTATACGAGAGACCCTAATTGATAGGCGCGGCGTAAAGAGTGGTTTAGGACT

AAAACTAATAAAGCTAAAGATCCTCCCAGCCGTCGCACGCATTCTGAGGACACGAAACTC

AACCACGAAAGTAGCTTTAACATACTCTCCTGACCCCACGAAAGCTAAGAAA

>162_Sample_ASV17

CACCGCGGTTATACGAAAGACCCTAGTTGATAGGCACGGCGTAAAGGGTGGTTAGGGATA

TATCAAAAATAAAGTTAAAGAGCCTCTAAGCTGTCGCACGCATCCCGAGAGCTCGAGACC

CAGACACGAAAGTAACTTTAAAATACCTCACCTGACCCCACGAAAGCTAAGAAA

>162_2_Sample_ASV27

CACCGCGGTTATACGAGAGGCCCAAGTTGATACTCCACGGCGTAAAGAGTGGTTAGAAAA

GACCTACTACTAAAGCCGAACGCCTTCAAAGCTGTTATACGCATCCGAAGGTGAGAAGCC

CACCCACGAAAGTGGCTTTACAACCTTGACTCCACGAAAGCTATGACA

>162_Sample_ASV18

CACCGCGGTTATACGAGAGACCCCAGTTGATAAACACGGCGTAAAGAGTGGTTAAGGGCA

CAAAACAAATAAAGTCAAACAACCTCTTAGCTGTTATACGCATTATGAGGATATGAAGCC

CCCCTACGAAAGTAACTTTACCAACCCCTGACCCCACGAAAGCTAAGAAA

>162_2_Sample_ASV28

CACCGCGGTTATACGAGAGACCCCAGTTGATAAACACGGCGTAAAGAGTGGTTAAGGGCA

CAAAACAAATAAAGTCAAACAACCTCTTAGCTGTTATACGCATTATGAGGATATGAAGCC

CCCCTACGAAAGTAACTTTACCAACCCCTGACCCCACGAAAGCTAAGAAA

>162_Sample_ASV19

CACCGCGGTTATACGAAAGACCCTAGTTTATAGGCACGGCGTAAAGGGTGGTTAGGGGAT

AAACAAAATAAAGCCAAAGACCTTCTAAGTCGTCATACACTCATGAAGGCACGAAGCCCA

AATACGAAAGTAACTTTACAACTTAGACCCGACTCCACGAAAGCTGGGGGA

>162_2_Sample_ASV29

CACCGCGGTTATACGAGAGACCCTAATTAATAGCTACGGCGTAAAGAGTGGTTTAGGACA

AAAATTAATAAAGCCGAAGTTTCTCCTAGCCGTTGTACGCACTCTGAGAACACGAAGCCC

CACTACGAAAGTAGCTTTACCAATAATCTCCTGACCCCACGAAAGCTAAGAAA

>162_2_Sample_ASV30

CACCGCGGTTATACGAGAGACCCTAATTAATAGATACGGCGTAAAGAGTGGTTTAGGACA

AAAATTAATAAAGCCGAAGTTTCTCCTAGCCGTTGTACGCACTCTGAGAACACGAAGCCC

CACTACGAAAGTAGCTTTACCAATAATCTCCTGACCCCACGAAAGCTAAGAAA

>162_2_Sample_ASV31

CACCGCGGTTATACGAGAGACCCTAATTAATAGCTACGGCGTAAAGAGTGGTTTAGGACA

AAAATTAATAAAGCCGAAGTTTCTCCTAGCCGTTGTACGCACTCTGAGAACACGAAGCCC

CACTACGAAAGTAGCTTTACCTATTATTTCCTGACCCCACGAAAGCTAAGAAA

>162_2_Sample_ASV32

CACCGCGGTTATACGAGAGACCCTAATTAATAGCTACGGCGTAAAGAGTGGTTTAGGGTA

AAAATTAATAAAGCCGAAGTTTCTCCTAGCCGTTGTACGCACTCTGAGAACACGAAGCCC

CACTACGAAAGTAGCTTTACCTATTATTTCCTGACCCCACGAAAGCTAAGAAA

>162_2_Sample_ASV33

CACCGCGGTTATACGAGAGACCCTAATTAATAGATACGGCGTAAAGAGTGGTTTAGGACA

AAAATTAATAAAGCCGAAGTTTCTCCTAGCCGTTGTACGCACTCTGAGAACACGAAGCCC

CACTACGAAAGTAGCTTTACCTATTATTTCCTGACCCCACGAAAGCTAAGAAA

>162_Sample_ASV20

CACCGCGGTTATACGAGAGACCCTAATTAATAGATACGGCGTAAAGAGTGGTTTAGGACA

AAAATTAATAAAGCCGAAGTTTCTCCTAGCCGTTGTACGCACTCTGAGAACACGAAGCCC

CACTACGAAAGTAGCTTTACCAATAATCTCCTGACCCCACGAAAGCTAAGAAA

>162_Sample_ASV21

CACCGCGGTTATACGAGAGACCCTAATTAATAGCTACGGCGTAAAGAGTGGTTTAGGGTA

AAAATTAATAAAGCCGAAGTTTCTCCTAGCCGTTGTACGCACTCTGAGAACACGAAGCCC

CACTACGAAAGTAGCTTTACCTATTATTTCCTGACCCCACGAAAGCTAAGAAA

>162_Sample_ASV22

CACCGCGGTTATACGAGAGACCCTAATTAATAGATACGGCGTAAAGAGTGGTTTAGGACA

AAAATTAATAAAGCCGAAGTTTCTCCTAGCCGTTGTACGCACTCTGAGAACACGAAGCCC

CACTACGAAAGTAGCTTTACCTATTATTTCCTGACCCCACGAAAGCTAAGAAA

>162_2_Sample_ASV34

CACCGCGGTTATACGAGAGACCCTAATTAATAGATACGGCGTAAAGAGTGGTTTAGGACA

AAAATTAATAAAGCCGAAGATTCTCCTGGCTGTTGCACGCACTCTGAGAACACGAAGCCC

CACTACGAAAGTAGCTTTACCTATTATTTCCTGACCCCACGAAAGCTAAGAAA

>162_2_Sample_ASV35

CACCGCGGTTATACGAGAGGCCCAAGTTGATAGACATCGGCGTAAAGAGTGGTTAAGGAA

GTCTCAAAACTAAAGCCGAATGCCTTCAAGACTGTTATACGTTTCCGAAGGTAAGAAGCC

CCACTACGAAAGTGGCTTTACTCCACCTGACTCCACGAAAGCTGTGAAA

>162_Sample_ASV23

CACCGCGGTTATACGAGAGGCCCAAGTTGATAGACATCGGCGTAAAGAGTGGTTAAGGAA

GTCTCAAAACTAAAGCCGAATGCCTTCAAGACTGTTATACGTTTCCGAAGGTAAGAAGCC

CCACTACGAAAGTGGCTTTACTCCACCTGACTCCACGAAAGCTGTGAAA

>162_Sample_ASV24

CACCGCGGTTATACGAGAGGCCCAAGTTGATAGACATCGGCGTAAAGAGTGGTTAAGGAA

GTCTCAAAACTAAAGCCGAATGCCTTCAAGACTGTTATACGTTTCCGAAGGTAAGAAGCC

CCACTACGAAAGTGGCTTTACTCCACCTGACTCCACGAAAGCTGGGACA

>162_2_Sample_ASV36

CACCGCGGTTATACGAGAGACTCCAGTTGATAGCTACGGCGTAAAGAGTGGTTTGGGGCC

CGCCCAAAATAAAGCCAAAGACCTCCCAAGCCGTCAAACGCACCCCGGAGGCACGAAGTC

CTAACGCGAAAGCAACTTTACCTTCCCCGACGCCACGAAAGCTAAGAAA

>162_2_Sample_ASV37

CACCGCGGTTATACGAAAGACCCTAGTTGATAGCTACGGCGTAAAGGGTGGTTAAGGTAT

ATAACAAATAAAGCTAAAGAGCCTCCAAGCCGTCGTACGCATTCTGAGAGCTCGAAACCC

TAACACGAAAGTAGCTTTAAAAAATAACACCTGACCCCACGAAAGCTAAGAAA

>162_2_Sample_ASV38

CACCGCGGTTATACGAAAGACCCTAGTTGATAGATCACGGCGTAAAGGGTGGTTAAGGAG

AACAAAAATAAAGCTAAAAATCCTCTAAGCTGTCATACGCTTTCCGAAGACATGAGACCC

AACCACGAAAGTAACTTTAAACTTTCCTGACACCACGAAAGCCAAGAAA

>162_Sample_ASV25

CACCGCGGTTATACGAAAGACCCTAGTTGATAGATCACGGCGTAAAGGGTGGTTAAGGAA

AACAAAAATAAAGCTAAAAATCCTCTAAGCTGTCATACGCTTTCCGAAGACATGAGATCC

AACCACGAAAGTAGCTTTAAAATTTCCTGACACCACGAAAGCCAAGGAA

>174_Sample_ASV1

CACCGCGGTTATACGAAAGACCCTAGTTGATGGGTGCGGCGTAAAGGGTGGTTAGGGACT

ACAAAAAATAGAGCTAAAGATCCTCTAGGTCGTCATACACATCACGAGGAAACGAAGCCC

AAATACGAAAGTAGCTCTAAATATAATCCTGACGCCACGAAAGCCAAGAAA

>174_Sample_ASV2

CACCGCGGTTATACGAGAGACCCTAGTTGATTAAAGCGGCGTAAAGAGTGGTTATGGAAC

TATTCTTTTAAAGCAGAAAACCTCTCAAACTGTTATACGCACCCAGAGGTCTGAATCCCT

CACACGAAAGTGACTTTATTTATGCCTACCAGAACCCACGAAAGCTGGGACA

>174_Sample_ASV3

CACCGCGGTTATACGAGAGACCCTAGTTGATTTAAGCGGCGTAAAGGGTGGTTATGGAAC

TATTCTTTTAAAGCAGAAAACCTCTCAAACTGTTATACGCACCCAGAGGTCTGAATCCCT

CACACGAAAGTGACTTTATTTATGCCTACCAGAACCCACGAAAGCTGGGACA

>174_Sample_ASV4

CACCGCGGTTATACGAGAGACCCTAGTTGATATACACGGCGTAAAGAGTGGTTTGGGGTA

TATAATAAATAAAGCCAAAGACCTTCTAGGCCGTTGAACGCACCACGAAGGAGCGAAGTC

CCAATACGAAAGTAGCTTTATAAAACCTGACGCCACGAAAGCTAAGAAA

>174_Sample_ASV5

CACCGCGGTTATACGAGAGGCCCAAGTTGACAGACACCGGCGTAAAGAGTGGCTAGGGAA

AAATTACTACTAAAGCCGAACACCTTCAGAACTGTCATACGTTCCCGAAGATAAGAAGCC

CCACCACGAAAGTGGCTTTATATTACCCGACCCCACGAAAGCTGTGAAA

>174_Sample_ASV6

CACCGCGGTTATACGAGAGGCCCAAGTTGACAGACACCGGCGTAAAGAGTGGCTAGGGAA

AAATTACTACTAAAGCCGAACACCTTCAGAACTGTCATACGTTCCCGAAGATAAGAAGCC

CCACCACGAAAGTGGCTTTATATTACCCGACCCCACGAAAGCTAAGACA

>174_Sample_ASV7

CACCGCGGTTATACGAGAGGCCCAAGTTGATAAGCCCGGCGTAAAGAGTGGTTATGGAGA

ATAAAACACTAAAGCTAAAGACCCCCTAGGCTGTTATACGCACCTGGCGGCTCGAACCAC

CTATACGAAAGTAGCTTTACCCCCCTTCCACCAGAATCCACGACAGCTGGGGCA

>174_Sample_ASV8

CACCGCGGTTATACGAGAGGCCCTAGTTGATAGCCACGGCGCAAAGAGTGGTTAAGGAGC

CCCACTAAATAAAGTCGAACACTTCCTAGGCCGTTATACGTTTTCTAGAAGCACGAAACC

CAATTACACGAAAGCAACTTTATACTAAACAACCTGACCCCACGAAAGCTAAGAAA

>174_Sample_ASV9

CACCGCGGTTATACGAGAGGCCCAAGTTGACAGACATCGGCGTAAAGAGTGGTTAAGGAA

ACCCCATTACTAAAGCCGAACGCCCTCAGAACTGTCATACGTTCCCGAAGGTAAGAAGCC

CCACTACGAAAGTGGCTTTAACCACACCCGACCCCACGAAAGCTAAGACA

>174_Sample_ASV10

CACCGCGGTTATACGAGAGGCCCAAGTTGACAGACATCGGCGTAAAGAGTGGTTAAGGAA

ACCCCATTACTAAAGCCGAACGCCCTCAGAACTGTCATACGTTCCCGAAGGTAAGAAGCC

CCACTACGAAAGTGGCTTTAACCACACCCGACCCCACGAAAGCTGTGAAA

>174_Sample_ASV11

CACCGCGGTTATACGAGAGGCCCAAGTTGATAGATATCGGCGTAAAGAGTGGTTAAGGAA

ACCCCGTTACTAAAGCCGAACGCCCTCAGAACTGTCATACGTTCCCGAAGGTAAGAAGCC

CCACTACGAAAGTGGCTTTAACCACACCCGACCCCACGAAAGCTGTGAAA

>174_Sample_ASV12

CACCGCGGTCACACGATTAACCCAAGTCAATAGAAGCCGGCGTAAAGAGTGTTTTAGATC

ACCCCCTCCCCAATAAAGCTAAAACTCACCTGAGTTGTAAAAAACTCCAGTTGACACAAA

ATAGACTACGAAAGTGGCTTTAACATATCTGAACACACAATAGCTAAGACC

>174_Sample_ASV13

CACCGCGGTTATACGAGAAACCCTAGTTGACAAATACGGCGTAAAGAGTGGTTAAGGGGT

ATCAAAAATAAAGTCAAACATTTTCCTAGTTGTTATAAGCATTTCGAGAACATGAAGCCC

AACCACGAAAGTAACTTTACCTTCACCTGACCCCACGAAAGCTAAGAAA

>174_Sample_ASV14

CACCGCGGTTATACGAGAGACCCTAGTTGATAAATACGGCGTAAAGAGTGGTTATGGGAA

AAACAAATAAAGTCAAACAACCTCTTAGCTGTTATACGCATTATGAGAGCATGAAGCCCC

CTCACGAAAGTAACTTTAATACCTCCTGACCCCACGAAAGCTAAGAAA

>174_Sample_ASV15

AACCGCGGTTATACGAGAGACCCTAATTGATAGCTCACGGCGTAAAGGGTGGTTTGGGGA

AACCTCAAAATAAAGCTAAAGACCTTCTAAGCCGTTACACGCATACCGAAGGCACGAGGC

CCTAACACGAAAGTAGCTTTACTATTACCCCTGACGCCACGAAAGCTAAGAAA

>174_Sample_ASV16

CACCGCGGTTATACGAAAGACCCTAGTTGATGGATCACGGCGTAAAGGGTGGTTAAGGGA

TATAAAAATAAAGCTAAAAATCCTCTTAGCTGTCATACGCTTTCCGAAGACATGAGGCCC

AATTACGAAAGTAGCTTTAAACCACCCTGACACCACGAAAGCCAAGAAA

>174_Sample_ASV17

CACCGCGGTTATACGAGAGGCCCAAGTTGATACTCCACGGCGTAAAGAGTGGTTAGAAAA

GACCTACTACTAAAGCCGAACGCCTTCAAAGCTGTTATACGCATCCGAAGGTGAGAAGCC

CACCCACGAAAGTGGCTTTACAACCTTGACTCCACGAAAGCTATGACA

>174_Sample_ASV18

CACCGCGGTTATACGAGAGACCCCAGTTGATAAACACGGCGTAAAGAGTGGTTAAGGGCA

CAAAACAAATAAAGTCAAACAACCTCTTAGCTGTTATACGCATTATGAGGATATGAAGCC

CCCCTACGAAAGTAACTTTACCAACCCCTGACCCCACGAAAGCTAAGAAA

>174_Sample_ASV19

CACCGCGGTTATACGAAAGACCCTAGTTTATAGGCACGGCGTAAAGGGTGGTTAGGGGAT

AAACAAAATAAAGCCAAAGACCTTCTAAGTCGTCATACACTCATGAAGGCACGAAGCCCA

AATACGAAAGTAACTTTACAACTTAGACCCGACTCCACGAAAGCTGTGAAA

>174_Sample_ASV20

CACCGCGGTTATACGAAAGACCCTAGTTTATAGGCACGGCGTAAAGGGTGGTTAGGGGAT

AAACAAAATAAAGCCAAAGACCTTCTAAGTCGTCATACACTCATGAAGGCACGAAGCCCA

AATACGAAAGTAACTTTACAACTTAGACCCGACTCCACGAAAGCTGGGGGA

>174_Sample_ASV21

CACCGCGGTTATACGAGAGACCCTAATTAATAGATACGGCGTAAAGAGTGGTTTAGGACA

AAAATTAATAAAGCCGAAGTTTCTCCTAGCCGTTGTACGCACTCTGAGAACACGAAGCCC

CACTACGAAAGTAGCTTTACCTATTATTTCCTGACCCCACGAAAGCTAAGAAA

>174_Sample_ASV22

CACCGCGGTTATACGAGAGGCCCAAGTTGATAGACATCGGCGTAAAGAGTGGTTAAGGAA

GTCTCAAAACTAAAGCCGAATGCCTTCAAGACTGTTATACGTTTCCGAAGGTAAGAAGCC

CCACTACGAAAGTGGCTTTATTCCACCTGACCCCACGAAAGCTGTGAAA

>174_Sample_ASV23

CACCGCGGTTATACGAGAGGCCCAAGTTGATAGACATCGGCGTAAAGAGTGGTTAAGGAA

GTCTCAAAACTAAAGCCGAATGCCTTCAAGACTGTTATACGTTTCCGAAGGTAAGAAGCC

CCACTACGAAAGTGGCTTTACTCCACCTGACTCCACGAAAGCTGTGAAA

>175_Sample_ASV1

CACCGCGGTTATACGAAAGACCCTAGTTGATGGGTGCGGCGTAAAGGGTGGTTAGGGACT

ACAAAAAATAGAGCTAAAGATCCTCTAGGTCGTCATACACATCACGAGGAAACGAAGCCC

AAATACGAAAGTAGCTCTAAATATAATCCTGACGCCACGAAAGCCAAGAAA

>175_Sample_ASV2

CACCGCGGTTATACGAGAGACCCTAGTTGATTAAAGCGGCGTAAAGAGTGGTTATGGAAC

TATTCTTTTAAAGCAGAAAACCTCTCAAACTGTTATACGCACCCAGAGGTCTGAATCCCT

CACACGAAAGTGACTTTATTTATGCCTACCAGAACCCACGAAAGCTGGGACA

>175_Sample_ASV3

CACCGCGGTTATACGAGAGACCCTAGTTGATTAAAACGGCGTAAAGAGTGGTTATGGAAC

TATTCTTTTAAAGCAGAAAACCTCTCAAACTGTTATACGCACCCAGAGGTCTGAATCCCT

CACACGAAAGTGACTTTATTTATGCCTACCAGAACCCACGAAAGCTGGGACA

>175_Sample_ASV4

CACCGCGGTTATACGAGAGACCCTAGTTGATTAAAGCGGCGTAAAGAGTGGTTATGGAAC

TATTCTTTTAAAGCAGAAAACCTCTCAAACTGTTATACGCACCCAGAGGTCTGAATCCCT

CACACGAAAGTGACTTTATTTATGCCTACCAGAACCCACGAAAGCTAAGACA

>175_Sample_ASV5

CACCGCGGTTATACGAGAGACCCTAGTTGATTAAAGCGGCGTAAAGAGTGGTTATGGAAC

TATTCTTTTAAAGCAGAAAACCTCTCAAACTGTTATACGCACCCAGAGGTCTGAATCCCT

CACACGAAAGTGACTTTATTTTGCCCACCAGAACCCACGAAAGCTAAGACA

>175_Sample_ASV6

CACCGCGGTTATACGAGAGACCCTAGTTGATTAAAACGGCGTAAAGAGTGGTTATGGAAC

TATTCTTTTAAAGCAGAAAACCTCTCAAACTGTTATACGCACCCAGAGGTCTGAATCCCT

CACACGAAAGTGACTTTATTTATGCCTACCAGAACCCACGAAAGCTAAGACA

>175_Sample_ASV7

CACCGCGGTTATACGAGAGACCCTAGTTGATTAAAGCGGCGTAAAGAGTGGTTATGGAAC

TATTCTTTTAAAGCAGAAAACCTCTCAAACTGTTATACGCACCCAGAGGTCTGAATCCCT

CACACGAAAGTAACTTTATTTTGCCCACCAGAACCCACGAAAGCTAAGACA

>175_Sample_ASV8

CACCGCGGTTATACGAGAGACCCTAGTTGATTAAAACGGCGTAAAGAGTGGTTATGGAAC

TATTCTTTTAAAGCAGAAAACCTCTCAAACTGTTATACGCACCCAGAGGTCTGAATCCCT

CACACGAAAGTAACTTTATTTTGCCCACCAGAACCCACGAAAGCTAAGACA

>175_Sample_ASV9

CACCGCGGTTATACGAAAGACCCTAGTTTATAGGTACGGCGTAAAGGGTGGTTAGGGGAC

AAACAAAATAAAGCCAAAGACCTCCTAAGCCGTCATACGCTCACGGAAGCACGAAGCCCA

AACACGAAAGTAGCTTTACCGAACATGCCCGACTCCACGAAAGCTGGGGAA

>175_Sample_ASV10

CACCGCGGTTATACGAGAGACCCTAGTTGATATACACGGCGTAAAGAGTGGTTTGGGGTA

TATAATAAATAAAGCCAAAGACCTTCTAGGCCGTTGAACGCACCACGAAGGAGCGAAGTC

CCAATACGAAAGTAGCTTTATAAAACCTGACGCCACGAAAGCTAAGAAA

>175_Sample_ASV11

CACCGCGGTTATACGAGAGGCCCAAGTTGACAGACACCGGCGTAAAGAGTGGCTAGGGAA

AAATTACTACTAAAGCCGAACACCTTCAGAACTGTCATACGTTCCCGAAGATAAGAAGCC

CCACCACGAAAGTGACTTTATATTACCCGACCCCACGAAAGCTGTGAAA

>175_Sample_ASV12

CACCGCGGTTATACGAGAGGCCCAAGTTGACAGACACCGGCGTAAAGAGTGGCTAGGGAA

AAATTACTACTAAAGCCGAACACCTTCAGAACTGTCATACGTTCCCGAAGATAAGAAGCC

CCACCACGAAAGTGGCTTTATATTACCCGACCCCACGAAAGCTGTGAAA

>175_Sample_ASV13

CACCGCGGTTATACGAGAGGCCCAAGTTGACAGACACCGGCGTAAAGAGTGGCTAGGGAA

AAATTACTACTAAAGCCGAACACCTTCAGAACTGTCATACGTTCCCGAAGATAAGAAGCC

CCACCACGAAAGTGGCTTTATATTACCCGACCCCACGAAAGCTAAGACA

>175_Sample_ASV14

CACCGCGGTTATACGAGAGGCCCAAGTTGACAGACACCGGCGTAAAGAGTGGCTAAGGAA

AAATTTCTACTAAAGCCGAACACCTTCAGAACTGTCATACGTTCCCGAAGAAAAGAAGCC

CCACCACGAAAGTGGCTTTATATTACCCGACCCCACGAAAGCTGTGAAA

>175_Sample_ASV15

CACCGCGGTTATACGAGAGGCCCAAGTTGATAAGCCCGGCGTAAAGAGTGGTTATGGAGA

ATAAAACACTAAAGCTAAAGACCCCCTAGGCTGTTATACGCACCTGGCGGCTCGAACCAC

CTATACGAAAGTAGCTTTACCCCCCTTCCACCAGAATCCACGACAGCTGGGGCA

>175_Sample_ASV16

CACCGCGGTTATACGAGAGGCCCAAGTTGACAGACATCGGCGTAAAGAGTGGTTAAGGAA

ACCCCATTACTAAAGCCGAACGCCCTCAGAACTGTCATACGTTCCCGAAGGTAAGAAGCC

CCACTACGAAAGTGGCTTTAACCACACCCGACCCCACGAAAGCTGTGAAA

>175_Sample_ASV17

CACCGCGGTTATACGAGAGGCCCAAGTTGACAGACATCGGCGTAAAGAGTGGTTAAGGAA

ACCCCATTACTAAAGCCGAACGCCCTCAGAACTGTCATACGTTCCCGAAGGTAAGAAGCC

CCACTACGAAAGTGGCTTTAACCACACCCGACCCCACGAAAGCTAAGACA

>175_Sample_ASV18

CACCGCGGTTATACGAGAAACCCTAGTTGACAAATACGGCGTAAAGAGTGGTTAAGGGGT

ATCAAAAATAAAGTCAAACATTTTCCTAGTTGTTATAAGCATTTCGAGAACATGAAGCCC

AACCACGAAAGTAACTTTACCTTCACCTGACCCCACGAAAGCTAAGAAA

>175_Sample_ASV19

CACCGCGGTTATACGAGAGACCCTAGTTGATAAATACGGCGTAAAGAGTGGTTATGGGAA

AAACAAATAAAGTCAAACAACCTCTTAGCTGTTATACGCATTATGAGAGCATGAAGCCCC

CTCACGAAAGTAACTTTAATACCTCCTGACCCCACGAAAGCTAAGAAA

>175_Sample_ASV20

AACCGCGGTTATACGAGAGACCCTAATTGATAGCTCACGGCGTAAAGGGTGGTTTGGGGA

AACCTCAAAATAAAGCTAAAGACCTTCTAAGCCGTTACACGCATACCGAAGGCACGAGGC

CCTAACACGAAAGTAGCTTTACTATTACCCCTGACGCCACGAAAGCTAAGAAA

>175_Sample_ASV21

CACCGCGGTTATACGAAAGACCCTAGTTGATGGATCACGGCGTAAAGGGTGGTTAAGGGA

TATAAAAATAAAGCTAAAAATCCTCTTAGCTGTCATACGCTTTCCGAAGACATGAGGCCC

AATTACGAAAGTAGCTTTAAACCACCCTGACACCACGAAAGCCAAGAAA

>175_Sample_ASV22

CACCGCGGTTATACGAGAGGCCCAAGTTGACAGGTACCGGCGTAAAGAGTGGTTAAGAAA

AAATATTAAACTAAAGCCGAACACCTTCAGAGCTGTCATACGTTTCCGAAGGCATGAAGC

CCCACCACGAAAGTGGCTTTATTTTGCCCGACTCCACGAAAGCTGTGAAA

>175_Sample_ASV23

CACCGCGGTTATACGAGAGGCCCTAGTTGATAACTACCGGCGTAAAGAGTGGTTATGGAA

AAATATTTAATAAAGCCGAACACCCCCTCAGCCGTCATACGCACCTGGGAGCACGAAGAC

CTACTGCGAAAGCAGCTTTAATTATGCCTGACCCCACGACAGCTAAGAAA

>175_Sample_ASV24

CACCGCGGTTATACGAGAGGCCCAAGTTGATACTCCACGGCGTAAAGAGTGGTTAGAAAA

GACCTACTACTAAAGCCGAACGCCTTCAAAGCTGTTATACGCATCCGAAGGTGAGAAGCC

CACCCACGAAAGTGGCTTTACAACCTTGACTCCACGAAAGCTAAGACA

>175_Sample_ASV25

CACCGCGGTTATACGAGAGGCCCAAGTTGATACTCCACGGCGTAAAGAGTGGTTAGAAAA

GACCTACTACTAAAGCCGAACGCCTTCAAAGCTGTTATACGCATCCGAAGGTGAGAAGCC

CACCCACGAAAGTGGCTTTACAACCTTGACTCCACGAAAGCTATGACA

>175_Sample_ASV26

CACCGCGGTTATACGAGAGACCCCAGTTGATAAACACGGCGTAAAGAGTGGTTAAGGGCA

CAAAACAAATAAAGTCAAACAACCTCTTAGCTGTTATACGCATTATGAGGATATGAAGCC

CCCCTACGAAAGTAACTTTACCAACCCCTGACCCCACGAAAGCTAAGAAA

>175_Sample_ASV27

CACCGCGGTTATACGAAAGACCCTAGTTTATAGGCACGGCGTAAAGGGTGGTTAGGGGAT

AAACAAAATAAAGCCAAAGACCTTCTAAGTCGTCATACACTCATGAAGGCACGAAGCCCA

AATACGAAAGTAACTTTACAACTTAGACCCGACTCCACGAAAGCTGGGGGA

>175_Sample_ASV28

CACCGCGGTTATACGAGAGACCCTAATTAATAGATACGGCGTAAAGAGTGGTTTAGGACA

AAAATTAATAAAGCCGAAGTTTCTCCTAGCCGTTGTACGCACTCTGAGAACACGAAGCCC

CACTACGAAAGTAGCTTTACCTATTATTTCCTGACCCCACGAAAGCTAAGAAA

>175_Sample_ASV29

CACCGCGGTTATACGAGAGGCCCAAGTTGATAGACATCGGCGTAAAGAGTGGTTAAGGAA

GTCTCAAAACTAAAGCCGAATGCCTTCAAGACTGTTATACGTTTCCGAAGGTAAGAAGCC

CCACTACGAAAGTGGCTTTACTCCACCTGACTCCACGAAAGCTGTGAAA

>176_Sample_ASV1

CACCGCGGTTATACGAAAGACCCTAGTTGATGGGTGCGGCGTAAAGGGTGGTTAGGGACT

ACAAAAAATAGAGCTAAAGATCCTCTAGGTCGTCATACACATCACGAGGAAACGAAGCCC

AAATACGAAAGTAGCTCTAAATATAATCCTGACGCCACGAAAGCCAAGAAA

>176_Sample_ASV2

CACCGCGGTTAAACGAGAGGCCCTAGTTGATGGTGCAACGGCGTAAAGGGTGGTTAAGGA

TAGCGAAATAATAAAGTCAAATGGCCCTCTGGCTGTCATACGCTTCTAGGAGTCCGAAGC

CCAGTATACGAAAGTAGCTTTAAGAAAGTCCACCTGACCCCACGAAAACTGAGGAA

>176_Sample_ASV3

CACCGCGGTTATACGAAAGACCCTAGTTGCTAGCCACGGCGTAAAGGGTGGTTAAGGACA

ACAACAAATAAAGCTAAAGATCCCCTAAGCCGTCATACGCATTCCGGGGGCACGAAGCCC

TAACACGAAAGTAGCTTTAAAAAATATACCTGACCCCACGAAAGCTAAGAAA

>176_Sample_ASV4

CACCGCGGTTATACGAAAGACCCTAGTTGATAGCCACGGCGTAAAGGGTGGTTAAGGAAC

CTAAAAAATAAAGCTAAAGACCCTCTAGGCCGTCATACGCATTACGAGGCAAACGAAACC

CCAACACGAAAGTAGCTTTAGACAAATATCCTGATGCCACGAAAGCCAAGAAA

>176_Sample_ASV5

CACCGCGGTTATACGAGAGGCCCAAGTTGACAGACACCGGCGTAAAGAGTGGCTAGGGAA

AAATTACTACTAAAGCCGAACACCTTCAGAACTGTCATACGTTCCCGAAGATAAGAAGCC

CCACCACGAAAGTGACTTTATATTACCCGACCCCACGAAAGCTGTGAAA

>176_Sample_ASV6

CACCGCGGTTATACGAGAGGCCCAAGTTGACAGACACCGGCGTAAAGAGTGGCTAGGGAA

AAATTACTACTAAAGCCGAACACCTTCAGAACTGTCATACGTTCCCGAAGATAAGAAGCC

CCACCACGAAAGTGGCTTTATATTACCCGACCCCACGAAAGCTGTGAAA

>176_Sample_ASV7

CACCGCGGTTATACGAGAGGCCCAAGTTGACAGACACCGGCGTAAAGAGTGGCTAGGGAA

AAATTACTACTAAAGCCGAACACCTTCAGAACTGTCATACGTTCCCGAAGATAAGAAGCC

CCACCACGAAAGTGGCTTTATATTACCCGACCCCACGAAAGCTAAGAAA

>176_Sample_ASV8

CACCGCGGTTATACGAGAGGCCCAAGTTGATAAGCCCGGCGTAAAGAGTGGTTATGGAGA

ATAAAACACTAAAGCTAAAGACCCCCTAGGCTGTTATACGCACCTGGCGGCTCGAACCAC

CTATACGAAAGTAGCTTTACCCCCCTTCCACCAGAATCCACGACAGCTGGGGCA

>176_Sample_ASV9

CACCGCGGTTATACGAGAGGCCCAAGTTGACAGACATCGGCGTAAAGAGTGGTTAAGGAA

ACCCCATTACTAAAGCCGAACGCCCTCAGAACTGTCATACGTTCCCGAAGGTAAGAAGCC

CCACTACGAAAGTGGCTTTAACCACACCCGACCCCACGAAAGCTGTGAAA

>176_Sample_ASV10

CACCGCGGTTAAACGAGAGGCCCTAGTTGATATTACAACGGCGTAAAGGGTGGTTAAGGA

AAGCAAGCAATAAAGCCGAATGGCCCTTTGGCTGTCATACGCTTCTAGGTGTCCGAAGCC

CAATATACGAAAGTAGCTTTAGAAAAGCCCACCTGACACCACGAAAGCTGAGGAA

>176_Sample_ASV11

CACCGCGGTTATACGAGAGACCCCAGTTGATAGCTACGGCGTAAAGAGTGGTTAAGGACC

CCATTTAATAAAGCCAAAGACCCCCCAAGCCGTCATACGCACACGAGGGCGCGAAGCTCG

CACACGAAAGTAGCTCTAATTTACCCGACGCCACGAAAGCTAAGGAA

>176_Sample_ASV12

CACCGCGGTTATACGAGAAACCCTAGTTGACAAATACGGCGTAAAGAGTGGTTAAGGGGT

ATCAAAAATAAAGTCAAACATTTTCCTAGTTGTTATAAGCATTTCGAGAACATGAAGCCC

AACCACGAAAGTAACTTTACCTTCACCTGACCCCACGAAAGCTAAGAAA

>176_Sample_ASV13

CACCGCGGTTATACGAGAGACCCTAGTTGATAAATACGGCGTAAAGAGTGGTTATGGGAA

AAACAAATAAAGTCAAACAACCTCTTAGCTGTTATACGCATTATGAGAGCATGAAGCCCC

CTCACGAAAGTAACTTTAATACCTCCTGACCCCACGAAAGCTAAGAAA

>176_Sample_ASV14

CACCGCGGTTATACGAGAGGCTCAAGTTGATAAACCCCGGCGTAAAGAGTGGTTAAGGAA

AAATAGAAACTAAAGCCGAATGCTTTCAAGACTGTTATACGTTCCCGAGAGTAAGAAGAC

CAATTACGAAAGTGGCTTTACTTTACCTGACCCCACGAAAGCTACGACA

>176_Sample_ASV15

CACCGCGGTTATACGAGAGACCCTAATTGATAGACACGGCGTAAAGAGTGGTTTGGGAAA

GCCCAAAAATAAAGCTAAAGACCTTCTAAGCCGTCACACGCACGCCGAAGGCACGAAGCC

CTGACACGAAAGTAGCTTTACTATTACCCCCGACGCCACGAAAGCTAAGAAA

>176_Sample_ASV16

CACCGCGGTTATACGAAAGACCCTAGTTTATAGATGCGGCGTAAAGGGTGGTTAAGGAAA

ACCTAAAATAATGCTAAAGACTCTCAAAGCTGTTATACGCTTCATGAGCACACGAAATCC

AAATACGAAAGTTGCCTTAATAAACTTTGCCTGACCCCACGAAAGCTGAAAAA

>176_Sample_ASV17

CACCGCGGTTATACGAGAGACCCTAGTTGATAGCTACGGCGTAAAGCGTGGTTAGGAGAA

CAAGATAAATAAAGTCAAACAATCTCTCGGCCGTTATACGTTATCTGAGAATATGAAGTC

CTACCACGAAAGTAACTTTAATTTTTCTGACCCCACGAAAACTGAGGAA

>176_Sample_ASV18

CACCGCGGTTAAACGAGAGGCCCTAGTTGATAGTACAACGGCGTAAAGGGTGGTTAAGGA

TAGTGAAATAATAAAGTCGAATGGCCCTTTGGCTGTCATACGCTTCTAGGAGTCCGAAGC

CCAATATACGAAAGTAACTTTAAGAGCGCCCACCTGACCCCACGAAAACTGAGAAA

>176_Sample_ASV19

CACCGCGGTTATACGAGAGACCCTAATTGATAGGCGCGGCGTAAAGAGTGGTTTAGGACT

AAAACTAATAAAGCTAAAGATCCTCCCAGCCGTCGCACGCATTCTGAGGACACGAAACTC

AACCACGAAAGTAGCTTTAACATACTCTCCTGACCCCACGAAAGCTAAGAAA

>176_Sample_ASV20

CACCGCGGTTATACGAAAGACCCTAGTTGATAGGCACGGCGTAAAGGGTGGTTAGGGATA

TATTAAAAATAAAGTTAAAGAGCCTCTAAGCTGTCGCACGCATCCCGAGAGCTCGAGACC

CAGACACGAAAGTAACTTTAAAATACCTCACCTGACCCCACGAAAGCTAAGAAA

>176_Sample_ASV21

CACCGCGGTTATACGAGAGGCCCAAGTTGATAGTCTTCGGCGTAAAGAGTGGTTAAAGAA

GCAATTAGACTAAGGCTAAACTCTTCCAAGGCTGTCATACGCACCCGGAAGCATGAAACC

CAACCACGAAAGTGGCCTTATTTACCTTGACCCCACGAAAGCTGTGAAA

>176_Sample_ASV22

CACCGCGGTTATACGAGAGACCCCAGTTGATAAACACGGCGTAAAGAGTGGTTAAGGGCA

CAAAACAAATAAAGTCAAACAACCTCTTAGCTGTTATACGCATTATGAGGATATGAAGCC

CCCCTACGAAAGTAACTTTACCAACCCCTGACCCCACGAAAGCTAAGAAA

>176_Sample_ASV23

CACCGCGGTTATACGAGAGACCCTAGTTGATATATACGGCGTAAAGAGTGGTTTGGAATA

ACCAAGTAATATAGCCAAAGACCTCCCAAGCCGTCACACGCACCCCGGAGGCACGAAGCC

CAAACACGAAAGTAGCTTTATTAACTTACCGACGCCACGAAAGCTAAGAAA

>176_Sample_ASV24

CACCGCGGTTATACGAAAGACCCTAGTTTATAGGCACGGCGTAAAGGGTGGTTAGGGGAT

AAACAAAATAAAGCCAAAGACCTTCTAAGTCGTCATACACTCATGAAGGCACGAAGCCCA

AATACGAAAGTAACTTTACAACTTAGACCCGACTCCACGAAAGCTGGGGGA

>176_Sample_ASV25

CACCGCGGTTATACGAGAGGCCCAAGTTGACAAACTCCGGCGTAAAGCGTGGTTAAGGCA

CACTCAAAAACTAAAGCCGAATATCTTCAGAGCAGTCATACGCATCCGAAGACACGAAGC

CCCACCACGAAAGTGGCTTTATGACACCTGACCCCACGAAAGCTAGGACA

>176_Sample_ASV26

CACCGCGGTTATACGAGAGACCCTAATTGATAGACTACGGCGTAAAGAGTGGTTTAGAAC

AAAAATTAATAAAGCCAAAGATCCTCCTGGCCGTCGCACGCATTTCGAGGATACGAAGCC

CCACTACGAAAGTAGCTTTACTACAACTTTTCTGACCCCACGAAAGCTAAGAAA

>176_Sample_ASV27

CACCGCGGTTATACGAAAGACCCTAGTTGATAGATCACGGCGTAAAGGGTGGTTAAGGAG

AACAAAAATAAAGCTAAAAATCCTCTAAGCTGTCATACGCTTTCCGAAGACATGAGACCC

AACCACGAAAGTAACTTTAAACTTTCCTGACACCACGAAAGCCAAGAAA

>177_Sample_ASV1

CACCGCGGTTATACGAGAGGCCCAAGTTGACAGACATCGGCGTAAAGAGTGGTTAAGGAA

AACCTTCAACTAAAGTCGAACGCTCTCAAGACTGTCGTACGTTTCCGAGAGTATGAAGCC

CCACCACGAAAGTGACTTTACCTCACCTGACTCCACGAAAGCTGCGAAA

>177_Sample_ASV2

CACCGCGGTTATACGAAAGACCCTAGTTGATGGGTGCGGCGTAAAGGGTGGTTAGGGACT

ACAAAAAATAGAGCTAAAGATCCTCTAGGTCGTCATACACATCACGAGGAAACGAAGCCC

AAATACGAAAGTAGCTCTAAATATAATCCTGACGCCACGAAAGCTGGGAAA

>177_Sample_ASV3

CACCGCGGTTATACGAAAGACCCTAGTTGATGGGTGCGGCGTAAAGGGTGGTTAGGGACT

ACAAAAAATAGAGCTAAAGATCCTCTAGGTCGTCATACACATCACGAGGAAACGAAGCCC

AAATACGAAAGTAGCTCTAAATATAATCCTGACGCCACGAAAGCCAAGAAA

>177_Sample_ASV4

CACCGCGGTTATACGAAAGACCCTAGTTGATAACTACGGCATAAAGGGTGGTTAGGGAGT

TTAAAAAATAAAGCCAAAGCTCTTCTAAGCTGTCATACGCTCTCTGAAAGAACGAGGCCC

AAACACGAAAGTAGCTTTAAATAAATAACCTGATACCACGAAAGCCAAGAAA

>177_Sample_ASV5

CACCGCGGTTATACGAGAGACCCTAGTTGATTAAAGCGGCGTAAAGAGTGGTTATGGAAC

TATTCTTTTAAAGCAGAAAACCTCTCAAACTGTTATACGCACCCAGAGGTCTGAATCCCT

CACACGAAAGTGACTTTATTTATGCCTACCAGAACCCACGAAAGCTGGGACA

>177_Sample_ASV6

CACCGCGGTTATACGAGAGGCCCAAGTTGACAGACACCGGCGTAAAGAGTGGCTAGGGAA

AAATTACTACTAAAGCCGAACACCTTCAGAACTGTCATACGTTCCCGAAGATAAGAAGCC

CCACCACGAAAGTGGCTTTATATTACCCGACCCCACGAAAGCTGTGAAA

>177_Sample_ASV7

CACCGCGATTATACGAGAGGCCCAAGTTGACAGACATCGGCGTAAAGAGTGGTTAAGGAA

AAATTACTACTAAAGCCGAACACCTTCAGAACTGTCATACGTTCCCGAAGATAAGAAGCC

CCACCACGAAAGTGGCTTTATATTACCCGACCCCACGAAAGCTGTGAAA

>177_Sample_ASV8

CACCGCGGTTATACGAAAGACCCTAGTTTATAGAAACGGCGTAAAGGGTGGTTAAGGGAC

AAACAAAATAAGGCCAAAGACTTCCTAAGCCGTCATACGCTCCACGAAAGCACGAAGCCC

AAACACGAAAGTAGCCTTAATAACTTCACCTGACCCCACGAAAGCTGGGAGA

>177_Sample_ASV9

CACCGCGGTTATACGAGAGGCCCAAGTTGATAAGCCCGGCGTAAAGAGTGGTTATGGAGA

ATAAAACACTAAAGCTAAAGACCCCCTAGGCTGTTATACGCACCTGGCGGCTCGAACCAC

CTATACGAAAGTAGCTTTACCCCCCTTCCACCAGAATCCACGACAGCTGGGGCA

>177_Sample_ASV10

CACCGCGGTTATACGAGAGGCCCTAGTTGATAGCCACGGCGCAAAGAGTGGTTAAGGAGC

CCCACTAAATAAAGTCGAACACTTCCTAGGCCGTTATACGTTTTCTAGAAGCACGAAACC

CAATTACACGAAAGCAACTTTATACTAAACAACCTGACCCCACGAAAGCTAAGAAA

>177_Sample_ASV11

CACCGCGGTTATACGAGAGGCCCAAGTTGACAGACATCGGCGTAAAGAGTGGTTAAGGAA

ACCCCATTACTAAAGCCGAACGCCCTCAGAACTGTCATACGTTCCCGAAGGTAAGAAGCC

CCACTACGAAAGTGGCTTTAACCACACCCGACCCCACGAAAGCTGTGAAA

>177_Sample_ASV12

CACCGCGGTTATACGAGAGGCCCAAGTTGACAGACACCGGCGTAAAGAGTGGTTAAGGAA

ATTCATCAACTAAAGCCGAATGCCCTCAGGACTGTTATACGTTTGCGAAGGTAAGAAGCC

CTACTACGAAAGTGGCTTTATTCCACCTGATTCCACGAAAGCTGCGGAA

>177_Sample_ASV13

CACCGCGGTTATACGAGAGGCCCCAGTTGATATTAGCGGCGTAAAGGGTGGTTAAGGACT

TTAATAACTAAAGCTAAACACTTCCCCGGCTGTTGTACGTAGACCGGAAGCTTGAAGCCC

AGACACGAAAGTAGCTTTATTCCCCCTGACCCCACGAAAGCTGAGAAA

>177_Sample_ASV14

CACCGCGGTCACACGATTAACCCAAGTCAATAGAAGCCGGCGTAAAGAGTGTTTTAGATC

ACCCCCTCCCCAATAAAGCTAAAACTCACCTGAGTTGTAAAAAACTCCAGTTGACACAAA

ATAGACTACGAAAGTGGCTTTAACATATCTGAACACACAATAGCTAAGACC

>177_Sample_ASV15

CACCGCGGTTATACGAGAAACCCTAGTTGACAAATACGGCGTAAAGAGTGGTTAAGGGGT

ATCAAAAATAAAGTCAAACATTTTCCTAGTTGTTATAAGCATTTCGAGAACATGAAGCCC

AACCACGAAAGTAACTTTACCTTCACCTGACCCCACGAAAGCTAAGAAA

>177_Sample_ASV16

CACCGCGGTTATACGAGAGACCCTAGTTGATAAATACGGCGTAAAGAGTGGTTATGGGAA

AAACAAATAAAGTCAAACAACCTCTTAGCTGTTATACGCATTATGAGAGCATGAAGCCCC

CTCACGAAAGTAACTTTAATACCTCCTGACCCCACGAAAGCTAAGAAA

>177_Sample_ASV17

CACCGCGGTTATACGAGAGACCCTAATTGATAGGTACGGCGTAAAGAGTGGTTTGGGATA

ACCCAAAAATAAAGCTAAAGACCTTCTAAGCCGTCACACGCATACCGAAGGCACGAGGCC

CCAACACGAAAGTAGCTTTACTATTACCCCGACGCCACGAAAGCTAAGAAA

>177_Sample_ASV18

CACCGCGGTTATACGAGAGGCCCTAGTTGATAACTACCGGCGTAAAGAGTGGTTATGGAA

AAATATTTAATAAAGCCGAACACCCCCTCAGCCGTCATACGCACCTGGGAGCACGAAGAC

CTACTGCGAAAGCAGCTTTAATTATGCCTGACCCCACGACAGCTAAGAAA

>177_Sample_ASV19

CACCGCGGTTATACGAGAGGCCCAAGTTGATACTCCACGGCGTAAAGAGTGGTTAGAAAA

GACCTACTACTAAAGCCGAACGCCTTCAAAGCTGTTATACGCATCCGAAGGTGAGAAGCC

CACCCACGAAAGTGGCTTTACAACCTTGACTCCACGAAAGCTATGACA

>177_Sample_ASV20

CACCGCGGTTATACGAGAGACCCCAGTTGATAAACACGGCGTAAAGAGTGGTTAAGGGCA

CAAAACAAATAAAGTCAAACAACCTCTTAGCTGTTATACGCATTATGAGGATATGAAGCC

CCCCTACGAAAGTAACTTTACCAACCCCTGACCCCACGAAAGCTAAGAAA

>177_Sample_ASV21

CACCGCGGTTATACGAGAGGCCCAAGTTGATAGACATCGGCGTAAAGAGTGGTTAAGGAA

GTCTCAAAACTAAAGCCGAATGCCTTCAAGACTGTTATACGTTTCCGAAGGTAAGAAGCC

CCACTACGAAAGTGGCTTTACTCCACCTGACTCCACGAAAGCTGTGAAA

>177_Sample_ASV22

CACCGCGGTTATACGAGAGACTCCAGTTGATAGCTACGGCGTAAAGAGTGGTTTGGGGCC

CGCCCAAAATAAAGCCAAAGACCTCCCAAGCCGTCAAACGCACCCCGGAGGCACGAAGTC

CTAACGCGAAAGCAACTTTACCTTCCCCGACGCCACGAAAGCTAAGAAA

>177_Sample_ASV23

CACCGCGGTTATACGAAAGACCCTAGTTGATAGCTACGGCGTAAAGGGTGGTTAAGGTAT

ATAACAAATAAAGCTAAAGAGCCTCTAAGCCGTCGTACGCATTCTGAGAGCTCGAAACCC

TAACACGAAAGTAGCTTTAAAAAATAACACCTGACCCCACGAAAGCTAAGAAA

>177_Sample_ASV24

CACCGCGGTTATACGAAAGACCCTAGTTGATAGCTACGGCGTAAAGGGTGGTTAAGGTAT

ATAACAAATAAAGCTAAAGAGCCTCCAAGCCGTCGTACGCATTCTGAGAGCTCGAAACCC

TAACACGAAAGTAGCTTTAAAAAATAACACCTGACCCCACGAAAGCTAAGAAA

>177_Sample_ASV25

CGCCGCGGTTATACGAAAGACCCTAGTTGATAGCTACGGCGTAAAGGGTGGTTAAGGTAT

ATAACAAATAAAGCTAAAGAGCCTCTAAGCCGTCGTACGCATTCTGAGAGCTCGAAACCC

TAACACGAAAGTAGCTTTAAAAAATAACACCTGACCCCACGAAAGCTAAGAAA

>177_Sample_ASV26

CACCGCGGTTATACGAAAGACCCTAGTTGATAGCTACGGCGTAAAGGGTGGTTAAGGTAT

ATAACAAATAAAGCTAAAGAGCCTCCAAGCCGTCGCACGCATTCTGAGAGCTCGAAACCC

TAACACGAAAGTAGCTTTAAAAAATAACACCTGACCCCACGAAAGCTAAGAAA

>177_Sample_ASV27

CACCGCGGTTATACGAAAGACCCTAGTTGATAGATCACGGCGTAAAGGGTGGTTAAGGAG

AACAAAAATAAAGCTAAAAATCCTCTAAGCTGTCATACGCTTTCCGAAGACATGAGACCC

AACCACGAAAGTAACTTTAAACTTTCCTGACACCACGAAAGCCAAGAAA

>178_Sample_ASV1

CACCGCGGTTATACGAAAGACCCTAGTTGATGGGTGCGGCGTAAAGGGTGGTTAGGGACT

ACAAAAAATAGAGCTAAAGATCCTCTAGGTCGTCATACACATCACGAGGAAACGAAGCCC

AAATACGAAAGTAGCTCTAAATATAATCCTGACGCCACGAAAGCCAAGAAA

>178_Sample_ASV2

CACCGCGGTTATACGAGAGACCCTAGTTGATTAAAGCGGCGTAAAGAGTGGTTATGGAAC

TATTCTTTTAAAGCAGAAAACCTCTCAAACTGTTATACGCACCCAGAGGTCTGAATCCCT

CACACGAAAGTGACTTTATTTATGCCTACCAGAACCCACGAAAGCCAAGAAA

>178_Sample_ASV3

CACCGCGGTTATACGAGAGACCCTAGTTGATTAAAGCGGCGTAAAGAGTGGTTATGGAAC

TATTCTTTTAAAGCAGAAAACCTCTCAAACTGTTATACGCACCCAGAGGTCTGAATCCCT

CACACGAAAGTGACTTTATTTATGCCTACCAGAACCCACGAAAGCTGGGACA

>178_Sample_ASV4

CACCGCGGTTATACGAGAGGCCCAAGTTGACAGACACCGGCGTAAAGAGTGGCTAGGGAA

AAATTACTACTAAAGCCGAACACCTTCAGAACTGTCATACGTTCCCGAAGATAAGAAGCC

CCACCACGAAAGTGGCTTTATATTACCCGACCCCACGAAAGCTGTGAAA

>178_Sample_ASV5

CACCGCGGTTATACGAGAGGCCCAAGTTGACAGACATCGGCGTAAAGAGTGGTTAAGGAA

AAATTACTGCTAAAGCCGAACACCCTCAGAACTGTCATACGTTCCCGAAGATAAGAAGCC

CCACCACGAAAGTGGCTTTATATTACCCGACCCCACGAAAGCTGTGAAA

>178_Sample_ASV6

CACCGCGGTTATACGAGAGGCCCAAGTTGACAGACACCGGCGTAAAGAGTGGCTAAGGAA

AAATTTCTACTAAAGCCGAACACCTTCAGAACTGTCATACGTTCCCGAAGAAAAGAAGCC

CCACCACGAAAGTGGCTTTATATTACCCGACCCCACGAAAGCTGTGAAG

>178_Sample_ASV7

CACCGCGGTTATACGAGAGGCCCAAGTTGACAGACACCGGCGTAAAGAGTGGCTAAGGAA

AAATTTCTACTAAAGCCGAACACCTTCAGAACTGTCATACGTTCCCGAAGAAAAGAAGCC

CCACCACGAAAGTGGCTTTATATTACCCGACCCCACGAAAGCTGTGAAA

>178_Sample_ASV8

CACCGCGGTTATACGAGAGGCCCAAGTTGATAAGCCCGGCGTAAAGAGTGGTTATGGAGA

ATAAAACACTAAAGCTAAAGACCCCCTAGGCTGTTATACGCACCTGGCGGCTCGAACCAC

CTATACGAAAGTAGCTTTACCCCCCTTCCACCAGAATCCACGACAGCTGGGGCA

>178_Sample_ASV9

CACCGCGGTTATACGAGAGGCCCTAGTTGATAGCCACGGCGCAAAGAGTGGTTAAGGAGC

CCCACTAAATAAAGTCGAACACTTCCTAGGCCGTTATACGTTTTCTAGAAGCACGAAACC

CAATTACACGAAAGCAACTTTATACTAAACAACCTGACCCCACGAAAGCTAAGAAA

>178_Sample_ASV10

CACCGCGGTTATACGAGAGGCCCAAGTTGACAGACATCGGCGTAAAGAGTGGTTAAGGAA

ACCCCATTACTAAAGCCGAACGCCCTCAGAACTGTCATACGTTCCCGAAGGTAAGAAGCC

CCACTACGAAAGTGGCTTTAACCACACCCGACCCCACGAAAGCTGTGAAA

>178_Sample_ASV11

CACCGCGGTTATACGAGAGGCCCAAGTTGATAGATATCGGCGTAAAGAGTGGTTAAGGAA

ACCCCGTTACTAAAGCCGAACGCCCTCAGAACTGTCATACGTTCCCGAAGGTAAGAAGCC

CCACTACGAAAGTGGCTTTAACCACACCCGACCCCACGAAAGCTGTGAAA

>178_Sample_ASV12

CACCGCGGTCACACGATTAACCCAAGTCAATAGAAGCCGGCGTAAAGAGTGTTTTAGATC

ACCCCCTCCCCAATAAAGCTAAAACTCACCTGAGTTGTAAAAAACTCCAGTTGACACAAA

ATAGGCTACGAAAGTGGCTTTAACATATCTGAACACACAATAGCTAAGACC

>178_Sample_ASV13

CACCGCGGTTATACGAAAGACCCCAGTTTATAGACACGGCGTAAAGGGTGGTTATAGGAC

AAACTAACATAGAGCCAAAGACCCCCTAAGCCGTCATACGCTCACGAGGGCTCGAAGCCC

CTATACGAAAGTAGCTCTACCAAAAAATGCTCGACTCCACGAAAGCTGGGACA

>178_Sample_ASV14

CACCGCGGTTATACGAGAGACCCTAGTTGATATATACGGCGTAAAGAGTGGTTATGGGAA

AAACAAATAAAGTCAAACAACCTCTTAGCTGTTATACGCATTATGAGAGCATGAAGCCCC

CTCACGAAAGTAACTTTAATACCTCCTGACCCCACGAAAGCTAAGAAA

>178_Sample_ASV15

CACCGCGGTTATACGAGAGACCCTAGTTGATAAATACGGCGTAAAGAGTGGTTATGGGAA

AAACAAATAAAGTCAAACAACCTCTTAGCTGTTATACGCATTATGAGAGCATGAAGCCCC

CTCACGAAAGTAACTTTAATACCTCCTGACCCCACGAAAGCTAAGAAA

>178_Sample_ASV16

CACCGCGGTTATACGAAAGACCCTAGTTGATGGATCACGGCGTAAAGGGTGGTTAAGGGA

TATAAAAATAAAGCTAAAAATCCTCTTAGCTGTCATACGCTTTCCGAAGACATGAGGCCC

AATTACGAAAGTAGCTTTAAACCACCCTGACACCACGAAAGCCAAGAAA

>178_Sample_ASV17

CACCGCGGTTATACGAGAGGCCCTAGTTGATAACTACCGGCGTAAAGAGTGGTTATGGAA

AAATATTTAATAAAGCCGAACACCCCCTCAGCCGTCATACGCACCTGGGAGCACGAAGAC

CTACTGCGAAAGCAGCTTTAATTATGCCTGACCCCACGACAGCTAAGAAA

>178_Sample_ASV18

CACCGCGGTTATACGAGAGGCCCAAGTTGATTATTACCGGCGTAAAGCGTGGTTAAGATT

TTTATAGGACTAAAGCCGAACCCCCTCAGAGCTGTTATACGCACCCGAAGGTTAGAAGTT

CAATCACGAAAGTGGCTTTACAACCCCGAACCCACGAAAGCTAAGATA

>178_Sample_ASV19

CACCGCGGTTATACGAGAGACCCTAATTGATAGGCGCGGCGTAAAGAGTGGTTTAGGACT

AAAACTAATAAAGCTAAAGATCCTCCCAGCCGTCGCACGCATTCTGAGGACACGAAACTC

AACCACGAAAGTAGCTTTAACATACTCTCCTGACCCCACGAAAGCTAAGAAA

>178_Sample_ASV20

CACCGCGGTTATACGAAAGACCCTAGTTGATAGGCACGGCGTAAAGGGTGGTTAGGGATA

TATCAAAAATAAAGTTAAAGAGCCTCTAAGCTGTCGCACGCATCCCGAGAGCTCGAGACC

CAGACACGAAAGTAACTTTAAAATACCTCACCTGACCCCACGAAAGCTAAGAAA

>178_Sample_ASV21

CACCGCGGTTATACGAGAGACCCCAGTTGATAAACACGGCGTAAAGAGTGGTTAAGGGCA

CAAAACAAATAAAGTCAAACAACCTCTTAGCTGTTATACGCATTATGAGGATATGAAGCC

CCCCTACGAAAGTAACTTTACCAACCCCTGACCCCACGAAAGCTAAGAAA

>178_Sample_ASV22

CACCGCGGTTATACGAAAGACCCTAGTTTATAGGCACGGCGTAAAGGGTGGTTAGGGGAT

AAACAAAATAAAGCCAAAGACCTTCTAAGTCGTCATACACTCATGAAGGCACGAAGCCCA

AATACGAAAGTAACTTTACAACTTAGACCCGACTCCACGAAAGCTGGGGGA

>178_Sample_ASV23

CACCGCGGTTATACGAGAGACCCTAATTAATAGCTACGGCGTAAAGAGTGGTTTAGGACA

AAAATTAATAAAGCCGAAGTTTCTCCTAGCCGTTGTACGCACTCTGAGAACACGAAGCCC

CACTACGAAAGTAGCTTTACCAATAATCTCCTGACCCCACGAAAGCTAAGAAA

>178_Sample_ASV24

CACCGCGGTTATACGAGAGACCCTAATTAATAGATACGGCGTAAAGAGTGGTTTAGGACA

AAAATTAATAAAGCCGAAGTTTCTCCTAGCCGTTGTACGCACTCTGAGAACACGAAGCCC

CACTACGAAAGTAGCTTTACCAATAATCTCCTGACCCCACGAAAGCTAAGAAA

>178_Sample_ASV25

CACCGCGGTTATACGAGAGACCCTAATTAATAGCTACGGCGTAAAGAGTGGTTTAGGGTA

AAAATTAATAAAGCCGAAGTTTCTCCTAGCCGTTGTACGCACTCTGAGAACACGAAGCCC

CACTACGAAAGTAGCTTTACCTATTATTTCCTGACCCCACGAAAGCTAAGAAA

>178_Sample_ASV26

CACCGCGGTTATACGAGAGACCCTAATTAATAGATACGGCGTAAAGAGTGGTTTAGGACA

AAAATTAATAAAGCCGAAGTTTCTCCTAGCCGTTGTACGCACTCTGAGAACACGAAGCCC

CACTACGAAAGTAGCTTTACCTATTATTTCCTGACCCCACGAAAGCTAAGAAA

>178_Sample_ASV27

CACCGCGGTTATACGAGAGACTCCAGTTGATAGCTACGGCGTAAAGAGTGGTTTGGGGCC

CGCCCAAAATAAAGCCAAAGACCTCCCAAGCCGTCAAACGCACCCCGGAGGCACGAAGTC

CTAACGCGAAAGCAACTTTACCTTCCCCGACGCCACGAAAGCTAAGAAA

>178_Sample_ASV28

CACCGCGGTTATACGAAAGACCCTAGTTGATAGATCACGGCGTAAAGGGTGGTTAAGGAA

AACAAAAATAAAGCTAAAAATCCTCTAAGCTGTCATACGCTTTCCGAAGACATGAGATCC

AACCACGAAAGTAGCTTTAAAATTTCCTGACACCACGAAAGCCAAGGAA

>181_Sample_ASV1

CACCGCGGTTATACGAAAGACCCAAGTTGATAAACACCGGCGTAAAGCGTGGTTAAGGAA

CAACACATAATTAAAGCCAAAGACCTCCTAGGCCGTTATACGCACTACGGAGACATGAAG

CCCAAACACGAAAGTAACTTTATTCTGCCCGACCCCACGAAAGCTAAGAAA

>181_Sample_ASV2

CACCGCGGTTATACGAAAGACCCTAGTTGATGGGTGCGGCGTAAAGGGTGGTTAGGGACT

ACAAAAAATAGAGCTAAAGATCCTCTAGGTCGTCATACACATCACGAGGAAACGAAGCCC

AAATACGAAAGTAGCTCTAAATATAATCCTGACGCCACGAAAGCCAAGAAA

>181_Sample_ASV3

CACCGCGGTTATACGAGAGACCCTAGTTGATTAAAGCGGCGTAAAGAGTGGTTATGGAAC

TATTCTTTTAAAGCAGAAAACCTCTCAAACTGTTATACGCACCCAGAGGTCTGAATCCCT

CACACGAAAGTGACTTTATTTATGCCTACCAGAACCCACGAAAGCTGGGACA

>181_Sample_ASV4

CACCGCGGTTATACGAAAGACCCTAGTTTATAGGTACGGCGTAAAGGGTGGTTAGGGGAC

AAACAAAATAAAGCCAAAGACCTCCTAAGCCGTCATACGCTCACGGAAGCACGAAGCCCA

AACACGAAAGTAGCTTTACCGAACATGCCCGACTCCACGAAAGCTGGGGAA

>181_Sample_ASV5

CACCGCGGTTATACGAAAGACCCTAGTTGATAGCCACGGCGTAAAGGGTGGTTAAGGAAC

CTAAAAAATAAAGCTAAAGACCCTCTAGGCCGTCATACGCATTACGAGGCAAACGAAACC

CCAACACGAAAGTAGCTTTAGACAAATATCCTGATGCCACGAAAGCCAAGAAA

>181_Sample_ASV6

CACCGCGGTTATACGAGAGACCCTAGTTGACAGACACCGGCGTAAAGAGTGGCTAGGGAA

AAATTACTACTAAAGCCGAACACCTTCAGAACTGTCATACGTTCCCGAAGATAAGAAGCC

CCACCACGAAAGTGGCTTTATATTACCCGACCCCACGAAAGCTAAGAAA

>181_Sample_ASV7

CACCGCGGTTATACGAGAGGCCCAAGTTGACAGACACCGGCGTAAAGAGTGGCTAGGGAA

AAATTACTACTAAAGCCGAACACCTTCAGAACTGTCATACGTTCCCGAAGATAAGAAGCC

CCACCACGAAAGTGGCTTTATATTACCCGACCCCACGAAAGCTAAGAAA

>181_Sample_ASV8

CACCGCGGTTATACGAGAGGCCCAAGTTGACAGACACCGGCGTAAAGAGTGGCTAGGGAA

AAATTACTACTAAAGCCGAACACCTTCAGAACTGTCATACGTTCCCGAAGATAAGAAGCC

CCACCACGAAAGTGGCTTTATATTACCCGACCCCACGAAAGCTGTGAAA

>181_Sample_ASV9

CACCGCGGTTATACGAGAGACCCTAGTTGACAGACACCGGCGTAAAGAGTGGCTAAGGAA

AAATTTCTACTAAAGCCGAACACCTTCAGAACTGTCATACGTTCCCGAAGAAAAGAAGCC

CCACCACGAAAGTGGCTTTATATTACCCGACCCCACGAAAGCTGTGAAA

>181_Sample_ASV10

CACCGCGGTTATACGAGAGGCCCAAGTTGACAGACACCGGCGTAAAGAGTGGCTAAGGAA

AAATTTCTACTAAAGCCGAACACCTTCAGAACTGTCATACGTTCCCGAAGAAAAGAAGCC

CCACCACGAAAGTGGCTTTATATTACCCGACCCCACGAAAGCTGTGAAA

>181_Sample_ASV11

CACCGCGGTTATACGAGAGGCCCAAGTTGACAGACACCGGCGTAAAGAGTGGCTAAGGAA

AAATTTCTACTAAAGCCGAACACCTTCAGAACTGTCATACGTTCCCGAAGAAAAGAAGCC

CCACCACGAAAGTGGCTTTATATTACCCGACCCCACGAAAGCTAAGAAA

>181_Sample_ASV12

CACCGCGGTTATACGAGAGGCTCAAGTTGACAGACATCGGCGTAAAGAGTGGTTAGGAAG

TTTTTGAAACTAAAGCCGAACGCCCTCAGAACTGTTATACGTACCCGAGGGTAAGAAGCC

CCACTACGAAAGTGGCTTTATATCTCCGACCCCACGAAAGCTGCGAAA

>181_Sample_ASV13

CACCGCGGTTAAACGAGAGACCCTAGTTGATCATCACGGCGTAAAGAGTGGTTAGGGGAT

TATCATAAATAAAGCCAAAGACCTCCCAAGCTGTCGCACGCATTCCGGAGGCGCGAAGCC

CACACACGAAAGTAGCTTTAACTATTGACCCTGATGCCACGAAAGCTAAGAAA

>181_Sample_ASV14

CACCGCGGTTATACGAGAGACCCTAGTTGATATATACGGCGTAAAGAGTGGTTTGGGATA

TTTCTCCAAAATAAAGCCGAAGATCTCCCAAGCCGTCACACGCATCCCGGAGACACGAAG

CCCTGATACGAAGGTAGCTTTATTAACACCCCGACGCCACGAAAGCTAAGAAA

>181_Sample_ASV15

CACCGCGGTTATACGAGAGACCCTAGTTGATATATACGGCGTAAAGAGTGGTTTGGGATA

TTTCTCCAAAATAAAGTCGAAGATCTCCCAAGCCGTCACACGCATCCCGGAGACACGAAG

CCCTGATACGAAGGTAGCTTTATTAACACCCCGACGCCACGAAAGCTAAGAAA

>181_Sample_ASV16

CACCGCGGTTATACGAGAGGCCCAAGTTGATAAGCCCGGCGTAAAGAGTGGTTATGGAGA

ATAAAACACTAAAGCTAAAGACCCCCTAGGCTGTTATACGCACCTGGCGGCTCGAACCAC

CTATACGAAAGTAGCTTTACCCCCCTTCCACCAGAATCCACGACAGCTGGGGCA

>181_Sample_ASV17

CACCGCGGTTATACGAGAGGCCCAAGTTGACAGACATCGGCGTAAAGAGTGGTTAAGGAA

ACCCCATTACTAAAGCCGAACGCCCTCAGAACTGTCATACGTTCCCGAAGGTAAGAAGCC

CCACTACGAAAGTGGCTTTAACCACACCCGACCCCACGAAAGCTGTGAAA

>181_Sample_ASV18

CACCGCGGTTATACGAGAGACCCCAGTTGATAGCTACGGCGTAAAGAGTGGTTAAGGACC

CCATTTAATAAAGCCAAAGACCCCCCAAGCCGTCATACGCACACGAGGGCGCGAAGCTCG

CACACGAAAGTAGCTCTAATTTACCCGACGCCACGAAAGCTAAGGAA

>181_Sample_ASV19

CACCGCGGTTATACGAGAGACCCTAGTTGATAAATACGGCGTAAAGAGTGGTTATGGGAA

AAACAAATAAAGTCAAACAACCTCTTAGCTGTTATACGCATTATGAGAGCATGAAGCCCC

CTCACGAAAGTAACTTTAATACCTCCTGACCCCACGAAAGCTAAGAAA

>181_Sample_ASV20

CACCGCGGTTATACGAGAGGCTCAAGTTGATAAACCCCGGCGTAAAGAGTGGTTAAGGAA

AAATAGAAACTAAAGCCGAATGCTTTCAAGACTGTTATACGTTCCCGAGAGTAAGAAGAC

CAATTACGAAAGTGGCTTTACTTTACCTGACCCCACGAAAGCTACGACA

>181_Sample_ASV21

CACCGCGGTTATACGAGAGACCCTAATTGATAGACACGGCGTAAAGAGTGGTTTGGGAAA

GCCCAAAAATAAAGCTAAAGACCTTCTAAGCCGTCACACGCACGCCGAAGGCACGAAGCC

CTGACACGAAAGTAGCTTTACTATTACCCCCGACGCCACGAAAGCTAAGAAA

>181_Sample_ASV22

CACCGCGGTTATACGAAAGACCCTAGTTTATAGATGCGGCGTAAAGGGTGGTTAAGGAAA

ACCTAAAATAATGCTAAAGACTCTCAAAGCTGTTATACGCTTCATGAGCACACGAAATCC

AAATACGAAAGTTGCCTTAATAAACTTTGCCTGACCCCACGAAAGCTGAAAAA

>181_Sample_ASV23

CACCGCGGTTATACGAGAGACCCTAGTTGATAGCTACGGCGTAAAGCGTGGTTAGGAGAA

CAAGATAAATAAAGTCAAACAATCTCTCGGCCGTTATACGTTATCTGAGAATATGAAGTC

CTACCACGAAAGTAACTTTAATTTTTCTGACCCCACGAAAACTGAGGAA

>181_Sample_ASV24

CACCGCGGTTAAACGAGAGGCCCTAGTTGATAGTACAACGGCGTAAAGGGTGGTTAAGGA

TAGTGAAATAATAAAGTCGAATGGCCCTTTGGCTGTCATACGCTTCTAGGAGTCCGAAGC

CCAATATACGAAAGTAACTTTAAGAGCGCCCACCTGACCCCACGAAAACTGAGAAA

>181_Sample_ASV25

CACCGCGGTTATACGAAAGACCCTAGTTGATAGGCACGGCGTAAAGGGTGGTTAGGGATA

TATTAAAAATAAAGTTAAAGAGCCTCTAAGCTGTCGCACGCATCCCGAGAGCTCGAGACC

CAGACACGAAAGTAACTTTAAAATACCTCACCTGACCCCACGAAAGCTAAGAAA

>181_Sample_ASV26

CACCGCGGTTATACGAGAGGCCCAAGTTGATACTCCACGGCGTAAAGAGTGGTTAGAAAA

GACCTACTACTAAAGCCGAACGCCTTCAAAGCTGTTATACGCATCCGAAGGTGAGAAGCC

CACCCACGAAAGTGGCTTTACAACCTTGACTCCACGAAAGCTATGACA

>181_Sample_ASV27

CACCGCGGTTATACGAGAGACCCCAGTTGATAAACACGGCGTAAAGAGTGGTTAAGGGCA

CAAAACAAATAAAGTCAAACAACCTCTTAGCTGTTATACGCATTATGAGGATATGAAGCC

CCCCTACGAAAGTAACTTTACCAACCCCTGACCCCACGAAAGCTAAGAAA

>181_Sample_ASV28

CACCGCGGTTATACGAGAGACCCTAGTTGATATATACGGCGTAAAGAGTGGTTTGGAATA

ACCAAGTAATATAGCCAAAGACCTCCCAAGCCGTCACACGCACCCCGGAGGCACGAAGCC

CAAACACGAAAGTAGCTTTATTAACTTACCGACGCCACGAAAGCTAAGAAA

>181_Sample_ASV29

CACCGCGGTTATACGAAAGACCCTAGTTTATAGGCACGGCGTAAAGGGTGGTTAGGGGAT

AAACAAAATAAAGCCAAAGACCTTCTAAGTCGTCATACACTCATGAAGGCACGAAGCCCA

AATACGAAAGTAACTTTACAACTTAGACCCGACTCCACGAAAGCTGGGGGA

>181_Sample_ASV30

CACCGCGGTTATACGAGAGACCCTAATTGATAGACTACGGCGTAAAGAGTGGTTTAGAAC

AAAAATTAATAAAGCCAAAGATCCTCCTGGCCGTCGCACGCATTTCGAGGATACGAAGCC

CCACTACGAAAGTAGCTTTACTACAACTTTTCTGACCCCACGAAAGCTAAGAAA

>181_Sample_ASV31

CACCGCGGTTATACGAAAGACCCTAGTTGATAGATCACGGCGTAAAGGGTGGTTAAGGAG

AACAAAAATAAAGCTAAAAATCCTCTAAGCTGTCATACGCTTTCCGAAGACATGAGACCC

AACCACGAAAGTAACTTTAAACTTTCCTGACACCACGAAAGCCAAGAAA

>182_Sample_ASV1

CACCGCGGTTATACGAAAGACCCTAGTTGATGGGTGCGGCGTAAAGGGTGGTTAGGGACT

ACAAAAAATAGAGCTAAAGATCCTCTAGGTCGTCATACACATCACGAGGAAACGAAGCCC

AAATACGAAAGTAGCTCTAAATATAATCCTGACGCCACGAAAGCCAAGAAA

>182_Sample_ASV2

CACCGCGGTTAAACGAGAGGCCCTAGTTGATGGTGCAACGGCGTAAAGGGTGGTTAAGGA

TAGCGAAATAATAAAGTCAAATGGCCCTCTGGCTGTCATACGCTTCTAGGAGTCCGAAGC

CCAGTATACGAAAGTAGCTTTAAGAAAGTCCACCTGACCCCACGAAAGCCAAGAAA

>182_Sample_ASV3

CACCGCGGTTATACGAGAGGCCCAAGTTGATGGTGCAACGGCGTAAAGGGTGGTTAAGGA

TAGCGAAATAATAAAGTCAAATGGCCCTCTGGCTGTCATACGCTTCTAGGAGTCCGAAGC

CCAGTATACGAAAGTAGCTTTAAGAAAGTCCACCTGACCCCACGAAAACTGAGGAA

>182_Sample_ASV4

CACCGCGGTTAAACGAGAGGCCCTAGTTGATGGTGCAACGGCGTAAAGGGTGGTTAAGGA

TAGCGAAATAATAAAGTCAAATGGCCCTCTGGCTGTCATACGCTTCTAGGAGTCCGAAGC

CCAGTATACGAAAGTAGCTTTAGAAAAGCCCACCTGACACCACGAAAGCTGTGAAA

>182_Sample_ASV5

CACCGCGGTTAAACGAGAGGCCCTAGTTGATGGTGCAACGGCGTAAAGGGTGGTTAAGGA

TAGCGAAATAATAAAGTCAAATGGCCCTCTGGCTGTCATACGCTTCTAGGAGTCCGAAGC

CCAGTATACGAAAGTAGCTTTAAGAAAGTCCACCTGACCCCACGAAAACTGAGGAA

>182_Sample_ASV6

CACCGCGGTTATACGAAAGACCCTAGTTGCTAGCCACGGCGTAAAGGGTGGTTAAGGACA

ACAACAAATAAAGCTAAAGATCCCCTAAGCCGTCATACGCATTCCGGGGGCACGAAGCCC

TAACACGAAAGTAGCTTTAAAAAATATACCTGACCCCACGAAAGCTAGGACA

>182_Sample_ASV7

CACCGCGGTTATACGAAAGACCCTAGTTGCTAGCCACGGCGTAAAGGGTGGTTAAGGACA

ACAACAAATAAAGCTAAAGATCCCCTAAGCCGTCATACGCATTCCGGGGGCACGAAGCCC

TAACACGAAAGTAGCTTTAAAAAATATACCTGACCCCACGAAAGCTACGACA

>182_Sample_ASV8

CACCGCGGTTATACGAAAGACCCTAGTTGCTAGCCACGGCGTAAAGGGTGGTTAAGGACA

ACAACAAATAAAGCTAAAGATCCCCTAAGCCGTCATACGCATTCCGGGGGCACGAAGCCC

TAACACGAAAGTAGCTTTAAAAAATATACCTGACCCCACGAAAGCTAAGAAA

>182_Sample_ASV9

CACCGCGGTTATACGAGAGACCCTAGTTGATTAAAGCGGCGTAAAGAGTGGTTATGGAAC

TATTCTTTTAAAGCAGAAAACCTCTCAAACTGTTATACGCACCCAGAGGTCTGAATCCCT

CACACGAAAGTGACTTTATTTATGCCTACCAGAACCCACGAAAGCTGGGACA

>182_Sample_ASV10

CACCGCGGTTATACGAGAGGCCCAAGTTGACAGACACCGGCGTAAAGAGTGGCTAGGGAA

AAATTACTACTAAAGCCGAACACCTTCAGAACTGTCATACGTTCCCGAAGATAAGAAGCC

CCACCACGAAAGTGGCTTTATATTACCCGACCCCACGAAAGCTGTGAAA

>182_Sample_ASV11

CACCGCGGTTATACGAGAGGCCCAAGTTGACAGACACCGGCGTAAAGAGTGGCTAGGGAA

AAATTACTACTAAAGCCGAACACCTTCAGAACTGTCATACGTTCCCGAAGATAAGAAGCC

CCACCACGAAAGTGGCTTTATATTACCCGACCCCACGAAAGCCAAGAAA

>182_Sample_ASV12

CACCGCGGTTATACGAGAGGCCCAAGTTGACAGACACCGGCGTAAAGAGTGGCTAAGGAA

AAATTTCTACTAAAGCCGAACACCTTCAGAACTGTCATACGTTCCCGAAGAAAAGAAGCC

CCACCACGAAAGTGGCTTTATATTACCCGACCCCACGAAAGCTGTGAAA

>182_Sample_ASV13

CACCGCGGTTATACGAGAGGCCCAAGTTGACAGACATCGGCGTAAAGAGTGGTTAGGAAG

TTTTTGAAACTAAAGCCGAACGCCCTCAGAACTGTTATACGTACCCGAGGGTAAGAAGCC

CCACTACGAAAGTGGCTTTATATCTCCGACCCCACGAAAGCTGTGAAA

>182_Sample_ASV14

CACCGCGGTTGTACGAGAGGCCCAAGTTGACAGACATCGGCGTAAAGAGTGGTTAGGAAG

TTTTTGAAACTAAAGCCGAACGCCCTCAGAACTGTTATACGTACCCGAGGGTAAGAAGCC

CCACTACGAAAGTGGCTTTATATCTCCGACCCCACGAAAGCTGTGAAA

>182_Sample_ASV15

CACCGCGGTTATACGAGAGGCTCAAGTTGACAGACATCGGCGTAAAGAGTGGTTAGGAAG

TTTTTGAAACTAAAGCCGAACGCCCTCAGAACTGTTATACGTACCCGAGGGTAAGAAGCC

CCACTACGAAAGTGGCTTTATATCTCCGACCCCACGAAAGCTGCGAAA

>182_Sample_ASV16

CACCGCGGTTATACGAGAGGCTCAAGTTGACAGACATCGGCGTAAAGAGTGGTTAGGAAG

TTTTTGAAACTAAAGCCGAACGCCCTCAGAACTGTTATACGTACCCGAGGGTAAGAAGCC

CCACTACGAAAGTGGCTTTATATCTCCGACACCACGAAAGCTGAGGAA

>182_Sample_ASV17

CACCGCGGTTAAACGAGAGGCCCAAGTTGACAGACATCGGCGTAAAGAGTGGTTAGGAAG

TTTTTGAAACTAAAGCCGAACGCCCTCAGAACTGTTATACGTACCCGAGGGTAAGAAGCC

CCACTACGAAAGTGGCTTTATATCTCCGACCCCACGAAAGCTGCGAAA

>182_Sample_ASV18

CACCGCGGTTATACGAGAGGCTCAAGTTGACAGACATCGGCGTAAAGAGTGGTTAGGAAG

TTTTTGAAACTAAAGCCGAACGCCCTCAGAACTGTTATACGTACCCGAGGGTAAGAAGCC

CCACTACGAAAGTGGCTTTATATCTCCGACCCCACGAAAGCTGAGAAA

>182_Sample_ASV19

CACCGCGGTTAAACGAGAGACCCTAGTTGATCATCACGGCGTAAAGAGTGGTTAGGGGAT

TATCATAAATAAAGCCAAAGACCTCCCAAGCTGTCGCACGCATTCCGGAGGCGCGAAGCC

CACACACGAAAGTAGCTTTAACTATTGACCCTGATGCCACGAAAGCTAAGAAA

>182_Sample_ASV20

CGCCGCGGTTAAACGAGAGGCCCTAGTTGATGGAACAACGGCGTAAAGGGTGGTTAAGGA

TAGTGAGACAATAAAGTCGAATGGCCCTTTGGCTGTCATACGCTTCTGGGAGCCTGAAGC

CCACTATACGAAAGTAGCTTTAACAACGTCCACCTGACCCCACGAAAACTGAGAAA

>182_Sample_ASV21

CGCCGCGGTTAAACGAGAGGCCCTAGTTGATGGAACAACGGCGTAAAGGGTGGTTAAGGA

TAGTGAGACAATAAAGTCGAATGGCCCTTTGGCTGTCATACGCTTCTGGGAGCCTGAAGC

CCACTATACGAAAGTAGCTTTAACAATGTCCACCTGACCCCACGAAAACTGAGAAA

>182_Sample_ASV22

CACCGCGGTTATACGAAAGACCCTAGTTGATAGATCACGGCGTAAAGGGTGGTTAGGGAA

TACAAAAATAAAGCTAAAAATCCTCTAAGCTGTCATACGCTTCCCGAAGACATGAAACCC

AACCACGAAAGTAGCTTTAAACTCTCCTGACACCACGAAAGCCAAGGAA

>182_Sample_ASV23

CACCGCGGTTATACGAGAGGCCCAAGTTGATAAGCCCGGCGTAAAGAGTGGTTATGGAGA

ATAAAACACTAAAGCTAAAGACCCCCTAGGCTGTTATACGCACCTGGCGGCTCGAACCAC

CTATACGAAAGTAGCTTTACCCCCCTTCCACCAGAATCCACGACAGCTGGGGCA

>182_Sample_ASV24

CACCGCGGTTAAACGAGAGGCCCAAGTTGATAAAATACGGCGTAAAGCGTGGTTAAAAGC

CCCCACTAAACTAAGACTAAACCTTTCCAAAGCTGTTATACGCACCCGGAAATATGAAAC

TCAACTACGAAAGTGGCCTTAATTTCCCCTTGACCCCACGAAAGCTGCGAAA

>182_Sample_ASV25

CACCGCGGTTATACGAGAGGCCCAAGTTGACAGACATCGGCGTAAAGAGTGGTTAAGGAA

ACCCCATTACTAAAGCCGAACGCCCTCAGAACTGTCATACGTTCCCGAAGGTAAGAAGCC

CCACTACGAAAGTGGCTTTAACCACACCCGACCCCACGAAAGCTGTGAAA

>182_Sample_ASV26

CACCGCGGTTAAACGAGAGGCCCTAGTTGATATTACAACGGCGTAAAGGGTGGTTAAGGA

AAGCAAGCAATAAAGCCGAATGGCCCTTTGGCTGTCATACGCTTCTAGGTGTCCGAAGCC

CAATATACGAAAGTAGCTTTAGAAAAGCCCACCTGACACCACGAAAGCTGAGGAA

>182_Sample_ASV27

CACCGCGGTTAAACGAGAGGCCCTAGTTGATATTACAACGGCGTAAAGGGTGGTTAAGGA

AAGCAAGCAATAAAGCCGAATGGCCCTTTGGCTGTCATACGCTTCTAGGTGTCCGAAGCC

CAATATACGAAAGTAGCTTTAGAAAAGCCCACCTGACGCCACGAAAGCCAAGAAA

>182_Sample_ASV28

CACCGCGGTTAAACGAGAGGCCCTAGTTGATATTACAACGGCGTAAAGGGTGGTTAAGGA

AAGCAAGCAATAAAGCCGAATGGCCCTTTGGCTGTCATACGCTTCTAGGTGTCCGAAGCC

CAATATACGAAAGTAGCTTTAGAAAAGCCCACCTGACACCACGAAAGCCAAGAAA

>182_Sample_ASV29

CACCGCGGTTAAACGAGAGGCCCTAGTTGATATTACAACGGCGTAAAGGGTGGTTAAGGA

AAGCAAGCAATAAAGTCAAATGGCCCTCTGGCTGTCATACGCTTCTAGGAGTCCGAAGCC

CAGTATACGAAAGTAGCTTTAGAAAAGCCCACCTGACACCACGAAAGCTGAGGAA

>182_Sample_ASV30

CACCGCGGTCACACGATTAACCCAAGTCAATAGAAGCCGGCGTAAAGAGTGTTTTAGATC

ACCCCCTCCCCAATAAAGCTAAAACTCACCTGAGTTGTAAAAAACTCCAGTTGACACAAA

ATAGACTACGAAAGTGGCTTTAACATATCTGAACACACAATAGCTAAGACC

>182_Sample_ASV31

CACCGCGGTTATACGAGAGGCCCTAGTTGATAAATACGGCGTAAAGAGTGGTTAGAGGAC

TCCCATAATAAAGCCAAAGACCCTCCGGGCCGTCACACGCATACGAGGGCACGAAGTTCA

CACACGAAAGTAGCTTTAAATTACCCGACGCCACGAAAGCTAAGAAA

>182_Sample_ASV32

CACCGCGGTTATACGAGAAACCCTAGTTGACAAATACGGCGTAAAGAGTGGTTAAGGGGT

ATCAAAAATAAAGTCAAACATTTTCCTAGTTGTTATAAGCATTTCGAGAACATGAAGCCC

AACCACGAAAGTAACTTTACCTTCACCTGACCCCACGAAAGCTAAGAAA

>182_Sample_ASV33

CACCGCGGTTATACGAAAGACCCTAGTTTATAAGTACGGCGTAAAGGGTGGTTAGGGGAC

AAACAAAATAAAGCCAAAGACCTCCTAAGCCGTCATACGCTTACGGAGGCACGAAGCCCA

AACACGAAAGTAGCTTTACCGAACATGCCCGACTCCACGAAAGCTGGGAAA

>182_Sample_ASV34

CACCGCGGTTATACGAGAGACCCTAGTTGATATATACGGCGTAAAGAGTGGTTATGGGAA

AAACAAATAAAGTCAAACAACCTCTTAGCTGTTATACGCATTATGAGAGCATGAAGCCCC

CTCACGAAAGTAACTTTAATACCTCCTGACCCCACGAAAGCTAAGAAA

>182_Sample_ASV35

CACCGCGGTTATACGAGAGACCCTAGTTGATAAATACGGCGTAAAGAGTGGTTATGGGAA

AAACAAATAAAGTCAAACAACCTCTTAGCTGTTATACGCATTATGAGAGCATGAAGCCCC

CTCACGAAAGTAACTTTAATACCTCCTGACCCCACGAAAGCTAAGAAA

>182_Sample_ASV36

CACCGCGGTTATACGAGAGGCTCAAGTTGATAAACCCCGGCGTAAAGAGTGGTTAAGGAA

AAATAGAAACTAAAGCCGAATGCTTTCAAGACTGTTATACGTTCCCGAGAGTAAGAAGAC

CAATTACGAAAGTGGCTTTACTTTACCTGACCCCACGAAAGCCAAGAAA

>182_Sample_ASV37

CACCGCGGTTATACGAGAGGCTCAAGTTGATAAACCCCGGCGTAAAGAGTGGTTAAGGAA

AAATAGAAACTAAAGCCGAATGCTTTCAAGACTGTTATACGTTCCCGAGAGTAAGAAGAC

CAATTACGAAAGTGGCTTCACTTTACCTGACCCCACGAAAGCTATGAAA

>182_Sample_ASV38

CACCGCGGTTATACGAGAGGCTCAAGTTGACAGACATCGGCGTAAAGAGTGGTTAAGGAA

AAATAGAAACTAAAGTCGAATGCTTTCAAGACTGTTATACGTTCCCGAGAGTAAGAAGAC

CAATTACGAAAGTGGCTTTACTTTACCTGACCCCACGAAAGCTACGACA

>182_Sample_ASV39

CACCGCGGTTATACGAGAGGCCCAAGTTGATAGTCTTCGGCGTAAAGAGTGGTTAAGGAA

AAATAGAAACTAAAGCCGAATGCTTTCAAGACTGTTATACGTTCCCGAGAGTAAGAAGAC

CAATTACGAAAGTGGCTTTACTTTACCTGACCCCACGAAAGCTACGACA

>182_Sample_ASV40

CACCGCGGTTATACGAGAGGCTCAAGTTGATAAACCCCGGCGTAAAGAGTGGTTAAGGAA

AAATAGAAACTAAAGCCGAATGCTTTCAAGACTGTTATACGTTCCCGAGAGTAAGAAGAC

CAATTACGAAAGTGGCTTTACTTTACCTGACCCCACGAAAGCTACGACA

>182_Sample_ASV41

CACCGCGGTTATACGAGAGGCTCAAGTTGATGAACCCCGGCGTAAAGAGTGGTTAAGGGA

GATCAAAACTAAAGCCGAATGCTTTCAAAGCTGTTATACGCTTCCGAAAGTAAGAAGCCC

AATCACGAAAGTGGCTTTACTTTACCTGACCCCACGAAAGCTACGACA

>182_Sample_ASV42

AACCGCGGTTATACGAGAGACCCTAATTGATAGCTCACGGCGTAAAGGGTGGTTTGGGGA

AACCTCAAAATAAAGCTAAAGACCTTCTAAGCCGTTACACGCATACCGAAGGCACGAGGC

CCTAACACGAAAGTAGCTTTACTATTACCCCTGACGCCACGAAAGCTAAGAAA

>182_Sample_ASV43

CACCGCGGTTATACGAGAGGCCCAAGTTGACAAACCCCGGCGTAAAGAGTGGTTAAGAAA

AATTTAAAACTAAAGCCGAATACCCTCAAAGCTGTTATACGCACCCGAAGGTAAGAAGCC

CAATCACGAAAGTGGCTTTACATTTCCCGAACCCACGAAAGCTATGAAA

>182_Sample_ASV44

CACCGCGGTTAAACGAGAGGCCCTAGTTGATAGTACAACGGCGTAAAGGGTGGTTAAGGA

TAGTGAAATAATAAAGTCGAATGGCCCTTTGGCTGTCATACGCTTCTAGGAGTCCGAAGC

CCAATATACGAAAGTAACTTTAAGAGCGCCCACCTGACCCCACGAAAACTGAGAAA

>182_Sample_ASV45

CACCGCAGTTATACGAGAGACCCTAATTGATAGGCGCGGCGTAAAGAGTGGTTTAGGACT

AAAACTAATAAAGCTAAAGATCCTCCCAGCCGTCGCACGCATTCTGAGGACACGAAACTC

AACCACGAAAGTAGCTTTAACATACTCTCCTGACCCCACGAAAGCTAAGAAA

>182_Sample_ASV46

CACCGCGGTTATACGAGAGACCCTAATTGATAGGCGCGGCGTAAAGAGTGGTTTAGGACT

AAAACTAATAAAGCTAAAGATCCTCCCAGCCGTCGCACGCATTCTGAGGACACGAAACTC

AACCACCAAAGTAGCTTTAACATACTCTCCTGACCCCACGAAAGCTAAGAAA

>182_Sample_ASV47

CACCGCGGTTATACGAGAGACCCTAATTGATAGGCGCGGCGTAAAGAGTGGTTTAGGACT

AAAACTAATAAAGCTAAAGATCCTCCCAGCCGTCGCACGCATTCTGAGGACACGAAACTC

AACCACGAAAGTAGCTTTAACATACTCTCCTGACCCCACGAAAGCTAAGAAA

>182_Sample_ASV48

CACCGCGGTTATACGAGAGACCCTAATTGATAGGCGCGGCGTAAAGAGTGGTTTAGGACT

AAAACTAATAAAGCTAAAGATCCTCCCAGCCGTCGCACGCATTCTGAGGATACGAAACTC

AACCACGAAAGTAGCTTTAACATACTCTCCTGACCCCACGAAAGCTAAGAAA

>182_Sample_ASV49

CACCGCGGTTATACGAGAGGCCCAAGTTGATACTCCACGGCGTAAAGAGTGGTTAGAAAA

GACCTACTACTAAAGCCGAACGCCTTCAAAGCTGTTATACGCATCCGAAGGTGAGAAGCC

CACCCACGAAAGTGGCTTTACAACCTTGACTCCACGAAAGCTATGACA

>182_Sample_ASV50

CACCGCGGTTATACGAGAGGCCCAAGTTGATAGTCTTCGGCGTAAAGAGTGGTTAAAGAA

GCAATTAGACTAAGGCTAAACTCTTCCAAGGCTGTCATACGCACCCGGAAGCATGAAACC

CAACCACGAAAGTGGCCTTATTTACCTTGACCCCACGAAAGCTACGACA

>182_Sample_ASV51

CACCGCGGTTATACGAGAGGCCCAAGTTGATAGTCTTCGGCGTAAAGAGTGGTTAAAGAA

GCAATTAGACTAAGGCTAAACTCTTCCAAGGCTGTCATACGCACCCGGAAGCATGAAACC

CAACCACGAAAGTGACCTTATTTACCTTGACCCCACGAAAGCTGTGAAA

>182_Sample_ASV52

CACCGCGGTTATACGAGAGGCACAAGTTGATAGTCTTCGGCGTAAAGAGTGGTTAAAGAA

GCAATTAGACTAAGGCTAAACTCTTCCAAGGCTGTCATACGCACCCGGAAGCATGAAACC

CAACCACGAAAGTGGCCTTATTTACCTTGACCCCACGAAAGCTACGACA

>182_Sample_ASV53

CACCGCGGTTATACGAGAGGCCCAAGTTGATAGTCTTCGGCGTAAAGAGTGGTTAAAGAA

GCAATTAGACTAAGGCTAAACTCTTCCAAGGCTGTCATACGCACCCGGAAGCATGAAACC

CAACCACGAAAGTGGCCTTATTTACCTTGACCCCACGAAAGCTGTGAAA

>182_Sample_ASV54

TACCGCGGTTATACGAGAGGCCCAAGTTGATAGTCTTCGGCGTAAAGAGTGGTTAAAGAA

GCAATTAGACTAAGGCTAAACCCTTCCAAAGCCGTCATACGCATCCGGAAGCATGAAACC

CAACCACGAAAGTGGCCTTATTTACCTTGACCCCACGAAAGCTGTGAAA

>182_Sample_ASV55

TACCGCGGTTATACGAGAGGCCCAAGTTGATAGTCCTCGGCGTAAAGAGTGGTTAGAGAA

TCTATTAAACTAAGGCTAAACCCTTCCAAAGCCGTCATACGCATCCGGAAGCATGAAACC

CAACCACGAAAGTGACCTTATTTACCTTGACCCCACGAAAGCTGCGAAA

>182_Sample_ASV56

TACCGCGGTTATACGAGAGGCCCAAGTTGATAGTCCTCGGCGTAAAGAGTGGTTAGAGAA

TCTATTAAACTAAGGCTAAACCCTTCCAAAGCCGTCATACGCATCCGGAAGCATGAAACC

CACCCACGAAAGTAGCCTTATTTACCTTGACCCCACGAAAGCTGTGGAA

>182_Sample_ASV57

TACCGCGGTTATACGAGAGGCCCAAGTTGATAGTCTTCGGCGTAAAGAGTGGTTAGAGAA

TCTATTAAACTAAGGCTAAACCCTTCCAAAGCCGTCATACGCATCCGGAAGCATGAAACC

CACCCACGAAAGTAGCCTTATTTACCTTGACCCCACGAAAGCTGTGGAA

>182_Sample_ASV58

CACCGCGGTTATACGAGAGACCCCAGTTGATAAACACGGCGTAAAGAGTGGTTAAGGGCA

CAAAACAAATAAAGTCAAACAACCTCTTAGCTGTTATACGCATTATGAGGATATGAAGCC

CCCCTACGAAAGTAACTTTACCAACCCCTGACCCCACGAAAGCTAAGAAA

>182_Sample_ASV59

CACCGCGGTTATACGAGAGACCCTAGTTGATATATACGGCGTAAAGAGTGGTTTGGAATA

ACCAAGTAATATAGCCAAAGACCTCCCAAGCCGTCACACGCACCCCGGAGGCACGAAGCC

CAAACACGAAAGTAGCTTTATTAACTTACCGACGCCACGAAAGCTAAGAAA

>182_Sample_ASV60

CACCGCGGTTATACGAAAGACCCTAGTTTATAGGCACGGCGTAAAGGGTGGTTAGGGGAT

AAACAAAATAAAGCCAAAGACCTTCTAAGTCGTCATACACTCATGAAGGCACGAAGCCCA

AATACGAAAGTAACTTTACAACTTAGACCCGACTCCACGAAAGCTGGGGGA

>182_Sample_ASV61

CACCGCGGTTAAACGAGAGGCCCTAGTTGATGGTGCAACGGCGTAAAGGGTGGTTAAGGA

AAGCAAGCAATAAAGCCGAATGGCCCTTTGGCTGTCATACGCTTCTAGGTGTCCGAAGCC

CAATATACGAAAGTAGCTTTAGAAAAGCCCACCTGACACCACGAAAGCTGAGGAA

>182_Sample_ASV62

CACCGCGGTTATACGAGAGACCCTAATTAATAGATACGGCGTAAAGAGTGGTTTAGGACA

AAAATTAATAAAGCCGAAGTTTCTCCTAGCCGTTGTACGCACTCTGAGAACACGAAGCCC

CACTACGAAAGTAGCTTTACCTATTATTTCCTGACCCCACGAAAGCTAAGAAA

>182_Sample_ASV63

CACCGCGGTTATACGAGAGGCCCAAGTTGATAGACATCGGCGTAAAGAGTGGTTAAGGAA

GTCTCAAAACTAAAGCCGAATGCCTTCAAGACTGTTATACGTTTCCGAAGGTAAGAAGCC

CCACTACGAAAGTGGCTTTACTCCACCTGACTCCACGAAAGCTGTGAAA

>182_Sample_ASV64

CACCGCGGTTATACGAGAGGCCCAAGTTGACAAACTCCGGCGTAAAGCGTGGTTAAGGCA

CACTCAAAAACTAAAGCCGAATATCTTCAGAGCAGTCATACGCATCCGAAGACACGAAGC

CCCACCACGAAAGTGGCTTTATGACACCTGACCCCACGAAAGCTAGGACA

>182_Sample_ASV65

CACCGCGGTTATACGAGAGGCCCAAGTTGACAAACTCCGGCGTAAAGCGTGGTTAAGGCA

CACTCAAAAACTAAAGCCGAATATCTTCAGAGCAGTCATACGCATCCGAAGACACGAAGC

CCCACCACGAAAGTGGCTTTATGACACCTGACCCCACGAAAGCCAAGAAA

>182_Sample_ASV66

CACCGCGGTTATACGAGAGGCTCAAGTTGATAAACCCCGGCGTAAAGCGTGGTTAAGGCA

CACTCAAAAACTAAAGCCGAATATCTTCAGAGCAGTCATACGCATCCGAAGACACGAAGC

CCCACCACGAAAGTGGCTTTATGACACCTGACCCCACGAAAGCTAGGACA

>182_Sample_ASV67

CACCGCGGTTATACGAAAGACCCTAGTTGATAGACCACGGCGTAAAGGGTGGTTAAGGAA

CACAAAAATAAAGCTAAAGATCCTCTAAGCTGTCATACGCTTCCCGAAGACATGAAACCC

AATCACGAAAGTAGCTTTAAACTGTCCTGACACCACGAAAGCCAAGAAA

>182_Sample_ASV68

CACCGCGGTTATACGAGAGGCCCAAGTTGACAGTCTTCGGCGTAAAGCGTGGTTAAAGAT

ATACTAAACTAAGGCTAAACTTCCCCAAGGCTGTCATACGCACCCGGAAACATGAGACCC

GACCACGAAAGTGGCCTTAATAccccccccccTTGACCCCACGAAAGCTATGAAA

>182_Sample_ASV69

CACCGCGGTTAAACGAGAGGCCCAAGTTGATAGTCCTCGGCGTAAAGAGTGGTTAAAGAT

ATACTAAACTAAGGCTAAACTTCCCCAAGGCTGTCATACGCACCCGGAAACATGAGACCC

GACCACGAAAGTGGCCTTAATAccccccccccTTGACCCCACGAAAGCTGAGGAA

>183_Sample_ASV1

CACCGCGGTTATACGAAAGACCCTAGTTGATGGGTGCGGCGTAAAGGGTGGTTAGGGACT

ACAAAAAATAGAGCTAAAGATCCTCTAGGTCGTCATACACATCACGAGGAAACGAAGCCC

AAATACGAAAGTAGCTCTAAATATAATCCTGACGCCACGAAAGCCAAGAAA

>183_Sample_ASV2

CACCGCGGTTATACGAGAGACCCTAGTTGATTAAAGCGGCGTAAAGAGTGGTTATGGAAC

TATTCTTTTAAAGCAGAAAACCTCTCAAACTGTTATACGCACCCAGAGGTCTGAATCCCT

CACACGAAAGTGACTTTATTTATGCCTACCAGAACCCACGAAAGCTGGGACA

>183_Sample_ASV3

CACCGCGGTTATACGAAAGACCCTAGTTTATAGGTACGGCGTAAAGGGTGGTTAGGGGAC

AAACAAAATAAAGCCAAAGACCTCCTAAGCCGTCATACGCTCACGGAAGCACGAAGCCCA

AACACGAAAGTAGCTTTACCGAACATGCCCGACTCCACGAAAGCTGGGACA

>183_Sample_ASV4

CACCGCGGTTATACGAAAGACCCTAGTTTATAGGTACGGCGTAAAGGGTGGTTAGGGGAC

AAACAAAATAAAGCCAAAGACCTCCTAAGCCGTCATACGCTCACGGAAGCACGAAGCCCA

AACACGAAAGTAGCTTTACCGAACATGCCCGACTCCACGAAAGCTGGGGAA

>183_Sample_ASV5

CACCGCGGTTATACGAAAGACCCTAGTTGATAGCCACGGCGTAAAGGGTGGTTAAGGAAC

CTAAAAAATAAAGCTAAAGACCCTCTAGGCCGTCATACGCATTACGAGGCAAACGAAACC

CCAACACGAAAGTAGCTTTAGACAAATATCCTGATGCCACGAAAGCCAAGAAA

>183_Sample_ASV6

CACCGCGGTTATACGAAAGACCCTAGTTGATAGCCACGGCGTAAAGGGTGGTTAAGGAAC

CTAAAAAATAAAGCTAAAGACCCTCTAGGCCGTCATACGCATTACGAGGCAAACGAAACC

CCAACACGAAAGTAGCTTTAGACAAATATCCTGATGCCACGAAAGCTGTGAAA

>183_Sample_ASV7

CACCGCGGTTATACGAGAGGCCCAAGTTGACAGACACCGGCGTAAAGAGTGGCTAGGGAA

AAATTACTACTAAAGCCGAACACCTTCAGAACTGTCATACGTTCCCGAAGATAAGAAGCC

CCACCACGAAAGTGGCTTTAACCACACCCGACCCCACGAAAGCTGTGAAA

>183_Sample_ASV8

CACCGCGGTTATACGAGAGGCCCAAGTTGACAGACACCGGCGTAAAGAGTGGCTAGGGAA

AAATTACTACTAAAGCCGAACACCTTCAGAACTGTCATACGTTCCCGAAGATAAGAAGCC

CCACCACGAAAGTGGCTTTATATTACCCGACCCCACGAAAGCTGTGAAA

>183_Sample_ASV9

CACCGCGGTTATACGAGAGACCCTAGTTGATATATACGGCGTAAAGAGTGGTTTGGGGTT

ATGCCTCCAAAATAAAGCCGAAGATCTCCCAAGCCGTCACACGCATCCCGGAGACACGAA

GCCCTAATACGAAGGTAGCTTTATTAATTCCCCGACGCCACGAAAGCTAAGAAA

>183_Sample_ASV10

CACCGCGGTTATACGAGAGACCCTAGTTGATATATACGGCGTAAAGAGTGGTTTGGGGTT

ATGCCTCCAAAATAAAGCCGAAGATCTCCCAAGCCGTCACACGCATCCCGGAGACACGAA

GCCCTAATACGAAGGTAGCTTTATTAGTACCCCGACGCCACGAAAGCTAAGAAA

>183_Sample_ASV11

CACCGCGGTTATACGAGAGGCCCAAGTTGATAAGCCCGGCGTAAAGAGTGGTTATGGAGA

ATAAAACACTAAAGCTAAAGACCCCCTAGGCTGTTATACGCACCTGGCGGCTCGAACCAC

CTATACGAAAGTAGCTTTACCCCCCTTCCACCAGAATCCACGACAGCTGGGGCA

>183_Sample_ASV12

CACCGCGGTTATACGAGAGGCCCTAGTTGATAGCCACGGCGCAAAGAGTGGTTAAGGAGC

CCCACTAAATAAAGTCGAACACTTCCTAGGCCGTTATACGTTTTCTAGAAGCACGAAACC

CAATTACACGAAAGCAACTTTATACTAAACAACCTGACCCCACGAAAGCTAAGAAA

>183_Sample_ASV13

CACCGCGGTTATACGAGAGGCCCAAGTTGACAGACATCGGCGTAAAGAGTGGTTAAGGAA

ACCCCATTACTAAAGCCGAACGCCCTCAGAACTGTCATACGTTCCCGAAGGTAAGAAGCC

CCACTACGAAAGTGGCTTTAACCACACCCGACCCCACGAAAGCTGTGAAA

>183_Sample_ASV14

CACCGCGGTTATACGAGAGGCCCAAGTTGATAGATATCGGCGTAAAGAGTGGTTAAGGAA

ACCCCGTTACTAAAGCCGAACGCCCTCAGAACTGTCATACGTTCCCGAAGGTAAGAAGCC

CCACTACGAAAGTGGCTTTAACCACACCCGACCCCACGAAAGCTGTGAAA

>183_Sample_ASV15

CACCGCGGTTATACGAGAGGCCCAAGTTGACAGACATCGGCGTAAAGAGTGGTTAAGGAA

ACCCCATTACTAAAGCCGAACGCCCTCAGAACTGTCATACGTTCCCGAAGGTAAGAAGCC

CCACTACGAAAGTGGCTTTATATTACCCGACCCCACGAAAGCTGTGAAA

>183_Sample_ASV16

CACCGCGGTTATACGAGAGGCCCAAGTTGACAGACACCGGCGTAAAGAGTGGTTAAGGAA

ATTCATCAACTAAAGCCGAATGCCCTCAGGACTGTTATACGTTTGCGAAGGTAAGAAGCC

CTACTACGAAAGTGGCTTTATTCCACCTGATTCCACGAAAGCTGCGGAA

>183_Sample_ASV17

CACCGCGGTCACACGATTAACCCAAGTCAATAGAAGCCGGCGTAAAGAGTGTTTTAGATC

ACCCCCTCCCCAATAAAGCTAAAACTCACCTGAGTTGTAAAAAACTCCAGTTGACACAAA

ATAGACTACGAAAATGGCTTTAACATATCTGAACACACAATAGCTAAGACC

>183_Sample_ASV18

CACCGCGGTTATACGAGAGGCCCCAGTTGATAAACACGGCGTAAAGGGTGGTTAAGGGAG

CATAAGAATAAAGCCGAAGGGCCCTTTGGCCGTTATACGCTTCTAGGTGTCCGAAGCCCA

AACACGAAAGTAGCTTTAGTTAAGCCCACCTGACTCCACGAAAACTGAGAAA

>183_Sample_ASV19

CACCGCGGTTATACGAGAGACCCTAGTTGATAAATACGGCGTAAAGAGTGGTTATGGGAA

AAACAAATAAAGTCAAACAACCTCTTAGCTGTTATACGCATTATGAGAGCATGAAGCCCC

CTCACGAAAGTAACTTTACCAACCCCTGACCCCACGAAAGCTGTGAAA

>183_Sample_ASV20

CACCGCGGTTATACGAGAGACCCTAGTTGATTTAAGCGGCGTAAAGAGTGGTTATGGGAA

AAACAAATAAAGTCAAACAACCTCTTAGCTGTTATACGCATTATGAGAGCATGAAGCCCC

CTCACGAAAGTAACTTTAATACCTCCTGACCCCACGAAAGCTGTGAAA

>183_Sample_ASV21

CACCGCGGTTATACGAGAGACCCCAGTTGATAAACACGGCGTAAAGAGTGGTTAAGGGCA

CAAAACAAATAAAGTCAAACAACCTCTTAGCTGTTATACGCATTATGAGAGCATGAAGCC

CCCTCACGAAAGTAACTTTAATACCTCCTGACCCCACGAAAGCTAAGAAA

>183_Sample_ASV22

CACCGCGGTTATACGAGAGACCCTAGTTGATAAATACGGCGTAAAGAGTGGTTATGGGAA

AAACAAATAAAGTCAAACAACCTCTTAGCTGTTATACGCATTATGAGAGCATGAAGCCCC

CTCACGAAAGTAACTTTAATACCTCCTGACCCCACGAAAGCTAAGAAA

>183_Sample_ASV23

CACCGCGGTTATACGAGAGGCTCAAGTTGATGAACCCCGGCGTAAAGAGTGGTTAAGGGA

GATCAAAACTAAAGCCGAATGCTTTCAAAGCTGTTATACGCTTCCGAAAGTAAGAAGCTC

AATCACGAAAGTGGCTTTACTTTACCTGACCCCACGAAAGCTACGACA

>183_Sample_ASV24

CACCGCGGTTATACGAAAGACCCTAGTTGATGGATCACGGCGTAAAGGGTGGTTAAGGGA

TATAAAAATAAAGCTAAAAATCCTCTTAGCTGTCATACGCTTTCCGAAGACATGAGGCCC

AATTACGAAAGTAGCTTTAAACCACCCTGACACCACGAAAGCCAAGAAA

>183_Sample_ASV25

CACCGCGGTTATACGAAAGACCCCAGTTTATAGATGCGGCGTAAAGGGTGGTTAAGAGAA

AATCAAAATAAGGCCAAAGATCCCCTAAGCAGTCATACGCTCCACGAGGACACGAAACCC

AAATACGAAAGTTGCCTTAATAAACTTCACCTGATCCCACGAAAGCTGAAAAA

>183_Sample_ASV26

CACCGCGGTTATACGAGAGGCCCAAGTTGACAAACCCCGGCGTAAAGAGTGGTTAAGGAA

AATTTAAAACTAAAGCCGAATGCCCCCAAAGCTGTTATACGCACCCGAGGGTAAGAAGCC

CAATCACGAAAGTGGCTTTACACTACCTGAACCCACGAAAGCTATGAAA

>183_Sample_ASV27

CACCGCGGTTATACGAGAGGCCCAAGTTGACAAACCCCGGCGTAAAGAGTGGTTAAGGAA

AATTTAAAACTAAAGCCGAATGCCCCCAAAGCTGTTATACGCACCCGAGGATAAGAAGCC

CAATCACGAAAGTGGCTTTACACTACCTGAACCCACGAAAGCTATGAAA

>183_Sample_ASV28

CACCGCGGTCATACGATTAACCCAAGTTAATAGGCACACGGCGTAAAGCGTGTTTAAGCA

CTACGCCAAATAAAGTTAAACTCCAATTAAGCTGTAAAAAGCCATAATCAAGACGAAAAT

AAATAACGAAAGTAACTTTACAACCGCTGAGACACGATAGCTAAGATC

>183_Sample_ASV29

CACCGCGGTTATACGAGAGGCCCTAGTTGATAACTACCGGCGTAAAGAGTGGTTATGGAA

AAATATTTAATAAAGCCGAACACCCCCTCAGCCGTCATACGCACCTGGGAGCACGAAGAC

CTACTGCGAAAGCAGCTTTAATTATGCCTGACCCCACGACAGCTAAGAAA

>183_Sample_ASV30

CACCGCGGTTATACGAAAGACCCTAGTTTATAGACACGGCGTAAAGGGTGGTTCGGGACT

AAACAAAATAAAGCCAAAGACCTCCTAAGCCGTCATACGCTCACGGAAGCACGAAGCCCA

AGTACGAAAGTAGCTTTACAAAACATGCCTGACTCCACGAAAGCTAGGAAA

>183_Sample_ASV31

CACCGCGGTTATACGAGAGGCCCAAGTTGATACTCCACGGCGTAAAGAGTGGTTAGAAAA

GACCTACTACTAAAGCCGAACGCCTTCAAAGCTGTTATACGCATCCGAAGGTGAGAAGCC

CACCCACGAAAGTGGCTTTACAACCTTGACTCCACGAAAGCTATGACA

>183_Sample_ASV32

CACCGCGGTTATACGAGAGACCCCAGTTGATAAACACGGCGTAAAGAGTGGTTAAGGGCA

CAAAACAAATAAAGTCAAACAACCTCTTAGCTGTTATACGCATTATGAGGATATGAAGCC

CCCCTACGAAAGTAACTTTACCAACCCCTGACCCCACGAAAGCTAAGAAA

>183_Sample_ASV33

CACCGCGGTTATACGAAAGACCCTAGTTTATAGGCACGGCGTAAAGGGTGGTTAGGGGAT

AAACAAAATAAAGCCAAAGACCTTCTAAGTCGTCATACACTCATGAAGGCACGAAGCCCA

AATACGAAAGTAACTTTACAACTTAGACCCGACTCCACGAAAGCTGGGGGA

>186_Sample_ASV1

CACCGCGGTTATACGAGAGGCCCAAGTTGACAGACATCGGCGTAAAGAGTGGTTAAGGAA

AACCTTCAACTAAAGTCGAACGCTCTCAAGACTGTCGTACGTTTCCGAGAGTATGAAGCC

CCACCACGAAAGTGACTTTACCTCACCTGACTCCACGAAAGCTGCGAAA

>186_Sample_ASV2

CACCACGGTTATACGAGAGGCCCAAGTTGAAAGACATCGGCGTAAAGAGTGGTTAAGGAA

AACCTTCTACTAAAGTCGAACGCTCTCAGGACTGTCGTACGTTTCCGAGAGTATGAAGCC

CCACCACGAAAGTGACTTTACCTCACCTGACTCCACGAAAGCTGCGAAA

>186_Sample_ASV3

CACCGCGGTTATACGAAAGACCCTAGTTGATGGGTGCGGCGTAAAGGGTGGTTAGGGACT

ACAAAAAATAGAGCTAAAGATCCTCTAGGTCGTCATACACATCACGAGGAAACGAAGCCC

AAATACGAAAGTAGCTCTAAATATAATCCTGACGCCACGAAAGCCAAGAAA

>186_Sample_ASV4

CACCGCGGTTATACGAAAGACCCTAGTTGATGGGTGCGGCGTAAAGGGTGGTTAGGGACT

ACAAAAAATAGAGCTAAAGATCCTCTAGGTCGTCATACACATCACGAGGAAACGAAGCCC

AAATACGAAAGTAGCTCTAAATATAATCCTGACGCCACGAAAGCTGTGAAA

>186_Sample_ASV5

CACCGCGGTTATACGAAAGACCCTAGTTGATAACTACGGCATAAAGGGTGGTTAGGGAGT

TTAAAAAATAAAGCCAAAGCTCTTCTAAGCTGTCATACGCTCTCTGAAAGAACGAGGCCC

AAACACGAAAGTAGCTTTAAATAAATAACCTGATACCACGAAAGCCAAGAAA

>186_Sample_ASV6

CACCGCGGTTATACGAGAGACCCTAGTTGATTAAAGCGGCGTAAAGAGTGGTTATGGAAC

TATTCTTTTAAAGCAGAAAACCTCTCAAACTGTTATACGCACCCAGAGGTCTGAATCCCT

CACACGAAAGTGACTTTATTTATGCCTACCAGAACCCACGAAGGCTGGGACG

>186_Sample_ASV7

CACCGCGGTTATACGAGAGACCCTAGTTGATTAAAGCGGCGTAAAGAGTGGTTATGGAAC

TATTCTTTTAAAGCAGAAAACCTCTCAAACTGTTATACGCACCCAGAGGTCTGAATCCCT

CACACGAAAGTGACTTTATTTATGCCTACCAGAACCCACGAAAGCTGGGACA

>186_Sample_ASV8

CACCGCGGTTATACGAAAGACCCTAGTTTATAGGTACGGCGTAAAGGGTGGTTAGGGGAC

AAACAAAATAAAGCCAAAGACCTCCTAAGCCGTCATACGCTCACGGAAGCACGAAGCCCA

AACACGAAAGTAGCTTTACCGAACATGCCCGACTCCACGAAAGCTGGGGAA

>186_Sample_ASV9

CACCGCGGTTATACGAAAGACCCTAGTTGATAGCCACGGCGTAAAGGGTGGTTAAGGAAC

CTAAAAAATAAAGCTAAAGACCCTCTAGGCCGTCATACGCATTACGAGGCAAACGAAACC

CCAACACGAAAGTAGCTTTAGACAAATATCCTGATGCCACGAAAGCCAAGAAA

>186_Sample_ASV10

CACCGCGATTATACGAGAGGCCCAAGTTGACAGACATCGGCGTAAAGAGTGGTTAAGGAA

AAATTACTACTAAAGCCGAACACCTTCAGAACTGTCATACGTTCCCGAAGATAAGAAGCC

CCACCACGAAAGTGGCTTTATATTACCCGACCCCACGAAAGCTGTGAAA

>186_Sample_ASV11

CACCGCGGTTATACGAGAGGCCCAAGTTGACAGACATCGGCGTAAAGAGTGGTTAAGGAA

AAATTACTGCTAAAGCCGAACACCCTCAGAACTGTCATACGTTCCCGAAGATAAGAAGCC

CCACCACGAAAGTGGCTTTATATTACCCGACCCCACGAAAGCTGTGAAA

>186_Sample_ASV12

CACCGCGGTTATACGAGAGGCCCAAGTTGACAGACACCGGCGTAAAGAGTGGCTAGGGAA

AAATTACTACTAAAGCCGAACACCTTCAGAACTGTCGTACGTTTCCGAGAGTATGAAGCC

CCACCACGAAAGTGGCTTTATATTACCCGACCCCACGAAAGCTGTGAAA

>186_Sample_ASV13

CACCGCGGTTATACGAGAGGCCCAAGTTGACAGACACCGGCGTAAAGAGTGGCTAGGGAA

AAATTACTACTAAAGCCGAACACCTTCAGAACTGTCATACGTTCCCGAAGATAAGAAGCC

CCACCACGAAAGTGGCTTTATATTACCCGACCCCACGAAAGCTGTGAAA

>186_Sample_ASV14

CACCGCGGTTATACGAAAGACTCTAGTTGACAAACACGGCGTAAAGGGTGGTTAGGATAA

CAACAAAATAAAGCTAAAGACTTACCAAGCCGTCATACGCCCATGTAAGAGCGAAAACCA

CAAACGAAAGTAACTTTATTACACCATCCGAATCCACGAAAGCTAAGAAA

>186_Sample_ASV15

CACCGCGGTTATACGAAAGACCCTAGTTTATAGAAACGGCGTAAAGGGTGGTTAAGGGAC

AAACAAAATAAGGCCAAAGACTTCCTAAGCCGTCATACGCTCCACGAAAGCACGAAGCCC

AAACACGAAAGTAGCCTTAATAACTTCACCTGACCCCACGAAAGCTGTGAAA

>186_Sample_ASV16

CACCGCGGTTATACGAAAGACCCTAGTTTATAGAAACGGCGTAAAGGGTGGTTAAGGGAC

AAACAAAATAAGGCCAAAGACTTCCTAAGCCGTCATACGCTCCACGAAAGCACGAAGCCC

AAACACGAAAGTAGCCTTAATAACTTCACCTGACCCCACGAAAGCTGGGAGA

>186_Sample_ASV17

CACCGCGGTTATACGAGAGGCCCAAGTTGATAAGCCCGGCGTAAAGAGTGGTTATGGAGA

ATAAAACACTAAAGCTAAAGACCCCCTAGGCTGTTATACGCACCTGGCGGCTCGAACCAC

CTATACGAAAGTAGCTTTACCCCCCTTCCACCAGAATCCACGACAGCTGGGGCA

>186_Sample_ASV18

CACCGCGGTTATACGAGAGGCCCAAGTTGATAAGCCCGGCGTAAAGAGTGGTTATGGAGA

ATAAAACACTAAAGCTAAAGACCCCCTAGGCTGTTATACGCACCTGGCGGCTCGAACCAC

CTATACGAAAGTAGCTTTACCCCCCTTCCACCAGAATCCATGACAGCTGGGGCA

>186_Sample_ASV19

CACCGCGGTTATACGAGAGGCCCAAGTTGACAGACATCGGCGTAAAGAGTGGTTAAGGAA

ACCCCATTACTAAAGCCGAACGCCCTCAGAACTGTCATACGTTCCCGAAGGTAAGAAGCC

CCACTACGAAAGTGGCTTTAACCACACCCGACCCCACGAAAGCTGTGAAA

>186_Sample_ASV20

CACCGCGGTTATACGAGAGGCCCAAGTTGATAGATATCGGCGTAAAGAGTGGTTAAGGAA

ACCCCGTTACTAAAGCCGAACGCCCTCAGAACTGTCATACGTTCCCGAAGGTAAGAAGCC

CCACTACGAAAGTGGCTTTAACCACACCCGACCCCACGAAAGCTGTGAAA

>186_Sample_ASV21

CACCGCGGTTATACGAGAGGCCCAAGTTGACAGACACCGGCGTAAAGAGTGGTTAAGGAA

ATTCATCAACTAAAGCCGAATGCCCTCAGGACTGTTATACGTTTGCGAAGGTAAGAAGCC

CTACTACGAAAGTGGCTTTATTCCACCTGATTCCACGAAAGCTGCGGAA

>186_Sample_ASV22

CACCGCGGTTATACGAGAGGCCCCAGTTGATATTAGCGGCGTAAAGGGTGGTTAAGGACT

TTAATAACTAAAGCTAAACACTTCCCCGGCTGTTGTACGTAGACCGGAAGCTTGAAGCCC

AGACACGAAAGTAGCTTTATTCCCCCTGACCCCACGAAAGCTGAGAAA

>186_Sample_ASV23

CACCGCGGTCACACGATTAACCCAAGTCAATAGAAGCCGGCGTAAAGAGTGTTTTAGATC

ACCCCCTCCCCAATAAAGCTAAAACTCACCTGAGTTGTAAAAAACTCCAGTTGACACAAA

ATAGACTACGAAAGTGGCTTTAACATATCTGAACACACAATAGCTAAGACC

>186_Sample_ASV24

CACCGCGGTTATACGAGAAACCCTAGTTGACAAATACGGCGTAAAGAGTGGTTAAGGGGT

ATCAAAAATAAAGTCAAACATTTTCCTAGTTGTTATAAGCATTTCGAGAACATGAAGCCC

AACCACGAAAGTAACTTTACCTTCACCTGACCCCACGAAAGCTAAGAAA

>186_Sample_ASV25

CACCGCGGTTATACGAGAGACCCTAGTTGATAAATACGGCGTAAAGAGTGGTTATGGGAA

AAACAAATAAAGTCAAACAACCTCTTAGCTGTTATACGCATTATGAGAGCATGAAGCCCC

CTCACGAAAGTAACTTTAATACCTCCTGACCCCACGAAAGCTAAGAAA

>186_Sample_ASV26

CACCGCGGTTATACGAGAGACCCTAATTGATAGGTACGGCGTAAAGAGTGGTTTGGGATA

ACCCAAAAATAAAGCTAAAGACCTTCTAAGCCGTCACACGCATACCGAAGGCACGAGGCC

CCAACACGAAAGTAGCTTTACTATTACCCCGACGCCACGAAAGCTAAGAAA

>186_Sample_ASV27

CACCGCGGTTATACGAGAGGCCCTAGTTGATAACTACCGGCGTAAAGAGTGGTTATGGAA

AAATATTTAATAAAGCCGAACACCCCCTCAGCCGTCATACGCACCTGGGAGCACGAAGAC

CTACTGCGAAAGCAGCTTTAATTATGCCTGACCCCACGACAGCTAAGAAA

>186_Sample_ASV28

CACCGCGGTTATACGAGAGACCCTAATTGATAGGCGCGGCGTAAAGAGTGGTTTAGGACT

AAAACTAATAAAGCTAAAGATCCTCCCAGCCGTCGCACGCATTCTGAGGACACGAAACTC

AACCACGAAAGTAGCTTTAACATACTCTCCTGACCCCACGAAAGCTAAGAAA

>186_Sample_ASV29

CACCGCGGTTATACGAGAGGCCCAAGTTGATACTCCACGGCGTAAAGAGTGGTTAGAAAA

GACCTACTACTAAAGCCGAACGCCTTCAAAGCTGTTATACGCATCCGAAGGTGAGAAGCC

CACCCACGAAAGTGGCTTTACAACCTTGACTCCACGAAAGCTATGACA

>186_Sample_ASV30

CACCGCGGTTATACGAGAGACCCCAGTTGATAAACACGGCGTAAAGAGTGGTTAAGGGCA

CAAAACAAATAAAGTCAAACAACCTCTTAGCTGTTATACGCATTATGAGGATATGAAGCC

CCCCTACGAAAGTAACTTTACCAACCCCTGACCCCACGAAAGCTAAGAAA

>186_Sample_ASV31

CACCGCGGTTATACGAAAGACCCTAGTTTATAGGCACGGCGTAAAGGGTGGTTAGGGGAT

AAACAAAATAAAGCCAAAGACCTTCTAAGTCGTCATACACTCATGAAGGCACGAAGCCCA

AATACGAAAGTAACTTTACAACTTAGACCCGACTCCACGAAAGCTGGGGGA

>186_Sample_ASV32

CACCGCGGTTATACGAGAGACCCTAATTAATAGATACGGCGTAAAGAGTGGTTTAGGACA

AAAATTAATAAAGCCGAAGTTTCTCCTAGCCGTTGTACGCACTCTGAGAACACGAAGCCC

CACTACGAAAGTAGCTTTACCTATTATTTCCTGACCCCACGAAAGCTAAGAAA

>186_Sample_ASV33

CACCGCGGTTATACGAGAGGCCCAAGTTGATAGACATCGGCGTAAAGAGTGGTTAAGGAA

GTCTCAAAACTAAAGCCGAATGCCTTCAAGACTGTTATACGTTTCCGAAGGTAAGAAGCC

CCACTACGAAAGTGGCTTTACTCCACCTGACTCCACGAAAGCTGTGAAA

>186_Sample_ASV34

CACCGCGGTTATACGAGAGACTCCAGTTGATAGCTACGGCGTAAAGAGTGGTTTGGGGCC

CGCCCAAAATAAAGCCAAAGACCTCCCAAGCCGTCAAACGCACCCCGGAGGCACGAAGTC

CTAACGCGAAAGCAACTTTACCTTCCCCGACGCCACGAAAGCTAAGAAA

>186_Sample_ASV35

CACCGCGGTTATACGAAAGACCCTAGTTGATAGCTACGGCGTAAAGGGTGGTTAAGGTAT

ATAACAAATAAAGCTAAAGAGCCTCCAAGCCGTCGTACGCATTCTGAGAGCTCGAAACCC

TAACACGAAAGTAGCTTTAAAAAATAACACCTGACCCCACGAAAGCTAAGAAA

>186_Sample_ASV36

CACCGCGGTTATACGAAAGACCCTAGTCGATAGCTACGGCGTAAAGGGTGGTTAAGGTAT

ATAACAAATAAAGCTAAAGAGCCTCCAAGCCGTCGTACGCATTCTGAGAGCTCGAAACCC

TAACACGAAAGTAGCTTTAAAAAATAACACCTGACCCCACGAAAGCTAAGAAA

>186_Sample_ASV37

CACCGCGGTTATACGAAAGACCCTAGTTGATAGATCACGGCGTAAAGGGTGGTTAAGGAG

AACAAAAATAAAGCTAAAAATCCTCTAAGCTGTCATACGCTTTCCGAAGACATGAGACCC

AACCACGAAAGTAACTTTAAACTTTCCTGACACCACGAAAGCCAAGAAA

>196_Sample_ASV1

CACCGCGGTTATACGAGAGGCCCAAGTTGACAGACATCGGCGTAAAGAGTGGTTAAGGAA

AACCTTCAACTAAAGTCGAACGCTCTCAAGACTGTCGTACGTTTCCGAGAGTATGAAGCC

CCACCACGAAAGTGACTTTACCTCACCTGACTCCACGAAAGCTGCGAAA

>196_Sample_ASV2

CACCACGGTTATACGAGAGGCCCAAGTTGAAAGACATCGGCGTAAAGAGTGGTTAAGGAA

AACCTTCTACTAAAGTCGAACGCTCTCAGGACTGTCGTACGTTTCCGAGAGTATGAAGCC

CCACCACGAAAGTGACTTTACCTCACCTGACTCCACGAAAGCTGCGAAA

>196_Sample_ASV3

CACCGCGGTTATACGAAAGACCCTAGTTGATGGGTGCGGCGTAAAGGGTGGTTAGGGACT

ACAAAAAATAGAGCTAAAGATCCTCTAGGTCGTCATACACATCACGAGGAAACGAAGCCC

AAATACGAAAGTAGCTCTAAATATAATCCTGACGCCACGAAAGCCAAGAAA

>196_Sample_ASV4

CACCGCGGTTATACGAGAGACCCTAGTTGATTAAAGCGGCGTAAAGAGTGGTTATGGAAC

TATTCTTTTAAAGCAGAAAACCTCTCAAACTGTTATACGCACCCAGAGGTCTGAATCCCT

CACACGAAAGTGACTTTATTTATGCCTACCAGAACCCACGAAAGCTGGGACA

>196_Sample_ASV5

CACCGCGGTTATACGAAAGACCCTAGTTTATAGGTACGGCGTAAAGGGTGGTTAGGGGAC

AAACAAAATAAAGCCAAAGACCTCCTAAGCCGTCATACGCTCACGGAAGCACGAAGCCCA

AACACGAAAGTAGCTTTACCGAACATGCCCGACTCCACGAAAGCTGGGGAA

>196_Sample_ASV6

CACCGCGGTTATACGAGAGGCCCAAGTTGACAGACACCGGCGTAAAGAGTGGCTAGGGAA

AAATTACTACTAAAGCCGAACACCTTCAGAACTGTCATACGTTCCCGAAGATAAGAAGCC

CCACCACGAAAGTGGCTTTATATTACCCGACCCCACGAAAGCTGTGAAA

>196_Sample_ASV7

CACCGCGGTTATACGAGAGGCCCAAGTTGACAGACACCGGCGTAAAGAGTGGCTAAGGAA

AAATTTCTACTAAAGCCGAACACCTTCAGAACTGTCATACGTTCCCGAAGAAAAGAAGCC

CCACCACGAAAGTGGCTTTATATTACCCGACCCCACGAAAGCTGTGAAA

>196_Sample_ASV8

CACCGCGGTTAAACGAGAGACCCTAGTTGATCATCACGGCGTAAAGAGTGGTTAGGGGAT

TATCATAAATAAAGCCAAAGACCTCCCAAGCTGTCGCACGCATTCCGGAGGCGCGAAGCC

CACACACGAAAGTAGCTTTAACTATTGACCCTGATGCCACGAAAGCTAAGAAA

>196_Sample_ASV9

CACCGCGGTTATACGAGAGGCCCAAGTTGATAAGCCCGGCGTAAAGAGTGGTTATGGAGA

ATAAAACACTAAAGCTAAAGACCCCCTAGGCTGTTATACGCACCTGGCGGCTCGAACCAC

CTATACGAAAGTAGCTTTACCCCCCTTCCACCAGAATCCACGACAGCTGGGGCA

>196_Sample_ASV10

CACCGCGGTTATACGAGAGGCCCAAGTTGACAGACATCGGCGTAAAGAGTGGTTAAGGAA

ACCCCATTACTAAAGCCGAACGCCCTCAGAACTGTCATACGTTCCCGAAGGTAAGAAGCC

CCACTACGAAAGTGGCTTTAACCACACCCGACCCCACGAAAGCTGTGAAA

>196_Sample_ASV11

CACCGCGGTTATACGAGAGGCCCTAGTTGATAAATACGGCGTAAAGAGTGGTTAGAGGAC

TCCCATAATAAAGCCAAAGACCCCCCGGGCCGTCACACGCATACGAGGGCACGAAGTTCA

CACACGAAAGTAGCTTTAAATTACCCGACGCCACGAAAGCTAAGAAA

>196_Sample_ASV12

GACCGCGGTTATACGAGAGACTCTAGTTGATAGATACGGCGTAAAGAGTGGTTTGGGATA

AACTATAAATAAAGCCAAAGACCTTCCAAGCTGTTAAACGCATACCGAAGGCACGAAGCC

CCAACACGAAAGTAGCTTTACTACTACCCCCGATGCCACGAAAGCTAAGACA

>196_Sample_ASV13

CACCGCGGTCATACGATTAACCCAAGTTAATAGGCACACGGCGTAAAGCGTGTTTAAGCA

CTATGCCAAATAAAGTTAAACTCCAATTAAGCTGTAAAAAGCCATAATCAAGACGAAAAT

AAATAACGAAAGTAACTTTACAACCGCTGAAACACGATAGCTAAGATC

>196_Sample_ASV14

CACCGCGGTTATACGAAAGACCCTAGTTTATAGACACGGCGTAAAGGGTGGTTCGGGACT

AAACAAAATAAAGCCAAAGACCTCCTAAGCCGTCATACGCTCACGGAAGCACGAAGCCCA

AGTACGAAAGTAGCTTTACAAAACATGCCTGACTCCACGAAAGCTAGGAAA

>196_Sample_ASV15

CACCGCGGTTATACGAAAGACCCTAGTTGATAGGCACGGCGTAAAGGGTGGTTAGGGATA

TATCAAAAATAAAGTTAAAGAGCCTCTAAGCTGTCGCACGCATCCCGAGAGCTCGAGACC

CAGACACGAAAGTAACTTTAAAATACCTCACCTGACCCCACGAAAGCTAAGAAA

>196_Sample_ASV16

CACCGCGGTTATACGAAAGACCCTAGTTGATAGCCACGGCGTAAAGGGTGGTTAAGGTAT

ACAACAAATAAAGCTAAATAGCCTCTAGGCCGTCGCACGCATTCCGAGAGCTCGAAACCC

AAAAACGAAAGTAGCTTTAAAATAAACAGACCTGACCCCACGAAAGCTAAGAAA

>196_Sample_ASV17

CACCGCGGTTATACGAGAGGCCCAAGTTGATACTCCACGGCGTAAAGAGTGGTTAGAAAA

GACCTACTACTAAAGCCGAACGCCTTCAAAGCTGTTATACGCATCCGAAGGTGAGAAGCC

CACCCACGAAAGTGGCTTTACAACCTTGACTCCACGAAAGCTATGACA

>196_Sample_ASV18

CACCGCGGTTATACGAGAGACCCCAGTTGATAAACACGGCGTAAAGAGTGGTTAAGGGCA

CAAAACAAATAAAGTCAAACAACCTCTTAGCTGTTATACGCATTATGAGGATATGAAGCC

CCCCTACGAAAGTAACTTTACCAACCCCTGACCCCACGAAAGCTAAGAAA

>196_Sample_ASV19

CACCGCGGTTATACGAAAGACCCTAGTTTATAGGCACGGCGTAAAGGGTGGTTAGGGGAT

AAACAAAATAAAGCCAAAGACCTTCTAAGTCGTCATACACTCATGAAGGCACGAAGCCCA

AATACGAAAGTAACTTTACAACTTAGACCCGACTCCACGAAAGCTGGGGGA

>196_Sample_ASV20

CACCGCGGTTATACGAAAGACCCTAGTTGATAGATCACGGCGTAAAGGGTGGTTAAGGAG

AACAAAAATAAAGCTAAAAATCTTCTAAGCTGTCATACGCTTTCCGAAGACATGAGACCC

AACCACGAAAGTAGCTTTAAACTTTCCTGACACCACGAAAGCCAAGAAA

>198_Sample_ASV1

TACCGCGGTTATACGAAAGACCCAAGTTAATAAACTACGGCGTAAAGCGTGGTTAGAGAC

CTCCAATAATTAAAGCCAAAGACCTTCTAAGCCGTCGCACGCCCCGCGAAGACACGAAGC

CCAAAAACGAAAGTAGCTTTATTCTTACTCGACCCCACGAAAGCTAGGGCA

>198_Sample_ASV2

CACCGCGGTTATACGAAAGACCCTAGTTGATGGGTGCGGCGTAAAGGGTGGTTAGGGACT

ACAAAAAATAGAGCTAAAGATCCTCTAGGTCGTCATACACATCACGAGGAAACGAAGCCC

AAATACGAAAGTAGCTCTAAATATAATCCTGACGCCACGAAAGCCAAGAAA

>198_Sample_ASV3

CACCGCGGTTATACGAGAGACCCTAGTTGATTAAAGCGGCGTAAAGAGTGGTTATGGAAC

TATTCTTTTAAAGCAGAAAACCTCTCAAACTGTTATACGCACCCAGAGGTCTGAATCCCT

CACACGAAAGTGACTTTATTTATGCCTACCAGAACCCACGAAAGCTGGGACA

>198_Sample_ASV4

CACCGCGATTATACGAGAGGCCCAAGTTGACAGACATCGGCGTAAAGAGTGGTTAAGGAA

AAATTACTACTAAAGCCGAACACCTTCAGAACTGTCATACGTTCCCGAAGATAAGAAGCC

CCACCACGAAAGTGGCTTTATATTACCCGACCCCACGAAAGCTGTGAAA

>198_Sample_ASV5

CACCGCGGTTATACGAGAGGCCCAAGTTGACAGACACCGGCGTAAAGAGTGGCTAGGGAA

AAATTACTACTAAAGCCGAACACCTTCAGAACTGTCATACGTTCCCGAAGATAAGAAGCC

CCACCACGAAAGTGGCTTTATATTACCCGACCCCACGAAAGCCAAGAAA

>198_Sample_ASV6

CACCGCGGTTATACGAGAGGCCCAAGTTGACAGACACCGGCGTAAAGAGTGGCTAGGGAA

AAATTACTACTAAAGCCGAACACCTTCAGAACTGTCATACGTTCCCGAAGATAAGAAGCC

CCACCACGAAAGTGGCTTTATATTACCCGACCCCACGAAAGCTGTGAAA

>198_Sample_ASV7

CACCGCGGTTATACGAGAGGCCCAAGTTGACAGACACCGGCGTAAAGAGTGGCTAAGGAA

AAATTTCTACTAAAGCCGAACACCTTCAGAACTGTCATACGTTCCCGAAGAAAAGAAGCC

CCACCACGAAAGTGGCTTTATATTACCCGACCCCACGAAAGCTGTGAAA

>198_Sample_ASV8

CACCGCGGTTATACGAAAGACTCTAGTTGACAAACACGGCGTAAAGGGTGGTTAGGATAA

CAACAAAATAAAGCTAAAGACTTACCAAGCCGTCATACGCCCATGTAAGAGCGAAAACCA

CAAACGAAAGTAACTTTATTACACCATCCGAATCCACGAAAGCTAAGAAA

>198_Sample_ASV9

CACCGCGGTTATACGAAAGACCCTAGTTGATAGATCACGGCGTAAAGGGTGGTTAGGGAA

TACAAAAATAAAGCTAAAAATCCTCTAAGCTGTCATACGCTTCCCGAAGACATGAAACCC

AACCACGAAAGTAGCTTTAAACTCTCCTGACACCACGAAAGCCAAGGAA

>198_Sample_ASV10

CACCGCGGTTATACGAAAGACCCTAGTTTATAGGCACGGCGTAAAGGGTGGTTAGGGAAT

ACAAAAATAAAGCTAAAAATCCTCTAAGCTGTCATACGCTTCCCGAAGACATGAAACCCA

ACCACGAAAGTAGCTTTAAACTCTCCTGACACCACGAAAGCCAAGGAA

>198_Sample_ASV11

CACCGCGGTTATACGAGAGGCCCAAGTTGATAAGCCCGGCGTAAAGAGTGGTTATGGAGA

ATAAAACACTAAAGCTAAAGACCCCCTAGGCTGTTATACGCACCTGGCGGCTCGAACCAC

CTATACGAAAGTAGCTTTACCCCCCTTCCACCAGAATCCACGACAGCTGGGGCA

>198_Sample_ASV12

CACCGCGGTTATACGAGAGGCCCAAGTTGACAGACATCGGCGTAAAGAGTGGTTAAGGAA

ACCCCATTACTAAAGCCGAACGCCCTCAGAACTGTCATACGTTCCCGAAGGTAAGAAGCC

CCACTACGAAAGTGGCTTTAACCACACCCGACCCCACGAAAGCTGTGAAA

>198_Sample_ASV13

CACCGCGGTTATACGAGAGGCCCCAGTTGATATTAGCGGCGTAAAGGGTGGTTAAGGACT

TTAATAACTAAAGCCAAACACTTCCCCGGCTGTTGTACGTAGACCGGAAGCTTGAAGCCC

AGACACGAAAGTAGCTTTATTTCCCCTGACCCCACGAAAGCTGAGAAA

>198_Sample_ASV14

CACCGCGGTTATACGAGAGGCCCCAGTTGATATTAGCGGCGTAAAGGGTGGTTAAGGACT

TTAATAACTAAAGCTAAACACTTCCCCGGCTGTTGTACGTAGACCGGAAGCTTGAAGCCC

AGACACGAAAGTAGCTTTATTTCCCCTGACCCCACGAAAGCTGAGAAA

>198_Sample_ASV15

CACCGCGGTTATACGAGAGGCCCCAGTTGATATTAGCGGCGTAAAGGGTGGTTAAGGACT

TTAATAACTAAAGCTAAACACTTCCCCGGCTGTTGTACGTAGACCGGAAGCTTGAAGCCC

AGACACGAAAGTAGCTTTATTCCCCCTGACCCCACGAAAGCTGAGAAA

>198_Sample_ASV16

CACCGCGGTCACACGATTAACCCAAGTCAATAGAAGCCGGCGTAAAGAGTGTTTTAGATC

ACCCCCTCCCCAATAAAGCTAAAACTCACCTGAGTTGTAAAAAACTCCAGTTGACACAAA

ATAGACTACGAAAGTGGCTTTAACATATCTGAACACACAATAGCTAAGACC

>198_Sample_ASV17

CACCGCGGTTATACGAAAGACCCCAGTTTATAGACACGGCGTAAAGGGTGGTTATAGGAC

AAACTAACATAGAGCCAAAGACCCCCTAAGCCGTCATACGCTCACGAGGGCTCGAAGCCC

CTATACGAAAGTAGCTCTACCAAAAAATGCTCGACTCCACGAAAGCTGGGACA

>198_Sample_ASV18

CACCGCGGTTATACGAGAGACCCTAGTTGATAAATACGGCGTAAAGAGTGGTTATGGGAA

AAACAAATAAAGTCAAACAACCTCTTAGCTGTTATACGCATTATGAGAGCATGAAGCCCC

CTCACGAAAGTAACTTTAATACCTCCTGACCCCACGAAAGCTAAGAAA

>198_Sample_ASV19

CACCGCGGTTATACGAGAGACCCTAATTGATAGGCACGGCGTAAAGAGTGGTTTAGGGTA

ACCCATAAATAAAGCTAAAGACCTTCTAAGCCGTCACACGCACACCGAAGGCACGAGACC

CCAACACGAAAGTAGCTTTACTTCTACCCCTGACGCCACGAAAGCTAAGAAA

>198_Sample_ASV20

CACCGCGGTTATACGAAAGACCCTAGTTGATGGATCACGGCGTAAAGGGTGGTTAAGGGA

TATAAAAATAAAGCTAAAAATCCTCTTAGCTGTCATACGCTTTCCGAAGACATGAGGCCC

AATTACGAAAGTAGCTTTAAACCACCCTGACACCACGAAAGCCAAGAAA

>198_Sample_ASV21

CACCGCGGTTATACGAAAGACCCCAGTTTATAGATGCGGCGTAAAGGGTGGTTAAGAGAA

AATCAAAATAAGGCCAAAGATCCCCTAAGCAGTCATACGCTCCACGAGGACACGAAACCC

AAATACGAAAGTTGCCTTAATAAACTTCACCTGATCCCACGAAAGCTGAAAAA

>198_Sample_ASV22

CACCGCGGTTATACGAAAGGCTCTAGTTTATAAATACGGCGTAAAGGGTGGTTAAGGAAA

AATTAAAATAATGCTAAAGACCCCCTAAGCAGTTATACGCTTCACGGAGGCACGAAACCC

AAATACGAAAGTTGCCTTAATAAACTTTACCTGACCCCACGAAAGCTGAGAAA

>198_Sample_ASV23

CACCGCGGTTATACGAAAGGCTCTAGTTTATAAATACGGCGTAAAGGGTGGTTAAGGAAA

AATTAAAATAATGCTAAAGACCCCCTAAGCAGTTATACGCTTCACGGAGGCACGAAACCC

AAATACGAAAGTTGCCTTAATAAACTTTACCTGACCCCACGAAAGCTGAAAAA

>198_Sample_ASV24

CACCGCGGTTATACGAGAGACTCTAGTTGATACCAACGGCGTAAAGAGTGGTTTGGGATG

CCCCTTAAAATAAAGCCAAAGACCTCCCAAGCCGTCAAACGCACCCCGGAGGCACGAAGC

CCCAACGCGAAAGCAACTTTACCTCCCTCCCGACGCCACGAAAGCTAAGAAA

>198_Sample_ASV25

CACCGCGGTTATACGAGAGACCCTAGTTGATAGCTACGGCGTAAAGCGTGGTTAGGAGAA

CAAGATAAATAAAGTCAAACAATCTCTCGGCCGTTATACGTTATCTGAGAATATGAAGTC

CTACCACGAAAGTAACTTTAATTTTTCTGACCCCACGAAAACTGAGGAA

>198_Sample_ASV26

CACCGCGGTCATACGATTAACCCAAGTTAATAGGCACACGGCGTAAAGCGTGTTTAAGCA

CTACGCCAAATAAAGTTAAACTCCAATTAAGCTGTAAAAAGCCATAATCAAGACGAAAAT

AAATAACGAAAGTAACTTTACAACCGCTGAAACACGATAGCTAAGATC

>198_Sample_ASV27

CACCGCGGTTATACGAGAGGCCCTAGTTGATAACTACCGGCGTAAAGAGTGGTTATGGAA

AAATATTTAATAAAGCCGAACACCCCCTCAGCCGTCATACGCACCTGGGAGCACGAAGAC

CTACTGCGAAAGCAGCTTTAATTATGCCTGACCCCACGACAGCTAAGAAA

>198_Sample_ASV28

CACCGCGGTTATACGAGAGGCCCAAGTTGATTATTACCGGCGTAAAGCGTGGTTAAGATT

TTTATAGGACTAAAGCCGAACCCCCTCAGAGCTGTTATACGCACCCGAAGGTTAGAAGTT

CAATCACGAAAGTGGCTTTACAACCCCGAACCCACGAAAGCTAAGATA

>198_Sample_ASV29

CACCGCGGTTATACGAGAGGCCCAAGTTGATACTCCACGGCGTAAAGAGTGGTTAGAAAA

GACCTACTACTAAAGCCGAACGCCTTCAAAGCTGTTATACGCATCCGAAGGTGAGAAGCC

CACCCACGAAAGTGGCTTTACAACCTTGACTCCACGAAAGCTATGACA

>198_Sample_ASV30

CACCGCGGTTATACGAGAGACCCCAGTTGATAAACACGGCGTAAAGAGTGGTTAAGGGCA

CAAAACAAATAAAGTCAAACAACCTCTTAGCTGTTATACGCATTATGAGGATATGAAGCC

CCCCTACGAAAGTAACTTTACCAACCCCTGACCCCACGAAAGCTAAGAAA

>198_Sample_ASV31

CACCGCGGTTATACGAAAGACCCTAGTTTATAGGCACGGCGTAAAGGGTGGTTAGGGGAT

AAACAAAATAAAGCCAAAGACCTTCTAAGTCGTCATACACTCATGAAGGCACGAAGCCCA

AATACGAAAGTAACTTTACAACTTAGACCCGACTCCACGAAAGCTGGGGGA

>198_Sample_ASV32

CACCGCGGTTATACGAAAGACCCTAGTTTATAGGCACGGCGTAAAGGGTGGTTAGGGGAT

AAACAAAATAAAGCCAAAGACCTTCTAAGTCGTCATACACTCATGAAGGCACGAAGCCCA

AATACGAAAGTAACTTTACAACTTAGACCCGACTCCACGAAAGCCAAGGAA

>198_Sample_ASV33

CACCGCGGTTATACGAAAGACCCTAGTTGATAGCTACGGCGTAAAGGGTGGTTAAGGTAT

ATAACAAATAAAGCTAAAGAGCCTCCAAGCCGTCGTACGCATTCTGAGAGCTCGAAACCC

TAACACGAAAGTAGCTTTAAAAAATAACACCTGACCCCACGAAAGCTAAGAAA

>198_Sample_ASV34

CACCGCGGTTATACGAAAGACCCTAGTTGATAGATCACGGCGTAAAGGGTGGTTAAGGAG

AACAAAAATAAAGCTAAAAATCTTCTAAGCTGTCATACGCTTTCCGAAGACATGAGACCC

AACCACGAAAGTAGCTTTAAACTTTCCTGACACCACGAAAGCCAAGAAA

>199_Sample_ASV1

CACCGCGGTTATACGAGAGGCCCAAGTTGACAGACATCGGCGTAAAGAGTGGTTAAGGAA

AACCTTCAACTAAAGTCGAACGCTCTCAAGACTGTCGTACGTTTCCGAGAGTATGAAGCC

CCACCACGAAAGTGACTTTACCTCACCTGACTCCACGAAAGCTGCGAAA

>199_Sample_ASV2

TACCGCGGTTATACGAAAGACCCAAGTTAATAAACTACGGCGTAAAGCGTGGTTAGAGAC

CTCCAATAATTAAAGCCAAAGACCTTCTAAGCCGTCGCACGCCCCGCGAAGACACGAAGC

CCAAAAACGAAAGTAGCTTTATTCTTACTCGACCCCACGAAAGCTAGGGCA

>199_Sample_ASV3

CACCGCGGTTATACGAAAGACCCTAGTTGATGGGTGCGGCGTAAAGGGTGGTTAGGGACT

ACAAAAAATAGAGCTAAAGATCCTCTAGGTCGTCATACACATCACGAGGAAACGAAGCCC

AAATACGAAAGTAGCTCTAAATATAATCCTGACGCCACGAAAGCCAAGAAA

>199_Sample_ASV4

CACCGCGGTTATACGAGAGACCCTAGTTGATTAAAGCGGCGTAAAGAGTGGTTATGGAAC

TATTCTTTTAAAGCAGAAAACCTCTCAAACTGTTATACGCACCCAGAGGTCTGAATCCCT

CACACGAAAGTGACTTTATTTATGCCTACCAGAACCCACGAAAGCTGGGACA

>199_Sample_ASV5

CACCGCGGTTATACGAGAGACCCTAGTTGATTAAAGCGGCGTAAAGAGTGGTTATGGAAC

TATTCTTTTAAAGCAGAAAACCTCTCAAACTGTTATACGCACCCAGAGGTCTGAATCCCT

CACACGAAAGTGACTTTATTTATGCCTACCAGAACCCACGAAAGCTATGACA

>199_Sample_ASV6

CACCGCGGTTATACGAAAGACCCTAGTTTATAGGTACGGCGTAAAGGGTGGTTAGGGGAC

AAACAAAATAAAGCCAAAGACCTCCTAAGCCGTCATACGCTCACGGAAGCACGAAGCCCA

AACACGAAAGTAGCTTTACCGAACATGCCCGACTCCACGAAAGCTGGGGAA

>199_Sample_ASV7

CACCGCGGTTATACGAGAGGCCCAAGTTGACAGACACCGGCGTAAAGAGTGGCTAGGGAA

AAATTACTACTAAAGCCGAACACCTTCAGAACTGTCATACGTTCCCGAAGATAAGAAGCC

CCACCACGAAAGTGGCTTTATATTACCCGACCCCACGAAAGCTGTGAAA

>199_Sample_ASV8

CACCGCGGTTATACGAGAGGCCCAAGTTGACAGACACCGGCGTAAAGAGTGGCTAGGGAA

AAATTACTACTAAAGCCGAACACCTTCAGAACTGTCATACGTTCCCGAAGATAAGAAGCC

CCACCACGAAAGTGGCTTTATATTACCCGACCCCACGAAAGCCAAGAAA

>199_Sample_ASV9

CACCGCGGTTATACGAGAGGCCCAAGTTGACAGACACCGGCGTAAAGAGTGGCTAAGGAA

AAATTTCTACTAAAGCCGAACACCTTCAGAACTGTCATACGTTCCCGAAGAAAAGAAGCC

CCACCACGAAAGTGGCTTTATATTACCCGACCCCACGAAAGCTGTGAAA

>199_Sample_ASV10

CACCGCGGTTAAACGAGAGACCCTAGTTGATCATCACGGCGTAAAGAGTGGTTAGGGGAT

TATCATAAATAAAGCCAAAGACCTCCCAAGCTGTCGCACGCATTCCGGAGGCGCGAAGCC

CACACACGAAAGTAGCTTTAACTATTGACCCTGATGCCACGAAAGCTAAGAAA

>199_Sample_ASV11

CACCGCGGTTATACGAGAGGCCCAAGTTGATAAGCCCGGCGTAAAGAGTGGTTATGGAGA

ATAAAACACTAAAGCTAAAGACCCCCTAGGCTGTTATACGCACCTGGCGGCTCGAACCAC

CTATACGAAAGTAGCTTTACCCCCCTTCCACCAGAATCCACGACAGCTGGGGCA

>199_Sample_ASV12

CACCGCGGTTATACGAGAGGCCCAAGTTGACAGACATCGGCGTAAAGAGTGGTTAAGGAA

ACCCCATTACTAAAGCCGAACGCCCTCAGAACTGTCATACGTTCCCGAAGGTAAGAAGCC

CCACTACGAAAGTGGCTTTAACCACACCCGACCCCACGAAAGCTGTGAAA

>199_Sample_ASV13

CACCGCGGTTATACGAGAGGCCCAAGTTGATAGATATCGGCGTAAAGAGTGGTTAAGGAA

ACCCCGTTACTAAAGCCGAACGCCCTCAGAACTGTCATACGTTCCCGAAGGTAAGAAGCC

CCACTACGAAAGTGGCTTTAACCACACCCGACCCCACGAAAGCTGTGAAA

>199_Sample_ASV14

CACCGCGGTTATACGAGAGGCCCTAGTTGATAAATACGGCGTAAAGAGTGGTTAGAGGAC

TCCCATAATAAAGCCAAAGACCCCCCGGGCCGTCACACGCATACGAGGGCACGAAGTTCA

CACACGAAAGTAGCTTTAAATTACCCGACGCCACGAAAGCTAAGAAA

>199_Sample_ASV15

GACCGCGGTTATACGAGAGACTCTAGTTGATAGATACGGCGTAAAGAGTGGTTTGGGATA

AACTATAAATAAAGCCAAAGACCTTCCAAGCTGTTAAACGCATACCGAAGGCACGAAGCC

CCAACACGAAAGTAGCTTTACTACTACCCCCGATGCCACGAAAGCTAAGACA

>199_Sample_ASV16

CACCGCGGTCATACGATTAACCCAAGTTAATAGGCACACGGCGTAAAGCGTGTTTAAGCA

CTATGCCAAATAAAGTTAAACTCCAATTAAGCTGTAAAAAGCCATAATCAAGACGAAAAT

AAATAACGAAAGTAACTTTACAACCGCTGAAACACGATAGCTAAGATC

>199_Sample_ASV17

CACCGCGGTTATACGAGAGGCCCTAGTTGATAACTACCGGCGTAAAGAGTGGTTATGGAA

AAATATTTAATAAAGCCGAACACCCCCTCAGCCGTCATACGCACCTGGGAGCACGAAGAC

CTACTGCGAAAGCAGCTTTAATTATGCCTGACCCCACGACAGCTAAGAAA

>199_Sample_ASV18

CACCGCGGTTATACGAAAGACCCTAGTTTATAGACACGGCGTAAAGGGTGGTTCGGGACT

AAACAAAATAAAGCCAAAGACCTCCTAAGCCGTCATACGCTCACGGAAGCACGAAGCCCA

AGTACGAAAGTAGCTTTACAAAACATGCCTGACTCCACGAAAGCTAGGAAA

>199_Sample_ASV19

CACCGCGGTTATACGAAAGACCCTAGTTGATAGGCACGGCGTAAAGGGTGGTTAGGGATA

TATCAAAAATAAAGTTAAAGAGCCTCTAAGCTGTCGCACGCATCCCGAGAGCTCGAGACC

CAGACACGAAAGTAACTTTAAAATACCTCACCTGACCCCACGAAAGCTAAGAAA

>199_Sample_ASV20

CACCGCGGTTATACGAAAGACCCTAGTTGATAGCCACGGCGTAAAGGGTGGTTAAGGTAT

ACAACAAATAAAGCTAAATAGCCTCTAGGCCGTCGCACGCATTCCGAGAGCTCGAAACCC

AAAAACGAAAGTAGCTTTAAAATAAACAGACCTGACCCCACGAAAGCTAAGAAA

>199_Sample_ASV21

CACCGCGGTTATACGAGAGGCCCAAGTTGATACTCCACGGCGTAAAGAGTGGTTAGAAAA

GACCTACTACTAAAGCCGAACGCCTTCAAAGCTGTTATACGCATCCGAAGGTGAGAAGCC

CACCCACGAAAGTGGCTTTACAACCTTGACTCCACGAAAGCTATGACA

>199_Sample_ASV22

CACCGCGGTTATACGAGAGACCCCAGTTGATAAACACGGCGTAAAGAGTGGTTAAGGGCA

CAAAACAAATAAAGTCAAACAACCTCTTAGCTGTTATACGCATTATGAGGATATGAAGCC

CCCCTACGAAAGTAACTTTACCAACCCCTGACCCCACGAAAGCTAAGAAA

>199_Sample_ASV23

CACCGCGGTTATACGAAAGACCCTAGTTTATAGGCACGGCGTAAAGGGTGGTTAGGGGAT

AAACAAAATAAAGCCAAAGACCTTCTAAGTCGTCATACACTCATGAAGGCACGAAGCCCA

AATACGAAAGTAACTTTACAACTTAGACCCGACTCCACGAAAGCTGGGGGA

>199_Sample_ASV24

CACCGCGGTTATACGAGAGACTCCAGTTGATAGCTACGGCGTAAAGAGTGGTTTGGGGCC

CGCCCAAAATAAAGCCAAAGACCTCCCAAGCCGTCAAACGCACCCCGGAGGCACGAAGTC

CTAACGCGAAAGCAACTTTACCTTCCCCGACGCCACGAAAGCTAAGAAA

>199_Sample_ASV25

CACCGCGGTTATACGAAAGACCCTAGTTGATAGATCACGGCGTAAAGGGTGGTTAAGGAG

AACAAAAATAAAGCTAAAAATCTTCTAAGCTGTCATACGCTTTCCGAAGACATGAGACCC

AACCACGAAAGTAGCTTTAAACTTTCCTGACACCACGAAAGCCAAGAAA

>200_Sample_ASV1

CACCGCGGTTATACGAAAGACCCTAGTTGATGGGTGCGGCGTAAAGGGTGGTTAGGGACT

ACAAAAAATAGAGCTAAAGATCCTCTAGGTCGTCATACACATCACGAGGAAACGAAGCCC

AAATACGAAAGTAGCTCTAAATATAATCCTGACGCCACGAAAGCCAAGAAA

>200_Sample_ASV2

CACCGCGGTTATACGAGAGACCCTAGTTGATTAAAGCGGCGTAAAGAGTGGTTATGGAAC

TATTCTTTTAAAGCAGAAAACCTCTCAAACTGTTATACGCACCCAGAGGTCTGAATCCCT

CACACGAAAGTGACTTTATTTATGCCTACCAGAACCCACGAAAGCTGGGACA

>200_Sample_ASV3

CACCGCGGTTATACGAAAGACCCTAGTTTATAGGTACGGCGTAAAGGGTGGTTAGGGGAC

AAACAAAATAAAGCCAAAGACCTCCTAAGCCGTCATACGCTCACGGAAGCACGAAGCCCA

AACACGAAAGTAGCTTTACCGAACATGCCCGACTCCACGAAAGCTGGGGAA

>200_Sample_ASV4

CACCGCGGTTATACGAAAGACCCTAGTTGATAGCCACGGCGTAAAGGGTGGTTAAGGAAC

CTAAAAAATAAAGCTAAAGACCCTCTAGGCCGTCATACGCATTACGAGGCAAACGAAACC

CCAACACGAAAGTAGCTTTAGACAAATATCCTGATGCCACGAAAGCCAAGAAA

>200_Sample_ASV5

CACCGCGGTTATACGAGAGGCCCAAGTTGACAGACACCGGCGTAAAGAGTGGCTAGGGAA

AAATTACTACTAAAGCCGAACACCTTCAGAACTGTCATACGTTCCCGAAGATAAGAAGCC

CCACCACGAAAGTGACTTTATATTACCCGACCCCACGAAAGCTGTGAAA

>200_Sample_ASV6

CACCGCGGTTATACGAGAGGCCCAAGTTGACAGACACCGGCGTAAAGAGTGGCTAGGGAA

AAATTACTACTAAAGCCGAACACCTTCAGAACTGTCATACGTTCCCGAAGATAAGAAGCC

CCACCACGAAAGTGGCTTTATATTACCCGACCCCACGAAAGCTGTGAAA

>200_Sample_ASV7

CACCGCGGTTATACGAGAGGCCCAAGTTGATAAGCCCGGCGTAAAGAGTGGTTATGGAGA

ATAAAACACTAAAGCTAAAGACCCCCTAGGCTGTTATACGCACCTGGCGGCTCGAACCAC

CTATACGAAAGTAGCTTTACCCCCCTTCCACCAGAATCCACGACAGCTGGGGCA

>200_Sample_ASV8

CACCGCGGTTATACGAGAGGCCCAAGTTGACAGACATCGGCGTAAAGAGTGGTTAAGGAA

ACCCCATTACTAAAGCCGAACGCCCTCAGAACTGTCATACGTTCCCGAAGGTAAGAAGCC

CCACTACGAAAGTGGCTTTAACCACACCCGACCCCACGAAAGCTGTGAAA

>200_Sample_ASV9

CACCGCGGTTATACGAGAGGCCCAAGTTGATAGATATCGGCGTAAAGAGTGGTTAAGGAA

ACCCCGTTACTAAAGCCGAACGCCCTCAGAACTGTCATACGTTCCCGAAGGTAAGAAGCC

CCACTACGAAAGTGGCTTTAACCACACCCGACCCCACGAAAGCTGTGAAA

>200_Sample_ASV10

CACCGCGGTCACACGATTAACCCAAGTCAATAGAAGCCGGCGTAAAGAGTGTTTTAGATC

ACCCCCTCCCCAATAAAGCTAAAACTCACCTGAGTTGTAAAAAACTCCAGTTGACACAAA

ATAGACTACGAAAGTGGCTTTAACATATCTGAACACACAATAGCTAAGACC

>200_Sample_ASV11

CACCGCGGTTATACGAGAGACCCCAGTTGATAGCTACGGCGTAAAGAGTGGTTAAGGACC

CCATTTAATAAAGCCAAAGACCCCCCAAGCCGTCATACGCACACGAGGGCGCGAAGCTCG

CACACGAAAGTAGCTCTAATTTACCCGACGCCACGAAAGCTAAGGAA

>200_Sample_ASV12

CACCGCGGTTATACGAGAGACCCTAGTTGATAAATACGGCGTAAAGAGTGGTTATGGGAA

AAACAAATAAAGTCAAACAACCTCTTAGCTGTTATACGCATTATGAGAGCATGAAGCCCC

CTCACGAAAGTAACTTTAATACCTCCTGACCCCACGAAAGCTAAGAAA

>200_Sample_ASV13

CACCGCGGTTATACGAGAGACCCCAGTTGATAAACACGGCGTAAAGAGTGGTTAAGGGCA

CAAAACAAATAAAGTCAAACAACCTCTTAGCTGTTATACGCATTATGAGAGCATGAAGCC

CCCTCACGAAAGTAACTTTAATACCTCCTGACCCCACGAAAGCTAAGAAA

>200_Sample_ASV14

CACCGCGGTTATACGAGAGACCCTAATTGATAGACACGGCGTAAAGAGTGGTTTGGGAAA

GCCCAAAAATAAAGCTAAAGACCTTCTAAGCCGTCACACGCACGCCGAAGGCACGAAGCC

CTGACACGAAAGTAGCTTTACTATTACCCCCGACGCCACGAAAGCTAAGAAA

>200_Sample_ASV15

CACCGCGGTTATACGAAAGACCCTAGTTTATAGATGCGGCGTAAAGGGTGGTTAAGGAAA

ACCTAAAATAATGCTAAAGACTCTCAAAGCTGTTATACGCTTCATGAGCACACGAAATCC

AAATACGAAAGTTGCCTTAATAAACTTTGCCTGACCCCACGAAAGCTGAAAAA

>200_Sample_ASV16

CACCGCGGTTATACGAGAGACCCTAGTTGATAGCTACGGCGTAAAGCGTGGTTAGGAGAA

CAAGATAAATAAAGTCAAACAATCTCTCGGCCGTTATACGTTATCTGAGAATATGAAGTC

CTACCACGAAAGTAACTTTAATTTTTCTGACCCCACGAAAACTGAGGAA

>200_Sample_ASV17

CACCGCGGTTAAACGAGAGGCCCTAGTTGATAGTACAACGGCGTAAAGGGTGGTTAAGGA

TAGTGAAATAATAAAGTCGAATGGCCCTTTGGCTGTCATACGCTTCTAGGAGTCCGAAGC

CCAATATACGAAAGTAACTTTAAGAGCGCCCACCTGACCCCACGAAAACTGAGAAA

>200_Sample_ASV18

CACCGCGGTTATACGATTAACCCAAGTTAATAGGCACACGGCGTAAAGCGTGTTTAAGCA

CTACGCCAAATAAAGTTAAACTCCAATTAAGCTGTAAAAAGCCATAATCAAGACGAAAAT

AAATAACGAAAGTAACTTTACAACCGCTGAAACACGATAGCTAAGATC

>200_Sample_ASV19

CACCGCGGTCATACGATTAACCCAAGTTAATAGGCACACGGCGTAAAGCGTGTTTAAGCA

CTACGCCAAATAAAGTTAAACTCCAATTAAGCTGTAAAAAGCCATAATCAAGACGAAAAT

AAATAACGAAAGTAACTTTACAACCGCTGAAACACGATAGCTAAGATC

>200_Sample_ASV20

CACCGCGGTTATACGAAAGACCCTAGTTGATAGGCACGGCGTAAAGGGTGGTTAGGGATA

TATTAAAAATAAAGTTAAAGAGCCTCTAAGCTGTCGCACGCATCCCGAGAGCTCGAGACC

CAGACACGAAAGTAACTTTAAAATACCTCACCTGACCCCACGAAAGCTAAGAAA

>200_Sample_ASV21

CACCGCGGTTATACGAGAGGCCCAAGTTGATACTCCACGGCGTAAAGAGTGGTTAGAAAA

GACCTACTACTAAAGCCGAACGCCTTCAAAGCTGTTATACGCATCCGAAGGTGAGAAGCC

CACCCACGAAAGTGGCTTTACAACCTTGACTCCACGAAAGCTATGACA

>200_Sample_ASV22

CACCGCGGTTATACGAGAGACCCCAGTTGATAAACACGGCGTAAAGAGTGGTTAAGGGCA

CAAAACAAATAAAGTCAAACAACCTCTTAGCTGTTATACGCATTATGAGGATATGAAGCC

CCCCTACGAAAGTAACTTTACCAACCCCTGACCCCACGAAAGCTAAGAAA

>200_Sample_ASV23

CACCGCGGTTATACGAGAGACCCTAGTTGATATATACGGCGTAAAGAGTGGTTTGGAATA

ACCAAGTAATATAGCCAAAGACCTCCCAAGCCGTCACACGCACCCCGGAGGCACGAAGCC

CAAACACGAAAGTAGCTTTATTAACTTACCGACGCCACGAAAGCTAAGAAA

>200_Sample_ASV24

CACCGCGGTTATACGAAAGACCCTAGTTTATAGGCACGGCGTAAAGGGTGGTTAGGGGAT

AAACAAAATAAAGCCAAAGACCTTCTAAGTCGTCATACACTCATGAAGGCACGAAGCCCA

AATACGAAAGTAACTTTACAACTTAGACCCGACTCCACGAAAGCTGGGGGA

>200_Sample_ASV25

CACCGCGGTTATACGAAAGACCCTAGTTGATAGCTACGGCGTAAAGGGTGGTTAAGGTAT

ATAACAAATAAAGCTAAAGAGCCTCCAAGCCGTCGTACGCATTCTGAGAGCTCGAAACCC

TAACACGAAAGTAGCTTTAAAAAATAACACCTGACCCCACGAAAGCTAAGAAA

>200_Sample_ASV26

CACCGCGGTTATACGAGAGACCCTAATTGATAGACTACGGCGTAAAGAGTGGTTTAGAAC

AAAAATTAATAAAGCCAAAGATCCTCCTGGCCGTCGCACGCATTTCGAGGATACGAAGCC

CCACTACGAAAGTAGCTTTACTACAACTTTTCTGACCCCACGAAAGCTAAGAAA

>200_Sample_ASV27

CACCGCGGTTATACGAAAGACCCTAGTTGATAGATCACGGCGTAAAGGGTGGTTAAGGAG

AACAAAAATAAAGCTAAAAATCCTCTAAGCTGTCATACGCTTTCCGAAGACATGAGACCC

AACCACGAAAGTAACTTTAAACTTTCCTGACACCACGAAAGCCAAGAAA

>227_Sample_ASV1

CACCGCGGTTATACGAAAGACCCTAGTTGATGGGTGCGGCGTAAAGGGTGGTTAGGGACT

ACAAAAAATAGAGCTAAAGATCCTCTAGGTCGTCATACACATCACGAGGAAACGAAGCCC

AAATACGAAAGTAGCTCTAAATATAATCCTGACGCCACGAAAGCCAAGAAA

>227_Sample_ASV2

CACCGCGGTTATACGAGAGACCCTAGTTGATTAAAGCGGCGTAAAGAGTGGTTATGGAAC

TATTCTTTTAAAGCAGAAAACCTCTCAAACTGTTATACGCACCCAGAGGTCTGAATCCCT

CACACGAAAGTGACTTTATTTATGCCTACCAGAACCCACGAAAGCTGGGACA

>227_Sample_ASV3

CACCGCGGTTATACGAGAGACCCTAGTTGATTAAAGCGGCGTAAAGAGTGGTTATGGAAC

TATTCTTTTAAAGCAGAAAACCTCTCAAACTGTTATACGCACCCAGAGGTCTGAATCCCT

CACACGAAAGTGACTTTATTTATGCCTACCAGAACCCACGAAAGCTATGACA

>227_Sample_ASV4

CACCGCGGTTATACGAAAGACCCTAGTTGATAGCCACGGCGTAAAGGGTGGTTAAGGAAC

CTAAAAAATAAAGCTAAAGACCCTCTAGGCCGTCATACGCATTACGAGGCAAACGAAACC

CCAACACGAAAGTAGCTTTAGACAAATATCCTGATGCCACGAAAGCCAAGAAA

>227_Sample_ASV5

CACCGCGGTTATACGAGAGGCCCAAGTTGACAGACACCGGCGTAAAGAGTGGCTAGGGAA

AAATTACTACTAAAGCCGAACACCTTCAGAACTGTCATACGTTCCCGAAGATAAGAAGCC

CCACCACGAAAGTGACTTTATATTACCCGACCCCACGAAAGCTAAGAAA

>227_Sample_ASV6

CACCGCGGTTATACGAGAGGCCCAAGTTGACAGACACCGGCGTAAAGAGTGGCTAGGGAA

AAATTACTACTAAAGCCGAACACCTTCAGAACTGTCATACGTTCCCGAAGATAAGAAGCC

CCACCACGAAAGTGGCTTTATATTACCCGACCCCACGAAAGCTGTGAAA

>227_Sample_ASV7

CACCGCGGTTATACGAGAGGCCCAAGTTGACAGACACCGGCGTAAAGAGTGGCTAGGGAA

AAATTACTACTAAAGCCGAACACCTTCAGAACTGTCATACGTTCCCGAAGATAAGAAGCC

CCACCACGAAAGTGGCTTTATATTACCCGACCCCACGAAAGCTAAGAAA

>227_Sample_ASV8

CACCGCGGTTATACGAGAGGCCCAAGTTGACAGACACCGGCGTAAAGAGTGGCTAAGGAA

AAATTTCTACTAAAGCCGAACACCTTCAGAACTGTCATACGTTCCCGAAGAAAAGAAGCC

CCACCACGAAAGTGGCTTTATATTACCCGACCCCACGAAAGCTGTGAAA

>227_Sample_ASV9

CACCGCGGTTATACGAGAGGCCCAAGTTGATAAGCCCGGCGTAAAGAGTGGTTATGGAGA

ATAAAACACTAAAGCTAAAGACCCCCTAGGCTGTTATACGCACCTGGCGGCTCGAACCAC

CTATACGAAAGTAGCTTTACCCCCCTTCCACCAGAATCCACGACAGCTGGGGCA

>227_Sample_ASV10

CACCGCGGTTATACGAGAGGCCCTAGTTGATAGCCACGGCGCAAAGAGTGGTTAAGGAGC

CCCACTAAATAAAGTCGAACACTTCCTAGGCCGTTATACGTTTTCTAGAAGCACGAAACC

CAATTACACGAAAGCAACTTTATACTAAACAACCTGACCCCACGAAAGCTAAGAAA

>227_Sample_ASV11

CACCGCGGTTATACGAGAGGCCCAAGTTGACAGACATCGGCGTAAAGAGTGGTTAAGGAA

ACCCCATTACTAAAGCCGAACGCCCTCAGAACTGTCATACGTTCCCGAAGGTAAGAAGCC

CCACTACGAAAGTGGCTTTAACCACACCCGACCCCACGAAAGCTGTGAAA

>227_Sample_ASV12

CACCGCGGTTATACGAGAGACCCCAGTTGATAGCTACGGCGTAAAGAGTGGTTAAGGACC

CCATTTAATAAAGCCAAAGACCCCCCAAGCCGTCATACGCACACGAGGGCGCGAAGCTCG

CACACGAAAGTAGCTCTAATTTACCCGACGCCACGAAAGCTAAGGAA

>227_Sample_ASV13

CACCGCGGTTATACGAGAAACCCTAGTTGACAAATACGGCGTAAAGAGTGGTTAAGGGGT

ATCAAAAATAAAGTCAAACATTTTCCTAGTTGTTATAAGCATTTCGAGAACATGAAGCCC

AACCACGAAAGTAACTTTACCTTCACCTGACCCCACGAAAGCTAAGAAA

>227_Sample_ASV14

CACCGCGGTTATACGAGAGACCCTAGTTGATAAATACGGCGTAAAGAGTGGTTATGGGAA

AAACAAATAAAGTCAAACAACCTCTTAGCTGTTATACGCATTATGAGAGCATGAAGCCCC

CTCACGAAAGTAACTTTAATACCTCCTGACCCCACGAAAGCTAAGAAA

>227_Sample_ASV15

CACCGCGGTTATACGAGAGACCCTAATTGATAGACACGGCGTAAAGAGTGGTTTGGGAAA

GCCCAAAAATAAAGCTAAAGACCTTCTAAGCCGTCACACGCACGCCGAAGGCACGAAGCC

CTGACACGAAAGTAGCTTTACTATTACCCCCGACGCCACGAAAGCTAAGAAA

>227_Sample_ASV16

CACCGCGGTTATACGAAAGACCCTAGTTTATAGATGCGGCGTAAAGGGTGGTTAAGGAAA

ACCTAAAATAATGCTAAAGACTCTCAAAGCTGTTATACGCTTCATGAGCACACGAAATCC

AAATACGAAAGTTGCCTTAATAAACTTTGCCTGACCCCACGAAAGCTGAAAAA

>227_Sample_ASV17

CACCGCGGTTATACGAGAGACCCTAGTTGATAGCTACGGCGTAAAGCGTGGTTAGGAGAA

CAAGATAAATAAAGTCAAACAATCTCTCGGCCGTTATACGTTATCTGAGAATATGAAGTC

CTACCACGAAAGTAACTTTAATTTTTCTGACCCCACGAAAACTGAGGAA

>227_Sample_ASV18

CACCGCGGTTAAACGAGAGGCCCTAGTTGATAGTACAACGGCGTAAAGGGTGGTTAAGGA

TAGTGAAATAATAAAGTCGAATGGCCCTTTGGCTGTCATACGCTTCTAGGAGTCCGAAGC

CCAATATACGAAAGTAACTTTAAGAGCGCCCACCTGACCCCACGAAAACTGAGAAA

>227_Sample_ASV19

CACCGCGGTTATACGAAAGACCCTAGTTGATAGGCACGGCGTAAAGGGTGGTTAGGGATA

TATTAAAAATAAAGTTAAAGAGCCTCTAAGCTGTCGCACGCATCCCGAGAGCTCGAGACC

CAGACACGAAAGTAACTTTAAAATACCTCACCTGACCCCACGAAAGCTAAGAAA

>227_Sample_ASV20

CACCGCGGTTATACGAGAGGCCCAAGTTGATACTCCACGGCGTAAAGAGTGGTTAGAAAA

GACCTACTACTAAAGCCGAACGCCTTCAAAGCTGTTATACGCATCCGAAGGTGAGAAGCC

CACCCACGAAAGTGGCTTTACAACCTTGACTCCACGAAAGCTATGACA

>227_Sample_ASV21

CACCGCGGTTATACGAGAGACCCCAGTTGATAAACACGGCGTAAAGAGTGGTTAAGGGCA

CAAAACAAATAAAGTCAAACAACCTCTTAGCTGTTATACGCATTATGAGGATATGAAGCC

CCCCTACGAAAGTAACTTTACCAACCCCTGACCCCACGAAAGCTAAGAAA

>227_Sample_ASV22

CACCGCGGTTATACGAGAGACCCTAGTTGATATATACGGCGTAAAGAGTGGTTTGGAATA

ACCAAGTAATATAGCCAAAGACCTCCCAAGCCGTCACACGCACCCCGGAGGCACGAAGCC

CAAACACGAAAGTAGCTTTATTAACTTACCGACGCCACGAAAGCTAAGAAA

>227_Sample_ASV23

CACCGCGGTTATACGAAAGACCCTAGTTTATAGGCACGGCGTAAAGGGTGGTTAGGGGAT

AAACAAAATAAAGCCAAAGACCTTCTAAGTCGTCATACACTCATGAAGGCACGAAGCCCA

AATACGAAAGTAACTTTACAACTTAGACCCGACTCCACGAAAGCTGGGGGA

>227_Sample_ASV24

CACCGCGGTTATACGAGAGACCCTAATTGATAGACTACGGCGTAAAGAGTGGTTTAGAAC

AAAAATTAATAAAGCCAAAGATCCTCCTGGCCGTCGCACGCATTTCGAGGATACGAAGCC

CCACTACGAAAGTAGCTTTACTACAACTTTTCTGACCCCACGAAAGCTAAGAAA

>227_Sample_ASV25

CACCGCGGTTATACGAAAGACCCTAGTTGATAGATCACGGCGTAAAGGGTGGTTAAGGAG

AACAAAAATAAAGCTAAAAATCCTCTAAGCTGTCATACGCTTTCCGAAGACATGAGACCC

AACCACGAAAGTAACTTTAAACTTTCCTGACACCACGAAAGCCAAGAAA

>228_Sample_ASV1

CACCGCGGTTATACGAAAGACCCTAGTTGATGGGTGCGGCGTAAAGGGTGGTTAGGGACT

ACAAAAAATAGAGCTAAAGATCCTCTAGGTCGTCATACACATCACGAGGAAACGAAGCCC

AAATACGAAAGTAGCTCTAAATATAATCCTGACGCCACGAAAGCCAAGAAA

>228_Sample_ASV2

CACCGCGGTTAAACGAGAGGCCCTAGTTGATGGTGCAACGGCGTAAAGGGTGGTTAAGGA

TAGCGAAATAATAAAGTCAAATGGCCCTCTGGCTGTCATACGCTTCTAGGAGTCCGAAGC

CCAGTATACGAAAGTAGCTTTAAGAAAGTCCACCTGACCCCACGAAAACTGAGGAA

>228_Sample_ASV3

CACCGCGGTTATACGAAAGACCCTAGTTGCTAGCCACGGCGTAAAGGGTGGTTAAGGACA

ACAACAAATAAAGCTAAAGATCCCCTAAGCCGTCATACGCATTCCGGGGGCACGAAGCCC

TAACACGAAAGTAGCTTTAAAAAATATACCTGACCCCACGAAAGCTAAGAAA

>228_Sample_ASV4

CACCGCGGTTATACGAGAGGCCCAAGTTGACAGACACCGGCGTAAAGAGTGGCTAGGGAA

AAATTACTACTAAAGCCGAACACCTTCAGAACTGTCATACGTTCCCGAAGATAAGAAGCC

CCACCACGAAAGTGGCTTTATATTACCCGACCCCACGAAAGCTAAGAAA

>228_Sample_ASV5

CACCGCGGTTATACGAGAGGCCCAAGTTGACAGACACCGGCGTAAAGAGTGGCTAGGGAA

AAATTACTACTAAAGCCGAACACCTTCAGAACTGTCATACGTTCCCGAAGATAAGAAGCC

CCACCACGAAAGTGGCTTTATATTACCCGACCCCACGAAAGCTGTGAAA

>228_Sample_ASV6

CACCGCGGTTATACGAGAGACCCTAGTTGATATATACGGCGTAAAGAGTGGTTTGGGATA

TTTCTCCAAAATAAAGCCGAAGATCTCCCAAGCCGTCACACGCATCCCGGAGACACGAAG

CCCTGATACGAAGGTAGCTTTATTAACACCCCGACGCCACGAAAGCTAAGAAA

>228_Sample_ASV7

CACCGCGGTTATACGAGAGGCCCAAGTTGATAAGCCCGGCGTAAAGAGTGGTTATGGAGA

ATAAAACACTAAAGCTAAAGACCCCCTAGGCTGTTATACGCACCTGGCGGCTCGAACCAC

CTATACGAAAGTAGCTTTACCCCCCTTCCACCAGAATCCACGACAGCTGGGGCA

>228_Sample_ASV8

CACCGCGGTTATACGAGAGGCCCTAGTTGATAGCCACGGCGCAAAGAGTGGTTAAGGAGC

CCCACTAAATAAAGTCGAACACTTCCTAGGCCGTTATACGTTTTCTAGAAGCACGAAACC

CAATTACACGAAAGCAACTTTATACTAAACAACCTGACCCCACGAAAGCTAAGAAA

>228_Sample_ASV9

CACCGCGGTTATACGAGAGGCCCAAGTTGACAGACATCGGCGTAAAGAGTGGTTAAGGAA

ACCCCATTACTAAAGCCGAACGCCCTCAGAACTGTCATACGTTCCCGAAGGTAAGAAGCC

CCACTACGAAAGTGGCTTTAACCACACCCGACCCCACGAAAGCTGTGAAA

>228_Sample_ASV10

CACCGCGGTTAAACGAGAGGCCCTAGTTGATATTACAACGGCGTAAAGGGTGGTTAAGGA

AAGCAAGCAATAAAGCCGAATGGCCCTTTGGCTGTCATACGCTTCTAGGTGTCCGAAGCC

CAATATACGAAAGTAGCTTTAGAAAAGCCCACCTGACACCACGAAAGCTGAGGAA

>228_Sample_ASV11

CACCGCGGTTATACGAGAAACCCTAGTTGACAAATACGGCGTAAAGAGTGGTTAAGGGGT

ATCAAAAATAAAGTCAAACATTTTCCTAGTTGTTATAAGCATTTCGAGAACATGAAGCCC

AACCACGAAAGTAACTTTACCTTCACCTGACCCCACGAAAGCTAAGAAA

>228_Sample_ASV12

CACCGCGGTTATACGAAAGACCCCAGTTTATAGACACGGCGTAAAGGGTGGTTATAGGAC

AAACTAACATAGAGCCAAAGACCCCCTAAGCCGTCATACGCTCACGAGGGCTCGAAGCCC

CTATACGAAAGTAGCTCTACCAAAAAATGCTCGACTCCACGAAAGCTGGGACA

>228_Sample_ASV13

CACCGCGGTTATACGAGAGACCCTAGTTGATAAATACGGCGTAAAGAGTGGTTATGGGAA

AAACAAATAAAGTCAAACAACCTCTTAGCTGTTATACGCATTATGAGAGCATGAAGCCCC

CTCACGAAAGTAACTTTAATACCTCCTGACCCCACGAAAGCTAAGAAA

>228_Sample_ASV14

CACCGCGGTTATACGAGAGGCTCAAGTTGATAAACCCCGGCGTAAAGAGTGGTTAAGGAA

AAATAGAAACTAAAGCCGAATGCTTTCAAGACTGTTATACGTTCCCGAGAGTAAGAAGAC

CAATTACGAAAGTGGCTTTACTTTACCTGACCCCACGAAAGCTACGACA

>228_Sample_ASV15

CACCGCGGTTATACGAAAGACCCTAGTTGATGGATCACGGCGTAAAGGGTGGTTAAGGGA

TATAAAAATAAAGCTAAAAATCCTCTTAGCTGTCATACGCTTTCCGAAGACATGAGGCCC

AATTACGAAAGTAGCTTTAAACCACCCTGACACCACGAAAGCCAAGAAA

>228_Sample_ASV16

CACCGCGGTTATACGAGAGGCCCTAGTTGATAACTACCGGCGTAAAGAGTGGTTATGGAA

AAATATTTAATAAAGCCGAACACCCCCTCAGCCGTCATACGCACCTGGGAGCACGAAGAC

CTACTGCGAAAGCAGCTTTAATTATGCCTGACCCCACGACAGCTAAGAAA

>228_Sample_ASV17

CACCGCGGTTATACGAGAGGCCCAAGTTGATTATTACCGGCGTAAAGCGTGGTTAAGATT

TTTATAGGACTAAAGCCGAACCCCCTCAGAGCTGTTATACGCACCCGAAGGTTAGAAGTT

CAATCACGAAAGTGGCTTTACAACCCCGAACCCACGAAAGCTAAGATA

>228_Sample_ASV18

CACCGCGGTTATACGAGAGACCCTAATTGATAGGCGCGGCGTAAAGAGTGGTTTAGGACT

AAAACTAATAAAGCTAAAGATCCTCCCAGCCGTCGCACGCATTCTGAGGACACGAAACTC

AACCACGAAAGTAGCTTTAACATACTCTCCTGACCCCACGAAAGCTAAGAAA

>228_Sample_ASV19

CACCGCGGTTATACGAGAGGCCCAAGTTGATAGTCTTCGGCGTAAAGAGTGGTTAAAGAA

GCAATTAGACTAAGGCTAAACTCTTCCAAGGCTGTCATACGCACCCGGAAGCATGAAACC

CAACCACGAAAGTGGCCTTATTTACCTTGACCCCACGAAAGCTGTGAAA

>228_Sample_ASV20

TACCGCGGTTATACGAGAGGCCCAAGTTGATAGTCCTCGGCGTAAAGAGTGGTTAGAGAA

TCTATTAAACTAAGGCTAAACCCTTCCAAAGCCGTCATACGCATCCGGAAGCATGAAACC

CACCCACGAAAGTAGCCTTATTTACCTTGACCCCACGAAAGCTGTGGAA

>228_Sample_ASV21

CACCGCGGTTATACGAGAGACCCCAGTTGATAAACACGGCGTAAAGAGTGGTTAAGGGCA

CAAAACAAATAAAGTCAAACAACCTCTTAGCTGTTATACGCATTATGAGGATATGAAGCC

CCCCTACGAAAGTAACTTTACCAACCCCTGACCCCACGAAAGCTAAGAAA

>228_Sample_ASV22

CACCGCGGTTATACGAAAGACCCTAGTTTATAGGCACGGCGTAAAGGGTGGTTAGGGGAT

AAACAAAATAAAGCCAAAGACCTTCTAAGTCGTCATACACTCATGAAGGCACGAAGCCCA

AATACGAAAGTAACTTTACAACTTAGACCCGACTCCACGAAAGCTGGGGGA

>228_Sample_ASV23

CACCGCGGTTATACGAGAGACCCTAATTAATAGATACGGCGTAAAGAGTGGTTTAGGACA

AAAATTAATAAAGCCGAAGTTTCTCCTAGCCGTTGTACGCACTCTGAGAACACGAAGCCC

CACTACGAAAGTAGCTTTACCTATTATTTCCTGACCCCACGAAAGCTAAGAAA

>228_Sample_ASV24

CACCGCGGTTATACGAGAGACCCTAATTAATAGCTACGGCGTAAAGAGTGGTTTAGGGTA

AAAATTAATAAAGCCGAAGTTTCTCCTAGCCGTTGTACGCACTCTGAGAACACGAAGCCC

CACTACGAAAGTAGCTTTACCTATTATTTCCTGACCCCACGAAAGCTAAGAAA

>228_Sample_ASV25

CACCGCGGTTATACGAGAGGCCCAAGTTGACAAACTCCGGCGTAAAGCGTGGTTAAGGCA

CACTCAAAAACTAAAGCCGAATATCTTCAGAGCAGTCATACGCATCCGAAGACACGAAGC

CCCACCACGAAAGTGGCTTTATGACACCTGACCCCACGAAAGCTAGGACA

>228_Sample_ASV26

CACCGCGGTTATACGAAAGACCCTAGTTGATAGATCACGGCGTAAAGGGTGGTTAAGGAA

AACAAAAATAAAGCTAAAAATCCTCTAAGCTGTCATACGCTTTCCGAAGACATGAGATCC

AACCACGAAAGTAGCTTTAAAATTTCCTGACACCACGAAAGCCAAGGAA

>236_Sample_ASV1

CACCGCGGTTATACGAAAGACCCTAGTTGATGGGTGCGGCGTAAAGGGTGGTTAGGGACT

ACAAAAAATAGAGCTAAAGATCCTCTAGGTCGTCATACACATCACGAGGAAACGAAGCCC

AAATACGAAAGTAGCTCTAAATATAATCCTGACGCCACGAAAGCCAAGAAA

>236_Sample_ASV2

CACCGCGGTTATACGAGAGACCCTAGTTGATTAAAGCGGCGTAAAGAGTGGTTATGGAAC

TATTCTTTTAAAGCAGAAAACCTCTCAAACTGTTATACGCACCCAGAGGTCTGAATCCCT

CACACGAAAGTGACTTTATTTATGCCTACCAGAACCCACGAAAGCTGGGACA

>236_Sample_ASV3

CACCGCGGTTATACGAAAGACCCTAGTTGATAGCCACGGCGTAAAGGGTGGTTAAGGAAC

CTAAAAAATAAAGCTAAAGACCCTCTAGGCCGTCATACGCATTACGAGGCAAACGAAACC

CCAACACGAAAGTAGCTTTAGACAAATATCCTGATGCCACGAAAGCCAAGAAA

>236_Sample_ASV4

CACCGCGGTTATACGAGAGGCCCAAGTTGACAGACACCGGCGTAAAGAGTGGCTAGGGAA

AAATTACTACTAAAGCCGAACACCTTCAGAACTGTCATACGTTCCCGAAGATAAGAAGCC

CCACCACGAAAGTGGCTTTATATTACCCGACCCCACGAAAGCTGTGAAA

>236_Sample_ASV5

CACCGCGGTTATACGAGAGGCCCAAGTTGACAGACACCGGCGTAAAGAGTGGCTAGGGAA

AAATTACTACTAAAGCCGAACACCTTCAGAACTGTCATACGTTCCCGAAGATAAGAAGCC

CCACCACGAAAGTGGCTTTATATTACCCGACCCCACGAAAGCCAAGAAA

>236_Sample_ASV6

CACCGCGGTTATACGAGAGGCCCAAGTTGACAGACACCGGCGTAAAGAGTGGCTAAGGAA

AAATTTCTACTAAAGCCGAACACCTTCAGAACTGTCATACGTTCCCGAAGAAAAGAAGCC

CCACCACGAAAGTGGCTTTATATTACCCGACCCCACGAAAGCTGTGAAA

>236_Sample_ASV7

CACCGCGGTTATACGAGAGGCCCAAGTTGATAAGCCCGGCGTAAAGAGTGGTTATGGAGA

ATAAAACACTAAAGCTAAAGACCCCCTAGGCTGTTATACGCACCTGGCGGCTCGAACCAC

CTATACGAAAGTAGCTTTACCCCCCTTCCACCAGAATCCACGACAGCTGGGGCA

>236_Sample_ASV8

CACCGCGGTTATACGAGAGGCCCAAGTTGACAGACATCGGCGTAAAGAGTGGTTAAGGAA

ACCCCATTACTAAAGCCGAACGCCCTCAGAACTGTCATACGTTCCCGAAGGTAAGAAGCC

CCACTACGAAAGTGGCTTTAACCACACCCGACCCCACGAAAGCTGTGAAA

>236_Sample_ASV9

CACCGCGGTTATACGAGAGGCCCAAGTTGACAGACATCGGCGTAAAGAGTGGTTAAGGAA

ACCCCATTACTAAAGCCGAACGCCCTCAGAACTGTCATACGTTCCCGAAGGTAAGAAGCC

CCACTACGAAAGTGGCTTTAACCACACCCGACCCCACGAAAGCTAAGAAA

>236_Sample_ASV10

CACCGCGGTTATACGAGAGACCCCAGTTGATAGCTACGGCGTAAAGAGTGGTTAAGGACC

CCATTTAATAAAGCCAAAGACCCCCCAAGCCGTCATACGCACACGAGGGCGCGAAGCTCG

CACACGAAAGTAGCTCTAATTTACCCGACGCCACGAAAGCTAAGGAA

>236_Sample_ASV11

CACCGCGGTTATACGAGAGACCCTAGTTGATAAATACGGCGTAAAGAGTGGTTATGGGAA

AAACAAATAAAGTCAAACAACCTCTTAGCTGTTATACGCATTATGAGAGCATGAAGCCCC

CTCACGAAAGTAACTTTAATACCTCCTGACCCCACGAAAGCTAAGAAA

>236_Sample_ASV12

CACCGCGGTTATACGAGAGACCCTAATTGATAGACACGGCGTAAAGAGTGGTTTGGGAAA

GCCCAAAAATAAAGCTAAAGACCTTCTAAGCCGTCACACGCACGCCGAAGGCACGAAGCC

CTGACACGAAAGTAGCTTTACTATTACCCCCGACGCCACGAAAGCTAAGAAA

>236_Sample_ASV13

CACCGCGGTTATACGAAAGACCCTAGTTTATAGATGCGGCGTAAAGGGTGGTTAAGGAAA

ACCTAAAATAATGCTAAAGACTCTCAAAGCTGTTATACGCTTCATGAGCACACGAAATCC

AAATACGAAAGTTGCCTTAATAAACTTTGCCTGACCCCACGAAAGCTGAAAAA

>236_Sample_ASV14

CACCGCGGTTATACGAGAGACCCTAGTTGATAGCTACGGCGTAAAGCGTGGTTAGGAGAA

CAAGATAAATAAAGTCAAACAATCTCTCGGCCGTTATACGTTATCTGAGAATATGAAGTC

CTACCACGAAAGTAACTTTAATTTTTCTGACCCCACGAAAACTGAGGAA

>236_Sample_ASV15

CACCGCGGTTAAACGAGAGGCCCTAGTTGATAGTACAACGGCGTAAAGGGTGGTTAAGGA

TAGTGAAATAATAAAGTCGAATGGCCCTTTGGCTGTCATACGCTTCTAGGAGTCCGAAGC

CCAATATACGAAAGTAACTTTAAGAGCGCCCACCTGACCCCACGAAAACTGAGAAA

>236_Sample_ASV16

CACCGCGGTTATACGAAAGACCCTAGTTGATAGGCACGGCGTAAAGGGTGGTTAGGGATA

TATTAAAAATAAAGTTAAAGAGCCTCTAAGCTGTCGCACGCATCCCGAGAGCTCGAGACC

CAGACACGAAAGTAACTTTAAAATACCTCACCTGACCCCACGAAAGCTAAGAAA

>236_Sample_ASV17

CACCGCGGTTATACGAGAGACCCCAGTTGATAAACACGGCGTAAAGAGTGGTTAAGGGCA

CAAAACAAATAAAGTCAAACAACCTCTTAGCTGTTATACGCATTATGAGGATATGAAGCC

CCCCTACGAAAGTAACTTTACCAACCCCTGACCCCACGAAAGCTAAGAAA

>236_Sample_ASV18

CACCGCGGTTATACGAGAGACCCTAGTTGATATATACGGCGTAAAGAGTGGTTTGGAATA

ACCAAGTAATATAGCCAAAGACCTCCCAAGCCGTCACACGCACCCCGGAGGCACGAAGCC

CAAACACGAAAGTAGCTTTATTAACTTACCGACGCCACGAAAGCTAAGAAA

>236_Sample_ASV19

CACCGCGGTTATACGAAAGACCCTAGTTTATAGGCACGGCGTAAAGGGTGGTTAGGGGAT

AAACAAAATAAAGCCAAAGACCTTCTAAGTCGTCATACACTCATGAAGGCACGAAGCCCA

AATACGAAAGTAACTTTACAACTTAGACCCGACTCCACGAAAGCTGGGGGA

>236_Sample_ASV20

CACCGCGGTTATACGAGAGGCCCAAGTTGATAGACATCGGCGTAAAGAGTGGTTAAGGAA

GTCTCAAAACTAAAGCCGAATGCCTTCAAGACTGTTATACGTTTCCGAAGGTAAGAAGCC

CCACTACGAAAGTGGCTTTACTCCACCTGACTCCACGAAAGCTGTGAAA

>236_Sample_ASV21

CACCGCGGTTATACGAGAGACCCTAATTGATAGACTACGGCGTAAAGAGTGGTTTAGAAC

AAAAATTAATAAAGCCAAAGATCCTCCTGGCCGTCGCACGCATTTCGAGGATACGAAGCC

CCACTACGAAAGTAGCTTTACTACAACTTTTCTGACCCCACGAAAGCTAAGAAA

>236_Sample_ASV22

CACCGCGGTTATACGAAAGACCCTAGTTGATAGATCACGGCGTAAAGGGTGGTTAAGGAG

AACAAAAATAAAGCTAAAAATCCTCTAAGCTGTCATACGCTTTCCGAAGACATGAGACCC

AACCACGAAAGTAACTTTAAACTTTCCTGACACCACGAAAGCCAAGAAA

>237_Sample_ASV1

CACCGCGGTTATACGAAAGACCCTAGTTGATGGGTGCGGCGTAAAGGGTGGTTAGGGACT

ACAAAAAATAGAGCTAAAGATCCTCTAGGTCGTCATACACATCACGAGGAAACGAAGCCC

AAATACGAAAGTAGCTCTAAATATAATCCTGACGCCACGAAAGCCAAGAAA

>237_Sample_ASV2

CACCGCGGTTATACGAGAGACCCTAGTTGATTAAAGCGGCGTAAAGAGTGGTTATGGAAC

TATTCTTTTAAAGCAGAAAACCTCTCAAACTGTTATACGCACCCAGAGGTCTGAATCCCT

CACACGAAAGTGACTTTATTTATGCCTACCAGAACCCACGAAAGCTGGGACA

>237_Sample_ASV3

CACCGCGGTTATACGAAAGACCCTAGTTTATAGGTACGGCGTAAAGGGTGGTTAGGGGAC

AAACAAAATAAAGCCAAAGACCTCCTAAGCCGTCATACGCTCACGGAAGCACGAAGCCCA

AACACGAAAGTAGCTTTACCGAACATGCCCGACTCCACGAAAGCTGGGGAA

>237_Sample_ASV4

CACCGCGGTTATACGAGAGGCCCAAGTTGACAGACACCGGCGTAAAGAGTGGCTAGGGAA

AAATTACTACTAAAGCCGAACACCTTCAGAACTGTCATACGTTCCCGAAGATAAGAAGCC

CCACCACGAAAGTGGCTTTATATTACCCGACCCCACGAAAGCTGTGAAA

>237_Sample_ASV5

CACCGCGGTTATACGAGAGGCCCAAGTTGACAGACACCGGCGTAAAGAGTGGCTAGGGAA

AAATTACTACTAAAGCCGAACACCTTCAGAACTGTCATACGTTCCCGAAGATAAGAAGCC

CCACCACGAAAGTGGCTTTATATTACCCGACCCCACGAAAGCTAAGAAA

>237_Sample_ASV6

CACCGCGGTTAAACGAGAGACCCTAGTTGATCATCACGGCGTAAAGAGTGGTTAGGGGAT

TATCATAAATAAAGCCAAAGACCTCCCAAGCTGTCGCACGCATTCCGGAGGCGCGAAGCC

CACACACGAAAGTAGCTTTAACTATTGACCCTGATGCCACGAAAGCTAAGAAA

>237_Sample_ASV7

CGCCGCGGTTAAACGAGAGGCCCTAGTTGATGGAACAACGGCGTAAAGGGTGGTTAAGGA

TAGTGAGACAATAAAGTCGAATGGCCCTTTGGCTGTCATACGCTTCTGGGAGCCTGAAGC

CCACTATACGAAAGTAGCTTTAACAACGTCCACCTGACCCCACGAAAACTGAGAAA

>237_Sample_ASV8

CGCCGCGGTTAAACGAGAGGCCCTAGTTGATGGAACAACGGCGTAAAGGGTGGTTAAGGA

TAGTGAGACAATAAAGTCGAATGGCCCTTTGGCTGTCATACGCTTCTGGGAGCCTGAAGC

CCACTATACGAAAGTAGCTTTAACAATGTCCACCTGACCCCACGAAAACTGAGAAA

>237_Sample_ASV9

CACCGCGGTTATACGAAAGACCCTAGTTGATAGATCACGGCGTAAAGGGTGGTTAGGGAA

TACAAAAATAAAGCTAAAAATCCTCTAAGCTGTCATACGCTTCCCGAAGACATGAAACCC

AACCACGAAAGTAGCTTTAAACTCTCCTGACACCACGAAAGCCAAGGAA

>237_Sample_ASV10

CACCGCGGTTATACGAGAGGCCCAAGTTGATAAGCCCGGCGTAAAGAGTGGTTATGGAGA

ATAAAACACTAAAGCTAAAGACCCCCTAGGCTGTTATACGCACCTGGCGGCTCGAACCAC

CTATACGAAAGTAGCTTTACCCCCCTTCCACCAGAATCCACGACAGCTGGGGCA

>237_Sample_ASV11

CACCGCGGTTATACGAGAGGCCCAAGTTGACAGACATCGGCGTAAAGAGTGGTTAAGGAA

ACCCCATTACTAAAGCCGAACGCCCTCAGAACTGTCATACGTTCCCGAAGGTAAGAAGCC

CCACTACGAAAGTGGCTTTAACCACACCCGACCCCACGAAAGCTGTGAAA

>237_Sample_ASV12

CACCGCGGTTATACGAGAGGCCCAAGTTGATACTCCACGGCGTAAAGAGTGGTTAAGGAA

ACCCCATTACTAAAGCCGAACGCCCTCAGAACTGTCATACGTTCCCGAAGGTAAGAAGCC

CCACTACGAAAGTGGCTTTAACCACACCCGACCCCACGAAAGCTGTGAAA

>237_Sample_ASV13

CACCGCGGTTAAACGAGAGGCCCTAGTTGATATTACAACGGCGTAAAGGGTGGTTAAGGA

AAGCAAGCAATAAAGCCGAATGGCCCTTTGGCTGTCATACGCTTCTAGGTGTCCGAAGCC

CAATATACGAAAGTAGCTTTAGAAAAGCCCACCTGACACCACGAAAGCTGAGGAA

>237_Sample_ASV14

CACCGCGGTTATACGAGAGGCCCTAGTTGATAAATACGGCGTAAAGAGTGGTTAGAGGAC

TCCCATAATAAAGCCAAAGACCCTCCGGGCCGTCACACGCATACGAGGGCACGAAGTTCA

CACACGAAAGTAGCTTTAAATTACCCGACGCCACGAAAGCTAAGAAA

>237_Sample_ASV15

CACCGCGGTTATACGAAAGACCCTAGTTTATAAGTACGGCGTAAAGGGTGGTTAGGGGAC

AAACAAAATAAAGCCAAAGACCTCCTAAGCCGTCATACGCTTACGGAGGCACGAAGCCCA

AACACGAAAGTAGCTTTACCGAACATGCCCGACTCCACGAAAGCTGGGAAA

>237_Sample_ASV16

CACCGCGGTTATACGAGAGACCCTAGTTGATAAATACGGCGTAAAGAGTGGTTATGGGAA

AAACAAATAAAGTCAAACAACCTCTTAGCTGTTATACGCATTATGAGAGCATGAAGCCCC

CTCACGAAAGTAACTTTAATACCTCCTGACCCCACGAAAGCTAAGAAA

>237_Sample_ASV17

CACCGCGGTTATACGAGAGGCTCAAGTTGATAAACCCCGGCGTAAAGAGTGGTTAAGGAA

AAATAGAAACTAAAGCCGAATGCTTTCAAGACTGTTATACGTTCCCGAGAGTAAGAAGAC

CAATTACGAAAGTGGCTTTACTTTACCTGACCCCACGAAAGCTACGACA

>237_Sample_ASV18

AACCGCGGTTATACGAGAGACCCTAATTGATAGCTCACGGCGTAAAGGGTGGTTTGGGGA

AACCTCAAAATAAAGCTAAAGACCTTCTAAGCCGTTACACGCATACCGAAGGCACGAGGC

CCTAACACGAAAGTAGCTTTACTATTACCCCTGACGCCACGAAAGCTAAGAAA

>237_Sample_ASV19

CACCGCGGTTATACGAAAGACCCTAGTTGATGGATCACGGCGTAAAGGGTGGTTAAGGGA

TATAAAAATAAAGCTAAAAATCCTCTTAGCTGTCATACGCTTTCCGAAGACATGAGGCCC

AATTACGAAAGTAGCTTTAAACCACCCTGACACCACGAAAGCCAAGAAA

>237_Sample_ASV20

CACCGCGGTTAAACGAGAGGCCCTAGTTGATAGTACAACGGCGTAAAGGGTGGTTAAGGA

TAGTGAAATAATAAAGTCGAATGGCCCTTTGGCTGTCATACGCTTCTAGGAGTCCGAAGC

CCAATATACGAAAGTAACTTTAAGAGCGCCCACCTGACCCCACGAAAACTGAGAAA

>237_Sample_ASV21

CACCGCGGTTATACGAAAGACCCTAGTTGATAGGCACGGCGTAAAGGGTGGTTAGGGGTA

CATCAAAAATAAAGTTAAAGAGCCTCTAAGCTGTCGCACGCATCCCGAGAGCTCGAGACC

CAAACACGAAAGTAACTTTAAAATACTTCACCTGACCCCACGAAAGCTAAGAAA

>237_Sample_ASV22

CACCGCGGTTATACGAGAGGCCCAAGTTGATACTCCACGGCGTAAAGAGTGGTTAGAAAA

GACCTACTACTAAAGCCGAACGCCTTCAAAGCTGTTATACGCATCCGAAGGTGAGAAGCC

CACCCACGAAAGTGGCTTTACAACCTTGACTCCACGAAAGCTATGACA

>237_Sample_ASV23

CACCGCGGTTATACGAGAGACCCCAGTTGATAAACACGGCGTAAAGAGTGGTTAAGGGCA

CAAAACAAATAAAGTCAAACAACCTCTTAGCTGTTATACGCATTATGAGGATATGAAGCC

CCCCTACGAAAGTAACTTTACCAACCCCTGACCCCACGAAAGCTAAGAAA

>237_Sample_ASV24

CACCGCGGTTATACGAGAGACCCTAGTTGATATATACGGCGTAAAGAGTGGTTTGGAATA

ACCAAGTAATATAGCCAAAGACCTCCCAAGCCGTCACACGCACCCCGGAGGCACGAAGCC

CAAACACGAAAGTAGCTTTATTAACTTACCGACGCCACGAAAGCTAAGAAA

>237_Sample_ASV25

CACCGCGGTTATACGAAAGACCCTAGTTTATAGGCACGGCGTAAAGGGTGGTTAGGGGAT

AAACAAAATAAAGCCAAAGACCTTCTAAGTCGTCATACACTCATGAAGGCACGAAGCCCA

AATACGAAAGTAACTTTACAACTTAGACCCGACTCCACGAAAGCTGGGGGA

>237_Sample_ASV26

CACCGCGGTTATACGAGAGGCCCAAGTTGATAGACATCGGCGTAAAGAGTGGTTAAGGAA

GTCTCAAAACTAAAGCCGAATGCCTTCAAGACTGTTATACGTTTCCGAAGGTAAGAAGCC

CCACTACGAAAGTGGCTTTACTCCACCTGACTCCACGAAAGCTGTGAAA

>237_Sample_ASV27

CACCGCGGTTATACGAGAGGCCCAAGTTGACAAACTCCGGCGTAAAGCGTGGTTAAGGCA

CACTCAAAAACTAAAGCCGAATATCTTCAGAGCAGTCATACGCATCCGAAGACACGAAGC

CCCACCACGAAAGTGGCTTTATGACACCTGACCCCACGAAAGCTAGGACA

>237_Sample_ASV28

CACCGCGGTTATACGAAAGACCCTAGTTGATAGACCACGGCGTAAAGGGTGGTTAAGGAA

CACAAAAATAAAGCTAAAGATCCTCTAAGCTGTCATACGCTTCCCGAAGACATGAAACCC

AATCACGAAAGTAGCTTTAAACTGTCCTGACACCACGAAAGCCAAGAAA

>238_Sample_ASV1

CACCGCGGTTATACGAAAGACCCTAGTTGATGGGTGCGGCGTAAAGGGTGGTTAGGGACT

ACAAAAAATAGAGCTAAAGATCCTCTAGGTCGTCATACACATCACGAGGAAACGAAGCCC

AAATACGAAAGTAGCTCTAAATATAATCCTGACGCCACGAAAGCCAAGAAA

>238_Sample_ASV2

CACCGCGGTTATACGAGAGACCCTAGTTGATTAAAGCGGCGTAAAGAGTGGTTATGGAAC

TATTCTTTTAAAGCAGAAAACCTCTCAAACTGTTATACGCACCCAGAGGTCTGAATCCCT

CACACGAAAGTGACTTTATTTATGCCTACCAGAACCCACGAAAGCTGGGACA

>238_Sample_ASV3

CACCGCGGTTATACGAAAGACCCTAGTTGATAGCCACGGCGTAAAGGGTGGTTAAGGAAC

CTAAAAAATAAAGCTAAAGACCCTCTAGGCCGTCATACGCATTACGAGGCAAACGAAACC

CCAACACGAAAGTAGCTTTAGACAAATATCCTGATGCCACGAAAGCCAAGAAA

>238_Sample_ASV4

CACCGCGGTTATACGAGAGGCCCAAGTTGACAGACACCGGCGTAAAGAGTGGCTAGGGAA

AAATTACTACTAAAGCCGAACACCTTCAGAACTGTCATACGTTCCCGAAGATAAGAAGCC

CCACCACGAAAGTGGCTTTATATTACCCGACCCCACGAAAGCTGTGAAA

>238_Sample_ASV5

CACCGCGGTTATACGAGAGGCCCAAGTTGATAAGCCCGGCGTAAAGAGTGGTTATGGAGA

ATAAAACACTAAAGCTAAAGACCCCCTAGGCTGTTATACGCACCTGGCGGCTCGAACCAC

CTATACGAAAGTAGCTTTACCCCCCTTCCACCAGAATCCACGACAGCTGGGGCA

>238_Sample_ASV6

CACCGCGGTTATACGAGAGGCCCTAGTTGATAGCCACGGCGCAAAGAGTGGTTAAGGAGC

CCCACTAAATAAAGTCGAACACTTCCTAGGCCGTTATACGTTTTCTAGAAGCACGAAACC

CAATTACACGAAAGCAACTTTATACTAAACAACCTGACCCCACGAAAGCTAAGAAA

>238_Sample_ASV7

CACCGCGGTTATACGAGAGGCCCAAGTTGACAGACATCGGCGTAAAGAGTGGTTAAGGAA

ACCCCATTACTAAAGCCGAACGCCCTCAGAACTGTCATACGTTCCCGAAGGTAAGAAGCC

CCACTACGAAAGTGGCTTTAACCACACCCGACCCCACGAAAGCTGTGAAA

>238_Sample_ASV8

CACCGCGGTTATACGAGAGACCCCAGTTGATAGCTACGGCGTAAAGAGTGGTTAAGGACC

CCATTTAATAAAGCCAAAGACCCCCCAAGCCGTCATACGCACACGAGGGCGCGAAGCTCG

CACACGAAAGTAGCTCTAATTTACCCGACGCCACGAAAGCTAAGGAA

>238_Sample_ASV9

CACCGCGGTTATACGAGAGACCCTAGTTGATAAATACGGCGTAAAGAGTGGTTATGGGAA

AAACAAATAAAGTCAAACAACCTCTTAGCTGTTATACGCATTATGAGAGCATGAAGCCCC

CTCACGAAAGTAACTTTAATACCTCCTGACCCCACGAAAGCTGAGAAA

>238_Sample_ASV10

CACCGCGGTTATACGAGAGACCCTAGTTGATAAATACGGCGTAAAGAGTGGTTATGGGAA

AAACAAATAAAGTCAAACAACCTCTTAGCTGTTATACGCATTATGAGAGCATGAAGCCCC

CTCACGAAAGTAACTTTAATACCTCCTGACCCCACGAAAGCTAAGAAA

>238_Sample_ASV11

CACCGCGGTTATACGAGAGACCCTAATTGATAGACACGGCGTAAAGAGTGGTTTGGGAAA

GCCCAAAAATAAAGCTAAAGACCTTCTAAGCCGTCACACGCACGCCGAAGGCACGAAGCC

CTGACACGAAAGTAGCTTTACTATTACCCCCGACGCCACGAAAGCTAAGAAA

>238_Sample_ASV12

CACCGCGGTTATACGAAAGACCCTAGTTTATAGATGCGGCGTAAAGGGTGGTTAAGGAAA

ACCTAAAATAATGCTAAAGACTCTCAAAGCTGTTATACGCTTCATGAGCACACGAAATCC

AAATACGAAAGTTGCCTTAATAAACTTTGCCTGACCCCACGAAAGCTGAAAAA

>238_Sample_ASV13

CACCGCGGTTATACGAGAGACCCTAGTTGATAGCTACGGCGTAAAGCGTGGTTAGGAGAA

CAAGATAAATAAAGTCAAACAATCTCTCGGCCGTTATACGTTATCTGAGAATATGAAGTC

CTACCACGAAAGTAACTTTAATTTTTCTGACCCCACGAAAACTGAGGAA

>238_Sample_ASV14

CACCGCGGTTAAACGAGAGGCCCTAGTTGATAGTACAACGGCGTAAAGGGTGGTTAAGGA

TAGTGAAATAATAAAGTCGAATGGCCCTTTGGCTGTCATACGCTTCTAGGAGTCCGAAGC

CCAATATACGAAAGTAACTTTAAGAGCGCCCACCTGACCCCACGAAAACTGAGAAA

>238_Sample_ASV15

CACCGCGGTTATACGAAAGACCCTAGTTGATAGGCACGGCGTAAAGGGTGGTTAGGGATA

TATTAAAAATAAAGTTAAAGAGCCTCTAAGCTGTCGCACGCATCCCGAGAGCTCGAGACC

CAGACACGAAAGTAACTTTAAAATACCTCACCTGACCCCACGAAAGCTAAGAAA

>238_Sample_ASV16

CACCGCGGTTATACGAGAGGCCCAAGTTGATACTCCACGGCGTAAAGAGTGGTTAGAAAA

GACCTACTACTAAAGCCGAACGCCTTCAAAGCTGTTATACGCATCCGAAGGTGAGAAGCC

CACCCACGAAAGTGGCTTTACAACCTTGACTCCACGAAAGCTATGACA

>238_Sample_ASV17

CACCGCGGTTATACGAGAGACCCCAGTTGATAAACACGGCGTAAAGAGTGGTTAAGGGCA

CAAAACAAATAAAGTCAAACAACCTCTTAGCTGTTATACGCATTATGAGGATATGAAGCC

CCCCTACGAAAGTAACTTTACCAACCCCTGACCCCACGAAAGCTAAGAAA

>238_Sample_ASV18

CACCGCGGTTATACGAGAGACCCTAGTTGATATATACGGCGTAAAGAGTGGTTTGGAATA

ACCAAGTAATATAGCCAAAGACCTCCCAAGCCGTCACACGCACCCCGGAGGCACGAAGCC

CAAACACGAAAGTAGCTTTATTAACTTACCGACGCCACGAAAGCTAAGAAA

>238_Sample_ASV19

CACCGCGGTTATACGAAAGACCCTAGTTTATAGGCACGGCGTAAAGGGTGGTTAGGGGAT

AAACAAAATAAAGCCAAAGACCTTCTAAGTCGTCATACACTCATGAAGGCACGAAGCCCA

AATACGAAAGTAACTTTACAACTTAGACCCGACTCCACGAAAGCTGGGGGA

>238_Sample_ASV20

CACCGCGGTTATACGAGAGACCCTAATTGATAGACTACGGCGTAAAGAGTGGTTTAGAAC

AAAAATTAATAAAGCCAAAGATCCTCCTGGCCGTCGCACGCATTTCGAGGATACGAAGCC

CCACTACGAAAGTAGCTTTACTACAACTTTTCTGACCCCACGAAAGCTAAGAAA

>238_Sample_ASV21

CACCGCGGTTATACGAAAGACCCTAGTTGATAGATCACGGCGTAAAGGGTGGTTAAGGAG

AACAAAAATAAAGCTAAAAATCCTCTAAGCTGTCATACGCTTTCCGAAGACATGAGACCC

AACCACGAAAGTAACTTTAAACTTTCCTGACACCACGAAAGCCAAGAAA

>239_Sample_ASV1

TACCGCGGTTATACGAAAGACCCAAGTTAATAAACTACGGCGTAAAGCGTGGTTAGAGAC

CTCCAATAATTAAAGCCAAAGACCTTCTAAGCCGTCGCACGCCCCGCGAAGACACGAAGC

CCAAAAACGAAAGTAGCTTTATTCTTACTCGACCCCACGAAAGCTAGGGCA

>239_Sample_ASV2

CACCGCGGTTATACGAAAGACCCTAGTTGATGGGTGCGGCGTAAAGGGTGGTTAGGGACT

ACAAAAAATAGAGCTAAAGATCCTCTAGGTCGTCATACACATCACGAGAAAACGAAGCCC

AAATACGAAAGTAGCTCTAAATATAATCCTGACGCCACGAAAGCCAAGAAA

>239_Sample_ASV3

CACCGCGGTTATACGAAAGACCCTAGTTGATGGGTGCGGCGTAAAGGGTGGTTAGGGACT

ACAAAAAATAGAGCTAAAGATCCTCTAGGTCGTCATACACATCACGAGGAAACGAAGCCC

AAATACGAAAGTAGCTCTAAATATAATCCTGACGCCACGAAAGCCAAGAAA

>239_Sample_ASV4

CACCGCGGTTAAACGAGAGGCCCTAGTTGATGGTGCAACGGCGTAAAGGGTGGTTAAGGA

TAGCGAAATAATAAAGTCAAATGGCCCTCTGGCTGTCATACGCTTCTAGGAGTCCGAAGC

CCAGTATACGAAAGTAGCTTTAAGAAAGTCCACCTGACCCCACGAAAACTGAGGAA

>239_Sample_ASV5

CACCGCGGTTATACGAAAGACCCTAGTTGCTAGCCACGGCGTAAAGGGTGGTTAAGGACA

ACAACAAATAAAGCTAAAGATCCCCTAAGCCGTCATACGCATTCCGGGGGCACGAAGCCC

TAACACGAAAGTAGCTTTAAAAAATATACCTGACCCCACGAAAGCTAAGAAA

>239_Sample_ASV6

CACCGCGGTTATACGAGAGACCCTAGTTGATTAAAGCGGCGTAAAGAGTGGTTATGGAAC

TATTCTTTTAAAGCAGAAAACCTCTCAAACTGTTATACGCACCCAGAGGTCTGAATCCCT

CACACGAAAGTGACTTTATTTATGCCTACCAGAACCCACGAAAGCTGGGACA

>239_Sample_ASV7

CACCGCGGTTATACGAGAGGCCCAAGTTGACAGACACCGGCGTAAAGAGTGGCTAGGGAA

AAATTACTACTAAAGCCGAACACCTTCAGAACTGTCATACGTTCCCGAAGATAAGAAGCC

CCACCACGAAAGTGACTTTATATTACCCGACCCCACGAAAGCTGTGAAA

>239_Sample_ASV8

CACCGCGGTTATACGAGAGGCCCAAGTTGACAGACACCGGCGTAAAGAGTGGCTAGGGAA

AAATTACTACTAAAGCCGAACACCTTCAGAACTGTCATACGTTCCCGAAGATAAGAAGCC

CCACCACGAAAGTGGCTTTATATTACCCGACCCCACGAAAGCTGTGAAA

>239_Sample_ASV9

CACCGCGGTTATACGAAAGACTCTAGTTGACAAACACGGCGTAAAGGGTGGTTAGGATAA

CAACAAAATAAAGCTAAAGACTTACCAAGCCGTCATACGCCCATGTAAGAGCGAAAACCA

CAAACGAAAGTAACTTTATTACACCATCCGAATCCACGAAAGCTAAGAAA

>239_Sample_ASV10

CACCGCGGTTATACGAAAGACCCTAGTTGATAGATCACGGCGTAAAGGGTGGTTAGGGAA

TACAAAAATAAAGCTAAAAATCCTCTAAGCTGTCATACGCTTCCCGAAGACATGAAACCC

AACCACGAAAGTAGCTTTAAACTCTCCTGACACCACGAAAGCCAAGGAA

>239_Sample_ASV11

CACCGCGGTTATACGAGAGACCCTAGTTGATAAGCCCGGCGTAAAGAGTGGTTATGGAGA

ATAAAACACTAAAGCTAAAGACCCCCTAGGCTGTTATACGCACCTGGCGGCTCGAACCAC

CTATACGAAAGTAGCTTTACCCCCCTTCCACCAGAATCCACGACAGCTGGGGCA

>239_Sample_ASV12

CACCGCGGTTATACGAGAGGCCCAAGTTGATAAGCCCGGCGTAAAGAGTGGTTATGGAGA

ATAAAACACTAAAGCTAAAGACCCCCTAGGCTGTTATACGCACCTGGCGGCTCGAACCAC

CTATACGAAAGTAGCTTTACCCCCCTTCCACCAGAATCCACGACAGCTGGGGCA

>239_Sample_ASV13

CACCGCGGTTATACGAGAGGCCCAAGTTGACAGACATCGGCGTAAAGAGTGGTTAAGGAA

ACCCCATTACTAAAGCCGAACGCCCTCAGAACTGTCATACGTTCCCGAAGGTAAGAAGCC

CCACTACGAAAGTGGCTTTAACCACACCCGACCCCACGAAAGCTGTGAAA

>239_Sample_ASV14

CACCGCGGTTATACGAGAGGCCCTAGTTGATATTACAACGGCGTAAAGGGTGGTTAAGGA

AAGCAAGCAATAAAGCCGAATGGCCCTTTGGCTGTCATACGCTTCTAGGTGTCCGAAGCC

CAATATACGAAAGTAGCTTTAGAAAAGCCCACCTGACACCACGAAAGCTGAGGAA

>239_Sample_ASV15

CACCGCGGTTAAACGAGAGGCCCTAGTTGATATTACAACGGCGTAAAGGGTGGTTAAGGA

AAGCAAGCAATAAAGCCGAATGGCCCTTTGGCTGTCATACGCTTCTAGGTGTCCGAAGCC

CAATATACGAAAGTAGCTTTAGAAAAGCCCACCTGACACCACGAAAGCTGAGGAA

>239_Sample_ASV16

CACCGCGGTTATACGAGAGGCCCCAGTTGATATTAGCGGCGTAAAGGGTGGTTAAGGACT

TTAATAACTAAAGCTAAACACTTCCCCGGCTGTTGTACGTAGACCGGAAGCTTGAAGCCC

AGACACGAAAGTAGCTTTATTTCCCCTGACCCCACGAAAGCTGAGAAA

>239_Sample_ASV17

CACCGCGGTTATACGAGAGGCCCCAGTTGATATTAGCGGCGTAAAGGGTGGTTAAGGACT

TTAATAACTAAAGCCAAACACTTCCCCGGCTGTTGTACGTAGACCGGAAGCTTGAAGCCC

AGACACGAAAGTAGCTTTATTTCCCCTGACCCCACGAAAGCTGAGAAA

>239_Sample_ASV18

CACCGCGGTTATACGAGAGGCCCCAGTTGATATTAGCGGCGTAAAGGGTGGTTAAGGACT

TTAATAACTAAAGCTAAACACTTCCCCGGCTGTTGTACGTAGACCGGAAGCTTGAAGCCC

AGACACGAAAGTAGCTTTATTCCCCCTGACCCCACGAAAGCTGAGAAA

>239_Sample_ASV19

CACCGCGGTCACACGATTAACCCAAGTCAATAGAAGCCGGCGTAAAGAGTGTTTTAGATC

ACCCCCTCCCCAATAAAGCTAAAACTCACCTGAGTTGTAAAAAACTCCAGTTGACACAAA

ATAGACTACGAAAGTGGCTTTAACATATCTGAACACACAATAGCTAAGACC

>239_Sample_ASV20

CACCGCGGTTATACGAGAAACCCTAGTTGACAAATACGGCGTAAAGAGTGGTTAAGGGGT

ATCAAAAATAAAGTCAAACATTTTCCTAGTTGTTATAAGCATTTCGAGAACATGAAGCCC

AACCACGAAAGTAACTTTACCTTCACCTGACCCCACGAAAGCTAAGAAA

>239_Sample_ASV21

CACCGCGGTTATACGAAAGACCCCAGTTTATAGACACGGCGTAAAGGGTGGTTATAGGAC

AAACTAACATAGAGCCAAAGACCCCCTAAGCCGTCATACGCTCACGAGGGCTCGAAGCCC

CTATACGAAAGTAGCTCTACCAAAAAATGCTCGACTCCACGAAAGCTGGGACA

>239_Sample_ASV22

CACCGCGGTTATACGAGAGACCCTAGTTGATAAATACGGCGTAAAGAGTGGTTATGGGAA

AAACAAATAAAGTCAAACAACCTCTTAGCTGTTATACGCATTATGAGAGCATGAAGCCCC

CTCACGAAAGTAACTTTAATACCTCCTGACCCCACGAAAGCTAAGAAA

>239_Sample_ASV23

CACCGCGGTTATACGAGAGGCTCAAGTTGATAAACCCCGGCGTAAAGAGTGGTTAAGGAA

AAATAGAAACTAAAGCCGAATGCTTTCAAGACTGTTATACGTTCCCGAGAGTAAGAAGAC

CAATTACGAAAGTGGCTTTACTTTACCTGACCCCACGAAAGCTACGACA

>239_Sample_ASV24

CACCGCGGTTATACGAGAGACCCTAATTGATAGGCACGGCGTAAAGAGTGGTTTAGGGTA

ACCCATAAATAAAGCTAAAGACCTTCTAAGCCGTCACACGCACACCGAAGGCACGAGACC

CCAACACGAAAGTAGCTTTACTTCTACCCCTGACGCCACGAAAGCTAAGAAA

>239_Sample_ASV25

CACCGCGGTTATACGAAAGACCCTAGTTGATGGATCACGGCGTAAAGGGTGGTTAAGGGA

TATAAAAATAAAGCTAAAAATCCTCTTAGCTGTCATACGCTTTCCGAAGACATGAGGCCC

AATTACGAAAGTAGCTTTAAACCACCCTGACACCACGAAAGCCAAGAAA

>239_Sample_ASV26

CACCGCGGTTATACGAAAGACCCCAGTTTATAGATGCGGCGTAAAGGGTGGTTAAGAGAA

AATCAAAATAAGGCCAAAGATCCCCTAAGCAGTCATACGCTCCACGAGGACACGAAACCC

AAATACGAAAGTTGCCTTAATAAACTTCACCTGATCCCACGAAAGCTGAAAAA

>239_Sample_ASV27

CACCGCGGTTATACGAAAGGCTCTAGTTTATAAATACGGCGTAAAGGGTGGTTAAGGAAA

AATTAAAATAATGCTAAAGACCCCCTAAGCAGTTATACGCTTCACGGAGGCACGAAACCC

AAATACGAAAGTTGCCTTAATAAACTTTACCTGACCCCACGAAAGCTGAAAAA

>239_Sample_ASV28

CACCGCGGTTATACGAGAGACTCTAGTTGATACCAACGGCGTAAAGAGTGGTTTGGGATG

CCCCTTAAAATAAAGCCAAAGACCTCCCAAGCCGTCAAACGCACCCCGGAGGCACGAAGC

CCCAACGCGAAAGCAACTTTACCTCCCTCCCGACGCCACGAAAGCTAAGAAA

>239_Sample_ASV29

CACCGCGGTTATACGAGAGACCCTAGTTGATAGCTACGGCGTAAAGCGTGGTTAGGAGAA

CAAGATAAATAAAGTCAAACAATCTCTCGGCCGTTATACGTTATCTGAGAATATGAAGTC

CTACCACGAAAGTAACTTTAATTTTTCTGACCCCACGAAAACTGAGGAA

>239_Sample_ASV30

CACCGCGGTTATACGAGAGGCCCTAGTTGATAACTACCGGCGTAAAGAGTGGTTATGGAA

AAATATTTAATAAAGCCGAACACCCCCTCAGCCGTCATACGCACCTGGGAGCACGAAGAC

CTACTGCGAAAGCAGCTTTAATTATGCCTGACCCCACGACAGCTAAGAAA

>239_Sample_ASV31

CACCGCGGTTATACGAGAGGCCCAAGTTGATTATTACCGGCGTAAAGCGTGGTTAAGATT

TTTATAGGACTAAAGCCGAACCCCCTCAGAGCTGTTATACGCACCCGAAGGTTAGAAGTT

CAATCACGAAAGTGGCTTTACAACCCCGAACCCACGAAAGCTAAGATA

>239_Sample_ASV32

CACCGCGGTTATACGAGAGGCCCAAGTTGATACTCCACGGCGTAAAGAGTGGTTAGAAAA

GACCTACTACTAAAGCCGAACGCCTTCAAAGCTGTTATACGCATCCGAAGGTGAGAAGCC

CACCCACGAAAGTGGCTTTACAACCTTGACTCCACGAAAGCTATGACA

>239_Sample_ASV33

CACCGCGGTTATACGAGAGGCCCAAGTTGATAGTCTTCGGCGTAAAGAGTGGTTAAAGAA

GCAATTAGACTAAGGCTAAACTCTTCCAAGGCTGTCATACGCACCCGGAAGCATGAAACC

CAACCACGAAAGTGGCCTTATTTACCTTGACCCCACGAAAGCTGTGAAA

>239_Sample_ASV34

CACCGCGGTTATACGAGAGACCCCAGTTGATAAACACGGCGTAAAGAGTGGTTAAGGGCA

CAAAACAAATAAAGTCAAACAACCTCTTAGCTGTTATACGCATTATGAGGATATGAAGCC

CCCCTACGAAAGTAACTTTACCAACCCCTGACCCCACGAAAGCTAAGAAA

>239_Sample_ASV35

CACCGCGGTTATACGAAAGACCCTAGTTTATAGGCACGGCGTAAAGGGTGGTTAGGGGAT

AAACAAAATAAAGCCAAAGACCTTCTAAGTCGTCATACACTCATGAAGGCACGAAGCCCA

AATACGAAAGTAACTTTACAACTTAGACCCGACTCCACGAAAGCTGGGGGA

>239_Sample_ASV36

CACCGCGGTTATACGAGAGACCCTAATTAATAGATACGGCGTAAAGAGTGGTTTAGGACA

AAAATTAATAAAGCCGAAGTTTCTCCTAGCCGTTGTACGCACTCTGAGAACACGAAGCCC

CACTACGAAAGTAGCTTTACCTATTATTTCCTGACCCCACGAAAGCTAAGAAA

>239_Sample_ASV37

CACCGCGGTTATACGAGAGGCCCAAGTTGACAAACTCCGGCGTAAAGCGTGGTTAAGGCA

CACTCAAAAACTAAAGCCGAATATCTTCAGAGCAGTCATACGCATCCGAAGACACGAAGC

CCCACCACGAAAGTGGCTTTATGACACCTGACCCCACGAAAGCTAGGACA

>239_Sample_ASV38

CACCGCGGTTATACGAAAGACCCTAGTTGATAGCTACGGCGTAAAGGGTGGTTAAGGTAT

ATAACAAATAAAGCTAAAGAGCCTCCAAGCCGTCGTACGCATTCTGAGAGCTCGAAACCC

TAACACGAAAGTAGCTTTAAAAAATAACACCTGACCCCACGAAAGCTAAGAAA

>239_Sample_ASV39

CACCGCGGTTATACGAAAGACCCTAGTTGATAGATCACGGCGTAAAGGGTGGTTAAGGAG

AACAAAAATAAAGCTAAAAATCTTCTAAGCTGTCATACGCTTTCCGAAGACATGAGACCC

AACCACGAAAGTAGCTTTAAACTTTCCTGACACCACGAAAGCCAAGAAA

>240_Sample_ASV1

CACCGCGGTTATACGAAAGACCCTAGTTGATGGGTGCGGCGTAAAGGGTGGTTAGGGACT

ACAAAAAATAGAGCTAAAGATCCTCTAGGTCGTCATACACATCACGAGGAAACGAAGCCC

AAATACGAAAGTAGCTCTAAATATAATCCTGACGCCACGAAAGCCAAGAAA

>240_Sample_ASV2

CACCGCGGTTATACGAGAGACCCTAGTTGATTAAAGCGGCGTAAAGAGTGGTTATGGAAC

TATTCTTTTAAAGCAGAAAACCTCTCAAACTGTTATACGCACCCAGAGGTCTGAATCCCT

CACACGAAAGTGACTTTATTTATGCCTACCAGAACCCACGAAAGCTGGGACA

>240_Sample_ASV3

CACCGCGGTTATACGAGAGGCCCAAGTTGACAGACACCGGCGTAAAGAGTGGCTAGGGAA

AAATTACTACTAAAGCCGAACACCTTCAGAACTGTCATACGTTCCCGAAGATAAGAAGCC

CCACCACGAAAGTGGCTTTATATTACCCGACCCCACGAAAGCTGTGAAA

>240_Sample_ASV4

CACCGCGGTTATACGAGAGGCCCAAGTTGACAGACACCGGCGTAAAGAGTGGCTAGGGAA

AAATTACTACTAAAGCCGAACACCTTCAGAACTGTCATACGTTCCCGAAGATAAGAAGCC

CCACCACGAAAGTGGCTTTATATTACCCGACCCCACGAAAGCTAAGAAA

>240_Sample_ASV5

CACCGCGATTATACGAGAGGCCCAAGTTGACAGACATCGGCGTAAAGAGTGGTTAAGGAA

AAATTACTACTAAAGCCGAACACCTTCAGAACTGTCATACGTTCCCGAAGATAAGAAGCC

CCACCACGAAAGTGGCTTTATATTACCCGACCCCACGAAAGCTGTGAAA

>240_Sample_ASV6

CACCGCGGTTATACGAGAGGCCCAAGTTGACAGACACCGGCGTAAAGAGTGGCTAGGGAA

AAATTACTACTAAAGCCGAACACCTTCAGAACTGTCATACGTTCCCGAAGATAAGAAGCT

CCACCACGAAAGTGGCTTTATAATACGTGACCCCACGAAAGCTGTGAAA

>240_Sample_ASV7

CACCGCGGTTATACGAGAGGCCCAAGTTGACAGACACCGGCGTAAAGAGTGGCTAGGGAA

AAATTACTACTAAAGCCGAACACCTTCAGAACTGTCATACGTTCCCGAAGATAAGAAGCC

CCACCACGAAAGTGGCTTTACAATGCCTGACCCCACGAAAGCTGTGAAA

>240_Sample_ASV8

CACCGCGGTTATACGAGAGGCCCAAGTTGACAGACACCGGCGTAAAGAGTGGCTAGGGAA

AAATTACTACTAAAGCCGAACACCCTCAGAACTGTCATACGTTCCAGAAGGCAAGAAGCT

CCACCACGAAAGTGGCTTTATATTACCCGACCCCACGAAAGCTGTGAAA

>240_Sample_ASV9

CACCGCGGTTATACGAGAGGCCCAAGTTGACAGACACCGGCGTAAAGAGTGGCTAAGGAA

AAATTTCTACTAAAGCCGAACACCTTCAGAACTGTCATACGTTCCCGAAGAAAAGAAGCC

CCACCACGAAAGTGGCTTTATATTACCCGACCCCACGAAAGCTGTGAAA

>240_Sample_ASV10

CACCGCGGTTAAACGAGAGACCCTAGTTGATCATCACGGCGTAAAGAGTGGTTAGGGGAT

TATCATAAATAAAGCCAAAGACCTCCCAAGCTGTCGCACGCATTCCGGAGGCGCGAAGCC

CACACACGAAAGTAGCTTTAACTATTGACCCTGATGCCACGAAAGCTAAGAAA

>240_Sample_ASV11

CGCCGCGGTTAAACGAGAGGCCCTAGTTGATGGAACAACGGCGTAAAGGGTGGTTAAGGA

TAGTGAGACAATAAAGTCGAATGGCCCTTTGGCTGTCATACGCTTCTGGGAGCCTGAAGC

CCACTATACGAAAGTAGCTTTAACAATGTCCACCTGACCCCACGAAAACTGAGAAA

>240_Sample_ASV12

CGCCGCGGTTAAACGAGAGGCCCTAGTTGATGGAACAACGGCGTAAAGGGTGGTTAAGGA

TAGTGAGACAATAAAGTCGAATGGCCCTTTGGCTGTCATACGCTTCTGGGAGCCTGAAGC

CCACTATACGAAAGTAGCTTTAACAACGTCCACCTGACCCCACGAAAACTGAGAAA

>240_Sample_ASV13

CACCGCGGTTATACGAAAGACCCTAGTTGATAGATCACGGCGTAAAGGGTGGTTAGGGAA

TACAAAAATAAAGCTAAAAATCCTCTAAGCTGTCATACGCTTCCCGAAGACATGAAACCC

AACCACGAAAGTAGCTTTAAACTCTCCTGACACCACGAAAGCCAAGGAA

>240_Sample_ASV14

CACCGCGGTTATACGAGAGGCCCAAGTTGATAAGCCCGGCGTAAAGAGTGGTTATGGAGA

ATAAAACACTAAAGCTAAAGACCCCCTAGGCTGTTATACGCACCTGGCGGCTCGAACCAC

CTATACGAAAGTAGCTTTACCCCCCTTCCACCAGAATCCACGACAGCTGGGGCA

>240_Sample_ASV15

CACCGCGGTTATACGAGAGGCCCAAGTTGACAGACATCGGCGTAAAGAGTGGTTAAGGAA

ACCCCATTACTAAAGCCGAACGCCCTCAGAACTGTCATACGTTCCCGAAGGTAAGAAGCC

CCACTACGAAAGTGGCTTTAACCACACCCGACCCCACGAAAGCTGTGAAA

>240_Sample_ASV16

CACCGCGGTTATACGAGAGGCCCAAGTTGATAGATATCGGCGTAAAGAGTGGTTAAGGAA

ACCCCGTTACTAAAGCCGAACGCCCTCAGAACTGTCATACGTTCCCGAAGGTAAGAAGCC

CCACTACGAAAGTGGCTTTAACCACACCCGACCCCACGAAAGCTGTGAAA

>240_Sample_ASV17

CACCGCGGTTAAACGAGAGGCCCTAGTTGATATTACAACGGCGTAAAGGGTGGTTAAGGA

AAGCAAGCAATAAAGCCGAATGGCCCTTTGGCTGTCATACGCTTCTAGGTGTCCGAAGCC

CAATATACGAAAGTAGCTTTAGAAAAGCCCACCTGACACCACGAAAGCTGAGGAA

>240_Sample_ASV18

CACCGCGGTTATACGAGAGGCCCTAGTTGATAAATACGGCGTAAAGAGTGGTTAGAGGAC

TCCCATAATAAAGCCAAAGACCCTCCGGGCCGTCACACGCATACGAGGGCACGAAGTTCA

CACACGAAAGTAGCTTTAAATTACCCGACGCCACGAAAGCTAAGAAA

>240_Sample_ASV19

CACCGCGGTTATACGAAAGACCCTAGTTTATAAGTACGGCGTAAAGGGTGGTTAGGGGAC

AAACAAAATAAAGCCAAAGACCTCCTAAGCCGTCATACGCTTACGGAGGCACGAAGCCCA

AACACGAAAGTAGCTTTACCGAACATGCCCGACTCCACGAAAGCTGGGAAA

>240_Sample_ASV20

CACCGCGGTTATACGAGAGACCCTAGTTGATAAATACGGCGTAAAGAGTGGTTATGGGAA

AAACAAATAAAGTCAAACAACCTCTTAGCTGTTATACGCATTATGAGAGCATGAAGCCCC

CTCACGAAAGTAACTTTAATACCTCCTGACCCCACGAAAGCTAAGAAA

>240_Sample_ASV21

CACCGCGGTTATACGAGAGGCTCAAGTTGATAAACCCCGGCGTAAAGAGTGGTTAAGGAA

AAATAGAAACTAAAGCCGAATGCTTTCAAGACTGTTATACGTTCCCGAGAGTAAGAAGAC

CAATTACGAAAGTGGCTTTACTTTACCTGACCCCACGAAAGCTACGACA

>240_Sample_ASV22

AACCGCGGTTATACGAGAGACCCTAATTGATAGCTCACGGCGTAAAGGGTGGTTTGGGGA

AACCTCAAAATAAAGCTAAAGACCTTCTAAGCCGTTACACGCATACCGAAGGCACGAGGC

CCTAACACGAAAGTAGCTTTACTATTACCCCTGACGCCACGAAAGCTAAGAAA

>240_Sample_ASV23

CACCGCGGTTATACGAAAGACCCTAGTTGATGGATCACGGCGTAAAGGGTGGTTAAGGGA

TATAAAAATAAAGCTAAAAATCCTCTTAGCTGTCATACGCTTTCCGAAGACATGAGGCCC

AATTACGAAAGTAGCTTTAAACCACCCTGACACCACGAAAGCCAAGAAA

>240_Sample_ASV24

CACCGCGGTTAAACGAGAGGCCCTAGTTGATAGTACAACGGCGTAAAGGGTGGTTAAGGA

TAGTGAAATAATAAAGTCGAATGGCCCTTTGGCTGTCATACGCTTCTAGGAGTCCGAAGC

CCAATATACGAAAGTAACTTTAAGAGCGCCCACCTGACCCCACGAAAACTGAGAAA

>240_Sample_ASV25

CACCGCGGTTATACGAGAGGCCCAAGTTGATACTCCACGGCGTAAAGAGTGGTTAGAAAA

GACCTACTACTAAAGCCGAACGCCTTCAAAGCTGTTATACGCATCCGAAGGTGAGAAGCC

CACCCACGAAAGTGGCTTTACAACCTTGACTCCACGAAAGCTATGACA

>240_Sample_ASV26

CACCGCGGTTATACGAGAGACCCCAGTTGATAAACACGGCGTAAAGAGTGGTTAAGGGCA

CAAAACAAATAAAGTCAAACAACCTCTTAGCTGTTATACGCATTATGAGGATATGAAGCC

CCCCTACGAAAGTAACTTTACCAACCCCTGACCCCACGAAAGCTAAGAAA

>240_Sample_ASV27

CACCGCGGTTATACGAGAGACCCTAGTTGATATATACGGCGTAAAGAGTGGTTTGGAATA

ACCAAGTAATATAGCCAAAGACCTCCCAAGCCGTCACACGCACCCCGGAGGCACGAAGCC

CAAACACGAAAGTAGCTTTATTAACTTACCGACGCCACGAAAGCTAAGAAA

>240_Sample_ASV28

CACCGCGGTTATACGAAAGACCCTAGTTTATAGGCACGGCGTAAAGGGTGGTTAGGGGAT

AAACAAAATAAAGCCAAAGACCTTCTAAGTCGTCATACACTCATGAAGGCACGAAGCCCA

AATACGAAAGTAACTTTACAACTTAGACCCGACTCCACGAAAGCTAAGAAA

>240_Sample_ASV29

CACCGCGGTTATACGAAAGACCCTAGTTTATAGGCACGGCGTAAAGGGTGGTTAGGGGAT

AAACAAAATAAAGCCAAAGACCTTCTAAGTCGTCATACACTCATGAAGGCACGAAGCCCA

AATACGAAAGTAACTTTACAACTTAGACCCGACTCCACGAAAGCTGGGGGA

>240_Sample_ASV30

CACCGCGGTTATACGAGAGGCCCAAGTTGATAGACATCGGCGTAAAGAGTGGTTAAGGAA

GTCTCAAAACTAAAGCCGAATGCCTTCAAGACTGTTATACGTTTCCGAAGGTAAGAAGCC

CCACTACGAAAGTGGCTTTACTCCACCTGACTCCACGAAAGCTGTGAAA

>240_Sample_ASV31

CACCGCGGTTATACGAGAGGCCCAAGTTGACAAACTCCGGCGTAAAGCGTGGTTAAGGCA

CACTCAAAAACTAAAGCCGAATATCTTCAGAGCAGTCATACGCATCCGAAGACACGAAGC

CCCACCACGAAAGTGGCTTTATGACACCTGACCCCACGAAAGCTAGGACA

>240_Sample_ASV32

CACCGCGGTTATACGAGAGACTCCAGTTGATAGCTACGGCGTAAAGAGTGGTTTGGGGCC

CGCCCAAAATAAAGCCAAAGACCTCCCAAGCCGTCAAACGCACCCCGGAGGCACGAAGTC

CTAACGCGAAAGCAACTTTACCTTCCCCGACGCCACGAAAGCTAAGAAA

>240_Sample_ASV33

CACCGCGGTTATACGAAAGACCCTAGTTGATAGACCACGGCGTAAAGGGTGGTTAAGGAA

CACAAAAATAAAGCTAAAGATCCTCTAAGCTGTCATACGCTTCCCGAAGACATGAAACCC

AATCACGAAAGTAGCTTTAAACTGTCCTGACACCACGAAAGCCAAGAAA

>242_Sample_ASV1

CACCGCGGTTATACGAGAGGCCCAAGTTGACAGACATCGGCGTAAAGAGTGGTTAAGGAA

AACCTTCAACTAAAGTCGAACGCTCTCAAGACTGTCGTACGTTTCCGAGAGTATGAAGCC

CCACCACGAAAGTGACTTTACCTCACCTGACTCCACGAAAGCTGCGAAA

>242_Sample_ASV2

CACCACGGTTATACGAGAGGCCCAAGTTGAAAGACATCGGCGTAAAGAGTGGTTAAGGAA

AACCTTCTACTAAAGTCGAACGCTCTCAGGACTGTCGTACGTTTCCGAGAGTATGAAGCC

CCACCACGAAAGTGACTTTACCTCACCTGACTCCACGAAAGCTGCGAAA

>242_Sample_ASV3

CACCGCGGTTATACGAAAGACCCTAGTTGATGGGTGCGGCGTAAAGGGTGGTTAGGGACT

ACAAAAAATAGAGCTAAAGATCCTCTAGGTCGTCATACACATCACGAGGAAACGAAGCCC

AAATACGAAAGTAGCTCTAAATATAATCCTGACGCCACGAAAGCCAAGAAA

>242_Sample_ASV4

CACCGCGGTTATACGAGAGACCCTAGTTGATTAAAGCGGCGTAAAGAGTGGTTATGGAAC

TATTCTTTTAAAGCAGAAAACCTCTCAAACTGTTATACGCACCCAGAGGTCTGAATCCCT

CACACGAAAGTGACTTTATTTATGCCTACCAGAACCCACGAAAGCTGGGACA

>242_Sample_ASV5

CACCGCGGTTATACGAAAGACCCTAGTTTATAGGTACGGCGTAAAGGGTGGTTAGGGGAC

AAACAAAATAAAGCCAAAGACCTCCTAAGCCGTCATACGCTCACGGAAGCACGAAGCCCA

AACACGAAAGTAGCTTTACCGAACATGCCCGACTCCACGAAAGCTGGGGAA

>242_Sample_ASV6

CACCGCGGTTATACGAAAGACCCTAGTTGATAGCCACGGCGTAAAGGGTGGTTAAGGAAC

CTAAAAAATAAAGCTAAAGACCCTCTAGGCCGTCATACGCATTACGAGGCAAACGAAACC

CCAACACGAAAGTAGCTTTAGACAAATATCCTGATGCCACGAAAGCCAAGAAA

>242_Sample_ASV7

CACCGCGGTTATACGAGAGGCCCAAGTTGACAGACACCGGCGTAAAGAGTGGCTAGGGAA

AAATTACTACTAAAGCCGAACACCTTCAGAACTGTCATACGTTCCCGAAGATAAGAAGCC

CCACCACGAAAGTGGCTTTATATTACCCGACCCCACGAAAGCTAAGAAA

>242_Sample_ASV8

CACCGCGGTTATACGAGAGGCCCAAGTTGACAGACACCGGCGTAAAGAGTGGCTAGGGAA

AAATTACTACTAAAGCCGAACACCTTCAGAACTGTCATACGTTCCCGAAGATAAGAAGCC

CCACCACGAAAGTGGCTTTATATTACCCGACCCCACGAAAGCTGTGAAA

>242_Sample_ASV9

CACCGCGGTTATACGAGAGGCCCAAGTTGACAGACACCGGCGTAAAGAGTGGCTAAGGAA

AAATTTCTACTAAAGCCGAACACCTTCAGAACTGTCATACGTTCCCGAAGAAAAGAAGCC

CCACCACGAAAGTGGCTTTATATTACCCGACCCCACGAAAGCTGTGAAA

>242_Sample_ASV10

CACCGCGGTTATACGAAAGACTCTAGTTGACAAACACGGCGTAAAGGGTGGTTAGGATAA

CAACAAAATAAAGCTAAAGACTTACCAAGCCGTCATACGCCCATGTAAGAGCGAAAACCA

CAAACGAAAGTAACTTTATTACACCATCCGAATCCACGAAAGCTAAGAAA

>242_Sample_ASV11

CACCGCGGTTAAACGAGAGACCCTAGTTGATCATCACGGCGTAAAGAGTGGTTAGGGGAT

TATCATAAATAAAGCCAAAGACCTCCCAAGCTGTCGCACGCATTCCGGAGGCGCGAAGCC

CACACACGAAAGTAGCTTTAACTATTGACCCTGATGCCACGAAAGCTAAGAAA

>242_Sample_ASV12

CGCCGCGGTTAAACGAGAGGCCCTAGTTGATGGAACAACGGCGTAAAGGGTGGTTAAGGA

TAGTGAGACAATAAAGTCGAATGGCCCTTTGGCTGTCATACGCTTCTGGGAGCCTGAAGC

CCACTATACGAAAGTAGCTTTAACAATGTCCACCTGACCCCACGAAAACTGAGAAA

>242_Sample_ASV13

CGCCGCGGTTAAACGAGAGGCCCTAGTTGATGGAACAACGGCGTAAAGGGTGGTTAAGGA

TAGTGAGACAATAAAGTCGAATGGCCCTTTGGCTGTCATACGCTTCTGGGAGCCTGAAGC

CCACTATACGAAAGTAGCTTTAACAACGTCCACCTGACCCCACGAAAACTGAGAAA

>242_Sample_ASV14

CACCGCGGTTATACGAAAGACCCTAGTTGATAGATCACGGCGTAAAGGGTGGTTAGGGAA

TACAAAAATAAAGCTAAAAATCCTCTAAGCTGTCATACGCTTCCCGAAGACATGAAACCC

AACCACGAAAGTAGCTTTAAACTCTCCTGACACCACGAAAGCCAAGGAA

>242_Sample_ASV15

CACCGCGGTTATACGAGAGGCCCAAGTTGATAAGCCCGGCGTAAAGAGTGGTTATGGAGA

ATAAAACACTAAAGCTAAAGACCCCCTAGGCTGTTATACGCACCTGGCGGCTCGAACCAC

CTATACGAAAGTAGCTTTACCCCCCTTCCACCAGAATCCACGACAGCTGGGGCA

>242_Sample_ASV16

CACCGCGGTTATACGAGAGGCCCAAGTTGACAGACATCGGCGTAAAGAGTGGTTAAGGAA

ACCCCATTACTAAAGCCGAACGCCCTCAGAACTGTCATACGTTCCCGAAGGTAAGAAGCC

CCACTACGAAAGTGGCTTTAACCACACCCGACCCCACGAAAGCTGTGAAA

>242_Sample_ASV17

CACCGCGGTTATACGAGAGGCCCAAGTTGATAGATATCGGCGTAAAGAGTGGTTAAGGAA

ACCCCGTTACTAAAGCCGAACGCCCTCAGAACTGTCATACGTTCCCGAAGGTAAGAAGCC

CCACTACGAAAGTGGCTTTAACCACACCCGACCCCACGAAAGCTGTGAAA

>242_Sample_ASV18

CACCGCGGTTAAACGAGAGGCCCTAGTTGATATTACAACGGCGTAAAGGGTGGTTAAGGA

AAGCAAGCAATAAAGCCGAATGGCCCTTTGGCTGTCATACGCTTCTAGGTGTCCGAAGCC

CAATATACGAAAGTAGCTTTAGAAAAGCCCACCTGACACCACGAAAGCTGAGGAA

>242_Sample_ASV19

CACCGCGGTTATACGAGAGGCCCTAGTTGATAAATACGGCGTAAAGAGTGGTTAGAGGAC

TCCCATAATAAAGCCAAAGACCCTCCGGGCCGTCACACGCATACGAGGGCACGAAGTTCA

CACACGAAAGTAGCTTTAAATTACCCGACGCCACGAAAGCTAAGAAA

>242_Sample_ASV20

CACCGCGGTTATACGAAAGACCCTAGTTTATAAGTACGGCGTAAAGGGTGGTTAGGGGAC

AAACAAAATAAAGCCAAAGACCTCCTAAGCCGTCATACGCTTACGGAGGCACGAAGCCCA

AACACGAAAGTAGCTTTACCGAACATGCCCGACTCCACGAAAGCTGGGAAA

>242_Sample_ASV21

CACCGCGGTTATACGAGAGACCCTAGTTGATAAATACGGCGTAAAGAGTGGTTATGGGAA

AAACAAATAAAGTCAAACAACCTCTTAGCTGTTATACGCATTATGAGAGCATGAAGCCCC

CTCACGAAAGTAACTTTAATACCTCCTGACCCCACGAAAGCTAAGAAA

>242_Sample_ASV22

CACCGCGGTTATACGAGAGGCTCAAGTTGATAAACCCCGGCGTAAAGAGTGGTTAAGGAA

AAATAGAAACTAAAGCCGAATGCTTTCAAGACTGTTATACGTTCCCGAGAGTAAGAAGAC

CAATTACGAAAGTGGCTTTACTTTACCTGACCCCACGAAAGCTACGACA

>242_Sample_ASV23

AACCGCGGTTATACGAGAGACCCTAATTGATAGCTCACGGCGTAAAGGGTGGTTTGGGGA

AACCTCAAAATAAAGCTAAAGACCTTCTAAGCCGTTACACGCATACCGAAGGCACGAGGC

CCTAACACGAAAGTAGCTTTACTATTACCCCTGACGCCACGAAAGCTAAGAAA

>242_Sample_ASV24

CACCGCGGTTATACGAAAGACCCTAGTTGATGGATCACGGCGTAAAGGGTGGTTAAGGGA

TATAAAAATAAAGCTAAAAATCCTCTTAGCTGTCATACGCTTTCCGAAGACATGAGGCCC

AATTACGAAAGTAGCTTTAAACCACCCTGACACCACGAAAGCCAAGAAA

>242_Sample_ASV25

CACCGCGGTTAAACGAGAGGCCCTAGTTGATAGTACAACGGCGTAAAGGGTGGTTAAGGA

TAGTGAAATAATAAAGTCGAATGGCCCTTTGGCTGTCATACGCTTCTAGGAGTCCGAAGC

CCAATATACGAAAGTAACTTTAAGAGCGCCCACCTGACCCCACGAAAACTGAGAAA

>242_Sample_ASV26

CACCGCGGTTATACGAGAGGCCCAAGTTGATACTCCACGGCGTAAAGAGTGGTTAGAAAA

GACCTACTACTAAAGCCGAACGCCTTCAAAGCTGTTATACGCATCCGAAGGTGAGAAGCC

CACCCACGAAAGTGGCTTTACAACCTTGACTCCACGAAAGCTATGACA

>242_Sample_ASV27

CACCGCGGTTATACGAGAGACCCCAGTTGATAAACACGGCGTAAAGAGTGGTTAAGGGCA

CAAAACAAATAAAGTCAAACAACCTCTTAGCTGTTATACGCATTATGAGGATATGAAGCC

CCCCTACGAAAGTAACTTTACCAACCCCTGACCCCACGAAAGCTAAGAAA

>242_Sample_ASV28

CACCGCGGTTATACGAGAGACCCTAGTTGATATATACGGCGTAAAGAGTGGTTTGGAATA

ACCAAGTAATATAGCCAAAGACCTCCCAAGCCGTCACACGCACCCCGGAGGCACGAAGCC

CAAACACGAAAGTAGCTTTATTAACTTACCGACGCCACGAAAGCTAAGAAA

>242_Sample_ASV29

CACCGCGGTTATACGAAAGACCCTAGTTTATAGGCACGGCGTAAAGGGTGGTTAGGGGAT

AAACAAAATAAAGCCAAAGACCTTCTAAGTCGTCATACACTCATGAAGGCACGAAGCCCA

AATACGAAAGTAACTTTACAACTTAGACCCGACTCCACGAAAGCTGGGGGA

>242_Sample_ASV30

CACCGCGGTTATACGAGAGGCCCAAGTTGATAGACATCGGCGTAAAGAGTGGTTAAGGAA

GTCTCAAAACTAAAGCCGAATGCCTTCAAGACTGTTATACGTTTCCGAAGGTAAGAAGCC

CCACTACGAAAGTGGCTTTACTCCACCTGACTCCACGAAAGCTGTGAAA

>242_Sample_ASV31

CACCGCGGTTATACGAGAGGCCCAAGTTGACAAACTCCGGCGTAAAGCGTGGTTAAGGCA

CACTCAAAAACTAAAGCCGAATATCTTCAGAGCAGTCATACGCATCCGAAGACACGAAGC

CCCACCACGAAAGTGGCTTTATGACACCTGACCCCACGAAAGCTAGGACA

>242_Sample_ASV32

CACCGCGGTTATACGAAAGACCCTAGTTGATAGACCACGGCGTAAAGGGTGGTTAAGGAA

CACAAAAATAAAGCTAAAGATCCTCTAAGCTGTCATACGCTTCCCGAAGACATGAAACCC

AATCACGAAAGTAGCTTTAAACTGTCCTGACACCACGAAAGCCAAGAAA

>243_Sample_ASV1

CACCGCGGTTATACGAAAGACCCTAGTTGATGGGTGCGGCGTAAAGGGTGGTTAGGGACT

ACAAAAAATAGAGCTAAAGATCCTCTAGGTCGTCATACACATCACGAGAAAACGAAGCCC

AAATACGAAAGTAGCTCTAAATATAATCCTGACGCCACGAAAGCCAAGAAA

>243_Sample_ASV2

CACCGCGGTTATACGAAAGACCCTAGTTGATGGGTGCGGCGTAAAGGGTGGTTAGGGACT

ACAAAAAATAGAGCTAAAGATCCTCTAGGTCGTCATACACATCACGAGGAAACGAAGCCC

AAATACGAAAGTAGCTCTAAATATAATCCTGACGCCACGAAAGCCAAGAAA

>243_Sample_ASV3

CACCGCGGTTATACGAGAGACCCTAGTTGATTAAAGCGGCGTAAAGAGTGGTTATGGAAC

TATTCTTTTAAAGCAGAAAACCTCTCAAACTGTTATACGCACCCAGAGGTCTGAATCCCT

CACACGAAAGTGACTTTATTTATGCCTACCAGAACCCACGAAAGCTGGGACA

>243_Sample_ASV4

CACCGCGGTTATACGAAAGACCCTAGTTTATAGGTACGGCGTAAAGGGTGGTTAGGGGAC

AAACAAAATAAAGCCAAAGACCTCCTAAGCCGTCATACGCTCACGGAAGCACGAAGCCCA

AACACGAAAGTAGCTTTACCGAACATGCCCGACTCCACGAAAGCTGGGGAA

>243_Sample_ASV5

CACCGCGGTTATACGAGAGACCCTAGTTGATATACACGGCGTAAAGAGTGGTTTGGGGTA

TATAATAAATAAAGCCAAAGACCTTCTAGGCCGTTGAACGCACCACGAAGGAGCGAAGTC

CCAATACGAAAGTAGCTTTATAAAACCTGACGCCACGAAAGCTAAGAAA

>243_Sample_ASV6

CACCGCGGTTATACGAGAGGCCCAAGTTGACAGACACCGGCGTAAAGAGTGGCTAGGGAA

AAATTACTACTAAAGCCGAACACCTTCAGAACTGTCATACGTTCCCGAAGATAAGAAGCC

CCACCACGAAAGTGGCTTTATATTACCCGACCCCACGAAAGCTAAGAAA

>243_Sample_ASV7

CACCGCGGTTATACGAGAGGCCCAAGTTGACAGACACCGGCGTAAAGAGTGGCTAGGGAA

AAATTACTACTAAAGCCGAACACCTTCAGAACTGTCATACGTTCCCGAAGATAAGAAGCC

CCACCACGAAAGTGGCTTTATATTACCCGACCCCACGAAAGCTGTGAAA

>243_Sample_ASV8

CACCGCGGTTAAACGAGAGACCCTAGTTGATCATCACGGCGTAAAGAGTGGTTAGGGGAT

TATCATAAATAAAGCCAAAGACCTCCCAAGCTGTCGCACGCATTCCGGAGGCGCGAAGCC

CACACACGAAAGTAGCTTTAACTATTGACCCTGATGCCACGAAAGCTAAGAAA

>243_Sample_ASV9

CACCGCGGTTATACGAGAGGCCCAAGTTGATAAGCCCGGCGTAAAGAGTGGTTATGGAGA

ATAAAACACTAAAGCTAAAGACCCCCTAGGCTGTTATACGCACCTGGCGGCTCGAACCAC

CTATACGAAAGTAGCTTTACCCCCCTTCCACCAGAATCCACGACAGCTGGGGCA

>243_Sample_ASV10

CACCGCGGTTATACGAGAGGCCCTAGTTGATAGCCACGGCGCAAAGAGTGGTTAAGGAGC

CCCACTAAATAAAGTCGAACACTTCCTAGGCCGTTATACGTTTTCTAGAAGCACGAAACC

CAATTACACGAAAGCAACTTTATACTAAACAACCTGACCCCACGAAAGCTAAGAAA

>243_Sample_ASV11

CACCGCGGTTATACGAGAGGCCCAAGTTGACAGACATCGGCGTAAAGAGTGGTTAAGGAA

ACCCCATTACTAAAGCCGAACGCCCTCAGAACTGTCATACGTTCCCGAAGGTAAGAAGCC

CCACTACGAAAGTGGCTTTAACCACACCCGACCCCACGACAGCTAAGAAA

>243_Sample_ASV12

CACCGCGGTTATACGAGAGGCCCAAGTTGACAGACATCGGCGTAAAGAGTGGTTAAGGAA

ACCCCATTACTAAAGCCGAACGCCCTCAGAACTGTCATACGTTCCCGAAGGTAAGAAGCC

CCACTACGAAAGTGGCTTTAACCACACCCGACCCCACGAAAGCTGTGAAA

>243_Sample_ASV13

CACCGCGGTTATACGAGAGACCCCAGTTGATAAACACGGCGTAAAGAGTGGTTAAGGAAA

CCCCATTACTAAAGCCGAACGCCCTCAGAACTGTCATACGTTCCCGAAGGTAAGAAGCCC

CACTACGAAAGTGGCTTTAACCACACCCGACCCCACGAAAGCTGTGAAA

>243_Sample_ASV14

CACCGCGGTCACACGATTAACCCAAGTCAATAGAAGCCGGCGTAAAGAGTGTTTTAGATC

ACCCCCTCCCCAATAAAGCTAAAACTCACCTGAGTTGTAAAAAACTCCAGTTGACACAAA

ATAGACTACGAAAGTGGCTTTAACATATCTGAACACACAATAGCTAAGACC

>243_Sample_ASV15

CACCGCGGTTATACGAGAAACCCTAGTTGACAAATACGGCGTAAAGAGTGGTTAAGGGGT

ATCAAAAATAAAGTCAAACATTTTCCTAGTTGTTATAAGCATTTCGAGAACATGAAGCCC

AACCACGAAAGTAACTTTACCTCCACCTGACCCCACGAAAGCTAAGAAA

>243_Sample_ASV16

CACCGCGGTTATACGAAAGACCCCAGTTTATAGACACGGCGTAAAGGGTGGTTATAGGAC

AAACTAACATAGAGCCAAAGACCCCCTAAGCCGTCATACGCTCACGAGGGCTCGAAGCCC

CTATACGAAAGTAGCTCTACCAAAAAATGCTCGACTCCACGAAAGCTGGGACA

>243_Sample_ASV17

CACCGCGGTTATACGAGAGACCCTAGTTGATAAATACGGCGTAAAGAGTGGTTATGGGAA

AAACAAATAAAGTCAAACAACCTCTTAGCTGTTATACGCATTATGAGAGCATGAAGCCCC

CTCACGAAAGTAACTTTAATACCTCCTGACCCCACGAAAGCTAAGAAA

>243_Sample_ASV18

AACCGCGGTTATACGAGAGACCCTAATTGATAGCTCACGGCGTAAAGGGTGGTTTGGGGA

AACCTCAAAATAAAGCTAAAGACCTTCTAAGCCGTTACACGCATACCGAAGGCACGAGGC

CCTAACACGAAAGTAGCTTTACTATTACCCCTGACGCCACGAAAGCTAAGAAA

>243_Sample_ASV19

CACCGCGGTTATACGAAAGACCCTAGTTGATGGATCACGGCGTAAAGGGTGGTTAAGGGA

TATAAAAATAAAGCTAAAAATCCTCTTAGCTGTCATACGCTTTCCGAAGACATGAGGCCC

AATTACGAAAGTAGCTTTAAACCACCCTGACACCACGAAAGCCAAGAAA

>243_Sample_ASV20

CACCGCGGTTATACGAGAGACCCTAGTTGATAGCTACGGCGTAAAGCGTGGTTAGGAGAA

CAAGATAAATAAAGTCAAACAATCTCTCGGCCGTTATACGTTATCTGAGAATATGAAGTC

CTACCACGAAAGTAACTTTAATTTTTCTGACCCCACGAAAACTGAGGAA

>243_Sample_ASV21

CACCGCGGTTATACGAGAGGCCCTAGTTGATAACTACCGGCGTAAAGAGTGGTTATGGAA

AAATATTTAATAAAGCCGAACACCCCCTCAGCCGTCATACGCACCTGGGAGCACGAAGAC

CTACTGCGAAAGCAGCTTTAATTATGCCTGACCCCACGACAGCTAAGAAA

>243_Sample_ASV22

CACCGCGGTTATACGAGAGGCCCAAGTTGATTATTACCGGCGTAAAGCGTGGTTAAGATT

TTTATAGGACTAAAGCCGAACCCCCTCAGAGCTGTTATACGCACCCGAAGGTTAGAAGTT

CAATCACGAAAGTGGCTTTACAACCCCGAACCCACGAAAGCTAAGATA

>243_Sample_ASV23

CACCGCGGTTATACGAGAGACCCTAATTGATAGGCGCGGCGTAAAGAGTGGTTTAGGACT

AAAACTAATAAAGCTAAAGATCCTCCCAGCCGTCGCACGCATTCTGAGGACACGAAACTC

AACCACGAAAGTAGCTTTAACATACTCTCCTGACCCCACGAAAGCTAAGAAA

>243_Sample_ASV24

CACCGCGGTTATACGAAAGACCCTAGTTGATAGGCACGGCGTAAAGGGTGGTTAGGGATA

TATCAAAAATAAAGTTAAAGAGCCTCTAAGCTGTCGCACGCATCCCGAGAGCTCGAGACC

CAGACACGAAAGTAACTTTAAAATACCTCACCTGACCCCACGAAAGCTAAGAAA

>243_Sample_ASV25

CACCGCGGTTATACGAGAGGCCCAAGTTGATACTCCACGGCGTAAAGAGTGGTTAGAAAA

GACCTACTACTAAAGCCGAACGCCTTCAAAGCTGTTATACGCATCCGAAGGTGAGAAGCC

CACCCACGAAAGTGGCTTTACAACCTTGACTCCACGAAAGCTATGACA

>243_Sample_ASV26

CACCGCGGTTATACGAGAGACCCCAGTTGATAAACACGGCGTAAAGAGTGGTTAAGGGCA

CAAAACAAATAAAGTCAAACAACCTCTTAGCTGTTATACGCATTATGAGGATATGAAGCC

CCCCTACGAAAGTAACTTTACCAACCCCTGACCCCACGAAAGCTAAGAAA

>243_Sample_ASV27

CACCGCGGTTATACGAAAGACCCTAGTTTATAGGCACGGCGTAAAGGGTGGTTAGGGGAT

AAACAAAATAAAGCCAAAGACCTTCTAAGTCGTCATACACTCATGAAGGCACGAAGCCCA

AATACGAAAGTAACTTTACAACTTAGACCCGACTCCACGAAAGCTGGGGGA

>243_Sample_ASV28

CACCGCGGTTATACGAGAGACCCTAATTAATAGATACGGCGTAAAGAGTGGTTTAGGGTA

AAAATTAATAAAGCCGAAGTTTCTCCTAGCCGTTGTACGCACTCTGAGAACACGAAGCCC

CACTACGAAAGTAGCTTTACCTATTATTTCCTGACCCCACGAAAGCTAAGAAA

>243_Sample_ASV29

CACCGCGGTTATACGAGAGACCCTAATTAATAGCTACGGCGTAAAGAGTGGTTTAGGACA

AAAATTAATAAAGCCGAAGTTTCTCCTAGCCGTTGTACGCACTCTGAGAACACGAAGCCC

CACTACGAAAGTAGCTTTACCAATAATCTCCTGACCCCACGAAAGCTAAGAAA

>243_Sample_ASV30

CACCGCGGTTATACGAGAGACCCTAATTAATAGATACGGCGTAAAGAGTGGTTTAGGACA

AAAATTAATAAAGCCGAAGTTTCTCCTAGCCGTTGTACGCACTCTGAGAACACGAAGCCC

CACTACGAAAGTAGCTTTACCAATAATCTCCTGACCCCACGAAAGCTAAGAAA

>243_Sample_ASV31

CACCGCGGTTATACGAGAGACCCTAATTAATAGATACGGCGTAAAGAGTGGTTTAGGACA

AAAATTAATAAAGCCGAAGTTTCTCCTAGCCGTTGTACGCACTCTGAGAACACGAAGCCC

CACTACGAAAGTAGCTTTACCTATTATTTCCTGACCCCACGAAAGCTAAGAAA

>243_Sample_ASV32

CACCGCGGTTATACGAGAGACCCTAATTAATAGCTACGGCGTAAAGAGTGGTTTAGGGTA

AAAATTAATAAAGCCGAAGTTTCTCCTAGCCGTTGTACGCACTCTGAGAACACGAAGCCC

CACTACGAAAGTAGCTTTACCTATTATTTCCTGACCCCACGAAAGCTAAGAAA

>243_Sample_ASV33

CACCGCGGTTATACGAGAGACCCTAATTAATAGCTACGGCGTAAAGAGTGGTTTAGGACA

AAAATTAATAAAGCCGAAGTTTCTCCTAGCCGTTGTACGCACTCTGAGAACACGAAGCCC

CACTACGAAAGTAGCTTTACCTATTATTTCCTGACCCCACGAAAGCTAAGAAA

>243_Sample_ASV34

CACCGCGGTTATACGAGAGGCCCAAGTTGATAGACATCGGCGTAAAGAGTGGTTAAGGAA

GTCTCAAAACTAAAGCCGAATGCCTTCAAGACTGTTATACGTTTCCGAAGGTAAGAAGCC

CCACTACGAAAGTGGCTTTACTCCACCTGACTCCACGAAAGCTGTGAAA

>243_Sample_ASV35

CACCGCGGTTATACGAAAGACCCTAGTTGATAGCTACGGCGTAAAGGGTGGTTAAGGTAT

ATAACAAATAAAGCTAAAGAGCCTCCAAGCCGTCGTACGCATTCTGAGAGCTCGAAACCC

TAACACGAAAGTAGCTTTAAAAAATAACACCTGACCCCACGAAAGCTAAGAAA

>243_Sample_ASV36

CACCGCGGTTATACGAAAGACCCTAGTTGATAGATCACGGCGTAAAGGGTGGTTAAGGAA

AACAAAAATAAAGCTAAAAATCCTCTAAGCTGTCATACGCTTTCCGAAGACATGAGATCC

AACCACGAAAGTAGCTTTAAAATTTCCTGACACCACGAAAGCCAAGGAA

>243_Sample_ASV37

CACCGCGGTTATACGAAAGACCCTAGTTGATAGATCACGGCGTAAAGGGTGGTTAAGGAG

AACAAAAATAAAGCTAAAAATCCTCTAAGCTGTCATACGCTTTCCGAAGACATGAGACCC

AACCACGAAAGTAACTTTAAACTTTCCTGACACCACGAAAGCCAAGAAA

>245_Sample_ASV1

TACCGCGGTTATACGAAAGACCCAAGTTAATAAACTACGGCGTAAAGCGTGGTTAGAGAC

CTCCAATAATTAAAGCCAAAGACCTTCTAAGCCGTCGCACGCCCCGCGAAGACACGAAGC

CCAAAAACGAAAGTAGCTTTATTCTTACTCGACCCCACGAAAGCTAGGGCA

>245_Sample_ASV2

CACCGCGGTTATACGAAAGACCCTAGTTGATGGGTGCGGCGTAAAGGGTGGTTAGGGACT

ACAAAAAATAGAGCTAAAGATCCTCTAGGTCGTCATACACATCACGAGGAAACGAAGCCC

AAATACGAAAGTAGCTCTAAATATAATCCTGACGCCACGAAAGCCAAGAAA

>245_Sample_ASV3

CACCGCGGTTATACGAGAGACCCTAGTTGATTAAAGCGGCGTAAAGAGTGGTTATGGAAC

TATTCTTTTAAAGCAGAAAACCTCTCAAACTGTTATACGCACCCAGAGGTCTGAATCCCT

CACACGAAAGTGACTTTATTTATGCCTACCAGAACCCACGAAAGCTGGGACA

>245_Sample_ASV4

CACCGCGGTTATACGAAAGACCCTAGTTTATAGGTACGGCGTAAAGGGTGGTTAGGGGAC

AAACAAAATAAAGCCAAAGACCTCCTAAGCCGTCATACGCTCACGGAAGCACGAAGCCCA

AACACGAAAGTAGCTTTACCGAACATGCCCGACTCCACGAAAGCTGGGGAA

>245_Sample_ASV5

CACCGCGGTTATACGAGAGGCCCAAGTTGACAGACACCGGCGTAAAGAGTGGCTAGGGAA

AAATTACTACTAAAGCCGAACACCTTCAGAACTGTCATACGTTCCCGAAGATAAGAAGCC

CCACCACGAAAGTGACTTTATATTACCCGACCCCACGAAAGCTGTGAAA

>245_Sample_ASV6

CATGGCGATTATACGAGAGGCCCAAGTTGACAGACACTGGCGTAAAGAGTGGCTAGGGAA

AAATTACTACTAAAGCCGAACACCTTCAGAACTGTCATACGTTCCCGAAGATAAGAAGCC

CCACCACGAAAGTGGCTTTATATTACCCGACCCCACGAAAGCTGTGAAA

>245_Sample_ASV7

CACCGCGGTTATACGAGAGGCCCAAGTTGACAGACACCGGCGTAAAGAGTGGCTAGGGAA

AAATTACTACTAAAGCCGAACACCTTCAGAACTGTCATACGTTCCCGAAGATAAGAAGCC

CCACCACGAAAGTGGCTTTATATTACCCGACCCCACGAAAGCTGTGAAA

>245_Sample_ASV8

CACCGCGGTTATACGAGAGGCCCAAGTTGACAGACACCGGCGTAAAGAGTGGCTAGGGAA

AAATTACTACTAAAGCCGAACACCTTCAGAACTGTCATACGTTCCCGAAGATAAGAAGCC

CCACCACGAAAGTGGCTTTATATTACCCGACCCCACGAAAGCTAAGAAA

>245_Sample_ASV9

CACCGCGGTTATACGAGAGGCCCAAGTTGACAGACACCGGCGTAAAGAGTGGCTAAGGAA

AAATTTCTACTAAAGCCGAACACCTTCAGAACTGTCATACGTTCCCGAAGAAAAGAAGCC

CCACCACGAAAGTGGCTTTATATTACCCGACCCCACGAAAGCTGTGAAA

>245_Sample_ASV10

CACCGCGGTTATACGAAAGACTCTAGTTGACAAACACGGCGTAAAGGGTGGTTAGGATAA

CAACAAAATAAAGCTAAAGACTTACCAAGCCGTCATACGCCCATGTAAGAGCGAAAACCA

CAAACGAAAGTAACTTTATTACACCATCCGAATCCACGAAAGCTAAGAAA

>245_Sample_ASV11

CACCGCGGTTAAACGAGAGACCCTAGTTGATCATCACGGCGTAAAGAGTGGTTAGGGGAT

TATCATAAATAAAGCCAAAGACCTCCCAAGCTGTCGCACGCATTCCGGAGGCGCGAAGCC

CACACACGAAAGTAGCTTTAACTATTGACCCTGATGCCACGAAAGCTAAGAAA

>245_Sample_ASV12

CACCGCGGTTATACGAAAGACCCTAGTTGATAGATCACGGCGTAAAGGGTGGTTAGGGAA

TACAAAAATAAAGCTAAAAATCCTCTAAGCTGTCATACGCTTCCCGAAGACATGAAACCC

AACCACGAAAGTAGCTTTAAACTCTCCTGACACCACGAAAGCCAAGGAA

>245_Sample_ASV13

CACCGCGGTTATACGAGAGGCCCAAGTTGATAAGCCCGGCGTAAAGAGTGGTTATGGAGA

ATAAAACACTAAAGCTAAAGACCCCCTAGGCTGTTATACGCACCTGGCGGCTCGAACCAC

CTATACGAAAGTAGCTTTACCCCCCTTCCACCAGAATCCACGACAGCTGGGGCA

>245_Sample_ASV14

CACCGCGGTTATACGAGAGGCCCAAGTTGACAGACATCGGCGTAAAGAGTGGTTAAGGAA

ACCCCATTACTAAAGCCGAACGCCCTCAGAACTGTCATACGTTCCCGAAGGTAAGAAGCC

CCACTACGAAAGTGGCTTTAACCACACCCGACCCCACGAAAGCTGTGAAA

>245_Sample_ASV15

CACCGCGGTTATACGAGAGGCCCAAGTTGATAGATATCGGCGTAAAGAGTGGTTAAGGAA

ACCCCGTTACTAAAGCCGAACGCCCTCAGAACTGTCATACGTTCCCGAAGGTAAGAAGCC

CCACTACGAAAGTGGCTTTAACCACACCCGACCCCACGAAAGCTGTGAAA

>245_Sample_ASV16

CACCGCGGTTATACGAGAGGCCCCAGTTGATATTAGCGGCGTAAAGGGTGGTTAAGGACT

TTAATAACTAAAGCCAAACACTTCCCCGGCTGTTGTACGTAGACCGGAAGCTTGAAGCCC

AGACACGAAAGTAGCTTTATTTCCCCTGACCCCACGAAAGCTGAGAAA

>245_Sample_ASV17

CACCGCGGTTATACGAGAGGCCCCAGTTGATATTAGCGGCGTAAAGGGTGGTTAAGGACT

TTAATAACTAAAGCTAAACACTTCCCCGGCTGTTGTACGTAGACCGGAAGCTTGAAGCCC

AGACACGAAAGTAGCTTTATTTCCCCTGACCCCACGAAAGCTGAGAAA

>245_Sample_ASV18

CACCGCGGTTATACGAGAGGCCCCAGTTGATATTAGCGGCGTAAAGGGTGGTTAAGGACT

TTAATAACTAAAGCTAAACACTTCCCCGGCTGTTGTACGTAGACCGGAAGCTTGAAGCCC

AGACACGAAAGTAGCTTTATTCCCCCTGACCCCACGAAAGCTGAGAAA

>245_Sample_ASV19

CACCGCGGTTATACGAAAGACCCCAGTTTATAGACACGGCGTAAAGGGTGGTTATAGGAC

AAACTAACATAGAGCCAAAGACCCCCTAAGCCGTCATACGCTCACGAGGGCTCGAAGCCC

CTATACGAAAGTAGCTCTACCAAAAAATGCTCGACTCCACGAAAGCTGGGACA

>245_Sample_ASV20

CACCGCGGTTATACGAGAGACCCTAGTTGATAAATACGGCGTAAAGAGTGGTTATGGGAA

AAACAAATAAAGTCAAACAACCTCTTAGCTGTTATACGCATTATGAGAGCATGAAGCCCC

CTCACGAAAGTAACTTTAATACCTCCTGACCCCACGAAAGCTAAGAAA

>245_Sample_ASV21

CACCGCGGTTATACGAGAGGCTCAAGTTGATAAACCCCGGCGTAAAGAGTGGTTAAGGAA

AAATAGAAACTAAAGCCGAATGCTTTCAAGACTGTTATACGTTCCCGAGAGTAAGAAGAC

CAATTACGAAAGTGGCTTTACTTTACCTGACCCCACGAAAGCTACGACA

>245_Sample_ASV22

CACCGCGGTTATACGAGAGACCCTAATTGATAGGCACGGCGTAAAGAGTGGTTTAGGGTA

ACCCATAAATAAAGCTAAAGACCTTCTAAGCCGTCACACGCACACCGAAGGCACGAGACC

CCAACACGAAAGTAGCTTTACTTCTACCCCTGACGCCACGAAAGCTAAGAAA

>245_Sample_ASV23

CACCGCGGTTATACGAAAGACCCTAGTTGATGGATCACGGCGTAAAGGGTGGTTAAGGGA

TATAAAAATAAAGCTAAAAATCCTCTTAGCTGTCATACGCTTTCCGAAGACATGAGGCCC

AATTACGAAAGTAGCTTTAAACCACCCTGACACCACGAAAGCCAAGAAA

>245_Sample_ASV24

CACCGCGGTTATACGAAAGACCCCAGTTTATAGATGCGGCGTAAAGGGTGGTTAAGAGAA

AATCAAAATAAGGCCAAAGATCCCCTAAGCAGTCATACGCTCCACGAGGACACGAAACCC

AAATACGAAAGTTGCCTTAATAAACTTCACCTGATCCCACGAAAGCTGAAAAA

>245_Sample_ASV25

CACCGCGGTTATACGAAAGGCTCTAGTTTATAAATACGGCGTAAAGGGTGGTTAAGGAAA

AATTAAAATAATGCTAAAGACCCCCTAAGCAGTTATACGCTTCACGGAGGCACGAAACCC

AAATACGAAAGTTGCCTTAATAAACTTTACCTGACCCCACGAAAGCTGAAAAA

>245_Sample_ASV26

CACCGCGGTTATACGAGAGACTCTAGTTGATACCAACGGCGTAAAGAGTGGTTTGGGATG

CCCCTTAAAATAAAGCCAAAGACCTCCCAAGCCGTCAAACGCACCCCGGAGGCACGAAGC

CCCAACGCGAAAGCAACTTTACCTCCCTCCCGACGCCACGAAAGCTAAGAAA

>245_Sample_ASV27

CACCGCGGTTATACGAGAGACCCTAGTTGATAGCTACGGCGTAAAGCGTGGTTAGGAGAA

CAAGATAAATAAAGTCAAACAATCTCTCGGCCGTTATACGTTATCTGAGAATATGAAGTC

CTACCACGAAAGTAACTTTAATTTTTCTGACCCCACGAAAACTGAGGAA

>245_Sample_ASV28

CACCGCGGTTATACGAGAGGCCCTAGTTGATAACTACCGGCGTAAAGAGTGGTTATGGAA

AAATATTTAATAAAGCCGAACACCCCCTCAGCCGTCATACGCACCTGGGAGCACGAAGAC

CTACTGCGAAAGCAGCTTTAATTATGCCTGACCCCACGACAGCTAAGAAA

>245_Sample_ASV29

CACCGCGGTTATACGAGAGGCCCAAGTTGATTATTACCGGCGTAAAGCGTGGTTAAGATT

TTTATAGGACTAAAGCCGAACCCCCTCAGAGCTGTTATACGCACCCGAAGGTTAGAAGTT

CAATCACGAAAGTGGCTTTACAACCCCGAACCCACGAAAGCTAAGATA

>245_Sample_ASV30

CACCGCGGTTATACGAGAGGCCCAAGTTGATACTCCACGGCGTAAAGAGTGGTTAGAAAA

GACCTACTACTAAAGCCGAACGCCTTCAAAGCTGTTATACGCATCCGAAGGTGAGAAGCC

CACCCACGAAAGTGGCTTTACAACCTTGACTCCACGAAAGCTATGACA

>245_Sample_ASV31

CACCGCGGTTATACGAGAGACCCCAGTTGATAAACACGGCGTAAAGAGTGGTTAAGGGCA

CAAAACAAATAAAGTCAAACAACCTCTTAGCTGTTATACGCATTATGAGGATATGAAGCC

CCCCTACGAAAGTAACTTTACCAACCCCTGACCCCACGAAAGCTAAGAAA

>245_Sample_ASV32

CACCGCGGTTATACGAAAGACCCTAGTTTATAGGCACGGCGTAAAGGGTGGTTAGGGGAT

AAACAAAATAAAGCCAAAGACCTTCTAAGTCGTCATACACTCATGAAGGCACGAAGCCCA

AATACGAAAGTAACTTTACAACTTAGACCCGACTCCACGAAAGCTGGGGGA

>245_Sample_ASV33

CACCGCGGTTATACGAGAGACCCTAATTAATAGATACGGCGTAAAGAGTGGTTTAGGACA

AAAATTAATAAAGCCGAAGTTTCTCCTAGCCGTTGTACGCACTCTGAGAACACGAAGCCC

CACTACGAAAGTAGCTTTACCTATTATTTCCTGACCCCACGAAAGCTAAGAAA

>245_Sample_ASV34

CACCGCGGTTATACGAGAGGCCCAAGTTGATAGACATCGGCGTAAAGAGTGGTTAAGGAA

GTCTCAAAACTAAAGCCGAATGCCTTCAAGACTGTTATACGTTTCCGAAGGTAAGAAGCC

CCACTACGAAAGTGGCTTTACTCCACCTGACTCCACGAAAGCTGTGAAA

>245_Sample_ASV35

CACCGCGGTTATACGAAAGACCCTAGTTGATAGCTACGGCGTAAAGGGTGGTTAAGGTAT

ATAACAAATAAAGCTAAAGAGCCTCCAAGCCGTCGTACGCATTCTGAGAGCTCGAAACCC

TAACACGAAAGTAGCTTTAAAAAATAACACCTGACCCCACGAAAGCTAAGAAA

>245_Sample_ASV36

CACCGCGGTTATACGAAAGACCCTAGTTGATAGATCACGGCGTAAAGGGTGGTTAAGGAG

AACAAAAATAAAGCTAAAAATCTTCTAAGCTGTCATACGCTTTCCGAAGACATGAGACCC

AACCACGAAAGTAGCTTTAAACTTTCCTGACACCACGAAAGCCAAGAAA

>250_Sample_ASV1

CACCGCGGTTATACGAGAGGCCCAAGTTGACAGACATCGGCGTAAAGAGTGGTTAAGGAA

AACCTTCAACTAAAGTCGAACGCTCTCAAGACTGTCGTACGTTTCCGAGAGTATGAAGCC

CCACCACGAAAGTGACTTTACCTCACCTGACTCCACGAAAGCTGCGAAA

>250_Sample_ASV2

CACCACGGTTATACGAGAGGCCCAAGTTGAAAGACATCGGCGTAAAGAGTGGTTAAGGAA

AACCTTCTACTAAAGTCGAACGCTCTCAGGACTGTCGTACGTTTCCGAGAGTATGAAGCC

CCACCACGAAAGTGACTTTACCTCACCTGACTCCACGAAAGCTGCGAAA

>250_Sample_ASV3

CACCGCGGTTATACGAAAGACCCTAGTTGATGGGTGCGGCGTAAAGGGTGGTTAGGGACT

ACAAAAAATAGAGCTAAAGATCCTCTAGGTCGTCATACACATCACGAGGAAACGAAGCCC

AAATACGAAAGTAGCTCTAAATATAATCCTGACGCCACGAAAGCCAAGAAA

>250_Sample_ASV4

CACCGCGGTTATACGAGAGACCCTAGTTGATTAAAGCGGCGTAAAGAGTGGTTATGGAAC

TATTCTTTTAAAGCAGAAAACCTCTCAAACTGTTATACGCACCCAGAGGTCTGAATCCCT

CACACGAAAGTGACTTTATTTATGCCTACCAGAACCCACGAAAGCTGGGACA

>250_Sample_ASV5

CACCGCGGTTATACGAAAGACCCTAGTTTATAGGTACGGCGTAAAGGGTGGTTAGGGGAC

AAACAAAATAAAGCCAAAGACCTCCTAAGCCGTCATACGCTCACGGAAGCACGAAGCCCA

AACACGAAAGTAGCTTTACCGAACATGCCCGACTCCACGAAAGCTGGGGAA

>250_Sample_ASV6

CACCGCGGTTATACGAAAGACCCTAGTTGATAGCCACGGCGTAAAGGGTGGTTAAGGAAC

CTAAAAAATAAAGCTAAAGACCCTCTAGGCCGTCATACGCATTACGAGGCAAACGAAACC

CCAACACGAAAGTAGCTTTAGACAAATATCCTGATGCCACGAAAGCCAAGAAA

>250_Sample_ASV7

CACCGCGGTTATACGAGAGGCCCAAGTTGACAGACACCGGCGTAAAGAGTGGCTAGGGAA

AAATTACTACTAAAGCCGAACACCTTCAGAACTGTCATACGTTCCCGAAGATAAGAAGCC

CCACCACGAAAGTGGCTTTATATTACCCGACCCCACGAAAGCTGTGAAA

>250_Sample_ASV8

CACCGCGGTTATACGAGAGGCCCAAGTTGACAGACACCGGCGTAAAGAGTGGCTAGGGAA

AAATTACTACTAAAGCCGAACACCTTCAGAACTGTCATACGTTCCCGAAGATAAGAAGCC

CCACCACGAAAGTGGCTTTATATTACCCGACCCCACGAAAGCCAAGAAA

>250_Sample_ASV9

CACCGCGGTTATACGAAAGACCCTAGTTGACAGACACCGGCGTAAAGAGTGGCTAGGGAA

AAATTACTACTAAAGCCGAACACCTTCAGAACTGTCATACGTTCCCGAAGATAAGAAGCC

CCACCACGAAAGTGGCTTTATATTACCCGACCCCACGAAAGCCAAGAAA

>250_Sample_ASV10

CACCGCGGTTATACGAGAGGCCCAAGTTGACAGACACCGGCGTAAAGAGTGGCTAAGGAA

AAATTTCTACTAAAGCCGAACACCTTCAGAACTGTCATACGTTCCCGAAGAAAAGAAGCC

CCACCACGAAAGTGGCTTTATATTACCCGACCCCACGAAAGCCAAGAAA

>250_Sample_ASV11

CACCGCGGTTATACGAAAGACTCTAGTTGACAAACACGGCGTAAAGGGTGGTTAGGATAA

CAACAAAATAAAGCTAAAGACTTACCAAGCCGTCATACGCCCATGTAAGAGCGAAAACCA

CAAACGAAAGTAACTTTATTACACCATCCGAATCCACGAAAGCTAAGAAA

>250_Sample_ASV12

CACCGCGGTTATACGAGAGGCCCAAGTTGATAAGCCCGGCGTAAAGAGTGGTTATGGAGA

ATAAAACACTAAAGCTAAAGACCCCCTAGGCTGTTATACGCACCTGGCGGCTCGAACCAC

CTATACGAAAGTAGCTTTACCCCCCTTCCACCAGAATCCACGACAGCTGGGGCA

>250_Sample_ASV13

CACCGCGGTTATACGAGAGGCCCTAGTTGATAGCCACGGCGCAAAGAGTGGTTAAGGAGC

CCCACTAAATAAAGTCGAACACTTCCTAGGCCGTTATACGTTTTCTAGAAGCACGAAACC

CAATTACACGAAAGCAACTTTATACTAAACAACCTGACCCCACGAAAGCTAAGAAA

>250_Sample_ASV14

CACCGCGGTTATACGAGAGGCCCAAGTTGACAGACATCGGCGTAAAGAGTGGTTAAGGAA

ACCCCATTACTAAAGCCGAACGCCCTCAGAACTGTCATACGTTCCCGAAGGTAAGAAGCC

CCACTACGAAAGTGGCTTTAACCACACCCGACCCCACGAAAGCTGTGAAA

>250_Sample_ASV15

CACCGCGGTTATACGAGAGGCCCAAGTTGATAGATATCGGCGTAAAGAGTGGTTAAGGAA

ACCCCGTTACTAAAGCCGAACGCCCTCAGAACTGTCATACGTTCCCGAAGGTAAGAAGCC

CCACTACGAAAGTGGCTTTAACCACACCCGACCCCACGAAAGCTGTGAAA

>250_Sample_ASV16

CACCGCGGTCACACGATTAACCCAAGTCAATAGAAGCCGGCGTAAAGAGTGTTTTAGATC

ACCCCCTCCCCAATAAAGCTAAAACTCACCTGAGTTGTAAAAAACTCCAGTTGACACAAA

ATAGACTACGAAAGTGGCTTTAACATATCTGAACACACAATAGCTAAGACC

>250_Sample_ASV17

CACCGCGGTTATACGAGAAACCCTAGTTGACAAATACGGCGTAAAGAGTGGTTAAGGGGT

ATCAAAAATAAAGTCAAACATTTTCCTAGTTGTTATAAGCATTTCGAGAACATGAAGCCC

AACCACGAAAGTAACTTTACCTTCACCTGACCCCACGAAAGCTAAGAAA

>250_Sample_ASV18

CACCGCGGTTATACGAAAGACCCCAGTTTATAGACACGGCGTAAAGGGTGGTTATAGGAC

AAACTAACATAGAGCCAAAGACCCCCTAAGCCGTCATACGCTCACGAGGGCTCGAAGCCC

CTATACGAAAGTAGCTCTACCAAAAAATGCTCGACTCCACGAAAGCTGGGACA

>250_Sample_ASV19

CACCGCGGTTATACGAGAGACCCTAGTTGATAAATACGGCGTAAAGAGTGGTTATGGGAA

AAACAAATAAAGTCAAACAACCTCTTAGCTGTTATACGCATTATGAGAGCATGAAGCCCC

CTCACGAAAGTAACTTTAATACCTCCTGACCCCACGAAAGCTAAGAAA

>250_Sample_ASV20

AACCGCGGTTATACGAGAGACCCTAATTGATAGCTCACGGCGTAAAGGGTGGTTTGGGGA

AACCTCAAAATAAAGCTAAAGACCTTCTAAGCCGTTACACGCATACCGAAGGCACGAGGC

CCTAACACGAAAGTAGCTTTACTATTACCCCTGACGCCACGAAAGCTAAGAAA

>250_Sample_ASV21

CACCGCGGTTATACGAAAGACCCTAGTTGATGGATCACGGCGTAAAGGGTGGTTAAGGGA

TATAAAAATAAAGCTAAAAATCCTCTTAGCTGTCATACGCTTTCCGAAGACATGAGGCCC

AATTACGAAAGTAGCTTTAAACCACCCTGACACCACGAAAGCCAAGAAA

>250_Sample_ASV22

CACCGCGGTTATACGAAAGACCCAAACTGATAGATGCCGGCGTAAAGAGTGGTTAAGTAT

CTTGATAGAACTAAAGCCGAACGCCCTCAAGACCGTCATACGTTTCCGAAGGTATGAAGC

CCAACTACGAAAGTAACTTTAACTGTATCCGACTCCACGAAAGCTGTGAAA

>250_Sample_ASV23

CACCGCGGTTATACGAGAGGCCCAAGTTGATAGACGCCGGCGCAAAGAGTGGTTAGGTAA

CCTGACAAACTAAAGCCGAACGCCCCCAAGACTGTTATACGTGCTCGGAGGTATGAAGCC

CAACTACGAAAGTGGCTTTAACTATGTCTGAACCCACGAAAGCTGTGAAA

>250_Sample_ASV24

CACCGCGGTTATACGAGAGGCCCTAGTTGATAACTACCGGCGTAAAGAGTGGTTATGGAA

AAATATTTAATAAAGCCGAACACCCCCTCAGCCGTCATACGCACCTGGGAGCACGAAGAC

CTACTGCGAAAGCAGCTTTAATTATGCCTGACCCCACGACAGCTAAGAAA

>250_Sample_ASV25

CACCGCGGTTATACGAGAGGCCCAAGTTGATTATTACCGGCGTAAAGCGTGGTTAAGATT

TTTATAGGACTAAAGCCGAACCCCCTCAGAGCTGTTATACGCACCCGAAGGTTAGAAGTT

CAATCACGAAAGTGGCTTTACAACCCCGAACCCACGAAAGCTAAGATA

>250_Sample_ASV26

CACCGCGGTTATACGAGAGACCCTAATTGATAGGCGCGGCGTAAAGAGTGGTTTAGGACT

AAAACTAATAAAGCTAAAGATCCTCCCAGCCGTCGCACGCATTCTGAGGACACGAAACTC

AACCACGAAAGTAGCTTTAACATACTCTCCTGACCCCACGAAAGCTAAGAAA

>250_Sample_ASV27

CACCGCGGTTATACGAAAGACCCTAGTTGATAGGCACGGCGTAAAGGGTGGTTAGGGATA

TATCAAAAATAAAGTTAAAGAGCCTCTAAGCTGTCGCACGCATCCCGAGAGCTCGAGACC

CAGACACGAAAGTAACTTTAAAATACCTCACCTGACCCCACGAAAGCTAAGAAA

>250_Sample_ASV28

CACCGCGGTTATACGAGAGGCCCAAGTTGATACTCCACGGCGTAAAGAGTGGTTAGAAAA

GACCTACTACTAAAGCCGAACGCCTTCAAAGCTGTTATACGCATCCGAAGGTGAGAAGCC

CACCCACGAAAGTGGCTTTACAACCTTGACTCCACGAAAGCTATGACA

>250_Sample_ASV29

CACCGCGGTTATACGAGAGACCCCAGTTGATAAACACGGCGTAAAGAGTGGTTAAGGGCA

CAAAACAAATAAAGTCAAACAACCTCTTAGCTGTTATACGCATTATGAGGATATGAAGCC

CCCCTACGAAAGTAACTTTACCAACCCCTGACCCCACGAAAGCTAAGAAA

>250_Sample_ASV30

CACCGCGGTTATACGAAAGACCCTAGTTTATAGGCACGGCGTAAAGGGTGGTTAGGGGAT

AAACAAAATAAAGCCAAAGACCTTCTAAGTCGTCATACACTCATGAAGGCACGAAGCCCA

AATACGAAAGTAACTTTACAACTTAGACCCGACTCCACGAAAGCTGGGGGA

>250_Sample_ASV31

CACCGCGGTTATACGAGAGACCCTAATTAATAGCTACGGCGTAAAGAGTGGTTTAGGACA

AAAATTAATAAAGCCGAAGTTTCTCCTAGCCGTTGTACGCACTCTGAGAACACGAAGCCC

CACTACGAAAGTAGCTTTACCTATTATTTCCTGACCCCACGAAAGCTAAGAAA

>250_Sample_ASV32

CACCGCGGTTATACGAGAGACCCTAATTAATAGATACGGCGTAAAGAGTGGTTTAGGACA

AAAATTAATAAAGCCGAAGTTTCTCCTAGCCGTTGTACGCACTCTGAGAACACGAAGCCC

CACTACGAAAGTAGCTTTACCAATAATCTCCTGACCCCACGAAAGCTAAGAAA

>250_Sample_ASV33

CACCGCGGTTATACGAGAGACCCTAATTAATAGCTACGGCGTAAAGAGTGGTTTAGGGTA

AAAATTAATAAAGCCGAAGTTTCTCCTAGCCGTTGTACGCACTCTGAGAACACGAAGCCC

CACTACGAAAGTAGCTTTACCTATTATTTCCTGACCCCACGAAAGCTAAGAAA

>250_Sample_ASV34

CACCGCGGTTATACGAGAGACCCTAATTAATAGATACGGCGTAAAGAGTGGTTTAGGACA

AAAATTAATAAAGCCGAAGTTTCTCCTAGCCGTTGTACGCACTCTGAGAACACGAAGCCC

CACTACGAAAGTAGCTTTACCTATTATTTCCTGACCCCACGAAAGCTAAGAAA

>250_Sample_ASV35

CACCGCGGTTATACGAGAGGCCCAAGTTGATAGACATCGGCGTAAAGAGTGGTTAAGGAA

GTCTCAAAACTAAAGCCGAATGCCTTCAAGACTGTTATACGTTTCCGAAGGTAAGAAGCC

CCACTACGAAAGTGGCTTTACTCCACCTGACTCCACGAAAGCTGTGAAA

>250_Sample_ASV36

CACCGCGGTTATACGAGAGGCCCAAGTTGATAGACATCGGCGTAAAGAGTGGTTAAGGAA

GTCTCAAAACTAAAGCCGAATGCCTTCAAGACTGTTATACGTTTCCGAAGGTAAGAAGCC

CCACTACGAAAGTGGCTTTACTCCACCTGACTCCACGAAAGCCAAGAAA

>250_Sample_ASV37

CACCGCGGTTATACGAGAGGCCCAAGTTGATAGACATCGGCGTAAAGAGTGGTTAAGGAA

GTCTCAAAACTAAAGCCGAATGCCTTCAAGACTGTTATACGTTTCCGAAGGTAAGAAGCC

CCACTACGAAAGTGGCTTTACTCCACCTGACGCCACGAAAGCCAAGAAA

>250_Sample_ASV38

CACCGCGGTTATACGAAAGACCCTAGTTGATAGCTACGGCGTAAAGGGTGGTTAAGGTAT

ATAACAAATAAAGCTAAAGAGCCTCCAAGCCGTCGTACGCATTCTGAGAGCTCGAAACCC

TAACACGAAAGTAGCTTTAAAAAATAACACCTGACCCCACGAAAGCTAAGAAA

>250_Sample_ASV39

CACCGCGGTTATACGAAAGACCCTAGTTGATAGCTACGGCGTAAAGGGTGGTTAAGGTAT

ATAACAAATAAAGCTAAAGAGCCTCCAAGCCGTCGTACGCATTCTGAGAGCTCGAAACCC

TAACACGAAAGTAGCTTTAAAAAATAACACCTGACCCCACGAAAGCCAAGAAA

>250_Sample_ASV40

CACCGCGGTTATACGAAAGACCCTAGTTGATAGATCACGGCGTAAAGGGTGGTTAAGGAA

AACAAAAATAAAGCTAAAAATCCTCTAAGCTGTCATACGCTTTCCGAAGACATGAGATCC

AACCACGAAAGTAGCTTTAAAATTTCCTGACACCACGAAAGCCAAGGAA

>262_Sample_ASV1

CACCGCGGTTATACGAAAGACCCTAGTTGATGGGTGCGGCGTAAAGGGTGGTTAGGGACT

ACAAAAAATAGAGCTAAAGATCCTCTAGGTCGTCATACACATCACGAGGAAACGAAGCCC

AAATACGAAAGTAGCTCTAAATATAATCCTGACGCCACGAAAGCCAAGAAA

>262_Sample_ASV2

CACCGCGGTTATACGAGAGACCCTAGTTGATTAAAGCGGCGTAAAGAGTGGTTATGGAAC

TATTCTTTTAAAGCAGAAAACCTCTCAAACTGTTATACGCACCCAGAGGTCTGAATCCCT

CACACGAAAGTGACTTTATTTATGCCTACCAGAACCCACGAAAGCTGTGAAA

>262_Sample_ASV3

CACCGCGGTTATACGAGAGACCCTAGTTGATTAAAGCGGCGTAAAGAGTGGTTATGGAAC

TATTCTTTTAAAGCAGAAAACCTCTCAAACTGTTATACGCACCCAGAGGTCTGAATCCCT

CACACGAAAGTGACTTTATTTATGCCTACCAGAACCCACGAAAGCTGGGACA

>262_Sample_ASV4

CACCGCGGTTATACGAAAGACCCTAGTTTATAGGTACGGCGTAAAGGGTGGTTAGGGGAC

AAACAAAATAAAGCCAAAGACCTCCTAAGCCGTCATACGCTCACGGAAGCACGAAGCCCA

AACACGAAAGTAGCTTTACCGAACATGCCCGACTCCACGAAAGCTGGGGAA

>262_Sample_ASV5

CACCGCGGTTATACGAGAGACCCTAGTTGATATACACGGCGTAAAGAGTGGTTTGGGGTA

TATAATAAATAAAGCCAAAGACCTTCTAGGCCGTTGAACGCACCACGAAGGAGCGAAGTC

CCAATACGAAAGTAGCTTTATAAAACCTGACGCCACGAAAGCTAAGAAA

>262_Sample_ASV6

CACCGCGGTTATACGAGAGGCCCAAGTTGACAGACACCGGCGTAAAGAGTGGCTAGGGAA

AAATTACTACTAAAGCCGAACACCTTCAGAACTGTCATACGTTCCCGAAGATAAGAAGCC

CCACCACGAAAGTGACTTTATATTACCCGACCCCACGAAAGCTGTGAAA

>262_Sample_ASV7

CACCGCGGTTATACGAGAGGCCCAAGTTGACAGACACCGGCGTAAAGAGTGGCTAGGGAA

AAATTACTACTAAAGCCGAACACCTTCAGAACTGTCATACGTTCCCGAAGATAAGAAGCC

CCACCACGAAAGTGGCTTTATATTACCCGACCCCACGAAAGCTGTGAAA

>262_Sample_ASV8

CACCGCGGTTATACGAGAGGCCCAAGTTGACAGACACCGGCGTAAAGAGTGGCTAAGGAA

AAATTTCTACTAAAGCCGAACACCTTCAGAACTGTCATACGTTCCCGAAGAAAAGAAGCC

CCACCACGAAAGTGGCTTTATATTACCCGACCCCACGAAAGCTGTGAAA

>262_Sample_ASV9

CACCGCGGTTATACGAGAGGCCCAAGTTGATAGACATCGGCGTAAAGAGTGGTTATGGAG

AATAAAACACTAAAGCTAAAGACCCCCTAGGCTGTTATACGCACCTGGCGGCTCGAACCA

CCTATACGAAAGTAGCTTTACCCCCCTTCCACCAGAATCCACGACAGCTGGGGCA

>262_Sample_ASV10

CACCGCGGTTATACGAGAGGCCCAAGTTGATAAGCCCGGCGTAAAGAGTGGTTATGGAGA

ATAAAACACTAAAGCTAAAGACCCCCTAGGCTGTTATACGCACCTGGCGGCTCGAACCAC

CTATACGAAAGTAGCTTTACCCCCCTTCCACCAGAATCCACGAAAGCTGTGAAA

>262_Sample_ASV11

CACCGCGGTTATACGAGAGGCCCAAGTTGATAAGCCCGGCGTAAAGAGTGGTTATGGAGA

ATAAAACACTAAAGCTAAAGACCCCCTAGGCTGTTATACGCACCTGGCGGCTCGAACCAC

CTATACGAAAGTAGCTTTACCCCCCTTCCACCAGAATCCACGACAGCTGTGAAA

>262_Sample_ASV12

CACCGCGGTTATACGAGAGGCCCAAGTTGATAAGCCCGGCGTAAAGAGTGGTTATGGAGA

ATAAAACACTAAAGCTAAAGACCCCCTAGGCTGTTATACGCACCTGGCGGCTCGAACCAC

CTATACGAAAGTAGCTTTACCCCCCTTCCACCAGAATCCACGACAGCTGGGGCA

>262_Sample_ASV13

CACCGCGGTTATACGAGAGGCCCAAGTTGACAGACATCGGCGTAAAGAGTGGTTAAGGAA

ACCCCATTACTAAAGCCGAACGCCCTCAGAACTGTCATACGTTCCCGAAGGTAAGAAGCC

CCACTACGAAAGTGGCTTTAACCACACCCGACCCCACGAAAGCTGTGAAA

>262_Sample_ASV14

CACCGCGGTTATACGAGAGGCCCAAGTTGACAGACACCGGCGTAAAGAGTGGTTAAGGAA

ATTCATCAACTAAAGCCGAATGCCCTCAGGACTGTTATACGTTTGCGAAGGTAAGAAGCC

CTACTACGAAAGTGGCTTTATTCCACCTGATTCCACGAAAGCTGCGGAA

>262_Sample_ASV15

CACCGCGGTTATACGAGAGGCCCCAGTTGATATTAGCGGCGTAAAGGGTGGTTAAGGACT

TTAATAACTAAAGCTAAACACTTCCCCGGCTGTTGTACGTAGACCGGAAGCTTGAAGCCC

AGACACGAAAGTAGCTTTATTTCCCCTGACCCCACGAAAGCTGAGAAA

>262_Sample_ASV16

CACCGCGGTTATACGAGAGGCCCCAGTTGATATTAGCGGCGTAAAGGGTGGTTAAGGACT

TTAATAACTAAAGCTAAACACTTCCCCGGCTGTTGTACGTAGACCGGAAGCTTGAAGCCC

AGACACGAAAGTAGCTTTATTCCCCCTGACCCCACGAAAGCTGAGAAA

>262_Sample_ASV17

CACCGCGGTTATACGAGAGGCCCCAGTTGATATTAGCGGCGTAAAGGGTGGTTAAGGACT

TTAATAACTAAAGCCAAACACTTCCCCGGCTGTTGTACGTAGACCGGAAGCTTGAAGCCC

AGACACGAAAGTAGCTTTATTTCCCCTGACCCCACGAAAGCTGAGAAA

>262_Sample_ASV18

CACCGCGGTCACACGATTAACCCAAGTCAATAGAAGCCGGCGTAAAGAGTGTTTTAGATC

ACCCCCTCCCCAATAAAGCTAAAACTCACCTGAGTTGTAAAAAACTCCAGTTGACACAAA

ATAGACTACGAAAGTGGCTTTAACATATCTGAACACACAATAGCTAAGACC

>262_Sample_ASV19

CACCGCGGTTATACGAGAAACCCTAGTTGACAAATACGGCGTAAAGAGTGGTTAAGGGGT

ATCAAAAATAAAGTCAAACATTTTCCTAGTTGTTATAAGCATTTCGAGAACATGAAGCCC

AACCACGAAAGTAACTTTACCTTCACCTGACCCCACGAAAGCTAAGAAA

>262_Sample_ASV20

CACCGCGGTTATACGAGAGACCCTAGTTGATAAATACGGCGTAAAGAGTGGTTATGGGAA

AAACAAATAAAGTCAAACAACCTCTTAGCTGTTATACGCATTATGAGAGCATGAAGCCCC

CTCACGAAAGTAACTTTAATACCTCCTGACCCCACGAAAGCTAAGAAA

>262_Sample_ASV21

CACCGCGGTTATACGAGAGACCCTAATTGATAGACACGGCGTAAAGAGTGGTTTGGGAAA

GCCCAAAAATAAAGCTAAAGACCTTCTAAGCCGTCACACGCACGCCGAAGGCACGAAGCC

CTGACACGAAAGTAGCTTTACTATTACCCCCGACGCCACGAAAGCTAAGAAA

>262_Sample_ASV22

CACCGCGGTTATACGAGAGACCCTAATTGATAGACACGGCGTAAAGAGTGGTTTGGGAAA

GCCCAAAAATAAAGCTAAAGACCTTCTAAGCCGTCACACGCACGCCGAAGGCACGAAGCC

CTGACACGAAAGTAGCTTTACTACTGCCCCTGACGCCACGAAAGCTAAGAAA

>262_Sample_ASV23

AACCGCGGTTATACGAGAGACCCTAATTGATAGCTCACGGCGTAAAGGGTGGTTTGGGGA

AACCTCAAAATAAAGCTAAAGACCTTCTAAGCCGTTACACGCATACCGAAGGCACGAGGC

CCTAACACGAAAGTAGCTTTACTATTACCCCTGACGCCACGAAAGCTAAGAAA

>262_Sample_ASV24

CACCGCGGTTATACGAGAGACCCTAATTGATAGACACGGCGTAAAGAGTGGTTTAGGACA

ACCCAAAAATAAAGCTAAAGACCTTCTAAGCCGTCACACGCACGCCGAAGGCACGAAGCC

CTGACACGAAAGTAGCTTTACTATTACCCCCGACGCCACGAAAGCTAAGAAA

>262_Sample_ASV25

CACCGCGGTTATACGAAAGACCCTAGTTGATGGATCACGGCGTAAAGGGTGGTTAAGGGA

TATAAAAATAAAGCTAAAAATCCTCTTAGCTGTCATACGCTTTCCGAAGACATGAGGCCC

AATTACGAAAGTAGCTTTAAACCACCCTGACACCACGAAAGCCAAGAAA

>262_Sample_ASV26

CACCGCGGTTATACGAAAGACCCTAGTTTATAGATGCGGCGTAAAGGGTGGTTAAGGAAA

ACCTAAAATAATGCTAAAGACTCTCAAAGCTGTTATACGCTTCATGAGCACACGAAATCC

AAATACGAAAGTTGCCTTAATAAACTTTGCCTGACCCCACGAAAGCTGAAAAA

>262_Sample_ASV27

CACCGCGGTTATACGAAAGGCTCTAGTTTATAAATACGGCGTAAAGGGTGGTTAAGGAAA

AATTAAAATAATGCTAAAGACCCCCTAAGCAGTTATACGCTTCACGGAGGCACGAAACCC

AAATACGAAAGTTGCCTTAATAAACTTTACCTGACCCCACGAAAGCTGAAAAA

>262_Sample_ASV28

CACCGCGGTTATACGAAAGGCTCTAGTTTATAAATACGGCGTAAAGGGTGGTTAAGGAAA

AATTAAAATAATGCTAAAGACCCCCTAAGCAGTTATACGCTTCACGGAGGCACGAAACCC

AAATACGAAAGTTGCCTTAATAAACTTTACCTGACCCCACGAAAGCTGAGAAA

>262_Sample_ASV29

CACCGCGGTTATACGAGTTGACCCCAGTTGATAAATACGGCGTAAAGAGTGGTTATGGGT

AGAACTAAATAAAGTCAAACAATCTTTCAGCTGTTATATGCAATATGAGAGCATGAAGCC

CCCTTACGAAAGTAACTTTAACAACTCCTGACCCCACGAAAGCTAAGAAA

>262_Sample_ASV30

CACCGCGGTTATACGAGAGACCCTAGTTGATAGCTACGGCGTAAAGCGTGGTTAGGAGAA

CAAGATAAATAAAGTCAAACAATCTCTCGGCCGTTATACGTTATCTGAGAATATGAAGTC

CTACCACGAAAGTAACTTTAATTTTTCTGACCCCACGAAAACTGAGGAA

>262_Sample_ASV31

CACCGCGGTTATACGAGAGGCCCAAGTTGATACTCCACGGCGTAAAGAGTGGTTAGAAAA

GACCTACTACTAAAGCCGAACGCCTTCAAAGCTGTTATACGCATCCGAAGGTGAGAAGCC

CACCCACGAAAGTGGCTTTACAACCTTGACTCCACGAAAGCTATGACA

>262_Sample_ASV32

CACCGCGGTTATACGAGAGACCCCAGTTGATAAACACGGCGTAAAGAGTGGTTAAGGGCA

CAAAACAAATAAAGTCAAACAACCTCTTAGCTGTTATACGCATTATGAGGATATGAAGCC

CCCCTACGAAAGTAACTTTACCAACCCCTGACCCCACGAAAGCTAAGAAA

>262_Sample_ASV33

CACCGCGGTTATACGAGAGACCCTAGTTGATATATACGGCGTAAAGAGTGGTTTGGAATA

ACCAAGTAATATAGCCAAAGACCTCCCAAGCCGTCACACGCACCCCGGAGGCACGAAGCC

CAAACACGAAAGTAGCTTTATTAACTTACCGACGCCACGAAAGCTAAGAAA

>262_Sample_ASV34

CACCGCGGTTATACGAAAGACCCTAGTTTATAGGCACGGCGTAAAGGGTGGTTAGGGGAT

AAACAAAATAAAGCCAAAGACCTTCTAAGTCGTCATACACTCATGAAGGCACGAAGCCCA

AATACGAAAGTAACTTTACAACTTAGACCCGACTCCACGAAAGCTGGGGGA

>262_Sample_ASV35

CACCGCGGTTATACGAGAGACCCTAATTAATAGATACGGCGTAAAGAGTGGTTTAGGACA

AAAATTAATAAAGCCGAAGTTTCTCCTAGCCGTTGTACGCACTCTGAGAACACGAAGCCC

CACTACGAAAGTAGCTTTACCTATTATTTCCTGACCCCACGAAAGCTAAGAAA

>262_Sample_ASV36

CACCGCGGTTATACGAGAGGCCCAAGTTGATAGACATCGGCGTAAAGAGTGGTTAAGGAA

GTCTCAAAACTAAAGCCGAATGCCTTCAAGACTGTTATACGTTTCCGAAGGTAAGAAGCC

CCACTACGAAAGTGGCTTTACTCCACCTGACTCCACGAAAGCTGTGAAA

>262_Sample_ASV37

CACCGCGGTTATACGAGAGGCCCAAGTTGATAGACATCGGCGTAAAGAGTGGTTAAGGAA

GTCTCAAAACTAAAGCCGAATGCCTTCAAGACTGTTATACGTTTCCGAAGGTAAGAAGCC

CCACTACGAAAGTGGCTTTATTCCACCTGACCCCACGAAAGCTGTGAAA

>262_Sample_ASV38

CACCGCGGTTATACGAGAGGCCCAAGTTGATAGACATCGGCGTAAAGAGTGGTTAAGGAA

GTCTCAAAACTAAAGCCGAATGCCTTCAAGACTGTTATACGTTTACGAAGGTAAGAAGCC

CCACTACGAAAGTGGCTTTATTCCACCTGACCCCACGAAAGCTGTGAAA

>262_Sample_ASV39

CACCGCGGTTATACGAGAGGCCCAAGTTGACAGATATCGGCGTAAAGAGTGGTTAAGGAA

ATCTTAAAACTAAAGCCGAATGCCTTCAAGACTGTTATACGTTTCCGAAGGTAAGAAGCC

CCACTACGAAAGTGGCTTTACTCCACCTGACTCCACGAAAGCTGTGAAA

>262_Sample_ASV40

CACCGCGGTTATACGAGAGGCCAAAGTTGACAGATATCGGCGTAAAGAGTGGTTAAGGAA

GTCTCAAAACTAAAGCCGAATGCCTTCAAGACTGTTATACGTTTCCGAAGGTAAGAAGCC

CCACTACGAAAGTGGCTTTACTCCACCTGACTCCACGAAAGCTGTGAAA

>262_Sample_ASV41

CACCGCGGTTATACGAGAGGCCAAAGTTGACAGATATCGGCGTAAAGAGTGGTTAAGGAA

ATCTTAAAACTAAAGCCGAATGCCTTCAAGACTGTTATACGTTTCCGAAGGTAAGAAGCC

CCACTACGAAAGTGGCTTTACTCCACCTGACTCCACGAAAGCTGTGAAA

>262_Sample_ASV42

CACCGCGGTTATACGAGAGGCCCAAGTTGATAGACATCGGCGTAAAGAGTGGTTAAGGAA

ATCTTAAAACTAAAGCCGAATGCCTTCAAGACTGTTATACGTTTACGAAGGTAAGAAGCC

CCACTACGAAAGTGGCTTTATTCCACCTGACCCCACGAAAGCTGTGAAA

>262_Sample_ASV43

CACCGCGGTTATACGAGAGGCCCAAGTTGACAGATATCGGCGTAAAGAGTGGTTAAGGAA

ATCTTAAAACTAAAGCCGAATGCCTTCAAGACTGTTATACGTTTACGAAGGTAAGAAGCC

CCACTACGAAAGTGGCTTTACTCCACCTGACTCCACGAAAGCTGTGAAA

>262_Sample_ASV44

CACCGCGGTTATACGAGAGACCCTAATTGATAGACTACGGCGTAAAGAGTGGTTTAGAAC

AAAAATTAATAAAGCCAAAGATCCTCCTGGCCGTCGCACGCATTTCGAGGATACGAAGCC

CCACTACGAAAGTAGCTTTACTACAACTTTTCTGACCCCACGAAAGCTAAGAAA

>263_Sample_ASV1

CACCGCGGTTATACGAGAGGCCCAAGTTGACAGACATCGGCGTAAAGAGTGGTTAAGGAA

AACCTTCAACTAAAGTCGAACGCTCTCAAGACTGTCGTACGTTTCCGAGAGTATGAAGCC

CCACCACGAAAGTGACTTTACCTCACCTGACTCCACGAAAGCTGCGAAA

>263_Sample_ASV2

CACCGCGGTTATACGAAAGACCCTAGTTGATGGGTGCGGCGTAAAGGGTGGTTAGGGACT

ACAAAAAATAGAGCTAAAGATCCTCTAGGTCGTCATACACATCACGAGGAAACGAAGCCC

AAATACGAAAGTAGCTCTAAATATAATCCTGACGCCACGAAAGCCAAGAAA

>263_Sample_ASV3

CACCGCGGTTATACGAAAGACCCTAGTTGATAACTACGGCATAAAGGGTGGTTAGGGAGT

TTAAAAAATAAAGCCAAAGCTCTTCTAAGCTGTCATACGCTCTCTGAAAGAACGAGGCCC

AAACACGAAAGTAGCTTTAAATAAATAACCTGATACCACGAAAGCCAAGAAA

>263_Sample_ASV4

CACCGCGGTTATACGAGAGACCCTAGTTGATTAAAGCGGCGTAAAGAGTGGTTATGGAAC

TATTCTTTTAAAGCAGAAAACCTCTCAAACTGTTATACGCACCCAGAGGTCTGAATCCCT

CACACGAAAGTGACTTTATTTATGCCTACCAGAACCCACGAAAGCTGGGACA

>263_Sample_ASV5

CACCGCGGTTATACGAGAGGCCCAAGTTGACAGACACCGGCGTAAAGAGTGGCTAGGGAA

AAATTACTACTAAAGCCGAACACCTTCAGAACTGTCATACGTTCCCGAAGATAAGAAGCC

CCACCACGAAAGTGACTTTATATTACCCGACCCCACGAAAGCTGTGAAA

>263_Sample_ASV6

CACCGCGGTTATACGAGAGGCCCAAGTTGACAGACACCGGCGTAAAGAGTGGCTAGGGAA

AAATTACTACTAAAGCCGAACACCTTCAGAACTGTCATACGTTCCCGAAGATAAGAAGCC

CCACCACGAAAGTGGCTTTATATTACCCGACCCCACGAAAGCTGTGAAA

>263_Sample_ASV7

CACCGCGGTTATACGAGAGGCCCAAGTTGACAGACACCGGCGTAAAGAGTGGCTAGGGAA

AAATTACTACTAAAGCCGAACACCTTCAGAACTGTCATACGTTCCCGAAGATAAGAAGCC

CCACCACGAAAGTGGCTTTATATTACCCGACCCCACGAAAGCTAAGAAA

>263_Sample_ASV8

CACCGCGGTTATACGAGAGGCCCAAGTTGACAGACACCGGCGTAAAGAGTGGCTAGGGAA

AAATTACTACTAAAGCCGAACACCCTCAGAACTGTCATACGTTCCCGAAGGTAAGAAGCC

CCACCACGAAAGTGGCTTTATAATACCCGACCCCACGAAAGCTGTGAAA

>263_Sample_ASV9

CACCGCGGTTATACGAGAGGCCCAAGTTGACAGACACCGGCGTAAAGAGTGGCTAAGGAA

AAATTTCTACTAAAGCCGAACACCTTCAGAACTGTCATACGTTCCCGAAGAAAAGAAGCC

CCACCACGAAAGTGGCTTTATATTACCCGACCCCACGAAAGCTGTGAAA

>263_Sample_ASV10

CACCGCGGTTATACGAAAGACTCTAGTTGACAAACACGGCGTAAAGGGTGGTTAGGATAA

CAACAAAATAAAGCTAAAGACTTACCAAGCCGTCATACGCCCATGTAAGAGCGAAAACCA

CAAACGAAAGTAACTTTATTACACCATCCGAATCCACGAAAGCTAAGAAA

>263_Sample_ASV11

CACCGCGGTTATACGAAAGACCCTAGTTTATAGAAACGGCGTAAAGGGTGGTTAAGGGAC

AAACAAAATAAGGCCAAAGACTTCCTAAGCCGTCATACGCTCCACGAAAGCACGAAGCCC

AAACACGAAAGTAGCCTTAATAACTTCACCTGACCCCACGAAAGCTGGGAGA

>263_Sample_ASV12

CACCGCGGTTATACGAGAGACCCTAGTTGATATATACGGCGTAAAGAGTGGTTTGGGATA

TTTCTCCAAAATAAAGCCGAAGATCTCCCAAGCCGTCACACGCATCCCGGAGACACGAAG

CCCTGATACGAAGGTAGCTTTATTAATTCCCCGACGCCACGAAAGCTAAGAAA

>263_Sample_ASV13

CACCGCGGTTATACGAGAGACCCTAGTTGATATATACGGCGTAAAGAGTGGTTTGGGATA

TTTCTCCAAAATAAAGTCGAAGATCTCCCAAGCCGTCACACGCATCCCGGAGACACGAAG

CCCTGATACGAAGGTAGCTTTATTAATTCCCCGACGCCACGAAAGCTAAGAAA

>263_Sample_ASV14

CACCGCGGTTATACGAGAGACCCTAGTTGATATATACGGCGTAAAGAGTGGTTTGGGATA

TTTCTCCAAAATAAAGTCGAAGATCTCCCAAGCCGTCACACGCATCCCGGAGACACGAAG

CCCTGATACGAAGGTAGCTTTATTAACACCCCGACGCCACGAAAGCTAAGAAA

>263_Sample_ASV15

CACCGCGGTTATACGAGAGACCCTAGTTGATATATACGGCGTAAAGAGTGGTTTGGGATA

TTTCTCCAAAATAAAGCCGAAGATCTCCCAAGCCGTCACACGCATCCCGGAGACACGAAG

CCCTGATACGAAGGTAGCTTTATTAACACCCCGACGCCACGAAAGCTAAGAAA

>263_Sample_ASV16

CACCGCGGTTATACGAAAGACCCTAGTTGATAGATCACGGCGTAAAGGGTGGTTAGGGAA

TACAAAAATAAAGCTAAAAATCCTCTAAGCTGTCATACGCTTCCCGAAGACATGAAACCC

AACCACGAAAGTAGCTTTAAACTCTCCTGACACCACGAAAGCCAAGGAA

>263_Sample_ASV17

CACCGCGGTTATACGAGAGGCCCAAGTTGATAAGCCCGGCGTAAAGAGTGGTTATGGAGA

ATAAAACACTAAAGCTAAAGACCCCCTAGGCTGTTATACGCACCTGGCGGCTCGAACCAC

CTATACGAAAGTAGCTTTACCCCCCTTCCACCAGAATCCACGACAGCTGGGGCA

>263_Sample_ASV18

CACCGCGGTTATACGAGAGGCCCAAGTTGACAGACATCGGCGTAAAGAGTGGTTAAGGAA

ACCCCATTACTAAAGCCGAACGCCCTCAGAACTGTCATACGTTCCCGAAGGTAAGAAGCC

CCACTACGAAAGTGGCTTTAACCACACCCGACCCCACGAAAGCTGTGAAA

>263_Sample_ASV19

CACCGCGGTTATACGAGAGGCCCAAGTTGATAGATATCGGCGTAAAGAGTGGTTAAGGAA

ACCCCGTTACTAAAGCCGAACGCCCTCAGAACTGTCATACGTTCCCGAAGGTAAGAAGCC

CCACTACGAAAGTGGCTTTAACCACACCCGACCCCACGAAAGCTGTGAAA

>263_Sample_ASV20

CACCGCGGTTATACGAGAGGCCCAAGTTGACAGACACCGGCGTAAAGAGTGGTTAAGGAA

ATTCATCAACTAAAGCCGAATGCCCTCAGGACTGTTATACGTTTGCGAAGGTAAGAAGCC

CTACTACGAAAGTGGCTTTATTCCACCTGATTCCACGAAAGCTGCGGAA

>263_Sample_ASV21

CACCGCGGTTATACGAGAGGCCCCAGTTGATATTAGCGGCGTAAAGGGTGGTTAAGGACT

TTAATAACTAAAGCTAAACACTTCCCCGGCTGTTGTACGTAGACCGGAAGCTTGAAGCCC

AGACACGAAAGTAGCTTTATTCCCCCTGACCCCACGAAAGCTGAGAAA

>263_Sample_ASV22

CACCGCGGTTATACGAGAGACCCCAGTTGATAGCTACGGCGTAAAGAGTGGTTAAGGACC

CCATTTAATAAAGCCAAAGACCCCCCAAGCCGTCATACGCACACGAGGGCGCGAAGCTCG

CACACGAAAGTAGCTCTAATTTACCCGACGCCACGAAAGCTAAGGAA

>263_Sample_ASV23

CACCGCGGTTATACGAGAAACCCTAGTTGACAAATACGGCGTAAAGAGTGGTTAAGGGGT

ATCAAAAATAAAGTCAAACATTTTCCTAGTTGTTATAAGCATTTCGAGAACATGAAGCCC

AACCACGAAAGTAACTTTACCTTCACCTGACCCCACGAAAGCTAAGAAA

>263_Sample_ASV24

GACCGCGGTTATACGAGAGACTCTAGTTGATAGATACGGCGTAAAGAGTGGTTTGGGATA

AACTATAAATAAAGCCAAAGACCTTCCAAGCTGTTAAACGCATACCGAAGGCACGAAGCC

CCAACACGAAAGTAGCTTTACTACTACCCCCGATGCCACGAAAGCTAAGACA

>263_Sample_ASV25

CACCGCGGTTATACGAGAGACCCTAATTGATAGGTACGGCGTAAAGAGTGGTTTGGGATA

ACCCAAAAATAAAGCTAAAGACCTTCTAAGCCGTCACACGCATACCGAAGGCACGAGGCC

CCAACACGAAAGTAGCTTTACTATTACCCCGACGCCACGAAAGCTAAGAAA

>263_Sample_ASV26

CACCGCGGTTATACGAAAGACCCTAGTTGATGGATCACGGCGTAAAGGGTGGTTAAGGGA

TATAAAAATAAAGCTAAAAATCCTCTTAGCTGTCATACGCTTTCCGAAGACATGAGGCCC

AATTACGAAAGTAGCTTTAAACCACCCTGACACCACGAAAGCCAAGAAA

>263_Sample_ASV27

CACCGCGGTTATACGAAAGACCCTAGTTGATGGATCACGGCGTAAAGGGTGGTTAAGGGA

TATAAAAATAAAGCTAAAAATCCTCTTAGCTGTCATACGCTTTCCGAAGACATGAGGCCC

AATTACGAAAGTAGCTTTAAACCACCCTGACACCACGAAAGCTGTGAAA

>263_Sample_ASV28

CACCGCGGTTATACGAGAGGCCCTAGTTGATAACTACCGGCGTAAAGAGTGGTTATGGAA

AAATATTTAATAAAGCCGAACACCCCCTCAGCCGTCATACGCACCTGGGAGCACGAAGAC

CTACTGCGAAAGCAGCTTTAATTATGCCTGACCCCACGACAGCTAAGAAA

>263_Sample_ASV29

CACCGCGGTTATACGAGAGGCCCAAGTTGATACTCCACGGCGTAAAGAGTGGTTAGAAAA

GACCTACTACTAAAGCCGAACGCCTTCAAAGCTGTTATACGCATCCGAAGGTGAGAAGCC

CACCCACGAAAGTGGCTTTACAACCTTGACTCCACGAAAGCTATGACA

>263_Sample_ASV30

CACCGCGGTTATACGAGAGACCCCAGTTGATAAACACGGCGTAAAGAGTGGTTAAGGGCA

CAAAACAAATAAAGTCAAACAACCTCTTAGCTGTTATACGCATTATGAGGATATGAAGCC

CCCCTACGAAAGTAACTTTACCAACCCCTGACCCCACGAAAGCTAAGAAA

>263_Sample_ASV31

CACCGCGGTTATACGAGAGGCCCAAGTTGATAGACATCGGCGTAAAGAGTGGTTAAGGAA

GTCTCAAAACTAAAGCCGAATGCCTTCAAGACTGTTATACGTTTCCGAAGGTAAGAAGCC

CCACTACGAAAGTGGCTTTACTCCACCTGACTCCACGAAAGCTGTGAAA

>263_Sample_ASV32

CACCGCGGTTATACGAGAGACTCCAGTTGATAGCTACGGCGTAAAGAGTGGTTTGGGGCC

CGCCCAAAATAAAGCCAAAGACCTCCCAAGCCGTCAAACGCACCCCGGAGGCACGAAGTC

CTAACGCGAAAGCAACTTTACCTTCCCCGACGCCACGAAAGCTAAGAAA

>263_Sample_ASV33

CACCGCGGTTATACGAAAGACCCTAGTTGATAGATCACGGCGTAAAGGGTGGTTAAGGAG

AACAAAAATAAAGCTAAAAATCCTCTAAGCTGTCATACGCTTTCCGAAGACATGAGACCC

AACCACGAAAGTAACTTTAAACTTTCCTGACACCACGAAAGCCAAGAAA

>263_Sample_ASV34

CACCGCGGTTATACGAAAGACCCTAGTTGATAGATCACGGCGTAAAGGGTGGTTAAGGAA

AACAAAAATAAAGCTAAAAATCCTCTAAGCTGTCATACGCTTTCCGAAGACATGAGATCC

AACCACGAAAGTAGCTTTAAAATTTCCTGACACCACGAAAGCCAAGGAA

>264_Sample_ASV1

CACCGCGGTTATACGAGAGGCCCAAGTTGACAGACATCGGCGTAAAGAGTGGTTAAGGAA

AACCTTCAACTAAAGTCGAACGCTCTCAAGACTGTCGTACGTTTCCGAGAGTATGAAGCC

CCACCACGAAAGTGACTTTACCTCACCTGACTCCACGAAAGCTGCGAAA

>264_Sample_ASV2

CACCGCGGTTATACGAAAGACCCTAGTTGATGGGTGCGGCGTAAAGGGTGGTTAGGGACT

ACAAAAAATAGAGCTAAAGATCCTCTAGGTCGTCATACACATCACGAGGAAACGAAGCCC

AAATACGAAAGTAGCTCTAAATATAATCCTGACGCCACGAAAGCCAAGAAA

>264_Sample_ASV3

CACCGCGGTTATACGAAAGACCCTAGTTGATAACTACGGCATAAAGGGTGGTTAGGGAGT

TTAAAAAATAAAGCCAAAGCTCTTCTAAGCTGTCATACGCTCTCTGAAAGAACGAGGCCC

AAACACGAAAGTAGCTTTAAATAAATAACCTGATACCACGAAAGCCAAGAAA

>264_Sample_ASV4

CACCGCGGTTATACGAGAGACCCTAGTTGATTAAAGCGGCGTAAAGAGTGGTTATGGAAC

TATTCTTTTAAAGCAGAAAACCTCTCAAACTGTTATACGCACCCAGAGGTCTGAATCCCT

CACACGAAAGTGACTTTATTTATGCCTACCAGAACCCACGAAAGCTGGGACA

>264_Sample_ASV5

CACCGCGGTTATACGAGAGGCCCAAGTTGACAGACACCGGCGTAAAGAGTGGCTAGGGAA

AAATTACTACTAAAGCCGAACACCTTCAGAACTGTCATACGTTCCCGAAGATAAGAAGCC

CCACCACGAAAGTGACTTTATATTACCCGACCCCACGAAAGCTGTGAAA

>264_Sample_ASV6

CACCGCGGTTATACGAGAGGCCCAAGTTGACAGACACCGGCGTAAAGAGTGGCTAGGGAA

AAATTACTACTAAAGCCGAACACCTTCAGAACTGTCATACGTTCCCGAAGATAAGAAGCC

CCACCACGAAAGTGGCTTTATATTACCCGACCCCACGAAAGCTGTGAAA

>264_Sample_ASV7

CACCGCGGTTATACGAGAGGCCCAAGTTGACAGACACCGGCGTAAAGAGTGGCTAGGGAA

AAATTACTACTAAAGCCGAACACCCTCAGAACTGTCATACGTTCCCGAAGGTAAGAAGCC

CCACCACGAAAGTGGCTTTATAATACCCGACCCCACGAAAGCTGTGAAA

>264_Sample_ASV8

CACCGCGGTTATACGAGAGGCCCAAGTTGACAGACACCGGCGTAAAGAGTGGCTAAGGAA

AAATTTCTACTAAAGCCGAACACCTTCAGAACTGTCATACGTTCCCGAAGATAAGAAGCC

CCACCACGAAAGTGGCTTTATATTACCCGACACCACGAAAGCTGTGAAA

>264_Sample_ASV9

CACCGCGGTTATACGAAAGACCCTAGTTTATAGAAACGGCGTAAAGGGTGGTTAAGGGAC

AAACAAAATAAGGCCAAAGACTTCCTAAGCCGTCATACGCTCCACGAAAGCACGAAGCCC

AAACACGAAAGTAGCCTTAATAACTTCACCTGACCCCACGAAAGCTGGGAGA

>264_Sample_ASV10

CACCGCGGTTAAACGAGAGACCCTAGTTGATCATCACGGCGTAAAGAGTGGTTAGGGGAT

TATCATAAATAAAGCCAAAGACCTCCCAAGCTGTCGCACGCATTCCGGAGGCGCGAAGCC

CACACACGAAAGTAGCTTTAACTATTGACCCTGATGCCACGAAAGCTAAGAAA

>264_Sample_ASV11

CACCGCGGTTATACGAGAGACCCTAGTTGATATATACGGCGTAAAGAGTGGTTTGGGATA

TTTCTCCAAAATAAAGTCGAAGATCTCCCAAGCCGTCACACGCATCCCGGAGACACGAAG

CCCTGATACGAAGGTAGCTTTATTAACACCCCGACGCCACGAAAGCTAAGAAA

>264_Sample_ASV12

CACCGCGGTTATACGAGAGACCCTAGTTGATATATACGGCGTAAAGAGTGGTTTGGGATA

TTTCTCCAAAATAAAGCCGAAGATCTCCCAAGCCGTCACACGCATCCCGGAGACACGAAG

CCCTGATACGAAGGTAGCTTTATTAACACCCCGACGCCACGAAAGCTAAGAAA

>264_Sample_ASV13

CACCGCGGTTATACGAGAGACCCTAGTTGATATATACGGCGTAAAGAGTGGTTTGGGGTT

ATGCCTCCAAAATAAAGTCGAAGATCTCCCAAGCCGTCACACGCACCCCGGAGACACGAA

GCCCTAATACGAAGGTAGCTTTATTAATTCCCCGACGCCACGAAAGCTAAGAAA

>264_Sample_ASV14

CACCGCGGTTATACGAGAGACCCTAGTTGATATATACGGCGTAAAGAGTGGTTTGGGGTT

ATGCCTCCAAAATAAAGCCGAAGATCTCCCAAGCCGTCACACGCACCCCGGAGACACGAA

GCCCTAATACGAAGGTAGCTTTATTAATTCCCCGACGCCACGAAAGCTAAGAAA

>264_Sample_ASV15

CACCGCGGTTATACGAGAGACCCTAGTTGATATATACGGCGTAAAGAGTGGTTTGGGATA

TTTCTCCAAAATAAAGCCGAAGATCTCCCAAGCCGTCACACGCATCCCGGAGACACGAAG

CCCTAATACGAAGGTAGCTTTATTAGTACCCCGACGCCACGAAAGCTAAGAAA

>264_Sample_ASV16

CACCGCGGTTATACGAGAGACCCTAGTTGATATATACGGCGTAAAGAGTGGTTTGGGGTT

ATGCCTCCAAAATAAAGCCGAAGATCTCCCAAGCCGTCACACGCATCCCGGAGACACGAA

GCCCTAATACGAAGGTAGCTTTATTAATTCCCCGACGCCACGAAAGCTAAGAAA

>264_Sample_ASV17

CACCGCGGTTATACGAGAGACCCTAGTTGATATATACGGCGTAAAGAGTGGTTTGGGGTT

ATGCCTCCAAAATAAAGCCGAAGATCTCCCAAGCCGTCACACGCATCCCGGAGACACGAA

GCCCTAATACGAAGGTAGCTTTATTAGTACCCCGACGCCACGAAAGCTAAGAAA

>264_Sample_ASV18

CACCGCGGTTATACGAGAGGCCCAAGTTGATAAGCCCGGCGTAAAGAGTGGTTATGGAGA

ATAAAACACTAAAGCTAAAGACCCCCTAGGCTGTTATACGCACCTGGCGGCTCGAACCAC

CTATACGAAAGTAGCTTTACCCCCCTTCCACCAGAATCCACGACAGCTGGGGCA

>264_Sample_ASV19

CACCGCGGTTATACGAGAGGCCCAAGTTGACAGACATCGGCGTAAAGAGTGGTTAAGGAA

ACCCCATTACTAAAGCCGAACGCCCTCAGAACTGTCATACGTTCCCGAAGGTAAGAAGCC

CCACTACGAAAGTGGCTTTAACCACACCCGACCCCACGAAAGCTGTGAAA

>264_Sample_ASV20

CACCGCGGTTATACGAGAGGCCCAAGTTGACAGACACCGGCGTAAAGAGTGGTTAAGGAA

ATTCATCAACTAAAGCCGAATGCCCTCAGGACTGTTATACGTTTGCGAAGGTAAGAAGCC

CTACTACGAAAGTGGCTTTATTCCACCTGATTCCACGAAAGCTGCGGAA

>264_Sample_ASV21

CACCGCGGTTATACGAGAGGCCCCAGTTGATATTAGCGGCGTAAAGGGTGGTTAAGGACT

TTAATAACTAAAGCTAAACACTTCCCCGGCTGTTGTACGTAGACCGGAAGCTTGAAGCCC

AGACACGAAAGTAGCTTTATTCCCCCTGACCCCACGAAAGCTGAGAAA

>264_Sample_ASV22

CACCGCGGTTATACGAGAAACCCTAGTTGACAAATACGGCGTAAAGAGTGGTTAAGGGGT

ATCAAAAATAAAGTCAAACATTTTCCTAGTTGTTATAAGCATTTCGAGAACATGAAGCCC

AACCACGAAAGTAACTTTACCTTCACCTGACCCCACGAAAGCTAAGAAA

>264_Sample_ASV23

CACCGCGGTTATACGAGAGACCCTAATTGATAGGTACGGCGTAAAGAGTGGTTTGGGATA

ACCCAAAAATAAAGCTAAAGACCTTCTAAGCCGTCACACGCATACCGAAGGCACGAGGCC

CCAACACGAAAGTAGCTTTACTATTACCCCGACGCCACGAAAGCTAAGAAA

>264_Sample_ASV24

CACCGCGGTTATACGAAAGACCCCAGTTTATAGATGCGGCGTAAAGGGTGGTTAAGAGAA

AATCAAAATAAGGCCAAAGATCCCCTAAGCAGTCATACGCTCCACGAGGACACGAAACCC

AAATACGAAAGTTGCCTTAATAAACTTCACCTGATCCCACGAAAGCTGAAAAA

>264_Sample_ASV25

CACCGCGGTTATACGAGAGGCCCTAGTTGATAACTACCGGCGTAAAGAGTGGTTATGGAA

AAATATTTAATAAAGCCGAACACCCCCTCAGCCGTCATACGCACCTGGGAGCACGAAGAC

CTACTGCGAAAGCAGCTTTAATTATGCCTGACCCCACGACAGCTAAGAAA

>264_Sample_ASV26

CACCGCGGTTATACGAAAGACCCTAGTTGATAGCCACGGCGTAAAGGGTGGTTAAGGTAT

ACAACAAATAAAGCTAAATAGCCTCTAGGCCGTCGCACGCATTCCGAGAGCTCGAAACCC

AAAAACGAAAGTAGCTTTAAAATAAACAGACCTGACCCCACGAAAGCTAAGAAA

>264_Sample_ASV27

CACCGCGGTTATACGAGAGACCCCAGTTGATAAACACGGCGTAAAGAGTGGTTAAGGGCA

CAAAACAAATAAAGTCAAACAACCTCTTAGCTGTTATACGCATTATGAGGATATGAAGCC

CCCCTACGAAAGTAACTTTACCAACCCCTGACCCCACGAAAGCTAAGAAA

>264_Sample_ASV28

CACCGCGGTTATACGAAAGACCCTAGTTTATAGGCACGGCGTAAAGGGTGGTTAGGGGAT

AAACAAAATAAAGCCAAAGACCTTCTAAGTCGTCATACACTCATGAAGGCACGAAGCCCA

AATACGAAAGTAACTTTACAACTTAGACCCGACTCCACGAAAGCTGGGGGA

>264_Sample_ASV29

CACCGCGGTTATACGAGAGGCCCAAGTTGATAGACATCGGCGTAAAGAGTGGTTAAGGAA

GTCTCAAAACTAAAGCCGAATGCCTTCAAGACTGTTATACGTTTCCGAAGGTAAGAAGCC

CCACTACGAAAGTGGCTTTACTCCACCTGACTCCACGAAAGCTGTGAAA

>264_Sample_ASV30

CACCGCGGTTATACGAGAGACTCCAGTTGATAGCTACGGCGTAAAGAGTGGTTTGGGGCC

CGCCCAAAATAAAGCCAAAGACCTCCCAAGCCGTCAAACGCACCCCGGAGGCACGAAGTC

CTAACGCGAAAGCAACTTTACCTTCCCCGACGCCACGAAAGCTAAGAAA

>264_Sample_ASV31

CACCGCGGTTATACGAGAGACCCTAATTGATAGACTACGGCGTAAAGAGTGGTTTAGAAC

AAAAATTAATAAAGCCAAAGATCCTCCTGGCCGTCGCACGCATTTCGAGGATACGAAGCC

CCACTACGAAAGTAGCTTTACTACAACTTTTCTGACCCCACGAAAGCTAAGAAA

>264_Sample_ASV32

CACCGCGGTTATACGAAAGACCCTAGTTGATAGATCACGGCGTAAAGGGTGGTTAAGGAG

AACAAAAATAAAGCTAAAAATCCTCTAAGCTGTCATACGCTTTCCGAAGACATGAGACCC

AACCACGAAAGTAACTTTAAACTTTCCTGACACCACGAAAGCCAAGAAA

>265_Sample_ASV1

CACCGCGGTTATACGAGAGGCCCAAGTTGACAGACATCGGCGTAAAGAGTGGTTAAGGAA

AACCTTCAACTAAAGTCGAACGCTCTCAAGACTGTCGTACGTTTCCGAGAGTATGAAGCC

CCACCACGAAAGTGACTTTACCTCACCTGACTCCACGAAAGCTGCGAAA

>265_Sample_ASV2

CACCGCGGTTATACGAAAGACCCTAGTTGATGGGTGCGGCGTAAAGGGTGGTTAGGGACT

ACAAAAAATAGAGCTAAAGATCCTCTAGGTCGTCATACACATCACGAGGAAACGAAGCCC

AAATACGAAAGTAGCTCTAAATATAATCCTGACGCCACGAAAGCCAAGAAA

>265_Sample_ASV3

CACCGCGGTTATACGAAAGACCCTAGTTGATAACTACGGCATAAAGGGTGGTTAGGGAGT

TTAAAAAATAAAGCCAAAGCTCTTCTAAGCTGTCATACGCTCTCTGAAAGAACGAGGCCC

AAACACGAAAGTAGCTTTAAATAAATAACCTGATACCACGAAAGCCAAGAAA

>265_Sample_ASV4

CACCGCGGTTATACGAGAGACCCTAGTTGATTAAAGCGGCGTAAAGAGTGGTTATGGAAC

TATTCTTTTAAAGCAGAAAACCTCTCAAACTGTTATACGCACCCAGAGGTCTGAATCCCT

CACACGAAAGTGACTTTATTTATGCCTACCAGAACCCACGAAAGCTGGGACA

>265_Sample_ASV5

CACCGCGGTTATACGAGAGGCCCAAGTTGACAGACACCGGCGTAAAGAGTGGCTAGGGAA

AAATTACTACTAAAGCCGAACACCTTCAGAACTGTCATACGTTCCCGAAGATAAGAAGCC

CCACCACGAAAGTGGCTTTATATTACCCGACCCCACGAAAGCTGTGAAA

>265_Sample_ASV6

CACCGCGGTTATACGAAAGACCCTAGTTTATAGAAACGGCGTAAAGGGTGGTTAAGGGAC

AAACAAAATAAGGCCAAAGACTTCCTAAGCCGTCATACGCTCCACGAAAGCACGAAGCCC

AAACACGAAAGTAGCCTTAATAACTTCACCTGACCCCACGAAAGCTGGGAGA

>265_Sample_ASV7

CACCGCGGTTATACGAGAGACCCTAGTTGATATATACGGCGTAAAGAGTGGTTTGGGATA

TTTCTCCAAAATAAAGTCGAAGATCTCCCAAGCCGTCACACGCATCCCGGAGACACGAAG

CCCTGATACGAAGGTAGCTTTATTAACACCCCGACGCCACGAAAGCTAAGAAA

>265_Sample_ASV8

CACCGCGGTTATACGAGAGACCCTAGTTGATATATACGGCGTAAAGAGTGGTTTGGGATA

TTTCTCCAAAATAAAGCCGAAGATCTCCCAAGCCGTCACACGCATCCCGGAGACACGAAG

CCCTGATACGAAGGTAGCTTTATTAACACCCCGACGCCACGAAAGCTAAGAAA

>265_Sample_ASV9

CACCGCGGTTATACGAAAGACCCTAGTTGATAGATCACGGCGTAAAGGGTGGTTAGGGAA

TACAAAAATAAAGCTAAAAATCCTCTAAGCTGTCATACGCTTCCCGAAGACATGAAACCC

AACCACGAAAGTAGCTTTAAACTCTCCTGACACCACGAAAGCCAAGGAA

>265_Sample_ASV10

CACCGCGGTTATACGAGAGGCCCAAGTTGATAAGCCCGGCGTAAAGAGTGGTTATGGAGA

ATAAAACACTAAAGCTAAAGACCCCCTAGGCTGTTATACGCACCTGGCGGCTCGAACCAC

CTATACGAAAGTAGCTTTACCCCCCTTCCACCAGAATCCACGACAGCTGGGGCA

>265_Sample_ASV11

CACCGCGGTTATACGAGAGGCCCAAGTTGACAGACATCGGCGTAAAGAGTGGTTAAGGAA

ACCCCATTACTAAAGCCGAACGCCCTCAGAACTGTCATACGTTCCCGAAGGTAAGAAGCC

CCACTACGAAAGTGGCTTTAACCACACCCGACCCCACGAAAGCTGTGAAA

>265_Sample_ASV12

CACCGCGGTTATACGAGAGGCCCAAGTTGACAGACACCGGCGTAAAGAGTGGTTAAGGAA

ATTCATCAACTAAAGCCGAATGCCCTCAGGACTGTTATACGTTTGCGAAGGTAAGAAGCC

CTACTACGAAAGTGGCTTTATTCCACCTGATTCCACGAAAGCTGCGGAA

>265_Sample_ASV13

CACCGCGGTTATACGAGAGGCCCCAGTTGATATTAGCGGCGTAAAGGGTGGTTAAGGACT

TTAATAACTAAAGCTAAACACTTCCCCGGCTGTTGTACGTAGACCGGAAGCTTGAAGCCC

AGACACGAAAGTAGCTTTATTCCCCCTGACCCCACGAAAGCTGAGAAA

>265_Sample_ASV14

CACCGCGGTTATACGAGAGGCCCTAGTTGATAAATACGGCGTAAAGAGTGGTTAGAGGAC

TCCCATAATAAAGCCAAAGACCCCCCGGGCCGTCACACGCATACGAGGGCACGAAGTTCA

CACACGAAAGTAGCTTTAAATTACCCGACGCCACGAAAGCTAAGAAA

>265_Sample_ASV15

CACCGCGGTTATACGAGAAACCCTAGTTGACAAATACGGCGTAAAGAGTGGTTAAGGGGT

ATCAAAAATAAAGTCAAACATTTTCCTAGTTGTTATAAGCATTTCGAGAACATGAAGCCC

AACCACGAAAGTAACTTTACCTTCACCTGACCCCACGAAAGCTAAGAAA

>265_Sample_ASV16

CACCGCGGTTATACGAGAGACCCTAATTGATAGGTACGGCGTAAAGAGTGGTTTGGGATA

ACCCAAAAATAAAGCTAAAGACCTTCTAAGCCGTCACACGCATACCGAAGGCACGAGGCC

CCAACACGAAAGTAGCTTTACTATTACCCCGACGCCACGAAAGCTAAGAAA

>265_Sample_ASV17

CACCGCGGTTATACGAAAGACCCCAGTTTATAGATGCGGCGTAAAGGGTGGTTAAGAGAA

AATCAAAATAAGGCCAAAGATCCCCTAAGCAGTCATACGCTCCACGAGGACACGAAACCC

AAATACGAAAGTTGCCTTAATAAACTTCACCTGATCCCACGAAAGCTGAAAAA

>265_Sample_ASV18

CACCGCGGTTATACGAGAGACTCTAGTTGATACCAACGGCGTAAAGAGTGGTTTGGGATG

CCCCTTAAAATAAAGCCAAAGACCTCCCAAGCCGTCAAACGCACCCCGGAGGCACGAAGC

CCCAACGCGAAAGCAACTTTACCTCCCTCCCGACGCCACGAAAGCTAAGAAA

>265_Sample_ASV19

CACCGCGGTTATACGAGAGGCCCTAGTTGATAACTACCGGCGTAAAGAGTGGTTATGGAA

AAATATTTAATAAAGCCGAACACCCCCTCAGCCGTCATACGCACCTGGGAGCACGAAGAC

CTACTGCGAAAGCAGCTTTAATTATGCCTGACCCCACGACAGCTAAGAAA

>265_Sample_ASV20

CACCGCGGTTATACGAGAGACCCCAGTTGATAAACACGGCGTAAAGAGTGGTTAAGGGCA

CAAAACAAATAAAGTCAAACAACCTCTTAGCTGTTATACGCATTATGAGGATATGAAGCC

CCCCTACGAAAGTAACTTTACCAACCCCTGACCCCACGAAAGCTAAGAAA

>265_Sample_ASV21

CACCGCGGTTATACGAGAGGCCCAAGTTGATAGACATCGGCGTAAAGAGTGGTTAAGGAA

GTCTCAAAACTAAAGCCGAATGCCTTCAAGACTGTTATACGTTTCCGAAGGTAAGAAGCC

CCACTACGAAAGTGGCTTTACTCCACCTGACTCCACGAAAGCTGTGAAA

>265_Sample_ASV22

CACCGCGGTTATACGAGAGACTCCAGTTGATAGCTACGGCGTAAAGAGTGGTTTGGGGCC

CGCCCAAAATAAAGCCAAAGACCTCCCAAGCCGTCAAACGCACCCCGGAGGCACGAAGTC

CTAACGCGAAAGCAACTTTACCTTCCCCGACGCCACGAAAGCTAAGAAA

>265_Sample_ASV23

CACCGCGGTTATACGAAAGACCCTAGTTGATAGATCACGGCGTAAAGGGTGGTTAAGGAG

AACAAAAATAAAGCTAAAAATCCTCTAAGCTGTCATACGCTTTCCGAAGACATGAGACCC

AACCACGAAAGTAACTTTAAACTTTCCTGACACCACGAAAGCCAAGAAA

>266_Sample_ASV1

CACCGCGGTTATACGAAAGACCCTAGTTGATGGGTGCGGCGTAAAGGGTGGTTAGGGACT

ACAAAAAATAGAGCTAAAGATCCTCTAGGTCGTCATACACATCACGAGGAAACGAAGCCC

AAATACGAAAGTAGCTCTAAATATAATCCTGACGCCACGAAAGCCAAGAAA

>266_Sample_ASV2

CACCGCGGTTATACGAGAGACCCTAGTTGATTAAAGCGGCGTAAAGAGTGGTTATGGAAC

TATTCTTTTAAAGCAGAAAACCTCTCAAACTGTTATACGCACCCAGAGGTCTGAATCCCT

CACACGAAAGTGACTTTATTTATGCCTACCAGAACCCACGAAAGCTGGGACA

>266_Sample_ASV3

CACCGCGGTTATACGAGAGGCCCAAGTTGACAGACACCGGCGTAAAGAGTGGCTAGGGAA

AAATTACTACTAAAGCCGAACACCTTCAGAACTGTCATACGTTCCCGAAGATAAGAAGCC

CCACCACGAAAGTGACTTTATATTACCCGACCCCACGAAAGCTGTGAAA

>266_Sample_ASV4

CACCGCGGTTATACGAGAGGCCCAAGTTGACAGACACCGGCGTAAAGAGTGGCTAGGGAA

AAATTACTACTAAAGCCGAACACCCTCAGAACTGTCATACGTTCCCGAAGGTAAGAAGCC

CCACCACGAAAGTGGCTTTATAATACCCGACCCCACGAAAGCTGAGAAA

>266_Sample_ASV5

CACCGCGGTTATACGAGAGGCCCAAGTTGACAGACACCGGCGTAAAGAGTGGCTAGGGAA

AAATTACTACTAAAGCCGAACACCCTCAGAACTGTCATACGTTCCCGAAGGTAAGAAGCC

CCACCACGAAAGTGGCTTTATAATACCCGACCCCACGAAAGCTGTGAAA

>266_Sample_ASV6

CACCGCGGTTATACGAGAGGCCCAAGTTGACAGACACCGGCGTAAAGAGTGGCTAGGGAA

AAATTACTACTAAAGCCGAACACCTTCAGAACTGTCATACGTTCCCGAAGATAAGAAGCC

CCACCACGAAAGTGGCTTTATATTACCCGACCCCACGAAAGCTGTGAAA

>266_Sample_ASV7

CACCGCGGTTATACGAGAGGCCCAAGTTGACAGACACCGGCGTAAAGAGTGGCTAAGGAA

AAATTTCTACTAAAGCCGAACACCTTCAGAACTGTCATACGTTCCCGAAGAAAAGAAGCC

CCACCACGAAAGTGGCTTTATATTACCCGACCCCACGAAAGCTGTGAAA

>266_Sample_ASV8

CACCGCGGTTATACGAGAGGCCCAAGTTGACAGACACCGGCGTAAAGAGTGGCTAAGGAA

AAATTTCTACTAAAGCCGAACACCTTCAGAACTGTCATACGTTCCCGAAGATAAGAAGCC

CCACCACGAAAGTGGCTTTATATTACCCGACACCACGAAAGCTGAGAAA

>266_Sample_ASV9

CACCGCGGTTATACGAGAGGCCCAAGTTGACAGACACCGGCGTAAAGAGTGGCTAAGGAA

AAATTTCTACTAAAGCCGAACACCTTCAGAACTGTCATACGTTCCCGAAGATAAGAAGCC

CCACCACGAAAGTGGCTTTATATTACCCGACACCACGAAAGCTGTGAAA

>266_Sample_ASV10

CACCGCGGTTAAACGAGAGACCCTAGTTGATCATCACGGCGTAAAGAGTGGTTAGGGGAT

TATCATAAATAAAGCCAAAGACCTCCCAAGCTGTCGCACGCATTCCGGAGGCGCGAAGCC

CACACACGAAAGTAGCTTTAACTATTGACCCTGATGCCACGAAAGCTAAGAAA

>266_Sample_ASV11

CACCGCGGTTATACGAAAGACCCTAGTTGATAGATCACGGCGTAAAGGGTGGTTAGGGAA

TACAAAAATAAAGCTAAAAATCCTCTAAGCTGTCATACGCTTCCCGAAGACATGAAACCC

AACCACGAAAGTAGCTTTAAACTCTCCTGACACCACGAAAGCCAAGGAA

>266_Sample_ASV12

CACCGCGGTTATACGAAAGACCCTAGTTTATAGGCACGGCGTAAAGGGTGGTTAGGGAAT

ACAAAAATAAAGCTAAAAATCCTCTAAGCTGTCATACGCTTCCCGAAGACATGAAACCCA

ACCACGAAAGTAGCTTTAAACTCTCCTGACACCACGAAAGCCAAGGAA

>266_Sample_ASV13

CACCGCGGTTATACGAGAGGCCCAAGTTGATAAGCCCGGCGTAAAGAGTGGTTATGGAGA

ATAAAACACTAAAGCTAAAGACCCCCTAGGCTGTTATACGCACCTGGCGGCTCGAACCAC

CTATACGAAAGTAGCTTTACCCCCCTTCCACCAGAATCCACGACAGCTGGGGCA

>266_Sample_ASV14

CACCGCGGTTATACGAGAGGCCCTAGTTGATAGCCACGGCGCAAAGAGTGGTTAAGGAGC

CCCACTAAATAAAGTCGAACACTTCCTAGGCCGTTATACGTTTTCTAGAAGCACGAAACC

CAATTACACGAAAGCAACTTTATACTAAACAACCTGACCCCACGAAAGCTAAGAAA

>266_Sample_ASV15

CACCGCGGTTATACGAGAGGCCCAAGTTGACAGACATCGGCGTAAAGAGTGGTTAAGGAA

ACCCCATTACTAAAGCCGAACGCCCTCAGAACTGTCATACGTTCCCGAAGGTAAGAAGCC

CCACTACGAAAGTGGCTTTAACCACACCCGACCCCACGAAAGCTAAGAAA

>266_Sample_ASV16

CACCGCGGTTATACGAGAGGCCCAAGTTGACAGACATCGGCGTAAAGAGTGGTTAAGGAA

ACCCCATTACTAAAGCCGAACGCCCTCAGAACTGTCATACGTTCCCGAAGGTAAGAAGCC

CCACTACGAAAGTGGCTTTAACCACACCCGACCCCACGAAAGCTGTGAAA

>266_Sample_ASV17

CACCGCGGTTATACGAAAGACCCCAGTTTATAGACACGGCGTAAAGGGTGGTTATAGGAC

AAACTAACATAGAGCCAAAGACCCCCTAAGCCGTCATACGCTCACGAGGGCTCGAAGCCC

CTATACGAAAGTAGCTCTACCAAAAAATGCTCGACTCCACGAAAGCTGGGACA

>266_Sample_ASV18

CACCGCGGTTATACGAGAGACCCTAATTGATAGACACGGCGTAAAGAGTGGTTTGGGAAA

GCCCAAAAATAAAGCTAAAGACCTTCTAAGCCGTCACACGCACGCCGAAGGCACGAAGCC

CTGACACGAAAGTAGCTTTACTATTACCCCCGACGCCACGAAAGCTAAGAAA

>266_Sample_ASV19

CACCGCGGTTATACGAAAGACCCTAGTTGATGGATCACGGCGTAAAGGGTGGTTAAGGGA

TATAAAAATAAAGCTAAAAATCCTCTTAGCTGTCATACGCTTTCCGAAGACATGAGGCCC

AATTACGAAAGTAGCTTTAAACCACCCTGACACCACGAAAGCCAAGAAA

>266_Sample_ASV20

CACCGCGGTTATACGAGAGGCCCTAGTTGATAACTACCGGCGTAAAGAGTGGTTATGGAA

AAATATTTAATAAAGCCGAACACCCCCTCAGCCGTCATACGCACCTGGGAGCACGAAGAC

CTACTGCGAAAGCAGCTTTAATTATGCCTGACCCCACGACAGCTAAGAAA

>266_Sample_ASV21

CACCGCGGTTATACGAGAGGCCCAAGTTGATTATTACCGGCGTAAAGCGTGGTTAAGATT

TTTATAGGACTAAAGCCGAACCCCCTCAGAGCTGTTATACGCACCCGAAGGTTAGAAGTT

CAATCACGAAAGTGGCTTTACAACCCCGAACCCACGAAAGCTAAGATA

>266_Sample_ASV22

CACCGCGGTTATACGAGAGACCCTAATTGATAGGCGCGGCGTAAAGAGTGGTTTAGGACT

AAAACTAATAAAGCTAAAGATCCTCCCAGCCGTCGCACGCATTCTGAGGACACGAAACTC

AACCACGAAAGTAGCTTTAACATACTCTCCTGACCCCACGAAAGCTAAGAAA

>266_Sample_ASV23

CACCGCGGTTATACGAAAGACCCTAGTTGATAGGCACGGCGTAAAGGGTGGTTAGGGATA

TATTAAAAATAAAGTTAAAGAGCCTCTAAGCTGTCGCACGCATCCCGAGAGCTCGAGACC

CAGACACGAAAGTAACTTTAAAATACCTCACCTGACCCCACGAAAGCTAAGAAA

>266_Sample_ASV24

CACCGCGGTTATACGAGAGGCCCAAGTTGATACTCCACGGCGTAAAGAGTGGTTAGAAAA

GACCTACTACTAAAGCCGAACGCCTTCAAAGCTGTTATACGCATCCGAAGGTGAGAAGCC

CACCCACGAAAGTGGCTTTACAACCTTGACTCCACGAAAGCTATGACA

>266_Sample_ASV25

CACCGCGGTTATACGAGAGACCCCAGTTGATAAACACGGCGTAAAGAGTGGTTAAGGGCA

CAAAACAAATAAAGTCAAACAACCTCTTAGCTGTTATACGCATTATGAGGATATGAAGCC

CCCCTACGAAAGTAACTTTACCAACCCCTGACCCCACGAAAGCTAAGAAA

>266_Sample_ASV26

CACCGCGGTTATACGAAAGACCCTAGTTTATAGGCACGGCGTAAAGGGTGGTTAGGGGAT

AAACAAAATAAAGCCAAAGACCTTCTAAGTCGTCATACACTCATGAAGGCACGAAGCCCA

AATACGAAAGTAACTTTACAACTTAGACCCGACTCCACGAAAGCCAAGGAA

>266_Sample_ASV27

CACCGCGGTTATACGAAAGACCCTAGTTTATAGGCACGGCGTAAAGGGTGGTTAGGGGAT

AAACAAAATAAAGCCAAAGACCTTCTAAGTCGTCATACACTCATGAAGGCACGAAGCCCA

AATACGAAAGTAACTTTACAACTTAGACCCGACTCCACGAAAGCTGGGGGA

>266_Sample_ASV28

CACCGCGGTTATACGAGAGACCCTAATTAATAGCTACGGCGTAAAGAGTGGTTTAGGGTA

AAAATTAATAAAGCCGAAGTTTCTCCTAGCCGTTGTACGCACTCTGAGAACACGAAGCCC

CACTACGAAAGTAGCTTTACCTATTATTTCCTGACCCCACGAAAGCTAAGAAA

>266_Sample_ASV29

CACCGCGGTTATACGAGAGACCCTAATTAATAGATACGGCGTAAAGAGTGGTTTAGGACA

AAAATTAATAAAGCCGAAGTTTCTCCTAGCCGTTGTACGCACTCTGAGAACACGAAGCCC

CACTACGAAAGTAGCTTTACCTATTATTTCCTGACCCCACGAAAGCTAAGAAA

>266_Sample_ASV30

CACCGCGGTTATACGAAAGACCCTAGTTGATAGATCACGGCGTAAAGGGTGGTTAAGGAA

AACAAAAATAAAGCTAAAAATCCTCTAAGCTGTCATACGCTTTCCGAAGACATGAGATCC

AACCACGAAAGTAGCTTTAAAATTTCCTGACACCACGAAAGCCAAGGAA

>267_Sample_ASV1

CACCGCGGTTATACGAGAGGCCCAAGTTGACAGACATCGGCGTAAAGAGTGGTTAAGGAA

AACCTTCAACTAAAGTCGAACGCTCTCAAGACTGTCGTACGTTTCCGAGAGTATGAAGCC

CCACCACGAAAGTGACTTTACCTCACCTGACTCCACGAAAGCTGCGAAA

>267_Sample_ASV2

CACCGCGGTTATACGAAAGACCCTAGTTGATGGGTGCGGCGTAAAGGGTGGTTAGGGACT

ACAAAAAATAGAGCTAAAGATCCTCTAGGTCGTCATACACATCACGAGGAAACGAAGCCC

AAATACGAAAGTAGCTCTAAATATAATCCTGACGCCACGAAAGCCAAGAAA

>267_Sample_ASV3

CACCGCGGTTATACGAAAGACCCTAGTTGATAACTACGGCATAAAGGGTGGTTAGGGAGT

TTAAAAAATAAAGCCAAAGCTCTTCTAAGCTGTCATACGCTCTCTGAAAGAACGAGGCCC

AAACACGAAAGTAGCTTTAAATAAATAACCTGATACCACGAAAGCCAAGAAA

>267_Sample_ASV4

CACCGCGGTTATACGAGAGACCCTAGTTGATTAAAGCGGCGTAAAGAGTGGTTATGGAAC

TATTCTTTTAAAGCAGAAAACCTCTCAAACTGTTATACGCACCCAGAGGTCTGAATCCCT

CACACGAAAGTGACTTTATTTATGCCTACCAGAACCCACGAAAGCTGGGACA

>267_Sample_ASV5

CACCGCGGTTATACGAAAGACCCAAGTTGACAGACACCGGCGTAAAGAGTGGCTAGGGAA

AAATTACTACTAAAGCCGAACACCTTCAGAACTGTCATACGTTCCCGAAGATAAGAAGCC

CCACCACGAAAGTGACTTTATATTACCCGACCCCACGAAAGCTAAGAAA

>267_Sample_ASV6

CACCGCGGTTATACGAGAGGCCCAAGTTGACAGACACCGGCGTAAAGAGTGGCTAGGGAA

AAATTACTACTAAAGCCGAACACCTTCAGAACTGTCATACGTTCCCGAAGATAAGAAGCC

CCACCACGAAAGTGACTTTATATTACCCGACCCCACGAAAGCTAAGAAA

>267_Sample_ASV7

CACCGCGGTTATACGAGAGGCCCAAGTTGACAGACACCGGCGTAAAGAGTGGCTAGGGAA

AAATTACTACTAAAGCCGAACACCTTCAGAACTGTCATACGTTCCCGAAGATAAGAAGCC

CCACCACGAAAGTGACTTTATATTACCCGACCCCACGAAAGCTGTGAAA

>267_Sample_ASV8

CATGGCGATTATACGAGAGGCCCAAGTTGACAGACACTGGCGTAAAGAGTGGCTAAGGAA

AAACTACTACTAAAGCCGAACACCTTCAGAACTGTCATACGTTCCCGAAGATAAGAAGCC

CCACCACGAAAGTGGCTTTATATTACCCGACCCCACGAAAGCTGTGAAA

>267_Sample_ASV9

CACCGCGGTTATACGAGAGGCCCAAGTTGACAGACACCGGCGTAAAGAGTGGCTAGGGAA

AAATTACTACTAAAGCCGAACACCCTCAGAACTGTCATACGTTCCCGAAGGTAAGAAGCC

CCACCACGAAAGTGGCTTTATAATACCCGACCCCACGAAAGCTGTGAAA

>267_Sample_ASV10

CACCGCGGTTATACGAGAGGCCCAAGTTGACAGACACCGGCGTAAAGAGTGGCTAGGGAA

AAATTACTACTAAAGCCGAACACCTTCAGAACTGTCATACGTTCCCGAAGATAAGAAGCC

CCACCACGAAAGTGGCTTTATATTACCCGACCCCACGAAAGCTGTGAAA

>267_Sample_ASV11

CACCGCGGTTATACGAAAGACCCTAGTTTATAGAAACGGCGTAAAGGGTGGTTAAGGGAC

AAACAAAATAAGGCCAAAGACTTCCTAAGCCGTCATACGCTCCACGAAAGCACGAAGCCC

AAACACGAAAGTAGCCTTAATAACTTCACCTGACCCCACGAAAGCTGGGAGA

>267_Sample_ASV12

CACCGCGGTTATACGAGAGACCCTAGTTGATATATACGGCGTAAAGAGTGGTTTGGGATA

TTTCTCCAAAATAAAGCCGAAGATCTCCCAAGCCGTCACACGCATCCCGGAGACACGAAG

CCCTGATACGAAGGTAGCTTTATTAACACCCCGACGCCACGAAAGCTAAGAAA

>267_Sample_ASV13

CACCGCGGTTATACGAGAGACCCTAGTTGATATATACGGCGTAAAGAGTGGTTTGGGATA

TTTCTCCAAAATAAAGTCGAAGATCTCCCAAGCCGTCACACGCATCCCGGAGACACGAAG

CCCTGATACGAAGGTAGCTTTATTAACACCCCGACGCCACGAAAGCTAAGAAA

>267_Sample_ASV14

CACCGCGGTTATACGAGAGGCCCAAGTTGATAAGCCCGGCGTAAAGAGTGGTTATGGAGA

ATAAAACACTAAAGCTAAAGACCCCCTAGGCTGTTATACGCACCTGGCGGCTCGAACCAC

CTATACGAAAGTAGCTTTACCCCCCTTCCACCAGAATCCACGACAGCTGGGGCA

>267_Sample_ASV15

CACCGCGGTTATACGAGAGGCCCAAGTTGACAGACATCGGCGTAAAGAGTGGTTAAGGAA

ACCCCATTACTAAAGCCGAACGCCCTCAGAACTGTCATACGTTCCCGAAGGTAAGAAGCC

CCACTACGAAAGTGGCTTTAACCACACCCGACCCCACGAAAGCTAAGAAA

>267_Sample_ASV16

CACCGCGGTTATACGAGAGGCCCAAGTTGACAGACATCGGCGTAAAGAGTGGTTAAGGAA

ACCCCATTACTAAAGCCGAACGCCCTCAGAACTGTCATACGTTCCCGAAGGTAAGAAGCC

CCACTACGAAAGTGGCTTTAACCACACCCGACCCCACGAAAGCTGTGAAA

>267_Sample_ASV17

CACCGCGGTTATACGAGAGGCCCAAGTTGATAGATATCGGCGTAAAGAGTGGTTAAGGAA

ACCCCGTTACTAAAGCCGAACGCCCTCAGAACTGTCATACGTTCCCGAAGGTAAGAAGCC

CCACTACGAAAGTGGCTTTAACCACACCCGACCCCACGAAAGCTGTGAAA

>267_Sample_ASV18

CACCGCGGTTATACGAGAGGCCCAAGTTGACAGACACCGGCGTAAAGAGTGGTTAAGGAA

ATTCATCAACTAAAGCCGAATGCCCTCAGGACTGTTATACGTTTGCGAAGGTAAGAAGCC

CTACTACGAAAGTGGCTTTATTCCACCTGATTCCACGAAAGCTAAGAAA

>267_Sample_ASV19

CACCGCGGTTATACGAGAGGCCCAAGTTGACAGACACCGGCGTAAAGAGTGGTTAAGGAA

ATTCATCAACTAAAGCCGAATGCCCTCAGGACTGTTATACGTTTGCGAAGGTAAGAAGCC

CTACTACGAAAGTGGCTTTATTCCACCTGATTCCACGAAAGCTGCGGAA

>267_Sample_ASV20

CACCGCGGTTATACGAGAGGCCCCAGTTGATATTAGCGGCGTAAAGGGTGGTTAAGGACT

TTAATAACTAAAGCTAAACACTTCCCCGGCTGTTGTACGTAGACCGGAAGCTTGAAGCCC

AGACACGAAAGTAGCTTTATTCCCCCTGACCCCACGAAAGCTGAGAAA

>267_Sample_ASV21

CACCGCGGTTATACGAGAAACCCTAGTTGACAAATACGGCGTAAAGAGTGGTTAAGGGGT

ATCAAAAATAAAGTCAAACATTTTCCTAGTTGTTATAAGCATTTCGAGAACATGAAGCCC

AACCACGAAAGTAACTTTACCTTCACCTGACCCCACGAAAGCTAAGAAA

>267_Sample_ASV22

CACCGCGGTTATACGAGAGACCCTAATTGATAGGTACGGCGTAAAGAGTGGTTTGGGATA

ACCCAAAAATAAAGCTAAAGACCTTCTAAGCCGTCACACGCATACCGAAGGCACGAGGCC

CCAACACGAAAGTAGCTTTACTATTACCCCGACGCCACGAAAGCTAAGAAA

>267_Sample_ASV23

CACCGCGGTTATACGAGAGGCCCTAGTTGATAACTACCGGCGTAAAGAGTGGTTATGGAA

AAATATTTAATAAAGCCGAACACCCCCTCAGCCGTCATACGCACCTGGGAGCACGAAGAC

CTACTGCGAAAGCAGCTTTAATTATGCCTGACCCCACGACAGCTAAGAAA

>267_Sample_ASV24

CACCGCGGTTATACGAGAGGCCCAAGTTGATACTCCACGGCGTAAAGAGTGGTTAGAAAA

GACCTACTACTAAAGCCGAACGCCTTCAAAGCTGTTATACGCATCCGAAGGTGAGAAGCC

CACCCACGAAAGTGGCTTTACAACCTTGACTCCACGAAAGCTATGACA

>267_Sample_ASV25

CACCGCGGTTATACGAGAGACCCCAGTTGATAAACACGGCGTAAAGAGTGGTTAAGGGCA

CAAAACAAATAAAGTCAAACAACCTCTTAGCTGTTATACGCATTATGAGGATATGAAGCC

CCCCTACGAAAGTAACTTTACCAACCCCTGACCCCACGAAAGCTAAGAAA

>267_Sample_ASV26

CACCGCGGTTATACGAGAGACCCTAGTTGATATATACGGCGTAAAGAGTGGTTTGGAATA

ACCAAGTAATATAGCCAAAGACCTCCCAAGCCGTCACACGCACCCCGGAGGCACGAAGCC

CAAACACGAAAGTAGCTTTATTAACTTACCGACGCCACGAAAGCTAAGAAA

>267_Sample_ASV27

CACCGCGGTTATACGAAAGACCCTAGTTTATAGGCACGGCGTAAAGGGTGGTTAGGGGAT

AAACAAAATAAAGCCAAAGACCTTCTAAGTCGTCATACACTCATGAAGGCACGAAGCCCA

AATACGAAAGTAACTTTACAACTTAGACCCGACTCCACGAAAGCTGGGGGA

>267_Sample_ASV28

CACCGCGGTTATACGAGAGACCCTAATTAATAGATACGGCGTAAAGAGTGGTTTAGGACA

AAAATTAATAAAGCCGAAGTTTCTCCTAGCCGTTGTACGCACTCTGAGAACACGAAGCCC

CACTACGAAAGTAGCTTTACCTATTATTTCCTGACCCCACGAAAGCTAAGAAA

>267_Sample_ASV29

CACCGCGGTTATACGAGAGACTCCAGTTGATAGCTACGGCGTAAAGAGTGGTTTGGGGCC

CGCCCAAAATAAAGCCAAAGACCTCCCAAGCCGTCAAACGCACCCCGGAGGCACGAAGTC

CTAACGCGAAAGCAACTTTACCTTCCCCGACGCCACGAAAGCTAAGAAA

>267_Sample_ASV30

CACCGCGGTTATACGAAAGACCCTAGTTGATAGATCACGGCGTAAAGGGTGGTTAAGGAG

AACAAAAATAAAGCTAAAAATCTTCTAAGCTGTCATACGCTTTCCGAAGACATGAGACCC

AACCACGAAAGTAGCTTTAAACTTTCCTGACACCACGAAAGCCAAGAAA

>268_Sample_ASV1

TACCGCGGTTATACGAAAGACCCAAGTTAATAAACTACGGCGTAAAGCGTGGTTAGAGAC

CTCCAATAATTAAAGCCAAAGACCTTCTAAGCCGTCGCACGCCCCGCGAAGACACGAAGC

CCAAAAACGAAAGTAGCTTTATTCTTACTCGACCCCACGAAAGCTAGGGCA

>268_Sample_ASV2

CACCGCGGTTATACGAAAGACCCTAGTTGATGGGTGCGGCGTAAAGGGTGGTTAGGGACT

ACAAAAAATAGAGCTAAAGATCCTCTAGGTCGTCATACACATCACGAGGAAACGAAGCCC

AAATACGAAAGTAGCTCTAAATATAATCCTGACGCCACGAAAGCCAAGAAA

>268_Sample_ASV3

CACCGCGGTTATACGAAAGACCCTAGTTGATAACTACGGCATAAAGGGTGGTTAGGGAGT

TTAAAAAATAAAGCCAAAGCTCTTCTAAGCTGTCATACGCTCTCTGAAAGAACGAGGCCC

AAACACGAAAGTAGCTTTAAATAAATAACCTGATACCACGAAAGCCAAGAAA

>268_Sample_ASV4

CACCGCGGTTATACGAGAGACCCTAGTTGATTAAAGCGGCGTAAAGAGTGGTTATGGAAC

TATTCTTTTAAAGCAGAAAACCTCTCAAACTGTTATACGCACCCAGAGGTCTGAATCCCT

CACACGAAAGTGACTTTATTTATGCCTACCAGAACCCACGAAAGCTGGGACA

>268_Sample_ASV5

CACCGCGGTTATACGAGAGGCCCAAGTTGACAGACACCGGCGTAAAGAGTGGCTAGGGAA

AAATTACTACTAAAGCCGAACACCTTCAGAACTGTCATACGTTCCCGAAGATAAGAAGCC

CCACCACGAAAGTGACTTTATATTACCCGACCCCACGAAAGCTGGGGGA

>268_Sample_ASV6

CACCGCGGTTATACGAGAGGCCCAAGTTGACAGACACCGGCGTAAAGAGTGGCTAGGGAA

AAATTACTACTAAAGCCGAACACCTTCAGAACTGTCATACGTTCCCGAAGATAAGAAGCC

CCACCACGAAAGTGACTTTATATTACCCGACCCCACGAAAGCTGTGAAA

>268_Sample_ASV7

CACCGCGGTTATACGAGAGGCCCAAGTTGACAGACACCGGCGTAAAGAGTGGCTAGGGAA

AAATTACTACTAAAGCCGAACACCTTCAGAACTGTCATACGTTCCCGAAGATAAGAAGCC

CCACCACGAAAGTGACTTTATATTACCCGACCCCACGAAAGCTAAGAAA

>268_Sample_ASV8

CACCGCGGTTATACGAGAGGCCCAAGTTGACAGACACCGGCGTAAAGAGTGGCTAGGGAA

AAATTACTACTAAAGCCGAACACCTTCAGAACTGTCATACGTTCCCGAAGATAAGAAGCC

CCACCACGAAAGTGGCTTTATATTACCCGACCCCACGAAAGCTGTGAAA

>268_Sample_ASV9

CACCGCGGTTATACGAGAGGCCCAAGTTGACAGACACCGGCGTAAAGAGTGGCTAAGGAA

AAATTTCTACTAAAGCCGAACACCTTCAGAACTGTCATACGTTCCCGAAGAAAAGAAGCC

CCACCACGAAAGTGGCTTTATATTACCCGACCCCACGAAAGCTGTGAAA

>268_Sample_ASV10

CACCGCGGTTATACGAAAGACTCTAGTTGACAAACACGGCGTAAAGGGTGGTTAGGATAA

CAACAAAATAAAGCTAAAGACTTACCAAGCCGTCATACGCCCATGTAAGAGCGAAAACCA

CAAACGAAAGTAACTTTATTACACCATCCGAATCCACGAAAGCTAAGAAA

>268_Sample_ASV11

CACCGCGGTTATACGAAAGACCCTAGTTGATAGATCACGGCGTAAAGGGTGGTTAGGGAA

TACAAAAATAAAGCTAAAAATCCTCTAAGCTGTCATACGCTTCCCGAAGACATGAAACCC

AACCACGAAAGTAGCTTTAAACTCTCCTGACACCACGAAAGCCAAGGAA

>268_Sample_ASV12

CACCGCGGTTATACGAGAGGCCCAAGTTGATAAGCCCGGCGTAAAGAGTGGTTATGGAGA

ATAAAACACTAAAGCTAAAGACCCCCTAGGCTGTTATACGCACCTGGCGGCTCGAACCAC

CTATACGAAAGTAGCTTTACCCCCCTTCCACCAGAATCCACGACAGCTGGGGCA

>268_Sample_ASV13

CACCGCGGTTATACGAGAGGCCCAAGTTGACAGACATCGGCGTAAAGAGTGGTTAAGGAA

ACCCCATTACTAAAGCCGAACGCCCTCAGAACTGTCATACGTTCCCGAAGGTAAGAAGCC

CCACTACGAAAGTGGCTTTAACCACACCCGACCCCACGAAAGCTGTGAAA

>268_Sample_ASV14

CACCGCGGTTATACGAGAGGCCCCAGTTGATATTAGCGGCGTAAAGGGTGGTTAAGGACT

TTAATAACTAAAGCTAAACACTTCCCCGGCTGTTGTACGTAGACCGGAAGCTTGAAGCCC

AGACACGAAAGTAGCTTTATTCCCCCTGACCCCACGAAAGCTGAGAAA

>268_Sample_ASV15

CACCGCGGTTATACGAGAGGCCCCAGTTGATATTAGCGGCGTAAAGGGTGGTTAAGGACT

TTAATAACTAAAGCCAAACACTTCCCCGGCTGTTGTACGTAGACCGGAAGCTTGAAGCCC

AGACACGAAAGTAGCTTTATTTCCCCTGACCCCACGAAAGCTGAGAAA

>268_Sample_ASV16

CACCGCGGTTATACGAGAGGCCCCAGTTGATATTAGCGGCGTAAAGGGTGGTTAAGGACT

TTAATAACTAAAGCTAAACACTTCCCCGGCTGTTGTACGTAGACCGGAAGCTTGAAGCCC

AGACACGAAAGTAGCTTTATTTCCCCTGACCCCACGAAAGCTGAGAAA

>268_Sample_ASV17

CACCGCGGTTATACGAAAGACCCCAGTTTATAGACACGGCGTAAAGGGTGGTTATAGGAC

AAACTAACATAGAGCCAAAGACCCCCTAAGCCGTCATACGCTCACGAGGGCTCGAAGCCC

CTATACGAAAGTAGCTCTACCAAAAAATGCTCGACTCCACGAAAGCTGGGACA

>268_Sample_ASV18

CACCGCGGTTATACGAGAGACCCTAATTGATAGGCACGGCGTAAAGAGTGGTTTAGGGTA

ACCCATAAATAAAGCTAAAGACCTTCTAAGCCGTCACACGCACACCGAAGGCACGAGACC

CCAACACGAAAGTAGCTTTACTTCTACCCCTGACGCCACGAAAGCTAAGAAA

>268_Sample_ASV19

CACCGCGGTTATACGAAAGACCCTAGTTGATGGATCACGGCGTAAAGGGTGGTTAAGGGA

TATAAAAATAAAGCTAAAAATCCTCTTAGCTGTCATACGCTTTCCGAAGACATGAGGCCC

AATTACGAAAGTAGCTTTAAACCACCCTGACACCACGAAAGCCAAGAAA

>268_Sample_ASV20

CACCGCGGTTATACGAAAGACCCCAGTTTATAGATGCGGCGTAAAGGGTGGTTAAGAGAA

AATCAAAATAAGGCCAAAGATCCCCTAAGCAGTCATACGCTCCACGAGGACACGAAACCC

AAATACGAAAGTTGCCTTAATAAACTTCACCTGATCCCACGAAAGCTGAAAAA

>268_Sample_ASV21

CACCGCGGTTATACGAAAGGCTCTAGTTTATAAATACGGCGTAAAGGGTGGTTAAGGAAA

AATTAAAATAATGCTAAAGACCCCCTAAGCAGTTATACGCTTCACGGAGGCACGAAACCC

AAATACGAAAGTTGCCTTAATAAACTTTACCTGACCCCACGAAAGCTGAAAAA

>268_Sample_ASV22

CACCGCGGTTATACGAAAGGCTCTAGTTTATAAATACGGCGTAAAGGGTGGTTAAGGAAA

AATTAAAATAATGCTAAAGACCCCCTAAGCAGTTATACGCTTCACGGAGGCACGAAACCC

AAATACGAAAGTTGCCTTAATAAACTTTACCTGACCCCACGAAAGCTGAGAAA

>268_Sample_ASV23

CACCGCGGTTATACGAGAGACTCTAGTTGATACCAACGGCGTAAAGAGTGGTTTGGGATG

CCCCTTAAAATAAAGCCAAAGACCTCCCAAGCCGTCAAACGCACCCCGGAGGCACGAAGC

CCCAACGCGAAAGCAACTTTACCTCCCTCCCGACGCCACGAAAGCTAAGAAA

>268_Sample_ASV24

CACCGCGGTTATACGAGAGACCCTAGTTGATAGCTACGGCGTAAAGCGTGGTTAGGAGAA

CAAGATAAATAAAGTCAAACAATCTCTCGGCCGTTATACGTTATCTGAGAATATGAAGTC

CTACCACGAAAGTAACTTTAATTTTTCTGACCCCACGAAAACTGAGGAA

>268_Sample_ASV25

CACCGCGGTTATACGAGAGGCCCTAGTTGATAACTACCGGCGTAAAGAGTGGTTATGGAA

AAATATTTAATAAAGCCGAACACCCCCTCAGCCGTCATACGCACCTGGGAGCACGAAGAC

CTACTGCGAAAGCAGCTTTAATTATGCCTGACCCCACGACAGCTAAGAAA

>268_Sample_ASV26

CACCGCGGTTATACGAAAGACCCTAGTTTATAGACACGGCGTAAAGGGTGGTTCGGGACT

AAACAAAATAAAGCCAAAGACCTCCTAAGCCGTCATACGCTCACGGAAGCACGAAGCCCA

AGTACGAAAGTAGCTTTACAAAACATGCCTGACTCCACGAAAGCTAGGAAA

>268_Sample_ASV27

CACCGCGGTTATACGAGAGGCCCAAGTTGATTATTACCGGCGTAAAGCGTGGTTAAGATT

TTTATAGGACTAAAGCCGAACCCCCTCAGAGCTGTTATACGCACCCGAAGGTTAGAAGTT

CAATCACGAAAGTGGCTTTACAACCCCGAACCCACGAAAGCTAAGATA

>268_Sample_ASV28

CACCGCGGTTATACGAGAGGCCCAAGTTGATACTCCACGGCGTAAAGAGTGGTTAGAAAA

GACCTACTACTAAAGCCGAACGCCTTCAAAGCTGTTATACGCATCCGAAGGTGAGAAGCC

CACCCACGAAAGTGGCTTTACAACCTTGACTCCACGAAAGCTATGACA

>268_Sample_ASV29

CACCGCGGTTATACGAGAGACCCCAGTTGATAAACACGGCGTAAAGAGTGGTTAAGGGCA

CAAAACAAATAAAGTCAAACAACCTCTTAGCTGTTATACGCATTATGAGGATATGAAGCC

CCCCTACGAAAGTAACTTTACCAACCCCTGACCCCACGAAAGCTAAGAAA

>268_Sample_ASV30

CACCGCGGTTATACGAAAGACCCTAGTTTATAGGCACGGCGTAAAGGGTGGTTAGGGGAT

AAACAAAATAAAGCCAAAGACCTTCTAAGTCGTCATACACTCATGAAGGCACGAAGCCCA

AATACGAAAGTAACTTTACAACTTAGACCCGACTCCACGAAAGCTGGGGGA

>268_Sample_ASV31

CACCGCGGTTATACGAAAGACCCTAGTTTATAGGCACGGCGTAAAGGGTGGTTAGGGGAT

AAACAAAATAAAGCCAAAGACCTTCTAAGTCGTCATACACTCATGAAGGCACGAAGCCCA

AATACGAAAGTAACTTTACAACTTAGACCCGACCCCACGAAAGCTGTGAAA

>268_Sample_ASV32

CACCGCGGTTATACGAGAGGCCCAAGTTGATAGACATCGGCGTAAAGAGTGGTTAAGGAA

GTCTCAAAACTAAAGCCGAATGCCTTCAAGACTGTTATACGTTTCCGAAGGTAAGAAGCC

CCACTACGAAAGTGGCTTTACTCCACCTGACTCCACGAAAGCTGTGAAA

>268_Sample_ASV33

CACCGCGGTTATACGAAAGACCCTAGTTGATAGCTACGGCGTAAAGGGTGGTTAAGGTAT

ATAACAAATAAAGCTAAAGAGCCTCCAAGCCGTCGTACGCATTCTGAGAGCTCGAAACCC

TAACACGAAAGTAGCTTTAAAAAATAACACCTGACCCCACGAAAGCTAAGAAA

>268_Sample_ASV34

CACCGCGGTTATACGAAAGACCCTAGTTGATAGACCACGGCGTAAAGGGTGGTTAAGGAA

CACAAAAATAAAGCTAAAGATCCTCTAAGCTGTCATACGCTTCCCGAAGACATGAAACCC

AATCACGAAAGTAGCTTTAAACTGTCCTGACACCACGAAAGCCAAGAAA

>268_Sample_ASV35

CACCGCGGTTATACGAAAGACCCTAGTTGATAGATCACGGCGTAAAGGGTGGTTAAGGAG

AACAAAAATAAAGCTAAAAATCTTCTAAGCTGTCATACGCTTTCCGAAGACATGAGACCC

AACCACGAAAGTAGCTTTAAACTTTCCTGACACCACGAAAGCCAAGAAA

>270_Sample_ASV1

CACCGCGGTTATACGAGAGGCCCAAGTTGACAGACATCGGCGTAAAAAGTGGTTAAGGAA

AACCTTCAACTAAAGTCGAACGCTCTCAAGACTGTCGTACGTTTCCGAGAGTATGAAGCC

CCACCACGAAAGTGACTTTACCTCACCTGACTCCACGAAAGCTGCGAAA

>270_Sample_ASV2

CACCGCGGTTATACGAGAGGCCCAAGTTGACAGACATCGGAGTAAAAAGTGGTTAAGGAA

AACCTTCAACTAAAGTCGAACGCTCTCAAGACTGTCGTACGTTTCCGAGAGTATGAAGCC

CCACCACGAAAGTGACTTTACCTCACCTGACTCCACGAAAGCTGCGAAA

>270_Sample_ASV3

CACCGCGGTTATACGAAAGACCCTAGTTGATGGGTGCGGCGTAAAGGGTGGTTAGGGACT

ACAAAAAATAGAGCTAAAGATCCTCTAGGTCGTCATACACATCACGAGAAAACGAAGCCC

AAATACGAAAGTAGCTCTAAATATAATCCTGACGCCACGAAAGCCAAGAAA

>270_Sample_ASV4

CACCGCGGTTATACGAAAGACCCTAGTTGATGGGTGCGGCGTAAAGGGTGGTTAGGGACT

ACAAAAAATAGAGCTAAAGATCCTCTAGGTCGTCATACACATCACGAGGAAACGAAGCCC

AAATACGAAAGTAGCTCTAAATATAATCCTGACGCCACGAAAGCCAAGAAA

>270_Sample_ASV5

CACCGCGGTTATACGAAAGACCCTAGTTGATAACTACGGCATAAAGGGTGGTTAGGGAGT

TTAAAAAATAAAGCCAAAGCTCTTCTAAGCTGTCATACGCTCTCTGAAAGAACGAGGCCC

AAACACGAAAGTAGCTTTAAATAAATAACCTGATACCACGAAAGCCAAGAAA

>270_Sample_ASV6

CACCGCGGTTATACGAGAGACCCTAGTTGATTAAAGCGGCGTAAAGAGTGGTTATGGAAC

TATTCTTTTAAAGCAGAAAACCTCTCAAACTGTTATACGCACCCAGAGGTCTGAATCCCT

CACACGAAAGTGACTTTATTTATGCCTACCAGAACCCACGAAAGCCAAGAAA

>270_Sample_ASV7

CACCGCGGTTATACGAGAGACCCTAGTTGATTAAAGCGGCGTAAAGAGTGGTTATGGAAC

TATTCTTTTAAAGCAGAAAACCTCTCAAACTGTTATACGCACCCAGAGGTCTGAATCCCT

CACACGAAAGTGACTTTATTTATGCCTACCAGAACCCACGAAAGCTGGGACA

>270_Sample_ASV8

CACCGCGGTTATACGAGAGGCCCAAGTTGACAGACACCGGCGTAAAGAGTGGCTAGGGAA

AAATTACTACTAAAGCCGAACACCTTCAGAACTGTCATACGTTCCCGAAGATAAGAAGCC

CCACCACGAAAGTGGCTTTATATTGTCCTGACACCACGAAAGCCAAGAAA

>270_Sample_ASV9

CATGGCGATTATACGAGAGGCCCAAGTTGACAGACACTGGCGTAAAGAGTGGCTAAGGAA

AAATTACTACTAAAGCCGAACACCTTCAGAACTGTCATACGTTCCCGAAGATAAGAAGCC

CCACCACGAAAGTGGCTTTATATTACCCGACCCCACGAAAGCTGTGAAA

>270_Sample_ASV10

CACCGCGGTTATACGAGAGGCCCAAGTTGACAGACACCGGCGTAAAGAGTGGCTAGGGAA

AAATTACTACTAAAGCCGAACACCTTCAGAACTGTCATACGTTCCCGAAGATAAGAAGCC

CCACCACGAAAGTGGCTTTATATTACCCGACACCACGAAAGCCAAGAAA

>270_Sample_ASV11

CACCGCGGTTATACGAGAGGCCCAAGTTGACAGACACCGGCGTAAAGAGTGGCTAGGGAA

AAATTACTACTAAAGCCGAACACCTTCAGAACTGTCATACGTTCCCGAAGATAAGAAGCC

CCACCACGAAAGTGGCTTTATATTACCCGACCCCACGAAAGCCAAGAAA

>270_Sample_ASV12

CACCGCGGTTATACGAGAGGCCCAAGTTGACAGACACCGGCGTAAAGAGTGGCTAGGGAA

AAATTACTACTAAAGCCGAACACCTTCAGAACTGTCATACGTTCCCGAAGATAAGAAGCC

CCACCACGAAAGTGGCTTTATATTACCCGACCCCACGAAAGCTGTGAAA

>270_Sample_ASV13

CATGGCGATTATACGAGAGGCCCAAGTTGACAGACACTGGCGTAAAGAGTGGCTAAGGAA

AAACTACTACTAAAGCCGAACACCTTCAGAACTGTCATACGTTCCCGAAGATAAGAAGCC

CCACCACGAAAGTGGCTTTATATTACCCGACCCCACGAAAGCTGTGAAA

>270_Sample_ASV14

CACCGCGGTTAAACGAGAGACCCTAGTTGATCATCACGGCGTAAAGAGTGGTTAGGGGAT

TATCATAAATAAAGCCAAAGACCTCCCAAGCTGTCGCACGCATTCCGGAGGCGCGAAGCC

CACACACGAAAGTAGCTTTAACTATTGACCCTGATGCCACGAAAGCTAAGAAA

>270_Sample_ASV15

CGCCGCGGTTAAACGAGAGGCCCTAGTTGATGGAACAACGGCGTAAAGGGTGGTTAAGGA

TAGTGAGACAATAAAGTCGAATGGCCCTTTGGCTGTCATACGCTTCTGGGAGCCTGAAGC

CCACTATACGAAAGTAGCTTTAACAACGTCCACCTGACCCCACGAAAACTGAGAAA

>270_Sample_ASV16

CACCGCGGTTATACGAAAGACCCTAGTTGATAGATCACGGCGTAAAGGGTGGTTAGGGAA

TACAAAAATAAAGCTAAAAATCCTCTAAGCTGTCATACGCTTCCCGAAGACATGAAACCC

AACCACGAAAGTAGCTTTAAACTCTCCTGACACCACGAAAGCCAAGGAA

>270_Sample_ASV17

CACCGCGGTTATACGAGAGGCCCAAGTTGATAAGCCCGGCGTAAAGAGTGGTTATGGAGA

ATAAAACACTAAAGCTAAAGACCCCCTAGGCTGTTATACGCACCTGGCGGCTCGAACCAC

CTATACGAAAGTAGCTTTACCCCCCTTCCACCAGAATCCACGACAGCTGGGGCA

>270_Sample_ASV18

CACCGCGGTTATACGAGAGGCCCAAGTTGACAGACATCGGCGTAAAGAGTGGTTAAGGAA

ACCCCATTACTAAAGCCGAACGCCCTCAGAACTGTCATACGTTCCCGAAGGTAAGAAGCC

CCACTACGAAAGTGGCTTTAACCACACCCGACCCCACGAAAGCTAAGAAA

>270_Sample_ASV19

CACCGCGGTTATACGAGAGGCCCAAGTTGACAGACATCGGCGTAAAGAGTGGTTAAGGAA

ACCCCATTACTAAAGCCGAACGCCCTCAGAACTGTCATACGTTCCCGAAGGTAAGAAGCC

CCACTACGAAAGTGGCTTTAACCACACCCGACCCCACGAAAGCTGTGAAA

>270_Sample_ASV20

CACCGCGGTTAAACGAGAGGCCCTAGTTGATATTACAACGGCGTAAAGGGTGGTTAAGGA

AAGCAAGCAATAAAGCCGAATGGCCCTTTGGCTGTCATACGCTTCTAGGTGTCCGAAGCC

CAATATACGAAAGTAGCTTTAGAAAAGCCCACCTGACACCACGAAAGCTGAGGAA

>270_Sample_ASV21

CACCGCGGTTATACGAGAGGCCCCAGTTGATATTAGCGGCGTAAAGGGTGGTTAAGGACT

TTAATAACTAAAGCTAAACACTTCCCCGGCTGTTGTACGTAGACCGGAAGCTTGAAGCCC

AGACACGAAAGTAGCTTTATTCCCCCTGACCCCACGAAAGCTGAGAAA

>270_Sample_ASV22

CACCGCGGTCACACGATTAACCCAAGTCAATAGAAGCCGGCGTAAAGAGTGTTTTAGATC

ACCCCCTCCCCAATAAAGCTAAAACTCACCTGAGTTGTAAAAAACTCCAGTTGACACAAA

ATAGACTACGAAAGTGGCTTTAACATATCTGAACACACAATAGCTAAGACC

>270_Sample_ASV23

CACCGCGGTTATACGAGAGGCCCTAGTTGATAAATACGGCGTAAAGAGTGGTTAGAGGAC

TCCCATAATAAAGCCAAAGACCCTCCGGGCCGTCACACGCATACGAGGGCACGAAGTTCA

CACACGAAAGTAGCTTTAAATTACCCGACGCCACGAAAGCTAAGAAA

>270_Sample_ASV24

CACCGCGGTTATACGAAAGACCCTAGTTTATAAGTACGGCGTAAAGGGTGGTTAGGGGAC

AAACAAAATAAAGCCAAAGACCTCCTAAGCCGTCATACGCTTACGGAGGCACGAAGCCCA

AACACGAAAGTAGCTTTACCGAACATGCCCGACTCCACGAAAGCTGGGAAA

>270_Sample_ASV25

CACCGCGGTTATACGAAAGACCCTAGTTTATAAGTACGGCGTAAAGGGTGGTTAGGGGAC

AAACAAAATAAAGCCAAAGACCTCCTAAGCCGTCATACGCTTACGGAGGCACGAAGCCCA

AACACGAAAGTAGCTTTACCGAACATGCCCGACTCCACGAAAGCTAAGAAA

>270_Sample_ASV26

CACCGCGGTTATACGAGAGACCCTAGTTGATAAATACGGCGTAAAGAGTGGTTATGGGAA

AAACAAATAAAGTCAAACAACCTCTTAGCTGTTATACGCATTATGAGAGCATGAAGCCCC

CTCACGAAAGTAACTTTAATACCTCCTGACCCCACGAAAGCTAAGAAA

>270_Sample_ASV27

CACCGCGGTTATACGAGAGGCTCAAGTTGATAAACCCCGGCGTAAAGAGTGGTTAAGGAA

AAATAGAAACTAAAGCCGAATGCTTTCAAGACTGTTATACGTTCCCGAGAGTAAGAAGAC

CAATTACGAAAGTGGCTTTACTTTACCTGACCCCACGAAAGCTACGACA

>270_Sample_ASV28

AACCGCGGTTATACGAGAGACCCTAATTGATAGCTCACGGCGTAAAGGGTGGTTTGGGGA

AACCTCAAAATAAAGCTAAAGACCTTCTAAGCCGTTACACGCATACCGAAGGCACGAGGC

CCTAACACGAAAGTAGCTTTACTATTACCCCTGACGCCACGAAAGCTAAGAAA

>270_Sample_ASV29

CACCGCGGTTAAACGAGAGGCCCTAGTTGATAGTACAACGGCGTAAAGGGTGGTTAAGGA

TAGTGAAATAATAAAGTCGAATGGCCCTTTGGCTGTCATACGCTTCTAGGAGTCCGAAGC

CCAATATACGAAAGTAACTTTAAGAGCGCCCACCTGACCCCACGAAAACTGAGAAA

>270_Sample_ASV30

CACCGCGGTTATACGAAAGACCCTAGTTTATAGACACGGCGTAAAGGGTGGTTCGGGACT

AAACAAAATAAAGCCAAAGACCTCCTAAGCCGTCATACGCTCACGGAAGCACGAAGCCCA

AGTACGAAAGTAGCTTTACAAAACATGCCTGACTCCACGAAAGCTAGGAAA

>270_Sample_ASV31

CACCGCGGTTATACGAAAGACCCTAGTTGATAGCCACGGCGTAAAGGGTGGTTAAGGTAT

ACAACAAATAAAGCTAAATAGCCTCTAGGCCGTCGCACGCATTCCGAGAGCTCGAAACCC

AAAAACGAAAGTAGCTTTAAAATAAACAGACCTGACCCCACGAAAGCTAAGAAA

>270_Sample_ASV32

CACCGCGGTTATACGAGAGGCCCAAGTTGATACTCCACGGCGTAAAGAGTGGTTAGAAAA

GACCTACTACTAAAGCCGAACGCCTTCAAAGCTGTTATACGCATCCGAAGGTGAGAAGCC

CACCCACGAAAGTGGCTTTACAACCTTGACTCCACGAAAGCTATGACA

>270_Sample_ASV33

CACCGCGGTTATACGAGAGGCCCAAGTTGATACTCCACGGCGTAAAGAGTGGTTAGAAAA

GACCTACTACTAAAGCCGAACGCCTTCAAAGCTGTTATACGCATCCGAAGGTGAGAAGCC

CACCCACGAAAGTGGCTTTACAACCTTGACTCCACGAAAGCCAAGAAA

>270_Sample_ASV34

CACCGCGGTTATACGAGAGACCCCAGTTGATAAACACGGCGTAAAGAGTGGTTAAGGGCA

CAAAACAAATAAAGTCAAACAACCTCTTAGCTGTTATACGCATTATGAGGATATGAAGCC

CCCCTACGAAAGTAACTTTACCAACCCCTGACCCCACGAAAGCTAAGAAA

>270_Sample_ASV35

CACCGCGGTTATACGAGAGACCCTAGTTGATATATACGGCGTAAAGAGTGGTTTGGAATA

ACCAAGTAATATAGCCAAAGACCTCCCAAGCCGTCACACGCACCCCGGAGGCACGAAGCC

CAAACACGAAAGTAGCTTTATTAACTTACCGACGCCACGAAAGCTAAGAAA

>270_Sample_ASV36

CACCGCGGTTATACGAAAGACCCTAGTTTATAGGCACGGCGTAAAGGGTGGTTAGGGGAT

AAACAAAATAAAGCCAAAGACCTTCTAAGTCGTCATACACTCATGAAGGCACGAAGCCCA

AATACGAAAGTAACTTTACAACTTAGACCCGACTCCACGAAAGCTGGGGGA

>270_Sample_ASV37

CACCGCGGTTATACGAGAGGCCCAAGTTGATAGACATCGGCGTAAAGAGTGGTTAAGGAA

GTCTCAAAACTAAAGCCGAATGCCTTCAAGACTGTTATACGTTTCCGAAGGTAAGAAGCC

CCACTACGAAAGTGGCTTTACTCCACCTGACTCCACGAAAGCTGTGAAA

>270_Sample_ASV38

CACCGCGGTTATACGAAAGACCCTAGTTGATAGACCACGGCGTAAAGGGTGGTTAAGGAA

CACAAAAATAAAGCTAAAGATCCTCTAAGCTGTCATACGCTTCCCGAAGACATGAAACCC

AATCACGAAAGTAGCTTTAAACTGTCCTGACACCACGAAAGCCAAGAAA

>270_Sample_ASV39

CACCGCGGTTATACGAAAGACCCTAGTTGATAGATCACGGCGTAAAGGGTGGTTAAGGAG

AACAAAAATAAAGCTAAAAATCTTCTAAGCTGTCATACGCTTTCCGAAGACATGAGACCC

AACCACGAAAGTAGCTTTAAACTGTCCTGACACCACGAAAGCCAAGAAA

>270_Sample_ASV40

CACCGCGGTTATACGAAAGACCCTAGTTGATAGATCACGGCGTAAAGGGTGGTTAAGGAG

AACAAAAATAAAGCTAAAAATCTTCTAAGCTGTCATACGCTTTCCGAAGACATGAGACCC

AACCACGAAAGTAGCTTTAAACTTTCCTGACACCACGAAAGCCAAGAAA

>271_Sample_ASV1

CACCGCGGTTATACGAAAGACCCTAGTTGATGGGTGCGGCGTAAAGGGTGGTTAGGGACT

ACAAAAAATAGAGCTAAAGATCCTCTAGGTCGTCATACACATCACGAGGAAACGAAGCCC

AAATACGAAAGTAGCTCTAAATATAATCCTGACGCCACGAAAGCCAAGAAA

>271_Sample_ASV2

CACCGCGGTTATACGAAAGACCCTAGTTGATGGGTGCGGCGTAAAGGGTGGTTAGGGACT

ACAAAAAATAGAGCTAAAGATCCTCTAGGTCGTCATACACATCACGAGAAAACGAAGCCC

AAATACGAAAGTAGCTCTAAATATAATCCTGACGCCACGAAAGCCAAGAAA

>271_Sample_ASV3

CACCGCGGTTATACGAGAGGCCCAAGTTGACAGACACCGGCGTAAAGAGTGGCTAGGGAA

AAATTACTACTAAAGCCGAACACCTTCAGAACTGTCATACGTTCCCGAAGATAAGAAGCC

CCACCACGAAAGTGACTTTATATTACCCGACCCCACGAAAGCCAAGAAA

>271_Sample_ASV4

CACCGCGGTTATACGAGAGGCCCAAGTTGACAGACACCGGCGTAAAGAGTGGCTAGGGAA

AAATTACTACTAAAGCCGAACACCTTCAGAACTGTCATACGTTCCCGAAGATAAGAAGCC

CCACCACGAAAGTGACTTTATATTACCCGACCCCACGAAAGCTGTGAAA

>271_Sample_ASV5

CACCGCGGTTATACGAGAGGCCCAAGTTGACAGACACCGGCGTAAAGAGTGGCTAGGGAA

AAATTACTACTAAAGCCGAACACCCTCAGAACTGTCATACGTTCCCGAAGGTAAGAAGCC

CCACCACGAAAGTGGCTTTATAATACCCGACCCCACGAAAGCTGTGAAA

>271_Sample_ASV6

CACCGCGGTTATACGAGAGGCCCAAGTTGACAGACACCGGCGTAAAGAGTGGCTAGGGAA

AAATTACTACTAAAGCCGAACACCTTCAGAACTGTCATACGTTCCCGAAGATAAGAAGCC

CCACCACGAAAGTGGCTTTATATTACCCGACCCCACGAAAGCTGTGAAA

>271_Sample_ASV7

CACCGCGGTTATACGAGAGGCCCAAGTTGACAGACACCGGCGTAAAGAGTGGCTAAGGAA

AAATTTCTACTAAAGCCGAACACCTTCAGAACTGTCATACGTTCCCGAAGATAAGAAGCC

CCACCACGAAAGTGGCTTTATATTACCCGACACCACGAAAGCTGTGAAA

>271_Sample_ASV8

CACCGCGGTTATACGAAAGACTCTAGTTGACAAACACGGCGTAAAGGGTGGTTAGGATAA

CAACAAAATAAAGCTAAAGACTTACCAAGCCGTCATACGCCCATGTAAGAGCGAAAACCA

CAAACGAAAGTAACTTTATTACACCATCCGAATCCACGAAAGCTAAGAAA

>271_Sample_ASV9

CACCGCGGTTATACGAGAGGCCCAAGTTGATAAGCCCGGCGTAAAGAGTGGTTATGGAGA

ATAAAACACTAAAGCTAAAGACCCCCTAGGCTGTTATACGCACCTGGCGGCTCGAACCAC

CTATACGAAAGTAGCTTTACCCCCCTTCCACCAGAATCCACGACAGCTGGGGCA

>271_Sample_ASV10

CACCGCGGTTATACGAGAGGCCCTAGTTGATAGCCACGGCGCAAAGAGTGGTTAAGGAGC

CCCACTAAATAAAGTCGAACACTTCCTAGGCCGTTATACGTTTTCTAGAAGCACGAAACC

CAATTACACGAAAGCAACTTTATACTAAACAACCTGACCCCACGAAAGCTAAGAAA

>271_Sample_ASV11

CACCGCGGTTATACGAGAGGCCCAAGTTGACAGACATCGGCGTAAAGAGTGGTTAAGGAA

ACCCCATTACTAAAGCCGAACGCCCTCAGAACTGTCATACGTTCCCGAAGGTAAGAAGCC

CCACTACGAAAGTGGCTTTAACCACACCCGACCCCACGAAAGCTGTGAAA

>271_Sample_ASV12

CACCGCGGTTATACGAGAGGCCCAAGTTGACAGACACCGGCGTAAAGAGTGGTTAAGGAA

ATTCATCAACTAAAGCCGAATGCCCTCAGGACTGTTATACGTTTGCGAAGGTAAGAAGCC

CTACTACGAAAGTGGCTTTATTCCACCTGATTCCACGAAAGCTGCGGAA

>271_Sample_ASV13

CACCGCGGTTATACGAGAGGCCCAAGTTGACAGACACCGGCGTAAAGAGTGGTTAAGGAA

ATTCATCAACTAAAGCCGAATGCCCTCAGGACTGTTATACGTTTGAGAAGGTAAGAAGCC

CCACTACGAAAGTGGCTTTATTACACCTGACTCCACGAAAGCTGCGGAA

>271_Sample_ASV14

CACCGCGGTTATACGAAAGACCCCAGTTTATAGACACGGCGTAAAGGGTGGTTATAGGAC

AAACTAACATAGAGCCAAAGACCCCCTAAGCCGTCATACGCTCACGAGGGCTCGAAGCCC

CTATACGAAAGTAGCTCTACCAAAAAATGCTCGACTCCACGAAAGCTGGGACA

>271_Sample_ASV15

CACCGCGGTTATACGAAAGACCCTAGTTTATAAGTACGGCGTAAAGGGTGGTTAGGGGAC

AAACAAAATAAAGCCAAAGACCTCCTAAGCCGTCATACGCTTACGGAGGCACGAAGCCCA

AACACGAAAGTAGCTTTACCGAACATGCCCGACTCCACGAAAGCTGGGAAA

>271_Sample_ASV16

CACCGCGGTTATACGAAAGACCCTAATTGATAGCTCACGGCGTAAAGGGTGGTTTGGGGA

AACCTCAAAATAAAGCTAAAGACCTTCTAAGCCGTTACACGCATACCGAAGGCACGAGGC

CCTAACACGAAAGTAGCTTTACTATTACCCCTGACGCCACGAAAGCTAAGAAA

>271_Sample_ASV17

CACCGCGGTTATACGAAAGACCCTAGTTGATAGACACGGCGTAAAGGGTGGTTTGGGGAA

ACCTCAAAATAAAGCTAAAGACCTTCTAAGCCGTTACACGCATACCGAAGGCACGAGGCC

CTAACACGAAAGTAGCTTTACTATTACCCCTGACGCCACGAAAGCTAAGAAA

>271_Sample_ASV18

CACCGCGGTTATACGAGAGACCCTAATTGATAGCTCACGGCGTAAAGGGTGGTTTGGGGA

AACCTCAAAATAAAGCTAAAGACCTTCTAAGCCGTTACACGCATACCGAAGGCACGAGGC

CCTAACACGAAAGTAGCTTTACTATTACCCCTGACGCCACGAAAGCTAAGAAA

>271_Sample_ASV19

AACCGCGGTTATACGAGAGACCCTAATTGATAGCTCACGGCGTAAAGGGTGGTTTGGGGA

AACCTCAAAATAAAGCTAAAGACCTTCTAAGCCGTTACACGCATACCGAAGGCACGAGGC

CCTAACACGAAAGTAGCTTTACTATTACCCCTGACGCCACGAAAGCTAAGAAA

>271_Sample_ASV20

CACCGCGGTTATACGAAAGACCCTAGTTGATGGATCACGGCGTAAAGGGTGGTTAAGGGA

TATAAAAATAAAGCTAAAAATCCTCTTAGCTGTCATACGCTTTCCGAAGACATGAGGCCC

AATTACGAAAGTAGCTTTAAACCACCCTGACACCACGAAAGCCAAGAAA

>271_Sample_ASV21

CACCGCGGTTATACGAGAGGCCCTAGTTGATAACTACCGGCGTAAAGAGTGGTTATGGAA

AAATATTTAATAAAGCCGAACACCCCCTCAGCCGTCATACGCACCTGGGAGCACGAAGAC

CTACTGCGAAAGCAGCTTTAATTATGCCTGACCCCACGACAGCTAAGAAA

>271_Sample_ASV22

CACCGCGGTTATACGAAAGACCCTAGTTTATAGACACGGCGTAAAGGGTGGTTCGGGACT

AAACAAAATAAAGCCAAAGACCTCCTAAGCCGTCATACGCTCACGGAAGCACGAAGCCCA

AGTACGAAAGTAGCTTTACAAAACATGCCTGACTCCACGAAAGCTAGGAAA

>271_Sample_ASV23

CACCGCGGTTATACGAGAGGCCCAAGTTGATTATTACCGGCGTAAAGCGTGGTTAAGATT

TTTATAGGACTAAAGCCGAACCCCCTCAGAGCTGTTATACGCACCCGAAGGTTAGAAGTT

CAATCACGAAAGTGGCTTTACAACCCCGAACCCACGAAAGCTAAGATA

>271_Sample_ASV24

CACCGCGGTTATACGAGAGACCCTAATTGATAGGCGCGGCGTAAAGAGTGGTTTAGGACT

AAAACTAATAAAGCTAAAGATCCTCCCAGCCGTCGCACGCATTCTGAGGACACGAAACTC

AACCACGAAAGTAGCTTTAACATACTCTCCTGACCCCACGAAAGCTAAGAAA

>271_Sample_ASV25

CACCGCGGTTATACGAGAGACCCTAGTTGATATATACGGCGTAAAGAGTGGTTTAGGACT

AAAACTAATAAAGCTAAAGATCCTCCCAGCCGTCGCACGCATTCTGAGGACACGAAACTC

AACCACGAAAGTAGCTTTAACATACTCTCCTGACCCCACGAAAGCTAAGAAA

>271_Sample_ASV26

CACCGCGGTTATACGAAAGACCCTAGTTGATAGGCACGGCGTAAAGGGTGGTTAGGGATA

TATCAAAAATAAAGTTAAAGAGCCTCTAAGCTGTCGCACGCATCCCGAGAGCTCGAGACC

CAGACACGAAAGTAACTTTAAAATACCTCACCTGACCCCACGAAAGCTAAGAAA

>271_Sample_ASV27

CACCGCGGTTATACGAAAGACCCTAGTTTATAGGCACGGCGTAAAGGGTGGTTAGGGGAT

AAACAAAATAAAGCCAAAGACCTTCTAAGTCGTCATACACTCATGAAGGCACGAAGCCCA

AATACGAAAGTAACTTTACAACTTAGACCCGACTCCACGAAAGCTGGGGGA

>271_Sample_ASV28

CACCGCGGTTATACGAAAGACCCTAGTTGATAGACACGGCGTAAAGGGTGGTTAAGGTAA

CTAATAAATAAAGCTAAAGAGCCTCTAAGCCGTCGCACGCATTCCGAGAGCTCGAAGCCC

AAACACGAAAGTAGCTTTAAAATAAAGCACACCTGACCCCACGAAAGCTAAGAAA

>271_Sample_ASV29

CACCGCGGTTATACGAAAGACCCTAGTTGATAGCCACGGCGTAAAGGGTGGTTAAGGTAA

CTAATAAATAAAGCTAAAGAGCCTCTAAGCCGTCGCACGCATTCCGAGAGCTCGAAGCCC

AAACACGAAAGTAGCTTTAAAATAAAGCACACCTGACCCCACGAAAGCTAAGAAA

>271_Sample_ASV30

CACCGCGGTTATACGAGAGACCCTAATTAATAGCTACGGCGTAAAGAGTGGTTTAGGGTA

AAAATTAATAAAGCCGAAGTTTCTCCTAGCCGTTGTACGCACTCTGAGAACACGAAGCCC

CACTACGAAAGTAGCTTTACCTATTATTTCCTGACCCCACGAAAGCTAAGAAA

>271_Sample_ASV31

CACCGCGGTTATACGAGAGACCCTAATTAATAGATACGGCGTAAAGAGTGGTTTAGGACA

AAAATTAATAAAGCCGAAGTTTCTCCTAGCCGTTGTACGCACTCTGAGAACACGAAGCCC

CACTACGAAAGTAGCTTTACCTATTATTTCCTGACCCCACGAAAGCTAAGAAA

>271_Sample_ASV32

CACCGCGGTTATACGAGAGACCCTAATTGATAGACTACGGCGTAAAGAGTGGTTTAGAAC

AAAAATTAATAAAGCCAAAGATCCTCCTGGCCGTCGCACGCATTTCGAGGATACGAAGCC

CCACTACGAAAGTAGCTTTACTACAACTTTTCTGACCCCACGAAAGCTAAGAAA

>271_Sample_ASV33

CACCGCGGTTATACGAAAGACCCTAGTTGATAGATCACGGCGTAAAGGGTGGTTAAGGAA

AACAAAAATAAAGCTAAAAATCCTCTAAGCTGTCATACGCTTTCCGAAGACATGAGATCC

AACCACGAAAGTAGCTTTAAAATTTCCTGACACCACGAAAGCCAAGGAA

>286_Sample_ASV1

CACCGCGGTTATACGAAAGACCCTAGTTGATGGGTGCGGCGTAAAGGGTGGTTAGGGACT

ACAAAAAATAGAGCTAAAGATCCTCTAGGTCGTCATACACATCACGAGGAAACGAAGCCC

AAATACGAAAGTAGCTCTAAATATAATCCTGACGCCACGAAAGCCAAGAAA

>286_Sample_ASV2

CACCGCGGTTATACGAGAGACCCTAGTTGATTAAAGCGGCGTAAAGAGTGGTTATGGAAC

TATTCTTTTAAAGCAGAAAACCTCTCAAACTGTTATACGCACCCAGAGGTCTGAATCCCT

CACACGAAAGTGACTTTATTTATGCCTACCAGAACCCACGAAAGCTGGGACA

>286_Sample_ASV3

CACCGCGGTTATACGAAAGACCCTAGTTTATAGGTACGGCGTAAAGGGTGGTTAGGGGAC

AAACAAAATAAAGCCAAAGACCTCCTAAGCCGTCATACGCTCACGGAAGCACGAAGCCCA

AACACGAAAGTAGCTTTACCGAACATGCCCGACTCCACGAAAGCTGGGGAA

>286_Sample_ASV4

CACCGCGGTTATACGAGAGACCCTAGTTGATATACACGGCGTAAAGAGTGGTTTGGGGTA

TATAATAAATAAAGCCAAAGACCTTCTAGGCCGTTGAACGCACCACGAAGGAGCGAAGTC

CCAATACGAAAGTAGCTTTATAAAACCTGACGCCACGAAAGCTAAGAAA

>286_Sample_ASV5

CACCGCGGTTATACGAGAGGCCCAAGTTGACAGACACCGGCGTAAAGAGTGGCTAGGGAA

AAATTACTACTAAAGCCGAACACCTTCAGAACTGTCATACGTTCCCGAAGATAAGAAGCC

CCACCACGAAAGTGACTTTATATTACCCGACCCCACGAAAGCTGTGAAA

>286_Sample_ASV6

CACCGCGGTTATACGAGAGGCCCAAGTTGACAGACACCGGCGTAAAGAGTGGCTAGGGAA

AAATTACTACTAAAGCCGAACACCTTCAGAACTGTCATACGTTCCCGAAGATAAGAAGCC

CCACCACGAAAGTGGCTTTATATTACCCGACCCCACGAAAGCTAAGAAA

>286_Sample_ASV7

CACCGCGGTTATACGAGAGGCCCAAGTTGACAGACACCGGCGTAAAGAGTGGCTAGGGAA

AAATTACTACTAAAGCCGAACACCTTCAGAACTGTCATACGTTCCCGAAGATAAGAAGCC

CCACCACGAAAGTGGCTTTATATTACCCGACCCCACGAAAGCTGTGAAA

>286_Sample_ASV8

CACCGCGGTTATACGAGAGGCCCAAGTTGACAGACACCGGCGTAAAGAGTGGCTAAGGAA

AAATTTCTACTAAAGCCGAACACCTTCAGAACTGTCATACGTTCCCGAAGAAAAGGAGCC

CCACCACGAAAGTGGCTTTATATTACCCGACCCCACGAAAGCTGTGAAA

>286_Sample_ASV9

CACCGCGGTTATACGAGAGGCCCAAGTTGACAGACACCGGCGTAAAGAGTGGCTAAGGAA

AAATTTCTACTAAAGCCGAACACCTTCAGAACTGTCATACGTTCCCGAAGAAAAGAAGCC

CCACCACGAAAGTGGCTTTATATTACCCGACCCCACGAAAGCTGTGAAA

>286_Sample_ASV10

CACCGCGGTTATACGAGAGGCCCAAGTTGATAAGCCCGGCGTAAAGAGTGGTTATGGAGA

ATAAAACACTAAAGCTAAAGACCCCCTAGGCTGTTATACGCACCTGGCGGCTCGAACCAC

CTATACGAAAGTAGCTTTACCCCCCTTCCACCAGAATCCACGACAGCTGGGGCA

>286_Sample_ASV11

CACCGCGGTTATACGAGAGGCCCAAGTTGACAGACATCGGCGTAAAGAGTGGTTAAGGAA

ACCCCATTACTAAAGCCGAACGCCCTCAGAACTGTCATACGTTCCCGAAGGTAAGAAGCC

CCACTACGAAAGTGGCTTTAACCACACCCGACCCCACGAAAGCTGTGAAA

>286_Sample_ASV12

CACCGCGGTTATACGAGAGGCCCAAGTTGATAGATATCGGCGTAAAGAGTGGTTAAGGAA

ACCCCATTACTAAAGCCGAACGCCCTCAGAACTGTCATACGTTCCCGAAGGTAAGAAGCC

CCACTACGAAAGTGGCTTTAACCACACCCGACCCCACGAAAGCTGTGAAA

>286_Sample_ASV13

CACCGCGGTTATACGAGAGGCCCAAGTTGATAGATATCGGCGTAAAGAGTGGTTAAGGAA

ACCCCGTTACTAAAGCCGAACGCCCTCAGAACTGTCATACGTTCCCGAAGGTAAGAAGCC

CCACTACGAAAGTGGCTTTAACCACACCCGACCCCACGAAAGCTGTGAAA

>286_Sample_ASV14

CACCGCGGTTATACGAGAGGCCCTAGTTGATAAATACGGCGTAAAGAGTGGTTAGAGGAC

TCCCATAATAAAGCCAAAGACCCTCCGGGCCGTCACACGCATACGAGGGCACGAAGTTCA

CACACGAAAGTAGCTTTAAATCACCCGACGCCACGAAAGCTAAGAAA

>286_Sample_ASV15

CACCGCGGTTATACGAGAAACCCTAGTTGACAAATACGGCGTAAAGAGTGGTTAGAGGAC

TCCCATAATAAAGCCAAAGACCCTCCGGGCCGTCACACGCATACGAGGGCACGAAGTTCA

CACACGAAAGTAGCTTTAAATCACCCGACGCCACGAAAGCTAAGAAA

>286_Sample_ASV16

CACCGCGGTTATACGAGAAACCCTAGTTGATAAATACGGCGTAAAGAGTGGTTAGAGGAC

TCCCATAATAAAGCCAAAGACCCTCCGGGCCGTCACACGCATACGAGGGCACGAAGTTCA

CACACGAAAGTAGCTTTAAATCACCCGACGCCACGAAAGCTAAGAAA

>286_Sample_ASV17

CACCGCGGTTATACGAGAAACCCTAGTTGACAAATACGGCGTAAAGAGTGGTTAAGGGGT

ATCAAAAATAAAGTCAAACATTTTCCTAGTTGTTATAAGCATTTCGAGAACATGAAGCCC

AACCACGAAAGTAACTTTACCTTCACCTGACCCCACGAAAGCTAAGAAA

>286_Sample_ASV18

CACCGCGGTTATACGAGAGACCCTAGTTGATAAATACGGCGTAAAGAGTGGTTATGGGAA

AAACAAATAAAGTCAAACAACCTCTTAGCTGTTATACGCATTATGAGAGCATGAAGCCCC

CTCACGAAAGTAACTTTAATACCTCCTGACCCCACGAAAGCTAAGAAA

>286_Sample_ASV19

CACCGCGGTTATACGAGAGACCCTAATTGATAGACACGGCGTAAAGAGTGGTTTGGGAAA

GCCCAAAAATAAAGCTAAAGACCTTCTAAGCCGTCACACGCACGCCGAAGGCACGAAGCC

CTGACACGAAAGTAGCTTTACTATTACCCCCGACGCCACGAAAGCTAAGAAA

>286_Sample_ASV20

AACCGCGGTTATACGAGAGACCCTAATTGATAGCTCACGGCGTAAAGGGTGGTTTGGGGA

AACCTCAAAATAAAGCTAAAGACCTTCTAAGCCGTTACACGCATACCGAAGGCACGAGGC

CCTAACACGAAAGTAGCTTTACTATTACCCCTGACGCCACGAAAGCTAAGAAA

>286_Sample_ASV21

CACCGCGGTTATACGAAAGACCCTAGTTGATGGATCACGGCGTAAAGGGTGGTTAAGGGA

TATAAAAATAAAGCTAAAAATCCTCTTAGCTGTCATACGCTTTCCGAAGACATGAGGCCC

AATTACGAAAGTAGCTTTAAACCACCCTGACACCACGAAAGCCAAGAAA

>286_Sample_ASV22

CACCGCGGTCATACGATTAACCCAAGTTAATAGGCACACGGCGTAAAGCGTGTTTAAGCA

CTACGCCAAATAAAGTTAAACTCCAATTAAGCTGTAAAAAGCCATAATCAAGACGAAAAT

AAATAACGAAAGTAACTTTACAACCGCTGAGACACGATAGCTAAGATC

>286_Sample_ASV23

CACCGCGGTCATACGATTAACCCAAGTTAATAGGCACACGGCGTAAAGCGTGTTTAAGCA

CTATGCCAAATAAAGTTAAACTCCAATTAAGCTGTAAAAAGCCATAATCAAGACGAAAAT

AAATAACGAAAGTAACTTTACAACCGCTGAAACACGATAGCTAAGATC

>286_Sample_ASV24

CACCGCGGTTATACGAGAGACCCCAGTTGATAAACACGGCGTAAAGAGTGGTTAAGGGCA

CAAAACAAATAAAGTCAAACAACCTCTTAGCTGTTATACGCATTATGAGGATATGAAGCC

CCCCTACGAAAGTAACTTTACCAACCCCTGACCCCACGAAAGCTAAGAAA

>286_Sample_ASV25

CACCGCGGTTATACGAAAGACCCTAGTTTATAGGCACGGCGTAAAGGGTGGTTAGGGGAT

AAACAAAATAAAGCCAAAGACCTTCTAAGTCGTCATACACTCATGAAGGCACGAAGCCCA

AATACGAAAGTAACTTTACAACTTAGACCCGACTCCACGAAAGCTGGGGGA

>286_Sample_ASV26

CACCGCGGTTATACGAGAGACCCTAATTAATAGATACGGCGTAAAGAGTGGTTTAGGACA

AAAATTAATAAAGCCGAAGTTTCTCCTAGCCGTTGTACGCACTCTGAGAACACGAAGCCC

CACTACGAAAGTAGCTTTACCTATTATTTCCTGACCCCACGAAAGCTAAGAAA

>286_Sample_ASV27

CACCGCGGTCATACGATTAACCCAAGTTAATAGGCACACGGCGTAAAGCGTGTTAAAGCA

CTATACTAAATAAAGTTAAATTCCAATTAAGCTGTAAAAAGCCATAATTGCAACAAAAAT

AAACAACGAAAGTAACTTTACAGTCGCTGAAACACGATAGCTAGGACC

>286_Sample_ASV28

CACCGCGGTTATACGAGAGGCCCAAGTTGATAGACATCGGCGTAAAGAGTGGTTAAGGAA

GTCTCAAAACTAAAGCCGAATGCCTTCAAGACTGTTATACGTTTCCGAAGGTAAGAAGCC

CCACTACGAAAGTGGCTTTACTCCACCTGACTCCACGAAAGCTGTGAAA

>286_Sample_ASV29

CACCGCGGTTATACGAAAGACCCTAGTTGATAGATCACGGCGTAAAGGGTGGTTAAGGAG

AACAAAAATAAAGCTAAAAATCCTCTAAGCTGTCATACGCTTTCCGAAGACATGAGACCC

AACCACGAAAGTAACTTTAAACTTTCCTGACACCACGAAAGCCAAGAAA

>300_Sample_ASV1

CACCGCGGTTATACGAGAGGCCCAAGTTGACAGACATCGGCGTAAAGAGTGGTTAAGGAA

AACCTTCAACTAAAGTCGAACGCTCTCAAGACTGTCGTACGTTTCCGAGAGTATGAAGCC

CCACCACGAAAGTGACTTTACCTCACCTGACTCCACGAAAGCTGCGAAA

>300_Sample_ASV2

CACCGCGGTTATACGAAAGACCCTAGTTGATGGGTGCGGCGTAAAGGGTGGTTAGGGACT

ACAAAAAATAGAGCTAAAGATCCTCTAGGTCGTCATACACATCACGAGGAAACGAAGCCC

AAATACGAAAGTAGCTCTAAATATAATCCTGACGCCACGAAAGCCAAGAAA

>300_Sample_ASV3

CACCGCGGTTATACGAAAGACCCTAGTTGATAACTACGGCATAAAGGGTGGTTAGGGAGT

TTAAAAAATAAAGCCAAAGCTCTTCTAAGCTGTCATACGCTCTCTGAAAGAACGAGGCCC

AAACACGAAAGTAGCTTTAAATAAATAACCTGATACCACGAAAGCCAAGAAA

>300_Sample_ASV4

CACCGCGGTTATACGAGAGACCCTAGTTGATTAAAGCGGCGTAAAGAGTGGTTATGGAAC

TATTCTTTTAAAGCAGAAAACCTCTCAAACTGTTATACGCACCCAGAGGTCTGAATCCCT

CACACGAAAGTGACTTTACTTATGCCTACCAGAACCCACGAAAGCTGGGACA

>300_Sample_ASV5

CACCGCGGTTATACGAGAGACCCTAGTTGATTAAAGCGGCGTAAAGAGTGGTTATGGAAC

TATTCTTTTAAAGCAGAAAACCTCTCAAACTGTTATACGCACCCAGAGGTCTGAATCCCT

CACACGAAAGTGACTTTATTTATGCCTACCAGAACCCACGAAAGCTGGGACA

>300_Sample_ASV6

CACCGCGGTTATACGAGAGACCCTAATTAATAATTACGGCGTAAAGAGTGGTTAAAAGTT

AATATAAATAAAGCCAAAGATTTTCCAAGCTGTTATACGCACTCGAAAACATGAAGCCTT

ATAACGAAAGTAGCTTTACAAACCTTTTGACCCCACGAAAGCTATGATA

>300_Sample_ASV7

CACCGCGGTTATACGAAAGACCCTAGTTGATAGACACCGGCGTAAAGAGTGGCTAGGGAA

AAATTACTACTAAAGCCGAACACCTTCAGAACTGTCATACGTTCCCGAAGATAAGAAGCC

CCACCACGAAAGTGGCTTTATATTACCCGACCCCACGAAAGCTGTGAAA

>300_Sample_ASV8

CACCGCGATTATACGAGAGGCCCAAGTTGACAGACATCGGCGTAAAGAGTGGTTAAGGAA

AAATTACTGCTAAAGCCGAACACCTTCAGAACTGTCATACGTTCCCGAAGATAAGAAGCC

CCACCACGAAAGTGGCTTTATATTACCCGACCCCACGAAAGCTGTGAAA

>300_Sample_ASV9

CATGGCGATTATACGAGAGGCCCAAGTTGACAGACACTGGCGTAAAGAGTGGCTAAGGAA

AAATTACTACTAAAGCCGAACACCTTCAGAACTGTCATACGTTCCCGAAGATAAGAAGCC

CCACCACGAAAGTGGCTTTATATTACCCGACCCCACGAAAGCTGTGAAA

>300_Sample_ASV10

CACCGCGGTTATACGAGAGGCCCAAGTTGACAGACACCGGCGTAAAGAGTGGCTAGGGAA

AAATTACTACTAAAGCCGAACACCTTCAGAACTGTCATACGTTCCCGAAGATAAGAAGCC

CCACCACGAAAGTGGCTTTATATTACCCGACCCCACGAAAGCCAAGAAA

>300_Sample_ASV11

CACCGCGGTTATACGAGAGGCCCAAGTTGACAGACACCGGCGTAAAGAGTGGCTAGGGAA

AAATTACTACTAAAGCCGAACACCTTCAGAACTGTCATACGTTCCCGAAGATAAGAAGCC

CCACCACGAAAGTGGCTTTATATTACCCGACCCCACGAAAGCTGTGAAA

>300_Sample_ASV12

CACCGCGGTTATACGAGAGGCCCAAGTTGACAGACACCGGCGTAAAGAGTGGCTAAGGAA

AAATTTCTACTAAAGCCGAACACCTTCAGAACTGTCATACGTTCCCGAAGAAAAGAAGCC

CCACCACGAAAGTGGCTTTATATTACCCGACCCCACGAAAGCTGTGAAA

>300_Sample_ASV13

CACCGCGGTTATACGAAAGACCCTAGTTTATAGAAACGGCGTAAAGGGTGGTTAAGGGAC

AAACAAAATAAGGCCAAAGACTTCCTAAGCCGTCATACGCTCCACGAAAGCACGAAGCCC

AAACACGAAAGTAGCCTTAATAACTTCACCTGACCCCACGAAAGCTGGGAGA

>300_Sample_ASV14

CACCGCGGTTATACGAGAGACCCTAGTTGATATATACGGCGTAAAGAGTGGTTTGGGATA

TTTCTCCAAAATAAAGCCGAAGATCTCCCAAGCCGTCACACGCATCCCGGAGACACGAAG

CCCTGATACGAAGGTAGCTTTATTAACACCCCGACGCCACGAAAGCTAAGAAA

>300_Sample_ASV15

CACCGCGGTTATACGAGAGGCCCAAGTTGATAAGCCCGGCGTAAAGAGTGGTTATGGAGA

ATAAAACACTAAAGCTAAAGACCCCCTAGGCTGTTATACGCACCTGGCGGCTCGAACCAC

CTATACGAAAGTAGCTTTACCCCCCTTCCACCAGAATCCACGACAGCTGGGGCA

>300_Sample_ASV16

CACCGCGGTTATACGAGAGGCCCAAGTTGACAGACATCGGCGTAAAGAGTGGTTAAGGAA

ACCCCATTACTAAAGCCGAACGCCCTCAGAACTGTCATACGTTCCCGAAGGTAAGAAGCC

CCACTACGAAAGTGGCTTTAACCACACCCGACCCCACGAAAGCTGTGAAA

>300_Sample_ASV17

CACCGCGGTTATACGAGAGGCCCAAGTTGACAGACACCGGCGTAAAGAGTGGTTAAGGAA

ATTCATCAACTAAAGCCGAATGCCCTCAGGACTGTTATACGTTTGCGAAGGTAAGAAGCC

CTACTACGAAAGTGGCTTTATTCCACCTGATTCCACGAAAGCTGCGGAA

>300_Sample_ASV18

CACCGCGGTTATACGAGAGGCCCCAGTTGATATTAGCGGCGTAAAGGGTGGTTAAGGACT

TTAATAACTAAAGCTAAACACTTCCCCGGCTGTTGTACGTAGACCGGAAGCTTGAAGCCC

AGACACGAAAGTAGCTTTATTCCCCCTGACCCCACGAAAGCTGAGAAA

>300_Sample_ASV19

CACCGCGGTTATACGAGAAACCCTAGTTGACAAATACGGCGTAAAGAGTGGTTAAGGGGT

ATCAAAAATAAAGTCAAACATTTTCCTAGTTGTTATAAGCATTTCGAGAACATGAAGCCC

AACCACGAAAGTAACTTTACCTTCACCTGACCCCACGAAAGCTAAGAAA

>300_Sample_ASV20

CACCGCGGTTATACGAGAGACCCTAATTGATAGGTACGGCGTAAAGAGTGGTTTGGGATA

ACCCAAAAATAAAGCTAAAGACCTTCTAAGCCGTCACACGCATACCGAAGGCACGAGGCC

CCAACACGAAAGTAGCTTTACTATTACCCCGACGCCACGAAAGCTAAGAAA

>300_Sample_ASV21

CACCGCGGTTATACGAGAGGCCCTAGTTGATAACTACCGGCGTAAAGAGTGGTTATGGAA

AAATATTTAATAAAGCCGAACACCCCCTCAGCCGTCATACGCACCTGGGAGCACGAAGAC

CTACTGCGAAAGCAGCTTTAATTATGCCTGACCCCACGACAGCTAAGAAA

>300_Sample_ASV22

CACCGCGGTTATACGAAAGACCCTAGTTTATAGACACGGCGTAAAGGGTGGTTCGGGACT

AAACAAAATAAAGCCAAAGACCTCCTAAGCCGTCATACGCTCACGGAAGCACGAAGCCCA

AGTACGAAAGTAGCTTTACAAAACATGCCTGACTCCACGAAAGCTAGGAAA

>300_Sample_ASV23

CACCGCGGTTATACGAGAGGCCCAAGTTGATACTCCACGGCGTAAAGAGTGGTTAGAAAA

GACCTACTACTAAAGCCGAACGCCTTCAAAGCTGTTATACGCATCCGAAGGTGAGAAGCC

CACCCACGAAAGTGGCTTTACAACCTTGACTCCACGAAAGCTATGACA

>300_Sample_ASV24

CACCGCGGTTATACGAGAGACCCCAGTTGATAAACACGGCGTAAAGAGTGGTTAAGGGCA

CAAAACAAATAAAGTCAAACAACCTCTTAGCTGTTATACGCATTATGAGGATATGAAGCC

CCCCTACGAAAGTAACTTTACCAACCCCTGACCCCACGAAAGCTAAGAAA

>300_Sample_ASV25

CACCGCGGTTATACGAAAGACCCTAGTTTATAGGCACGGCGTAAAGGGTGGTTAGGGGAT

AAACAAAATAAAGCCAAAGACCTTCTAAGTCGTCATACACTCATGAAGGCACGAAGCCCA

AATACGAAAGTAACTTTACAACTTAGACCCGACTCCACGAAAGCTGGGGGA

>300_Sample_ASV26

CACCGCGGTTATACGAGAGGCCCAAGTTGATAGACATCGGCGTAAAGAGTGGTTAAGGAA

GTCTCAAAACTAAAGCCGAATGCCTTCAAGACTGTTATACGTTTCCGAAGGTAAGAAGCC

CCACTACGAAAGTGGCTTTACTCCACCTGACTCCACGAAAGCTGTGAAA

>300_Sample_ASV27

CACCGCGGTTATACGAGAGACTCCAGTTGATAGCTACGGCGTAAAGAGTGGTTTGGGGCC

CGCCCAAAATAAAGCCAAAGACCTCCCAAGCCGTCAAACGCACCCCGGAGGCACGAAGTC

CTAACGCGAAAGCAACTTTACCTTCCCCGACGCCACGAAAGCTAAGAAA

>300_Sample_ASV28

CACCGCGGTTATACGAAAGACCCTAGTTGATAGACCACGGCGTAAAGGGTGGTTAAGGAA

CACAAAAATAAAGCTAAAGATCCTCTAAGCTGTCATACGCTTCCCGAAGACATGAAACCC

AATCACGAAAGTAGCTTTAAACTGTCCTGACACCACGAAAGCCAAGAAA

>300_Sample_ASV29

CACCGCGGTTATACGAAAGACCCTAGTTGATAGATCACGGCGTAAAGGGTGGTTAAGGAG

AACAAAAATAAAGCTAAAAATCCTCTAAGCTGTCATACGCTTTCCGAAGACATGAGACCC

AACCACGAAAGTAACTTTAAACTTTCCTGACACCACGAAAGCCAAGAAA

>308_Sample_ASV1

CACCGCGGTTATACGAAAGACCCTAGTTGATGGGTGCGGCGTAAAGGGTGGTTAGGGACT

ACAAAAAATAGAGCTAAAGATCCTCTAGGTCGTCATACACATCACGAGGAAACGAAGCCC

AAATACGAAAGTAGCTCTAAATATAATCCTGACGCCACGAAAGCCAAGAAA

>308_Sample_ASV2

CACCGCGGTTATACGAGAGACCCTAGTTGATTAAAGCGGCGTAAAGAGTGGTTATGGAAC

TATTCTTTTAAAGCAGAAAACCTCTCAAACTGTTATACGCACCCAGAGGTCTGAATCCCT

CACACGAAAGTGACTTTATTTATGCCTACCAGAACCCACGAAAGCTGGGACA

>308_Sample_ASV3

CACCGCGGTTATACGAGAGACCCTAGTTGATATACACGGCGTAAAGAGTGGTTTGGGGTA

TATAATAAATAAAGCCAAAGACCTTCTAGGCCGTTGAACGCACCACGAAGGAGCGAAGTC

CCAATACGAAAGTAGCTTTATAAAACCTGACGCCACGAAAGCTAAGAAA

>308_Sample_ASV4

CACCGCGGTTATACGAGAGGCCCAAGTTGACAGACACCGGCGTAAAGAGTGGCTAGGGAA

AAATTACTACTAAAGCCGAACACCTTCAGAACTGTCATACGTTCCCGAAGATAAGAAGCC

CCACCACGAAAGTGACTTTATATTACCCGACCCCACGAAAGCTGTGAAA

>308_Sample_ASV5

CACCGCGGTTATACGAGAGGCCCAAGTTGACAGACACCGGCGTAAAGAGTGGCTAGGGAA

AAATTACTACTAAAGCCGAACACCTTCAGAACTGTCATACGTTCCCGAAGATAAGAAGCC

CCACCACGAAAGTGGCTTTATATTACCCGACCCCACGAAAGCTGTGAAA

>308_Sample_ASV6

CACCGCGGTTATACGAGAGGCCCAAGTTGACAGACACCGGCGTAAAGAGTGGCTAGGGAA

AAATTACTACTAAAGCCGAACACCTTCAGAACTGTCATACGTTCCCGAAGATAAGAAGCC

CCACCACGAAAGTGGCTTTATATTACCCGACCCCACGAAAGCTAAGAAA

>308_Sample_ASV7

CACCGCGGTTATACGAGAGGCCCAAGTTGACAGACACCGGCGTAAAGAGTGGCTAAGGAA

AAATTTCTACTAAAGCCGAACACCTTCAGAACTGTCATACGTTCCCGAAGAAAAGAAGCC

CCACCACGAAAGTGGCTTTATATTACCCGACCCCACGAAAGCTGTGAAA

>308_Sample_ASV8

CACCGCGGTTATACGAAAGACCCTAGTTGATAGATCACGGCGTAAAGGGTGGTTAGGGAA

TACAAAAATAAAGCTAAAAATCCTCTAAGCTGTCATACGCTTCCCGAAGACATGAAACCC

AACCACGAAAGTAGCTTTAAACTCTCCTGACACCACGAAAGCCAAGGAA

>308_Sample_ASV9

CACCGCGGTTATACGAGAGGCCCAAGTTGATAAGCCCGGCGTAAAGAGTGGTTATGGAGA

ATAAAACACTAAAGCTAAAGACCCCCTAGGCTGTTATACGCACCTGGCGGCTCGAACCAC

CTATACGAAAGTAGCTTTACCCCCCTTCCACCAGAATCCACGACAGCTGGGGCA

>308_Sample_ASV10

CACCGCGGTTATACGAGAGGCCCAAGTTGACAGACATCGGCGTAAAGAGTGGTTAAGGAA

ACCCCATTACTAAAGCCGAACGCCCTCAGAACTGTCATACGTTCCCGAAGGTAAGAAGCC

CCACTACGAAAGTGGCTTTAACCACACCCGACCCCACGAAAGCTGTGAAA

>308_Sample_ASV11

CACCGCGGTTATACGAGAGGCCCAAGTTGATAGATATCGGCGTAAAGAGTGGTTAAGGAA

ACCCCGTTACTAAAGCCGAACGCCCTCAGAACTGTCATACGTTCCCGAAGGTAAGAAGCC

CCACTACGAAAGTGGCTTTAACCACACCCGACCCCACGAAAGCTGTGAAA

>308_Sample_ASV12

CACCGCGGTTATACGAGAGGCCCTAGTTGATAAATACGGCGTAAAGAGTGGTTAGAGGAC

TCCCATAATAAAGCCAAAGACCCCCCGGGCCGTCACACGCATACGAGGGCACGAAGTTCA

CACACGAAAGTAGCTTTAAATTACCCGACGCCACGAAAGCTAAGAAA

>308_Sample_ASV13

CACCGCGGTTATACGAGAAACCCTAGTTGACAAATACGGCGTAAAGAGTGGTTAAGGGGT

ATCAAAAATAAAGTCAAACATTTTCCTAGTTGTTATAAGCATTTCGAGAACATGAAGCCC

AACCACGAAAGTAACTTTACCTTCACCTGACCCCACGAAAGCTAAGAAA

>308_Sample_ASV14

CACCGCGGTTATACGAGAGACCCTAGTTGATAAATACGGCGTAAAGAGTGGTTATGGGAA

AAACAAATAAAGTCAAACAACCTCTTAGCTGTTATACGCATTATGAGAGCATGAAGCCCC

CTCACGAAAGTAACTTTAATACCTCCTGACCCCACGAAAGCTAAGAAA

>308_Sample_ASV15

AACCGCGGTTATACGAGAGACCCTAATTGATAGCTCACGGCGTAAAGGGTGGTTTGGGGA

AACCTCAAAATAAAGCTAAAGACCTTCTAAGCCGTTACACGCATACCGAAGGCACGAGGC

CCTAACACGAAAGTAGCTTTACTATTACCCCTGACGCCACGAAAGCTAAGAAA

>308_Sample_ASV16

CACCGCGGTTATACGAAAGACCCTAGTTGATGGATCACGGCGTAAAGGGTGGTTAAGGGA

TATAAAAATAAAGCTAAAAATCCTCTTAGCTGTCATACGCTTTCCGAAGACATGAGGCCC

AATTACGAAAGTAGCTTTAAACCACCCTGACACCACGAAAGCCAAGAAA

>308_Sample_ASV17

CACCGCGGTTATACGAAAGACCCCAGTTTATAGATGCGGCGTAAAGGGTGGTTAAGAGAA

AATCAAAATAAGGCCAAAGATCCCCTAAGCAGTCATACGCTCCACGAGGACACGAAACCC

AAATACGAAAGTTGCCTTAATAAACTTCACCTGATCCCACGAAAGCTGAAAAA

>308_Sample_ASV18

CACCGCGGTTATACGAGAGACTCTAGTTGATACCAACGGCGTAAAGAGTGGTTTGGGATG

CCCCTTAAAATAAAGCCAAAGACCTCCCAAGCCGTCAAACGCACCCCGGAGGCACGAAGC

CCCAACGCGAAAGCAACTTTACCTCCCTCCCGACGCCACGAAAGCTAAGAAA

>308_Sample_ASV19

CACCGCGGTTATACGAGAGGCCCAAGTTGATACTCCACGGCGTAAAGAGTGGTTAGAAAA

GACCTACTACTAAAGCCGAACGCCTTCAAAGCTGTTATACGCATCCGAAGGTGAGAAGCC

CACCCACGAAAGTGGCTTTACAACCTTGACTCCACGAAAGCTATGACA

>308_Sample_ASV20

CACCGCGGTTATACGAAAGACCCTAGTTTATAGGCACGGCGTAAAGGGTGGTTAGGGGAT

AAACAAAATAAAGCCAAAGACCTTCTAAGTCGTCATACACTCATGAAGGCACGAAGCCCA

AATACGAAAGTAACTTTACAACTTAGACCCGACTCCACGAAAGCTGGGGGA

>308_Sample_ASV21

CACCGCGGTTATACGAGAGACCCTAATTAATAGATACGGCGTAAAGAGTGGTTTAGGACA

AAAATTAATAAAGCCGAAGTTTCTCCTAGCCGTTGTACGCACTCTGAGAACACGAAGCCC

CACTACGAAAGTAGCTTTACCTATTATTTCCTGACCCCACGAAAGCTAAGAAA

>308_Sample_ASV22

CACCGCGGTTATACGAGAGGCCCAAGTTGATAGACATCGGCGTAAAGAGTGGTTAAGGAA

GTCTCAAAACTAAAGCCGAATGCCTTCAAGACTGTTATACGTTTCCGAAGGTAAGAAGCC

CCACTACGAAAGTGGCTTTACTCCACCTGACTCCACGAAAGCTGTGAAA

>330_Sample_ASV1

CACCGCGGTTATACGAGAGGCCCAAGTTGACAGACATCGGCGTAAAGAGTGGTTAAGGAA

AACCTTCAACTAAAGTCGAACGCTCTCAAGACTGTCGTACGTTTCCGAGAGTATGAAGCC

CCACCACGAAAGTGACTTTACCTCACCTGACTCCACGAAAGCTGCGAAA

>330_Sample_ASV2

CACCGCGGTTATACGAAAGACCCTAGTTGATGGGTGCGGCGTAAAGGGTGGTTAGGGACT

ACAAAAAATAGAGCTAAAGATCCTCTAGGTCGTCATACACATCACGAGGAAACGAAGCCC

AAATACGAAAGTAGCTCTAAATATAATCCTGACGCCACGAAAGCCAAGAAA

>330_Sample_ASV3

CACCGCGGTTATACGAAAGACCCTAGTTGATGGGTGCGGCGTAAAGGGTGGTTAGGGACT

ACAAAAAATAGAGCTAAAGATCCTCTAGGTCGTCATACACATCACGAGAAAACGAAGCCC

AAATACGAAAGTAGCTCTAAATATAATCCTGACGCCACGAAAGCCAAGAAA

>330_Sample_ASV4

CACCGCGGTTAAACGAGAGGCCCTAGTTGATGGTGCAACGGCGTAAAGGGTGGTTAAGGA

TAGCGAAATAATAAAGTCAAATGGCCCTCTGGCTGTCATACGCTTCTAGGAGTCCGAAGC

CCAGTATACGAAAGTAGCTTTAAGAAAGTCCACCTGACCCCACGAAAACTGAGGAA

>330_Sample_ASV5

CACCGCGGTTATACGAAAGACCCTAGTTGATAACTACGGCATAAAGGGTGGTTAGGGAGT

TTAAAAAATAAAGCCAAAGCTCTTCTAAGCTGTCATACGCTCTCTGAAAGAACGAGGCCC

AAACACGAAAGTAGCTTTAAATAAATAACCTGATACCACGAAAGCCAAGAAA

>330_Sample_ASV6

CACCGCGGTTATACGAGAGGCCCAAGTTGATTAAAGCGGCGTAAAGAGTGGTTATGGAAC

TATTCTTTTAAAGCAGAAAACCTCTCAAACTGTTATACGCACCCAGAGGTCTGAATCCCT

CACACGAAAGTGACTTTATTTATGCCTACCAGAACCCACGAAAGCTGGGACA

>330_Sample_ASV7

CACCGCGGTTATACGAGAGACCCTAGTTGATTAAAGCGGCGTAAAGAGTGGTTATGGAAC

TATTCTTTTAAAGCAGAAAACCTCTCAAACTGTTATACGCACCCAGAGGTCTGAATCCCT

CACACGAAAGTGACTTTATTTATGCCTACCAGAACCCACGAAAGCTGGGACA

>330_Sample_ASV8

CACCGCGGTTATACGAGAGACCCTAGTTGATTAAAGCGGCGTAAAGAGTGGTTATGGAAC

TATTCTTTTAAAGCAGAAAACCTCTCAAACTGTTATACGCACCCAGAGGTCTGAATCCCT

CACACGAAAGTGACTTTATTTATGCCTACCAGAACCCACGAAAGCTGTGAAA

>330_Sample_ASV9

CACCGCGGTTATACGAAAGACCCTAGTTTATAGGTACGGCGTAAAGGGTGGTTAGGGGAC

AAACAAAATAAAGCCAAAGACCTCCTAAGCCGTCATACGCTCACGGAAGCACGAAGCCCA

AACACGAAAGTAGCTTTACCGAACATGCCCGACTCCACGAAAGCTGGGGAA

>330_Sample_ASV10

CACCGCGGTTATACGAAAGACCCTAGTTGATAGCCACGGCGTAAAGGGTGGTTAAGGAAC

CTAAAAAATAAAGCTAAAGACCCTCTAGGCCGTCATACGCATTACGAGGCAAACGAAACC

CCAACACGAAAGTAGCTTTAGACAAATATCCTGATGCCACGAAAGCCAAGAAA

>330_Sample_ASV11

CACCGCGGTTATACGAGAGGCCCAAGTTGACAGACACCGGCGTAAAGAGTGGCTAGGGAA

AAATTACTACTAAAGCCGAACACCTTCAGAACTGTCATACGTTCCCGAAGATAAGAAGCC

CCACCACGAAAGTGGCTTTATATTACCCGACCCCACGAAAGCTGTGAAA

>330_Sample_ASV12

CATGGCGATTATACGAGAGGCCCAAGTTGACAGACACCGGCGTAAAGAGTGGCTAGGGAA

AAATTACTACTAAAGCCGAACACCTTCAGAACTGTCATACGTTCCCGAAGATAAGAAGCC

CCACCACGAAAGTGGCTTTATATTACCCGACCCCACGAAAGCTGTGAAA

>330_Sample_ASV13

CACCGCGGTTATACGAGAGGCCCAAGTTGACAGACACCGGCGTAAAGAGTGGCTAGGGAA

AAATTACTACTAAAGCCGAACACCTTCAGAACTGTCATACGTTCCCGAAGATAAGAAGCC

CCACCACGAAAGTGGCTTTACAATGCCTGACCCCACGAAAGCTGTGAAA

>330_Sample_ASV14

CACCGCGGTTATACGAGAGGCCCAAGTTGACAGACACCGGCGTAAAGAGTGGCTAAGGAA

AAACTACTACTAAAGCCGAAAACCCTCAGAACTGTTATACGTTCCCGAAGATAAGAAGCC

CCACCACGAAAGTGGCTTTATATTACCCGACCCCACGAAAGCTGTGAAA

>330_Sample_ASV15

CACCGCGGTTATACGAGAGGCCCAAGTTGACAGACACTGGCGTAAAGAGTGGCTAAGGAA

AAACTACTACTAAAGCCGAAAACCCTCAGAACTGTCATACGTTCCCGAAGATAAGAAGCC

CCACCACGAAAGTGGCTTTATATTACCCGACCCCACGAAAGCTGTGAAA

>330_Sample_ASV16

CATGGCGATTATACGAGAGGCCCAAGTTGACAGACACTGGCGTAAAGAGTGGCTAAGGAA

AAATTACTACTAAAGCCGAACACCTTCAGAACTGTCATACGTTCCCGAAGATAAGAAGCC

CCACCACGAAAGTGGCTTTATATTACCCGACCCCACGAAAGCTGTGAAA

>330_Sample_ASV17

CATGGCGATTATACGAGAGGCCCAAGTTGACAGACACTGGCGTAAAGAGTGGCTAAGGAA

AAACTACTACTAAAGCCGAACACCTTCAGAACTGTCATACGTTCCCGAAGATAAGAAGCC

CCACCACGAAAGTGGCTTTATATTACCCGACCCCACGAAAGCTGTGAAA

>330_Sample_ASV18

CATGGCGATTATACGAGAGGCCCAAGTTGACAGACACTGGCGTAAAGAGTGGCTAGGGAA

AAATTACTACTAAAGCCGAACACCTTCAGAACTGTCATACGTTCCCGAAGATAAGAAGCC

CCACCACGAAAGTGGCTTTATATTACCCGACCCCACGAAAGCTGTGAAA

>330_Sample_ASV19

CACCGCGGTTATACGAGAGGCCCAAGTTGACAGACACCGGCGTAAAGAGTGGCTAGGGAA

AAATTACTACTAAAGCCGAACACCTTCAGAACTGTCATACGTTCCCGAAGATAAGAAGCT

CCACCACGAAAGTGGCTTTACAATGCCTGACCCCACGAAAGCTGTGAAA

>330_Sample_ASV20

CACCGCGGTTATACGAGAGGCCCAAGTTGACAGACACCGGCGTAAAGAGTGGTTAAGGAA

ACCCCATTACTAAAGCCGAACACCTTCAGAACTGTCATACGTTCCCGAAGATAAGAAGCC

CCACCACGAAAGTGGCTTTATATTACCCGACCCCACGAAAGCTGTGAAA

>330_Sample_ASV21

CACCGCGGTTATACGAAAGACCCTAGTTTATAGAAACGGCGTAAAGGGTGGTTAAGGGAC

AAACAAAATAAGGCCAAAGACTTCCTAAGCCGTCATACGCTCCACGAAAGCACGAAGCCC

AAACACGAAAGTAGCCTTAATAACTTCACCTGACCCCACGAAAGCTGTGAAA

>330_Sample_ASV22

CACCGCGGTTATACGAAAGACCCTAGTTTATAGAAACGGCGTAAAGGGTGGTTAAGGGAC

AAACAAAATAAGGCCAAAGACTTCCTAAGCCGTCATACGCTCCACGAAAGCACGAAGCCC

AAACACGAAAGTAGCCTTAATAACTTCACCTGACCCCACGAAAGCTGGGAGA

>330_Sample_ASV23

CACCGCGGTTATACGAGAGACCCTAGTTGATATATACGGCGTAAAGAGTGGTTTGGGATA

TTTCTCCAAAATAAAGTCGAAGATCTCCCAAGCCGTCACACGCATCCCGGAGACACGAAG

CCCTGATACGAAGGTAGCTTTATTAACACCCCGACGCCACGAAAGCTAAGAAA

>330_Sample_ASV24

CACCGCGGTTATACGAGAGACCCTAGTTGATATATACGGCGTAAAGAGTGGTTTGGGATA

TTTCTCCAAAATAAAGCCGAAGATCTCCCAAGCCGTCACACGCATCCCGGAGACACGAAG

CCCTGATACGAAGGTAGCTTTATTAACACCCCGACGCCACGAAAGCTAAGAAA

>330_Sample_ASV25

CACCGCGGTTATACGAGAGGCCCAAGTTGATAAGCCCGGCGTAAAGAGTGGTTATGGAGA

ATAAAACACTAAAGCTAAAGACCCCCTAGGCTGTTATACGCACCTGGCGGCTCGAACCAC

CTATACGAAAGTAGCTTTACCCCCCTTCCACCAGAATCCACGACAGCTGGGGCA

>330_Sample_ASV26

CACCGCGGTTATACGAGAGGCCCAAGTTGACAGACATCGGCGTAAAGAGTGGTTAAGGAA

ACCCCATTACTAAAGCCGAACGCCCTCAGAACTGTCATACGTTCCCGAAGGTAAGAAGCC

CCACTACGAAAGTGGCTTTAACCACACCCGACCCCACGAAAGCTGTGAAA

>330_Sample_ASV27

CACCGCGGTTATACGAGAGGCCCAAGTTGACAGACATCGGCGTAAAGAGTGGTTAAGGAA

ACCCCATTACTAAAGCCGAACGCCCTCAGAACTGTCATACGTTCCCGAAGGTAAGAAGCC

CCACTACGAAAGTGGCTTTATATTACCCGACCCCACGAAAGCTGTGAAA

>330_Sample_ASV28

CACCGCGGTTATACGAGAGGCCCAAGTTGATAGATATCGGCGTAAAGAGTGGTTAAGGAA

ACCCCGTTACTAAAGCCGAACGCCCTCAGAACTGTCATACGTTCCCGAAGGTAAGAAGCC

CCACTACGAAAGTGGCTTTAACCACACCCGACCCCACGAAAGCTGTGAAA

>330_Sample_ASV29

CACCGCGGTTATACGAGAGGCCCAAGTTGACAGACACCGGCGTAAAGAGTGGTTAAGGAA

ATTCATCAACTAAAGCCGAATGCCCTCAGGACTGTTATACGTTTGCGAAGGTAAGAAGCC

CTACTACGAAAGTGGCTTTATTCCACCTGATTCCACGAAAGCTGCGGAA

>330_Sample_ASV30

CACCGCGGTTATACGAGAGGCCCCAGTTGATATTAGCGGCGTAAAGGGTGGTTAAGGACT

TTAATAACTAAAGCTAAACACTTCCCCGGCTGTTGTACGTAGACCGGAAGCTTGAAGCCC

AGACACGAAAGTAGCTTTATTCCCCCTGACCCCACGAAAGCTGAGAAA

>330_Sample_ASV31

CACCGCGGTTATACGAGAAACCCTAGTTGACAAATACGGCGTAAAGAGTGGTTAAGGGGT

ATCAAAAATAAAGTCAAACATTTTCCTAGTTGTTATAAGCATTTCGAGAACATGAAGCCC

AACCACGAAAGTAACTTTACCTTCACCTGACCCCACGAAAGCTAAGAAA

>330_Sample_ASV32

CACCGCGGTTATACGAGAGACCCTAGTTGATAAATACGGCGTAAAGAGTGGTTATGGGAA

AAACAAATAAAGTCAAACAACCTCTTAGCTGTTATACGCATTATGAGAGCATGAAGCCCC

CTCACGAAAGTAACTTTAATACCTCCTGACCCCACGAAAGCTGTGAAA

>330_Sample_ASV33

CACCGCGGTTATACGAGAGACCCTAGTTGATAAATACGGCGTAAAGAGTGGTTATGGGAA

AAACAAATAAAGTCAAACAACCTCTTAGCTGTTATACGCATTATGAGAGCATGAAGCCCC

CTCACGAAAGTAACTTTAATACCTCCTGACCCCACGAAAGCTAAGAAA

>330_Sample_ASV34

CACCGCGGTTATACGAGAGACCCTAATTGATAGGTACGGCGTAAAGAGTGGTTTGGGATA

ACCCAAAAATAAAGCTAAAGACCTTCTAAGCCGTCACACGCATACCGAAGGCACGAGGCC

CCAACACGAAAGTAGCTTTACTATTACCCCGACGCCACGAAAGCTAAGAAA

>330_Sample_ASV35

CACCGCGGTTATACGAGAGGCCCTAGTTGATAACTACCGGCGTAAAGAGTGGTTATGGAA

AAATATTTAATAAAGCCGAACACCCCCTCAGCCGTCATACGCACCTGGGAGCACGAAGAC

CTACTGCGAAAGCAGCTTTAATTATGCCTGACCCCACGACAGCTAAGAAA

>330_Sample_ASV36

CACCGCGGTTATACGAGAGGCCCAAGTTGATACTCCACGGCGTAAAGAGTGGTTAGAAAA

GACCTACTACTAAAGCCGAACGCCTTCAAAGCTGTTATACGCATCCGAAGGTGAGAAGCC

CACCCACGAAAGTGGCTTTACAACCTTGACTCCACGAAAGCTGTGAAA

>330_Sample_ASV37

CACCGCGGTTATACGAGAGGCCCAAGTTGATACTCCACGGCGTAAAGAGTGGTTAGAAAA

GACCTACTACTAAAGCCGAACGCCTTCAAAGCTGTTATACGCATCCGAAGGTGAGAAGCC

CACCCACGAAAGTGGCTTTACAACCTTGACTCCACGAAAGCTATGACA

>330_Sample_ASV38

CACCGCGGTTATACGAGAGACCCCAGTTGATAAACACGGCGTAAAGAGTGGTTAAGGGCA

CAAAACAAATAAAGTCAAACAACCTCTTAGCTGTTATACGCATTATGAGGATATGAAGCC

CCCCTACGAAAGTAACTTTACCAACCCCTGACCCCACGAAAGCTAAGAAA

>330_Sample_ASV39

CACCGCGGTTATACGAGAGACCCCAGTTGATAAACACGGCGTAAAGAGTGGTTAAGGGCA

CAAAACAAATAAAGTCAAACAACCTCTTAGCTGTTATACGCATTATGAGGATATGAAGCC

CCCCTACGAAAGTAACTTTACCAACCCCCGACCCCACGAAAGCTGTGAAA

>330_Sample_ASV40

CACCGCGGTTATACGAGAGACCCCAGTTGATAAACACGGCGTAAAGAGTGGTTAAGGGCA

CAAAACAAATAAAGTCAAACAACCTCTTAGCTGTTATACGCATTATGAGGATATGAAGCC

CCCCTACGAAAGTAACTTTACCAACCCCTGACCCCACGAAAGCTGTGAAA

>330_Sample_ASV41

CACCGCGGTTATACGAAAGACCCTAGTTTATAGGCACGGCGTAAAGGGTGGTTAGGGGAT

AAACAAAATAAAGCCAAAGACCTTCTAAGTCGTCATACACTCATGAAGGCACGAAGCCCA

AATACGAAAGTAACTTTACAACTTAGACCCGACTCCACGAAAGCTGGGGGA

>330_Sample_ASV42

CACCGCGGTTATACGAGAGGCCCAAGTTGATAGACATCGGCGTAAAGAGTGGTTAAGGAA

GTCTCAAAACTAAAGCCGAATGCCTTCAAGACTGTTATACGTTTCCGAAGGTAAGAAGCC

CCACTACGAAAGTGGCTTTACTCCACCTGACTCCACGAAAGCTGTGAAA

>330_Sample_ASV43

CACCGCGGTTATACGAGAGACTCCAGTTGATAGCTACGGCGTAAAGAGTGGTTTGGGGCC

CGCCCAAAATAAAGCCAAAGACCTCCCAAGCCGTCAAACGCACCCCGGAGGCACGAAGTC

CTAACGCGAAAGCAACTTTACCTTCCCCGACGCCACGAAAGCTAAGAAA

>330_Sample_ASV44

CACCGCGGTTATACGAAAGACCCTAGTTGATAGCTACGGCGTAAAGGGTGGTTAAGGTAT

ATAACAAATAAAGCTAAAGAGCCTCCAAGCCGTCGTACGCATTCTGAGAGCTCGAAACCC

TAACACGAAAGTAGCTTTAAAAAATAACACCTGACCCCACGAAAGCTAAGAAA

>330_Sample_ASV45

CACCGCGGTTATACGAAAGACCCTAGTTGATAGATCACGGCGTAAAGGGTGGTTAAGGAG

AACAAAAATAAAGCTAAAAATCCTCTAAGCTGTCATACGCTTTCCGAAGACATGAGACCC

AACCACGAAAGTAACTTTAAACTTTCCTGACACCACGAAAGCCAAGAAA

>331_Sample_ASV1

CACCGCGGTTATACGAAAGACCCTAGTTGATGGGTGCGGCGTAAAGGGTGGTTAGGGACT

ACAAAAAATAGAGCTAAAGATCCTCTAGGTCGTCATACACATCACGAGGAAACGAAGCCC

AAATACGAAAGTAGCTCTAAATATAATCCTGACGCCACGAAAGCCAAGAAA

>331_Sample_ASV2

CACCGCGGTTATACGAGAGACCCTAGTTGATTAAAGCGGCGTAAAGAGTGGTTATGGAAC

TATTCTTTTAAAGCAGAAAACCTCTCAAACTGTTATACGCACCCAGAGGTCTGAATCCCT

CACACGAAAGTGACTTTATTTATGCCTACCAGAACCCACGAAAGCTGGGACA

>331_Sample_ASV3

CACCGCGGTTATACGAGAGACCCTAGTTGATATACACGGCGTAAAGAGTGGTTTGGGGTA

TATAATAAATAAAGCCAAAGACCTTCTAGGCCGTTGAACGCACCACGAAGGAGCGAAGTC

CCAATACGAAAGTAGCTTTATAAAACCTGACGCCACGAAAGCTAAGAAA

>331_Sample_ASV4

CATGGCGATTATACGAGAGGCCCAAGTTGACAGACACTGGCGTAAAGAGTGGCTAAGGAA

AAACTACTACTAAAGCCGAACACCTTCAGAACTGTCATACGTTCCCGAAGATAAGAAGCC

CCACCACGAAAGTGACTTTATATTACCCGACCCCACGAAAGCTGTGAAA

>331_Sample_ASV5

CACCGCGGTTATACGAGAGGCCCAAGTTGACAGACACCGGCGTAAAGAGTGGCTAGGGAA

AAATTACTACTAAAGCCGAACACCTTCAGAACTGTCATACGTTCCCGAAGATAAGAAGCC

CCACCACGAAAGTGACTTTATATTACCCGACCCCACGAAAGCTGTGAAA

>331_Sample_ASV6

CACCGCGGTTATACGAAAGACTCTAGTTGACAAACACGGCGTAAAGGGTGGTTAGGATAA

CAACAAAATAAAGCTAAAGACTTACCAAGCCGTCATACGCCCATGTAAGAGCGAAAACCA

CAAACGAAAGTAACTTTATTACACCATCCGAATCCACGAAAGCTAAGAAA

>331_Sample_ASV7

CACCGCGGTTATACGAGAGACCCTAGTTGATATATACGGCGTAAAGAGTGGTTTGGGATA

TTTCTCCAAAATAAAGTCGAAGATCTCCCAAGCCGTCACACGCATCCCGGAGACACGAAG

CCCTGATACGAAGGTAGCTTTATTAACACCCCGACGCCACGAAAGCTAAGAAA

>331_Sample_ASV8

CACCGCGGTTATACGAGAGACCCTAGTTGATATATACGGCGTAAAGAGTGGTTTGGGATA

TTTCTCCAAAATAAAGCCGAAGATCTCCCAAGCCGTCACACGCATCCCGGAGACACGAAG

CCCTGATACGAAGGTAGCTTTATTAACACCCCGACGCCACGAAAGCTAAGAAA

>331_Sample_ASV9

CACCGCGGTTATACGAGAGACCCTAGTTGATATATACGGCGTAAAGAGTGGTTTGGGATA

TTTCTCCAAAATAAAGCCGAAGATCTCCCAAGCCGTCACACGCATCCCGGAGACACGAAG

CCCTGATACGAAGGTAGCTTTATTAATTCCCCGACGCCACGAAAGCTAAGAAA

>331_Sample_ASV10

CACCGCGGTTATACGAGAGGCCCAAGTTGATAAGCCCGGCGTAAAGAGTGGTTATGGAGA

ATAAAACACTAAAGCTAAAGACCCCCTAGGCTGTTATACGCACCTGGCGGCTCGAACCAC

CTATACGAAAGTAGCTTTACCCCCCTTCCACCAGAATCCACGACAGCTGGGGCA

>331_Sample_ASV11

CACCGCGGTTATACGAGAGGCCCAAGTTGACAGACACCGGCGTAAAGAGTGGTTATGGAG

AATAAAACACTAAAGCTAAAGACCCCCTAGGCTGTTATACGCACCTGGCGGCTCGAACCA

CCTATACGAAAGTAGCTTTACCCCCCTTCCACCAGAATCCACGACAGCTGGGGCA

>331_Sample_ASV12

CACCGCGGTTATACGAGAGGCCCAAGTTGACAGACATCGGCGTAAAGAGTGGTTAAGGAA

ACCCCATTACTAAAGCCGAACGCCCTCAGAACTGTCATACGTTCCCGAAGGTAAGAAGCC

CCACTACGAAAGTGGCTTTAACCACACCCGACCCCACGAAAGCTGTGAAA

>331_Sample_ASV13

CACCGCGGTTATACGAGAGACCCCAGTTGATAGCTACGGCGTAAAGAGTGGTTAAGGACC

CCATTTAATAAAGCCAAAGACCCCCCAAGCCGTCATACGCACACGAGGGCGCGAAGCTCG

CACACGAAAGTAGCTCTAATTTACCCGACGCCACGAAAGCTAAGGAA

>331_Sample_ASV14

CACCGCGGTTATACGAGAAACCCTAGTTGACAAATACGGCGTAAAGAGTGGTTAAGGGGT

ATCAAAAATAAAGTCAAACATTTTCCTAGTTGTTATAAGCATTTCGAGAACATGAAGCCC

AACCACGAAAGTAACTTTACCTTCACCTGACCCCACGAAAGCTAAGAAA

>331_Sample_ASV15

GACCGCGGTTATACGAGAGACTCTAGTTGATAGATACGGCGTAAAGAGTGGTTTGGGATA

AACTATAAATAAAGCCAAAGACCTTCCAAGCTGTTAAACGCATACCGAAGGCACGAAGCC

CCAACACGAAAGTAGCTTTACTACTACCCCCGATGCCACGAAAGCTAAGACA

>331_Sample_ASV16

CACCGCGGTTATACGAGAGACCCTAGTTGATAAATACGGCGTAAAGAGTGGTTATGGGAA

AAACAAATAAAGTCAAACAACCTCTTAGCTGTTATACGCATTATGAGAGCATGAAGCCCC

CTCACGAAAGTAACTTTAATACCTCCTGACCCCACGAAAGCTAAGAAA

>331_Sample_ASV17

AACCGCGGTTATACGAGAGACCCTAATTGATAGCTCACGGCGTAAAGGGTGGTTTGGGGA

AACCTCAAAATAAAGCTAAAGACCTTCTAAGCCGTTACACGCATACCGAAGGCACGAGGC

CCTAACACGAAAGTAGCTTTACTATTACCCCTGACGCCACGAAAGCTAAGAAA

>331_Sample_ASV18

CACCGCGGTTATACGAAAGACCCTAGTTGATGGATCACGGCGTAAAGGGTGGTTAAGGGA

TATAAAAATAAAGCTAAAAATCCTCTTAGCTGTCATACGCTTTCCGAAGACATGAGGCCC

AATTACGAAAGTAGCTTTAAACCACCCTGACACCACGAAAGCCAAGAAA

>331_Sample_ASV19

CACCGCGGTTATACGAAAGACCCTAGTTGATGGATCACGGCGTAAAGGGTGGTTAAGGGA

TATAAAAATAAAGCTAAAAATCCTCTTAGCTGTCATACGCTTTCCGAAGACATGAGGCCC

AATTACGAAAGTAGCTTTAAACCACCCTGACACCACGAAAGCTGTGAAA

>331_Sample_ASV20

CACCGCGGTTATACGAGAGGCCCAAGTTGATACTCCACGGCGTAAAGAGTGGTTAGAAAA

GACCTACTACTAAAGCCGAACGCCTTCAAAGCTGTTATACGCATCCGAAGGTGAGAAGCC

CACCCACGAAAGTGGCTTTACAACCTTGACTCCACGAAAGCTATGACA

>331_Sample_ASV21

CACCGCGGTTATACGAGAGACCCCAGTTGATAAACACGGCGTAAAGAGTGGTTAAGGGCA

CAAAACAAATAAAGTCAAACAACCTCTTAGCTGTTATACGCATTATGAGGATATGAAGCC

CCCCTACGAAAGTAACTTTACCAACCCCTGACCCCACGAAAGCTAAGAAA

>331_Sample_ASV22

CACCGCGGTTATACGAAAGACCCTAGTTTATAGGCACGGCGTAAAGGGTGGTTAGGGGAT

AAACAAAATAAAGCCAAAGACCTTCTAAGTCGTCATACACTCATGAAGGCACGAAGCCCA

AATACGAAAGTAACTTTACAACTTAGACCCGACTCCACGAAAGCTGGGGGA

>331_Sample_ASV23

CACCGCGGTTATACGAGAGACCCTAATTAATAGATACGGCGTAAAGAGTGGTTTAGGACA

AAAATTAATAAAGCCGAAGTTTCTCCTAGCCGTTGTACGCACTCTGAGAACACGAAGCCC

CACTACGAAAGTAGCTTTACCTATTATTTCCTGACCCCACGAAAGCTAAGAAA

>331_Sample_ASV24

CACCGCGGTTATACGAGAGGCCCAAGTTGATAGACATCGGCGTAAAGAGTGGTTAAGGAA

GTCTCAAAACTAAAGCCGAATGCCTTCAAGACTGTTATACGTTTCCGAAGGTAAGAAGCC

CCACTACGAAAGTGGCTTTACTCCACCTGACTCCACGAAAGCTGTGAAA

>331_Sample_ASV25

CACCGCGGTTATACGAGAGGCCCAAGTTGATAGACATCGGCGTAAAGAGTGGTTAAGGAA

GTCTCAAAACTAAAGCCGAATGCCTTCAAGACTGTTATACGTTTCCGAAGGTAAGAAGCC

CCACTACGAAAGTGGCTTTATTCCACCTGACCCCACGAAAGCTGTGAAA

>331_Sample_ASV26

CACCGCGGTTATACGAAAGACCCTAGTTGATAGATCACGGCGTAAAGGGTGGTTAAGGAA

AACAAAAATAAAGCTAAAAATCCTCTAAGCTGTCATACGCTTTCCGAAGACATGAGATCC

AACCACGAAAGTAGCTTTAAAATTTCCTGACACCACGAAAGCCAAGGAA

>332_Sample_ASV1

CACCGCGGTTATACGAAAGACCCTAGTTGATGGGTGCGGCGTAAAGGGTGGTTAGGGACT

ACAAAAAATAGAGCTAAAGATCCTCTAGGTCGTCATACACATCACGAGGAAACGAAGCCC

AAATACGAAAGTAGCTCTAAATATAATCCTGACGCCACGAAAGCCAAGAAA

>332_Sample_ASV2

CACCGCGGTTAAACGAGAGGCCCTAGTTGATGGTGCAACGGCGTAAAGGGTGGTTAAGGA

TAGCGAAATAATAAAGTCAAATGGCCCTCTGGCTGTCATACGCTTCTAGGAGTCCGAAGC

CCAGTATACGAAAGTAGCTTTAAGAAAGTCCACCTGACCCCACGAAAACTGAGGAA

>332_Sample_ASV3

CACCGCGGTTATACGAAAGACCCTAGTTGATTAAAGCGGCGTAAAGAGTGGTTATGGAAC

TATTCTTTTAAAGCAGAAAACCTCTCAAACTGTTATACGCACCCAGAGGTCTGAATCCCT

CACACGAAAGTGACTTTATTTATGCCTACCAGAACCCACGAAAGCTGGGGGA

>332_Sample_ASV4

CACCGCGGTTATACGAGAGACCCTAGTTGATTAAAGCGGCGTAAAGAGTGGTTATGGAAC

TATTCTTTTAAAGCAGAAAACCTCTCAAACTGTTATACGCACCCAGAGGTCTGAATCCCT

CACACGAAAGTGACTTTATTTATGCCTACCAGAACCCACGAAAGCTGGGGGA

>332_Sample_ASV5

CACCGCGGTTATACGAGAGACCCTAGTTGATTAAAGCGGCGTAAAGAGTGGTTATGGAAC

TATTCTTTTAAAGCAGAAAACCTCTCAAACTGTTATACGCACCCAGAGGTCTGAATCCCT

CACACGAAAGTGACTTTATTTATGCCTACCAGAACCCACGAAAGCTGGGACA

>332_Sample_ASV6

CACCGCGGTTATACGAAAGACCCTAGTTTATAGGTACGGCGTAAAGGGTGGTTAGGGGAC

AAACAAAATAAAGCCAAAGACCTCCTAAGCCGTCATACGCTCACGGAAGCACGAAGCCCA

AACACGAAAGTAGCTTTACCGAACATGCCCGACTCCACGAAAGCTGGGGAA

>332_Sample_ASV7

CACCGCGGTTATACGAAAGACCCTAGTTTATAGGCACGGCGTAAAGGGTGGTTAGGGGAT

AAACAAAATAAAGCCAAAGACCTCCTAAGCCGTCATACGCTCACGGAAGCACGAAGCCCA

AACACGAAAGTAGCTTTACCGAACATGCCCGACTCCACGAAAGCTGGGGAA

>332_Sample_ASV8

CACCGCGGTTATACGAAAGACCCTAGTTTATAGGCACGGCGTAAAGGGTGGTTAGGGGAT

AAACAAAATAAAGCCAAAGACCTTCTAAGTCGTCATACGCTCACGGAAGCACGAAGCCCA

AACACGAAAGTAGCTTTACCGAACATGCCCGACTCCACGAAAGCTGGGGAA

>332_Sample_ASV9

CACCGCGGTTATACGAGAGACCCTAGTTGATATACACGGCGTAAAGAGTGGTTTGGGGTA

TATAATAAATAAAGCCAAAGACCTTCTAGGCCGTTGAACGCACCACGAAGGAGCGAAGTC

CCAATACGAAAGTAGCTTTATAAAACCTGACGCCACGAAAGCTAAGAAA

>332_Sample_ASV10

CACCGCGGTTATACGAGAGGCCCAAGTTGACAGACACCGGCGTAAAGAGTGGCTAGGGAA

AAATTACTACTAAAGCCGAACACCTTCAGAACTGTCATACGTTCCCGAAGATAAGAAGCC

CCACCACGAAAGTGACTTTATATTACCCGACCCCACGAAAGCTGTGAAA

>332_Sample_ASV11

CACCGCGGTTATACGAGAGGCCCAAGTTGACAGACACCGGCGTAAAGAGTGGCTAGGGAA

AAATTACTACTAAAGCCGAACACCTTCAGAACTGTCATACGTTCCCGAAGATAAGAAGCC

CCACCACGAAAGTGACTTTATATTACCCGACCCCACGAAAGCTGGGGGA

>332_Sample_ASV12

CACCGCGATTATACGAGAGGCCCAAGTTGACAGACATCGGCGTAAAGAGTGGTTAAGGAA

AAATTACTACTAAAGCCGAACACCTTCAGAACTGTCATACGTTCCCGAAGATAAGAAGCC

CCACCACGAAAGTGGCTTTATATTACCCGACCCCACGAAAGCTGTGAAA

>332_Sample_ASV13

CACCGCGGTTATACGAGAGGCCCAAGTTGACAGACACCGGCGTAAAGAGTGGCTAGGGAA

AAATTACTACTAAAGCCGAACACCTTCAGAACTGTCATACGTTCCCGAAGATAAGAAGCC

CCACCACGAAAGTGGCTTTATATTACCCGACCCCACGAAAGCTGTGAAA

>332_Sample_ASV14

CACCGCGATTATACGAGAGGCCCAAGTTGACAGACATCGGCGTAAAGAGTGGCTAGGGAA

AAATTACTACTAAAGCCGAACACCTTCAGAACTGTCATACGTTCCCGAAGATAAGAAGCC

CCACCACGAAAGTGGCTTTATATTACCCGACCCCACGAAAGCTGTGAAA

>332_Sample_ASV15

CACCGCGGTTATACGAGAGGCCCAAGTTGACAGACACCGGCGTAAAGAGTGGCTAAGGAA

AAATTTCTACTAAAGCCGAACACCTTCAGAACTGTCATACGTTCCCGAAGATAAGAAGCC

CCACCACGAAAGTGGCTTTATATTACCCGACACCACGAAAGCTGTGAAA

>332_Sample_ASV16

CACCGCGGTTATACGAGAGGCCCAAGTTGACAGACACCGGCGTAAAGAGTGGCTAAGGAA

AAATTTCTACTAAAGCCGAACACCTTCAGAACTGTCATACGTTCCCGAAGAAAAGAAGCC

CCACCACGAAAGTGGCTTTATATTACCCGACCCCACGAAAGCTGTGAAA

>332_Sample_ASV17

CACCGCGGTTATACGAAAGACCCTAGTTTATAGAAACGGCGTAAAGGGTGGTTAAGGGAC

AAACAAAATAAGGCCAAAGACTTCCTAAGCCGTCATACGCTCCACGAAAGCACGAAGCCC

AAACACGAAAGTAGCCTTAATAACTTCACCTGACCCCACGAAAGCTGGGAGA

>332_Sample_ASV18

CACCGCGGTTATACGAAAGACCCTAGTTTATAGGCACGGCGTAAAGGGTGGTTAAGGGAC

AAACAAAATAAGGCCAAAGACTTCCTAAGCCGTCATACGCTCCACGAAAGCACGAAGCCC

AAACACGAAAGTAGCCTTAATAACTTCACCTGACCCCACGAAAGCTGGGAGA

>332_Sample_ASV19

CACCGCGGTTATACGAGAGGCCCAAGTTGATAAGCCCGGCGTAAAGAGTGGTTATGGAGA

ATAAAACACTAAAGCTAAAGACCCCCTAGGCTGTTATACGCACCTGGCGGCTCGAACCAC

CTATACGAAAGTAGCTTTACCCCCCTTCCACCAGAATCCACGACAGCTGGGGCA

>332_Sample_ASV20

CACCGCGGTTATACGAGAGGCCCAAGTTGACAGACATCGGCGTAAAGAGTGGTTAAGGAA

ACCCCATTACTAAAGCCGAACGCCCTCAGAACTGTCATACGTTCCCGAAGGTAAGAAGCC

CCACTACGAAAGTGGCTTTAACCACACCCGACCCCACGAAAGCTGTGAAA

>332_Sample_ASV21

CACCGCGGTTATACGAGAGGCCCAAGTTGACAGACATCGGCGTAAAGAGTGGTTAAGGAA

ACCCCATTACTAAAGCCGAACGCCCTCAGAACTGTCATACGTTCCCGAAGGTAAGAAGCC

CCACTACGAAAGTGGCTTTAACCACACCCGACCCCACGAAAGCTGGGGGA

>332_Sample_ASV22

CACCGCGGTTATACGAGAGGCCCAAGTTGACAGACATCGGCGTAAAGAGTGGTTAAGGAA

ACCCCATTACTAAAGCCGAACGCCCTCAGAACTGTCATACGTTCCCGAAGGTAAGAAGCC

CCACTACGAAAGTGGCTTTAACCACACCCGACTCCACGAAAGCTGGGGGA

>332_Sample_ASV23

CACCGCGGTTATACGAGAGGCCCAAGTTGATAGATATCGGCGTAAAGAGTGGTTAAGGAA

ACCCCGTTACTAAAGCCGAACGCCCTCAGAACTGTCATACGTTCCCGAAGGTAAGAAGCC

CCACTACGAAAGTGGCTTTAACCACACCCGACCCCACGAAAGCTGTGAAA

>332_Sample_ASV24

CACCGCGGTTAAACGAGAGGCCCTAGTTGATATTACAACGGCGTAAAGGGTGGTTAAGGA

AAGCAAGCAATAAAGCCGAATGGCCCTTTGGCTGTCATACGCTTCTAGGTGTCCGAAGCC

CAATATACGAAAGTAGCTTTAGAAAAGCCCACCTGACACCACGAAAGCTGAGGAA

>332_Sample_ASV25

CACCGCGGTCACACGATTAACCCAAGTCAATAGAAGCCGGCGTAAAGAGTGTTTTAGATC

ACCCCCTCCCCAATAAAGCTAAAACTCACCTGAGTTGTAAAAAACTCCAGTTGACACAAA

ATAGACTACGAAAGTGGCTTTAACATATCTGAACACACAATAGCTAAGACC

>332_Sample_ASV26

CACCGCGGTTATACGAGAAACCCTAGTTGACAAATACGGCGTAAAGAGTGGTTAAGGGGT

ATCAAAAATAAAGTCAAACATTTTCCTAGTTGTTATAAGCATTTCGAGAACATGAAGCCC

AACCACGAAAGTAACTTTACCTTCACCTGACCCCACGAAAGCTAAGAAA

>332_Sample_ASV27

CACCGCGGTTATACGAAAGACCCCAGTTTATAGACACGGCGTAAAGGGTGGTTATAGGAC

AAACTAACATAGAGCCAAAGACCCCCTAAGCCGTCATACGCTCACGAGGGCTCGAAGCCC

CTATACGAAAGTAGCTCTACCAAAAAATGCTCGACTCCACGAAAGCTGGGACA

>332_Sample_ASV28

CACCGCGGTTATACGAGAGACCCTAGTTGATAAATACGGCGTAAAGAGTGGTTATGGGAA

AAACAAATAAAGTCAAACAACCTCTTAGCTGTTATACGCATTATGAGAGCATGAAGCCCC

CTCACGAAAGTAACTTTAATACCTCCTGACCCCACGAAAGCTAAGAAA

>332_Sample_ASV29

CACCGCGGTTATACGAGAGGCTCAAGTTGATAAACCCCGGCGTAAAGAGTGGTTAAGGAA

AAATAGAAACTAAAGCCGAATGCTTTCAAGACTGTTATACGTTCCCGAGAGTAAGAAGAC

CAATTACGAAAGTGGCTTTACTTTACCTGACCCCACGAAAGCTACGACA

>332_Sample_ASV30

AACCGCGGTTATACGAGAGACCCTAATTGATAGCTCACGGCGTAAAGGGTGGTTTGGGGA

AACCTCAAAATAAAGCTAAAGACCTTCTAAGCCGTTACACGCATACCGAAGGCACGAGGC

CCTAACACGAAAGTAGCTTTACTATTACCCCTGACGCCACGAAAGCTAAGAAA

>332_Sample_ASV31

CACCGCGGTTATACGAAAGACCCTAGTTGATGGATCACGGCGTAAAGGGTGGTTAAGGGA

TATAAAAATAAAGCTAAAAATCCTCTTAGCTGTCATACGCTTTCCGAAGACATGAGGCCC

AATTACGAAAGTAGCTTTAAACCACCCTGACACCACGAAAGCCAAGAAA

>332_Sample_ASV32

CACCGCGGTTATACGAGAGGCCCTAGTTGATAACTACCGGCGTAAAGAGTGGTTATGGAA

AAATATTTAATAAAGCCGAACACCCCCTCAGCCGTCATACGCACCTGGGAGCACGAAGAC

CTACTGCGAAAGCAGCTTTAATTATGCCTGACCCCACGACAGCTAAGAAA

>332_Sample_ASV33

CACCGCGGTTATACGAGAGGCCCAAGTTGATACTCCACGGCGTAAAGAGTGGTTAGAAAA

GACCTACTACTAAAGCCGAACGCCTTCAAAGCTGTTATACGCATCCGAAGGTGAGAAGCC

CACCCACGAAAGTGGCTTTACAACCTTGACTCCACGAAAGCTATGACA

>332_Sample_ASV34

CACCGCGGTTATACGAGAGACCCCAGTTGATAAACACGGCGTAAAGAGTGGTTAAGGGCA

CAAAACAAATAAAGTCAAACAACCTCTTAGCTGTTATACGCATTATGAGGATATGAAGCC

CCCCTACGAAAGTAACTTTACCAACCCCTGACCCCACGAAAGCTAAGAAA

>332_Sample_ASV35

CACCGCGGTTATACGAAAGACCCTAGTTTATAGGCACGGCGTAAAGGGTGGTTAGGGGAT

AAACAAAATAAAGCCAAAGACCTTCTAAGTCGTCATACACTCATGAAGGCACGAAGCCCA

AATACGAAAGTAACTTTACAACTTAGACCCGACTCCACGAAAGCTGGGGGA

>332_Sample_ASV36

CACCGCGGTTATACGAGAGACCCTAATTAATAGATACGGCGTAAAGAGTGGTTTAGGACA

AAAATTAATAAAGCCGAAGTTTCTCCTAGCCGTTGTACGCACTCTGAGAACACGAAGCCC

CACTACGAAAGTAGCTTTACCTATTATTTCCTGACCCCACGAAAGCTAAGAAA

>332_Sample_ASV37

CACCGCGGTTATACGAGAGGCCCAAGTTGATAGACATCGGCGTAAAGAGTGGTTAAGGAA

GTCTCAAAACTAAAGCCGAATGCCTTCAAGACTGTTATACGTTTCCGAAGGTAAGAAGCC

CCACTACGAAAGTGGCTTTACTCCACCTGACTCCACGAAAGCTGTGAAA

>332_Sample_ASV38

CACCGCGGTTATACGAGAGGCCCAAGTTGATAGACATCGGCGTAAAGAGTGGTTAAGGAA

GTCTCAAAACTAAAGCCGAATGCCTTCAAGACTGTTATACGTTTACGAAGGTAAGAAGCC

CCACTACGAAAGTGGCTTTATTCCACCTGACCCCACGAAAGCTGTGAAA

>332_Sample_ASV39

CACCGCGGTTATACGAGAGGCCCAAGTTGATAGACATCGGCGTAAAGAGTGGTTAAGGAA

GTCTCAAAACTAAAGCCGAATGCCTTCAAGACTGTTATACGTTTCCGAAGGTAAGAAGCC

CCACTACGAAAGTGGCTTTATTCCACCTGACCCCACGAAAGCTGTGAAA

>332_Sample_ASV40

CACCGCGGTTATACGAGAGGCCCAAGTTGACAAACTCCGGCGTAAAGCGTGGTTAAGGCA

CACTCAAAAACTAAAGCCGAATATCTTCAGAGCAGTCATACGCATCCGAAGACACGAAGC

CCCACCACGAAAGTGGCTTTATGACACCTGACCCCACGAAAGCTAGGACA

>332_Sample_ASV41

CACCGCGGTCATACGATTAACCCAAATTAATAGATCCACGGCGTAAAGAGTGTTTAAGaa

aaaaaaaCCACAATAGAGTTAAATTATAACTAAGCTGTAAAAAGCCCTAGTTAAAATAAA

ATAACCCACGAAAGTGACTCTAATAATCCTGACACACGATAGCTAGGACC

>332_Sample_ASV42

CACCGCGGTTATACGAAAGACCCTAGTTGATAGATCACGGCGTAAAGGGTGGTTAAGGAA

AACAAAAATAAAGCTAAAAATCCTCTAAGCTGTCATACGCTTTCCGAAGACATGAGATCC

AACCACGAAAGTAGCTTTAAAATTTCCTGACACCACGAAAGCCAAGGAA

>335_Sample_ASV1

CACCGCGGTTATACGAAAGACCCTAGTTGATGGGTGCGGCGTAAAGGGTGGTTAGGGACT

ACAAAAAATAGAGCTAAAGATCCTCTAGGTCGTCATACACATCACGAGGAAACGAAGCCC

AAATACGAAAGTAGCTCTAAATATAATCCTGACGCCACGAAAGCCAAGAAA

>335_Sample_ASV2

CACCGCGGTTAAACGAGAGGCCCTAGTTGATGGTGCAACGGCGTAAAGGGTGGTTAAGGA

TAGCGAAATAATAAAGTCAAATGGCCCTCTGGCTGTCATACGCTTCTAGGAGTCCGAAGC

CCAGTATACGAAAGTAGCTTTAAGAAAGTCCACCTGACCCCACGAAAACTGAGGAA

>335_Sample_ASV3

CACCGCGGTTATACGAGAGACCCTAGTTGATTAAAGCGGCGTAAAGAGTGGTTATGGAAC

TATTCTTTTAAAGCAGAAAACCTCTCAAACTGTTATACGCACCCAGAGGTCTGAATCCCT

CACACGAAAGTGACTTTATTTATGCCTACCAGAACCCACGAAAGCTGGGACA

>335_Sample_ASV4

CACCGCGGTTATACGAAAGACCCTAGTTTATAGGTACGGCGTAAAGGGTGGTTAGGGGAC

AAACAAAATAAAGCCAAAGACCTCCTAAGCCGTCATACGCTCACGGAAGCACGAAGCCCA

AACACGAAAGTAGCTTTACCGAACATGCCCGACTCCACGAAAGCTGGGGAA

>335_Sample_ASV5

CACCGCGGTTATACGAGAGGCCCAAGTTGACAGACACCGGCGTAAAGAGTGGCTAGGGAA

AAATTACTACTAAAGCCGAACACCTTCAGAACTGTCATACGTTCCCGAAGATAAGAAGCC

CCACCACGAAAGTGGCTTTATATTACCCGACCCCACGAAAGCTGTGAAA

>335_Sample_ASV6

CACCGCGGTTATACGAGAGGCCCAAGTTGACAGACACCGGCGTAAAGAGTGGCTAAGGAA

AAATTTCTACTAAAGCCGAACACCTTCAGAACTGTCATACGTTCCCGAAGAAAAGAAGCC

CCACCACGAAAGTGGCTTTATATTACCCGACCCCACGAAAGCTGTGAAA

>335_Sample_ASV7

CACCGCGGTTATACGAGAGGCCCAAGTTGATAAGCCCGGCGTAAAGAGTGGTTATGGAGA

ATAAAACACTAAAGCTAAAGACCCCCTAGGCTGTTATACGCACCTGGCGGCTCGAACCAC

CTATACGAAAGTAGCTTTACCCCCCTTCCACCAGAATCCACGACAGCTGGGGCA

>335_Sample_ASV8

CACCGCGGTTATACGAGAGGCCCTAGTTGATAGCCACGGCGCAAAGAGTGGTTAAGGAGC

CCCACTAAATAAAGTCGAACACTTCCTAGGCCGTTATACGTTTTCTAGAAGCACGAAACC

CAATTACACGAAAGCAACTTTATACTAAACAACCTGACCCCACGAAAGCTAAGAAA

>335_Sample_ASV9

CACCGCGGTTATACGAGAGGCCCAAGTTGACAGACATCGGCGTAAAGAGTGGTTAAGGAA

ACCCCATTACTAAAGCCGAACGCCCTCAGAACTGTCATACGTTCCCGAAGGTAAGAAGCC

CCACTACGAAAGTGGCTTTAACCACACCCGACCCCACGAAAGCTGTGAAA

>335_Sample_ASV10

CACCGCGGTTATACGAGAGGCCCAAGTTGATAGATATCGGCGTAAAGAGTGGTTAAGGAA

ACCCCGTTACTAAAGCCGAACGCCCTCAGAACTGTCATACGTTCCCGAAGGTAAGAAGCC

CCACTACGAAAGTGGCTTTAACCACACCCGACCCCACGAAAGCTGTGAAA

>335_Sample_ASV11

CACCGCGGTTAAACGAGAGGCCCTAGTTGATATTACAACGGCGTAAAGGGTGGTTAAGGA

AAGCAAGCAATAAAGCCGAATGGCCCTTTGGCTGTCATACGCTTCTAGGTGTCCGAAGCC

CAATATACGAAAGTAGCTTTAGAAAAGCCCACCTGACACCACGAAAGCTGAGGAA

>335_Sample_ASV12

CACCGCGGTTATACGAAAGACCCCAGTTTATAGACACGGCGTAAAGGGTGGTTATAGGAC

AAACTAACATAGAGCCAAAGACCCCCTAAGCCGTCATACGCTCACGAGGGCTCGAAGCCC

CTATACGAAAGTAGCTCTACCAAAAAATGCTCGACTCCACGAAAGCTGGGACA

>335_Sample_ASV13

CACCGCGGTTATACGAGAGACCCTAGTTGATAAATACGGCGTAAAGAGTGGTTATGGGAA

AAACAAATAAAGTCAAACAACCTCTTAGCTGTTATACGCATTATGAGAGCATGAAGCCCC

CTCACGAAAGTAACTTTAATACCTCCTGACCCCACGAAAGCTAAGAAA

>335_Sample_ASV14

CACCGCGGTTATACGAGAGGCTCAAGTTGATAAACCCCGGCGTAAAGAGTGGTTAAGGAA

AAATAGAAACTAAAGCCGAATGCTTTCAAGACTGTTATACGTTCCCGAGAGTAAGAAGAC

CAATTACGAAAGTGGCTTTACTTTACCTGACCCCACGAAAGCTACGACA

>335_Sample_ASV15

AACCGCGGTTATACGAGAGACCCTAATTGATAGCTCACGGCGTAAAGGGTGGTTTGGGGA

AACCTCAAAATAAAGCTAAAGACCTTCTAAGCCGTTACACGCATACCGAAGGCACGAGGC

CCTAACACGAAAGTAGCTTTACTATTACCCCTGACGCCACGAAAGCTAAGAAA

>335_Sample_ASV16

CACCGCGGTTATACGAGAGACCCTAATTGATAGCTCACGGCGTAAAGGGTGGTTTGGGGA

AACCTCAAAATAAAGCTAAAGACCTTCTAAGCCGTTACACGCATACCGAAGGCACGAGGC

CCTAACACGAAAGTAGCTTTACTATTACCCCTGACGCCACGAAAGCTAAGAAA

>335_Sample_ASV17

CACCGCGGTTATACGAAAGACCCTAGTTGATGGATCACGGCGTAAAGGGTGGTTAAGGGA

TATAAAAATAAAGCTAAAAATCCTCTTAGCTGTCATACGCTTTCCGAAGACATGAGGCCC

AATTACGAAAGTAGCTTTAAACCACCCTGACACCACGAAAGCCAAGAAA

>335_Sample_ASV18

CACCGCGGTTATACGAGAGGCCCTAGTTGATAACTACCGGCGTAAAGAGTGGTTATGGAA

AAATATTTAATAAAGCCGAACACCCCCTCAGCCGTCATACGCACCTGGGAGCACGAAGAC

CTACTGCGAAAGCAGCTTTAATTATGCCTGACCCCACGACAGCTAAGAAA

>335_Sample_ASV19

CACCGCGGTTATACGAGAGGCCCAAGTTGATTATTACCGGCGTAAAGCGTGGTTAAGATT

TTTATAGGACTAAAGCCGAACCCCCTCAGAGCTGTTATACGCACCCGAAGGTTAGAAGTT

CAATCACGAAAGTGGCTTTACAACCCCGAACCCACGAAAGCTAAGATA

>335_Sample_ASV20

CACCGCGGTTATACGAGAGACCCTAATTGATAGGCGCGGCGTAAAGAGTGGTTTAGGACT

AAAACTAATAAAGCTAAAGATCCTCCCAGCCGTCGCACGCATTCTGAGGACACGAAACTC

AACCACGAAAGTAGCTTTAACATACTCTCCTGACCCCACGAAAGCTAAGAAA

>335_Sample_ASV21

CACCGCGGTTATACGAAAGACCCTAGTTGATAGGCACGGCGTAAAGGGTGGTTAGGGATA

TATCAAAAATAAAGTTAAAGAGCCTCTAAGCTGTCGCACGCATCCCGAGAGCTCGAGACC

CAGACACGAAAGTAACTTTAAAATACCTCACCTGACCCCACGAAAGCTAAGAAA

>335_Sample_ASV22

CACCGCGGTTATACGAGAGGCCCAAGTTGATACTCCACGGCGTAAAGAGTGGTTAGAAAA

GACCTACTACTAAAGCCGAACGCCTTCAAAGCTGTTATACGCATCCGAAGGTGAGAAGCC

CACCCACGAAAGTGGCTTTACAACCTTGACTCCACGAAAGCTATGACA

>335_Sample_ASV23

CACCGCGGTTATACGAGAGACCCCAGTTGATAAACACGGCGTAAAGAGTGGTTAAGGGCA

CAAAACAAATAAAGTCAAACAACCTCTTAGCTGTTATACGCATTATGAGGATATGAAGCC

CCCCTACGAAAGTAACTTTACCAACCCCTGACCCCACGAAAGCTAAGAAA

>335_Sample_ASV24

CACCGCGGTTATACGAAAGACCCTAGTTTATAGGCACGGCGTAAAGGGTGGTTAGGGGAT

AAACAAAATAAAGCCAAAGACCTTCTAAGTCGTCATACACTCATGAAGGCACGAAGCCCA

AATACGAAAGTAACTTTACAACTTAGACCCGACTCCACGAAAGCTGGGGGA

>335_Sample_ASV25

CACCGCGGTTATACGAGAGACCCTAATTAATAGATACGGCGTAAAGAGTGGTTTAGGGTA

AAAATTAATAAAGCCGAAGTTTCTCCTAGCCGTTGTACGCACTCTGAGAACACGAAGCCC

CACTACGAAAGTAGCTTTACCTATTATTTCCTGACCCCACGAAAGCTAAGAAA

>335_Sample_ASV26

CACCGCGGTTATACGAGAGACCCTAATTAATAGCTACGGCGTAAAGAGTGGTTTAGGACA

AAAATTAATAAAGCCGAAGTTTCTCCTAGCCGTTGTACGCACTCTGAGAACACGAAGCCC

CACTACGAAAGTAGCTTTACCTATTATTTCCTGACCCCACGAAAGCTAAGAAA

>335_Sample_ASV27

CACCGCGGTTATACGAGAGACCCTAATTAATAGCTACGGCGTAAAGAGTGGTTTAGGGTA

AAAATTAATAAAGCCGAAGTTTCTCCTAGCCGTTGTACGCACTCTGAGAACACGAAGCCC

CACTACGAAAGTAGCTTTACCTATTATTTCCTGACCCCACGAAAGCTAAGAAA

>335_Sample_ASV28

CACCGCGGTTATACGAGAGACCCTAATTAATAGATACGGCGTAAAGAGTGGTTTAGGACA

AAAATTAATAAAGCCGAAGTTTCTCCTAGCCGTTGTACGCACTCTGAGAACACGAAGCCC

CACTACGAAAGTAGCTTTACCTATTATTTCCTGACCCCACGAAAGCTAAGAAA

>335_Sample_ASV29

CACCGCGGTTATACGAGAGGCCCAAGTTGACAAACTCCGGCGTAAAGCGTGGTTAAGGCA

CACTCAAAAACTAAAGCCGAATATCTTCAGAGCAGTCATACGCATCCGAAGACACGAAGC

CCCACCACGAAAGTGGCTTTATGACACCTGACCCCACGAAAGCTAGGACA

>335_Sample_ASV30

CACCGCGGTTATACGAAAGACCCTAGTTGATAGATCACGGCGTAAAGGGTGGTTAAGGAA

AACAAAAATAAAGCTAAAAATCCTCTAAGCTGTCATACGCTTTCCGAAGACATGAGATCC

AACCACGAAAGTAGCTTTAAAATTTCCTGACACCACGAAAGCCAAGGAA

>336_Sample_ASV1

CACCACGGTTATACGAGAGGCCCAAGTTGAAAGACATCGGCGTAAAGAGTGGTTAAGGAA

AACCTTCTACTAAAGTCGAACGCTCTCAGGACTGTCGTACGTTTCCGAGAGTATGAAGCC

CCACCACGAAAGTGACTTTACCTCACCTGACTCCACGAAAGCTGCGAAA

>336_Sample_ASV2

CACCGCGGTTATACGAGAGGCCCAAGTTGACAGACATCGGCGTAAAGAGTGGTTAAGGAA

AACCTTCAACTAAAGTCGAACGCTCTCAAGACTGTCGTACGTTTCCGAGAGTATGAAGCC

CCACCACGAAAGTGACTTTACCTCACCTGACTCCACGAAAGCTGCGAAA

>336_Sample_ASV3

CACCGCGGTTATACGAAAGACCCTAGTTGATGGGTGCGGCGTAAAGGGTGGTTAGGGACT

ACAAAAAATAGAGCTAAAGATCCTCTAGGTCGTCATACACATCACGAGGAAACGAAGCCC

AAATACGAAAGTAGCTCTAAATATAATCCTGACGCCACGAAAGCCAAGAAA

>336_Sample_ASV4

CACCGCGGTTATACGAGAGACCCTAGTTGATTAAAGCGGCGTAAAGAGTGGTTATGGAAC

TATTCTTTTAAAGCAGAAAACCTCTCAAACTGTTATACGCACCCAGAGGTCTGAATCCCT

CACACGAAAGTGACTTTATTTATGCCTACCAGAACCCACGAAAGCTGGGACA

>336_Sample_ASV5

CACCGCGGTTATACGAAAGACCCTAGTTTATAGGTACGGCGTAAAGGGTGGTTAGGGGAC

AAACAAAATAAAGCCAAAGACCTCCTAAGCCGTCATACGCTCACGGAAGCACGAAGCCCA

AACACGAAAGTAGCTTTACCGAACATGCCCGACTCCACGAAAGCTGGGGAA

>336_Sample_ASV6

CACCGCGGTTATACGAAAGACCCTAGTTGATAGCCACGGCGTAAAGGGTGGTTAAGGAAC

CTAAAAAATAAAGCTAAAGACCCTCTAGGCCGTCATACGCATTACGAGGCAAACGAAACC

CCAACACGAAAGTAGCTTTAGACAAATATCCTGATGCCACGAAAGCCAAGAAA

>336_Sample_ASV7

CACCGCGGTTATACGAGAGGCCCAAGTTGACAGACACCGGCGTAAAGAGTGGCTAGGGAA

AAATTACTACTAAAGCCGAACACCTTCAGAACTGTCATACGTTCCCGAAGATAAGAAGCC

CCACCACGAAAGTGACTTTATATTACCCGACCCCACGAAAGCTGGGGGA

>336_Sample_ASV8

CACCGCGGTTATACGAGAGGCCCAAGTTGACAGACACCGGCGTAAAGAGTGGCTAGGGAA

AAATTACTACTAAAGCCGAACACCTTCAGAACTGTCATACGTTCCCGAAGATAAGAAGCC

CCACCACGAAAGTGACTTTATATTACCCGACCCCACGAAAGCTGTGAAA

>336_Sample_ASV9

CACCGCGGTTATACGAGAGGCCCAAGTTGACAGACACCGGCGTAAAGAGTGGCTAGGGAA

AAATTACTACTAAAGCCGAACACCTTCAGAACTGTCATACGTTCCCGAAGATAAGAAGCC

CCACCACGAAAGTGGCTTTATATTACCCGACCCCACGAAAGCTGGGGGA

>336_Sample_ASV10

CACCGCGGTTATACGAGAGGCCCAAGTTGACAGACACCGGCGTAAAGAGTGGCTAGGGAA

AAATTACTACTAAAGCCGAACACCCTCAGAACTGTCATACGTTCCCGAAGGTAAGAAGCC

CCACCACGAAAGTGGCTTTATAATACCCGACCCCACGAAAGCTGTGAAA

>336_Sample_ASV11

CACCGCGGTTATACGAGAGGCCCAAGTTGACAGACACCGGCGTAAAGAGTGGCTAGGGAA

AAATTACTACTAAAGCCGAACACCTTCAGAACTGTCATACGTTCCCGAAGATAAGAAGCC

CCACCACGAAAGTGGCTTTATATTACCCGACCCCACGAAAGCTGTGAAA

>336_Sample_ASV12

CACCGCGGTTATACGAGAGGCCCAAGTTGACAGACACCGGCGTAAAGAGTGGCTAAGGAA

AAATTTCTACTAAAGCCGAACACCTTCAGAACTGTCATACGTTCCCGAAGATAAGAAGCC

CCACCACGAAAGTGGCTTTATATTACCCGACACCACGAAAGCTGTGAAA

>336_Sample_ASV13

CACCGCGGTTATACGAAAGACTCTAGTTGACAAACACGGCGTAAAGGGTGGTTAGGATAA

CAACAAAATAAAGCTAAAGACTTACCAAGCCGTCATACGCCCATGTAAGAGCGAAAACCA

CAAACGAAAGTAACTTTATTACACCATCCGAATCCACGAAAGCTAAGAAA

>336_Sample_ASV14

CACCGCGGTTAAACGAGAGACCCTAGTTGATCATCACGGCGTAAAGAGTGGTTAGGGGAT

TATCATAAATAAAGCCAAAGACCTCCCAAGCTGTCGCACGCATTCCGGAGGCGCGAAGCC

CACACACGAAAGTAGCTTTAACTATTGACCCTGATGCCACGAAAGCTAAGAAA

>336_Sample_ASV15

CACCGCGGTTATACGAGAGGCCCAAGTTGATAAGCCCGGCGTAAAGAGTGGTTATGGAGA

ATAAAACACTAAAGCTAAAGACCCCCTAGGCTGTTATACGCACCTGGCGGCTCGAACCAC

CTATACGAAAGTAGCTTTACCCCCCTTCCACCAGAATCCACGACAGCTGGGGCA

>336_Sample_ASV16

CACCGCGGTTATACGAGAGGCCCAAGTTGACAGACATCGGCGTAAAGAGTGGTTAAGGAA

ACCCCATTACTAAAGCCGAACGCCCTCAGAACTGTCATACGTTCCCGAAGGTAAGAAGCC

CCACTACGAAAGTGGCTTTAACCACACCCGACCCCACGAAAGCTGTGAAA

>336_Sample_ASV17

CACCGCGGTTATACGAGAGGCCCAAGTTGATAGATATCGGCGTAAAGAGTGGTTAAGGAA

ACCCCGTTACTAAAGCCGAACGCCCTCAGAACTGTCATACGTTCCCGAAGGTAAGAAGCC

CCACTACGAAAGTGGCTTTAACCACACCCGACCCCACGAAAGCTGTGAAA

>336_Sample_ASV18

CACCGCGGTTATACGAGAGGCCCCAGTTGATATTAGCGGCGTAAAGGGTGGTTAAGGACT

TTAATAACTAAAGCTAAACACTTCCCCGGCTGTTGTACGTAGACCGGAAGCTTGAAGCCC

AGACACGAAAGTAGCTTTATTCCCCCTGACCCCACGAAAGCTGAGAAA

>336_Sample_ASV19

CACCGCGGTTATACGAGAGGCCCTAGTTGATAAATACGGCGTAAAGAGTGGTTAGAGGAC

TCCCATAATAAAGCCAAAGACCCCCCGGGCCGTCACACGCATACGAGGGCACGAAGTTCA

CACACGAAAGTAGCTTTAAATTACCCGACGCCACGAAAGCTAAGAAA

>336_Sample_ASV20

GACCGCGGTTATACGAGAGACTCTAGTTGATAGATACGGCGTAAAGAGTGGTTTGGGATA

AACTATAAATAAAGCCAAAGACCTTCCAAGCTGTTAAACGCATACCGAAGGCACGAAGCC

CCAACACGAAAGTAGCTTTACTACTACCCCCGATGCCACGAAAGCTAAGACA

>336_Sample_ASV21

CACCGCGGTTATACGAGAGACCCTAGTTGATAAATACGGCGTAAAGAGTGGTTATGGGAA

AAACAAATAAAGTCAAACAACCTCTTAGCTGTTATACGCATTATGAGAGCATGAAGCCCC

CTCACGAAAGTAACTTTAATACCTCCTGACCCCACGAAAGCTAAGAAA

>336_Sample_ASV22

CACCGCGGTTATACGAGAGGCTCAAGTTGATAAACCCCGGCGTAAAGAGTGGTTAAGGAA

AAATAGAAACTAAAGCCGAATGCTTTCAAGACTGTTATACGTTCCCGAGAGTAAGAAGAC

CAATTACGAAAGTGGCTTTACTTTACCTGACCCCACGAAAGCTACGACA

>336_Sample_ASV23

CACCGCGGTCATACGATTAACCCAAGTTAATAGGCACACGGCGTAAAGCGTGTTTAAGCA

CTATGCCAAATAAAGTTAAACTCCAATTAAGCTGTAAAAAGCCATAATCAAGACGAAAAT

AAATAACGAAAGTAACTTTACAACCGCTGAAACACGATAGCTAAGATC

>336_Sample_ASV24

CACCGCGGTTATACGAGAGGCCCTAGTTGATAACTACCGGCGTAAAGAGTGGTTATGGAA

AAATATTTAATAAAGCCGAACACCCCCTCAGCCGTCATACGCACCTGGGAGCACGAAGAC

CTACTGCGAAAGCAGCTTTAATTATGCCTGACCCCACGACAGCTAAGAAA

>336_Sample_ASV25

CACCGCGGTTATACGAAAGACCCTAGTTTATAGACACGGCGTAAAGGGTGGTTCGGGACT

AAACAAAATAAAGCCAAAGACCTCCTAAGCCGTCATACGCTCACGGAAGCACGAAGCCCA

AGTACGAAAGTAGCTTTACAAAACATGCCTGACTCCACGAAAGCTAGGAAA

>336_Sample_ASV26

CACCGCGGTTATACGAAAGACCCTAGTTGATAGCCACGGCGTAAAGGGTGGTTAAGGTAT

ACAACAAATAAAGCTAAATAGCCTCTAGGCCGTCGCACGCATTCCGAGAGCTCGAAACCC

AAAAACGAAAGTAGCTTTAAAATAAACAGACCTGACCCCACGAAAGCTAAGAAA

>336_Sample_ASV27

CACCGCGGTTATACGAGAGGCCCAAGTTGATACTCCACGGCGTAAAGAGTGGTTAGAAAA

GACCTACTACTAAAGCCGAACGCCTTCAAAGCTGTTATACGCATCCGAAGGTGAGAAGCC

CACCCACGAAAGTGGCTTTACAACCTTGACTCCACGAAAGCTATGACA

>336_Sample_ASV28

CACCGCGGTTATACGAGAGACCCCAGTTGATAAACACGGCGTAAAGAGTGGTTAAGGGCA

CAAAACAAATAAAGTCAAACAACCTCTTAGCTGTTATACGCATTATGAGGATATGAAGCC

CCCCTACGAAAGTAACTTTACCAACCCCTGACCCCACGAAAGCTGGGGGA

>336_Sample_ASV29

CACCGCGGTTATACGAGAGACCCCAGTTGATAAACACGGCGTAAAGAGTGGTTAAGGGCA

CAAAACAAATAAAGTCAAACAACCTCTTAGCTGTTATACGCATTATGAGGATATGAAGCC

CCCCTACGAAAGTAACTTTACCAACCCCTGACCCCACGAAAGCTAAGAAA

>336_Sample_ASV30

CACCGCGGTTATACGAAAGACCCTAGTTTATAGGCACGGCGTAAAGGGTGGTTAGGGGAT

AAACAAAATAAAGCCAAAGACCTTCTAAGTCGTCATACACTCATGAAGGCACGAAGCCCA

AATACGAAAGTAACTTTACAACTTAGACCCGACTCCACGAAAGCTGGGGGA

>336_Sample_ASV31

CACCGCGGTTATACGAAAGACCCTAGTTGATAGATCACGGCGTAAAGGGTGGTTAAGGAG

AACAAAAATAAAGCTAAAAATCTTCTAAGCTGTCATACGCTTTCCGAAGACATGAGACCC

AACCACGAAAGTAGCTTTAAACTTTCCTGACACCACGAAAGCCAAGAAA

>337_Sample_ASV1

CACCGCGGTTATACGAAAGACCCTAGTTGATGGGTGCGGCGTAAAGGGTGGTTAGGGACT

ACAAAAAATAGAGCTAAAGATCCTCTAGGTCGTCATACACATCACGAGGAAACGAAGCCC

AAATACGAAAGTAGCTCTAAATATAATCCTGACGCCACGAAAGCCAAGAAA

>337_Sample_ASV2

CACCGCGGTTATACGAAAGACCCTAGTTGATAACTACGGCATAAAGGGTGGTTAGGGAGT

TTAAAAAATAAAGCCAAAGCTCTTCTAAGCTGTCATACGCTCTCTGAAAGAACGAGGCCC

AAACACGAAAGTAGCTTTAAATAAATAACCTGATACCACGAAAGCCAAGAAA

>337_Sample_ASV3

CACCGCGGTTATACGAGAGACCCTAGTTGATTAAAGCGGCGTAAAGAGTGGTTATGGAAC

TATTCTTTTAAAGCAGAAAACCTCTCAAACTGTTATACGCACCCAGAGGTCTGAATCCCT

CACACGAAAGTGACTTTATTTATGCCTACCAGAACCCACGAAAGCTGGGACA

>337_Sample_ASV4

CACCGCGGTTATACGAGAGACCCTAGTTGATATACACGGCGTAAAGAGTGGTTTGGGGTA

TATAATAAATAAAGCCAAAGACCTTCTAGGCCGTTGAACGCACCACGAAGGAGCGAAGTC

CCAATACGAAAGTAGCTTTATAAAACCTGACGCCACGAAAGCTAAGAAA

>337_Sample_ASV5

CACCGCGGTTATACGAGAGGCCCAAGTTGACAGACACCGGCGTAAAGAGTGGCTAGGGAA

AAATTACTACTAAAGCCGAACACCTTCAGAACTGTCATACGTTCCCGAAGATAAGAAGCC

CCACCACGAAAGTGACTTTATATTACCCGACCCCACGAAAGCTGGGGGA

>337_Sample_ASV6

CACCGCGGTTATACGAGAGGCCCAAGTTGACAGACACCGGCGTAAAGAGTGGCTAGGGAA

AAATTACTACTAAAGCCGAACACCTTCAGAACTGTCATACGTTCCCGAAGATAAGAAGCC

CCACCACGAAAGTGACTTTATATTACCCGACCCCACGAAAGCTGTGAAA

>337_Sample_ASV7

CACCGCGGTTATACGAGAGGCCCAAGTTGACAGACACCGGCGTAAAGAGTGGCTAGGGAA

AAATTACTACTAAAGCCGAACACCTTCAGAACTGTCATACGTTCCCGAAGATAAGAAGCC

CCACCACGAAAGTGGCTTTATATTACCCGACCCCACGAAAGCTGTGAAA

>337_Sample_ASV8

CACCGCGGTTATACGAGAGGCCCAAGTTGATAAGCCCGGCGTAAAGAGTGGTTATGGAGA

ATAAAACACTAAAGCTAAAGACCCCCTAGGCTGTTATACGCACCTGGCGGCTCGAACCAC

CTATACGAAAGTAGCTTTACCCCCCTTCCACCAGAATCCACGACAGCTGGGGCA

>337_Sample_ASV9

CACCGCGGTTATACGAGAGGCCCAAGTTGACAGACATCGGCGTAAAGAGTGGTTAAGGAA

ACCCCATTACTAAAGCCGAACGCCCTCAGAACTGTCATACGTTCCCGAAGGTAAGAAGCC

CCACTACGAAAGTGGCTTTAACCACACCCGACCCCACGAAAGCTGTGAAA

>337_Sample_ASV10

CACCGCGGTTATACGAGAAACCCTAGTTGACAAATACGGCGTAAAGAGTGGTTAAGGGGT

ATCAAAAATAAAGTCAAACATTTTCCTAGTTGTTATAAGCATTTCGAGAACATGAAGCCC

AACCACGAAAGTAACTTTACCTTCACCTGACCCCACGAAAGCTAAGAAA

>337_Sample_ASV11

CACCGCGGTTATACGAGAGACCCTAGTTGATAAATACGGCGTAAAGAGTGGTTATGGGAA

AAACAAATAAAGTCAAACAACCTCTTAGCTGTTATACGCATTATGAGAGCATGAAGCCCC

CTCACGAAAGTAACTTTAATACCTCCTGACCCCACGAAAGCTAAGAAA

>337_Sample_ASV12

AACCGCGGTTATACGAGAGACCCTAATTGATAGCTCACGGCGTAAAGGGTGGTTTGGGGA

AACCTCAAAATAAAGCTAAAGACCTTCTAAGCCGTTACACGCATACCGAAGGCACGAGGC

CCTAACACGAAAGTAGCTTTACTATTACCCCTGACGCCACGAAAGCTAAGAAA

>337_Sample_ASV13

CACCGCGGTTATACGAAAGACCCTAGTTGATGGATCACGGCGTAAAGGGTGGTTAAGGGA

TATAAAAATAAAGCTAAAAATCCTCTTAGCTGTCATACGCTTTCCGAAGACATGAGGCCC

AATTACGAAAGTAGCTTTAAACCACCCTGACACCACGAAAGCCAAGAAA

>337_Sample_ASV14

CACCGCGGTTATACGAGAGGCCCTAGTTGATAACTACCGGCGTAAAGAGTGGTTATGGAA

AAATATTTAATAAAGCCGAACACCCCCTCAGCCGTCATACGCACCTGGGAGCACGAAGAC

CTACTGCGAAAGCAGCTTTAATTATGCCTGACCCCACGACAGCTAAGAAA

>337_Sample_ASV15

CACCGCGGTTATACGAAAGACCCTAGTTTATAGACACGGCGTAAAGGGTGGTTCGGGACT

AAACAAAATAAAGCCAAAGACCTCCTAAGCCGTCATACGCTCACGGAAGCACGAAGCCCA

AGTACGAAAGTAGCTTTACAAAACATGCCTGACTCCACGAAAGCTAGGAAA

>337_Sample_ASV16

CACCGCGGTTATACGAGAGGCCCAAGTTGATACTCCACGGCGTAAAGAGTGGTTAGAAAA

GACCTACTACTAAAGCCGAACGCCTTCAAAGCTGTTATACGCATCCGAAGGTGAGAAGCC

CACCCACGAAAGTGGCTTTACAACCTTGACTCCACGAAAGCTATGACA

>337_Sample_ASV17

CACCGCGGTTATACGAGAGACCCCAGTTGATAAACACGGCGTAAAGAGTGGTTAAGGGCA

CAAAACAAATAAAGTCAAACAACCTCTTAGCTGTTATACGCATTATGAGGATATGAAGCC

CCCCTACGAAAGTAACTTTACCAACCCCTGACCCCACGAAAGCTAAGAAA

>337_Sample_ASV18

CACCGCGGTTATACGAAAGACCCTAGTTTATAGGCACGGCGTAAAGGGTGGTTAGGGGAT

AAACAAAATAAAGCCAAAGACCTTCTAAGTCGTCATACACTCATGAAGGCACGAAGCCCA

AATACGAAAGTAACTTTACAACTTAGACCCGACTCCACGAAAGCTGGGGGA

>337_Sample_ASV19

CACCGCGGTTATACGAGAGACCCTAATTAATAGATACGGCGTAAAGAGTGGTTTAGGACA

AAAATTAATAAAGCCGAAGTTTCTCCTAGCCGTTGTACGCACTCTGAGAACACGAAGCCC

CACTACGAAAGTAGCTTTACCTATTATTTCCTGACCCCACGAAAGCTAAGAAA

>337_Sample_ASV20

CACCGCGGTTATACGAGAGGCCCAAGTTGATAGACATCGGCGTAAAGAGTGGTTAAGGAA

GTCTCAAAACTAAAGCCGAATGCCTTCAAGACTGTTATACGTTTCCGAAGGTAAGAAGCC

CCACTACGAAAGTGGCTTTATTCCACCTGACCCCACGAAAGCTGTGAAA

>337_Sample_ASV21

CACCGCGGTTATACGAGAGGCCCAAGTTGATAGACATCGGCGTAAAGAGTGGTTAAGGAA

GTCTCAAAACTAAAGCCGAATGCCTTCAAGACTGTTATACGTTTCCGAAGGTAAGAAGCC

CCACTACGAAAGTGGCTTTACTCCACCTGACTCCACGAAAGCTGTGAAA

>337_Sample_ASV22

CACCGCGGTTATACGAAAGACCCTAGTTGATAGACCACGGCGTAAAGGGTGGTTAAGGAA

CACAAAAATAAAGCTAAAGATCCTCTAAGCTGTCATACGCTTCCCGAAGACATGAAACCC

AATCACGAAAGTAGCTTTAAACTGTCCTGACACCACGAAAGCCAAGAAA

>341_Sample_ASV1

CACCGCGGTTATACGAAAGACCCTAGTTGATGGGTGCGGCGTAAAGGGTGGTTAGGGACT

ACAAAAAATAGAGCTAAAGATCCTCTAGGTCGTCATACACATCACGAGGAAACGAAGCCC

AAATACGAAAGTAGCTCTAAATATAATCCTGACGCCACGAAAGCCAAGAAA

>341_Sample_ASV2

CACCGCGGTTATACGAGAGACCCTAGTTGATTAAAGCGGCGTAAAGAGTGGTTATGGAAC

TATTCTTTTAAAGCAGAAAACCTCTCAAACTGTTATACGCACCCAGAGGTCTGAATCCCT

CACACGAAAGTGACTTTATTTATGCCTACCAGAACCCACGAAAGCTGGGACA

>341_Sample_ASV3

CACCGCGGTTATACGAAAGACCCTAGTTTATAGGTACGGCGTAAAGGGTGGTTAGGGGAC

AAACAAAATAAAGCCAAAGACCTCCTAAGCCGTCATACGCTCACGGAAGCACGAAGCCCA

AACACGAAAGTAGCTTTACCGAACATGCCCGACTCCACGAAAGCTGGGGAA

>341_Sample_ASV4

CATGGCGATTATACGAGAGGCCCAAGTTGACAGACACTGGCGTAAAGAGTGGCTAGGGAA

AAATTACTACTAAAGCCGAACACCTTCAGAACTGTCATACGTTCCCGAAGATAAGAAGCC

CCACCACGAAAGTGGCTTTATATTACCCGACCCCACGAAAGCTGTGAAA

>341_Sample_ASV5

CATGGCGATTATACGAGAGGCCCAAGTTGACAGACACTGGCGTAAAGAGTGGCTAAGGAA

AAACTACTACTAAAGCCGAACACCTTCAGAACTGTCATACGTTCCCGAAGATAAGAAGCC

CCACCACGAAAGTGGCTTTATATTACCCGACCCCACGAAAGCTGTGAAA

>341_Sample_ASV6

CATGGCGATTATACGAGAGGCCCAAGTTGACAGACACCGGCGTAAAGAGTGGCTAGGGAA

AAATTACTACTAAAGCCGAACACCTTCAGAACTGTCATACGTTCCCGAAGATAAGAAGCC

CCACCACGAAAGTGGCTTTATATTACCCGACCCCACGAAAGCTGTGAAA

>341_Sample_ASV7

CACCGCGGTTATACGAGAGGCCCAAGTTGACAGACACCGGCGTAAAGAGTGGCTAGGGAA

AAATTACTACTAAAGCCGAACACCTTCAGAACTGTCATACGTTCCCGAAGATAAGAAGCC

CCACCACGAAAGTGGCTTTATATTACCCGACCCCACGAAAGCTGTGAAA

>341_Sample_ASV8

CACCGCGGTTATACGAGAGACCCTAGTTGATATATACGGCGTAAAGAGTGGTTTGGGGTT

ATGCCTCCAAAATAAAGTCGAAGATCTCCCAAGCCGTCACACGCACCCCGGAGACACGAA

GCCCTAATACGAAGGTAGCTTTATTAATTCCCCGACGCCACGAAAGCTAAGAAA

>341_Sample_ASV9

CACCGCGGTTATACGAGAGACCCTAGTTGATATATACGGCGTAAAGAGTGGTTTGGGGTT

ATGCCTCCAAAATAAAGCCGAAGATCTCCCAAGCCGTCACACGCATCCCGGAGACACGAA

GCCCTAATACGAAGGTAGCTTTATTAATTCCCCGACGCCACGAAAGCTAAGAAA

>341_Sample_ASV10

CACCGCGGTTATACGAGAGACCCTAGTTGATATATACGGCGTAAAGAGTGGTTTGGGGTT

ATGCCTCCAAAATAAAGCCGAAGATCTCCCAAGCCGTCACACGCATCCCGGAGACACGAA

GCCCTAATACGAAGGTAGCTTTATTAGTACCCCGACGCCACGAAAGCTAAGAAA

>341_Sample_ASV11

CACCGCGGTTATACGAGAGGCCCAAGTTGATAAGCCCGGCGTAAAGAGTGGTTATGGAGA

ATAAAACACTAAAGCTAAAGACCCCCTAGGCTGTTATACGCACCTGGCGGCTCGAACCAC

CTATACGAAAGTAGCTTTACCCCCCTTCCACCAGAATCCACGACAGCTGGGGCA

>341_Sample_ASV12

CACCGCGGTTATACGAGAGGCCCTAGTTGATAGCCACGGCGCAAAGAGTGGTTAAGGAGC

CCCACTAAATAAAGTCGAACACTTCCTAGGCCGTTATACGTTTTCTAGAAGCACGAAACC

CAATTACACGAAAGCAACTTTATACTAAACAACCTGACCCCACGAAAGCTAAGAAA

>341_Sample_ASV13

CACCGCGGTTATACGAGAGGCCCAAGTTGACAGACATCGGCGTAAAGAGTGGTTAAGGAA

ACCCCATTACTAAAGCCGAACGCCCTCAGAACTGTCATACGTTCCCGAAGGTAAGAAGCC

CCACTACGAAAGTGGCTTTAACCACACCCGACCCCACGAAAGCTGTGAAA

>341_Sample_ASV14

CACCGCGGTTATACGAGAGGCCCAAGTTGACAGACATCGGCGTAAAGAGTGGTTAAGGAA

ACCCCATTACTAAAGCCGAACGCCCTCAGAACTGTCATACGTTCCCGAAGGTAAGAAGCC

CCACTACGAAAGTGGCTTTAACCACACCCGACCCCACGAAAGCTGGGACA

>341_Sample_ASV15

CACCGCGGTTATACGAGAGGCCCAAGTTGATAGATATCGGCGTAAAGAGTGGTTAAGGAA

ACCCCGTTACTAAAGCCGAACGCCCTCAGAACTGTCATACGTTCCCGAAGGTAAGAAGCC

CCACTACGAAAGTGGCTTTAACCACACCCGACCCCACGAAAGCTGTGAAA

>341_Sample_ASV16

CACCGCGGTTATACGAGAGGCCCAAGTTGACAGACACCGGCGTAAAGAGTGGTTAAGGAA

ATTCATCAACTAAAGCCGAATGCCCTCAGGACTGTTATACGTTTGCGAAGGTAAGAAGCC

CTACTACGAAAGTGGCTTTATTCCACCTGATTCCACGAAAGCTGCGGAA

>341_Sample_ASV17

CACCGCGGTTATACGAAAGACCCCAGTTTATAGACACGGCGTAAAGGGTGGTTATAGGAC

AAACTAACATAGAGCCAAAGACCCCCTAAGCCGTCATACGCTCACGAGGGCTCGAAGCCC

CTATACGAAAGTAGCTCTACCAAAAAATGCTCGACTCCACGAAAGCTGGGACA

>341_Sample_ASV18

CACCGCGGTTATACGAGAGGCCCCAGTTGATAAACACGGCGTAAAGGGTGGTTAAGGGAG

CATAAGAATAAAGCCGAAGGGCCCTTTGGCCGTTATACGCTTCTAGGTGTCCGAAGCCCA

AACACGAAAGTAGCTTTAGTTAAGCCCACCTGACTCCACGAAAACTGAGAAA

>341_Sample_ASV19

CACCGCGGTTATACGAGAGACCCTAGTTGATAAATACGGCGTAAAGAGTGGTTATGGGAA

AAACAAATAAAGTCAAACAACCTCTTAGCTGTTATACGCATTATGAGAGCATGAAGCCCC

CTCACGAAAGTAACTTTAATACCTCCTGACCCCACGAAAGCTAAGAAA

>341_Sample_ASV20

CACCGCGGTTATACGAGAGGCCCAAGTTGACAAACCCCGGCGTAAAGAGTGGTTAAGGGA

GATCAAAACTAAAGCCGAATGCTTTCAAAGCTGTTATACGCTTCCGAAAGTAAGAAGCTC

AATCACGAAAGTGGCTTTACTTTACCTGACCCCACGAAAGCTACGACA

>341_Sample_ASV21

CACCGCGGTTATACGAGAGGCTCAAGTTGATGAACCCCGGCGTAAAGAGTGGTTAAGGGA

GATCAAAACTAAAGCCGAATGCTTTCAAAGCTGTTATACGCTTCCGAAAGTAAGAAGCTC

AATCACGAAAGTGGCTTTACTTTACCTGACCCCACGAAAGCTACGACA

>341_Sample_ASV22

CACCGCGGTTATACGAAAGACCCTAGTTGATGGATCACGGCGTAAAGGGTGGTTAAGGGA

TATAAAAATAAAGCTAAAAATCCTCTTAGCTGTCATACGCTTTCCGAAGACATGAGGCCC

AATTACGAAAGTAGCTTTAAACCACCCTGACACCACGAAAGCCAAGAAA

>341_Sample_ASV23

CACCGCGGTTATACGAAAGACCCCAGTTTATAGATGCGGCGTAAAGGGTGGTTAAGAGAA

AATCAAAATAAGGCCAAAGATCCCCTAAGCAGTCATACGCTCCACGAGGACACGAAACCC

AAATACGAAAGTTGCCTTAATAAACTTCACCTGATCCCACGAAAGCTGAAAAA

>341_Sample_ASV24

CACCGCGGTTATACGAGAGGCCCAAGTTGACAAACCCCGGCGTAAAGAGTGGTTAAGGAA

AATTTAAAACTAAAGCCGAATGCCCCCAAAGCTGTTATACGCACCCGAGGATAAGAAGCC

CAATCACGAAAGTGGCTTTACACTACCTGAACCCACGAAAGCTATGAAA

>341_Sample_ASV25

CACCGCGGTTATACGAGAGGCCCAAGTTGACAAACCCCGGCGTAAAGAGTGGTTAAGGAA

AATTTAAAACTAAAGCCGAATGCCCCCAAAGCTGTTATACGCACCCGAGGGTAAGAAGCC

CAATCACGAAAGTGGCTTTACACTACCTGAACCCACGAAAGCTATGAAA

>341_Sample_ASV26

CACCGCGGTTATACGAGAGGCCCTAGTTGATAACTACCGGCGTAAAGAGTGGTTATGGAA

AAATATTTAATAAAGCCGAACACCCCCTCAGCCGTCATACGCACCTGGGAGCACGAAGAC

CTACTGCGAAAGCAGCTTTAATTATGCCTGACCCCACGACAGCTAAGAAA

>341_Sample_ASV27

CACCGCGGTTATACGAAAGACCCTAGTTTATAGACACGGCGTAAAGGGTGGTTCGGGACT

AAACAAAATAAAGCCAAAGACCTCCTAAGCCGTCATACGCTCACGGAAGCACGAAGCCCA

AGTACGAAAGTAGCTTTACAAAACATGCCTGACTCCACGAAAGCTAGGAAA

>341_Sample_ASV28

CACCGCGGTTATACGAGAGGCCCAAGTTGATACTCCACGGCGTAAAGAGTGGTTAGAAAA

GACCTACTACTAAAGCCGAACGCCTTCAAAGCTGTTATACGCATCCGAAGGTGAGAAGCC

CACCCACGAAAGTGGCTTTACAACCTTGACTCCACGAAAGCTATGACA

>341_Sample_ASV29

CACCGCGGTTATACGAGAGACCCCAGTTGATAAACACGGCGTAAAGAGTGGTTAAGGGCA

CAAAACAAATAAAGTCAAACAACCTCTTAGCTGTTATACGCATTATGAGGATATGAAGCC

CCCCTACGAAAGTAACTTTACCAACCCCTGACCCCACGAAAGCTAAGAAA

>341_Sample_ASV30

CACCGCGGTTATACGAGAGACCCTAGTTGATATATACGGCGTAAAGAGTGGTTTGGAATA

ACCAAGTAATATAGCCAAAGACCTCCCAAGCCGTCACACGCACCCCGGAGGCACGAAGCC

CAAACACGAAAGTAGCTTTATTAACTTACCGACGCCACGAAAGCTAAGAAA

>341_Sample_ASV31

CACCGCGGTTATACGAAAGACCCTAGTTTATAGGCACGGCGTAAAGGGTGGTTAGGGGAT

AAACAAAATAAAGCCAAAGACCTTCTAAGTCGTCATACACTCATGAAGGCACGAAGCCCA

AATACGAAAGTAACTTTACAACTTAGACCCGACTCCACGAAAGCTGGGGGA

>342_Sample_ASV1

CACCGCGGTTATACGAAAGACCCTAGTTGATGGGTGCGGCGTAAAGGGTGGTTAGGGACT

ACAAAAAATAGAGCTAAAGATCCTCTAGGTCGTCATACACATCACGAGGAAACGAAGCCC

AAATACGAAAGTAGCTCTAAATATAATCCTGACGCCACGAAAGCCAAGAAA

>342_Sample_ASV2

CACCGCGGTTATACGAGAGACCCTAGTTGATTAAAGCGGCGTAAAGAGTGGTTATGGAAC

TATTCTTTTAAAGCAGAAAACCTCTCAAACTGTTATACGCACCCAGAGGTCTGAATCCCT

CACACGAAAGTGACTTTATTTATGCCTACCAGAACCCACGAAAGCTAAGAAA

>342_Sample_ASV3

CACCGCGGTTATACGAGAGACCCTAGTTGATTAAAGCGGCGTAAAGAGTGGTTATGGAAC

TATTCTTTTAAAGCAGAAAACCTCTCAAACTGTTATACGCACCCAGAGGTCTGAATCCCT

CACACGAAAGTGACTTTATTTATGCCTACCAGAACCCACGAAAGCTGGGACA

>342_Sample_ASV4

CACCGCGGTTATACGAGAGGCCCAAGTTGACAGACACCGGCGTAAAGAGTGGCTAGGGAA

AAATTACTACTAAAGCCGAACACCTTCAGAACTGTCATACGTTCCCGAAGATAAGAAGCC

CCACCACGAAAGTGGCTTTATATTACCCGACCCCACGAAAGCTGTGAAA

>342_Sample_ASV5

CACCGCGGTTATACGAGAGGCCCAAGTTGACAGACACCGGCGTAAAGAGTGGCTAGGGAA

AAATTACTACTAAAGCCGAACACCTTCAGAACTGTCATACGTTCCCGAAGATAAGAAGCC

CCACCACGAAAGTGGCTTTATATTACCCGACCCCACGAAAGCTAAGAAA

>342_Sample_ASV6

CACCGCGGTTATACGAGAGACCCTAGTTGACAGACACCGGCGTAAAGAGTGGCTAGGGAA

AAATTACTACTAAAGCCGAACACCTTCAGAACTGTCATACGTTCCCGAAGATAAGAAGCC

CCACCACGAAAGTGGCTTTATATTACCCGACCCCACGAAAGCTAAGAAA

>342_Sample_ASV7

CACCGCGGTTATACGAAAGACTCTAGTTGACAAACACGGCGTAAAGGGTGGTTAGGATAA

CAACAAAATAAAGCTAAAGACTTACCAAGCCGTCATACGCCCATGTAAGAGCGAAAACCA

CAAACGAAAGTAACTTTATTACACCATCCGAATCCACGAAAGCTAAGAAA

>342_Sample_ASV8

CACCGCGGTTATACGAGAGGCCCAAGTTGATAAGCCCGGCGTAAAGAGTGGTTATGGAGA

ATAAAACACTAAAGCTAAAGACCCCCTAGGCTGTTATACGCACCTGGCGGCTCGAACCAC

CTATACGAAAGTAGCTTTACCCCCCTTCCACCAGAATCCACGACAGCTGGGGCA

>342_Sample_ASV9

CACCGCGGTTATACGAGAGGCCCTAGTTGATAGCCACGGCGCAAAGAGTGGTTAAGGAGC

CCCACTAAATAAAGTCGAACACTTCCTAGGCCGTTATACGTTTTCTAGAAGCACGAAACC

CAATTACACGAAAGCAACTTTATACTAAACAACCTGACCCCACGAAAGCTAAGAAA

>342_Sample_ASV10

CACCGCGGTTATACGAGAGGCCCAAGTTGACAGACATCGGCGTAAAGAGTGGTTAAGGAA

ACCCCATTACTAAAGCCGAACGCCCTCAGAACTGTCATACGTTCCCGAAGGTAAGAAGCC

CCACTACGAAAGTGGCTTTAACCACACCCGACCCCACGAAAGCTAAGAAA

>342_Sample_ASV11

CACCGCGGTTATACGAGAGGCCCAAGTTGACAGACATCGGCGTAAAGAGTGGTTAAGGAA

ACCCCATTACTAAAGCCGAACGCCCTCAGAACTGTCATACGTTCCCGAAGGTAAGAAGCC

CCACTACGAAAGTGGCTTTAACCACACCCGACCCCACGAAAGCTGTGAAA

>342_Sample_ASV12

CACCGCGGTTATACGAAAGACCCCAGTTTATAGACACGGCGTAAAGGGTGGTTATAGGAC

AAACTAACATAGAGCCAAAGACCCCCTAAGCCGTCATACGCTCACGAGGGCTCGAAGCCC

CTATACGAAAGTAGCTCTACCAAAAAATGCTCGACTCCACGAAAGCTGGGACA

>342_Sample_ASV13

AACCGCGGTTATACGAGAGACCCTAATTGATAGCTCACGGCGTAAAGGGTGGTTTGGGGA

AACCTCAAAATAAAGCTAAAGACCTTCTAAGCCGTTACACGCATACCGAAGGCACGAGGC

CCTAACACGAAAGTAGCTTTACTATTACCCCTGACGCCACGAAAGCTAAGAAA

>342_Sample_ASV14

CACCGCGGTTATACGAAAGACCCTAGTTGATGGATCACGGCGTAAAGGGTGGTTAAGGGA

TATAAAAATAAAGCTAAAAATCCTCTTAGCTGTCATACGCTTTCCGAAGACATGAGGCCC

AATTACGAAAGTAGCTTTAAACCACCCTGACACCACGAAAGCCAAGAAA

>342_Sample_ASV15

CACCGCGGTTATACGAGAGGCCCTAGTTGATAACTACCGGCGTAAAGAGTGGTTATGGAA

AAATATTTAATAAAGCCGAACACCCCCTCAGCCGTCATACGCACCTGGGAGCACGAAGAC

CTACTGCGAAAGCAGCTTTAATTATGCCTGACCCCACGACAGCTAAGAAA

>342_Sample_ASV16

CACCGCGGTTATACGAGAGGCCCAAGTTGATTATTACCGGCGTAAAGCGTGGTTAAGATT

TTTATAGGACTAAAGCCGAACCCCCTCAGAGCTGTTATACGCACCCGAAGGTTAGAAGTT

CAATCACGAAAGTGGCTTTACAACCCCGAACCCACGAAAGCTAAGATA

>342_Sample_ASV17

CACCGCGGTTATACGAGAGACCCTAATTGATAGGCGCGGCGTAAAGAGTGGTTTAGGACT

AAAACTAATAAAGCTAAAGATCCTCCCAGCCGTCGCACGCATTCTGAGGACACGAAACTC

AACCACGAAAGTAGCTTTAACATACTCTCCTGACCCCACGAAAGCTAAGAAA

>342_Sample_ASV18

CACCGCGGTTATACGAAAGACCCTAGTTGATAGGCACGGCGTAAAGGGTGGTTAGGGATA

TATCAAAAATAAAGTTAAAGAGCCTCTAAGCTGTCGCACGCATCCCGAGAGCTCGAGACC

CAGACACGAAAGTAACTTTAAAATACCTCACCTGACCCCACGAAAGCTAAGAAA

>342_Sample_ASV19

CACCGCGGTTATACGAGAGGCCCAAGTTGATACTCCACGGCGTAAAGAGTGGTTAGAAAA

GACCTACTACTAAAGCCGAACGCCTTCAAAGCTGTTATACGCATCCGAAGGTGAGAAGCC

CACCCACGAAAGTGGCTTTACAACCTTGACTCCACGAAAGCTAAGAAA

>342_Sample_ASV20

CACCGCGGTTATACGAGAGGCCCAAGTTGATACTCCACGGCGTAAAGAGTGGTTAGAAAA

GACCTACTACTAAAGCCGAACGCCTTCAAAGCTGTTATACGCATCCGAAGGTGAGAAGCC

CACCCACGAAAGTGGCTTTACAACCTTGACTCCACGAAAGCTATGACA

>342_Sample_ASV21

CACCGCGGTTATACGAGAGACCCCAGTTGATAAACACGGCGTAAAGAGTGGTTAAGGGCA

CAAAACAAATAAAGTCAAACAACCTCTTAGCTGTTATACGCATTATGAGGATATGAAGCC

CCCCTACGAAAGTAACTTTACCAACCCCTGACCCCACGAAAGCTAAGAAA

>342_Sample_ASV22

CACCGCGGTTATACGAGAGACCCTAGTTGATATATACGGCGTAAAGAGTGGTTTGGAATA

ACCAAGTAATATAGCCAAAGACCTCCCAAGCCGTCACACGCACCCCGGAGGCACGAAGCC

CAAACACGAAAGTAGCTTTATTAACTTACCGACGCCACGAAAGCTAAGAAA

>342_Sample_ASV23

CACCGCGGTTATACGAAAGACCCTAGTTTATAGGCACGGCGTAAAGGGTGGTTAGGGGAT

AAACAAAATAAAGCCAAAGACCTTCTAAGTCGTCATACACTCATGAAGGCACGAAGCCCA

AATACGAAAGTAACTTTACAACTTAGACCCGACTCCACGAAAGCTGGGGGA

>342_Sample_ASV24

CACCGCGGTTATACGAGAGACCCTAATTAATAGATACGGCGTAAAGAGTGGTTTAGGACA

AAAATTAATAAAGCCGAAGTTTCTCCTAGCCGTTGTACGCACTCTGAGAACACGAAGCCC

CACTACGAAAGTAGCTTTACCTATTATTTCCTGACCCCACGAAAGCTAAGAAA

>342_Sample_ASV25

CACCGCGGTTATACGAGAGACCCTAATTAATAGCTACGGCGTAAAGAGTGGTTTAGGGTA

AAAATTAATAAAGCCGAAGTTTCTCCTAGCCGTTGTACGCACTCTGAGAACACGAAGCCC

CACTACGAAAGTAGCTTTACCTATTATTTCCTGACCCCACGAAAGCTAAGAAA

>342_Sample_ASV26

CACCGCGGTTATACGAGAGACCCTAATTAATAGCTACGGCGTAAAGAGTGGTTTAGGACA

AAAATTAATAAAGCCGAAGTTTCTCCTAGCCGTTGTACGCACTCTGAGAACACGAAGCCC

CACTACGAAAGTAGCTTTACCTATTATTTCCTGACCCCACGAAAGCTAAGAAA

>342_Sample_ASV27

CACCGCGGTTATACGAAAGACCCTAGTTGATAGATCACGGCGTAAAGGGTGGTTAAGGAA

AACAAAAATAAAGCTAAAAATCCTCTAAGCTGTCATACGCTTTCCGAAGACATGAGATCC

AACCACGAAAGTAGCTTTAAAATTTCCTGACACCACGAAAGCCAAGGAA

>344_2_Sample_ASV1

CACCGCGGTTATACGAGAGGCCCAAGTTGACAGACATCGGCGTAAAGAGTGGTTAAGGAA

AACCTTCAACTAAAGTCGAACGCTCTCAAGACTGTCGTACGTTTCCGAGAGTATGAAGCC

CCACCACGAAAGTGACTTTACCTCACCTGACTCCACGAAAGCTGCGAAA

>344_Sample_ASV1

CACCGCGGTTATATGAAAGACCCTAGTTGATGGGTGCGGCGTAAAGGGTGGTTAGGGACT

ACAAAAAATAGAGCTAAAGATCCTCTAGGTCGTCATACACATCACGAGGAAACGAAGCCC

AAATACGAAAGTAGCTCTAAATATAATCCTGACGCCACGAAAGCCAAGAAA

>344_Sample_ASV2

CACCGCGGTTATACGAAAGACCCTAGTTGATGGGTGCGGCGTAAAGGGTGGTTAGGGACT

ACAAAAAATAGAGCTAAAGATCCTCTAGGTCGTCATACACATCACGAGGAAACGAAGCCC

AAATACGAAAGTAGCTCTAAATATAATCCTGACGCCACGAAAGCCAAGAAA

>344_2_Sample_ASV2

CACCGCGGTTATACGAAAGACCCTAGTTGATGGGTGCGGCGTAAAGGGTGGTTAGGGACT

ACAAAAAATAGAGCTAAAGATCCTCTAGGTCGTCATACACATCACGAGGAAACGAAGCCC

AAATACGAAAGTAGCTCTAAATATAATCCTGACGCCACGAAAGCCAAGAAA

>344_2_Sample_ASV3

CACCGCGGTTATACGAGAGACCCTAGTTGATTAAAGCGGCGTAAAGAGTGGTTATGGAAC

TATTCTTTTAAAGCAGAAAACCTCTCAAACTGTTATACGCACCCAGAGGTCTGAATCCCT

CACACGAAAGTGACTTTATTTATGCCTACCAGAACCCACGAAAGCTGGGACA

>344_Sample_ASV3

CACCGCGGTTATACGAGAGACCCTAGTTGATTAAAGCGGCGTAAAGAGTGGTTATGGAAC

TATTCTTTTAAAGCAGAAAACCTCTCAAACTGTTATACGCACCCAGAGGTCTGAATCCCT

CACACGAAAGTGACTTTATTTATGCCTACCAGAACCCACGAAAGCTGGGACA

>344_Sample_ASV4

CACCGCGGTTATACGAAAGACCCTAGTTTATAGGTACGGCGTAAAGGGTGGTTAGGGGAC

AAACAAAATAAAGCCAAAGACCTCCTAAGCCGTCATACGCTCACGGAAGCACGAAGCCCA

AACACGAAAGTAGCTTTACCGAACATGCCCGACTCCACGAAAGCTGGGGAA

>344_2_Sample_ASV4

CACCGCGGTTATACGAAAGACCCTAGTTTATAGGTACGGCGTAAAGGGTGGTTAGGGGAC

AAACAAAATAAAGCCAAAGACCTCCTAAGCCGTCATACGCTCACGGAAGCACGAAGCCCA

AACACGAAAGTAGCTTTACCGAACATGCCCGACTCCACGAAAGCTGGGGAA

>344_Sample_ASV5

CACCGCGGTTATACGAGAGACCCTAGTTGATATACACGGCGTAAAGAGTGGTTTGGGGTA

TATAATAAATAAAGCCAAAGACCTTCTAGGCCGTTGAACGCACCACGAAGGAGCGAAGTC

CCAATACGAAAGTAGCTTTATAAAACCTGACGCCACGAAAGCTAAGAAA

>344_Sample_ASV6

CACCGCGGTTATACGAGAGGCCCAAGTTGACAGACACCGGCGTAAAGAGTGGCTAGGGAA

AAATTACTACTAAAGCCGAACACCTTCAGAACTGTCATACGTTCCCGAAGATAAGAAGCC

CCACCACGAAAGTGGCTTTATATTACCCGACCCCACGAAAGCTGAAAAA

>344_Sample_ASV7

CACCGCGGTTATACGAGAGGCCCAAGTTGACAGACACCGGCGTAAAGAGTGGCTAGGGAA

AAATTACTACTAAAGCCGAACACCTTCAGAACTGTCATACGTTCCCGAAGATAAGAAGCC

CCACCACGAAAGTGGCTTTATATTACCCGACCCCACGAAAGCTGTGAAA

>344_2_Sample_ASV5

CACCGCGATTATACGAGAGGCCCAAGTTGACAGACATCGGCGTAAAGAGTGGTTAAGGAA

AAATTACTACTAAAGCCGAACACCTTCAGAACTGTCATACGTTCCCGAAGATAAGAAGCC

CCACCACGAAAGTGGCTTTATATTACCCGACCCCACGAAAGCTGTGAAA

>344_2_Sample_ASV6

CACCGCGGTTATACGAGAGGCCCAAGTTGACAGACACCGGCGTAAAGAGTGGCTAGGGAA

AAATTACTACTAAAGCCGAACACCTTCAGAACTGTCATACGTTCCCGAAGATAAGAAGCC

CCACCACGAAAGTGGCTTTATATTACCCGACCCCACGAAAGCTGTGAAA

>344_2_Sample_ASV7

CACCGCGGTTATACGAGAGGCCCAAGTTGACAGACACCGGCGTAAAGAGTGGCTAGGGAA

AAATTACTACTAAAGCCGAACACCTTCAGAACTGTCATACGTTCCCGAAGATAAGAAGCC

CCACCACGAAAGTGGCTTTATATTACCCGACCCCACGAAAGCTGAAAAA

>344_2_Sample_ASV8

CACCGCGGTTATACGAAAGGCCCTAGTTGACAGACACCGGCGTAAAGAGTGGCTAGGGAA

AAATTACTACTAAAGCCGAACACCTTCAGAACTGTCATACGTTCCCGAAGATAAGAAGCC

CCACCACGAAAGTGGCTTTATATTACCCGACCCCACGAAAGCTGAAAAA

>344_2_Sample_ASV9

CACCGCGGTTATACGAGAGGCCCAAGTTGACAGACACCGGCGTAAAGAGTGGCTAAGGAA

AAATTTCTACTAAAGCCGAACACCTTCAGAACTGTCATACGTTCCCGAAGAAAAGAAGCC

CCACCACGAAAGTGGCTTTATATTACCCGACCCCACGAAAGCTGTGAAA

>344_Sample_ASV8

CACCGCGGTTAAACGAGAGACCCTAGTTGATCATCACGGCGTAAAGAGTGGTTAGGGGAT

TATCATAAATAAAGCCAAAGACCTCCCAAGCTGTCGCACGCATTCCGGAGGCGCGAAGCC

CACACACGAAAGTAGCTTTAACTATTGACCCTGATGCCACGAAAGCTAAGAAA

>344_2_Sample_ASV10

CACCGCGGTTAAACGAGAGACCCTAGTTGATCATCACGGCGTAAAGAGTGGTTAGGGGAT

TATCATAAATAAAGCCAAAGACCTCCCAAGCTGTCGCACGCATTCCGGAGGCGCGAAGCC

CACACACGAAAGTAGCTTTAACTATTGACCCTGATGCCACGAAAGCTAAGAAA

>344_Sample_ASV9

CACCGCGGTTATACGAGAGACCCTAGTTGATATATACGGCGTAAAGAGTGGTTTGGGGTT

ATGCCTCCAAAATAAAGCCGAAGATCTCCCAAGCCGTCACACGCATCCCGGAGACACGAA

GCCCTAATACGAAGGTAGCTTTATTAATTCCCCGACGCCACGAAAGCTAAGAAA

>344_Sample_ASV10

CACCGCGGTTATACGAGAGACCCTAGTTGATATATACGGCGTAAAGAGTGGTTTGGGGTT

ATGCCTCCAAAATAAAGCCGAAGATCTCCCAAGCCGTCACACGCATCCCGGAGACACGAA

GCCCTAATACGAAGGTAGCTTTATTAGTACCCCGACGCCACGAAAGCTAAGAAA

>344_Sample_ASV11

CACCGCGGTTATACGAGAGGCCCAAGTTGATAAGCCCGGCGTAAAGAGTGGTTATGGAGA

ATAAAACACTAAAGCTAAAGACCCCCTAGGCTGTTATACGCACCTGGCGGCTCGAACCAC

CTATACGAAAGTAGCTTTACCCCCCTTCCACCAGAATCCACGACAGCTGGGGCA

>344_2_Sample_ASV11

CACCGCGGTTATACGAGAGGCCCAAGTTGATAAGCCCGGCGTAAAGAGTGGTTATGGAGA

ATAAAACACTAAAGCTAAAGACCCCCTAGGCTGTTATACGCACCTGGCGGCTCGAACCAC

CTATACGAAAGTAGCTTTACCCCCCTTCCACCAGAATCCACGACAGCTGGGGCA

>344_Sample_ASV12

CACCGCGGTTATACGAGAGGCCCTAGTTGATAGCCACGGCGCAAAGAGTGGTTAAGGAGC

CCCACTAAATAAAGTCGAACACTTCCTAGGCCGTTATACGTTTTCTAGAAGCACGAAACC

CAATTACACGAAAGCAACTTTATACTAAACAACCTGACCCCACGAAAGCTAAGAAA

>344_2_Sample_ASV12

CACCGCGGTTATACGAGAGGCCCAAGTTGACAGACATCGGCGTAAAGAGTGGTTAAGGAA

ACCCCATTACTAAAGCCGAACGCCCTCAGAACTGTCATACGTTCCCGAAGGTAAGAAGCC

CCACTACGAAAGTGGCTTTAACCACACCCGACCCCACGAAAGCTAAGAAA

>344_2_Sample_ASV13

CACCGCGGTTATACGAGAGGCCCAAGTTGACAGACATCGGCGTAAAGAGTGGTTAAGGAA

ACCCCATTACTAAAGCCGAACGCCCTCAGAACTGTCATACGTTCCCGAAGGTAAGAAGCC

CCACTACGAAAGTGGCTTTAACCACACCCGACCCCACGAAAGCTGAAAAA

>344_2_Sample_ASV14

CACCGCGGTTATACGAGAGGCCCAAGTTGACAGACATCGGCGTAAAGAGTGGTTAAGGAA

ACCCCATTACTAAAGCCGAACGCCCTCAGAACTGTCATACGTTCCCGAAGGTAAGAAGCC

CCACTACGAAAGTGGCTTTAACCACACCCGACCCCACGAAAGCTGTGAAA

>344_Sample_ASV13

CACCGCGGTTATACGAGAGGCCCAAGTTGACAGACATCGGCGTAAAGAGTGGTTAAGGAA

ACCCCATTACTAAAGCCGAACGCCCTCAGAACTGTCATACGTTCCCGAAGGTAAGAAGCC

CCACTACGAAAGTGGCTTTAACCACACCCGACCCCACGAAAGCTGTGAAA

>344_Sample_ASV14

CACCGCGGTTATACGAGAGGCCCAAGTTGACAGACATCGGCGTAAAGAGTGGTTAAGGAA

ACCCCATTACTAAAGCCGAACGCCCTCAGAACTGTCATACGTTCCCGAAGGTAAGAAGCC

CCACTACGAAAGTGGCTTTAACCACACCCGACCCCACGACAGCTAAGAAA

>344_Sample_ASV15

CACCGCGGTTATACGAGAGGCCCAAGTTGATAGATATCGGCGTAAAGAGTGGTTAAGGAA

ACCCCGTTACTAAAGCCGAACGCCCTCAGAACTGTCATACGTTCCCGAAGGTAAGAAGCC

CCACTACGAAAGTGGCTTTAACCACACCCGACCCCACGAAAGCTGTGAAA

>344_2_Sample_ASV15

CACCGCGGTTATACGAGAGGCCCAAGTTGATAGATATCGGCGTAAAGAGTGGTTAAGGAA

ACCCCGTTACTAAAGCCGAACGCCCTCAGAACTGTCATACGTTCCCGAAGGTAAGAAGCC

CCACTACGAAAGTGGCTTTAACCACACCCGACCCCACGAAAGCTGTGAAA

>344_Sample_ASV16

CACCGCGGTTATACGAGAGGCCCAAGTTGACAGACACCGGCGTAAAGAGTGGTTAAGGAA

ATTCATCAACTAAAGCCGAATGCCCTCAGGACTGTTATACGTTTGCGAAGGTAAGAAGCC

CTACTACGAAAGTGGCTTTATTCCACCTGATTCCACGAAAGCTGCGGAA

>344_Sample_ASV17

CACCGCGGTCACACGATTAACCCAAGTCAATAGAAGCCGGCGTAAAGAGTGTTTTAGATC

ACCCCCTCCCCAATAAAGCTAAAACTCACCTGAGTTGTAAAAAACTCCAGTTGACACAAA

ATAGACTACGAAAGTGGCTTTAACATATCTGAACACACAATAGCTAAGACC

>344_2_Sample_ASV16

CACCGCGGTTATACGAGAGGCCCTAGTTGATAAATACGGCGTAAAGAGTGGTTAGAGGAC

TCCCATAATAAAGCCAAAGACCCCCCGGGCCGTCACACGCATACGAGGGCACGAAGTTCA

CACACGAAAGTAGCTTTAAATTACCCGACGCCACGAAAGCTAAGAAA

>344_2_Sample_ASV17

CACCGCGGTTATACGAGAAACCCTAGTTGACAAATACGGCGTAAAGAGTGGTTAAGGGGT

ATCAAAAATAAAGTCAAACATTTTCCTAGTTGTTATAAGCATTTCGAGAACATGAAGCCC

AACCACGAAAGTAACTTTACCTTCACCTGACCCCACGAAAGCTAAGAAA

>344_Sample_ASV18

CACCGCGGTTATACGAGAAACCCTAGTTGACAAATACGGCGTAAAGAGTGGTTAAGGGGT

ATCAAAAATAAAGTCAAACATTTTCCTAGTTGTTATAAGCATTTCGAGAACATGAAGCCC

AACCACGAAAGTAACTTTACCTCCACCTGACCCCACGAAAGCTAAGAAA

>344_2_Sample_ASV18

GACCGCGGTTATACGAGAGACTCTAGTTGATAGATACGGCGTAAAGAGTGGTTTGGGATA

AACTATAAATAAAGCCAAAGACCTTCCAAGCTGTTAAACGCATACCGAAGGCACGAAGCC

CCAACACGAAAGTAGCTTTACTACTACCCCCGATGCCACGAAAGCTAAGACA

>344_Sample_ASV19

CACCGCGGTTATACGAAAGACCCCAGTTTATAGACACGGCGTAAAGGGTGGTTATAGGAC

AAACTAACATAGAGCCAAAGACCCCCTAAGCCGTCATACGCTCACGAGGGCTCGAAGCCC

CTATACGAAAGTAGCTCTACCAAAAAATGCTCGACTCCACGAAAGCTGGGACA

>344_Sample_ASV20

CACCGCGGTTATACGAGAGGCCCCAGTTGATAAACACGGCGTAAAGGGTGGTTAAGGGAG

CATAAGAATAAAGCCGAAGGGCCCTTTGGCCGTTATACGCTTCTAGGTGTCCGAAGCCCA

AACACGAAAGTAGCTTTAGTTAAGCCCACCTGACTCCACGAAAACTGAGAAA

>344_2_Sample_ASV19

CACCGCGGTTATACGAGAGACCCTAGTTGATAAATACGGCGTAAAGAGTGGTTATGGGAA

AAACAAATAAAGTCAAACAACCTCTTAGCTGTTATACGCATTATGAGAGCATGAAGCCCC

CTCACGAAAGTAACTTTAATACCTCCTGACCCCACGAAAGCTAAGAAA

>344_Sample_ASV21

CACCGCGGTTATACGAGAGACCCCAGTTGATAAACACGGCGTAAAGAGTGGTTATGGGAA

AAACAAATAAAGTCAAACAACCTCTTAGCTGTTATACGCATTATGAGAGCATGAAGCCCC

CTCACGAAAGTAACTTTAATACCTCCTGACCCCACGAAAGCTAAGAAA

>344_Sample_ASV22

CACCGCGGTTATACGAGAGACCCCAGTTGATAAACACGGCGTAAAGAGTGGTTAAGGGCA

CAAAACAAATAAAGTCAAACAACCTCTTAGCTGTTATACGCATTATGAGAGCATGAAGCC

CCCTCACGAAAGTAACTTTAATACCTCCTGACCCCACGAAAGCTAAGAAA

>344_Sample_ASV23

CACCGCGGTTATACGAGAGACCCTAGTTGATAAATACGGCGTAAAGAGTGGTTATGGGAA

AAACAAATAAAGTCAAACAACCTCTTAGCTGTTATACGCATTATGAGAGCATGAAGCCCC

CTCACGAAAGTAACTTTAATACCTCCTGACCCCACGAAAGCTAAGAAA

>344_Sample_ASV24

CACCGCGGTTATACGAGAGGCCCAAGTTGACAAACCCCGGCGTAAAGAGTGGTTAAGGGA

GATCAAAACTAAAGCCGAATGCTTTCAAAGCTGTTATACGCTTCCGAAAGTAAGAAGCTC

AATCACGAAAGTGGCTTTACTTTACCTGACCCCACGAAAGCTACGACA

>344_Sample_ASV25

CACCGCGGTTATACGAGAGGCTCAAGTTGATGAACCCCGGCGTAAAGAGTGGTTAAGGGA

GATCAAAACTAAAGCCGAATGCTTTCAAAGCTGTTATACGCTTCCGAAAGTAAGAAGCTC

AATCACGAAAGTGGCTTTACACTACCTGAACCCACGAAAGCTATGAAA

>344_Sample_ASV26

CACCGCGGTTATACGAGAGGCTCAAGTTGATGAACCCCGGCGTAAAGAGTGGTTAAGGGA

GATCAAAACTAAAGCCGAATGCTTTCAAAGCTGTTATACGCTTCCGAAAGTAAGAAGCTC

AATCACGAAAGTGGCTTTACTTTACCTGACCCCACGAAAGCTACGACA

>344_Sample_ASV27

CACCGCGGTTATACGAAAGACCCTAGTTGATGGATCACGGCGTAAAGGGTGGTTAAGGGA

TATAAAAATAAAGCTAAAAATCCTCTTAGCTGTCATACGCTTTCCGAAGACATGAGGCCC

AATTACGAAAGTAGCTTTAAACCACCCTGACACCACGAAAGCCAAGAAA

>344_Sample_ASV28

CACCGCGGTTATACGAAAGACCCCAGTTTATAGATGCGGCGTAAAGGGTGGTTAAGAGAA

AATCAAAATAAGGCCAAAGATCCCCTAAGCAGTCATACGCTCCACGAGGACACGAAACCC

AAATACGAAAGTTGCCTTAATAAACTTCACCTGATCCCACGAAAGCTGAAAAA

>344_Sample_ASV29

CACCGCGGTTATACGAGAGGCCCAAGTTGACAAACCCCGGCGTAAAGAGTGGTTAAGGAA

AATTTAAAACTAAAGCCGAATGCCCCCAAAGCTGTTATACGCACCCGAGGGTAAGAAGCC

CAATCACGAAAGTGGCTTTACACTACCTGAACCCACGAAAGCTATGAAA

>344_Sample_ASV30

CACCGCGGTTATACGAGAGGCCCAAGTTGACAAACCCCGGCGTAAAGAGTGGTTAAGGAA

AATTTAAAACTAAAGCCGAATGCCCCCAAAGCTGTTATACGCACCCGAGGATAAGAAGCC

CAATCACGAAAGTGGCTTTACACTACCTGAACCCACGAAAGCTATGAAA

>344_Sample_ASV31

CACCGCGGTTATACGAGAGGCCCAAGTTGACAAACCCCGGCGTAAAGAGTGGTTAAGGAA

AATTTAAAACTAAAGCCGAATGCCCCCAAAGCTGTTATACGCACCCGAGGATAAGAAGCC

CAATCACGAAAGTGGCTTTACTTTACCTGACCCCACGAAAGCTACGACA

>344_Sample_ASV32

CACCGCGGTTATACGAGAGACCCTAGTTGATAGCTACGGCGTAAAGCGTGGTTAGGAGAA

CAAGATAAATAAAGTCAAACAATCTCTCGGCCGTTATACGTTATCTGAGAATATGAAGTC

CTACCACGAAAGTAACTTTAATTTTTCTGACCCCACGAAAACTGAGGAA

>344_2_Sample_ASV20

CACCGCGGTCATACGATTAACCCAAGTTAATAGGCACACGGCGTAAAGCGTGTTTAAGCA

CTATGCCAAATAAAGTTAAACTCCAATTAAGCTGTAAAAAGCCATAATCAAGACGAAAAT

AAATAACGAAAGTAACTTTACAACCGCTGAAACACGATAGCTAAGATC

>344_Sample_ASV33

CACCGCGGTTATACGAGAGGCCCTAGTTGATAACTACCGGCGTAAAGAGTGGTTATGGAA

AAATATTTAATAAAGCCGAACACCCCCTCAGCCGTCATACGCACCTGGGAGCACGAAGAC

CTACTGCGAAAGCAGCTTTAATTATGCCTGACCCCACGACAGCTAAGAAA

>344_2_Sample_ASV21

CACCGCGGTTATACGAAAGACCCTAGTTTATAGACACGGCGTAAAGGGTGGTTCGGGACT

AAACAAAATAAAGCCAAAGACCTCCTAAGCCGTCATACGCTCACGGAAGCACGAAGCCCA

AGTACGAAAGTAGCTTTACAAAACATGCCTGACTCCACGAAAGCTAGGAAA

>344_Sample_ASV34

CACCGCGGTTATACGAAAGACCCTAGTTTATAGACACGGCGTAAAGGGTGGTTCGGGACT

AAACAAAATAAAGCCAAAGACCTCCTAAGCCGTCATACGCTCACGGAAGCACGAAGCCCA

AGTACGAAAGTAGCTTTACAAAACATGCCTGACTCCACGAAAGCTAGGAAA

>344_2_Sample_ASV22

CACCGCGGTTATACGAGAGACCCTAGTTGATATATACGGCGTAAAGAGTGGTTTAGGACT

AAAACTAATAAAGCTAAAGATCCTCCCAGCCGTCGCACGCATTCTGAGGACACGAAACTC

AACCACGAAAGTAGCTTTAACATACTCTCCTGACCCCACGAAAGCTAAGAAA

>344_2_Sample_ASV23

CACCGCGGTTATACGAGAGACCCTAATTGATAGGCGCGGCGTAAAGAGTGGTTTAGGACT

AAAACTAATAAAGCTAAAGATCCTCCCAGCCGTCGCACGCATTCTGAGGACACGAAACTC

AACCACGAAAGTAGCTTTAACATACTCTCCTGACCCCACGAAAGCTAAGAAA

>344_2_Sample_ASV24

CACCGCGGTTATACGAAAGACCCTAGTTGATAGGCACGGCGTAAAGGGTGGTTAGGGATA

TATCAAAAATAAAGTTAAAGAGCCTCTAAGCTGTCGCACGCATCCCGAGAGCTCGAGACC

CAGACACGAAAGTAACTTTAAAATACCTCACCTGACCCCACGAAAGCTAAGAAA

>344_2_Sample_ASV25

CACCGCGGTTATACGAAAGACCCTAGTTGATAGCCACGGCGTAAAGGGTGGTTAAGGTAT

ACAACAAATAAAGCTAAATAGCCTCTAGGCCGTCGCACGCATTCCGAGAGCTCGAAACCC

AAAAACGAAAGTAGCTTTAAAATAAACAGACCTGACCCCACGAAAGCTAAGAAA

>344_2_Sample_ASV26

CACCGCGGTTATACGAGAGGCCCAAGTTGATACTCCACGGCGTAAAGAGTGGTTAGAAAA

GACCTACTACTAAAGCCGAACGCCTTCAAAGCTGTTATACGCATCCGAAGGTGAGAAGCC

CACCCACGAAAGTGGCTTTACAACCTTGACTCCACGAAAGCTATGACA

>344_2_Sample_ASV27

CACCGCGGTTATACGAGAGGCCCAAGTTGATACTCCACGGCGTAAAGAGTGGTTAGAAAA

GACCTACTACTAAAGCCGAACGCCTTCAAAGCTGTTATACGCATCCGAAGGTGAGAAGCC

CACCCACGAAAGTGGCTTTACAACCTTGACTCCACGAAAGCTGGGACA

>344_Sample_ASV35

CACCGCGGTTATACGAGAGGCCCAAGTTGATACTCCACGGCGTAAAGAGTGGTTAGAAAA

GACCTACTACTAAAGCCGAACGCCTTCAAAGCTGTTATACGCATCCGAAGGTGAGAAGCC

CACCCACGAAAGTGGCTTTACAACCTTGACTCCACGAAAGCTATGACA

>344_2_Sample_ASV28

CACCGCGGTTATACGAGAGACCCCAGTTGATAAACACGGCGTAAAGAGTGGTTAAGGGCA

CAAAACAAATAAAGTCAAACAACCTCTTAGCTGTTATACGCATTATGAGGATATGAAGCC

CCCCTACGAAAGTAACTTTACCAACCCCTGACCCCACGAAAGCTAAGAAA

>344_Sample_ASV36

CACCGCGGTTATACGAGAGACCCCAGTTGATAAACACGGCGTAAAGAGTGGTTAAGGGCA

CAAAACAAATAAAGTCAAACAACCTCTTAGCTGTTATACGCATTATGAGGATATGAAGCC

CCCCTACGAAAGTAACTTTACCAACCCCTGACCCCACGAAAGCTAAGAAA

>344_2_Sample_ASV29

CACCGCGGTTATACGAAAGACCCTAGTTTATAGGCACGGCGTAAAGGGTGGTTAGGGGAT

AAACAAAATAAAGCCAAAGACCTTCTAAGTCGTCATACACTCATGAAGGCACGAAGCCCA

AATACGAAAGTAACTTTACAACTTAGACCCGACTCCACGAAAGCTGGGGGA

>344_Sample_ASV37

CACCGCGGTTATACGAAAGACCCTAGTTTATAGGCACGGCGTAAAGGGTGGTTAGGGGAT

AAACAAAATAAAGCCAAAGACCTTCTAAGTCGTCATACACTCATGAAGGCACGAAGCCCA

AATACGAAAGTAACTTTACAACTTAGACCCGACTCCACGAAAGCTGGGGGA

>344_Sample_ASV38

CACCGCGGTTATACGAGAGACCCTAATTAATAGATACGGCGTAAAGAGTGGTTTAGGACA

AAAATTAATAAAGCCGAAGTTTCTCCTAGCCGTTGTACGCACTCTGAGAACACGAAGCCC

CACTACGAAAGTAGCTTTACCAATAATCTCCTGACCCCACGAAAGCTAAGAAA

>344_Sample_ASV39

CACCGCGGTTATACGAGAGACCCTAATTAATAGATACGGCGTAAAGAGTGGTTTAGGACA

AAAATTAATAAAGCCGAAGTTTCTCCTAGCCGTTGTACGCACTCTGAGAACACGAAGCCC

CACTACGAAAGTAGCTTTACCTATTATTTCCTGACCCCACGAAAGCTAAGAAA

>344_Sample_ASV40

CACCGCGGTTATACGAGAGACCCTAATTAATAGCTACGGCGTAAAGAGTGGTTTAGGGTA

AAAATTAATAAAGCCGAAGTTTCTCCTAGCCGTTGTACGCACTCTGAGAACACGAAGCCC

CACTACGAAAGTAGCTTTACCTATTATTTCCTGACCCCACGAAAGCTAAGAAA

>344_Sample_ASV41

CACCGCGGTTATACGAGAGACCCTAATTAATAGCTACGGCGTAAAGAGTGGTTTAGGACA

AAAATTAATAAAGCCGAAGTTTCTCCTAGCCGTTGTACGCACTCTGAGAACACGAAGCCC

CACTACGAAAGTAGCTTTACCAATAATCTCCTGACCCCACGAAAGCTAAGAAA

>344_Sample_ASV42

CACCGCGGTTATACGAGAGACCCTAATTAATAGATACGGCGTAAAGAGTGGTTTAGGGTA

AAAATTAATAAAGCCGAAGTTTCTCCTAGCCGTTGTACGCACTCTGAGAACACGAAGCCC

CACTACGAAAGTAGCTTTACCTATTATTTCCTGACCCCACGAAAGCTAAGAAA

>344_Sample_ASV43

CACCGCGGTTATACGAGAGACCCTAATTAATAGCTACGGCGTAAAGAGTGGTTTAGGACA

AAAATTAATAAAGCCGAAGTTTCTCCTAGCCGTTGTACGCACTCTGAGAACACGAAGCCC

CACTACGAAAGTAGCTTTACCTATTATTTCCTGACCCCACGAAAGCTAAGAAA

>344_Sample_ASV44

CACCGCGGTTATACGAGAGGCCCAAGTTGATAGACATCGGCGTAAAGAGTGGTTAAGGAA

GTCTCAAAACTAAAGCCGAATGCCTTCAAGACTGTTATACGTTTCCGAAGGTAAGAAGCC

CCACTACGAAAGTGGCTTTACTCCACCTGACTCCACGAAAGCTGTGAAA

>344_Sample_ASV45

CACCGCGGTTATACGAGAGGCCCAAGTTGACAAACTCCGGCGTAAAGCGTGGTTAAGGCA

CACTCAAAAACTAAAGCCGAATATCTTCAGAGCAGTCATACGCATCCGAAGACACGAAGC

CCCACCACGAAAGTGGCTTTATGACACCTGACCCCACGAAAGCTAGGACA

>344_2_Sample_ASV30

CACCGCGGTTATACGAAAGACCCTAGTTGATAGATCACGGCGTAAAGGGTGGTTAAGGAG

AACAAAAATAAAGCTAAAAATCTTCTAAGCTGTCATACGCTTTCCGAAGACATGAGACCC

AACCACGAAAGTAGCTTTAAACTTTCCTGACACCACGAAAGCCAAGAAA

>344_Sample_ASV46

CACCGCGGTTATACGAAAGACCCTAGTTGATAGATCACGGCGTAAAGGGTGGTTAAGGAG

AACAAAAATAAAGCTAAAAATCCTCTAAGCTGTCATACGCTTTCCGAAGACATGAGACCC

AACCACGAAAGTAACTTTAAACTTTCCTGACACCACGAAAGCCAAGAAA

>347_Sample_ASV1

CACCGCGGTTATACGAGAGGCCCAAGTTGACAGACATCGGCGTAAAGAGTGGTTAAGGAA

AACCTTCAACTAAAGTCGAACGCTCTCAAGACTGTCGTACGTTTCCGAGAGTATGAAGCC

CCACCACGAAAGTGACTTTACCTCACCTGACTCCACGAAAGCTGCGAAA

>347_Sample_ASV2

CACCGCGGTTATACGAAAGACCCTAGTTGATGGGTGCGGCGTAAAGGGTGGTTAGGGACT

ACAAAAAATAGAGCTAAAGATCCTCTAGGTCGTCATACACATCACGAGGAAACGAAGCCC

AAATACGAAAGTAGCTCTAAATATAATCCTGACGCCACGAAAGCCAAGAAA

>347_Sample_ASV3

CACCGCGGTTATACGAGAGACCCTAGTTGATTAAAGCGGCGTAAAGAGTGGTTATGGAAC

TATTCTTTTAAAGCAGAAAACCTCTCAAACTGTTATACGCACCCAGAGGTCTGAATCCCT

CACACGAAAGTGACTTTATTTATGCCTACCAGAACCCACGAAAGCTGGGACA

>347_Sample_ASV4

CACCGCGGTTATACGAGAGACCCTAGTTGATTAAAGCGGCGTAAAGAGTGGTTATGGAAC

TATTCTTTTAAAGCAGAAAACCTCTCAAACTGTTATACGCACCCAGAGGTCTGAATCCCT

CACACGAAAGTGACTTTATTTATGCCTACCAGAACCCACGAAAGCTATGACA

>347_Sample_ASV5

CACCGCGGTTATACGAAAGACCCTAGTTTATAGGTACGGCGTAAAGGGTGGTTAGGGGAC

AAACAAAATAAAGCCAAAGACCTCCTAAGCCGTCATACGCTCACGGAAGCACGAAGCCCA

AACACGAAAGTAGCTTTACCGAACATGCCCGACTCCACGAAAGCTGGGGAA

>347_Sample_ASV6

CACCGCGGTTATACGAGAGGCCCAAGTTGACAGACACCGGCGTAAAGAGTGGCTAGGGAA

AAATTACTACTAAAGCCGAACACCTTCAGAACTGTCATACGTTCCCGAAGATAAGAAGCC

CCACCACGAAAGTGGCTTTATATTACCCGACCCCACGAAAGCTGTGAAA

>347_Sample_ASV7

CACCGCGGTTATACGAGAGGCCCAAGTTGACAGACACCGGCGTAAAGAGTGGCTAAGGAA

AAATTTCTACTAAAGCCGAACACCTTCAGAACTGTCATACGTTCCCGAAGAAAAGAAGCC

CCACCACGAAAGTGGCTTTATATTACCCGACCCCACGAAAGCTGTGAAA

>347_Sample_ASV8

CACCGCGGTTAAACGAGAGACCCTAGTTGATCATCACGGCGTAAAGAGTGGTTAGGGGAT

TATCATAAATAAAGCCAAAGACCTCCCAAGCTGTCGCACGCATTCCGGAGGCGCGAAGCC

CACACACGAAAGTAGCTTTAACTATTGACCCTGATGCCACGAAAGCTAAGAAA

>347_Sample_ASV9

CACCGCGGTTATACGAGAGGCCCAAGTTGATAAGCCCGGCGTAAAGAGTGGTTATGGAGA

ATAAAACACTAAAGCTAAAGACCCCCTAGGCTGTTATACGCACCTGGCGGCTCGAACCAC

CTATACGAAAGTAGCTTTACCCCCCTTCCACCAGAATCCACGACAGCTGGGGCA

>347_Sample_ASV10

CACCGCGGTTATACGAGAGGCCCAAGTTGACAGACATCGGCGTAAAGAGTGGTTAAGGAA

ACCCCATTACTAAAGCCGAACGCCCTCAGAACTGTCATACGTTCCCGAAGGTAAGAAGCC

CCACTACGAAAGTGGCTTTAACCACACCCGACCCCACGAAAGCTGTGAAA

>347_Sample_ASV11

CACCGCGGTTATACGAGAGGCCCAAGTTGACAGACATCGGCGTAAAGAGTGGTTAAGGAA

ACCCCATTACTAAAGCCGAACGCCCTCAGAACTGTCATACGTTCCCGAAGGTAAGAAGCC

CCACTACGAAAGTGGCTTTAACCACACCCGACCCCACGAAAGCTAAGAAA

>347_Sample_ASV12

CACCGCGGTTATACGAGAGGCCCAAGTTGATAGATATCGGCGTAAAGAGTGGTTAAGGAA

ACCCCGTTACTAAAGCCGAACGCCCTCAGAACTGTCATACGTTCCCGAAGGTAAGAAGCC

CCACTACGAAAGTGGCTTTAACCACACCCGACCCCACGAAAGCTGTGAAA

>347_Sample_ASV13

CACCGCGGTTATACGAGAGGCCCAAGTTGATAGATATCGGCGTAAAGAGTGGTTAAGGAA

ACCCCGTTACTAAAGCCGAACGCCCTCAGAACTGTCATACGTTCCCGAAGGTAAGAAGCC

CCACTACGAAAGTGGCTTTAACCACACCCGACCCCACGAAAGCTAAGAAA

>347_Sample_ASV14

CACCGCGGTCACACGATTAACCCAAGTCAATAGAAGCCGGCGTAAAGAGTGTTTTAGATC

ACCCCCTCCCCAATAAAGCTAAAACTCACCTGAGTTGTAAAAAACTCCAGTTGACACAAA

ATAGACTACGAAAGTGGCTTTAACATATCTGAACACACAATAGCTAAGACC

>347_Sample_ASV15

CACCGCGGTTATACGAGAGGCCCTAGTTGATAAATACGGCGTAAAGAGTGGTTAGAGGAC

TCCCATAATAAAGCCAAAGACCCCCCGGGCCGTCACACGCATACGAGGGCACGAAGTTCA

CACACGAAAGTAGCTTTAAATTACCCGACGCCACGAAAGCTAAGAAA

>347_Sample_ASV16

GACCGCGGTTATACGAGAGACTCTAGTTGATAGATACGGCGTAAAGAGTGGTTTGGGATA

AACTATAAATAAAGCCAAAGACCTTCCAAGCTGTTAAACGCATACCGAAGGCACGAAGCC

CCAACACGAAAGTAGCTTTACTACTACCCCCGATGCCACGAAAGCTAAGACA

>347_Sample_ASV17

CACCGCGGTTATACGAGAGACCCTAGTTGATAAATACGGCGTAAAGAGTGGTTATGGGAA

AAACAAATAAAGTCAAACAACCTCTTAGCTGTTATACGCATTATGAGAGCATGAAGCCCC

CTCACGAAAGTAACTTTAATACCTCCTGACCCCACGAAAGCTAAGAAA

>347_Sample_ASV18

CACCGCGGTCATACGATTAACCCAAGTTAATAGGCACACGGCGTAAAGCGTGTTTAAGCA

CTATGCCAAATAAAGTTAAACTCCAATTAAGCTGTAAAAAGCCATAATCAAGACGAAAAT

AAATAACGAAAGTAACTTTACAACCGCTGAAACACGATAGCTAAGATC

>347_Sample_ASV19

CACCGCGGTTATACGAAAGACCCTAGTTTATAGACACGGCGTAAAGGGTGGTTCGGGACT

AAACAAAATAAAGCCAAAGACCTCCTAAGCCGTCATACGCTCACGGAAGCACGAAGCCCA

AGTACGAAAGTAGCTTTACAAAACATGCCTGACTCCACGAAAGCTAGGAAA

>347_Sample_ASV20

CACCGCGGTTATACGAAAGACCCTAGTTGATAGGCACGGCGTAAAGGGTGGTTAGGGATA

TATCAAAAATAAAGTTAAAGAGCCTCTAAGCTGTCGCACGCATCCCGAGAGCTCGAGACC

CAGACACGAAAGTAACTTTAAAATACCTCACCTGACCCCACGAAAGCTAAGAAA

>347_Sample_ASV21

CACCGCGGTTATACGAAAGACCCTAGTTGATAGCCACGGCGTAAAGGGTGGTTAAGGTAT

ACAACAAATAAAGCTAAATAGCCTCTAGGCCGTCGCACGCATTCCGAGAGCTCGAAACCC

AAAAACGAAAGTAGCTTTAAAATAAACAGACCTGACCCCACGAAAGCTAAGAAA

>347_Sample_ASV22

CACCGCGGTTATACGAGAGGCCCAAGTTGATACTCCACGGCGTAAAGAGTGGTTAGAAAA

GACCTACTACTAAAGCCGAACGCCTTCAAAGCTGTTATACGCATCCGAAGGTGAGAAGCC

CACCCACGAAAGTGGCTTTACAACCTTGACTCCACGAAAGCTATGACA

>347_Sample_ASV23

CACCGCGGTTATACGAGAGACCCCAGTTGATAAACACGGCGTAAAGAGTGGTTAAGGGCA

CAAAACAAATAAAGTCAAACAACCTCTTAGCTGTTATACGCATTATGAGGATATGAAGCC

CCCCTACGAAAGTAACTTTACCAACCCCTGACCCCACGAAAGCTAAGAAA

>347_Sample_ASV24

CACCGCGGTTATACGAAAGACCCTAGTTTATAGGCACGGCGTAAAGGGTGGTTAGGGGAT

AAACAAAATAAAGCCAAAGACCTTCTAAGTCGTCATACACTCATGAAGGCACGAAGCCCA

AATACGAAAGTAACTTTACAACTTAGACCCGACTCCACGAAAGCTGGGGGA

>347_Sample_ASV25

CACCGCGGTTATACGAGAGACCCTAATTAATAGATACGGCGTAAAGAGTGGTTTAGGACA

AAAATTAATAAAGCCGAAGTTTCTCCTAGCCGTTGTACGCACTCTGAGAACACGAAGCCC

CACTACGAAAGTAGCTTTACCTATTATTTCCTGACCCCACGAAAGCTAAGAAA

>347_Sample_ASV26

CACCGCGGTTATACGAGAGGCCCAAGTTGATAGACATCGGCGTAAAGAGTGGTTAAGGAA

GTCTCAAAACTAAAGCCGAATGCCTTCAAGACTGTTATACGTTTCCGAAGGTAAGAAGCC

CCACTACGAAAGTGGCTTTACTCCACCTGACTCCACGAAAGCTGTGAAA

>347_Sample_ASV27

CACCGCGGTTATACGAAAGACCCTAGTTGATAGATCACGGCGTAAAGGGTGGTTAAGGAG

AACAAAAATAAAGCTAAAAATCTTCTAAGCTGTCATACGCTTTCCGAAGACATGAGACCC

AACCACGAAAGTAGCTTTAAACTTTCCTGACACCACGAAAGCCAAGAAA

>348_Sample_ASV1

CACCGCGGTTATACGAAAGACCCTAGTTGATGGGTGCGGCGTAAAGGGTGGTTAGGGACT

ACAAAAAATAGAGCTAAAGATCCTCTAGGTCGTCATACACATCACGAGGAAACGAAGCCC

AAATACGAAAGTAGCTCTAAATATAATCCTGACGCCACGAAAGCCAAGAAA

>348_Sample_ASV2

CACCGCGGTTATACGAGAGACCCTAGTTGATTAAAGCGGCGTAAAGAGTGGTTATGGAAC

TATTCTTTTAAAGCAGAAAACCTCTCAAACTGTTATACGCACCCAGAGGTCTGAATCCCT

CACACGAAAGTGACTTTATTTATGCCTACCAGAACCCACGAAAGCTGGGACA

>348_Sample_ASV3

CACCGCGGTTATACGAAAGACCCTAGTTTATAGGTACGGCGTAAAGGGTGGTTAGGGGAC

AAACAAAATAAAGCCAAAGACCTCCTAAGCCGTCATACGCTCACGGAAGCACGAAGCCCA

AACACGAAAGTAGCTTTACCGAACATGCCCGACTCCACGAAAGCTGGGGAA

>348_Sample_ASV4

CACCGCGGTTATACGAGAGGCCCAAGTTGACAGACACCGGCGTAAAGAGTGGCTAGGGAA

AAATTACTACTAAAGCCGAACACCTTCAGAACTGTCATACGTTCCCGAAGATAAGAAGCC

CCACCACGAAAGTGGCTTTATATTACCCGACCCCACGAAAGCTGTGAAA

>348_Sample_ASV5

CATGGCGATTATACGAGAGGCCCAAGTTGACAGACACTGGCGTAAAGAGTGGCTAGGGAA

AAATTACTACTAAAGCCGAACACCTTCAGAACTGTCATACGTTCCCGAAGATAAGAAGCC

CCACCACGAAAGTGGCTTTATATTACCCGACCCCACGAAAGCTGTGAAA

>348_Sample_ASV6

CACCGCGGTTATACGAGAGACCCTAGTTGATATATACGGCGTAAAGAGTGGTTTGGGGTT

ATGCCTCCAAAATAAAGCCGAAGATCTCCCAAGCCGTCACACGCATCCCGGAGACACGAA

GCCCTAATACGAAGGTAGCTTTATTAATTCCCCGACGCCACGAAAGCTAAGAAA

>348_Sample_ASV7

CACCGCGGTTATACGAGAGACCCTAGTTGATATATACGGCGTAAAGAGTGGTTTGGGGTT

ATGCCTCCAAAATAAAGCCGAAGATCTCCCAAGCCGTCACACGCATCCCGGAGACACGAA

GCCCTAATACGAAGGTAGCTTTATTAGTACCCCGACGCCACGAAAGCTAAGAAA

>348_Sample_ASV8

CACCGCGGTTATACGAGAGGCCCAAGTTGATAAGCCCGGCGTAAAGAGTGGTTATGGAGA

ATAAAACACTAAAGCTAAAGACCCCCTAGGCTGTTATACGCACCTGGCGGCTCGAACCAC

CTATACGAAAGTAGCTTTACCCCCCTTCCACCAGAATCCACGACAGCTGGGGCA

>348_Sample_ASV9

CACCGCGGTTATACGAGAGGCCCTAGTTGATAGCCACGGCGCAAAGAGTGGTTAAGGAGC

CCCACTAAATAAAGTCGAACACTTCCTAGGCCGTTATACGTTTTCTAGAAGCACGAAACC

CAATTACACGAAAGCAACTTTATACTAAACAACCTGACCCCACGAAAGCTAAGAAA

>348_Sample_ASV10

CACCGCGGTTATACGAGAGGCCCAAGTTGACAGACATCGGCGTAAAGAGTGGTTAAGGAA

ACCCCATTACTAAAGCCGAACGCCCTCAGAACTGTCATACGTTCCCGAAGGTAAGAAGCC

CCACTACGAAAGTGGCTTTAACCACACCCGACCCCACGAAAGCTGTGAAA

>348_Sample_ASV11

CACCGCGGTTATACGAGAGGCCCAAGTTGATAGATATCGGCGTAAAGAGTGGTTAAGGAA

ACCCCGTTACTAAAGCCGAACGCCCTCAGAACTGTCATACGTTCCCGAAGGTAAGAAGCC

CCACTACGAAAGTGGCTTTAACCACACCCGACCCCACGAAAGCTGTGAAA

>348_Sample_ASV12

CACCGCGGTTATACGAGAGGCCCAAGTTGACAGACACCGGCGTAAAGAGTGGTTAAGGAA

ATTCATCAACTAAAGCCGAATGCCCTCAGGACTGTTATACGTTTGCGAAGGTAAGAAGCC

CTACTACGAAAGTGGCTTTATTCCACCTGATTCCACGAAAGCTGCGGAA

>348_Sample_ASV13

CACCGCGGTTATACGAGAGGCCCTAGTTGATAAATACGGCGTAAAGAGTGGTTAGAGGAC

TCCCATAATAAAGCCAAAGACCCTCCGGGCCGTCACACGCATACGAGGGCACGAAGTTCA

CACACGAAAGTAGCTTTAAATTACCCGACGCCACGAAAGCTAAGAAA

>348_Sample_ASV14

CACCGCGGTTATACGAAAGACCCCAGTTTATAGACACGGCGTAAAGGGTGGTTATAGGAC

AAACTAACATAGAGCCAAAGACCCCCTAAGCCGTCATACGCTCACGAGGGCTCGAAGCCC

CTATACGAAAGTAGCTCTACCAAAAAATGCTCGACTCCACGAAAGCTGGGACA

>348_Sample_ASV15

CACCGCGGTTATACGAGAGGCCCCAGTTGATAAACACGGCGTAAAGGGTGGTTAAGGGAG

CATAAGAATAAAGCCGAAGGGCCCTTTGGCCGTTATACGCTTCTAGGTGTCCGAAGCCCA

AACACGAAAGTAGCTTTAGTTAAGCCCACCTGACTCCACGAAAACTGAGAAA

>348_Sample_ASV16

CACCGCGGTTATACGAGAGACCCTAGTTGATAAATACGGCGTAAAGAGTGGTTATGGGAA

AAACAAATAAAGTCAAACAACCTCTTAGCTGTTATACGCATTATGAGAGCATGAAGCCCC

CTCACGAAAGTAACTTTAATACCTCCTGACCCCACGAAAGCTAAGAAA

>348_Sample_ASV17

CACCGCGGTTATACGAGAGGCTCAAGTTGATAAACCCCGGCGTAAAGAGTGGTTAAGGAA

AAATAGAAACTAAAGCCGAATGCTTTCAAGACTGTTATACGTTCCCGAGAGTAAGAAGAC

CAATTACGAAAGTGGCTTTACTTTACCTGACCCCACGAAAGCTACGACA

>348_Sample_ASV18

CACCGCGGTTATACGAGAGGCTCAAGTTGATGAACCCCGGCGTAAAGAGTGGTTAAGGGA

GATCAAAACTAAAGCCGAATGCTTTCAAAGCTGTTATACGCTTCCGAAAGTAAGAAGCTC

AATCACGAAAGTGGCTTTACTTTACCTGACCCCACGAAAGCTACGACA

>348_Sample_ASV19

CACCGCGGTTATACGAGAGGCCCAAGTTGACAAACCCCGGCGTAAAGAGTGGTTAAGGGA

GATCAAAACTAAAGCCGAATGCTTTCAAAGCTGTTATACGCTTCCGAAAGTAAGAAGCTC

AATCACGAAAGTGGCTTTACTTTACCTGACCCCACGAAAGCTACGACA

>348_Sample_ASV20

CACCGCGGTTATACGAAAGACCCTAGTTGATGGATCACGGCGTAAAGGGTGGTTAAGGGA

TATAAAAATAAAGCTAAAAATCCTCTTAGCTGTCATACGCTTTCCGAAGACATGAGGCCC

AATTACGAAAGTAGCTTTAAACCACCCTGACACCACGAAAGCCAAGAAA

>348_Sample_ASV21

CACCGCGGTTATACGAAAGACCCCAGTTTATAGATGCGGCGTAAAGGGTGGTTAAGAGAA

AATCAAAATAAGGCCAAAGATCCCCTAAGCAGTCATACGCTCCACGAGGACACGAAACCC

AAATACGAAAGTTGCCTTAATAAACTTCACCTGATCCCACGAAAGCTGAAAAA

>348_Sample_ASV22

CACCGCGGTTATACGAGAGGCCCAAGTTGACAAACCCCGGCGTAAAGAGTGGTTAAGGAA

AATTTAAAACTAAAGCCGAATGCCCCCAAAGCTGTTATACGCACCCGAGGATAAGAAGCC

CAATCACGAAAGTGGCTTTACACTACCTGAACCCACGAAAGCTATGAAA

>348_Sample_ASV23

CACCGCGGTTATACGAGAGGCCCAAGTTGACAAACCCCGGCGTAAAGAGTGGTTAAGGAA

AATTTAAAACTAAAGCCGAATGCCCCCAAAGCTGTTATACGCACCCGAGGGTAAGAAGCC

CAATCACGAAAGTGGCTTTACACTACCTGAACCCACGAAAGCTATGAAA

>348_Sample_ASV24

CACCGCGGTTATACGAGAGACCCTAGTTGATAGCTACGGCGTAAAGCGTGGTTAGGAGAA

CAAGATAAATAAAGTCAAACAATCTCTCGGCCGTTATACGTTATCTGAGAATATGAAGTC

CTACCACGAAAGTAACTTTAATTTTTCTGACCCCACGAAAACTGAGGAA

>348_Sample_ASV25

CACCGCGGTTATACGAGAGGCCCTAGTTGATAACTACCGGCGTAAAGAGTGGTTATGGAA

AAATATTTAATAAAGCCGAACACCCCCTCAGCCGTCATACGCACCTGGGAGCACGAAGAC

CTACTGCGAAAGCAGCTTTAATTATGCCTGACCCCACGACAGCTAAGAAA

>348_Sample_ASV26

CACCGCGGTTATACGAAAGACCCTAGTTTATAGACACGGCGTAAAGGGTGGTTCGGGACT

AAACAAAATAAAGCCAAAGACCTCCTAAGCCGTCATACGCTCACGGAAGCACGAAGCCCA

AGTACGAAAGTAGCTTTACAAAACATGCCTGACTCCACGAAAGCTAGGAAA

>348_Sample_ASV27

CACCGCGGTTATACGAGAGGCCCAAGTTGATACTCCACGGCGTAAAGAGTGGTTAGAAAA

GACCTACTACTAAAGCCGAACGCCTTCAAAGCTGTTATACGCATCCGAAGGTGAGAAGCC

CACCCACGAAAGTGGCTTTACAACCTTGACTCCACGAAAGCTATGACA

>348_Sample_ASV28

CACCGCGGTTATACGAGAGACCCCAGTTGATAAACACGGCGTAAAGAGTGGTTAAGGGCA

CAAAACAAATAAAGTCAAACAACCTCTTAGCTGTTATACGCATTATGAGGATATGAAGCC

CCCCTACGAAAGTAACTTTACCAACCCCTGACCCCACGAAAGCTAAGAAA

>348_Sample_ASV29

CACCGCGGTTATACGAAAGACCCTAGTTTATAGGCACGGCGTAAAGGGTGGTTAGGGGAT

AAACAAAATAAAGCCAAAGACCTTCTAAGTCGTCATACACTCATGAAGGCACGAAGCCCA

AATACGAAAGTAACTTTACAACTTAGACCCGACTCCACGAAAGCTGTGAAA

>348_Sample_ASV30

CACCGCGGTTATACGAAAGACCCTAGTTTATAGGCACGGCGTAAAGGGTGGTTAGGGGAT

AAACAAAATAAAGCCAAAGACCTTCTAAGTCGTCATACACTCATGAAGGCACGAAGCCCA

AATACGAAAGTAACTTTACAACTTAGACCCGACTCCACGAAAGCTGGGGGA

>348_Sample_ASV31

CACCGCGGTTATACGAGAGGCCCAAGTTGATAGACATCGGCGTAAAGAGTGGTTAAGGAA

GTCTCAAAACTAAAGCCGAATGCCTTCAAGACTGTTATACGTTTCCGAAGGTAAGAAGCC

CCACTACGAAAGTGGCTTTACTCCACCTGACTCCACGAAAGCTGTGAAA

>348_Sample_ASV32

CACCGCGGTTATACGAGAGGCCCAAGTTGATAGACATCGGCGTAAAGAGTGGTTAAGGAA

GTCTCAAAACTAAAGCCGAATGCCTTCAAGACTGTTATACGTTTCCGAAGGTAAGAAGCC

CCACTACGAAAGTGGCTTTACTCCACCTGACTCCACGAAAGCTAAGAAA

>348_Sample_ASV33

CACCGCGGTTATACGAGAGACTCCAGTTGATAGCTACGGCGTAAAGAGTGGTTTGGGGCC

CGCCCAAAATAAAGCCAAAGACCTCCCAAGCCGTCAAACGCACCCCGGAGGCACGAAGTC

CTAACGCGAAAGCAACTTTACCTTCCCCGACGCCACGAAAGCTAAGAAA

>359_Sample_ASV1

CACCGCGGTTATACGAAAGACCCTAGTTGATGGGTGCGGCGTAAAGGGTGGTTAGGGACT

ACAAAAAATAGAGCTAAAGATCCTCTAGGTCGTCATACACATCACGAGGAAACGAAGCCC

AAATACGAAAGTAGCTCTAAATATAATCCTGACGCCACGAAAGCCAAGAAA

>359_Sample_ASV2

CACCGCGGTTATACGAGAGACCCTAGTTGATTAAAGCGGCGTAAAGAGTGGTTATGGAAC

TATTCTTTTAAAGCAGAAAACCTCTCAAACTGTTATACGCACCCAGAGGTCTGAATCCCT

CACACGAAAGTGACTTTATTTATGCCTACCAGAACCCACGAAAGCTGGGACA

>359_Sample_ASV3

CACCGCGGTTATACGAAAGACCCTAGTTTATAGGTACGGCGTAAAGGGTGGTTAGGGGAC

AAACAAAATAAAGCCAAAGACCTCCTAAGCCGTCATACGCTCACGGAAGCACGAAGCCCA

AACACGAAAGTAGCTTTACCGAACATGCCCGACTCCACGAAAGCTGGGGAA

>359_Sample_ASV4

CACCGCGGTTATACGAGAGGCCCAAGTTGACAGACACCGGCGTAAAGAGTGGCTAGGGAA

AAATTACTACTAAAGCCGAACACCTTCAGAACTGTCATACGTTCCCGAAGATAAGAAGCC

CCACCACGAAAGTGGCTTTATATTACCCGACCCCACGAAAGCTGTGAAA

>359_Sample_ASV5

CACCGCGGTTATACGAGAGGCCCAAGTTGACAGACACCGGCGTAAAGAGTGGCTAAGGAA

AAATTTCTACTAAAGCCGAACACCTTCAGAACTGTCATACGTTCCCGAAGAAAAGAAGCC

CCACCACGAAAGTGGCTTTATATTACCCGACCCCACGAAAGCTGTGAAA

>359_Sample_ASV6

CACCGCGGTTATACGAGAGACCCTAGTTGATATATACGGCGTAAAGAGTGGTTTGGGGTT

ATGCCTCCAAAATAAAGCCGAAGATCTCCCAAGCCGTCACACGCATCCCGGAGACACGAA

GCCCTAATACGAAGGTAGCTTTATTAGTACCCCGACGCCACGAAAGCTAAGAAA

>359_Sample_ASV7

CACCGCGGTTATACGAGAGGCCCAAGTTGATAAGCCCGGCGTAAAGAGTGGTTATGGAGA

ATAAAACACTAAAGCTAAAGACCCCCTAGGCTGTTATACGCACCTGGCGGCTCGAACCAC

CTATACGAAAGTAGCTTTACCCCCCTTCCACCAGAATCCACGACAGCTGGGGCA

>359_Sample_ASV8

CACCGCGGTTATACGAGAGGCCCTAGTTGATAGCCACGGCGCAAAGAGTGGTTAAGGAGC

CCCACTAAATAAAGTCGAACACTTCCTAGGCCGTTATACGTTTTCTAGAAGCACGAAACC

CAATTACACGAAAGCAACTTTATACTAAACAACCTGACCCCACGAAAGCTAAGAAA

>359_Sample_ASV9

CACCGCGGTTATACGAGAGGCCCAAGTTGACAGACATCGGCGTAAAGAGTGGTTAAGGAA

ACCCCATTACTAAAGCCGAACGCCCTCAGAACTGTCATACGTTCCCGAAGGTAAGAAGCC

CCACTACGAAAGTGGCTTTAACCACACCCGACCCCACGAAAGCTGTGAAA

>359_Sample_ASV10

CACCGCGGTTATACGAGAGGCCCAAGTTGATAGATATCGGCGTAAAGAGTGGTTAAGGAA

ACCCCGTTACTAAAGCCGAACGCCCTCAGAACTGTCATACGTTCCCGAAGGTAAGAAGCC

CCACTACGAAAGTGGCTTTAACCACACCCGACCCCACGAAAGCTGTGAAA

>359_Sample_ASV11

CACCGCGGTTATACGAGAGGCCCAAGTTGACAGACACCGGCGTAAAGAGTGGTTAAGGAA

ATTCATCAACTAAAGCCGAATGCCCTCAGGACTGTTATACGTTTGCGAAGGTAAGAAGCC

CTACTACGAAAGTGGCTTTATTCCACCTGATTCCACGAAAGCTGCGGAA

>359_Sample_ASV12

CACCGCGGTCACACGATTAACCCAAGTCAATAGAAGCCGGCGTAAAGAGTGTTTTAGATC

ACCCCCTCCCCAATAAAGCTAAAACTCACCTGAGTTGTAAAAAACTCCAGTTGACACAAA

ATAGACTACGAAAGTGGCTTTAACATATCTGAACACACAATAGCTAAGACC

>359_Sample_ASV13

CACCGCGGTTATACGAGAGGCCCCAGTTGATAAACACGGCGTAAAGGGTGGTTAAGGGAG

CATAAGAATAAAGCCGAAGGGCCCTTTGGCCGTTATACGCTTCTAGGTGTCCGAAGCCCA

AACACGAAAGTAGCTTTAGTTAAGCCCACCTGACTCCACGAAAACTGAGAAA

>359_Sample_ASV14

CACCGCGGTTATACGAGAGACCCTAGTTGATAAATACGGCGTAAAGAGTGGTTATGGGAA

AAACAAATAAAGTCAAACAACCTCTTAGCTGTTATACGCATTATGAGAGCATGAAGCCCC

CTCACGAAAGTAACTTTAATACCTCCTGACCCCACGAAAGCTAAGAAA

>359_Sample_ASV15

CACCGCGGTTATACGAGAGGCTCAAGTTGATGAACCCCGGCGTAAAGAGTGGTTAAGGGA

GATCAAAACTAAAGCCGAATGCTTTCAAAGCTGTTATACGCTTCCGAAAGTAAGAAGCTC

AATCACGAAAGTGGCTTTACTTTACCTGACCCCACGAAAGCTACGACA

>359_Sample_ASV16

CACCGCGGTTATACGAGAGGCCCAAGTTGACAAACCCCGGCGTAAAGAGTGGTTAAGGGA

GATCAAAACTAAAGCCGAATGCTTTCAAAGCTGTTATACGCTTCCGAAAGTAAGAAGCTC

AATCACGAAAGTGGCTTTACTTTACCTGACCCCACGAAAGCTACGACA

>359_Sample_ASV17

CACCGCGGTTATACGAAAGACCCTAGTTGATGGATCACGGCGTAAAGGGTGGTTAAGGGA

TATAAAAATAAAGCTAAAAATCCTCTTAGCTGTCATACGCTTTCCGAAGACATGAGGCCC

AATTACGAAAGTAGCTTTAAACCACCCTGACACCACGAAAGCCAAGAAA

>359_Sample_ASV18

CACCGCGGTTATACGAAAGACCCCAGTTTATAGATGCGGCGTAAAGGGTGGTTAAGAGAA

AATCAAAATAAGGCCAAAGATCCCCTAAGCAGTCATACGCTCCACGAGGACACGAAACCC

AAATACGAAAGTTGCCTTAATAAACTTCACCTGATCCCACGAAAGCTGAAAAA

>359_Sample_ASV19

CACCGCGGTTATACGAGAGGCCCAAGTTGACAAACCCCGGCGTAAAGAGTGGTTAAGGAA

AATTTAAAACTAAAGCCGAATGCCCCCAAAGCTGTTATACGCACCCGAGGGTAAGAAGCC

CAATCACGAAAGTGGCTTTACACTACCTGAACCCACGAAAGCTATGAAA

>359_Sample_ASV20

CACCGCGGTTATACGAGAGGCCCAAGTTGACAAACCCCGGCGTAAAGAGTGGTTAAGGAA

AATTTAAAACTAAAGCCGAATGCCCCCAAAGCTGTTATACGCACCCGAGGATAAGAAGCC

CAATCACGAAAGTGGCTTTACACTACCTGAACCCACGAAAGCTATGAAA

>359_Sample_ASV21

CACCGCGGTCATACGATTAACCCAAGTTAATAGGCACACGGCGTAAAGCGTGTTTAAGCA

CTACGCCAAATAAAGTTAAACTCCAATTAAGCTGTAAAAAGCCATAATCAAGACGAAAAT

AAATAACGAAAGTAACTTTACAACCGCTGAAACACGATAGCTAAGATC

>359_Sample_ASV22

CACCGCGGTTATACGAGAGGCCCTAGTTGATAACTACCGGCGTAAAGAGTGGTTATGGAA

AAATATTTAATAAAGCCGAACACCCCCTCAGCCGTCATACGCACCTGGGAGCACGAAGAC

CTACTGCGAAAGCAGCTTTAATTATGCCTGACCCCACGACAGCTAAGAAA

>359_Sample_ASV23

CACCGCGGTTATACGAAAGACCCTAGTTTATAGACACGGCGTAAAGGGTGGTTCGGGACT

AAACAAAATAAAGCCAAAGACCTCCTAAGCCGTCATACGCTCACGGAAGCACGAAGCCCA

AGTACGAAAGTAGCTTTACAAAACATGCCTGACTCCACGAAAGCTAGGAAA

>359_Sample_ASV24

CACCGCGGTTATACGAGAGGCCCAAGTTGATACTCCACGGCGTAAAGAGTGGTTAGAAAA

GACCTACTACTAAAGCCGAACGCCTTCAAAGCTGTTATACGCATCCGAAGGTGAGAAGCC

CACCCACGAAAGTGGCTTTACAACCTTGACTCCACGAAAGCTATGACA

>359_Sample_ASV25

CACCGCGGTTATACGAGAGACCCCAGTTGATAAACACGGCGTAAAGAGTGGTTAAGGGCA

CAAAACAAATAAAGTCAAACAACCTCTTAGCTGTTATACGCATTATGAGGATATGAAGCC

CCCCTACGAAAGTAACTTTACCAACCCCTGACCCCACGAAAGCTAAGAAA

>359_Sample_ASV26

CACCGCGGTTATACGAAAGACCCTAGTTTATAGGCACGGCGTAAAGGGTGGTTAGGGGAT

AAACAAAATAAAGCCAAAGACCTTCTAAGTCGTCATACACTCATGAAGGCACGAAGCCCA

AATACGAAAGTAACTTTACAACTTAGACCCGACTCCACGAAAGCTGGGGGA

>366_Sample_ASV1

TACCGCGGTTATACGAAAGACCCAAGTTAATAAACTACGGCGTAAAGCGTGGTTAGAGAC

CTCCAATAATTAAAGCCAAAGACCTTCTAAGCCGTCGCACGCCCCGCGAAGACACGAAGC

CCAAAAACGAAAGTAGCTTTATTCTTACTCGACCCCACGAAAGCTAGGGCA

>366_Sample_ASV2

CACCGCGGTTATACGAAAGACCCTAGTTGATGGGTGCGGCGTAAAGGGTGGTTAGGGACT

ACAAAAAATAGAGCTAAAGATCCTCTAGGTCGTCATACACATCACGAGGAAACGAAGCCC

AAATACGAAAGTAGCTCTAAATATAATCCTGACGCCACGAAAGCCAAGAAA

>366_Sample_ASV3

CACCGCGGTTATACGAGAGACCCTAGTTGATTAAAGCGGCGTAAAGAGTGGTTATGGAAC

TATTCTTTTAAAGCAGAAAACCTCTCAAACTGTTATACGCACCCAGAGGTCTGAATCCCT

CACACGAAAGTGACTTTATTTATGCCTACCAGAACCCACGAAAGCTGGGACA

>366_Sample_ASV4

CACCGCGGTTATACGAGAGACCCTAGTTGATTTAAGCGGCGTAAAGGGTGGTTATGGAAC

TATTCTTTTAAAGCAGAAAACCTCTCAAACTGTTATACGCACCCAGAGGTCTGAATCCCT

CACACGAAAGTGACTTTATTTATGCCTACCAGAACCCACGAAAGCTGGGACA

>366_Sample_ASV5

CACCGCGGTTATACGAAAGACCCTAGTTTATAGGTACGGCGTAAAGGGTGGTTAGGGGAC

AAACAAAATAAAGCCAAAGACCTCCTAAGCCGTCATACGCTCACGGAAGCACGAAGCCCA

AACACGAAAGTAGCTTTACCGAACATGCCCGACTCCACGAAAGCTGGGGAA

>366_Sample_ASV6

CACCGCGGTTATACGAAAGACCCTAGTTTATAGGTACGGCGTAAAGGGTGATTAGGGGAC

AAACAAAATAAAGCCAAAGACCTCCTAAGCCGTCATACGCTCACGGAAGCACGAAGCCCA

AACACGAAAGTAGCTTTACCGAACATGCCCGACTCCACGAAAGCTGGGGAA

>366_Sample_ASV7

CACCGCGGTCATACGATTAACCCAAACTAATAGGCCTACGGCGTAAAGCGTGTTCAAGAT

ACTTTAACACTAAAGTTAAAACTTAACTAAGCCGTAAAAAGCTACAGTTATCATAAAATA

AACCACGAAGGTGACTTTATAATAATCTGACTACACGATAGCTAAGACC

>366_Sample_ASV8

CACCGCGATTATACGAGAGGCCCAAGTTGACAGACATCGGCGTAAAGAGTGGTTAAGGAA

AAATTACTACTAAAGCCGAACACCTTCAGAACTGTCATACGTTCCCGAAGATAAGAAGCC

CCACCACGAAAGTGGCTTTATATTACCCGACCCCACGAAAGCTGTGAAA

>366_Sample_ASV9

CATGGCGATTATACGAGAGGCCCAAGTTGACAGACACCGGCGTAAAGAGTGGCTAGGGAA

AAATTACTACTAAAGCCGAACACCTTCAGAACTGTCATACGTTCCCGAAGATAAGAAGCC

CCACCACGAAAGTGGCTTTATATTACCCGACCCCACGAAAGCTGGGACA

>366_Sample_ASV10

CACCGCGGTTATACGAGAGGCCCAAGTTGACAGACATCGGCGTAAAGAGTGGTTAAGGAA

AAATTACTGCTAAAGCCGAACACCCTCAGAACTGTCATACGTTCCCGAAGATAAGAAGCC

CCACCACGAAAGTGGCTTTATATTACCCGACCCCACGAAAGCTGTGAAA

>366_Sample_ASV11

CACCGCGATTATACGAGAGGCCCAAGTTGACAGACATCGGCGTAAAGAGTGGTTAAGGAA

AAATTACTACTAAAGCCGAACACCTTCAGAACTGTCATACGTTCCCGAAGATAAGAAGCC

CCACCACGAAAGTGGCTTTATATTACCCGACCCCACGAAAGCTGGGACA

>366_Sample_ASV12

CATGGCGATTATACGAGAGGCCCAAGTTGACAGACACTGGCGTAAAGAGTGGCTAAGGAA

AAACTACTACTAAAGCCGAACACCTTCAGAACTGTCATACGTTCCCGAAGATAAGAAGCC

CCACCACGAAAGTGGCTTTATATTACCCGACCCCACGAAAGCTGGGACA

>366_Sample_ASV13

CATGGCGATTATACGAGAGGCCCAAGTTGACAGACACCGGCGTAAAGAGTGGCTAGGGAA

AAATTACTACTAAAGCCGAACACCTTCAGAACTGTCATACGTTCCCGAAGATAAGAAGCC

CCACCACGAAAGTGGCTTTATATTACCCGACCCCACGAAAGCTGTGAAA

>366_Sample_ASV14

CACCGCGGTTATACGAGAGGCCCAAGTTGACAGACACCGGCGTAAAGAGTGGCTAGGGAA

AAATTACTACTAAAGCCGAACACCTTCAGAACTGTCATACGTTCCCGAAGATAAGAAGCC

CCACCACGAAAGTGGCTTTATATTACCCGACCCCACGAAAGCTGTGAAA

>366_Sample_ASV15

CACCGCGGTTATACGAAAGACTCTAGTTGACAAACACGGCGTAAAGGGTGGTTAGGATAA

CAACAAAATAAAGCTAAAGACTTACCAAGCCGTCATACGCCCATGTAAGAGCGAAAACCA

CAAACGAAAGTAACTTTATTACACCATCCGAATCCACGAAAGCTAAGAAA

>366_Sample_ASV16

CACCGCGGTTATACGAAAGACCCTAGTTTATAGGCACGGCGTAAAGGGTGGTTAGGGAAT

ACAAAAATAAAGCTAAAAATCCTCTAAGCTGTCATACGCTTCCCGAAGACATGAAACCCA

ACCACGAAAGTAGCTTTAAACTCTCCTGACACCACGAAAGCCAAGGAA

>366_Sample_ASV17

CACCGCGGTTATACGAAAGACCCTAGTTGATAGATCACGGCGTAAAGGGTGGTTAGGGAA

TACAAAAATAAAGCTAAAAATCCTCTAAGCTGTCATACGCTTCCCGAAGACATGAAACCC

AACCACGAAAGTAGCTTTAAACTCTCCTGACACCACGAAAGCCAAGGAA

>366_Sample_ASV18

CACCGCGGTTATACGAGAGGCCCAAGTTGATAAGCCCGGCGTAAAGAGTGGTTATGGAGA

ATAAAACACTAAAGCTAAAGACCCCCTAGGCTGTTATACGCACCTGGCGGCTCGAACCAC

CTATACGAAAGTAGCTTTACCCCCCTTCCACCAGAATCCACGACAGCTGGGGCA

>366_Sample_ASV19

CACCGCGGTTATACGAGAGGCCCTAGTTGATAGCCACGGCGCAAAGAGTGGTTAAGGAGC

CCCACTAAATAAAGTCGAACACTTCCTAGGCCGTTATACGTTTTCTAGAAGCACGAAACC

CAATTACACGAAAGCAACTTTATACTAAACAACCTGACCCCACGAAAGCTAAGAAA

>366_Sample_ASV20

CACCGCGGTTATACGAGAGGCCCAAGTTGACAGACATCGGCGTAAAGAGTGGTTAAGGAA

ACCCCATTACTAAAGCCGAACGCCCTCAGAACTGTCATACGTTCCCGAAGGTAAGAAGCC

CCACTACGAAAGTGGCTTTAACCACACCCGACCCCACGAAAGCTGTGAAA

>366_Sample_ASV21

CACCGCGGTTATACGAGAGGCCCAAGTTGACAGACACCGGCGTAAAGAGTGGTTAAGGAA

ATTCATCAACTAAAGCCGAATGCCCTCAGGACTGTTATACGTTTGCGAAGGTAAGAAGCC

CTACTACGAAAGTGGCTTTATTCCACCTGATTCCACGAAAGCTGCGGAA

>366_Sample_ASV22

CACCGCGGTTATACGAGAGGCCCCAGTTGATATTAGCGGCGTAAAGGGTGGTTAAGGACT

TTAATAACTAAAGCCAAACACTTCCCCGGCTGTTGTACGTAGACCGGAAGCTTGAAGCCC

AGACACGAAAGTAGCTTTATTTCCCCTGACCCCACGAAAGCTGAGAAA

>366_Sample_ASV23

CACCGCGGTTATACGAGAGGCCCCAGTTGATATTAGCGGCGTAAAGGGTGGTTAAGGACT

TTAATAACTAAAGCTAAACACTTCCCCGGCTGTTGTACGTAGACCGGAAGCTTGAAGCCC

AGACACGAAAGTAGCTTTATTCCCCCTGACCCCACGAAAGCTGAGAAA

>366_Sample_ASV24

CACCGCGGTTATACGAGAGGCCCCAGTTGATATTAGCGGCGTAAAGGGTGGTTAAGGACT

TTAATAACTAAAGCTAAACACTTCCCCGGCTGTTGTACGTAGACCGGAAGCTTGAAGCCC

AGACACGAAAGTAGCTTTATTTCCCCTGACCCCACGAAAGCTGAGAAA

>366_Sample_ASV25

CACCGCGGTCACACGATTAACCCAAGTCAATAGAAGCCGGCGTAAAGAGTGTTTTAGATC

ACCCCCTCCCCAATAAAGCTAAAACTCACCTGAGTTGTAAAAAACTCCAGTTGACACAAA

ATAGACTACGAAAGTGGCTTTAACATATCTGAACACACAATAGCTAAGACC

>366_Sample_ASV26

CACCGCGGTTATACGAAAGACCCCAGTTTATAGACACGGCGTAAAGGGTGGTTATAGGAC

AAACTAACATAGAGCCAAAGACCCCCTAAGCCGTCATACGCTCACGAGGGCTCGAAGCCC

CTATACGAAAGTAGCTCTACCAAAAAATGCTCGACTCCACGAAAGCTGGGACA

>366_Sample_ASV27

CACCGCGGTTATACGAGAGACCCTAGTTGATAAATACGGCGTAAAGAGTGGTTATGGGAA

AAACAAATAAAGTCAAACAACCTCTTAGCTGTTATACGCATTATGAGAGCATGAAGCCCC

CTCACGAAAGTAACTTTAATACCTCCTGACCCCACGAAAGCTAAGAAA

>366_Sample_ASV28

CACCGCGGTTATACGAGAGACCCTAATTGATAGGCACGGCGTAAAGAGTGGTTTAGGGTA

ACCCATAAATAAAGCTAAAGACCTTCTAAGCCGTCACACGCACACCGAAGGCACGAGACC

CCAACACGAAAGTAGCTTTACTTCTACCCCTGACGCCACGAAAGCTAAGAAA

>366_Sample_ASV29

CACCGCGGTTATACGAAAGACCCTAGTTGATGGATCACGGCGTAAAGGGTGGTTAAGGGA

TATAAAAATAAAGCTAAAAATCCTCTTAGCTGTCATACGCTTTCCGAAGACATGAGGCCC

AATTACGAAAGTAGCTTTAAACCACCCTGACACCACGAAAGCCAAGAAA

>366_Sample_ASV30

CACCGCGGTTATACGAAAGACCCCAGTTTATAGATGCGGCGTAAAGGGTGGTTAAGAGAA

AATCAAAATAAGGCCAAAGATCCCCTAAGCAGTCATACGCTCCACGAGGACACGAAACCC

AAATACGAAAGTTGCCTTAATAAACTTCACCTGATCCCACGAAAGCTGAAAAA

>366_Sample_ASV31

CACCGCGGTTATACGAAAGGCTCTAGTTTATAAATACGGCGTAAAGGGTGGTTAAGGAAA

AATTAAAATAATGCTAAAGACCCCCTAAGCAGTTATACGCTTCACGGAGGCACGAAACCC

AAATACGAAAGTTGCCTTAATAAACTTTACCTGACCCCACGAAAGCTGAAAAA

>366_Sample_ASV32

CACCGCGGTTATACGAAAGGCTCTAGTTTATAAATACGGCGTAAAGGGTGGTTAAGGAAA

AATTAAAATAATGCTAAAGACCCCCTAAGCAGTTATACGCTTCACGGAGGCACGAAACCC

AAATACGAAAGTTGCCTTAATAAACTTTACCTGACCCCACGAAAGCTGAGAAA

>366_Sample_ASV33

CACCGCGGTTATACGAGAGACTCTAGTTGATACCAACGGCGTAAAGAGTGGTTTGGGATG

CCCCTTAAAATAAAGCCAAAGACCTCCCAAGCCGTCAAACGCACCCCGGAGGCACGAAGC

CCCAACGCGAAAGCAACTTTACCTCCCTCCCGACGCCACGAAAGCTAAGAAA

>366_Sample_ASV34

CACCGCGGTTATACGAGAGACCCTAGTTGATAGCTACGGCGTAAAGCGTGGTTAGGAGAA

CAAGATAAATAAAGTCAAACAATCTCTCGGCCGTTATACGTTATCTGAGAATATGAAGTC

CTACCACGAAAGTAACTTTAATTTTTCTGACCCCACGAAAACTGAGGAA

>366_Sample_ASV35

CACCGCGGTTATACGAGAGGCCCTAGTTGATAACTACCGGCGTAAAGAGTGGTTATGGAA

AAATATTTAATAAAGCCGAACACCCCCTCAGCCGTCATACGCACCTGGGAGCACGAAGAC

CTACTGCGAAAGCAGCTTTAATTATGCCTGACCCCACGACAGCTAAGAAA

>366_Sample_ASV36

CACCGCGGTTATACGAGAGGCCCAAGTTGATTATTACCGGCGTAAAGCGTGGTTAAGATT

TTTATAGGACTAAAGCCGAACCCCCTCAGAGCTGTTATACGCACCCGAAGGTTAGAAGTT

CAATCACGAAAGTGGCTTTACAACCCCGAACCCACGAAAGCTAAGATA

>366_Sample_ASV37

CACCGCGGTTATACGAGAGGCCCAAGTTGATACTCCACGGCGTAAAGAGTGGTTAGAAAA

GACCTACTACTAAAGCCGAACGCCTTCAAAGCTGTTATACGCATCCGAAGGTGAGAAGCC

CACCCACGAAAGTGGCTTTACAACCTTGACTCCACGAAAGCTATGACA

>366_Sample_ASV38

CACCGCGGTTATACGAGAGGCCCAAGTTGATACTCCACGGCGTAAAGAGTGGTTAGAAAA

GACCTACTACTAAAGCCGAACGCCTTCAAAGCTGTTATACGCATCCGAAGGTGAGAAGCC

CACCCACGAAAGTGGCTTTACAATGCCTGACCCCACGAAAGCTGTGAAA

>366_Sample_ASV39

CACCGCGGTTATACGAGAGACCCCAGTTGATAAACACGGCGTAAAGAGTGGTTAAGGGCA

CAAAACAAATAAAGTCAAACAACCTCTTAGCTGTTATACGCATTATGAGGATATGAAGCC

CCCCTACGAAAGTAACTTTACCAACCCCTGACCCCACGAAAGCTAAGAAA

>366_Sample_ASV40

CACCGCGGTTATACGAGAGACCCCAGTTGATAAACACGGCGTAAAGAGTGGTTAAGGGCA

CAAAACAAATAAAGTCAAACAACCTCTTAGCTGTTATACGCATTATGAGGATATGAAGCC

CCCCTACGAAAGTAACTTTACCAACCCCTGACCCCACGAAAGCTGTGAAA

>366_Sample_ASV41

CACCGCGGTTATACGAAAGACCCTAGTTTATAGGCACGGCGTAAAGGGTGGTTAGGGGAT

AAACAAAATAAAGCCAAAGACCTTCTAAGTCGTCATACACTCATGAAGGCACGAAGCCCA

AATACGAAAGTAACTTTACAACTTAGACCCGACTCCACGAAAGCTGGGGGA

>366_Sample_ASV42

CACCGCGGTTATACGAAAGACCCTAGTTGATAGCTACGGCGTAAAGGGTGGTTAAGGTAT

ATAACAAATAAAGCTAAAGAGCCTCCAAGCCGTCGTACGCATTCTGAGAGCTCGAAACCC

TAACACGAAAGTAGCTTTAAAAAATAACACCTGACCCCACGAAAGCTAAGAAA

>366_Sample_ASV43

CACCGCGGTCATACGATTAACCCAAATTAATAGATCCACGGCGTAAAGAGTGTTTAAGaa

aaaaaaTCACAATAGAGTTAAATTATAACTAAGCTGTAAAAAGCCCTAGTTAAAATAAAA

TAACCCACGAAAGTGACTCTAATAATCCTGACACACGATAGCTAGGACC

>366_Sample_ASV44

CACCGCGGTTATACGAAAGACCCTAGTTGATAGATCACGGCGTAAAGGGTGGTTAAGGAG

AACAAAAATAAAGCTAAAAATCTTCTAAGCTGTCATACGCTTTCCGAAGACATGAGACCC

AACCACGAAAGTAGCTTTAAACTTTCCTGACACCACGAAAGCCAAGAAA

>366_Sample_ASV45

CACCGCGGTTATACGAAAGACCCTAGTTGATAGATCACGGCGTAAAGGGTGGTTAAGGAG

AACAAAAATAAAGCTAAAAATCCTCTAAGCTGTCATACGCTTTCCGAAGACATGAGACCC

AACCACGAAAGTAACTTTAAACTTTCCTGACACCACGAAAGCCAAGAAA

>368_2_Sample_ASV1

CACCGCGGTTATACGAGAGGCCCAAGTTGACAGACATCGGCGTAAAGAGTGGTTAAGGAA

AACCTTCAACTAAAGTCGAACGCTCTCAAGACTGTCGTACGTTTCCGAGAGTATGAAGCC

CCACCACGAAAGTGACTTTACCTCACCTGACTCCACGAAAGCTGCGAAA

>368_2_Sample_ASV2

CACCACGGTTATACGAGAGGCCCAAGTTGAAAGACATCGGCGTAAAGAGTGGTTAAGGAA

AACCTTCTACTAAAGTCGAACGCTCTCAGGACTGTCGTACGTTTCCGAGAGTATGAAGCC

CCACCACGAAAGTGACTTTACCTCACCTGACTCCACGAAAGCTGCGAAA

>368_Sample_ASV1

CACCGCGGTTATACGAGAGGCCCAAGTTGACAGACATCGGCGTAAAGAGTGGTTAAGGAA

AACCTTCAACTAAAGTCGAACGCTCTCAAGACTGTCGTACGTTTCCGAGAGTATGAAGCC

CCACCACGAAAGTGACTTTACCTCACCTGACTCCACGAAAGCTGCGAAA

>368_3_Sample_ASV1

TACCGCGGTTATACGAAAGACCCAAGTTAATAAACTACGGCGTAAAGCGTGGTTAGAGAC

CTCCAATAATTAAAGCCAAAGACCTTCTAAGCCGTCGCACGCCCCGCGAAGACACGAAGC

CCAAAAACGAAAGTAGCTTTATTCTTACTCGACCCCACGAAAGCTAGGGCA

>368_3_Sample_ASV2

CACCGCGGTTATACGAAAGACCCTAGTTGATGGGTGCGGCGTAAAGGGTGGTTAGGGACT

ACAAAAAATAGAGCTAAAGATCCTCTAGGTCGTCATACACATCACGAGGAAACGAAGCCC

AAATACGAAAGTAGCTCTAAATATAATCCTGACGCCACGAAAGCCAAGAAA

>368_3_Sample_ASV3

CACCGCGGTTATACGAAAGACCCTAGTTGATGGGTGCGGCGTAAAGGGTGGTTAGGGACT

ACAAAAAATAGAGCTAAAGATCCTCTAGGTCGTCATACACATCACGAGAAAACGAAGCCC

AAATACGAAAGTAGCTCTAAATATAATCCTGACGCCACGAAAGCCAAGAAA

>368_Sample_ASV2

CACCGCGGTTATACGAAAGACCCTAGTTGATGGGTGCGGCGTAAAGGGTGGTTAGGGACT

ACAAAAAATAGAGCTAAAGATCCTCTAGGTCGTCATACACATCACGAGGAAACGAAGCCC

AAATACGAAAGTAGCTCTAAATATAATCCTGACGCCACGAAAGCCAAGAAA

>368_2_Sample_ASV3

CACCGCGGTTATACGAAAGACCCTAGTTGATGGGTGCGGCGTAAAGGGTGGTTAGGGACT

ACAAAAAATAGAGCTAAAGATCCTCTAGGTCGTCATACACATCACGAGGAAACGAAGCCC

AAATACGAAAGTAGCTCTAAATATAATCCTGACGCCACGAAAGCCAAGAAA

>368_Sample_ASV3

CACCGCGGTTATACGAAAGACCCTAGTTGATAACTACGGCATAAAGGGTGGTTAGGGAGT

TTAAAAAATAAAGCCAAAGCTCTTCTAAGCTGTCATACGCTCTCTGAAAGAACGAGGCCC

AAACACGAAAGTAGCTTTAAATAAATAACCTGATACCACGAAAGCCAAGAAA

>368_Sample_ASV4

CACCGCGGTTATACGAGAGACCCTAGTTGATTAAAGCGGCGTAAAGAGTGGTTATGGAAC

TATTCTTTTAAAGCAGAAAACCTCTCAAACTGTTATACGCACCCAGAGGTCTGAATCCCT

CACACGAAAGTGACTTTATTTATGCCTACCAGAACCCACGAAAGCTGGGACA

>368_Sample_ASV5

CACCGCGGTTATACGAGAGACCCTAGTTGATTAAAGCGGCGTAAAGAGTGGTTATGGAAC

TATTCTTTTAAAGCAGAAAACCTCTCAAACTGTTATACGCACCCAGAGGTCTGAATCCCT

CACACGAAAGTGACTTTATTTATGCCTACCAGAACCCACGAAAGCTGTGAAA

>368_2_Sample_ASV4

CACCGCGGTTATACGAGAGACCCTAGTTGATTAAAGCGGCGTAAAGAGTGGTTATGGAAC

TATTCTTTTAAAGCAGAAAACCTCTCAAACTGTTATACGCACCCAGAGGTCTGAATCCCT

CACACGAAAGTGACTTTATTTATGCCTACCAGAACCCACGAAAGCTGGGACA

>368_2_Sample_ASV5

CACCGCGGTTATACGAGAGACCCTAGTTGATTAAAGCGGCGTAAAGAGTGGTTATGGAAC

TATTCTTTTAAAGCAGAAAACCTCTCAAACTGTTATACGCACCCAGAGGTCTGAATCCCT

CACACGAAAGTGACTTTATTTATGCCTACCAGAACCCACGAAAGCTGTGAAA

>368_4_Sample_ASV1

CACCGCGGTTATACGAGAGACCCTAGTTGATTAAAGCGGCGTAAAGAGTGGTTATGGAAC

TATTCTTTTAAAGCAGAAAACCTCTCAAACTGTTATACGCACCCAGAGGTCTGAATCCCT

CACACGAAAGTGACTTTATTTATGCCTACCAGAACCCACGAAAGCTGTGAAA

>368_4_Sample_ASV2

CACCGCGGTTATACGAGAGACCCTAGTTGATTAAAGCGGCGTAAAGAGTGGTTATGGAAC

TATTCTTTTAAAGCAGAAAACCTCTCAAACTGTTATACGCACCCAGAGGTCTGAATCCCT

CACACGAAAGTGACTTTATTTATGCCTACCAGAACCCACGAAAGCTAAGAAA

>368_4_Sample_ASV3

CACCGCGGTTATACGAGAGACCCTAGTTGATTAAAGCGGCGTAAAGAGTGGTTATGGAAC

TATTCTTTTAAAGCAGAAAACCTCTCAAACTGTTATACGCACCCAGAGGTCTGAATCCCT

CACACGAAAGTGACTTTATTTATGCCTACCAGAACCCACGAAAGCTGGGACA

>368_3_Sample_ASV4

CACCGCGGTTATACGAGAGACCCTAGTTGATTAAAGCGGCGTAAAGAGTGGTTATGGAAC

TATTCTTTTAAAGCAGAAAACCTCTCAAACTGTTATACGCACCCAGAGGTCTGAATCCCT

CACACGAAAGTGACTTTATTTATGCCTACCAGAACCCACGAAAGCTGGGACA

>368_2_Sample_ASV6

CACCGCGGTTATACGAAAGACCCTAGTTTATAGGTACGGCGTAAAGGGTGGTTAGGGGAC

AAACAAAATAAAGCCAAAGACCTCCTAAGCCGTCATACGCTCACGGAAGCACGAAGCCCA

AACACGAAAGTAGCTTTACCGAACATGCCCGACTCCACGAAAGCTGTGAAA

>368_2_Sample_ASV7

CACCGCGGTTATACGAAAGACCCTAGTTTATAGGTACGGCGTAAAGGGTGGTTAGGGGAC

AAACAAAATAAAGCCAAAGACCTCCTAAGCCGTCATACGCTCACGGAAGCACGAAGCCCA

AACACGAAAGTAGCTTTACCGAACATGCCCGACTCCACGAAAGCTGGGGAA

>368_4_Sample_ASV4

CACCGCGGTTATACGAAAGACCCTAGTTTATAGGTACGGCGTAAAGGGTGGTTAGGGGAC

AAACAAAATAAAGCCAAAGACCTCCTAAGCCGTCATACGCTCACGGAAGCACGAAGCCCA

AACACGAAAGTAGCTTTACCGAACATGCCCGACTCCACGAAAGCTGGGGAA

>368_2_Sample_ASV8

CACCGCGGTTATACGAAAGACCCTAGTTTATAGGTACGGCGTAAAGGGTGGTTAGGGGAC

AAACAAAATAAAGCCAAAGACCTCCTAAGCCGTCATACGCTCACGGAAGCACGAAGCCCA

AACACGAAAGTAGCTTTACCGAACATGCCCGACCCCACGAAAGCTGTGAAA

>368_2_Sample_ASV9

CACCGCGGTTATACGAAAGACCCTAGTTTATAGGTACGGCGTAAAGGGTGGTTAGGGGAC

AAACAAAATAAAGCCAAAGACCTCCTAAGCCGTCATACGCTCACGGAAGCACGAAGCCCA

AACACGAAAGTAGCTTTACCGAACATGCCTGACCCCACGAAAGCTAAGAAA

>368_2_Sample_ASV10

CACCGCGGTTATACGAAAGACCCTAGTTTATAGGTACGGCGTAAAGGGTGGTTAGGGGAC

AAACAAAATAAAGCCAAAGACCTCCTAAGCCGTCATACGCTCACGGAAGCACGAAGCCCA

AACACGAAAGTAGCTTTACCGAACATGCCCGACTCCACGAAAGCTATGACA

>368_2_Sample_ASV11

CACCGCGGTTATACGAAAGACCCTAGTTTATAGGTACGGCGTAAAGGGTGGTTAGGGGAC

AAACAAAATAAAGCCAAAGACCTCCTAAGCCGTCATACGCTCACGGAAGCACGAAGCCCA

AACACGAAAGTAGCTTTACCGAACATGCCCGACTCCACGAAAGCTAAGACA

>368_Sample_ASV6

CACCGCGGTTATACGAAAGACCCTAGTTTATAGGTACGGCGTAAAGGGTGGTTAGGGGAC

AAACAAAATAAAGCCAAAGACCTCCTAAGCCGTCATACGCTCACGGAAGCACGAAGCCCA

AACACGAAAGTAGCTTTACCGAACATGCCCGACTCCACGAAAGCTGGGGAA

>368_2_Sample_ASV12

CACCGCGGTTATACGAAAGACCCTAGTTGATAGCCACGGCGTAAAGGGTGGTTAAGGAAC

CTAAAAAATAAAGCTAAAGACCCTCTAGGCCGTCATACGCATTACGAGGCAAACGAAACC

CCAACACGAAAGTAGCTTTAGACAAATATCCTGATGCCACGAAAGCCAAGAAA

>368_2_Sample_ASV13

CACCGCGGTCATACGATTAACCCAAACTAATAGGCCTACGGCGTAAAGCGTGTTCAAGAT

ACTTTTACACTAAAGTTAAAACTTAACTAAGCCGTAAAAAGCTACAGTTATCATAAAATA

AACCACGAAAGTGACTTTATAATAATCTGACTACACGATAGCTAAGACC

>368_2_Sample_ASV14

CACCGCGGTTATACGAGAGACCCTAGTTGATATACACGGCGTAAAGAGTGGTTTGGGGTA

TATAATAAATAAAGCCAAAGACCTTCTAGGCCGTTGAACGCACCACGAAGGAGCGAAGTC

CCAATACGAAAGTAGCTTTATAAAACCTGACGCCACGAAAGCTAAGAAA

>368_2_Sample_ASV15

CACCGCGGTTATACGAGAGGCCCAAGTTGACAGACACCGGCGTAAAGAGTGGCTAGGGAA

AAATTACTACTAAAGCCGAACACCTTCAGAACTGTCATACGTTCCCGAAGATAAGAAGCC

CCACCACGAAAGTGACTTTATATTACCCGACCCCACGAAAGCTGTGAAA

>368_3_Sample_ASV5

CACCGCGGTTATACGAGAGGCCCAAGTTGACAGACACCGGCGTAAAGAGTGGCTAGGGAA

AAATTACTACTAAAGCCGAACACCTTCAGAACTGTCATACGTTCCCGAAGATAAGAAGCC

CCACCACGAAAGTGGCTTTATATTACCCGACCCCACGAAAGCTGTGAAA

>368_3_Sample_ASV6

CACCGCGATTATACGAGAGGCCCAAGTTGACAGACATCGGCGTAAAGAGTGGTTAAGGAA

AAATTACTACTAAAGCCGAACACCTTCAGAACTGTCATACGTTCCCGAAGATAAGAAGCC

CCACCACGAAAGTGGCTTTATATTACCCGACCCCACGAAAGCTGTGAAA

>368_3_Sample_ASV7

CACCGCGGTTATACGAGAGGCCCAAGTTGACAGACACCGGCGTAAAGAGTGGCTAGGGAA

AAATTACTACTAAAGCCGAACACCTTCAGAACTGTCATACGTTCCCGAAGATAAGAAGCC

CCACCACGAAAGTGGCTTTACAATGCCTGACCCCACGAAAGCTGTGAAA

>368_3_Sample_ASV8

CATGGCGATTATACGAGAGGCCCAAGTTGACAGACACTGGCGTAAAGAGTGGCTAAGGAA

AAATTACTACTAAAGCCGAACACCTTCAGAACTGTCATACGTTCCCGAAGATAAGAAGCC

CCACCACGAAAGTGGCTTTATATTACCCGACCCCACGAAAGCTGTGAAA

>368_3_Sample_ASV9

CATGGCGATTATACGAGAGGCCCAAGTTGACAGACACTGGCGTAAAGAGTGGCTAGGGAA

AAATTACTACTAAAGCCGAACACCTTCAGAACTGTCATACGTTCCCGAAGATAAGAAGCC

CCACCACGAAAGTGGCTTTATATTACCCGACCCCACGAAAGCTGTGAAA

>368_3_Sample_ASV10

CATGGCGATTATACGAGAGGCCCAAGTTGACAGACACTGGCGTAAAGAGTGGCTAAGGAA

AAACTACTACTAAAGCCGAACACCTTCAGAACTGTCATACGTTCCCGAAGATAAGAAGCC

CCACCACGAAAGTGGCTTTATATTACCCGACCCCACGAAAGCTGTGAAA

>368_4_Sample_ASV5

CACCGCGGTTATACGAGAGGCCCAAGTTGACAGACACCGGCGTAAAGAGTGGCTAGGGAA

AAATTACTACTAAAGCCGAACACCTTCAGAACTGTCATACGTTCCCGAAGATAAGAAGCC

CCACCACGAAAGTGGCTTTATATTACCCGACCCCACGAAAGCTAAGAAA

>368_4_Sample_ASV6

CACCGCGGTTATACGAGAGGCCCAAGTTGACAGACACCGGCGTAAAGAGTGGCTAGGGAA

AAATTACTACTAAAGCCGAACACCTTCAGAACTGTCATACGTTCCCGAAGATAAGAAGCC

CCACCACGAAAGTGGCTTTATATTACCCGACCCCACGAAAGCTGTGAAA

>368_4_Sample_ASV7

CACCGCGATTATACGAGAGGCCCAAGTTGACAGACATCGGCGTAAAGAGTGGTTAAGGAA

AAATTACTACTAAAGCCGAACACCTTCAGAACTGTCATACGTTCCCGAAGATAAGAAGCC

CCACCACGAAAGTGGCTTTATATTACCCGACCCCACGAAAGCTGTGAAA

>368_Sample_ASV7

CACCGCGGTTATACGAGAGGCCCAAGTTGACAGACACCGGCGTAAAGAGTGGCTAGGGAA

AAATTACTACTAAAGCCGAACACCTTCAGAACTGTCATACGTTCCCGAAGATAAGAAGCC

CCACCACGAAAGTGGCTTTACAATGCCTGACCCCACGAAAGCTGTGAAA

>368_Sample_ASV8

CACCGCGGTTATACGAGAGGCCCAAGTTGACAGACACCGGCGTAAAGAGTGGCTAGGGAA

AAATTACTACTAAAGCCGAACACCTTCAGAACTGTCATACGTTCCCGAAGATAAGAAGCC

CCACCACGAAAGTGGCTTTATATTACCCGACCCCACGAAAGCTGTGAAA

>368_2_Sample_ASV16

CACCGCGGTTATACGAGAGGCCCAAGTTGACAGACACCGGCGTAAAGAGTGGCTAGGGAA

AAATTACTACTAAAGCCGAACACCTTCAGAACTGTCATACGTTCCCGAAGATAAGAAGCC

CCACCACGAAAGTGGCTTTATATTACCCGACCCCACGAAAGCTGTGAAA

>368_Sample_ASV9

CACCGCGATTATACGAGAGGCCCAAGTTGACAGACATCGGCGTAAAGAGTGGTTAAGGAA

AAATTACTACTAAAGCCGAACACCTTCAGAACTGTCATACGTTCCCGAAGATAAGAAGCC

CCACCACGAAAGTGGCTTTATATTACCCGACCCCACGAAAGCTGTGAAA

>368_4_Sample_ASV8

CACCGCGGTTATACGAGAGGCCCAAGTTGACAGACACCGGCGTAAAGAGTGGCTAAGGAA

AAATTTCTACTAAAGCCGAACACCTTCAGAACTGTCATACGTTCCCGAAGAAAAGAAGCC

CCACCACGAAAGTGGCTTTATATTACCCGACCCCACGAAAGCTGTGAAA

>368_2_Sample_ASV17

CACCGCGGTTATACGAGAGGCCCAAGTTGACAGACACCGGCGTAAAGAGTGGCTAAGGAA

AAATTTCTACTAAAGCCGAACACCTTCAGAACTGTCATACGTTCCCGAAGATAAGAAGCC

CCACCACGAAAGTGGCTTTATATTACCCGACACCACGAAAGCTGTGAAA

>368_2_Sample_ASV18

CACCGCGGTTATACGAGAGGCCCAAGTTGACAGACACCGGCGTAAAGAGTGGCTAAGGAA

AAATTTCTACTAAAGCCGAACACCTTCAGAACTGTCATACGTTCCCGAAGAAAAGAAGCC

CCACCACGAAAGTGGCTTTATATTACCCGACCCCACGAAAGCTGTGAAA

>368_3_Sample_ASV11

CACCGCGGTTATACGAAAGACTCTAGTTGACAAACACGGCGTAAAGGGTGGTTAGGATAA

CAACAAAATAAAGCTAAAGACTTACCAAGCCGTCATACGCCCATGTAAGAGCGAAAACCA

CAAACGAAAGTAACTTTATTACACCATCCGAATCCACGAAAGCTAAGAAA

>368_2_Sample_ASV19

CACCGCGGTTATACGAAAGACTCTAGTTGACAAACACGGCGTAAAGGGTGGTTAGGATAA

CAACAAAATAAAGCTAAAGACTTACCAAGCCGTCATACGCCCATGTAAGAGCGAAAACCA

CAAACGAAAGTAACTTTATTACACCATCCGAATCCACGAAAGCTAAGAAA

>368_Sample_ASV10

CACCGCGGTTATACGAAAGACCCTAGTTTATAGAAACGGCGTAAAGGGTGGTTAAGGGAC

AAACAAAATAAGGCCAAAGACTTCCTAAGCCGTCATACGCTCCACGAAAGCACGAAGCCC

AAACACGAAAGTAGCCTTAATAACTTCACCTGACCCCACGAAAGCTGGGAGA

>368_4_Sample_ASV9

CACCGCGGTTATACGAGAGACCCTAGTTGATATATACGGCGTAAAGAGTGGTTTGGGGTT

ATGCCTCCAAAATAAAGCCGAAGATCTCCCAAGCCGTCACACGCATCCCGGAGACACGAA

GCCCTAATACGAAGGTAGCTTTATTAGTACCCCGACGCCACGAAAGCTAAGAAA

>368_4_Sample_ASV10

CACCGCGGTTATACGAGAGACCCTAGTTGATATATACGGCGTAAAGAGTGGTTTGGGGTT

ATGCCTCCAAAATAAAGCCGAAGATCTCCCAAGCCGTCACACGCACCCCGGAGACACGAA

GCCCTAATACGAAGGTAGCTTTATTAATTCCCCGACGCCACGAAAGCTAAGAAA

>368_3_Sample_ASV12

CACCGCGGTTATACGAAAGACCCTAGTTGATAGATCACGGCGTAAAGGGTGGTTAGGGAA

TACAAAAATAAAGCTAAAAATCCTCTAAGCTGTCATACGCTTCCCGAAGACATGAAACCC

AACCACGAAAGTAGCTTTAAACTCTCCTGACACCACGAAAGCCAAGGAA

>368_3_Sample_ASV13

CACCGCGGTTATACGAGAGGCCCAAGTTGATAAGCCCGGCGTAAAGAGTGGTTATGGAGA

ATAAAACACTAAAGCTAAAGACCCCCTAGGCTGTTATACGCACCTGGCGGCTCGAACCAC

CTATACGAAAGTAGCTTTACCCCCCTTCCACCAGAATCCACGACAGCTGGGGCA

>368_2_Sample_ASV20

CACCGCGGTTATACGAGAGGCCCAAGTTGATAAGCCCGGCGTAAAGAGTGGTTATGGAGA

ATAAAACACTAAAGCTAAAGACCCCCTAGGCTGTTATACGCACCTGGCGGCTCGAACCAC

CTATACGAAAGTAGCTTTACCCCCCTTCCACCAGAATCCACGACAGCTGGGGCA

>368_Sample_ASV11

CACCGCGGTTATACGAGAGGCCCAAGTTGATAAGCCCGGCGTAAAGAGTGGTTATGGAGA

ATAAAACACTAAAGCTAAAGACCCCCTAGGCTGTTATACGCACCTGGCGGCTCGAACCAC

CTATACGAAAGTAGCTTTACCCCCCTTCCACCAGAATCCACGACAGCTGGGGCA

>368_4_Sample_ASV11

CACCGCGGTTATACGAGAGGCCCAAGTTGATAAGCCCGGCGTAAAGAGTGGTTATGGAGA

ATAAAACACTAAAGCTAAAGACCCCCTAGGCTGTTATACGCACCTGGCGGCTCGAACCAC

CTATACGAAAGTAGCTTTACCCCCCTTCCACCAGAATCCACGACAGCTGGGGCA

>368_4_Sample_ASV12

CACCGCGGTTATACGAGAGGCCCTAGTTGATAGCCACGGCGCAAAGAGTGGTTAAGGAGC

CCCACTAAATAAAGTCGAACACTTCCTAGGCCGTTATACGTTTTCTAGAAGCACGAAACC

CAATTACACGAAAGCAACTTTATACTAAACAACCTGACCCCACGAAAGCTAAGAAA

>368_3_Sample_ASV14

CACCGCGGTTATACGAAAGACCCTAGTTGATAGCCACGGCGCAAAGAGTGGTTAAGGAGC

CCCACTAAATAAAGTCGAACACTTCCTAGGCCGTTATACGTTTTCTAGAAGCACGAAACC

CAATTACACGAAAGCAACTTTATACTAAACAACCTGACCCCACGAAAGCTAAGAAA

>368_3_Sample_ASV15

CACCGCGGTTATACGAGAGGCCCTAGTTGATAGCCACGGCGCAAAGAGTGGTTAAGGAGC

CCCACTAAATAAAGTCGAACACTTCCTAGGCCGTTATACGTTTTCTAGAAGCACGAAACC

CAATTACACGAAAGCAACTTTATACTAAACAACCTGACCCCACGAAAGCTAAGAAA

>368_4_Sample_ASV13

CACCGCGGTTATACGAGAGGCCCAAGTTGACAGACATCGGCGTAAAGAGTGGTTAAGGAA

ACCCCATTACTAAAGCCGAACGCCCTCAGAACTGTCATACGTTCCCGAAGGTAAGAAGCC

CCACTACGAAAGTGGCTTTAACCACACCCGACCCCACGAAAGCTGTGAAA

>368_2_Sample_ASV21

CACCGCGGTTATACGAGAGGCCCAAGTTGACAGACATCGGCGTAAAGAGTGGTTAAGGAA

ACCCCATTACTAAAGCCGAACGCCCTCAGAACTGTCATACGTTCCCGAAGGTAAGAAGCC

CCACTACGAAAGTGGCTTTAACCACACCCGACCCCACGAAAGCTGTGAAA

>368_Sample_ASV12

CACCGCGGTTATACGAGAGGCCCAAGTTGACAGACATCGGCGTAAAGAGTGGTTAAGGAA

ACCCCATTACTAAAGCCGAACGCCCTCAGAACTGTCATACGTTCCCGAAGGTAAGAAGCC

CCACTACGAAAGTGGCTTTAACCACACCCGACCCCACGAAAGCTGTGAAA

>368_3_Sample_ASV16

CACCGCGGTTATACGAGAGGCCCAAGTTGACAGACATCGGCGTAAAGAGTGGTTAAGGAA

ACCCCATTACTAAAGCCGAACGCCCTCAGAACTGTCATACGTTCCCGAAGGTAAGAAGCC

CCACTACGAAAGTGGCTTTAACCACACCCGACCCCACGAAAGCTGTGAAA

>368_Sample_ASV13

CACCGCGGTTATACGAGAGGCCCAAGTTGATAGATATCGGCGTAAAGAGTGGTTAAGGAA

ACCCCGTTACTAAAGCCGAACGCCCTCAGAACTGTCATACGTTCCCGAAGGTAAGAAGCC

CCACTACGAAAGTGGCTTTAACCACACCCGACCCCACGAAAGCTGTGAAA

>368_Sample_ASV14

CACCGCGGTTATACGAGAGGCCCAAGTTGACAGACATCGGCGTAAAGAGTGGTTAAGGAA

ACCCCATTACTAAAGCCGAACGCCCTCAGAACTGTCATACGTTCCCGAAGGTAAGAAGCC

CCACTACGAAAGTGGCTTTACTCCACCTGACTCCACGAAAGCTGTGAAA

>368_2_Sample_ASV22

CACCGCGGTTATACGAGAGGCCCAAGTTGATAGATATCGGCGTAAAGAGTGGTTAAGGAA

ACCCCGTTACTAAAGCCGAACGCCCTCAGAACTGTCATACGTTCCCGAAGGTAAGAAGCC

CCACTACGAAAGTGGCTTTAACCACACCCGACCCCACGAAAGCTGTGAAA

>368_2_Sample_ASV23

CACCGCGGTTATACGAGAGGCCCAAGTTGACAGACATCGGCGTAAAGAGTGGTTAAGGAA

ACCCCATTACTAAAGCCGAACGCCCTCAGAACTGTCATACGTTCCCGAAGGTAAGAAGCC

CCACTACGAAAGTGGCTTTATATTACCCGACCCCACGAAAGCTGTGAAA

>368_4_Sample_ASV14

CACCGCGGTTAAACGAGAGGCCCTAGTTGATATTACAACGGCGTAAAGGGTGGTTAAGGA

AAGCAAGCAATAAAGCCGAATGGCCCTTTGGCTGTCATACGCTTCTAGGTGTCCGAAGCC

CAATATACGAAAGTAGCTTTAGAAAAGCCCACCTGACACCACGAAAGCTGAGGAA

>368_3_Sample_ASV17

CACCGCGGTTAAACGAGAGGCCCTAGTTGATATTACAACGGCGTAAAGGGTGGTTAAGGA

AAGCAAGCAATAAAGCCGAATGGCCCTTTGGCTGTCATACGCTTCTAGGTGTCCGAAGCC

CAATATACGAAAGTAGCTTTAGAAAAGCCCACCTGACACCACGAAAGCTGAGGAA

>368_Sample_ASV15

CACCGCGGTTATACGAGAGGCCCAAGTTGACAGACACCGGCGTAAAGAGTGGTTAAGGAA

ATTCATCAACTAAAGCCGAATGCCCTCAGGACTGTTATACGTTTGCGAAGGTAAGAAGCC

CTACTACGAAAGTGGCTTTATTCCACCTGATTCCACGAAAGCTGCGGAA

>368_4_Sample_ASV15

CACCGCGGTTATACGAGAGGCCCAAGTTGACAGACACCGGCGTAAAGAGTGGTTAAGGAA

ATTCATCAACTAAAGCCGAATGCCCTCAGGACTGTTATACGTTTGCGAAGGTAAGAAGCC

CTACTACGAAAGTGGCTTTATTCCACCTGATTCCACGAAAGCTGCGGAA

>368_Sample_ASV16

CACCGCGGTTATACGAGAGGCCCCAGTTGATATTAGCGGCGTAAAGGGTGGTTAAGGACT

TTAATAACTAAAGCTAAACACTTCCCCGGCTGTTGTACGTAGACCGGAAGCTTGAAGCCC

AGACACGAAAGTAGCTTTATTCCCCCTGACCCCACGAAAGCTGAGAAA

>368_3_Sample_ASV18

CACCGCGGTTATACGAGAGGCCCCAGTTGATATTAGCGGCGTAAAGGGTGGTTAAGGACT

TTAATAACTAAAGCTAAACACTTCCCCGGCTGTTGTACGTAGACCGGAAGCTTGAAGCCC

AGACACGAAAGTAGCTTTATTCCCCCTGACCCCACGAAAGCTGAGAAA

>368_3_Sample_ASV19

CACCGCGGTTATACGAGAGGCCCCAGTTGATATTAGCGGCGTAAAGGGTGGTTAAGGACT

TTAATAACTAAAGCCAAACACTTCCCCGGCTGTTGTACGTAGACCGGAAGCTTGAAGCCC

AGACACGAAAGTAGCTTTATTTCCCCTGACCCCACGAAAGCTGAGAAA

>368_3_Sample_ASV20

CACCGCGGTTATACGAGAGGCCCCAGTTGATATTAGCGGCGTAAAGGGTGGTTAAGGACT

TTAATAACTAAAGCTAAACACTTCCCCGGCTGTTGTACGTAGACCGGAAGCTTGAAGCCC

AGACACGAAAGTAGCTTTATTTCCCCTGACCCCACGAAAGCTGAGAAA

>368_2_Sample_ASV24

CACCGCGGTCACACGATTAACCCAAGTCAATAGAAGCCGGCGTAAAGAGTGTTTTAGATC

ACCCCCTCCCCAATAAAGCTAAAACTCACCTGAGTTGTAAAAAACTCCAGTTGACACAAA

ATAGACTACGAAAGTGGCTTTAACATATCTGAACACACAATAGCTAAGACC

>368_2_Sample_ASV25

CACCGCGGTTATACGAGAAACCCTAGTTGACAAATACGGCGTAAAGAGTGGTTAAGGGGT

ATCAAAAATAAAGTCAAACATTTTCCTAGTTGTTATAAGCATTTCGAGAACATGAAGCCC

AACCACGAAAGTAACTTTACCTTCACCTGACCCCACGAAAGCTAAGAAA

>368_4_Sample_ASV16

CACCGCGGTTATACGAGAAACCCTAGTTGACAAATACGGCGTAAAGAGTGGTTAAGGGGT

ATCAAAAATAAAGTCAAACATTTTCCTAGTTGTTATAAGCATTTCGAGAACATGAAGCCC

AACCACGAAAGTAACTTTACCTTCACCTGACCCCACGAAAGCTAAGAAA

>368_Sample_ASV17

CACCGCGGTTATACGAGAAACCCTAGTTGACAAATACGGCGTAAAGAGTGGTTAAGGGGT

ATCAAAAATAAAGTCAAACATTTTCCTAGTTGTTATAAGCATTTCGAGAACATGAAGCCC

AACCACGAAAGTAACTTTACCTTCACCTGACCCCACGAAAGCTAAGAAA

>368_4_Sample_ASV17

CACCGCGGTTATACGAAAGACCCCAGTTTATAGACACGGCGTAAAGGGTGGTTATAGGAC

AAACTAACATAGAGCCAAAGACCCCCTAAGCCGTCATACGCTCACGAGGGCTCGAAGCCC

CTATACGAAAGTAGCTCTACCAAAAAATGCTCGACTCCACGAAAGCTGGGACA

>368_3_Sample_ASV21

CACCGCGGTTATACGAAAGACCCCAGTTTATAGACACGGCGTAAAGGGTGGTTATAGGAC

AAACTAACATAGAGCCAAAGACCCCCTAAGCCGTCATACGCTCACGAGGGCTCGAAGCCC

CTATACGAAAGTAGCTCTACCAAAAAATGCTCGACTCCACGAAAGCTGGGACA

>368_3_Sample_ASV22

CACCGCGGTTATACGAAAGACCCTAGTTGATAGCTACGGCGTAAAGGGTGGTTAGGGGAC

AAACAAAATAAAGCCAAAGACCTCCTAAGCCGTCATACGCTTACGGAGGCACGAAGCCCA

AACACGAAAGTAGCTTTACCGAACATGCCCGACTCCACGAAAGCTGGGAAA

>368_3_Sample_ASV23

CACCGCGGTTATACGAAAGACCCTAGTTTATAAGTACGGCGTAAAGGGTGGTTAGGGGAC

AAACAAAATAAAGCCAAAGACCTCCTAAGCCGTCATACGCTTACGGAGGCACGAAGCCCA

AACACGAAAGTAGCTTTACCGAACATGCCCGACTCCACGAAAGCTGGGAAA

>368_3_Sample_ASV24

CACCGCGGTTATACGAAAGACCCTAGTTTATAAGTACGGCGTAAAGGGTGGTTAGGGGAC

AAACAAAATAAAGCCAAAGACCTCCTAAGCCGTCATACGCTTACGGAGGCACGAAGCCCA

AACACGAAAGTAGCTTTACCGAACATGCCCGACTCCACGAAAGCTAAGAAA

>368_3_Sample_ASV25

CACCGCGGTTATACGAAAGACCCTAGTTGATAGCTACGGCGTAAAGGGTGGTTAGGGGAC

AAACAAAATAAAGCCAAAGACCTCCTAAGCCGTCATACGCTTACGGAGGCACGAAGCCCA

AACACGAAAGTAGCTTTACCGAACATGCCCGACTCCACGAAAGCTAAGAAA

>368_3_Sample_ASV26

CACCGCGGTTATACGAAAGACCCTAGTTTATAAGTACGGCGTAAAGGGTGGTTAGGGGAC

AAACAAAATAAAGCCAAAGACCTCCTAAGCCGTCATACGCTTACGGAGGCACGAAGCCCA

AACACGAAAGTAGCTTTACCGAACATGCCCGACCCCACGAAAGCTAAGAAA

>368_4_Sample_ASV18

CACCGCGGTTATACGAGAGGCCCCAGTTGATAAACACGGCGTAAAGGGTGGTTAAGGGAG

CATAAGAATAAAGCCGAAGGGCCCTTTGGCCGTTATACGCTTCTAGGTGTCCGAAGCCCA

AACACGAAAGTAGCTTTAGTTAAGCCCACCTGACTCCACGAAAACTGAGAAA

>368_Sample_ASV18

CACCGCGGTTATACGAGAGACCCTAGTTGATAAATACGGCGTAAAGAGTGGTTATGGGAA

AAACAAATAAAGTCAAACAACCTCTTAGCTGTTATACGCATTATGAGAGCATGAAGCCCC

CTCACGAAAGTAACTTTAATACCTCCTGACCCCACGAAAGCTAAGAAA

>368_Sample_ASV19

CACCGCGGTTATACGAGAGACCCTAGTTGATATATACGGCGTAAAGAGTGGTTATGGGAA

AAACAAATAAAGTCAAACAACCTCTTAGCTGTTATACGCATTATGAGAGCATGAAGCCCC

CTCACGAAAGTAACTTTAATACCTCCTGACCCCACGAAAGCTAAGAAA

>368_2_Sample_ASV26

CACCGCGGTTATACGAGAGACCCTAGTTGATAAATACGGCGTAAAGAGTGGTTATGGGAA

AAACAAATAAAGTCAAACAACCTCTTAGCTGTTATACGCATTATGAGAGCATGAAGCCCC

CTCACGAAAGTAACTTTAATACCTCCTGACCCCACGAAAGCTAAGAAA

>368_4_Sample_ASV19

CACCGCGGTTATACGAGAGACCCTAGTTGATAAATACGGCGTAAAGAGTGGTTATGGGAA

AAACAAATAAAGTCAAACAACCTCTTAGCTGTTATACGCATTATGAGAGCATGAAGCCCC

CTCACGAAAGTAACTTTAATACCTCCTGACCCCACGAAAGCTAAGAAA

>368_3_Sample_ASV27

CACCGCGGTTATACGAGAGACCCTAGTTGATAAATACGGCGTAAAGAGTGGTTATGGGAA

AAACAAATAAAGTCAAACAACCTCTTAGCTGTTATACGCATTATGAGAGCATGAAGCCCC

CTCACGAAAGTAACTTTAATACCTCCTGACCCCACGAAAGCTAAGAAA

>368_4_Sample_ASV20

CACCGCGGTTATACGAGAGGCTCAAGTTGATAAACCCCGGCGTAAAGAGTGGTTAAGGAA

AAATAGAAACTAAAGCCGAATGCTTTCAAGACTGTTATACGTTCCCGAGAGTAAGAAGAC

CAATTACGAAAGTGGCTTTACTTTACCTGACCCCACGAAAGCTACGACA

>368_4_Sample_ASV21

CACCGCGGTTATACGAGAGGCTCAAGTTGATGAACCCCGGCGTAAAGAGTGGTTAAGGGA

GATCAAAACTAAAGCCGAATGCTTTCAAAGCTGTTATACGCTTCCGAAAGTAAGAAGCTC

AATCACGAAAGTGGCTTTACTTTACCTGACCCCACGAAAGCTACGACA

>368_2_Sample_ASV27

AACCGCGGTTATACGAGAGACCCTAATTGATAGCTCACGGCGTAAAGGGTGGTTTGGGGA

AACCTCAAAATAAAGCTAAAGACCTTCTAAGCCGTTACACGCATACCGAAGGCACGAGGC

CCTAACACGAAAGTAGCTTTACTATTACCCCTGACGCCACGAAAGCTAAGAAA

>368_Sample_ASV20

CACCGCGGTTATACGAGAGACCCTAATTGATAGGTACGGCGTAAAGAGTGGTTTGGGATA

ACCCAAAAATAAAGCTAAAGACCTTCTAAGCCGTCACACGCATACCGAAGGCACGAGGCC

CCAACACGAAAGTAGCTTTACTATTACCCCGACGCCACGAAAGCTAAGAAA

>368_3_Sample_ASV28

CACCGCGGTTATACGAGAGACCCTAATTGATAGGCACGGCGTAAAGAGTGGTTTAGGGTA

ACCCATAAATAAAGCTAAAGACCTTCTAAGCCGTCACACGCACACCGAAGGCACGAGACC

CCAACACGAAAGTAGCTTTACTTCTACCCCTGACGCCACGAAAGCTAAGAAA

>368_4_Sample_ASV22

CACCGCGGTTATACGAAAGACCCTAGTTGATGGATCACGGCGTAAAGGGTGGTTAAGGGA

TATAAAAATAAAGCTAAAAATCCTCTTAGCTGTCATACGCTTTCCGAAGACATGAGGCCC

AATTACGAAAGTAGCTTTAAACCACCCTGACACCACGAAAGCCAAGAAA

>368_3_Sample_ASV29

CACCGCGGTTATACGAAAGACCCTAGTTGATGGATCACGGCGTAAAGGGTGGTTAAGGGA

TATAAAAATAAAGCTAAAAATCCTCTTAGCTGTCATACGCTTTCCGAAGACATGAGGCCC

AATTACGAAAGTAGCTTTAAACCACCCTGACACCACGAAAGCCAAGAAA

>368_2_Sample_ASV28

CACCGCGGTTATACGAAAGACCCTAGTTGATGGATCACGGCGTAAAGGGTGGTTAAGGGA

TATAAAAATAAAGCTAAAAATCCTCTTAGCTGTCATACGCTTTCCGAAGACATGAGGCCC

AATTACGAAAGTAGCTTTAAACCACCCTGACACCACGAAAGCCAAGAAA

>368_3_Sample_ASV30

CACCGCGGTTATACGAAAGACCCCAGTTTATAGATGCGGCGTAAAGGGTGGTTAAGAGAA

AATCAAAATAAGGCCAAAGATCCCCTAAGCAGTCATACGCTCCACGAGGACACGAAACCC

AAATACGAAAGTTGCCTTAATAAACTTCACCTGATCCCACGAAAGCTGAAAAA

>368_4_Sample_ASV23

CACCGCGGTTATACGAAAGACCCCAGTTTATAGATGCGGCGTAAAGGGTGGTTAAGAGAA

AATCAAAATAAGGCCAAAGATCCCCTAAGCAGTCATACGCTCCACGAGGACACGAAACCC

AAATACGAAAGTTGCCTTAATAAACTTCACCTGATCCCACGAAAGCTGAAAAA

>368_3_Sample_ASV31

CACCGCGGTTATACGAAAGGCTCTAGTTTATAAATACGGCGTAAAGGGTGGTTAAGGAAA

AATTAAAATAATGCTAAAGACCCCCTAAGCAGTTATACGCTTCACGGAGGCACGAAACCC

AAATACGAAAGTTGCCTTAATAAACTTTACCTGACCCCACGAAAGCTGAAAAA

>368_2_Sample_ASV29

CACCGCGGTTATACGAGAGGCCCAAGTTGACAGGTACCGGCGTAAAGAGTGGTTAAGAAA

AAATATTAAACTAAAGCCGAACACCTTCAGAGCTGTCATACGTTTCCGAAGGCATGAAGC

CCCACCACGAAAGTGGCTTTATTTTGCCCGACTCCACGAAAGCTGTGAAA

>368_3_Sample_ASV32

CACCGCGGTTATACGAGAGACTCTAGTTGATACCAACGGCGTAAAGAGTGGTTTGGGATG

CCCCTTAAAATAAAGCCAAAGACCTCCCAAGCCGTCAAACGCACCCCGGAGGCACGAAGC

CCCAACGCGAAAGCAACTTTACCTCCCTCCCGACGCCACGAAAGCTAAGAAA

>368_4_Sample_ASV24

CACCGCGGTTATACGAGAGGCCCAAGTTGACAAACCCCGGCGTAAAGAGTGGTTAAGGAA

AATTTAAAACTAAAGCCGAATGCCCCCAAAGCTGTTATACGCACCCGAGGATAAGAAGCC

CAATCACGAAAGTGGCTTTACACTACCTGAACCCACGAAAGCTATGAAA

>368_4_Sample_ASV25

CACCGCGGTTATACGAGAGGCCCAAGTTGACAAACCCCGGCGTAAAGAGTGGTTAAGGAA

AATTTAAAACTAAAGCCGAATGCCCCCAAAGCTGTTATACGCACCCGAGGGTAAGAAGCC

CAATCACGAAAGTGGCTTTACACTACCTGAACCCACGAAAGCTATGAAA

>368_3_Sample_ASV33

CACCGCGGTTATACGAGAGACCCTAGTTGATAGCTACGGCGTAAAGCGTGGTTAGGAGAA

CAAGATAAATAAAGTCAAACAATCTCTCGGCCGTTATACGTTATCTGAGAATATGAAGTC

CTACCACGAAAGTAACTTTAATTTTTCTGACCCCACGAAAACTGAGGAA

>368_Sample_ASV21

CACCGCGGTTATACGAGAGGCCCTAGTTGATAACTACCGGCGTAAAGAGTGGTTATGGAA

AAATATTTAATAAAGCCGAACACCCCCTCAGCCGTCATACGCACCTGGGAGCACGAAGAC

CTACTGCGAAAGCAGCTTTAATTATGCCTGACCCCACGACAGCTAAGAAA

>368_3_Sample_ASV34

CACCGCGGTTATACGAGAGGCCCTAGTTGATAACTACCGGCGTAAAGAGTGGTTATGGAA

AAATATTTAATAAAGCCGAACACCCCCTCAGCCGTCATACGCACCTGGGAGCACGAAGAC

CTACTGCGAAAGCAGCTTTAATTATGCCTGACCCCACGACAGCTAAGAAA

>368_4_Sample_ASV26

CACCGCGGTTATACGAGAGGCCCTAGTTGATAACTACCGGCGTAAAGAGTGGTTATGGAA

AAATATTTAATAAAGCCGAACACCCCCTCAGCCGTCATACGCACCTGGGAGCACGAAGAC

CTACTGCGAAAGCAGCTTTAATTATGCCTGACCCCACGACAGCTAAGAAA

>368_4_Sample_ASV27

CACCGCGGTTATACGAAAGACCCTAGTTTATAGACACGGCGTAAAGGGTGGTTCGGGACT

AAACAAAATAAAGCCAAAGACCTCCTAAGCCGTCATACGCTCACGGAAGCACGAAGCCCA

AGTACGAAAGTAGCTTTACAAAACATGCCTGACTCCACGAAAGCTAGGAAA

>368_3_Sample_ASV35

CACCGCGGTTATACGAGAGGCCCAAGTTGATTATTACCGGCGTAAAGCGTGGTTAAGATT

TTTATAGGACTAAAGCCGAACCCCCTCAGAGCTGTTATACGCACCCGAAGGTTAGAAGTT

CAATCACGAAAGTGGCTTTACAACCCCGAACCCACGAAAGCTAAGATA

>368_4_Sample_ASV28

CACCGCGGTTATACGAGAGACCCTAATTGATAGGCGCGGCGTAAAGAGTGGTTTAGGACT

AAAACTAATAAAGCTAAAGATCCTCCCAGCCGTCGCACGCATTCTGAGGACACGAAACTC

AACCACGAAAGTAGCTTTAACATACTCTCCTGACCCCACGAAAGCTAAGAAA

>368_2_Sample_ASV30

CACCGCGGTTATACGAGAGGCCCAAGTTGATACTCCACGGCGTAAAGAGTGGTTAGAAAA

GACCTACTACTAAAGCCGAACGCCTTCAAAGCTGTTATACGCATCCGAAGGTGAGAAGCC

CACCCACGAAAGTGGCTTTACAACCTTGACTCCACGAAAGCTATGACA

>368_4_Sample_ASV29

CACCGCGGTTATACGAGAGGCCCAAGTTGATACTCCACGGCGTAAAGAGTGGTTAGAAAA

GACCTACTACTAAAGCCGAACGCCTTCAAAGCTGTTATACGCATCCGAAGGTGAGAAGCC

CACCCACGAAAGTGGCTTTACAACCTTGACTCCACGAAAGCTATGACA

>368_Sample_ASV22

CACCGCGGTTATACGAGAGGCCCAAGTTGATACTCCACGGCGTAAAGAGTGGTTAGAAAA

GACCTACTACTAAAGCCGAACGCCTTCAAAGCTGTTATACGCATCCGAAGGTGAGAAGCC

CACCCACGAAAGTGGCTTTACAACCTTGACTCCACGAAAGCTATGACA

>368_Sample_ASV23

CACCGCGGTTATACGAGAGGCCCAAGTTGATACTCCACGGCGTAAAGAGTGGTTAGAAAA

GACCTACTACTAAAGCCGAACGCCTTCAAAGCTGTTATACGCATCCGAAGGTGAGAAGCC

CACCCACGAAAGTGGCTTTACAACCTTGACTCCACGAAAGCTGTGAAA

>368_3_Sample_ASV36

CACCGCGGTTATACGAGAGGCCCAAGTTGATACTCCACGGCGTAAAGAGTGGTTAGAAAA

GACCTACTACTAAAGCCGAACGCCTTCAAAGCTGTTATACGCATCCGAAGGTGAGAAGCC

CACCCACGAAAGTGGCTTTACAACCTTGACTCCACGAAAGCTATGACA

>368_4_Sample_ASV30

CACCGCGGTTATACGAGAGACCCCAGTTGATAAACACGGCGTAAAGAGTGGTTAAGGGCA

CAAAACAAATAAAGTCAAACAACCTCTTAGCTGTTATACGCATTATGAGGATATGAAGCC

CCCCTACGAAAGTAACTTTACCAACCCCTGACCCCACGAAAGCTAAGAAA

>368_3_Sample_ASV37

CACCGCGGTTATACGAGAGACCCCAGTTGATAAACACGGCGTAAAGAGTGGTTAAGGGCA

CAAAACAAATAAAGTCAAACAACCTCTTAGCTGTTATACGCATTATGAGGATATGAAGCC

CCCCTACGAAAGTAACTTTACCAACCCCTGACCCCACGAAAGCTGTGAAA

>368_3_Sample_ASV38

CACCGCGGTTATACGAGAGACCCCAGTTGATAAACACGGCGTAAAGAGTGGTTAAGGGCA

CAAAACAAATAAAGTCAAACAACCTCTTAGCTGTTATACGCATTATGAGGATATGAAGCC

CCCCTACGAAAGTAACTTTACCAACCCCTGACCCCACGAAAGCTAAGAAA

>368_2_Sample_ASV31

CACCGCGGTTATACGAGAGACCCCAGTTGATAAACACGGCGTAAAGAGTGGTTAAGGGCA

CAAAACAAATAAAGTCAAACAACCTCTTAGCTGTTATACGCATTATGAGGATATGAAGCC

CCCCTACGAAAGTAACTTTACCAACCCCTGACCCCACGAAAGCTAAGAAA

>368_Sample_ASV24

CACCGCGGTTATACGAGAGACCCCAGTTGATAAACACGGCGTAAAGAGTGGTTAAGGGCA

CAAAACAAATAAAGTCAAACAACCTCTTAGCTGTTATACGCATTATGAGGATATGAAGCC

CCCCTACGAAAGTAACTTTACCAACCCCTGACCCCACGAAAGCTAAGAAA

>368_2_Sample_ASV32

CACCGCGGTTATACGAAAGACCCTAGTTTATAGGCACGGCGTAAAGGGTGGTTAGGGGAT

AAACAAAATAAAGCCAAAGACCTTCTAAGTCGTCATACACTCATGAAGGCACGAAGCCCA

AATACGAAAGTAACTTTACAACTTAGACCCGACTCCACGAAAGCTGGGGGA

>368_3_Sample_ASV39

CACCGCGGTTATACGAAAGACCCTAGTTTATAGGCACGGCGTAAAGGGTGGTTAGGGGAT

AAACAAAATAAAGCCAAAGACCTTCTAAGTCGTCATACACTCATGAAGGCACGAAGCCCA

AATACGAAAGTAACTTTACAACTTAGACCCGACTCCACGAAAGCTGGGGGA

>368_4_Sample_ASV31

CACCGCGGTTATACGAAAGACCCTAGTTTATAGGCACGGCGTAAAGGGTGGTTAGGGGAT

AAACAAAATAAAGCCAAAGACCTTCTAAGTCGTCATACACTCATGAAGGCACGAAGCCCA

AATACGAAAGTAACTTTACAACTTAGACCCGACTCCACGAAAGCTGGGGGA

>368_2_Sample_ASV33

CACCGCGGTTATACGAGAGACCCTAATTAATAGATACGGCGTAAAGAGTGGTTTAGGACA

AAAATTAATAAAGCCGAAGTTTCTCCTAGCCGTTGTACGCACTCTGAGAACACGAAGCCC

CACTACGAAAGTAGCTTTACCTATTATTTCCTGACCCCACGAAAGCTAAGAAA

>368_3_Sample_ASV40

CACCGCGGTTATACGAGAGGCCCAAGTTGATAGACATCGGCGTAAAGAGTGGTTAAGGAA

GTCTCAAAACTAAAGCCGAATGCCTTCAAGACTGTTATACGTTTCCGAAGGTAAGAAGCC

CCACTACGAAAGTGGCTTTACTCCACCTGACTCCACGAAAGCTGTGAAA

>368_2_Sample_ASV34

CACCGCGGTTATACGAGAGGCCCAAGTTGATAGACATCGGCGTAAAGAGTGGTTAAGGAA

GTCTCAAAACTAAAGCCGAATGCCTTCAAGACTGTTATACGTTTCCGAAGGTAAGAAGCC

CCACTACGAAAGTGGCTTTACTCCACCTGACTCCACGAAAGCTAAGAAA

>368_2_Sample_ASV35

CACCGCGGTTATACGAGAGGCCCAAGTTGATAGACATCGGCGTAAAGAGTGGTTAAGGAA

GTCTCAAAACTAAAGCCGAATGCCTTCAAGACTGTTATACGTTTCCGAAGGTAAGAAGCC

CCACTACGAAAGTGGCTTTATTCCACCTGACCCCACGAAAGCTGTGAAA

>368_2_Sample_ASV36

CACCGCGGTTATACGAGAGGCCCAAGTTAATAGACATCGGCGTAAAGAGTGGTTAAGGAA

GTCTCAAAACTAAAGCCGAATGCCTTCAAGACTGTTATACGTTTCCGAAGGTAAGAAGCC

CCACTACGAAAGTGGCTTTACTCCACCTGACTCCACGAAAGCTGTGAAA

>368_2_Sample_ASV37

CACCGCGGTTATACGAGAGGCCCAAGTTGATAGACATCGGCGTAAAGAGTGGTTAAGGAA

GTCTCAAAACTAAAGCCGAATGCCTTCAAGACTGTTATACGTTTCCGAAGGTAAGAAGCC

CCACTACGAAAGTGGCTTTACTCCACCTGACTCCACGAAAGCTGTGAAA

>368_Sample_ASV25

CACCGCGGTTATACGAGAGGCCCAAGTTGACAGACATCGGCGTAAAGAGTGGTTAAGGAA

GTCTCAAAACTAAAGCCGAATGCCTTCAAGACTGTTATACGTTTCCGAAGGTAAGAAGCC

CCACTACGAAAGTGGCTTTACTCCACCTGACTCCACGAAAGCTGTGAAA

>368_Sample_ASV26

CACCGCGGTTATACGAGAGGCCCAAGTTGATAGACATCGGCGTAAAGAGTGGTTAAGGAA

GTCTCAAAACTAAAGCCGAATGCCTTCAAGACTGTTATACGTTTCCGAAGGTAAGAAGCC

CCACTACGAAAGTGGCTTTAACCACACCCGACCCCACGAAAGCTGTGAAA

>368_Sample_ASV27

CACCGCGGTTATACGAGAGGCCCAAGTTGATAGACATCGGCGTAAAGAGTGGTTAAGGAA

GTCTCAAAACTAAAGCCGAATGCCTTCAAGACTGTTATACGTTTCCGAAGGTAAGAAGCC

CCACTACGAAAGTGGCTTTACTCCACCTGACTCCACGAAAGCTGTGAAA

>368_Sample_ASV28

CACCGCGGTTATACGAGAGACTCCAGTTGATAGCTACGGCGTAAAGAGTGGTTTGGGGCC

CGCCCAAAATAAAGCCAAAGACCTCCCAAGCCGTCAAACGCACCCCGGAGGCACGAAGTC

CTAACGCGAAAGCAACTTTACCTTCCCCGACGCCACGAAAGCTAAGAAA

>368_3_Sample_ASV41

CACCGCGGTTATACGAAAGACCCTAGTTGATAGCTACGGCGTAAAGGGTGGTTAAGGTAT

ATAACAAATAAAGCTAAAGAGCCTCCAAGCCGTCGTACGCATTCTGAGAGCTCGAAACCC

TAACACGAAAGTAGCTTTAAAAAATAACACCTGACCCCACGAAAGCTAAGAAA

>368_Sample_ASV29

CACCGCGGTTATACGAAAGACCCTAGTTGATAGATCACGGCGTAAAGGGTGGTTAAGGAG

AACAAAAATAAAGCTAAAAATCCTCTAAGCTGTCATACGCTTTCCGAAGACATGAGACCC

AACCACGAAAGTAACTTTAAACTTTCCTGACACCACGAAAGCCAAGAAA

>368_3_Sample_ASV42

CACCGCGGTTATACGAAAGACCCTAGTTGATAGATCACGGCGTAAAGGGTGGTTAAGGAG

AACAAAAATAAAGCTAAAAATCTTCTAAGCTGTCATACGCTTTCCGAAGACATGAGACCC

AACCACGAAAGTAGCTTTAAACTTTCCTGACACCACGAAAGCCAAGAAA

>370_Sample_ASV1

CACCGCGGTTATACGAGAGGCCCAAGTTGACAGACATCGGCGTAAAGAGTGGTTAAGGAA

AACCTTCAACTAAAGTCGAACGCTCTCAAGACTGTCGTACGTTTCCGAGAGTATGAAGCC

CCACCACGAAAGTGACTTTACCTCACCTGACTCCACGAAAGCTGCGAAA

>370_Sample_ASV2

CACCGCGGTTATACGAAAGACCCTAGTTGATGGGTGCGGCGTAAAGGGTGGTTAGGGACT

ACAAAAAATAGAGCTAAAGATCCTCTAGGTCGTCATACACATCACGAGGAAACGAAGCCC

AAATACGAAAGTAGCTCTAAATATAATCCTGACGCCACGAAAGCCAAGAAA

>370_Sample_ASV3

CACCGCGGTTATACGAGAGACCCTAGTTGATTAAAGCGGCGTAAAGAGTGGTTATGGAAC

TATTCTTTTAAAGCAGAAAACCTCTCAAACTGTTATACGCACCCAGAGGTCTGAATCCCT

CACACGAAAGTGACTTTATTTATGCCTACCAGAACCCACGAAAGCTGGGACA

>370_Sample_ASV4

CACCGCGGTTATACGAAAGACCCTAGTTTATAGGTACGGCGTAAAGGGTGGTTAGGGGAC

AAACAAAATAAAGCCAAAGACCTCCTAAGCCGTCATACGCTCACGGAAGCACGAAGCCCA

AACACGAAAGTAGCTTTACCGAACATGCCCGACTCCACGAAAGCTGGGGAA

>370_Sample_ASV5

CATGGCGATTATACGAGAGGCCCAAGTTGACAGACACTGGCGTAAAGAGTGGCTAGGGAA

AAATTACTACTAAAGCCGAACACCTTCAGAACTGTCATACGTTCCCGAAGATAAGAAGCC

CCACCACGAAAGTGGCTTTATATTACCCGACCCCACGAAAGCTGTGAAA

>370_Sample_ASV6

CACCGCGGTTATACGAGAGGCCCAAGTTGACAGACACCGGCGTAAAGAGTGGCTAGGGAA

AAATTACTACTAAAGCCGAACACCTTCAGAACTGTCATACGTTCCCGAAGATAAGAAGCC

CCACCACGAAAGTGGCTTTATATTACCCGACCCCACGAAAGCTGTGAAA

>370_Sample_ASV7

CACCGCGGTTATACGAGAGGCCCAAGTTGACAGACACCGGCGTAAAGAGTGGTTAAGGAA

ACCCCATTACTAAAGCCGAACACCTTCAGAACTGTCATACGTTCCCGAAGATAAGAAGCC

CCACCACGAAAGTGGCTTTATATTACCCGACCCCACGAAAGCTGTGAAA

>370_Sample_ASV8

CACCGCGGTTAAACGAGAGACCCTAGTTGATCATCACGGCGTAAAGAGTGGTTAGGGGAT

TATCATAAATAAAGCCAAAGACCTCCCAAGCTGTCGCACGCATTCCGGAGGCGCGAAGCC

CACACACGAAAGTAGCTTTAACTATTGACCCTGATGCCACGAAAGCTAAGAAA

>370_Sample_ASV9

CACCGCGGTTATACGAGAGGCCCAAGTTGATAAGCCCGGCGTAAAGAGTGGTTATGGAGA

ATAAAACACTAAAGCTAAAGACCCCCTAGGCTGTTATACGCACCTGGCGGCTCGAACCAC

CTATACGAAAGTAGCTTTACCCCCCTTCCACCAGAATCCACGACAGCTGGGGCA

>370_Sample_ASV10

CACCGCGGTTATACGAGAGGCCCAAGTTGACAGACATCGGCGTAAAGAGTGGTTAAGGAA

ACCCCATTACTAAAGCCGAACGCCCTCAGAACTGTCATACGTTCCCGAAGGTAAGAAGCC

CCACTACGAAAGTGGCTTTAACCACACCCGACCCCACGAAAGCTGTGAAA

>370_Sample_ASV11

CACCGCGGTTATACGAGAGGCCCAAGTTGACAGACATCGGCGTAAAGAGTGGTTAAGGAA

ACCCCATTACTAAAGCCGAACGCCCTCAGAACTGTCATACGTTCCCGAAGGTAAGAAGCC

CCACTACGAAAGTGGCTTTAACCACACCCGACCCCACGAAAGCTAAGAAA

>370_Sample_ASV12

CACCGCGGTTATACGAGAGGCCCAAGTTGATAGATATCGGCGTAAAGAGTGGTTAAGGAA

ACCCCGTTACTAAAGCCGAACGCCCTCAGAACTGTCATACGTTCCCGAAGGTAAGAAGCC

CCACTACGAAAGTGGCTTTAACCACACCCGACCCCACGAAAGCTGTGAAA

>370_Sample_ASV13

CACCGCGGTTATACGAGAGGCCCTAGTTGATAAATACGGCGTAAAGAGTGGTTAGAGGAC

TCCCATAATAAAGCCAAAGACCCCCCGGGCCGTCACACGCATACGAGGGCACGAAGTTCA

CACACGAAAGTAGCTTTAAATTACCCGACGCCACGAAAGCTAAGAAA

>370_Sample_ASV14

GACCGCGGTTATACGAGAGACTCTAGTTGATAGATACGGCGTAAAGAGTGGTTTGGGATA

AACTATAAATAAAGCCAAAGACCTTCCAAGCTGTTAAACGCATACCGAAGGCACGAAGCC

CCAACACGAAAGTAGCTTTACTACTACCCCCGATGCCACGAAAGCTAAGACA

>370_Sample_ASV15

CACCGCGGTTATACGAGAGACCCTAGTTGATAAATACGGCGTAAAGAGTGGTTATGGGAA

AAACAAATAAAGTCAAACAACCTCTTAGCTGTTATACGCATTATGAGAGCATGAAGCCCC

CTCACGAAAGTAACTTTAATACCTCCTGACCCCACGAAAGCTAAGAAA

>370_Sample_ASV16

CACCGCGGTCATACGATTAACCCAAGTTAATAGGCACACGGCGTAAAGCGTGTTTAAGCA

CTATGCCAAATAAAGTTAAACTCCAATTAAGCTGTAAAAAGCCATAATCAAGACGAAAAT

AAATAACGAAAGTAACTTTACAACCGCTGAAACACGATAGCTAAGATC

>370_Sample_ASV17

CACCGCGGTTATACGAAAGACCCTAGTTTATAGACACGGCGTAAAGGGTGGTTCGGGACT

AAACAAAATAAAGCCAAAGACCTCCTAAGCCGTCATACGCTCACGGAAGCACGAAGCCCA

AGTACGAAAGTAGCTTTACAAAACATGCCTGACTCCACGAAAGCTAGGAAA

>370_Sample_ASV18

CACCGCGGTTATACGAAAGACCCTAGTTGATAGGCACGGCGTAAAGGGTGGTTAGGGATA

TATCAAAAATAAAGTTAAAGAGCCTCTAAGCTGTCGCACGCATCCCGAGAGCTCGAGACC

CAGACACGAAAGTAACTTTAAAATACCTCACCTGACCCCACGAAAGCTAAGAAA

>370_Sample_ASV19

CACCGCGGTTATACGAAAGACCCTAGTTGATAGCCACGGCGTAAAGGGTGGTTAAGGTAT

ACAACAAATAAAGCTAAATAGCCTCTAGGCCGTCGCACGCATTCCGAGAGCTCGAAACCC

AAAAACGAAAGTAGCTTTAAAATAAACAGACCTGACCCCACGAAAGCTAAGAAA

>370_Sample_ASV20

CACCGCGGTTATACGAGAGGCCCAAGTTGATACTCCACGGCGTAAAGAGTGGTTAGAAAA

GACCTACTACTAAAGCCGAACGCCTTCAAAGCTGTTATACGCATCCGAAGGTGAGAAGCC

CACCCACGAAAGTGGCTTTACAACCTTGACTCCACGAAAGCTATGACA

>370_Sample_ASV21

CACCGCGGTTATACGAGAGACCCCAGTTGATAAACACGGCGTAAAGAGTGGTTAAGGGCA

CAAAACAAATAAAGTCAAACAACCTCTTAGCTGTTATACGCATTATGAGGATATGAAGCC

CCCCTACGAAAGTAACTTTACCAACCCCTGACCCCACGAAAGCTAAGAAA

>370_Sample_ASV22

CACCGCGGTTATACGAGAGACCCTAGTTGATATATACGGCGTAAAGAGTGGTTTGGAATA

ACCAAGTAATATAGCCAAAGACCTCCCAAGCCGTCACACGCACCCCGGAGGCACGAAGCC

CAAACACGAAAGTAGCTTTATTAACTTACCGACGCCACGAAAGCTAAGAAA

>370_Sample_ASV23

CACCGCGGTTATACGAAAGACCCTAGTTTATAGGCACGGCGTAAAGGGTGGTTAGGGGAT

AAACAAAATAAAGCCAAAGACCTTCTAAGTCGTCATACACTCATGAAGGCACGAAGCCCA

AATACGAAAGTAACTTTACAACTTAGACCCGACTCCACGAAAGCTGGGGGA

>370_Sample_ASV24

CACCGCGGTTATACGAAAGACCCTAGTTGATAGATCACGGCGTAAAGGGTGGTTAAGGAG

AACAAAAATAAAGCTAAAAATCTTCTAAGCTGTCATACGCTTTCCGAAGACATGAGACCC

AACCACGAAAGTAGCTTTAAACTTTCCTGACACCACGAAAGCCAAGAAA

>372_Sample_ASV1

TACCGCGGTTATACGAAAGACCCAAGTTAATAAACTACGGCGTAAAGCGTGGTTAGAGAC

CTCCAATAATTAAAGCCAAAGACCTTCTAAGCCGTCGCACGCCCCGCGAAGACACGAAGC

CCAAAAACGAAAGTAGCTTTATTCTTACTCGACCCCACGAAAGCTAGGGCA

>372_Sample_ASV2

CACCGCGGTTATACGAAAGACCCTAGTTGATGGGTGCGGCGTAAAGGGTGGTTAGGGACT

ACAAAAAATAGAGCTAAAGATCCTCTAGGTCGTCATACACATCACGAGGAAACGAAGCCC

AAATACGAAAGTAGCTCTAAATATAATCCTGACGCCACGAAAGCCAAGAAA

>372_Sample_ASV3

CACCGCGGTTATACGAAAGACCCTAGTTGATGGGTGCGGCGTAAAGGGTGGTTAGGGACT

ACAAAAAATAGAGCTAAAGATCCTCTAGGTCGTCATACACATCACGAGGAAACGAAGCCC

AAATACGAAAGTAGCTCTAAATATAATCCTGACGCCACGAAAGCTGTGAAA

>372_Sample_ASV4

CACCGCGGTTATACGAGAGACCCTAGTTGATTAAAGCGGCGTAAAGAGTGGTTATGGAAC

TATTCTTTTAAAGCAGAAAACCTCTCAAACTGTTATACGCACCCAGAGGTCTGAATCCCT

CACACGAAAGTGACTTTATTTATGCCTACCAGAACCCACGAAAGCTGGGACA

>372_Sample_ASV5

CACCGCGGTTATACGAGAGGCCCAAGTTGACAGACACCGGCGTAAAGAGTGGCTAGGGAA

AAATTACTACTAAAGCCGAACACCTTCAGAACTGTCATACGTTCCCGAAGATAAGAAGCC

CCACCACGAAAGTGGCTTTATATTACCCGACCCCACGAAAGCTGTGAAA

>372_Sample_ASV6

CACCGCGGTTATACGAGAGGCCCTAGTTGATAGCCACGGCGCAAAGAGTGGTTAAGGAGC

CCCACTAAATAAAGTCGAACACTTCCTAGGCCGTTATACGTTTTCTAGAAGCACGAAACC

CAATTACACGAAAGCAACTTTATACTAAACAACCTGACCCCACGAAAGCTAAGAAA

>372_Sample_ASV7

CACCGCGGTTATACGAGAGGCCCAAGTTGACAGACATCGGCGTAAAGAGTGGTTAAGGAA

ACCCCATTACTAAAGCCGAACGCCCTCAGAACTGTCATACGTTCCCGAAGGTAAGAAGCC

CCACTACGAAAGTGGCTTTAACCACACCCGACCCCACGAAAGCTGTGAAA

>372_Sample_ASV8

CACCGCGGTTATACGAGAGGCCCAAGTTGACAGACATCGGCGTAAAGAGTGGTTAAGGAA

ACCCCATTACTAAAGCCGAACGCCCTCAGAACTGTCATACGTTCCCGAAGGTAAGAAGCC

CCACTACGAAAGTGGCTTTAACCACACCCGACCCCACGAAAGCTAAGAAA

>372_Sample_ASV9

CACCGCGGTTATACGAGAGGCCCAAGTTGATAGATATCGGCGTAAAGAGTGGTTAAGGAA

ACCCCGTTACTAAAGCCGAACGCCCTCAGAACTGTCATACGTTCCCGAAGGTAAGAAGCC

CCACTACGAAAGTGGCTTTAACCACACCCGACCCCACGAAAGCTGTGAAA

>372_Sample_ASV10

CACCGCGGTTATACGAAAGACCCCAGTTTATAGACACGGCGTAAAGGGTGGTTATAGGAC

AAACTAACATAGAGCCAAAGACCCCCTAAGCCGTCATACGCTCACGAGGGCTCGAAGCCC

CTATACGAAAGTAGCTCTACCAAAAAATGCTCGACTCCACGAAAGCTGGGACA

>372_Sample_ASV11

CACCGCGGTTATACGAAAGACCCTAGTTGATGGATCACGGCGTAAAGGGTGGTTAAGGGA

TATAAAAATAAAGCTAAAAATCCTCTTAGCTGTCATACGCTTTCCGAAGACATGAGGCCC

AATTACGAAAGTAGCTTTAAACCACCCTGACACCACGAAAGCCAAGAAA

>372_Sample_ASV12

CACCGCGGTTATACGAGAGGCCCTAGTTGATAACTACCGGCGTAAAGAGTGGTTATGGAA

AAATATTTAATAAAGCCGAACACCCCCTCAGCCGTCATACGCACCTGGGAGCACGAAGAC

CTACTGCGAAAGCAGCTTTAATTATGCCTGACCCCACGACAGCTAAGAAA

>372_Sample_ASV13

CACCGCGGTTATACGAGAGGCCCAAGTTGATTATTACCGGCGTAAAGCGTGGTTAAGATT

TTTATAGGACTAAAGCCGAACCCCCTCAGAGCTGTTATACGCACCCGAAGGTTAGAAGTT

CAATCACGAAAGTGGCTTTACAACCCCGAACCCACGAAAGCTAAGATA

>372_Sample_ASV14

CACCGCGGTTATACGAGAGACCCTAATTGATAGGCGCGGCGTAAAGAGTGGTTTAGGACT

AAAACTAATAAAGCTAAAGATCCTCCCAGCCGTCGCACGCATTCTGAGGACACGAAACTC

AACCACGAAAGTAGCTTTAACATACTCTCCTGACCCCACGAAAGCTAAGAAA

>372_Sample_ASV15

CACCGCGGTTATACGAAAGACCCTAGTTGATAGGCACGGCGTAAAGGGTGGTTAGGGATA

TATCAAAAATAAAGTTAAAGAGCCTCTAAGCTGTCGCACGCATCCCGAGAGCTCGAGACC

CAGACACGAAAGTAACTTTAAAATACCTCACCTGACCCCACGAAAGCTAAGAAA

>372_Sample_ASV16

CACCGCGGTTATACGAGAGACCCCAGTTGATAAACACGGCGTAAAGAGTGGTTAAGGGCA

CAAAACAAATAAAGTCAAACAACCTCTTAGCTGTTATACGCATTATGAGGATATGAAGCC

CCCCTACGAAAGTAACTTTACCAACCCCTGACCCCACGAAAGCTAAGAAA

>372_Sample_ASV17

CACCGCGGTTATACGAAAGACCCTAGTTTATAGGCACGGCGTAAAGGGTGGTTAGGGGAT

AAACAAAATAAAGCCAAAGACCTTCTAAGTCGTCATACACTCATGAAGGCACGAAGCCCA

AATACGAAAGTAACTTTACAACTTAGACCCGACTCCACGAAAGCTGGGGGA

>372_Sample_ASV18

CACCGCGGTTATACGAGAGACCCTAATTAATAGCTACGGCGTAAAGAGTGGTTTAGGGTA

AAAATTAATAAAGCCGAAGTTTCTCCTAGCCGTTGTACGCACTCTGAGAACACGAAGCCC

CACTACGAAAGTAGCTTTACCTATTATTTCCTGACCCCACGAAAGCTAAGAAA

>372_Sample_ASV19

CACCGCGGTTATACGAGAGACCCTAATTAATAGATACGGCGTAAAGAGTGGTTTAGGACA

AAAATTAATAAAGCCGAAGTTTCTCCTAGCCGTTGTACGCACTCTGAGAACACGAAGCCC

CACTACGAAAGTAGCTTTACCTATTATTTCCTGACCCCACGAAAGCTAAGAAA

>372_Sample_ASV20

CACCGCGGTTATACGAAAGACCCTAGTTGATAGATCACGGCGTAAAGGGTGGTTAAGGAA

AACAAAAATAAAGCTAAAAATCCTCTAAGCTGTCATACGCTTTCCGAAGACATGAGATCC

AACCACGAAAGTAGCTTTAAAATTTCCTGACACCACGAAAGCCAAGGAA

>385_Sample_ASV1

CACCGCGGTTATACGAGAGGCCCAAGTTGACAGACATCGGCGTAAAGAGTGGTTAAGGAA

AACCTTCAACTAAAGTCGAACGCTCTCAAGACTGTCGTACGTTTCCGAGAGTATGAAGCC

CCACCACGAAAGTGACTTTACCTCACCTGACTCCACGAAAGCTGCGAAA

>385_Sample_ASV2

CACCGCGGTTATACGAAAGACCCTAGTTGATGGGTGCGGCGTAAAGGGTGGTTAGGGACT

ACAAAAAATAGAGCTAAAGATCCTCTAGGTCGTCATACACATCACGAGGAAACGAAGCCC

AAATACGAAAGTAGCTCTAAATATAATCCTGACGCCACGAAAGCCAAGAAA

>385_Sample_ASV3

CACCGCGGTTATACGAGAGACCCTAGTTGATTAAAGCGGCGTAAAGAGTGGTTATGGAAC

TATTCTTTTAAAGCAGAAAACCTCTCAAACTGTTATACGCACCCAGAGGTCTGAATCCCT

CACACGAAAGTGACTTTATTTATGCCTACCAGAACCCACGAAAGCTGTGAAA

>385_Sample_ASV4

CACCGCGGTTATACGAGAGACCCTAGTTGATTAAAGCGGCGTAAAGAGTGGTTATGGAAC

TATTCTTTTAAAGCAGAAAACCTCTCAAACTGTTATACGCACCCAGAGGTCTGAATCCCT

CACACGAAAGTGACTTTATTTATGCCTACCAGAACCCACGAAAGCTGGGACA

>385_Sample_ASV5

CACCGCGGTTATACGAGAGACCCTAGTTGATTTAAGCGGCGTAAAGGGTGGTTATGGAAC

TATTCTTTTAAAGCAGAAAACCTCTCAAACTGTTATACGCACCCAGAGGTCTGAATCCCT

CACACGAAAGTGACTTTATTTTTGCCCACCAGAATCCACGAAAGCTGGGACA

>385_Sample_ASV6

CACCGCGGTTATACGAAAGACCCTAGTTTATAGGTACGGCGTAAAGGGTGGTTAGGGGAC

AAACAAAATAAAGCCAAAGACCTCCTAAGCCGTCATACGCTCACGGAAGCACGAAGCCCA

AACACGAAAGTAGCTTTACCGAACATGCCCGACTCCACGAAAGCTGGGGAA

>385_Sample_ASV7

CACCGCGGTTATACGAAAGACCCTAGTTTATAGGTACGGCGTAAAGGGTGGTTAGGGGAC

AAACAAAATAAAGCCAAAGACCTCCTAAGCCGTCATACGCTCACGGAAGCACGAAGCCCA

AACACGAAAGTAGCTTTACCGAACATGCCCGACCCCACGAAAGCTAAGAAA

>385_Sample_ASV8

CACCGCGGTTATACGAAAGACCCTAGTTTATAGGTACGGCGTAAAGGGTGGTTAGGGGAC

AAACAAAATAAAGCCAAAGACCTCCTAAGCCGTCATACGCTCACGGAAGCACGAAGCCCA

AACACGAAAGTAGCTTTACCGAACATGCCCGACTCCACGAAAGCTAAGAAA

>385_Sample_ASV9

CACCGCGGTTATACGAAAGACCCTAGTTGATAGCCACGGCGTAAAGGGTGGTTAAGGAAC

CTAAAAAATAAAGCTAAAGACCCTCTAGGCCGTCATACGCATTACGAGGCAAACGAAACC

CCAACACGAAAGTAGCTTTAGACAAATATCCTGATGCCACGAAAGCCAAGAAA

>385_Sample_ASV10

CACCGCGGTTATACGAAAGACCCTAGTTGATAGCCACGGCGTAAAGGGTGGTTAAGGAAC

CTAAAAAATAAAGCTAAAGACCCTCTAGGCCGTCATACGCATTACGAGGCAAACGAAACC

CCAACACGAAAGTAGCTTTAGACAAATATCCTGATGCCACGAAAGCTGTGAAA

>385_Sample_ASV11

CACCGCGGTTATACGAGAGACCCTAGTTGATATACACGGCGTAAAGAGTGGTTTGGGGTA

TATAATAAATAAAGCCAAAGACCTTCTAGGCCGTTGAACGCACCACGAAGGAGCGAAGTC

CCAATACGAAAGTAGCTTTATAAAACCTGACGCCACGAAAGCTAAGAAA

>385_Sample_ASV12

CACCGCGGTTATACGAGAGGCCCAAGTTGACAGACACCGGCGTAAAGAGTGGCTAGGGAA

AAATTACTACTAAAGCCGAACACCTTCAGAACTGTCATACGTTCCCGAAGATAAGAAGCC

CCACCACGAAAGTGGCTTTATATTACCCGACCCCACGAAAGCTGTGAAA

>385_Sample_ASV13

CACCGCGGTTATACGAGAGGCCCAAGTTGACAGACACCGGCGTAAAGAGTGGCTAAGGAA

AAATTTCTACTAAAGCCGAACACCTTCAGAACTGTCATACGTTCCCGAAGAAAAGAAGCC

CCACCACGAAAGTGGCTTTATATTACCCGACCCCACGAAAGCTGTGAAA

>385_Sample_ASV14

CACCGCGGTTAAACGAGAGACCCTAGTTGATCATCACGGCGTAAAGAGTGGTTAGGGGAT

TATCATAAATAAAGCCAAAGACCTCCCAAGCTGTCGCACGCATTCCGGAGGCGCGAAGCC

CACACACGAAAGTAGCTTTAACTATTGACCCTGATGCCACGAAAGCTAAGAAA

>385_Sample_ASV15

CACCGCGGTTATACGAGAGGCCCAAGTTGATAAGCCCGGCGTAAAGAGTGGTTATGGAGA

ATAAAACACTAAAGCTAAAGACCCCCTAGGCTGTTATACGCACCTGGCGGCTCGAACCAC

CTATACGAAAGTAGCTTTACCCCCCTTCCACCAGAATCCACGACAGCTGGGGCA

>385_Sample_ASV16

CACCGCGGTTATACGAGAGGCCCAAGTTGACAGACATCGGCGTAAAGAGTGGTTAAGGAA

ACCCCATTACTAAAGCCGAACGCCCTCAGAACTGTCATACGTTCCCGAAGGTAAGAAGCC

CCACTACGAAAGTGGCTTTAACCACACCCGACCCCACGAAAGCTGTGAAA

>385_Sample_ASV17

CACCGCGGTTATACGAGAGGCCCAAGTTGATAGATATCGGCGTAAAGAGTGGTTAAGGAA

ACCCCGTTACTAAAGCCGAACGCCCTCAGAACTGTCATACGTTCCCGAAGGTAAGAAGCC

CCACTACGAAAGTGGCTTTAACCACACCCGACCCCACGAAAGCTGTGAAA

>385_Sample_ASV18

CACCGCGGTCACACGATTAACCCAAGTCAATAGAAGCCGGCGTAAAGAGTGTTTTAGATC

ACCCCCTCCCCAATAAAGCTAAAACTCACCTGAGTTGTAAAAAACTCCAGTTGACACAAA

ATAGACTACGAAAGTGGCTTTAACATATCTGAACACACAATAGCTAAGACC

>385_Sample_ASV19

CACCGCGGTTATACGAGAGGCCCTAGTTGATAAATACGGCGTAAAGAGTGGTTAGAGGAC

TCCCATAATAAAGCCAAAGACCCCCCGGGCCGTCACACGCATACGAGGGCACGAAGTTCA

CACACGAAAGTAGCTTTAAATTACCCGACGCCACGAAAGCTAAGAAA

>385_Sample_ASV20

CACCGCGGTTATACGAGAAACCCTAGTTGACAAATACGGCGTAAAGAGTGGTTAAGGGGT

ATCAAAAATAAAGTCAAACATTTTCCTAGTTGTTATAAGCATTTCGAGAACATGAAGCCC

AACCACGAAAGTAACTTTACCTCCACCTGACCCCACGAAAGCTAAGAAA

>385_Sample_ASV21

GACCGCGGTTATACGAGAGACTCTAGTTGATAGATACGGCGTAAAGAGTGGTTTGGGATA

AACTATAAATAAAGCCAAAGACCTTCCAAGCTGTTAAACGCATACCGAAGGCACGAAGCC

CCAACACGAAAGTAGCTTTACTACTACCCCCGATGCCACGAAAGCTAAGACA

>385_Sample_ASV22

CACCGCGGTTATACGAGAGACCCTAGTTGATAAATACGGCGTAAAGAGTGGTTATGGGAA

AAACAAATAAAGTCAAACAACCTCTTAGCTGTTATACGCATTATGAGAGCATGAAGCCCC

CTCACGAAAGTAACTTTAATACCTCCTGACCCCACGAAAGCTAAGAAA

>385_Sample_ASV23

CACCGCGGTTATACGAGAGACCCCAGTTGATAAACACGGCGTAAAGAGTGGTTAAGGGCA

CAAAACAAATAAAGTCAAACAACCTCTTAGCTGTTATACGCATTATGAGAGCATGAAGCC

CCCTCACGAAAGTAACTTTAATACCTCCTGACCCCACGAAAGCTAAGAAA

>385_Sample_ASV24

CACCGCGGTTATACGAGAGACCCTAGTTGATAGCTACGGCGTAAAGCGTGGTTAGGAGAA

CAAGATAAATAAAGTCAAACAATCTCTCGGCCGTTATACGTTATCTGAGAATATGAAGTC

CTACCACGAAAGTAACTTTAATTTTTCTGACCCCACGAAAACTGAGGAA

>385_Sample_ASV25

CACCGCGGTCATACGATTAACCCAAGTTAATAGGCACACGGCGTAAAGCGTGTTTAAGCA

CTATGCCAAATAAAGTTAAACTCCAATTAAGCTGTAAAAAGCCATAATCAAGACGAAAAT

AAATAACGAAAGTAACTTTACAACCGCTGAAACACGATAGCTAAGATC

>385_Sample_ASV26

CACCGCGGTTATACGAGAGGCCCTAGTTGATAACTACCGGCGTAAAGAGTGGTTATGGAA

AAATATTTAATAAAGCCGAACACCCCCTCAGCCGTCATACGCACCTGGGAGCACGAAGAC

CTACTGCGAAAGCAGCTTTAATTATGCCTGACCCCACGACAGCTAAGAAA

>385_Sample_ASV27

CACCGCGGTTATACGAAAGACCCTAGTTTATAGACACGGCGTAAAGGGTGGTTCGGGACT

AAACAAAATAAAGCCAAAGACCTCCTAAGCCGTCATACGCTCACGGAAGCACGAAGCCCA

AGTACGAAAGTAGCTTTACAAAACATGCCTGACTCCACGAAAGCTAGGAAA

>385_Sample_ASV28

CACCGCGGTTATACGAAAGACCCTAGTTGATAGGCACGGCGTAAAGGGTGGTTAGGGATA

TATCAAAAATAAAGTTAAAGAGCCTCTAAGCTGTCGCACGCATCCCGAGAGCTCGAGACC

CAGACACGAAAGTAACTTTAAAATACCTCACCTGACCCCACGAAAGCTAAGAAA

>385_Sample_ASV29

CACCGCGGTTATACGAAAGACCCTAGTTGATAGCCACGGCGTAAAGGGTGGTTAAGGTAT

ACAACAAATAAAGCTAAATAGCCTCTAGGCCGTCGCACGCATTCCGAGAGCTCGAAACCC

AAAAACGAAAGTAGCTTTAAAATAAACAGACCTGACCCCACGAAAGCTAAGAAA

>385_Sample_ASV30

CACCGCGGTTATACGAGAGGCCCAAGTTGATACTCCACGGCGTAAAGAGTGGTTAGAAAA

GACCTACTACTAAAGCCGAACGCCTTCAAAGCTGTTATACGCATCCGAAGGTGAGAAGCC

CACCCACGAAAGTGGCTTTACAACCTTGACTCCACGAAAGCTATGACA

>385_Sample_ASV31

CACCGCGGTTATACGAGAGACCCCAGTTGATAAACACGGCGTAAAGAGTGGTTAAGGGCA

CAAAACAAATAAAGTCAAACAACCTCTTAGCTGTTATACGCATTATGAGGATATGAAGCC

CCCCTACGAAAGTAACTTTACCAACCCCTGACCCCACGAAAGCTAAGAAA

>385_Sample_ASV32

CACCGCGGTTATACGAAAGACCCTAGTTTATAGGCACGGCGTAAAGGGTGGTTAGGGGAT

AAACAAAATAAAGCCAAAGACCTTCTAAGTCGTCATACACTCATGAAGGCACGAAGCCCA

AATACGAAAGTAACTTTACAACTTAGACCCGACTCCACGAAAGCTGGGGGA

>385_Sample_ASV33

CACCGCGGTTATACGAGAGACCCTAATTAATAGCTACGGCGTAAAGAGTGGTTTAGGACA

AAAATTAATAAAGCCGAAGTTTCTCCTAGCCGTTGTACGCACTCTGAGAACACGAAGCCC

CACTACGAAAGTAGCTTTACCAATAATCTCCTGACCCCACGAAAGCTAAGAAA

>385_Sample_ASV34

CACCGCGGTTATACGAGAGACCCTAATTAATAGATACGGCGTAAAGAGTGGTTTAGGACA

AAAATTAATAAAGCCGAAGTTTCTCCTAGCCGTTGTACGCACTCTGAGAACACGAAGCCC

CACTACGAAAGTAGCTTTACCAATAATCTCCTGACCCCACGAAAGCTAAGAAA

>385_Sample_ASV35

CACCGCGGTTATACGAGAGACCCTAATTAATAGCTACGGCGTAAAGAGTGGTTTAGGACA

AAAATTAATAAAGCCGAAGTTTCTCCTAGCCGTTGTACGCACTCTGAGAACACGAAGCCC

CACTACGAAAGTAGCTTTACCTATTATTTCCTGACCCCACGAAAGCTAAGAAA

>385_Sample_ASV36

CACCGCGGTTATACGAGAGACCCTAATTAATAGCTACGGCGTAAAGAGTGGTTTAGGGTA

AAAATTAATAAAGCCGAAGTTTCTCCTAGCCGTTGTACGCACTCTGAGAACACGAAGCCC

CACTACGAAAGTAGCTTTACCTATTATTTCCTGACCCCACGAAAGCTAAGAAA

>385_Sample_ASV37

CACCGCGGTTATACGAGAGACCCTAATTAATAGATACGGCGTAAAGAGTGGTTTAGGACA

AAAATTAATAAAGCCGAAGTTTCTCCTAGCCGTTGTACGCACTCTGAGAACACGAAGCCC

CACTACGAAAGTAGCTTTACCTATTATTTCCTGACCCCACGAAAGCTAAGAAA

>385_Sample_ASV38

CACCGCGGTTATACGAGAGGCCCAAGTTGATAGACATCGGCGTAAAGAGTGGTTAAGGAA

GTCTCAAAACTAAAGCCGAATGCCTTCAAGACTGTTATACGTTTCCGAAGGTAAGAAGCC

CCACTACGAAAGTGGCTTTACTCCACCTGACTCCACGAAAGCTGTGAAA

>385_Sample_ASV39

CACCGCGGTTATACGAGAGACTCCAGTTGATAGCTACGGCGTAAAGAGTGGTTTGGGGCC

CGCCCAAAATAAAGCCAAAGACCTCCCAAGCCGTCAAACGCACCCCGGAGGCACGAAGTC

CTAACGCGAAAGCAACTTTACCTTCCCCGACGCCACGAAAGCTAAGAAA

>385_Sample_ASV40

CACCGCGGTTATACGAAAGACCCTAGTTGATAGCTACGGCGTAAAGGGTGGTTAAGGTAT

ATAACAAATAAAGCTAAAGAGCCTCCAAGCCGTCGTACGCATTCTGAGAGCTCGAAACCC

TAACACGAAAGTAGCTTTAAAAAATAACACCTGACCCCACGAAAGCTAAGAAA

>385_Sample_ASV41

CACCGCGGTTATACGAAAGACCCTAGTTGATAGATCACGGCGTAAAGGGTGGTTAAGGAG

AACAAAAATAAAGCTAAAAATCCTCTAAGCTGTCATACGCTTTCCGAAGACATGAGACCC

AACCACGAAAGTAACTTTAAACTTTCCTGACACCACGAAAGCCAAGAAA

>385_Sample_ASV42

CACCGCGGTTATACGAAAGACCCTAGTTGATAGATCACGGCGTAAAGGGTGGTTAAGGAG

AACAAAAATAAAGCTAAAAATCTTCTAAGCTGTCATACGCTTTCCGAAGACATGAGACCC

AACCACGAAAGTAGCTTTAAACTTTCCTGACACCACGAAAGCCAAGAAA

>396_Sample_ASV1

TACCGCGGTTATACGAAAGACCCAAGTTAATAAACTACGGCGTAAAGCGTGGTTAGAGAC

CTCCAATAATTAAAGCCAAAGACCTTCTAAGCCGTCGCACGCCCCGCGAAGACACGAAGC

CCAAAAACGAAAGTAGCTTTATTCTTACTCGACCCCACGAAAGCTAGGGCA

>396_Sample_ASV2

CACCGCGGTTATACGAAAGACCCTAGTTGATGGGTGCGGCGTAAAGGGTGGTTAGGGACT

ACAAAAAATAGAGCTAAAGATCCTCTAGGTCGTCATACACATCACGAGGAAACGAAGCCC

AAATACGAAAGTAGCTCTAAATATAATCCTGACGCCACGAAAGCCAAGAAA

>396_Sample_ASV3

CACCGCGGTTATACGAGAGACCCTAGTTGATTAAAGCGGCGTAAAGAGTGGTTATGGAAC

TATTCTTTTAAAGCAGAAAACCTCTCAAACTGTTATACGCACCCAGAGGTCTGAATCCCT

CACACGAAAGTGACTTTATTTATGCCTACCAGAACCCACGAAAGCTATGACA

>396_Sample_ASV4

CACCGCGGTTATACGAGAGACCCTAGTTGATTAAAGCGGCGTAAAGAGTGGTTATGGAAC

TATTCTTTTAAAGCAGAAAACCTCTCAAACTGTTATACGCACCCAGAGGTCTGAATCCCT

CACACGAAAGTGACTTTATTTATGCCTACCAGAACCCACGAAAGCTGGGACA

>396_Sample_ASV5

CACCGCGGTTATACGAGAGGCCCAAGTTGACAGACACCGGCGTAAAGAGTGGCTAGGGAA

AAATTACTACTAAAGCCGAACACCTTCAGAACTGTCATACGTTCCCGAAGATAAGAAGCC

CCACCACGAAAGTGACTTTATATTACCCGACCCCACGAAAGCTGTGAAA

>396_Sample_ASV6

CACCGCGGTTATACGAGAGGCCCAAGTTGACAGACACCGGCGTAAAGAGTGGCTAGGGAA

AAATTACTACTAAAGCCGAACACCTTCAGAACTGTCATACGTTCCCGAAGATAAGAAGCC

CCACCACGAAAGTGGCTTTATATTACCCGACCCCACGAAAGCTGTGAAA

>396_Sample_ASV7

CACCGCGGTTATACGAGAGGCCCAAGTTGACAGACACCGGCGTAAAGAGTGGCTAGGGAA

AAATTACTACTAAAGCCGAACACCTTCAGAACTGTCATACGTTCCCGAAGATAAGAAGCC

CCACCACGAAAGTGGCTTTAACCACACCCGACCCCACGAAAGCTGTGAAA

>396_Sample_ASV8

CACCGCGGTTATACGAGAGGCCCAAGTTGACAGACATCGGCGTAAAGAGTGGTTAAGGAA

ACCCCATTACTAAAGCCGAACACCTTCAGAACTGTCATACGTTCCCGAAGATAAGAAGCC

CCACCACGAAAGTGGCTTTATATTACCCGACCCCACGAAAGCTGTGAAA

>396_Sample_ASV9

CACCGCGGTTATACGAAAGACTCTAGTTGACAAACACGGCGTAAAGGGTGGTTAGGATAA

CAACAAAATAAAGCTAAAGACTTACCAAGCCGTCATACGCCCATGTAAGAGCGAAAACCA

CAAACGAAAGTAACTTTATTACACCATCCGAATCCACGAAAGCTAAGAAA

>396_Sample_ASV10

CACCGCGGTTATACGAAAGACCCTAGTTGATAGATCACGGCGTAAAGGGTGGTTAGGGAA

TACAAAAATAAAGCTAAAAATCCTCTAAGCTGTCATACGCTTCCCGAAGACATGAAACCC

AACCACGAAAGTAGCTTTAAACTCTCCTGACACCACGAAAGCCAAGGAA

>396_Sample_ASV11

CACCGCGGTTATACGAGAGGCCCAAGTTGATAAGCCCGGCGTAAAGAGTGGTTATGGAGA

ATAAAACACTAAAGCTAAAGACCCCCTAGGCTGTTATACGCACCTGGCGGCTCGAACCAC

CTATACGAAAGTAGCTTTACCCCCCTTCCACCAGAATCCACGACAGCTGGGGCA

>396_Sample_ASV12

CACCGCGGTTATACGAGAGGCCCTAGTTGATAGCCACGGCGCAAAGAGTGGTTAAGGAGC

CCCACTAAATAAAGTCGAACACTTCCTAGGCCGTTATACGTTTTCTAGAAGCACGAAACC

CAATTACACGAAAGCAACTTTATACTAAACAACCTGACCCCACGAAAGCTAAGAAA

>396_Sample_ASV13

CACCGCGGTTATACGAGAGGCCCAAGTTGACAGACATCGGCGTAAAGAGTGGTTAAGGAA

ACCCCATTACTAAAGCCGAACGCCCTCAGAACTGTCATACGTTCCCGAAGGTAAGAAGCC

CCACTACGAAAGTGGCTTTAACCACACCCGACCCCACGAAAGCTGTGAAA

>396_Sample_ASV14

CACCGCGGTTATACGAGAGGCCCCAGTTGATATTAGCGGCGTAAAGGGTGGTTAAGGACT

TTAATAACTAAAGCCAAACACTTCCCCGGCTGTTGTACGTAGACCGGAAGCTTGAAGCCC

AGACACGAAAGTAGCTTTATTTCCCCTGACCCCACGAAAGCTGAGAAA

>396_Sample_ASV15

CACCGCGGTTATACGAGAGGCCCCAGTTGATATTAGCGGCGTAAAGGGTGGTTAAGGACT

TTAATAACTAAAGCTAAACACTTCCCCGGCTGTTGTACGTAGACCGGAAGCTTGAAGCCC

AGACACGAAAGTAGCTTTATTCCCCCTGACCCCACGAAAGCTGAGAAA

>396_Sample_ASV16

CACCGCGGTTATACGAGAGGCCCCAGTTGATATTAGCGGCGTAAAGGGTGGTTAAGGACT

TTAATAACTAAAGCTAAACACTTCCCCGGCTGTTGTACGTAGACCGGAAGCTTGAAGCCC

AGACACGAAAGTAGCTTTATTTCCCCTGACCCCACGAAAGCTGAGAAA

>396_Sample_ASV17

CACCGCGGTTATACGAGAGACCCCAGTTGATATTAGCGGCGTAAAGGGTGGTTAAGGACT

TTAATAACTAAAGCTAAACACTTCCCCGGCTGTTGTACGTAGACCGGAAGCTTGAAGCCC

AGACACGAAAGTAGCTTTATTTCCCCTGACCCCACGAAAGCTGAGAAA

>396_Sample_ASV18

CACCGCGGTTATACGAGAAACCCTAGTTGACAAATACGGCGTAAAGAGTGGTTAAGGGGT

ATCAAAAATAAAGTCAAACATTTTCCTAGTTGTTATAAGCATTTCGAGAACATGAAGCCC

AACCACGAAAGTAACTTTACCTTCACCTGACCCCACGAAAGCTAAGAAA

>396_Sample_ASV19

CACCGCGGTTATACGAAAGACCCCAGTTTATAGACACGGCGTAAAGGGTGGTTATAGGAC

AAACTAACATAGAGCCAAAGACCCCCTAAGCCGTCATACGCTCACGAGGGCTCGAAGCCC

CTATACGAAAGTAGCTCTACCAAAAAATGCTCGACTCCACGAAAGCTGGGACA

>396_Sample_ASV20

CACCGCGGTTATACGAGAGACCCTAATTGATAGGCACGGCGTAAAGAGTGGTTTAGGGTA

ACCCATAAATAAAGCTAAAGACCTTCTAAGCCGTCACACGCACACCGAAGGCACGAGACC

CCAACACGAAAGTAGCTTTACTTCTACCCCTGACGCCACGAAAGCTAAGAAA

>396_Sample_ASV21

CACCGCGGTTATACGAAAGACCCTAGTTGATGGATCACGGCGTAAAGGGTGGTTAAGGGA

TATAAAAATAAAGCTAAAAATCCTCTTAGCTGTCATACGCTTTCCGAAGACATGAGGCCC

AATTACGAAAGTAGCTTTAAACCACCCTGACACCACGAAAGCCAAGAAA

>396_Sample_ASV22

CACCGCGGTTATACGAAAGACCCCAGTTTATAGATGCGGCGTAAAGGGTGGTTAAGAGAA

AATCAAAATAAGGCCAAAGATCCCCTAAGCAGTCATACGCTCCACGAGGACACGAAACCC

AAATACGAAAGTTGCCTTAATAAACTTCACCTGATCCCACGAAAGCTGAAAAA

>396_Sample_ASV23

CACCGCGGTTATACGAAAGGCTCTAGTTTATAAATACGGCGTAAAGGGTGGTTAAGGAAA

AATTAAAATAATGCTAAAGACCCCCTAAGCAGTTATACGCTTCACGGAGGCACGAAACCC

AAATACGAAAGTTGCCTTAATAAACTTTACCTGACCCCACGAAAGCTGAAAAA

>396_Sample_ASV24

CACCGCGGTTATACGAAAGGCTCTAGTTTATAAATACGGCGTAAAGGGTGGTTAAGGAAA

AATTAAAATAATGCTAAAGACCCCCTAAGCAGTTATACGCTTCACGGAGGCACGAAACCC

AAATACGAAAGTTGCCTTAATAAACTTTACCTGACCCCACGAAAGCTGAGAAA

>396_Sample_ASV25

CACCGCGGTTATACGAGAGACTCTAGTTGATACCAACGGCGTAAAGAGTGGTTTGGGATG

CCCCTTAAAATAAAGCCAAAGACCTCCCAAGCCGTCAAACGCACCCCGGAGGCACGAAGC

CCCAACGCGAAAGCAACTTTACCTCCCTCCCGACGCCACGAAAGCTAAGAAA

>396_Sample_ASV26

CACCGCGGTTATACGAGAGACCCTAGTTGATAGCTACGGCGTAAAGCGTGGTTAGGAGAA

CAAGATAAATAAAGTCAAACAATCTCTCGGCCGTTATACGTTATCTGAGAATATGAAGTC

CTACCACGAAAGTAACTTTAATTTTTCTGACCCCACGAAAACTGAGGAA

>396_Sample_ASV27

CACCGCGGTTATACGAGAGGCCCTAGTTGATAACTACCGGCGTAAAGAGTGGTTATGGAA

AAATATTTAATAAAGCCGAACACCCCCTCAGCCGTCATACGCACCTGGGAGCACGAAGAC

CTACTGCGAAAGCAGCTTTAATTATGCCTGACCCCACGACAGCTAAGAAA

>396_Sample_ASV28

CACCGCGGTTATACGAGAGGCCCAAGTTGATTATTACCGGCGTAAAGCGTGGTTAAGATT

TTTATAGGACTAAAGCCGAACCCCCTCAGAGCTGTTATACGCACCCGAAGGTTAGAAGTT

CAATCACGAAAGTGGCTTTACAACCCCGAACCCACGAAAGCTAAGATA

>396_Sample_ASV29

CACCGCGGTTATACGAGAGGCCCAAGTTGATACTCCACGGCGTAAAGAGTGGTTAGAAAA

GACCTACTACTAAAGCCGAACGCCTTCAAAGCTGTTATACGCATCCGAAGGTGAGAAGCC

CACCCACGAAAGTGGCTTTACAACCTTGACTCCACGAAAGCTATGACA

>396_Sample_ASV30

CACCGCGGTTATACGAGAGACCCCAGTTGATAAACACGGCGTAAAGAGTGGTTAAGGGCA

CAAAACAAATAAAGTCAAACAACCTCTTAGCTGTTATACGCATTATGAGGATATGAAGCC

CCCCTACGAAAGTAACTTTACCAACCCCTGACCCCACGAAAGCTAAGAAA

>396_Sample_ASV31

CACCGCGGTTATACGAAAGACCCTAGTTTATAGGCACGGCGTAAAGGGTGGTTAGGGGAT

AAACAAAATAAAGCCAAAGACCTTCTAAGTCGTCATACACTCATGAAGGCACGAAGCCCA

AATACGAAAGTAACTTTACAACTTAGACCCGACTCCACGAAAGCTGGGGGA

>396_Sample_ASV32

CACCGCGGTTATACGAAAGACCCTAGTTGATAGCTACGGCGTAAAGGGTGGTTAAGGTAT

ATAACAAATAAAGCTAAAGAGCCTCCAAGCCGTCGTACGCATTCTGAGAGCTCGAAACCC

TAACACGAAAGTAGCTTTAAAAAATAACACCTGACCCCACGAAAGCTAAGAAA

>396_Sample_ASV33

CACCGCGGTTATACGAAAGACCCTAGTTGATAGATCACGGCGTAAAGGGTGGTTAAGGAG

AACAAAAATAAAGCTAAAAATCTTCTAAGCTGTCATACGCTTTCCGAAGACATGAGACCC

AACCACGAAAGTAGCTTTAAACTTTCCTGACACCACGAAAGCCAAGAAA

>397_Sample_ASV1

TACCGCGGTTATACGAAAGACCCAAGTTAATAAACTACGGCGTAAAGCGTGGTTAGAGAC

CTCCAATAATTAAAGCCAAAGACCTTCTAAGCCGTCGCACGCCCCGCGAAGACACGAAGC

CCAAAAACGAAAGTAGCTTTATTCTTACTCGACCCCACGAAAGCTAGGGCA

>397_Sample_ASV2

CACCGCGGTTATACGAAAGACCCTAGTTGATGGGTGCGGCGTAAAGGGTGGTTAGGGACT

ACAAAAAATAGAGCTAAAGATCCTCTAGGTCGTCATACACATCACGAGGAAACGAAGCCC

AAATACGAAAGTAGCTCTAAATATAATCCTGACGCCACGAAAGCCAAGAAA

>397_Sample_ASV3

CACCGCGGTTATACGAGAGACCCTAGTTGATTAAAGCGGCGTAAAGAGTGGTTATGGAAC

TATTCTTTTAAAGCAGAAAACCTCTCAAACTGTTATACGCACCCAGAGGTCTGAATCCCT

CACACGAAAGTGACTTTATTTATGCCTACCAGAACCCACGAAAGCTGGGACA

>397_Sample_ASV4

CACCGCGGTTATACGAAAGACCCTAGTTTATAGGTACGGCGTAAAGGGTGGTTAGGGGAC

AAACAAAATAAAGCCAAAGACCTCCTAAGCCGTCATACGCTCACGGAAGCACGAAGCCCA

AACACGAAAGTAGCTTTACCGAACATGCCCGACTCCACGAAAGCTGGGGAA

>397_Sample_ASV5

CACCGCGGTTATACGAGAGGCCCAAGTTGACAGACACCGGCGTAAAGAGTGGCTAGGGAA

AAATTACTACTAAAGCCGAACACCTTCAGAACTGTCATACGTTCCCGAAGATAAGAAGCC

CCACCACGAAAGTGACTTTATATTACCCGACCCCACGAAAGCTGTGAAA

>397_Sample_ASV6

CACCGCGGTTATACGAGAGGCCCAAGTTGACAGACACCGGCGTAAAGAGTGGCTAGGGAA

AAATTACTACTAAAGCCGAACACCTTCAGAACTGTCATACGTTCCCGAAGATAAGAAGCC

CCACCACGAAAGTGGCTTTATATTACCCGACCCCACGAAAGCTGTGATA

>397_Sample_ASV7

CACCGCGGTTATACGAGAGGCCCAAGTTGACAGACACCGGCGTAAAGAGTGGCTAGGGAA

AAATTACTACTAAAGCCGAACACCTTCAGAACTGTCATACGTTCCCGAAGATAAGAAGCC

CCACCACGAAAGTGGCTTTATATTACCCGACCCCACGAAAGCTGTGAAA

>397_Sample_ASV8

CACCGCGGTTATACGAAAGACTCTAGTTGACAAACACGGCGTAAAGGGTGGTTAGGATAA

CAACAAAATAAAGCTAAAGACTTACCAAGCCGTCATACGCCCATGTAAGAGCGAAAACCA

CAAACGAAAGTAACTTTATTACACCATCCGAATCCACGAAAGCTAAGAAA

>397_Sample_ASV9

CACCGCGGTTATACGAAAGACCCTAGTTGATAGATCACGGCGTAAAGGGTGGTTAGGGAA

TACAAAAATAAAGCTAAAAATCCTCTAAGCTGTCATACGCTTCCCGAAGACATGAAACCC

AACCACGAAAGTAGCTTTAAACTCTCCTGACACCACGAAAGCCAAGGAA

>397_Sample_ASV10

CACCGCGGTTATACGAGAGGCCCAAGTTGACAGACATCGGCGTAAAGAGTGGTTAAGGAA

ACCCCATTACTAAAGCCGAACGCCCTCAGAACTGTCATACGTTCCCGAAGGTAAGAAGCC

CCACTACGAAAGTGGCTTTAACCACACCCGACCCCACGAAAGCTGTGAAA

>397_Sample_ASV11

CACCGCGGTTATACGAGAGGCCCAAGTTGATAGATATCGGCGTAAAGAGTGGTTAAGGAA

ACCCCGTTACTAAAGCCGAACGCCCTCAGAACTGTCATACGTTCCCGAAGGTAAGAAGCC

CCACTACGAAAGTGGCTTTAACCACACCCGACCCCACGAAAGCTGTGAAA

>397_Sample_ASV12

CACCGCGGTTATACGAGAGGCCCCAGTTGATATTAGCGGCGTAAAGGGTGGTTAAGGACT

TTAATAACTAAAGCCAAACACTTCCCCGGCTGTTGTACGTAGACCGGAAGCTTGAAGCCC

AGACACGAAAGTAGCTTTATTTCCCCTGACCCCACGAAAGCTGAGAAA

>397_Sample_ASV13

CACCGCGGTTATACGAGAGGCCCCAGTTGATATTAGCGGCGTAAAGGGTGGTTAAGGACT

TTAATAACTAAAGCTAAACACTTCCCCGGCTGTTGTACGTAGACCGGAAGCTTGAAGCCC

AGACACGAAAGTAGCTTTATTCCCCCTGACCCCACGAAAGCTGAGAAA

>397_Sample_ASV14

CACCGCGGTTATACGAGAGGCCCCAGTTGATATTAGCGGCGTAAAGGGTGGTTAAGGACT

TTAATAACTAAAGCTAAACACTTCCCCGGCTGTTGTACGTAGACCGGAAGCTTGAAGCCC

AGACACGAAAGTAGCTTTATTTCCCCTGACCCCACGAAAGCTGAGAAA

>397_Sample_ASV15

CACCGCGGTTATACGAAAGACCCCAGTTTATAGACACGGCGTAAAGGGTGGTTATAGGAC

AAACTAACATAGAGCCAAAGACCCCCTAAGCCGTCATACGCTCACGAGGGCTCGAAGCCC

CTATACGAAAGTAGCTCTACCAAAAAATGCTCGACTCCACGAAAGCTGGGACA

>397_Sample_ASV16

CACCGCGGTTATACGAGAGACCCTAGTTGATAAATACGGCGTAAAGAGTGGTTATGGGAA

AAACAAATAAAGTCAAACAACCTCTTAGCTGTTATACGCATTATGAGAGCATGAAGCCCC

CTCACGAAAGTAACTTTAATACCTCCTGACCCCACGAAAGCTAAGAAA

>397_Sample_ASV17

CACCGCGGTTATACGAGAGACCCTAATTGATAGGCACGGCGTAAAGAGTGGTTTAGGGTA

ACCCATAAATAAAGCTAAAGACCTTCTAAGCCGTCACACGCACACCGAAGGCACGAGACC

CCAACACGAAAGTAGCTTTACTTCTACCCCTGACGCCACGAAAGCTAAGAAA

>397_Sample_ASV18

CACCGCGGTTATACGAAAGACCCTAGTTGATGGATCACGGCGTAAAGGGTGGTTAAGGGA

TATAAAAATAAAGCTAAAAATCCTCTTAGCTGTCATACGCTTTCCGAAGACATGAGGCCC

AATTACGAAAGTAGCTTTAAACCACCCTGACACCACGAAAGCCAAGAAA

>397_Sample_ASV19

CACCGCGGTTATACGAAAGACCCCAGTTTATAGATGCGGCGTAAAGGGTGGTTAAGAGAA

AATCAAAATAAGGCCAAAGATCCCCTAAGCAGTCATACGCTCCACGAGGACACGAAACCC

AAATACGAAAGTTGCCTTAATAAACTTCACCTGATCCCACGAAAGCTGAAAAA

>397_Sample_ASV20

CACCGCGGTTATACGAAAGGCTCTAGTTTATAAATACGGCGTAAAGGGTGGTTAAGGAAA

AATTAAAATAATGCTAAAGACCCCCTAAGCAGTTATACGCTTCACGGAGGCACGAAACCC

AAATACGAAAGTTGCCTTAATAAACTTTACCTGACCCCACGAAAGCTGAAAAA

>397_Sample_ASV21

CACCGCGGTTATACGAGAGACTCTAGTTGATACCAACGGCGTAAAGAGTGGTTTGGGATG

CCCCTTAAAATAAAGCCAAAGACCTCCCAAGCCGTCAAACGCACCCCGGAGGCACGAAGC

CCCAACGCGAAAGCAACTTTACCTCCCTCCCGACGCCACGAAAGCTAAGAAA

>397_Sample_ASV22

CACCGCGGTTATACGAGAGACCCTAGTTGATAGCTACGGCGTAAAGCGTGGTTAGGAGAA

CAAGATAAATAAAGTCAAACAATCTCTCGGCCGTTATACGTTATCTGAGAATATGAAGTC

CTACCACGAAAGTAACTTTAATTTTTCTGACCCCACGAAAACTGAGGAA

>397_Sample_ASV23

CACCGCGGTCATACGATTAACCCAAGTTAATAGGCACACGGCGTAAAGCGTGTTTAAGCA

CTACGCCAAATAAAGTTAAACTCCAATTAAGCTGTAAAAAGCCATAATCAAGACGAAAAT

AAATAACGAAAGTAACTTTACAACCGCTGAAACACGATAGCTAAGATC

>397_Sample_ASV24

CACCGCGGTTATACGAGAGGCCCTAGTTGATAACTACCGGCGTAAAGAGTGGTTATGGAA

AAATATTTAATAAAGCCGAACACCCCCTCAGCCGTCATACGCACCTGGGAGCACGAAGAC

CTACTGCGAAAGCAGCTTTAATTATGCCTGACCCCACGACAGCTAAGAAA

>397_Sample_ASV25

CACCGCGGTTATACGAGAGGCCCAAGTTGATTATTACCGGCGTAAAGCGTGGTTAAGATT

TTTATAGGACTAAAGCCGAACCCCCTCAGAGCTGTTATACGCACCCGAAGGTTAGAAGTT

CAATCACGAAAGTGGCTTTACAACCCCGAACCCACGAAAGCTAAGATA

>397_Sample_ASV26

CACCGCGGTTATACGAGAGGCCCAAGTTGATACTCCACGGCGTAAAGAGTGGTTAGAAAA

GACCTACTACTAAAGCCGAACGCCTTCAAAGCTGTTATACGCATCCGAAGGTGAGAAGCC

CACCCACGAAAGTGGCTTTACAACCTTGACTCCACGAAAGCTATGACA

>397_Sample_ASV27

CACCGCGGTTATACGAGAGACCCCAGTTGATAAACACGGCGTAAAGAGTGGTTAAGGGCA

CAAAACAAATAAAGTCAAACAACCTCTTAGCTGTTATACGCATTATGAGGATATGAAGCC

CCCCTACGAAAGTAACTTTACCAACCCCTGACCCCACGAAAGCTGTGAAA

>397_Sample_ASV28

CACCGCGGTTATACGAGAGACCCCAGTTGATAAACACGGCGTAAAGAGTGGTTAAGGGCA

CAAAACAAATAAAGTCAAACAACCTCTTAGCTGTTATACGCATTATGAGGATATGAAGCC

CCCCTACGAAAGTAACTTTACCAACCCCTGACCCCACGAAAGCTAAGAAA

>397_Sample_ASV29

CACCGCGGTTATACGAAAGACCCTAGTTTATAGGCACGGCGTAAAGGGTGGTTAGGGGAT

AAACAAAATAAAGCCAAAGACCTTCTAAGTCGTCATACACTCATGAAGGCACGAAGCCCA

AATACGAAAGTAACTTTACAACTTAGACCCGACTCCACGAAAGCTGGGGGA

>397_Sample_ASV30

CACCGCGGTTATACGAGAGACCCTAATTAATAGATACGGCGTAAAGAGTGGTTTAGGACA

AAAATTAATAAAGCCGAAGTTTCTCCTAGCCGTTGTACGCACTCTGAGAACACGAAGCCC

CACTACGAAAGTAGCTTTACCTATTATTTCCTGACCCCACGAAAGCTAAGAAA

>397_Sample_ASV31

CACCGCGGTTATACGAAAGACCCTAGTTGATAGCTACGGCGTAAAGGGTGGTTAAGGTAT

ATAACAAATAAAGCTAAAGAGCCTCCAAGCCGTCGTACGCATTCTGAGAGCTCGAAACCC

TAACACGAAAGTAGCTTTAAAAAATAACACCTGACCCCACGAAAGCTAAGAAA

>397_Sample_ASV32

CACCGCGGTTATACGAAAGACCCTAGTTGATAGATCACGGCGTAAAGGGTGGTTAAGGAG

AACAAAAATAAAGCTAAAAATCTTCTAAGCTGTCATACGCTTTCCGAAGACATGAGACCC

AACCACGAAAGTAGCTTTAAACTTTCCTGACACCACGAAAGCCAAGAAA

>401_2_Sample_ASV1

CACCGCGGTTATACGAGAGGCCCAAGTTGACAGACATCGGCGTAAAGAGTGGTTAAGGAA

AACCTTCAACTAAAGTCGAACGCTCTCAAGACTGTCGTACGTTTCCGAGAGTATGAAGCC

CCACCACGAAAGTGACTTTACCTCACCTGACTCCACGAAAGCTGCGAAA

>401_Sample_ASV1

CACCGCGGTTATACGAAAGACCCTAGTTGATGGGTGCGGCGTAAAGGGTGGTTAGGGACT

ACAAAAAATAGAGCTAAAGATCCTCTAGGTCGTCATACACATCACGAGGAAACGAAGCCC

AAATACGAAAGTAGCTCTAAATATAATCCTGACGCCACGAAAGCCAAGAAA

>401_2_Sample_ASV2

CACCGCGGTTATACGAAAGACCCTAGTTGATGGGTGCGGCGTAAAGGGTGGTTAGGGACT

ACAAAAAATAGAGCTAAAGATCCTCTAGGTCGTCATACACATCACGAGGAAACGAAGCCC

AAATACGAAAGTAGCTCTAAATATAATCCTGACGCCACGAAAGCCAAGAAA

>401_2_Sample_ASV3

CACCGCGGTTATACGAAAGACCCTAGTTGATAACTACGGCATAAAGGGTGGTTAGGGAGT

TTAAAAAATAAAGCCAAAGCTCTTCTAAGCTGTCATACGCTCTCTGAAAGAACGAGGCCC

AAACACGAAAGTAGCTTTAAATAAATAACCTGATACCACGAAAGCCAAGAAA

>401_2_Sample_ASV4

CACCGCGGTTATACGAGAGACCCTAGTTGATTAAAGCGGCGTAAAGAGTGGTTATGGAAC

TATTCTTTTAAAGCAGAAAACCTCTCAAACTGTTATACGCACCCAGAGGTCTGAATCCCT

CACACGAAAGTGACTTTATTTATGCCTACCAGAACCCACGAAAGCTGGGACA

>401_Sample_ASV2

CACCGCGGTTATACGAGAGACCCTAGTTGATTAAAGCGGCGTAAAGAGTGGTTATGGAAC

TATTCTTTTAAAGCAGAAAACCTCTCAAACTGTTATACGCACCCAGAGGTCTGAATCCCT

CACACGAAAGTGACTTTATTTATGCCTACCAGAACCCACGAAAGCTGGGACA

>401_Sample_ASV3

CACCGCGGTTATACGAGAGACCCTAGTTGATAAATACGGCGTAAAGAGTGGTTATGGAAC

TATTCTTTTAAAGCAGAAAACCTCTCAAACTGTTATACGCACCCAGAGGTCTGAATCCCT

CACACGAAAGTGACTTTATTTATGCCTACCAGAACCCACGAAAGCTGGGACA

>401_Sample_ASV4

CACCGCGGTTATACGAAAGACCCTAGTTTATAGGTACGGCGTAAAGGGTGGTTAGGGGAC

AAACAAAATAAAGCCAAAGACCTCCTAAGCCGTCATACGCTCACGGAAGCACGAAGCCCA

AACACGAAAGTAGCTTTACCGAACATGCCCGACTCCACGAAAGCTGGGGAA

>401_Sample_ASV5

CACCGCGGTTATACGAGAGACCCTAGTTGATATACACGGCGTAAAGAGTGGTTTGGGGTA

TATAATAAATAAAGCCAAAGACCTTCTAGGCCGTTGAACGCACCACGAAGGAGCGAAGTC

CCAATACGAAAGTAGCTTTATAAAACCTGACGCCACGAAAGCTAAGAAA

>401_Sample_ASV6

CACCGCGGTTATACGAGAGGCCCAAGTTGACAGACACCGGCGTAAAGAGTGGCTAGGGAA

AAATTACTACTAAAGCCGAACACCTTCAGAACTGTCATACGTTCCCGAAGATAAGAAGCC

CCACCACGAAAGTGACTTTATATTACCCGACCCCACGAAAGCTGTGAAA

>401_Sample_ASV7

CACCGCGGTTATACGAGAGGCCCAAGTTGACAGACACCGGCGTAAAGAGTGGCTAGGGAA

AAATTACTACTAAAGCCGAACACCTTCAGAACTGTCATACGTTCCCGAAGATAAGAAGCC

CCACCACGAAAGTGGCTTTATATTACCCGACCCCACGAAAGCTGTGAAA

>401_Sample_ASV8

CACCGCGGTTATACGAGAGGCCCAAGTTGACAGACACCGGCGTAAAGAGTGGCTAGGGAA

AAATTACTACTAAAGCCGAACACCTTCAGAACTGTCATACGTTCCCGAAGATAAGAAGCC

CCACCACGAAAGTGGCTTTATATTACCCGACCCCACGAAAGCTAAGAAA

>401_2_Sample_ASV5

CACCGCGGTTATACGAGAGGCCCAAGTTGACAGACACCGGCGTAAAGAGTGGCTAGGGAA

AAATTACTACTAAAGCCGAACACCTTCAGAACTGTCATACGTTCCCGAAGATAAGAAGCC

CCACCACGAAAGTGGCTTTATATTACCCGACCCCACGAAAGCTGTGAAA

>401_2_Sample_ASV6

CACCGCGATTATACGAGAGGCCCAAGTTGACAGACATCGGCGTAAAGAGTGGTTAAGGAA

AAATTACTACTAAAGCCGAACACCTTCAGAACTGTCATACGTTCCCGAAGATAAGAAGCC

CCACCACGAAAGTGGCTTTATATTACCCGACCCCACGAAAGCTGTGAAA

>401_Sample_ASV9

CACCGCGGTTATACGAGAGGCCCAAGTTGACAGACACCGGCGTAAAGAGTGGCTAAGGAA

AAATTTCTACTAAAGCCGAACACCTTCAGAACTGTCATACGTTCCCGAAGAAAAGAAGCC

CCACCACGAAAGTGGCTTTATATTACCCGACCCCACGAAAGCTGTGAAA

>401_2_Sample_ASV7

CACCGCGGTTATACGAGAGGCCCAAGTTGACAGACACCGGCGTAAAGAGTGGCTAAGGAA

AAATTTCTACTAAAGCCGAACACCTTCAGAACTGTCATACGTTCCCGAAGAAAAGAAGCC

CCACCACGAAAGTGGCTTTATATTACCCGACCCCACGAAAGCTGTGAAA

>401_2_Sample_ASV8

CACCGCGGTTATACGAAAGACCCTAGTTTATAGAAACGGCGTAAAGGGTGGTTAAGGGAC

AAACAAAATAAGGCCAAAGACTTCCTAAGCCGTCATACGCTCCACGAAAGCACGAAGCCC

AAACACGAAAGTAGCCTTAATAACTTCACCTGACCCCACGAAAGCTGGGAGA

>401_Sample_ASV10

CACCGCGGTTATACGAGAGGCCCAAGTTGATAAGCCCGGCGTAAAGAGTGGTTATGGAGA

ATAAAACACTAAAGCTAAAGACCCCCTAGGCTGTTATACGCACCTGGCGGCTCGAACCAC

CTATACGAAAGTAGCTTTACCCCCCTTCCACCAGAATCCACGACAGCTGGGGCA

>401_2_Sample_ASV9

CACCGCGGTTATACGAGAGGCCCAAGTTGATAAGCCCGGCGTAAAGAGTGGTTATGGAGA

ATAAAACACTAAAGCTAAAGACCCCCTAGGCTGTTATACGCACCTGGCGGCTCGAACCAC

CTATACGAAAGTAGCTTTACCCCCCTTCCACCAGAATCCACGACAGCTGGGGCA

>401_2_Sample_ASV10

CACCGCGGTTATACGAGAGGCCCAAGTTGACAGACATCGGCGTAAAGAGTGGTTAAGGAA

ACCCCATTACTAAAGCCGAACGCCCTCAGAACTGTCATACGTTCCCGAAGGTAAGAAGCC

CCACTACGAAAGTGGCTTTAACCACACCCGACCCCACGAAAGCTGTGAAA

>401_Sample_ASV11

CACCGCGGTTATACGAGAGGCCCAAGTTGACAGACATCGGCGTAAAGAGTGGTTAAGGAA

ACCCCATTACTAAAGCCGAACGCCCTCAGAACTGTCATACGTTCCCGAAGGTAAGAAGCC

CCACTACGAAAGTGGCTTTAACCACACCCGACCCCACGAAAGCTAAGAAA

>401_Sample_ASV12

CACCGCGGTTATACGAGAGGCCCAAGTTGACAGACATCGGCGTAAAGAGTGGTTAAGGAA

ACCCCATTACTAAAGCCGAACGCCCTCAGAACTGTCATACGTTCCCGAAGGTAAGAAGCC

CCACTACGAAAGTGGCTTTAACCACACCCGACCCCACGAAAGCTGTGAAA

>401_Sample_ASV13

CACCGCGGTTATACGAGAGGCCCAAGTTGATAGATATCGGCGTAAAGAGTGGTTAAGGAA

ACCCCGTTACTAAAGCCGAACGCCCTCAGAACTGTCATACGTTCCCGAAGGTAAGAAGCC

CCACTACGAAAGTGGCTTTAACCACACCCGACCCCACGAAAGCTGTGAAA

>401_2_Sample_ASV11

CACCGCGGTTATACGAGAGGCCCAAGTTGACAGACACCGGCGTAAAGAGTGGTTAAGGAA

ATTCATCAACTAAAGCCGAATGCCCTCAGGACTGTTATACGTTTGCGAAGGTAAGAAGCC

CTACTACGAAAGTGGCTTTATTCCACCTGATTCCACGAAAGCTGCGGAA

>401_2_Sample_ASV12

CACCGCGGTTATACGAGAGGCCCCAGTTGATATTAGCGGCGTAAAGGGTGGTTAAGGACT

TTAATAACTAAAGCTAAACACTTCCCCGGCTGTTGTACGTAGACCGGAAGCTTGAAGCCC

AGACACGAAAGTAGCTTTATTCCCCCTGACCCCACGAAAGCTGAGAAA

>401_Sample_ASV14

CACCGCGGTTATACGAGAAACCCTAGTTGACAAATACGGCGTAAAGAGTGGTTAAGGGGT

ATCAAAAATAAAGTCAAACATTTTCCTAGTTGTTATAAGCATTTCGAGAACATGAAGCCC

AACCACGAAAGTAACTTTACCTTCACCTGACCCCACGAAAGCTAAGAAA

>401_2_Sample_ASV13

CACCGCGGTTATACGAGAAACCCTAGTTGACAAATACGGCGTAAAGAGTGGTTAAGGGGT

ATCAAAAATAAAGTCAAACATTTTCCTAGTTGTTATAAGCATTTCGAGAACATGAAGCCC

AACCACGAAAGTAACTTTACCTTCACCTGACCCCACGAAAGCTAAGAAA

>401_2_Sample_ASV14

CACCGCGGTTATACGAGAGACCCTAGTTGATAAATACGGCGTAAAGAGTGGTTATGGGAA

AAACAAATAAAGTCAAACAACCTCTTAGCTGTTATACGCATTATGAGAGCATGAAGCCCC

CTCACGAAAGTAACTTTAATACCTCCTGACCCCACGAAAGCTAAGAAA

>401_2_Sample_ASV15

CACCGCGGTTATACGAGAGACCCTAGTTGATATATACGGCGTAAAGAGTGGTTATGGGAA

AAACAAATAAAGTCAAACAACCTCTTAGCTGTTATACGCATTATGAGAGCATGAAGCCCC

CTCACGAAAGTAACTTTAATACCTCCTGACCCCACGAAAGCTAAGAAA

>401_Sample_ASV15

CACCGCGGTTATACGAGAGACCCTAGTTGATAAATACGGCGTAAAGAGTGGTTATGGGAA

AAACAAATAAAGTCAAACAACCTCTTAGCTGTTATACGCATTATGAGAGCATGAAGCCCC

CTCACGAAAGTAACTTTAATACCTCCTGACCCCACGAAAGCTAAGAAA

>401_Sample_ASV16

AACCGCGGTTATACGAGAGACCCTAATTGATAGCTCACGGCGTAAAGGGTGGTTTGGGGA

AACCTCAAAATAAAGCTAAAGACCTTCTAAGCCGTTACACGCATACCGAAGGCACGAGGC

CCTAACACGAAAGTAGCTTTACTATTACCCCTGACGCCACGAAAGCTAAGAAA

>401_2_Sample_ASV16

CACCGCGGTTATACGAGAGACCCTAATTGATAGGTACGGCGTAAAGAGTGGTTTGGGATA

ACCCAAAAATAAAGCTAAAGACCTTCTAAGCCGTCACACGCATACCGAAGGCACGAGGCC

CCAACACGAAAGTAGCTTTACTATTACCCCGACGCCACGAAAGCTAAGAAA

>401_Sample_ASV17

CACCGCGGTTATACGAAAGACCCTAGTTGATGGATCACGGCGTAAAGGGTGGTTAAGGGA

TATAAAAATAAAGCTAAAAATCCTCTTAGCTGTCATACGCTTTCCGAAGACATGAGGCCC

AATTACGAAAGTAGCTTTAAACCACCCTGACACCACGAAAGCCAAGAAA

>401_2_Sample_ASV17

CACCGCGGTTATACGAGAGGCCCTAGTTGATAACTACCGGCGTAAAGAGTGGTTATGGAA

AAATATTTAATAAAGCCGAACACCCCCTCAGCCGTCATACGCACCTGGGAGCACGAAGAC

CTACTGCGAAAGCAGCTTTAATTATGCCTGACCCCACGACAGCTAAGAAA

>401_2_Sample_ASV18

CACCGCGGTTATACGAGAGACCCTAATTGATAGGCGCGGCGTAAAGAGTGGTTTAGGACT

AAAACTAATAAAGCTAAAGATCCTCCCAGCCGTCGCACGCATTCTGAGGACACGAAACTC

AACCACGAAAGTAGCTTTAACATACTCTCCTGACCCCACGAAAGCTAAGAAA

>401_2_Sample_ASV19

CACCGCGGTTATACGAGAGACCCTAGTTGATATATACGGCGTAAAGAGTGGTTTAGGACT

AAAACTAATAAAGCTAAAGATCCTCCCAGCCGTCGCACGCATTCTGAGGACACGAAACTC

AACCACGAAAGTAGCTTTAACATACTCTCCTGACCCCACGAAAGCTAAGAAA

>401_2_Sample_ASV20

CACCGCGGTTATACGAGAGGCCCAAGTTGATACTCCACGGCGTAAAGAGTGGTTAGAAAA

GACCTACTACTAAAGCCGAACGCCTTCAAAGCTGTTATACGCATCCGAAGGTGAGAAGCC

CACCCACGAAAGTGGCTTTACAACCTTGACTCCACGAAAGCTATGACA

>401_Sample_ASV18

CACCGCGGTTATACGAGAGGCCCAAGTTGATACTCCACGGCGTAAAGAGTGGTTAGAAAA

GACCTACTACTAAAGCCGAACGCCTTCAAAGCTGTTATACGCATCCGAAGGTGAGAAGCC

CACCCACGAAAGTGGCTTTACAACCTTGACTCCACGAAAGCTATGACA

>401_Sample_ASV19

CACCGCGGTTATACGAGAGACCCCAGTTGATAAACACGGCGTAAAGAGTGGTTAAGGGCA

CAAAACAAATAAAGTCAAACAACCTCTTAGCTGTTATACGCATTATGAGGATATGAAGCC

CCCCTACGAAAGTAACTTTACCAACCCCTGACCCCACGAAAGCTAAGAAA

>401_2_Sample_ASV21

CACCGCGGTTATACGAGAGACCCCAGTTGATAAACACGGCGTAAAGAGTGGTTAAGGGCA

CAAAACAAATAAAGTCAAACAACCTCTTAGCTGTTATACGCATTATGAGGATATGAAGCC

CCCCTACGAAAGTAACTTTACCAACCCCTGACCCCACGAAAGCTAAGAAA

>401_Sample_ASV20

CACCGCGGTTATACGAAAGACCCTAGTTTATAGGCACGGCGTAAAGGGTGGTTAGGGGAT

AAACAAAATAAAGCCAAAGACCTTCTAAGTCGTCATACACTCATGAAGGCACGAAGCCCA

AATACGAAAGTAACTTTACAACTTAGACCCGACTCCACGAAAGCTGGGGGA

>401_Sample_ASV21

CACCGCGGTTATACGAGAGACCCTAATTAATAGATACGGCGTAAAGAGTGGTTTAGGACA

AAAATTAATAAAGCCGAAGTTTCTCCTAGCCGTTGTACGCACTCTGAGAACACGAAGCCC

CACTACGAAAGTAGCTTTACCTATTATTTCCTGACCCCACGAAAGCTAAGAAA

>401_2_Sample_ASV22

CACCGCGGTTATACGAGAGGCCCAAGTTGATAGACATCGGCGTAAAGAGTGGTTAAGGAA

GTCTCAAAACTAAAGCCGAATGCCTTCAAGACTGTTATACGTTTCCGAAGGTAAGAAGCC

CCACTACGAAAGTGGCTTTACTCCACCTGACTCCACGAAAGCTGTGAAA

>401_Sample_ASV22

CACCGCGGTTATACGAGAGGCCCAAGTTGATAGACATCGGCGTAAAGAGTGGTTAAGGAA

GTCTCAAAACTAAAGCCGAATGCCTTCAAGACTGTTATACGTTTCCGAAGGTAAGAAGCC

CCACTACGAAAGTGGCTTTATTCCACCTGACCCCACGAAAGCTGTGAAA

>401_Sample_ASV23

CACCGCGGTTATACGAGAGGCCCAAGTTGATAGACATCGGCGTAAAGAGTGGTTAAGGAA

GTCTCAAAACTAAAGCCGAATGCCTTCAAGACTGTTATACGTTTCCGAAGGTAAGAAGCC

CCACTACGAAAGTGGCTTTACTCCACCTGACTCCACGAAAGCTGTGAAA

>401_2_Sample_ASV23

CACCGCGGTTATACGAGAGACTCCAGTTGATAGCTACGGCGTAAAGAGTGGTTTGGGGCC

CGCCCAAAATAAAGCCAAAGACCTCCCAAGCCGTCAAACGCACCCCGGAGGCACGAAGTC

CTAACGCGAAAGCAACTTTACCTTCCCCGACGCCACGAAAGCTAAGAAA

>401_2_Sample_ASV24

CACCGCGGTTATACGAGAGACTCCAGTTGATAGCTACGGCGTAAAGAGTGGTTTGGGGCC

CGCCCAAAATAAAGCCAAAGACCTCCCAAGCCGTCAAACGCACCCCGGAGGCACGAAGTC

CTAACGCGAAAGCAACTTTACCTTCCCCGACCCCACGAAAGCTGTGAAA

>401_2_Sample_ASV25

CACCGCGGTTATACGAAAGACCCTAGTTGATAGATCACGGCGTAAAGGGTGGTTAAGGAG

AACAAAAATAAAGCTAAAAATCCTCTAAGCTGTCATACGCTTTCCGAAGACATGAGACCC

AACCACGAAAGTAACTTTAAACTTTCCTGACACCACGAAAGCCAAGAAA

>405_Sample_ASV1

CACCGCGGTTATACGAAAGACCCTAGTTGATGGGTGCGGCGTAAAGGGTGGTTAGGGACT

ACAAAAAATAGAGCTAAAGATCCTCTAGGTCGTCATACACATCACGAGGAAACGAAGCCC

AAATACGAAAGTAGCTCTAAATATAATCCTGACGCCACGAAAGCCAAGAAA

>405_Sample_ASV2

CACCGCGGTTATACGAGAGACCCTAGTTGATTAAAGCGGCGTAAAGAGTGGTTATGGAAC

TATTCTTTTAAAGCAGAAAACCTCTCAAACTGTTATACGCACCCAGAGGTCTGAATCCCT

CACACGAAAGTGACTTTATTTATGCCTACCAGAACCCACGAAAGCTGGGACA

>405_Sample_ASV3

CACCGCGGTTATACGAAAGACCCTAGTTTATAGGTACGGCGTAAAGGGTGGTTAGGGGAC

AAACAAAATAAAGCCAAAGACCTCCTAAGCCGTCATACGCTCACGGAAGCACGAAGCCCA

AACACGAAAGTAGCTTTACCGAACATGCCCGACTCCACGAAAGCTGGGGAA

>405_Sample_ASV4

CACCGCGGTTATACGAAAGACCCTAGTTGATAGCCACGGCGTAAAGGGTGGTTAAGGAAC

CTAAAAAATAAAGCTAAAGACCCTCTAGGCCGTCATACGCATTACGAGGCAAACGAAACC

CCAACACGAAAGTAGCTTTAGACAAATATCCTGATGCCACGAAAGCCAAGAAA

>405_Sample_ASV5

CACCGCGGTTATACGAGAGACCCTAGTTGATATACACGGCGTAAAGAGTGGTTTGGGGTA

TATAATAAATAAAGCCAAAGACCTTCTAGGCCGTTGAACGCACCACGAAGGAGCGAAGTC

CCAATACGAAAGTAGCTTTATAAAACCTGACGCCACGAAAGCTAAGAAA

>405_Sample_ASV6

CACCGCGGTTATACGAGAGGCCCAAGTTGACAGACACCGGCGTAAAGAGTGGCTAGGGAA

AAATTACTACTAAAGCCGAACACCTTCAGAACTGTCATACGTTCCCGAAGATAAGAAGCC

CCACCACGAAAGTGGCTTTATATTACCCGACCCCACGAAAGCCAAGAAA

>405_Sample_ASV7

CACCGCGGTTATACGAGAGGCCCAAGTTGACAGACACCGGCGTAAAGAGTGGCTAGGGAA

AAATTACTACTAAAGCCGAACACCTTCAGAACTGTCATACGTTCCCGAAGATAAGAAGCC

CCACCACGAAAGTGGCTTTATATTACCCGACCCCACGAAAGCTGTGAAA

>405_Sample_ASV8

CACCGCGGTTATACTAGAGTCCCAAGTTGACAGACACCGGCGTAAAGAGTGGCTAGGGAA

AAATTACTACTAAAGCCGAACACCTTCAGAACTGTCATACGTTCCCGAAGATAAGAAGCC

CCACCACGAAAGTGGCTTTATATTACCCGACCCCACGAAAGCTGTGAAA

>405_Sample_ASV9

CACCGCGGTTATACGAGAGGCCCAAGTTGACAGACACCGGCGTAAAGAGTGGCTAAGGAA

AAATTTCTACTAAAGCCGAACACCTTCAGAACTGTCATACGTTCCCGAAGAAAAGAAGCC

CCACCACGAAAGTGGCTTTATATTACCCGACCCCACGAAAGCTGTGAAA

>405_Sample_ASV10

CACCGCGGTTAAACGAGAGACCCTAGTTGATCATCACGGCGTAAAGAGTGGTTAGGGGAT

TATCATAAATAAAGCCAAAGACCTCCCAAGCTGTCGCACGCATTCCGGAGGCGCGAAGCC

CACACACGAAAGTAGCTTTAACTATTGACCCTGATGCCACGAAAGCTAAGAAA

>405_Sample_ASV11

CACCGCGGTTATACGAGAGGCCCAAGTTGATAAGCCCGGCGTAAAGAGTGGTTATGGAGA

ATAAAACACTAAAGCTAAAGACCCCCTAGGCTGTTATACGCACCTGGCGGCTCGAACCAC

CTATACGAAAGTAGCTTTACCCCCCTTCCACCAGAATCCACGACAGCTGGGGCA

>405_Sample_ASV12

CACCGCGGTTATACGAGAGGCCCAAGTTGACAGACATCGGCGTAAAGAGTGGTTAAGGAA

ACCCCATTACTAAAGCCGAACGCCCTCAGAACTGTCATACGTTCCCGAAGGTAAGAAGCC

CCACTACGAAAGTGGCTTTAACCACACCCGACCCCACGAAAGCTGTGAAA

>405_Sample_ASV13

CACCGCGGTCACACGATTAACCCAAGTCAATAGAAGCCGGCGTAAAGAGTGTTTTAGATC

ACCCCCTCCCCAATAAAGCTAAAACTCACCTGAGTTGTAAAAAACTCCAGTTGACACAAA

ATAGACTACGAAAGTGGCTTTAACATATCTGAACACACAATAGCTAAGACC

>405_Sample_ASV14

CACCGCGGTTATACGAGAGACCCCAGTTGATAGCTACGGCGTAAAGAGTGGTTAAGGACC

CCATTTAATAAAGCCAAAGACCCCCCAAGCCGTCATACGCACACGAGGGCGCGAAGCTCG

CACACGAAAGTAGCTCTAATTTACCCGACGCCACGAAAGCTAAGGAA

>405_Sample_ASV15

CACCGCGGTTATACGAGAAACCCTAGTTGACAAATACGGCGTAAAGAGTGGTTAAGGGGT

ATCAAAAATAAAGTCAAACATTTTCCTAGTTGTTATAAGCATTTCGAGAACATGAAGCCC

AACCACGAAAGTAACTTTACCTCCACCTGACCCCACGAAAGCTAAGAAA

>405_Sample_ASV16

CACCGCGGTTATACGAGAGACCCTAGTTGATAAATACGGCGTAAAGAGTGGTTATGGGAA

AAACAAATAAAGTCAAACAACCTCTTAGCTGTTATACGCATTATGAGAGCATGAAGCCCC

CTCACGAAAGTAACTTTAATACCTCCTGACCCCACGAAAGCTAAGAAA

>405_Sample_ASV17

CACCGCGGTTATACGAGAGACCCCAGTTGATAAACACGGCGTAAAGAGTGGTTAAGGGCA

CAAAACAAATAAAGTCAAACAACCTCTTAGCTGTTATACGCATTATGAGAGCATGAAGCC

CCCTCACGAAAGTAACTTTAATACCTCCTGACCCCACGAAAGCTAAGAAA

>405_Sample_ASV18

CACCGCGGTTATACGAGAGACCCTAATTGATAGACACGGCGTAAAGAGTGGTTTGGGAAA

GCCCAAAAATAAAGCTAAAGACCTTCTAAGCCGTCACACGCACGCCGAAGGCACGAAGCC

CTGACACGAAAGTAGCTTTACTATTACCCCCGACGCCACGAAAGCTAAGAAA

>405_Sample_ASV19

CACCGCGGTTATACGAAAGACCCTAGTTTATAGATGCGGCGTAAAGGGTGGTTAAGGAAA

ACCTAAAATAATGCTAAAGACTCTCAAAGCTGTTATACGCTTCATGAGCACACGAAATCC

AAATACGAAAGTTGCCTTAATAAACTTTGCCTGACCCCACGAAAGCTGAAAAA

>405_Sample_ASV20

CACCGCGGTTATACGAGAGACCCTAGTTGATAGCTACGGCGTAAAGCGTGGTTAGGAGAA

CAAGATAAATAAAGTCAAACAATCTCTCGGCCGTTATACGTTATCTGAGAATATGAAGTC

CTACCACGAAAGTAACTTTAATTTTTCTGACCCCACGAAAACTGAGGAA

>405_Sample_ASV21

CACCGCGGTTAAACGAGAGGCCCTAGTTGATAGTACAACGGCGTAAAGGGTGGTTAAGGA

TAGTGAAATAATAAAGTCGAATGGCCCTTTGGCTGTCATACGCTTCTAGGAGTCCGAAGC

CCAATATACGAAAGTAACTTTAAGAGCGCCCACCTGACCCCACGAAAACTGAGAAA

>405_Sample_ASV22

CACCGCGGTCATACGATTAACCCAAGTTAATAGGCACACGGCGTAAAGCGTGTTTAAGCA

CTACGCCAAATAAAGTTAAACTCCAATTAAGCTGTAAAAAGCCATAATCAAGACGAAAAT

AAATAACGAAAGTAACTTTACAACCGCTGAAACACGATAGCTAAGATC

>405_Sample_ASV23

CACCGCGGTTATACGAGAGGCCCTAGTTGATAACTACCGGCGTAAAGAGTGGTTATGGAA

AAATATTTAATAAAGCCGAACACCCCCTCAGCCGTCATACGCACCTGGGAGCACGAAGAC

CTACTGCGAAAGCAGCTTTAATTATGCCTGACCCCACGACAGCTAAGAAA

>405_Sample_ASV24

CACCGCGGTTATACGAAAGACCCTAGTTGATAGGCACGGCGTAAAGGGTGGTTAGGGATA

TATTAAAAATAAAGTTAAAGAGCCTCTAAGCTGTCGCACGCATCCCGAGAGCTCGAGACC

CAGACACGAAAGTAACTTTAAAATACCTCACCTGACCCCACGAAAGCTAAGAAA

>405_Sample_ASV25

CACCGCGGTTATACGAGAGGCCCAAGTTGATACTCCACGGCGTAAAGAGTGGTTAGAAAA

GACCTACTACTAAAGCCGAACGCCTTCAAAGCTGTTATACGCATCCGAAGGTGAGAAGCC

CACCCACGAAAGTGGCTTTACAACCTTGACTCCACGAAAGCTATGACA

>405_Sample_ASV26

CACCGCGGTTATACGAGAGACCCCAGTTGATAAACACGGCGTAAAGAGTGGTTAAGGGCA

CAAAACAAATAAAGTCAAACAACCTCTTAGCTGTTATACGCATTATGAGGATATGAAGCC

CCCCTACGAAAGTAACTTTACCAACCCCTGACCCCACGAAAGCTAAGAAA

>405_Sample_ASV27

CACCGCGGTTATACGAGAGACCCTAGTTGATATATACGGCGTAAAGAGTGGTTTGGAATA

ACCAAGTAATATAGCCAAAGACCTCCCAAGCCGTCACACGCACCCCGGAGGCACGAAGCC

CAAACACGAAAGTAGCTTTATTAACTTACCGACGCCACGAAAGCTAAGAAA

>405_Sample_ASV28

CACCGCGGTTATACGAAAGACCCTAGTTTATAGGCACGGCGTAAAGGGTGGTTAGGGGAT

AAACAAAATAAAGCCAAAGACCTTCTAAGTCGTCATACACTCATGAAGGCACGAAGCCCA

AATACGAAAGTAACTTTACAACTTAGACCCGACTCCACGAAAGCTGGGGGA

>405_Sample_ASV29

CACCGCGGTTATACGAGAGACCCTAATTAATAGCTACGGCGTAAAGAGTGGTTTAGGACA

AAAATTAATAAAGCCGAAGTTTCTCCTAGCCGTTGTACGCACTCTGAGAACACGAAGCCC

CACTACGAAAGTAGCTTTACCAATAATCTCCTGACCCCACGAAAGCTAAGAAA

>405_Sample_ASV30

CACCGCGGTTATACGAGAGACCCTAATTAATAGATACGGCGTAAAGAGTGGTTTAGGACA

AAAATTAATAAAGCCGAAGTTTCTCCTAGCCGTTGTACGCACTCTGAGAACACGAAGCCC

CACTACGAAAGTAGCTTTACCAATAATCTCCTGACCCCACGAAAGCTAAGAAA

>405_Sample_ASV31

CACCGCGGTTATACGAGAGACCCTAATTAATAGCTACGGCGTAAAGAGTGGTTTAGGACA

AAAATTAATAAAGCCGAAGTTTCTCCTAGCCGTTGTACGCACTCTGAGAACACGAAGCCC

CACTACGAAAGTAGCTTTACCTATTATTTCCTGACCCCACGAAAGCTAAGAAA

>405_Sample_ASV32

CACCGCGGTTATACGAGAGACCCTAATTAATAGCTACGGCGTAAAGAGTGGTTTAGGGTA

AAAATTAATAAAGCCGAAGTTTCTCCTAGCCGTTGTACGCACTCTGAGAACACGAAGCCC

CACTACGAAAGTAGCTTTACCTATTATTTCCTGACCCCACGAAAGCTAAGAAA

>405_Sample_ASV33

CACCGCGGTTATACGAGAGACCCTAATTAATAGATACGGCGTAAAGAGTGGTTTAGGACA

AAAATTAATAAAGCCGAAGTTTCTCCTAGCCGTTGTACGCACTCTGAGAACACGAAGCCC

CACTACGAAAGTAGCTTTACCTATTATTTCCTGACCCCACGAAAGCTAAGAAA

>405_Sample_ASV34

CACCGCGGTTATACGAGAGGCCCAAGTTGATAGACATCGGCGTAAAGAGTGGTTAAGGAA

GTCTCAAAACTAAAGCCGAATGCCTTCAAGACTGTTATACGTTTCCGAAGGTAAGAAGCC

CCACTACGAAAGTGGCTTTACTCCACCTGACTCCACGAAAGCTGTGAAA

>405_Sample_ASV35

CACCGCGGTTATACGAAAGACCCTAGTTGATAGCTACGGCGTAAAGGGTGGTTAAGGTAT

ATAACAAATAAAGCTAAAGAGCCTCCAAGCCGTCGTACGCATTCTGAGAGCTCGAAACCC

TAACACGAAAGTAGCTTTAAAAAATAACACCTGACCCCACGAAAGCTAAGAAA

>405_Sample_ASV36

CACCGCGGTTATACGAGAGACCCTAATTGATAGACTACGGCGTAAAGAGTGGTTTAGAAC

AAAAATTAATAAAGCCAAAGATCCTCCTGGCCGTCGCACGCATTTCGAGGATACGAAGCC

CCACTACGAAAGTAGCTTTACTACAACTTTTCTGACCCCACGAAAGCTAAGAAA

>405_Sample_ASV37

CACCGCGGTTATACGAAAGACCCTAGTTGATAGATCACGGCGTAAAGGGTGGTTAAGGAG

AACAAAAATAAAGCTAAAAATCCTCTAAGCTGTCATACGCTTTCCGAAGACATGAGACCC

AACCACGAAAGTAACTTTAAACTTTCCTGACACCACGAAAGCCAAGAAA

>418_Sample_ASV1

CACCGCGGTTATACGAAAGACCCTAGTTGATGGGTGCGGCGTAAAGGGTGGTTAGGGACT

ACAAAAAATAGAGCTAAAGATCCTCTAGGTCGTCATACACATCACGAGGAAACGAAGCCC

AAATACGAAAGTAGCTCTAAATATAATCCTGACGCCACGAAAGCCAAGAAA

>418_Sample_ASV2

CACCGCGGTTAAACGAGAGGCCCTAGTTGATGGTGCAACGGCGTAAAGGGTGGTTAAGGA

TAGCGAAATAATAAAGTCAAATGGCCCTCTGGCTGTCATACGCTTCTAGGAGTCCGAAGC

CCAGTATACGAAAGTAGCTTTAAGAAAGTCCACCTGACCCCACGAAAACTGAGGAA

>418_Sample_ASV3

CACCGCGGTTATACGAAAGACCCTAGTTGCTAGCCACGGCGTAAAGGGTGGTTAAGGACA

ACAACAAATAAAGCTAAAGATCCCCTAAGCCGTCATACGCATTCCGGGGGCACGAAGCCC

TAACACGAAAGTAGCTTTAAAAAATATACCTGACCCCACGAAAGCTAAGAAA

>418_Sample_ASV4

CACCGCGGTTATACGAGAGACCCTAGTTGATTAAAGCGGCGTAAAGAGTGGTTATGGAAC

TATTCTTTTAAAGCAGAAAACCTCTCAAACTGTTATACGCACCCAGAGGTCTGAATCCCT

CACACGAAAGTGACTTTATTTATGCCTACCAGAACCCACGAAAGCTGGGACA

>418_Sample_ASV5

CACCGCGGTTATACGAGAGACCCTAGTTGATATACACGGCGTAAAGAGTGGTTTGGGGTA

TATAATAAATAAAGCCAAAGACCTTCTAGGCCGTTGAACGCACCACGAAGGAGCGAAGTC

CCAATACGAAAGTAGCTTTATAAAACCTGACGCCACGAAAGCTAAGAAA

>418_Sample_ASV6

CACCGCGGTTATACGAGAGGCCCAAGTTGACAGACACCGGCGTAAAGAGTGGCTAGGGAA

AAATTACTACTAAAGCCGAACACCTTCAGAACTGTCATACGTTCCCGAAGATAAGAAGCC

CCACCACGAAAGTGACTTTATATTACCCGACCCCACGAAAGCTGTGAAA

>418_Sample_ASV7

CACCGCGGTTATACGAGAGGCCCAAGTTGACAGACACCGGCGTAAAGAGTGGCTAGGGAA

AAATTACTACTAAAGCCGAACACCTTCAGAACTGTCATACGTTCCCGAAGATAAGAAGCC

CCACCACGAAAGTGGCTTTATATTACCCGACCCCACGAAAGCTAAGAAA

>418_Sample_ASV8

CACCGCGGTTATACGAGAGGCCCAAGTTGACAGACACCGGCGTAAAGAGTGGCTAGGGAA

AAATTACTACTAAAGCCGAACACCTTCAGAACTGTCATACGTTCCCGAAGATAAGAAGCC

CCACCACGAAAGTGGCTTTATATTACCCGACCCCACGAAAGCTGTGAAA

>418_Sample_ASV9

CACCGCGGTTATACGAGAGGCCCAAGTTGACAGACACCGGCGTAAAGAGTGGCTAAGGAA

AAATTTCTACTAAAGCCGAACACCTTCAGAACTGTCATACGTTCCCGAAGAAAAGAAGCC

CCACCACGAAAGTGGCTTTATATTACCCGACCCCACGAAAGCTGTGAAA

>418_Sample_ASV10

CACCGCGGTTATACGAGAGGCTCAAGTTGACAGACATCGGCGTAAAGAGTGGTTAGGAAG

TTTTTGAAACTAAAGCCGAACGCCCTCAGAACTGTTATACGTACCCGAGGGTAAGAAGCC

CCACTACGAAAGTGGCTTTATATCTCCGACCCCACGAAAGCTGCGAAA

>418_Sample_ASV11

CACCGCGGTTATACGAGAGGCCCAAGTTGATAAGCCCGGCGTAAAGAGTGGTTATGGAGA

ATAAAACACTAAAGCTAAAGACCCCCTAGGCTGTTATACGCACCTGGCGGCTCGAACCAC

CTATACGAAAGTAGCTTTACCCCCCTTCCACCAGAATCCACGACAGCTGGGGCA

>418_Sample_ASV12

CACCGCGGTTATACGAGAGGCCCAAGTTGACAGACATCGGCGTAAAGAGTGGTTAAGGAA

ACCCCATTACTAAAGCCGAACGCCCTCAGAACTGTCATACGTTCCCGAAGGTAAGAAGCC

CCACTACGAAAGTGGCTTTAACCACACCCGACCCCACGAAAGCTGTGAAA

>418_Sample_ASV13

CACCGCGGTTAAACGAGAGGCCCTAGTTGATATTACAACGGCGTAAAGGGTGGTTAAGGA

AAGCAAGCAATAAAGCCGAATGGCCCTTTGGCTGTCATACGCTTCTAGGTGTCCGAAGCC

CAATATACGAAAGTAGCTTTAGAAAAGCCCACCTGACACCACGAAAGCTGAGGAA

>418_Sample_ASV14

CACCGCGGTTATACGAGAAACCCTAGTTGACAAATACGGCGTAAAGAGTGGTTAAGGGGT

ATCAAAAATAAAGTCAAACATTTTCCTAGTTGTTATAAGCATTTCGAGAACATGAAGCCC

AACCACGAAAGTAACTTTACCTTCACCTGACCCCACGAAAGCTAAGAAA

>418_Sample_ASV15

CACCGCGGTTATACGAGAGACCCTAGTTGATAAATACGGCGTAAAGAGTGGTTATGGGAA

AAACAAATAAAGTCAAACAACCTCTTAGCTGTTATACGCATTATGAGAGCATGAAGCCCC

CTCACGAAAGTAACTTTAATACCTCCTGACCCCACGAAAGCTAAGAAA

>418_Sample_ASV16

CACCGCGGTTATACGAGAGGCTCAAGTTGATAAACCCCGGCGTAAAGAGTGGTTAAGGAA

AAATAGAAACTAAAGCCGAATGCTTTCAAGACTGTTATACGTTCCCGAGAGTAAGAAGAC

CAATTACGAAAGTGGCTTTACTTTACCTGACCCCACGAAAGCTACGACA

>418_Sample_ASV17

AACCGCGGTTATACGAGAGACCCTAATTGATAGCTCACGGCGTAAAGGGTGGTTTGGGGA

AACCTCAAAATAAAGCTAAAGACCTTCTAAGCCGTTACACGCATACCGAAGGCACGAGGC

CCTAACACGAAAGTAGCTTTACTATTACCCCTGACGCCACGAAAGCTAAGAAA

>418_Sample_ASV18

CACCGCGGTTATACGAAAGACCCTAGTTGATGGATCACGGCGTAAAGGGTGGTTAAGGGA

TATAAAAATAAAGCTAAAAATCCTCTTAGCTGTCATACGCTTTCCGAAGACATGAGGCCC

AATTACGAAAGTAGCTTTAAACCACCCTGACACCACGAAAGCCAAGAAA

>418_Sample_ASV19

CACCGCGGTTATACGAGAGACCCTAATTGATAGGCGCGGCGTAAAGAGTGGTTTAGGACT

AAAACTAATAAAGCTAAAGATCCTCCCAGCCGTCGCACGCATTCTGAGGACACGAAACTC

AACCACGAAAGTAGCTTTAACATACTCTCCTGACCCCACGAAAGCTAAGAAA

>418_Sample_ASV20

CACCGCGGTTATACGAGAGGCCCAAGTTGATAGTCTTCGGCGTAAAGAGTGGTTAAAGAA

GCAATTAGACTAAGGCTAAACTCTTCCAAGGCTGTCATACGCACCCGGAAGCATGAAACC

CAACCACGAAAGTGGCCTTATTTACCTTGACCCCACGAAAGCTGTGAAA

>418_Sample_ASV21

CACCGCGGTTATACGAGAGACCCCAGTTGATAAACACGGCGTAAAGAGTGGTTAAGGGCA

CAAAACAAATAAAGTCAAACAACCTCTTAGCTGTTATACGCATTATGAGGATATGAAGCC

CCCCTACGAAAGTAACTTTACCAACCCCTGACCCCACGAAAGCTAAGAAA

>418_Sample_ASV22

CACCGCGGTTATACGAAAGACCCTAGTTTATAGGCACGGCGTAAAGGGTGGTTAGGGGAT

AAACAAAATAAAGCCAAAGACCTTCTAAGTCGTCATACACTCATGAAGGCACGAAGCCCA

AATACGAAAGTAACTTTACAACTTAGACCCGACTCCACGAAAGCTGGGGGA

>418_Sample_ASV23

CACCGCGGTTATACGAGAGACCCTAATTAATAGATACGGCGTAAAGAGTGGTTTAGGACA

AAAATTAATAAAGCCGAAGTTTCTCCTAGCCGTTGTACGCACTCTGAGAACACGAAGCCC

CACTACGAAAGTAGCTTTACCTATTATTTCCTGACCCCACGAAAGCTAAGAAA

>418_Sample_ASV24

CACCGCGGTTATACGAGAGGCCCAAGTTGATAGACATCGGCGTAAAGAGTGGTTAAGGAA

GTCTCAAAACTAAAGCCGAATGCCTTCAAGACTGTTATACGTTTCCGAAGGTAAGAAGCC

CCACTACGAAAGTGGCTTTACTCCACCTGACTCCACGAAAGCTGTGAAA

>418_Sample_ASV25

CACCGCGGTTATACGAGAGGCCCAAGTTGATAGACATCGGCGTAAAGAGTGGTTAAGGAA

GTCTCAAAACTAAAGCCGAATGCCTTCAAGACTGTTATACGTTTCCGAAGGTAAGAAGCC

CCACTACGAAAGTGGCTTTACTCCACCTGACTCCACGAAAGCTAAGAAA

>418_Sample_ASV26

CACCGCGGTTATACGAGAGGCCCAAGTTGACAAACTCCGGCGTAAAGCGTGGTTAAGGCA

CACTCAAAAACTAAAGCCGAATATCTTCAGAGCAGTCATACGCATCCGAAGACACGAAGC

CCCACCACGAAAGTGGCTTTATGACACCTGACCCCACGAAAGCTAGGACA

>418_Sample_ASV27

CACCGCGGTTATACGAAAGACCCTAGTTGATAGCTACGGCGTAAAGGGTGGTTAAGGTAT

ATAACAAATAAAGCTAAAGAGCCTCCAAGCCGTCGTACGCATTCTGAGAGCTCGAAACCC

TAACACGAAAGTAGCTTTAAAAAATAACACCTGACCCCACGAAAGCTAAGAAA

>418_Sample_ASV28

CACCGCGGTTATACGAAAGACCCTAGTTGATAGATCACGGCGTAAAGGGTGGTTAAGGAG

AACAAAAATAAAGCTAAAAATCCTCTAAGCTGTCATACGCTTTCCGAAGACATGAGACCC

AACCACGAAAGTAACTTTAAACTTTCCTGACACCACGAAAGCCAAGAAA

>428_Sample_ASV1

CACCGCGGTTATACGAGAGGCCCAAGTTGACAGACATCGGCGTAAAGAGTGGTTAAGGAA

AACCTTCAACTAAAGTCGAACGCTCTCAAGACTGTCGTACGTTTCCGAGAGTATGAAGCC

CCACCACGAAAGTGACTTTACCTCACCTGACTCCACGAAAGCTGCGAAA

>428_Sample_ASV2

CACCGCGGTTATACGAAAGACCCTAGTTGATGGGTGCGGCGTAAAGGGTGGTTAGGGACT

ACAAAAAATAGAGCTAAAGATCCTCTAGGTCGTCATACACATCACGAGGAAACGAAGCCC

AAATACGAAAGTAGCTCTAAATATAATCCTGACGCCACGAAAGCCAAGAAA

>428_Sample_ASV3

CACCGCGGTTATACGAAAGACCCTAGTTGATAACTACGGCATAAAGGGTGGTTAGGGAGT

TTAAAAAATAAAGCCAAAGCTCTTCTAAGCTGTCATACGCTCTCTGAAAGAACGAGGCCC

AAACACGAAAGTAGCTTTAAATAAATAACCTGATACCACGAAAGCCAAGAAA

>428_Sample_ASV4

CACCGCGGTTATACGAGAGACCCTAGTTGATTAAAGCGGCGTAAAGAGTGGTTATGGAAC

TATTCTTTTAAAGCAGAAAACCTCTCAAACTGTTATACGCACCCAGAGGTCTGAATCCCT

CACACGAAAGTGACTTTATTTATGCCTACCAGAACCCACGAAAGCTGGGACA

>428_Sample_ASV5

CACCGCGGTTATACGAAAGACCCTAGTTTATAGGTACGGCGTAAAGGGTGGTTAGGGGAC

AAACAAAATAAAGCCAAAGACCTCCTAAGCCGTCATACGCTCACGGAAGCACGAAGCCCA

AACACGAAAGTAGCTTTACCGAACATGCCCGACTCCACGAAAGCTGGGGAA

>428_Sample_ASV6

CACCGCGGTTATACGAGAGGCCCAAGTTGACAGACACCGGCGTAAAGAGTGGCTAGGGAA

AAATTACTACTAAAGCCGAACACCTTCAGAACTGTCATACGTTCCCGAAGATAAGAAGCC

CCACCACGAAAGTGGCTTTATATTACCCGACCCCACGAAAGCTGTGAAA

>428_Sample_ASV7

CACCGCGGTTATACGAGAGGCCCAAGTTGACAGACACCGGCGTAAAGAGTGGCTAAGGAA

AAATTTCTACTAAAGCCGAACACCTTCAGAACTGTCATACGTTCCCGAAGAAAAGAAGCC

CCACCACGAAAGTGGCTTTATATTACCCGACCCCACGAAAGCTGTGAAA

>428_Sample_ASV8

CACCGCGGTTATACGAAAGACCCTAGTTTATAGAAACGGCGTAAAGGGTGGTTAAGGGAC

AAACAAAATAAGGCCAAAGACTTCCTAAGCCGTCATACGCTCCACGAAAGCACGAAGCCC

AAACACGAAAGTAGCCTTAATAACTTCACCTGACCCCACGAAAGCTGGGAGA

>428_Sample_ASV9

CACCGCGGTTAAACGAGAGACCCTAGTTGATCATCACGGCGTAAAGAGTGGTTAGGGGAT

TATCATAAATAAAGCCAAAGACCTCCCAAGCTGTCGCACGCATTCCGGAGGCGCGAAGCC

CACACACGAAAGTAGCTTTAACTATTGACCCTGATGCCACGAAAGCTAAGAAA

>428_Sample_ASV10

CACCGCGGTTATACGAGAGACCCTAGTTGATATATACGGCGTAAAGAGTGGTTTGGGATA

TTTCTCCAAAATAAAGTCGAAGATCTCCCAAGCCGTCACACGCATCCCGGAGACACGAAG

CCCTGATACGAAGGTAGCTTTATTAACACCCCGACGCCACGAAAGCTAAGAAA

>428_Sample_ASV11

CACCGCGGTTATACGAGAGACCCTAGTTGATATATACGGCGTAAAGAGTGGTTTGGGATA

TTTCTCCAAAATAAAGCCGAAGATCTCCCAAGCCGTCACACGCATCCCGGAGACACGAAG

CCCTGATACGAAGGTAGCTTTATTAACACCCCGACGCCACGAAAGCTAAGAAA

>428_Sample_ASV12

CACCGCGGTTATACGAGAGGCCCAAGTTGATAAGCCCGGCGTAAAGAGTGGTTATGGAGA

ATAAAACACTAAAGCTAAAGACCCCCTAGGCTGTTATACGCACCTGGCGGCTCGAACCAC

CTATACGAAAGTAGCTTTACCCCCCTTCCACCAGAATCCACGACAGCTGGGGCA

>428_Sample_ASV13

CACCGCGGTTATACGAGAGGCCCAAGTTGACAGACATCGGCGTAAAGAGTGGTTAAGGAA

ACCCCATTACTAAAGCCGAACGCCCTCAGAACTGTCATACGTTCCCGAAGGTAAGAAGCC

CCACTACGAAAGTGGCTTTAACCACACCCGACCCCACGACAGCTAAGAAA

>428_Sample_ASV14

CACCGCGGTTATACGAGAGGCCCAAGTTGACAGACATCGGCGTAAAGAGTGGTTAAGGAA

ACCCCATTACTAAAGCCGAACGCCCTCAGAACTGTCATACGTTCCCGAAGGTAAGAAGCC

CCACTACGAAAGTGGCTTTAACCACACCCGACCCCACGAAAGCTAAGAAA

>428_Sample_ASV15

CACCGCGGTTATACGAGAGGCCCAAGTTGACAGACATCGGCGTAAAGAGTGGTTAAGGAA

ACCCCATTACTAAAGCCGAACGCCCTCAGAACTGTCATACGTTCCCGAAGGTAAGAAGCC

CCACTACGAAAGTGGCTTTAACCACACCCGACCCCACGAAAGCTGTGAAA

>428_Sample_ASV16

CACCGCGGTTATACGAGAGGCCCAAGTTGACAGACACCGGCGTAAAGAGTGGTTAAGGAA

ATTCATCAACTAAAGCCGAATGCCCTCAGGACTGTTATACGTTTGCGAAGGTAAGAAGCC

CTACTACGAAAGTGGCTTTATTCCACCTGATTCCACGAAAGCTGCGGAA

>428_Sample_ASV17

CACCGCGGTTATACGAGAGGCCCCAGTTGATATTAGCGGCGTAAAGGGTGGTTAAGGACT

TTAATAACTAAAGCTAAACACTTCCCCGGCTGTTGTACGTAGACCGGAAGCTTGAAGCCC

AGACACGAAAGTAGCTTTATTCCCCCTGACCCCACGAAAGCTGAGAAA

>428_Sample_ASV18

CACCGCGGTTATACGAGAAACCCTAGTTGACAAATACGGCGTAAAGAGTGGTTAAGGGGT

ATCAAAAATAAAGTCAAACATTTTCCTAGTTGTTATAAGCATTTCGAGAACATGAAGCCC

AACCACGAAAGTAACTTTACCTTCACCTGACCCCACGAAAGCTAAGAAA

>428_Sample_ASV19

CACCGCGGTTATACGAGAGACCCTAGTTGATAAATACGGCGTAAAGAGTGGTTATGGGAA

AAACAAATAAAGTCAAACAACCTCTTAGCTGTTATACGCATTATGAGAGCATGAAGCCCC

CTCACGAAAGTAACTTTAATACCTCCTGACCCCACGAAAGCTAAGAAA

>428_Sample_ASV20

CACCGCGGTTATACGAGAGACCCTAATTGATAGGTACGGCGTAAAGAGTGGTTTGGGATA

ACCCAAAAATAAAGCTAAAGACCTTCTAAGCCGTCACACGCATACCGAAGGCACGAGGCC

CCAACACGAAAGTAGCTTTACTATTACCCCGACGCCACGAAAGCTAAGAAA

>428_Sample_ASV21

CACCGCGGTTATACGAGAGGCCCAAGTTGACAGGTACCGGCGTAAAGAGTGGTTAAGAAA

AAATATTAAACTAAAGCCGAACACCTTCAGAGCTGTCATACGTTTCCGAAGGCATGAAGC

CCCACCACGAAAGTGGCTTTATTTTGCCCGACTCCACGAAAGCTGTGAAA

>428_Sample_ASV22

CACCGCGGTTATACGAGAGGCCCAAGTTGACAGGTACCGGCGTAAAGAGTGGTTAAGAAA

AAATATTAAACTAAAGCCGAACACCTTCAGAGCTGTCATACGTTTCCGAAGGCATGAAGC

CCCACCACGAAAGTGGCTTTATTTTGCCCGACTCCACGAAAGCTAAGAAA

>428_Sample_ASV23

CACCGCGGTTATACGAGAGGCCCTAGTTGATAACTACCGGCGTAAAGAGTGGTTATGGAA

AAATATTTAATAAAGCCGAACACCCCCTCAGCCGTCATACGCACCTGGGAGCACGAAGAC

CTACTGCGAAAGCAGCTTTAATTATGCCTGACCCCACGACAGCTAAGAAA

>428_Sample_ASV24

CACCGCGGTTATACGAGAGGCCCAAGTTGATACTCCACGGCGTAAAGAGTGGTTAGAAAA

GACCTACTACTAAAGCCGAACGCCTTCAAAGCTGTTATACGCATCCGAAGGTGAGAAGCC

CACCCACGAAAGTGGCTTTACAACCTTGACTCCACGAAAGCTATGACA

>428_Sample_ASV25

CACCGCGGTTATACGAGAGACCCCAGTTGATAAACACGGCGTAAAGAGTGGTTAAGGGCA

CAAAACAAATAAAGTCAAACAACCTCTTAGCTGTTATACGCATTATGAGGATATGAAGCC

CCCCTACGAAAGTAACTTTACCAACCCCTGACCCCACGAAAGCTAAGAAA

>428_Sample_ASV26

CACCGCGGTTATACGAGAGGCCCAAGTTGATAGACATCGGCGTAAAGAGTGGTTAAGGAA

GTCTCAAAACTAAAGCCGAATGCCTTCAAGACTGTTATACGTTTCCGAAGGTAAGAAGCC

CCACTACGAAAGTGGCTTTACTCCACCTGACTCCACGAAAGCTGTGAAA

>428_Sample_ASV27

CACCGCGGTTATACGAGAGACTCCAGTTGATAGCTACGGCGTAAAGAGTGGTTTGGGGCC

CGCCCAAAATAAAGCCAAAGACCTCCCAAGCCGTCAAACGCACCCCGGAGGCACGAAGTC

CTAACGCGAAAGCAACTTTACCTTCCCCGACGCCACGAAAGCTAAGAAA

>428_Sample_ASV28

CACCGCGGTTATACGAAAGACCCTAGTTGATAGATCACGGCGTAAAGGGTGGTTAAGGAG

AACAAAAATAAAGCTAAAAATCCTCTAAGCTGTCATACGCTTTCCGAAGACATGAGACCC

AACCACGAAAGTAACTTTAAACTTTCCTGACACCACGAAAGCCAAGAAA

>453_Sample_ASV1

CACCGCGGTTATACGAAAGACCCTAGTTGATGGGTGCGGCGTAAAGGGTGGTTAGGGACT

ACAAAAAATAGAGCTAAAGATCCTCTAGGTCGTCATACACATCACGAGGAAACGAAGCCC

AAATACGAAAGTAGCTCTAAATATAATCCTGACGCCACGAAAGCCAAGAAA

>453_Sample_ASV2

CACCGCGGTTATACGAAAGACCCTAGTTGATGGGTGCGGCGTAAAGGGTGGTTAGGGACT

ACAAAAAATAGAGCTAAAGATCCTCTAGGTCGTCATACACATCACGAGAAAACGAAGCCC

AAATACGAAAGTAGCTCTAAATATAATCCTGACGCCACGAAAGCCAAGAAA

>453_Sample_ASV3

CACCGCGGTTATACGAGAGACCCTAGTTGATTAAAGCGGCGTAAAGAGTGGTTATGGAAC

TATTCTTTTAAAGCAGAAAACCTCTCAAACTGTTATACGCACCCAGAGGTCTGAATCCCT

CACACGAAAGTGACTTTATTTATGCCTACCAGAACCCACGAAAGCTGGGACA

>453_Sample_ASV4

CACCGCGGTTATACGAGAGGCCCAAGTTGACAGACACCGGCGTAAAGAGTGGCTAGGGAA

AAATTACTACTAAAGCCGAACACCTTCAGAACTGTCATACGTTCCCGAAGATAAGAAGCC

CCACCACGAAAGTGGCTTTATATTACCCGACCCCACGAAAGCTGTGAAA

>453_Sample_ASV5

CACCGCGGTTATACGAGAGGCCCAAGTTGACAGACACCGGCGTAAAGAGTGGCTAGGGAA

AAATTACTACTAAAGCCGAACACCCTCAGAACTGTCATACGTTCCAGAAGGCAAGAAGCT

CCACCACGAAAGTGGCTTTATATTACCCGACCCCACGAAAGCTGTGAAA

>453_Sample_ASV6

CACCGCGGTTATACGAGAGGCCCAAGTTGACAGACATCGGCGTAAAGAGTGGTTAAGGAA

AAATTACTGCTAAAGCCGAACACCCTCAGAACTGTCATACGTTCCCGAAGATAAGAAGCC

CCACCACGAAAGTGGCTTTATATTACCCGACCCCACGAAAGCTGTGAAA

>453_Sample_ASV7

CACCGCGATTATACGAGAGGCCCAAGTTGACAGACATCGGCGTAAAGAGTGGTTAAGGAA

AAATTACTGCTAAAGCCGAACACCTTCAGAACTGTCATACGTTCCCGAAGATAAGAAGCC

CCACCACGAAAGTGGCTTTATATTACCCGACCCCACGAAAGCTGTGAAA

>453_Sample_ASV8

CATGGCGATTATACGAGAGGCCCAAGTTGACAGACACTGGCGTAAAGAGTGGCTAAGGAA

AAACTACTACTAAAGCCGAACACCTTCAGAACTGTCATACGTTCCCGAAGATAAGAAGCC

CCACCACGAAAGTGGCTTTATATTACCCGACCCCACGAAAGCTGTGAAA

>453_Sample_ASV9

CACCGCGGTTATACGAGAGGCCCAAGTTGATAAGCCCGGCGTAAAGAGTGGTTATGGAGA

ATAAAACACTAAAGCTAAAGACCCCCTAGGCTGTTATACGCACCTGGCGGCTCGAACCAC

CTATACGAAAGTAGCTTTACCCCCCTTCCACCAGAATCCACGACAGCTGGGGCA

>453_Sample_ASV10

CACCGCGGTTATACGAGAGGCCCTAGTTGATAGCCACGGCGCAAAGAGTGGTTAAGGAGC

CCCACTAAATAAAGTCGAACACTTCCTAGGCCGTTATACGTTTTCTAGAAGCACGAAACC

CAATTACACGAAAGCAACTTTATACTAAACAACCTGACCCCACGAAAGCTAAGAAA

>453_Sample_ASV11

CACCGCGGTTATACGAGAGGCCCAAGTTGACAGACATCGGCGTAAAGAGTGGTTAAGGAA

ACCCCATTACTAAAGCCGAACGCCCTCAGAACTGTCATACGTTCCCGAAGGTAAGAAGCC

CCACTACGAAAGTGGCTTTAACCACACCCGACCCCACGAAAGCTGTGAAA

>453_Sample_ASV12

CACCGCGGTTATACGAGAGGCCCAAGTTGACAGACATCGGCGTAAAGAGTGGTTAAGGAA

ACCCCATTACTAAAGCCGAACGCCCTCAGAACTGTCATACGTTCCCGAAGGTAAGAAGCC

CCACTACGAAAGTGGCTTTAACCACACCCGACCCCACGAAAGCTAAGAAA

>453_Sample_ASV13

CACCGCGGTTATACGAAAGACCCCAGTTTATAGACACGGCGTAAAGGGTGGTTATAGGAC

AAACTAACATAGAGCCAAAGACCCCCTAAGCCGTCATACGCTCACGAGGGCTCGAAGCCC

CTATACGAAAGTAGCTCTACCAAAAAATGCTCGACTCCACGAAAGCTGGGACA

>453_Sample_ASV14

CACCGCGGTTATACGAGAGACCCTAGTTGATAAATACGGCGTAAAGAGTGGTTATGGGAA

AAACAAATAAAGTCAAACAACCTCTTAGCTGTTATACGCATTATGAGAGCATGAAGCCCC

CTCACGAAAGTAACTTTAATACCTCCTGACCCCACGAAAGCTAAGAAA

>453_Sample_ASV15

CACCGCGGTTATACGAAAGACCCTAGTTGATGGATCACGGCGTAAAGGGTGGTTAAGGGA

TATAAAAATAAAGCTAAAAATCCTCTTAGCTGTCATACGCTTTCCGAAGACATGAGGCCC

AATTACGAAAGTAGCTTTAAACCACCCTGACACCACGAAAGCCAAGAAA

>453_Sample_ASV16

CACCGCGGTTATACGAGAGGCCCTAGTTGATAACTACCGGCGTAAAGAGTGGTTATGGAA

AAATATTTAATAAAGCCGAACACCCCCTCAGCCGTCATACGCACCTGGGAGCACGAAGAC

CTACTGCGAAAGCAGCTTTAATTATGCCTGACCCCACGACAGCTAAGAAA

>453_Sample_ASV17

CACCGCGGTTATACGAGAGGCCCAAGTTGATTATTACCGGCGTAAAGCGTGGTTAAGATT

TTTATAGGACTAAAGCCGAACCCCCTCAGAGCTGTTATACGCACCCGAAGGTTAGAAGTT

CAATCACGAAAGTGGCTTTACAACCCCGAACCCACGAAAGCTAAGATA

>453_Sample_ASV18

CACCGCGGTTATACGAGAGACCCTAATTGATAGGCGCGGCGTAAAGAGTGGTTTAGGACT

AAAACTAATAAAGCTAAAGATCCTCCCAGCCGTCGCACGCATTCTGAGGACACGAAACTC

AACCACGAAAGTAGCTTTAACATACTCTCCTGACCCCACGAAAGCTAAGAAA

>453_Sample_ASV19

CACCGCGGTTATACGAAAGACCCTAGTTGATAGGCACGGCGTAAAGGGTGGTTAGGGATA

TATCAAAAATAAAGTTAAAGAGCCTCTAAGCTGTCGCACGCATCCCGAGAGCTCGAGACC

CAGACACGAAAGTAACTTTAAAATACCTCACCTGACCCCACGAAAGCTAAGAAA

>453_Sample_ASV20

CACCGCGGTTATACGAGAGGCCCAAGTTGATACTCCACGGCGTAAAGAGTGGTTAGAAAA

GACCTACTACTAAAGCCGAACGCCTTCAAAGCTGTTATACGCATCCGAAGGTGAGAAGCC

CACCCACGAAAGTGGCTTTACAACCTTGACTCCACGAAAGCTATGACA

>453_Sample_ASV21

CACCGCGGTTATACGAGAGACCCCAGTTGATAAACACGGCGTAAAGAGTGGTTAAGGGCA

CAAAACAAATAAAGTCAAACAACCTCTTAGCTGTTATACGCATTATGAGGATATGAAGCC

CCCCTACGAAAGTAACTTTACCAACCCCTGACCCCACGAAAGCTAAGAAA

>453_Sample_ASV22

CACCGCGGTTATACGAAAGACCCTAGTTTATAGGCACGGCGTAAAGGGTGGTTAGGGGAT

AAACAAAATAAAGCCAAAGACCTTCTAAGTCGTCATACACTCATGAAGGCACGAAGCCCA

AATACGAAAGTAACTTTACAACTTAGACCCGACTCCACGAAAGCTGGGGGA

>453_Sample_ASV23

CACCGCGGTTATACGAGAGACCCTAATTAATAGCTACGGCGTAAAGAGTGGTTTAGGACA

AAAATTAATAAAGCCGAAGTTTCTCCTAGCCGTTGTACGCACTCTGAGAACACGAAGCCC

CACTACGAAAGTAGCTTTACCTATTATTTCCTGACCCCACGAAAGCTAAGAAA

>453_Sample_ASV24

CACCGCGGTTATACGAGAGACCCTAATTAATAGCTACGGCGTAAAGAGTGGTTTAGGGTA

AAAATTAATAAAGCCGAAGTTTCTCCTAGCCGTTGTACGCACTCTGAGAACACGAAGCCC

CACTACGAAAGTAGCTTTACCTATTATTTCCTGACCCCACGAAAGCTAAGAAA

>453_Sample_ASV25

CACCGCGGTTATACGAGAGACCCTAATTAATAGATACGGCGTAAAGAGTGGTTTAGGACA

AAAATTAATAAAGCCGAAGTTTCTCCTAGCCGTTGTACGCACTCTGAGAACACGAAGCCC

CACTACGAAAGTAGCTTTACCTATTATTTCCTGACCCCACGAAAGCTAAGAAA

>453_Sample_ASV26

CACCGCGGTTATACGAGAGACCCTAATTAATAGATACGGCGTAAAGAGTGGTTTAGGACA

AAAATTAATAAAGCCGAAGTTTCTCCTAGCCGTTGTACGCACTCTGAGAACACGAAGCCC

CACTACGAAAGTAGCTTTACCAATAATCTCCTGACCCCACGAAAGCTAAGAAA

>453_Sample_ASV27

CACCGCGGTTATACGAAAGACCCTAGTTGATAGATCACGGCGTAAAGGGTGGTTAAGGAA

AACAAAAATAAAGCTAAAAATCCTCTAAGCTGTCATACGCTTTCCGAAGACATGAGATCC

AACCACGAAAGTAGCTTTAAAATTTCCTGACACCACGAAAGCCAAGGAA

>495_Sample_ASV1

CACCGCGGTTATACGAGAGGCCCAAGTTGACAGACATCGGCGTAAAGAGTGGTTAAGGAA

AACCTTCAACTAAAGTCGAACGCTCTCAAGACTGTCGTACGTTTCCGAGAGTATGAAGCC

CCACCACGAAAGTGACTTTACCTCACCTGACTCCACGAAAGCTGCGAAA

>495_Sample_ASV2

CACCACGGTTATACGAGAGGCCCAAGTTGAAAGACATCGGCGTAAAGAGTGGTTAAGGAA

AACCTTCTACTAAAGTCGAACGCTCTCAGGACTGTCGTACGTTTCCGAGAGTATGAAGCC

CCACCACGAAAGTGACTTTACCTCACCTGACTCCACGAAAGCTGCGAAA

>495_Sample_ASV3

CACCGCGGTTATACGAAAGACCCTAGTTGATGGGTGCGGCGTAAAGGGTGGTTAGGGACT

ACAAAAAATAGAGCTAAAGATCCTCTAGGTCGTCATACACATCACGAGGAAACGAAGCCC

AAATACGAAAGTAGCTCTAAATATAATCCTGACGCCACGAAAGCCAAGAAA

>495_Sample_ASV4

CACCGCGGTTATACGAGAGACCCTAGTTGATTAAAGCGGCGTAAAGAGTGGTTATGGAAC

TATTCTTTTAAAGCAGAAAACCTCTCAAACTGTTATACGCACCCAGAGGTCTGAATCCCT

CACACGAAAGTGACTTTATTTATGCCTACCAGAACCCACGAAAGCTGGGACA

>495_Sample_ASV5

CACCGCGGTTATACGAAAGACCCTAGTTTATAGGTACGGCGTAAAGGGTGGTTAGGGGAC

AAACAAAATAAAGCCAAAGACCTCCTAAGCCGTCATACGCTCACGGAAGCACGAAGCCCA

AACACGAAAGTAGCTTTACCGAACATGCCCGACTCCACGAAAGCTGGGGAA

>495_Sample_ASV6

CACCGCGGTTATACGAAAGACCCTAGTTGATAGCCACGGCGTAAAGGGTGGTTAAGGAAC

CTAAAAAATAAAGCTAAAGACCCTCTAGGCCGTCATACGCATTACGAGGCAAACGAAACC

CCAACACGAAAGTAGCTTTAGACAAATATCCTGATGCCACGAAAGCCAAGAAA

>495_Sample_ASV7

CACCGCGGTTATACGAGAGGCCCAAGTTGACAGACACCGGCGTAAAGAGTGGCTAGGGAA

AAATTACTACTAAAGCCGAACACCTTCAGAACTGTCATACGTTCCCGAAGATAAGAAGCC

CCACCACGAAAGTGGCTTTATATTACCCGACCCCACGAAAGCTGTGAAA

>495_Sample_ASV8

CACCGCGGTTATACGAAAGACTCTAGTTGACAAACACGGCGTAAAGGGTGGTTAGGATAA

CAACAAAATAAAGCTAAAGACTTACCAAGCCGTCATACGCCCATGTAAGAGCGAAAACCA

CAAACGAAAGTAACTTTATTACACCATCCGAATCCACGAAAGCTAAGAAA

>495_Sample_ASV9

CACCGCGGTTATACGAGAGACCCTAGTTGATATATACGGCGTAAAGAGTGGTTTGGGATA

TTTCTCCAAAATAAAGCCGAAGATCTCCCAAGCCGTCACACGCATCCCGGAGACACGAAG

CCCTAATACGAAGGTAGCTTTATTAGTACCCCGACGCCACGAAAGCTAAGAAA

>495_Sample_ASV10

CACCGCGGTTATACGAGAGACCCTAGTTGATATATACGGCGTAAAGAGTGGTTTGGGGTT

ATGCCTCCAAAATAAAGCCGAAGATCTCCCAAGCCGTCACACGCATCCCGGAGACACGAA

GCCCTAATACGAAGGTAGCTTTATTAGTACCCCGACGCCACGAAAGCTAAGAAA

>495_Sample_ASV11

CACCGCGGTTATACGAGAGACCCTAGTTGATATATACGGCGTAAAGAGTGGTTTGGGGTT

ATGCCTCCAAAATAAAGCCGAAGATCTCCCAAGCCGTCACACGCATCCCGGAGACACGAA

GCCCTAATACGAAGGTAGCTTTATTAATTCCCCGACGCCACGAAAGCTAAGAAA

>495_Sample_ASV12

CACCGCGGTTATACGAGAGGCCCAAGTTGATAAGCCCGGCGTAAAGAGTGGTTATGGAGA

ATAAAACACTAAAGCTAAAGACCCCCTAGGCTGTTATACGCACCTGGCGGCTCGAACCAC

CTATACGAAAGTAGCTTTACCCCCCTTCCACCAGAATCCACGACAGCTGGGGCA

>495_Sample_ASV13

CACCGCGGTTATACGAGAGGCCCTAGTTGATAGCCACGGCGCAAAGAGTGGTTAAGGAGC

CCCACTAAATAAAGTCGAACACTTCCTAGGCCGTTATACGTTTTCTAGAAGCACGAAACC

CAATTACACGAAAGCAACTTTATACTAAACAACCTGACCCCACGAAAGCTAAGAAA

>495_Sample_ASV14

CACCGCGGTTATACGAGAGGCCCAAGTTGACAGACATCGGCGTAAAGAGTGGTTAAGGAA

ACCCCATTACTAAAGCCGAACGCCCTCAGAACTGTCATACGTTCCCGAAGGTAAGAAGCC

CCACTACGAAAGTGGCTTTAACCACACCCGACCCCACGAAAGCTGTGAAA

>495_Sample_ASV15

CACCGCGGTTATACGAGAGGCCCAAGTTGATAGATATCGGCGTAAAGAGTGGTTAAGGAA

ACCCCGTTACTAAAGCCGAACGCCCTCAGAACTGTCATACGTTCCCGAAGGTAAGAAGCC

CCACTACGAAAGTGGCTTTAACCACACCCGACCCCACGAAAGCTGTGAAA

>495_Sample_ASV16

CACCGCGGTTATACGAGAGGCCCAAGTTGACAGACACCGGCGTAAAGAGTGGTTAAGGAA

ATTCATCAACTAAAGCCGAATGCCCTCAGGACTGTTATACGTTTGCGAAGGTAAGAAGCC

CTACTACGAAAGTGGCTTTATTCCACCTGATTCCACGAAAGCTGCGGAA

>495_Sample_ASV17

CACCGCGGTTATACGAAAGACCCCAGTTTATAGACACGGCGTAAAGGGTGGTTATAGGAC

AAACTAACATAGAGCCAAAGACCCCCTAAGCCGTCATACGCTCACGAGGGCTCGAAGCCC

CTATACGAAAGTAGCTCTACCAAAAAATGCTCGACTCCACGAAAGCTGGGACA

>495_Sample_ASV18

CACCGCGGTTATACGAGAGGCCCCAGTTGATAAACACGGCGTAAAGGGTGGTTAAGGGAG

CATAAGAATAAAGCCGAAGGGCCCTTTGGCCGTTATACGCTTCTAGGTGTCCGAAGCCCA

AACACGAAAGTAGCTTTAGTTAAGCCCACCTGACTCCACGAAAACTGAGAAA

>495_Sample_ASV19

CACCGCGGTTATACGAGAGACCCTAGTTGATAAATACGGCGTAAAGAGTGGTTATGGGAA

AAACAAATAAAGTCAAACAACCTCTTAGCTGTTATACGCATTATGAGAGCATGAAGCCCC

CTCACGAAAGTAACTTTAATACCTCCTGACCCCACGAAAGCTAAGAAA

>495_Sample_ASV20

CACCGCGGTTATACGAGAGGCCCAAGTTGACAAACCCCGGCGTAAAGAGTGGTTAAGGGA

GATCAAAACTAAAGCCGAATGCTTTCAAAGCTGTTATACGCTTCCGAAAGTAAGAAGCTC

AATCACGAAAGTGGCTTTACTTTACCTGACCCCACGAAAGCTACGACA

>495_Sample_ASV21

CACCGCGGTTATACGAGAGGCTCAAGTTGATGAACCCCGGCGTAAAGAGTGGTTAAGGGA

GATCAAAACTAAAGCCGAATGCTTTCAAAGCTGTTATACGCTTCCGAAAGTAAGAAGCTC

AATCACGAAAGTGGCTTTACTTTACCTGACCCCACGAAAGCTACGACA

>495_Sample_ASV22

CACCGCGGTTATACGAGAGACCCTAATTGATAGACACGGCGTAAAGAGTGGTTTAGGACA

ACCCAAAAATAAAGCTAAAGACCTTCTAAGCCGTCACACGCACGCCGAAGGCACGAAGCC

CTGACACGAAAGTAGCTTTACTATTACCCCCGACGCCACGAAAGCTAAGAAA

>495_Sample_ASV23

CACCGCGGTTATACGAAAGACCCTAGTTGATGGATCACGGCGTAAAGGGTGGTTAAGGGA

TATAAAAATAAAGCTAAAAATCCTCTTAGCTGTCATACGCTTTCCGAAGACATGAGGCCC

AATTACGAAAGTAGCTTTAAACCACCCTGACACCACGAAAGCCAAGAAA

>495_Sample_ASV24

CACCGCGGTTATACGAAAGACCCCAGTTTATAGATGCGGCGTAAAGGGTGGTTAAGAGAA

AATCAAAATAAGGCCAAAGATCCCCTAAGCAGTCATACGCTCCACGAGGACACGAAACCC

AAATACGAAAGTTGCCTTAATAAACTTCACCTGATCCCACGAAAGCTGAAAAA

>495_Sample_ASV25

CACCGCGGTTATACGAGAGGCCCAAGTTGACAAACCCCGGCGTAAAGAGTGGTTAAGGAA

AATTTAAAACTAAAGCCGAATGCCCCCAAAGCTGTTATACGCACCCGAGGATAAGAAGCC

CAATCACGAAAGTGGCTTTACACTACCTGAACCCACGAAAGCTATGAAA

>495_Sample_ASV26

CACCGCGGTTATACGAGAGGCCCAAGTTGACAAACCCCGGCGTAAAGAGTGGTTAAGGAA

AATTTAAAACTAAAGCCGAATGCCCCCAAAGCTGTTATACGCACCCGAGGGTAAGAAGCC

CAATCACGAAAGTGGCTTTACACTACCTGAACCCACGAAAGCTATGAAA

>495_Sample_ASV27

CACCGCGGTTATACGAGAGACCCTAGTTGATAGCTACGGCGTAAAGCGTGGTTAGGAGAA

CAAGATAAATAAAGTCAAACAATCTCTCGGCCGTTATACGTTATCTGAGAATATGAAGTC

CTACCACGAAAGTAACTTTAATTTTTCTGACCCCACGAAAACTGAGGAA

>495_Sample_ASV28

CACCGCGGTTATACGAGAGGCCCTAGTTGATAACTACCGGCGTAAAGAGTGGTTATGGAA

AAATATTTAATAAAGCCGAACACCCCCTCAGCCGTCATACGCACCTGGGAGCACGAAGAC

CTACTGCGAAAGCAGCTTTAATTATGCCTGACCCCACGACAGCTAAGAAA

>495_Sample_ASV29

CACCGCGGTTATACGAAAGACCCTAGTTTATAGACACGGCGTAAAGGGTGGTTCGGGACT

AAACAAAATAAAGCCAAAGACCTCCTAAGCCGTCATACGCTCACGGAAGCACGAAGCCCA

AGTACGAAAGTAGCTTTACAAAACATGCCTGACTCCACGAAAGCTAGGAAA

>495_Sample_ASV30

CACCGCGGTTATACGAGAGGCCCAAGTTGATTATTACCGGCGTAAAGCGTGGTTAAGATT

TTTATAGAACTAAAGCCGAACCCCCTCAGAGCTGTTATACGCACCCGAAGGTTAGAAGTT

CAATCACGAAAGTGGCTTTACAACCCCGAACCCACGAAAGCTAAGATA

>495_Sample_ASV31

CACCGCGGTTATACGAGAGGCCCAAGTTGATACTCCACGGCGTAAAGAGTGGTTAGAAAA

GACCTACTACTAAAGCCGAACGCCTTCAAAGCTGTTATACGCATCCGAAGGTGAGAAGCC

CACCCACGAAAGTGGCTTTACAACCTTGACTCCACGAAAGCTATGACA

>495_Sample_ASV32

CACCGCGGTTATACGAGAGACCCCAGTTGATAAACACGGCGTAAAGAGTGGTTAAGGGCA

CAAAACAAATAAAGTCAAACAACCTCTTAGCTGTTATACGCATTATGAGGATATGAAGCC

CCCCTACGAAAGTAACTTTACCAACCCCTGACCCCACGAAAGCTAAGAAA

>495_Sample_ASV33

CACCGCGGTTATACGAGAGACCCTAGTTGATATATACGGCGTAAAGAGTGGTTTGGAATA

ACCAAGTAATATAGCCAAAGACCTCCCAAGCCGTCACACGCACCCCGGAGGCACGAAGCC

CAAACACGAAAGTAGCTTTATTAACTTACCGACGCCACGAAAGCTAAGAAA

>495_Sample_ASV34

CACCGCGGTTATACGAAAGACCCTAGTTTATAGGCACGGCGTAAAGGGTGGTTAGGGGAT

AAACAAAATAAAGCCAAAGACCTTCTAAGTCGTCATACACTCATGAAGGCACGAAGCCCA

AATACGAAAGTAACTTTACAACTTAGACCCGACTCCACGAAAGCTGGGGGA

>500_Sample_ASV1

CACCGCGGTTATACGAGAGGCCCAAGTTGACAGACATCGGCGTAAAGAGTGGTTAAGGAA

AACCTTCAACTAAAGTCGAACGCTCTCAAGACTGTCGTACGTTTCCGAGAGTATGAAGCC

CCACCACGAAAGTGACTTTACCTCACCTGACTCCACGAAAGCTGCGAAA

>500_Sample_ASV2

CACCGCGGTTATACGAAAGACCCTAGTTGATGGGTGCGGCGTAAAGGGTGGTTAGGGACT

ACAAAAAATAGAGCTAAAGATCCTCTAGGTCGTCATACACATCACGAGGAAACGAAGCCC

AAATACGAAAGTAGCTCTAAATATAATCCTGACGCCACGAAAGCCAAGAAA

>500_Sample_ASV3

CACCGCGGTTATACGAGAGACCCTAGTTGATTAAAGCGGCGTAAAGAGTGGTTATGGAAC

TATTCTTTTAAAGCAGAAAACCTCTCAAACTGTTATACGCACCCAGAGGTCTGAATCCCT

CACACGAAAGTGACTTTATTTATGCCTACCAGAACCCACGAAAGCTATGACA

>500_Sample_ASV4

CACCGCGGTTATACGAGAGACCCTAGTTGATTAAAGCGGCGTAAAGAGTGGTTATGGAAC

TATTCTTTTAAAGCAGAAAACCTCTCAAACTGTTATACGCACCCAGAGGTCTGAATCCCT

CACACGAAAGTGACTTTATTTATGCCTACCAGAACCCACGAAAGCTGGGACA

>500_Sample_ASV5

CACCGCGGTTATACGAAAGACCCTAGTTTATAGGTACGGCGTAAAGGGTGGTTAGGGGAC

AAACAAAATAAAGCCAAAGACCTCCTAAGCCGTCATACGCTCACGGAAGCACGAAGCCCA

AACACGAAAGTAGCTTTACCGAACATGCCCGACTCCACGAAAGCTGGGGAA

>500_Sample_ASV6

CACCGCGGTTATACGAGAGGCCCAAGTTGACAGACACCGGCGTAAAGAGTGGCTAGGGAA

AAATTACTACTAAAGCCGAACACCTTCAGAACTGTCATACGTTCCCGAAGATAAGAAGCC

CCACCACGAAAGTGGCTTTATATTACCCGACCCCACGAAAGCTGTGAAA

>500_Sample_ASV7

CACCGCGGTTAAACGAGAGACCCTAGTTGATCATCACGGCGTAAAGAGTGGTTAGGGGAT

TATCATAAATAAAGCCAAAGACCTCCCAAGCTGTCGCACGCATTCCGGAGGCGCGAAGCC

CACACACGAAAGTAGCTTTAACTATTGACCCTGATGCCACGAAAGCTAAGAAA

>500_Sample_ASV8

CACCGCGGTTATACGAGAGGCCCAAGTTGATAAGCCCGGCGTAAAGAGTGGTTATGGAGA

ATAAAACACTAAAGCTAAAGACCCCCTAGGCTGTTATACGCACCTGGCGGCTCGAACCAC

CTATACGAAAGTAGCTTTACCCCCCTTCCACCAGAATCCACGACAGCTGGGGCA

>500_Sample_ASV9

CACCGCGGTTATACGAGAGGCCCAAGTTGACAGACATCGGCGTAAAGAGTGGTTAAGGAA

ACCCCATTACTAAAGCCGAACGCCCTCAGAACTGTCATACGTTCCCGAAGGTAAGAAGCC

CCACTACGAAAGTGGCTTTAACCACACCCGACCCCACGAAAGCTGTGAAA

>500_Sample_ASV10

CACCGCGGTTATACGAGAGGCCCAAGTTGATAGATATCGGCGTAAAGAGTGGTTAAGGAA

ACCCCGTTACTAAAGCCGAACGCCCTCAGAACTGTCATACGTTCCCGAAGGTAAGAAGCC

CCACTACGAAAGTGGCTTTAACCACACCCGACCCCACGAAAGCTGTGAAA

>500_Sample_ASV11

CACCGCGGTCACACGATTAACCCAAGTCAATAGAAGCCGGCGTAAAGAGTGTTTTAGATC

ACCCCCTCCCCAATAAAGCTAAAACTCACCTGAGTTGTAAAAAACTCCAGTTGACACAAA

ATAGACTACGAAAGTGGCTTTAACATATCTGAACACACAATAGCTAAGACC

>500_Sample_ASV12

CACCGCGGTTATACGAGAGGCCCTAGTTGATAAATACGGCGTAAAGAGTGGTTAGAGGAC

TCCCATAATAAAGCCAAAGACCCTCCGGGCCGTCACACGCATACGAGGGCACGAAGTTCA

CACACGAAAGTAGCTTTAAATTACCCGACGCCACGAAAGCTAAGAAA

>500_Sample_ASV13

CACCGCGGTTATACGAGAGGCCCTAGTTGATAAATACGGCGTAAAGAGTGGTTAGAGGAC

TCCCATAATAAAGCCAAAGACCCCCCGGGCCGTCACACGCATACGAGGGCACGAAGTTCA

CACACGAAAGTAGCTTTAAATTACCCGACGCCACGAAAGCTAAGAAA

>500_Sample_ASV14

GACCGCGGTTATACGAGAGACTCTAGTTGATAGATACGGCGTAAAGAGTGGTTTGGGATA

AACTATAAATAAAGCCAAAGACCTTCCAAGCTGTTAAACGCATACCGAAGGCACGAAGCC

CCAACACGAAAGTAGCTTTACTACTACCCCCGATGCCACGAAAGCTAAGACA

>500_Sample_ASV15

CACCGCGGTTATACGAAAGACCCCAGTTTATAGACACGGCGTAAAGGGTGGTTATAGGAC

AAACTAACATAGAGCCAAAGACCCCCTAAGCCGTCATACGCTCACGAGGGCTCGAAGCCC

CTATACGAAAGTAGCTCTACCAAAAAATGCTCGACTCCACGAAAGCTGGGACA

>500_Sample_ASV16

CACCGCGGTTATACGAGAGACCCTAGTTGATAAATACGGCGTAAAGAGTGGTTATGGGAA

AAACAAATAAAGTCAAACAACCTCTTAGCTGTTATACGCATTATGAGAGCATGAAGCCCC

CTCACGAAAGTAACTTTAATACCTCCTGACCCCACGAAAGCTAAGAAA

>500_Sample_ASV17

CACCGCGGTTATACGAAAGACCCTAGTTGATGGATCACGGCGTAAAGGGTGGTTAAGGGA

TATAAAAATAAAGCTAAAAATCCTCTTAGCTGTCATACGCTTTCCGAAGACATGAGGCCC

AATTACGAAAGTAGCTTTAAACCACCCTGACACCACGAAAGCCAAGAAA

>500_Sample_ASV18

CACCGCGGTTATACGAGAGACCCTAGTTGATAGCTACGGCGTAAAGCGTGGTTAGGAGAA

CAAGATAAATAAAGTCAAACAATCTCTCGGCCGTTATACGTTATCTGAGAATATGAAGTC

CTACCACGAAAGTAACTTTAATTTTTCTGACCCCACGAAAACTGAGGAA

>500_Sample_ASV19

CACCGCGGTCATACGATTAACCCAAGTTAATAGGCACACGGCGTAAAGCGTGTTTAAGCA

CTATGCCAAATAAAGTTAAACTCCAATTAAGCTGTAAAAAGCCATAATCAAGACGAAAAT

AAATAACGAAAGTAACTTTACAACCGCTGAAACACGATAGCTAAGATC

>500_Sample_ASV20

CACCGCGGTTATACGAAAGACCCTAGTTTATAGACACGGCGTAAAGGGTGGTTCGGGACT

AAACAAAATAAAGCCAAAGACCTCCTAAGCCGTCATACGCTCACGGAAGCACGAAGCCCA

AGTACGAAAGTAGCTTTACAAAACATGCCTGACTCCACGAAAGCTAGGAAA

>500_Sample_ASV21

CACCGCGGTTATACGAAAGACCCTAGTTGATAGGCACGGCGTAAAGGGTGGTTAGGGATA

TATCAAAAATAAAGTTAAAGAGCCTCTAAGCTGTCGCACGCATCCCGAGAGCTCGAGACC

CAGACACGAAAGTAACTTTAAAATACCTCACCTGACCCCACGAAAGCTAAGAAA

>500_Sample_ASV22

CACCGCGGTTATACGAAAGACCCTAGTTGATAGCCACGGCGTAAAGGGTGGTTAAGGTAT

ACAACAAATAAAGCTAAATAGCCTCTAGGCCGTCGCACGCATTCCGAGAGCTCGAAACCC

AAAAACGAAAGTAGCTTTAAAATAAACAGACCTGACCCCACGAAAGCTAAGAAA

>500_Sample_ASV23

CACCGCGGTTATACGAGAGGCCCAAGTTGATACTCCACGGCGTAAAGAGTGGTTAGAAAA

GACCTACTACTAAAGCCGAACGCCTTCAAAGCTGTTATACGCATCCGAAGGTGAGAAGCC

CACCCACGAAAGTGGCTTTACAACCTTGACTCCACGAAAGCTATGACA

>500_Sample_ASV24

CACCGCGGTTATACGAGAGACCCCAGTTGATAAACACGGCGTAAAGAGTGGTTAAGGGCA

CAAAACAAATAAAGTCAAACAACCTCTTAGCTGTTATACGCATTATGAGGATATGAAGCC

CCCCTACGAAAGTAACTTTACCAACCCCTGACCCCACGAAAGCTAAGAAA

>500_Sample_ASV25

CACCGCGGTTATACGAAAGACCCTAGTTTATAGGCACGGCGTAAAGGGTGGTTAGGGGAT

AAACAAAATAAAGCCAAAGACCTTCTAAGTCGTCATACACTCATGAAGGCACGAAGCCCA

AATACGAAAGTAACTTTACAACTTAGACCCGACTCCACGAAAGCTGGGGGA

>500_Sample_ASV26

CACCGCGGTTATACGAGAGGCCCAAGTTGATAGACATCGGCGTAAAGAGTGGTTAAGGAA

GTCTCAAAACTAAAGCCGAATGCCTTCAAGACTGTTATACGTTTCCGAAGGTAAGAAGCC

CCACTACGAAAGTGGCTTTACTCCACCTGACTCCACGAAAGCTGTGAAA

>500_Sample_ASV27

CACCGCGGTTATACGAGAGACTCCAGTTGATAGCTACGGCGTAAAGAGTGGTTTGGGGCC

CGCCCAAAATAAAGCCAAAGACCTCCCAAGCCGTCAAACGCACCCCGGAGGCACGAAGTC

CTAACGCGAAAGCAACTTTACCTTCCCCGACGCCACGAAAGCTAAGAAA

>500_Sample_ASV28

CACCGCGGTTATACGAAAGACCCTAGTTGATAGATCACGGCGTAAAGGGTGGTTAAGGAG

AACAAAAATAAAGCTAAAAATCTTCTAAGCTGTCATACGCTTTCCGAAGACATGAGACCC

AACCACGAAAGTAGCTTTAAACTTTCCTGACACCACGAAAGCCAAGAAA

>505_Sample_ASV1

CACCGCGGTTATACGAAAGACCCTAGTTGATGGGTGCGGCGTAAAGGGTGGTTAGGGACT

ACAAAAAATAGAGCTAAAGATCCTCTAGGTCGTCATACACATCACGAGGAAACGAAGCCC

AAATACGAAAGTAGCTCTAAATATAATCCTGACGCCACGAAAGCCAAGAAA

>505_Sample_ASV2

CACCGCGGTTATACGAGAGACCCTAGTTGATTAAAGCGGCGTAAAGAGTGGTTATGGAAC

TATTCTTTTAAAGCAGAAAACCTCTCAAACTGTTATACGCACCCAGAGGTCTGAATCCCT

CACACGAAAGTGACTTTATTTATGCCTACCAGAACCCACGAAAGCTGGGACA

>505_Sample_ASV3

CACCGCGGTTATACGAGAGACCCTAGTTGATTAAAGCGGCGTAAAGAGTGGTTATGGAAC

TATTCTTTTAAAGCAGAAAACCTCTCAAACTGTTATACGCACCCAGAGGTCTGAATCCCT

CACACGAAAGTGACTTTATTTATGCCTACCAGAACCCACGAAAGCTGTGAAA

>505_Sample_ASV4

CACCGCGGTTATACGAAAGACCCTAGTTTATAGGTACGGCGTAAAGGGTGGTTAGGGGAC

AAACAAAATAAAGCCAAAGACCTCCTAAGCCGTCATACGCTCACGGAAGCACGAAGCCCA

AACACGAAAGTAGCTTTACCGAACATGCCCGACTCCACGAAAGCTGGGGAA

>505_Sample_ASV5

CACCGCGGTTATACGAGAGACCCTAGTTGATATACACGGCGTAAAGAGTGGTTTGGGGTA

TATAATAAATAAAGCCAAAGACCTTCTAGGCCGTTGAACGCACCACGAAGGAGCGAAGTC

CCAATACGAAAGTAGCTTTATAAAACCTGACGCCACGAAAGCTAAGAAA

>505_Sample_ASV6

CACCGCGGTTATACGAGAGGCCCAAGTTGACAGACACCGGCGTAAAGAGTGGCTAGGGAA

AAATTACTACTAAAGCCGAACACCTTCAGAACTGTCATACGTTCCCGAAGATAAGAAGCC

CCACCACGAAAGTGACTTTATATTACCCGACCCCACGAAAGCTGTGAAA

>505_Sample_ASV7

CACCGCGGTTATACGAGAGGCCCAAGTTGACAGACACCGGCGTAAAGAGTGGCTAGGGAA

AAATTACTACTAAAGCCGAACACCTTCAGAACTGTCATACGTTCCCGAAGATAAGAAGCC

CCACCACGAAAGTGGCTTTAACCACACCCGACCCCACGAAAGCTGTGAAA

>505_Sample_ASV8

CACCGCGGTTATACGAGAGGCCCAAGTTGACAGACACCGGCGTAAAGAGTGGCTAGGGAA

AAATTACTACTAAAGCCGAACACCTTCAGAACTGTCATACGTTCCCGAAGATAAGAAGCC

CCACCACGAAAGTGGCTTTATATTACCCGACCCCACGAAAGCTGTGAAA

>505_Sample_ASV9

CACCGCGGTTATACGAGAGGCCCAAGTTGACAGACACCGGCGTAAAGAGTGGCTAAGGAA

AAATTTCTACTAAAGCCGAACACCTTCAGAACTGTCATACGTTCCCGAAGAAAAGAAGCC

CCACCACGAAAGTGGCTTTATATTACCCGACCCCACGAAAGCTGTGAAA

>505_Sample_ASV10

CACCGCGGTTATACGAGAGGCCCAAGTTGACAGACATCGGCGTAAAGAGTGGTTAAGGAA

ACCCCATTACTAAAGCCGAACACCTTCAGAACTGTCATACGTTCCCGAAGATAAGAAGCC

CCACCACGAAAGTGGCTTTATATTACCCGACCCCACGAAAGCTGTGAAA

>505_Sample_ASV11

CACCGCGGTTATACGAGAGGCCCAAGTTGATAAGCCCGGCGTAAAGAGTGGTTATGGAGA

ATAAAACACTAAAGCTAAAGACCCCCTAGGCTGTTATACGCACCTGGCGGCTCGAACCAC

CTATACGAAAGTAGCTTTACCCCCCTTCCACCAGAATCCACGACAGCTGGGGCA

>505_Sample_ASV12

CACCGCGGTTATACGAGAGGCCCAAGTTGACAGACATCGGCGTAAAGAGTGGTTAAGGAA

ACCCCATTACTAAAGCCGAACGCCCTCAGAACTGTCATACGTTCCCGAAGGTAAGAAGCC

CCACTACGAAAGTGGCTTTAACCACACCCGACCCCACGAAAGCTGTGAAA

>505_Sample_ASV13

CACCGCGGTTATACGAGAGGCCCAAGTTGATAGATATCGGCGTAAAGAGTGGTTAAGGAA

ACCCCGTTACTAAAGCCGAACGCCCTCAGAACTGTCATACGTTCCCGAAGGTAAGAAGCC

CCACTACGAAAGTGGCTTTAACCACACCCGACCCCACGAAAGCTGTGAAA

>505_Sample_ASV14

CACCGCGGTTATACGAGAAACCCTAGTTGACAAATACGGCGTAAAGAGTGGTTAAGGGGT

ATCAAAAATAAAGTCAAACATTTTCCTAGTTGTTATAAGCATTTCGAGAACATGAAGCCC

AACCACGAAAGTAACTTTACCTTCACCTGACCCCACGAAAGCTAAGAAA

>505_Sample_ASV15

CACCGCGGTTATACGAGAGACCCTAGTTGATAAATACGGCGTAAAGAGTGGTTATGGGAA

AAACAAATAAAGTCAAACAACCTCTTAGCTGTTATACGCATTATGAGAGCATGAAGCCCC

CTCACGAAAGTAACTTTAATACCTCCTGACCCCACGAAAGCTAAGAAA

>505_Sample_ASV16

CACCGCGGTTATACGAGAGGCTCAAGTTGATAAACCCCGGCGTAAAGAGTGGTCAAGGAA

AAATAGAAACTAAAGCCGAATGCTTTCAAGACTGTTATACGTTCCCGAGAGTAAGAAGAC

CAATTACGAAAGTGGCTTTACTTTACCTGACCCCACGAAAGCTACGACA

>505_Sample_ASV17

AACCGCGGTTATACGAGAGACCCTAATTGATAGCTCACGGCGTAAAGGGTGGTTTGGGGA

AACCTCAAAATAAAGCTAAAGACCTTCTAAGCCGTTACACGCATACCGAAGGCACGAGGC

CCTAACACGAAAGTAGCTTTACTATTACCCCTGACGCCACGAAAGCTAAGAAA

>505_Sample_ASV18

CACCGCGGTTATACGAAAGACCCTAGTTGATGGATCACGGCGTAAAGGGTGGTTAAGGGA

TATAAAAATAAAGCTAAAAATCCTCTTAGCTGTCATACGCTTTCCGAAGACATGAGGCCC

AATTACGAAAGTAGCTTTAAACCACCCTGACACCACGAAAGCCAAGAAA

>505_Sample_ASV19

CACCGCGGTCATACGATTAACCCAAGTTAATAGGCACACGGCGTAAAGCGTGTTTAAGCA

CTACGCCAAATAAAGTTAAACTCCAATTAAGCTGTAAAAAGCCATAATCAAGACGAAAAT

AAATAACGAAAGTAACTTTACAACCGCTGAAACACGATAGCTAAGATC

>505_Sample_ASV20

CACCGCGGTTATACGAGAGGCCCAAGTTGATACTCCACGGCGTAAAGAGTGGTTAGAAAA

GACCTACTACTAAAGCCGAACGCCTTCAAAGCTGTTATACGCATCCGAAGGTGAGAAGCC

CACCCACGAAAGTGGCTTTACAACCTTGACTCCACGAAAGCTATGACA

>505_Sample_ASV21

CACCGCGGTTATACGAGAGGCCCAAGTTGATACTCCACGGCGTAAAGAGTGGTTAGAAAA

GACCTACTACTAAAGCCGAACGCCTTCAAAGCTGTTATACGCATCCGAAGGTGAGAAGCC

CACCCACGAAAGTGGCTTTACAACCTTGACTCCACGAAAGCTGTGAAA

>505_Sample_ASV22

CACCGCGGTTATACGAGAGACCCCAGTTGATAAACACGGCGTAAAGAGTGGTTAAGGGCA

CAAAACAAATAAAGTCAAACAACCTCTTAGCTGTTATACGCATTATGAGGATATGAAGCC

CCCCTACGAAAGTAACTTTACCAACCCCTGACCCCACGAAAGCTAAGAAA

>505_Sample_ASV23

CACCGCGGTTATACGAGAGACCCTAGTTGATATATACGGCGTAAAGAGTGGTTTGGAATA

ACCAAGTAATATAGCCAAAGACCTCCCAAGCCGTCACACGCACCCCGGAGGCACGAAGCC

CAAACACGAAAGTAGCTTTATTAACTTACCGACGCCACGAAAGCTAAGAAA

>505_Sample_ASV24

CACCGCGGTTATACGAAAGACCCTAGTTTATAGGCACGGCGTAAAGGGTGGTTAGGGGAT

AAACAAAATAAAGCCAAAGACCTTCTAAGTCGTCATACACTCATGAAGGCACGAAGCCCA

AATACGAAAGTAACTTTACAACTTAGACCCGACTCCACGAAAGCTGGGGGA

>505_Sample_ASV25

CACCGCGGTTATACGAGAGACCCTAATTAATAGATACGGCGTAAAGAGTGGTTTAGGACA

AAAATTAATAAAGCCGAAGTTTCTCCTAGCCGTTGTACGCACTCTGAGAACACGAAGCCC

CACTACGAAAGTAGCTTTACCTATTATTTCCTGACCCCACGAAAGCTAAGAAA

>505_Sample_ASV26

CACCGCGGTTATACGAGAGGCCCAAGTTGATAGACATCGGCGTAAAGAGTGGTTAAGGAA

GTCTCAAAACTAAAGCCGAATGCCTTCAAGACTGTTATACGTTTACGAAGGTAAGAAGCC

CCACTACGAAAGTGGCTTTATTCCACCTGACCCCACGAAAGCTGTGAAA

>505_Sample_ASV27

CACCGCGGTTATACGAGAGGCCCAAGTTGATAGACATCGGCGTAAAGAGTGGTTAAGGAA

GTCTCAAAACTAAAGCCGAATGCCTTCAAGACTGTTATACGTTTCCGAAGGTAAGAAGCC

CCACTACGAAAGTGGCTTTATTCCACCTGACCCCACGAAAGCTGTGAAA

>505_Sample_ASV28

CACCGCGGTTATACGAGAGGCCCAAGTTGATAGACATCGGCGTAAAGAGTGGTTAAGGAA

GTCTCAAAACTAAAGCCGAATGCCTTCAAGACTGTTATACGTTTCCGAAGGTAAGAAGCC

CCACTACGAAAGTGGCTTTACTCCACCTGACTCCACGAAAGCTGTGAAA

>522_Sample_ASV1

Sequence

>546_Sample_ASV1

TACCGCGGTTATACGAAAGACCCAAGTTAATAAACTACGGCGTAAAGCGTGGTTAGAGAC

CTCCAATAATTAAAGCCAAAGACCTTCTAAGCCGTCGCACGCCCCGCGAAGACACGAAGC

CCAAAAACGAAAGTAGCTTTATTCTTACTCGACCCCACGAAAGCTAGGGCA

>546_Sample_ASV2

CACCGCGGTTATACGAAAGACCCTAGTTGATGGGTGCGGCGTAAAGGGTGGTTAGGGACT

ACAAAAAATAGAGCTAAAGATCCTCTAGGTCGTCATACACATCACGAGGAAACGAAGCCC

AAATACGAAAGTAGCTCTAAATATAATCCTGACGCCACGAAAGCCAAGAAA

>546_Sample_ASV3

CACCGCGGTTATACGAGAGACCCTAGTTGATTAAAGCGGCGTAAAGAGTGGTTATGGAAC

TATTCTTTTAAAGCAGAAAACCTCTCAAACTGTTATACGCACCCAGAGGTCTGAATCCCT

CACACGAAAGTGACTTTATTTATGCCTACCAGAACCCACGAAAGCTGGGACA

>546_Sample_ASV4

CATGGCGATTATACGAGAGGCCCAAGTTGACAGACACTGGCGTAAAGAGTGGCTAAGGAA

AAACTACTACTAAAGCCGAACACCTTCAGAACTGTCATACGTTCCCGAAGATAAGAAGCC

CCACCACGAAAGTGGCTTTATATTACCCGACCCCACGAAAGCTGTGAAA

>546_Sample_ASV5

CACCGCGGTTATACGAGAGGCCCAAGTTGACAGACACCGGCGTAAAGAGTGGCTAGGGAA

AAATTACTACTAAAGCCGAACACCTTCAGAACTGTCATACGTTCCCGAAGATAAGAAGCC

CCACCACGAAAGTGGCTTTACAATGCCTGACCCCACGAAAGCTGTGAAA

>546_Sample_ASV6

CATGGCGATTATACGAGAGGCCCAAGTTGACAGACACTGGCGTAAAGAGTGGCTAGGGAA

AAATTACTACTAAAGCCGAACACCTTCAGAACTGTCATACGTTCCCGAAGATAAGAAGCC

CCACCACGAAAGTGGCTTTATATTACCCGACCCCACGAAAGCTGTGAAA

>546_Sample_ASV7

CATGGCGATTATACGAGAGGCCCAAGTTGACAGACACCGGCGTAAAGAGTGGCTAGGGAA

AAATTACTACTAAAGCCGAACACCTTCAGAACTGTCATACGTTCCCGAAGATAAGAAGCC

CCACCACGAAAGTGGCTTTATATTACCCGACCCCACGAAAGCTGTGAAA

>546_Sample_ASV8

CACCGCGGTTATACGAGAGGCCCAAGTTGACAGACACCGGCGTAAAGAGTGGCTAGGGAA

AAATTACTACTAAAGCCGAACACCTTCAGAACTGTCATACGTTCCCGAAGATAAGAAGCC

CCACCACGAAAGTGGCTTTATATTACCCGACCCCACGAAAGCTGTGAAA

>546_Sample_ASV9

CACCGCGGTTATACGAAAGACTCTAGTTGACAAACACGGCGTAAAGGGTGGTTAGGATAA

CAACAAAATAAAGCTAAAGACTTACCAAGCCGTCATACGCCCATGTAAGAGCGAAAACCA

CAAACGAAAGTAACTTTATTACACCATCCGAATCCACGAAAGCTAAGAAA

>546_Sample_ASV10

CACCGCGGTTATACGAAAGACCCTAGTTGATAGATCACGGCGTAAAGGGTGGTTAGGGAA

TACAAAAATAAAGCTAAAAATCCTCTAAGCTGTCATACGCTTCCCGAAGACATGAAACCC

AACCACGAAAGTAGCTTTAAACTCTCCTGACACCACGAAAGCCAAGGAA

>546_Sample_ASV11

CACCGCGGTTATACGAGAGGCCCAAGTTGATAAGCCCGGCGTAAAGAGTGGTTATGGAGA

ATAAAACACTAAAGCTAAAGACCCCCTAGGCTGTTATACGCACCTGGCGGCTCGAACCAC

CTATACGAAAGTAGCTTTACCCCCCTTCCACCAGAATCCACGACAGCTGGGGCA

>546_Sample_ASV12

CACCGCGGTTATACGAGAGGCCCAAGTTGACAGACATCGGCGTAAAGAGTGGTTAAGGAA

ACCCCATTACTAAAGCCGAACGCCCTCAGAACTGTCATACGTTCCCGAAGGTAAGAAGCC

CCACTACGAAAGTGGCTTTAACCACACCCGACCCCACGAAAGCTGTGAAA

>546_Sample_ASV13

CACCGCGGTTATACGAGAGGCCCAAGTTGATAGATATCGGCGTAAAGAGTGGTTAAGGAA

ACCCCGTTACTAAAGCCGAACGCCCTCAGAACTGTCATACGTTCCCGAAGGTAAGAAGCC

CCACTACGAAAGTGGCTTTAACCACACCCGACCCCACGAAAGCTGTGAAA

>546_Sample_ASV14

CACCGCGGTTATACGAGAGGCCCCAGTTGATATTAGCGGCGTAAAGGGTGGTTAAGGACT

TTAATAACTAAAGCCAAACACTTCCCCGGCTGTTGTACGTAGACCGGAAGCTTGAAGCCC

AGACACGAAAGTAGCTTTATTTCCCCTGACCCCACGAAAGCTGAGAAA

>546_Sample_ASV15

CACCGCGGTTATACGAGAGGCCCCAGTTGATATTAGCGGCGTAAAGGGTGGTTAAGGACT

TTAATAACTAAAGCTAAACACTTCCCCGGCTGTTGTACGTAGACCGGAAGCTTGAAGCCC

AGACACGAAAGTAGCTTTATTTCCCCTGACCCCACGAAAGCTGAGAAA

>546_Sample_ASV16

CACCGCGGTTATACGAGAGGCCCCAGTTGATATTAGCGGCGTAAAGGGTGGTTAAGGACT

TTAATAACTAAAGCTAAACACTTCCCCGGCTGTTGTACGTAGACCGGAAGCTTGAAGCCC

AGACACGAAAGTAGCTTTATTCCCCCTGACCCCACGAAAGCTGAGAAA

>546_Sample_ASV17

CACCGCGGTTATACGAGAGGCCCTAGTTGATAAATACGGCGTAAAGAGTGGTTAGAGGAC

TCCCATAATAAAGCCAAAGACCCTCCGGGCCGTCACACGCATACGAGGGCACGAAGTTCA

CACACGAAAGTAGCTTTAAATTACCCGACGCCACGAAAGCTAAGAAA

>546_Sample_ASV18

CACCGCGGTTATACGAGAAACCCTAGTTGACAAATACGGCGTAAAGAGTGGTTAAGGGGT

ATCAAAAATAAAGTCAAACATTTTCCTAGTTGTTATAAGCATTTCGAGAACATGAAGCCC

AACCACGAAAGTAACTTTACCTTCACCTGATCCCACGAAAGCTAAGAAA

>546_Sample_ASV19

CACCGCGGTTATACGAGAAACCCTAGTTGACAAATACGGCGTAAAGAGTGGTTAAGGGGT

ATCAAAAATAAAGTCAAACATTTTCCTAGTTGTTATAAGCATTTCGAGAACATGAAGCCC

AACCACGAAAGTAACTTTACCTTCACCTGACCCCACGAAAGCTAAGAAA

>546_Sample_ASV20

CACCGCGGTTATACGAAAGACCCCAGTTTATAGACACGGCGTAAAGGGTGGTTATAGGAC

AAACTAACATAGAGCCAAAGACCCCCTAAGCCGTCATACGCTCACGAGGGCTCGAAGCCC

CTATACGAAAGTAGCTCTACCAAAAAATGCTCGACTCCACGAAAGCTGGGACA

>546_Sample_ASV21

CACCGCGGTTATACGAGAGACCCTAATTGATAGGCACGGCGTAAAGAGTGGTTTAGGGTA

ACCCATAAATAAAGCTAAAGACCTTCTAAGCCGTCACACGCACACCGAAGGCACGAGACC

CCAACACGAAAGTAGCTTTACTTCTACCCCTGACGCCACGAAAGCTAAGAAA

>546_Sample_ASV22

CACCGCGGTTATACGAAAGACCCTAGTTGATGGATCACGGCGTAAAGGGTGGTTAAGGGA

TATAAAAATAAAGCTAAAAATCCTCTTAGCTGTCATACGCTTTCCGAAGACATGAGGCCC

AATTACGAAAGTAGCTTTAAACCACCCTGACACCACGAAAGCCAAGAAA

>546_Sample_ASV23

CACCGCGGTTATACGAAAGACCCCAGTTTATAGATGCGGCGTAAAGGGTGGTTAAGAGAA

AATCAAAATAAGGCCAAAGATCCCCTAAGCAGTCATACGCTCCACGAGGACACGAAACCC

AAATACGAAAGTTGCCTTAATAAACTTCACCTGATCCCACGAAAGCTGAAAAA

>546_Sample_ASV24

CACCGCGGTTATACGAAAGGCTCTAGTTTATAAATACGGCGTAAAGGGTGGTTAAGGAAA

AATTAAAATAATGCTAAAGACCCCCTAAGCAGTTATACGCTTCACGGAGGCACGAAACCC

AAATACGAAAGTTGCCTTAATAAACTTTACCTGACCCCACGAAAGCTGAGAAA

>546_Sample_ASV25

CACCGCGGTTATACGAAAGGCTCTAGTTTATAAATACGGCGTAAAGGGTGGTTAAGGAAA

AATTAAAATAATGCTAAAGACCCCCTAAGCAGTTATACGCTTCACGGAGGCACGAAACCC

AAATACGAAAGTTGCCTTAATAAACTTTACCTGACCCCACGAAAGCTGAAAAA

>546_Sample_ASV26

CACCGCGGTTATACGAGAGACTCTAGTTGATACCAACGGCGTAAAGAGTGGTTTGGGATG

CCCCTTAAAATAAAGCCAAAGACCTCCCAAGCCGTCAAACGCACCCCGGAGGCACGAAGC

CCCAACGCGAAAGCAACTTTACCTCCCTCCCGACGCCACGAAAGCTAAGAAA

>546_Sample_ASV27

CACCGCGGTTATACGAGAGACCCTAGTTGATAGCTACGGCGTAAAGCGTGGTTAGGAGAA

CAAGATAAATAAAGTCAAACAATCTCTCGGCCGTTATACGTTATCTGAGAATATGAAGTC

CTACCACGAAAGTAACTTTAATTTTTCTGACCCCACGAAAACTGAGGAA

>546_Sample_ASV28

CACCGCGGTTATACGAGAGACCCTAGTTGATAGCTACGGCGTAAAGCGTGGTTAGGAGAA

CAAGATAAATAAAGTCAAACAATCTCTCGGCCGTTATACGTTATCTGAGAATATGAAGTC

CTACCACGAAAGTAACTTTAATTTTTCTGACCCCACGAAAGCTGTGAAA

>546_Sample_ASV29

CACCGCGGTTATACGAGAGGCCCTAGTTGATAACTACCGGCGTAAAGAGTGGTTATGGAA

AAATATTTAATAAAGCCGAACACCCCCTCAGCCGTCATACGCACCTGGGAGCACGAAGAC

CTACTGCGAAAGCAGCTTTAATTATGCCTGACCCCACGACAGCTAAGAAA

>546_Sample_ASV30

CACCGCGGTTATACGAGAGGCCCAAGTTGATTATTACCGGCGTAAAGCGTGGTTAAGATT

TTTATAGGACTAAAGCCGAACCCCCTCAGAGCTGTTATACGCACCCGAAGGTTAGAAGTT

CAATCACGAAAGTGGCTTTACAACCCCGAACCCACGAAAGCTAAGATA

>546_Sample_ASV31

CACCGCGGTTATACGAGAGGCCCAAGTTGATACTCCACGGCGTAAAGAGTGGTTAGAAAA

GACCTACTACTAAAGCCGAACGCCTTCAAAGCTGTTATACGCATCCGAAGGTGAGAAGCC

CACCCACGAAAGTGGCTTTACAACCTTGACTCCACGAAAGCTATGACA

>546_Sample_ASV32

CACCGCGGTTATACGAGAGACCCCAGTTGATAAACACGGCGTAAAGAGTGGTTAAGGGCA

CAAAACAAATAAAGTCAAACAACCTCTTAGCTGTTATACGCATTATGAGGATATGAAGCC

CCCCTACGAAAGTAACTTTACCAACCCCTGACCCCACGAAAGCTAAGAAA

>546_Sample_ASV33

CACCGCGGTTATACGAGAGACCCCAGTTGATAAACACGGCGTAAAGAGTGGTTAAGGGCA

CAAAACAAATAAAGTCAAACAACCTCTTAGCTGTTATACGCATTATGAGGATATGAAGCC

CCCCTACGAAAGTAACTTTACCAACCCCTGACCCCACGAAAGCTGTGAAA

>546_Sample_ASV34

CACCGCGGTTATACGAGAGACCCTAGTTGATATATACGGCGTAAAGAGTGGTTTGGAATA

ACCAAGTAATATAGCCAAAGACCTCCCAAGCCGTCACACGCACCCCGGAGGCACGAAGCC

CAAACACGAAAGTAGCTTTATTAACTTACCGACGCCACGAAAGCTAAGAAA

>546_Sample_ASV35

CACCGCGGTTATACGAGAGACCCTAGTTGATATATACGGCGTAAAGAGTGGTTTGGAATA

ACCAAGTAATATAGCCAAAGACCTCCCAAGCCGTCACACGCACCCCGGAGGCACGAAGCC

CAAACACGAAAGTAGCTTTATTAACTTACCGACGCCACGAAAGCTGTGAAA

>546_Sample_ASV36

CACCGCGGTTATACGAAAGACCCTAGTTTATAGGCACGGCGTAAAGGGTGGTTAGGGGAT

AAACAAAATAAAGCCAAAGACCTTCTAAGTCGTCATACACTCATGAAGGCACGAAGCCCA

AATACGAAAGTAACTTTACAACTTAGACCCGACCCCACGAAAGCTGTGAAA

>546_Sample_ASV37

CACCGCGGTTATACGAAAGACCCTAGTTTATAGGCACGGCGTAAAGGGTGGTTAGGGGAT

AAACAAAATAAAGCCAAAGACCTTCTAAGTCGTCATACACTCATGAAGGCACGAAGCCCA

AATACGAAAGTAACTTTACAACTTAGACCCGACTCCACGAAAGCTGTGAAA

>546_Sample_ASV38

CACCGCGGTTATACGAAAGACCCTAGTTTATAGGCACGGCGTAAAGGGTGGTTAGGGGAT

AAACAAAATAAAGCCAAAGACCTTCTAAGTCGTCATACACTCATGAAGGCACGAAGCCCA

AATACGAAAGTAACTTTACAACTTAGACCCGACTCCACGAAAGCTGGGGGA

>546_Sample_ASV39

CACCGCGGTTATACGAGAGGCCCAAGTTGATAGACATCGGCGTAAAGAGTGGTTAAGGAA

GTCTCAAAACTAAAGCCGAATGCCTTCAAGACTGTTATACGTTTCCGAAGGTAAGAAGCC

CCACTACGAAAGTGGCTTTACTCCACCTGACTCCACGAAAGCTGTGAAA

>546_Sample_ASV40

CACCGCGGTTATACGAGAGACTCCAGTTGATAGCTACGGCGTAAAGAGTGGTTTGGGGCC

CGCCCAAAATAAAGCCAAAGACCTCCCAAGCCGTCAAACGCACCCCGGAGGCACGAAGTC

CTAACGCGAAAGCAACTTTACCTTCCCCGACGCCACGAAAGCTAAGAAA

>546_Sample_ASV41

CACCGCGGTTATACGAAAGACCCTAGTTGATAGCTACGGCGTAAAGGGTGGTTAAGGTAT

ATAACAAATAAAGCTAAAGAGCCTCCAAGCCGTCGTACGCATTCTGAGAGCTCGAAACCC

TAACACGAAAGTAGCTTTAAAAAATAACACCTGACCCCACGAAAGCTAAGAAA

>546_Sample_ASV42

CACCGCGGTCATACGATTAACCCAAATTAATAGATCCACGGCGTAAAGAGTGTTTAAGaa

aaaaaaCCACAATAGAGTTAAATTATAACTAAGCTGTAAAAAGCCCTAGTTAAAATAAAA

TAACCCACGAAAGTGACTCTAATAATCCTGACACACGATAGCTAGGACC

>546_Sample_ASV43

CACCGCGGTTATACGAAAGACCCTAGTTGATAGATCACGGCGTAAAGGGTGGTTAAGGAG

AACAAAAATAAAGCTAAAAATCTTCTAAGCTGTCATACGCTTTCCGAAGACATGAGACCC

AACCACGAAAGTAGCTTTAAACTTTCCTGACACCACGAAAGCCAAGAAA

>551_Sample_ASV1

CACCGCGGTTATACGAGAGGCCCAAGTTGACAGACATCGGCGTAAAGAGTGGTTAAGGAA

AACCTTCAACTAAAGTCGAACGCTCTCAAGACTGTCGTACGTTTCCGAGAGTATGAAGCC

CCACCACGAAAGTGACTTTACCTCACCTGACTCCACGAAAGCTGCGAAA

>551_Sample_ASV2

CACCGCGGTTATACGAAAGACCCTAGTTGATGGGTGCGGCGTAAAGGGTGGTTAGGGACT

ACAAAAAATAGAGCTAAAGATCCTCTAGGTCGTCATACACATCACGAGGAAACGAAGCCC

AAATACGAAAGTAGCTCTAAATATAATCCTGACGCCACGAAAGCCAAGAAA

>551_Sample_ASV3

CACCGCGGTTATACGAAAGACCCTAGTTGATGGGTGCGGCGTAAAGGGTGGTTAGGGACT

ACAAAAAATAGAGCTAAAGATCCTCTAGGTCGTCATACACATCACGAGGAAACGAAGCCC

AAGTACGAAAGTAGCTCTAAATATAATCCTGACGCCACGAAAGCCAAGAAA

>551_Sample_ASV4

CACCGCGGTTATACGAGAGACCCTAGTTGATTAAAGCGGCGTAAAGAGTGGTTATGGAAC

TATTCTTTTAAAGCAGAAAACCTCTCAAACTGTTATACGCACCCAGAGGTCTGAATCCCT

CACACGAAAGTGACTTTATTTATGCCTACCAGAACCCACGAAAGCTATGACA

>551_Sample_ASV5

CACCGCGGTTATACGAGAGACCCTAGTTGATTAAAGCGGCGTAAAGAGTGGTTATGGAAC

TATTCTTTTAAAGCAGAAAACCTCTCAAACTGTTATACGCACCCAGAGGTCTGAATCCCT

CACACGAAAGTGACTTTATTTATGCCTACCAGAACCCACGAAAGCTGGGACA

>551_Sample_ASV6

CACCGCGGTTATACGAAAGACCCTAGTTTATAGGTACGGCGTAAAGGGTGGTTAGGGGAC

AAACAAAATAAAGCCAAAGACCTCCTAAGCCGTCATACGCTCACGGAAGCACGAAGCCCA

AACACGAAAGTAGCTTTACCGAACATGCCCGACTCCACGAAAGCTGGGGAA

>551_Sample_ASV7

CACCGCGGTTATACGAGAGGCCCAAGTTGACAGACACCGGCGTAAAGAGTGGCTAGGGAA

AAATTACTACTAAAGCCGAACACCTTCAGAACTGTCATACGTTCCCGAAGATAAGAAGCC

CCACCACGAAAGTGGCTTTATATTACCCGACCCCACGAAAGCTGTGAAA

>551_Sample_ASV8

CACCGCGGTTAAACGAGAGACCCTAGTTGATCATCACGGCGTAAAGAGTGGTTAGGGGAT

TATCATAAATAAAGCCAAAGACCTCCCAAGCTGTCGCACGCATTCCGGAGGCGCGAAGCC

CACACACGAAAGTAGCTTTAACTATTGACCCTGATGCCACGAAAGCTAAGAAA

>551_Sample_ASV9

CACCGCGGTTATACGAGAGGCCCAAGTTGATAAGCCCGGCGTAAAGAGTGGTTATGGAGA

ATAAAACACTAAAGCTAAAGACCCCCTAGGCTGTTATACGCACCTGGCGGCTCGAACCAC

CTATACGAAAGTAGCTTTACCCCCCTTCCACCAGAATCCACGACAGCTGGGGCA

>551_Sample_ASV10

CACCGCGGTTATACGAGAGGCCCTAGTTGATAGCCACGGCGCAAAGAGTGGTTAAGGAGC

CCCACTAAATAAAGTCGAACACTTCCTAGGCCGTTATACGTTTTCTAGAAGCACGAAACC

CAATTACACGAAAGCAACTTTATACTAAACAACCTGACCCCACGAAAGCTAAGAAA

>551_Sample_ASV11

CACCGCGGTTATACGAGAGGCCCAAGTTGACAGACATCGGCGTAAAGAGTGGTTAAGGAA

ACCCCATTACTAAAGCCGAACGCCCTCAGAACTGTCATACGTTCCCGAAGGTAAGAAGCC

CCACTACGAAAGTGGCTTTAACCACACCCGACCCCACGAAAGCTGTGAAA

>551_Sample_ASV12

CACCGCGGTTATACGAGAGGCCCTAGTTGATAAATACGGCGTAAAGAGTGGTTAGAGGAC

TCCCATAATAAAGCCAAAGACCCCCCGGGCCGTCACACGCATACGAGGGCACGAAGTTCA

CACACGAAAGTAGCTTTAAATTACCCGACGCCACGAAAGCTAAGAAA

>551_Sample_ASV13

CACCGCGGTTATACGAGAAACCCTAGTTGACAAATACGGCGTAAAGAGTGGTTAAGGGGT

ATCAAAAATAAAGTCAAACATTTTCCTAGTTGTTATAAGCATTTCGAGAACATGAAGCCC

AACCACGAAAGTAACTTTACCTTCACCTGACCCCACGAAAGCTAAGAAA

>551_Sample_ASV14

GACCGCGGTTATACGAGAGACTCTAGTTGATAGATACGGCGTAAAGAGTGGTTTGGGATA

AACTATAAATAAAGCCAAAGACCTTCCAAGCTGTTAAACGCATACCGAAGGCACGAAGCC

CCAACACGAAAGTAGCTTTACTACTACCCCCGATGCCACGAAAGCTAAGACA

>551_Sample_ASV15

CACCGCGGTTATACGAGAGACCCTAGTTGATAAATACGGCGTAAAGAGTGGTTATGGGAA

AAACAAATAAAGTCAAACAACCTCTTAGCTGTTATACGCATTATGAGAGCATGAAGCCCC

CTCACGAAAGTAACTTTAATACCTCCTGACCCCACGAAAGCTAAGAAA

>551_Sample_ASV16

CACCGCGGTCATACGATTAACCCAAGTTAATAGGCACACGGCGTAAAGCGTGTTTAAGCA

CTATGCCAAATAAAGTTAAACTCCAATTAAGCTGTAAAAAGCCATAATCAAGACGAAAAT

AAATAACGAAAGTAACTTTACAACCGCTGAAACACGATAGCTAAGATC

>551_Sample_ASV17

CACCGCGGTTATACGAAAGACCCTAGTTTATAGACACGGCGTAAAGGGTGGTTCGGGACT

AAACAAAATAAAGCCAAAGACCTCCTAAGCCGTCATACGCTCACGGAAGCACGAAGCCCA

AGTACGAAAGTAGCTTTACAAAACATGCCTGACTCCACGAAAGCTAGGAAA

>551_Sample_ASV18

CACCGCGGTTATACGAAAGACCCTAGTTGATAGGCACGGCGTAAAGGGTGGTTAGGGATA

TATCAAAAATAAAGTTAAAGAGCCTCTAAGCTGTCGCACGCATCCCGAGAGCTCGAGACC

CAGACACGAAAGTAACTTTAAAATACCTCACCTGACCCCACGAAAGCTAAGAAA

>551_Sample_ASV19

CACCGCGGTTATACGAAAGACCCTAGTTGATAGCCACGGCGTAAAGGGTGGTTAAGGTAT

ACAACAAATAAAGCTAAATAGCCTCTAGGCCGTCGCACGCATTCCGAGAGCTCGAAACCC

AAAAACGAAAGTAGCTTTAAAATAAACAGACCTGACCCCACGAAAGCTAAGAAA

>551_Sample_ASV20

CACCGCGGTTATACGAGAGGCCCAAGTTGATACTCCACGGCGTAAAGAGTGGTTAGAAAA

GACCTACTACTAAAGCCGAACGCCTTCAAAGCTGTTATACGCATCCGAAGGTGAGAAGCC

CACCCACGAAAGTGGCTTTACAACCTTGACTCCACGAAAGCTGGGACA

>551_Sample_ASV21

CACCGCGGTTATACGAGAGGCCCAAGTTGATACTCCACGGCGTAAAGAGTGGTTAGAAAA

GACCTACTACTAAAGCCGAACGCCTTCAAAGCTGTTATACGCATCCGAAGGTGAGAAGCC

CACCCACGAAAGTGGCTTTACAACCTTGACTCCACGAAAGCTATGACA

>551_Sample_ASV22

CACCGCGGTTATACGAGAGACCCCAGTTGATAAACACGGCGTAAAGAGTGGTTAAGGGCA

CAAAACAAATAAAGTCAAACAACCTCTTAGCTGTTATACGCATTATGAGGATATGAAGCC

CCCCTACGAAAGTAACTTTACCAACCCCTGACCCCACGAAAGCTAAGAAA

>551_Sample_ASV23

CACCGCGGTTATACGAAAGACCCTAGTTTATAGGCACGGCGTAAAGGGTGGTTAGGGGAT

AAACAAAATAAAGCCAAAGACCTTCTAAGTCGTCATACACTCATGAAGGCACGAAGCCCA

AATACGAAAGTAACTTTACAACTTAGACCCGACTCCACGAAAGCTGGGGGA

>551_Sample_ASV24

CACCGCGGTTATACGAAAGACCCTAGTTGATAGATCACGGCGTAAAGGGTGGTTAAGGAG

AACAAAAATAAAGCTAAAAATCTTCTAAGCTGTCATACGCTTTCCGAAGACATGAGACCC

AACCACGAAAGTAGCTTTAAACTTTCCTGACACCACGAAAGCCAAGAAA

>572_Sample_ASV1

CACCGCGGTTATACGAAAGACCCTAGTTGATGGGTGCGGCGTAAAGGGTGGTTAGGGACT

ACAAAAAATAGAGCTAAAGATCCTCTAGGTCGTCATACACATCACGAGGAAACGAAGCCC

AAATACGAAAGTAGCTCTAAATATAATCCTGACGCCACGAAAGCCAAGAAA

>572_Sample_ASV2

CACCGCGGTTATACGAGAGACCCTAGTTGATATACACGGCGTAAAGAGTGGTTTGGGGTA

TATAATAAATAAAGCCAAAGACCTTCTAGGCCGTTGAACGCACCACGAAGGAGCGAAGTC

CCAATACGAAAGTAGCTTTATAAAACCTGACGCCACGAAAGCTAAGAAA

>572_Sample_ASV3

CACCGCGGTTATACGAGAGGCCCAAGTTGACAGACACCGGCGTAAAGAGTGGCTAGGGAA

AAATTACTACTAAAGCCGAACACCTTCAGAACTGTCATACGTTCCCGAAGATAAGAAGCC

CCACCACGAAAGTGACTTTATATTACCCGACCCCACGAAAGCTGTGAAA

>572_Sample_ASV4

CACCGCGGTTATACGAGAGGCCCAAGTTGACAGACACCGGCGTAAAGAGTGGCTAGGGAA

AAATTACTACTAAAGCCGAACACCTTCAGAACTGTCATACGTTCCCGAAGATAAGAAGCC

CCACCACGAAAGTGGCTTTATATTACCCGACCCCACGAAAGCTGTGAAA

>572_Sample_ASV5

CACCGCGGTTATACGAGAGGCCCAAGTTGACAGACACCGGCGTAAAGAGTGGCTAGGGAA

AAATTACTACTAAAGCCGAACACCTTCAGAACTGTCATACGTTCCCGAAGATAAGAAGCC

CCACCACGAAAGTGGCTTTACAATGCCTGACCCCACGAAAGCTGTGAAA

>572_Sample_ASV6

CACCGCGGTTATACGAGAGGCCCAAGTTGATAAGCCCGGCGTAAAGAGTGGTTATGGAGA

ATAAAACACTAAAGCTAAAGACCCCCTAGGCTGTTATACGCACCTGGCGGCTCGAACCAC

CTATACGAAAGTAGCTTTACCCCCCTTCCACCAGAATCCACGACAGCTGGGGCA

>572_Sample_ASV7

CACCGCGGTTATACGAGAGGCCCAAGTTGACAGACATCGGCGTAAAGAGTGGTTAAGGAA

ACCCCATTACTAAAGCCGAACGCCCTCAGAACTGTCATACGTTCCCGAAGGTAAGAAGCC

CCACTACGAAAGTGGCTTTAACCACACCCGACCCCACGAAAGCTGTGAAA

>572_Sample_ASV8

CACCGCGGTTATACGAGAAACCCTAGTTGACAAATACGGCGTAAAGAGTGGTTAAGGGGT

ATCAAAAATAAAGTCAAACATTTTCCTAGTTGTTATAAGCATTTCGAGAACATGAAGCCC

AACCACGAAAGTAACTTTACCTTCACCTGACCCCACGAAAGCTAAGAAA

>572_Sample_ASV9

CACCGCGGTTATACGAAAGACCCCAGTTTATAGACACGGCGTAAAGGGTGGTTATAGGAC

AAACTAACATAGAGCCAAAGACCCCCTAAGCCGTCATACGCTCACGAGGGCTCGAAGCCC

CTATACGAAAGTAGCTCTACCAAAAAATGCTCGACTCCACGAAAGCTGGGACA

>572_Sample_ASV10

CACCGCGGTTATACGAGAGACCCTAGTTGATAAATACGGCGTAAAGAGTGGTTATGGGAA

AAACAAATAAAGTCAAACAACCTCTTAGCTGTTATACGCATTATGAGAGCATGAAGCCCC

CTCACGAAAGTAACTTTAATACCTCCTGACCCCACGAAAGCTAAGAAA

>572_Sample_ASV11

AACCGCGGTTATACGAGAGACCCTAATTGATAGCTCACGGCGTAAAGGGTGGTTTGGGGA

AACCTCAAAATAAAGCTAAAGACCTTCTAAGCCGTTACACGCATACCGAAGGCACGAGGC

CCTAACACGAAAGTAGCTTTACTATTACCCCTGACGCCACGAAAGCTAAGAAA

>572_Sample_ASV12

CACCGCGGTTATACGAAAGACCCTAGTTGATGGATCACGGCGTAAAGGGTGGTTAAGGGA

TATAAAAATAAAGCTAAAAATCCTCTTAGCTGTCATACGCTTTCCGAAGACATGAGGCCC

AATTACGAAAGTAGCTTTAAACCACCCTGACACCACGAAAGCCAAGAAA

>572_Sample_ASV13

CACCGCGGTTATACGAGAGACCCTAGTTGATAGCTACGGCGTAAAGCGTGGTTAGGAGAA

CAAGATAAATAAAGTCAAACAATCTCTCGGCCGTTATACGTTATCTGAGAATATGAAGTC

CTACCACGAAAGTAACTTTAATTTTTCTGACCCCACGAAAACTGAGGAA

>572_Sample_ASV14

CACCGCGGTTATACGAGAGGCCCAAGTTGATACTCCACGGCGTAAAGAGTGGTTAGAAAA

GACCTACTACTAAAGCCGAACGCCTTCAAAGCTGTTATACGCATCCGAAGGTGAGAAGCC

CACCCACGAAAGTGGCTTTACAACCTTGACTCCACGAAAGCTATGACA

>572_Sample_ASV15

CACCGCGGTTATACGAGAGACCCCAGTTGATAAACACGGCGTAAAGAGTGGTTAAGGGCA

CAAAACAAATAAAGTCAAACAACCTCTTAGCTGTTATACGCATTATGAGGATATGAAGCC

CCCCTACGAAAGTAACTTTACCAACCCCTGACCCCACGAAAGCTAAGAAA

>572_Sample_ASV16

CACCGCGGTTATACGAGAGACCCTAGTTGATATATACGGCGTAAAGAGTGGTTTGGAATA

ACCAAGTAATATAGCCAAAGACCTCCCAAGCCGTCACACGCACCCCGGAGGCACGAAGCC

CAAACACGAAAGTAGCTTTATTAACTTACCGACGCCACGAAAGCTAAGAAA

>572_Sample_ASV17

CACCGCGGTTATACGAAAGACCCTAGTTTATAGGCACGGCGTAAAGGGTGGTTAGGGGAT

AAACAAAATAAAGCCAAAGACCTTCTAAGTCGTCATACACTCATGAAGGCACGAAGCCCA

AATACGAAAGTAACTTTACAACTTAGACCCGACTCCACGAAAGCTGGGGGA

>572_Sample_ASV18

CACCGCGGTTATACGAGAGACCCTAATTAATAGATACGGCGTAAAGAGTGGTTTAGGACA

AAAATTAATAAAGCCGAAGTTTCTCCTAGCCGTTGTACGCACTCTGAGAACACGAAGCCC

CACTACGAAAGTAGCTTTACCTATTATTTCCTGACCCCACGAAAGCTAAGAAA

>572_Sample_ASV19

CACCGCGGTTATACGAGAGGCCCAAGTTGATAGACATCGGCGTAAAGAGTGGTTAAGGAA

GTCTCAAAACTAAAGCCGAATGCCTTCAAGACTGTTATACGTTTCCGAAGGTAAGAAGCC

CCACTACGAAAGTGGCTTTACTCCACCTGACTCCACGAAAGCTGTGAAA

>572_Sample_ASV20

CACCGCGGTTATACGAAAGACCCTAGTTGATAGATCACGGCGTAAAGGGTGGTTAAGGAG

AACAAAAATAAAGCTAAAAATCTTCTAAGCTGTCATACGCTTTCCGAAGACATGAGACCC

AACCACGAAAGTAGCTTTAAACTTTCCTGACACCACGAAAGCCAAGAAA

>576_Sample_ASV1

CACCGCGGTTATACGAAAGACCCTAGTTGATGGGTGCGGCGTAAAGGGTGGTTAGGGACT

ACAAAAAATAGAGCTAAAGATCCTCTAGGTCGTCATACACATCACGAGGAAACGAAGCCC

AAATACGAAAGTAGCTCTAAATATAATCCTGACGCCACGAAAGCCAAGAAA

>576_Sample_ASV2

CACCGCGGTTATACGAGAGACCCTAGTTGATTAAAGCGGCGTAAAGAGTGGTTATGGAAC

TATTCTTTTAAAGCAGAAAACCTCTCAAACTGTTATACGCACCCAGAGGTCTGAATCCCT

CACACGAAAGTGACTTTATTTATGCCTACCAGAACCCACGAAAGCTATGACA

>576_Sample_ASV3

CACCGCGGTTATACGAGAGACCCTAGTTGATTAAAGCGGCGTAAAGAGTGGTTATGGAAC

TATTCTTTTAAAGCAGAAAACCTCTCAAACTGTTATACGCACCCAGAGGTCTGAATCCCT

CACACGAAAGTGACTTTATTTATGCCTACCAGAACCCACGAAAGCTGGGACA

>576_Sample_ASV4

CACCGCGGTTATACGAGAGACCCTAGTTGATATACACGGCGTAAAGAGTGGTTTGGGGTA

TATAATAAATAAAGCCAAAGACCTTCTAGGCCGTTGAACGCACCACGAAGGAGCGAAGTC

CCAATACGAAAGTAGCTTTATAAAACCTGACGCCACGAAAGCTAAGAAA

>576_Sample_ASV5

CACCGCGGTTATACGAGAGGCCCAAGTTGACAGACACCGGCGTAAAGAGTGGCTAGGGAA

AAATTACTACTAAAGCCGAACACCTTCAGAACTGTCATACGTTCCCGAAGATAAGAAGCC

CCACCACGAAAGTGACTTTATATTACCCGACCCCACGAAAGCTGTGAAA

>576_Sample_ASV6

CACCGCGGTTATACGAGAGGCCCAAGTTGACAGACACCGGCGTAAAGAGTGGCTAGGGAA

AAATTACTACTAAAGCCGAACACCTTCAGAACTGTCATACGTTCCCGAAGATAAGAAGCC

CCACCACGAAAGTGGCTTTATATTACCCGACCCCACGAAAGCTGTGAAA

>576_Sample_ASV7

CACCGCGGTTATACGAGAGGCCCAAGTTGATAAGCCCGGCGTAAAGAGTGGTTATGGAGA

ATAAAACACTAAAGCTAAAGACCCCCTAGGCTGTTATACGCACCTGGCGGCTCGAACCAC

CTATACGAAAGTAGCTTTACCCCCCTTCCACCAGAATCCACGACAGCTGGGGCA

>576_Sample_ASV8

CACCGCGGTTATACGAGAGGCCCTAGTTGATAGCCACGGCGCAAAGAGTGGTTAAGGAGC

CCCACTAAATAAAGTCGAACACTTCCTAGGCCGTTATACGTTTTCTAGAAGCACGAAACC

CAATTACACGAAAGCAACTTTATACTAAACAACCTGACCCCACGAAAGCTAAGAAA

>576_Sample_ASV9

CACCGCGGTTATACGAGAGGCCCAAGTTGACAGACATCGGCGTAAAGAGTGGTTAAGGAA

ACCCCATTACTAAAGCCGAACGCCCTCAGAACTGTCATACGTTCCCGAAGGTAAGAAGCC

CCACTACGAAAGTGGCTTTAACCACACCCGACCCCACGAAAGCTGTGAAA

>576_Sample_ASV10

CACCGCGGTTATACGAGAAACCCTAGTTGACAAATACGGCGTAAAGAGTGGTTAAGGGGT

ATCAAAAATAAAGTCAAACATTTTCCTAGTTGTTATAAGCATTTCGAGAACATGAAGCCC

AACCACGAAAGTAACTTTACCTTCACCTGACCCCACGAAAGCTAAGAAA

>576_Sample_ASV11

CACCGCGGTTATACGAGAGACCCTAGTTGATAAATACGGCGTAAAGAGTGGTTATGGGAA

AAACAAATAAAGTCAAACAACCTCTTAGCTGTTATACGCATTATGAGAGCATGAAGCCCC

CTCACGAAAGTAACTTTAATACCTCCTGACCCCACGAAAGCTAAGAAA

>576_Sample_ASV12

AACCGCGGTTATACGAGAGACCCTAATTGATAGCTCACGGCGTAAAGGGTGGTTTGGGGA

AACCTCAAAATAAAGCTAAAGACCTTCTAAGCCGTTACACGCATACCGAAGGCACGAGGC

CCTAACACGAAAGTAGCTTTACTATTACCCCTGACGCCACGAAAGCTAAGAAA

>576_Sample_ASV13

CACCGCGGTTATACGAAAGACCCTAGTTGATGGATCACGGCGTAAAGGGTGGTTAAGGGA

TATAAAAATAAAGCTAAAAATCCTCTTAGCTGTCATACGCTTTCCGAAGACATGAGGCCC

AATTACGAAAGTAGCTTTAAACCACCCTGACACCACGAAAGCCAAGAAA

>576_Sample_ASV14

CACCGCGGTTATACGAGAGGCCCAAGTTGATACTCCACGGCGTAAAGAGTGGTTAGAAAA

GACCTACTACTAAAGCCGAACGCCTTCAAAGCTGTTATACGCATCCGAAGGTGAGAAGCC

CACCCACGAAAGTGGCTTTACAACCTTGACTCCACGAAAGCTGGGACA

>576_Sample_ASV15

CACCGCGGTTATACGAGAGGCCCAAGTTGATACTCCACGGCGTAAAGAGTGGTTAGAAAA

GACCTACTACTAAAGCCGAACGCCTTCAAAGCTGTTATACGCATCCGAAGGTGAGAAGCC

CACCCACGAAAGTGGCTTTACAACCTTGACTCCACGAAAGCTATGACA

>576_Sample_ASV16

CACCGCGGTTATACGAGAGACCCCAGTTGATAAACACGGCGTAAAGAGTGGTTAAGGGCA

CAAAACAAATAAAGTCAAACAACCTCTTAGCTGTTATACGCATTATGAGGATATGAAGCC

CCCCTACGAAAGTAACTTTACCAACCCCTGACCCCACGAAAGCTAAGAAA

>576_Sample_ASV17

CACCGCGGTTATACGAAAGACCCTAGTTTATAGGCACGGCGTAAAGGGTGGTTAGGGGAT

AAACAAAATAAAGCCAAAGACCTTCTAAGTCGTCATACACTCATGAAGGCACGAAGCCCA

AATACGAAAGTAACTTTACAACTTAGACCCGACTCCACGAAAGCTGGGGGA

>576_Sample_ASV18

CACCGCGGTTATACGAGAGACCCTAATTAATAGATACGGCGTAAAGAGTGGTTTAGGACA

AAAATTAATAAAGCCGAAGTTTCTCCTAGCCGTTGTACGCACTCTGAGAACACGAAGCCC

CACTACGAAAGTAGCTTTACCTATTATTTCCTGACCCCACGAAAGCTAAGAAA

>576_Sample_ASV19

CACCGCGGTTATACGAGAGACCCTAATTAATAGATACGGCGTAAAGAGTGGTTTAGGACA

AAAATTAATAAAGCCGAAGTTTCTCCTAGCCGTTGTACGCACTCTGAGAACACGAAGCCC

CACTACGAAAGTAGCTTTACCTATTATTTCCTGACCCCACGAAAGCTGTGACA

>576_Sample_ASV20

CACCGCGGTTATACGAGAGGCCCAAGTTGATAGACATCGGCGTAAAGAGTGGTTAAGGAA

GTCTCAAAACTAAAGCCGAATGCCTTCAAGACTGTTATACGTTTCCGAAGGTAAGAAGCC

CCACTACGAAAGTGGCTTTACTCCACCTGACTCCACGAAAGCTGTGAAA

>576_Sample_ASV21

CACCGCGGTTATACGAGAGGCCCAAGTTGATAGACATCGGCGTAAAGAGTGGTTAAGGAA

GTCTCAAAACTAAAGCCGAATGCCTTCAAGACTGTTATACGTTTACGAAGGTAAGAAGCC

CCACTACGAAAGTGGCTTTATTCCACCTGACCCCACGAAAGCTGTGAAA

>582_Sample_ASV1

CACCGCGGTTATACGAAAGACCCTAGTTGATGGGTGCGGCGTAAAGGGTGGTTAGGGACT

ACAAAAAATAGAGCTAAAGATCCTCTAGGTCGTCATACACATCACGAGGAAACGAAGCCC

AAATACGAAAGTAGCTCTAAATATAATCCTGACGCCACGAAAGCCAAGAAA

>582_Sample_ASV2

CACCGCGGTTATACGAGAGACCCTAGTTGATTAAAGCGGCGTAAAGAGTGGTTATGGAAC

TATTCTTTTAAAGCAGAAAACCTCTCAAACTGTTATACGCACCCAGAGGTCTGAATCCCT

CACACGAAAGTGACTTTATTTATGCCTACCAGAACCCACGAAAGCTGGGACA

>582_Sample_ASV3

CACCGCGGTTATACGAGAGACCCTAGTTGATATACACGGCGTAAAGAGTGGTTTGGGGTA

TATAATAAATAAAGCCAAAGACCTTCTAGGCCGTTGAACGCACCACGAAGGAGCGAAGTC

CCAATACGAAAGTAGCTTTATAAAACCTGACGCCACGAAAGCTAAGAAA

>582_Sample_ASV4

CACCGCGGTTATACGAGAGGCCCAAGTTGACAGACACCGGCGTAAAGAGTGGCTAGGGAA

AAATTACTACTAAAGCCGAACACCTTCAGAACTGTCATACGTTCCCGAAGATAAGAAGCC

CCACCACGAAAGTGACTTTATATTACCCGACCCCACGAAAGCTGTGAAA

>582_Sample_ASV5

CACCGCGGTTATACGAGAGGCCCAAGTTGACAGACACCGGCGTAAAGAGTGGCTAGGGAA

AAATTACTACTAAAGCCGAACACCTTCAGAACTGTCATACGTTCCCGAAGATAAGAAGCC

CCACCACGAAAGTGGCTTTATATTACCCGACCCCACGAAAGCTGTGAAA

>582_Sample_ASV6

CACCGCGGTTATACGAGAGGCCCAAGTTGATAAGCCCGGCGTAAAGAGTGGTTATGGAGA

ATAAAACACTAAAGCTAAAGACCCCCTAGGCTGTTATACGCACCTGGCGGCTCGAACCAC

CTATACGAAAGTAGCTTTACCCCCCTTCCACCAGAATCCACGACAGCTGGGGCA

>582_Sample_ASV7

CACCGCGGTTATACGAGAGGCCCAAGTTGACAGACATCGGCGTAAAGAGTGGTTAAGGAA

ACCCCATTACTAAAGCCGAACGCCCTCAGAACTGTCATACGTTCCCGAAGGTAAGAAGCC

CCACTACGAAAGTGGCTTTAACCACACCCGACCCCACGAAAGCTGTGAAA

>582_Sample_ASV8

CACCGCGGTTATACGAGAGGCCCAAGTTGATAGATATCGGCGTAAAGAGTGGTTAAGGAA

ACCCCGTTACTAAAGCCGAACGCCCTCAGAACTGTCATACGTTCCCGAAGGTAAGAAGCC

CCACTACGAAAGTGGCTTTAACCACACCCGACCCCACGAAAGCTGTGAAA

>582_Sample_ASV9

CACCGCGGTTATACGAGAAACCCTAGTTGACAAATACGGCGTAAAGAGTGGTTAAGGGGT

ATCAAAAATAAAGTCAAACATTTTCCTAGTTGTTATAAGCATTTCGAGAACATGAAGCCC

AACCACGAAAGTAACTTTACCTTCACCTGACCCCACGAAAGCTAAGAAA

>582_Sample_ASV10

CACCGCGGTTATACGAGAGACCCTAGTTGATAAATACGGCGTAAAGAGTGGTTATGGGAA

AAACAAATAAAGTCAAACAACCTCTTAGCTGTTATACGCATTATGAGAGCATGAAGCCCC

CTCACGAAAGTAACTTTAATACCTCCTGACCCCACGAAAGCTAAGAAA

>582_Sample_ASV11

AACCGCGGTTATACGAGAGACCCTAATTGATAGCTCACGGCGTAAAGGGTGGTTTGGGGA

AACCTCAAAATAAAGCTAAAGACCTTCTAAGCCGTTACACGCATACCGAAGGCACGAGGC

CCTAACACGAAAGTAGCTTTACTATTACCCCTGACGCCACGAAAGCTAAGAAA

>582_Sample_ASV12

CACCGCGGTTATACGAAAGACCCTAGTTGATGGATCACGGCGTAAAGGGTGGTTAAGGGA

TATAAAAATAAAGCTAAAAATCCTCTTAGCTGTCATACGCTTTCCGAAGACATGAGGCCC

AATTACGAAAGTAGCTTTAAACCACCCTGACACCACGAAAGCCAAGAAA

>582_Sample_ASV13

CACCGCGGTTATACGAGAGGCCCAAGTTGATACTCCACGGCGTAAAGAGTGGTTAGAAAA

GACCTACTACTAAAGCCGAACGCCTTCAAAGCTGTTATACGCATCCGAAGGTGAGAAGCC

CACCCACGAAAGTGGCTTTACAACCTTGACTCCACGAAAGCTATGACA

>582_Sample_ASV14

CACCGCGGTTATACGAGAGACCCCAGTTGATAAACACGGCGTAAAGAGTGGTTAAGGGCA

CAAAACAAATAAAGTCAAACAACCTCTTAGCTGTTATACGCATTATGAGGATATGAAGCC

CCCCTACGAAAGTAACTTTACCAACCCCTGACCCCACGAAAGCTAAGAAA

>582_Sample_ASV15

CACCGCGGTTATACGAGAGACCCTAGTTGATATATACGGCGTAAAGAGTGGTTTGGAATA

ACCAAGTAATATAGCCAAAGACCTCCCAAGCCGTCACACGCACCCCGGAGGCACGAAGCC

CAAACACGAAAGTAGCTTTATTAACTTACCGACGCCACGAAAGCTAAGAAA

>582_Sample_ASV16

CACCGCGGTTATACGAAAGACCCTAGTTTATAGGCACGGCGTAAAGGGTGGTTAGGGGAT

AAACAAAATAAAGCCAAAGACCTTCTAAGTCGTCATACACTCATGAAGGCACGAAGCCCA

AATACGAAAGTAACTTTACAACTTAGACCCGACTCCACGAAAGCTGGGGGA

>582_Sample_ASV17

CACCGCGGTTATACGAGAGACCCTAATTAATAGATACGGCGTAAAGAGTGGTTTAGGACA

AAAATTAATAAAGCCGAAGTTTCTCCTAGCCGTTGTACGCACTCTGAGAACACGAAGCCC

CACTACGAAAGTAGCTTTACCTATTATTTCCTGACCCCACGAAAGCTAAGAAA

>582_Sample_ASV18

CACCGCGGTTATACGAGAGGCCCAAGTTGATAGACATCGGCGTAAAGAGTGGTTAAGGAA

GTCTCAAAACTAAAGCCGAATGCCTTCAAGACTGTTATACGTTTACGAAGGTAAGAAGCC

CCACTACGAAAGTGGCTTTATTCCACCTGACCCCACGAAAGCTGTGAAA

>582_Sample_ASV19

CACCGCGGTTATACGAGAGGCCCAAGTTGATAGACATCGGCGTAAAGAGTGGTTAAGGAA

GTCTCAAAACTAAAGCCGAATGCCTTCAAGACTGTTATACGTTTCCGAAGGTAAGAAGCC

CCACTACGAAAGTGGCTTTATTCCACCTGACCCCACGAAAGCTGTGAAA

>582_Sample_ASV20

CACCGCGGTTATACGAGAGGCCCAAGTTGATAGACATCGGCGTAAAGAGTGGTTAAGGAA

GTCTCAAAACTAAAGCCGAATGCCTTCAAGACTGTTATACGTTTCCGAAGGTAAGAAGCC

CCACTACGAAAGTGGCTTTACTCCACCTGACTCCACGAAAGCTGTGAAA

>592_Sample_ASV1

CACCGCGGTTATACGAAAGACCCTAGTTGATGGGTGCGGCGTAAAGGGTGGTTAGGGACT

ACAAAAAATAGAGCTAAAGATCCTCTAGGTCGTCATACACATCACGAGGAAACGAAGCCC

AAATACGAAAGTAGCTCTAAATATAATCCTGACGCCACGAAAGCCAAGAAA

>592_Sample_ASV2

CACCGCGGTTAAACGAGAGGCCCTAGTTGATGGTGCAACGGCGTAAAGGGTGGTTAAGGA

TAGCGAAATAATAAAGTCAAATGGCCCTCTGGCTGTCATACGCTTCTAGGAGTCCGAAGC

CCAGTATACGAAAGTAGCTTTAAGAAAGTCCACCTGACCCCACGAAAACTGAGGAA

>592_Sample_ASV3

CACCGCGGTTATACGAGAGACCCTAGTTGATTAAAGCGGCGTAAAGAGTGGTTATGGAAC

TATTCTTTTAAAGCAGAAAACCTCTCAAACTGTTATACGCACCCAGAGGTCTGAATCCCT

CACACGAAAGTGACTTTATTTATGCCTACCAGAACCCACGAAAGCTGGGACA

>592_Sample_ASV4

CACCGCGGTTATACGAAAGACCCTAGTTTATAGGTACGGCGTAAAGGGTGGTTAGGGGAC

AAACAAAATAAAGCCAAAGACCTCCTAAGCCGTCATACGCTCACGGAAGCACGAAGCCCA

AACACGAAAGTAGCTTTACCGAACATGCCCGACTCCACGAAAGCTGGGGAA

>592_Sample_ASV5

CACCGCGGTTATACGAGAGACCCTAGTTGATATACACGGCGTAAAGAGTGGTTTGGGGTA

TATAATAAATAAAGCCAAAGACCTTCTAGGCCGTTGAACGCACCACGAAGGAGCGAAGTC

CCAATACGAAAGTAGCTTTATAAAACCTGACGCCACGAAAGCTAAGAAA

>592_Sample_ASV6

CACCGCGGTTATACGAGAGGCCCAAGTTGACAGACACCGGCGTAAAGAGTGGCTAGGGAA

AAATTACTACTAAAGCCGAACACCTTCAGAACTGTCATACGTTCCCGAAGATAAGAAGCC

CCACCACGAAAGTGACTTTATATTACCCGACCCCACGAAAGCTGTGAAA

>592_Sample_ASV7

CACCGCGGTTATACGAGAGGCCCAAGTTGACAGACACCGGCGTAAAGAGTGGCTAGGGAA

AAATTACTACTAAAGCCGAACACCTTCAGAACTGTCATACGTTCCCGAAGATAAGAAGCC

CCACCACGAAAGTGGCTTTATATTACCCGACCCCACGAAAGCTGTGAAA

>592_Sample_ASV8

CACCGCGGTTAAACGAGAGACCCTAGTTGATCATCACGGCGTAAAGAGTGGTTAGGGGAT

TATCATAAATAAAGCCAAAGACCTCCCAAGCTGTCGCACGCATTCCGGAGGCGCGAAGCC

CACACACGAAAGTAGCTTTAACTATTGACCCTGATGCCACGAAAGCTAAGAAA

>592_Sample_ASV9

CACCGCGGTTATACGAGAGGCCCAAGTTGATAAGCCCGGCGTAAAGAGTGGTTATGGAGA

ATAAAACACTAAAGCTAAAGACCCCCTAGGCTGTTATACGCACCTGGCGGCTCGAACCAC

CTATACGAAAGTAGCTTTACCCCCCTTCCACCAGAATCCACGACAGCTGGGGCA

>592_Sample_ASV10

CACCGCGGTTATACGAGAGGCCCAAGTTGACAGACATCGGCGTAAAGAGTGGTTAAGGAA

ACCCCATTACTAAAGCCGAACGCCCTCAGAACTGTCATACGTTCCCGAAGGTAAGAAGCC

CCACTACGAAAGTGGCTTTAACCACACCCGACCCCACGAAAGCTGTGAAA

>592_Sample_ASV11

CACCGCGGTTATACGAGAGGCCCAAGTTGATAGATATCGGCGTAAAGAGTGGTTAAGGAA

ACCCCGTTACTAAAGCCGAACGCCCTCAGAACTGTCATACGTTCCCGAAGGTAAGAAGCC

CCACTACGAAAGTGGCTTTAACCACACCCGACCCCACGAAAGCTGTGAAA

>592_Sample_ASV12

CACCGCGGTTAAACGAGAGGCCCTAGTTGATATTACAACGGCGTAAAGGGTGGTTAAGGA

AAGCAAGCAATAAAGCCGAATGGCCCTTTGGCTGTCATACGCTTCTAGGTGTCCGAAGCC

CAATATACGAAAGTAGCTTTAGAAAAGCCCACCTGACACCACGAAAGCTGAGGAA

>592_Sample_ASV13

CACCGCGGTTAAACGAGAGGCCCTAGTTGATATTACAACGGCGTAAAGGGTGGTTAAGGA

AAGCAAGCAATAAAGCCGAATGGCCCTTTGGCTGTCATACGCTTCTAGGTGTCCGAAGCC

CAATATACGAAAGTAGCTTTAGAAAAGCCCACCTGACACCACGAAAGCTGTGACA

>592_Sample_ASV14

CACCGCGGTTATACGAGAAACCCTAGTTGACAAATACGGCGTAAAGAGTGGTTAAGGGGT

ATCAAAAATAAAGTCAAACATTTTCCTAGTTGTTATAAGCATTTCGAGAACATGAAGCCC

AACCACGAAAGTAACTTTACCTTCACCTGACCCCACGAAAGCTAAGAAA

>592_Sample_ASV15

CACCGCGGTTATACGAGAGACCCTAGTTGATAAATACGGCGTAAAGAGTGGTTATGGGAA

AAACAAATAAAGTCAAACAACCTCTTAGCTGTTATACGCATTATGAGAGCATGAAGCCCC

CTCACGAAAGTAACTTTAATACCTCCTGACCCCACGAAAGCTAAGAAA

>592_Sample_ASV16

CACCGCGGTTATACGAGAGGCTCAAGTTGATAAACCCCGGCGTAAAGAGTGGTTAAGGAA

AAATAGAAACTAAAGCCGAATGCTTTCAAGACTGTTATACGTTCCCGAGAGTAAGAAGAC

CAATTACGAAAGTGGCTTTACTTTACCTGACCCCACGAAAGCTACGACA

>592_Sample_ASV17

AACCGCGGTTATACGAGAGACCCTAATTGATAGCTCACGGCGTAAAGGGTGGTTTGGGGA

AACCTCAAAATAAAGCTAAAGACCTTCTAAGCCGTTACACGCATACCGAAGGCACGAGGC

CCTAACACGAAAGTAGCTTTACTATTACCCCTGACGCCACGAAAGCTAAGAAA

>592_Sample_ASV18

CACCGCGGTTATACGAAAGACCCTAGTTGATGGATCACGGCGTAAAGGGTGGTTAAGGGA

TATAAAAATAAAGCTAAAAATCCTCTTAGCTGTCATACGCTTTCCGAAGACATGAGGCCC

AATTACGAAAGTAGCTTTAAACCACCCTGACACCACGAAAGCCAAGAAA

>592_Sample_ASV19

CACCGCGGTTATACGAGAGACCCTAGTTGATAGCTACGGCGTAAAGCGTGGTTAGGAGAA

CAAGATAAATAAAGTCAAACAATCTCTCGGCCGTTATACGTTATCTGAGAATATGAAGTC

CTACCACGAAAGTAACTTTAATTTTTCTGACCCCACGAAAACTGAGGAA

>592_Sample_ASV20

CACCGCGGTTATACGAGAGGCCCAAGTTGATACTCCACGGCGTAAAGAGTGGTTAGAAAA

GACCTACTACTAAAGCCGAACGCCTTCAAAGCTGTTATACGCATCCGAAGGTGAGAAGCC

CACCCACGAAAGTGGCTTTACAACCTTGACTCCACGAAAGCTATGACA

>592_Sample_ASV21

CACCGCGGTTATACGAGAGACCCCAGTTGATAAACACGGCGTAAAGAGTGGTTAAGGGCA

CAAAACAAATAAAGTCAAACAACCTCTTAGCTGTTATACGCATTATGAGGATATGAAGCC

CCCCTACGAAAGTAACTTTACCAACCCCTGACCCCACGAAAGCTAAGAAA

>592_Sample_ASV22

CACCGCGGTTATACGAAAGACCCTAGTTTATAGGCACGGCGTAAAGGGTGGTTAGGGGAT

AAACAAAATAAAGCCAAAGACCTTCTAAGTCGTCATACACTCATGAAGGCACGAAGCCCA

AATACGAAAGTAACTTTACAACTTAGACCCGACTCCACGAAAGCTGGGGGA

>592_Sample_ASV23

CACCGCGGTTATACGAGAGACCCTAATTAATAGATACGGCGTAAAGAGTGGTTTAGGACA

AAAATTAATAAAGCCGAAGTTTCTCCTAGCCGTTGTACGCACTCTGAGAACACGAAGCCC

CACTACGAAAGTAGCTTTACCTATTATTTCCTGACCCCACGAAAGCTGTGACA

>592_Sample_ASV24

CACCGCGGTTATACGAGAGACCCTAATTAATAGATACGGCGTAAAGAGTGGTTTAGGACA

AAAATTAATAAAGCCGAAGTTTCTCCTAGCCGTTGTACGCACTCTGAGAACACGAAGCCC

CACTACGAAAGTAGCTTTACCTATTATTTCCTGACCCCACGAAAGCTAAGAAA

>592_Sample_ASV25

CACCGCGGTTATACGAGAGGCCCAAGTTGATAGACATCGGCGTAAAGAGTGGTTAAGGAA

GTCTCAAAACTAAAGCCGAATGCCTTCAAGACTGTTATACGTTTCCGAAGGTAAGAAGCC

CCACTACGAAAGTGGCTTTATTCCACCTGACCCCACGAAAGCTGTGAAA

>592_Sample_ASV26

CACCGCGGTTATACGAGAGGCCCAAGTTGATAGACATCGGCGTAAAGAGTGGTTAAGGAA

GTCTCAAAACTAAAGCCGAATGCCTTCAAGACTGTTATACGTTTCCGAAGGTAAGAAGCC

CCACTACGAAAGTGGCTTTACTCCACCTGACTCCACGAAAGCTGTGAAA

>592_Sample_ASV27

CACCGCGGTTATACGAGAGGCCCAAGTTGACAAACTCCGGCGTAAAGCGTGGTTAAGGCA

CACTCAAAAACTAAAGCCGAATATCTTCAGAGCAGTCATACGCATCCGAAGACACGAAGC

CCCACCACGAAAGTGGCTTTATGACACCTGACCCCACGAAAGCTAGGACA

>614_Sample_ASV1

CACCGCGGTTATACGAGAGGCCCAAGTTGACAGACATCGGCGTAAAGAGTGGTTAAGGAA

AACCTTCAACTAAAGTCGAACGCTCTCAAGACTGTCGTACGTTTCCGAGAGTATGAAGCC

CCACCACGAAAGTGACTTTACCTCACCTGACTCCACGAAAGCTGCGAAA

>614_Sample_ASV2

CACCACGGTTATACGAGAGGCCCAAGTTGAAAGACATCGGCGTAAAGAGTGGTTAAGGAA

AACCTTCTACTAAAGTCGAACGCTCTCAGGACTGTCGTACGTTTCCGAGAGTATGAAGCC

CCACCACGAAAGTGACTTTACCTCACCTGACTCCACGAAAGCTGCGAAA

>614_Sample_ASV3

TACCGCGGTTATACGAAAGACCCAAGTTAATAAACTACGGCGTAAAGCGTGGTTAGAGAC

CTCCAATAATTAAAGCCAAAGACCTTCTAAGCCGTCGCACGCCCCGCGAAGACACGAAGC

CCAAAAACGAAAGTAGCTTTATTCTTACTCGACCCCACGAAAGCTAGGGCA

>614_Sample_ASV4

CACCGCGGTTATACGAAAGACCCTAGTTGATGGGTGCGGCGTAAAGGGTGGTTAGGGACT

ACAAAAAATAGAGCTAAAGATCCTCTAGGTCGTCATACACATCACGAGGAAACGAAGCCC

AAATACGAAAGTAGCTCTAAATATAATCCTGACGCCACGAAAGCCAAGAAA

>614_Sample_ASV5

CACCGCGGTTAAACGAGAGGCCCTAGTTGATGGTGCAACGGCGTAAAGGGTGGTTAAGGA

TAGCGAAATAATAAAGTCAAATGGCCCTCTGGCTGTCATACGCTTCTAGGAGTCCGAAGC

CCAGTATACGAAAGTAGCTTTAAGAAAGTCCACCTGACCCCACGAAAACTGAGGAA

>614_Sample_ASV6

CACCGCGGTTATACGAGAGACCCTAGTTGATTAAAGCGGCGTAAAGAGTGGTTATGGAAC

TATTCTTTTAAAGCAGAAAACCTCTCAAACTGTTATACGCACCCAGAGGTCTGAATCCCT

CACACGAAAGTGACTTTATTTATGCCTACCAGAACCCACGAAAGCTGGGACA

>614_Sample_ASV7

CACCGCGGTTATACGAAAGACCCTAGTTTATAGGTACGGCGTAAAGGGTGGTTAGGGGAC

AAACAAAATAAAGCCAAAGACCTCCTAAGCCGTCATACGCTCACGGAAGCACGAAGCCCA

AACACGAAAGTAGCTTTACCGAACATGCCCGACTCCACGAAAGCTGGGGAA

>614_Sample_ASV8

CACCGCGGTTATACGAAAGACCCTAGTTGATAGCCACGGCGTAAAGGGTGGTTAAGGAAC

CTAAAAAATAAAGCTAAAGACCCTCTAGGCCGTCATACGCATTACGAGGCAAACGAAACC

CCAACACGAAAGTAGCTTTAGACAAATATCCTGATGCCACGAAAGCCAAGAAA

>614_Sample_ASV9

CACCGCGGTTATACGAGAGGCCCAAGTTGACAGACACCGGCGTAAAGAGTGGCTAGGGAA

AAATTACTACTAAAGCCGAACACCTTCAGAACTGTCATACGTTCCCGAAGATAAGAAGCC

CCACCACGAAAGTGGCTTTATATTACCCGACCCCACGAAAGCTAAGAAA

>614_Sample_ASV10

CACCGCGGTTATACGAGAGGCCCAAGTTGACAGACACCGGCGTAAAGAGTGGCTAGGGAA

AAATTACTACTAAAGCCGAACACCTTCAGAACTGTCATACGTTCCCGAAGATAAGAAGCC

CCACCACGAAAGTGGCTTTATATTACCCGAATCCACGAAAGCTAAGAAA

>614_Sample_ASV11

CACCGCGGTTATACGAAAGACTCTAGTTGACAGACACCGGCGTAAAGAGTGGCTAGGGAA

AAATTACTACTAAAGCCGAACACCTTCAGAACTGTCATACGTTCCCGAAGATAAGAAGCC

CCACCACGAAAGTGGCTTTATATTACCCGACCCCACGAAAGCTAAGAAA

>614_Sample_ASV12

CACCGCGGTTATACGAGAGGCCCAAGTTGACAGACACCGGCGTAAAGAGTGGCTAGGGAA

AAATTACTACTAAAGCCGAACACCTTCAGAACTGTCATACGTTCCCGAAGATAAGAAGCC

CCACCACGAAAGTGGCTTTATATTACCCGACCCCACGAAAGCTGTGAAA

>614_Sample_ASV13

CACCGCGGTTATACGAAAGACTCTAGTTGACAAACACGGCGTAAAGGGTGGTTAGGATAA

CAACAAAATAAAGCTAAAGACTTACCAAGCCGTCATACGCCCATGTAAGAGCGAAAACCA

CAAACGAAAGTAACTTTATTACACCATCCGAATCCACGAAAGCTAAGAAA

>614_Sample_ASV14

CACCGCGGTTATACGAAAGACCCTAGTTGATAGATCACGGCGTAAAGGGTGGTTAGGGAA

TACAAAAATAAAGCTAAAAATCCTCTAAGCTGTCATACGCTTCCCGAAGACATGAAACCC

AACCACGAAAGTAGCTTTAAACTCTCCTGACACCACGAAAGCCAAGGAA

>614_Sample_ASV15

CACCGCGGTTATACGAAAGACCCTAGTTTATAGGCACGGCGTAAAGGGTGGTTAGGGAAT

ACAAAAATAAAGCTAAAAATCCTCTAAGCTGTCATACGCTTCCCGAAGACATGAAACCCA

ACCACGAAAGTAGCTTTAAACTCTCCTGACACCACGAAAGCCAAGGAA

>614_Sample_ASV16

CACCGCGGTTATACGAGAGGCCCAAGTTGATAAGCCCGGCGTAAAGAGTGGTTATGGAGA

ATAAAACACTAAAGCTAAAGACCCCCTAGGCTGTTATACGCACCTGGCGGCTCGAACCAC

CTATACGAAAGTAGCTTTACCCCCCTTCCACCAGAATCCACGACAGCTGGGGCA

>614_Sample_ASV17

CACCGCGGTTATACGAGAGGCCCTAGTTGATAGCCACGGCGCAAAGAGTGGTTAAGGAGC

CCCACTAAATAAAGTCGAACACTTCCTAGGCCGTTATACGTTTTCTAGAAGCACGAAACC

CAATTACACGAAAGCAACTTTATACTAAACAACCTGACCCCACGAAAGCTAAGAAA

>614_Sample_ASV18

CACCGCGGTTATACGAGAGGCCCAAGTTGACAGACATCGGCGTAAAGAGTGGTTAAGGAA

ACCCCATTACTAAAGCCGAACGCCCTCAGAACTGTCATACGTTCCCGAAGGTAAGAAGCC

CCACTACGAAAGTGGCTTTAACCACACCCGACCCCACGAAAGCTGTGAAA

>614_Sample_ASV19

CACCGCGGTTATACGAGAGGCCCAAGTTGATAGATATCGGCGTAAAGAGTGGTTAAGGAA

ACCCCGTTACTAAAGCCGAACGCCCTCAGAACTGTCATACGTTCCCGAAGGTAAGAAGCC

CCACTACGAAAGTGGCTTTAACCACACCCGACCCCACGAAAGCTGTGAAA

>614_Sample_ASV20

CACCGCGGTTATACGAGAGGCCCCAGTTGATATTAGCGGCGTAAAGGGTGGTTAAGGACT

TTAATAACTAAAGCTAAACACTTCCCCGGCTGTTGTACGTAGACCGGAAGCTTGAAGCCC

AGACACGAAAGTAGCTTTATTTCCCCTGACCCCACGAAAGCTGAGAAA

>614_Sample_ASV21

CACCGCGGTTATACGAGAGGCCCCAGTTGATATTAGCGGCGTAAAGGGTGGTTAAGGACT

TTAATAACTAAAGCTAAACACTTCCCCGGCTGTTGTACGTAGACCGGAAGCTTGAAGCCC

AGACACGAAAGTAGCTTTATTCCCCCTGACCCCACGAAAGCTGAGAAA

>614_Sample_ASV22

CACCGCGGTTATACGAGAGGCCCCAGTTGATATTAGCGGCGTAAAGGGTGGTTAAGGACT

TTAATAACTAAAGCCAAACACTTCCCCGGCTGTTGTACGTAGACCGGAAGCTTGAAGCCC

AGACACGAAAGTAGCTTTATTTCCCCTGACCCCACGAAAGCTGAGAAA

>614_Sample_ASV23

CACCGCGGTCACACGATTAACCCAAGTCAATAGAAGCCGGCGTAAAGAGTGTTTTAGATC

ACCCCCTCCCCAATAAAGCTAAAACTCACCTGAGTTGTAAAAAACTCCAGTTGACACAAA

ATAGACTACGAAAGTGGCTTTAACATATCTGAACACACAATAGCTAAGACC

>614_Sample_ASV24

CACCGCGGTTATACGAAAGACCCCAGTTTATAGACACGGCGTAAAGGGTGGTTATAGGAC

AAACTAACATAGAGCCAAAGACCCCCTAAGCCGTCATACGCTCACGAGGGCTCGAAGCCC

CTATACGAAAGTAGCTCTACCAAAAAATGCTCGACTCCACGAAAGCTGGGACA

>614_Sample_ASV25

CACCGCGGTTATACGAGAGACCCTAGTTGATAAATACGGCGTAAAGAGTGGTTATGGGAA

AAACAAATAAAGTCAAACAACCTCTTAGCTGTTATACGCATTATGAGAGCATGAAGCCCC

CTCACGAAAGTAACTTTAATACCTCCTGACCCCACGAAAGCTAAGAAA

>614_Sample_ASV26

CACCGCGGTTATACGAGAGACCCTAATTGATAGGCACGGCGTAAAGAGTGGTTTAGGGTA

ACCCATAAATAAAGCTAAAGACCTTCTAAGCCGTCACACGCACACCGAAGGCACGAGACC

CCAACACGAAAGTAGCTTTACTTCTACCCCTGACGCCACGAAAGCTAAGAAA

>614_Sample_ASV27

CACCGCGGTTATACGAAAGACCCTAGTTGATGGATCACGGCGTAAAGGGTGGTTAAGGGA

TATAAAAATAAAGCTAAAAATCCTCTTAGCTGTCATACGCTTTCCGAAGACATGAGGCCC

AATTACGAAAGTAGCTTTAAACCACCCTGACACCACGAAAGCCAAGAAA

>614_Sample_ASV28

CACCGCGGTTATACGAAAGACCCCAGTTTATAGATGCGGCGTAAAGGGTGGTTAAGAGAA

AATCAAAATAAGGCCAAAGATCCCCTAAGCAGTCATACGCTCCACGAGGACACGAAACCC

AAATACGAAAGTTGCCTTAATAAACTTCACCTGATCCCACGAAAGCTGAAAAA

>614_Sample_ASV29

CACCGCGGTTATACGAAAGGCTCTAGTTTATAAATACGGCGTAAAGGGTGGTTAAGGAAA

AATTAAAATAATGCTAAAGACCCCCTAAGCAGTTATACGCTTCACGGAGGCACGAAACCC

AAATACGAAAGTTGCCTTAATAAACTTTACCTGACCCCACGAAAGCTGAAAAA

>614_Sample_ASV30

CACCGCGGTTATACGAGAGACTCTAGTTGATACCAACGGCGTAAAGAGTGGTTTGGGATG

CCCCTTAAAATAAAGCCAAAGACCTCCCAAGCCGTCAAACGCACCCCGGAGGCACGAAGC

CCCAACGCGAAAGCAACTTTACCTCCCTCCCGACGCCACGAAAGCTAAGAAA

>614_Sample_ASV31

CACCGCGGTTATACGAGAGACCCTAGTTGATAGCTACGGCGTAAAGCGTGGTTAGGAGAA

CAAGATAAATAAAGTCAAACAATCTCTCGGCCGTTATACGTTATCTGAGAATATGAAGTC

CTACCACGAAAGTAACTTTAATTTTTCTGACCCCACGAAAACTGAGGAA

>614_Sample_ASV32

CACCGCGGTTATACGAGAGGCCCTAGTTGATAACTACCGGCGTAAAGAGTGGTTATGGAA

AAATATTTAATAAAGCCGAACACCCCCTCAGCCGTCATACGCACCTGGGAGCACGAAGAC

CTACTGCGAAAGCAGCTTTAATTATGCCTGACCCCACGACAGCTAAGAAA

>614_Sample_ASV33

CACCGCGGTTATACGAGAGGCCCAAGTTGATTATTACCGGCGTAAAGCGTGGTTAAGATT

TTTATAGGACTAAAGCCGAACCCCCTCAGAGCTGTTATACGCACCCGAAGGTTAGAAGTT

CAATCACGAAAGTGGCTTTACAACCCCGAACCCACGAAAGCTAAGATA

>614_Sample_ASV34

CACCGCGGTTATACGAGAGGCCCAAGTTGATACTCCACGGCGTAAAGAGTGGTTAGAAAA

GACCTACTACTAAAGCCGAACGCCTTCAAAGCTGTTATACGCATCCGAAGGTGAGAAGCC

CACCCACGAAAGTGGCTTTACAACCTTGACTCCACGAAAGCTATGACA

>614_Sample_ASV35

CACCGCGGTTATACGAGAGACCCCAGTTGATAAACACGGCGTAAAGAGTGGTTAAGGGCA

CAAAACAAATAAAGTCAAACAACCTCTTAGCTGTTATACGCATTATGAGGATATGAAGCC

CCCCTACGAAAGTAACTTTACCAACCCCTGACCCCACGAAAGCTAAGAAA

>614_Sample_ASV36

CACCGCGGTTATACGAAAGACCCTAGTTTATAGGCACGGCGTAAAGGGTGGTTAGGGGAT

AAACAAAATAAAGCCAAAGACCTTCTAAGTCGTCATACACTCATGAAGGCACGAAGCCCA

AATACGAAAGTAACTTTACAACTTAGACCCGACTCCACGAAAGCTGGGGGA

>614_Sample_ASV37

CACCGCGGTTATACGAGAGACTCCAGTTGATAGCTACGGCGTAAAGAGTGGTTTGGGGCC

CCACCCAAAATAAAGCCAAAGACCTCCCAAGCCGTCAAACGCACCCCGGAGGCACGAAGT

CCTAACGCGAAAGCAACTTTACCTCCCCCGACGCCACGAAAGCTAAGAAA

>614_Sample_ASV38

CACCGCGGTTATACGAGAGACCCTAATTAATAGATACGGCGTAAAGAGTGGTTTAGGACA

AAAATTAATAAAGCCGAAGTTTCTCCTAGCCGTTGTACGCACTCTGAGAACACGAAGCCC

CACTACGAAAGTAGCTTTACCTATTATTTCCTGACCCCACGAAAGCTAAGAAA

>614_Sample_ASV39

CACCGCGGTTATACGAAAGACCCTAGTTGATAGCTACGGCGTAAAGGGTGGTTAAGGTAT

ATAACAAATAAAGCTAAAGAGCCTCCAAGCCGTCGTACGCATTCTGAGAGCTCGAAACCC

TAACACGAAAGTAGCTTTAAAAAATAACACCTGACCCCACGAAAGCTAAGAAA

>614_Sample_ASV40

CACCGCGGTTATACGAAAGACCCTAGTTGATAGATCACGGCGTAAAGGGTGGTTAAGGAG

AACAAAAATAAAGCTAAAAATCTTCTAAGCTGTCATACGCTTTCCGAAGACATGAGACCC

AACCACGAAAGTAGCTTTAAACTTTCCTGACACCACGAAAGCCAAGAAA

>620_Sample_ASV1

TACCGCGGTTATACGAAAGACCCAAGTTAATAAACTACGGCGTAAAGCGTGGTTAGAGAC

CTCCAATAATTAAAGCCAAAGACCTTCTAAGCCGTCGCACGCCCCGCGAAGACACGAAGC

CCAAAAACGAAAGTAGCTTTATTCTTACTCGACCCCACGAAAGCTAGGGCA

>620_Sample_ASV2

CACCGCGGTTATACGAAAGACCCTAGTTGATGGGTGCGGCGTAAAGGGTGGTTAGGGACT

ACAAAAAATAGAGCTAAAGATCCTCTAGGTCGTCATACACATCACGAGGAAACGAAGCCC

AAATACGAAAGTAGCTCTAAATATAATCCTGACGCCACGAAAGCCAAGAAA

>620_Sample_ASV3

CACCGCGGTTATACGAGAGACCCTAGTTGATTAAAGCGGCGTAAAGAGTGGTTATGGAAC

TATTCTTTTAAAGCAGAAAACCTCTCAAACTGTTATACGCACCCAGAGGTCTGAATCCCT

CACACGAAAGTGACTTTATTTATGCCTACCAGAACCCACGAAAGCTGGGACA

>620_Sample_ASV4

CACCGCGGTTATACGAAAGACCCTAGTTTATAGGTACGGCGTAAAGGGTGGTTAGGGGAC

AAACAAAATAAAGCCAAAGACCTCCTAAGCCGTCATACGCTCACGGAAGCACGAAGCCCA

AACACGAAAGTAGCTTTACCGAACATGCCCGACTCCACGAAAGCTGGGGAA

>620_Sample_ASV5

CACCGCGGTTATACGAGAGGCCCAAGTTGACAGACACCGGCGTAAAGAGTGGCTAGGGAA

AAATTACTACTAAAGCCGAACACCTTCAGAACTGTCATACGTTCCCGAAGATAAGAAGCC

CCACCACGAAAGTGGCTTTATATTACCCGACCCCACGAAAGCTGTGAAA

>620_Sample_ASV6

CACCGCGGTTATACGAGAGGCCCAAGTTGACAGACACCGGCGTAAAGAGTGGCTAAGGAA

AAATTTCTACTAAAGCCGAACACCTTCAGAACTGTCATACGTTCCCGAAGAAAAGAAGCC

CCACCACGAAAGTGGCTTTATATTACCCGACCCCACGAAAGCTGTGAAA

>620_Sample_ASV7

CACCGCGGTTATACGAAAGACTCTAGTTGACAAACACGGCGTAAAGGGTGGTTAGGATAA

CAACAAAATAAAGCTAAAGACTTACCAAGCCGTCATACGCCCATGTAAGAGCGAAAACCA

CAAACGAAAGTAACTTTATTACACCATCCGAATCCACGAAAGCTAAGAAA

>620_Sample_ASV8

CACCGCGGTTATACGAAAGACCCTAGTTGATAGATCACGGCGTAAAGGGTGGTTAGGGAA

TACAAAAATAAAGCTAAAAATCCTCTAAGCTGTCATACGCTTCCCGAAGACATGAAACCC

AACCACGAAAGTAGCTTTAAACTCTCCTGACACCACGAAAGCCAAGGAA

>620_Sample_ASV9

CACCGCGGTTATACGAGAGGCCCAAGTTGATAAGCCCGGCGTAAAGAGTGGTTATGGAGA

ATAAAACACTAAAGCTAAAGACCCCCTAGGCTGTTATACGCACCTGGCGGCTCGAACCAC

CTATACGAAAGTAGCTTTACCCCCCTTCCACCAGAATCCACGACAGCTGGGGCA

>620_Sample_ASV10

CACCGCGGTTATACGAGAGGCCCTAGTTGATAGCCACGGCGCAAAGAGTGGTTAAGGAGC

CCCACTAAATAAAGTCGAACACTTCCTAGGCCGTTATACGTTTTCTAGAAGCACGAAACC

CAATTACACGAAAGCAACTTTATACTAAACAACCTGACCCCACGAAAGCTAAGAAA

>620_Sample_ASV11

CACCGCGGTTATACGAGAGGCCCAAGTTGACAGACATCGGCGTAAAGAGTGGTTAAGGAA

ACCCCATTACTAAAGCCGAACGCCCTCAGAACTGTCATACGTTCCCGAAGGTAAGAAGCC

CCACTACGAAAGTGGCTTTAACCACACCCGACCCCACGAAAGCTAAGAAA

>620_Sample_ASV12

CACCGCGGTTATACGAGAGGCCCAAGTTGACAGACATCGGCGTAAAGAGTGGTTAAGGAA

ACCCCATTACTAAAGCCGAACGCCCTCAGAACTGTCATACGTTCCCGAAGGTAAGAAGCC

CCACTACGAAAGTGGCTTTAACCACACCCGACCCCACGAAAGCTGTGAAA

>620_Sample_ASV13

CGCCGCGGTTATACGAGAGGCCCAAGTTGACAGACATCGGCGTAAAGAGTGGTTAAGGAA

ACCCCATTACTAAAGCCGAACGCCCTCAGAACTGTCATACGTTTCCGAAGGTAAGAAGCC

CCACTACGAAAGTGGCTTTACCCACACCCGACCCCACGAAAGCTAAGAAA

>620_Sample_ASV14

CACCGCGGTTATACGAGAGGCCCAAGTTGATAGATATCGGCGTAAAGAGTGGTTAAGGAA

ACCCCGTTACTAAAGCCGAACGCCCTCAGAACTGTCATACGTTCCCGAAGGTAAGAAGCC

CCACTACGAAAGTGGCTTTAACCACACCCGACCCCACGAAAGCTAAGAAA

>620_Sample_ASV15

CACCGCGGTTATACGAGAGGCCCAAGTTGACAGACATCGGCGTAAAGAGTGGTTAAGGAA

ACCCCATTACCAAAGCCGAACGCCCTCAGAACTGTCATACGTTCCCGAAGGTAAGAAGCC

CCACTACGAAAGTGGCTTTACTCCACCTGACTCCACGAAAGCTGTGAAA

>620_Sample_ASV16

CACCGCGGTTATACGAGAGGCCCAAGTTGATAGATATCGGCGTAAAGAGTGGTTAAGGAA

ACCCCGTTACTAAAGCCGAACGCCCTCAGAACTGTCATACGTTCCCGAAGGTAAGAAGCC

CCACTACGAAAGTGGCTTTAACCACACCCGACCCCACGAAAGCTGTGAAA

>620_Sample_ASV17

CACCGCGGTTATACGAGAGGCCCCAGTTGATATTAGCGGCGTAAAGGGTGGTTAAGGACT

TTAATAACTAAAGCTAAACACTTCCCCGGCTGTTGTACGTAGACCGGAAGCTTGAAGCCC

AGACACGAAAGTAGCTTTATTTCCCCTGACCCCACGAAAGCTGAGAAA

>620_Sample_ASV18

CACCGCGGTTATACGAGAGGCCCCAGTTGATATTAGCGGCGTAAAGGGTGGTTAAGGACT

TTAATAACTAAAGCCAAACACTTCCCCGGCTGTTGTACGTAGACCGGAAGCTTGAAGCCC

AGACACGAAAGTAGCTTTATTTCCCCTGACCCCACGAAAGCTGAGAAA

>620_Sample_ASV19

CACCGCGGTTATACGAGAGGCCCCAGTTGATATTAGCGGCGTAAAGGGTGGTTAAGGACT

TTAATAACTAAAGCTAAACACTTCCCCGGCTGTTGTACGTAGACCGGAAGCTTGAAGCCC

AGACACGAAAGTAGCTTTATTCCCCCTGACCCCACGAAAGCTGAGAAA

>620_Sample_ASV20

CACCGCGGTTATACGAAAGACCCCAGTTTATAGACACGGCGTAAAGGGTGGTTATAGGAC

AAACTAACATAGAGCCAAAGACCCCCTAAGCCGTCATACGCTCACGAGGGCTCGAAGCCC

CTATACGAAAGTAGCTCTACCAAAAAATGCTCGACTCCACGAAAGCTGGGACA

>620_Sample_ASV21

CACCGCGGTTATACGAGAGACCCTAATTGATAGGCACGGCGTAAAGAGTGGTTTAGGGTA

ACCCATAAATAAAGCTAAAGACCTTCTAAGCCGTCACACGCACACCGAAGGCACGAGACC

CCAACACGAAAGTAGCTTTACTTCTACCCCTGACGCCACGAAAGCTAAGAAA

>620_Sample_ASV22

CACCGCGGTTATACGAAAGACCCTAGTTGATGGATCACGGCGTAAAGGGTGGTTAAGGGA

TATAAAAATAAAGCTAAAAATCCTCTTAGCTGTCATACGCTTTCCGAAGACATGAGGCCC

AATTACGAAAGTAGCTTTAAACCACCCTGACACCACGAAAGCCAAGAAA

>620_Sample_ASV23

CACCGCGGTTATACGAAAGACCCCAGTTTATAGATGCGGCGTAAAGGGTGGTTAAGAGAA

AATCAAAATAAGGCCAAAGATCCCCTAAGCAGTCATACGCTCCACGAGGACACGAAACCC

AAATACGAAAGTTGCCTTAATAAACTTCACCTGATCCCACGAAAGCTGAAAAA

>620_Sample_ASV24

CACCGCGGTTATACGAAAGGCTCTAGTTTATAAATACGGCGTAAAGGGTGGTTAAGGAAA

AATTAAAATAATGCTAAAGACCCCCTAAGCAGTTATACGCTTCACGGAGGCACGAAACCC

AAATACGAAAGTTGCCTTAATAAACTTTACCTGACCCCACGAAAGCTGAAAAA

>620_Sample_ASV25

CACCGCGGTTATACGAGAGACTCTAGTTGATACCAACGGCGTAAAGAGTGGTTTGGGATG

CCCCTTAAAATAAAGCCAAAGACCTCCCAAGCCGTCAAACGCACCCCGGAGGCACGAAGC

CCCAACGCGAAAGCAACTTTACCTCCCTCCCGACGCCACGAAAGCTAAGAAA

>620_Sample_ASV26

CACCGCGGTTATACGAGAGACCCTAGTTGATAGCTACGGCGTAAAGCGTGGTTAGGAGAA

CAAGATAAATAAAGTCAAACAATCTCTCGGCCGTTATACGTTATCTGAGAATATGAAGTC

CTACCACGAAAGTAACTTTAATTTTTCTGACCCCACGAAAACTGAGGAA

>620_Sample_ASV27

CACCGCGGTTATACGAGAGGCCCTAGTTGATAACTACCGGCGTAAAGAGTGGTTATGGAA

AAATATTTAATAAAGCCGAACACCCCCTCAGCCGTCATACGCACCTGGGAGCACGAAGAC

CTACTGCGAAAGCAGCTTTAATTATGCCTGACCCCACGACAGCTAAGAAA

>620_Sample_ASV28

CACCGCGGTTATACGAGAGGCCCAAGTTGATTATTACCGGCGTAAAGCGTGGTTAAGATT

TTTATAGGACTAAAGCCGAACCCCCTCAGAGCTGTTATACGCACCCGAAGGTTAGAAGTT

CAATCACGAAAGTGGCTTTACAACCCCGAACCCACGAAAGCTAAGATA

>620_Sample_ASV29

CACCGCGGTTATACGAGAGGCCCAAGTTGATACTCCACGGCGTAAAGAGTGGTTAGAAAA

GACCTACTACTAAAGCCGAACGCCTTCAAAGCTGTTATACGCATCCGAAGGTGAGAAGCC

CACCCACGAAAGTGGCTTTACAACCTTGACTCCACGAAAGCTATGACA

>620_Sample_ASV30

CACCGCGGTTATACGAGAGACCCCAGTTGATAAACACGGCGTAAAGAGTGGTTAAGGGCA

CAAAACAAATAAGGTCAAACAACCTCTTAGCTGTTATACGCATTATGAGGATATGAAGCC

CCCCTACGAAAGTAACTTTACCAACCCCTGACCCCACGAAAGCTGAAAAA

>620_Sample_ASV31

CACCGCGGTTATACGAGAGACCCCAGTTGATAAACACGGCGTAAAGAGTGGTTAAGGGCA

CAAAACAAATAAAGTCAAACAACCTCTTAGCTGTTATACGCATTATGAGGATATGAAGCC

CCCCTACGAAAGTAACTTTACCAACCCCTGACCCCACGAAAGCTAAGAAA

>620_Sample_ASV32

CACCGCGGTTATACGAAAGACCCTAGTTTATAGGCACGGCGTAAAGGGTGGTTAGGGGAT

AAACAAAATAAAGCCAAAGACCTTCTAAGTCGTCATACACTCATGAAGGCACGAAGCCCA

AATACGAAAGTAACTTTACAACTTAGACCCGACTCCACGAAAGCTGGGGGA

>620_Sample_ASV33

CACCGCGGTTATACGAGAGACCCTAATTAATAGATACGGCGTAAAGAGTGGTTTAGGACA

AAAATTAATAAAGCCGAAGTTTCTCCTAGCCGTTGTACGCACTCTGAGAACACGAAGCCC

CACTACGAAAGTAGCTTTACCTATTATTTCCTGACCCCACGAAAGCTAAGAAA

>620_Sample_ASV34

CACCGCGGTTATACGAGAGGCCCAAGTTGATAGACATCGGCGTAAAGAGTGGTTAAGGAA

GTCTCAAAACTAAAGCCGAATGCCTTCAAGACTGTTATACGTTTCCGAAGGTAAGAAGCC

CCACTACGAAAGTGGCTTTACTCCACCTGACTCCACGAAAGCTGTGAAA

>620_Sample_ASV35

CACCGCGGTTATACGAAAGACCCTAGTTGATAGCTACGGCGTAAAGGGTGGTTAAGGTAT

ATAACAAATAAAGCTAAAGAGCCTCCAAGCCGTCGTACGCATTCTGAGAGCTCGAAACCC

TAACACGAAAGTAGCTTTAAAAAATAACACCTGACCCCACGAAAGCTAAGAAA

>620_Sample_ASV36

CACCGCGGTTATACGAAAGACCCTAGTTGATAGATCACGGCGTAAAGGGTGGTTAAGGAG

AACAAAAATAAAGCTAAAAATCTTCTAAGCTGTCATACGCTTTCCGAAGACATGAGACCC

AACCACGAAAGTAGCTTTAAACTTTCCTGACACCACGAAAGCCAAGAAA

>628_Sample_ASV1

CACCGCGGTTATACGAAAGACCCTAGTTGATGGGTGCGGCGTAAAGGGTGGTTAGGGACT

ACAAAAAATAGAGCTAAAGATCCTCTAGGTCGTCATACACATCACGAGGAAACGAAGCCC

AAATACGAAAGTAGCTCTAAATATAATCCTGACGCCACGAAAGCCAAGAAA

>628_Sample_ASV2

CACCGCGGTTATACGAGAGACCCTAGTTGATTAAAGCGGCGTAAAGAGTGGTTATGGAAC

TATTCTTTTAAAGCAGAAAACCTCTCAAACTGTTATACGCACCCAGAGGTCTGAATCCCT

CACACGAAAGTGACTTTATTTATGCCTACCAGAACCCACGAAAGCTGGGACA

>628_Sample_ASV3

CACCGCGGTTATACGAGAGACCCTAGTTGATTAAAGCGGCGTAAAGAGTGGTTATGGAAC

TATTCTTTTAAAGCAGAAAACCTCTCAAACTGTTATACGCACCCAGAGGTCTAAATCCCT

CACACGAAAGTGACTTTATTTATGCCTACCAGAACCCACGAAAGCTGGGACA

>628_Sample_ASV4

CACCGCGGTTATACGAGAGACCCTAGTTGATTAAAGCGGCGTAAAGAGTGGTTATGGAAC

TATTCTTTTAAAGCAGAAAACCTCTCAAACTGTTATACGCACCCAGAGGTCTGAATCCCT

CACACGAAAGTGACTTTATCTATGCCTACCAGAACCCACGAAAGCTGGGACA

>628_Sample_ASV5

CACCGCGGTTATACGAGAGACCCTAGTTGATATACACGGCGTAAAGAGTGGTTTGGGGTA

TATAATAAATAAAGCCAAAGACCTTCTAGGCCGTTGAACGCACCACGAAGGAGCGAAGTC

CCAATACGAAAGTAGCTTTATAAAACCTGACGCCACGAAAGCTAAGAAA

>628_Sample_ASV6

CACCGCGGTTATACGAGAGGCCCAAGTTGACAGACACCGGCGTAAAGAGTGGCTAGGGAA

AAATTACTACTAAAGCCGAACACCTTCAGAACTGTCATACGTTCCCGAAGATAAGAAGCC

CCACCACGAAAGTGACTTTATATTACCCGACCCCACGAAAGCTGTGAAA

>628_Sample_ASV7

CACCGCGGTTATACGAGAGGCCCAAGTTGACAGACACCGGCGTAAAGAGTGGCTAGGGAA

AAATTACTACTAAAGCCGAACACCTTCAGAACTGTCATACGTTCCCGAAGATAAGAAGCC

CCACCACGAAAGTGGCTTTATATTACCCGACCCCACGAAAGCTAAGAAA

>628_Sample_ASV8

CATGGCGATTATACGAGAGGCCCAAGTTGACAGACACTGGCGTAAAGAGTGGCTAAGGAA

AAACTACTACTAAAGCCGAACACCTTCAGAACTGTCATACGTTCCCGAAGATAAGAAGCC

CCACCACGAAAGTGGCTTTATATTACCCGACCCCACGAAAGCTGTGAAA

>628_Sample_ASV9

CACCGCGGTTATACGAGAGGCCCAAGTTGACAGACACCGGCGTAAAGAGTGGCTAGGGAA

AAATTACTACTAAAGCCGAACACCTTCAGAACTGTCATACGTTCCCGAAGATAAGAAGCC

CCACCACGAAAGTGGCTTTACAATGCCTGACCCCACGAAAGCTGTGAAA

>628_Sample_ASV10

CACCGCGGTTATACGAGAGACCCTAGTTGACAGACACCGGCGTAAAGAGTGGCTAGGGAA

AAATTACTACTAAAGCCGAACACCTTCAGAACTGTCATACGTTCCCGAAGATAAGAAGCC

CCACCACGAAAGTGGCTTTATATTACCCGACCCCACGAAAGCTAAGAAA

>628_Sample_ASV11

CACCGCGGTTATACGAGAGGCCCAAGTTGACAGACACCGGCGTAAAGAGTGGCTAGGGAA

AAATTACTACTAAAGCCGAACACCTTCAGAACTGTCATACGTTCCCGAAGATAAGAAGCC

CCACCACGAAAGTGGCTTTATATTACCCGACCCCACGAAAGCTGTGAAA

>628_Sample_ASV12

CACCGCGGTTATACGAGAGGCCCAAGTTGACAGACACCGGCGTAAAGAGTGGCTAAGGAA

AAATTTCTACTAAAGCCGAACACCTTCAGAACTGTCATACGTTCCCGAAGAAAAGAAGCC

CCACCACGAAAGTGGCTTTATATTACCCGACCCCACGAAAGCTGTGAAA

>628_Sample_ASV13

CACCGCGGTTATACGAGAGGCCCAAGTTGATAAGCCCGGCGTAAAGAGTGGTTATGGAGA

ATAAAACACTAAAGCTAAAGACCCCCTAGGCTGTTATACGCACCTGGCGGCTCGAACCAC

CTATACGAAAGTAGCTTTACCCCCCTTCCACCAGAATCCACGACAGCTGGGGCA

>628_Sample_ASV14

CACCGCGGTTATACGAGAGGCCCTAGTTGATAGCCACGGCGCAAAGAGTGGTTAAGGAGC

CCCACTAAATAAAGTCGAACACTTCCTAGGCCGTTATACGTTTTCTAGAAGCACGAAACC

CAATTACACGAAAGCAACTTTATACTAAACAACCTGACCCCACGAAAGCTAAGAAA

>628_Sample_ASV15

CACCGCGGTTATACGAGAGGCCCAAGTTGACAGACATCGGCGTAAAGAGTGGTTAAGGAA

ACCCCATTACTAAAGCCGAACGCCCTCAGAACTGTCATACGTTCCCGAAGGTAAGAAGCC

CCACTACGAAAGTGGCTTTAACCACACCCGACCCCACGAAAGCTGTGAAA

>628_Sample_ASV16

CACCGCGGTTATACGAGAGGCCCAAGTTGATAGATATCGGCGTAAAGAGTGGTTAAGGAA

ACCCCGTTACTAAAGCCGAACGCCCTCAGAACTGTCATACGTTCCCGAAGGTAAGAAGCC

CCACTACGAAAGTGGCTTTAACCACACCCGACCCCACGAAAGCTGTGAAA

>628_Sample_ASV17

CACCGCGGTTATACGAGAGGCCCCAGTTGATATTAGCGGCGTAAAGGGTGGTTAAGGACT

TTAATAACTAAAGCTAAACACTTCCCCGGCTGTTGTACGTAGACCGGAAGCTTGAAGCCC

AGACACGAAAGTAGCTTTATTCCCCCTGACCCCACGAAAGCTGAGAAA

>628_Sample_ASV18

CACCGCGGTCACACGATTAACCCAAGTCAATAGAAGCCGGCGTAAAGAGTGTTTTAGATC

ACCCCCTCCCCAATAAAGCTAAAACTCACCTGAGTTGTAAAAAACTCCAGTTGACACAAA

ATAGACTACGAAAGTGGCTTTAACATATCTGAACACACAATAGCTAAGACC

>628_Sample_ASV19

CACCGCGGTTATACGAGAAACCCTAGTTGACAAATACGGCGTAAAGAGTGGTTAAGGGGT

ATCAAAAATAAAGTCAAACATTTTCCTAGTTGTTATAAGCATTTCGAGAACATGAAGCCC

AACCACGAAAGTAACTTTACCTTCACCTGACCCCACGAAAGCTAAGAAA

>628_Sample_ASV20

CACCGCGGTTATACGAAAGACCCCAGTTTATAGACACGGCGTAAAGGGTGGTTATAGGAC

AAACTAACATAGAGCCAAAGACCCCCTAAGCCGTCATACGCTCACGAGGGCTCGAAGCCC

CTATACGAAAGTAGCTCTACCAAAAAATGCTCGACTCCACGAAAGCTGGGACA

>628_Sample_ASV21

CACCGCGGTTATACGAGAGACCCTAGTTGATAAATACGGCGTAAAGAGTGGTTATGGGAA

AAACAAATAAAGTCAAACAACCTCTTAGCTGTTATACGCATTATGAGAGCATGAAGCCCC

CTCACGAAAGTAACTTTAATACCTCCTGACCCCACGAAAGCTAAGAAA

>628_Sample_ASV22

CACCGCGGTTATACGAGAGACCCTAGTTGATATATACGGCGTAAAGAGTGGTTATGGGAA

AAACAAATAAAGTCAAACAACCTCTTAGCTGTTATACGCATTATGAGAGCATGAAGCCCC

CTCACGAAAGTAACTTTAATACCTCCTGACCCCACGAAAGCTAAGAAA

>628_Sample_ASV23

AACCGCGGTTATACGAGAGACCCTAATTGATAGCTCACGGCGTAAAGGGTGGTTTGGGGA

AACCTCAAAATAAAGCTAAAGACCTTCTAAGCCGTTACACGCATACCGAAGGCACGAGGC

CCTAACACGAAAGTAGCTTTACTATTACCCCTGACGCCACGAAAGCTAAGAAA

>628_Sample_ASV24

CACCGCGGTTATACGAAAGACCCTAGTTGATGGATCACGGCGTAAAGGGTGGTTAAGGGA

TATAAAAATAAAGCTAAAAATCCTCTTAGCTGTCATACGCTTTCCGAAGACATGAGGCCC

AATTACGAAAGTAGCTTTAAACCACCCTGACACCACGAAAGCCAAGAAA

>628_Sample_ASV25

CACCGCGGTTATACGAGAGACCCTAGTTGATAGCCACGGCGTAAAGGGTGGTTAAGGTAT

ACAACAAATAAAGCTAAATAGCCTCTAGGCCGTCGCACGCATTCCGAGAGCTCGAAACCC

AAAAACGAAAGTAGCTTTAAAATAAACAGACCTGACCCCACGAAAGCTAAGAAA

>628_Sample_ASV26

CACCGCGGTTATACGAAAGACCCTAGTTGATAGCCACGGCGTAAAGGGTGGTTAAGGTAT

ACAACAAATAAAGCTAAATAGCCTCTAGGCCGTCGCACGCATTCCGAGAGCTCGAAACCC

AAAAACGAAAGTAGCTTTAAAATAAACAGACCTGACCCCACGAAAGCTAAGAAA

>628_Sample_ASV27

CACCGCGGTTATACGAGAGGCCCAAGTTGATACTCCACGGCGTAAAGAGTGGTTAGAAAA

GACCTACTACTAAAGCCGAACGCCTTCAAAGCTGTTATACGCATCCGAAGGTGAGAAGCC

CACCCACGAAAGTGGCTTTACAACCTTGACTCCACGAAAGCTATGACA

>628_Sample_ASV28

CACCGCGGTTATACGAGAGACCCCAGTTGATAAACACGGCGTAAAGAGTGGTTAAGGGCA

CAAAACAAATAAAGTCAAACAACCTCTTAGCTGTTATACGCATTATGAGGATATGAAGCC

CCCCTACGAAAGTAACTTTACCAACCCCTGACCCCACGAAAGCTAAGAAA

>628_Sample_ASV29

CACCGCGGTTATACGAAAGACCCTAGTTTATAGGCACGGCGTAAAGGGTGGTTAGGGGAT

AAACAAAATAAAGCCAAAGACCTTCTAAGTCGTCATACACTCATGAAGGCACGAAGCCCA

AATACGAAAGTAACTTTACAACTTAGACCCGACTCCACGAAAGCTGGGGGA

>628_Sample_ASV30

CACCGCGGTTATACGAGAGACCCTAATTAATAGATACGGCGTAAAGAGTGGTTTAGGACA

AAAATTAATAAAGCCGAAGTTTCTCCTAGCCGTTGTACGCACTCTGAGAACACGAAGCCC

CACTACGAAAGTAGCTTTACCTATTATTTCCTGACCCCACGAAAGCTAAGAAA

>628_Sample_ASV31

CACCGCGGTTATACGAGAGGCCCAAGTTGATAGACATCGGCGTAAAGAGTGGTTAAGGAA

GTCTCAAAACTAAAGCCGAATGCCTTCAAGACTGTTATACGTTTCCGAAGGTAAGAAGCC

CCACTACGAAAGTGGCTTTATTCCACCTGACCCCACGAAAGCTGTGAAA

>628_Sample_ASV32

CACCGCGGTTATACGAGAGGCCCAAGTTGATAGACATCGGCGTAAAGAGTGGTTAAGGAA

GTCTCAAAACTAAAGCCGAATGCCTTCAAGACTGTTATACGTTTCCGAAGGTAAGAAGCC

CCACTACGAAAGTGGCTTTACTCCACCTGACTCCACGAAAGCTGTGAAA

>628_Sample_ASV33

CACCGCGGTTATACGAGAGACTCCAGTTGATAGCTACGGCGTAAAGAGTGGTTTGGGGCC

CGCCCAAAATAAAGCCAAAGACCTCCCAAGCCGTCAAACGCACCCCGGAGGCACGAAGTC

CTAACGCGAAAGCAACTTTACCTTCCCCGACGCCACGAAAGCTAAGAAA

>628_Sample_ASV34

CACCGCGGTTATACGAAAGACCCTAGTTGATAGATCACGGCGTAAAGGGTGGTTAAGGAG

AACAAAAATAAAGCTAAAAATCCTCTAAGCTGTCATACGCTTTCCGAAGACATGAGACCC

AACCACGAAAGTAACTTTAAACTTTCCTGACACCACGAAAGCCAAGAAA

>631_Sample_ASV1

TACCGCGGTTATACGAAAGACCCAAGTTAATAAACTACGGCGTAAAGCGTGGTTAGAGAC

CTCCAATAATTAAAGCCAAAGACCTTCTAAGCCGTCGCACGCCCCGCGAAGACACGAAGC

CCAAAAACGAAAGTAGCTTTATTCTTACTCGACCCCACGAAAGCTAGGGCA

>631_Sample_ASV2

CACCGCGGTTATACGAAAGACCCTAGTTGATGGGTGCGGCGTAAAGGGTGGTTAGGGACT

ACAAAAAATAGAGCTAAAGATCCTCTAGGTCGTCATACACATCACGAGGAAACGAAGCCC

AAATACGAAAGTAGCTCTAAATATAATCCTGACGCCACGAAAGCCAAGAAA

>631_Sample_ASV3

CACCGCGGTTATACGAGAGACCCTAGTTGATTAAAGCGGCGTAAAGAGTGGTTATGGAAC

TATTCTTTTAAAGCAGAAAACCTCTCAAACTGTTATACGCACCCAGAGGTCTGAATCCCT

CACACGAAAGTGACTTTATTTATGCCTACCAGAACCCACGAAAGCTGGGACA

>631_Sample_ASV4

CATGGCGATTATACGAGAGGCCCAAGTTGACAGACACCGGCGTAAAGAGTGGCTAGGGAA

AAATTACTACTAAAGCCGAACACCTTCAGAACTGTCATACGTTCCCGAAGATAAGAAGCC

CCACCACGAAAGTGGCTTTATATTACCCGACCCCACGAAAGCTGTGAAA

>631_Sample_ASV5

CACCGCGGTTATACGAGAGGCCCAAGTTGACAGACACCGGCGTAAAGAGTGGCTAGGGAA

AAATTACTACTAAAGCCGAACACCTTCAGAACTGTCATACGTTCCCGAAGATAAGAAGCC

CCACCACGAAAGTGGCTTTATATTACCCGACCCCACGAAAGCTGTGAAA

>631_Sample_ASV6

CACCGCGGTTATACGAGAGGCCCAAGTTGACAGACACCGGCGTAAAGAGTGGCTAGGGAA

AAATTACTACTAAAGCCGAACACCTTCAGAACTGTCATACGTTCCCGAAGATAAGAAGCC

CCACCACGAAAGTGGCTTTATAATACGTGACCCCACGAAAGCTGTGAAA

>631_Sample_ASV7

CACCGCGATTATACGAGAGGCCCAAGTTGACAGACATCGGCGTAAAGAGTGGTTAAGGAA

AAATTACTGCTAAAGCCGAACACCTTCAGAACTGTCATACGTTCCCGAAGATAAGAAGCC

CCACCACGAAAGTGGCTTTATATTACCCGACCCCACGAAAGCTGTGAAA

>631_Sample_ASV8

CACCGCGATTATACGAGAGGCCCAAGTTGACAGACATCGGCGTAAAGAGTGGTTAAGGAA

AAATTACTACTAAAGCCGAACACCTTCAGAACTGTCATACGTTCCCGAAGATAAGAAGCC

CCACCACGAAAGTGGCTTTATATTACCCGACCCCACGAAAGCTGTGAAA

>631_Sample_ASV9

CACCGCGGTTATACGAGAGGCCCAAGTTGACAGACACCGGCGTAAAGAGTGGCTAGGGAA

AAATTACTACTAAAGCCGAACACCCTCAGAACTGTCATACGTTCCAGAAGGCAAGAAGCT

CCACCACGAAAGTGGCTTTATATTACCCGACCCCACGAAAGCTGTGAAA

>631_Sample_ASV10

CATGGCGATTATACGAGAGGCCCAAGTTGACAGACACTGGCGTAAAGAGTGGCTAAGGAA

AAACTACTACTAAAGCCGAACACCTTCAGAACTGTCATACGTTCCCGAAGATAAGAAGCC

CCACCACGAAAGTGGCTTTATATTACCCGACCCCACGAAAGCTGTGAAA

>631_Sample_ASV11

CATGGCGATTATACGAGAGGCCCAAGTTGACAGACACTGGCGTAAAGAGTGGCTAGGGAA

AAATTACTACTAAAGCCGAACACCTTCAGAACTGTCATACGTTCCCGAAGATAAGAAGCC

CCACCACGAAAGTGGCTTTATATTACCCGACCCCACGAAAGCTGTGAAA

>631_Sample_ASV12

CACCGCGGTTATACGAGAGGCCCAAGTTGACAGACACCGGCGTAAAGAGTGGCTAGGGAA

AAATTACTACTAAAGCCGAACACCTTCAGAACTGTCATACGTTCCAGAAGGCAAGAAGCT

CCACCACGAAAGTGGCTTTATATTACCCGACCCCACGAAAGCTGTGAAA

>631_Sample_ASV13

CACCGCGGTTATACGAGAGGCCCAAGTTGACAGACACCGGCGTAAAGAGTGGCTAGGGAA

AAATTACTACTAAAGCCGAACACCTTCAGAACTGTCATACGTTCCCGAAGATAAGAAGCC

CCACCACGAAAGTGGCTTTACAATGCCTGACCCCACGAAAGCTGTGAAA

>631_Sample_ASV14

CACCGCGGTTATACGAGAGGCCCAAGTTGACAGACACCGGCGTAAAGAGTGGCTAGGGAA

AAATTACTACTAAAGCCGAACACCTTCAGAACTGTCATACGTTCCCGAAGATAAGAAGCT

CCACCACGAAAGTGGCTTTACAATGCCTGACCCCACGAAAGCTGTGAAA

>631_Sample_ASV15

CACCGCGGTTATACGAGAGGCCCAAGTTGACAGACATCGGCGTAAAGAGTGGTTAAGGAA

AAATTACTGCTAAAGCCGAACACCCTCAGAACTGTCATACGTTCCCGAAGATAAGAAGCC

CCACCACGAAAGTGGCTTTATATTACCCGACCCCACGAAAGCTGTGAAA

>631_Sample_ASV16

CACCGCGGTTATACGAGAGGCCCAAGTTGACAGACACTGGCGTAAAGAGTGGCTAAGGAA

AAACTACTACTAAAGCCGAAAACCCTCAGAACTGTCATACGTTCCCGAAGATAAGAAGCC

CCACCACGAAAGTGGCTTTATATTACCCGACCCCACGAAAGCTGTGAAA

>631_Sample_ASV17

CACCGCGGTTATACGAGAGGCCCAAGTTGACAGACACCGGCGTAAAGAGTGGCTAGGGAA

AAATTACTGCTAAAGCCGAACACCCTCAGAACTGTCATACGTTCCAGAAGGCAAGAAGCC

CCACCACGAAAGTGGCTTTATATTACCCGACCCCACGAAAGCTGTGAAA

>631_Sample_ASV18

CATGGCGATTATACGAGAGGCCCAAGTTGACAGACACTGGCGTAAAGAGTGGCTAAGGAA

AAATTACTACTAAAGCCGAACACCTTCAGAACTGTCATACGTTCCCGAAGATAAGAAGCC

CCACCACGAAAGTGGCTTTATATTACCCGACCCCACGAAAGCTGTGAAA

>631_Sample_ASV19

CACCGCGGTTATACGAAAGACTCTAGTTGACAAACACGGCGTAAAGGGTGGTTAGGATAA

CAACAAAATAAAGCTAAAGACTTACCAAGCCGTCATACGCCCATGTAAGAGCGAAAACCA

CAAACGAAAGTAACTTTATTACACCATCCGAATCCACGAAAGCTAAGAAA

>631_Sample_ASV20

CACCGCGGTTATACGAAAGACCCTAGTTGATAGATCACGGCGTAAAGGGTGGTTAGGGAA

TACAAAAATAAAGCTAAAAATCCTCTAAGCTGTCATACGCTTCCCGAAGACATGAAACCC

AACCACGAAAGTAGCTTTAAACTCTCCTGACACCACGAAAGCCAAGGAA

>631_Sample_ASV21

CACCGCGGTTATACGAGAGGCCCAAGTTGATAAGCCCGGCGTAAAGAGTGGTTATGGAGA

ATAAAACACTAAAGCTAAAGACCCCCTAGGCTGTTATACGCACCTGGCGGCTCGAACCAC

CTATACGAAAGTAGCTTTACCCCCCTTCCACCAGAATCCACGACAGCTGGGGCA

>631_Sample_ASV22

CACCGCGGTTATACGAGAGGCCCAAGTTGACAGACATCGGCGTAAAGAGTGGTTAAGGAA

ACCCCATTACTAAAGCCGAACGCCCTCAGAACTGTCATACGTTCCCGAAGGTAAGAAGCC

CCACTACGAAAGTGGCTTTAACCACACCCGACCCCACGAAAGCTGTGAAA

>631_Sample_ASV23

CACCGCGGTTATACGAGAGGCCCCAGTTGATATTAGCGGCGTAAAGGGTGGTTAAGGACT

TTAATAACTAAAGCCAAACACTTCCCCGGCTGTTGTACGTAGACCGGAAGCTTGAAGCCC

AGACACGAAAGTAGCTTTATTTCCCCTGACCCCACGAAAGCTGAGAAA

>631_Sample_ASV24

CACCGCGGTTATACGAGAGGCCCCAGTTGATATTAGCGGCGTAAAGGGTGGTTAAGGACT

TTAATAACTAAAGCTAAACACTTCCCCGGCTGTTGTACGTAGACCGGAAGCTTGAAGCCC

AGACACGAAAGTAGCTTTATTTCCCCTGACCCCACGAAAGCTGAGAAA

>631_Sample_ASV25

CACCGCGGTTATACGAGAGGCCCCAGTTGATATTAGCGGCGTAAAGGGTGGTTAAGGACT

TTAATAACTAAAGCTAAACACTTCCCCGGCTGTTGTACGTAGACCGGAAGCTTGAAGCCC

AGACACGAAAGTAGCTTTATTCCCCCTGACCCCACGAAAGCTGAGAAA

>631_Sample_ASV26

CACCGCGGTTATACGAAAGACCCCAGTTTATAGACACGGCGTAAAGGGTGGTTATAGGAC

AAACTAACATAGAGCCAAAGACCCCCTAAGCCGTCATACGCTCACGAGGGCTCGAAGCCC

CTATACGAAAGTAGCTCTACCAAAAAATGCTCGACTCCACGAAAGCTGGGACA

>631_Sample_ASV27

CACCGCGGTTATACGAGAGACCCTAGTTGATAAATACGGCGTAAAGAGTGGTTATGGGAA

AAACAAATAAAGTCAAACAACCTCTTAGCTGTTATACGCATTATGAGAGCATGAAGCCCC

CTCACGAAAGTAACTTTAATACCTCCTGACCCCACGAAAGCTAAGAAA

>631_Sample_ASV28

CACCGCGGTTATACGAGAGACCCTAGTTGATATATACGGCGTAAAGAGTGGTTATGGGAA

AAACAAATAAAGTCAAACAACCTCTTAGCTGTTATACGCATTATGAGAGCATGAAGCCCC

CTCACGAAAGTAACTTTAATACCTCCTGACCCCACGAAAGCTAAGAAA

>631_Sample_ASV29

CACCGCGGTTATACGAGAGACCCTAATTGATAGGCACGGCGTAAAGAGTGGTTTAGGGTA

ACCCATAAATAAAGCTAAAGACCTTCTAAGCCGTCACACGCACACCGAAGGCACGAGACC

CCAACACGAAAGTAGCTTTACTTCTACCCCTGACGCCACGAAAGCTAAGAAA

>631_Sample_ASV30

CACCGCGGTTATACGAAAGACCCTAGTTGATGGATCACGGCGTAAAGGGTGGTTAAGGGA

TATAAAAATAAAGCTAAAAATCCTCTTAGCTGTCATACGCTTTCCGAAGACATGAGGCCC

AATTACGAAAGTAGCTTTAAACCACCCTGACACCACGAAAGCTGTGAAA

>631_Sample_ASV31

CACCGCGGTTATACGAAAGACCCTAGTTGATGGATCACGGCGTAAAGGGTGGTTAAGGGA

TATAAAAATAAAGCTAAAAATCCTCTTAGCTGTCATACGCTTTCCGAAGACATGAGGCCC

AATTACGAAAGTAGCTTTAAACCACCCTGACACCACGAAAGCCAAGAAA

>631_Sample_ASV32

CACCGCGGTTATACGAAAGACCCCAGTTTATAGATGCGGCGTAAAGGGTGGTTAAGAGAA

AATCAAAATAAGGCCAAAGATCCCCTAAGCAGTCATACGCTCCACGAGGACACGAAACCC

AAATACGAAAGTTGCCTTAATAAACTTCACCTGATCCCACGAAAGCTGAAAAA

>631_Sample_ASV33

CACCGCGGTTATACGAAAGGCTCTAGTTTATAAATACGGCGTAAAGGGTGGTTAAGGAAA

AATTAAAATAATGCTAAAGACCCCCTAAGCAGTTATACGCTTCACGGAGGCACGAAACCC

AAATACGAAAGTTGCCTTAATAAACTTTACCTGACCCCACGAAAGCTGAGAAA

>631_Sample_ASV34

CACCGCGGTTATACGAAAGGCTCTAGTTTATAAATACGGCGTAAAGGGTGGTTAAGGAAA

AATTAAAATAATGCTAAAGACCCCCTAAGCAGTTATACGCTTCACGGAGGCACGAAACCC

AAATACGAAAGTTGCCTTAATAAACTTTACCTGACCCCACGAAAGCTGAAAAA

>631_Sample_ASV35

CACCGCGGTTATACGAGAGACTCTAGTTGATACCAACGGCGTAAAGAGTGGTTTGGGATG

CCCCTTAAAATAAAGCCAAAGACCTCCCAAGCCGTCAAACGCACCCCGGAGGCACGAAGC

CCCAACGCGAAAGCAACTTTACCTCCCTCCCGACGCCACGAAAGCTAAGAAA

>631_Sample_ASV36

CACCGCGGTTATACGAGAGACCCTAGTTGATAGCTACGGCGTAAAGCGTGGTTAGGAGAA

CAAGATAAATAAAGTCAAACAATCTCTCGGCCGTTATACGTTATCTGAGAATATGAAGTC

CTACCACGAAAGTAACTTTAATTTTTCTGACCCCACGAAAACTGAGGAA

>631_Sample_ASV37

CACCGCGGTTATACGAGAGGCCCTAGTTGATAACTACCGGCGTAAAGAGTGGTTATGGAA

AAATATTTAATAAAGCCGAACACCCCCTCAGCCGTCATACGCACCTGGGAGCACGAAGAC

CTACTGCGAAAGCAGCTTTAATTATGCCTGACCCCACGACAGCTAAGAAA

>631_Sample_ASV38

CACCGCGGTTATACGAGAGGCCCAAGTTGATTATTACCGGCGTAAAGCGTGGTTAAGATT

TTTATAGGACTAAAGCCGAACCCCCTCAGAGCTGTTATACGCACCCGAAGGTTAGAAGTT

CAATCACGAAAGTGGCTTTACAACCCCGAACCCACGAAAGCTAAGATA

>631_Sample_ASV39

CACCGCGGTTATACGAGAGGCCCAAGTTGATACTCCACGGCGTAAAGAGTGGTTAGAAAA

GACCTACTACTAAAGCCGAACGCCTTCAAAGCTGTTATACGCATCCGAAGGTGAGAAGCC

CACCCACGAAAGTGGCTTTACAACCTTGACTCCACGAAAGCTATGACA

>631_Sample_ASV40

CACCGCGGTTATACGAGAGACCCCAGTTGATAAACACGGCGTAAAGAGTGGTTAAGGGCA

CAAAACAAATAAAGTCAAACAACCTCTTAGCTGTTATACGCATTATGAGGATATGAAGCC

CCCCTACGAAAGTAACTTTACCAACCCCTGACCCCACGAAAGCTAAGAAA

>631_Sample_ASV41

CACCGCGGTTATACGAAAGACCCTAGTTTATAGGCACGGCGTAAAGGGTGGTTAGGGGAT

AAACAAAATAAAGCCAAAGACCTTCTAAGTCGTCATACACTCATGAAGGCACGAAGCCCA

AATACGAAAGTAACTTTACAACTTAGACCCGACTCCACGAAAGCTGGGGGA

>631_Sample_ASV42

CACCGCGGTTATACGAGAGACTCCAGTTGATAGCTACGGCGTAAAGAGTGGTTTGGGGCC

CGCCCAAAATAAAGCCAAAGACCTCCCAAGCCGTCAAACGCACCCCGGAGGCACGAAGTC

CTAACGCGAAAGCAACTTTACCTTCCCCGACGCCACGAAAGCTAAGAAA

>631_Sample_ASV43

CACCGCGGTTATACGAAAGACCCTAGTTGATAGCTACGGCGTAAAGGGTGGTTAAGGTAT

ATAACAAATAAAGCTAAAGAGCCTCCAAGCCGTCGTACGCATTCTGAGAGCTCGAAACCC

TAACACGAAAGTAGCTTTAAAAAATAACACCTGACCCCACGAAAGCTAAGAAA

>631_Sample_ASV44

CACCGCGGTTATACGAAAGACCCTAGTTGATAGATCACGGCGTAAAGGGTGGTTAAGGAG

AACAAAAATAAAGCTAAAAATCTTCTAAGCTGTCATACGCTTTCCGAAGACATGAGACCC

AACCACGAAAGTAGCTTTAAACTTTCCTGACACCACGAAAGCCAAGAAA

>665_Sample_ASV1

CACCGCGGTTATACGAAAGACCCTAGTTGATGGGTGCGGCGTAAAGGGTGGTTAGGGACT

ACAAAAAATAGAGCTAAAGATCCTCTAGGTCGTCATACACATCACGAGGAAACGAAGCCC

AAATACGAAAGTAGCTCTAAATATAATCCTGACGCCACGAAAGCCAAGAAA

>665_Sample_ASV2

CACCGCGGTTATACGAGAGACCCTAGTTGATTAAAGCGGCGTAAAGAGTGGTTATGGAAC

TATTCTTTTAAAGCAGAAAACCTCTCAAACTGTTATACGCACCCAGAGGTCTGAATCCCT

CACACGAAAGTGACTTTATTTATGCCTACCAGAACCCACGAAAGCTGGGACA

>665_Sample_ASV3

CACCGCGGTTATACGAAAGACCCTAGTTTATAGGTACGGCGTAAAGGGTGGTTAGGGGAC

AAACAAAATAAAGCCAAAGACCTCCTAAGCCGTCATACGCTCACGGAAGCACGAAGCCCA

AACACGAAAGTAGCTTTACCGAACATGCCCGACTCCACGAAAGCTGGGGAA

>665_Sample_ASV4

CACCGCGGTTATACGAGAGGCCCAAGTTGACAGACACCGGCGTAAAGAGTGGCTAGGGAA

AAATTACTACTAAAGCCGAACACCTTCAGAACTGTCATACGTTCCCGAAGATAAGAAGCC

CCACCACGAAAGTGGCTTTATATTACCCGACCCCACGAAAGCTGTGAAA

>665_Sample_ASV5

CACCGCGATTATACGAGAGGCCCAAGTTGACAGACATCGGCGTAAAGAGTGGTTAAGGAA

AAATTACTACTAAAGCCGAACACCTTCAGAACTGTCATACGTTCCCGAAGATAAGAAGCC

CCACCACGAAAGTGGCTTTATATTACCCGACCCCACGAAAGCTGTGAAA

>665_Sample_ASV6

CACCGCGGTTATACGAGAGGCCCAAGTTGACAGACACCGGCGTAAAGAGTGGCTAAGGAA

AAATTTCTACTAAAGCCGAGCACCTTCAGAACTGTCATACGTTCCCGAAGAAAAGAAGCC

CCACCACGAAAGTGGCTTTATATTACCCGACCCCACGAAAGCTGTGAAA

>665_Sample_ASV7

CACCGCGGTTATACGAAAGACTCTAGTTGACAAACACGGCGTAAAGGGTGGTTAGGATAA

CAACAAAATAAAGCTAAAGACTTACCAAGCCGTCATACGCCCATGTAAGAGCGAAAACCA

CAAACGAAAGTAACTTTATTACACCATCCGAATCCACGAAAGCTAAGAAA

>665_Sample_ASV8

CACCGCGGTTATACGAGAGACCCTAGTTGATATATACGGCGTAAAGAGTGGTTTGGGGTT

ATGCCTCCAAAATAAAGCCGAAGATCTCCCAAGCCGTCACACGCATCCCGGAGACACGAA

GCCCTAATACGAAGGTAGCTTTATTAGTACCCCGACGCCACGAAAGCTAAGAAA

>665_Sample_ASV9

CACCGCGGTTATACGAGAGACCCTAGTTGATATATACGGCGTAAAGAGTGGTTTGGGGTT

ATGCCTCCAAAATAAAGCCGAAGATCTCCCAAGCCGTCACACGCACCCCGGAGACACGAA

GCCCTAATACGAAGGTAGCTTTATTAATTCCCCGACGCCACGAAAGCTAAGAAA

>665_Sample_ASV10

CACCGCGGTTATACGAGAGACCCTAGTTGATATATACGGCGTAAAGAGTGGTTTGGGATA

TTTCTCCAAAATAAAGCCGAAGATCTCCCAAGCCGTCACACGCATCCCGGAGACACGAAG

CCCTAATACGAAGGTAGCTTTATTAGTACCCCGACGCCACGAAAGCTAAGAAA

>665_Sample_ASV11

CACCGCGGTTATACGAGAGGCCCAAGTTGATAAGCCCGGCGTAAAGAGTGGTTATGGAGA

ATAAAACACTAAAGCTAAAGACCCCCTAGGCTGTTATACGCACCTGGCGGCTCGAACCAC

CTATACGAAAGTAGCTTTACCCCCCTTCCACCAGAATCCACGACAGCTGGGGCA

>665_Sample_ASV12

CACCGCGGTTATACGAGAGGCCCTAGTTGATAGCCACGGCGCAAAGAGTGGTTAAGGAGC

CCCACTAAATAAAGTCGAACACTTCCTAGGCCGTTATACGTTTTCTAGAAGCACGAAACC

CAATTACACGAAAGCAACTTTATACTAAACAACCTGACCCCACGAAAGCTAAGAAA

>665_Sample_ASV13

CACCGCGGTTATACGAGAGGCCCAAGTTGACAGACATCGGCGTAAAGAGTGGTTAAGGAA

ACCCCATTACTAAAGCCGAACGCCCTCAGAACTGTCATACGTTCCCGAAGGTAAGAAGCC

CCACTACGAAAGTGGCTTTAACCACACCCGACCCCACGAAAGCTGTGAAA

>665_Sample_ASV14

CACCGCGGTTATACGAGAGGCCCAAGTTGACAGACACCGGCGTAAAGAGTGGTTAAGGAA

ATTCATCAACTAAAGCCGAATGCCCTCAGGACTGTTATACGTTTGCGAAGGTAAGAAGCC

CTACTACGAAAGTGGCTTTATTCCACCTGATTCCACGAAAGCTGCGGAA

>665_Sample_ASV15

CACCGCGGTTATACGAAAGACCCCAGTTTATAGACACGGCGTAAAGGGTGGTTATAGGAC

AAACTAACATAGAGCCAAAGACCCCCTAAGCCGTCATACGCTCACGAGGGCTCGAAGCCC

CTATACGAAAGTAGCTCTACCAAAAAATGCTCGACTCCACGAAAGCTGGGACA

>665_Sample_ASV16

CACCGCGGTTATACGAGAGGCCCCAGTTGATAAACACGGCGTAAAGGGTGGTTAAGGGAG

CATAAGAATAAAGCCGAAGGGCCCTTTGGCCGTTATACGCTTCTAGGTGTCCGAAGCCCA

AACACGAAAGTAGCTTTAGTTAAGCCCACCTGACTCCACGAAAACTGAGAAA

>665_Sample_ASV17

CACCGCGGTTATACGAGAGACCCTAGTTGATAAATACGGCGTAAAGAGTGGTTATGGGAA

AAACAAATAAAGTCAAACAACCTCTTAGCTGTTATACGCATTATGAGAGCATGAAGCCCC

CTCACGAAAGTAACTTTAATACCTCCTGACCCCACGAAAGCTAAGAAA

>665_Sample_ASV18

CACCGCGGTTATACGAGAGGCTCAAGTTGATGAACCCCGGCGTAAAGAGTGGTTAAGGGA

GATCAAAACTAAAGCCGAATGCTTTCAAAGCTGTTATACGCTTCCGAAAGTAAGAAGCTC

AATCACGAAAGTGGCTTTACTTTACCTGACCCCACGAAAGCTACGACA

>665_Sample_ASV19

CACCGCGGTTATACGAAAGACCCTAGTTGATGGATCACGGCGTAAAGGGTGGTTAAGGGA

TATAAAAATAAAGCTAAAAATCCTCTTAGCTGTCATACGCTTTCCGAAGACATGAGGCCC

AATTACGAAAGTAGCTTTAAACCACCCTGACACCACGAAAGCCAAGAAA

>665_Sample_ASV20

CACCGCGGTTATACGAAAGACCCCAGTTTATAGATGCGGCGTAAAGGGTGGTTAAGAGAA

AATCAAAATAAGGCCAAAGATCCCCTAAGCAGTCATACGCTCCACGAGGACACGAAACCC

AAATACGAAAGTTGCCTTAATAAACTTCACCTGATCCCACGAAAGCTGAAAAA

>665_Sample_ASV21

CACCGCGGTTATACGAGAGGCTCAAGTTGATGAACCCCGGCGTAAAGAGTGGTTAAGGAA

AATTTAAAACTAAAGCCGAATGCCCCCAAAGCTGTTATACGCACCCGAGGATAAGAAGCC

CAATCACGAAAGTGGCTTTACACTACCTGAACCCACGAAAGCTATGAAA

>665_Sample_ASV22

CACCGCGGTTATACGAGAGGCCCAAGTTGACAAACCCCGGCGTAAAGAGTGGTTAAGGAA

AATTTAAAACTAAAGCCGAATGCCCCCAAAGCTGTTATACGCACCCGAGGGTAAGAAGCC

CAATCACGAAAGTGGCTTTACACTACCTGAACCCACGAAAGCTATGAAA

>665_Sample_ASV23

CACCGCGGTTATACGAGAGGCCCAAGTTGACAAACCCCGGCGTAAAGAGTGGTTAAGGAA

AATTTAAAACTAAAGCCGAATGCCCCCAAAGCTGTTATACGCACCCGAGGATAAGAAGCC

CAATCACGAAAGTGGCTTTACACTACCTGAACCCACGAAAGCTATGAAA

>665_Sample_ASV24

CACCGCGGTTATACGAGAGGCCCTAGTTGATAACTACCGGCGTAAAGAGTGGTTATGGAA

AAATATTTAATAAAGCCGAACACCCCCTCAGCCGTCATACGCACCTGGGAGCACGAAGAC

CTACTGCGAAAGCAGCTTTAATTATGCCTGACCCCACGACAGCTAAGAAA

>665_Sample_ASV25

CACCGCGGTTATACGAAAGACCCTAGTTTATAGACACGGCGTAAAGGGTGGTTCGGGACT

AAACAAAATAAAGCCAAAGACCTCCTAAGCCGTCATACGCTCACGGAAGCACGAAGCCCA

AGTACGAAAGTAGCTTTACAAAACATGCCTGACTCCACGAAAGCTAGGAAA

>665_Sample_ASV26

CACCGCGGTTATACGAGAGGCCCAAGTTGATACTCCACGGCGTAAAGAGTGGTTAGAAAA

GACCTACTACTAAAGCCGAACGCCTTCAAAGCTGTTATACGCATCCGAAGGTGAGAAGCC

CACCCACGAAAGTGGCTTTACAACCTTGACTCCACGAAAGCTATGACA

>665_Sample_ASV27

CACCGCGGTTATACGAGAGACCCCAGTTGATAAACACGGCGTAAAGAGTGGTTAAGGGCA

CAAAACAAATAAAGTCAAACAACCTCTTAGCTGTTATACGCATTATGAGGATATGAAGCC

CCCCTACGAAAGTAACTTTACCAACCCCTGACCCCACGAAAGCTAAGAAA

>665_Sample_ASV28

CACCGCGGTTATACGAAAGACCCTAGTTTATAGGCACGGCGTAAAGGGTGGTTAGGGGAT

AAACAAAATAAAGCCAAAGACCTTCTAAGTCGTCATACACTCATGAAGGCACGAAGCCCA

AATACGAAAGTAACTTTACAACTTAGACCCGACTCCACGAAAGCTGGGGGA

>665_Sample_ASV29

CACCGCGGTTATACGAGAGACCCTAATTAATAGATACGGCGTAAAGAGTGGTTTAGGACA

AAAATTAATAAAGCCGAAGTTTCTCCTAGCCGTTGTACGCACTCTGAGAACACGAAGCCC

CACTACGAAAGTAGCTTTACCTATTATTTCCTGACCCCACGAAAGCTAAGAAA

>672_Sample_ASV1

CACCGCGGTTATACGAGAGGCCCAAGTTGACAGACATCGGCGTAAAGAGTGGTTAAGGAA

AACCTTCAACTAAAGTCGAACGCTCTCAAGACTGTCGTACGTTTCCGAGAGTATGAAGCC

CCACCACGAAAGTGACTTTACCTCACCTGACTCCACGAAAGCTGCGAAA

>672_Sample_ASV2

CACCGCGGTTATACGAAAGACCCTAGTTGATGGGTGCGGCGTAAAGGGTGGTTAGGGACT

ACAAAAAATAGAGCTAAAGATCCTCTAGGTCGTCATACACATCACGAGGAAACGAAGCCC

AAATACGAAAGTAGCTCTAAATATAATCCTGACGCCACGAAAGCCAAGAAA

>672_Sample_ASV3

CACCGCGGTTATACGAAAGACCCTAGTTGATGGGTGCGGCGTAAAGGGTGGTTAGGGACT

ACAAAAAATAGAGCTAAAGATCCTCTAGGTCGTCATACACATCACGAGAAAACGAAGCCC

AAATACGAAAGTAGCTCTAAATATAATCCTGACGCCACGAAAGCCAAGAAA

>672_Sample_ASV4

CACCGCGGTTATACGAGAGACCCTAGTTGATTAAAGCGGCGTAAAGAGTGGTTATGGAAC

TATTCTTTTAAAGCAGAAAACCTCTCAAACTGTTATACGCACCCAGAGGTCTGAATCCCT

CACACGAAAGTGACTTTATTTATGCCTACCAGAACCCACGAAAGCTGGGACA

>672_Sample_ASV5

CACCGCGGTTATACGAAAGACCCTAGTTTATAGGTACGGCGTAAAGGGTGGTTAGGGGAC

AAACAAAATAAAGCCAAAGACCTCCTAAGCCGTCATACGCTCACGGAAGCACGAAGCCCA

AACACGAAAGTAGCTTTACCGAACATGCCCGACTCCACGAAAGCTGGGGAA

>672_Sample_ASV6

CACCGCGGTTATACGAGAGGCCCAAGTTGACAGACACCGGCGTAAAGAGTGGCTAGGGAA

AAATTACTGCTAAAGCCGAACACCCTCAGAACTGTCATACGTTCCAGAAGGCAAGAAGCC

CCACCACGAAAGTGGCTTTATATTACCCGACCCCACGAAAGCTGTGAAA

>672_Sample_ASV7

CACCGCGGTTATACGAGAGGCCCAAGTTGACAGACATCGGCGTAAAGAGTGGTTAAGGAA

AAATTACTGCTAAAGCCGAACACCTTCAGAACTGTCATACGTTCCCGAAGATAAGAAGCC

CCACCACGAAAGTGGCTTTATATTACCCGACCCCACGAAAGCTGTGAAA

>672_Sample_ASV8

CACCGCGGTTATACGAGAGGCCCAAGTTGACAGACACCGGCGTAAAGAGTGGCTAGGGAA

AAATTACTACTAAAGCCGAACACCTTCAGAACTGTCATACGTTCCAGAAGGCAAGAAGCT

CCACCACGAAAGTGGCTTTATATTACCCGACCCCACGAAAGCTGTGAAA

>672_Sample_ASV9

CACCGCGATTATACGAGAGGCCCAAGTTGACAGACATCGGCGTAAAGAGTGGTTAAGGAA

AAATTACTACTAAAGCCGAACACCTTCAGAACTGTCATACGTTCCCGAAGATAAGAAGCC

CCACCACGAAAGTGGCTTTATATTACCCGACCCCACGAAAGCTGTGAAA

>672_Sample_ASV10

CACCGCGATTATACGAGAGGCCCAAGTTGACAGACATCGGCGTAAAGAGTGGTTAAGGAA

AAATTACTGCTAAAGCCGAACACCTTCAGAACTGTCATACGTTCCCGAAGATAAGAAGCC

CCACCACGAAAGTGGCTTTATATTACCCGACCCCACGAAAGCTGTGAAA

>672_Sample_ASV11

CACCGCGGTTATACGAGAGGCCCAAGTTGACAGACATCGGCGTAAAGAGTGGTTAAGGAA

AAATTACTGCTAAAGCCGAACACCCTCAGAACTGTCATACGTTCCCGAAGATAAGAAGCC

CCACCACGAAAGTGGCTTTATATTACCCGACCCCACGAAAGCTGTGAAA

>672_Sample_ASV12

CACCGCGGTTATACGAGAGGCCCAAGTTGACAGACACCGGCGTAAAGAGTGGCTAGGGAA

AAATTACTACTAAAGCCGAACACCTTCAGAACTGTCATACGTTCCCGAAGATAAGAAGCC

CCACCACGAAAGTGGCTTTATAATACGTGACCCCACGAAAGCTGTGAAA

>672_Sample_ASV13

CACCGCGGTTATACGAGAGGCCCAAGTTGACAGACACCGGCGTAAAGAGTGGCTAGGGAA

AAATTACTACTAAAGCCGAACACCTTCAGAACTGTCATACGTTCCCGAAGATAAGAAGCC

CCACCACGAAAGTGGCTTTATATTACCCGACCCCACGAAAGCTGTGAAA

>672_Sample_ASV14

CACCGCGGTTATACGAGAGGCCCAAGTTGACAGACACCGGCGTAAAGAGTGGCTAGGGAA

AAATTACTACTAAAGCCGAACACCCTCAGAACTGTCATACGTTCCAGAAGGCAAGAAGCT

CCACCACGAAAGTGGCTTTATATTACCCGACCCCACGAAAGCTGTGAAA

>672_Sample_ASV15

CACCGCGGTTATACGAGAGGCCCAAGTTGACAGACACCGGCGTAAAGAGTGGCTAGGGAA

AAATTACTACTAAAGCCGAACACCTTCAGAACTGTCATACGTTCCCGAAGATAAGAAGCT

CCACCACGAAAGTGGCTTTATAATACGTGACCCCACGAAAGCTGTGAAA

>672_Sample_ASV16

CACCGCGGTTAAACGAGAGACCCTAGTTGATCATCACGGCGTAAAGAGTGGTTAGGGGAT

TATCATAAATAAAGCCAAAGACCTCCCAAGCTGTCGCACGCATTCCGGAGGCGCGAAGCC

CACACACGAAAGTAGCTTTAACTATTGACCCTGATGCCACGAAAGCTAAGAAA

>672_Sample_ASV17

CACCGCGGTTATACGAGAGGCCCAAGTTGATAAGCCCGGCGTAAAGAGTGGTTATGGAGA

ATAAAACACTAAAGCTAAAGACCCCCTAGGCTGTTATACGCACCTGGCGGCTCGAACCAC

CTATACGAAAGTAGCTTTACCCCCCTTCCACCAGAATCCACGACAGCTGGGGCA

>672_Sample_ASV18

CACCGCGGTTATACGAGAGGCCCAAGTTGACAGACATCGGCGTAAAGAGTGGTTAAGGAA

ACCCCATTACTAAAGCCGAACGCCCTCAGAACTGTCATACGTTCCCGAAGGTAAGAAGCC

CCACTACGAAAGTGGCTTTAACCACACCCGACCCCACGAAAGCTGTGAAA

>672_Sample_ASV19

CACCGCGGTTATACGAGAGGCCCTAGTTGATAAATACGGCGTAAAGAGTGGTTAGAGGAC

TCCCATAATAAAGCCAAAGACCCCCCGGGCCGTCACACGCATACGAGGGCACGAAGTTCA

CACACGAAAGTAGCTTTAAATTACCCGACGCCACGAAAGCTAAGAAA

>672_Sample_ASV20

GACCGCGGTTATACGAGAGACTCTAGTTGATAGATACGGCGTAAAGAGTGGTTTGGGATA

AACTATAAATAAAGCCAAAGACCTTCCAAGCTGTTAAACGCATACCGAAGGCACGAAGCC

CCAACACGAAAGTAGCTTTACTACTACCCCCGATGCCACGAAAGCTAAGACA

>672_Sample_ASV21

CACCGCGGTTATACGAGAGACCCTAGTTGATAAATACGGCGTAAAGAGTGGTTATGGGAA

AAACAAATAAAGTCAAACAACCTCTTAGCTGTTATACGCATTATGAGAGCATGAAGCCCC

CTCACGAAAGTAACTTTAATACCTCCTGACCCCACGAAAGCTAAGAAA

>672_Sample_ASV22

CACCGCGGTCATACGATTAACCCAAGTTAATAGGCACACGGCGTAAAGCGTGTTTAAGCA

CTATGCCAAATAAAGTTAAACTCCAATTAAGCTGTAAAAAGCCATAATCAAGACGAAAAT

AAATAACGAAAGTAACTTTACAACCGCTGAAACACGATAGCTAAGATC

>672_Sample_ASV23

CACCGCGGTTATACGAAAGACCCTAGTTTATAGACACGGCGTAAAGGGTGGTTCGGGACT

AAACAAAATAAAGCCAAAGACCTCCTAAGCCGTCATACGCTCACGGAAGCACGAAGCCCA

AGTACGAAAGTAGCTTTACAAAACATGCCTGACTCCACGAAAGCTAGGAAA

>672_Sample_ASV24

CACCGCGGTTATACGAAAGACCCTAGTTGATAGGCACGGCGTAAAGGGTGGTTAGGGATA

TATCAAAAATAAAGTTAAAGAGCCTCTAAGCTGTCGCACGCATCCCGAGAGCTCGAGACC

CAGACACGAAAGTAACTTTAAAATACCTCACCTGACCCCACGAAAGCTAAGAAA

>672_Sample_ASV25

CACCGCGGTTATACGAAAGACCCTAGTTGATAGCCACGGCGTAAAGGGTGGTTAAGGTAT

ACAACAAATAAAGCTAAATAGCCTCTAGGCCGTCGCACGCATTCCGAGAGCTCGAAACCC

AAAAACGAAAGTAGCTTTAAAATAAACAGACCTGACCCCACGAAAGCTAAGAAA

>672_Sample_ASV26

CACCGCGGTTATACGAGAGGCCCAAGTTGATACTCCACGGCGTAAAGAGTGGTTAGAAAA

GACCTACTACTAAAGCCGAACGCCTTCAAAGCTGTTATACGCATCCGAAGGTGAGAAGCC

CACCCACGAAAGTGGCTTTACAACCTTGACTCCACGAAAGCTATGACA

>672_Sample_ASV27

CACCGCGGTTATACGAGAGACCCCAGTTGATAAACACGGCGTAAAGAGTGGTTAAGGGCA

CAAAACAAATAAAGTCAAACAACCTCTTAGCTGTTATACGCATTATGAGGATATGAAGCC

CCCCTACGAAAGTAACTTTACCAACCCCTGACCCCACGAAAGCTAAGAAA

>672_Sample_ASV28

CACCGCGGTTATACGAAAGACCCTAGTTTATAGGCACGGCGTAAAGGGTGGTTAGGGGAT

AAACAAAATAAAGCCAAAGACCTTCTAAGTCGTCATACACTCATGAAGGCACGAAGCCCA

AATACGAAAGTAACTTTACAACTTAGACCCGACTCCACGAAAGCTGGGGGA

>672_Sample_ASV29

CACCGCGGTTATACGAAAGACCCTAGTTGATAGCTACGGCGTAAAGGGTGGTTAAGGTAT

ATAACAAATAAAGCTAAAGAGCCTCCAAGCCGTCGTACGCATTCTGAGAGCTCGAAACCC

TAACACGAAAGTAGCTTTAAAAAATAACACCTGACCCCACGAAAGCTAAGAAA

>672_Sample_ASV30

CACCGCGGTTATACGAAAGACCCTAGTTGATAGATCACGGCGTAAAGGGTGGTTAAGGAG

AACAAAAATAAAGCTAAAAATCTTCTAAGCTGTCATACGCTTTCCGAAGACATGAGACCC

AACCACGAAAGTAGCTTTAAACTTTCCTGACACCACGAAAGCCAAGAAA

>698_Sample_ASV1

CACCGCGGTTATACGAGAGACCCTAGTTGATTAAAGCGGCGTAAAGAGTGGTTATGGAAC

TATTCTTTTAAAGCAGAAAACCTCTCAAACTGTTATACGCACCCAGAGGTCTGAATCCCT

CACACGAAAGTGACTTTATTTATGCCTACCAGAACCCACGAAAGCTGGGACA

>698_Sample_ASV2

CACCGCGGTTATACGAGAGACCCTAGTTGATTAAAGCGGCGTAAAGAGTGGTTATGGAAC

TATTCTTTTAAAGCAGAAAACCTCTCAAACTGTTATACGCACCCAGAGGTCTGAATCCCT

CACACGAAAGTGACTTTATTTATGCCTACCAGAACCCACGAAAGCTAAGAAA

>698_Sample_ASV3

CACCGCGGTTATACGAAAGACCCTAGTTTATAGGTACGGCGTAAAGGGTGGTTAGGGGAC

AAACAAAATAAAGCCAAAGACCTCCTAAGCCGTCATACGCTCACGGAAGCACGAAGCCCA

AACACGAAAGTAGCTTTACCGAACATGCCCGACTCCACGAAAGCTGGGGAA

>698_Sample_ASV4

CACCGCGGTTATACGAGAGGCCCAAGTTGACAGACACCGGCGTAAAGAGTGGCTAGGGAA

AAATTACTACTAAAGCCGAACACCTTCAGAACTGTCATACGTTCCCGAAGATAAGAAGCC

CCACCACGAAAGTGGCTTTATATTACCCGACCCCACGAAAGCTGTGAAA

>698_Sample_ASV5

CACCGCGATTATACGAGAGGCCCAAGTTGACAGACATCGGCGTAAAGAGTGGTTAAGGAA

AAATTACTACTAAAGCCGAACACCTTCAGAACTGTCATACGTTCCCGAAGATAAGAAGCC

CCACCACGAAAGTGGCTTTATATTACCCGACCCCACGAAAGCTGTGAAA

>698_Sample_ASV6

CACCGCGGTTATACGAGAGACCCTAGTTGATATATACGGCGTAAAGAGTGGTTTGGGATA

TTTCTCCAAAATAAAGCCGAAGATCTCCCAAGCCGTCACACGCATCCCGGAGACACGAAG

CCCTAATACGAAGGTAGCTTTATTAGTACCCCGACGCCACGAAAGCTAAGAAA

>698_Sample_ASV7

CACCGCGGTTATACGAGAGACCCTAGTTGATATATACGGCGTAAAGAGTGGTTTGGGGTT

ATGCCTCCAAAATAAAGCCGAAGATCTCCCAAGCCGTCACACGCATCCCGGAGACACGAA

GCCCTAATACGAAGGTAGCTTTATTAGTACCCCGACGCCACGAAAGCTAAGAAA

>698_Sample_ASV8

CACCGCGGTTATACGAGAGGCCCAAGTTGATAAGCCCGGCGTAAAGAGTGGTTATGGAGA

ATAAAACACTAAAGCTAAAGACCCCCTAGGCTGTTATACGCACCTGGCGGCTCGAACCAC

CTATACGAAAGTAGCTTTACCCCCCTTCCACCAGAATCCACGACAGCTGGGGCA

>698_Sample_ASV9

CACCGCGGTTATACGAGAGGCCCTAGTTGATAGCCACGGCGCAAAGAGTGGTTAAGGAGC

CCCACTAAATAAAGTCGAACACTTCCTAGGCCGTTATACGTTTTCTAGAAGCACGAAACC

CAATTACACGAAAGCAACTTTATACTAAACAACCTGACCCCACGAAAGCTAAGAAA

>698_Sample_ASV10

CACCGCGGTTATACGAGAGGCCCAAGTTGACAGACATCGGCGTAAAGAGTGGTTAAGGAA

ACCCCATTACTAAAGCCGAACGCCCTCAGAACTGTCATACGTTCCCGAAGGTAAGAAGCC

CCACTACGAAAGTGGCTTTAACCACACCCGACCCCACGAAAGCTGTGAAA

>698_Sample_ASV11

CACCGCGGTTATACGAGAGGCCCAAGTTGATAGATATCGGCGTAAAGAGTGGTTAAGGAA

ACCCCGTTACTAAAGCCGAACGCCCTCAGAACTGTCATACGTTCCCGAAGGTAAGAAGCC

CCACTACGAAAGTGGCTTTAACCACACCCGACCCCACGAAAGCTGTGAAA

>698_Sample_ASV12

CACCGCGGTTAAACGAGAGGCCCTAGTTGATATTACAACGGCGTAAAGGGTGGTTAAGGA

AAGCAAGCAATAAAGCCGAATGGCCCTTTGGCTGTCATACGCTTCTAGGTGTCCGAAGCC

CAATATACGAAAGTAGCTTTAGAAAAGCCCACCTGACACCACGAAAGCTGAGGAA

>698_Sample_ASV13

CACCGCGGTTATACGAGAGGCCCAAGTTGACAGACACCGGCGTAAAGAGTGGTTAAGGAA

ATTCATCAACTAAAGCCGAATGCCCTCAGGACTGTTATACGTTTGCGAAGGTAAGAAGCC

CTACTACGAAAGTGGCTTTATTCCACCTGATTCCACGAAAGCTGCGGAA

>698_Sample_ASV14

CACCGCGGTTATACGAGAGGCCCCAGTTGATAAACACGGCGTAAAGGGTGGTTAAGGGAG

CATAAGAATAAAGCCGAAGGGCCCTTTGGCCGTTATACGCTTCTAGGTGTCCGAAGCCCA

AACACGAAAGTAGCTTTAGTTAAGCCCACCTGACTCCACGAAAACTGAGAAA

>698_Sample_ASV15

CACCGCGGTTATACGAGAGACCCTAGTTGATAAATACGGCGTAAAGAGTGGTTATGGGAA

AAACAAATAAAGTCAAACAACCTCTTAGCTGTTATACGCATTATGAGAGCATGAAGCCCC

CTCACGAAAGTAACTTTAATACCTCCTGACCCCACGAAAGCTAAGAAA

>698_Sample_ASV16

CACCGCGGTTATACGAGAGGCTCAAGTTGATGAACCCCGGCGTAAAGAGTGGTTAAGGGA

GATCAAAACTAAAGCCGAATGCTTTCAAAGCTGTTATACGCTTCCGAAAGTAAGAAGCTC

AATCACGAAAGTGGCTTTACACTACCTGAACCCACGAAAGCTATGAAA

>698_Sample_ASV17

CACCGCGGTTATACGAGAGGCTCAAGTTGATGAACCCCGGCGTAAAGAGTGGTTAAGGGA

GATCAAAACTAAAGCCGAATGCTTTCAAAGCTGTTATACGCTTCCGAAAGTAAGAAGCTC

AATCACGAAAGTGGCTTTACTTTACCTGACCCCACGAAAGCTACGACA

>698_Sample_ASV18

CACCGCGGTTATACGAAAGACCCTAGTTGATGGATCACGGCGTAAAGGGTGGTTAAGGGA

TATAAAAATAAAGCTAAAAATCCTCTTAGCTGTCATACGCTTTCCGAAGACATGAGGCCC

AATTACGAAAGTAGCTTTAAACCACCCTGACACCACGAAAGCCAAGAAA

>698_Sample_ASV19

CACCGCGGTTATACGAAAGACCCCAGTTTATAGATGCGGCGTAAAGGGTGGTTAAGAGAA

AATCAAAATAAGGCCAAAGATCCCCTAAGCAGTCATACGCTCCACGAGGACACGAAACCC

AAATACGAAAGTTGCCTTAATAAACTTCACCTGATCCCACGAAAGCTGAAAAA

>698_Sample_ASV20

CACCGCGGTTATACGAGAGGCCCAAGTTGACAAACCCCGGCGTAAAGAGTGGTTAAGGAA

AATTTAAAACTAAAGCCGAATGCCCCCAAAGCTGTTATACGCACCCGAGGGTAAGAAGCC

CAATCACGAAAGTGGCTTTACACTACCTGAACCCACGAAAGCTATGAAA

>698_Sample_ASV21

CACCGCGGTTATACGAGAGGCCCAAGTTGACAAACCCCGGCGTAAAGAGTGGTTAAGGAA

AATTTAAAACTAAAGCCGAATGCCCCCAAAGCTGTTATACGCACCCGAGGATAAGAAGCC

CAATCACGAAAGTGGCTTTACACTACCTGAACCCACGAAAGCTATGAAA

>698_Sample_ASV22

CACCGCGGTTATACGAGAGGCTCAAGTTGATGAACCCCGGCGTAAAGAGTGGTTAAGGAA

AATTTAAAACTAAAGCCGAATGCCCCCAAAGCTGTTATACGCACCCGAGGATAAGAAGCC

CAATCACGAAAGTGGCTTTACACTACCTGAACCCACGAAAGCTATGAAA

>698_Sample_ASV23

CACCGCGGTTATACGAGAGGCCCTAGTTGATAACTACCGGCGTAAAGAGTGGTTATGGAA

AAATATTTAATAAAGCCGAACACCCCCTCAGCCGTCATACGCACCTGGGAGCACGAAGAC

CTACTGCGAAAGCAGCTTTAATTATGCCTGACCCCACGACAGCTAAGAAA

>698_Sample_ASV24

CACCGCGGTTATACGAAAGACCCTAGTTTATAGACACGGCGTAAAGGGTGGTTCGGGACT

AAACAAAATAAAGCCAAAGACCTCCTAAGCCGTCATACGCTCACGGAAGCACGAAGCCCA

AGTACGAAAGTAGCTTTACAAAACATGCCTGACTCCACGAAAGCTAGGAAA

>698_Sample_ASV25

CACCGCGGTTATACGAGAGGCCCAAGTTGATACTCCACGGCGTAAAGAGTGGTTAGAAAA

GACCTACTACTAAAGCCGAACGCCTTCAAAGCTGTTATACGCATCCGAAGGTGAGAAGCC

CACCCACGAAAGTGGCTTTACAACCTTGACTCCACGAAAGCTATGACA

>698_Sample_ASV26

CACCGCGGTTATACGAGAGACCCCAGTTGATAAACACGGCGTAAAGAGTGGTTAAGGGCA

CAAAACAAATAAAGTCAAACAACCTCTTAGCTGTTATACGCATTATGAGGATATGAAGCC

CCCCTACGAAAGTAACTTTACCAACCCCTGACCCCACGAAAGCTAAGAAA

>698_Sample_ASV27

CACCGCGGTTATACGAGAGACCCTAGTTGATATATACGGCGTAAAGAGTGGTTTGGAATA

ACCAAGTAATATAGCCAAAGACCTCCCAAGCCGTCACACGCACCCCGGAGGCACGAAGCC

CAAACACGAAAGTAGCTTTATTAACTTACCGACGCCACGAAAGCTAAGAAA

>698_Sample_ASV28

CACCGCGGTTATACGAAAGACCCTAGTTTATAGGCACGGCGTAAAGGGTGGTTAGGGGAT

AAACAAAATAAAGCCAAAGACCTTCTAAGTCGTCATACACTCATGAAGGCACGAAGCCCA

AATACGAAAGTAACTTTACAACTTAGACCCGACTCCACGAAAGCTGGGGGA

>698_Sample_ASV29

CACCGCGGTTATACGAGAGACCCTAATTAATAGATACGGCGTAAAGAGTGGTTTAGGACA

AAAATTAATAAAGCCGAAGTTTCTCCTAGCCGTTGTACGCACTCTGAGAACACGAAGCCC

CACTACGAAAGTAGCTTTACCTATTATTTCCTGACCCCACGAAAGCTAAGAAA

>698_Sample_ASV30

CACCGCGGTTATACGAAAGACCCTAGTTGATAGATCACGGCGTAAAGGGTGGTTAAGGAG

AACAAAAATAAAGCTAAAAATCTTCTAAGCTGTCATACGCTTTCCGAAGACATGAGACCC

AACCACGAAAGTAGCTTTAAACTTTCCTGACACCACGAAAGCCAAGAAA

>702_Sample_ASV1

CACCGCGGTTATACGAAAGACCCTAGTTGATGGGTGCGGCGTAAAGGGTGGTTAGGGACT

ACAAAAAATAGAGCTAAAGATCCTCTAGGTCGTCATACACATCACGAGGAAACGAAGCCC

AAATACGAAAGTAGCTCTAAATATAATCCTGACGCCACGAAAGCCAAGAAA

>702_Sample_ASV2

CACCGCGGTTATACGAAAGACCCTAGTTGCTAGCCACGGCGTAAAGGGTGGTTAAGGACA

ACAACAAATAAAGCTAAAGATCCCCTAAGCCGTCATACGCATTCCGGGGGCACGAAGCCC

TAACACGAAAGTAGCTTTAAAAAATATACCTGACCCCACGAAAGCTAAGAAA

>702_Sample_ASV3

CACCGCGGTTATACGAGAGACCCTAGTTGATTAAAGCGGCGTAAAGAGTGGTTATGGAAC

TATTCTTTTAAAGCAGAAAACCTCTCAAACTGTTATACGCACCCAGAGGTCTGAATCCCT

CACACGAAAGTGACTTTATTTATGCCTACCAGAACCCACGAAAGCTGGGACA

>702_Sample_ASV4

CACCGCGGTTATACGAAAGACCCTAGTTTATAGGTACGGCGTAAAGGGTGGTTAGGGGAC

AAACAAAATAAAGCCAAAGACCTCCTAAGCCGTCATACGCTCACGGAAGCACGAAGCCCA

AACACGAAAGTAGCTTTACCGAACATGCCCGACTCCACGAAAGCTGGGGAA

>702_Sample_ASV5

CACCGCGGTTATACGAGAGACCCTAGTTGATATACACGGCGTAAAGAGTGGTTTGGGGTA

TATAATAAATAAAGCCAAAGACCTTCTAGGCCGTTGAACGCACCACGAAGGAGCGAAGTC

CCAATACGAAAGTAGCTTTATAAAACCTGACGCCACGAAAGCTAAGAAA

>702_Sample_ASV6

CACCGCGGTTATACGAGAGACCCTAGTTGATAGACACGGCGTAAAGAGTGGCTAGGGAAA

AATTACTACTAAAGCCGAACACCTTCAGAACTGTCATACGTTCCCGAAGATAAGAAGCCC

CACCACGAAAGTGGCTTTATATTACCCGACCCCACGAAAGCTGTGAAA

>702_Sample_ASV7

CACCGCGGTTATACGAGAGGCCCAAGTTGACAGACACCGGCGTAAAGAGTGGCTAGGGAA

AAATTACTACTAAAGCCGAACACCTTCAGAACTGTCATACGTTCCCGAAGATAAGAAGCC

CCACCACGAAAGTGGCTTTATATTACCCGACCCCACGAAAGCTAAGAAA

>702_Sample_ASV8

CACCGCGGTTATACGAGAGGCCCAAGTTGACAGACACCGGCGTAAAGAGTGGCTAGGGAA

AAATTACTACTAAAGCCGAACACCTTCAGAACTGTCATACGTTCCCGAAGATAAGAAGCC

CCACCACGAAAGTGGCTTTATATTACCCGACCCCACGAAAGCTGTGAAA

>702_Sample_ASV9

CACCGCGGTTATACGAGAGACCCTAGTTGATAGACACGGCGTAAAGAGTGGCTAGGGAAA

AATTACTACTAAAGCCGAACACCTTCAGAACTGTCATACGTTCCCGAAGATAAGAAGCCC

CACCACGAAAGTGGCTTTATATTACCCGACCCCACGAAAGCTAAGAAA

>702_Sample_ASV10

CACCGCGGTTATACGAGAGGCCCAAGTTGACAGACACCGGCGTAAAGAGTGGCTAGGGAA

AAATTACTACTAAAGCCGAACACCTTCAGAACTGTCATACGTTCCCGAAGATAAGAAGCC

CCACCACGAAAGTGGCTTTAAACCAACCCGACCCCACGAAAGCTAAGAAA

>702_Sample_ASV11

CACCGCGGTTATACGAGAGGCCCAAGTTGACAGACACCGGCGTAAAGAGTGGCTAAGGAA

AAATTTCTACTAAAGCCGAACACCTTCAGAACTGTCATACGTTCCCGAAGAAAAGAAGCC

CCACCACGAAAGTGGCTTTATATTACCCGACCCCACGAAAGCTGTGAAA

>702_Sample_ASV12

CACCGCGGTTAAACGAGAGACCCTAGTTGATCATCACGGCGTAAAGAGTGGTTAGGGGAT

TATCATAAATAAAGCCAAAGACCTCCCAAGCTGTCGCACGCATTCCGGAGGCGCGAAGCC

CACACACGAAAGTAGCTTTAACTATTGACCCTGATGCCACGAAAGCTAAGAAA

>702_Sample_ASV13

CGCCGCGGTTAAACGAGAGGCCCTAGTTGATGGAACAACGGCGTAAAGGGTGGTTAAGGA

TAGTGAGACAATAAAGTCGAATGGCCCTTTGGCTGTCATACGCTTCTGGGAGCCTGAAGC

CCACTATACGAAAGTAGCTTTAACAACGTCCACCTGACCCCACGAAAACTGAGAAA

>702_Sample_ASV14

CGCCGCGGTTAAACGAGAGGCCCTAGTTGATGGAACAACGGCGTAAAGGGTGGTTAAGGA

TAGTGAGACAATAAAGTCGAATGGCCCTTTGGCTGTCATACGCTTCTGGGAGCCTGAAGC

CCACTATACGAAAGTAGCTTTAACAATGTCCACCTGACCCCACGAAAACTGAGAAA

>702_Sample_ASV15

CACCGCGGTTATACGAAAGACCCTAGTTGATAGATCACGGCGTAAAGGGTGGTTAGGGAA

TACAAAAATAAAGCTAAAAATCCTCTAAGCTGTCATACGCTTCCCGAAGACATGAAACCC

AACCACGAAAGTAGCTTTAAACTCTCCTGACACCACGAAAGCCAAGGAA

>702_Sample_ASV16

CACCGCGGTTATACGAGAGGCCCAAGTTGATAAGCCCGGCGTAAAGAGTGGTTATGGAGA

ATAAAACACTAAAGCTAAAGACCCCCTAGGCTGTTATACGCACCTGGCGGCTCGAACCAC

CTATACGAAAGTAGCTTTACCCCCCTTCCACCAGAATCCACGACAGCTGGGGCA

>702_Sample_ASV17

CACCGCGGTTATACGAGAGGCCCAAGTTGACAGACATCGGCGTAAAGAGTGGTTAAGGAA

ACCCCATTACTAAAGCCGAACGCCCTCAGAACTGTCATACGTTCCCGAAGGTAAGAAGCC

CCACTACGAAAGTGGCTTTAACCACACCCGACCCCACGAAAGCTGTGAAA

>702_Sample_ASV18

CACCGCGGTTAAACGAGAGGCCCTAGTTGATATTACAACGGCGTAAAGGGTGGTTAAGGA

AAGCAAGCAATAAAGCCGAATGGCCCTTTGGCTGTCATACGCTTCTAGGTGTCCGAAGCC

CAATATACGAAAGTAGCTTTAGAAAAGCCCACCTGACACCACGAAAGCTGAGGAA

>702_Sample_ASV19

CACCGCGGTCACACGATTAACCCAAGTCAATAGAAGCCGGCGTAAAGAGTGTTTTAGATC

ACCCCCTCCCCAATAAAGCTAAAACTCACCTGAGTTGTAAAAAACTCCAGTTGACACAAA

ATAGACTACGAAAGTGGCTTTAACATATCTGAACACACAATAGCTAAGACC

>702_Sample_ASV20

CACCGCGGTTATACGAGAGGCCCTAGTTGATAAATACGGCGTAAAGAGTGGTTAGAGGAC

TCCCATAATAAAGCCAAAGACCCTCCGGGCCGTCACACGCATACGAGGGCACGAAGTTCA

CACACGAAAGTAGCTTTAAATTACCCGACGCCACGAAAGCTAAGAAA

>702_Sample_ASV21

CACCGCGGTTATACGAGAAACCCTAGTTGACAAATACGGCGTAAAGAGTGGTTAAGGGGT

ATCAAAAATAAAGTCAAACATTTTCCTAGTTGTTATAAGCATTTCGAGAACATGAAGCCC

AACCACGAAAGTAACTTTACCTCCACCTGACCCCACGAAAGCTAAGAAA

>702_Sample_ASV22

CACCGCGGTTATACGAAAGACCCCAGTTTATAGACACGGCGTAAAGGGTGGTTATAGGAC

AAACTAACATAGAGCCAAAGACCCCCTAAGCCGTCATACGCTCACGAGGGCTCGAAGCCC

CTATACGAAAGTAGCTCTACCAAAAAATGCTCGACTCCACGAAAGCTGGGACA

>702_Sample_ASV23

CACCGCGGTTATACGAAAGACCCTAGTTTATAAGTACGGCGTAAAGGGTGGTTAGGGGAC

AAACAAAATAAAGCCAAAGACCTCCTAAGCCGTCATACGCTTACGGAGGCACGAAGCCCA

AACACGAAAGTAGCTTTACCGAACATGCCCGACTCCACGAAAGCTGGGAAA

>702_Sample_ASV24

CACCGCGGTTATACGAGAGACCCTAGTTGATAAATACGGCGTAAAGAGTGGTTATGGGAA

AAACAAATAAAGTCAAACAACCTCTTAGCTGTTATACGCATTATGAGAGCATGAAGCCCC

CTCACGAAAGTAACTTTAATACCTCCTGACCCCACGAAAGCTAAGAAA

>702_Sample_ASV25

CACCGCGGTTATACGAGAGGCTCAAGTTGATAAACCCCGGCGTAAAGAGTGGTTAAGGAA

AAATAGAAACTAAAGCCGAATGCTTTCAAGACTGTTATACGTTCCCGAGAGTAAGAAGAC

CAATTACGAAAGTGGCTTTACTTTACCTGACCCCACGAAAGCTACGACA

>702_Sample_ASV26

AACCGCGGTTATACGAGAGACCCTAATTGATAGCTCACGGCGTAAAGGGTGGTTTGGGGA

AACCTCAAAATAAAGCTAAAGACCTTCTAAGCCGTTACACGCATACCGAAGGCACGAGGC

CCTAACACGAAAGTAGCTTTACTATTACCCCTGACGCCACGAAAGCTAAGAAA

>702_Sample_ASV27

CACCGCGGTTATACGAAAGACCCTAGTTGATGGATCACGGCGTAAAGGGTGGTTAAGGGA

TATAAAAATAAAGCTAAAAATCCTCTTAGCTGTCATACGCTTTCCGAAGACATGAGGCCC

AATTACGAAAGTAGCTTTAAACCACCCTGACACCACGAAAGCCAAGAAA

>702_Sample_ASV28

CACCGCGGTTATACGAGAGACCCTAGTTGATAGCTACGGCGTAAAGCGTGGTTAGGAGAA

CAAGATAAATAAAGTCAAACAATCTCTCGGCCGTTATACGTTATCTGAGAATATGAAGTC

CTACCACGAAAGTAACTTTAATTTTTCTGACCCCACGAAAACTGAGGAA

>702_Sample_ASV29

CACCGCGGTTATACGAGAGACCCTAGTTGATAGCTACGGCGTAAAGCGTGGTTAGGAGAA

CAAGATAAATAAAGTCAAACAATCTCTCGGCCGTTATACGTTATCTGAGAATATGAAGTC

CTACCACGAAAGTAACTTTAATTTTTCTGACCCCACGAAAGCTAAGAAA

>702_Sample_ASV30

CACCGCGGTTAAACGAGAGGCCCTAGTTGATAGTACAACGGCGTAAAGGGTGGTTAAGGA

TAGTGAAATAATAAAGTCGAATGGCCCTTTGGCTGTCATACGCTTCTAGGAGTCCGAAGC

CCAATATACGAAAGTAACTTTAAGAGCGCCCACCTGACCCCACGAAAACTGAGAAA

>702_Sample_ASV31

CACCGCGGTCATACGATTAACCCAAGTTAATAGGCACACGGCGTAAAGCGTGTTTAAGCA

CTACGCCAAATAAAGTTAAACTCCAATTAAGCTGTAAAAAGCCATAATCAAGACGAAAAT

AAATAACGAAAGTAACTTTACAACCGCTGAAACACGATAGCTAAGATC

>702_Sample_ASV32

CACCGCGGTTATACGAGAGGCCCTAGTTGATAACTACCGGCGTAAAGAGTGGTTATGGAA

AAATATTTAATAAAGCCGAACACCCCCTCAGCCGTCATACGCACCTGGGAGCACGAAGAC

CTACTGCGAAAGCAGCTTTAATTATGCCTGACCCCACGACAGCTAAGAAA

>702_Sample_ASV33

CACCGCGGTTATACGAGAGGCCCAAGTTGATACTCCACGGCGTAAAGAGTGGTTAGAAAA

GACCTACTACTAAAGCCGAACGCCTTCAAAGCTGTTATACGCATCCGAAGGTGAGAAGCC

CACCCACGAAAGTGGCTTTACAACCTTGACTCCACGAAAGCTATGACA

>702_Sample_ASV34

CACCGCGGTTATACGAGAGACCCCAGTTGATAAACACGGCGTAAAGAGTGGTTAAGGGCA

CAAAACAAATAAAGTCAAACAACCTCTTAGCTGTTATACGCATTATGAGGATATGAAGCC

CCCCTACGAAAGTAACTTTACCAACCCCTGACCCCACGAAAGCTAAGAAA

>702_Sample_ASV35

CACCGCGGTTATACGAGAGACCCTAGTTGATATATACGGCGTAAAGAGTGGTTTGGAATA

ACCAAGTAATATAGCCAAAGACCTCCCAAGCCGTCACACGCACCCCGGAGGCACGAAGCC

CAAACACGAAAGTAGCTTTATTAACTTACCGACGCCACGAAAGCTAAGAAA

>702_Sample_ASV36

CACCGCGGTTATACGAAAGACCCTAGTTTATAGGCACGGCGTAAAGGGTGGTTAGGGGAT

AAACAAAATAAAGCCAAAGACCTTCTAAGTCGTCATACACTCATGAAGGCACGAAGCCCA

AATACGAAAGTAACTTTACAACTTAGACCCGACTCCACGAAAGCTGGGGGA

>702_Sample_ASV37

CACCGCGGTTATACGAAAGACCCTAGTTTATAGGCACGGCGTAAAGGGTGGTTAGGGGAT

AAACAAAATAAAGCCAAAGACCTTCTAAGTCGTCATACACTCATGAAGGCACGAAGCCCA

AATACGAAAGTAACTTTACAACTTAGACCCGACTCCACGAAAGCTAAGAAA

>702_Sample_ASV38

CACCGCGGTTATACGAGAGACCCTAATTAATAGATACGGCGTAAAGAGTGGTTTAGGACA

AAAATTAATAAAGCCGAAGTTTCTCCTAGCCGTTGTACGCACTCTGAGAACACGAAGCCC

CACTACGAAAGTAGCTTTACCTATTATTTCCTGACCCCACGAAAGCTAAGAAA

>702_Sample_ASV39

CACCGCGGTTATACGAGAGACCCTAATTAATAGCTACGGCGTAAAGAGTGGTTTAGGGTA

AAAATTAATAAAGCCGAAGTTTCTCCTAGCCGTTGTACGCACTCTGAGAACACGAAGCCC

CACTACGAAAGTAGCTTTACCTATTATTTCCTGACCCCACGAAAGCTAAGAAA

>702_Sample_ASV40

CACCGCGGTTATACGAGAGACCCTAATTAATAGATACGGCGTAAAGAGTGGTTTAGGGTA

AAAATTAATAAAGCCGAAGTTTCTCCTAGCCGTTGTACGCACTCTGAGAACACGAAGCCC

CACTACGAAAGTAGCTTTACCTATTATTTCCTGACCCCACGAAAGCTAAGAAA

>702_Sample_ASV41

CACCGCGGTTATACGAGAGACCCTAATTAATAGCTACGGCGTAAAGAGTGGTTTAGGGTA

AAAATTAATAAAGCCGAAGATTCTCCTAGCCGTTGTACGCACTCTGAGAACACGAAGCCC

CACTACGAAAGTAGCTTTACCTATTATTTCCTGACCCCACGAAAGCTAAGAAA

>702_Sample_ASV42

CACCGCGGTTATACGAGAGACCCTAATTAATAGCTACGGCGTAAAGAGTGGTTTAGGACA

AAAATTAATAAAGCCGAAGTTTCTCCTAGCCGTTGTACGCACTCTGAGAACACGAAGCCC

CACTACGAAAGTAGCTTTACCAATAATCTCCTGACCCCACGAAAGCTAAGAAA

>702_Sample_ASV43

CACCGCGGTTATACGAGAGACCCTAATTAATAGATACGGCGTAAAGAGTGGTTTAGGACA

AAAATTAATAAAGCCGAAGTTTCTCCTAGCCGTTGTACGCACTCTGAGAACACGAAGCCC

CACTACGAAAGTAGCTTTACCAATAATCTCCTGACCCCACGAAAGCTAAGAAA

>702_Sample_ASV44

CACCGCGGTTATACGAGAGACCCTAATTAATAGCTACGGCGTAAAGAGTGGTTTAGGACA

AAAATTAATAAAGCCGAAGTTTCTCCTAGCCGTTGTACGCACTCTGAGAACACGAAGCCC

CACTACGAAAGTAGCTTTACCTATTATTTCCTGACCCCACGAAAGCTAAGAAA

>702_Sample_ASV45

CACCGCGGTTATACGAGAGGCCCAAGTTGATAGACATCGGCGTAAAGAGTGGTTAAGGAA

GTCTCAAAACTAAAGCCGAATGCCTTCAAGACTGTTATACGTTTCCGAAGGTAAGAAGCC

CCACTACGAAAGTGGCTTTACTCCACCTGACTCCACGAAAGCTGTGAAA

>702_Sample_ASV46

CACCGCGGTTATACGAGAGACTCCAGTTGATAGCTACGGCGTAAAGAGTGGTTTGGGGCC

CGCCCAAAATAAAGCCAAAGACCTCCCAAGCCGTCAAACGCACCCCGGAGGCACGAAGTC

CTAACGCGAAAGCAACTTTACCTTCCCCGACGCCACGAAAGCTAAGAAA

>702_Sample_ASV47

CACCGCGGTTATACGAAAGACCCTAGTTGATAGCTACGGCGTAAAGGGTGGTTAAGGTAT

ATAACAAATAAAGCTAAAGAGCCTCCAAGCCGTCGTACGCATTCTGAGAGCTCGAAACCC

TAACACGAAAGTAGCTTTAAAAAATAACACCTGACCCCACGAAAGCTAAGAAA

>702_Sample_ASV48

CACCGCGGTTATACGAAAGACCCTAGTTGATAGACCACGGCGTAAAGGGTGGTTAAGGAA

CACAAAAATAAAGCTAAAGATCCTCTAAGCTGTCATACGCTTCCCGAAGACATGAAACCC

AATCACGAAAGTAGCTTTAAACTGTCCTGACACCACGAAAGCCAAGAAA

>702_Sample_ASV49

CACCGCGGTTATACGAAAGACCCTAGTTGATAGATCACGGCGTAAAGGGTGGTTAAGGAG

AACAAAAATAAAGCTAAAAATCCTCTAAGCTGTCATACGCTTTCCGAAGACATGAGACCC

AACCACGAAAGTAACTTTAAACTTTCCTGACACCACGAAAGCCAAGAAA

>704_Sample_ASV1

CACCGCGGTTATACGAAAGACCCTAGTTGATGGGTGCGGCGTAAAGGGTGGTTAGGGACT

ACAAAAAATAGAGCTAAAGATCCTCTAGGTCGTCATACACATCACGAGGAAACGAAGCCC

AAATACGAAAGTAGCTCTAAATATAATCCTGACGCCACGAAAGCCAAGAAA

>704_Sample_ASV2

CACCGCGGTTATACGAGAGACCCTAGTTGATTAAAGCGGCGTAAAGAGTGGTTATGGAAC

TATTCTTTTAAAGCAGAAAACCTCTCAAACTGTTATACGCACCCAGAGGTCTGAATCCCT

CACACGAAAGTGACTTTATTTATGCCTACCAGAACCCACGAAAGCTGGGACA

>704_Sample_ASV3

CACCGCGGTTATACGAGAGGCCCAAGTTGACAGACACCGGCGTAAAGAGTGGCTAGGGAA

AAATTACTACTAAAGCCGAACACCTTCAGAACTGTCATACGTTCCCGAAGATAAGAAGCC

CCACCACGAAAGTGGCTTTATATTACCCGACCCCACGAAAGCTGTGAAA

>704_Sample_ASV4

CACCGCGGTTATACGAGAGGCCCAAGTTGACAGACACCGGCGTAAAGAGTGGCTAGGGAA

AAATTACTACTAAAGCCGAACACCTTCAGAACTGTCATACGTTCCCGAAGATAAGAAGCC

CCACCACGAAAGTGGCTTTATATTACCCGACCCCACGAAAGCTAAGAAA

>704_Sample_ASV5

CACCGCGGTTATACGAGAGACCCTAGTTGACAGACACCGGCGTAAAGAGTGGCTAGGGAA

AAATTACTACTAAAGCCGAACACCTTCAGAACTGTCATACGTTCCCGAAGATAAGAAGCC

CCACCACGAAAGTGGCTTTATATTACCCGACCCCACGAAAGCTAAGAAA

>704_Sample_ASV6

CACCGCGGTTATACGAGAGGCCCAAGTTGACAGACACCGGCGTAAAGAGTGGCTAAGGAA

AAATTTCTACTAAAGCCGAACACCTTCAGAACTGTCATACGTTCCCGAAGAAAAGAAGCC

CCACCACGAAAGTGGCTTTATATTACCCGACCCCACGAAAGCTGTGAAA

>704_Sample_ASV7

CACCGCGGTTAAACGAGAGACCCTAGTTGATCATCACGGCGTAAAGAGTGGTTAGGGGAT

TATCATAAATAAAGCCAAAGACCTCCCAAGCTGTCGCACGCATTCCGGAGGCGCGAAGCC

CACACACGAAAGTAGCTTTAACTATTGACCCTGATGCCACGAAAGCTAAGAAA

>704_Sample_ASV8

CGCCGCGGTTAAACGAGAGGCCCTAGTTGATGGAACAACGGCGTAAAGGGTGGTTAAGGA

TAGTGAGACAATAAAGTCGAATGGCCCTTTGGCTGTCATACGCTTCTGGGAGCCTGAAGC

CCACTATACGAAAGTAGCTTTAACAATGTCCACCTGACCCCACGAAAACTGAGAAA

>704_Sample_ASV9

CACCGCGGTTATACGAAAGACCCTAGTTGATAGATCACGGCGTAAAGGGTGGTTAGGGAA

TACAAAAATAAAGCTAAAAATCCTCTAAGCTGTCATACGCTTCCCGAAGACATGAAACCC

AACCACGAAAGTAGCTTTAAACTCTCCTGACACCACGAAAGCCAAGGAA

>704_Sample_ASV10

CACCGCGGTTATACGAGAGGCCCAAGTTGATAAGCCCGGCGTAAAGAGTGGTTATGGAGA

ATAAAACACTAAAGCTAAAGACCCCCTAGGCTGTTATACGCACCTGGCGGCTCGAACCAC

CTATACGAAAGTAGCTTTACCCCCCTTCCACCAGAATCCACGACAGCTGGGGCA

>704_Sample_ASV11

CACCGCGGTTATACGAGAGGCCCTAGTTGATAGCCACGGCGCAAAGAGTGGTTAAGGAGC

CCCACTAAATAAAGTCGAACACTTCCTAGGCCGTTATACGTTTTCTAGAAGCACGAAACC

CAATTACACGAAAGCAACTTTATACTAAACAACCTGACCCCACGAAAGCTAAGAAA

>704_Sample_ASV12

CACCGCGGTCATACAAGAAACCCAAATCAATAGCTACCCGGCGTAAAGAGTGGCCACATG

TTATCTGCACCAGCTAAGATTAAAATGCAACCAAGCTGTCATAAGCCTAAGATCCACCTA

AACCCAACCCAAATCCATCTTAGCCTCAACGATTAATTTTAACCCACGAAAGCTAGGACC

>704_Sample_ASV13

CACCGCGGTTATACGAGAGGCCCAAGTTGACAGACATCGGCGTAAAGAGTGGTTAAGGAA

ACCCCATTACTAAAGCCGAACGCCCTCAGAACTGTCATACGTTCCCGAAGGTAAGAAGCC

CCACTACGAAAGTGGCTTTAACCACACCCGACCCCACGAAAGCTGTGAAA

>704_Sample_ASV14

CACCGCGGTTAAACGAGAGGCCCTAGTTGATATTACAACGGCGTAAAGGGTGGTTAAGGA

AAGCAAGCAATAAAGCCGAATGGCCCTTTGGCTGTCATACGCTTCTAGGTGTCCGAAGCC

CAATATACGAAAGTAGCTTTAGAAAAGCCCACCTGACACCACGAAAGCTGAGGAA

>704_Sample_ASV15

CACCGCGGTTATACGAGAGGCCCTAGTTGATAAATACGGCGTAAAGAGTGGTTAGAGGAC

TCCCATAATAAAGCCAAAGACCCTCCGGGCCGTCACACGCATACGAGGGCACGAAGTTCA

CACACGAAAGTAGCTTTAAATTACCCGACGCCACGAAAGCTAAGAAA

>704_Sample_ASV16

CACCGCGGTTATACGAGAAACCCTAGTTGACAAATACGGCGTAAAGAGTGGTTAAGGGGT

ATCAAAAATAAAGTCAAACATTTTCCTAGTTGTTATAAGCATTTCGAGAACATGAAGCCC

AACCACGAAAGTAACTTTACCTTCACCTGACCCCACGAAAGCTAAGAAA

>704_Sample_ASV17

CACCGCGGTTATACGAAAGACCCTAGTTTATAAGTACGGCGTAAAGGGTGGTTAGGGGAC

AAACAAAATAAAGCCAAAGACCTCCTAAGCCGTCATACGCTTACGGAGGCACGAAGCCCA

AACACGAAAGTAGCTTTACCGAACATGCCCGACTCCACGAAAGCTGGGAAA

>704_Sample_ASV18

CACCGCGGTTATACGAGAGACCCTAGTTGATAAATACGGCGTAAAGAGTGGTTATGGGAA

AAACAAATAAAGTCAAACAACCTCTTAGCTGTTATACGCATTATGAGAGCATGAAGCCCC

CTCACGAAAGTAACTTTAATACCTCCTGACCCCACGAAAGCTAAGAAA

>704_Sample_ASV19

CACCGCGGTTATACGAGAGGCTCAAGTTGATAAACCCCGGCGTAAAGAGTGGTTAAGGAA

AAATAGAAACTAAAGCCGAATGCTTTCAAGACTGTTATACGTTCCCGAGAGTAAGAAGAC

CAATTACGAAAGTGGCTTTACTTTACCTGACCCCACGAAAGCTACGACA

>704_Sample_ASV20

AACCGCGGTTATACGAGAGACCCTAATTGATAGCTCACGGCGTAAAGGGTGGTTTGGGGA

AACCTCAAAATAAAGCTAAAGACCTTCTAAGCCGTTACACGCATACCGAAGGCACGAGGC

CCTAACACGAAAGTAGCTTTACTATTACCCCTGACGCCACGAAAGCTAAGAAA

>704_Sample_ASV21

CACCGCGGTTAAACGAGAGGCCCTAGTTGATAGTACAACGGCGTAAAGGGTGGTTAAGGA

TAGTGAAATAATAAAGTCGAATGGCCCTTTGGCTGTCATACGCTTCTAGGAGTCCGAAGC

CCAATATACGAAAGTAACTTTAAGAGCGCCCACCTGACCCCACGAAAACTGAGAAA

>704_Sample_ASV22

CACCGCGGTTATACGAAAGACCCTAGTTGATAGCCACGGCGTAAAGGGTGGTTAAGGTAT

ACAACAAATAAAGCTAAATAGCCTCTAGGCCGTCGCACGCATTCCGAGAGCTCGAAACCC

AAAAACGAAAGTAGCTTTAAAATAAACAGACCTGACCCCACGAAAGCTAAGAAA

>704_Sample_ASV23

CACCGCGGTTATACGAGAGGCCCAAGTTGATACTCCACGGCGTAAAGAGTGGTTAGAAAA

GACCTACTACTAAAGCCGAACGCCTTCAAAGCTGTTATACGCATCCGAAGGTGAGAAGCC

CACCCACGAAAGTGGCTTTACAACCTTGACTCCACGAAAGCTATGACA

>704_Sample_ASV24

CACCGCGGTTATACGAGAGACCCCAGTTGATAAACACGGCGTAAAGAGTGGTTAAGGGCA

CAAAACAAATAAAGTCAAACAACCTCTTAGCTGTTATACGCATTATGAGGATATGAAGCC

CCCCTACGAAAGTAACTTTACCAACCCCTGACCCCACGAAAGCTAAGAAA

>704_Sample_ASV25

CACCGCGGTTATACGAGAGACCCTAGTTGATATATACGGCGTAAAGAGTGGTTAAGGGCA

CAAAACAAATAAAGTCAAACAACCTCTTAGCTGTTATACGCATTATGAGGATATGAAGCC

CCCCTACGAAAGTAACTTTACCAACCCCTGACCCCACGAAAGCTAAGAAA

>704_Sample_ASV26

CACCGCGGTTATACGAGAGACCCTAGTTGATATATACGGCGTAAAGAGTGGTTTGGAATA

ACCAAGTAATATAGCCAAAGACCTCCCAAGCCGTCACACGCACCCCGGAGGCACGAAGCC

CAAACACGAAAGTAGCTTTATTAACTTACCGACGCCACGAAAGCTAAGAAA

>704_Sample_ASV27

CACCGCGGTTATACGAAAGACCCTAGTTTATAGGCACGGCGTAAAGGGTGGTTAGGGGAT

AAACAAAATAAAGCCAAAGACCTTCTAAGTCGTCATACACTCATGAAGGCACGAAGCCCA

AATACGAAAGTAACTTTACAACTTAGACCCGACTCCACGAAAGCTAAGAAA

>704_Sample_ASV28

CACCGCGGTTATACGAAAGACCCTAGTTTATAGGCACGGCGTAAAGGGTGGTTAGGGGAT

AAACAAAATAAAGCCAAAGACCTTCTAAGTCGTCATACACTCATGAAGGCACGAAGCCCA

AATACGAAAGTAACTTTACAACTTAGACCCGACTCCACGAAAGCTGGGGGA

>704_Sample_ASV29

CACCGCGGTTATACGAGAGGCCCAAGTTGATAGACATCGGCGTAAAGAGTGGTTAAGGAA

GTCTCAAAACTAAAGCCGAATGCCTTCAAGACTGTTATACGTTTCCGAAGGTAAGAAGCC

CCACTACGAAAGTGGCTTTACTCCACCTGACTCCACGAAAGCTGTGAAA

>704_Sample_ASV30

CACCGCGGTTATACGAGAGGCCCAAGTTGACAAACTCCGGCGTAAAGCGTGGTTAAGGCA

CACTCAAAAACTAAAGCCGAATATCTTCAGAGCAGTCATACGCATCCGAAGACACGAAGC

CCCACCACGAAAGTGGCTTTATGACACCTGACCCCACGAAAGCTAGGACA

>704_Sample_ASV31

CACCGCGGTTATACGAAAGACCCTAGTTGATAGACCACGGCGTAAAGGGTGGTTAAGGAA

CACAAAAATAAAGCTAAAGATCCTCTAAGCTGTCATACGCTTCCCGAAGACATGAAACCC

AATCACGAAAGTAGCTTTAAACTGTCCTGACACCACGAAAGCCAAGAAA

>707_Sample_ASV1

CACCGCGGTTATACGAAAGACCCTAGTTGATGGGTGCGGCGTAAAGGGTGGTTAGGGACT

ACAAAAAATAGAGCTAAAGATCCTCTAGGTCGTCATACACATCACGAGGAAACGAAGCCC

AAATACGAAAGTAGCTCTAAATATAATCCTGACGCCACGAAAGCCAAGAAA

>707_Sample_ASV2

CACCGCGGTTATACGAAAGACCCTAGTTGCTAGCCACGGCGTAAAGGGTGGTTAAGGACA

ACAACAAATAAAGCTAAAGATCCCCTAAGCCGTCATACGCATTCCGGGGGCACGAAGCCC

TAACACGAAAGTAGCTTTAAAAAATATACCTGACCCCACGAAAGCTAAGAAA

>707_Sample_ASV3

CACCGCGGTTATACGAGAGACCCTAGTTGATTAAAGCGGCGTAAAGAGTGGTTATGGAAC

TATTCTTTTAAAGCAGAAAACCTCTCAAACTGTTATACGCACCCAGAGGTCTGAATCCCT

CACACGAAAGTGACTTTATTTATGCCTACCAGAACCCACGAAAGCTGTGAAA

>707_Sample_ASV4

CACCGCGGTTATACGAGAGACCCTAGTTGATTAAAGCGGCGTAAAGAGTGGTTATGGAAC

TATTCTTTTAAAGCAGAAAACCTCTCAAACTGTTATACGCACCCAGAGGTCTGAATCCCT

CACACGAAAGTGACTTTATTTATGCCTACCAGAACCCACGAAAGCTGGGACA

>707_Sample_ASV5

CACCGCGGTTATACGAGAGGCCCAAGTTGACAGACACCGGCGTAAAGAGTGGCTAGGGAA

AAATTACTACTAAAGCCGAACACCTTCAGAACTGTCATACGTTCCCGAAGATAAGAAGCC

CCACCACGAAAGTGGCTTTATATTACCCGACCCCACGAAAGCTGTGAAA

>707_Sample_ASV6

CACCGCGGTTAAACGAGAGACCCTAGTTGATCATCACGGCGTAAAGAGTGGTTAGGGGAT

TATCATAAATAAAGCCAAAGACCTCCCAAGCTGTCGCACGCATTCCGGAGGCGCGAAGCC

CACACACGAAAGTAGCTTTAACTATTGACCCTGATGCCACGAAAGCTAAGAAA

>707_Sample_ASV7

CGCCGCGGTTAAACGAGAGGCCCTAGTTGATAGAACAACGGCGTAAAGGGTGGTTAAGGA

TAGCGAAATAATAAAGTCGAATGGCCCTTTGGCTGTCATACGCTTCTGGGAGTCTGAAGC

CCAATATACGAAAGTAGCTTTAATACCGTCCACCTGACCCCACGAAAACTGAGAAA

>707_Sample_ASV8

CGCCGCGGTTAAACGAGAGGCCCTAGTTGATGGAACAACGGCGTAAAGGGTGGTTAAGGA

TAGTGAGACAATAAAGTCGAATGGCCCTTTGGCTGTCATACGCTTCTGGGAGCCTGAAGC

CCACTATACGAAAGTAGCTTTAACAATGTCCACCTGACCCCACGAAAACTGAGAAA

>707_Sample_ASV9

CGCCGCGGTTAAACGAGAGGCCCTAGTTGATGGAACAACGGCGTAAAGGGTGGTTAAGGA

TAGTGAGACAATAAAGTCGAATGGCCCTTTGGCTGTCATACGCTTCTGGGAGCCTGAAGC

CCACTATACGAAAGTAGCTTTAACAACGTCCACCTGACCCCACGAAAACTGAGAAA

>707_Sample_ASV10

CACCGCGGTTATACGAAAGACCCTAGTTGATAGATCACGGCGTAAAGGGTGGTTAGGGAA

TACAAAAATAAAGCTAAAAATCCTCTAAGCTGTCATACGCTTCCCGAAGACATGAAACCC

AACCACGAAAGTAGCTTTAAACTCTCCTGACACCACGAAAGCCAAGGAA

>707_Sample_ASV11

CACCGCGGTTATACGAGAGGCCCAAGTTGATAAGCCCGGCGTAAAGAGTGGTTATGGAGA

ATAAAACACTAAAGCTAAAGACCCCCTAGGCTGTTATACGCACCTGGCGGCTCGAACCAC

CTATACGAAAGTAGCTTTACCCCCCTTCCACCAGAATCCACGACAGCTGGGGCA

>707_Sample_ASV12

CACCGCGGTTATACGAGAGGCCCTAGTTGATAGCCACGGCGCAAAGAGTGGTTAAGGAGC

CCCACTAAATAAAGTCGAACACTTCCTAGGCCGTTATACGTTTTCTAGAAGCACGAAACC

CAATTACACGAAAGCAACTTTATACTAAACAACCTGACCCCACGAAAGCTAAGAAA

>707_Sample_ASV13

CACCGCGGTTATACGAGAGGCCCAAGTTGACAGACATCGGCGTAAAGAGTGGTTAAGGAA

ACCCCATTACTAAAGCCGAACGCCCTCAGAACTGTCATACGTTCCCGAAGGTAAGAAGCC

CCACTACGAAAGTGGCTTTAACCACACCCGACCCCACGAAAGCTGTGAAA

>707_Sample_ASV14

CACCGCGGTTAAACGAGAGGCCCTAGTTGATATTACAACGGCGTAAAGGGTGGTTAAGGA

AAGCAAGCAATAAAGCCGAATGGCCCTTTGGCTGTCATACGCTTCTAGGTGTCCGAAGCC

CAATATACGAAAGTAGCTTTAGAAAAGCCCACCTGACACCACGAAAGCTGAGGAA

>707_Sample_ASV15

CACCGCGGTTATACGAGAGGCCCTAGTTGATAAATACGGCGTAAAGAGTGGTTAGAGGAC

TCCCATAATAAAGCCAAAGACCCTCCGGGCCGTCACACGCATACGAGGGCACGAAGTTCA

CACACGAAAGTAGCTTTAAATTACCCGACGCCACGAAAGCTAAGAAA

>707_Sample_ASV16

CACCGCGGTTATACGAAAGACCCCAGTTTATAGACACGGCGTAAAGGGTGGTTATAGGAC

AAACTAACATAGAGCCAAAGACCCCCTAAGCCGTCATACGCTCACGAGGGCTCGAAGCCC

CTATACGAAAGTAGCTCTACCAAAAAATGCTCGACTCCACGAAAGCTGGGACA

>707_Sample_ASV17

CACCGCGGTTATACGAAAGACCCTAGTTTATAAGTACGGCGTAAAGGGTGGTTAGGGGAC

AAACAAAATAAAGCCAAAGACCTCCTAAGCCGTCATACGCTTACGGAGGCACGAAGCCCA

AACACGAAAGTAGCTTTACCGAACATGCCCGACTCCACGAAAGCTGGGAAA

>707_Sample_ASV18

CACCGCGGTTATACGAGAGACCCTAGTTGATAAATACGGCGTAAAGAGTGGTTATGGGAA

AAACAAATAAAGTCAAACAACCTCTTAGCTGTTATACGCATTATGAGAGCATGAAGCCCC

CTCACGAAAGTAACTTTAATACCTCCTGACCCCACGAAAGCTAAGAAA

>707_Sample_ASV19

CACCGCGGTTATACGAGAGGCTCAAGTTGATAAACCCCGGCGTAAAGAGTGGTTAAGGAA

AAATAGAAACTAAAGCCGAATGCTTTCAAGACTGTTATACGTTCCCGAGAGTAAGAAGAC

CAATTACGAAAGTGGCTTTACTTTACCTGACCCCACGAAAGCTACGACA

>707_Sample_ASV20

AACCGCGGTTATACGAGAGACCCTAATTGATAGCTCACGGCGTAAAGGGTGGTTTGGGGA

AACCTCAAAATAAAGCTAAAGACCTTCTAAGCCGTTACACGCATACCGAAGGCACGAGGC

CCTAACACGAAAGTAGCTTTACTATTACCCCTGACGCCACGAAAGCTAAGAAA

>707_Sample_ASV21

CACCGCGGTTATACGAAAGACCCTAGTTGATGGATCACGGCGTAAAGGGTGGTTAAGGGA

TATAAAAATAAAGCTAAAAATCCTCTTAGCTGTCATACGCTTTCCGAAGACATGAGGCCC

AATTACGAAAGTAGCTTTAAACCACCCTGACACCACGAAAGCCAAGAAA

>707_Sample_ASV22

CACCGCGGTTAAACGAGAGGCCCTAGTTGATAGTACAACGGCGTAAAGGGTGGTTAAGGA

TAGTGAAATAATAAAGTCGAATGGCCCTTTGGCTGTCATACGCTTCTAGGAGTCCGAAGC

CCAATATACGAAAGTAACTTTAAGAGCGCCCACCTGACCCCACGAAAACTGAGAAA

>707_Sample_ASV23

CACCGCGGTTATACGAGAGGCCCAAGTTGATACTCCACGGCGTAAAGAGTGGTTAGAAAA

GACCTACTACTAAAGCCGAACGCCTTCAAAGCTGTTATACGCATCCGAAGGTGAGAAGCC

CACCCACGAAAGTGGCTTTACAACCTTGACTCCACGAAAGCTATGACA

>707_Sample_ASV24

CACCGCGGTTATACGAGAGACCCCAGTTGATAAACACGGCGTAAAGAGTGGTTAAGGGCA

CAAAACAAATAAAGTCAAACAACCTCTTAGCTGTTATACGCATTATGAGGATATGAAGCC

CCCCTACGAAAGTAACTTTACCAACCCCTGACCCCACGAAAGCTAAGAAA

>707_Sample_ASV25

CACCGCGGTTATACGAGAGACCCTAGTTGATATATACGGCGTAAAGAGTGGTTTGGAATA

ACCAAGTAATATAGCCAAAGACCTCCCAAGCCGTCACACGCACCCCGGAGGCACGAAGCC

CAAACACGAAAGTAGCTTTATTAACTTACCGACGCCACGAAAGCTAAGAAA

>707_Sample_ASV26

CACCGCGGTTATACGAAAGACCCTAGTTTATAGGCACGGCGTAAAGGGTGGTTAGGGGAT

AAACAAAATAAAGCCAAAGACCTTCTAAGTCGTCATACACTCATGAAGGCACGAAGCCCA

AATACGAAAGTAACTTTACAACTTAGACCCGACTCCACGAAAGCTGGGGGA

>707_Sample_ASV27

CACCGCGGTTATACGAGAGGCCCAAGTTGATAGACATCGGCGTAAAGAGTGGTTAAGGAA

GTCTCAAAACTAAAGCCGAATGCCTTCAAGACTGTTATACGTTTCCGAAGGTAAGAAGCC

CCACTACGAAAGTGGCTTTACTCCACCTGACTCCACGAAAGCTGTGAAA

>707_Sample_ASV28

CACCGCGGTTATACGAAAGACCCTAGTTGATAGACCACGGCGTAAAGGGTGGTTAAGGAA

CACAAAAATAAAGCTAAAGATCCTCTAAGCTGTCATACGCTTCCCGAAGACATGAAACCC

AATCACGAAAGTAGCTTTAAACTGTCCTGACACCACGAAAGCCAAGAAA

>709_Sample_ASV1

CACCGCGGTTATACGAAAGACCCTAGTTGATGGGTGCGGCGTAAAGGGTGGTTAGGGACT

ACAAAAAATAGAGCTAAAGATCCTCTAGGTCGTCATACACATCACGAGGAAACGAAGCCC

AAATACGAAAGTAGCTCTAAATATAATCCTGACGCCACGAAAGCCAAGAAA

>709_Sample_ASV2

CACCGCGGTTATACGAGAGACCCTAGTTGATTAAAGCGGCGTAAAGAGTGGTTATGGAAC

TATTCTTTTAAAGCAGAAAACCTCTCAAACTGTTATACGCACCCAGAGGTCTGAATCCCT

CACACGAAAGTGACTTTATTTATGCCTACCAGAACCCACGAAAGCTGGGACA

>709_Sample_ASV3

CACCGCGGTTATACGAAAGACCCTAGTTTATAGGTACGGCGTAAAGGGTGGTTAGGGGAC

AAACAAAATAAAGCCAAAGACCTCCTAAGCCGTCATACGCTCACGGAAGCACGAAGCCCA

AACACGAAAGTAGCTTTACCGAACATGCCCGACTCCACGAAAGCTGGGGAA

>709_Sample_ASV4

CACCGCGGTTATACGAAAGACCCTAGTTGATAGCCACGGCGTAAAGGGTGGTTAAGGAAC

CTAAAAAATAAAGCTAAAGACCCTCTAGGCCGTCATACGCATTACGAGGCAAACGAAACC

CCAACACGAAAGTAGCTTTAGACAAATATCCTGATGCCACGAAAGCCAAGAAA

>709_Sample_ASV5

CACCGCGGTTATACGAGAGGCCCAAGTTGACAGACACCGGCGTAAAGAGTGGCTAGGGAA

AAATTACTACTAAAGCCGAACACCTTCAGAACTGTCATACGTTCCCGAAGATAAGAAGCC

CCACCACGAAAGTGGCTTTATATTACCCGACCCCACGAAAGCTGTGAAA

>709_Sample_ASV6

CACCGCGGTTATACGAGAGGCCCAAGTTGACAGACACCGGCGTAAAGAGTGGCTAAGGAA

AAATTTCTACTAAAGCCGAACACCTTCAGAACTGTCATACGTTCCCGAAGAAAAGAAGCC

CCACCACGAAAGTGGCTTTATATTACCCGACCCCACGAAAGCTGTGAAA

>709_Sample_ASV7

CACCGCGGTTATACGAGAGGCCCAAGTTGATAAGCCCGGCGTAAAGAGTGGTTATGGAGA

ATAAAACACTAAAGCTAAAGACCCCCTAGGCTGTTATACGCACCTGGCGGCTCGAACCAC

CTATACGAAAGTAGCTTTACCCCCCTTCCACCAGAATCCACGACAGCTGGGGCA

>709_Sample_ASV8

CACCGCGGTTATACGAGAGGCCCAAGTTGACAGACATCGGCGTAAAGAGTGGTTAAGGAA

ACCCCATTACTAAAGCCGAACGCCCTCAGAACTGTCATACGTTCCCGAAGGTAAGAAGCC

CCACTACGAAAGTGGCTTTAACCACACCCGACCCCACGAAAGCTGTGAAA

>709_Sample_ASV9

CACCGCGGTTATACGAGAGACCCCAGTTGATAGCTACGGCGTAAAGAGTGGTTAAGGACC

CCATTTAATAAAGCCAAAGACCCCCCAAGCCGTCATACGCACACGAGGGCGCGAAGCTCG

CACACGAAAGTAGCTCTAATTTACCCGACGCCACGAAAGCTAAGGAA

>709_Sample_ASV10

CACCGCGGTTATACGAGAGACCCTAGTTGATAAATACGGCGTAAAGAGTGGTTATGGGAA

AAACAAATAAAGTCAAACAACCTCTTAGCTGTTATACGCATTATGAGAGCATGAAGCCCC

CTCACGAAAGTAACTTTAATACCTCCTGACCCCACGAAAGCTAAGAAA

>709_Sample_ASV11

CACCGCGGTTATACGAGAGACCCTAATTGATAGACACGGCGTAAAGAGTGGTTTGGGAAA

GCCCAAAAATAAAGCTAAAGACCTTCTAAGCCGTCACACGCACGCCGAAGGCACGAAGCC

CTGACACGAAAGTAGCTTTACTATTACCCCCGACGCCACGAAAGCTAAGAAA

>709_Sample_ASV12

CACCGCGGTTATACGAAAGACCCTAGTTTATAGATGCGGCGTAAAGGGTGGTTAAGGAAA

ACCTAAAATAATGCTAAAGACTCTCAAAGCTGTTATACGCTTCATGAGCACACGAAATCC

AAATACGAAAGTTGCCTTAATAAACTTTGCCTGACCCCACGAAAGCTGAAAAA

>709_Sample_ASV13

CACCGCGGTTATACGAGAGGCCCAAGTTGACAGGTACCGGCGTAAAGAGTGGTTAAGAAA

AAATATTAAACTAAAGCCGAACACCTTCAGAGCTGTCATACGTTTCCGAAGGCATGAAGC

CCCACCACGAAAGTGGCTTTATTTTGCCCGACTCCACGAAAGCTGTGAAA

>709_Sample_ASV14

CACCGCGGTTATACGAGAGACCCTAGTTGATAGCTACGGCGTAAAGCGTGGTTAGGAGAA

CAAGATAAATAAAGTCAAACAATCTCTCGGCCGTTATACGTTATCTGAGAATATGAAGTC

CTACCACGAAAGTAACTTTAATTTTTCTGACCCCACGAAAACTGAGGAA

>709_Sample_ASV15

CACCGCGGTTAAACGAGAGGCCCTAGTTGATAGTACAACGGCGTAAAGGGTGGTTAAGGA

TAGTGAAATAATAAAGTCGAATGGCCCTTTGGCTGTCATACGCTTCTAGGAGTCCGAAGC

CCAATATACGAAAGTAACTTTAAGAGCGCCCACCTGACCCCACGAAAACTGAGAAA

>709_Sample_ASV16

CACCGCGGTTATACGAGAGGCCCTAGTTGATAACTACCGGCGTAAAGAGTGGTTATGGAA

AAATATTTAATAAAGCCGAACACCCCCTCAGCCGTCATACGCACCTGGGAGCACGAAGAC

CTACTGCGAAAGCAGCTTTAATTATGCCTGACCCCACGACAGCTAAGAAA

>709_Sample_ASV17

CACCGCGGTTATACGAAAGACCCTAGTTGATAGGCACGGCGTAAAGGGTGGTTAGGGATA

TATTAAAAATAAAGTTAAAGAGCCTCTAAGCTGTCGCACGCATCCCGAGAGCTCGAGACC

CAGACACGAAAGTAACTTTAAAATACCTCACCTGACCCCACGAAAGCTAAGAAA

>709_Sample_ASV18

CACCGCGGTTATACGAGAGACCCCAGTTGATAAACACGGCGTAAAGAGTGGTTAAGGGCA

CAAAACAAATAAAGTCAAACAACCTCTTAGCTGTTATACGCATTATGAGGATATGAAGCC

CCCCTACGAAAGTAACTTTACCAACCCCTGACCCCACGAAAGCTAAGAAA

>709_Sample_ASV19

CACCGCGGTTATACGAGAGACCCTAGTTGATATATACGGCGTAAAGAGTGGTTTGGAATA

ACCAAGTAATATAGCCAAAGACCTCCCAAGCCGTCACACGCACCCCGGAGGCACGAAGCC

CAAACACGAAAGTAGCTTTATTAACTTACCGACGCCACGAAAGCTAAGAAA

>709_Sample_ASV20

CACCGCGGTTATACGAAAGACCCTAGTTTATAGGCACGGCGTAAAGGGTGGTTAGGGGAT

AAACAAAATAAAGCCAAAGACCTTCTAAGTCGTCATACACTCATGAAGGCACGAAGCCCA

AATACGAAAGTAACTTTACAACTTAGACCCGACTCCACGAAAGCTGGGGGA

>709_Sample_ASV21

CACCGCGGTTATACGAGAGGCCCAAGTTGATAGACATCGGCGTAAAGAGTGGTTAAGGAA

GTCTCAAAACTAAAGCCGAATGCCTTCAAGACTGTTATACGTTTCCGAAGGTAAGAAGCC

CCACTACGAAAGTGGCTTTACTCCACCTGACTCCACGAAAGCTGTGAAA

>709_Sample_ASV22

CACCGCGGTTATACGAGAGACCCTAATTGATAGACTACGGCGTAAAGAGTGGTTTAGAAC

AAAAATTAATAAAGCCAAAGATCCTCCTGGCCGTCGCACGCATTTCGAGGATACGAAGCC

CCACTACGAAAGTAGCTTTACTACAACTTTTCTGACCCCACGAAAGCTAAGAAA

>709_Sample_ASV23

CACCGCGGTTATACGAAAGACCCTAGTTGATAGATCACGGCGTAAAGGGTGGTTAAGGAG

AACAAAAATAAAGCTAAAAATCCTCTAAGCTGTCATACGCTTTCCGAAGACATGAGACCC

AACCACGAAAGTAACTTTAAACTTTCCTGACACCACGAAAGCCAAGAAA

>710_Sample_ASV1

CACCGCGGTTATACGAAAGACCCTAGTTGATGGGTGCGGCGTAAAGGGTGGTTAGGGACT

ACAAAAAATAGAGCTAAAGATCCTCTAGGTCGTCATACACATCACGAGGAAACGAAGCCC

AAATACGAAAGTAGCTCTAAATATAATCCTGACGCCACGAAAGCCAAGAAA

>710_Sample_ASV2

CACCGCGGTTATACGAGAGACCCTAGTTGATTAAAGCGGCGTAAAGAGTGGTTATGGAAC

TATTCTTTTAAAGCAGAAAACCTCTCAAACTGTTATACGCACCCAGAGGTCTGAATCCCT

CACACGAAAGTGACTTTATTTATGCCTACCAGAACCCACGAAAGCTGGGACA

>710_Sample_ASV3

CACCGCGGTTATACGAGAGGCCCAAGTTGACAGACACCGGCGTAAAGAGTGGCTAGGGAA

AAATTACTACTAAAGCCGAACACCTTCAGAACTGTCATACGTTCCCGAAGATAAGAAGCC

CCACCACGAAAGTGGCTTTATATTACCCGACCCCACGAAAGCTGTGAAA

>710_Sample_ASV4

CACCGCGGTTATACGAGAGGCCCAAGTTGACAGACACCGGCGTAAAGAGTGGCTAGGGAA

AAATTACTACTAAAGCCGAACACCTTCAGAACTGTCATACGTTCCCGAAGATAAGAAGCC

CCACCACGAAAGTGGCTTTATATTACCCGACCCCACGAAAGCCAAGAAA

>710_Sample_ASV5

CACCGCGGTTATACGAGAGGCCCAAGTTGACAGACACCGGCGTAAAGAGTGGCTAAGGAA

AAATTTCTACTAAAGCCGAACACCTTCAGAACTGTCATACGTTCCCGAAGAAAAGAAGCC

CCACCACGAAAGTGGCTTTATATTACCCGACCCCACGAAAGCTGTGAAA

>710_Sample_ASV6

CACCGCGGTTAAACGAGAGACCCTAGTTGATCATCACGGCGTAAAGAGTGGTTAGGGGAT

TATCATAAATAAAGCCAAAGACCTCCCAAGCTGTCGCACGCATTCCGGAGGCGCGAAGCC

CACACACGAAAGTAGCTTTAACTATTGACCCTGATGCCACGAAAGCTAAGAAA

>710_Sample_ASV7

CGCCGCGGTTAAACGAGAGGCCCTAGTTGATGGAACAACGGCGTAAAGGGTGGTTAAGGA

TAGTGAGACAATAAAGTCGAATGGCCCTTTGGCTGTCATACGCTTCTGGGAGCCTGAAGC

CCACTATACGAAAGTAGCTTTAACAATGTCCACCTGACCCCACGAAAACTGAGAAA

>710_Sample_ASV8

CACCGCGGTTATACGAAAGACCCTAGTTGATAGATCACGGCGTAAAGGGTGGTTAGGGAA

TACAAAAATAAAGCTAAAAATCCTCTAAGCTGTCATACGCTTCCCGAAGACATGAAACCC

AACCACGAAAGTAGCTTTAAACTCTCCTGACACCACGAAAGCCAAGGAA

>710_Sample_ASV9

CACCGCGGTTATACGAGAGGCCCAAGTTGATAAGCCCGGCGTAAAGAGTGGTTATGGAGA

ATAAAACACTAAAGCTAAAGACCCCCTAGGCTGTTATACGCACCTGGCGGCTCGAACCAC

CTATACGAAAGTAGCTTTACCCCCCTTCCACCAGAATCCACGACAGCTGGGGCA

>710_Sample_ASV10

CACCGCGGTTATACGAGAGGCCCAAGTTGACAGACATCGGCGTAAAGAGTGGTTAAGGAA

ACCCCATTACTAAAGCCGAACGCCCTCAGAACTGTCATACGTTCCCGAAGGTAAGAAGCC

CCACTACGAAAGTGGCTTTAACCACACCCGACCCCACGAAAGCTGTGAAA

>710_Sample_ASV11

CACCGCGGTTAAACGAGAGGCCCTAGTTGATATTACAACGGCGTAAAGGGTGGTTAAGGA

AAGCAAGCAATAAAGCCGAATGGCCCTTTGGCTGTCATACGCTTCTAGGTGTCCGAAGCC

CAATATACGAAAGTAGCTTTAGAAAAGCCCACCTGACACCACGAAAGCTGAGGAA

>710_Sample_ASV12

CACCGCGGTTATACGAGAGGCCCTAGTTGATAAATACGGCGTAAAGAGTGGTTAGAGGAC

TCCCATAATAAAGCCAAAGACCCTCCGGGCCGTCACACGCATACGAGGGCACGAAGTTCA

CACACGAAAGTAGCTTTAAATTACCCGACGCCACGAAAGCTAAGAAA

>710_Sample_ASV13

CACCGCGGTTATACGAAAGACCCCAGTTTATAGACACGGCGTAAAGGGTGGTTATAGGAC

AAACTAACATAGAGCCAAAGACCCCCTAAGCCGTCATACGCTCACGAGGGCTCGAAGCCC

CTATACGAAAGTAGCTCTACCAAAAAATGCTCGACTCCACGAAAGCTGGGACA

>710_Sample_ASV14

CACCGCGGTTATACGAAAGACCCTAGTTTATAAGTACGGCGTAAAGGGTGGTTAGGGGAC

AAACAAAATAAAGCCAAAGACCTCCTAAGCCGTCATACGCTTACGGAGGCACGAAGCCCA

AACACGAAAGTAGCTTTACCGAACATGCCCGACTCCACGAAAGCTGGGAAA

>710_Sample_ASV15

CACCGCGGTTATACGAGAGACCCTAGTTGATAAATACGGCGTAAAGAGTGGTTATGGGAA

AAACAAATAAAGTCAAACAACCTCTTAGCTGTTATACGCATTATGAGAGCATGAAGCCCC

CTCACGAAAGTAACTTTAATACCTCCTGACCCCACGAAAGCTAAGAAA

>710_Sample_ASV16

AACCGCGGTTATACGAGAGACCCTAATTGATAGCTCACGGCGTAAAGGGTGGTTTGGGGA

AACCTCAAAATAAAGCTAAAGACCTTCTAAGCCGTTACACGCATACCGAAGGCACGAGGC

CCTAACACGAAAGTAGCTTTACTATTACCCCTGACGCCACGAAAGCTAAGAAA

>710_Sample_ASV17

CACCGCGGTTATACGAAAGACCCTAGTTGATGGATCACGGCGTAAAGGGTGGTTAAGGGA

TATAAAAATAAAGCTAAAAATCCTCTTAGCTGTCATACGCTTTCCGAAGACATGAGGCCC

AATTACGAAAGTAGCTTTAAACCACCCTGACACCACGAAAGCCAAGAAA

>710_Sample_ASV18

CACCGCGGTTATACGAGAGACCCTAGTTGATAGCTACGGCGTAAAGCGTGGTTAGGAGAA

CAAGATAAATAAAGTCAAACAATCTCTCGGCCGTTATACGTTATCTGAGAATATGAAGTC

CTACCACGAAAGTAACTTTAATTTTTCTGACCCCACGAAAACTGAGGAA

>710_Sample_ASV19

CACCGCGGTTAAACGAGAGGCCCTAGTTGATAGTACAACGGCGTAAAGGGTGGTTAAGGA

TAGTGAAATAATAAAGTCGAATGGCCCTTTGGCTGTCATACGCTTCTAGGAGTCCGAAGC

CCAATATACGAAAGTAACTTTAAGAGCGCCCACCTGACCCCACGAAAACTGAGAAA

>710_Sample_ASV20

CACCGCGGTTATACGAGAGGCCCAAGTTGATACTCCACGGCGTAAAGAGTGGTTAGAAAA

GACCTACTACTAAAGCCGAACGCCTTCAAAGCTGTTATACGCATCCGAAGGTGAGAAGCC

CACCCACGAAAGTGGCTTTACAACCTTGACTCCACGAAAGCTATGACA

>710_Sample_ASV21

CACCGCGGTTATACGAGAGACCCCAGTTGATAAACACGGCGTAAAGAGTGGTTAAGGGCA

CAAAACAAATAAAGTCAAACAACCTCTTAGCTGTTATACGCATTATGAGGATATGAAGCC

CCCCTACGAAAGTAACTTTACCAACCCCTGACCCCACGAAAGCTAAGAAA

>710_Sample_ASV22

CACCGCGGTTATACGAGAGACCCTAGTTGATATATACGGCGTAAAGAGTGGTTTGGAATA

ACCAAGTAATATAGCCAAAGACCTCCCAAGCCGTCACACGCACCCCGGAGGCACGAAGCC

CAAACACGAAAGTAGCTTTATTAACTTACCGACGCCACGAAAGCTAAGAAA

>710_Sample_ASV23

CACCGCGGTTATACGAAAGACCCTAGTTTATAGGCACGGCGTAAAGGGTGGTTAGGGGAT

AAACAAAATAAAGCCAAAGACCTTCTAAGTCGTCATACACTCATGAAGGCACGAAGCCCA

AATACGAAAGTAACTTTACAACTTAGACCCGACTCCACGAAAGCTGTGATA

>710_Sample_ASV24

CACCGCGGTTATACGAAAGACCCTAGTTTATAGGCACGGCGTAAAGGGTGGTTAGGGGAT

AAACAAAATAAAGCCAAAGACCTTCTAAGTCGTCATACACTCATGAAGGCACGAAGCCCA

AATACGAAAGTAACTTTACAACTTAGACCCGACTCCACGAAAGCTGGGGGA

>710_Sample_ASV25

CACCGCGGTTATACGAGAGACCCTAATTAATAGATACGGCGTAAAGAGTGGTTTAGGACA

AAAATTAATAAAGCCGAAGTTTCTCCTAGCCGTTGTACGCACTCTGAGAACACGAAGCCC

CACTACGAAAGTAGCTTTACCTATTATTTCCTGACCCCACGAAAGCTAAGAAA

>710_Sample_ASV26

CACCGCGGTTATACGAGAGGCCCAAGTTGATAGACATCGGCGTAAAGAGTGGTTAAGGAA

GTCTCAAAACTAAAGCCGAATGCCTTCAAGACTGTTATACGTTTCCGAAGGTAAGAAGCC

CCACTACGAAAGTGGCTTTACTCCACCTGACTCCACGAAAGCTGTGAAA

>710_Sample_ASV27

CACCGCGGTTATACGAAAGACCCTAGTTGATAGACCACGGCGTAAAGGGTGGTTAAGGAA

CACAAAAATAAAGCTAAAGATCCTCTAAGCTGTCATACGCTTCCCGAAGACATGAAACCC

AATCACGAAAGTAGCTTTAAACTGTCCTGACACCACGAAAGCCAAGAAA

>715_Sample_ASV1

CACCGCGGTTATACGAGAGGCCCAAGTTGACAGACATCGGCGTAAAGAGTGGTTAAGGAA

AACCTTCAACTAAAGTCGAACGCTCTCAAGACTGTCGTACGTTTCCGAGAGTATGAAGCC

CCACCACGAAAGTGACTTTACCTCACCTGACTCCACGAAAGCTGCGAAA

>715_Sample_ASV2

CACCGCGGTTATACGAAAGACCCTAGTTGATGGGTGCGGCGTAAAGGGTGGTTAGGGACT

ACAAAAAATAGAGCTAAAGATCCTCTAGGTCGTCATACACATCACGAGGAAACGAAGCCC

AAATACGAAAGTAGCTCTAAATATAATCCTGACGCCACGAAAGCCAAGAAA

>715_Sample_ASV3

CACCGCGGTTATACGAAAGACCCTAGTTGATAACTACGGCATAAAGGGTGGTTAGGGAGT

TTAAAAAATAAAGCCAAAGCTCTTCTAAGCTGTCATACGCTCTCTGAAAGAACGAGGCCC

AAACACGAAAGTAGCTTTAAATAAATAACCTGATACCACGAAAGCCAAGAAA

>715_Sample_ASV4

CACCGCGGTTATACGAGAGACCCTAGTTGATTAAAGCGGCGTAAAGAGTGGTTATGGAAC

TATTCTTTTAAAGCAGAAAACCTCTCAAACTGTTATACGCACCCAGAGGTCTGAATCCCT

CACACGAAAGTGACTTTATTTATGCCTACCAGAACCCACGAAAGCTGGGACA

>715_Sample_ASV5

CACCGCGGTTATACGAGAGACCCTAGTTGATTAAAGCGGCGTAAAGAGTGGTTATGGAAC

TATTCTTTTAAAGCAGAAAACCTCTCAAACTGTTATACGCACCCAGAGGTCTGAATCCCT

CACACGAAAGTGACTTTATTTATGCCTACCAGAACCCACGAAAGCTATGACA

>715_Sample_ASV6

CACCGCGGTTATACGAGAGGCCCAAGTTGACAGACACCGGCGTAAAGAGTGGCTAGGGAA

AAATTACTACTAAAGCCGAACACCTTCAGAACTGTCATACGTTCCCGAAGATAAGAAGCC

CCACCACGAAAGTGGCTTTATATTACCCGACCCCACGAAAGCTGTGAAA

>715_Sample_ASV7

CACCGCGGTTATACGAGAGGCCCAAGTTGACAGACACCGGCGTAAAGAGTGGCTAGGGAA

AAATTACTACTAAAGCCGAACACCTTCAGAACTGTCATACGTTCCCGAAGATAAGAAGCC

CCACCACGAAAGTGGCTTTATATTACCCGACCCCACGAAAGCTAAGAAA

>715_Sample_ASV8

CACCGCGGTTATACGAAAGACCCTAGTTTATAGAAACGGCGTAAAGGGTGGTTAAGGGAC

AAACAAAATAAGGCCAAAGACTTCCTAAGCCGTCATACGCTCCACGAAAGCACGAAGCCC

AAACACGAAAGTAGCCTTAATAACTTCACCTGACCCCACGAAAGCTGGGAGA

>715_Sample_ASV9

CACCGCGGTTATACGAGAGGCCCAAGTTGATAAGCCCGGCGTAAAGAGTGGTTATGGAGA

ATAAAACACTAAAGCTAAAGACCCCCTAGGCTGTTATACGCACCTGGCGGCTCGAACCAC

CTATACGAAAGTAGCTTTACCCCCCTTCCACCAGAATCCACGACAGCTGGGGCA

>715_Sample_ASV10

CACCGCGGTTATACGAGAGGCCCTAGTTGATAGCCACGGCGCAAAGAGTGGTTAAGGAGC

CCCACTAAATAAAGTCGAACACTTCCTAGGCCGTTATACGTTTTCTAGAAGCACGAAACC

CAATTACACGAAAGCAACTTTATACTAAACAACCTGACCCCACGAAAGCTAAGAAA

>715_Sample_ASV11

CACCGCGGTTATACGAGAGGCCCAAGTTGACAGACATCGGCGTAAAGAGTGGTTAAGGAA

ACCCCATTACTAAAGCCGAACGCCCTCAGAACTGTCATACGTTCCCGAAGGTAAGAAGCC

CCACTACGAAAGTGGCTTTAACCACACCCGACCCCACGAAAGCTGTGAAA

>715_Sample_ASV12

CACCGCGGTTATACGAGAGGCCCAAGTTGACAGACACCGGCGTAAAGAGTGGTTAAGGAA

ATTCATCAACTAAAGCCGAATGCCCTCAGGACTGTTATACGTTTGCGAAGGTAAGAAGCC

CTACTACGAAAGTGGCTTTATTCCACCTGATTCCACGAAAGCTAAGAAA

>715_Sample_ASV13

CACCGCGGTTATACGAGAGGCCCAAGTTGACAGACACCGGCGTAAAGAGTGGTTAAGGAA

ATTCATCAACTAAAGCCGAATGCCCTCAGGACTGTTATACGTTTGCGAAGGTAAGAAGCC

CTACTACGAAAGTGGCTTTATTCCACCTGATTCCACGAAAGCTGCGGAA

>715_Sample_ASV14

CACCGCGGTTATACGAGAGGCCCCAGTTGATATTAGCGGCGTAAAGGGTGGTTAAGGACT

TTAATAACTAAAGCTAAACACTTCCCCGGCTGTTGTACGTAGACCGGAAGCTTGAAGCCC

AGACACGAAAGTAGCTTTATTCCCCCTGACCCCACGAAAGCTGAGAAA

>715_Sample_ASV15

CACCGCGGTTATACGAGAAACCCTAGTTGACAAATACGGCGTAAAGAGTGGTTAAGGGGT

ATCAAAAATAAAGTCAAACATTTTCCTAGTTGTTATAAGCATTTCGAGAACATGAAGCCC

AACCACGAAAGTAACTTTACCTTCACCTGACCCCACGAAAGCTAAGAAA

>715_Sample_ASV16

CACCGCGGTTATACGAGAGACCCTAATTGATAGGTACGGCGTAAAGAGTGGTTTGGGATA

ACCCAAAAATAAAGCTAAAGACCTTCTAAGCCGTCACACGCATACCGAAGGCACGAGGCC

CCAACACGAAAGTAGCTTTACTATTACCCCGACGCCACGAAAGCTAAGAAA

>715_Sample_ASV17

CACCGCGGTTATACGAGAGGCCCTAGTTGATAACTACCGGCGTAAAGAGTGGTTATGGAA

AAATATTTAATAAAGCCGAACACCCCCTCAGCCGTCATACGCACCTGGGAGCACGAAGAC

CTACTGCGAAAGCAGCTTTAATTATGCCTGACCCCACGACAGCTAAGAAA

>715_Sample_ASV18

CACCGCGGTTATACGAGAGGCCCAAGTTGATACTCCACGGCGTAAAGAGTGGTTAGAAAA

GACCTACTACTAAAGCCGAACGCCTTCAAAGCTGTTATACGCATCCGAAGGTGAGAAGCC

CACCCACGAAAGTGGCTTTACAACCTTGACTCCACGAAAGCTATGACA

>715_Sample_ASV19

CACCGCGGTTATACGAGAGACCCCAGTTGATAAACACGGCGTAAAGAGTGGTTAAGGGCA

CAAAACAAATAAAGTCAAACAACCTCTTAGCTGTTATACGCATTATGAGGATATGAAGCC

CCCCTACGAAAGTAACTTTACCAACCCCTGACCCCACGAAAGCTAAGAAA

>715_Sample_ASV20

CACCGCGGTTATACGAGAGACTCCAGTTGATAGCTACGGCGTAAAGAGTGGTTTGGGGCC

CGCCCAAAATAAAGCCAAAGACCTCCCAAGCCGTCAAACGCACCCCGGAGGCACGAAGTC

CTAACGCGAAAGCAACTTTACCTTCCCCGACGCCACGAAAGCTAAGAAA

>715_Sample_ASV21

CACCGCGGTTATACGAAAGACCCTAGTTGATAGATCACGGCGTAAAGGGTGGTTAAGGAG

AACAAAAATAAAGCTAAAAATCCTCTAAGCTGTCATACGCTTTCCGAAGACATGAGACCC

AACCACGAAAGTAACTTTAAACTTTCCTGACACCACGAAAGCCAAGAAA

>720_Sample_ASV1

CACCGCGGTTATACGAGAGGCCCAAGTTGACAGACATCGGCGTAAAGAGTGGTTAAGGAA

AACCTTCAACTAAAGTCGAACGCTCTCAAGACTGTCGTACGTTTCCGAGAGTATGAAGCC

CCACCACGAAAGTGACTTTACCTCACCTGACTCCACGAAAGCTGCGAAA

>720_Sample_ASV2

CACCGCGGTTATACGAAAGACCCTAGTTGATGGGTGCGGCGTAAAGGGTGGTTAGGGACT

ACAAAAAATAGAGCTAAAGATCCTCTAGGTCGTCATACACATCACGAGGAAACGAAGCCC

AAATACGAAAGTAGCTCTAAATATAATCCTGACGCCACGAAAGCCAAGAAA

>720_Sample_ASV3

CACCGCGGTTATACGAAAGACCCTAGTTGATAACTACGGCATAAAGGGTGGTTAGGGAGT

TTAAAAAATAAAGCCAAAGCTCTTCTAAGCTGTCATACGCTCTCTGAAAGAACGAGGCCC

AAACACGAAAGTAGCTTTAAATAAATAACCTGATACCACGAAAGCCAAGAAA

>720_Sample_ASV4

CACCGCGGTTATACGAGAGACCCTAGTTGATTAAAGCGGCGTAAAGAGTGGTTATGGAAC

TATTCTTTTAAAGCAGAAAACCTCTCAAACTGTTATACGCACCCAGAGGTCTGAATCCCT

CACACGAAAGTGACTTTATTTATGCCTACCAGAACCCACGAAAGCTGGGACA

>720_Sample_ASV5

CACCGCGGTTATACGAAAGACCCTAGTTTATAGGTACGGCGTAAAGGGTGGTTAGGGGAC

AAACAAAATAAAGCCAAAGACCTCCTAAGCCGTCATACGCTCACGGAAGCACGAAGCCCA

AACACGAAAGTAGCTTTACCGAACATGCCCGACTCCACGAAAGCTGGGGAA

>720_Sample_ASV6

CACCGCGGTTATACGAGAGGCCCAAGTTGACAGACACCGGCGTAAAGAGTGGCTAGGGAA

AAATTACTACTAAAGCCGAACACCTTCAGAACTGTCATACGTTCCCGAAGATAAGAAGCC

CCACCACGAAAGTGGCTTTATATTACCCGACCCCACGAAAGCTGTGAAA

>720_Sample_ASV7

CACCGCGGTTATACGAAAGACTCTAGTTGACAAACACGGCGTAAAGGGTGGTTAGGATAA

CAACAAAATAAAGCTAAAGACTTACCAAGCCGTCATACGCCCATGTAAGAGCGAAAACCA

CAAACGAAAGTAACTTTATTACACCATCCGAATCCACGAAAGCTAAGAAA

>720_Sample_ASV8

CACCGCGGTTATACGAAAGACCCTAGTTTATAGAAACGGCGTAAAGGGTGGTTAAGGGAC

AAACAAAATAAGGCCAAAGACTTCCTAAGCCGTCATACGCTCCACGAAAGCACGAAGCCC

AAACACGAAAGTAGCCTTAATAACTTCACCTGACCCCACGAAAGCTGGGAGA

>720_Sample_ASV9

CACCGCGGTTATACGAGAGGCCCAAGTTGATAAGCCCGGCGTAAAGAGTGGTTATGGAGA

ATAAAACACTAAAGCTAAAGACCCCCTAGGCTGTTATACGCACCTGGCGGCTCGAACCAC

CTATACGAAAGTAGCTTTACCCCCCTTCCACCAGAATCCACGACAGCTGGGGCA

>720_Sample_ASV10

CACCGCGGTTATACGAGAGGCCCTAGTTGATAGCCACGGCGCAAAGAGTGGTTAAGGAGC

CCCACTAAATAAAGTCGAACACTTCCTAGGCCGTTATACGTTTTCTAGAAGCACGAAACC

CAATTACACGAAAGCAACTTTATACTAAACAACCTGACCCCACGAAAGCTAAGAAA

>720_Sample_ASV11

CACCGCGGTTATACGAGAGGCCCAAGTTGACAGACATCGGCGTAAAGAGTGGTTAAGGAA

ACCCCATTACTAAAGCCGAACGCCCTCAGAACTGTCATACGTTCCCGAAGGTAAGAAGCC

CCACTACGAAAGTGGCTTTAACCACACCCGACCCCACGAAAGCTGTGAAA

>720_Sample_ASV12

CACCGCGGTTATACGAGAGGCCCAAGTTGATAGATATCGGCGTAAAGAGTGGTTAAGGAA

ACCCCGTTACTAAAGCCGAACGCCCTCAGAACTGTCATACGTTCCCGAAGGTAAGAAGCC

CCACTACGAAAGTGGCTTTAACCACACCCGACCCCACGAAAGCTGTGAAA

>720_Sample_ASV13

CACCGCGGTTATACGAGAGGCCCAAGTTGACAGACACCGGCGTAAAGAGTGGTTAAGGAA

ATTCATCAACTAAAGCCGAATGCCCTCAGGACTGTTATACGTTTGCGAAGGTAAGAAGCC

CTACTACGAAAGTGGCTTTATTCCACCTGATTCCACGAAAGCTGCGGAA

>720_Sample_ASV14

CACCGCGGTTATACGAGAGGCCCCAGTTGATATTAGCGGCGTAAAGGGTGGTTAAGGACT

TTAATAACTAAAGCTAAACACTTCCCCGGCTGTTGTACGTAGACCGGAAGCTTGAAGCCC

AGACACGAAAGTAGCTTTATTCCCCCTGACCCCACGAAAGCTGAGAAA

>720_Sample_ASV15

CACCGCGGTTATACGAGAAACCCTAGTTGACAAATACGGCGTAAAGAGTGGTTAAGGGGT

ATCAAAAATAAAGTCAAACATTTTCCTAGTTGTTATAAGCATTTCGAGAACATGAAGCCC

AACCACGAAAGTAACTTTACCTTCACCTGACCCCACGAAAGCTAAGAAA

>720_Sample_ASV16

CACCGCGGTTATACGAGAGACCCTAGTTGATAAATACGGCGTAAAGAGTGGTTATGGGAA

AAACAAATAAAGTCAAACAACCTCTTAGCTGTTATACGCATTATGAGAGCATGAAGCCCC

CTCACGAAAGTAACTTTAATACCTCCTGACCCCACGAAAGCTAAGAAA

>720_Sample_ASV17

CACCGCGGTTATACGAGAGACCCTAATTGATAGGTACGGCGTAAAGAGTGGTTTGGGATA

ACCCAAAAATAAAGCTAAAGACCTTCTAAGCCGTCACACGCATACCGAAGGCACGAGGCC

CCAACACGAAAGTAGCTTTACTATTACCCCGACGCCACGAAAGCTAAGAAA

>720_Sample_ASV18

CACCGCGGTTATACGAGAGGCCCTAGTTGATAACTACCGGCGTAAAGAGTGGTTATGGAA

AAATATTTAATAAAGCCGAACACCCCCTCAGCCGTCATACGCACCTGGGAGCACGAAGAC

CTACTGCGAAAGCAGCTTTAATTATGCCTGACCCCACGACAGCTAAGAAA

>720_Sample_ASV19

CACCGCGGTTATACGAGAGGCCCAAGTTGATACTCCACGGCGTAAAGAGTGGTTAGAAAA

GACCTACTACTAAAGCCGAACGCCTTCAAAGCTGTTATACGCATCCGAAGGTGAGAAGCC

CACCCACGAAAGTGGCTTTACAACCTTGACTCCACGAAAGCTATGACA

>720_Sample_ASV20

CACCGCGGTTATACGAGAGACCCCAGTTGATAAACACGGCGTAAAGAGTGGTTAAGGGCA

CAAAACAAATAAAGTCAAACAACCTCTTAGCTGTTATACGCATTATGAGGATATGAAGCC

CCCCTACGAAAGTAACTTTACCAACCCCTGACCCCACGAAAGCTAAGAAA

>720_Sample_ASV21

CACCGCGGTTATACGAGAGACCCTAATTAATAGATACGGCGTAAAGAGTGGTTTAGGACA

AAAATTAATAAAGCCGAAGTTTCTCCTAGCCGTTGTACGCACTCTGAGAACACGAAGCCC

CACTACGAAAGTAGCTTTACCTATTATTTCCTGACCCCACGAAAGCTAAGAAA

>720_Sample_ASV22

CACCGCGGTTATACGAGAGACTCCAGTTGATAGCTACGGCGTAAAGAGTGGTTTGGGGCC

CGCCCAAAATAAAGCCAAAGACCTCCCAAGCCGTCAAACGCACCCCGGAGGCACGAAGTC

CTAACGCGAAAGCAACTTTACCTTCCCCGACGCCACGAAAGCTAAGAAA

>720_Sample_ASV23

CACCGCGGTTATACGAAAGACCCTAGTTGATAGCTACGGCGTAAAGGGTGGTTAAGGTAT

ATAACAAATAAAGCTAAAGAGCCTCCAAGCCGTCGTACGCATTCTGAGAGCTCGAAACCC

TAACACGAAAGTAGCTTTAAAAAATAACACCTGACCCCACGAAAGCTAAGAAA

>736_Sample_ASV1

CACCGCGGTTATACGAAAGACCCTAGTTGATGGGTGCGGCGTAAAGGGTGGTTAGGGACT

ACAAAAAATAGAGCTAAAGATCCTCTAGGTCGTCATACACATCACGAGGAAACGAAGCCC

AAATACGAAAGTAGCTCTAAATATAATCCTGACGCCACGAAAGCCAAGAAA

>736_Sample_ASV2

CACCGCGGTTATACGAAAGACCCTAGTTGATGGGTGCGGCGTAAAGGGTGGTTAGGGACT

ACAAAAAATAGAGCTAAAGATCCTCTAGGTCGTCATACACATCACGAGAAAACGAAGCCC

AAATACGAAAGTAGCTCTAAATATAATCCTGACGCCACGAAAGCCAAGAAA

>736_Sample_ASV3

CACCGCGGTTATACGAGAGACCCTAGTTGATTAAAGCGGCGTAAAGAGTGGTTATGGAAC

TATTCTTTTAAAGCAGAAAACCTCTCAAACTGTTATACGCACCCAGAGGTCTGAATCCCT

CACACGAAAGTGACTTTATTTATGCCTACCAGAACCCACGAAAGCTGGGACA

>736_Sample_ASV4

CACCGCGGTTATACGAGAGACCCTAGTTGATTAAAGCGGCGTAAAGAGTGGTTATGGAAC

TATTCTTTTAAAGCAGAAAACCTCTCAAACTGTTATACGCACCCAGAGGTCTGAATCCCT

CACACGAAAGTGACTTTATTTATGCCTACCAGAACCCACGAAAGCTATGACA

>736_Sample_ASV5

CACCGCGGTTATACGAGAGGCCCAAGTTGACAGACACCGGCGTAAAGAGTGGCTAGGGAA

AAATTACTACTAAAGCCGAACACCTTCAGAACTGTCATACGTTCCCGAAGATAAGAAGCC

CCACCACGAAAGTGGCTTTATATTACCCGACCCCACGAAAGCTGTGAAA

>736_Sample_ASV6

CACCGCGGTTATACGAGAGGCCCAAGTTGACAGACACCGGCGTAAAGAGTGGCTAGGGAA

AAATTACTACTAAAGCCGAACACCTTCAGAACTGTCATACGTTCCCGAAGATAAGAAGCC

CCACCACGAAAGTGGCTTTATATTACCCGACCCCACGAAAGCTAAGAAA

>736_Sample_ASV7

CACCGCGGTTATACGAAAGACTCTAGTTGACAAACACGGCGTAAAGGGTGGTTAGGATAA

CAACAAAATAAAGCTAAAGACTTACCAAGCCGTCATACGCCCATGTAAGAGCGAAAACCA

CAAACGAAAGTAACTTTATTACACCATCCGAATCCACGAAAGCTAAGAAA

>736_Sample_ASV8

CACCGCGGTTATACGAGAGGCCCTAGTTGATCATCACGGCGTAAAGAGTGGTTAGGGGAT

TATCATAAATAAAGCCAAAGACCTCCCAAGCTGTCGCACGCATTCCGGAGGCGCGAAGCC

CACACACGAAAGTAGCTTTAACTATTGACCCTGATGCCACGAAAGCTAAGAAA

>736_Sample_ASV9

CACCGCGGTTAAACGAGAGACCCTAGTTGATCATCACGGCGTAAAGAGTGGTTAGGGGAT

TATCATAAATAAAGCCAAAGACCTCCCAAGCTGTCGCACGCATTCCGGAGGCGCGAAGCC

CACACACGAAAGTAGCTTTAACTATTGACCCTGATGCCACGAAAGCTAAGAAA

>736_Sample_ASV10

CACCGCGGTTATACGAAAGACCCTAGTTGATAGATCACGGCGTAAAGGGTGGTTAGGGAA

TACAAAAATAAAGCTAAAAATCCTCTAAGCTGTCATACGCTTCCCGAAGACATGAAACCC

AACCACGAAAGTAGCTTTAAACTCTCCTGACACCACGAAAGCCAAGGAA

>736_Sample_ASV11

CACCGCGGTTATACGAGAGGCCCAAGTTGATAAGCCCGGCGTAAAGAGTGGTTATGGAGA

ATAAAACACTAAAGCTAAAGACCCCCTAGGCTGTTATACGCACCTGGCGGCTCGAACCAC

CTATACGAAAGTAGCTTTACCCCCCTTCCACCAGAATCCACGACAGCTGGGGCA

>736_Sample_ASV12

CACCGCGGTTATACGAGAGGCCCTAGTTGATAGCCACGGCGCAAAGAGTGGTTAAGGAGC

CCCACTAAATAAAGTCGAACACTTCCTAGGCCGTTATACGTTTTCTAGAAGCACGAAACC

CAATTACACGAAAGCAACTTTATACTAAACAACCTGACCCCACGAAAGCTAAGAAA

>736_Sample_ASV13

CACCGCGGTTATACGAGAGGCCCAAGTTGACAGACATCGGCGTAAAGAGTGGTTAAGGAA

ACCCCATTACTAAAGCCGAACGCCCTCAGAACTGTCATACGTTCCCGAAGGTAAGAAGCC

CCACTACGAAAGTGGCTTTAACCACACCCGACCCCACGAAAGCTGTGAAA

>736_Sample_ASV14

CACCGCGGTTATACGAGAAACCCTAGTTGACAAATACGGCGTAAAGAGTGGTTAAGGGGT

ATCAAAAATAAAGTCAAACATTTTCCTAGTTGTTATAAGCATTTCGAGAACATGAAGCCC

AACCACGAAAGTAACTTTACCTTCACCTGACCCCACGAAAGCTAAGAAA

>736_Sample_ASV15

GACCGCGGTTATACGAGAGACTCTAGTTGATAGATACGGCGTAAAGAGTGGTTTGGGATA

AACTATAAATAAAGCCAAAGACCTTCCAAGCTGTTAAACGCATACCGAAGGCACGAAGCC

CCAACACGAAAGTAGCTTTACTACTACCCCCGATGCCACGAAAGCTAAGACA

>736_Sample_ASV16

CACCGCGGTTATACGAAAGACCCCAGTTTATAGACACGGCGTAAAGGGTGGTTATAGGAC

AAACTAACATAGAGCCAAAGACCCCCTAAGCCGTCATACGCTCACGAGGGCTCGAAGCCC

CTATACGAAAGTAGCTCTACCAAAAAATGCTCGACTCCACGAAAGCTGGGACA

>736_Sample_ASV17

AACCGCGGTTATACGAGAGACCCTAATTGATAGCTCACGGCGTAAAGGGTGGTTTGGGGA

AACCTCAAAATAAAGCTAAAGACCTTCTAAGCCGTTACACGCATACCGAAGGCACGAGGC

CCTAACACGAAAGTAGCTTTACTATTACCCCTGACGCCACGAAAGCTAAGAAA

>736_Sample_ASV18

CACCGCGGTTATACGAAAGACCCTAGTTGATGGATCACGGCGTAAAGGGTGGTTAAGGGA

TATAAAAATAAAGCTAAAAATCCTCTTAGCTGTCATACGCTTTCCGAAGACATGAGGCCC

AATTACGAAAGTAGCTTTAAACCACCCTGACACCACGAAAGCCAAGAAA

>736_Sample_ASV19

CACCGCGGTTATACGAGAGGCCCTAGTTGATAACTACCGGCGTAAAGAGTGGTTATGGAA

AAATATTTAATAAAGCCGAACACCCCCTCAGCCGTCATACGCACCTGGGAGCACGAAGAC

CTACTGCGAAAGCAGCTTTAATTATGCCTGACCCCACGACAGCTAAGAAA

>736_Sample_ASV20

CACCGCGGTTATACGAGAGGCCCAAGTTGATTATTACCGGCGTAAAGCGTGGTTAAGATT

TTTATAGGACTAAAGCCGAACCCCCTCAGAGCTGTTATACGCACCCGAAGGTTAGAAGTT

CAATCACGAAAGTGGCTTTACAACCCCGAACCCACGAAAGCTAAGATA

>736_Sample_ASV21

CACCGCGGTTATACGAGAGACCCTAATTGATAGGCGCGGCGTAAAGAGTGGTTTAGGACT

AAAACTAATAAAGCTAAAGATCCTCCCAGCCGTCGCACGCATTCTGAGGACACGAAACTC

AACCACGAAAGTAGCTTTAACATACTCTCCTGACCCCACGAAAGCTAAGAAA

>736_Sample_ASV22

CACCGCGGTTATACGAAAGACCCTAGTTGATAGGCACGGCGTAAAGGGTGGTTAGGGATA

TATCAAAAATAAAGTTAAAGAGCCTCTAAGCTGTCGCACGCATCCCGAGAGCTCGAGACC

CAGACACGAAAGTAACTTTAAAATACCTCACCTGACCCCACGAAAGCTAAGAAA

>736_Sample_ASV23

CACCGCGGTTATACGAGAGGCCCTAGTTGATAGCCACGGCGTAAAGGGTGGTTAAGGTAT

ACAACAAATAAAGCTAAATAGCCTCTAGGCCGTCGCACGCATTCCGAGAGCTCGAAACCC

AAAAACGAAAGTAGCTTTAAAATAAACAGACCTGACCCCACGAAAGCTAAGAAA

>736_Sample_ASV24

CACCGCGGTTATACGAGAGGCCCTAGTTGATAGCCACGGCGCAAAGAGTGGTTAAGGTAT

ACAACAAATAAAGCTAAATAGCCTCTAGGCCGTCGCACGCATTCCGAGAGCTCGAAACCC

AAAAACGAAAGTAGCTTTAAAATAAACAGACCTGACCCCACGAAAGCTAAGAAA

>736_Sample_ASV25

CACCGCGGTTATACGAAAGACCCTAGTTGATAGCCACGGCGTAAAGGGTGGTTAAGGTAT

ACAACAAATAAAGCTAAATAGCCTCTAGGCCGTCGCACGCATTCCGAGAGCTCGAAACCC

AAAAACGAAAGTAGCTTTAAAATAAACAGACCTGACCCCACGAAAGCTAAGAAA

>736_Sample_ASV26

CACCGCGGTTATACGAGAGGCCCAAGTTGATACTCCACGGCGTAAAGAGTGGTTAGAAAA

GACCTACTACTAAAGCCGAACGCCTTCAAAGCTGTTATACGCATCCGAAGGTGAGAAGCC

CACCCACGAAAGTGGCTTTACAACCTTGACTCCACGAAAGCTATGACA

>736_Sample_ASV27

CACCGCGGTTATACGAGAGACCCCAGTTGATAAACACGGCGTAAAGAGTGGTTAAGGGCA

CAAAACAAATAAAGTCAAACAACCTCTTAGCTGTTATACGCATTATGAGGATATGAAGCC

CCCCTACGAAAGTAACTTTACCAACCCCTGACCCCACGAAAGCTAAGAAA

>736_Sample_ASV28

CACCGCGGTTATACGAAAGACCCTAGTTTATAGGCACGGCGTAAAGGGTGGTTAGGGGAT

AAACAAAATAAAGCCAAAGACCTTCTAAGTCGTCATACACTCATGAAGGCACGAAGCCCA

AATACGAAAGTAACTTTACAACTTAGACCCGACTCCACGAAAGCTGGGGGA

>736_Sample_ASV29

CACCGCGGTTATACGAAAGACCCTAGTTTATAGGCACGGCGTAAAGGGTGGTTAGGGGAT

AAACAAAATAAAGCCAAAGACCTTCTAAGTCGTCATACACTCATGAAGGCACGAAGCCCA

AATACGAAAGTAACTTTACAACTTAGACCCGACTCCACGAAAGCTAAGAAA

>736_Sample_ASV30

CACCGCGGTTATACGAGAGACCCTAATTAATAGATACGGCGTAAAGAGTGGTTTAGGACA

AAAATTAATAAAGCCGAAGTTTCTCCTAGCCGTTGTACGCACTCTGAGAACACGAAGCCC

CACTACGAAAGTAGCTTTACCTATTATTTCCTGACCCCACGAAAGCTAAGAAA

>736_Sample_ASV31

CACCGCGGTTATACGAGAGACCCTAATTAATAGCTACGGCGTAAAGAGTGGTTTAGGGTA

AAAATTAATAAAGCCGAAGTTTCTCCTAGCCGTTGTACGCACTCTGAGAACACGAAGCCC

CACTACGAAAGTAGCTTTACCTATTATTTCCTGACCCCACGAAAGCTAAGAAA

>736_Sample_ASV32

CACCGCGGTTATACGAGAGACCCTAATTAATAGATACGGCGTAAAGAGTGGTTTAGGACA

AAAATTAATAAAGCCGAAGTTTCTCCTAGCCGTTGTACGCACTCTGAGAACACGAAGCCC

CACTACGAAAGTAGCTTTACCAATAATCTCCTGACCCCACGAAAGCTAAGAAA

>736_Sample_ASV33

CACCGCGGTTATACGAAAGACCCTAGTTGATAGATCACGGCGTAAAGGGTGGTTAAGGAA

AACAAAAATAAAGCTAAAAATCCTCTAAGCTGTCATACGCTTTCCGAAGACATGAGATCC

AACCACGAAAGTAGCTTTAAAATTTCCTGACACCACGAAAGCCAAGGAA

>744_Sample_ASV1

TACCGCGGTTATACGAAAGACCCAAGTTAATAAACTACGGCGTAAAGCGTGGTTAGAGAC

CTCCAATAATTAAAGCCAAAGACCTTCTAAGCCGTCGCACGCCCCGCGAAGACACGAAGC

CCAAAAACGAAAGTAGCTTTATTCTTACTCGACCCCACGAAAGCTAGGGCA

>744_Sample_ASV2

CACCGCGGTTATACGAAAGACCCTAGTTGATGGGTGCGGCGTAAAGGGTGGTTAGGGACT

ACAAAAAATAGAGCTAAAGATCCTCTAGGTCGTCATACACATCACGAGGAAACGAAGCCC

AAATACGAAAGTAGCTCTAAATATAATCCTGACGCCACGAAAGCCAAGAAA

>744_Sample_ASV3

CACCGCGGTTATACGAGAGACCCTAGTTGATTAAAGCGGCGTAAAGAGTGGTTATGGAAC

TATTCTTTTAAAGCAGAAAACCTCTCAAACTGTTATACGCACCCAGAGGTCTGAATCCCT

CACACGAAAGTGACTTTATTTATGCCTACCAGAACCCACGAAAGCTGGGACA

>744_Sample_ASV4

CACCGCGGTTATACGAGAGGCCCAAGTTGACAGACACCGGCGTAAAGAGTGGCTAGGGAA

AAATTACTACTAAAGCCGAACACCTTCAGAACTGTCATACGTTCCCGAAGATAAGAAGCC

CCACCACGAAAGTGACTTTATATTACCCGACCCCACGAAAGCTGTGAAA

>744_Sample_ASV5

CACCGCGGTTATACGAGAGGCCCAAGTTGACAGACACCGGCGTAAAGAGTGGCTAGGGAA

AAATTACTACTAAAGCCGAACACCCTCAGAACTGTCATACGTTCCCGAAGGTAAGAAGCC

CCACCACGAAAGTGGCTTTATAATACCCGACCCCACGAAAGCTGTGAAA

>744_Sample_ASV6

CACCGCGGTTATACGAGAGGCCCAAGTTGACAGACACCGGCGTAAAGAGTGGCTAGGGAA

AAATTACTACTAAAGCCGAACACCTTCAGAACTGTCATACGTTCCCGAAGATAAGAAGCC

CCACCACGAAAGTGGCTTTATATTACCCGACTCCACGAAAGCTGGGACA

>744_Sample_ASV7

CACCGCGGTTATACGAGAGGCCCAAGTTGACAGACACCGGCGTAAAGAGTGGCTAGGGAA

AAATTACTACTAAAGCCGAACACCTTCAGAACTGTCATACGTTCCCGAAGATAAGAAGCC

CCACCACGAAAGTGGCTTTATATTACCCGACCCCACGAAAGCTGGGACA

>744_Sample_ASV8

CACCGCGGTTATACGAGAGGCCCAAGTTGACAGACACCGGCGTAAAGAGTGGCTAGGGAA

AAATTACTACTAAAGCCGAACACCTTCAGAACTGTCATACGTTCCCGAAGATAAGAAGCC

CCACCACGAAAGTGGCTTTATATTACCCGACCCCACGAAAGCTGTGAAA

>744_Sample_ASV9

CACCGCGGTTATACGAAAGACTCTAGTTGACAAACACGGCGTAAAGGGTGGTTAGGATAA

CAACAAAATAAAGCTAAAGACTTACCAAGCCGTCATACGCCCATGTAAGAGCGAAAACCA

CAAACGAAAGTAACTTTATTACACCATCCGAATCCACGAAAGCTAAGAAA

>744_Sample_ASV10

CACCGCGGTTATACGAAAGACCCTAGTTGATAGATCACGGCGTAAAGGGTGGTTAGGGAA

TACAAAAATAAAGCTAAAAATCCTCTAAGCTGTCATACGCTTCCCGAAGACATGAAACCC

AACCACGAAAGTAGCTTTAAACTCTCCTGACACCACGAAAGCCAAGGAA

>744_Sample_ASV11

CACCGCGGTTATACGAGAGGCCCAAGTTGACAGACATCGGCGTAAAGAGTGGTTAAGGAA

ACCCCATTACTAAAGCCGAACGCCCTCAGAACTGTCATACGTTCCCGAAGGTAAGAAGCC

CCACTACGAAAGTGGCTTTAACCACACCCGACCCCACGAAAGCTGTGAAA

>744_Sample_ASV12

CACCGCGGTTATACGAGAGGCCCCAGTTGATATTAGCGGCGTAAAGGGTGGTTAAGGACT

TTAATAACTAAAGCCAAACACTTCCCCGGCTGTTGTACGTAGACCGGAAGCTTGAAGCCC

AGACACGAAAGTAGCTTTATTTCCCCTGACCCCACGAAAGCTGAGAAA

>744_Sample_ASV13

CACCGCGGTTATACGAGAGGCCCCAGTTGATATTAGCGGCGTAAAGGGTGGTTAAGGACT

TTAATAACTAAAGCTAAACACTTCCCCGGCTGTTGTACGTAGACCGGAAGCTTGAAGCCC

AGACACGAAAGTAGCTTTATTCCCCCTGACCCCACGAAAGCTGAGAAA

>744_Sample_ASV14

CACCGCGGTTATACGAGAGGCCCCAGTTGATATTAGCGGCGTAAAGGGTGGTTAAGGACT

TTAATAACTAAAGCTAAACACTTCCCCGGCTGTTGTACGTAGACCGGAAGCTTGAAGCCC

AGACACGAAAGTAGCTTTATTTCCCCTGACCCCACGAAAGCTGAGAAA

>744_Sample_ASV15

CACCGCGGTTATACGAAAGACCCCAGTTTATAGACACGGCGTAAAGGGTGGTTATAGGAC

AAACTAACATAGAGCCAAAGACCCCCTAAGCCGTCATACGCTCACGAGGGCTCGAAGCCC

CTATACGAAAGTAGCTCTACCAAAAAATGCTCGACTCCACGAAAGCTGGGACA

>744_Sample_ASV16

CACCGCGGTTATACGAGAGACCCTAATTGATAGGCACGGCGTAAAGAGTGGTTTAGGGTA

ACCCATAAATAAAGCTAAAGACCTTCTAAGCCGTCACACGCACACCGAAGGCACGAGACC

CCAACACGAAAGTAGCTTTACTTCTACCCCTGACGCCACGAAAGCTAAGAAA

>744_Sample_ASV17

CACCGCGGTTATACGAAAGACCCTAGTTGATGGATCACGGCGTAAAGGGTGGTTAAGGGA

TATAAAAATAAAGCTAAAAATCCTCTTAGCTGTCATACGCTTTCCGAAGACATGAGGCCC

AATTACGAAAGTAGCTTTAAACCACCCTGACACCACGAAAGCCAAGAAA

>744_Sample_ASV18

CACCGCGGTTATACGAAAGACCCCAGTTTATAGATGCGGCGTAAAGGGTGGTTAAGAGAA

AATCAAAATAAGGCCAAAGATCCCCTAAGCAGTCATACGCTCCACGAGGACACGAAACCC

AAATACGAAAGTTGCCTTAATAAACTTCACCTGATCCCACGAAAGCTGAAAAA

>744_Sample_ASV19

CACCGCGGTTATACGAAAGGCTCTAGTTTATAAATACGGCGTAAAGGGTGGTTAAGGAAA

AATTAAAATAATGCTAAAGACCCCCTAAGCAGTTATACGCTTCACGGAGGCACGAAACCC

AAATACGAAAGTTGCCTTAATAAACTTTACCTGACCCCACGAAAGCTGAAAAA

>744_Sample_ASV20

CACCGCGGTTATACGAAAGGCTCTAGTTTATAAATACGGCGTAAAGGGTGGTTAAGGAAA

AATTAAAATAATGCTAAAGACCCCCTAAGCAGTTATACGCTTCACGGAGGCACGAAACCC

AAATACGAAAGTTGCCTTAATAAACTTTACCTGACCCCACGAAAGCTGAGAAA

>744_Sample_ASV21

CACCGCGGTTATACGAGAGACTCTAGTTGATACCAACGGCGTAAAGAGTGGTTTGGGATG

CCCCTTAAAATAAAGCCAAAGACCTCCCAAGCCGTCAAACGCACCCCGGAGGCACGAAGC

CCCAACGCGAAAGCAACTTTACCTCCCTCCCGACGCCACGAAAGCTAAGAAA

>744_Sample_ASV22

CACCGCGGTTATACGAGAGACCCTAGTTGATAGCTACGGCGTAAAGCGTGGTTAGGAGAA

CAAGATAAATAAAGTCAAACAATCTCTCGGCCGTTATACGTTATCTGAGAATATGAAGTC

CTACCACGAAAGTAACTTTAATTTTTCTGACCCCACGAAAACTGAGGAA

>744_Sample_ASV23

CACCGCGGTTATACGAGAGGCCCTAGTTGATAACTACCGGCGTAAAGAGTGGTTATGGAA

AAATATTTAATAAAGCCGAACACCCCCTCAGCCGTCATACGCACCTGGGAGCACGAAGAC

CTACTGCGAAAGCAGCTTTAATTATGCCTGACCCCACGACAGCTAAGAAA

>744_Sample_ASV24

CACCGCGGTTATACGAGAGGCCCAAGTTGATTATTACCGGCGTAAAGCGTGGTTAAGATT

TTTATAGGACTAAAGCCGAACCCCCTCAGAGCTGTTATACGCACCCGAAGGTTAGAAGTT

CAATCACGAAAGTGGCTTTACAACCCCGAACCCACGAAAGCTAAGATA

>744_Sample_ASV25

CACCGCGGTTATACGAGAGGCCCAAGTTGATACTCCACGGCGTAAAGAGTGGTTAGAAAA

GACCTACTACTAAAGCCGAACGCCTTCAAAGCTGTTATACGCATCCGAAGGTGAGAAGCC

CACCCACGAAAGTGGCTTTACAACCTTGACTCCACGAAAGCTATGACA

>744_Sample_ASV26

CACCGCGGTTATACGAGAGACCCCAGTTGATAAACACGGCGTAAAGAGTGGTTAAGGGCA

CAAAACAAATAAAGTCAAACAACCTCTTAGCTGTTATACGCATTATGAGGATATGAAGCC

CCCCTACGAAAGTAACTTTACCAACCCCTGACCCCACGAAAGCTAAGAAA

>744_Sample_ASV27

CACCGCGGTTATACGAAAGACCCTAGTTTATAGGCACGGCGTAAAGGGTGGTTAGGGGAT

AAACAAAATAAAGCCAAAGACCTTCTAAGTCGTCATACACTCATGAAGGCACGAAGCCCA

AATACGAAAGTAACTTTACAACTTAGACCCGACTCCACGAAAGCTGGGGGA

>744_Sample_ASV28

CACCGCGGTTATACGAAAGACCCTAGTTTATAGGCACGGCGTAAAGGGTGGTTAGGGGAT

AAACAAAATAAAGCCAAAGACCTTCTAAGTCGTCATACACTCATGAAGGCACGAAGCCCA

AATACGAAAGTAACTTTACAACTTAGACCCGACTCCACGAAAGCCAAGGAA

>744_Sample_ASV29

CACCGCGGTTATACGAAAGACCCTAGTTGATAGCTACGGCGTAAAGGGTGGTTAAGGTAT

ATAACAAATAAAGCTAAAGAGCCTCCAAGCCGTCGTACGCATTCTGAGAGCTCGAAACCC

TAACACGAAAGTAGCTTTAAAAAATAACACCTGACCCCACGAAAGCTAAGAAA

>744_Sample_ASV30

CACCGCGGTTATACGAAAGACCCTAGTTGATAGATCACGGCGTAAAGGGTGGTTAAGGAG

AACAAAAATAAAGCTAAAAATCTTCTAAGCTGTCATACGCTTTCCGAAGACATGAGACCC

AACCACGAAAGTAGCTTTAAACTTTCCTGACACCACGAAAGCCAAGAAA

>745_Sample_ASV1

CACCGCGGTTATACGAGAGGCCCAAGTTGACAGACATCGGCGTAAAGAGTGGTTAAGGAA

AACCTTCAACTAAAGTCGAACGCTCTCAAGACTGTCGTACGTTTCCGAGAGTATGAAGCC

CCACCACGAAAGTGACTTTACCTCACCTGACTCCACGAAAGCTGCGAAA

>745_Sample_ASV2

CACCGCGGTTATACGAAAGACCCTAGTTGATGGGTGCGGCGTAAAGGGTGGTTAGGGACT

ACAAAAAATAGAGCTAAAGATCCTCTAGGTCGTCATACACATCACGAGGAAACGAAGCCC

AAATACGAAAGTAGCTCTAAATATAATCCTGACGCCACGAAAGCCAAGAAA

>745_Sample_ASV3

CACCGCGGTTATACGAGAGACCCTAGTTGATTAAAGCGGCGTAAAGAGTGGTTATGGAAC

TATTCTTTTAAAGCAGAAAACCTCTCAAACTGTTATACGCACCCAGAGGTCTGAATCCCT

CACACGAAAGTGACTTTATTTATGCCTACCAGAACCCACGAAAGCTATGACA

>745_Sample_ASV4

CACCGCGGTTATACGAGAGACCCTAGTTGATTAAAGCGGCGTAAAGAGTGGTTATGGAAC

TATTCTTTTAAAGCAGAAAACCTCTCAAACTGTTATACGCACCCAGAGGTCTGAATCCCT

CACACGAAAGTGACTTTATTTATGCCTACCAGAACCCACGAAAGCTGGGACA

>745_Sample_ASV5

CACCGCGGTTATACGAAAGACCCTAGTTTATAGGTACGGCGTAAAGGGTGGTTAGGGGAC

AAACAAAATAAAGCCAAAGACCTCCTAAGCCGTCATACGCTCACGGAAGCACGAAGCCCA

AACACGAAAGTAGCTTTACCGAACATGCCCGACTCCACGAAAGCTGGGGAA

>745_Sample_ASV6

CACCGCGGTTATACGAGAGGCCCAAGTTGACAGACACCGGCGTAAAGAGTGGCTAGGGAA

AAATTACTACTAAAGCCGAACACCTTCAGAACTGTCATACGTTCCCGAAGATAAGAAGCC

CCACCACGAAAGTGGCTTTATATTACCCGACCCCACGAAAGCTGTGAAA

>745_Sample_ASV7

CACCGCGGTTATACGAGAGGCCCAAGTTGACAGACACCGGCGTAAAGAGTGGCTAGGGAA

AAATTACTACTAAAGCCGAACACCTTCAGAACTGTCATACGTTCCCGAAGATAAGAAGCC

CCACCACGAAAGTGGCTTTATATTACCCGACCCCACGAAAGCTAAGAAA

>745_Sample_ASV8

CACCGCGATTATACGAGAGGCCCAAGTTGACAGACATCGGCGTAAAGAGTGGTTAAGGAA

AAATTACTACTAAAGCCGAACACCTTCAGAACTGTCATACGTTCCCGAAGATAAGAAGCC

CCACCACGAAAGTGGCTTTATATTACCCGACCCCACGAAAGCTGTGAAA

>745_Sample_ASV9

CACCGCGGTTATACGAGAGGCCCAAGTTGACAGACACCGGCGTAAAGAGTGGCTAGGGAA

AAATTACTACTAAAGCCGAACACCTTCAGAACTGTCATACGTTCCCGAAGATAAGAAGCC

CCACCACGAAAGTGGCTTTACAATGCCTGACCCCACGAAAGCTGTGAAA

>745_Sample_ASV10

CACCGCGGTTAAACGAGAGACCCTAGTTGATCATCACGGCGTAAAGAGTGGTTAGGGGAT

TATCATAAATAAAGCCAAAGACCTCCCAAGCTGTCGCACGCATTCCGGAGGCGCGAAGCC

CACACACGAAAGTAGCTTTAACTATTGACCCTGATGCCACGAAAGCTAAGAAA

>745_Sample_ASV11

CACCGCGGTTATACGAGAGGCCCAAGTTGATAAGCCCGGCGTAAAGAGTGGTTATGGAGA

ATAAAACACTAAAGCTAAAGACCCCCTAGGCTGTTATACGCACCTGGCGGCTCGAACCAC

CTATACGAAAGTAGCTTTACCCCCCTTCCACCAGAATCCACGACAGCTGGGGCA

>745_Sample_ASV12

CACCGCGGTTATACGAGAGGCCCAAGTTGACAGACATCGGCGTAAAGAGTGGTTAAGGAA

ACCCCATTACTAAAGCCGAACGCCCTCAGAACTGTCATACGTTCCCGAAGGTAAGAAGCC

CCACTACGAAAGTGGCTTTAACCACACCCGACCCCACGAAAGCTGTGAAA

>745_Sample_ASV13

CACCGCGGTTATACGAGAGGCCCAAGTTGATAGATATCGGCGTAAAGAGTGGTTAAGGAA

ACCCCGTTACTAAAGCCGAACGCCCTCAGAACTGTCATACGTTCCCGAAGGTAAGAAGCC

CCACTACGAAAGTGGCTTTAACCACACCCGACCCCACGAAAGCTGTGAAA

>745_Sample_ASV14

CACCGCGGTTATACGAGAGGCCCTAGTTGATAAATACGGCGTAAAGAGTGGTTAGAGGAC

TCCCATAATAAAGCCAAAGACCCCCCGGGCCGTCACACGCATACGAGGGCACGAAGTTCA

CACACGAAAGTAGCTTTAAATTACCCGACGCCACGAAAGCTAAGAAA

>745_Sample_ASV15

GACCGCGGTTATACGAGAGACTCTAGTTGATAGATACGGCGTAAAGAGTGGTTTGGGATA

AACTATAAATAAAGCCAAAGACCTTCCAAGCTGTTAAACGCATACCGAAGGCACGAAGCC

CCAACACGAAAGTAGCTTTACTACTACCCCCGATGCCACGAAAGCTAAGACA

>745_Sample_ASV16

CACCGCGGTTATACGAGAGACCCCAGTTTATAGACACGGCGTAAAGGGTGGTTATAGGAC

AAACTAACATAGAGCCAAAGACCCCCTAAGCCGTCATACGCTCACGAGGGCTCGAAGCCC

CTATACGAAAGTAGCTCTACCAAAAAATGCTCGACTCCACGAAAGCTGGGACA

>745_Sample_ASV17

CACCGCGGTTATACGAAAGACCCCAGTTTATAGACACGGCGTAAAGGGTGGTTATAGGAC

AAACTAACATAGAGCCAAAGACCCCCTAAGCCGTCATACGCTCACGAGGGCTCGAAGCCC

CTATACGAAAGTAGCTCTACCAAAAAATGCTCGACTCCACGAAAGCTGGGACA

>745_Sample_ASV18

CACCGCGGTTATACGAAAGACCCCAGTTTATAGACACGGCGTAAAGGGTGGTTATAGGAC

AAACTAACATAGAGCCAAAGACCCCCTAAGCCGTCATACGCTCACGAGGGCTCGAAGCCC

CTATACGAAAGTAGCTCTACCAAAAAATGCTCGACTCCACGAAAGCTAAGAAA

>745_Sample_ASV19

CACCGCGGTTATACGAGAGACCCTAGTTGATAAATACGGCGTAAAGAGTGGTTATGGGAA

AAACAAATAAAGTCAAACAACCTCTTAGCTGTTATACGCATTATGAGAGCATGAAGCCCC

CTCACGAAAGTAACTTTAATACCTCCTGACCCCACGAAAGCTAAGAAA

>745_Sample_ASV20

CACCGCGGTCATACGATTAACCCAAGTTAATAGGCACACGGCGTAAAGCGTGTTTAAGCA

CTATGCCAAATAAAGTTAAACTCCAATTAAGCTGTAAAAAGCCATAATCAAGACGAAAAT

AAATAACGAAAGTAACTTTACAACCGCTGAAACACGATAGCTAAGATC

>745_Sample_ASV21

CACCGCGGTTATACGAAAGACCCTAGTTTATAGACACGGCGTAAAGGGTGGTTCGGGACT

AAACAAAATAAAGCCAAAGACCTCCTAAGCCGTCATACGCTCACGGAAGCACGAAGCCCA

AGTACGAAAGTAGCTTTACAAAACATGCCTGACTCCACGAAAGCTAGGAAA

>745_Sample_ASV22

CACCGCGGTTATACGAAAGACCCTAGTTGATAGGCACGGCGTAAAGGGTGGTTAGGGATA

TATCAAAAATAAAGTTAAAGAGCCTCTAAGCTGTCGCACGCATCCCGAGAGCTCGAGACC

CAGACACGAAAGTAACTTTAAAATACCTCACCTGACCCCACGAAAGCTAAGAAA

>745_Sample_ASV23

CACCGCGGTTATACGAAAGACCCTAGTTGATAGACACGGCGTAAAGGGTGGTTAGGGATA

CATCAAAAATAAAGTTAAAGAGCCTCTAAGCTGTCGCACGCATCCTGAGAGCTCGAGACC

CAAACACGAAAGTAACTTTAAAATACTTCACCTGACCCCACGAAAGCTAAGAAA

>745_Sample_ASV24

CACCGCGGTTATACGAAAGACCCTAGTTGATAGCCACGGCGTAAAGGGTGGTTAAGGTAT

ACAACAAATAAAGCTAAATAGCCTCTAGGCCGTCGCACGCATTCCGAGAGCTCGAAACCC

AAAAACGAAAGTAGCTTTAAAATAAACAGACCTGACCCCACGAAAGCTAAGAAA

>745_Sample_ASV25

CACCGCGGTTATACGAGAGGCCCAAGTTGATACTCCACGGCGTAAAGAGTGGTTAGAAAA

GACCTACTACTAAAGCCGAACGCCTTCAAAGCTGTTATACGCATCCGAAGGTGAGAAGCC

CACCCACGAAAGTGGCTTTACAACCTTGACTCCACGAAAGCTGGGACA

>745_Sample_ASV26

CACCGCGGTTATACGAGAGGCCCAAGTTGATACTCCACGGCGTAAAGAGTGGTTAGAAAA

GACCTACTACTAAAGCCGAACGCCTTCAAAGCTGTTATACGCATCCGAAGGTGAGAAGCC

CACCCACGAAAGTGGCTTTACAACCTTGACTCCACGAAAGCTATGACA

>745_Sample_ASV27

CACCGCGGTTATACGAGAGACCCCAGTTGATAAACACGGCGTAAAGAGTGGTTAAGGGCA

CAAAACAAATAAAGTCAAACAACCTCTTAGCTGTTATACGCATTATGAGGATATGAAGCC

CCCCTACGAAAGTAACTTTACCAACCCCTGACCCCACGAAAGCTAAGAAA

>745_Sample_ASV28

CACCGCGGTTATACGAAAGACCCTAGTTTATAGGCACGGCGTAAAGGGTGGTTAGGGGAT

AAACAAAATAAAGCCAAAGACCTTCTAAGTCGTCATACACTCATGAAGGCACGAAGCCCA

AATACGAAAGTAACTTTACAACTTAGACCCGACTCCACGAAAGCTGGGGGA

>745_Sample_ASV29

CACCGCGGTTATACGAGAGGCCCAAGTTGATAGACATCGGCGTAAAGAGTGGTTAAGGAA

GTCTCAAAACTAAAGCCGAATGCCTTCAAGACTGTTATACGTTTCCGAAGGTAAGAAGCC

CCACTACGAAAGTGGCTTTACTCCACCTGACTCCACGAAAGCTGTGAAA

>745_Sample_ASV30

CACCGCGGTTATACGAGAGACCCCAGTTGATAAACACGGCGTAAAGAGTGGTTTGGGGCC

CGCCCAAAATAAAGCCAAAGACCTCCCAAGCCGTCAAACGCACCCCGGAGGCACGAAGTC

CTAACGCGAAAGCAACTTTACCTTCCCCGACGCCACGAAAGCTAAGAAA

>745_Sample_ASV31

CACCGCGGTTATACGAGAGACTCCAGTTGATAGCTACGGCGTAAAGAGTGGTTTGGGGCC

CGCCCAAAATAAAGCCAAAGACCTCCCAAGCCGTCAAACGCACCCCGGAGGCACGAAGTC

CTAACGCGAAAGCAACTTTACCTTCCCCGACGCCACGAAAGCTAAGAAA

>745_Sample_ASV32

CACCGCGGTTATACGAAAGACCCTAGTTGATAGATCACGGCGTAAAGGGTGGTTAAGGAG

AACAAAAATAAAGCTAAAAATCTTCTAAGCTGTCATACGCTTTCCGAAGACATGAGACCC

AACCACGAAAGTAGCTTTAAACTTTCCTGACACCACGAAAGCCAAGAAA

>754_Sample_ASV1

CACCGCGGTTATACGAAAGACCCTAGTTGATGGGTGCGGCGTAAAGGGTGGTTAGGGACT

ACAAAAAATAGAGCTAAAGATCCTCTAGGTCGTCATACACATCACGAGGAAACGAAGCCC

AAATACGAAAGTAGCTCTAAATATAATCCTGACGCCACGAAAGCCAAGAAA

>754_Sample_ASV2

CACCGCGGTTATACGAGAGACCCTAGTTGATATACACGGCGTAAAGAGTGGTTTGGGGTA

TATAATAAATAAAGCCAAAGACCTTCTAGGCCGTTGAACGCACCACGAAGGAGCGAAGTC

CCAATACGAAAGTAGCTTTATAAAACCTGACGCCACGAAAGCTAAGAAA

>754_Sample_ASV3

CACCGCGGTTATACGAGAGGCCCAAGTTGACAGACACCGGCGTAAAGAGTGGCTAGGGAA

AAATTACTACTAAAGCCGAACACCTTCAGAACTGTCATACGTTCCCGAAGATAAGAAGCC

CCACCACGAAAGTGACTTTATATTACCCGACCCCACGAAAGCTGTGAAA

>754_Sample_ASV4

CACCGCGGTTATACGAGAGGCCCAAGTTGACAGACACCGGCGTAAAGAGTGGCTAGGGAA

AAATTACTACTAAAGCCGAACACCTTCAGAACTGTCATACGTTCCCGAAGATAAGAAGCC

CCACCACGAAAGTGGCTTTATATTACCCGACCCCACGAAAGCTGTGAAA

>754_Sample_ASV5

CACCGCGGTTATACGAGAGGCCCAAGTTGACAGACACCGGCGTAAAGAGTGGCTAGGGAA

AAATTACTACTAAAGCCGAACACCTTCAGAACTGTCATACGTTCCCGAAGATAAGAAGCC

CCACCACGAAAGTGGCTTTATATTACCCGACCCCACGAAAGCTGTGACA

>754_Sample_ASV6

CACCGCGGTTATACGAGAGGCCCAAGTTGATAAGCCCGGCGTAAAGAGTGGTTATGGAGA

ATAAAACACTAAAGCTAAAGACCCCCTAGGCTGTTATACGCACCTGGCGGCTCGAACCAC

CTATACGAAAGTAGCTTTACCCCCCTTCCACCAGAATCCACGACAGCTGGGGCA

>754_Sample_ASV7

CACCGCGGTTATACGAGAGGCCCAAGTTGACAGACATCGGCGTAAAGAGTGGTTAAGGAA

ACCCCATTACTAAAGCCGAACGCCCTCAGAACTGTCATACGTTCCCGAAGGTAAGAAGCC

CCACTACGAAAGTGGCTTTAACCACACCCGACCCCACGAAAGCTGTGAAA

>754_Sample_ASV8

CACCGCGGTTATACGAGAAACCCTAGTTGACAAATACGGCGTAAAGAGTGGTTAAGGGGT

ATCAAAAATAAAGTCAAACATTTTCCTAGTTGTTATAAGCATTTCGAGAACATGAAGCCC

AACCACGAAAGTAACTTTACCTTCACCTGACCCCACGAAAGCTAAGAAA

>754_Sample_ASV9

CACCGCGGTTATACGAAAGACCCCAGTTTATAGACACGGCGTAAAGGGTGGTTATAGGAC

AAACTAACATAGAGCCAAAGACCCCCTAAGCCGTCATACGCTCACGAGGGCTCGAAGCCC

CTATACGAAAGTAGCTCTACCAAAAAATGCTCGACTCCACGAAAGCTGGGACA

>754_Sample_ASV10

CACCGCGGTTATACGAGAGACCCTAGTTGATAAATACGGCGTAAAGAGTGGTTATGGGAA

AAACAAATAAAGTCAAACAACCTCTTAGCTGTTATACGCATTATGAGAGCATGAAGCCCC

CTCACGAAAGTAACTTTAATACCTCCTGACCCCACGAAAGCTAAGAAA

>754_Sample_ASV11

AACCGCGGTTATACGAGAGACCCTAATTGATAGCTCACGGCGTAAAGGGTGGTTTGGGGA

AACCTCAAAATAAAGCTAAAGACCTTCTAAGCCGTTACACGCATACCGAAGGCACGAGGC

CCTAACACGAAAGTAGCTTTACTATTACCCCTGACGCCACGAAAGCTAAGAAA

>754_Sample_ASV12

CACCGCGGTTATACGAAAGACCCTAGTTGATGGATCACGGCGTAAAGGGTGGTTAAGGGA

TATAAAAATAAAGCTAAAAATCCTCTTAGCTGTCATACGCTTTCCGAAGACATGAGGCCC

AATTACGAAAGTAGCTTTAAACCACCCTGACACCACGAAAGCCAAGAAA

>754_Sample_ASV13

CACCGCGGTTATACGAGAGACCCTAGTTGATAGCTACGGCGTAAAGCGTGGTTAGGAGAA

CAAGATAAATAAAGTCAAACAATCTCTCGGCCGTTATACGTTATCTGAGAATATGAAGTC

CTACCACGAAAGTAACTTTAATTTTTCTGACCCCACGAAAACTGAGGAA

>754_Sample_ASV14

CACCGCGGTTATACGAGAGACCCCAGTTGATAAACACGGCGTAAAGAGTGGTTAAGGGCA

CAAAACAAATAAAGTCAAACAACCTCTTAGCTGTTATACGCATTATGAGGATATGAAGCC

CCCCTACGAAAGTAACTTTACCAACCCCTGACCCCACGAAAGCTAAGAAA

>754_Sample_ASV15

CACCGCGGTTATACGAAAGACCCTAGTTTATAGGCACGGCGTAAAGGGTGGTTAGGGGAT

AAACAAAATAAAGCCAAAGACCTTCTAAGTCGTCATACACTCATGAAGGCACGAAGCCCA

AATACGAAAGTAACTTTACAACTTAGACCCGACTCCACGAAAGCTGGGGGA

>754_Sample_ASV16

CACCGCGGTTATACGAGAGACCCTAATTAATAGATACGGCGTAAAGAGTGGTTTAGGACA

AAAATTAATAAAGCCGAAGTTTCTCCTAGCCGTTGTACGCACTCTGAGAACACGAAGCCC

CACTACGAAAGTAGCTTTACCTATTATTTCCTGACCCCACGAAAGCTAAGAAA

>754_Sample_ASV17

CACCGCGGTTATACGAGAGGCCCAAGTTGATAGACATCGGCGTAAAGAGTGGTTAAGGAA

GTCTCAAAACTAAAGCCGAATGCCTTCAAGACTGTTATACGTTTCCGAAGGTAAGAAGCC

CCACTACGAAAGTGGCTTTACTCCACCTGACTCCACGAAAGCTGTGAAA

>760_Sample_ASV1

CACCGCGGTTATACGAAAGACCCTAGTTGATGGGTGCGGCGTAAAGGGTGGTTAGGGACT

ACAAAAAATAGAGCTAAAGATCCTCTAGGTCGTCATACACATCACGAGGAAACGAAGCCC

AAATACGAAAGTAGCTCTAAATATAATCCTGACGCCACGAAAGCCAAGAAA

>760_Sample_ASV2

CACCGCGGTTAAACGAGAGGCCCTAGTTGATGGTGCAACGGCGTAAAGGGTGGTTAAGGA

TAGCGAAATAATAAAGTCAAATGGCCCTCTGGCTGTCATACGCTTCTAGGAGTCCGAAGC

CCAGTATACGAAAGTAGCTTTAAGAAAGTCCACCTGACCCCACGAAAACTGAGGAA

>760_Sample_ASV3

CACCGCGGTTATACGAGAGACCCTAGTTGATTAAAGCGGCGTAAAGAGTGGTTATGGAAC

TATTCTTTTAAAGCAGAAAACCTCTCAAACTGTTATACGCACCCAGAGGTCTGAATCCCT

CACACGAAAGTGACTTTATTTATGCCTACCAGAACCCACGAAAGCTGGGACA

>760_Sample_ASV4

CACCGCGGTTATACGAAAGACCCTAGTTTATAGGTACGGCGTAAAGGGTGGTTAGGGGAC

AAACAAAATAAAGCCAAAGACCTCCTAAGCCGTCATACGCTCACGGAAGCACGAAGCCCA

AACACGAAAGTAGCTTTACCGAACATGCCCGACTCCACGAAAGCTGGGGAA

>760_Sample_ASV5

CACCGCGGTTATACGAGAGGCCCAAGTTGACAGACACCGGCGTAAAGAGTGGCTAGGGAA

AAATTACTACTAAAGCCGAACACCTTCAGAACTGTCATACGTTCCCGAAGATAAGAAGCC

CCACCACGAAAGTGGCTTTATATTACCCGACCCCACGAAAGCTGTGAAA

>760_Sample_ASV6

CACCGCGGTTATACGAGAGACCCCAGTTGATAAACACGGCGTAAAGAGTGGCTAGGGAAA

AATTACTACTAAAGCCGAACACCTTCAGAACTGTCATACGTTCCCGAAGATAAGAAGCCC

CACCACGAAAGTGGCTTTATATTACCCGACCCCACGAAAGCTGTGAAA

>760_Sample_ASV7

CACCGCGGTTATACGAGAGGCCCAAGTTGACAGACACCGGCGTAAAGAGTGGCTAGGGAA

AAATTACTACTAAAGCCGAACACCTTCAGAACTGTCATACGTTCCCGAAGATAAGAAGCC

CCACCACGAAAGTGGCTTTATATTACCCGACCCCACGAAAGCTAAGAAA

>760_Sample_ASV8

CACCGCGGTTATACGAGAGACCCTAGTTGATATATACGGCGTAAAGAGTGGTTTGGGGTT

ATGCCTCCAAAATAAAGCCGAAGATCTCCCAAGCCGTCACACGCATCCCGGAGACACGAA

GCCCTAATACGAAGGTAGCTTTATTAATTCCCCGACGCCACGAAAGCTAAGAAA

>760_Sample_ASV9

CACCGCGGTTATACGAGAGACCCTAGTTGATATATACGGCGTAAAGAGTGGTTTGGGATA

TTTCTCCAAAATAAAGCCGAAGATCTCCCAAGCCGTCACACGCATCCCGGAGACACGAAG

CCCTAATACGAAGGTAGCTTTATTAGTACCCCGACGCCACGAAAGCTAAGAAA

>760_Sample_ASV10

CACCGCGGTTATACGAGAGACCCTAGTTGATATATACGGCGTAAAGAGTGGTTTGGGGTT

ATGCCTCCAAAATAAAGCCGAAGATCTCCCAAGCCGTCACACGCATCCCGGAGACACGAA

GCCCTAATACGAAGGTAGCTTTATTAGTACCCCGACGCCACGAAAGCTAAGAAA

>760_Sample_ASV11

CACCGCGGTTATACGAGAGGCCCAAGTTGATAAGCCCGGCGTAAAGAGTGGTTATGGAGA

ATAAAACACTAAAGCTAAAGACCCCCTAGGCTGTTATACGCACCTGGCGGCTCGAACCAC

CTATACGAAAGTAGCTTTACCCCCCTTCCACCAGAATCCACGACAGCTGGGGCA

>760_Sample_ASV12

CACCGCGGTTATACGAGAGGCCCTAGTTGATAGCCACGGCGCAAAGAGTGGTTAAGGAGC

CCCACTAAATAAAGTCGAACACTTCCTAGGCCGTTATACGTTTTCTAGAAGCACGAAACC

CAATTACACGAAAGCAACTTTATACTAAACAACCTGACCCCACGAAAGCTAAGAAA

>760_Sample_ASV13

CACCGCGGTTATACGAGAGGCCCAAGTTGACAGACATCGGCGTAAAGAGTGGTTAAGGAA

ACCCCATTACTAAAGCCGAACGCCCTCAGAACTGTCATACGTTCCCGAAGGTAAGAAGCC

CCACTACGAAAGTGGCTTTAACCACACCCGACCCCACGAAAGCTGTGAAA

>760_Sample_ASV14

CACCGCGGTTATACGAGAGGCCCAAGTTGATAGATATCGGCGTAAAGAGTGGTTAAGGAA

ACCCCGTTACTAAAGCCGAACGCCCTCAGAACTGTCATACGTTCCCGAAGGTAAGAAGCC

CCACTACGAAAGTGGCTTTAACCACACCCGACCCCACGAAAGCTGTGAAA

>760_Sample_ASV15

CACCGCGGTTATACGAGAGGCCCAAGTTGACAGACACCGGCGTAAAGAGTGGTTAAGGAA

ATTCATCAACTAAAGCCGAATGCCCTCAGGACTGTTATACGTTTGCGAAGGTAAGAAGCC

CTACTACGAAAGTGGCTTTATTCCACCTGATTCCACGAAAGCTGCGGAA

>760_Sample_ASV16

CACCGCGGTTATACGAAAGACCCCAGTTTATAGACACGGCGTAAAGGGTGGTTATAGGAC

AAACTAACATAGAGCCAAAGACCCCCTAAGCCGTCATACGCTCACGAGGGCTCGAAGCCC

CTATACGAAAGTAGCTCTACCAAAAAATGCTCGACTCCACGAAAGCTGGGACA

>760_Sample_ASV17

CACCGCGGTTATACGAGAGGCCCCAGTTGATAAACACGGCGTAAAGGGTGGTTAAGGGAG

CATAAGAATAAAGCCGAAGGGCCCTTTGGCCGTTATACGCTTCTAGGTGTCCGAAGCCCA

AACACGAAAGTAGCTTTAGTTAAGCCCACCTGACTCCACGAAAACTGAGAAA

>760_Sample_ASV18

CACCGCGGTTATACGAGAGACCCTAGTTGATAAATACGGCGTAAAGAGTGGTTATGGGAA

AAACAAATAAAGTCAAACAACCTCTTAGCTGTTATACGCATTATGAGAGCATGAAGCCCC

CTCACGAAAGTAACTTTAATACCTCCTGACCCCACGAAAGCTAAGAAA

>760_Sample_ASV19

CACCGCGGTTATACGAGAGACCCTAGTTGATATATACGGCGTAAAGAGTGGTTATGGGAA

AAACAAATAAAGTCAAACAACCTCTTAGCTGTTATACGCATTATGAGAGCATGAAGCCCC

CTCACGAAAGTAACTTTAATACCTCCTGACCCCACGAAAGCTAAGAAA

>760_Sample_ASV20

CACCGCGGTTATACGAGAGGCTCAAGTTGATGAACCCCGGCGTAAAGAGTGGTTAAGGGA

GATCAAAACTAAAGCCGAATGCTTTCAAAGCTGTTATACGCTTCCGAAAGTAAGAAGCTC

AATCACGAAAGTGGCTTTACTTTACCTGACCCCACGAAAGCTACGACA

>760_Sample_ASV21

CACCGCGGTTATACGAGAGGCCCAAGTTGACAAACCCCGGCGTAAAGAGTGGTTAAGGGA

GATCAAAACTAAAGCCGAATGCTTTCAAAGCTGTTATACGCTTCCGAAAGTAAGAAGCTC

AATCACGAAAGTGGCTTTACTTTACCTGACCCCACGAAAGCTACGACA

>760_Sample_ASV22

CACCGCGGTTATACGAGAGGCTCAAGTTGATGAACCCCGGCGTAAAGAGTGGTTAAGGGA

GATCAAAACTAAAGCCGAATGCTTTCAAAGCTGTTATACGCTTCCGAAAGTAAGAAGCTC

AATCACGAAAGTGGCTTTACACTACCTGAACCCACGAAAGCTATGAAA

>760_Sample_ASV23

CACCGCGGTTATACGAAAGACCCTAGTTGATGGATCACGGCGTAAAGGGTGGTTAAGGGA

TATAAAAATAAAGCTAAAAATCCTCTTAGCTGTCATACGCTTTCCGAAGACATGAGGCCC

AATTACGAAAGTAGCTTTAAACCACCCTGACACCACGAAAGCCAAGAAA

>760_Sample_ASV24

CACCGCGGTTATACGAAAGACCCCAGTTTATAGATGCGGCGTAAAGGGTGGTTAAGAGAA

AATCAAAATAAGGCCAAAGATCCCCTAAGCAGTCATACGCTCCACGAGGACACGAAACCC

AAATACGAAAGTTGCCTTAATAAACTTCACCTGATCCCACGAAAGCTGAAAAA

>760_Sample_ASV25

CACCGCGGTTATACGAGAGGCCCAAGTTGACAAACCCCGGCGTAAAGAGTGGTTAAGGAA

AATTTAAAACTAAAGCCGAATGCCCCCAAAGCTGTTATACGCACCCGAGGATAAGAAGCC

CAATCACGAAAGTGGCTTTACACTACCTGAACCCACGAAAGCTATGAAA

>760_Sample_ASV26

CACCGCGGTTATACGAGAGGCCCAAGTTGACAAACCCCGGCGTAAAGAGTGGTTAAGGAA

AATTTAAAACTAAAGCCGAATGCCCCCAAAGCTGTTATACGCACCCGAGGGTAAGAAGCC

CAATCACGAAAGTGGCTTTACACTACCTGAACCCACGAAAGCTATGAAA

>760_Sample_ASV27

CACCGCGGTTATACGAGAGGCCCTAGTTGATAACTACCGGCGTAAAGAGTGGTTATGGAA

AAATATTTAATAAAGCCGAACACCCCCTCAGCCGTCATACGCACCTGGGAGCACGAAGAC

CTACTGCGAAAGCAGCTTTAATTATGCCTGACCCCACGACAGCTAAGAAA

>760_Sample_ASV28

CACCGCGGTTATACGAAAGACCCTAGTTTATAGACACGGCGTAAAGGGTGGTTCGGGACT

AAACAAAATAAAGCCAAAGACCTCCTAAGCCGTCATACGCTCACGGAAGCACGAAGCCCA

AGTACGAAAGTAGCTTTACAAAACATGCCTGACTCCACGAAAGCTAGGAAA

>760_Sample_ASV29

CACCGCGGTTATACGAGAGGCCCAAGTTGATACTCCACGGCGTAAAGAGTGGTTAGAAAA

GACCTACTACTAAAGCCGAACGCCTTCAAAGCTGTTATACGCATCCGAAGGTGAGAAGCC

CACCCACGAAAGTGGCTTTACAACCTTGACTCCACGAAAGCTATGACA

>760_Sample_ASV30

CACCGCGGTTATACGAGAGACCCCAGTTGATAAACACGGCGTAAAGAGTGGTTAAGGGCA

CAAAACAAATAAAGTCAAACAACCTCTTAGCTGTTATACGCATTATGAGGATATGAAGCC

CCCCTACGAAAGTAACTTTACCAACCCCTGACCCCACGAAAGCTAAGAAA

>760_Sample_ASV31

CACCGCGGTTATACGAAAGACCCTAGTTTATAGGCACGGCGTAAAGGGTGGTTAGGGGAT

AAACAAAATAAAGCCAAAGACCTTCTAAGTCGTCATACACTCATGAAGGCACGAAGCCCA

AATACGAAAGTAACTTTACAACTTAGACCCGACTCCACGAAAGCTGGGGGA

>760_Sample_ASV32

CACCGCGGTTATACGAGAGGCCCAAGTTGACAAACTCCGGCGTAAAGCGTGGTTAAGGCA

CACTCAAAAACTAAAGCCGAATATCTTCAGAGCAGTCATACGCATCCGAAGACACGAAGC

CCCACCACGAAAGTGGCTTTATGACACCTGACCCCACGAAAGCTAGGACA

>760_Sample_ASV33

CACCGCGGTTATACGAGAGACTCCAGTTGATAGCTACGGCGTAAAGAGTGGTTTGGGGCC

CGCCCAAAATAAAGCCAAAGACCTCCCAAGCCGTCAAACGCACCCCGGAGGCACGAAGTC

CTAACGCGAAAGCAACTTTACCTTCCCCGACGCCACGAAAGCTAAGAAA

>762_Sample_ASV1

CACCGCGGTTATACGAGAGGCCCAAGTTGACAGACATCGGCGTAAAGAGTGGTTAAGGAA

AACCTTCAACTAAAGTCGAACGCTCTCAAGACTGTCGTACGTTTCCGAGAGTATGAAGCC

CCACCACGAAAGTGACTTTACCTCACCTGACTCCACGAAAGCTGCGAAA

>762_Sample_ASV2

CACCGCGGTTATACGAAAGACCCTAGTTGATGGGTGCGGCGTAAAGGGTGGTTAGGGACT

ACAAAAAATAGAGCTAAAGATCCTCTAGGTCGTCATACACATCACGAGGAAACGAAGCCC

AAATACGAAAGTAGCTCTAAATATAATCCTGACGCCACGAAAGCCAAGAAA

>762_Sample_ASV3

CACCGCGGTTATACGAAAGACCCTAGTTGATAACTACGGCATAAAGGGTGGTTAGGGAGT

TTAAAAAATAAAGCCAAAGCTCTTCTAAGCTGTCATACGCTCTCTGAAAGAACGAGGCCC

AAACACGAAAGTAGCTTTAAATAAATAACCTGATACCACGAAAGCCAAGAAA

>762_Sample_ASV4

CACCGCGGTTATACGAGAGACCCTAGTTGATTAAAGCGGCGTAAAGAGTGGTTATGGAAC

TATTCTTTTAAAGCAGAAAACCTCTCAAACTGTTATACGCACCCAGAGGTCTGAATCCCT

CACACGAAAGTGACTTTATTTATGCCTACCAGAACCCACGAAAGCTGGGACA

>762_Sample_ASV5

CACCGCGGTTATACGAGAGGCCCAAGTTGACAGACACCGGCGTAAAGAGTGGCTAGGGAA

AAATTACTACTAAAGCCGAACACCTTCAGAACTGTCATACGTTCCCGAAGATAAGAAGCC

CCACCACGAAAGTGGCTTTATATTACCCGACCCCACGAAAGCTGAGAAA

>762_Sample_ASV6

CACCGCGGTTATACGAGAGGCCCAAGTTGACAGACACCGGCGTAAAGAGTGGCTAGGGAA

AAATTACTACTAAAGCCGAACACCTTCAGAACTGTCATACGTTCCCGAAGATAAGAAGCC

CCACCACGAAAGTGGCTTTATATTACCCGACCCCACGAAAGCTGTGAAA

>762_Sample_ASV7

CACCGCGGTTATACGAGAGGCCCAAGTTGACAGACACCGGCGTAAAGAGTGGCTAAGGAA

AAATTTCTACTAAAGCCGAACACCTTCAGAACTGTCATACGTTCCCGAAGAAAAGAAGCC

CCACCACGAAAGTGGCTTTATATTACCCGACCCCACGAAAGCTGAGAAA

>762_Sample_ASV8

CACCGCGGTTATACGAGAGGCCCAAGTTGACAGACACCGGCGTAAAGAGTGGCTAAGGAA

AAATTTCTACTAAAGCCGAACACCTTCAGAACTGTCATACGTTCCCGAAGAAAAGAAGCC

CCACCACGAAAGTGGCTTTATATTACCCGACCCCACGAAAGCTGTGAAA

>762_Sample_ASV9

CACCGCGGTTATACGAAAGACCCTAGTTTATAGAAACGGCGTAAAGGGTGGTTAAGGGAC

AAACAAAATAAGGCCAAAGACTTCCTAAGCCGTCATACGCTCCACGAAAGCACGAAGCCC

AAACACGAAAGTAGCCTTAATAACTTCACCTGACCCCACGAAAGCTGGGAGA

>762_Sample_ASV10

CACCGCGGTTAAACGAGAGACCCTAGTTGATCATCACGGCGTAAAGAGTGGTTAGGGGAT

TATCATAAATAAAGCCAAAGACCTCCCAAGCTGTCGCACGCATTCCGGAGGCGCGAAGCC

CACACACGAAAGTAGCTTTAACTATTGACCCTGATGCCACGAAAGCTAAGAAA

>762_Sample_ASV11

CACCGCGGTTATACGAGAGACCCTAGTTGATATATACGGCGTAAAGAGTGGTTTGGGATA

TTTCTCCAAAATAAAGTCGAAGATCTCCCAAGCCGTCACACGCATCCCGGAGACACGAAG

CCCTGATACGAAGGTAGCTTTATTAACACCCCGACGCCACGAAAGCTAAGAAA

>762_Sample_ASV12

CACCGCGGTTATACGAGAGACCCTAGTTGATATATACGGCGTAAAGAGTGGTTTGGGATA

TTTCTCCAAAATAAAGCCGAAGATCTCCCAAGCCGTCACACGCATCCCGGAGACACGAAG

CCCTGATACGAAGGTAGCTTTATTAACACCCCGACGCCACGAAAGCTAAGAAA

>762_Sample_ASV13

CACCGCGGTTATACGAGAGGCCCAAGTTGATAAGCCCGGCGTAAAGAGTGGTTATGGAGA

ATAAAACACTAAAGCTAAAGACCCCCTAGGCTGTTATACGCACCTGGCGGCTCGAACCAC

CTATACGAAAGTAGCTTTACCCCCCTTCCACCAGAATCCACGACAGCTGGGGCA

>762_Sample_ASV14

CACCGCGGTTATACGAGAGGCCCAAGTTGACAGACATCGGCGTAAAGAGTGGTTAAGGAA

ACCCCATTACTAAAGCCGAACGCCCTCAGAACTGTCATACGTTCCCGAAGGTAAGAAGCC

CCACTACGAAAGTGGCTTTAACCACACCCGACCCCACGAAAGCTGTGAAA

>762_Sample_ASV15

CACCGCGGTTATACGAGAGGCCCAAGTTGATAGATATCGGCGTAAAGAGTGGTTAAGGAA

ACCCCGTTACTAAAGCCGAACGCCCTCAGAACTGTCATACGTTCCCGAAGGTAAGAAGCC

CCACTACGAAAGTGGCTTTAACCACACCCGACCCCACGAAAGCTGTGAAA

>762_Sample_ASV16

CACCGCGGTTATACGAGAGGCCCAAGTTGACAGACACCGGCGTAAAGAGTGGTTAAGGAA

ATTCATCAACTAAAGCCGAATGCCCTCAGGACTGTTATACGTTTGCGAAGGTAAGAAGCC

CTACTACGAAAGTGGCTTTATTCCACCTGATTCCACGAAAGCTGCGGAA

>762_Sample_ASV17

CACCGCGGTTATACGAGAGGCCCCAGTTGATATTAGCGGCGTAAAGGGTGGTTAAGGACT

TTAATAACTAAAGCTAAACACTTCCCCGGCTGTTGTACGTAGACCGGAAGCTTGAAGCCC

AGACACGAAAGTAGCTTTATTCCCCCTGACCCCACGAAAGCTGAGAAA

>762_Sample_ASV18

CACCGCGGTTATACGAGAAACCCTAGTTGACAAATACGGCGTAAAGAGTGGTTAAGGGGT

ATCAAAAATAAAGTCAAACATTTTCCTAGTTGTTATAAGCATTTCGAGAACATGAAGCCC

AACCACGAAAGTAACTTTACCTTCACCTGACCCCACGAAAGCTAAGAAA

>762_Sample_ASV19

CACCGCGGTTATACGAAAGACCCCAGTTTATAGACACGGCGTAAAGGGTGGTTATAGGAC

AAACTAACATAGAGCCAAAGACCCCCTAAGCCGTCATACGCTCACGAGGGCTCGAAGCCC

CTATACGAAAGTAGCTCTACCAAAAAATGCTCGACTCCACGAAAGCTGGGACA

>762_Sample_ASV20

CACCGCGGTTATACGAAAGACCCCAGTTTATAGACACGGCGTAAAGGGTGGTTATAGGAC

AAACTAACATAGAGCCAAAGACCCCCTAAGCCGTCATACGCTCACGAGGGCTCGAAGCCC

CTATACGAAAGTAGCTCTACCAAAAAATGCTCGACTCCACGAAAGCTGAGAAA

>762_Sample_ASV21

CACCGCGGTTATACGAGAGACCCTAATTGATAGGTACGGCGTAAAGAGTGGTTTGGGATA

ACCCAAAAATAAAGCTAAAGACCTTCTAAGCCGTCACACGCATACCGAAGGCACGAGGCC

CCAACACGAAAGTAGCTTTACTATTACCCCGACGCCACGAAAGCTAAGAAA

>762_Sample_ASV22

CACCGCGGTTATACGAGAGGCCCTAGTTGATAACTACCGGCGTAAAGAGTGGTTATGGAA

AAATATTTAATAAAGCCGAACACCCCCTCAGCCGTCATACGCACCTGGGAGCACGAAGAC

CTACTGCGAAAGCAGCTTTAATTATGCCTGACCCCACGACAGCTAAGAAA

>762_Sample_ASV23

CACCGCGGTTATACGAGAGGCCCCAGTTGATAAACACGGCGTAAAGAGTGGTTAAGGGCA

CAAAACAAATAAAGTCAAACAACCTCTTAGCTGTTATACGCATTATGAGGATATGAAGCC

CCCCTACGAAAGTAACTTTACCAACCCCTGACCCCACGAAAGCTGAGAAA

>762_Sample_ASV24

CACCGCGGTTATACGAGAGGCCCCAGTTGATAAACACGGCGTAAAGAGTGGTTAAGGGCA

CAAAACAAATAAAGTCAAACAACCTCTTAGCTGTTATACGCATTATGAGGATATGAAGCC

CCCCTACGAAAGTAACTTTACCAACCCCTGACCCCACGAAAGCTAAGAAA

>762_Sample_ASV25

CACCGCGGTTATACGAGAGACCCCAGTTGATAAACACGGCGTAAAGAGTGGTTAAGGGCA

CAAAACAAATAAAGTCAAACAACCTCTTAGCTGTTATACGCATTATGAGGATATGAAGCC

CCCCTACGAAAGTAACTTTACCAACCCCTGACCCCACGAAAGCTGAGAAA

>762_Sample_ASV26

CACCGCGGTTATACGAGAGACCCCAGTTGATAAACACGGCGTAAAGAGTGGTTAAGGGCA

CAAAACAAATAAAGTCAAACAACCTCTTAGCTGTTATACGCATTATGAGGATATGAAGCC

CCCCTACGAAAGTAACTTTACCAACCCCTGACCCCACGAAAGCTAAGAAA

>762_Sample_ASV27

CACCGCGGTTATACGAGAGACCCTAGTTGATATATACGGCGTAAAGAGTGGTTTGGAATA

ACCAAGTAATATAGCCAAAGACCTCCCAAGCCGTCACACGCACCCCGGAGGCACGAAGCC

CAAACACGAAAGTAGCTTTATTAACTTACCGACGCCACGAAAGCTAAGAAA

>762_Sample_ASV28

CACCGCGGTTATACGAGAGACCCTAATTAATAGATACGGCGTAAAGAGTGGTTTAGGACA

AAAATTAATAAAGCCGAAGTTTCTCCTAGCCGTTGTACGCACTCTGAGAACACGAAGCCC

CACTACGAAAGTAGCTTTACCTATTATTTCCTGACCCCACGAAAGCTAAGAAA

>762_Sample_ASV29

CACCGCGGTTATACGAGAGGCCCAAGTTGATAGACATCGGCGTAAAGAGTGGTTAAGGAA

GTCTCAAAACTAAAGCCGAATGCCTTCAAGACTGTTATACGTTTCCGAAGGTAAGAAGCC

CCACTACGAAAGTGGCTTTACTCCACCTGACTCCACGAAAGCTGTGAAA

>762_Sample_ASV30

CACCGCGGTTATACGAGAGACTCCAGTTGATAGCTACGGCGTAAAGAGTGGTTTGGGGCC

CGCCCAAAATAAAGCCAAAGACCTCCCAAGCCGTCAAACGCACCCCGGAGGCACGAAGTC

CTAACGCGAAAGCAACTTTACCTTCCCCGACGCCACGAAAGCTAAGAAA

>762_Sample_ASV31

CACCGCGGTTATACGAAAGACCCTAGTTGATAGATCACGGCGTAAAGGGTGGTTAAGGAG

AACAAAAATAAAGCTAAAAATCCTCTAAGCTGTCATACGCTTTCCGAAGACATGAGACCC

AACCACGAAAGTAACTTTAAACTTTCCTGACACCACGAAAGCCAAGAAA

>782_Sample_ASV1

CACCGCGGTTATACGAGAGGCCCAAGTTGACAGACATCGGCGTAAAGAGTGGTTAAGGAA

AACCTTCAACTAAAGTCGAACGCTCTCAAGACTGTCGTACGTTTCCGAGAGTATGAAGCC

CCACCACGAAAGTGACTTTACCTCACCTGACTCCACGAAAGCTGCGAAA

>782_Sample_ASV2

CACCGCGGTTATACGAAAGACCCTAGTTGATGGGTGCGGCGTAAAGGGTGGTTAGGGACT

ACAAAAAATAGAGCTAAAGATCCTCTAGGTCGTCATACACATCACGAGGAAACGAAGCCC

AAATACGAAAGTAGCTCTAAATATAATCCTGACGCCACGAAAGCCAAGAAA

>782_Sample_ASV3

CACCGCGGTTATACGAAAGACCCTAGTTGATGGGTGCGGCGTAAAGGGTGGTTAGGGACT

ACAAAAAATAGAGCTAAAGATCCTCTAGGTCGTCATACACATCACGAGGAAACGAAGCCC

AAGTACGAAAGTAGCTCTAAATATAATCCTGACGCCACGAAAGCCAAGAAA

>782_Sample_ASV4

CACCGCGGTTATACGAGAGACCCTAGTTGATTAAAGCGGCGTAAAGAGTGGTTATGGAAC

TATTCTTTTAAAGCAGAAAACCTCTCAAACTGTTATACGCACCCAGAGGTCTGAATCCCT

CACACGAAAGTGACTTTATTTATGCCTACCAGAACCCACGAAAGCTGGGACA

>782_Sample_ASV5

CACCGCGGTTATACGAGAGACCCTAGTTGATTAAAGCGGCGTAAAGAGTGGTTATGGAAC

TATTCTTTTAAAGCAGAAAACCTCTCAAACTGTTATACGCACCCAGAGGTCTGAATCCCT

CACACGAAAGTGACTTTATTTATGCCTACCAGAACCCACGAAAGCTATGACA

>782_Sample_ASV6

CACCGCGGTTATACGAAAGACCCTAGTTTATAGGTACGGCGTAAAGGGTGGTTAGGGGAC

AAACAAAATAAAGCCAAAGACCTCCTAAGCCGTCATACGCTCACGGAAGCACGAAGCCCA

AACACGAAAGTAGCTTTACCGAACATGCCCGACTCCACGAAAGCTGGGGAA

>782_Sample_ASV7

CATGGCGATTATACGAGAGGCCCAAGTTGACAGACACTGGCGTAAAGAGTGGCTAAGGAA

AAACTACTACTAAAGCCGAACACCTTCAGAACTGTCATACGTTCCCGAAGATAAGAAGCC

CCACCACGAAAGTGACTTTATATTACCCGACCCCACGAAAGCTGTGAAA

>782_Sample_ASV8

CACCGCGGTTATACGAGAGGCCCAAGTTGACAGACACCGGCGTAAAGAGTGGCTAGGGAA

AAATTACTACTAAAGCCGAACACCTTCAGAACTGTCATACGTTCCCGAAGATAAGAAGCC

CCACCACGAAAGTGACTTTATATTACCCGACCCCACGAAAGCTAAGACA

>782_Sample_ASV9

CACCGCGGTTATACGAGAGGCCCAAGTTGACAGACACCGGCGTAAAGAGTGGCTAGGGAA

AAATTACTACTAAAGCCGAACACCTTCAGAACTGTCATACGTTCCCGAAGATAAGAAGCC

CCACCACGAAAGTGACTTTATATTACCCGACCCCACGAAAGCTGTGAAA

>782_Sample_ASV10

CACCGCGGTTATACGAGAGGCCCAAGTTGACAGACACCGGCGTAAAGAGTGGCTAGGGAA

AAATTACTACTAAAGCCGAACACCCTCAGAACTGTCATACGTTCCCGAAGGTAAGAAGCC

CCACCACGAAAGTGGCTTTATAATACCCGACCCCACGAAAGCTGTGAAA

>782_Sample_ASV11

CACCGCGGTTATACGAGAGGCCCAAGTTGACAGACACCGGCGTAAAGAGTGGCTAGGGAA

AAATTACTACTAAAGCCGAACACCTTCAGAACTGTCATACGTTCCCGAAGATAAGAAGCC

CCACCACGAAAGTGGCTTTATATTACCCGACCCCACGAAAGCTGTGAAA

>782_Sample_ASV12

CACCGCGGTTATACGAGAGGCCCAAGTTGACAGACACCGGCGTAAAGAGTGGCTAAGGAA

AAATTTCTACTAAAGCCGAACACCTTCAGAACTGTCATACGTTCCCGAAGATAAGAAGCC

CCACCACGAAAGTGGCTTTATATTACCCGACACCACGAAAGCTGTGAAA

>782_Sample_ASV13

CACCGCGGTTATACGAGAGGCCCAAGTTGACAGACACCGGCGTAAAGAGTGGCTAAGGAA

AAATTTCTACTAAAGCCGAACACCTTCAGAACTGTCATATGTTCCCGAAGATAAGAAGCC

CCACCACGAAAGTGGCTTTATATTACCCGACACCACGAAAGCTGTGAAA

>782_Sample_ASV14

CACCGCGGTTATACGAAAGACTCTAGTTGACAAACACGGCGTAAAGGGTGGTTAGGATAA

CAACAAAATAAAGCTAAAGACTTACCAAGCCGTCATACGCCCATGTAAGAGCGAAAACCA

CAAACGAAAGTAACTTTATTACACCATCCGAATCCACGAAAGCTAAGAAA

>782_Sample_ASV15

CACCGCGGTTAAACGAGAGACCCTAGTTGATCATCACGGCGTAAAGAGTGGTTAGGGGAT

TATCATAAATAAAGCCAAAGACCTCCCAAGCTGTCGCACGCATTCCGGAGGCGCGAAGCC

CACACACGAAAGTAGCTTTAACTATTGACCCTGATGCCACGAAAGCTAAGAAA

>782_Sample_ASV16

CACCGCGGTTATACGAGAGACCCTAGTTGATATATACGGCGTAAAGAGTGGTTTGGGATA

TTTCTCCAAAATAAAGCCGAAGATCTCCCAAGCCGTCACACGCATCCCGGAGACACGAAG

CCCTGATACGAAGGTAGCTTTATTAACACCCCGACGCCACGAAAGCTAAGAAA

>782_Sample_ASV17

CACCGCGGTTATACGAGAGACCCTAGTTGATATATACGGCGTAAAGAGTGGTTTGGGATA

TTTCTCCAAAATAAAGTCGAAGATCTCCCAAGCCGTCACACGCATCCCGGAGACACGAAG

CCCTGATACGAAGGTAGCTTTATTAATTCCCCGACGCCACGAAAGCTAAGAAA

>782_Sample_ASV18

CACCGCGGTTATACGAGAGACCCTAGTTGATATATACGGCGTAAAGAGTGGTTTGGGATA

TTTCTCCAAAATAAAGTCGAAGATCTCCCAAGCCGTCACACGCATCCCGGAGACACGAAG

CCCTGATACGAAGGTAGCTTTATTAACACCCCGACGCCACGAAAGCTAAGAAA

>782_Sample_ASV19

CACCGCGGTTATACGAAAGACCCTAGTTGATAGATCACGGCGTAAAGGGTGGTTAGGGAA

TACAAAAATAAAGCTAAAAATCCTCTAAGCTGTCATACGCTTCCCGAAGACATGAAACCC

AACCACGAAAGTAGCTTTAAACTCTCCTGACACCACGAAAGCCAAGGAA

>782_Sample_ASV20

CACCGCGGTTATACGAGAGGCCCAAGTTGATAAGCCCGGCGTAAAGAGTGGTTATGGAGA

ATAAAACACTAAAGCTAAAGACCCCCTAGGCTGTTATACGCACCTGGCGGCTCGAACCAC

CTATACGAAAGTAGCTTTACCCCCCTTCCACCAGAATCCACGACAGCTGGGGCA

>782_Sample_ASV21

CACCGCGGTTATACGAGAGGCCCAAGTTGACAGACATCGGCGTAAAGAGTGGTTAAGGAA

ACCCCATTACTAAAGCCGAACGCCCTCAGAACTGTCATACGTTCCCGAAGGTAAGAAGCC

CCACTACGAAAGTGGCTTTAACCACACCCGACCCCACGAAAGCTGTGAAA

>782_Sample_ASV22

CACCGCGGTTATACGAGAGGCCCAAGTTGATAGATATCGGCGTAAAGAGTGGTTAAGGAA

ACCCCGTTACTAAAGCCGAACGCCCTCAGAACTGTCATACGTTCCCGAAGGTAAGAAGCC

CCACTACGAAAGTGGCTTTAACCACACCCGACCCCACGAAAGCTGTGAAA

>782_Sample_ASV23

CACCGCGGTTATACGAGAGGCCCAAGTTGACAGACACCGGCGTAAAGAGTGGTTAAGGAA

ATTCATCAACTAAAGCCGAATGCCCTCAGGACTGTTATACGTTTGCGAAGGTAAGAAGCC

CTACTACGAAAGTGGCTTTATTCCACCTGATTCCACGAAAGCTGCGGAA

>782_Sample_ASV24

CACCGCGGTTATACGAGAGACCCCAGTTGATAGCTACGGCGTAAAGAGTGGTTAAGGACC

CCATTTAATAAAGCCAAAGACCCCCCAAGCCGTCATACGCACACGAGGGCGCGAAGCTCG

CACACGAAAGTAGCTCTAATTTACCCGACGCCACGAAAGCTAAGGAA

>782_Sample_ASV25

CACCGCGGTTATACGAGAGGCCCTAGTTGATAAATACGGCGTAAAGAGTGGTTAGAGGAC

TCCCATAATAAAGCCAAAGACCCCCCGGGCCGTCACACGCATACGAGGGCACGAAGTTCA

CACACGAAAGTAGCTTTAAATTACCCGACGCCACGAAAGCTAAGAAA

>782_Sample_ASV26

GACCGCGGTTATACGAGAGACTCTAGTTGATAGATACGGCGTAAAGAGTGGTTTGGGATA

AACTATAAATAAAGCCAAAGACCTTCCAAGCTGTTAAACGCATACCGAAGGCACGAAGCC

CCAACACGAAAGTAGCTTTACTACTACCCCCGATGCCACGAAAGCTAAGACA

>782_Sample_ASV27

CACCGCGGTTATACGAGAGACCCTAGTTGATAAATACGGCGTAAAGAGTGGTTATGGGAA

AAACAAATAAAGTCAAACAACCTCTTAGCTGTTATACGCATTATGAGAGCATGAAGCCCC

CTCACGAAAGTAACTTTAATACCTCCTGACCCCACGAAAGCTAAGAAA

>782_Sample_ASV28

CACCGCGGTTATACGAAAGACCCTAGTTGATGGATCACGGCGTAAAGGGTGGTTAAGGGA

TATAAAAATAAAGCTAAAAATCCTCTTAGCTGTCATACGCTTTCCGAAGACATGAGGCCC

AATTACGAAAGTAGCTTTAAACCACCCTGACACCACGAAAGCCAAGAAA

>782_Sample_ASV29

CACCGCGGTCATACGATTAACCCAAGTTAATAGGCACACGGCGTAAAGCGTGTTTAAGCA

CTATGCCAAATAAAGTTAAACTCCAATTAAGCTGTAAAAAGCCATAATCAAGACGAAAAT

AAATAACGAAAGTAACTTTACAACCGCTGAAACACGATAGCTAAGATC

>782_Sample_ASV30

CACCGCGGTTATACGAAAGACCCTAGTTTATAGACACGGCGTAAAGGGTGGTTCGGGACT

AAACAAAATAAAGCCAAAGACCTCCTAAGCCGTCATACGCTCACGGAAGCACGAAGCCCA

AGTACGAAAGTAGCTTTACAAAACATGCCTGACTCCACGAAAGCTAGGAAA

>782_Sample_ASV31

CACCGCGGTTATACGAAAGACCCTAGTTGATAGGCACGGCGTAAAGGGTGGTTAGGGATA

TATCAAAAATAAAGTTAAAGAGCCTCTAAGCTGTCGCACGCATCCCGAGAGCTCGAGACC

CAGACACGAAAGTAACTTTAAAATACCTCACCTGACCCCACGAAAGCTAAGAAA

>782_Sample_ASV32

CACCGCGGTTATACGAAAGACCCTAGTTGATAGCCACGGCGTAAAGGGTGGTTAAGGTAT

ACAACAAATAAAGCTAAATAGCCTCTAGGCCGTCGCACGCATTCCGAGAGCTCGAAACCC

AAAAACGAAAGTAGCTTTAAAATAAACAGACCTGACCCCACGAAAGCTAAGAAA

>782_Sample_ASV33

CACCGCGGTTATACGAGAGGCCCAAGTTGATACTCCACGGCGTAAAGAGTGGTTAGAAAA

GACCTACTACTAAAGCCGAACGCCTTCAAAGCTGTTATACGCATCCGAAGGTGAGAAGCC

CACCCACGAAAGTGGCTTTACAACCTTGACTCCACGAAAGCTATGACA

>782_Sample_ASV34

CACCGCGGTTATACGAGAGACCCCAGTTGATAAACACGGCGTAAAGAGTGGTTAAGGGCA

CAAAACAAATAAAGTCAAACAACCTCTTAGCTGTTATACGCATTATGAGGATATGAAGCC

CCCCTACGAAAGTAACTTTACCAACCCCTGACCCCACGAAAGCTAAGAAA

>782_Sample_ASV35

CACCGCGGTTATACGAAAGACCCTAGTTTATAGGCACGGCGTAAAGGGTGGTTAGGGGAT

AAACAAAATAAAGCCAAAGACCTTCTAAGTCGTCATACACTCATGAAGGCACGAAGCCCA

AATACGAAAGTAACTTTACAACTTAGACCCGACTCCACGAAAGCTGGGGGA

>782_Sample_ASV36

CACCGCGGTTATACGAAAGACCCTAGTTTATAGGCACGGCGTAAAGGGTGGTTAGGGGAT

AAACAAAATAAAGCCAAAGACCTTCTAAGTCGTCATACACTCATGAAGGCACGAAGCCCA

AATACGAAAGTAACTTTACAACTTAGACCCGACTCCACGAAAGCTAAGACA

>782_Sample_ASV37

CACCGCGGTTATACGAAAGACCCTAGTTGATAGATCACGGCGTAAAGGGTGGTTAAGGAA

AACAAAAATAAAGCTAAAAATCCTCTAAGCTGTCATACGCTTTCCGAAGACATGAGATCC

AACCACGAAAGTAGCTTTAAAATTTCCTGACACCACGAAAGCCAAGGAA

>782_Sample_ASV38

CACCGCGGTTATACGAAAGACCCTAGTTGATAGATCACGGCGTAAAGGGTGGTTAAGGAG

AACAAAAATAAAGCTAAAAATCTTCTAAGCTGTCATACGCTTTCCGAAGACATGAGACCC

AACCACGAAAGTAGCTTTAAACTTTCCTGACACCACGAAAGCCAAGAAA

>784_Sample_ASV1

CACCGCGGTTATACGAAAGACCCTAGTTGATGGGTGCGGCGTAAAGGGTGGTTAGGGACT

ACAAAAAATAGAGCTAAAGATCCTCTAGGTCGTCATACACATCACGAGGAAACGAAGCCC

AAATACGAAAGTAGCTCTAAATATAATCCTGACGCCACGAAAGCCAAGAAA

>784_Sample_ASV2

CACCGCGGTTATACGAGAGACCCTAGTTGATTAAAGCGGCGTAAAGAGTGGTTATGGAAC

TATTCTTTTAAAGCAGAAAACCTCTCAAACTGTTATACGCACCCAGAGGTCTGAATCCCT

CACACGAAAGTGACTTTATTTATGCCTACCAGAACCCACGAAAGCTGGGACA

>784_Sample_ASV3

CACCGCGGTTATACGAGAGGCCCAAGTTGACAGACACCGGCGTAAAGAGTGGCTAGGGAA

AAATTACTACTAAAGCCGAACACCTTCAGAACTGTCATACGTTCCCGAAGATAAGAAGCC

CCACCACGAAAGTGGCTTTATATTACCCGACCCCACGAAAGCTGTGAAA

>784_Sample_ASV4

CACCGAGGTTATACGAGAGGCCCAAGTTGACAGACACCGGCGTAAAGAGTAGCTAGGGAA

AAATTACTACTAAAGCCGAACACCTTCAGAACTGTAATACGTTCCCGAAGATAAGAAGCC

CCACCACGAAAGTGGCTTTATATTACCCGACCCCACGAAGGCTGTGAAA

>784_Sample_ASV5

CACCGCGGTTATACGAGAGGCCCAAGTTGACAGACACCGGCGTAAAGAGTGGCTAAGGAA

AAATTTCTACTAAAGCCGAACACCTTCAGAACTGTCATACGTTCCCGAAGAAAAGAAGCC

CCACCACGAAAGTGGCTTTATATTACCCGACCCCACGAAAGCTGTGAAA

>784_Sample_ASV6

CACCGCGGTTATACGAGAGACCCAAGTTGACAGACACCGGCGTAAAGAGTGGCTAAGGAA

AAATTTCTACTAAAGCCGAACACCTTCAGAACTGTCATACGTTCCCGAAGAAAATAAGCC

CCACCACGAAAGTGGCTTTATATTACCCGACCCCACGAAAGCTGTGAAA

>784_Sample_ASV7

CACCGCGGTTAAACGAGAGACCCTAGTTGATCATCACGGCGTAAAGAGTGGTTAGGGGAT

TATCATAAATAAAGCCAAAGACCTCCCAAGCTGTCGCACGCATTCCGGAGGCGCGAAGCC

CACACACGAAAGTAGCTTTAACTATTGACCCTGATGCCACGAAAGCTAAGAAA

>784_Sample_ASV8

CGCCGCGGTTAAACGAGAGGCCCTAGTTGATGGAACAACGGCGTAAAGGGTGGTTAAGGA

TAGTGAGACAATAAAGTCGAATGGCCCTTTGGCTGTCATACGCTTCTGGGAGCCTGAAGC

CCACTATACGAAAGTAGCTTTAACAACGTCCACCTGACCCCACGAAAACTGAGAAA

>784_Sample_ASV9

CACCGCGGTTATACGAAAGACCCTAGTTGATAGATCACGGCGTAAAGGGTGGTTAGGGAA

TACAAAAATAAAGCTAAAAATCCTCTAAGCTGTCATACGCTTCCCGAAGACATGAAACCC

AACCACGAAAGTAGCTTTAAACTCTCCTGACACCACGAAAGCCAAGGAA

>784_Sample_ASV10

CACCGCGGTTATACGAGAGGCCCAAGTTGATAAGCCCGGCGTAAAGAGTGGTTATGGAGA

ATAAAACACTAAAGCTAAAGACCCCCTAGGCTGTTATACGCACCTGGCGGCTCGAACCAC

CTATACGAAAGTAGCTTTACCCCCCTTCCACCAGAATCCACGACAGCTGGGGCA

>784_Sample_ASV11

CACCGCGGTTATACGAGAGGCCCAAGTTGACAGACATCGGCGTAAAGAGTGGTTAAGGAA

ACCCCATTACTAAAGCCGAACGCCCTCAGAACTGTCATACGTTCCCGAAGGTAAGAAGCC

CCACTACGAAAGTGGCTTTAACCACACCCGACCCCACGAAAGCTGTGAAA

>784_Sample_ASV12

CACCGCGGTTAAACGAGAGGCCCTAGTTGATATTACAACGGCGTAAAGGGTGGTTAAGGA

AAGCAAGCAATAAAGCCGAATGGCCCTTTGGCTGTCATACGCTTCTAGGTGTCCGAAGCC

CAATATACGAAAGTAGCTTTAGAAAAGCCCACCTGACACCACGAAAGCTGAGGAA

>784_Sample_ASV13

CACCGCGGTTATACGAGAGGCCCAAGTTGACAGACACCGGCGTAAAGAGTGGTTAAGGAA

ATTCATCAACTAAAGCCGAATGCCCTCAGGACTGTTATACGTTTGCGAAGGTAAGAAGCC

CTACTACGAAAGTGGCTTTATTCCACCTGATTCCACGAAAGCTGCGGAA

>784_Sample_ASV14

CACCGCGGTTATACGAGAGGCCCCAGTTGATATTAGCGGCGTAAAGGGTGGTTAAGGACT

TTAATAACTAAAGCTAAACACTTCCCCGGCTGTTGTACGTAGACCGGAAGCTTGAAGCCC

AGACACGAAAGTAGCTTTATTCCCCCTGACCCCACGAAAGCTGAGAAA

>784_Sample_ASV15

CACCGCGGTCACACGATTAACCCAAGTCAATAGAAGCCGGCGTAAAGAGTGTTTTAGATC

ACCCCCTCCCCAATAAAGCTAAAACTCACCTGAGTTGTAAAAAACTCCAGTTGACACAAA

ATAGACTACGAAAGTGGCTTTAACATATCTGAACACACAATAGCTAAGACC

>784_Sample_ASV16

CACCGCGGTTATACGAGAGGCCCTAGTTGATAAATACGGCGTAAAGAGTGGTTAGAGGAC

TCCCATAATAAAGCCAAAGACCCTCCGGGCCGTCACACGCATACGAGGGCACGAAGTTCA

CACACGAAAGTAGCTTTAAATTACCCGACGCCACGAAAGCTAAGAAA

>784_Sample_ASV17

CACCGCGGTTATACGAAAGACCCTAGTTTATAAGTACGGCGTAAAGGGTGGTTAGGGGAC

AAACAAAATAAAGCCAAAGACCTCCTAAGCCGTCATACGCTTACGGAGGCACGAAGCCCA

AACACGAAAGTAGCTTTACCGAACATGCCCGACTCCACGAAAGCTGGGAAA

>784_Sample_ASV18

CACCGCGGTTATACGAGAGACCCTAGTTGATAAATACGGCGTAAAGAGTGGTTATGGGAA

AAACAAATAAAGTCAAACAACCTCTTAGCTGTTATACGCATTATGAGAGCATGAAGCCCC

CTCACGAAAGTAACTTTAATACCTCCTGACCCCACGAAAGCTAAGAAA

>784_Sample_ASV19

CACCGCGGTTATACGAGAGGCTCAAGTTGATAAACCCCGGCGTAAAGAGTGGTTAAGGAA

AAATAGAAACTAAAGCCGAATGCTTTCAAGACTGTTATACGTTCCCGAGAGTAAGAAGAC

CAATTACGAAAGTGGCTTTACTTTACCTGACCCCACGAAAGCTACGACA

>784_Sample_ASV20

CACCGCGGTTATACGAGAGACCCTAATTGATAGACACGGCGTAAAGAGTGGTTTGGGAAA

GCCCAAAAATAAAGCTAAAGACCTTCTAAGCCGTCACACGCACGCCGAAGGCACGAAGCC

CTGACACGAAAGTAGCTTTACTATTACCCCTGACGCCACGAAAGCTAAGAAA

>784_Sample_ASV21

CACCGCGGTTATACGAGAGACCCTAATTGATAGACACGGCGTAAAGAGTGGTTTGGGAAA

GCCCAAAAATAAAGCTAAAGACCTTCTAAGCCGTCACACGCACGCCGAAGGCACGAAGCC

CTGACACGAAAGTAGCTTTACTATTACCCCCGACGCCACGAAAGCTAAGAAA

>784_Sample_ASV22

CACCGCGGTTATACGAGAGACCCTAATTGATAGACACGGCGTAAAGAGTGGTTTGGGAAA

GCCCAAAAATAAAGCTAAAGACCTTCTAAGCCGTCACACGCACGCCGAAGGCACGAAGCC

CTGACACGAAGGTAGCTTTACTATTACCCCCGACGCCACGAAAGCTAAGAAA

>784_Sample_ASV23

AACCGCGGTTATACGAGAGACCCTAATTGATAGCTCACGGCGTAAAGGGTGGTTTGGGGA

AACCTCAAAATAAAGCTAAAGACCTTCTAAGCCGTTACACGCATACCGAAGGCACGAGGC

CCTAACACGAAAGTAGCTTTACTATTACCCCTGACGCCACGACAGCTGGGGCA

>784_Sample_ASV24

AACCGCGGTTATACGAGAGACCCTAATTGATAGCTCACGGCGTAAAGGGTGGTTTGGGGA

AACCTCAAAATAAAGCTAAAGACCTTCTAAGCCGTTACACGCATACCGAAGGCACGAGGC

CCTAACACGAAAGTAGCTTTACTATTACCCCTGACGCCACGAAAGCTAAGAAA

>784_Sample_ASV25

CACCGCGGTTATACGAAAGACCCTAGTTGATGGATCACGGCGTAAAGGGTGGTTAAGGGA

TATAAAAATAAAGCTAAAAATCCTCTTAGCTGTCATACGCTTTCCGAAGACATGAGGCCC

AATTACGAAAGTAGCTTTAAACCACCCTGACACCACGAAAGCCAAGAAA

>784_Sample_ASV26

CACCGCGGTTATACGAAAGACCCTAGTTTATAGATGCGGCGTAAAGGGTGGTTAAGGAAA

ACCTAAAATAATGCTAAAGACTCTCAAAGCTGTTATACGCTTCATGAGCACACGAAATCC

AAATACGAAAGTTGCCTTAATAAACTTTGCCTGACCCCACGAAAGCTGAAAAA

>784_Sample_ASV27

CACCGCGGTTATACGAAAGACCCCAGTTTATAGATGCGGCGTAAAGGGTGGTTAAGAGAA

AATCAAAATAAGGCCAAAGATCCCCTAAGCAGTCATACGCTCCACGAGGACACGAAACCC

AAATACGAAAGTTGCCTTAATAAACTTCACCTGATCCCACGAAAGCTGAAAAA

>784_Sample_ASV28

CACCGCGGTTATACGAAAGGCTCTAGTTTATAAATACGGCGTAAAGGGTGGTTAAGGAAA

AATTAAAATAATGCTAAAGACCCCCTAAGCAGTTATACGCTTCACGGAGGCACGAAACCC

AAATACGAAAGTTGCCTTAATAAACTTTACCTGACCCCACGAAAGCTGAGAAA

>784_Sample_ASV29

CACCGCGGTTATACGAAAGGCTCTAGTTTATAAATACGGCGTAAAGGGTGGTTAAGGAAA

AATTAAAATAATGCTAAAGACCCCCTAAGCAGTTATACGCTTCACGGAGGCACGAAACCC

AAATACGAAAGTTGCCTTAATAAACTTTACCTGACCCCACGAAAGCTGAAAAA

>784_Sample_ASV30

CACCGCGGTTAAACGAGAGGCCCTAGTTGATAGTACAACGGCGTAAAGGGTGGTTAAGGA

TAGTGAAATAATAAAGTCGAATGGCCCTTTGGCTGTCATACGCTTCTAGGAGTCCGAAGC

CCAATATACGAAAGTAACTTTAAGAGCGCCCACCTGACCCCACGAAAACTGAGAAA

>784_Sample_ASV31

CACCGCGGTTATACGAAAGACCCTAGTTGATAGGCACGGCGTAAAGGGTGGTTAGGGATA

TATCAAAAATAAAGTTAAAGAGCCTCTAAGCTGTCGCACGCATCCCGAGAGCTCGAGACC

CAGACACGAAAGTAACTTTAAAATACCTCACCTGACCCCACGAAAGCTAAGAAA

>784_Sample_ASV32

CACCGCGGTTATACGAAAGACCCTAGTTGATAGGCACGGCGTAAAGGGTGGTTAGGGATA

TATTAAAAATAAAGTTAAAGAGCCTCTAAGCTGTCGCACGCATCCCGAGAGCTCGAGACC

CAGACACGAAAGTAACTTTAAAATACCTCACCTGACCCCACGAAAGCTAAGAAA

>784_Sample_ASV33

CACCGCGGTTATACGAAAGACCCTAGTTGATAGGCACGGCGTAAAGGGTGGTTAGGGATA

CACCAAAAATAAAGTTAAAGAGCCTCTAAGCTGTCGCACGCATCCCGAGAGCTCGAGACC

CAGACACGAAAGTAACTTTAAAATACCTCACCTGACCCCACGAAAGCTAAGAAA

>784_Sample_ASV34

CACCGCGGTTATACGAAAGACCCTAGTTGATAGCCACGGCGTAAAGGGTGGTTAAGGTAT

ACAACAAATAAAGCTAAATAGCCTCTAGGCCGTCGCACGCATTCCGAGAGCTCGAAACCC

AAAAACGAAAGTAGCTTTAAAATAAACAGACCTGACCCCACGAAAGCTAAGAAA

>784_Sample_ASV35

CACCGCGGTTATACGAGAGGCCCAAGTTGATACTCCACGGCGTAAAGAGTGGTTAGAAAA

GACCTACTACTAAAGCCGAACGCCTTCAAAGCTGTTATACGCATCCGAAGGTGAGAAGCC

CACCCACGAAAGTGGCTTTACAACCTTGACTCCACGAAAGCTATGACA

>784_Sample_ASV36

CACCGCGGTTATACGAGAGACCCCAGTTGATAAACACGGCGTAAAGAGTGGTTAAGGGCA

CAAAACAAATAAAGTCAAACAACCTCTTAGCTGTTATACGCATTATGAGGATATGAAGCC

CCCCTACGAAAGTAACTTTACCAACCCCTGACCCCACGAAAGCTAAGAAA

>784_Sample_ASV37

CACCGCGGTTATACGAGAGACCCTAGTTGATATATACGGCGTAAAGAGTGGTTTGGAATA

ACCAAGTAATATAGCCAAAGACCTCCCAAGCCGTCACACGCACCCCGGAGGCACGAAGCC

CAAACACGAAAGTAGCTTTATTAACTTACCGACGCCACGAAAGCTAAGAAA

>784_Sample_ASV38

CACCGCGGTTATACGAAAGACCCTAGTTTATAGGCACGGCGTAAAGGGTGGTTAGGGGAT

AAACAAAATAAAGCCAAAGACCTTCTAAGTCGTCATACACTCATGAAGGCACGAAGCCCA

AATACGAAAGTAACTTTACAACTTAGACCCGACTCCACGAAAGCTGGGGGA

>784_Sample_ASV39

CACCGCGGTTATACGAAAGACCCTAGTTTATAGGCACGGCGTAAAGGGTGGTTAGGGGAT

AAACAAAATAAAGCCAAAGACCTTCTAAGTCGTCATACACTCATGAAGGCACGAAGCCCA

AATACGAAAGTAACTTTACAACTTAGACCCGACGCCACGAAAGCTAAGAAA

>784_Sample_ASV40

CACCGCGGTTATACGAAAGACCCTAGTTTATAGGCACGGCGTAAAGGGTGGTTAGGGGAT

AAACAAAATAAAGCCAAAGACCTTCTAAGTCGTCATACACTCATGAAGGCACGAAGCCCA

AATACGAAAGTAACTTTACAACTTAGACCCGACTCCACGAAAGCTAAGAAA

>784_Sample_ASV41

CACCGCGGTTATACGAAAGACCCTAGTTTATAGGCACGGCGTAAAGGGTGGTTAGGGGAT

AAACAAAATAAAGCCAAAGACCTTCTAAGTCGTCATACACTCATGAAGGCACGAAGCCCA

AATACGAAAGTAACTTTACAACTTAGACCCGACCCCACGAAAGCTGTGAAA

>784_Sample_ASV42

CACCGCGGTTAAACGAGAGGCCCTAGTTGATGGTGCAACGGCGTAAAGGGTGGTTAAGGA

AAGCAAGCAATAAAGCCGAATGGCCCTTTGGCTGTCATACGCTTCTAGGTGTCCGAAGCC

CAATATACGAAAGTAGCTTTAGAAAAGCCCACCTGACACCACGAAAGCTGAGGAA

>784_Sample_ASV43

CACCGCGGTTATACGAGAGGCCCAAGTTGATAGACATCGGCGTAAAGAGTGGTTAAGGAA

GTCTCAAAACTAAAGCCGAATGCCTTCAAGACTGTTATACGTTTCCGAAGGTAAGAAGCC

CCACTACGAAAGTGGCTTTACTCCACCTGACTCCACGAAAGCTGTGAAA

>784_Sample_ASV44

CACCGCGGTTATACGAGAGGCCCAAGTTGATAGACATCGGCGTAAAGAGTGGTTAAGGAA

GTCTCAAAACTAAAGCCGAATGCCTTCAAGACTGTTATACGTTTCCGAAGGTAAGAAGCC

CCACTACGAAAGTGGCTTTATTCCACCTGACCCCACGAAAGCTGTGAAA

>784_Sample_ASV45

CACCGCGGTTATACGAGAGGCCAAAGTTGACAGATATCGGCGTAAAGAGTGGTTAAGGAA

ATCTTAAAACTAAAGCCGAATGCCTTCAAGACTGTTATACGTTTCCGAAGGTAAGAAGCC

CCACTACGAAAGTGGCTTTACTCCACCTGACTCCACGAAAGCTGTGAAA

>784_Sample_ASV46

CACCGCGGTTATACGAGAGGCCCAAGTTGATAGACATCGGCGTAAAGAGTGGTTAAGGAA

GTCTCAAAACTAAAGCCGAATGCCTTCAAGACTGTTATACGTTTACGAAGGTAAGAAGCC

CCACTACGAAAGTGGCTTTATTCCACCTGACCCCACGAAAGCTGTGAAA

>784_Sample_ASV47

CACCGCGGTTATACGAGAGGCCCAAGTTGACAGATATCGGCGTAAAGAGTGGTTAAGGAA

ATCTTAAAACTAAAGCCGAATGCCTTCAAGACTGTTATACGTTTACGAAGGTAAGAAGCC

CCACTACGAAAGTGGCTTTACTCCACCTGACTCCACGAAAGCTGTGAAA

>784_Sample_ASV48

CACCGCGGTTATACGAGAGGCCCAAGTTGACAGATATCGGCGTAAAGAGTGGTTAAGGAA

ATCTTAAAACTAAAGCCGAATGCCTTCAAGACTGTTATACGTTTCCGAAGGTAAGAAGCC

CCACTACGAAAGTGGCTTTACTCCACCTGACTCCACGAAAGCTGTGAAA

>784_Sample_ASV49

CACCGCGGTTATACGAGAGGCCCAAGTTGATAGACATCGGCGTAAAGAGTGGTTAAGGAA

ATCTTAAAACTAAAGCCGAATGCCTTCAAGACTGTTATACGTTTACGAAGGTAAGAAGCC

CCACTACGAAAGTGGCTTTATTCCACCTGACCCCACGAAAGCTGTGAAA

>784_Sample_ASV50

CACCGCGGTTATACGAGAGGCCAAAGTTGACAGATATCGGCGTAAAGAGTGGTTAAGGAA

GTCTCAAAACTAAAGCCGAATGCCTTCAAGACTGTTATACGTTTCCGAAGGTAAGAAGCC

CCACTACGAAAGTGGCTTTACTCCACCTGACTCCACGAAAGCTGTGAAA

>784_Sample_ASV51

CACCGCGGTTATACGAGAGACTCCAGTTGATAGCTACGGCGTAAAGAGTGGTTTGGGGCC

CGCCCAAAATAAAGCCAAAGACCTCCCAAGCCGTCAAACGCACCCCGGAGGCACGAAGTC

CTAACGCGAAAGCAACTTTACCTTCCCCGACGCCACGAAAGCTAAGAAA

>784_Sample_ASV52

CACCGCGGTTATACGAAAGACCCTAGTTGATAGACCACGGCGTAAAGGGTGGTTAAGGAA

CACAAAAATAAAGCTAAAGATCCTCTAAGCTGTCATACGCTTCCCGAAGACATGAAACCC

AATCACGAAAGTAGCTTTAAACTGTCCTGACACCACGAAAGCCAAGAAA

>784_Sample_ASV53

CACCGCGGTTATACGAGAGACCCTAATTGATAGACTACGGCGTAAAGAGTGGTTTAGAAC

AAAAATTAATAAAGCCAAAGATCCTCCTGGCCGTCGCACGCATTTCGAGGATACGAAGCC

CCACTACGAAAGTAGCTTTACTACAACTTTTCTGACCCCACGAAAGCTAAGAAA

>784_Sample_ASV54

CACCGCGGTTATACGAAAGACCCTAGTTGATAGATCACGGCGTAAAGGGTGGTTAAGGAA

AACAAAAATAAAGCTAAAAATCCTCTAAGCTGTCATACGCTTTCCGAAGACATGAGATCC

AACCACGAAAGTAGCTTTAAAATTTCCTGACACCACGAAAGCCAAGGAA

>790_Sample_ASV1

CACCGCGGTTATACGAGAGACCCTAGTTGATTAAAGCGGCGTAAAGAGTGGTTATGGAAC

TATTCTTTTAAAGCAGAAAACCTCTCAAACTGTTATACGCACCCAGAGGTCTGAATCCCT

CACACGAAAGTGACTTTATTTATGCCTACCAGAACCCACGAAAGCTGGGACA

>790_Sample_ASV2

CACCGCGGTTATACGAGAGACCCTAGTTGATTAAAGCGGCGTAAAGAGTGGTTATGGAAC

TATTCTTTTAAAGCAGAAAACCTCTCAAACTGTTATACGCACCCAGAGGTCTGAATCCCT

CACACGAAAGTGACTTTATTTATGCCTACCAGAACCCACGAAAGCTGGGACA

>790_Sample_ASV3

CACCGCGGTTATACGAAAGACCCTAGTTTATAGGTACGGCGTAAAGGGTGGTTAGGGGAC

AAACAAAATAAAGCCAAAGACCTCCTAAGCCGTCATACGCTCACGGAAGCACGAAGCCCA

AACACGAAAGTAGCTTTACCGAACATGCCCGACTCCACGAAAGCTGGGGAA

>790_Sample_ASV4

CACCGCGGTTATACGAAAGACCCTAGTTTATAGGTACGGCGTAAAGGGTGGTTAGGGGAC

AAACAAAATAAAGCCAAAGACCTCCTAAGCCGTCATACGCTCACGGAAGCACGAAGCCCA

AACACGAAAGTAGCTTTACCGAACATGCCCGACTCCACGAAAGCTGGGGAA

>790_Sample_ASV5

CACCGCGGTTATACGAGAGGCCCAAGTTGACAGACACCGGCGTAAAGAGTGGCTAGGGAA

AAATTACTACTAAAGCCGAACACCTTCAGAACTGTCATACGTTCCCGAAGATAAGAAGCC

CCACCACGAAAGTGACTTTATATTACCCGACCCCACGAAAGCTGTGAAA

>790_Sample_ASV6

CACCGCGGTTATACGAGAGGCCCAAGTTGACAGACACCGGCGTAAAGAGTGGCTAGGGAA

AAATTACTACTAAAGCCGAACACCTTCAGAACTGTCATACGTTCCCGAAGATAAGAAGCC

CCACCACGAAAGTGACTTTATATTACCCGACCCCACGAAAGCTGTGAAA

>790_Sample_ASV7

CACCGCGGTTATACGAGAGGCCCAAGTTGACAGACACCGGCGTAAAGAGTGGCTAGGGAA

AAATTACTACTAAAGCCGAACACCTTCAGAACTGTCATACGTTCCCGAAGATAAGAAGCC

CCACCACGAAAGTGACTTTATATTACCCGACCCCACGAAAGCTAAGAAA

>790_Sample_ASV8

CACCGCGGTTATACGAGAGGCCCAAGTTGACAGACACCGGCGTAAAGAGTGGCTAGGGAA

AAATTACTACTAAAGCCGAACACCTTCAGAACTGTCATACGTTCCCGAAGATAAGAAGCC

CCACCACGAAAGTGACTTTATATTACCCGACCCCACGAAAGCTAAGAAA

>790_Sample_ASV9

CACCGCGGTTATACGAGAGGCCCAAGTTGACAGACACCGGCGTAAAGAGTGGCTAGGGAA

AAATTACTACTAAAGCCGAACACCTTCAGAACTGTCATACGTTCCCGAAGATAAGAAGCC

CCACCACGAAAGTGGCTTTATATTACCCGACCCCACGAAAGCTGTGAAA

>790_Sample_ASV10

CACCGCGGTTATACGAGAGGCCCAAGTTGACAGACACCGGCGTAAAGAGTGGCTAGGGAA

AAATTACTACTAAAGCCGAACACCTTCAGAACTGTCATACGTTCCCGAAGATAAGAAGCC

CCACCACGAAAGTGGCTTTATATTACCCGACCCCACGAAAGCTGTGAAA

>790_Sample_ASV11

CACCGCGGTTATACGAGAGACCCAAGTTGACAGACACCGGCGTAAAGAGTGGCTAAGGAA

AAATTTCTACTAAAGCCGAACACCTTCAGAACTGTCATACGTTCCCGAAGAAAATAAGCC

CCACCACGAAAGTGGCTTTATATTACCCGACCCCACGAAAGCTGTGAAA

>790_Sample_ASV12

CACCGCGGTTATACGAGAGGCCCAAGTTGACAGACACCGGCGTAAAGAGTGGCTAAGGAA

AAATTTCTACTAAAGCCGAACACCTTCAGAACTGTCATACGTTCCCGAAGAAAAGAAGCC

CCACCACGAAAGTGGCTTTATATTACCCGACCCCACGAAAGCTGTGAAA

>790_Sample_ASV13

CACCGCGGTTATACGAGAGACCCAAGTTGACAGACACCGGCGTAAAGAGTGGCTAAGGAA

AAATTTCTACTAAAGCCGAACACCTTCAGAACTGTCATACGTTCCCGAAGAAAATAAGCC

CCACCACGAAAGTGGCTTTATATTACCCGACCCCACGAAAGCTGTGAAA

>790_Sample_ASV14

CACCGCGGTTATACGAGAGGCCCAAGTTGACAGACACCGGCGTAAAGAGTGGCTAAGGAA

AAATTTCTACTAAAGCCGAACACCTTCAGAACTGTCATACGTTCCCGAAGAAAAGAAGCC

CCACCACGAAAGTGGCTTTATATTACCCGACCCCACGAAAGCTGTGAAA

>790_Sample_ASV15

CACCGCGGTTATACGAGAGACCCTAGTTGATATATACGGCGTAAAGAGTGGTTTGGGGTT

ATGCCTCCAAAATAAAGCCGAAGATCTCCCAAGCCGTCACACGCATCCCGGAGACACGAA

GCCCTAATACGAAGGTAGCTTTATTAGTACCCCGACGCCACGAAAGCTAAGAAA

>790_Sample_ASV16

CACCGCGGTTATACGAGAGACCCTAGTTGATATATACGGCGTAAAGAGTGGTTTGGGGTT

ATGCCTCCAAAATAAAGCCGAAGATCTCCCAAGCCGTCACACGCATCCCGGAGACACGAA

GCCCTAATACGAAGGTAGCTTTATTAATTCCCCGACGCCACGAAAGCTAAGAAA

>790_Sample_ASV17

CACCGCGGTTATACGAGAGACCCTAGTTGATATATACGGCGTAAAGAGTGGTTTGGGGTT

ATGCCTCCAAAATAAAGCCGAAGATCTCCCAAGCCGTCACACGCATCCCGGAGACACGAA

GCCCTAATACGAAGGTAGCTTTATTAGTACCCCGACGCCACGAAAGCTAAGAAA

>790_Sample_ASV18

CACCGCGGTTATACGAGAGACCCTAGTTGATATATACGGCGTAAAGAGTGGTTTGGGGTT

ATGCCTCCAAAATAAAGCCGAAGATCTCCCAAGCCGTCACACGCATCCCGGAGACACGAA

GCCCTAATACGAAGGTAGCTTTATTAATTCCCCGACGCCACGAAAGCTAAGAAA

>790_Sample_ASV19

CACCGCGGTTATACGAGAGGCCCAAGTTGATAAGCCCGGCGTAAAGAGTGGTTATGGAGA

ATAAAACACTAAAGCTAAAGACCCCCTAGGCTGTTATACGCACCTGGCGGCTCGAACCAC

CTATACGAAAGTAGCTTTACCCCCCTTCCACCAGAATCCACGACAGCTGGGGCA

>790_Sample_ASV20

CACCGCGGTTATACGAGAGGCCCAAGTTGATAAGCCCGGCGTAAAGAGTGGTTATGGAGA

ATAAAACACTAAAGCTAAAGACCCCCTAGGCTGTTATACGCACCTGGCGGCTCGAACCAC

CTATACGAAAGTAGCTTTACCCCCCTTCCACCAGAATCCACGACAGCTGGGGCA

>790_Sample_ASV21

CACCGCGGTTATACGAGAGGCCCTAGTTGATAGCCACGGCGCAAAGAGTGGTTAAGGAGC

CCCACTAAATAAAGTCGAACACTTCCTAGGCCGTTATACGTTTTCTAGAAGCACGAAACC

CAATTACACGAAAGCAACTTTATACTAAACAACCTGACCCCACGAAAGCTAAGAAA

>790_Sample_ASV22

CACCGCGGTTATACGAGAGGCCCTAGTTGATAGCCACGGCGCAAAGAGTGGTTAAGGAGC

CCCACTAAATAAAGTCGAACACTTCCTAGGCCGTTATACGTTTTCTAGAAGCACGAAACC

CAATTACACGAAAGCAACTTTATACTAAACAACCTGACCCCACGAAAGCTAAGAAA

>790_Sample_ASV23

CACCGCGGTTATACGAGAGGCCCAAGTTGACAGACATCGGCGTAAAGAGTGGTTAAGGAA

ACCCCATTACTAAAGCCGAACGCCCTCAGAACTGTCATACGTTCCCGAAGGTAAGAAGCC

CCACTACGAAAGTGGCTTTAACCACACCCGACCCCACGAAAGCTGTGAAA

>790_Sample_ASV24

CACCGCGGTTATACGAGAGGCCCAAGTTGACAGACATCGGCGTAAAGAGTGGTTAAGGAA

ACCCCATTACTAAAGCCGAACGCCCTCAGAACTGTCATACGTTCCCGAAGGTAAGAAGCC

CCACTACGAAAGTGGCTTTAACCACACCCGACCCCACGAAAGCTGTGAAA

>790_Sample_ASV25

CACCGCGGTTATACGAGAGGCCCAAGTTGATAGATATCGGCGTAAAGAGTGGTTAAGGAA

ACCCCGTTACTAAAGCCGAACGCCCTCAGAACTGTCATACGTTCCCGAAGGTAAGAAGCC

CCACTACGAAAGTGGCTTTAACCACACCCGACCCCACGAAAGCTGTGAAA

>790_Sample_ASV26

CACCGCGGTTATACGAGAGGCCCAAGTTGATAGATATCGGCGTAAAGAGTGGTTAAGGAA

ACCCCGTTACTAAAGCCGAACGCCCTCAGAACTGTCATACGTTCCCGAAGGTAAGAAGCC

CCACTACGAAAGTGGCTTTAACCACACCCGACCCCACGAAAGCTGTGAAA

>790_Sample_ASV27

CACCGCGGTTATACGAGAGGCCCAAGTTGACAGACACCGGCGTAAAGAGTGGTTAAGGAA

ATTCATCAACTAAAGCCGAATGCCCTCAGGACTGTTATACGTTTGCGAAGGTAAGAAGCC

CTACTACGAAAGTGGCTTTATTCCACCTGATTCCACGAAAGCTGCGGAA

>790_Sample_ASV28

CACCGCGGTTATACGAGAGGCCCAAGTTGACAGACACCGGCGTAAAGAGTGGTTAAGGAA

ATTCATCAACTAAAGCCGAATGCCCTCAGGACTGTTATACGTTTGCGAAGGTAAGAAGCC

CTACTACGAAAGTGGCTTTATTCCACCTGATTCCACGAAAGCTGCGGAA

>790_Sample_ASV29

CACCGCGGTTATACGAGAGACCCCAGTTGATATTAGCGGCGTAAAGGGTGGTTAAGGACT

TTAATAACTAAAGCTAAACACTTCCCCGGCTGTTGTACGTAGACCGGAAGCTTGAAGCCC

AGACACGAAAGTAGCTTTATTTCCCCTGACCCCACGAAAGCTGAGAAA

>790_Sample_ASV30

CACCGCGGTTATACGAGAGGCCCCAGTTGATATTAGCGGCGTAAAGGGTGGTTAAGGACT

TTAATAACTAAAGCCAAACACTTCCCCGGCTGTTGTACGTAGACCGGAAGCTTGAAGCCC

AGACACGAAAGTAGCTTTATTTCCCCTGACCCCACGAAAGCTGAGAAA

>790_Sample_ASV31

CACCGCGGTTATACGAGAGGCCCCAGTTGATATTAGCGGCGTAAAGGGTGGTTAAGGACT

TTAATAACTAAAGCTAAACACTTCCCCGGCTGTTGTACGTAGACCGGAAGCTTGAAGCCC

AGACACGAAAGTAGCTTTATTCCCCCTGACCCCACGAAAGCTGAGAAA

>790_Sample_ASV32

CACCGCGGTTATACGAGAGGCCCCAGTTGATATTAGCGGCGTAAAGGGTGGTTAAGGACT

TTAATAACTAAAGCTAAACACTTCCCCGGCTGTTGTACGTAGACCGGAAGCTTGAAGCCC

AGACACGAAAGTAGCTTTATTTCCCCTGACCCCACGAAAGCTGAGAAA

>790_Sample_ASV33

CACCGCGGTTATACGAGAGACCCCAGTTGATATTAGCGGCGTAAAGGGTGGTTAAGGACT

TTAATAACTAAAGCTAAACACTTCCCCGGCTGTTGTACGTAGACCGGAAGCTTGAAGCCC

AGACACGAAAGTAGCTTTATTTCCCCTGACCCCACGAAAGCTGAGAAA

>790_Sample_ASV34

CACCGCGGTTATACGAGAGGCCCCAGTTGATATTAGCGGCGTAAAGGGTGGTTAAGGACT

TTAATAACTAAAGCTAAACACTTCCCCGGCTGTTGTACGTAGACCGGAAGCTTGAAGCCC

AGACACGAAAGTAGCTTTATTCCCCCTGACCCCACGAAAGCTGAGAAA

>790_Sample_ASV35

CACCGCGGTTATACGAGAGGCCCCAGTTGATATTAGCGGCGTAAAGGGTGGTTAAGGACT

TTAATAACTAAAGCCAAACACTTCCCCGGCTGTTGTACGTAGACCGGAAGCTTGAAGCCC

AGACACGAAAGTAGCTTTATTTCCCCTGACCCCACGAAAGCTGAGAAA

>790_Sample_ASV36

CACCGCGGTTATACGAGAGGCCCCAGTTGATATTAGCGGCGTAAAGGGTGGTTAAGGACT

TTAATAACTAAAGCTAAACACTTCCCCGGCTGTTGTACGTAGACCGGAAGCTTGAAGCCC

AGACACGAAAGTAGCTTTATTTCCCCTGACCCCACGAAAGCTGAGAAA

>790_Sample_ASV37

CACCGCGGTCACACGATTAACCCAAGTCAATAGAAGCCGGCGTAAAGAGTGTTTTAGATC

ACCCCCTCCCCAATAAAGCTAAAACTCACCTGAGTTGTAAAAAACTCCAGTTGACACAAA

ATAGACTACGAAAGTGGCTTTAACATATCTGAACACACAATAGCTAAGACC

>790_Sample_ASV38

CACCGCGGTCACACGATTAACCCAAGTCAATAGAAGCCGGCGTAAAGAGTGTTTTAGATC

ACCCCCTCCCCAATAAAGCTAAAACTCACCTGAGTTGTAAAAAACTCCAGTTGACACAAA

ATAGACTACGAAAGTGGCTTTAACATATCTGAACACACAATAGCTAAGACC

>790_Sample_ASV39

CACCGCGGTTATACGAAAGACCCCAGTTTATAGACACGGCGTAAAGGGTGGTTATAGGAC

AAACTAACATAGAGCCAAAGACCCCCTAAGCCGTCATACGCTCACGAGGGCTCGAAGCCC

CTATACGAAAGTAGCTCTACCAAAAAATGCTCGACTCCACGAAAGCTGGGACA

>790_Sample_ASV40

CACCGCGGTTATACGAAAGACCCCAGTTTATAGACACGGCGTAAAGGGTGGTTATAGGAC

AAACTAACATAGAGCCAAAGACCCCCTAAGCCGTCATACGCTCACGAGGGCTCGAAGCCC

CTATACGAAAGTAGCTCTACCAAAAAATGCTCGACTCCACGAAAGCTGGGACA

>790_Sample_ASV41

CACCGCGGTTATACGAGAGGCCCCAGTTGATAAACACGGCGTAAAGGGTGGTTAAGGGAG

CATAAGAATAAAGCCGAAGGGCCCTTTGGCCGTTATACGCTTCTAGGTGTCCGAAGCCCA

AACACGAAAGTAGCTTTAGTTAAGCCCACCTGACTCCACGAAAACTGAGAAA

>790_Sample_ASV42

CACCGCGGTTATACGAGAGGCCCCAGTTGATAAACACGGCGTAAAGGGTGGTTAAGGGAG

CATAAGAATAAAGCCGAAGGGCCCTTTGGCCGTTATACGCTTCTAGGTGTCCGAAGCCCA

AACACGAAAGTAGCTTTAGTTAAGCCCACCTGACTCCACGAAAACTGAGAAA

>790_Sample_ASV43

CACCGCGGTTATACGAGAGACCCTAGTTGATAAATACGGCGTAAAGAGTGGTTATGGGAA

AAACAAATAAAGTCAAACAACCTCTTAGCTGTTATACGCATTATGAGAGCATGAAGCCCC

CTCACGAAAGTAACTTTAATACCTCCTGACCCCACGAAAGCTAAGAAA

>790_Sample_ASV44

CACCGCGGTTATACGAGAGACCCTAGTTGATAAATACGGCGTAAAGAGTGGTTATGGGAA

AAACAAATAAAGTCAAACAACCTCTTAGCTGTTATACGCATTATGAGAGCATGAAGCCCC

CTCACGAAAGTAACTTTAATACCTCCTGACCCCACGAAAGCTAAGAAA

>790_Sample_ASV45

CACCGCGGTTATACGAGAGGCTCAAGTTGATAAACCCCGGCGTAAAGAGTGGTTAAGGAA

AAATAGAAACTAAAGCCGAATGCTTTCAAGACTGTTATACGTTCCCGAGAGTAAGAAGAC

CAATTACGAAAGTGGCTTTACTTTACCTGACCCCACGAAAGCTACGACA

>790_Sample_ASV46

CACCGCGGTTATACGAGAGGCTCAAGTTGATAAACCCCGGCGTAAAGAGTGGTTAAGGAA

AAATAGAAACTAAAGCCGAATGCTTTCAAGACTGTTATACGTTCCCGAGAGTAAGAAGAC

CAATTACGAAAGTGGCTTTACTTTACCTGACCCCACGAAAGCTACGACA

>790_Sample_ASV47

CACCGCGGTTATACGAGAGGCTCAAGTTGATGAACCCCGGCGTAAAGAGTGGTTAAGGGA

GATCAAAACTAAAGCCGAATGCTTTCAAAGCTGTTATACGCTTCCGAAAGTAAGAAGCTC

AATCACGAAAGTGGCTTTACTTTACCTGACCCCACGAAAGCTACGACA

>790_Sample_ASV48

CACCGCGGTTATACGAGAGGCTCAAGTTGATGAACCCCGGCGTAAAGAGTGGTTAAGGGA

GATCAAAACTAAAGCCGAATGCTTTCAAAGCTGTTATACGCTTCCGAAAGTAAGAAGCTC

AATCACGAAAGTGGCTTTACTTTACCTGACCCCACGAAAGCTACGACA

>790_Sample_ASV49

CACCGCGGTTATACGAGAGACCCTAATTGATAGACACGGCGTAAAGAGTGGTTTGGGAAA

GCCCAAAAATAAAGCTAAAGACCTTCTAAGCCGTCACACGCACGCCGAAGGCACGAAGCC

CTGACACGAAAGTAGCTTTACTATTACCCCCGACGCCACGAAAGCTAAGAAA

>790_Sample_ASV50

CACCGCGGTTATACGAGAGACCCTAATTGATAGACACGGCGTAAAGAGTGGTTTGGGAAA

GCCCAAAAATAAAGCTAAAGACCTTCTAAGCCGTCACACGCACGCCGAAGGCACGAAGCC

CTGACACGAAAGTAGCTTTACTACTGCCCCTGACGCCACGAAAGCTAAGAAA

>790_Sample_ASV51

CACCGCGGTTATACGAGAGACCCTAATTGATAGACACGGCGTAAAGAGTGGTTTGGGAAA

GCCCAAAAATAAAGCTAAAGACCTTCTAAGCCGTCACACGCACGCCGAAGGCACGAAGCC

CCAACACGAAAGTAGCTTTACTACTGCCCCTGACGCCACGAAAGCTAAGAAA

>790_Sample_ASV52

CACCGCGGTTATACGAGAGACCCTAATTGATAGACACGGCGTAAAGAGTGGTTTGGGAAA

GCCCAAAAATAAAGCTAAAGACCTTCTAAGCCGTCACACGCACGCCGAAGGCACGAAGCC

CCAACACGAAAGTAGCTTTACTACTGCCCCTGACGCCACGAAAGCTAAGAAA

>790_Sample_ASV53

CACCGCGGTTATACGAGAGACCCTAATTGATAGACACGGCGTAAAGAGTGGTTTGGGAAA

GCCCAAAAATAAAGCTAAAGACCTTCTAAGCCGTCACACGCACGCCGAAGGCACGAAGCC

CTGACACGAAAGTAGCTTTACTACTGCCCCTGACGCCACGAAAGCTAAGAAA

>790_Sample_ASV54

CACCGCGGTTATACGAGAGACCCTAATTGATAGACACGGCGTAAAGAGTGGTTTGGGAAA

GCCCAAAAATAAAGCTAAAGACCTTCTAAGCCGTCACACGCACGCCGAAGGCACGAAGCC

CTGACACGAAAGTAGCTTTACTATTACCCCCGACGCCACGAAAGCTAAGAAA

>790_Sample_ASV55

AACCGCGGTTATACGAGAGACCCTAATTGATAGCTCACGGCGTAAAGGGTGGTTTGGGGA

AACCTCAAAATAAAGCTAAAGACCTTCTAAGCCGTTACACGCATACCGAAGGCACGAGGC

CCTAACACGAAAGTAGCTTTACTATTACCCCTGACGCCACGAAAGCTAAGAAA

>790_Sample_ASV56

AACCGCGGTTATACGAGAGACCCTAATTGATAGCTCACGGCGTAAAGGGTGGTTTGGGGA

AACCTCAAAATAAAGCTAAAGACCTTCTAAGCCGTTACACGCATACCGAAGGCACGAGGC

CCTAACACGAAAGTAGCTTTACTATTACCCCTGACGCCACGAAAGCTAAGAAA

>790_Sample_ASV57

CACCGCGGTTATACGAGAGACCCTAATTGATAGACACGGCGTAAAGAGTGGTTTAGGACA

ACCCAAAAATAAAGCTAAAGACCTTCTAAGCCGTCACACGCACGCCGAAGGCACGAAGCC

CTGACACGAAAGTAGCTTTACTATTACCCCCGACGCCACGAAAGCTAAGAAA

>790_Sample_ASV58

CACCGCGGTTATACGAGAGACCCTAATTGATAGACACGGCGTAAAGAGTGGTTTAGGACA

ACCCAAAAATAAAGCTAAAGACCTTCTAAGCCGTCACACGCACGCCGAAGGCACGAAGCC

CTGACACGAAAGTAGCTTTACTATTACCCCCGACGCCACGAAAGCTAAGAAA

>790_Sample_ASV59

CACCGCGGTTATACGAGAGACCCTAATTGATAGACACGGCGTAAAGAGTGGTTTAGGACA

ACCCAAAAATAAAGCTAAAGACCTTCTAAGCCGTCACACGCATACCGAAGGCACGAAGCC

CTGACACGAAAGTAGCTTTACTATTACCCCCGACGCCACGAAAGCTAAGAAA

>790_Sample_ASV60

CACCGCGGTTATACGAGAGACCCTAATTGATAGACACGGCGTAAAGAGTGGTTTAGGACA

ACCCAAAAATAAAGCTAAAGACCTTCTAAGCCGTCACACGCATACCGAAGGCACGAAGCC

CTGACACGAAAGTAGCTTTACTATTACCCCCGACGCCACGAAAGCTAAGAAA

>790_Sample_ASV61

CACCGCGGTTATACGAAAGACCCTAGTTGATGGATCACGGCGTAAAGGGTGGTTAAGGGA

TATAAAAATAAAGCTAAAAATCCTCTTAGCTGTCATACGCTTTCCGAAGACATGAGGCCC

AATTACGAAAGTAGCTTTAAACCACCCTGACACCACGAAAGCCAAGAAA

>790_Sample_ASV62

CACCGCGGTTATACGAAAGACCCTAGTTGATGGATCACGGCGTAAAGGGTGGTTAAGGGA

TATAAAAATAAAGCTAAAAATCCTCTTAGCTGTCATACGCTTTCCGAAGACATGAGGCCC

AATTACGAAAGTAGCTTTAAACCACCCTGACACCACGAAAGCCAAGAAA

>790_Sample_ASV63

CACCGCGGTTATACGAAAGACCCTAGTTTATAGATGCGGCGTAAAGGGTGGTTAAGGAAA

ACCTAAAATAATGCTAAAGACTCTCAAAGCTGTTATACGCTTCATGAGCACACGAAATCC

AAATACGAAAGTTGCCTTAATAAACTTTGCCTGACCCCACGAAAGCTGAAAAA

>790_Sample_ASV64

CACCGCGGTTATACGAAAGACCCTAGTTTATAGATGCGGCGTAAAGGGTGGTTAAGGAAA

ACCTAAAATAATGCTAAAGACTCTCAAAGCTGTTATACGCTTCATGAGCACACGAAATCC

AAATACGAAAGTTGCCTTAATAAACTTTGCCTGACCCCACGAAAGCTGAAAAA

>790_Sample_ASV65

CACCGCGGTTATACGAAAGACCCCAGTTTATAGATGCGGCGTAAAGGGTGGTTAAGAGAA

AATCAAAATAAGGCCAAAGATCCCCTAAGCAGTCATACGCTCCACGAGGACACGAAACCC

AAATACGAAAGTTGCCTTAATAAACTTCACCTGATCCCACGAAAGCTGAAAAA

>790_Sample_ASV66

CACCGCGGTTATACGAAAGACCCCAGTTTATAGATGCGGCGTAAAGGGTGGTTAAGAGAA

AATCAAAATAAGGCCAAAGATCCCCTAAGCAGTCATACGCTCCACGAGGACACGAAACCC

AAATACGAAAGTTGCCTTAATAAACTTCACCTGATCCCACGAAAGCTGAAAAA

>790_Sample_ASV67

CACCGCGGTTATACGAAAGGCTCTAGTTTATAAATACGGCGTAAAGGGTGGTTAAGGAAA

AATTAAAATAATGCTAAAGACCCCCTAAGCAGTTATACGCTTCACGGAGGCACGAAACCC

AAATACGAAAGTTGCCTTAATAAACTTTACCTGACCCCACGAAAGCTGAGAAA

>790_Sample_ASV68

CACCGCGGTTATACGAAAGGCTCTAGTTTATAAATACGGCGTAAAGGGTGGTTAAGGAAA

AATTAAAATAATGCTAAAGACCCCCTAAGCAGTTATACGCTTCACGGAGGCACGAAACCC

AAATACGAAAGTTGCCTTAATAAACTTTACCTGACCCCACGAAAGCTGAAAAA

>790_Sample_ASV69

CACCGCGGTTATACGAAAGGCTCTAGTTTATAAATACGGCGTAAAGGGTGGTTAAGGAAA

AATTAAAATAATGCTAAAGACCCCCTAAGCAGTTATACGCTTCACGGAGGCACGAAACCC

AAATACGAAAGTTGCCTTAATAAACTTTACCTGACCCCACGAAAGCTGAGAAA

>790_Sample_ASV70

CACCGCGGTTATACGAAAGGCTCTAGTTTATAAATACGGCGTAAAGGGTGGTTAAGGAAA

AATTAAAATAATGCTAAAGACCCCCTAAGCAGTTATACGCTTCACGGAGGCACGAAACCC

AAATACGAAAGTTGCCTTAATAAACTTTACCTGACCCCACGAAAGCTGAAAAA

>790_Sample_ASV71

CACCGCGGTTATACGAGAGGCCCAAGTTGACAAACCCCGGCGTAAAGAGTGGTTAAGGAA

AATTTAAAACTAAAGCCGAATGCCCCCAAAGCTGTTATACGCACCCGAGGATAAGAAGCC

CAATCACGAAAGTGGCTTTACACTACCTGAACCCACGAAAGCTATGAAA

>790_Sample_ASV72

CACCGCGGTTATACGAGAGGCCCAAGTTGACAAACCCCGGCGTAAAGAGTGGTTAAGGAA

AATTTAAAACTAAAGCCGAATGCCCCCAAAGCTGTTATACGCACCCGAGGATAAGAAGCC

CAATCACGAAAGTGGCTTTACACTACCTGAACCCACGAAAGCTATGAAA

>790_Sample_ASV73

CACCGCGGTTATACGAGAGGCCCAAGTTGACAAACCCCGGCGTAAAGAGTGGTTAAGGAA

AATTTAAAACTAAAGCCGAATGCCCCCAAAGCTGTTATACGCACCCGAGGGTAAGAAGCC

CAATCACGAAAGTGGCTTTACACTACCTGAACCCACGAAAGCTATGAAA

>790_Sample_ASV74

CACCGCGGTTATACGAGAGGCCCAAGTTGACAAACCCCGGCGTAAAGAGTGGTTAAGGAA

AATTTAAAACTAAAGCCGAATGCCCCCAAAGCTGTTATACGCACCCGAGGGTAAGAAGCC

CAATCACGAAAGTGGCTTTACACTACCTGAACCCACGAAAGCTATGAAA

>790_Sample_ASV75

CACCGCGGTTATACGAGAGGCCCTAGTTGATAACTACCGGCGTAAAGAGTGGTTATGGAA

AAATATTTAATAAAGCCGAACACCCCCTCAGCCGTCATACGCACCTGGGAGCACGAAGAC

CTACTGCGAAAGCAGCTTTAATTATGCCTGACCCCACGACAGCTAAGAAA

>790_Sample_ASV76

CACCGCGGTTATACGAGAGGCCCTAGTTGATAACTACCGGCGTAAAGAGTGGTTATGGAA

AAATATTTAATAAAGCCGAACACCCCCTCAGCCGTCATACGCACCTGGGAGCACGAAGAC

CTACTGCGAAAGCAGCTTTAATTATGCCTGACCCCACGACAGCTAAGAAA

>790_Sample_ASV77

CACCGCGGTTATACGAAAGACCCTAGTTTATAGACACGGCGTAAAGGGTGGTTCGGGACT

AAACAAAATAAAGCCAAAGACCTCCTAAGCCGTCATACGCTCACGGAAGCACGAAGCCCA

AGTACGAAAGTAGCTTTACAAAACATGCCTGACTCCACGAAAGCTAGGAAA

>790_Sample_ASV78

CACCGCGGTTATACGAAAGACCCTAGTTTATAGACACGGCGTAAAGGGTGGTTCGGGACT

AAACAAAATAAAGCCAAAGACCTCCTAAGCCGTCATACGCTCACGGAAGCACGAAGCCCA

AGTACGAAAGTAGCTTTACAAAACATGCCTGACTCCACGAAAGCTAGGAAA

>790_Sample_ASV79

CACCGCGGTTATACGAAAGACCCTAGTTGATAGGCACGGCGTAAAGGGTGGTTAGGGATA

TATTAAAAATAAAGTTAAAGAGCCTCTAAGCTGTCGCACGCATCCCGAGAGCTCGAGACC

CAGACACGAAAGTAACTTTAAAATACCTCACCTGACCCCACGAAAGCTAAGAAA

>790_Sample_ASV80

CACCGCGGTTATACGAAAGACCCTAGTTGATAGGCACGGCGTAAAGGGTGGTTAGGGATA

TATTAAAAATAAAGTTAAAGAGCCTCTAAGCTGTCGCACGCATCCCGAGAGCTCGAGACC

CAGACACGAAAGTAACTTTAAAATACCTCACCTGACCCCACGAAAGCTAAGAAA

>790_Sample_ASV81

CACCGCGGTTATACGAGAGGCCCAAGTTGATACTCCACGGCGTAAAGAGTGGTTAGAAAA

GACCTACTACTAAAGCCGAACGCCTTCAAAGCTGTTATACGCATCCGAAGGTGAGAAGCC

CACCCACGAAAGTGGCTTTACAACCTTGACTCCACGAAAGCTATGACA

>790_Sample_ASV82

CACCGCGGTTATACGAGAGGCCCAAGTTGATACTCCACGGCGTAAAGAGTGGTTAGAAAA

GACCTACTACTAAAGCCGAACGCCTTCAAAGCTGTTATACGCATCCGAAGGTGAGAAGCC

CACCCACGAAAGTGGCTTTACAACCTTGACTCCACGAAAGCTATGACA

>790_Sample_ASV83

CACCGCGGTTATACGAGAGACCCCAGTTGATAAACACGGCGTAAAGAGTGGTTAAGGGCA

CAAAACAAATAAAGTCAAACAACCTCTTAGCTGTTATACGCATTATGAGGATATGAAGCC

CCCCTACGAAAGTAACTTTACCAACCCCTGACCCCACGAAAGCTAAGAAA

>790_Sample_ASV84

CACCGCGGTTATACGAGAGACCCCAGTTGATAAACACGGCGTAAAGAGTGGTTAAGGGCA

CAAAACAAATAAAGTCAAACAACCTCTTAGCTGTTATACGCATTATGAGGATATGAAGCC

CCCCTACGAAAGTAACTTTACCAACCCCTGACCCCACGAAAGCTAAGAAA

>790_Sample_ASV85

CACCGCGGTTATACGAGAGACCCTAGTTGATATATACGGCGTAAAGAGTGGTTTGGAATA

ACCAAGTAATATAGCCAAAGACCTCCCAAGCCGTCACACGCACCCCGGAGGCACGAAGCC

CAAACACGAAAGTAGCTTTATTAACTTACCGACGCCACGAAAGCTAAGAAA

>790_Sample_ASV86

CACCGCGGTTATACGAGAGACCCTAGTTGATATATACGGCGTAAAGAGTGGTTTGGAATA

ACCAAGTAATATAGCCAAAGACCTCCCAAGCCGTCACACGCACCCCGGAGGCACGAAGCC

CAAACACGAAAGTAGCTTTATTAACTTACCGACGCCACGAAAGCTAAGAAA

>790_Sample_ASV87

CACCGCGGTTATACGAAAGACCCTAGTTTATAGGCACGGCGTAAAGGGTGGTTAGGGGAT

AAACAAAATAAAGCCAAAGACCTTCTAAGTCGTCATACACTCATGAAGGCACGAAGCCCA

AATACGAAAGTAACTTTACAACTTAGACCCGACTCCACGAAAGCTAAGAAA

>790_Sample_ASV88

CACCGCGGTTATACGAAAGACCCTAGTTTATAGGCACGGCGTAAAGGGTGGTTAGGGGAT

AAACAAAATAAAGCCAAAGACCTTCTAAGTCGTCATACACTCATGAAGGCACGAAGCCCA

AATACGAAAGTAACTTTACAACTTAGACCCGACTCCACGAAAGCTGGGGGA

>790_Sample_ASV89

CACCGCGGTTATACGAAAGACCCTAGTTTATAGGCACGGCGTAAAGGGTGGTTAGGGGAT

AAACAAAATAAAGCCAAAGACCTTCTAAGTCGTCATACACTCATGAAGGCACGAAGCCCA

AATACGAAAGTAACTTTACAACTTAGACCCGACTCCACGAAAGCTAAGAAA

>790_Sample_ASV90

CACCGCGGTTATACGAAAGACCCTAGTTTATAGGCACGGCGTAAAGGGTGGTTAGGGGAT

AAACAAAATAAAGCCAAAGACCTTCTAAGTCGTCATACACTCATGAAGGCACGAAGCCCA

AATACGAAAGTAACTTTACAACTTAGACCCGACTCCACGAAAGCTGGGGGA

>790_Sample_ASV91

CACCGCGGTTATACGAGAGGCCCAAGTTGACAGATATCGGCGTAAAGAGTGGTTAAGGAA

ATCTTAAAACTAAAGCCGAATGCCTTCAAGACTGTTATACGTTTACGAAGGTAAGAAGCC

CCACTACGAAAGTGGCTTTACTCCACCTGACTCCACGAAAGCTGTGAAA

>790_Sample_ASV92

CACCGCGGTTATACGAGAGGCCCAAGTTGATAGACATCGGCGTAAAGAGTGGTTAAGGAA

ATCTTAAAACTAAAGCCGAATGCCTTCAAGACTGTTATACGTTTACGAAGGTAAGAAGCC

CCACTACGAAAGTGGCTTTACTCCACCTGACTCCACGAAAGCTGTGAAA

>790_Sample_ASV93

CACCGCGGTTATACGAGAGGCCAAAGTTGACAGATATCGGCGTAAAGAGTGGTTAAGGAA

ATCTTAAAACTAAAGCCGAATGCCTTCAAGACTGTTATACGTTTCCGAAGGTAAGAAGCC

CCACTACGAAAGTGGCTTTACTCCACCTGACTCCACGAAAGCTGTGAAA

>790_Sample_ASV94

CACCGCGGTTATACGAGAGGCCCAAGTTGATAGACATCGGCGTAAAGAGTGGTTAAGGAA

GTCTCAAAACTAAAGCCGAATGCCTTCAAGACTGTTATACGTTTACGAAGGTAAGAAGCC

CCACTACGAAAGTGGCTTTATTCCACCTGACCCCACGAAAGCTGTGAAA

>790_Sample_ASV95

CACCGCGGTTATACGAGAGGCCCAAGTTGACAGATATCGGCGTAAAGAGTGGTTAAGGAA

ATCTTAAAACTAAAGCCGAATGCCTTCAAGACTGTTATACGTTTCCGAAGGTAAGAAGCC

CCACTACGAAAGTGGCTTTACTCCACCTGACTCCACGAAAGCTGTGAAA

>790_Sample_ASV96

CACCGCGGTTATACGAGAGGCCAAAGTTGACAGATATCGGCGTAAAGAGTGGTTAAGGAA

GTCTCAAAACTAAAGCCGAATGCCTTCAAGACTGTTATACGTTTCCGAAGGTAAGAAGCC

CCACTACGAAAGTGGCTTTACTCCACCTGACTCCACGAAAGCTGTGAAA

>790_Sample_ASV97

CACCGCGGTTATACGAGAGGCCCAAGTTGATAGACATCGGCGTAAAGAGTGGTTAAGGAA

GTCTCAAAACTAAAGCCGAATGCCTTCAAGACTGTTATACGTTTCCGAAGGTAAGAAGCC

CCACTACGAAAGTGGCTTTATTCCACCTGACCCCACGAAAGCTGTGAAA

>790_Sample_ASV98

CACCGCGGTTATACGAGAGGCCCAAGTTGATAGACATCGGCGTAAAGAGTGGTTAAGGAA

GTCTCAAAACTAAAGCCGAATGCCTTCAAGACTGTTATACGTTTCCGAAGGTAAGAAGCC

CCACTACGAAAGTGGCTTTACTCCACCTGACTCCACGAAAGCTGTGAAA

>790_Sample_ASV99

CACCGCGGTTATACGAGAGGCCCAAGTTGACAGATATCGGCGTAAAGAGTGGTTAAGGAA

ATCTTAAAACTAAAGCCGAATGCCTTCAAGACTGTTATACGTTTACGAAGGTAAGAAGCC

CCACTACGAAAGTGGCTTTACTCCACCTGACTCCACGAAAGCTGTGAAA

>790_Sample_ASV100

CACCGCGGTTATACGAGAGGCCAAAGTTGACAGATATCGGCGTAAAGAGTGGTTAAGGAA

ATCTTAAAACTAAAGCCGAATGCCTTCAAGACTGTTATACGTTTCCGAAGGTAAGAAGCC

CCACTACGAAAGTGGCTTTACTCCACCTGACTCCACGAAAGCTGTGAAA

>790_Sample_ASV101

CACCGCGGTTATACGAGAGGCCCAAGTTGATAGACATCGGCGTAAAGAGTGGTTAAGGAA

GTCTCAAAACTAAAGCCGAATGCCTTCAAGACTGTTATACGTTTACGAAGGTAAGAAGCC

CCACTACGAAAGTGGCTTTATTCCACCTGACCCCACGAAAGCTGTGAAA

>790_Sample_ASV102

CACCGCGGTTATACGAGAGGCCCAAGTTGACAGATATCGGCGTAAAGAGTGGTTAAGGAA

ATCTTAAAACTAAAGCCGAATGCCTTCAAGACTGTTATACGTTTCCGAAGGTAAGAAGCC

CCACTACGAAAGTGGCTTTACTCCACCTGACTCCACGAAAGCTGTGAAA

>790_Sample_ASV103

CACCGCGGTTATACGAGAGGCCAAAGTTGACAGATATCGGCGTAAAGAGTGGTTAAGGAA

GTCTCAAAACTAAAGCCGAATGCCTTCAAGACTGTTATACGTTTCCGAAGGTAAGAAGCC

CCACTACGAAAGTGGCTTTACTCCACCTGACTCCACGAAAGCTGTGAAA

>790_Sample_ASV104

CACCGCGGTTATACGAGAGGCCCAAGTTGATAGACATCGGCGTAAAGAGTGGTTAAGGAA

GTCTCAAAACTAAAGCCGAATGCCTTCAAGACTGTTATACGTTTCCGAAGGTAAGAAGCC

CCACTACGAAAGTGGCTTTATTCCACCTGACCCCACGAAAGCTGTGAAA

>790_Sample_ASV105

CACCGCGGTTATACGAGAGGCCCAAGTTGATAGACATCGGCGTAAAGAGTGGTTAAGGAA

GTCTCAAAACTAAAGCCGAATGCCTTCAAGACTGTTATACGTTTCCGAAGGTAAGAAGCC

CCACTACGAAAGTGGCTTTACTCCACCTGACTCCACGAAAGCTGTGAAA

>790_Sample_ASV106

CACCGCGGTTATACGAGAGGCCCAAGTTGATAGACATCGGCGTAAAGAGTGGTTAAGGAA

ATCTTAAAACTAAAGCCGAATGCCTTCAAGACTGTTATACGTTTACGAAGGTAAGAAGCC

CCACTACGAAAGTGGCTTTACTCCACCTGACTCCACGAAAGCTGTGAAA

>790_Sample_ASV107

CACCGCGGTTATACGAGAGACCCTAATTGATAGACTACGGCGTAAAGAGTGGTTTAGAAC

AAAAATTAATAAAGCCAAAGATCCTCCTGGCCGTCGCACGCATTTCGAGGATACGAAGCC

CCACTACGAAAGTAGCTTTACTACAACTTTTCTGACCCCACGAAAGCTAAGAAA

>790_Sample_ASV108

CACCGCGGTTATACGAGAGACCCTAATTGATAGACTACGGCGTAAAGAGTGGTTTAGAAC

AAAAATTAATAAAGCCAAAGATCCTCCTGGCCGTCGCACGCATTTCGAGGATACGAAGCC

CCACTACGAAAGTAGCTTTACTACAACTTTTCTGACCCCACGAAAGCTAAGAAA

>791_Sample_ASV1

CACCGCGGTTATACGAAAGACCCTAGTTGATGGGTGCGGCGTAAAGGGTGGTTAGGGACT

ACAAAAAATAGAGCTAAAGATCCTCTAGGTCGTCATACACATCACGAGGAAACGAAGCCC

AAATACGAAAGTAGCTCTAAATATAATCCTGACGCCACGAAAGCCAAGAAA

>791_Sample_ASV2

CACCGCGGTTATACGAGAGACCCTAGTTGATTAAAGCGGCGTAAAGAGTGGTTATGGAAC

TATTCTTTTAAAGCAGAAAACCTCTCAAACTGTTATACGCACCCAGAGGTCTGAATCCCT

CACACGAAAGTGACTTTATTTATGCCTACCAGAACCCACGAAAGCTGGGACA

>791_Sample_ASV3

CACCGCGGTTATACGAGAGACCCTAGTTGATATACACGGCGTAAAGAGTGGTTTGGGGTA

TATAATAAATAAAGCCAAAGACCTTCTAGGCCGTTGAACGCACCACGAAGGAGCGAAGTC

CCAATACGAAAGTAGCTTTATAAAACCTGACGCCACGAAAGCTAAGAAA

>791_Sample_ASV4

CACCGCGGTTATACGAGAGGCCCAAGTTGACAGACACCGGCGTAAAGAGTGGCTAGGGAA

AAATTACTACTAAAGCCGAACACCTTCAGAACTGTCATACGTTCCCGAAGATAAGAAGCC

CCACCACGAAAGTGACTTTATATTACCCGACCCCACGAAAGCTGTGAAA

>791_Sample_ASV5

CACCGCGGTTATACGAGAGGCCCAAGTTGACAGACACCGGCGTAAAGAGTGGCTAGGGAA

AAATTACTACTAAAGCCGAACACCTTCAGAACTGTCATACGTTCCCGAAGATAAGAAGCC

CCACCACGAAAGTGACTTTATATTACCCGACCCCACGAAAGCCAAGAAA

>791_Sample_ASV6

CACCGCGGTTATACGAGAGGCCCAAGTTGACAGACACCGGCGTAAAGAGTGGCTAGGGAA

AAATTACTACTAAAGCCGAACACCTTCAGAACTGTCATACGTTCCCGAAGATAAGAAGCC

CCACCACGAAAGTGGCTTTATATTACCCGACCCCACGAAAGCTGTGAAA

>791_Sample_ASV7

CACCGCGGTTATACGAGAGACCCAAGTTGACAGACACCGGCGTAAAGAGTGGCTAAGGAA

AAATTTCTACTAAAGCCGAACACCTTCAGAACTGTCATACGTTCCCGAAGAAAATAAGCC

CCACCACGAAAGTGGCTTTATATTACCCGACCCCACGAAAGCTGTGAAA

>791_Sample_ASV8

CACCGCGGTTATACGAGAGGCCCAAGTTGATAAGCCCGGCGTAAAGAGTGGTTATGGAGA

ATAAAACACTAAAGCTAAAGACCCCCTAGGCTGTTATACGCACCTGGCGGCTCGAACCAC

CTATACGAAAGTAGCTTTACCCCCCTTCCACCAGAATCCACGACAGCTGGGGCA

>791_Sample_ASV9

CACCGCGGTTATACGAGAGGCCCAAGTTGACAGACATCGGCGTAAAGAGTGGTTAAGGAA

ACCCCATTACTAAAGCCGAACGCCCTCAGAACTGTCATACGTTCCCGAAGGTAAGAAGCC

CCACTACGAAAGTGGCTTTAACCACACCCGACCCCACGAAAGCTGTGAAA

>791_Sample_ASV10

CACCGCGGTTATACGAGAGGCCCAAGTTGATAGATATCGGCGTAAAGAGTGGTTAAGGAA

ACCCCGTTACTAAAGCCGAACGCCCTCAGAACTGTCATACGTTCCCGAAGGTAAGAAGCC

CCACTACGAAAGTGGCTTTAACCACACCCGACCCCACGAAAGCTGTGAAA

>791_Sample_ASV11

CACCGCGGTCACACGATTAACCCAAGTCAATAGAAGCCGGCGTAAAGAGTGTTTTAGATC

ACCCCCTCCCCAATAAAGCTAAAACTCACCTGAGTTGTAAAAAACTCCAGTTGACACAAA

ATAGACTACGAAAGTGGCTTTAACATATCTGAACACACAATAGCTAAGACC

>791_Sample_ASV12

CACCGCGGTTATACGAGAGGCCCTAGTTGATAAATACGGCGTAAAGAGTGGTTAGAGGAC

TCCCATAATAAAGCCAAAGACCCTCCGGGCCGTCACACGCATACGAGGGCACGAAGTTCA

CACACGAAAGTAGCTTTAAATTACCCGACGCCACGAAAGCTAAGAAA

>791_Sample_ASV13

CACCGCGGTTATACGAGAAACCCTAGTTGACAAATACGGCGTAAAGAGTGGTTAAGGGGT

ATCAAAAATAAAGTCAAACATTTTCCTAGTTGTTATAAGCATTTCGAGAACATGAAGCCC

AACCACGAAAGTAACTTTACCTTCACCTGACCCCACGAAAGCTAAGAAA

>791_Sample_ASV14

CACCGCGGTTATACGAAAGACCCCAGTTTATAGACACGGCGTAAAGGGTGGTTATAGGAC

AAACTAACATAGAGCCAAAGACCCCCTAAGCCGTCATACGCTCACGAGGGCTCGAAGCCC

CTATACGAAAGTAGCTCTACCAAAAAATGCTCGACTCCACGAAAGCTGGGACA

>791_Sample_ASV15

CACCGCGGTTATACGAGAGACCCTAGTTGATAAATACGGCGTAAAGAGTGGTTATGGGAA

AAACAAATAAAGTCAAACAACCTCTTAGCTGTTATACGCATTATGAGAGCATGAAGCCCC

CTCACGAAAGTAACTTTAATACCTCCTGACCCCACGAAAGCTAAGAAA

>791_Sample_ASV16

AACCGCGGTTATACGAGAGACCCTAATTGATAGCTCACGGCGTAAAGGGTGGTTTGGGGA

AACCTCAAAATAAAGCTAAAGACCTTCTAAGCCGTTACACGCATACCGAAGGCACGAGGC

CCTAACACGAAAGTAGCTTTACTATTACCCCTGACGCCACGAAAGCTAAGAAA

>791_Sample_ASV17

CACCGCGGTTATACGAAAGACCCTAGTTGATGGATCACGGCGTAAAGGGTGGTTAAGGGA

TATAAAAATAAAGCTAAAAATCCTCTTAGCTGTCATACGCTTTCCGAAGACATGAGGCCC

AATTACGAAAGTAGCTTTAAACCACCCTGACACCACGAAAGCCAAGAAA

>791_Sample_ASV18

CACCGCGGTTATACGAGAGACCCTAGTTGATAGCTACGGCGTAAAGCGTGGTTAGGAGAA

CAAGATAAATAAAGTCAAACAATCTCTCGGCCGTTATACGTTATCTGAGAATATGAAGTC

CTACCACGAAAGTAACTTTAATTTTTCTGACCCCACGAAAACTGAGGAA

>791_Sample_ASV19

CACCGCGGTCATACGATTAACCCAAGTTAATAGGCACACGGCGTAAAGCGTGTTTAAGCA

CTACGCCAAATAAAGTTAAACTCCAATTAAGCTGTAAAAAGCCATAATCAAGACGAAAAT

AAATAACGAAAGTAACTTTACAACCGCTGAAACACGATAGCTAAGATC

>791_Sample_ASV20

CACCGCGGTTATACGAGAGGCCCAAGTTGATACTCCACGGCGTAAAGAGTGGTTAGAAAA

GACCTACTACTAAAGCCGAACGCCTTCAAAGCTGTTATACGCATCCGAAGGTGAGAAGCC

CACCCACGAAAGTGGCTTTACAACCTTGACTCCACGAAAGCTATGACA

>791_Sample_ASV21

CACCGCGGTTATACGAGAGACCCCAGTTGATAAACACGGCGTAAAGAGTGGTTAAGGGCA

CAAAACAAATAAAGTCAAACAACCTCTTAGCTGTTATACGCATTATGAGGATATGAAGCC

CCCCTACGAAAGTAACTTTACCAACCCCTGACCCCACGAAAGCTAAGAAA

>791_Sample_ASV22

CACCGCGGTTATACGAAAGACCCTAGTTTATAGGCACGGCGTAAAGGGTGGTTAGGGGAT

AAACAAAATAAAGCCAAAGACCTTCTAAGTCGTCATACACTCATGAAGGCACGAAGCCCA

AATACGAAAGTAACTTTACAACTTAGACCCGACTCCACGAAAGCTGGGGGA

>791_Sample_ASV23

CACCGCGGTTATACGAGAGACCCTAATTAATAGATACGGCGTAAAGAGTGGTTTAGGACA

AAAATTAATAAAGCCGAAGTTTCTCCTAGCCGTTGTACGCACTCTGAGAACACGAAGCCC

CACTACGAAAGTAGCTTTACCTATTATTTCCTGACCCCACGAAAGCTAAGAAA

>791_Sample_ASV24

CACCGCGGTTATACGAGAGGCCCAAGTTGATAGACATCGGCGTAAAGAGTGGTTAAGGAA

GTCTCAAAACTAAAGCCGAATGCCTTCAAGACTGTTATACGTTTACGAAGGTAAGAAGCC

CCACTACGAAAGTGGCTTTATTCCACCTGACCCCACGAAAGCTGTGAAA

>791_Sample_ASV25

CACCGCGGTTATACGAGAGGCCCAAGTTGATAGACATCGGCGTAAAGAGTGGTTAAGGAA

GTCTCAAAACTAAAGCCGAATGCCTTCAAGACTGTTATACGTTTCCGAAGGTAAGAAGCC

CCACTACGAAAGTGGCTTTATTCCACCTGACCCCACGAAAGCTGTGAAA

>791_Sample_ASV26

CACCGCGGTTATACGAGAGGCCCAAGTTGATAGACATCGGCGTAAAGAGTGGTTAAGGAA

GTCTCAAAACTAAAGCCGAATGCCTTCAAGACTGTTATACGTTTCCGAAGGTAAGAAGCC

CCACTACGAAAGTGGCTTTACTCCACCTGACTCCACGAAAGCTGTGAAA

>791_Sample_ASV27

CACCGCGGTTATACGAGAGACTCCAGTTGATAGCTACGGCGTAAAGAGTGGTTTGGGGCC

CGCCCAAAATAAAGCCAAAGACCTCCCAAGCCGTCAAACGCACCCCGGAGGCACGAAGTC

CTAACGCGAAAGCAACTTTACCTTCCCCGACGCCACGAAAGCTAAGAAA

>792_Sample_ASV1

TACCGCGGTTATACGAAAGACCCAAGTTAATAAACTACGGCGTAAAGCGTGGTTAGAGAC

CTCCAATAATTAAAGCCAAAGACCTTCTAAGCCGTCGCACGCCCCGCGAAGACACGAAGC

CCAAAAACGAAAGTAGCTTTATTCTTACTCGACCCCACGAAAGCTAGGGCA

>792_Sample_ASV2

CACCGCGGTTATACGAAAGACCCTAGTTGATGGGTGCGGCGTAAAGGGTGGTTAGGGACT

ACAAAAAATAGAGCTAAAGATCCTCTAGGTCGTCATACACATCACGAGGAAACGAAGCCC

AAATACGAAAGTAGCTCTAAATATAATCCTGACGCCACGAAAGCCAAGAAA

>792_Sample_ASV3

CACCGCGGTTATACGAGAGACCCTAGTTGATTAAAGCGGCGTAAAGAGTGGTTATGGAAC

TATTCTTTTAAAGCAGAAAACCTCTCAAACTGTTATACGCACCCAGAGGTCTGAATCCCT

CACACGAAAGTGACTTTATTTATGCCTACCAGAACCCACGAAAGCTGGGACA

>792_Sample_ASV4

CACCGCGGTTATACGAGAGGCCCAAGTTGACAGACACCGGCGTAAAGAGTGGCTAGGGAA

AAATTACTACTAAAGCCGAACACCTTCAGAACTGTCATACGTTCCCGAAGATAAGAAGCC

CCACCACGAAAGTGACTTTATATTACCCGACCCCACGAAAGCTAAGAAA

>792_Sample_ASV5

CACCGCGGTTATACGAGAGGCCCAAGTTGACAGACACCGGCGTAAAGAGTGGCTAGGGAA

AAATTACTACTAAAGCCGAACACCTTCAGAACTGTCATACGTTCCCGAAGATAAGAAGCC

CCACCACGAAAGTGACTTTATATTACCCGACCCCACGAAAGCTGTGAAA

>792_Sample_ASV6

CACCGCGGTTATACGAGAGGCCCAAGTTGACAGACACCGGCGTAAAGAGTGGCTAGGGAA

AAATTACTACTAAAGCCGAACACCTTCAGAACTGTCATACGTTCCCGAAGATAAGAAGCC

CCACCACGAAAGTGGCTTTATATTACCCGACCCCACGAAAGCTAAGAAA

>792_Sample_ASV7

CACCGCGGTTATACGAGAGGCCCAAGTTGACAGACACCGGCGTAAAGAGTGGCTAGGGAA

AAATTACTACTAAAGCCGAACACCTTCAGAACTGTCATACGTTCCCGAAGATAAGAAGCC

CCACCACGAAAGTGGCTTTATATTACCCGACCCCACGAAAGCTGTGAAA

>792_Sample_ASV8

CACCGCGGTTATACGAGAGGCCCAAGTTGACAGACACCGGCGTAAAGAGTGGCTAAGGAA

AAATTTCTACTAAAGCCGAACACCTTCAGAACTGTCATACGTTCCCGAAGAAAAGAAGCC

CCACCACGAAAGTGGCTTTATATTACCCGACCCCACGAAAGCTGTGAAA

>792_Sample_ASV9

CACCGCGGTTATACGAAAGACTCTAGTTGACAAACACGGCGTAAAGGGTGGTTAGGATAA

CAACAAAATAAAGCTAAAGACTTACCAAGCCGTCATACGCCCATGTAAGAGCGAAAACCA

CAAACGAAAGTAACTTTATTACACCATCCGAATCCACGAAAGCTAAGAAA

>792_Sample_ASV10

CACCGCGGTTATACGAAAGACCCTAGTTGATAGATCACGGCGTAAAGGGTGGTTAGGGAA

TACAAAAATAAAGCTAAAAATCCTCTAAGCTGTCATACGCTTCCCGAAGACATGAAACCC

AACCACGAAAGTAGCTTTAAACTCTCCTGACACCACGAAAGCCAAGGAA

>792_Sample_ASV11

CACCGCGGTTATACGAAAGACCCTAGTTTATAGGCACGGCGTAAAGGGTGGTTAGGGAAT

ACAAAAATAAAGCTAAAAATCCTCTAAGCTGTCATACGCTTCCCGAAGACATGAAACCCA

ACCACGAAAGTAGCTTTAAACTCTCCTGACACCACGAAAGCCAAGGAA

>792_Sample_ASV12

CACCGCGGTTATACGAGAGGCCCAAGTTGATAAGCCCGGCGTAAAGAGTGGTTATGGAGA

ATAAAACACTAAAGCTAAAGACCCCCTAGGCTGTTATACGCACCTGGCGGCTCGAACCAC

CTATACGAAAGTAGCTTTACCCCCCTTCCACCAGAATCCACGACAGCTGGGGCA

>792_Sample_ASV13

CACCGCGGTTATACGAGAGACCCTAATTGATAGGCACGGCGTAAAGAGTGGTTATGGAGA

ATAAAACACTAAAGCTAAAGACCCCCTAGGCTGTTATACGCACCTGGCGGCTCGAACCAC

CTATACGAAAGTAGCTTTACCCCCCTTCCACCAGAATCCACGACAGCTGGGGCA

>792_Sample_ASV14

CACCGCGGTTATACGAGAGGCCCAAGTTGACAGACATCGGCGTAAAGAGTGGTTAAGGAA

ACCCCATTACTAAAGCCGAACGCCCTCAGAACTGTCATACGTTCCCGAAGGTAAGAAGCC

CCACTACGAAAGTGGCTTTAACCACACCCGACCCCACGAAAGCTGTGAAA

>792_Sample_ASV15

CACCGCGGTTATACGAGAGGCCCAAGTTGACAGACACCGGCGTAAAGAGTGGTTAAGGAA

ATTCATCAACTAAAGCCGAATGCCCTCAGGACTGTTATACGTTTGCGAAGGTAAGAAGCC

CTACTACGAAAGTGGCTTTATTCCACCTGATTCCACGAAAGCTGCGGAA

>792_Sample_ASV16

CACCGCGGTTATACGAGAGGCCCCAGTTGATATTAGCGGCGTAAAGGGTGGTTAAGGACT

TTAATAACTAAAGCCAAACACTTCCCCGGCTGTTGTACGTAGACCGGAAGCTTGAAGCCC

AGACACGAAAGTAGCTTTATTTCCCCTGACCCCACGAAAGCTGAGAAA

>792_Sample_ASV17

CACCGCGGTTATACGAGAGGCCCCAGTTGATATTAGCGGCGTAAAGGGTGGTTAAGGACT

TTAATAACTAAAGCTAAACACTTCCCCGGCTGTTGTACGTAGACCGGAAGCTTGAAGCCC

AGACACGAAAGTAGCTTTATTCCCCCTGACCCCACGAAAGCTGAGAAA

>792_Sample_ASV18

CACCGCGGTTATACGAGAGGCCCCAGTTGATATTAGCGGCGTAAAGGGTGGTTAAGGACT

TTAATAACTAAAGCTAAACACTTCCCCGGCTGTTGTACGTAGACCGGAAGCTTGAAGCCC

AGACACGAAAGTAGCTTTATTTCCCCTGACCCCACGAAAGCTGAGAAA

>792_Sample_ASV19

CACCGCGGTTATACGAAAGACCCCAGTTTATAGACACGGCGTAAAGGGTGGTTATAGGAC

AAACTAACATAGAGCCAAAGACCCCCTAAGCCGTCATACGCTCACGAGGGCTCGAAGCCC

CTATACGAAAGTAGCTCTACCAAAAAATGCTCGACTCCACGAAAGCTGGGACA

>792_Sample_ASV20

CACCGCGGTTATACGAGAGACCCTAATTGATAGGTACGGCGTAAAGAGTGGTTTGGGATA

ACCCAAAAATAAAGCTAAAGACCTTCTAAGCCGTCACACGCATACCGAAGGCACGAGGCC

CCAACACGAAAGTAGCTTTACTATTACCCCGACGCCACGAAAGCTAAGAAA

>792_Sample_ASV21

CACCGCGGTTATACGAGAGACCCTAATTGATAGGCACGGCGTAAAGAGTGGTTTAGGGTA

ACCCATAAATAAAGCTAAAGACCTTCTAAGCCGTCACACGCACACCGAAGGCACGAGACC

CCAACACGAAAGTAGCTTTACTTCTACCCCTGACGCCACGAAAGCTAAGAAA

>792_Sample_ASV22

CACCGCGGTTATACGAAAGACCCTAGTTGATGGATCACGGCGTAAAGGGTGGTTAAGGGA

TATAAAAATAAAGCTAAAAATCCTCTTAGCTGTCATACGCTTTCCGAAGACATGAGGCCC

AATTACGAAAGTAGCTTTAAACCACCCTGACACCACGAAAGCCAAGAAA

>792_Sample_ASV23

CACCGCGGTTATACGAAAGACCCCAGTTTATAGATGCGGCGTAAAGGGTGGTTAAGAGAA

AATCAAAATAAGGCCAAAGATCCCCTAAGCAGTCATACGCTCCACGAGGACACGAAACCC

AAATACGAAAGTTGCCTTAATAAACTTCACCTGATCCCACGAAAGCTGAAAAA

>792_Sample_ASV24

CACCGCGGTTATACGAAAGGCTCTAGTTTATAAATACGGCGTAAAGGGTGGTTAAGGAAA

AATTAAAATAATGCTAAAGACCCCCTAAGCAGTTATACGCTTCACGGAGGCACGAAACCC

AAATACGAAAGTTGCCTTAATAAACTTTACCTGACCCCACGAAAGCTGAAAAA

>792_Sample_ASV25

CACCGCGGTTATACGAGAGACTCTAGTTGATACCAACGGCGTAAAGAGTGGTTTGGGATG

CCCCTTAAAATAAAGCCAAAGACCTCCCAAGCCGTCAAACGCACCCCGGAGGCACGAAGC

CCCAACGCGAAAGCAACTTTACCTCCCTCCCGACGCCACGAAAGCTAAGAAA

>792_Sample_ASV26

CACCGCGGTTATACGAGAGACCCTAGTTGATAGCTACGGCGTAAAGCGTGGTTAGGAGAA

CAAGATAAATAAAGTCAAACAATCTCTCGGCCGTTATACGTTATCTGAGAATATGAAGTC

CTACCACGAAAGTAACTTTAATTTTTCTGACCCCACGAAAACTGAGGAA

>792_Sample_ASV27

CACCGCGGTTATACGAGAGGCCCTAGTTGATAACTACCGGCGTAAAGAGTGGTTATGGAA

AAATATTTAATAAAGCCGAACACCCCCTCAGCCGTCATACGCACCTGGGAGCACGAAGAC

CTACTGCGAAAGCAGCTTTAATTATGCCTGACCCCACGACAGCTAAGAAA

>792_Sample_ASV28

CACCGCGGTTATACGAGAGGCCCAAGTTGATTATTACCGGCGTAAAGCGTGGTTAAGATT

TTTATAGGACTAAAGCCGAACCCCCTCAGAGCTGTTATACGCACCCGAAGGTTAGAAGTT

CAATCACGAAAGTGGCTTTACAACCCCGAACCCACGAAAGCTAAGATA

>792_Sample_ASV29

CACCGCGGTTATACGAGAGGCCCAAGTTGATACTCCACGGCGTAAAGAGTGGTTAGAAAA

GACCTACTACTAAAGCCGAACGCCTTCAAAGCTGTTATACGCATCCGAAGGTGAGAAGCC

CACCCACGAAAGTGGCTTTACAACCTTGACTCCACGAAAGCTATGACA

>792_Sample_ASV30

CACCGCGGTTATACGAGAGACCCCAGTTGATAAACACGGCGTAAAGAGTGGTTAAGGGCA

CAAAACAAATAAAGTCAAACAACCTCTTAGCTGTTATACGCATTATGAGGATATGAAGCC

CCCCTACGAAAGTAACTTTACCAACCCCTGACCCCACGAAAGCTAAGAAA

>792_Sample_ASV31

CACCGCGGTTATACGAAAGACCCTAGTTTATAGGCACGGCGTAAAGGGTGGTTAGGGGAT

AAACAAAATAAAGCCAAAGACCTTCTAAGTCGTCATACACTCATGAAGGCACGAAGCCCA

AATACGAAAGTAACTTTACAACTTAGACCCGACTCCACGAAAGCTGGGGGA

>792_Sample_ASV32

CACCGCGGTTATACGAAAGACCCTAGTTGATAGCTACGGCGTAAAGGGTGGTTAAGGTAT

ATAACAAATAAAGCTAAAGAGCCTCCAAGCCGTCGTACGCATTCTGAGAGCTCGAAACCC

TAACACGAAAGTAGCTTTAAAAAATAACACCTGACCCCACGAAAGCTAAGAAA

>792_Sample_ASV33

CACCGCGGTTATACGAAAGACCCTAGTTGATAGATCACGGCGTAAAGGGTGGTTAAGGAG

AACAAAAATAAAGCTAAAAATCTTCTAAGCTGTCATACGCTTTCCGAAGACATGAGACCC

AACCACGAAAGTAGCTTTAAACTTTCCTGACACCACGAAAGCCAAGAAA

>795_Sample_ASV1

CACCGCGGTTATACGAAAGACCCTAGTTGATGGGTGCGGCGTAAAGGGTGGTTAGGGACT

ACAAAAAATAGAGCTAAAGATCCTCTAGGTCGTCATACACATCACGAGAAAACGAAGCCC

AAATACGAAAGTAGCTCTAAATATAATCCTGACGCCACGAAAGCCAAGAAA

>795_Sample_ASV2

CACCGCGGTTATACGAAAGACCCTAGTTGATGGGTGCGGCGTAAAGGGTGGTTAGGGACT

ACAAAAAATAGAGCTAAAGATCCTCTAGGTCGTCATACACATCACGAGGAAACGAAGCCC

AAATACGAAAGTAGCTCTAAATATAATCCTGACGCCACGAAAGCCAAGAAA

>795_Sample_ASV3

CACCGCGGTTATACGAGAGACCCTAGTTGATTAAAGCGGCGTAAAGAGTGGTTATGGAAC

TATTCTTTTAAAGCAGAAAACCTCTCAAACTGTTATACGCACCCAGAGGTCTGAATCCCT

CACACGAAAGTGACTTTATTTATGCCTACCAGAACCCACGAAAGCTGGGACA

>795_Sample_ASV4

CACCGCGGTTATACGAAAGACCCTAGTTTATAGGTACGGCGTAAAGGGTGGTTAGGGGAC

AAACAAAATAAAGCCAAAGACCTCCTAAGCCGTCATACGCTCACGGAAGCACGAAGCCCA

AACACGAAAGTAGCTTTACCGAACATGCCCGACTCCACGAAAGCTGGGGAA

>795_Sample_ASV5

CACCGCGATTATACGAGAGGCCCAAGTTGACAGACATCGGCGTAAAGAGTGGTTAAGGAA

AAATTACTACTAAAGCCGAACACCTTCAGAACTGTCATACGTTCCCGAAGATAAGAAGCC

CCACCACGAAAGTGGCTTTATATTACCCGACCCCACGAAAGCTGTGAAA

>795_Sample_ASV6

CACCGCGGTTATACGAGAGGCCCAAGTTGACAGACACCGGCGTAAAGAGTGGCTAGGGAA

AAATTACTACTAAAGCCGAACACCTTCAGAACTGTCATACGTTCCCGAAGATAAGAAGCC

CCACCACGAAAGTGGCTTTATATTACCCGACCCCACGAAAGCTGTGAAA

>795_Sample_ASV7

CACCGCGGTTATACGAGAGGCCCAAGTTGACAGACACCGGCGTAAAGAGTGGCTAAGGAA

AAATTTCTACTAAAGCCGAACACCTTCAGAACTGTCATACGTTCCCGAAGATAAGAAGCC

CCACCACGAAAGTGGCTTTATATTACCCGACCCCACGAAAGCTGTGAAA

>795_Sample_ASV8

CACCGCGGTTATACGAGAGGCCCAAGTTGACAGACACCGGCGTAAAGAGTGGCTAAGGAA

AAATTTCTACTAAAGCCGAACACCTTCAGAACTGTCATACGTTCCCGAAGAAAAGAAGCC

CCACCACGAAAGTGGCTTTATATTACCCGACCCCACGAAAGCTGTGAAA

>795_Sample_ASV9

CACCGCGGTTAAACGAGAGACCCTAGTTGATCATCACGGCGTAAAGAGTGGTTAGGGGAT

TATCATAAATAAAGCCAAAGACCTCCCAAGCTGTCGCACGCATTCCGGAGGCGCGAAGCC

CACACACGAAAGTAGCTTTAACTATTGACCCTGATGCCACGAAAGCTAAGAAA

>795_Sample_ASV10

CACCGCGGTTATACGAAAGACCCTAGTTGATAGATCACGGCGTAAAGGGTGGTTAGGGAA

TACAAAAATAAAGCTAAAAATCCTCTAAGCTGTCATACGCTTCCCGAAGACATGAAACCC

AACCACGAAAGTAGCTTTAAACTCTCCTGACACCACGAAAGCCAAGGAA

>795_Sample_ASV11

CACCGCGGTTATACGAGAGGCCCAAGTTGATAAGCCCGGCGTAAAGAGTGGTTATGGAGA

ATAAAACACTAAAGCTAAAGACCCCCTAGGCTGTTATACGCACCTGGCGGCTCGAACCAC

CTATACGAAAGTAGCTTTACCCCCCTTCCACCAGAATCCACGACAGCTGGGGCA

>795_Sample_ASV12

CACCGCGGTTATACGAGAGGCCCAAGTTGACAGACATCGGCGTAAAGAGTGGTTAAGGAA

ACCCCATTACTAAAGCCGAACGCCCTCAGAACTGTCATACGTTCCCGAAGGTAAGAAGCC

CCACTACGAAAGTGGCTTTAACCACACCCGACCCCACGAAAGCTGTGAAA

>795_Sample_ASV13

CACCGCGGTTAAACGAGAGGCCCTAGTTGATATTACAACGGCGTAAAGGGTGGTTAAGGA

AAGCAAGCAATAAAGCCGAATGGCCCTTTGGCTGTCATACGCTTCTAGGTGTCCGAAGCC

CAATATACGAAAGTAGCTTTAGAAAAGCCCACCTGACACCACGAAAGCTGAGGAA

>795_Sample_ASV14

CACCGCGGTCACACGATTAACCCAAGTCAATAGAAGCCGGCGTAAAGAGTGTTTTAGATC

ACCCCCTCCCCAATAAAGCTAAAACTCACCTGAGTTGTAAAAAACTCCAGTTGACACAAA

ATAGACTACGAAAGTGGCTTTAACATATCTGAACACACAATAGCTAAGACC

>795_Sample_ASV15

CACCGCGGTTATACGAGAGGCCCTAGTTGATAAATACGGCGTAAAGAGTGGTTAGAGGAC

TCCCATAATAAAGCCAAAGACCCTCCGGGCCGTCACACGCATACGAGGGCACGAAGTTCA

CACACGAAAGTAGCTTTAAATTACCCGACGCCACGAAAGCTAAGAAA

>795_Sample_ASV16

CACCGCGGTTATACGAGAAACCCTAGTTGACAAATACGGCGTAAAGAGTGGTTAAGGGGT

ATCAAAAATAAAGTCAAACATTTTCCTAGTTGTTATAAGCATTTCGAGAACATGAAGCCC

AACCACGAAAGTAACTTTACCTTCACCTGACCCCACGAAAGCTAAGAAA

>795_Sample_ASV17

CACCGCGGTTATACGAAAGACCCTAGTTTATAAGTACGGCGTAAAGGGTGGTTAGGGGAC

AAACAAAATAAAGCCAAAGACCTCCTAAGCCGTCATACGCTTACGGAGGCACGAAGCCCA

AACACGAAAGTAGCTTTACCGAACATGCCCGACTCCACGAAAGCTGGGAAA

>795_Sample_ASV18

CACCGCGGTTATACGAGAGACCCTAGTTGATAAATACGGCGTAAAGAGTGGTTATGGGAA

AAACAAATAAAGTCAAACAACCTCTTAGCTGTTATACGCATTATGAGAGCATGAAGCCCC

CTCACGAAAGTAACTTTAATACCTCCTGACCCCACGAAAGCTAAGAAA

>795_Sample_ASV19

CACCGCGGTTATACGAGAGGCTCAAGTTGATAAACCCCGGCGTAAAGAGTGGTTAAGGAA

AAATAGAAACTAAAGCCGAATGCTTTCAAGACTGTTATACGTTCCCGAGAGTAAGAAGAC

CAATTACGAAAGTGGCTTTACTTTACCTGACCCCACGAAAGCTACGACA

>795_Sample_ASV20

CACCGCGGTTATACGAGAGACCCTAATTGATAGACACGGCGTAAAGAGTGGTTTGGGAAA

GCCCAAAAATAAAGCTAAAGACCTTCTAAGCCGTCACACGCACGCCGAAGGCACGAAGCC

CTGACACGAAAGTAGCTTTACTATTACCCCCGACGCCACGAAAGCTAAGAAA

>795_Sample_ASV21

AACCGCGGTTATACGAGAGACCCTAATTGATAGCTCACGGCGTAAAGGGTGGTTTGGGGA

AACCTCAAAATAAAGCTAAAGACCTTCTAAGCCGTTACACGCATACCGAAGGCACGAGGC

CCTAACACGAAAGTAGCTTTACTATTACCCCTGACGCCACGAAAGCTAAGAAA

>795_Sample_ASV22

CACCGCGGTTATACGAAAGACCCTAGTTGATGGATCACGGCGTAAAGGGTGGTTAAGGGA

TATAAAAATAAAGCTAAAAATCCTCTTAGCTGTCATACGCTTTCCGAAGACATGAGGCCC

AATTACGAAAGTAGCTTTAAACCACCCTGACACCACGAAAGCCAAGAAA

>795_Sample_ASV23

CACCGCGGTTAAACGAGAGGCCCTAGTTGATAGTACAACGGCGTAAAGGGTGGTTAAGGA

TAGTGAAATAATAAAGTCGAATGGCCCTTTGGCTGTCATACGCTTCTAGGAGTCCGAAGC

CCAATATACGAAAGTAACTTTAAGAGCGCCCACCTGACCCCACGAAAACTGAGAAA

>795_Sample_ASV24

CACCGCGGTCATACGATTAACCCAAGTTAATAGGCACACGGCGTAAAGCGTGTTTAAGCA

CTACGCCAAATAAAGTTAAACTCCAATTAAGCTGTAAAAAGCCATAATCAAGACGAAAAT

AAATAACGAAAGTAACTTTACAACCGCTGAAACACGATAGCTAAGATC

>795_Sample_ASV25

CACCGCGGTCATACGATTAACCCAAGTTAATAGGCACACGGCGTAAAGCGTGTTTAAGCA

CTATGCCAAATAAAGTTAAACTCCAATTAAGCTGTAAAAAGCCATAATCAAGACGAAAAT

AAATAACGAAAGTAACTTTACAACCGCTGAAACACGATAGCTAAGATC

>795_Sample_ASV26

CACCGCGGTTATACGAGAGGCCCAAGTTGATACTCCACGGCGTAAAGAGTGGTTAGAAAA

GACCTACTACTAAAGCCGAACGCCTTCAAAGCTGTTATACGCATCCGAAGGTGAGAAGCC

CACCCACGAAAGTGGCTTTACAACCTTGACTCCACGAAAGCTATGACA

>795_Sample_ASV27

CACCGCGGTTATACGAGAGACCCCAGTTGATAAACACGGCGTAAAGAGTGGTTAAGGGCA

CAAAACAAATAAAGTCAAACAACCTCTTAGCTGTTATACGCATTATGAGGATATGAAGCC

CCCCTACGAAAGTAACTTTACCAACCCCTGACCCCACGAAAGCTAAGAAA

>795_Sample_ASV28

CACCGCGGTTATACGAGAGACCCTAGTTGATATATACGGCGTAAAGAGTGGTTTGGAATA

ACCAAGTAATATAGCCAAAGACCTCCCAAGCCGTCACACGCACCCCGGAGGCACGAAGCC

CAAACACGAAAGTAGCTTTATTAACTTACCGACGCCACGAAAGCTAAGAAA

>795_Sample_ASV29

CACCGCGGTTATACGAAAGACCCTAGTTTATAGGCACGGCGTAAAGGGTGGTTAGGGGAT

AAACAAAATAAAGCCAAAGACCTTCTAAGTCGTCATACACTCATGAAGGCACGAAGCCCA

AATACGAAAGTAACTTTACAACTTAGACCCGACTCCACGAAAGCTGGGGGA

>795_Sample_ASV30

CACCGCGGTCATACGATTAACCCAAGTTAATAGGCACACGGCGTAAAGCGTGTTAAAGCA

CTATACTAAATAAAGTTAAATTCCAATTAAGCTGTAAAAAGCCATAATTGCAACAAAAAT

AAACAACGAAAGTAACTTTACAGTCGCTGAAACACGATAGCTAGGACC

>795_Sample_ASV31

CACCGCGGTTATACGAGAGGCCCAAGTTGATAGACATCGGCGTAAAGAGTGGTTAAGGAA

GTCTCAAAACTAAAGCCGAATGCCTTCAAGACTGTTATACGTTTCCGAAGGTAAGAAGCC

CCACTACGAAAGTGGCTTTACTCCACCTGACTCCACGAAAGCTGTGAAA

>795_Sample_ASV32

CACCGCGGTTATACGAGAGGCCCAAGTTGACAAACTCCGGCGTAAAGCGTGGTTAAGGCA

CACTCAAAAACTAAAGCCGAATATCTTCAGAGCAGTCATACGCATCCGAAGACACGAAGC

CCCACCACGAAAGTGGCTTTATGACACCTGACCCCACGAAAGCTAGGACA

>795_Sample_ASV33

CACCGCGGTTATACGAAAGACCCTAGTTGATAGACCACGGCGTAAAGGGTGGTTAAGGAA

CACAAAAATAAAGCTAAAGATCCTCTAAGCTGTCATACGCTTCCCGAAGACATGAAACCC

AATCACGAAAGTAGCTTTAAACTGTCCTGACACCACGAAAGCCAAGAAA

>795_Sample_ASV34

CACCGCGGTTATACGAAAGACCCTAGTTGATAGATCACGGCGTAAAGGGTGGTTAAGGAG

AACAAAAATAAAGCTAAAAATCCTCTAAGCTGTCATACGCTTTCCGAAGACATGAGACCC

AACCACGAAAGTAACTTTAAACTTTCCTGACACCACGAAAGCCAAGAAA

>797_Sample_ASV1

CACCGCGGTTATACGAAAGACCCTAGTTGATGGGTGCGGCGTAAAGGGTGGTTAGGGACT

ACAAAAAATAGAGCTAAAGATCCTCTAGGTCGTCATACACATCACGAGAAAACGAAGCCC

AAATACGAAAGTAGCTCTAAATATAATCCTGACGCCACGAAAGCCAAGAAA

>797_Sample_ASV2

CACCGCGGTTATACGAAAGACCCTAGTTGATGGGTGCGGCGTAAAGGGTGGTTAGGGACT

ACAAAAAATAGAGCTAAAGATCCTCTAGGTCGTCATACACATCACGAGGAAACGAAGCCC

AAATACGAAAGTAGCTCTAAATATAATCCTGACGCCACGAAAGCCAAGAAA

>797_Sample_ASV3

CACCGCGGTTAAACGAGAGGCCCTAGTTGATGGTGCAACGGCGTAAAGGGTGGTTAAGGA

AAGCAAGCAATAAAGCCGAATGGCCCTTTGGCTGTCATACGCTTCTAGGTGTCCGAAGCC

CAATATACGAAAGTAGCTTTAAGAAAGTCCACCTGACCCCACGAAAACTGAGGAA

>797_Sample_ASV4

CACCGCGGTTATACGAAAGACCCTAGTTGATTAAAGCGGCGTAAAGAGTGGTTATGGAAC

TATTCTTTTAAAGCAGAAAACCTCTCAAACTGTTATACGCACCCAGGGGTCTGAATCCCT

CACACGAAAGTGACTTTATTTATGCCTACCAGAACCCACGAAAGCTGGGACA

>797_Sample_ASV5

CACCGCGGTTATACGAGAGACCCTAGTTGATTAAAGCGGCGTAAAGAGTGGTTATGGAAC

TATTCTTTTAAAGCAGAAAACCTCTCAAACTGTTATACGCACCCAGGGGTCTGAATCCCT

CACACGAAAGTGACTTTATTTATGCCTACCAGAACCCACGAAAGCTGGGACA

>797_Sample_ASV6

CACCGCGGTTATACGAGAGACCCTAGTTGATTAAAGCGGCGTAAAGAGTGGTTATGGAAC

TATTCTTTTAAAGCAGAAAACCTCTCAAACTGTTACACGCACCCAGAGGTCTAAATCCCT

CACACGAAAGTGACTTTATTTATGCCTACCAGAACCCACGAAAGCTGGGACA

>797_Sample_ASV7

CACCGCGGTTATACGAGAGACCCTAGTTGATTAAAGCGGCGTAAAGAGTGGTTATGGAAC

TATTCTTTTAAAGCAGAAAACCTCTCAAACTGTTATACGCACCCAGAGGTCTGAATCCCT

CACACGAAAGTGACTTTATTTATGCCTACCAGAACCCACGAAAGCTGGGACA

>797_Sample_ASV8

CACCGCGGTTATACGAGAGGCCCAAGTTGACAGACACCGGCGTAAAGAGTGGCTAGGGAA

AAATTACTACTAAAGCCGAACACCTTCAGAACTGTCATACGTTCCCGAAGATAAGAAGCC

CCACCACGAAAGTGACTTTATATTACCCGACCCCACGAAAGCTGTGAAA

>797_Sample_ASV9

CATGGCGATTATACGAGAGGCCCAAGTTGACAGACACTGGCGTAAAGAGTGGCTAAGGAA

AAACTACTACTAAAGCCGAACACCTTCAGAACTGTCATACGTTCCCGAAGATAAGAAGCC

CCACCACGAAAGTGACTTTATATTACCCGACCCCACGAAAGCTGTGAAA

>797_Sample_ASV10

CACCGCGGTTATACGAGAGGCCCAAGTTGACAGACACCGGCGTAAAGAGTGGCTAGGGAA

AAATTACTACTAAAGCCGAACACCTTCAGAACTGTCATACGTTCCCGAAGATAAGAAGCC

CCACCACGAAAGTGACTTTATATTACCCGACCCCACGAAAGCTGGGGAA

>797_Sample_ASV11

CACCGCGGTTATACGAGAGGCCCAAGTTGACAGACACCGGCGTAAAGAGTGGCTAGGGAA

AAATTACTACTAAAGCCGAACACCTTCAGAACTGTCATACGTTCCCGAAGATAAGAAGCC

CCACCACGAAAGTGGCTTTATATTACCCGACCCCACGAAAGCTGTGAAA

>797_Sample_ASV12

CACCGCGGTTATACGAGAGGCCCAAGTTGACAGACACCGGCGTAAAGAGTGGCTAGGGAA

AAATTACTACTAAAGCCGAACACCCTCAGAACTGTCATACGTTCCCGAAGGTAAGAAGCC

CCACCACGAAAGTGGCTTTATAATACCCGACCCCACGAAAGCTGTGAAA

>797_Sample_ASV13

CACCGCGGTTATACGAGAGGCCCAAGTTGACAGACACCGGCGTAAAGAGTGGCTAAGGAA

AAATTTCTACTAAAGCCGAACACCTTCAGAACTGTCATACGTTCCCGAAGATAAGAAGCC

CCACCACGAAAGTGGCTTTATATTACCCGACACCACGAAAGCTGTGAAA

>797_Sample_ASV14

CACCGCGGTTATACGAGAGGCCCAAGTTGACAGACACCGGCGTAAAGAGTGGCTAAGGAA

AAATTTCTACTAAAGCCGAACACCTTCAGAACTGTCATACGTTCCCGAAGAAAAGAAGCC

CCACCACGAAAGTGGCTTTATATTACCCGACCCCACGAAAGCTGTGAAA

>797_Sample_ASV15

CACCGCGGTTATACGAGAGGCCCAAGTTGACAGACACCGGCGTAAAGAGTGGCTAAGGAA

AAATTTCTACTAAAGCCGAACACCTTCAGAACTGTCATACGTTCCCGAAGAAAAGAAGCC

CCACCACGAAAGTGGCTTTATATTACCCGACTCCACGAAAGCTGGGGAA

>797_Sample_ASV16

CACCGCGGTTATACGAGAGGCCCAAGTTGACAGACACCGGCGTAAAGAGTGGCTAAGGAA

AAATTTCTACCAAAGCCGAACACCTTCAGAACTGTCATACGTTCCCGAAGAAAAGAAGCC

CCACCACGAAAGTGGCTTTATATTACCCGACCCCACGAAAGCTGTGAAG

>797_Sample_ASV17

CACCGCGGTTATACAAGAGGCCCAAGTTGACAGACACCGGCGTAAAGAGTGGCTAAGGAA

AAATTTCTACTAAAGCCGAACACCTTCAGAACTGTCATACGTTCCCGAAGAAAAGAAGCC

CCACCACGAAAGTGGCTTTATATTACCCGACCCCACGAAAGCTGTGAAA

>797_Sample_ASV18

CACCGCGGTTATACGAAAGACTCTAGTTGACAAACACGGCGTAAAGGGTGGTTAGGATAA

CAACAAAATAAAGCTAAAGACTTACCAAGCCGTCATACGCCCATGTAAGAGCGAAAACCA

CAAACGAAAGTAACTTTATTACACCATCCGAATCCACGAAAGCTAAGAAA

>797_Sample_ASV19

CACCGCGGTTATACGAGAGACCCTAGTTGATATATACGGCGTAAAGAGTGGTTTGGGGTT

ATGCCTCCAAAATAAAGCCGAAGATCTCCCAAGCCGTCACACGCATCCCGGAGACACGAA

GCCCTAATACGAAGGTAGCTTTATTAATTCCCCGACGCCACGAAAGCTAAGAAA

>797_Sample_ASV20

CACCGCGGTTATACGAGAGACCCTAGTTGATATATACGGCGTAAAGAGTGGTTTGGGGTT

ATGCCTCCAAAATAAAGCCGAAGATCTCCCAAGCCGTCACACGCACCCCGGAGACACGAA

GCCCTAATACGAAGGTAGCTTTATTAATTCCCCGACGCCACGAAAGCTAAGAAA

>797_Sample_ASV21

CACCGCGGTTATACGAGAGACCCTAGTTGATATATACGGCGTAAAGAGTGGTTTGGGGTT

ATGCCTCCAAAATAAAGTCGAAGATCTCCCAAGCCGTCACACGCACCCCGGAGACACGAA

GCCCTAATACGAAGGTAGCTTTATTAATTCCCCGACGCCACGAAAGCTAAGAAA

>797_Sample_ASV22

CACCGCGGTTATACGAGAGACCCTAGTTGATATATACGGCGTAAAGAGTGGTTTGGGATA

TTTCTCCAAAATAAAGCCGAAGATCTCCCAAGCCGTCACACGCATCCCGGAGACACGAAG

CCCTAATACGAAGGTAGCTTTATTAGTACCCCGACGCCACGAAAGCTAAGAAA

>797_Sample_ASV23

CACCGCGGTTATACGAGAGACCCTAGTTGATATATACGGCGTAAAGAGTGGTTTGGGGTT

ATGCCTCCAAAATAAAGCCGAAGATCTCCCAAGCCGTCACACGCATCCCGGAGACACGAA

GCCCTAATACGAAGGTAGCTTTATTAGTACCCCGACGCCACGAAAGCTAAGAAA

>797_Sample_ASV24

CACCGCGGTTATACGAAAGACCCTAGTTGATAGATCACGGCGTAAAGGGTGGTTAGGGAA

TACAAAAATAAAGCTAAAAATCCTCTAAGCTGTCATACGCTTCCCGAAGACATGAAACCC

AACCACGAAAGTAGCTTTAAACTCTCCTGACACCACGAAAGCCAAGGAA

>797_Sample_ASV25

CACCGCGGTTATACGAAAGACCCTAGTTTATAAGCACGGCGTAAAGGGTGGTTAGGGAAT

ACAAAAATAAAGCTAAAAATCCTCTAAGCTGTCATACGCTTCCCGAAGACATGAAACCCA

ACCACGAAAGTAGCTTTAAACTCTCCTGACACCACGAAAGCCAAGGAA

>797_Sample_ASV26

CACCGCGGTTATACGAGAGGCCCAAGTTGATAAGCCCGGCGTAAAGAGTGGTTATGGAGA

ATAAAACACTAAAGCTAAAGACCCCCTAGGCTGTTATACGCACCTGGCGGCTCGAACCAC

CTATACGAAAGTAGCTTTACCCCCCTTCCACCAGAATCCACGACAGCTGGGGCA

>797_Sample_ASV27

CACCGCGGTTATACGAGAGGCCCTAGTTGATAGCCACGGCGCAAAGAGTGGTTAAGGAGC

CCCACTAAATAAAGTCGAACACTTCCTAGGCCGTTATACGTTTTCTAGAAGCACGAAACC

CAATTACACGAAAGCAACTTTATACTAAACAACCTGACCCCACGAAAGCTAAGAAA

>797_Sample_ASV28

CACCGCGGTTATACGAGAGGCCCAAGTTGACAGACATCGGCGTAAAGAGTGGTTAAGGAA

ACCCCATTACTAAAGCCGAACGCCCTCAGAACTGTCATACGTTCCCGAAGGTAAGAAGCC

CCACTACGAAAGTGGCTTTAACCACACCCGACCCCACGAAAGCTAAGAAA

>797_Sample_ASV29

CACCGCGGTTATACGAGAGGCCCAAGTTGACAGACATCGGCGTAAAGAGTGGTTAAGGAA

ACCCCATTACTAAAGCCGAACGCCCTCAGAACTGTCATACGTTCCCGAAGGTAAGAAGCC

CCACTACGAAAGTGGCTTTAACCACACCCGACCCCACGAAAGCTGTGAAA

>797_Sample_ASV30

CACCGCGGTTATACGAGAGGCCCCAGTTGATATTAGCGGCGTAAAGGGTGGTTAAGGACT

TTAATAACTAAAGCTAAACACTTCCCCGGCTGTTGTACGTAGACCGGAAGCTTGAAGCCC

AGACACGAAAGTAGCTTTATTCCCCCTGACCCCACGAAAGCTGAGAAA

>797_Sample_ASV31

CACCGCGGTTATACGAGAAACCCTAGTTGACAAATACGGCGTAAAGAGTGGTTAAGGGGT

ATCAAAAATAAAGTCAAACATTTTCCTAGTTGTTATAAGCATTTCGAGAACATGAAGCCC

AACCACGAAAGTAACTTTACCTTCACCTGACCCCACGAAAGCTAAGAAA

>797_Sample_ASV32

GACCGCGGTTATACGAGAGACTCTAGTTGATAGATACGGCGTAAAGAGTGGTTTGGGATA

AACTATAAATAAAGCCAAAGACCTTCCAAGCTGTTAAACGCATACCGAAGGCACGAAGCC

CCAACACGAAAGTAGCTTTACTACTACCCCCGATGCCACGAAAGCTAAGACA

>797_Sample_ASV33

CACCGCGGTTATACGAAAGACCCCAGTTTATAGACACGGCGTAAAGGGTGGTTATAGGAC

AAACTAACATAGAGCCAAAGACCCCCTAAGCCGTCATACGCTCACGAGGGCTCGAAGCCC

CTATACGAAAGTAGCTCTACCAAAAAATGCTCGACTCCACGAAAGCTGGGACA

>797_Sample_ASV34

CACCGCGGTTATACGAGAGACCCTAATTGATAGACACGGCGTAAAGAGTGGTTTGGGAAA

GCCCAAAAATAAAGCTAAAGACCTTCTAAGCCGTCACACGCACGCCGAAGGCACGAAGCC

CTGACACGAAAGTAGCTTTACTATTACCCCCGACGCCACGAAAGCTAAGAAA

>797_Sample_ASV35

CACCGCGGTTATACGAAAGACCCTAGTTGATGGATCACGGCGTAAAGGGTGGTTAAGGGA

TATAAAAATAAAGCTAAAAATCCTCTTAGCTGTCATACGCTTTCCGAAGACATGAGGCCC

AATTACGAAAGTAGCTTTAAACCACCCTGACACCACGAAAGCCAAGAAA

>797_Sample_ASV36

CACCGCGGTTATACGAAAGACCCCAGTTTATAGATGCGGCGTAAAGGGTGGTTAAGAGAA

AATCAAAATAAGGCCAAAGATCCCCTAAGCAGTCATACGCTCCACGAGGACACGAAACCC

AAATACGAAAGTTGCCTTAATAAACTTCACCTGATCCCACGAAAGCTGAAAAA

>797_Sample_ASV37

CACCGCGGTTATACGAGAGGCCCTAGTTGATAACTACCGGCGTAAAGAGTGGTTATGGAA

AAATATTTAATAAAGCCGAACACCCCCTCAGCCGTCATACGCACCTGGGAGCACGAAGAC

CTACTGCGAAAGCAGCTTTAATTATGCCTGACCCCACGACAGCTAAGAAA

>797_Sample_ASV38

CACCGCGGTTATACGAGAGGCCCAAGTTGATTATTACCGGCGTAAAGCGTGGTTAAGATT

TTTATAGGACTAAAGCCGAACCCCCTCAGAGCTGTTATACGCACCCGAAGGTTAGAAGTT

CAATCACGAAAGTGGCTTTACAACCCCGAACCCACGAAAGCTAAGATA

>797_Sample_ASV39

CACCGCGGTTATACGAGAGACCCTAATTGATAGGCGCGGCGTAAAGAGTGGTTTAGGACT

AAAACTAATAAAGCTAAAGATCCTCCCAGCCGTCGCACGCATTCTGAGGACACGAAACTC

AACCACGAAAGTAGCTTTAACATACTCTCCTGACCCCACGAAAGCTAAGAAA

>797_Sample_ASV40

CACCGCGGTTATACGAAAGACCCTAGTTGATAGGCACGGCGTAAAGGGTGGTTAGGGATA

TATCAAAAATAAAGTTAAAGAGCCTCTAAGCTGTCGCACGCATCCCGAGAGCTCGAGACC

CAGACACGAAAGTAACTTTAAAATACCTCACCTGACCCCACGAAAGCTAAGAAA

>797_Sample_ASV41

CACCGCGGTTATACGAAAGACCCTAGTTGATAGCCACGGCGTAAAGGGTGGTTAAGGTAT

ACAACAAATAAAGCTAAATAGCCTCTAGGCCGTCGCACGCATTCCGAGAGCTCGAAACCC

AAAAACGAAAGTAGCTTTAAAATAAACAGACCTGACCCCACGAAAGCTAAGAAA

>797_Sample_ASV42

CACCGCGGTTATACGAGAGGCCCAAGTTGATACTCCACGGCGTAAAGAGTGGTTAGAAAA

GACCTACTACTAAAGCCGAACGCCTTCAAAGCTGTTATACGCATCCGAAGGTGAGAAGCC

CACCCACGAAAGTGGCTTTACAACCTTGACTCCACGAAAGCTATGACA

>797_Sample_ASV43

CACCGCGGTTATACGAGAGACCCTAGTTGATATATACGGCGTAAAGAGTGGTTTGGAATA

ACCAAGTAATATAGCCAAAGACCTCCCAAGCCGTCACACGCACCCCGGAGGCACGAAGCC

CAAACACGAAAGTAGCTTTATTAACTTACCGACGCCACGAAAGCTAAGAAA

>797_Sample_ASV44

CACCGCGGTTATACGAAAGACCCTAGTTTATAGGCACGGCGTAAAGGGTGGTTAGGGGAT

AAACAAAATAAAGCCAAAGACCTTCTAAGTCGTCATACACTCATGAAGGCACGAAGCCCA

AATACGAAAGTAACTTTACAACTTAGACCCGACTCCACGAAAGCTGGGGGA

>797_Sample_ASV45

CACCGCGGTTATACGAGAGACCCTAATTAATAGCTACGGCGTAAAGAGTGGTTTAGGACA

AAAATTAATAAAGCCGAAGTTTCTCCTAGCCGTTGTACGCACTCTGAGAACACGAAGCCC

CACTACGAAAGTAGCTTTACCTATTATTTCCTGACCCCACGAAAGCTAAGAAA

>797_Sample_ASV46

CACCGCGGTTATACGAGAGACCCTAATTAATAGATACGGCGTAAAGAGTGGTTTAGGACA

AAAATTAATAAAGCCGAAGTTTCTCCTAGCCGTTGTACGCACTCTGAGAACACGAAGCCC

CACTACGAAAGTAGCTTTACCTATTATTTCCTGACCCCACGAAAGCTAAGAAA

>797_Sample_ASV47

CACCGCGGTTATACGAGAGACCCTAATTAATAGCTACGGCGTAAAGAGTGGTTTAGGGTA

AAAATTAATAAAGCCGAAGTTTCTCCTAGCCGTTGTACGCACTCTGAGAACACGAAGCCC

CACTACGAAAGTAGCTTTACCTATTATTTCCTGACCCCACGAAAGCTAAGAAA

>797_Sample_ASV48

CACCGCGGTTATACGAGAGACCCTAATTGATAGACTACGGCGTAAAGAGTGGTTTAGAAC

AAAAATTAATAAAGCCAAAGATCCTCCTGGCCGTCGCACGCATTTCGAGGATACGAAGCC

CCACTACGAAAGTAGCTTTACTACAACTTTTCTGACCCCACGAAAGCTAAGAAA

>797_Sample_ASV49

CACCGCGGTTATACGAGAGACCCTAATTGATAGACTACGGCGTAAAGAGTGGTTTAGAAC

AAAAATTAATAAAGCCAAAGATCCTCCTGGCCGTCGCACGCATTTCGAGGATACGAAGCC

CCACTACGAAAGTAGCTTTACTACAACTTTTCTGACCCCACGAAAGCTGGGGAA

>797_Sample_ASV50

CACCGCGGTTATACGAAAGACCCTAGTTGATAGATCACGGCGTAAAGGGTGGTTAAGGAA

AACAAAAATAAAGCTAAAAATCCTCTAAGCTGTCATACGCTTTCCGAAGACATGAGATCC

AACCACGAAAGTAGCTTTAAAATTTCCTGACACCACGAAAGCCAAGGAA

>798_Sample_ASV1

CACCGCGGTTATACGAAAGACCCTAGTTGATGGGTGCGGCGTAAAGGGTGGTTAGGGACT

ACAAAAAATAGAGCTAAAGATCCTCTAGGTCGTCATACACATCACGAGAAAACGAAGCCC

AAATACGAAAGTAGCTCTAAATATAATCCTGACGCCACGAAAGCCAAGAAA

>798_Sample_ASV2

CACCGCGGTTATACGAAAGACCCTAGTTGATGGGTGCGGCGTAAAGGGTGGTTAGGGACT

ACAAAAAATAGAGCTAAAGATCCTCTAGGTCGTCATACACATCACGAGGAAACGAAGCCC

AAATACGAAAGTAGCTCTAAATATAATCCTGACGCCACGAAAGCCAAGAAA

>798_Sample_ASV3

CACCGCGGTTATACGAGAGGCCCAAGTTGACAGACACCGGCGTAAAGAGTGGCTAGGGAA

AAATTACTACTAAAGCCGAACACCTTCAGAACTGTCATACGTTCCCGAAGATAAGAAGCC

CCACCACGAAAGTGACTTTATATTACCCGACCCCACGAAAGCTGGGGGA

>798_Sample_ASV4

CACCGCGGTTATACGAGAGGCCCAAGTTGACAGACACCGGCGTAAAGAGTGGCTAGGGAA

AAATTACTACTAAAGCCGAACACCTTCAGAACTGTCATACGTTCCCGAAGATAAGAAGCC

CCACCACGAAAGTGACTTTATATTACCCGACCCCACGAAAGCTGTGAAA

>798_Sample_ASV5

CACCGCGGTTATACGAGAGGCCCAAGTTGACAGACACCGGCGTAAAGAGTGGCTAGGGAA

AAATTACTACTAAAGCCGAACACCTTCAGAACTGTCATACGTTCCCGAAGATAAGAAGCC

CCACCACGAAAGTGGCTTTATATTACCCGACCCCACGAAAGCTGTGAAA

>798_Sample_ASV6

CACCGCGGTTATACGAAAGACTCTAGTTGACAAACACGGCGTAAAGGGTGGTTAGGATAA

CAACAAAATAAAGCTAAAGACTTACCAAGCCGTCATACGCCCATGTAAGAGCGAAAACCA

CAAACGAAAGTAACTTTATTACACCATCCGAATCCACGAAAGCTAAGAAA

>798_Sample_ASV7

CACCGCGGTTATACGAGAGGCCCAAGTTGATAAGCCCGGCGTAAAGAGTGGTTATGGAGA

ATAAAACACTAAAGCTAAAGACCCCCTAGGCTGTTATACGCACCTGGCGGCTCGAACCAC

CTATACGAAAGTAGCTTTACCCCCCTTCCACCAGAATCCACGACAGCTGGGGCA

>798_Sample_ASV8

CACCGCGGTTATACGAGAGGCCCTAGTTGATAGCCACGGCGCAAAGAGTGGTTAAGGAGC

CCCACTAAATAAAGTCGAACACTTCCTAGGCCGTTATACGTTTTCTAGAAGCACGAAACC

CAATTACACGAAAGCAACTTTATACTAAACAACCTGACCCCACGAAAGCTAAGAAA

>798_Sample_ASV9

CACCGCGGTTATACGAGAGGCCCAAGTTGACAGACATCGGCGTAAAGAGTGGTTAAGGAA

ACCCCATTACTAAAGCCGAACGCCCTCAGAACTGTCATACGTTCCCGAAGGTAAGAAGCC

CCACTACGAAAGTGGCTTTAACCACACCCGACCCCACGAAAGCTAAGAAA

>798_Sample_ASV10

CACCGCGGTTATACGAGAGGCCCAAGTTGACAGACATCGGCGTAAAGAGTGGTTAAGGAA

ACCCCATTACTAAAGCCGAACGCCCTCAGAACTGTCATACGTTCCCGAAGGTAAGAAGCC

CCACTACGAAAGTGGCTTTAACCACACCCGACCCCACGAAAGCTGTGAAA

>798_Sample_ASV11

GACCGCGGTTATACGAGAGACTCTAGTTGATAGATACGGCGTAAAGAGTGGTTTGGGATA

AACTATAAATAAAGCCAAAGACCTTCCAAGCTGTTAAACGCATACCGAAGGCACGAAGCC

CCAACACGAAAGTAGCTTTACTACTACCCCCGATGCCACGAAAGCTAAGACA

>798_Sample_ASV12

CACCGCGGTTATACGAAAGACCCCAGTTTATAGACACGGCGTAAAGGGTGGTTATAGGAC

AAACTAACATAGAGCCAAAGACCCCCTAAGCCGTCATACGCTCACGAGGGCTCGAAGCCC

CTATACGAAAGTAGCTCTACCAAAAAATGCTCGACTCCACGAAAGCTGGGACA

>798_Sample_ASV13

AACCGCGGTTATACGAGAGACCCTAATTGATAGCTCACGGCGTAAAGGGTGGTTTGGGGA

AACCTCAAAATAAAGCTAAAGACCTTCTAAGCCGTTACACGCATACCGAAGGCACGAGGC

CCTAACACGAAAGTAGCTTTACTATTACCCCTGACGCCACGAAAGCTAAGAAA

>798_Sample_ASV14

CACCGCGGTTATACGAAAGACCCTAGTTGATGGATCACGGCGTAAAGGGTGGTTAAGGGA

TATAAAAATAAAGCTAAAAATCCTCTTAGCTGTCATACGCTTTCCGAAGACATGAGGCCC

AATTACGAAAGTAGCTTTAAACCACCCTGACACCACGAAAGCCAAGAAA

>798_Sample_ASV15

CACCGCGGTTATACGAGAGGCCCTAGTTGATAACTACCGGCGTAAAGAGTGGTTATGGAA

AAATATTTAATAAAGCCGAACACCCCCTCAGCCGTCATACGCACCTGGGAGCACGAAGAC

CTACTGCGAAAGCAGCTTTAATTATGCCTGACCCCACGACAGCTAAGAAA

>798_Sample_ASV16

CACCGCGGTTATACGAAAGACCCTAGTTTATAGACACGGCGTAAAGGGTGGTTCGGGACT

AAACAAAATAAAGCCAAAGACCTCCTAAGCCGTCATACGCTCACGGAAGCACGAAGCCCA

AGTACGAAAGTAGCTTTACAAAACATGCCTGACTCCACGAAAGCTAGGAAA

>798_Sample_ASV17

CACCGCGGTTATACGAGAGGCCCAAGTTGATTATTACCGGCGTAAAGCGTGGTTAAGATT

TTTATAGGACTAAAGCCGAACCCCCTCAGAGCTGTTATACGCACCCGAAGGTTAGAAGTT

CAATCACGAAAGTGGCTTTACAACCCCGAACCCACGAAAGCTAAGATA

>798_Sample_ASV18

CACCGCGGTTATACGAGAGACCCTAATTGATAGGCGCGGCGTAAAGAGTGGTTTAGGACT

AAAACTAATAAAGCTAAAGATCCTCCCAGCCGTCGCACGCATTCTGAGGACACGAAACTC

AACCACGAAAGTAGCTTTAACATACTCTCCTGACCCCACGAAAGCTAAGAAA

>798_Sample_ASV19

CACCGCGGTTATACGAAAGACCCTAGTTGATAGGCACGGCGTAAAGGGTGGTTAGGGATA

TATCAAAAATAAAGTTAAAGAGCCTCTAAGCTGTCGCACGCATCCCGAGAGCTCGAGACC

CAGACACGAAAGTAACTTTAAAATACCTCACCTGACCCCACGAAAGCTAAGAAA

>798_Sample_ASV20

CACCGCGGTTATACGAGAGACCCCAGTTGATAAACACGGCGTAAAGAGTGGTTAAGGGCA

CAAAACAAATAAAGTCAAACAACCTCTTAGCTGTTATACGCATTATGAGGATATGAAGCC

CCCCTACGAAAGTAACTTTACCAACCCCTGACCCCACGAAAGCTAAGAAA

>798_Sample_ASV21

CACCGCGGTTATACGAGAGACCCTAGTTGATATATACGGCGTAAAGAGTGGTTTGGAATA

ACCAAGTAATATAGCCAAAGACCTCCCAAGCCGTCACACGCACCCCGGAGGCACGAAGCC

CAAACACGAAAGTAGCTTTATTAACTTACCGACGCCACGAAAGCTAAGAAA

>798_Sample_ASV22

CACCGCGGTTATACGAAAGACCCTAGTTTATAGGCACGGCGTAAAGGGTGGTTAGGGGAT

AAACAAAATAAAGCCAAAGACCTTCTAAGTCGTCATACACTCATGAAGGCACGAAGCCCA

AATACGAAAGTAACTTTACAACTTAGACCCGACTCCACGAAAGCTGGGGGA

>798_Sample_ASV23

CACCGCGGTTATACGAGAGACCCTAATTAATAGATACGGCGTAAAGAGTGGTTTAGGACA

AAAATTAATAAAGCCGAAGTTTCTCCTAGCCGTTGTACGCACTCTGAGAACACGAAGCCC

CACTACGAAAGTAGCTTTACCTATTATTTCCTGACCCCACGAAAGCTAAGAAA

>798_Sample_ASV24

CACCGCGGTTATACGAGAGACCCTAATTAATAGCTACGGCGTAAAGAGTGGTTTAGGGTA

AAAATTAATAAAGCCGAAGTTTCTCCTAGCCGTTGTACGCACTCTGAGAACACGAAGCCC

CACTACGAAAGTAGCTTTACCTATTATTTCCTGACCCCACGAAAGCTAAGAAA

>798_Sample_ASV25

CACCGCGGTTATACGAGAGACCCTAATTGATAGACTACGGCGTAAAGAGTGGTTTAGAAC

AAAAATTAATAAAGCCAAAGATCCTCCTGGCCGTCGCACGCATTTCGAGGATACGAAGCC

CCACTACGAAAGTAGCTTTACTACAACTTTTCTGACCCCACGAAAGCTAAGAAA

>798_Sample_ASV26

CACCGCGGTTATACGAAAGACCCTAGTTGATAGATCACGGCGTAAAGGGTGGTTAAGGAG

AACAAAAATAAAGCTAAAAATCTTCTAAGCTGTCATACGCTTTCCGAAGACATGAGACCC

AACCACGAAAGTAGCTTTAAACTTTCCTGACACCACGAAAGCCAAGAAA

>798_Sample_ASV27

CACCGCGGTTATACGAAAGACCCTAGTTGATAGATCACGGCGTAAAGGGTGGTTAAGGAA

AACAAAAATAAAGCTAAAAATCCTCTAAGCTGTCATACGCTTTCCGAAGACATGAGATCC

AACCACGAAAGTAGCTTTAAAATTTCCTGACACCACGAAAGCCAAGGAA

>801_Sample_ASV1

CACCGCGGTTATACGAAAGACCCTAGTTGATGGGTGCGGCGTAAAGGGTGGTTAGGGACT

ACAAAAAATAGAGCTAAAGATCCTCTAGGTCGTCATACACATCACGAGGAAACGAAGCCC

AAATACGAAAGTAGCTCTAAATATAATCCTGACGCCACGAAAGCCAAGAAA

>801_Sample_ASV2

CACCGCGGTTATACGAAAGACCCTAGTTGATAGCCACGGCGTAAAGGGTGGTTAAGGAAC

CTAAAAAATAAAGCTAAAGACCCTCTAGGCCGTCATACGCATTACGAGGCAAACGAAACC

CCAACACGAAAGTAGCTTTAGACAAATATCCTGATGCCACGAAAGCCAAGAAA

>801_Sample_ASV3

CACCGCGGTTATACGAGAGGCCCAAGTTGACAGACACCGGCGTAAAGAGTGGCTAGGGAA

AAATTACTACTAAAGCCGAACACCTTCAGAACTGTCATACGTTCCCGAAGATAAGAAGCC

CCACCACGAAAGTGACTTTATATTACCCGACCCCACGAAAGCTGTGAAA

>801_Sample_ASV4

CACCGCGGTTATACGAGAGGCCCAAGTTGACAGACACCGGCGTAAAGAGTGGCTAGGGAA

AAATTACTACTAAAGCCGAACACCTTCAGAACTGTCATACGTTCCCGAAGATAAGAAGCC

CCACCACGAAAGTGACTTTATATTACCCGACCCCACGAAAGCTAAGAAA

>801_Sample_ASV5

CACCGCGGTTATACGAGAGGCCCAAGTTGACAGACACCGGCGTAAAGAGTGGCTAGGGAA

AAATTACTACTAAAGCCGAACACCTTCAGAACTGTCATACGTTCCCGAAGATAAGAAGCC

CCACCACGAAAGTGACTTTATATTACCCGACCCCACGAAAGCCAAGAAA

>801_Sample_ASV6

CACCGCGGTTATACGAGAGGCCCAAGTTGACAGACACCGGCGTAAAGAGTGGCTAGGGAA

AAATTACTACTAAAGCCGAACACCTTCAGAACTGTCATACGTTCCCGAAGATAAGAAGCC

CCACCACGAAAGTGGCTTTATATTACCCGACCCCACGAAAGCTGTGAAA

>801_Sample_ASV7

CACCGCGGTTATACGAGAGGCCCAAGTTGACAGACACCGGCGTAAAGAGTGGCTAGGGAA

AAATTACTACTAAAGCCGAACACCCTCAGAACTGTCATACGTTCCCGAAGGTAAGAAGCC

CCACCACGAAAGTGGCTTTATAATACCCGACCCCACGAAAGCTGTGAAA

>801_Sample_ASV8

CACCGCGGTTATACGAGAGGCCCAAGTTGACAGACACCGGCGTAAAGAGTGGCTAGGGAA

AAATTACTACTAAAGCCGAACACCTTCAGAACTGTCATACGTTCCCGAAGATAAGAAGCC

CCACCACGAAAGTGGCTTTATATTACCCGACCCCACGAAAGCTAAGAAA

>801_Sample_ASV9

CACCGCGGTTATACGAGAGGCCCAAGTTGACAGACACCGGCGTAAAGAGTGGCTAAGGAA

AAATTTCTACTAAAGCCGAACACCTTCAGAACTGTCATACGTTCCCGAAGATAAGAAGCC

CCACCACGAAAGTGGCTTTATATTACCCGACACCACGAAAGCTGTGAAA

>801_Sample_ASV10

CACCGCGGTTATACGAGAGGCCCAAGTTGATAAGCCCGGCGTAAAGAGTGGTTATGGAGA

ATAAAACACTAAAGCTAAAGACCCCCTAGGCTGTTATACGCACCTGGCGGCTCGAACCAC

CTATACGAAAGTAGCTTTACCCCCCTTCCACCAGAATCCACGACAGCTGGGGCA

>801_Sample_ASV11

CACCGCGGTTATACGAGAGGCCCAAGTTGACAGACATCGGCGTAAAGAGTGGTTAAGGAA

ACCCCATTACTAAAGCCGAACGCCCTCAGAACTGTCATACGTTCCCGAAGGTAAGAAGCC

CCACTACGAAAGTGGCTTTAACCACACCCGACCCCACGAAAGCTGTGAAA

>801_Sample_ASV12

CACCGCGGTTATACGAGAGGCCCAAGTTGACAGACATCGGCGTAAAGAGTGGTTAAGGAA

ACCCCATTACTAAAGCCGAACGCCCTCAGAACTGTCATACGTTCCCGAAGGTAAGAAGCC

CCACTACGAAAGTGGCTTTAACCACACCCGACCCCACGAAAGCTAAGAAA

>801_Sample_ASV13

CACCGCGGTTATACGAGAGGCCCAAGTTGACAGACACCGGCGTAAAGAGTGGTTAAGGAA

ATTCATCAACTAAAGCCGAATGCCCTCAGGACTGTTATACGTTTGCGAAGGTAAGAAGCC

CTACTACGAAAGTGGCTTTATTCCACCTGATTCCACGAAAGCTGCGGAA

>801_Sample_ASV14

CACCGCGGTTATACGAGAGGCCCCAGTTGATATTAGCGGCGTAAAGGGTGGTTAAGGACT

TTAATAACTAAAGCTAAACACTTCCCCGGCTGTTGTACGTAGACCGGAAGCTTGAAGCCC

AGACACGAAAGTAGCTTTATTCCCCCTGACCCCACGAAAGCTGAGAAA

>801_Sample_ASV15

CACCGCGGTTATACGAGAGACCCCAGTTGATAGCTACGGCGTAAAGAGTGGTTAAGGACC

CCATTTAATAAAGCCAAAGACCCCCCAAGCCGTCATACGCACACGAGGGCGCGAAGCTCG

CACACGAAAGTAGCTCTAATTTACCCGACGCCACGAAAGCTAAGGAA

>801_Sample_ASV16

CACCGCGGTTATACGAAAGACCCTAGTTTATAAGTACGGCGTAAAGGGTGGTTAGGGGAC

AAACAAAATAAAGCCAAAGACCTCCTAAGCCGTCATACGCTTACGGAGGCACGAAGCCCA

AACACGAAAGTAGCTTTACCGAACATGCCCGACTCCACGAAAGCTGGGAAA

>801_Sample_ASV17

CACCGCGGTTATACGAGAGACCCTAGTTGATAAATACGGCGTAAAGAGTGGTTATGGGAA

AAACAAATAAAGTCAAACAACCTCTTAGCTGTTATACGCATTATGAGAGCATGAAGCCCC

CTCACGAAAGTAACTTTAATACCTCCTGACCCCACGAAAGCTAAGAAA

>801_Sample_ASV18

CACCGCGGTTATACGAGAGACCCTAATTGATAGACACGGCGTAAAGAGTGGTTTGGGAAA

GCCCAAAAATAAAGCTAAAGACCTTCTAAGCCGTCACACGCACGCCGAAGGCACGAAGCC

CTGACACGAAAGTAGCTTTACTATTACCCCCGACGCCACGAAAGCTAAGAAA

>801_Sample_ASV19

AACCGCGGTTATACGAGAGACCCTAATTGATAGCTCACGGCGTAAAGGGTGGTTTGGGGA

AACCTCAAAATAAAGCTAAAGACCTTCTAAGCCGTTACACGCATACCGAAGGCACGAGGC

CCTAACACGAAAGTAGCTTTACTATTACCCCTGACGCCACGAAAGCTAAGAAA

>801_Sample_ASV20

CACCGCGGTTATACGAAAGACCCTAGTTGATGGATCACGGCGTAAAGGGTGGTTAAGGGA

TATAAAAATAAAGCTAAAAATCCTCTTAGCTGTCATACGCTTTCCGAAGACATGAGGCCC

AATTACGAAAGTAGCTTTAAACCACCCTGACACCACGAAAGCCAAGAAA

>801_Sample_ASV21

CACCGCGGTTATACGAAAGACCCTAGTTTATAGATGCGGCGTAAAGGGTGGTTAAGGAAA

ACCTAAAATAATGCTAAAGACTCTCAAAGCTGTTATACGCTTCATGAGCACACGAAATCC

AAATACGAAAGTTGCCTTAATAAACTTTGCCTGACCCCACGAAAGCTGAAAAA

>801_Sample_ASV22

CACCGCGGTTATACGAGAGACCCTAGTTGATAGCTACGGCGTAAAGCGTGGTTAGGAGAA

CAAGATAAATAAAGTCAAACAATCTCTCGGCCGTTATACGTTATCTGAGAATATGAAGTC

CTACCACGAAAGTAACTTTAATTTTTCTGACCCCACGAAAACTGAGGAA

>801_Sample_ASV23

CACCGCGGTTAAACGAGAGGCCCTAGTTGATAGTACAACGGCGTAAAGGGTGGTTAAGGA

TAGTGAAATAATAAAGTCGAATGGCCCTTTGGCTGTCATACGCTTCTAGGAGTCCGAAGC

CCAATATACGAAAGTAACTTTAAGAGCGCCCACCTGACCCCACGAAAACTGAGAAA

>801_Sample_ASV24

CACCGCGGTTATACGAAAGACCCTAGTTTATAGACACGGCGTAAAGGGTGGTTCGGGACT

AAACAAAATAAAGCCAAAGACCTCCTAAGCCGTCATACGCTCACGGAAGCACGAAGCCCA

AGTACGAAAGTAGCTTTACAAAACATGCCTGACTCCACGAAAGCTAGGAAA

>801_Sample_ASV25

CACCGCGGTTATACGAAAGACCCTAGTTGATAGGCACGGCGTAAAGGGTGGTTAGGGATA

TATTAAAAATAAAGTTAAAGAGCCTCTAAGCTGTCGCACGCATCCCGAGAGCTCGAGACC

CAGACACGAAAGTAACTTTAAAATACCTCACCTGACCCCACGAAAGCTAAGAAA

>801_Sample_ASV26

CACCGCGGTTATACGAGAGACCCCAGTTGATAAACACGGCGTAAAGAGTGGTTAAGGGCA

CAAAACAAATAAAGTCAAACAACCTCTTAGCTGTTATACGCATTATGAGGATATGAAGCC

CCCCTACGAAAGTAACTTTACCAACCCCTGACCCCACGAAAGCTAAGAAA

>801_Sample_ASV27

CACCGCGGTTATACGAGAGACCCTAGTTGATATATACGGCGTAAAGAGTGGTTTGGAATA

ACCAAGTAATATAGCCAAAGACCTCCCAAGCCGTCACACGCACCCCGGAGGCACGAAGCC

CAAACACGAAAGTAGCTTTATTAACTTACCGACGCCACGAAAGCTAAGAAA

>801_Sample_ASV28

CACCGCGGTTATACGAAAGACCCTAGTTTATAGGCACGGCGTAAAGGGTGGTTAGGGGAT

AAACAAAATAAAGCCAAAGACCTTCTAAGTCGTCATACACTCATGAAGGCACGAAGCCCA

AATACGAAAGTAACTTTACAACTTAGACCCGACTCCACGAAAGCTAAGAAA

>801_Sample_ASV29

CACCGCGGTTATACGAAAGACCCTAGTTTATAGGCACGGCGTAAAGGGTGGTTAGGGGAT

AAACAAAATAAAGCCAAAGACCTTCTAAGTCGTCATACACTCATGAAGGCACGAAGCCCA

AATACGAAAGTAACTTTACAACTTAGACCCGACTCCACGAAAGCTGGGGGA

>801_Sample_ASV30

CACCGCGGTTATACGAGAGACCCTAATTGATAGACTACGGCGTAAAGAGTGGTTTAGAAC

AAAAATTAATAAAGCCAAAGATCCTCCTGGCCGTCGCACGCATTTCGAGGATACGAAGCC

CCACTACGAAAGTAGCTTTACTACAACTTTTCTGACCCCACGAAAGCTAAGAAA

>801_Sample_ASV31

CACCGCGGTTATACGAAAGACCCTAGTTGATAGATCACGGCGTAAAGGGTGGTTAAGGAA

AACAAAAATAAAGCTAAAAATCCTCTAAGCTGTCATACGCTTTCCGAAGACATGAGATCC

AACCACGAAAGTAGCTTTAAAATTTCCTGACACCACGAAAGCCAAGGAA

>801_Sample_ASV32

CACCGCGGTTATACGAAAGACCCTAGTTGATAGATCACGGCGTAAAGGGTGGTTAAGGAG

AACAAAAATAAAGCTAAAAATCCTCTAAGCTGTCATACGCTTTCCGAAGACATGAGACCC

AACCACGAAAGTAACTTTAAACTTTCCTGACACCACGAAAGCCAAGAAA

>803_Sample_ASV1

CACCGCGGTTATACGAAAGACCCTAGTTGATGGGTGCGGCGTAAAGGGTGGTTAGGGACT

ACAAAAAATAGAGCTAAAGATCCTCTAGGTCGTCATACACATCACGAGGAAACGAAGCCC

AAATACGAAAGTAGCTCTAAATATAATCCTGACGCCACGAAAGCCAAGAAA

>803_Sample_ASV2

CACCGCGGTTATACGAGAGACCCTAGTTGATTAAAGCGGCGTAAAGAGTGGTTATGGAAC

TATTCTTTTAAAGCAGAAAACCTCTCAAACTGTTATACGCACCCAGAGGTCTGAATCCCT

CACACGAAAGTGACTTTATTTATGCCTACCAGAACCCACGAAAGCTGGGACA

>803_Sample_ASV3

CACCGCGGTTATACGAAAGACCCTAGTTTATAGGTACGGCGTAAAGGGTGGTTAGGGGAC

AAACAAAATAAAGCCAAAGACCTCCTAAGCCGTCATACGCTCACGGAAGCACGAAGCCCA

AACACGAAAGTAGCTTTACCGAACATGCCCGACTCCACGAAAGCTGGGGAA

>803_Sample_ASV4

CACCGCGGTTATACGAGAGGCCCAAGTTGACAGACACCGGCGTAAAGAGTGGCTAGGGAA

AAATTACTACTAAAGCCGAACACCTTCAGAACTGTCATACGTTCCCGAAGATAAGAAGCC

CCACCACGAAAGTGACTTTATATTACCCGACCCCACGAAAGCTGTGAAA

>803_Sample_ASV5

CACCGCGATTATACGAGAGGCCCAAGTTGACAGACATCGGCGTAAAGAGTGGTTAAGGAA

AAATTACTACTAAAGCCGAACACCTTCAGAACTGTCATACGTTCCCGAAGATAAGAAGCC

CCACCACGAAAGTGGCTTTATATTACCCGACCCCACGAAAGCTGTGAAA

>803_Sample_ASV6

CACCGCGGTTATACGAGAGGCCCAAGTTGACAGACACCGGCGTAAAGAGTGGCTAGGGAA

AAATTACTACTAAAGCCGAACACCTTCAGAACTGTCATACGTTCCCGAAGATAAGAAGCC

CCACCACGAAAGTGGCTTTATATTACCCGACCCCACGAAAGCTGTGAAA

>803_Sample_ASV7

CACCGCGGTTATACGAGAGGCCCAAGTTGACAGACACCGGCGTAAAGAGTGGCTAGGGAA

AAATTACTACTAAAGCCGAACACCTTCAGAACTGTCATACGTTCCCGAAGATAAGAAGCC

CCACCACGAAAGTGGCTTTATATTACCCGACCCCACGAAAGCTGGGGGA

>803_Sample_ASV8

CACCGCGGTTATACGAGAGGCCCAAGTTGACAGACACCGGCGTAAAGAGTGGCTAAGGAA

AAATTTCTACTAAAGCCGAACACCTTCAGAACTGTCATACGTTCCCGAAGAAAAGAAGCC

CCACCACGAAAGTGGCTTTATATTACCCGACCCCACGAAAGCTGTGAAA

>803_Sample_ASV9

CACCGCGGTTAAACGAGAGACCCTAGTTGATCATCACGGCGTAAAGAGTGGTTAGGGGAT

TATCATAAATAAAGCCAAAGACCTCCCAAGCTGTCGCACGCATTCCGGAGGCGCGAAGCC

CACACACGAAAGTAGCTTTAACTATTGACCCTGATGCCACGAAAGCTAAGAAA

>803_Sample_ASV10

CGCCGCGGTTAAACGAGAGGCCCTAGTTGATGGAACAACGGCGTAAAGGGTGGTTAAGGA

TAGTGAGACAATAAAGTCGAATGGCCCTTTGGCTGTCATACGCTTCTGGGAGCCTGAAGC

CCACTATACGAAAGTAGCTTTAACAATGTCCACCTGACCCCACGAAAACTGAGAAA

>803_Sample_ASV11

CACCGCGGTTATACGAAAGACCCTAGTTGATAGATCACGGCGTAAAGGGTGGTTAGGGAA

TACAAAAATAAAGCTAAAAATCCTCTAAGCTGTCATACGCTTCCCGAAGACATGAAACCC

AACCACGAAAGTAGCTTTAAACTCTCCTGACACCACGAAAGCCAAGGAA

>803_Sample_ASV12

CACCGCGGTTATACGAGAGGCCCAAGTTGATAAGCCCGGCGTAAAGAGTGGTTATGGAGA

ATAAAACACTAAAGCTAAAGACCCCCTAGGCTGTTATACGCACCTGGCGGCTCGAACCAC

CTATACGAAAGTAGCTTTACCCCCCTTCCACCAGAATCCACGACAGCTGGGGCA

>803_Sample_ASV13

CACCGCGGTTATACGAGAGGCCCAAGTTGACAGACATCGGCGTAAAGAGTGGTTAAGGAA

ACCCCATTACTAAAGCCGAACGCCCTCAGAACTGTCATACGTTCCCGAAGGTAAGAAGCC

CCACTACGAAAGTGGCTTTAACCACACCCGACCCCACGAAAGCTGTGAAA

>803_Sample_ASV14

CACCGCGGTTAAACGAGAGGCCCTAGTTGATATTACAACGGCGTAAAGGGTGGTTAAGGA

AAGCAAGCAATAAAGCCGAATGGCCCTTTGGCTGTCATACGCTTCTAGGTGTCCGAAGCC

CAATATACGAAAGTAGCTTTAGAAAAGCCCACCTGACACCACGAAAGCTGAGGAA

>803_Sample_ASV15

CACCGCGGTTATACGAGAGGCCCAAGTTGACAGACACCGGCGTAAAGAGTGGTTAAGGAA

ATTCATCAACTAAAGCCGAATGCCCTCAGGACTGTTATACGTTTGCGAAGGTAAGAAGCC

CTACTACGAAAGTGGCTTTATTCCACCTGATTCCACGAAAGCTGCGGAA

>803_Sample_ASV16

CACCGCGGTCACACGATTAACCCAAGTCAATAGAAGCCGGCGTAAAGAGTGTTTTAGATC

ACCCCCTCCCCAATAAAGCTAAAACTCACCTGAGTTGTAAAAAACTCCAGTTGACACAAA

ATAGACTACGAAAGTGGCTTTAACATATCTGAACACACAATAGCTAAGACC

>803_Sample_ASV17

CACCGCGGTTATACGAGAGGCCCTAGTTGATAAATACGGCGTAAAGAGTGGTTAGAGGAC

TCCCATAATAAAGCCAAAGACCCTCCGGGCCGTCACACGCATACGAGGGCACGAAGTTCA

CACACGAAAGTAGCTTTAAATTACCCGACGCCACGAAAGCTAAGAAA

>803_Sample_ASV18

CACCGCGGTTATACGAAAGACCCTAGTTTATAAGTACGGCGTAAAGGGTGGTTAGGGGAC

AAACAAAATAAAGCCAAAGACCTCCTAAGCCGTCATACGCTTACGGAGGCACGAAGCCCA

AACACGAAAGTAGCTTTACCGAACATGCCCGACTCCACGAAAGCTGGGAAA

>803_Sample_ASV19

CACCGCGGTTATACGAGAGACCCTAGTTGATAAATACGGCGTAAAGAGTGGTTATGGGAA

AAACAAATAAAGTCAAACAACCTCTTAGCTGTTATACGCATTATGAGAGCATGAAGCCCC

CTCACGAAAGTAACTTTAATACCTCCTGACCCCACGAAAGCTAAGAAA

>803_Sample_ASV20

CACCGCGGTTATACGAGAGGCTCAAGTTGATAAACCCCGGCGTAAAGAGTGGTTAAGGAA

AAATAGAAACTAAAGCCGAATGCTTTCAAGACTGTTATACGTTCCCGAGAGTAAGAAGAC

CAATTACGAAAGTGGCTTTACTTTACCTGACCCCACGAAAGCTACGACA

>803_Sample_ASV21

CACCGCGGTTATACGAGAGACCCTAATTGATAGACACGGCGTAAAGAGTGGTTTGGGAAA

GCCCAAAAATAAAGCTAAAGACCTTCTAAGCCGTCACACGCACGCCGAAGGCACGAAGCC

CTGACACGAAAGTAGCTTTACTATTACCCCCGACGCCACGAAAGCTAAGAAA

>803_Sample_ASV22

AACCGCGGTTATACGAGAGACCCTAATTGATAGCTCACGGCGTAAAGGGTGGTTTGGGGA

AACCTCAAAATAAAGCTAAAGACCTTCTAAGCCGTTACACGCATACCGAAGGCACGAGGC

CCTAACACGAAAGTAGCTTTACTATTACCCCTGACGCCACGAAAGCTAAGAAA

>803_Sample_ASV23

CACCGCGGTTATACGAGAGACCCTAATTGATAGGTACGGCGTAAAGAGTGGTTTGGGATA

ACCCAAAAATAAAGCTAAAGACCTTCTAAGCCGTCACACGCATACCGAAGGCACGAGGCC

CCAACACGAAAGTAGCTTTACTATTACCCCGACGCCACGAAAGCTAAGAAA

>803_Sample_ASV24

CACCGCGGTTATACGAGAGACCCTAATTGATAGGCACGGCGTAAAGAGTGGTTTAGGGTA

ACCCATAAATAAAGCTAAAGACCTTCTAAGCCGTCACACGCACACCGAAGGCACGAGACC

CCAACACGAAAGTAGCTTTACTTCTACCCCTGACGCCACGAAAGCTAAGAAA

>803_Sample_ASV25

CACCGCGGTTATACGAGAGACCCTAGTTGATAGCTACGGCGTAAAGCGTGGTTAGGAGAA

CAAGATAAATAAAGTCAAACAATCTCTCGGCCGTTATACGTTATCTGAGAATATGAAGTC

CTACCACGAAAGTAACTTTAATTTTTCTGACCCCACGAAAACTGAGGAA

>803_Sample_ASV26

CACCGCGGTTAAACGAGAGGCCCTAGTTGATAGTACAACGGCGTAAAGGGTGGTTAAGGA

TAGTGAAATAATAAAGTCGAATGGCCCTTTGGCTGTCATACGCTTCTAGGAGTCCGAAGC

CCAATATACGAAAGTAACTTTAAGAGCGCCCACCTGACCCCACGAAAACTGAGAAA

>803_Sample_ASV27

CACCGCGGTTATACGAGAGGCCCAAGTTGATACTCCACGGCGTAAAGAGTGGTTAGAAAA

GACCTACTACTAAAGCCGAACGCCTTCAAAGCTGTTATACGCATCCGAAGGTGAGAAGCC

CACCCACGAAAGTGGCTTTACAACCTTGACTCCACGAAAGCTATGACA

>803_Sample_ASV28

CACCGCGGTTATACGAGAGACCCCAGTTGATAAACACGGCGTAAAGAGTGGTTAAGGGCA

CAAAACAAATAAAGTCAAACAACCTCTTAGCTGTTATACGCATTATGAGGATATGAAGCC

CCCCTACGAAAGTAACTTTACCAACCCCTGACCCCACGAAAGCTAAGAAA

>803_Sample_ASV29

CACCGCGGTTATACGAGAGACCCTAGTTGATATATACGGCGTAAAGAGTGGTTTGGAATA

ACCAAGTAATATAGCCAAAGACCTCCCAAGCCGTCACACGCACCCCGGAGGCACGAAGCC

CAAACACGAAAGTAGCTTTATTAACTTACCGACGCCACGAAAGCTAAGAAA

>803_Sample_ASV30

CACCGCGGTTATACGAAAGACCCTAGTTTATAGGCACGGCGTAAAGGGTGGTTAGGGGAT

AAACAAAATAAAGCCAAAGACCTTCTAAGTCGTCATACACTCATGAAGGCACGAAGCCCA

AATACGAAAGTAACTTTACAACTTAGACCCGACTCCACGAAAGCTGGGGGA

>803_Sample_ASV31

CACCGCGGTTATACGAGAGGCCCAAGTTGATAGACATCGGCGTAAAGAGTGGTTAAGGAA

GTCTCAAAACTAAAGCCGAATGCCTTCAAGACTGTTATACGTTTCCGAAGGTAAGAAGCC

CCACTACGAAAGTGGCTTTACTCCACCTGACTCCACGAAAGCTGTGAAA

>803_Sample_ASV32

CACCGCGGTTATACGAGAGGCCCAAGTTGACAAACTCCGGCGTAAAGCGTGGTTAAGGCA

CACTCAAAAACTAAAGCCGAATATCTTCAGAGCAGTCATACGCATCCGAAGACACGAAGC

CCCACCACGAAAGTGGCTTTATGACACCTGACCCCACGAAAGCTAGGACA

>803_Sample_ASV33

CACCGCGGTTATACGAAAGACCCTAGTTGATAGACCACGGCGTAAAGGGTGGTTAAGGAA

CACAAAAATAAAGCTAAAGATCCTCTAAGCTGTCATACGCTTCCCGAAGACATGAAACCC

AATCACGAAAGTAGCTTTAAACTGTCCTGACACCACGAAAGCCAAGAAA

>804_Sample_ASV1

CACCGCGGTTATACGAGAGGCCCAAGTTGACAGACATCGGCGTAAAGAGTGGTTAAGGAA

AACCTTCAACTAAAGTCGAACGCTCTCAAGACTGTCGTACGTTTCCGAGAGTATGAAGCC

CCACCACGAAAGTGACTTTACCTCACCTGACTCCACGAAAGCTGCGAAA

>804_Sample_ASV2

CACCGCGGTTATACGAAAGACCCTAGTTGATGGGTGCGGCGTAAAGGGTGGTTAGGGACT

ACAAAAAATAGAGCTAAAGATCCTCTAGGTCGTCATACACATCACGAGGAAACGAAGCCC

AAATACGAAAGTAGCTCTAAATATAATCCTGACGCCACGAAAGCCAAGAAA

>804_Sample_ASV3

CACCGCGGTTATACGAAAGACCCTAGTTGATGGGTGCGGCGTAAAGGGTGGTTAGGGACT

ACAAAAAATAGAGCTAAAGATCCTCTAGGTCGTCATACACATCACGAGGAAACGAAGCCC

AAGTACGAAAGTAGCTCTAAATATAATCCTGACGCCACGAAAGCCAAGAAA

>804_Sample_ASV4

CACCGCGGTTATACGAAAGACCCTAGTTGATGGGTGCGGCGTAAAGGGTGGTTAGGGACT

ACAAAAAATAGAGCTAAAGATCCTCTAGGTCGTCATACACATCACGAGAAAACGAAGCCC

AAATACGAAAGTAGCTCTAAATATAATCCTGACGCCACGAAAGCCAAGAAA

>804_Sample_ASV5

CACCGCGGTTATACGAGAGACCCTAGTTGATTAAAGCGGCGTAAAGAGTGGTTATGGAAC

TATTCTTTTAAAGCAGAAAACCTCTCAAACTGTTATACGCACCCAGAGGTCTGAATCCCT

CACACGAAAGTGACTTTATTTATGCCTACCAGAACCCACGAAAGCTGGGACA

>804_Sample_ASV6

CACCGCGGTTATACGAAAGACCCTAGTTTATAGGTACGGCGTAAAGGGTGGTTAGGGGAC

AAACAAAATAAAGCCAAAGACCTCCTAAGCCGTCATACGCTCACGGAAGCACGAAGCCCA

AACACGAAAGTAGCTTTACCGAACATGCCCGACTCCACGAAAGCTGGGGAA

>804_Sample_ASV7

CACCGCGATTATACGAGAGGCCCAAGTTGACAGACATCGGCGTAAAGAGTGGTTAAGGAA

AAATTACTACTAAAGCCGAACACCTTCAGAACTGTCATACGTTCCCGAAGATAAGAAGCC

CCACCACGAAAGTGGCTTTATATTACCCGACCCCACGAAAGCTGTGAAA

>804_Sample_ASV8

CACCGCGGTTATACGAGAGGCCCAAGTTGACAGACACCGGCGTAAAGAGTGGCTAGGGAA

AAATTACTACTAAAGCCGAACACCTTCAGAACTGTCATACGTTCCCGAAGATAAGAAGCC

CCACCACGAAAGTGGCTTTATATTACCCGACCCCACGAAAGCTGGGGGA

>804_Sample_ASV9

CACCGCGATTATACGAGAGGCCCAGGTTGACAGACATCGGCGTAAAGAGTGGTTAAGGAA

AAATTACTACTAAAGCCGAACACCTTCAGAACTGTCATACGTTCCCGAAGATAAGAAGCC

CCACCACGAAAGTGGCTTTATATTACCCGACCCCACGAAAGCTGTGAAA

>804_Sample_ASV10

CACCGCGGTTATACGAGAGGCCCAAGTTGACAGACACCGGCGTAAAGAGTGGCTAGGGAA

AAATTACTACTAAAGCCGAACACCTTCAGAACTGTCATACGTTCCCGAAGATAAGAAGCC

CCACCACGAAAGTGGCTTTATATTACCCGACCCCACGAAAGCTGTGAAA

>804_Sample_ASV11

CACCGCGGTTATACGAGAGGCCCAAGTTGACAGACACCGGCGTAAAGAGTGGCTAAGGAA

AAATTTCTACTAAAGCCGAACACCTTCAGAACTGTCATACGTTCCCGAAGAAAAGAAGCC

CCACCACGAAAGTGGCTTTATATTACCCGACCCCACGAAAGCTGGGGGA

>804_Sample_ASV12

CACCGCGGTTATACGAGAGGCCCAAGTTGACAGACACCGGCGTAAAGAGTGGCTAAGGAA

AAATTTCTACTAAAGCCGAACACCTTCAGAACTGTCATACGTTCCCGAAGAAAAGAAGCC

CCACCACGAAAGTGGCTTTATATTACCCGACCCCACGAAAGCTGTGAAA

>804_Sample_ASV13

CACCGCGGTTATACGAAAGACTCTAGTTGACAAACACGGCGTAAAGGGTGGTTAGGATAA

CAACAAAATAAAGCTAAAGACTTACCAAGCCGTCATACGCCCATGTAAGAGCGAAAACCA

CAAACGAAAGTAACTTTATTACACCATCCGAATCCACGAAAGCTAAGAAA

>804_Sample_ASV14

CACCGCGGTTAAACGAGAGACCCTAGTTGATCATCACGGCGTAAAGAGTGGTTAGGGGAT

TATCATAAATAAAGCCAAAGACCTCCCAAGCTGTCGCACGCATTCCGGAGGCGCGAAGCC

CACACACGAAAGTAGCTTTAACTATTGACCCTGATGCCACGAAAGCTAAGAAA

>804_Sample_ASV15

CACCGCGGTTATACGAGAGGCCCAAGTTGATAAGCCCGGCGTAAAGAGTGGTTATGGAGA

ATAAAACACTAAAGCTAAAGACCCCCTAGGCTGTTATACGCACCTGGCGGCTCGAACCAC

CTATACGAAAGTAGCTTTACCCCCCTTCCACCAGAATCCACGACAGCTGGGGCA

>804_Sample_ASV16

CACCGCGGTTATACGAGAGGCCCAAGTTGACAGACATCGGCGTAAAGAGTGGTTAAGGAA

ACCCCATTACTAAAGCCGAACGCCCTCAGAACTGTCATACGTTCCCGAAGGTAAGAAGCC

CCACTACGAAAGTGGCTTTAACCACACCCGACCCCACGAAAGCTGGGGGA

>804_Sample_ASV17

CACCGCGGTTATACGAGAGGCCCAAGTTGACAGACATCGGCGTAAAGAGTGGTTAAGGAA

ACCCCATTACTAAAGCCGAACGCCCTCAGAACTGTCATACGTTCCCGAAGGTAAGAAGCC

CCACTACGAAAGTGGCTTTAACCACACCCGACCCCACGAAAGCTGTGAAA

>804_Sample_ASV18

CACCGCGGTTATACGAGAGGCCCAAGTTGATAGATATCGGCGTAAAGAGTGGTTAAGGAA

ACCCCGTTACTAAAGCCGAACGCCCTCAGAACTGTCATACGTTCCCGAAGGTAAGAAGCC

CCACTACGAAAGTGGCTTTAACCACACCCGACCCCACGAAAGCTGTGAAA

>804_Sample_ASV19

CACCGCGGTTATACGAGAGGCCCAAGTTGACAGACACCGGCGTAAAGAGTGGTTAAGGAA

ATTCATCAACTAAAGCCGAATGCCCTCAGGACTGTTATACGTTTGCGAAGGTAAGAAGCC

CTACTACGAAAGTGGCTTTATTCCACCTGATTCCACGAAAGCTGCGGAA

>804_Sample_ASV20

CACCGCGGTTATACGAGAGGCCCCAGTTGATATTAGCGGCGTAAAGGGTGGTTAAGGACT

TTAATAACTAAAGCTAAACACTTCCCCGGCTGTTGTACGTAGACCGGAAGCTTGAAGCCC

AGACACGAAAGTAGCTTTATTTCCCCTGACCCCACGAAAGCTGAGAAA

>804_Sample_ASV21

CACCGCGGTTATACGAGAGGCCCCAGTTGATATTAGCGGCGTAAAGGGTGGTTAAGGACT

TTAATAACTAAAGCTAAACACTTCCCCGGCTGTTGTACGTAGACCGGAAGCTTGAAGCCC

AGACACGAAAGTAGCTTTATTCCCCCTGACCCCACGAAAGCTGAGAAA

>804_Sample_ASV22

CACCGCGGTTATACGAGAGGCCCCAGTTGATATTAGCGGCGTAAAGGGTGGTTAAGGACT

TTAATAACTAAAGCCAAACACTTCCCCGGCTGTTGTACGTAGACCGGAAGCTTGAAGCCC

AGACACGAAAGTAGCTTTATTTCCCCTGACCCCACGAAAGCTGAGAAA

>804_Sample_ASV23

CACCGCGGTCACACGATTAACCCAAGTCAATAGAAGCCGGCGTAAAGAGTGTTTTAGATC

ACCCCCTCCCCAATAAAGCTAAAACTCACCTGAGTTGTAAAAAACTCCAGTTGACACAAA

ATAGACTACGAAAGTGGCTTTAACATATCTGAACACACAATAGCTAAGACC

>804_Sample_ASV24

CACCGCGGTTATACGAGAGGCCCTAGTTGATAAATACGGCGTAAAGAGTGGTTAGAGGAC

TCCCATAATAAAGCCAAAGACCCCCCGGGCCGTCACACGCATACGAGGGCACGAAGTTCA

CACACGAAAGTAGCTTTAAATTACCCGACGCCACGAAAGCTAAGAAA

>804_Sample_ASV25

CACCGCGGTTATACGAGAAACCCTAGTTGACAAATACGGCGTAAAGAGTGGTTAAGGGGT

ATCAAAAATAAAGTCAAACATTTTCCTAGTTGTTATAAGCATTTCGAGAACATGAAGCCC

AACCACGAAAGTAACTTTACCTTCACTTGACCCCACGAAAGCTAAGAAA

>804_Sample_ASV26

CACCGCGGTTGTACGAGAAACCCTAGTTGACAAATACGGCGTAAAGAGTGGTTAAGGGGT

ATCAAAAATAAAGTCAAACATTTTCCTAGTCGTTATAAGCATTTCGAGAACATGAAGCCC

AACCACGAAAGTAACTTTACCTTCACCTGACCCCACGAAAGCTAAGAAA

>804_Sample_ASV27

CACCGCGGTTATACGAGAAACCCTAGTTGACAAATACGGCGTAAAGAGTGGTTAAGGGGT

ATCAAAAATAAAGTCAAAGATTTTCCTAGTTGTTATAAGCATTTCGAGAACATGAAGCCC

AACCACGAAAGTAACTTTACCTTCACCTGACCCCACGAAAGCTAAGAAA

>804_Sample_ASV28

CACCGCGGTTATACGAGAAACCCTAGTTGACAAATACGGCGTAAAGAGTGGTTAAGGGGT

ATCAAAAATAAAGTCAAACATTTTCCTAGTTGTTATAAGCATTTCGAGAACATGATGCCC

AACCACGAAAGTAACTTTACCTTCACCTGACCCCACGAAAGCTAAGAGA

>804_Sample_ASV29

CACCGCGGTTATACGAGAAACCCTAGTTGACAAATACGGCGTAAAGAGTGGTTAAGGGGT

ATCAAAAATAAAGTCAAACATTTTCCTAGTTGTTATAAGCATTTCGAGAACATGAAGCCC

AACCACGAAAGTAACTTTACCTTCACCTGACCCCACGAAAGCTAAGAAA

>804_Sample_ASV30

GACCGCGGTTATACGAGAGACTCTAGTTGATAGATACGGCGTAAAGAGTGGTTTGGGATA

AACTATAAATAAAGCCAAAGACCTTCCAAGCTGTTAAACGCATACCGAAGGCACGAAGCC

CCAACACGAAAGTAGCTTTACTACTACCCCCGATGCCACGAAAGCTAAGACA

>804_Sample_ASV31

CACCGCGGTTATACGAAAGACCCCAGTTTATAGACACGGCGTAAAGGGTGGTTATAGGAC

AAACTAACATAGAGCCAAAGACCCCCTAAGCCGTCATACGCTCACGAGGGCTCGAAGCCC

CTATACGAAAGTAGCTCTACCAAAAAATGCTCGACTCCACGAAAGCTGGGACA

>804_Sample_ASV32

CACCGCGGTTATACGAGAGACCCTAGTTGATAAATACGGCGTAAAGAGTGGTTATGGGAA

AAACAAATAAAGTCAAACAACCTCTTAGCTGTTATACGCATTATGAGAGCATGAAGCCCC

CTCACGAAGGTAACTTTAATACCTCCTGACCCCACGAAAGCTAAGAAA

>804_Sample_ASV33

CACCGCGGTTATACGAGAGGCTCAAGTTGATAAACCCCGGCGTAAAGAGTGGTTAAGGAA

AAATAGAAACTAAAGCCGAATGCTTTCAAGACTGTTATACGTTCCCGAGAGTAAGAAGAC

CAATTACGAAAGTGGCTTTACTTTACCTGACCCCACGAAAGCTACGACA

>804_Sample_ASV34

CACCGCGGTTATACGAGAGACCCTAATTGATAGACACGGCGTAAAGAGTGGTTTGGGAAA

GCCCAAAAATAAAGCTAAAGACCTTCTAAGCCGTCACACGCACGCCGAAGGCACGAAGCC

CTGACACGAAAGTAGCTTTACTATTACCCCCGACGCCACGAAAGCTAAGAAA

>804_Sample_ASV35

CACCGCGGTTATACGAGAGACCCTAATTGATAGACACGGCGTAAAGAGTGGTTTGGGAAA

GCCCAAAAATAAAGCTAAAGACCTTCTAAGCCGTCACACGCACGCCGAAGGCACGAAGCC

CTGACACGAAAGTAGCTTTACTACTGCCCCTGACGCCACGAAAGCTAAGAAA

>804_Sample_ASV36

AACCGCGGTTATACGAGAGACCCTAATTGATAGCTCACGGCGTAAAGGGTGGTTTGGGGA

AACCTCAAAATAAAGCTAAAGACCTTCTAAGCCGTTACACGCATACCGAAGGCACGAGGC

CCTAACACGAAAGTAGCTTTACTATTACCCCTGACGCCACGAAAGCTAAGAAA

>804_Sample_ASV37

CACCGCGGTTATACGAGAGACCCTAATTGATAGACACGGCGTAAAGAGTGGTTTAGGACA

ACCCAAAAATAAAGCTAAAGACCTTCTAAGCCGTCACACGCACGCCGAAGGCACGAAGCC

CTGACACGAAAGTAGCTTTACTATTACCCCCGACGCCACGAAAGCTAAGAAA

>804_Sample_ASV38

CACCGCGGTTATACGAAAGACCCTAGTTTATAGGCACGGCGTAAAGAGTGGTTTAGGGTA

ACCCATAAATAAAGCTAAAGACCTTCTAAGCCGTCACACGCACACCGAAGGCACGAGACC

CCAACACGAAAGTAGCTTTACTTCTACCCCTGACGCCACGAAAGCTAAGAAA

>804_Sample_ASV39

CACCGCGGTTATACGAGAGACCCTAATTGATAGGCACGGCGTAAAGAGTGGTTTAGGGTA

ACCCATAAATAAAGCTAAAGACCTTCTAAGCCGTCACACGCACACCGAAGGCACGAGACC

CCAACACGAAAGTAGCTTTACTTCTACCCCTGACGCCACGAAAGCTAAGAAA

>804_Sample_ASV40

CACCGCGGTTATACGAAAGACCCTAGTTGATGGATCACGGCGTAAAGGGTGGTTAAGGGA

TATAAAAATAAAGCTAAAAATCCTCTTAGCTGTCATACGCTTTCCGAAGACATGAGGCCC

AATTACGAAAGTAGCTTTAAACCACCCTGACACCACGAAAGCCAAGAAA

>804_Sample_ASV41

CACCGCGGTTATACGAAAGACCCTAGTTTATAGATGCGGCGTAAAGGGTGGTTAAGGAAA

ACCTAAAATAATGCTAAAGACTCTCAAAGCTGTTATACGCTTCATGAGCACACGAAATCC

AAATACGAAAGTTGCCTTAATAAACTTTGCCTGACCCCACGAAAGCTGAAAAA

>804_Sample_ASV42

CACCGCGGTTATACGAAAGACCCCAGTTTATAGATGCGGCGTAAAGGGTGGTTAAGAGAA

AATCAAAATAAGGCCAAAGATCCCCTAAGCAGTCATACGCTCCACGAGGACACGAAACCC

AAATACGAAAGTTGCCTTAATAAACTTCACCTGATCCCACGAAAGCTGAAAAA

>804_Sample_ASV43

CACCGCGGTTATACGAAAGGCTCTAGTTTATAAATACGGCGTAAAGGGTGGTTAAGGAAA

AATTAAAATAATGCTAAAGACCCCCTAAGCAGTTATACGCTTCACGGAGGCACGAAACCC

AAATACGAAAGTTGCCTTAATAAACTTTACCTGACCCCACGAAAGCTGAAAAA

>804_Sample_ASV44

CACCGCGGTTATACGAGAGACCCTAGTTGATAGCTACGGCGTAAAGCGTGGTTAGGAGAA

CAAGATAAATAAAGTCAAACAATCTCTCGGCCGTTATACGTTATCTGAGAATATGAAGTC

CTACCACGAAAGTAACTTTAATTTTTCTGACCCCACGAAAACTGAGGAA

>804_Sample_ASV45

CACCGCGGTCATACGATTAACCCAAGTTAATAGGCACACGGCGTAAAGCGTGTTTAAGCA

CTATGCCAAATAAAGTTAAACTCCAATTAAGCTGTAAAAAGCCATAATCAAGACGAAAAT

AAATAACGAAAGTAACTTTACAACCGCTGAAACACGATAGCTAAGATC

>804_Sample_ASV46

CACCGCGGTTATACGAAAGACCCTAGTTTATAGACACGGCGTAAAGGGTGGTTCGGGACT

AAACAAAATAAAGCCAAAGACCTCCTAAGCCGTCATACGCTCACGGAAGCACGAAGCCCA

AGTACGAAAGTAGCTTTACAAAACATGCCTGACTCCACGAAAGCTAGGAAA

>804_Sample_ASV47

CACCGCGGTTATACGAAAGACCCTAGTTGATAGGCACGGCGTAAAGGGTGGTTAGGGATA

TATCAAAAATAAAGTTAAAGAGCCTCTAAGCTGTCGCACGCATCCCGAGAGCTCGAGACC

CAGACACGAAAGTAACTTTAAAATACCTCACCTGACCCCACGAAAGCTAAGAAA

>804_Sample_ASV48

CACCGCGGTTATACGAAAGACCCTAGTTGATAGGCACGGCGTAAAGGGTGGTTAGGGATA

TATTAAAAATAAAGTTAAAGAGCCTCTAAGCTGTCGCACGCATCCCGAGAGCTCGAGACC

CAGACACGAAAGTAACTTTAAAATACCTCACCTGACCCCACGAAAGCTAAGAAA

>804_Sample_ASV49

CACCGCGGTTATACGAAAGACCCTAGTTGATAGCCACGGCGTAAAGGGTGGTTAAGGTAT

ACAACAAATAAAGCTAAATAGCCTCTAGGCCGTCGCACGCATTCCGAGAGCTCGAAACCC

AAAAACGAAAGTAGCTTTAAAATAAACAGACCTGACCCCACGAAAGCTAAGAAA

>804_Sample_ASV50

CACCGCGGTTATACGAGAGGCCCAAGTTGATACTCCACGGCGTAAAGAGTGGTTAGAAAA

GACCTACTACTAAAGCCGAACGCCTTCAAAGCTGTTATACGCATCCGAAGGTGAGAAGCC

CACCCACGAAAGTGGCTTTACAACCTTGACTCCACGAAAGCTATGACA

>804_Sample_ASV51

CACCGCGGTTATACGAGAGACCCCAGTTGATAAACACGGCGTAAAGAGTGGTTAAGGGCA

CAAAACAAATAAAGTCAAACAACCTCTTAGCTGTTATACGCATTATGAGGATATGAAGCC

CCCCTACGAAAGTAACTTTACCAACCCCTGACCCCACGAAAGCTAAGAAA

>804_Sample_ASV52

CACCGCGGTTATACGAGAGACCCTAGTTGATATATACGGCGTAAAGAGTGGTTTGGAATA

ACCAAGTAATATAGCCAAAGACCTCCCAAGCCGTCACACGCACCCCGGAGGCACGAAGCC

CAAACACGAAAGTAGCTTTATTAACTTACCGACGCCACGAAAGCTAAGAAA

>804_Sample_ASV53

CACCGCGGTTATACGAAAGACCCTAGTTTATAGGCACGGCGTAAAGGGTGGTTAGGGGAT

AAACAAAATAAAGCCAAAGACCTTCTAAGTCGTCATACACTCATGAAGGCACGAAGCCCA

AATACGAAAGTAACTTTACAACTTAGACCCGACTCCACGAAAGCTGGGGGA

>804_Sample_ASV54

CACCGCGGTTATACGAAAGACCCTAGTTTATAGGCACGGCGTAAAGGGTGGTTAGGGGAT

AAACAAAATAAAGCCAAAGACCTTCTAAGTCGTCATACACTCATGAAGGCACGAAGCCCA

AATACGAAAGTAACTTTACAACTTAGACCCGACCCCACGAAAGCTGTGAAA

>804_Sample_ASV55

CACCGCGGTTATACGAGAGGCCCAAGTTGACAGATATCGGCGTAAAGAGTGGTTAAGGAA

ATCTTAAAACTAAAGCCGAATGCCTTCAAGACTGTTATACGTTTCCGAAGGTAAGAAGCC

CCACTACGAAAGTGGCTTTACTCCACCTGACTCCACGAAAGCTGTGAAA

>804_Sample_ASV56

CACCGCGGTTATACGAGAGGCCCAAGTTGACAGATATCGGCGTAAAGAGTGGTTAAGGAA

ATCTTAAAACTAAAGCCGAATGCCTTCAAGACTGTTATACGTTTACGAAGGTAAGAAGCC

CCACTACGAAAGTGGCTTTACTCCACCTGACTCCACGAAAGCTGTGAAA

>804_Sample_ASV57

CACCGCGGTTATACGAGAGGCCCAAGTTGATAGACATCGGCGTAAAGAGTGGTTAAGGAA

GTCTCAAAACTAAAGCCGAATGCCTTCAAGACTGTTATACGTTTCCGAAGGTAAGAAGCC

CCACTACGAAAGTGGCTTTACTCCACCTGACTCCACGAAAGCTGGGGGA

>804_Sample_ASV58

CACCGCGGTTATACGAGAGGCCAAAGTTGACAGATATCGGCGTAAAGAGTGGTTAAGGAA

GTCTCAAAACTAAAGCCGAATGCCTTCAAGACTGTTATACGTTTCCGAAGGTAAGAAGCC

CCACTACGAAAGTGGCTTTACTCCACCTGACTCCACGAAAGCTGTGAAA

>804_Sample_ASV59

CACCGCGGTTATACGAGAGGCCAAAGTTGACAGATATCGGCGTAAAGAGTGGTTAAGGAA

ATCTTAAAACTAAAGCCGAATGCCTTCAAGACTGTTATACGTTTCCGAAGGTAAGAAGCC

CCACTACGAAAGTGGCTTTACTCCACCTGACTCCACGAAAGCTGTGAAA

>804_Sample_ASV60

CACCGCGGTTATACGAGAGGCCCAAGTTGATAGACATCGGCGTAAAGAGTGGTTAAGGAA

GTCTCAAAACTAAAGCCGAATGCCTTCAAGACTGTTATACGTTTCCGAAGGTAAGAAGCC

CCACTACGAAAGTGGCTTTACTCCACCTGACTCCACGAAAGCTGTGAAA

>804_Sample_ASV61

CACCGCGGTTATACGAGAGGCCCAAGTTGATAGACATCGGCGTAAAGAGTGGTTAAGGAA

GTCTCAAAACTAAAGCCGAATGCCTTCAAGACTGTTATACGTTTACGAAGGTAAGAAGCC

CCACTACGAAAGTGGCTTTATTCCACCTGACCCCACGAAAGCTGTGAAA

>804_Sample_ASV62

CACCGCGGTTATACGAGAGACTCCAGTTGATAGCTACGGCGTAAAGAGTGGTTTGGGGCC

CGCCCAAAATAAAGCCAAAGACCTCCCAAGCCGTCAAACGCACCCCGGAGGCACGAAGTC

CTAACGCGAAAGCAACTTTACCTTCCCCGACGCCACGAAAGCTAAGAAA

>804_Sample_ASV63

CACCGCGGTTATACGAGAGACCCTAATTGATAGACTACGGCGTAAAGAGTGGTTTAGAAC

AAAAATTAATAAAGCCAAAGATCCTCCTGGCCGTCGCACGCATTTCGAGGATACGAAGCC

CCACTACGAAAGTAGCTTTACTACAACTTTTCTGACCCCACGAAAGCTAAGAAA

>804_Sample_ASV64

CACCGCGGTTATACGAAAGACCCTAGTTGATAGATCACGGCGTAAAGGGTGGTTAAGGAG

AACAAAAATAAAGCTAAAAATCTTCTAAGCTGTCATACGCTTTCCGAAGACATGAGACCC

AACCACGAAAGTAGCTTTAAACTTTCCTGACACCACGAAAGCCAAGAAA

>814_Sample_ASV1

CACCGCGGTTATACGAAAGACCCTAGTTGATGGGTGCGGCGTAAAGGGTGGTTAGGGACT

ACAAAAAATAGAGCTAAAGATCCTCTAGGTCGTCATACACATCACGAGAAAACGAAGCCC

AAATACGAAAGTAGCTCTAAATATAATCCTGACGCCACGAAAGCCAAGAAA

>814_Sample_ASV2

CACCGCGGTTATACGAAAGACCCTAGTTGATGGGTGCGGCGTAAAGGGTGGTTAGGGACT

ACAAAAAATAGAGCTAAAGATCCTCTAGGTCGTCATACACATCACGAGGAAACGAAGCCC

AAATACGAAAGTAGCTCTAAATATAATCCTGACGCCACGAAAGCCAAGAAA

>814_Sample_ASV3

CACCGCGGTTATACGAGAGACCCTAGTTGATTAAAGCGGCGTAAAGAGTGGTTATGGAAC

TATTCTTTTAAAGCAGAAAACCTCTCAAACTGTTATACGCACCCAGAGGTCTGAATCCCT

CACACGAAAGTGACTTTATTTATGCCTACCAGAACCCACGAAAGCTGGGACA

>814_Sample_ASV4

CACCGCGATTATACGAGAGGCCCAAGTTGACAGACATCGGCGTAAAGAGTGGTTAAGGAA

AAATTACTACTAAAGCCGAACACCTTCAGAACTGTCATACGTTCCCGAAGATAAGAAGCC

CCACCACGAAAGTGACTTTATATTACCCGACCCCACGAAAGCTGTGAAA

>814_Sample_ASV5

CACCGCGGTTATACGAGAGGCCCAAGTTGACAGACACCGGCGTAAAGAGTGGCTAGGGAA

AAATTACTACTAAAGCCGAACACCTTCAGAACTGTCATACGTTCCCGAAGATAAGAAGCC

CCACCACGAAAGTGACTTTATATTACCCGACCCCACGAAAGCTGTGAAA

>814_Sample_ASV6

CACCGCGGTTATACGAGAGGCCCAAGTTGACAGACACCGGCGTAAAGAGTGGCTAGGGAA

AAATTACTACTAAAGCCGAACACCCTCAGAACTGTCATACGTTCCCGAAGGTAAGAAGCC

CCACCACGAAAGTGGCTTTATAATACCCGACCCCACGAAAGCTGTGAAA

>814_Sample_ASV7

CACCGCGGTTATACGAGAGGCCCAAGTTGACAGACACCGGCGTAAAGAGTGGCTAGGGAA

AAATTACTACTAAAGCCGAACACCTTCAGAACTGTCATACGTTCCCGAAGATAAGAAGCC

CCACCACGAAAGTGGCTTTATATTACCCGACCCCACGAAAGCTGTGAAA

>814_Sample_ASV8

CACCGCGGTTATACGAGAGGCCCAAGTTGACAGACACCGGCGTAAAGAGTGGCTAGGGAA

AAATTACTACTAAAGCCGAACACCTTCAGAACTGTCATACGTTCCCGAAGATAAGAAGCC

CCACCACGAAAGTGGCTTTATATTACCCGACCCCACGAAAGCTAAGGAA

>814_Sample_ASV9

CACCGCGGTTATACGAGAGGCCCAAGTTGACAGACACCGGCGTAAAGAGTGGCTAAGGAA

AAATTTCTACTAAAGCCGAACACCTTCAGAACTGTCATACGTTCCCGAAGATAAGAAGCC

CCACCACGAAAGTGGCTTTATATTACCCGACACCACGAAAGCTGTGAAA

>814_Sample_ASV10

CACCGCGGTTATACGAGAGGCCCAAGTTGACAGACACCGGCGTAAAGAGTGGCTAAGGAA

AAATTTCTACTAAAGCCGAACACCTTCAGAACTGTCATACGTTCCCGAAGAAAAGAAGCC

CCACCACGAAAGTGGCTTTATATTACCCGACCCCACGAAAGCTGTGAAA

>814_Sample_ASV11

CACCGCGGTTATACGAAAGACTCTAGTTGACAAACACGGCGTAAAGGGTGGTTAGGATAA

CAACAAAATAAAGCTAAAGACTTACCAAGCCGTCATACGCCCATGTAAGAGCGAAAACCA

CAAACGAAAGTAACTTTATTACACCATCCGAATCCACGAAAGCTAAGAAA

>814_Sample_ASV12

CACCGCGGTTAAACGAGAGACCCTAGTTGATCATCACGGCGTAAAGAGTGGTTAGGGGAT

TATCATAAATAAAGCCAAAGACCTCCCAAGCTGTCGCACGCATTCCGGAGGCGCGAAGCC

CACACACGAAAGTAGCTTTAACTATTGACCCTGATGCCACGAAAGCTAAGAAA

>814_Sample_ASV13

CACCGCGGTTATACGAGAGACCCTAGTTGATATATACGGCGTAAAGAGTGGTTTGGGATA

TTTCTCCAAAATAAAGTCGAAGATCTCCCAAGCCGTCACACGCATCCCGGAGACACGAAG

CCCTGATACGAAGGTAGCTTTATTAACACCCCGACGCCACGAAAGCTAAGAAA

>814_Sample_ASV14

CACCGCGGTTATACGAGAGACCCTAGTTGATATATACGGCGTAAAGAGTGGTTTGGGATA

TTTCTCCAAAATAAAGTCGAAGATCTCCCAAGCCGTCACACGCATCCCGGAGACACGAAG

CCCTGATACGAAGGTAGCTTTATTAATTCCCCGACGCCACGAAAGCTAAGAAA

>814_Sample_ASV15

CACCGCGGTTATACGAGAGACCCTAGTTGATATATACGGCGTAAAGAGTGGTTTGGGATA

TTTCTCCAAAATAAAGCCGAAGATCTCCCAAGCCGTCACACGCATCCCGGAGACACGAAG

CCCTGATACGAAGGTAGCTTTATTAACACCCCGACGCCACGAAAGCTAAGAAA

>814_Sample_ASV16

CGCCGCGGTTAAACGAGAGGCCCTAGTTGATGGAACAACGGCGTAAAGGGTGGTTAAGGA

TAGTGAGACAATAAAGTCGAATGGCCCTTTGGCTGTCATACGCTTCTGGGAGCCTGAAGC

CCACTATACGAAAGTAGCTTTAACAATGTCCACCTGACCCCACGAAAACTGAGAAA

>814_Sample_ASV17

CACCGCGGTTATACGAAAGACCCTAGTTGATAGATCACGGCGTAAAGGGTGGTTAGGGAA

TACAAAAATAAAGCTAAAAATCCTCTAAGCTGTCATACGCTTCCCGAAGACATGAAACCC

AACCACGAAAGTAGCTTTAAACTCTCCTGACACCACGAAAGCCAAGGAA

>814_Sample_ASV18

CACCGCGGTTATACGAGAGGCCCAAGTTGATAAGCCCGGCGTAAAGAGTGGTTATGGAGA

ATAAAACACTAAAGCTAAAGACCCCCTAGGCTGTTATACGCACCTGGCGGCTCGAACCAC

CTATACGAAAGTAGCTTTACCCCCCTTCCACCAGAATCCACGACAGCTGGGGCA

>814_Sample_ASV19

CACCGCGGTTATACGAGAGGCCCAAGTTGATAAGCCCGGCGTAAAGAGTGGTTATGGAGA

ATAAAACACTAAAGCTAAAGACCCCCTAGGCTGTTATACGCACCTGGCGGCTCGAACCAC

CTATACGAAAGTAGCTTTACCCCCCTTCCACCAGAATCCACGAAAGCTAAGGAA

>814_Sample_ASV20

CACCGCGGTTATACGAGAGGCCCAAGTTGACAGACATCGGCGTAAAGAGTGGTTAAGGAA

ACCCCATTACTAAAGCCGAACGCCCTCAGAACTGTCATACGTTCCCGAAGGTAAGAAGCC

CCACTACGAAAGTGGCTTTAACCACACCCGACCCCACGAAAGCTGTGAAA

>814_Sample_ASV21

CACCGCGGTTAAACGAGAGGCCCTAGTTGATATTACAACGGCGTAAAGGGTGGTTAAGGA

AAGCAAGCAATAAAGCCGAATGGCCCTTTGGCTGTCATACGCTTCTAGGTGTCCGAAGCC

CAATATACGAAAGTAGCTTTAGAAAAGCCCACCTGACACCACGAAAGCTGAGGAA

>814_Sample_ASV22

CACCGCGGTTATACGAGAGGCCCAAGTTGACAGACACCGGCGTAAAGAGTGGTTAAGGAA

ATTCATCAACTAAAGCCGAATGCCCTCAGGACTGTTATACGTTTGCGAAGGTAAGAAGCC

CTACTACGAAAGTGGCTTTATTCCACCTGATTCCACGAAAGCTGCGGAA

>814_Sample_ASV23

CACCGCGGTTATACGAGAGACCCCAGTTGATAGCTACGGCGTAAAGAGTGGTTAAGGACC

CCATTTAATAAAGCCAAAGACCCCCCAAGCCGTCATACGCACACGAGGGCGCGAAGCTCG

CACACGAAAGTAGCTCTAATTTACCCGACGCCACGAAAGCTAAGGAA

>814_Sample_ASV24

CACCGCGGTTATACGAGAGGCCCTAGTTGATAAATACGGCGTAAAGAGTGGTTAGAGGAC

TCCCATAATAAAGCCAAAGACCCTCCGGGCCGTCACACGCATACGAGGGCACGAAGTTCA

CACACGAAAGTAGCTTTAAATTACCCGACGCCACGAAAGCTAAGAAA

>814_Sample_ASV25

CACCGCGGTTATACGAGAAACCCTAGTTGACAAATACGGCGTAAAGAGTGGTTAAGGGGT

ATCAAAAATAAAGTCAAACATTTTCCTAGTTGTTATAAGCATTTCGAGAACATGAAGCCC

AACCACGAAAGTAACTTTACCTTCACCTGACCCCACGAAAGCTAAGAAA

>814_Sample_ASV26

GACCGCGGTTATACGAGAGACTCTAGTTGATAGATACGGCGTAAAGAGTGGTTTGGGATA

AACTATAAATAAAGCCAAAGACCTTCCAAGCTGTTAAACGCATACCGAAGGCACGAAGCC

CCAACACGAAAGTAGCTTTACTACTACCCCCGATGCCACGAAAGCTAAGACA

>814_Sample_ASV27

CACCGCGGTTATACGAAAGACCCTAGTTTATAAGTACGGCGTAAAGGGTGGTTAGGGGAC

AAACAAAATAAAGCCAAAGACCTCCTAAGCCGTCATACGCTTACGGAGGCACGAAGCCCA

AACACGAAAGTAGCTTTACCGAACATGCCCGACTCCACGAAAGCTGGGAAA

>814_Sample_ASV28

CACCGCGGTTATACGAGAGACCCTAGTTGATAAATACGGCGTAAAGAGTGGTTATGGGAA

AAACAAATAAAGTCAAACAACCTCTTAGCTGTTATACGCATTATGAGAGCATGAAGCCCC

CTCACGAAAGTAACTTTAATACCTCCTGACCCCACGAAAGCTAAGAAA

>814_Sample_ASV29

CACCGCGGTTATACGAGAGGCTCAAGTTGATAAACCCCGGCGTAAAGAGTGGTTAAGGAA

AAATAGAAACTAAAGCCGAATGCTTTCAAGACTGTTATACGTTCCCGAGAGTAAGAAGAC

CAATTACGAAAGTGGCTTTACTTTACCTGACCCCACGAAAGCTACGACA

>814_Sample_ASV30

AACCGCGGTTATACGAGAGACCCTAATTGATAGCTCACGGCGTAAAGGGTGGTTTGGGGA

AACCTCAAAATAAAGCTAAAGACCTTCTAAGCCGTTACACGCATACCGAAGGCACGAGGC

CCTAACACGAAAGTAGCTTTACTATTACCCCTGACGCCACGAAAGCTAAGAAA

>814_Sample_ASV31

CACCGCGGTTATACGAAAGACCCTAGTTGATGGATCACGGCGTAAAGGGTGGTTAAGGGA

TATAAAAATAAAGCTAAAAATCCTCTTAGCTGTCATACGCTTTCCGAAGACATGAGGCCC

AATTACGAAAGTAGCTTTAAACCACCCTGACACCACGAAAGCCAAGAAA

>814_Sample_ASV32

CACCGCGGTTATACGAAAGACCCTAGTTGATGGATCACGGCGTAAAGGGTGGTTAAGGGA

TATAAAAATAAAGCTAAAAATCCTCTTAGCTGTCATACGCTTTCCGAAGACATGAGGCCC

AATTACGAAAGTAGCTTTAAACCACCCTGACACCACGAAAGCTGTGAAA

>814_Sample_ASV33

CACCGCGGTTATACGAGAGACCCTAGTTGATAGCTACGGCGTAAAGCGTGGTTAGGAGAA

CAAGATAAATAAAGTCAAACAATCTCTCGGCCGTTATACGTTATCTGAGAATATGAAGTC

CTACCACGAAAGTAACTTTAATTTTTCTGACCCCACGAAAACTGAGGAA

>814_Sample_ASV34

CACCGCGGTTAAACGAGAGGCCCTAGTTGATAGTACAACGGCGTAAAGGGTGGTTAAGGA

TAGTGAAATAATAAAGTCGAATGGCCCTTTGGCTGTCATACGCTTCTAGGAGTCCGAAGC

CCAATATACGAAAGTAACTTTAAGAGCGCCCACCTGACCCCACGAAAACTGAGAAA

>814_Sample_ASV35

CACCGCGGTTATACGAAAGACCCTAGTTTATAGACACGGCGTAAAGGGTGGTTCGGGACT

AAACAAAATAAAGCCAAAGACCTCCTAAGCCGTCATACGCTCACGGAAGCACGAAGCCCA

AGTACGAAAGTAGCTTTACAAAACATGCCTGACTCCACGAAAGCTAGGAAA

>814_Sample_ASV36

CACCGCGGTTATACGAAAGACCCCAGTTGATAGGCACGGCGTAAAGGGTGGTTCGGGACT

AAACAAAATAAAGCCAAAGACCTCCTAAGCCGTCATACGCTCACGGAAGCACGAAGCCCA

AGTACGAAAGTAGCTTTACAAAACATGCCTGACTCCACGAAAGCTAGGAAA

>814_Sample_ASV37

CACCGCGGTTATACGAGAGGCCCAAGTTGATACTCCACGGCGTAAAGAGTGGTTAGAAAA

GACCTACTACTAAAGCCGAACGCCTTCAAAGCTGTTATACGCATCCGAAGGTGAGAAGCC

CACCCACGAAAGTGGCTTTACAACCTTGACTCCACGAAAGCTATGACA

>814_Sample_ASV38

CACCGCGGTTATACGAGAGACCCCAGTTGATAAACACGGCGTAAAGAGTGGTTAAGGGCA

CAAAACAAATAAAGTCAAACAACCTCTTAGCTGTTATACGCATTATGAGGATATGAAGCC

CCCCTACGAAAGTAACTTTACCAACCCCTGACCCCACGAAAGCTAAGAAA

>814_Sample_ASV39

CACCGCGGTTATACGAGAGACCCTAGTTGATATATACGGCGTAAAGAGTGGTTTGGAATA

ACCAAGTAATATAGCCAAAGACCTCCCAAGCCGTCACACGCACCCCGGAGGCACGAAGCC

CAAACACGAAAGTAGCTTTATTAACTTACCGACGCCACGAAAGCTAAGAAA

>814_Sample_ASV40

CACCGCGGTTATACGAAAGACCCTAGTTTATAGGCACGGCGTAAAGGGTGGTTAGGGGAT

AAACAAAATAAAGCCAAAGACCTTCTAAGTCGTCATACACTCATGAAGGCACGAAGCCCA

AATACGAAAGTAACTTTACAACTTAGACCCGACTCCACGAAAGCTGGGGGA

>814_Sample_ASV41

CACCGCGGTTATACGAAAGACCCTAGTTTATAGGCACGGCGTAAAGGGTGGTTAGGGGAT

AAACAAAATAAAGCCAAAGACCTTCTAAGTCGTCATACACTCATGAAGGCACGAAGCCCA

AATACGAAAGTAACTTTACAACTTAGACCCGACTCCACGAAAGCTAAGGAA

>814_Sample_ASV42

CACCGCGGTTATACGAAAGACCCCAGTTGATAGGCACGGCGTAAAGGGTGGTTAGGGGAT

AAACAAAATAAAGCCAAAGACCTTCTAAGTCGTCATACACTCATGAAGGCACGAAGCCCA

AATACGAAAGTAACTTTACAACTTAGACCCGACTCCACGAAAGCTGGGGGA

>814_Sample_ASV43

CACCGCGGTTATACGAGAGGCCCAAGTTGATAGACATCGGCGTAAAGAGTGGTTAAGGAA

GTCTCAAAACTAAAGCCGAATGCCTTCAAGACTGTTATACGTTTCCGAAGGTAAGAAGCC

CCACTACGAAAGTGGCTTTACTCCACCTGACTCCACGAAAGCTGTGAAA

>814_Sample_ASV44

CACCGCGGTTATACGAGAGGCCCAAGTTGACAAACTCCGGCGTAAAGCGTGGTTAAGGCA

CACTCAAAAACTAAAGCCGAATATCTTCAGAGCAGTCATACGCATCCGAAGACACGAAGC

CCCACCACGAAAGTGGCTTTATGACACCTGACCCCACGAAAGCTAGGACA

>814_Sample_ASV45

CACCGCGGTTATACGAAAGACCCTAGTTGATAGACCACGGCGTAAAGGGTGGTTAAGGAA

CACAAAAATAAAGCTAAAGATCCTCTAAGCTGTCATACGCTTCCCGAAGACATGAAACCC

AATCACGAAAGTAGCTTTAAACTGTCCTGACACCACGAAAGCCAAGAAA

>814_Sample_ASV46

CACCGCGGTTATACGAAAGACCCTAGTTGATAGATCACGGCGTAAAGGGTGGTTAAGGAA

AACAAAAATAAAGCTAAAAATCCTCTAAGCTGTCATACGCTTTCCGAAGACATGAGATCC

AACCACGAAAGTAGCTTTAAAATTTCCTGACACCACGAAAGCCAAGGAA

>815_Sample_ASV1

CACCGCGGTTATACGAAAGACCCTAGTTGATGGGTGCGGCGTAAAGGGTGGTTAGGGACT

ACAAAAAATAGAGCTAAAGATCCTCTAGGTCGTCATACACATCACGAGGAAACGAAGCCC

AAATACGAAAGTAGCTCTAAATATAATCCTGACGCCACGAAAGCCAAGAAA

>815_Sample_ASV2

CACCGCGGTTATACGAGAGACCCTAGTTGATTAAAGCGGCGTAAAGAGTGGTTATGGAAC

TATTCTTTTAAAGCAGAAAACCTCTCAAACTGTTATACGCACCCAGAGGTCTGAATCCCT

CACACGAAAGTGACTTTATTTATGCCTACCAGAACCCACGAAAGCTGGGACA

>815_Sample_ASV3

CACCGCGGTTATACGAGAGACCCTAGTTGATTAAAGCGGCGTAAAGAGTGGTTATGGAAC

TATTCTTTTAAAGCAGAAAACCTCTCAAACTGTTATACGCACCCAGAGGTCTGAATCCCT

CACACGAAAGTGACTTTATTTATGCCTACCAGAACCCACGAAAGCTGTGAAA

>815_Sample_ASV4

CACCGCGGTTATACGAGAGGCCCAAGTTGACAGACACCGGCGTAAAGAGTGGCTAGGGAA

AAATTACTACTAAAGCCGAACACCTTCAGAACTGTCATACGTTCCCGAAGATAAGAAGCC

CCACCACGAAAGTGACTTTATATTACCCGACCCCACGAAAGCTGTGAAA

>815_Sample_ASV5

CACCGCGGTTATACGAGAGGCCCAAGTTGACAGACACCGGCGTAAAGAGTGGCTAGGGAA

AAATTACTACTAAAGCCGAACACCTTCAGAACTGTCATACGTTCCCGAAGATAAGAAGCC

CCACCACGAAAGTGGCTTTATATTACCCGACCCCACGAAAGCTGTGAAA

>815_Sample_ASV6

CACCGCGGTTATACGAGAGGCCCAAGTTGACAGACACCGGCGTAAAGAGTGGCTAGGGAA

AAATTACTACTAAAGCCGAACACCCTCAGAACTGTCATACGTTCCCGAAGGTAAGAAGCC

CCACCACGAAAGTGGCTTTATAATACCCGACCCCACGAAAGCTGTGAAA

>815_Sample_ASV7

CACCGCGGTTATACGAGAGGCCCAAGTTGACAGACACCGGCGTAAAGAGTGGCTAAGGAA

AAATTTCTACTAAAGCCGAACACCTTCAGAACTGTCATACGTTCCCGAAGAAAAGAAGCC

CCACCACGAAAGTGGCTTTATATTACCCGACCCCACGAAAGCTGTGAAA

>815_Sample_ASV8

CACCGCGGTTAAACGAGAGACCCTAGTTGATCATCACGGCGTAAAGAGTGGTTAGGGGAT

TATCATAAATAAAGCCAAAGACCTCCCAAGCTGTCGCACGCATTCCGGAGGCGCGAAGCC

CACACACGAAAGTAGCTTTAACTATTGACCCTGATGCCACGAAAGCTAAGAAA

>815_Sample_ASV9

CGCCGCGGTTAAACGAGAGGCCCTAGTTGATGGAACAACGGCGTAAAGGGTGGTTAAGGA

TAGTGAGACAATAAAGTCGAATGGCCCTTTGGCTGTCATACGCTTCTGGGAGCCTGAAGC

CCACTATACGAAAGTAGCTTTAACAACGTCCACCTGACCCCACGAAAACTGAGAAA

>815_Sample_ASV10

CGCCGCGGTTAAACGAGAGGCCCTAGTTGATGGAACAACGGCGTAAAGGGTGGTTAAGGA

TAGTGAGACAATAAAGTCGAATGGCCCTTTGGCTGTCATACGCTTCTGGGAGCCTGAAGC

CCACTATACGAAAGTAGCTTTAACAATGTCCACCTGACCCCACGAAAACTGAGAAA

>815_Sample_ASV11

CACCGCGGTTATACGAAAGACCCTAGTTGATAGATCACGGCGTAAAGGGTGGTTAGGGAA

TACAAAAATAAAGCTAAAAATCCTCTAAGCTGTCATACGCTTCCCGAAGACATGAAACCC

AACCACGAAAGTAGCTTTAAACTCTCCTGACACCACGAAAGCCAAGGAA

>815_Sample_ASV12

CACCGCGGTTATACGAGAGGCCCAAGTTGATAAGCCCGGCGTAAAGAGTGGTTATGGAGA

ATAAAACACTAAAGCTAAAGACCCCCTAGGCTGTTATACGCACCTGGCGGCTCGAACCAC

CTATACGAAAGTAGCTTTACCCCCCTTCCACCAGAATCCACGACAGCTGGGGCA

>815_Sample_ASV13

CACCGCGGTTATACGAGAGGCCCAAGTTGATAAGCCCGGCGTAAAGAGTGGTTATGGAGA

ATAAAACACTAAAGCTAAAGACCCCCTAGGCTGTTATACGCACCTGGCGGCTCGAACCAC

CTATACGAAAGTAGCTTTACCCCCCTTCCACCAGAATCCACGAAAGCTGGGAAA

>815_Sample_ASV14

CACCGCGGTTATACGAGAGGCCCAAGTTGACAGACATCGGCGTAAAGAGTGGTTAAGGAA

ACCCCATTACTAAAGCCGAACGCCCTCAGAACTGTCATACGTTCCCGAAGGTAAGAAGCC

CCACTACGAAAGTGGCTTTAACCACACCCGACCCCACGAAAGCTGGGAAA

>815_Sample_ASV15

CACCGCGGTTATACGAGAGGCCCAAGTTGACAGACATCGGCGTAAAGAGTGGTTAAGGAA

ACCCCATTACTAAAGCCGAACGCCCTCAGAACTGTCATACGTTCCCGAAGGTAAGAAGCC

CCACTACGAAAGTGGCTTTAACCACACCCGACCCCACGAAAGCTGTGAAA

>815_Sample_ASV16

CACCGCGGTTAAACGAGAGGCCCTAGTTGATATTACAACGGCGTAAAGGGTGGTTAAGGA

AAGCAAGCAATAAAGCCGAATGGCCCTTTGGCTGTCATACGCTTCTAGGTGTCCGAAGCC

CAATATACGAAAGTAGCTTTAGAAAAGCCCACCTGACACCACGAAAGCTGAGGAA

>815_Sample_ASV17

CACCGCGGTTATACGAGAGGCCCAAGTTGACAGACACCGGCGTAAAGAGTGGTTAAGGAA

ATTCATCAACTAAAGCCGAATGCCCTCAGGACTGTTATACGTTTGCGAAGGTAAGAAGCC

CTACTACGAAAGTGGCTTTATTCCACCTGATTCCACGAAAGCTGCGGAA

>815_Sample_ASV18

CACCGCGGTTATACGAGAGGCCCCAGTTGATATTAGCGGCGTAAAGGGTGGTTAAGGACT

TTAATAACTAAAGCTAAACACTTCCCCGGCTGTTGTACGTAGACCGGAAGCTTGAAGCCC

AGACACGAAAGTAGCTTTATTCCCCCTGACCCCACGAAAGCTGAGAAA

>815_Sample_ASV19

CACCGCGGTCACACGATTAACCCAAGTCAATAGAAACCGGCGTAAAGAGTGTTTTAGATC

ACCCCCTCCCCAATAAAGCTAAAACTCACCTGAGTTGTAAAAAACTCCAGTTGACACAAA

ATAGACTACGAAAGTGGCTTTAACATATCTGAACACACAATAGCTAAGACC

>815_Sample_ASV20

CACCGCGGTTATACGAGAGGCCCTAGTTGATAAATACGGCGTAAAGAGTGGTTAGAGGAC

TCCCATAATAAAGCCAAAGACCCTCCGGGCCGTCACACGCATACGAGGGCACGAAGTTCA

CACACGAAAGTAGCTTTAAATTACCCGACGCCACGAAAGCTAAGAAA

>815_Sample_ASV21

CACCGCGGTTATACGAAAGACCCTAGTTTATAAGTACGGCGTAAAGGGTGGTTAGGGGAC

AAACAAAATAAAGCCAAAGACCTCCTAAGCCGTCATACGCTTACGGAGGCACGAAGCCCA

AACACGAAAGTAGCTTTACCGAACATGCCCGACTCCACGAAAGCTGGGAAA

>815_Sample_ASV22

CACCGCGGTTATACGAGAGACCCTAGTTGATAAATACGGCGTAAAGAGTGGTTATGGGAA

AAACAAATAAAGTCAAACAACCTCTTAGCTGTTATACGCATTATGAGAGCATGAAGCCCC

CTCACGAAAGTAACTTTAATACCTCCTGACCCCACGAAAGCTAAGAAA

>815_Sample_ASV23

CACCGCGGTTATACGAGAGGCTCAAGTTGATAAACCCCGGCGTAAAGAGTGGTTAAGGAA

AAATAGAAACTAAAGCCGAATGCTTTCAAGACTGTTATACGTTCCCGAGAGTAAGAAGAC

CAATTACGAAAGTGGCTTTACTTTACCTGACCCCACGAAAGCTACGACA

>815_Sample_ASV24

CACCGCGGTTATACGAGAGACCCTAATTGATAGACACGGCGTAAAGAGTGGTTTGGGAAA

GCCCAAAAATAAAGCTAAAGACCTTCTAAGCCGTCACACGCACGCCGAAGGCACGAAGCC

CTGACACGAAAGTAGCTTTACTATTACCCCCGACGCCACGAAAGCTAAGAAA

>815_Sample_ASV25

AACCGCGGTTATACGAGAGACCCTAATTGATAGCTCACGGCGTAAAGGGTGGTTTGGGGA

AACCTCAAAATAAAGCTAAAGACCTTCTAAGCCGTTACACGCATACCGAAGGCACGAGGC

CCTAACACGAAAGTAGCTTTACTATTACCCCTGACGCCACGAAAGCTAAGAAA

>815_Sample_ASV26

CACCGCGGTTATACGAAAGACCCTAGTTGATGGATCACGGCGTAAAGGGTGGTTAAGGGA

TATAAAAATAAAGCTAAAAATCCTCTTAGCTGTCATACGCTTTCCGAAGACATGAGGCCC

AATTACGAAAGTAGCTTTAAACCACCCTGACACCACGAAAGCCAAGAAA

>815_Sample_ASV27

CACCGCGGTTATACGAAAGACCCCAGTTTATAGATGCGGCGTAAAGGGTGGTTAAGAGAA

AATCAAAATAAGGCCAAAGATCCCCTAAGCAGTCATACGCTCCACGAGGACACGAAACCC

AAATACGAAAGTTGCCTTAATAAACTTCACCTGATCCCACGAAAGCTGAAAAA

>815_Sample_ASV28

CACCGCGGTTAAACGAGAGGCCCTAGTTGATAGTACAACGGCGTAAAGGGTGGTTAAGGA

TAGTGAAATAATAAAGTCGAATGGCCCTTTGGCTGTCATACGCTTCTAGGAGTCCGAAGC

CCAATATACGAAAGTAACTTTAAGAGCGCCCACCTGACCCCACGAAAACTGAGAAA

>815_Sample_ASV29

CACCGCGGTTATACGAAAGACCCTAGTTTATAGACACGGCGTAAAGGGTGGTTCGGGACT

AAACAAAATAAAGCCAAAGACCTCCTAAGCCGTCATACGCTCACGGAAGCACGAAGCCCA

AGTACGAAAGTAGCTTTACAAAACATGCCTGACTCCACGAAAGCTAGGAAA

>815_Sample_ASV30

CACCGCGGTTATACGAAAGACCCTAGTTTATAAGTACGGCGTAAAGGGTGGTTAGGGATA

TATTAAAAATAAAGTTAAAGAGCCTCTAAGCTGTCGCACGCATCCCGAGAGCTCGAGACC

CAGACACGAAAGTAACTTTAAAATACCTCACCTGACCCCACGAAAGCTAAGAAA

>815_Sample_ASV31

CACCGCGGTTATACGAAAGACCCTAGTTGATAGGCACGGCGTAAAGGGTGGTTAGGGATA

TATCAAAAATAAAGTTAAAGAGCCTCTAAGCTGTCGCACGCATCCCGAGAGCTCGAGACC

CAGACACGAAAGTAACTTTAAAATACCTCACCTGACCCCACGAAAGCTAAGAAA

>815_Sample_ASV32

CACCGCGGTTATACGAAAGACCCTAGTTGATAGGCACGGCGTAAAGGGTGGTTAGGGATA

TATTAAAAATAAAGTTAAAGAGCCTCTAAGCTGTCGCACGCATCCCGAGAGCTCGAGACC

CAGACACGAAAGTAACTTTAAAATACCTCACCTGACCCCACGAAAGCTAAGAAA

>815_Sample_ASV33

CACCGCGGTTATACGAGAGGCCCAAGTTGATACTCCACGGCGTAAAGAGTGGTTAGAAAA

GACCTACTACTAAAGCCGAACGCCTTCAAAGCTGTTATACGCATCCGAAGGTGAGAAGCC

CACCCACGAAAGTGGCTTTACAACCTTGACTCCACGAAAGCTATGACA

>815_Sample_ASV34

TACCGCGGTTATACGAGAGGCCCAAGTTGATAGTCTTCGGCGTAAAGAGTGGTTAAAGAA

GCAATTAGACTAAGGCTAAACCCTTCCAAAGCCGTCATACGCATCCGGAAGCATGAAACC

CACCCACGAAAGTAGCCTTATTTACCTTGACCCCACGAAAGCTGTGGAA

>815_Sample_ASV35

CACCGCGGTTATACGAGAGACCCCAGTTGATAAACACGGCGTAAAGAGTGGTTAAGGGCA

CAAAACAAATAAAGTCAAACAACCTCTTAGCTGTTATACGCATTATGAGGATATGAAGCC

CCCCTACGAAAGTAACTTTACCAACCCCTGACCCCACGAAAGCTAAGAAA

>815_Sample_ASV36

CACCGCGGTTATACGAGAGACCCTAGTTGATATATACGGCGTAAAGAGTGGTTTGGAATA

ACCAAGTAATATAGCCAAAGACCTCCCAAGCCGTCACACGCACCCCGGAGGCACGAAGCC

CAAACACGAAAGTAGCTTTATTAACTTACCGACGCCACGAAAGCTAAGAAA

>815_Sample_ASV37

CACCGCGGTTATACGAAAGACCCTAGTTTATAGGCACGGCGTAAAGGGTGGTTAGGGGAT

AAACAAAATAAAGCCAAAGACCTTCTAAGTCGTCATACACTCATGAAGGCACGAAGCCCA

AATACGAAAGTAACTTTACAACTTAGACCCGACTCCACGAAAGCTGGGGGA

>815_Sample_ASV38

CACCGCGGTTATACGAAAGACCCTAGTTTATAAGTACGGCGTAAAGGGTGGTTAGGGGAC

AAACAAAATAAAGCCAAAGACCTTCTAAGTCGTCATACACTCATGAAGGCACGAAGCCCA

AATACGAAAGTAACTTTACAACTTAGACCCGACTCCACGAAAGCTGGGAAA

>815_Sample_ASV39

CACCGCGGTTATACGAAAGACCCTAGTTGATAGCCACGGCGTAAAGGGTGGTTAAGGTAA

CTAATAAATAAAGCTAAAGAGCCTCTAAGCCGTCGCACGCATTCCGAGAGCTCGAAGCCC

AAACACGAAAGTAGCTTTAAAATAAAGCACACCTGACCCCACGAAAGCTAAGAAA

>815_Sample_ASV40

CACCGCGGTTATACGAGAGGCCCAAGTTGATAGACATCGGCGTAAAGAGTGGTTAAGGAA

GTCTCAAAACTAAAGCCGAATGCCTTCAAGACTGTTATACGTTTCCGAAGGTAAGAAGCC

CCACTACGAAAGTGGCTTTACTCCACCTGACTCCACGAAAGCTGTGAAA

>815_Sample_ASV41

CACCGCGGTTATACGAAAGACCCTAGTTGATAGACCACGGCGTAAAGGGTGGTTAAGGAA

CACAAAAATAAAGCTAAAGATCCTCTAAGCTGTCATACGCTTCCCGAAGACATGAAACCC

AATCACGAAAGTAGCTTTAAACTGTCCTGACACCACGAAAGCCAAGAAA

>815_Sample_ASV42

CACCGCGGTTATACGAGAGACCCTAATTGATAGACTACGGCGTAAAGAGTGGTTTAGAAC

AAAAATTAATAAAGCCAAAGATCCTCCTGGCCGTCGCACGCATTTCGAGGATACGAAGCC

CCACTACGAAAGTAGCTTTACTACAACTTTTCTGACCCCACGAAAGCTAAGAAA

>820_Sample_ASV1

CACCGCGGTTATACGAGAGGCCCAAGTTGACAGACATCGGCGTAAAGAGTGGTTAAGGAA

AACCTTCAACTAAAGTCGAACGCTCTCAAGACTGTCGTACGTTTCCGAGAGTATGAAGCC

CCACCACGAAAGTGACTTTACCTCACCTGACTCCACGAAAGCTGCGAAA

>820_Sample_ASV2

CACCACGGTTATACGAGAGGCCCAAGTTGAAAGACATCGGCGTAAAGAGTGGTTAAGGAA

AACCTTCTACTAAAGTCGAACGCTCTCAGGACTGTCGTACGTTTCCGAGAGTATGAAGCC

CCACCACGAAAGTGACTTTACCTCACCTGACTCCACGAAAGCTGCGAAA

>820_Sample_ASV3

CACCGCGGTTATACGAGAGACCCTAGTTGATTAAAGCGGCGTAAAGAGTGGTTATGGAAC

TATTCTTTTAAAGCAGAAAACCTCTCAAACTGTTATACGCACCCAGAGGTCTGAATCCCT

CACACGAAAGTGACTTTATTTATGCCTACCAGAACCCACGAAAGCTGGGACA

>820_Sample_ASV4

CACCGCGGTTATACGAAAGACCCTAGTTTATAGGTACGGCGTAAAGGGTGGTTAGGGGAC

AAACAAAATAAAGCCAAAGACCTCCTAAGCCGTCATACGCTCACGGAAGCACGAAGCCCA

AACACGAAAGTAGCTTTACCGAACATGCCCGACTCCACGAAAGCTGGGGAA

>820_Sample_ASV5

CACCGCGGTTATACGAGAGGCCCAAGTTGACAGACACCGGCGTAAAGAGTGGCTAGGGAA

AAATTACTACTAAAGCCGAACACCTTCAGAACTGTCATACGTTCCCGAAGATAAGAAGCC

CCACCACGAAAGTGACTTTATATTACCCGACCCCACGAAAGCTGGGGGA

>820_Sample_ASV6

CACCGCGGTTATACGAGAGGCCCAAGTTGACAGACACCGGCGTAAAGAGTGGCTAGGGAA

AAATTACTACTAAAGCCGAACACCTTCAGAACTGTCATACGTTCCCGAAGATAAGAAGCC

CCACCACGAAAGTGACTTTATATTACCCGACCCCACGAAAGCTGTGAAA

>820_Sample_ASV7

CACCGCGATTATACGAGAGGCCCAAGTTGACAGACATCGGCGTAAAGAGTGGTTAAGGAA

AAATTACTACTAAAGCCGAACACCTTCAGAACTGTCATACGTTCCCGAAGATAAGAAGCC

CCACCACGAAAGTGGCTTTATATTACCCGACCCCACGAAAGCTGTGAAA

>820_Sample_ASV8

CACCGCGGTTATACGAGAGGCCCAAGTTGACAGACACCGGCGTAAAGAGTGGCTAGGGAA

AAATTACTACTAAAGCCGAACACCTTCAGAACTGTCATACGTTCCCGAAGATAAGAAGCC

CCACCACGAAAGTGGCTTTATATTACCCGACCCCACGAAAGCTGTGAAA

>820_Sample_ASV9

CACCGCGGTTATACGAGAGGCCCAAGTTGACAGACACCGGCGTAAAGAGTGGCTAAGGAA

AAATTTCTACTAAAGCCGAACACCTTCAGAACTGTCATACGTTCCCGAAGAAAAGAAGCC

CCACCACGAAAGTGGCTTTATATTACCCGACCCCACGAAAGCTGTGAAA

>820_Sample_ASV10

CACCGCGGTTATACGAGAGACCCTAGTTGATATATACGGCGTAAAGAGTGGTTTGGGGTT

ATGCCTCCAAAATAAAGTCGAAGATCTCCCAAGCCGTCACACGCACCCCGGAGACACGAA

GCCCTAATACGAAGGTAGCTTTATTAATTCCCCGACGCCACGAAAGCTAAGAAA

>820_Sample_ASV11

CACCGCGGTTATACGAGAGACCCTAGTTGATATATACGGCGTAAAGAGTGGTTTGGGGTT

ATGCCTCCAAAATAAAGCCGAAGATCTCCCAAGCCGTCACACGCACCCCGGAGACACGAA

GCCCTAATACGAAGGTAGCTTTATTAATTCCCCGACGCCACGAAAGCTAAGAAA

>820_Sample_ASV12

CACCGCGGTTATACGAGAGACCCTAGTTGATATATACGGCGTAAAGAGTGGTTTGGGGTT

ATGCCTCCAAAATAAAGCCGAAGATCTCCCAAGCCGTCACACGCATCCCGGAGACACGAA

GCCCTAATACGAAGGTAGCTTTATTAATTCCCCGACGCCACGAAAGCTAAGAAA

>820_Sample_ASV13

CACCGCGGTTATACGAGAGACCCTAGTTGATATATACGGCGTAAAGAGTGGTTTGGGGTT

ATGCCTCCAAAATAAAGCCGAAGATCTCCCAAGCCGTCACACGCATCCCGGAGACACGAA

GCCCTAATACGAAGGTAGCTTTATTAGTACCCCGACGCCACGAAAGCTAAGAAA

>820_Sample_ASV14

CACCGCGGTTATACGAAAGACCCTAGTTTATAGGCACGGCGTAAAGGGTGGTTAGGGAAT

ACAAAAATAAAGCTAAAAATCCTCTAAGCTGTCATACGCTTCCCGAAGACATGAAACCCA

ACCACGAAAGTAGCTTTAAACTCTCCTGACACCACGAAAGCCAAGGAA

>820_Sample_ASV15

CACCGCGGTTATACGAAAGACCCTAGTTGATAGATCACGGCGTAAAGGGTGGTTAGGGAA

TACAAAAATAAAGCTAAAAATCCTCTAAGCTGTCATACGCTTCCCGAAGACATGAAACCC

AACCACGAAAGTAGCTTTAAACTCTCCTGACACCACGAAAGCCAAGGAA

>820_Sample_ASV16

CACCGCGGTTATACGAGAGGCCCAAGTTGATAAGCCCGGCGTAAAGAGTGGTTATGGAGA

ATAAAACACTAAAGCTAAAGACCCCCTAGGCTGTTATACGCACCTGGCGGCTCGAACCAC

CTATACGAAAGTAGCTTTACCCCCCTTCCACCAGAATCCACGACAGCTGGGGCA

>820_Sample_ASV17

CACCGCGGTTATACGAGAGGCCCTAGTTGATAGCCACGGCGCAAAGAGTGGTTAAGGAGC

CCCACTAAATAAAGTCGAACACTTCCTAGGCCGTTATACGTTTTCTAGAAGCACGAAACC

CAATTACACGAAAGCAACTTTATACTAAACAACCTGACCCCACGAAAGCTAAGAAA

>820_Sample_ASV18

CACCGCGGTTATACGAGAGGCCCAAGTTGACAGACATCGGCGTAAAGAGTGGTTAAGGAA

ACCCCATTACTAAAGCCGAACGCCCTCAGAACTGTCATACGTTCCCGAAGGTAAGAAGCC

CCACTACGAAAGTGGCTTTAACCACACCCGACCCCACGAAAGCTGTGAAA

>820_Sample_ASV19

CACCGCGGTTATACGAGAGGCCCAAGTTGACAGACACCGGCGTAAAGAGTGGTTAAGGAA

ATTCATCAACTAAAGCCGAATGCCCTCAGGACTGTTATACGTTTGCGAAGGTAAGAAGCC

CTACTACGAAAGTGGCTTTATTCCACCTGATTCCACGAAAGCTGCGGAA

>820_Sample_ASV20

CACCGCGGTTATACGAGAGGCCCCAGTTGATATTAGCGGCGTAAAGGGTGGTTAAGGACT

TTAATAACTAAAGCTAAACACTTCCCCGGCTGTTGTACGTAGACCGGAAGCTTGAAGCCC

AGACACGAAAGTAGCTTTATTCCCCCTGACCCCACGAAAGCTGAGAAA

>820_Sample_ASV21

CACCGCGGTTATACGAGAGGCCCCAGTTGATAAACACGGCGTAAAGGGTGGTTAAGGGAG

CATAAGAATAAAGCCGAAGGGCCCTTTGGCCGTTATACGCTTCTAGGTGTCCGAAGCCCA

AACACGAAAGTAGCTTTAGTTAAGCCCACCTGACTCCACGAAAACTGAGAAA

>820_Sample_ASV22

CACCGCGGTTATACGAGAGACCCTAGTTGATAAATACGGCGTAAAGAGTGGTTATGGGAA

AAACAAATAAAGTCAAACAACCTCTTAGCTGTTATACGCATTATGAGAGCATGAAGCCCC

CTCACGAAAGTAACTTTAATACCTCCTGACCCCACGAAAGCTAAGAAA

>820_Sample_ASV23

CACCGCGGTTATACGAGAGGCTCAAGTTGATAAACCCCGGCGTAAAGAGTGGTTAAGGAA

AAATAGAAACTAAAGCCGAATGCTTTCAAGACTGTTATACGTTCCCGAGAGTAAGAAGAC

CAATTACGAAAGTGGCTTTACTTTACCTGACCCCACGAAAGCTACGACA

>820_Sample_ASV24

CACCGCGGTTATACGAGAGGCTCAAGTTGATGAACCCCGGCGTAAAGAGTGGTTAAGGGA

GATCAAAACTAAAGCCGAATGCTTTCAAAGCTGTTATACGCTTCCGAAAGTAAGAAGCTC

AATCACGAAAGTGGCTTTACTTTACCTGACCCCACGAAAGCTACGACA

>820_Sample_ASV25

CACCGCGGTTATACGAGAGACCCTAATTGATAGACACGGCGTAAAGAGTGGTTTGGGAAA

GCCCAAAAATAAAGCTAAAGACCTTCTAAGCCGTCACACGCACGCCGAAGGCACGAAGCC

CTGACACGAAAGTAGCTTTACTATTACCCCCGACGCCACGAAAGCTAAGAAA

>820_Sample_ASV26

CACCGCGGTTATACGAAAGACCCTAGTTGATGGATCACGGCGTAAAGGGTGGTTAAGGGA

TATAAAAATAAAGCTAAAAATCCTCTTAGCTGTCATACGCTTTCCGAAGACATGAGGCCC

AATTACGAAAGTAGCTTTAAACCACCCTGACACCACGAAAGCCAAGAAA

>820_Sample_ASV27

CACCGCGGTTATACGAAAGACCCCAGTTTATAGATGCGGCGTAAAGGGTGGTTAAGAGAA

AATCAAAATAAGGCCAAAGATCCCCTAAGCAGTCATACGCTCCACGAGGACACGAAACCC

AAATACGAAAGTTGCCTTAATAAACTTCACCTGATCCCACGAAAGCTGAAAAA

>820_Sample_ASV28

CACCGCGGTTATACGAGAGGCCCAAGTTGACAAACCCCGGCGTAAAGAGTGGTTAAGGAA

AATTTAAAACTAAAGCCGAATGCCCCCAAAGCTGTTATACGCACCCGAGGGTAAGAAGCC

CAATCACGAAAGTGGCTTTACACTACCTGAACCCACGAAAGCTATGAAA

>820_Sample_ASV29

CACCGCGGTTATACGAGAGGCTCAAGTTGATGAACCCCGGCGTAAAGAGTGGTTAAGGAA

AATTTAAAACTAAAGCCGAATGCCCCCAAAGCTGTTATACGCACCCGAGGATAAGAAGCC

CAATCACGAAAGTGGCTTTACACTACCTGAACCCACGAAAGCTATGAAA

>820_Sample_ASV30

CACCGCGGTTATACGAGAGGCCCAAGTTGACAAACCCCGGCGTAAAGAGTGGTTAAGGAA

AATTTAAAACTAAAGCCGAATGCCCCCAAAGCTGTTATACGCACCCGAGGATAAGAAGCC

CAATCACGAAAGTGGCTTTACACTACCTGAACCCACGAAAGCTATGAAA

>820_Sample_ASV31

CACCGCGGTTATACGAGAGGCCCTAGTTGATAACTACCGGCGTAAAGAGTGGTTATGGAA

AAATATTTAATAAAGCCGAACACCCCCTCAGCCGTCATACGCACCTGGGAGCACGAAGAC

CTACTGCGAAAGCAGCTTTAATTATGCCTGACCCCACGACAGCTAAGAAA

>820_Sample_ASV32

CACCGCGGTTATACGAAAGACCCTAGTTTATAGGCACGGCGTAAAGGGTGGTTAGGGGAT

AAACAAAATAAAGCCAAAGACCTCCTAAGCCGTCATACGCTCACGGAAGCACGAAGCCCA

AGTACGAAAGTAGCTTTACAAAACATGCCTGACTCCACGAAAGCTAGGAAA

>820_Sample_ASV33

CACCGCGGTTATACGAAAGACCCTAGTTTATAGACACGGCGTAAAGGGTGGTTCGGGACT

AAACAAAATAAAGCCAAAGACCTCCTAAGCCGTCATACGCTCACGGAAGCACGAAGCCCA

AGTACGAAAGTAGCTTTACAAAACATGCCTGACTCCACGAAAGCTAGGAAA

>820_Sample_ASV34

CACCGCGGTTATACGAAAGACCCTAGTTTATAGACACGGCGTAAAGGGTGGTTCGGGACT

AAACAAAATAAAGCCAAAGACCTCCTAAGCCGTCATACGCTCACGGAAGCACGAAGCCCA

AGTACGAAAGTAGCTTTACAAAACATGCCTGACTCCACGAAAGCTGGGGGA

>820_Sample_ASV35

CACCGCGGTTATACGAAAGACCCTAGTTGATAGGCACGGCGTAAAGGGTGGTTAGGGATA

TATTAAAAATAAAGTTAAAGAGCCTCTAAGCTGTCGCACGCATCCCGAGAGCTCGAGACC

CAGACACGAAAGTAACTTTAAAATACCTCACCTGACCCCACGAAAGCTAAGAAA

>820_Sample_ASV36

CACCGCGGTTATACGAAAGACCCTAGTTGATAGGCACGGCGTAAAGGGTGGTTAGGGATA

TATCAAAAATAAAGTTAAAGAGCCTCTAAGCTGTCGCACGCATCCCGAGAGCTCGAGACC

CAGACACGAAAGTAACTTTAAAATACCTCACCTGACCCCACGAAAGCTAAGAAA

>820_Sample_ASV37

CACCGCGGTTATACGAGAGGCCCAAGTTGATACTCCACGGCGTAAAGAGTGGTTAGAAAA

GACCTACTACTAAAGCCGAACGCCTTCAAAGCTGTTATACGCATCCGAAGGTGAGAAGCC

CACCCACGAAAGTGGCTTTACAACCTTGACTCCACGAAAGCTATGACA

>820_Sample_ASV38

CACCGCGGTTATACGAGAGACCCCAGTTGATAAACACGGCGTAAAGAGTGGTTAAGGGCA

CAAAACAAATAAAGTCAAACAACCTCTTAGCTGTTATACGCATTATGAGGATATGAAGCC

CCCCTACGAAAGTAACTTTACCAACCCCTGACCCCACGAAAGCTAAGAAA

>820_Sample_ASV39

CACCGCGGTTATACGAGAGACCCTAGTTGATATATACGGCGTAAAGAGTGGTTTGGAATA

ACCAAGTAATATAGCCAAAGACCTCCCAAGCCGTCACACGCACCCCGGAGGCACGAAGCC

CAAACACGAAAGTAGCTTTATTAACTTACCGACGCCACGAAAGCTGTGAAA

>820_Sample_ASV40

CACCGCGGTTATACGAAAGACCCTAGTTTATAGGCACGGCGTAAAGGGTGGTTAGGGGAT

AAACAAAATAAAGCCAAAGACCTTCTAAGTCGTCATACACTCATGAAGGCACGAAGCCCA

AATACGAAAGTAACTTTACAACTTAGACCCGACTCCACGAAAGCTGGGGGA

>820_Sample_ASV41

CACCGCGGTTATACGAAAGACCCTAGTTTATAGGCACGGCGTAAAGGGTGGTTAGGGGAT

AAACAAAATAAAGCCAAAGACCTCCTAAGCCGTCATACGCTCACGGAAGCACGAAGCCCA

AGTACGAAAGTAGCTTTACAAAACATGCCTGACTCCACGAAAGCTGGGGGA

>822_Sample_ASV1

CACCGCGGTTATACGAAAGACCCTAGTTGATGGGTGCGGCGTAAAGGGTGGTTAGGGACT

ACAAAAAATAGAGCTAAAGATCCTCTAGGTCGTCATACACATCACGAGGAAACGAAGCCC

AAATACGAAAGTAGCTCTAAATATAATCCTGACGCCACGAAAGCCAAGAAA

>822_Sample_ASV2

CACCGCGGTTATACGAGAGACCCTAGTTGATTAAAGCGGCGTAAAGAGTGGTTATGGAAC

TATTCTTTTAAAGCAGAAAACCTCTCAAACTGTTATACGCACCCAGAGGTCTGAATCCCT

CACACGAAAGTGACTTTATTTATGCCTACCAGAACCCACGAAAGCTGGGACA

>822_Sample_ASV3

CACCGCGGTTATACGAAAGACCCTAGTTGATAGCCACGGCGTAAAGGGTGGTTAAGGAAC

CTAAAAAATAAAGCTAAAGACCCTCTAGGCCGTCATACGCATTACGAGGCAAACGAAACC

CCAACACGAAAGTAGCTTTAGACAAATATCCTGATGCCACGAAAGCCAAGAAA

>822_Sample_ASV4

CACCGCGGTTATACGAGAGGCCCAAGTTGACAGACACCGGCGTAAAGAGTGGCTAGGGAA

AAATTACTACTAAAGCCGAACACCTTCAGAACTGTCATACGTTCCCGAAGATAAGAAGCC

CCACCACGAAAGTGACTTTATATTACCCGACCCCACGAAAGCTGTGAAA

>822_Sample_ASV5

CACCGCGGTTATACGAAAGACCCAAGTTGACAGACACCGGCGTAAAGAGTGGCTAGGGAA

AAATTACTACTAAAGCCGAACACCTTCAGAACTGTCATACGTTCCCGAAGATAAGAAGCC

CCACCACGAAAGTGGCTTTATATTACCCGACCCCACGAAAGCTGTGAAA

>822_Sample_ASV6

CACCGCGGTTATACGAGAGGCCCAAGTTGACAGACACCGGCGTAAAGAGTGGCTAGGGAA

AAATTACTACTAAAGCCGAACACCTTCAGAACTGTCATACGTTCCCGAAGATAAGAAGCC

CCACCACGAAAGTGGCTTTATATTACCCGACCCCACGAAAGCTAAGAAA

>822_Sample_ASV7

CACCGCGGTTATACGAGAGGCCCAAGTTGACAGACACCGGCGTAAAGAGTGGCTAGGGAA

AAATTACTACTAAAGCCGAACACCTTCAGAACTGTCATACGTTCCCGAAGATAAGAAGCC

CCACCACGAAAGTGGCTTTATATTACCCGACCCCACGAAAGCTGTGAAA

>822_Sample_ASV8

CACCGCGGTTATACGAGAGGCCCAAGTTGACAGACACCGGCGTAAAGAGTGGCTAAGGAA

AAATTTCTACTAAAGCCGAACACCTTCAGAACTGTCATACGTTCCCGAAGAAAAGAAGCC

CCACCACGAAAGTGGCTTTATATTACCCGACCCCACGAAAGCTGTGAAA

>822_Sample_ASV9

CACCGCGGTTATACGAAAGACCCTAGTTGATAGATCACGGCGTAAAGGGTGGTTAGGGAA

TACAAAAATAAAGCTAAAAATCCTCTAAGCTGTCATACGCTTCCCGAAGACATGAAACCC

AACCACGAAAGTAGCTTTAAACTCTCCTGACACCACGAAAGCCAAGGAA

>822_Sample_ASV10

CACCGCGGTTATACGAAAGACCCAAGTTGATAGTCACGGCGTAAAGAGTGGTTATGGAGA

ATAAAACACTAAAGCTAAAGACCCCCTAGGCTGTTATACGCACCTGGCGGCTCGAACCAC

CTATACGAAAGTAGCTTTACCCCCCTTCCACCAGAATCCACGACAGCTGGGGCA

>822_Sample_ASV11

CACCGCGGTTATACGAGAGGCCCAAGTTGATAAGCCCGGCGTAAAGAGTGGTTATGGAGA

ATAAAACACTAAAGCTAAAGACCCCCTAGGCTGTTATACGCACCTGGCGGCTCGAACCAC

CTATACGAAAGTAGCTTTACCCCCCTTCCACCAGAATCCACGACAGCTGGGGCA

>822_Sample_ASV12

CACCGCGGTTATACGAGAGGCCCAAGTTGACAGACATCGGCGTAAAGAGTGGTTAAGGAA

ACCCCATTACTAAAGCCGAACGCCCTCAGAACTGTCATACGTTCCCGAAGGTAAGAAGCC

CCACTACGAAAGTGGCTTTAACCACACCCGACCCCACGAAAGCTGTGAAA

>822_Sample_ASV13

CACCGCGGTTATACGAGAGACCCCAGTTGATAGCTACGGCGTAAAGAGTGGTTAAGGACC

CCATTTAATAAAGCCAAAGACCCCCCAAGCCGTCATACGCACACGAGGGCGCGAAGCTCG

CACACGAAAGTAGCTCTAATTTACCCGACGCCACGAAAGCTAAGGAA

>822_Sample_ASV14

CACCGCGGTTATACGAGAGGCCCTAGTTGATAAATACGGCGTAAAGAGTGGTTAGAGGAC

TCCCATAATAAAGCCAAAGACCCCCCGGGCCGTCACACGCATACGAGGGCACGAAGTTCA

CACACGAAAGTAGCTTTAAATTACCCGACGCCACGAAAGCTAAGAAA

>822_Sample_ASV15

CACCGCGGTTATACGAGAGACCCTAGTTGATAAATACGGCGTAAAGAGTGGTTATGGGAA

AAACAAATAAAGTCAAACAACCTCTTAGCTGTTATACGCATTATGAGAGCATGAAGCCCC

CTCACGAAAGTAACTTTAATACCTCCTGACCCCACGAAAGCTAAGAAA

>822_Sample_ASV16

CACCGCGGTTATACGAGAGACCCTAATTGATAGACACGGCGTAAAGAGTGGTTTGGGAAA

GCCCAAAAATAAAGCTAAAGACCTTCTAAGCCGTCACACGCACGCCGAAGGCACGAAGCC

CTGACACGAAAGTAGCTTTACTATTACCCCCGACGCCACGAAAGCTAAGAAA

>822_Sample_ASV17

CACCGCGGTTATACGAAAGACCCTAGTTTATAGATGCGGCGTAAAGGGTGGTTAAGGAAA

ACCTAAAATAATGCTAAAGACTCTCAAAGCTGTTATACGCTTCATGAGCACACGAAATCC

AAATACGAAAGTTGCCTTAATAAACTTTGCCTGACCCCACGAAAGCTGAAAAA

>822_Sample_ASV18

CACCGCGGTTATACGAAAGACCCCAGTTTATAGATGCGGCGTAAAGGGTGGTTAAGAGAA

AATCAAAATAAGGCCAAAGATCCCCTAAGCAGTCATACGCTCCACGAGGACACGAAACCC

AAATACGAAAGTTGCCTTAATAAACTTCACCTGATCCCACGAAAGCTGAAAAA

>822_Sample_ASV19

CACCGCGGTTATACGAGAGACTCTAGTTGATACCAACGGCGTAAAGAGTGGTTTGGGATG

CCCCTTAAAATAAAGCCAAAGACCTCCCAAGCCGTCAAACGCACCCCGGAGGCACGAAGC

CCCAACGCGAAAGCAACTTTACCTCCCTCCCGACGCCACGAAAGCTAAGAAA

>822_Sample_ASV20

CACCGCGGTTATACGAGAGACCCTAGTTGATAGCTACGGCGTAAAGCGTGGTTAGGAGAA

CAAGATAAATAAAGTCAAACAATCTCTCGGCCGTTATACGTTATCTGAGAATATGAAGTC

CTACCACGAAAGTAACTTTAATTTTTCTGACCCCACGAAAACTGAGGAA

>822_Sample_ASV21

CACCGCGGTTAAACGAGAGGCCCTAGTTGATAGTACAACGGCGTAAAGGGTGGTTAAGGA

TAGTGAAATAATAAAGTCGAATGGCCCTTTGGCTGTCATACGCTTCTAGGAGTCCGAAGC

CCAATATACGAAAGTAACTTTAAGAGCGCCCACCTGACCCCACGAAAACTGAGAAA

>822_Sample_ASV22

CACCGCGGTTATACGAAAGACCCTAGTTGATAGGCACGGCGTAAAGGGTGGTTAGGGATA

TATTAAAAATAAAGTTAAAGAGCCTCTAAGCTGTCGCACGCATCCCGAGAGCTCGAGACC

CAGACACGAAAGTAACTTTAAAATACCTCACCTGACCCCACGAAAGCTAAGAAA

>822_Sample_ASV23

CACCGCGGTTATACGAGAGGCCCAAGTTGATACTCCACGGCGTAAAGAGTGGTTAGAAAA

GACCTACTACTAAAGCCGAACGCCTTCAAAGCTGTTATACGCATCCGAAGGTGAGAAGCC

CACCCACGAAAGTGGCTTTACAACCTTGACTCCACGAAAGCTATGACA

>822_Sample_ASV24

CACCGCGGTTATACGAGAGGCCCAAGTTGATACTCCACGGCGTAAAGAGTGGTTAGAAAA

GACCTACTACTAAAGCCGAACGCCTTCAAAGCTGTTATACGCATCCGAAGGTGAGAAGCC

CACCCACGAAAGTGGCTTTACAACCTTGACTCCACGAAAGCTAAGAAA

>822_Sample_ASV25

CACCGCGGTTATACGAGAGACCCCAGTTGATAAACACGGCGTAAAGAGTGGTTAAGGGCA

CAAAACAAATAAAGTCAAACAACCTCTTAGCTGTTATACGCATTATGAGGATATGAAGCC

CCCCTACGAAAGTAACTTTACCAACCCCTGACCCCACGAAAGCTAAGAAA

>822_Sample_ASV26

CACCGCGGTTATACGAGAGACCCTAGTTGATATATACGGCGTAAAGAGTGGTTTGGAATA

ACCAAGTAATATAGCCAAAGACCTCCCAAGCCGTCACACGCACCCCGGAGGCACGAAGCC

CAAACACGAAAGTAGCTTTATTAACTTACCGACGCCACGAAAGCTAAGAAA

>822_Sample_ASV27

CACCGCGGTTATACGAAAGACCCTAGTTTATAGGCACGGCGTAAAGGGTGGTTAGGGGAT

AAACAAAATAAAGCCAAAGACCTTCTAAGTCGTCATACACTCATGAAGGCACGAAGCCCA

AATACGAAAGTAACTTTACAACTTAGACCCGACTCCACGAAAGCTAAGAAA

>822_Sample_ASV28

CACCGCGGTTATACGAAAGACCCTAGTTTATAGGCACGGCGTAAAGGGTGGTTAGGGGAT

AAACAAAATAAAGCCAAAGACCTTCTAAGTCGTCATACACTCATGAAGGCACGAAGCCCA

AATACGAAAGTAACTTTACAACTTAGACCCGACTCCACGAAAGCTGGGGGA

>822_Sample_ASV29

CACCGCGGTTATACGAGAGACCCTAATTGATAGACTACGGCGTAAAGAGTGGTTTAGAAC

AAAAATTAATAAAGCCAAAGATCCTCCTGGCCGTCGCACGCATTTCGAGGATACGAAGCC

CCACTACGAAAGTAGCTTTACTACAACTTTTCTGACCCCACGAAAGCTAAGAAA

>822_Sample_ASV30

CACCGCGGTTATACGAAAGACCCTAGTTGATAGATCACGGCGTAAAGGGTGGTTAAGGAG

AACAAAAATAAAGCTAAAAATCCTCTAAGCTGTCATACGCTTTCCGAAGACATGAGACCC

AACCACGAAAGTAACTTTAAACTTTCCTGACACCACGAAAGCCAAGAAA

>830_Sample_ASV1

CACCGCAGTTATACGAGAGGCCCAAGTTGACAGACATCGGCGTAAAGAGTGGTTAAGGAA

AACCTTCAACTAAAGTCGAACGCTCTCAAAACTGTCGTACGTTTCCGAGAGTATGAAGCC

CCACCACGAAAGTGACTTTACCTCACCTGACTCCACGAAAGCTGCGAAA

>830_Sample_ASV2

CACCGCGGTTATACGAGAGGCCCAAGTTGACAGACATCGGCGTAAAGAGTGGTTAAGGAA

AACCTTCAACTAAAGTCGAACGCTCTCAAGACTGTCGTACGTTTCCGAGAGTATGAAGCC

CCACCACGAAAGTGACTTTACCTCACATGACTCCACGAAAGCTGCGAAA

>830_Sample_ASV3

CACCGCGGTTATACGAGAGGCCCAAGTTGACAGACATCGGCGTAAAGAGTGGTTAAGGAA

AACCTTCAACTAAAGTCGAACGCTCTCAAGACTGTCGTACGTTTCCGAGAGTATGAAGCC

CCACCACGAAAGTGACTTTACCTCACCTGACTCCACGAAAGCTGCGAAA

>830_Sample_ASV4

CACCGCGGTTATACGAAAGACCCTAGTTGATGGGTGCGGCGTAAAGGGTGGTTAGGGACT

ACAAAAAATAGAGCTAAAGATCCTCTAGGTCGTCATACACATCACGAGAAAACGAAGCCC

AAATACGAAAGTAGCTCTAAATATAATCCTGACGCCACGAAAGCCAAGAAA

>830_Sample_ASV5

CACCGCGGTTATACGAAAGACCCTAGTTGATGGGTGCGGCGTAAAGGGTGGTTAGGGACT

ACAAAAAATAGAGCTAAAGATCCTCTAGGTCGTCATACACATCACGAGGAAACGAAGCCC

AAATACGAAAGTAGCTCTAAATATAATCCTGACGCCACGAAAGCCAAGAAA

>830_Sample_ASV6

CACCGCGGTTATACGAGAGACCCTAGTTGATTAAAGCGGCGTAAAGAGTGGTTATGGAAC

TATTCTTTTAAAGCAGAAAACCTCTCAAACTGTTATACGCACCCAGAGGTCTGAATCCCT

CACACGAAAGTGACTTTATTTATGCCTACCAGAACCCACGAAAGCTGTGAAA

>830_Sample_ASV7

CACCGCGGTTATACGAGAGACCCTAGTTGATTAAAGCGGCGTAAAGAGTGGTTATGGAAC

TATTCTTTTAAAGCAGAAAACCTCTCAAACTGTTATACGCACCCAGAGGTCTGAATCCCT

CACACGAAAGTGACTTTATTTATGCCTACCAGAACCCACGAAAGCTGGGACA

>830_Sample_ASV8

AGCCGCGGTAATACGAGAGGCCCAAGTTGACAGACACCGGCGTAAAGAGTGGCTAGGGAA

AAATTACTACTAAAGCCGAACACCTTCAGAACTGTCATACGTTCCCGAAGATAAGAAGCC

CCACCACGAAAGTGGCTTTATATTACCCGACCCCACGAAAGCTGTGAAA

>830_Sample_ASV9

CATGGCGATTATACGAGAGGCCCAAGTTGACAGACACTGGCGTAAAGAGTGGCTAAGGAA

AAATTACTACTAAAGCCGAACACCTTCAGAACTGTCATACGTTCCCGAAGATAAGAAGCC

CCACCACGAAAGTGGCTTTATATTACCCGACCCCACGAAAGCTGTGAAA

>830_Sample_ASV10

CATGGCGATTATACGAGAGGCCCAAGTTGACAGACACTGGCGTAAAGAGTGGCTAAGGAA

AAACTACTACTAAAGCCGAACACCTTCAGAACTGTCATACGTTCCCGAAGATAAGAAGCC

CCACCACGAAAGTGGCTTTATATTACCCGACCCCACGAAAGCTGTGAAA

>830_Sample_ASV11

CACCGCGGTTATACGAGAGGCCCAAGTTGACAGACACCGGCGTAAAGAGTGGCTAGGGAA

AAATTACTACTAAAGCCGAACACCTTCAGAACTGTCATACGTTCCCGAAGATAAGAAGCC

CCACCACGAAAGTGGCTTTATATTACCCGACCCCACGAAAGCTGTGAAA

>830_Sample_ASV12

CACCGCGGTTATACGAGAGGCCCAAGTTGACAGACACCGGCGTAAAGAGTGGCTAGGGAA

AAATTACTACTAAAGCCGAACACCTTCAGAACTGTCATACGTTCCCGAAGATAAGAAGCC

CCACCACGAAAGTGGCTTTATATTACCCGACCCCACGAAAGCGTGGGGAG

>830_Sample_ASV13

CACCGCGGTTATACGAGAGGCCCAAGTTGACAGACACCGGCGTAAAGAGTGGCTAGGGAA

AAATTACTACTAAAGCCGAACACCTTCAGAACTGTCATACGTTCCCGAAGATAAGAAGCC

CCACCACGAAAGTGGCTTTATATTACCCGACCCCTCGAAAGCGTGGGGAG

>830_Sample_ASV14

CACCGCGGTTATACGAAAGACCCTAGTTTATAGAAACGGCGTAAAGGGTGGTTAAGGGAC

AAACAAAATAAGGCCAAAGACTTCCTAAGCCGTCATACGCTCCACGAAAGCACGAAGCCC

AAACACGAAAGTAGCCTTAATAACTTCACCTGACCCCACGAAAGCTGGGAGA

>830_Sample_ASV15

CACCGCGGTTATACGAGAGGCCCAAGTTGATAAGCCCGGCGTAAAGAGTGGTTATGGAGA

ATAAAACACTAAAGCTAAAGACCCCCTAGGCTGTTATACGCACCTGGCGGCTCGAACCAC

CTATACGAAAGTAGCTTTACCCCCCTTCCACCAGAATCCACGACAGCTGGGGCA

>830_Sample_ASV16

CACCGCGGTTATACGAGAGGCCCTAGTTGATAGCCACGGCGCAAAGAGTGGTTAAGGAGC

CCCACTAAATAAAGTCGAACACTTCCTAGGCCGTTATACGTTTTCTAGAAGCACGAAACC

CAATTACACGAAAGCAACTTTATACTAAACAACCTGACCCCACGAAAGCTAAGAAA

>830_Sample_ASV17

CACCGCGGTTATACGAGAGGCCCAAGTTGACAGACATCGGCGTAAAGAGTGGTTAAGGAA

ACCCCATTACTAAAGCCGAACGCCCTCAGAACTGTCATACGTTCCCGAAGGTAAGAAGCC

CCACTACGAAAGTGGCTTTAACCACACCCGACCCCACGAAAGCTGTGAAA

>830_Sample_ASV18

CACCGCGGTTATACGAGAGGCCCAAGTTGACAGACATCGGCGTAAAGAGTGGTTAAGGAA

ACCCCATTACTAAAGCCGAACGCCCTCAGAACTGTCATACGTTCCCGAAGGTAAGAAGCC

CCACTACGAAAGTGGCTTTAACCACACCCGACCCCACGAAAGCTAAGAAA

>830_Sample_ASV19

CACCGCGGTTATACGAGAGGCCCCAGTTGATATTAGCGGCGTAAAGGGTGGTTAAGGACT

TTAATAACTAAAGCTAAACACTTCCCCGGCTGTTGTACGTAGACCGGAAGCTTGAAGCCC

AGACACGAAAGTAGCTTTATTCCCCCTGACCCCACGAAAGCTGAGAAA

>830_Sample_ASV20

CACCGCGGTCACACGATTAACCCAAGTCAATAGAAGCCGGCGTAAAGAGTGTTTTAGATC

ACCCCCTCCCCAATAAAGCTAAAACTCACCTGAGTTGTAAAAAACTCCAGTTGACACAAA

ATAGACTACGAAAGTGGCTTTAACATATCTGAACACACAATAGCTAAGACC

>830_Sample_ASV21

CACCGCGGTTATACGAAAGACCCCAGTTTATAGACACGGCGTAAAGGGTGGTTATAGGAC

AAACTAACATAGAGCCAAAGACCCCCTAAGCCGTCATACGCTCACGAGGGCTCGAAGCCC

CTATACGAAAGTAGCTCTACCAAAAAATGCTCGACTCCACGAAAGCTGGGACA

>830_Sample_ASV22

AACCGCGGTTATACGAGAGACCCTAATTGATAGCTCACGGCGTAAAGGGTGGTTTGGGGA

AACCTCAAAATAAAGCTAAAGACCTTCTAAGCCGTTACACGCATACCGAAGGCACGAGGC

CCTAACACGAAAGTAGCTTTACTATTACCCCTGACGCCACGAAAGCTAAGAAA

>830_Sample_ASV23

CACCGCGGTTATACGAAAGACCCTAGTTGATGGATCACGGCGTAAAGGGTGGTTAAGGGA

TATAAAAATAAAGCTAAAAATCCTCTTAGCTGTCATACGCTTTCCGAAGACATGAGGCCC

AATTACGAAAGTAGCTTTAAACCACCCTGACACCACGAAAGCCAAGAAA

>830_Sample_ASV24

CACCGCGGTTATACGAGAGACTCTAGTTGATACCAACGGCGTAAAGAGTGGTTTGGGATG

CCCCTTAAAATAAAGCCAAAGACCTCCCAAGCCGTCAAACGCACCCCGGAGGCACGAAGC

CCCAACGCGAAAGCAACTTTACCTCCCTCCCGACGCCACGAAAGCTAAGAAA

>830_Sample_ASV25

CACCGCGGTTATACGAGAGGCCCTAGTTGATAACTACCGGCGTAAAGAGTGGTTATGGAA

AAATATTTAATAAAGCCGAACACCCCCTCAGCCGTCATACGCACCTGGGAGCACGAAGAC

CTACTGCGAAAGCAGCTTTAATTATGCCTGACCCCACGACAGCTAAGAAA

>830_Sample_ASV26

CACCGCGGTTATACGAGAGGCCCAAGTTGATTATTACCGGCGTAAAGCGTGGTTAAGATT

TTTATAGGACTAAAGCCGAACCCCCTCAGAGCTGTTATACGCACCCGAAGGTTAGAAGTT

CAATCACGAAAGTGGCTTTACAACCCCGAACCCACGAAAGCTAAGATA

>830_Sample_ASV27

CACCGCGGTTATACGAGAGACCCTAATTGATAGGCGCGGCGTAAAGAGTGGTTTAGGACT

AAAACTAATAAAGCTAAAGATCCTCCCAGCCGTCGCACGCATTCTGAGGACACGAAACTC

AACCACGAAAGTAGCTTTAACATACTCTCCTGACCCCACGAAAGCTAAGAAA

>830_Sample_ASV28

CACCGCGGTTATACGAAAGACCCTAGTTGATAGGCACGGCGTAAAGGGTGGTTAGGGATA

TATCAAAAATAAAGTTAAAGAGCCTCTAAGCTGTCGCACGCATCCCGAGAGCTCGAGACC

CAGACACGAAAGTAACTTTAAAATACCTCACCTGACCCCACGAAAGCTAAGAAA

>830_Sample_ASV29

CACCGCGGTTATACGAAAGACCCTAGTTGATAGGCACGGCGTAAAGGGTGGTTAGGGATA

CACCAAAAATAAAGTTAAAGAGCCTCTAAGCTGTCGCACGCATCCCGAGAGCTCGAGACC

CAGACACGAAAGTAACTTTAAAATACCTCACCTGACCCCACGAAAGCTAAGAAA

>830_Sample_ASV30

CACCGCGGTTATACGAAAGACCCTAGTTGATAGCCACGGCGTAAAGGGTGGTTAAGGTAT

ACAACAAATAAAGCTAAATAGCCTCTAGGCCGTCGCACGCATTCCGAGAGCTCGAAACCC

AAAAACGAAAGTAGCTTTAAAATAAACAGACCTGACCCCACGAAAGCTAAGAAA

>830_Sample_ASV31

CACCGCGGTTATACGAAAGACCCTAGTTGATAGCCACGGCGTAAAGGGTGGTTAAGGTAT

ACAACAAATAAAGCTAAATAGCCTCTAGGCCGTCGCACGCATTCCGAGAGCTCGAAACCC

AAAAACGAAAGTAGCTTTAAAATAAACAGACCTGACCCCACGAAAGCTGTGAAA

>830_Sample_ASV32

CACCGCGGTTATACGAGAGGCCCAAGTTGATAGCCACGGCGTAAAGGGTGGTTAAGGTAT

ACAACAAATAAAGCTAAATAGCCTCTAGGCCGTCGCACGCATTCCGAGAGCTCGAAACCC

AAAAACGAAAGTAGCTTTAAAATAAACAGACCTGACCCCACGAAAGCTAAGAAA

>830_Sample_ASV33

CACCGCGGTTATACGAAAGACCCTAGTTTATAGGCACGGCGTAAAGGGTGGTTAGGGGAT

AAACAAAATAAAGCCAAAGACCTTCTAAGTCGTCATACACTCATGAAGGCACGAAGCCCA

AATACGAAAGTAACTTTACAACTTAGACCCGACTCCACGAAAGCTGGGGGA

>830_Sample_ASV34

CACCGCGGTTATACGAGAGACCCTAATTAATAGATACGGCGTAAAGAGTGGTTTAGGACA

AAAATTAATAAAGCCGAAGTTTCTCCTAGCCGTTGTACGCACTCTGAGAACACGAAGCCC

CACTACGAAAGTAGCTTTACCTATTATTTCCTGACCCCACGAAAGCTAAGAAA

>830_Sample_ASV35

CACCGCGGTTATACGAGAGACCCTAATTAATAGCTACGGCGTAAAGAGTGGTTTAGGGTA

AAAATTAATAAAGCCGAAGTTTCTCCTAGCCGTTGTACGCACTCTGAGAACACGAAGCCC

CACTACGAAAGTAGCTTTACCTATTATTTCCTGACCCCACGAAAGCTAAGAAA

>830_Sample_ASV36

CACCGCGGTTATACGAAAGACCCTAGTTGATAGATCACGGCGTAAAGGGTGGTTAAGGAA

AACAAAAATAAAGCTAAAAATCCTCTAAGCTGTCATACGCTTTCCGAAGACATGAGATCC

AACCACGAAAGTAGCTTTAAAATTTCCTGACACCACGAAAGCCAAGGAA

>831_Sample_ASV1

CACCGCGGTTATACGAAAGACCCTAGTTGATGGGTGCGGCGTAAAGGGTGGTTAGGGACT

ACAAAAAATAGAGCTAAAGATCCTCTAGGTCGTCATACACATCACGAGGAAACGAAGCCC

AAATACGAAAGTAGCTCTAAATATAATCCTGACGCCACGAAAGCCAAGAAA

>831_Sample_ASV2

CACCGCGGTTATACGAGAGACCCTAGTTGATTAAAGCGGCGTAAAGAGTGGTTATGGAAC

TATTCTTTTAAAGCAGAAAACCTCTCAAACTGTTATACGCACCCAGAGGTCTGAATCCCT

CACACGAAAGTGACTTTATTTATGCCTACCAGAACCCACGAAAGCTGGGACA

>831_Sample_ASV3

CACCGCGGTTATACGAGAGGCCCAAGTTGACAGACACCGGCGTAAAGAGTGGCTAGGGAA

AAATTACTACTAAAGCCGAACACCTTCAGAACTGTCATACGTTCCCGAAGATAAGAAGCC

CCACCACGAAAGTGGCTTTATATTACCCGACCCCACGAAAGCTAAGAAA

>831_Sample_ASV4

CACCGCGGTTATACGAGAGGCCCAAGTTGACAGACACCGGCGTAAAGAGTGGCTAGGGAA

AAATTACTACTAAAGCCGAACACCTTCAGAACTGTCATACGTTCCCGAAGATAAGAAGCC

CCACCACGAAAGTGGCTTTATATTACCCGACCCCACGAAAGCTGTGAAA

>831_Sample_ASV5

CACCGCGGTTATACGAGAGGCCCAAGTTGACAGACACCGGCGTAAAGAGTGGCTAAGGAA

AAATTTCTACTAAAGCCGAACACCTTCAGAACTGTCATACGTTCCCGAAGAAAAGAAGCC

CCACCACGAAAGTGGCTTTATATTACCCGACCCCACGAAAGCTGTGAAA

>831_Sample_ASV6

CACCGCGGTTAAACGAGAGACCCTAGTTGATCATCACGGCGTAAAGAGTGGTTAGGGGAT

TATCATAAATAAAGCCAAAGACCTCCCAAGCTGTCGCACGCATTCCGGAGGCGCGAAGCC

CACACACGAAAGTAGCTTTAACTATTGACCCTGATGCCACGAAAGCTAAGAAA

>831_Sample_ASV7

CACCGCGGTTATACGAAAGACCCTAGTTGATAGATCACGGCGTAAAGGGTGGTTAGGGAA

TACAAAAATAAAGCTAAAAATCCTCTAAGCTGTCATACGCTTCCCGAAGACATGAAACCC

AACCACGAAAGTAGCTTTAAACTCTCCTGACACCACGAAAGCCAAGGAA

>831_Sample_ASV8

CACCGCGGTTATACGAGAGGCCCAAGTTGATAAGCCCGGCGTAAAGAGTGGTTATGGAGA

ATAAAACACTAAAGCTAAAGACCCCCTAGGCTGTTATACGCACCTGGCGGCTCGAACCAC

CTATACGAAAGTAGCTTTACCCCCCTTCCACCAGAATCCACGACAGCTGGGGCA

>831_Sample_ASV9

CACCGCGGTTATACGAGAGGCCCAAGTTGACAGACATCGGCGTAAAGAGTGGTTAAGGAA

ACCCCATTACTAAAGCCGAACGCCCTCAGAACTGTCATACGTTCCCGAAGGTAAGAAGCC

CCACTACGAAAGTGGCTTTAACCACACCCGACCCCACGAAAGCTGTGAAA

>831_Sample_ASV10

CACCGCGGTTAAACGAGAGGCCCTAGTTGATATTACAACGGCGTAAAGGGTGGTTAAGGA

AAGCAAGCAATAAAGCCGAATGGCCCTTTGGCTGTCATACGCTTCTAGGTGTCCGAAGCC

CAATATACGAAAGTAGCTTTAGAAAAGCCCACCTGACACCACGAAAGCTGAGGAA

>831_Sample_ASV11

CACCGCGGTTATACGAGAGGCCCTAGTTGATAAATACGGCGTAAAGAGTGGTTAGAGGAC

TCCCATAATAAAGCCAAAGACCCCCCGGGCCGTCACACGCATACGAGGGCACGAAGTTCA

CACACGAAAGTAGCTTTAAATTACCCGACGCCACGAAAGCTAAGAAA

>831_Sample_ASV12

CACCGCGGTTATACGAAAGACCCTAGTTTATAAGTACGGCGTAAAGGGTGGTTAGGGGAC

AAACAAAATAAAGCCAAAGACCTCCTAAGCCGTCATACGCTTACGGAGGCACGAAGCCCA

AACACGAAAGTAGCTTTACCGAACATGCCCGACTCCACGAAAGCTGGGAAA

>831_Sample_ASV13

CACCGCGGTTATACGAGAGACCCTAGTTGATAAATACGGCGTAAAGAGTGGTTATGGGAA

AAACAAATAAAGTCAAACAACCTCTTAGCTGTTATACGCATTATGAGAGCATGAAGCCCC

CTCACGAAAGTAACTTTAATACCTCCTGACCCCACGAAAGCTAAGAAA

>831_Sample_ASV14

CACCGCGGTTATACGAGAGGCTCAAGTTGATAAACCCCGGCGTAAAGAGTGGTTAAGGAA

AAATAGAAACTAAAGCCGAATGCTTTCAAGACTGTTATACGTTCCCGAGAGTAAGAAGAC

CAATTACGAAAGTGGCTTTACTTTACCTGACCCCACGAAAGCTACGACA

>831_Sample_ASV15

AACCGCGGTTATACGAGAGACCCTAATTGATAGCTCACGGCGTAAAGGGTGGTTTGGGGA

AACCTCAAAATAAAGCTAAAGACCTTCTAAGCCGTTACACGCATACCGAAGGCACGAGGC

CCTAACACGAAAGTAGCTTTACTATTACCCCTGACGCCACGAAAGCTAAGAAA

>831_Sample_ASV16

CACCGCGGTTATACGAAAGACCCTAGTTGATGGATCACGGCGTAAAGGGTGGTTAAGGGA

TATAAAAATAAAGCTAAAAATCCTCTTAGCTGTCATACGCTTTCCGAAGACATGAGGCCC

AATTACGAAAGTAGCTTTAAACCACCCTGACACCACGAAAGCCAAGAAA

>831_Sample_ASV17

CACCGCGGTTATACGAAAGACCCCAGTTTATAGATGCGGCGTAAAGGGTGGTTAAGAGAA

AATCAAAATAAGGCCAAAGATCCCCTAAGCAGTCATACGCTCCACGAGGACACGAAACCC

AAATACGAAAGTTGCCTTAATAAACTTCACCTGATCCCACGAAAGCTGAAAAA

>831_Sample_ASV18

CACCGCGGTTATACGAGAGACTCTAGTTGATACCAACGGCGTAAAGAGTGGTTTGGGATG

CCCCTTAAAATAAAGCCAAAGACCTCCCAAGCCGTCAAACGCACCCCGGAGGCACGAAGC

CCCAACGCGAAAGCAACTTTACCTCCCTCCCGACGCCACGAAAGCTAAGAAA

>831_Sample_ASV19

CACCGCGGTTAAACGAGAGGCCCTAGTTGATAGTACAACGGCGTAAAGGGTGGTTAAGGA

TAGTGAAATAATAAAGTCGAATGGCCCTTTGGCTGTCATACGCTTCTAGGAGTCCGAAGC

CCAATATACGAAAGTAACTTTAAGAGCGCCCACCTGACCCCACGAAAACTGAGAAA

>831_Sample_ASV20

CACCGCGGTTATACGAGAGGCCCAAGTTGATACTCCACGGCGTAAAGAGTGGTTAGAAAA

GACCTACTACTAAAGCCGAACGCCTTCAAAGCTGTTATACGCATCCGAAGGTGAGAAGCC

CACCCACGAAAGTGGCTTTACAACCTTGACTCCACGAAAGCTATGACA

>831_Sample_ASV21

CACCGCGGTTATACGAGAGACCCCAGTTGATAAACACGGCGTAAAGAGTGGTTAAGGGCA

CAAAACAAATAAAGTCAAACAACCTCTTAGCTGTTATACGCATTATGAGGATATGAAGCC

CCCCTACGAAAGTAACTTTACCAACCCCTGACCCCACGAAAGCTAAGAAA

>831_Sample_ASV22

CACCGCGGTTATACGAGAGACCCTAGTTGATATATACGGCGTAAAGAGTGGTTTGGAATA

ACCAAGTAATATAGCCAAAGACCTCCCAAGCCGTCACACGCACCCCGGAGGCACGAAGCC

CAAACACGAAAGTAGCTTTATTAACTTACCGACGCCACGAAAGCTAAGAAA

>831_Sample_ASV23

CACCGCGGTTATACGAAAGACCCTAGTTTATAGGCACGGCGTAAAGGGTGGTTAGGGGAT

AAACAAAATAAAGCCAAAGACCTTCTAAGTCGTCATACACTCATGAAGGCACGAAGCCCA

AATACGAAAGTAACTTTACAACTTAGACCCGACGCCACGAAAGCTAAGAAA

>831_Sample_ASV24

CACCGCGGTTATACGAAAGACCCTAGTTTATAGGCACGGCGTAAAGGGTGGTTAGGGGAT

AAACAAAATAAAGCCAAAGACCTTCTAAGTCGTCATACACTCATGAAGGCACGAAGCCCA

AATACGAAAGTAACTTTACAACTTAGACCCGACTCCACGAAAGCTAAGAAA

>831_Sample_ASV25

CACCGCGGTTATACGAAAGACCCTAGTTTATAGGCACGGCGTAAAGGGTGGTTAGGGGAT

AAACAAAATAAAGCCAAAGACCTTCTAAGTCGTCATACACTCATGAAGGCACGAAGCCCA

AATACGAAAGTAACTTTACAACTTAGACCCGACTCCACGAAAGCTGGGGGA

>831_Sample_ASV26

CACCGCGGTTATACGAGAGGCCCAAGTTGATAGACATCGGCGTAAAGAGTGGTTAAGGAA

GTCTCAAAACTAAAGCCGAATGCCTTCAAGACTGTTATACGTTTCCGAAGGTAAGAAGCC

CCACTACGAAAGTGGCTTTACTCCACCTGACTCCACGAAAGCTGTGAAA

>831_Sample_ASV27

CACCGCGGTTATACGAAAGACCCTAGTTGATAGACCACGGCGTAAAGGGTGGTTAAGGAA

CACAAAAATAAAGCTAAAGATCCTCTAAGCTGTCATACGCTTCCCGAAGACATGAAACCC

AATCACGAAAGTAGCTTTAAACTGTCCTGACACCACGAAAGCCAAGAAA

>831_Sample_ASV28

CACCGCGGTTATACGAGAGACCCTAATTGATAGACTACGGCGTAAAGAGTGGTTTAGAAC

AAAAATTAATAAAGCCAAAGATCCTCCTGGCCGTCGCACGCATTTCGAGGATACGAAGCC

CCACTACGAAAGTAGCTTTACTACAACTTTTCTGACCCCACGAAAGCTAAGAAA

>835_Sample_ASV1

CACCGCGGTTATACGAAAGACCCTAGTTGATGGGTGCGGCGTAAAGGGTGGTTAGGGACT

ACAAAAAATAGAGCTAAAGATCCTCTAGGTCGTCATACACATCACGAGGAAACGAAGCCC

AAATACGAAAGTAGCTCTAAATATAATCCTGACGCCACGAAAGCCAAGAAA

>835_Sample_ASV2

CACCGCGGTTATACGAAAGACCCTAGTTGATGGGTGCGGCGTAAAGGGTGGTTAGGGACT

ACAAAAAATAGAGCTAAAGATCCTCTAGGTCGTCATACACATCACGAGAAAACGAAGCCC

AAATACGAAAGTAGCTCTAAATATAATCCTGACGCCACGAAAGCCAAGAAA

>835_Sample_ASV3

CACCGCGGTTATACGAGAGACCCTAGTTGATTAAAGCGGCGTAAAGAGTGGTTATGGAAC

TATTCTTTTAAAGCAGAAAACCTCTCAAACTGTTATACGCACCCAGAGGTCTGAATCCCT

CACACGAAAGTGACTTTATTTATGCCTACCAGAACCCACGAAAGCTGGGACA

>835_Sample_ASV4

CACCGCGGTTATACGAAAGACCCTAGTTTATAGGTACGGCGTAAAGGGTGGTTAGGGGAC

AAACAAAATAAAGCCAAAGACCTCCTAAGCCGTCATACGCTCACGGAAGCACGAAGCCCA

AACACGAAAGTAGCTTTACCGAACATGCCCGACTCCACGAAAGCTGGGGAA

>835_Sample_ASV5

CACCGCGGTTATACGAGAGGCCCAAGTTGACAGACACCGGCGTAAAGAGTGGCTAGGGAA

AAATTACTACTAAAGCCGAACACCTTCAGAACTGTCATACGTTCCCGAAGATAAGAAGCC

CCACCACGAAAGTGACTTTATATTACCCGACCCCACGAAAGCCAAGAAA

>835_Sample_ASV6

CACCGCGGTTATACGAGAGGCCCAAGTTGACAGACACCGGCGTAAAGAGTGGCTAGGGAA

AAATTACTACTAAAGCCGAACACCTTCAGAACTGTCATACGTTCCCGAAGATAAGAAGCC

CCACCACGAAAGTGACTTTATATTACCCGACCCCACGAAAGCTGTGAAA

>835_Sample_ASV7

CACCGCGGTTATACGAGAGGCCCAAGTTGACAGACACCGGCGTAAAGAGTGGCTAGGGAA

AAATTACTACTAAAGCCGAACACCTTCAGAACTGTCATACGTTCCCGAAGATAAGAAGCC

CCACCACGAAAGTGGCTTTATATTACCCGACCCCACGAAAGCTGTGAAA

>835_Sample_ASV8

CACCGCGGTTATACGAGAGGCCCAAGTTGACAGACACCGGCGTAAAGAGTGGCTAAGGAA

AAATTTCTACTAAAGCCGAACACCTTCAGAACTGTCATACGTTCCCGAAGATAAGAAGCC

CCACCACGAAAGTGGCTTTATATTACCCGACCCCACGAAAGCTGTGAAA

>835_Sample_ASV9

CACCGCGGTTATACGAGAGGCCCAAGTTGACAGACACCGGCGTAAAGAGTGGCTAAGGAA

AAATTTCTACTAAAGCCGAACACCTTCAGAACTGTCATACGTTCCCGAAGAAAAGAAGCC

CCACCACGAAAGTGGCTTTATATTACCCGACCCCACGAAAGCTGTGAAA

>835_Sample_ASV10

CACCGCGGTTATACGAGAGGCCCAAGTTGATAAGCCCGGCGTAAAGAGTGGTTATGGAGA

ATAAAACACTAAAGCTAAAGACCCCCTAGGCTGTTATACGCACCTGGCGGCTCGAACCAC

CTATACGAAAGTAGCTTTACCCCCCTTCCACCAGAATCCACGACAGCTGGGGCA

>835_Sample_ASV11

CACCGCGGTTATACGAGAGGCCCTAGTTGATAGCCACGGCGCAAAGAGTGGTTAAGGAGC

CCCACTAAATAAAGTCGAACACTTCCTAGGCCGTTATACGTTTTCTAGAAGCACGAAACC

CAATTACACGAAAGCAACTTTATACTAAACAACCTGACCCCACGAAAGCTAAGAAA

>835_Sample_ASV12

CACCGCGGTTATACGAGAGGCCCAAGTTGACAGACATCGGCGTAAAGAGTGGTTAAGGAA

ACCCCATTACTAAAGCCGAACGCCCTCAGAACTGTCATACGTTCCCGAAGGTAAGAAGCC

CCACTACGAAAGTGGCTTTAACCACACCCGACCCCACGAAAGCTGTGAAA

>835_Sample_ASV13

CACCGCGGTTATACGAGAGGCCCAAGTTGACAGACATCGGCGTAAAGAGTGGTTAAGGAA

ACCCCATTACTAAAGCCGAACGCCCTCAGAACTGTCATACGTTCCCGAAGGTAAGAAGCC

CCACTACGAAAGTGGCTTTAACCACACCCGACCCCACGAAAGCTAAGAAA

>835_Sample_ASV14

CACCGCGGTTATACGAGAGGCCCCAGTTGATATTAGCGGCGTAAAGGGTGGTTAAGGACT

TTAATAACTAAAGCTAAACACTTCCCCGGCTGTTGTACGTAGACCGGAAGCTTGAAGCCC

AGACACGAAAGTAGCTTTATTCCCCCTGACCCCACGAAAGCTGAGAAA

>835_Sample_ASV15

CACCGCGGTTATACGAGAAACCCTAGTTGACAAATACGGCGTAAAGAGTGGTTAAGGGGT

ATCAAAAATAAAGTCAAACATTTTCCTAGTTGTTATAAGCATTTCGAGAACATGAAGCCC

AACCACGAAAGTAACTTTACCTTCACCTGACCCCACGAAAGCTAAGAAA

>835_Sample_ASV16

CACCGCGGTTATACGAAAGACCCCAGTTTATAGACACGGCGTAAAGGGTGGTTATAGGAC

AAACTAACATAGAGCCAAAGACCCCCTAAGCCGTCATACGCTCACGAGGGCTCGAAGCCC

CTATACGAAAGTAGCTCTACCAAAAAATGCTCGACTCCACGAAAGCTGGGACA

>835_Sample_ASV17

CACCGCGGTTATACGAGAGACCCTAATTGATAGACACGGCGTAAAGAGTGGTTTGGGAAA

GCCCAAAAATAAAGCTAAAGACCTTCTAAGCCGTCACACGCACGCCGAAGGCACGAAGCC

CTGACACGAAAGTAGCTTTACTATTACCCCCGACGCCACGAAAGCTAAGAAA

>835_Sample_ASV18

CACCGCGGTTATACGAAAGACCCTAGTTGATGGATCACGGCGTAAAGGGTGGTTAAGGGA

TATAAAAATAAAGCTAAAAATCCTCTTAGCTGTCATACGCTTTCCGAAGACATGAGGCCC

AATTACGAAAGTAGCTTTAAACCACCCTGACACCACGAAAGCCAAGAAA

>835_Sample_ASV19

CACCGCGGTCATACGATTAACCCAAGTTAATAGGCACACGGCGTAAAGCGTGTTTAAGCA

CTACGCCAAATAAAGTTAAACTCCAATTAAGCTGTAAAAAGCCATAATCAAGACGAAAAT

AAATAACGAAAGTAACTTTACAACCGCTGAAACACGATAGCTAAGATC

>835_Sample_ASV20

CACCGCGGTCATACGATTAACCCAAGTTAATAGGCACACGGCGTAAAGCGTGTTTAAGCA

CTATGCCAAATAAAGTTAAACTCCAATTAAGCTGTAAAAAGCCATAATCAAGACGAAAAT

AAATAACGAAAGTAACTTTACAACCGCTGAAACACGATAGCTAAGATC

>835_Sample_ASV21

CACCGCGGTTATACGAGAGGCCCTAGTTGATAACTACCGGCGTAAAGAGTGGTTATGGAA

AAATATTTAATAAAGCCGAACACCCCCTCAGCCGTCATACGCACCTGGGAGCACGAAGAC

CTACTGCGAAAGCAGCTTTAATTATGCCTGACCCCACGACAGCTAAGAAA

>835_Sample_ASV22

CACCGCGGTTATACGAGAGGCCCAAGTTGATTATTACCGGCGTAAAGCGTGGTTAAGATT

TTTATAGGACTAAAGCCGAACCCCCTCAGAGCTGTTATACGCACCCGAAGGTTAGAAGTT

CAATCACGAAAGTGGCTTTACAACCCCGAACCCACGAAAGCTAAGATA

>835_Sample_ASV23

CACCGCGGTTATACGAGAGACCCTAATTGATAGGCGCGGCGTAAAGAGTGGTTTAGGACT

AAAACTAATAAAGCTAAAGATCCTCCCAGCCGTCGCACGCATTCTGAGGACACGAAACTC

AACCACGAAAGTAGCTTTAACATACTCTCCTGACCCCACGAAAGCTAAGAAA

>835_Sample_ASV24

CACCGCGGTTATACGAAAGACCCTAGTTGATAGGCACGGCGTAAAGGGTGGTTAGGGATA

TATCAAAAATAAAGTTAAAGAGCCTCTAAGCTGTCGCACGCATCCCGAGAGCTCGAGACC

CAGACACGAAAGTAACTTTAAAATACCTCACCTGACCCCACGAAAGCTAAGAAA

>835_Sample_ASV25

CACCGCGGTTATACGAGAGACCCCAGTTGATAAACACGGCGTAAAGAGTGGTTAAGGGCA

CAAAACAAATAAAGTCAAACAACCTCTTAGCTGTTATACGCATTATGAGGATATGAAGCC

CCCCTACGAAAGTAACTTTACCAACCCCTGACCCCACGAAAGCTAAGAAA

>835_Sample_ASV26

CACCGCGGTTATACGAGAGACCCTAGTTGATATATACGGCGTAAAGAGTGGTTTGGAATA

ACCAAGTAATATAGCCAAAGACCTCCCAAGCCGTCACACGCACCCCGGAGGCACGAAGCC

CAAACACGAAAGTAGCTTTATTAACTTACCGACGCCACGAAAGCTAAGAAA

>835_Sample_ASV27

CACCGCGGTTATACGAAAGACCCTAGTTTATAGGCACGGCGTAAAGGGTGGTTAGGGGAT

AAACAAAATAAAGCCAAAGACCTTCTAAGTCGTCATACACTCATGAAGGCACGAAGCCCA

AATACGAAAGTAACTTTACAACTTAGACCCGACTCCACGAAAGCTGGGGGA

>835_Sample_ASV28

CACCGCGGTTATACGAGAGACCCTAATTAATAGCTACGGCGTAAAGAGTGGTTTAGGGTA

AAAATTAATAAAGCCGAAGTTTCTCCTAGCCGTTGTACGCACTCTGAGAACACGAAGCCC

CACTACGAAAGTAGCTTTACCTATTATTTCCTGACCCCACGAAAGCTAAGAAA

>835_Sample_ASV29

CACCGCGGTTATACGAGAGACCCTAATTAATAGATACGGCGTAAAGAGTGGTTTAGGACA

AAAATTAATAAAGCCGAAGTTTCTCCTAGCCGTTGTACGCACTCTGAGAACACGAAGCCC

CACTACGAAAGTAGCTTTACCTATTATTTCCTGACCCCACGAAAGCTAAGAAA

>835_Sample_ASV30

CACCGCGGTCATACGATTAACCCAAGTTAATAGGCACACGGCGTAAAGCGTGTTAAAGCA

CTATACTAAATAAAGTTAAATTCCAATTAAGCTGTAAAAAGCCATAATTGCAACAAAAAT

AAACAACGAAAGTAACTTTACAGTCGCTGAAACACGATAGCTAGGACC

>835_Sample_ASV31

CACCGCGGTTATACGAGAGACCCTAATTGATAGACTACGGCGTAAAGAGTGGTTTAGAAC

AAAAATTAATAAAGCCAAAGATCCTCCTGGCCGTCGCACGCATTTCGAGGATACGAAGCC

CCACTACGAAAGTAGCTTTACTACAACTTTTCTGACCCCACGAAAGCTAAGAAA

>835_Sample_ASV32

CACCGCGGTTATACGAAAGACCCTAGTTGATAGATCACGGCGTAAAGGGTGGTTAAGGAG

AACAAAAATAAAGCTAAAAATCCTCTAAGCTGTCATACGCTTTCCGAAGACATGAGACCC

AACCACGAAAGTAACTTTAAACTTTCCTGACACCACGAAAGCCAAGAAA

>835_Sample_ASV33

CACCGCGGTTATACGAAAGACCCTAGTTGATAGATCACGGCGTAAAGGGTGGTTAAGGAA

AACAAAAATAAAGCTAAAAATCCTCTAAGCTGTCATACGCTTTCCGAAGACATGAGATCC

AACCACGAAAGTAGCTTTAAAATTTCCTGACACCACGAAAGCCAAGGAA

>838_Sample_ASV1

CACCACGGTTATACGAGAGGCCCAAGTTGAAAGACATCGGCGTAAAGAGTGGTTAAGGAA

AACCTTCTACTAAAGTCGAACGCTCTCAGGACTGTCGTACGTTTCCGAGAGTATGAAGCC

CCACCACGAAAGTGACTTTACCTCACCTGACTCCACGAAAGCTGCGAAA

>838_Sample_ASV2

CACCGCGGTTATACGAGAGGCCCAAGTTGACAGACATCGGCGTAAAGAGTGGTTAAGGAA

AACCTTCAACTAAAGTCGAACGCTCTCAAGACTGTCGTACGTTTCCGAGAGTATGAAGCC

CCACCACGAAAGTGACTTTACCTCACCTGACTCCACGAAAGCTGCGAAA

>838_Sample_ASV3

CACCGCGGTTATACGAAAGACCCTAGTTGATGGGTGCGGCGTAAAGGGTGGTTAGGGACT

ACAAAAAATAGAGCTAAAGATCCTCTAGGTCGTCATACACATCACGAGGAAACGAAGCCC

AAATACGAAAGTAGCTCTAAATATAATCCTGACGCCACGAAAGCCAAGAAA

>838_Sample_ASV4

CACCGCGGTTATACGAAAGACCCTAGTTGATAACTACGGCATAAAGGGTGGTTAGGGAGT

TTAAAAAATAAAGCCAAAGCTCTTCTAAGCTGTCATACGCTCTCTGAAAGAACGAGGCCC

AAACACGAAAGTAGCTTTAAATAAATAACCTGATACCACGAAAGCCAAGAAA

>838_Sample_ASV5

CACCGCGGTTATACGAGAGACCCTAGTTGATTAAAGCGGCGTAAAGAGTGGTTATGGAAC

TATTCTTTTAAAGCAGAAAACCTCTCAAACTGTTATACGCACCCAGAGGTCTGAATCCCT

CACACGAAAGTGACTTTATTTATGCCTACCAGAACCCACGAAAGCTGGGACA

>838_Sample_ASV6

CACCGCGGTTATACGAGAGGCCCAAGTTGACAGACACCGGCGTAAAGAGTGGCTAGGGAA

AAATTACTACTAAAGCCGAACACCTTCAGAACTGTCATACGTTCCCGAAGATAAGAAGCC

CCACCACGAAAGTGGCTTTATATTACCCGACCCCACGAAAGCTGTGAAA

>838_Sample_ASV7

CACCGCGATTATACGAGAGGCCCAAGTTGACAGACATCGGCGTAAAGAGTGGTTAAGGAA

AAATTACTACTAAAGCCGAACACCTTCAGAACTGTCATACGTTCCCGAAGATAAGAAGCC

CCACCACGAAAGTGGCTTTATATTACCCGACCCCACGAAAGCTGTGAAA

>838_Sample_ASV8

CACCGCGGTTATACGAGGGGCCTAAGTTGACAGACACCGGCGTAAAGAGTGGCTAGGGAA

AAATTACTACTAAAGCCGAACACCCTCAGAACTGTCATACGTTCCCGAAGATAAGAAGCC

CCACCACGAAAGTGGCTTTATATTACCCGACCCCACGAAAGCTGTGAAA

>838_Sample_ASV9

CACCGCGGTTATACGAGAGGCCCAAGTTGACAGACACCGGCGTAAAGAGTGGCTAAGGAA

AAATTTCTACTAAAGCCGAACACCTTCAGAACTGTCATACGTTCCCGAAGAAAAGAAGCC

CCACCACGAAAGTGGCTTTATATTACCCGACCCCACGAAAGCTGTGAAA

>838_Sample_ASV10

CACCGCGGTTATACGAGAGGCCCAAGTTGACAGACACCGGCGTAAAGAGTGGCTAAGGAG

AAATTTCTACTAAAGCCGAACACCTTCAGAACTGTCATACGTTCCCGAAGAAAAGAAGCC

CCACCACGAAAGTGGCTTTATATTACCCGACCCCACGAAAGCTGTGAAA

>838_Sample_ASV11

CACCGCGGTTATACGAAAGACCCTAGTTTATAGAAACGGCGTAAAGGGTGGTTAAGGGAC

AAACAAAATAAGGCCAAAGACTTCCTAAGCCGTCATACGCTCCACGAAAGCACGAAGCCC

AAACACGAAAGTAGCCTTAATAACTTCACCTGACCCCACGAAAGCTGGGAGA

>838_Sample_ASV12

CACCGCGGTTATACGAAAGACCCTAGTTTATAGAAACGGCGTAAAGGGTGGTTAAGGGAC

AAACAAAATAAGGCCAAAGACTTCCTAAGCCGTCATACGCTCCACGAAAGCACGAAGCCC

AAACACGAAAGTAGCCTTAATAACTTCACCTGACCCCACGAAAGCTGTGAAA

>838_Sample_ASV13

CACCGCGGTTATACGAGAGGCCCAAGTTGATAAGCCCGGCGTAAAGAGTGGTTATGGAGA

ATAAAACACTAAAGCTAAAGACCCCCTAGGCTGTTATACGCACCTGGCGGCTCGAACCAC

CTATACGAAAGTAGCTTTACCCCCCTTCCACCAGAATCCACGACAGCTGGGGCA

>838_Sample_ASV14

CACCGCGGTTATACGAGAGGCCCAAGTTGATAAGCCCGGCGTAAAGAGTGGTTATGGAGA

ATAAAACACTAAAGCTAAAGACCCCCTAGGCTGTTATACGCACCTGGCGGCTCGAACCAC

CTATACGAAAGTAGCTTTACCCCCCTTCCACCAGAATCCACGAAAGCTAAGAAA

>838_Sample_ASV15

CACCGCGGTTATACGAGAGGCCCAAGTTGATAAGCCCGGCGTAAAGAGTGGTTATGGAGA

ATAAAACACTAAAGCTAAAGACCCCCTAGGCTGTTATACGCACCTGGCGGCTCGAACCAC

CTATACGAAAGTAGCTTTACCCCCCTTCCACCTGACTCCACGAAAGCTAAGAAA

>838_Sample_ASV16

CACCGCGGTTATACGAGAGGCCCAAGTTGACAGACATCGGCGTAAAGAGTGGTTAAGGAA

ACCCCATTACTAAAGCCGAACGCCCTCAGAACTGTCATACGTTCCCGAAGGTAAGAAGCC

CCACTACGAAAGTGGCTTTAACCACACCCGACCCCACGAAAGCTGTGAAA

>838_Sample_ASV17

CACCGCGGTTATACGAGAGGCCCAAGTTGACAGACACCGGCGTAAAGAGTGGTTAAGGAA

ATTCATCAACTAAAGCCGAATGCCCTCAGGACTGTTATACGTTTGCGAAGGTAAGAAGCC

CTACTACGAAAGTGGCTTTATTCCACCTGATTCCACGAAAGCTGCGGAA

>838_Sample_ASV18

CACCGCGGTTATACGAGAGGCCCCAGTTGATATTAGCGGCGTAAAGGGTGGTTAAGGACT

TTAATAACTAAAGCTAAACACTTCCCCGGCTGTTGTACGTAGACCGGAAGCTTGAAGCCC

AGACACGAAAGTAGCTTTATTCCCCCTGACCCCACGAAAGCTGAGAAA

>838_Sample_ASV19

CACCGCGGTCACACGATTAACCCAAGTCAATAGAAGCCGGCGTAAAGAGTGTTTTAGATC

ACCCCCTCCCCAATAAAGCTAAAACTCACCTGAGTTGTAAAAAACTCCAGTTGACACAAA

ATAGACTACGAAAGTGGCTTTAACATATCTGAACACACAATAGCTAAGACC

>838_Sample_ASV20

CACCGCGGTTATACGAGAGACCCCAGTTGATAGCTACGGCGTAAAGAGTGGTTAAGGACC

CCATTTAATAAAGCCAAAGACCCCCCAAGCCGTCATACGCACACGAGGGCGCGAAGCTCG

CACACGAAAGTAGCTCTAATTTACCCGACGCCACGAAAGCTAAGGAA

>838_Sample_ASV21

CACCGCGGTTATACGAGAAACCCTAGTTGACAAATACGGCGTAAAGAGTGGTTAAGGGGT

ATCAAAAATAAAGTCAAACATTTTCCTAGTTGTTATAAGCATTTCGAGAACATGAAGCCC

AACCACGAAAGTAACTTTACCTTCACCTGACCCCACGAAAGCTAAGAAA

>838_Sample_ASV22

CACCGCGGTTATACGAGAGGCTCAAGTTGATAAACCCCGGCGTAAAGAGTGGTTAAGGAA

AAATAGAAACTAAAGCCGAATGCTTTCAAGACTGTTATACGTTCCCGAGAGTAAGAAGAC

CAATTACGAAAGTGGCTTTACTTTACCTGACCCCACGAAAGCTACGACA

>838_Sample_ASV23

AACCGCGGTTATACGAGAGACCCTAATTGATAGCTCACGGCGTAAAGGGTGGTTTGGGGA

AACCTCAAAATAAAGCTAAAGACCTTCTAAGCCGTTACACGCATACCGAAGGCACGAGGC

CCTAACACGAAAGTAGCTTTACTATTACCCCTGACGCCACGAAAGCTAAGAAA

>838_Sample_ASV24

CACCGCGGTTATACGAGAGACCCTAATTGATAGGTACGGCGTAAAGAGTGGTTTGGGATA

ACCCAAAAATAAAGCTAAAGACCTTCTAAGCCGTCACACGCATACCGAAGGCACGAGGCC

CCAACACGAAAGTAGCTTTACTATTACCCCGACGCCACGAAAGCTAAGAAA

>838_Sample_ASV25

CACCGCGGTTATACGAGAGACCCTAATTGATAGGCACGGCGTAAAGAGTGGTTTAGGGTA

ACCCATAAATAAAGCTAAAGACCTTCTAAGCCGTCACACGCACACCGAAGGCACGAGACC

CCAACACGAAAGTAGCTTTACTTCTACCCCTGACGCCACGAAAGCTAAGAAA

>838_Sample_ASV26

CACCGCGGTTATACGAAAGACCCTAGTTGATGGATCACGGCGTAAAGGGTGGTTAAGGGA

TATAAAAATAAAGCTAAAAATCCTCTTAGCTGTCATACGCTTTCCGAAGACATGAGGCCC

AATTACGAAAGTAGCTTTAAACCACCCTGACACCACGAAAGCCAAGAAA

>838_Sample_ASV27

CACCGCGGTTATACGAAAGACCCCAGTTTATAGATGCGGCGTAAAGGGTGGTTAAGAGAA

AATCAAAATAAGGCCAAAGATCCCCTAAGCAGTCATACGCTCCACGAGGACACGAAACCC

AAATACGAAAGTTGCCTTAATAAACTTCACCTGATCCCACGAAAGCTGAAAAA

>838_Sample_ASV28

CACCGCGGTTATACGAGAGGCCCTAGTTGATAACTACCGGCGTAAAGAGTGGTTATGGAA

AAATATTTAATAAAGCCGAACACCCCCTCAGCCGTCATACGCACCTGGGAGCACGAAGAC

CTACTGCGAAAGCAGCTTTAATTATGCCTGACCCCACGACAGCTAAGAAA

>838_Sample_ASV29

CACCGCGGTTATACGAAAGACCCTAGTTTATAGACACGGCGTAAAGGGTGGTTCGGGACT

AAACAAAATAAAGCCAAAGACCTCCTAAGCCGTCATACGCTCACGGAAGCACGAAGCCCA

AGTACGAAAGTAGCTTTACAAAACATGCCTGACTCCACGAAAGCTAGGAAA

>838_Sample_ASV30

CACCGCGGTTATACGAGAGGCCCAAGTTGATACTCCACGGCGTAAAGAGTGGTTAGAAAA

GACCTACTACTAAAGCCGAACGCCTTCAAAGCTGTTATACGCATCCGAAGGTGAGAAGCC

CACCCACGAAAGTGGCTTTACAACCTTGACTCCACGAAAGCTATGACA

>838_Sample_ASV31

CACCGCGGTTATACGAGAGACCCCAGTTGATAAACACGGCGTAAAGAGTGGTTAAGGGCA

CAAAACAAATAAAGTCAAACAACCTCTTAGCTGTTATACGCATTATGAGGATATGAAGCC

CCCCTACGAAAGTAACTTTACCAACCCCTGACCCCACGAAAGCTAAGAAA

>838_Sample_ASV32

CACCGCGGTTATACGAAAGACCCTAGTTTATAGGCACGGCGTAAAGGGTGGTTAGGGGAT

AAACAAAATAAAGCCAAAGACCTTCTAAGTCGTCATACACTCATGAAGGCACGAAGCCCA

AATACGAAAGTAACTTTACAACTTAGACCCGACTCCACGAAAGCTGGGGGA

>838_Sample_ASV33

CACCGCGGTTATACGAGAGACTCCAGTTGATAGCTACGGCGTAAAGAGTGGTTTGGGGCC

CGCCCAAAATAAAGCCAAAGACCTCCCAAGCCGTCAAACGCACCCCGGAGGCACGAAGTC

CTAACGCGAAAGCAACTTTACCTTCCCCGACGCCACGAAAGCTAAGAAA

>838_Sample_ASV34

CACCGCGGTTATACGAAAGACCCTAGTTGATAGATCACGGCGTAAAGGGTGGTTAAGGAG

AACAAAAATAAAGCTAAAAATCCTCTAAGCTGTCATACGCTTTCCGAAGACATGAGACCC

AACCACGAAAGTAACTTTAAACTTTCCTGACACCACGAAAGCCAAGAAA

>842_Sample_ASV1

CACCACGGTTATACGAGAGGCCCAAGTTGAAAGACATCGGCGTAAAGAGTGGTTAAGGAA

AACCTTCTACTAAAGTCGAACGCTCTCAGGACTGTCGTACGTTTCCGAGAGTATGAAGCC

CCACCACGAAAGTGACTTTACCTCACCTGACTCCACGAAAGCTGCGAAA

>842_Sample_ASV2

CACCGCGGTTATACGAGAGGCCCAAGTTGACAGACATCGGCGTAAAGAGTGGTTAAGGAA

AACCTTCAACTAAAGTCGAACGCTCTCAAGACTGTCGTACGTTTCCGAGAGTATGAAGCC

CCACCACGAAAGTGACTTTACCTCACCTGACTCCACGAAAGCTGCGAAA

>842_Sample_ASV3

CACCGCGGTTATACGAGAGGCCCAAGTTGACAGACATCGGCGTAAAGAGTGGTTAAGGAA

AACCTTCAACTAAAGTCGAACGCTCTCAAGACTGTCGTACGTTTCCGAGAGTATGAAGCC

CCACCACGAAAGTGACTTTACCTCACCTGACTCCACGAAAGCTAAGAAA

>842_Sample_ASV4

CACCGCGGTTATACGAAAGACCCTAGTTGATGGGTGCGGCGTAAAGGGTGGTTAGGGACT

ACAAAAAATAGAGCTAAAGATCCTCTAGGTCGTCATACACATCACGAGGAAACGAAGCCC

AAATACGAAAGTAGCTCTAAATATAATCCTGACGCCACGAAAGCCAAGAAA

>842_Sample_ASV5

CACCGCGGTTATACGAGAGACCCTAGTTGATTAAAGCGGCGTAAAGAGTGGTTATGGAAC

TATTCTTTTAAAGCAGAAAACCTCTCAAACTGTTATACGCACCCAGAGGTCTGAATCCCT

CACACGAAAGTGACTTTATTTATGCCTACCAGAACCCACGAAAGCTGGGACA

>842_Sample_ASV6

CACCGCGGTTATACGAAAGACCCTAGTTGATAGCCACGGCGTAAAGGGTGGTTAAGGAAC

CTAAAAAATAAAGCTAAAGACCCTCTAGGCCGTCATACGCATTACGAGGCAAACGAAACC

CCAACACGAAAGTAGCTTTAGACAAATATCCTGATGCCACGAAAGCCAAGAAA

>842_Sample_ASV7

CACCGCGGTTATACGAGAGGCCCAAGTTGACAGACACCGGCGTAAAGAGTGGCTAGGGAA

AAATTACTACTAAAGCCGAACACCTTCAGAACTGTCATACGTTCCCGAAGATAAGAAGCC

CCACCACGAAAGTGACTTTATATTACCCGACCCCACGAAAGCTAAGAAA

>842_Sample_ASV8

CACCGCGGTTATACGAGAGGCCCAAGTTGACAGACACCGGCGTAAAGAGTGGCTAGGGAA

AAATTACTACTAAAGCCGAACACCTTCAGAACTGTCATACGTTCCCGAAGATAAGAAGCC

CCACCACGAAAGTGACTTTATATTACCCGACCCCACGAAAGCTGTGAAA

>842_Sample_ASV9

CACCGCGGTTATACGAGAGGCCCAAGTTGACAGACACCGGCGTAAAGAGTGGCTAGGGAA

AAATTACTACTAAAGCCGAACACCCTCAGAACTGTCATACGTTCCCGAAGGTAAGAAGCC

CCACTACGAAAGTGGCTTTATAATACCCGACCCCACGAAAGCTGTGAAA

>842_Sample_ASV10

CACCGCGGTTATACGAGAGGCCCAAGTTGACAGACACCGGCGTAAAGAGTGGCTAGGGAA

AAATTACTACTAAAGCCGAACACCCTCAGAACTGTCATACGTTCCCGAAGGTAAGAAGCC

CCACCACGAAAGTGGCTTTATAATACCCGACCCCACGAAAGCTGTGAAA

>842_Sample_ASV11

CACCGCGGTTATACGAGAGGCCCAAGTTGACAGACACCGGCGTAAAGAGTGGCTAGGGAA

AAATTACTACTAAAGCCGAACACCTTCAGAACTGTCATACGTTCCCGAAGATAAGAAGCC

CCACCACGAAAGTGGCTTTATATTACCCGACCCCACGAAAGCTGTGAAA

>842_Sample_ASV12

CACCGCGGTTATACGAGAGGCCCAAGTTGACAGACACCGGCGTAAAGAGTGGCTAAGGAA

AAATTTCTACTAAAGCCGAACACCTTCAGAACTGTCATACGTTCCCGAAGATAAGAAGCC

CCACCACGAAAGTGGCTTTATATTACCCGACACCACGAAAGCTGTGAAA

>842_Sample_ASV13

CACCGCGGTTATACGAGAGGCCCAAGTTGACAGACACCGGCGTAAAGAGTGGCTAAGGAA

AAATTTCTACTAAAGCCGAACACCTTCAGAACTGTCATACGTTCCCGAAGAAAAGAAGCC

CCACCACGAAAGTGGCTTTATATTACCCGACCCCACGAAAGCTGTGAAA

>842_Sample_ASV14

CACCGCGGTTATACGAGAGGCCCAAGTTGATAAGCCCGGCGTAAAGAGTGGTTATGGAGA

ATAAAACACTAAAGCTAAAGACCCCCTAGGCTGTTATACGCACCTGGCGGCTCGAACCAC

CTATACGAAAGTAGCTTTACCCCCCTTCCACCAGAATCCACGACAGCTGGGGCA

>842_Sample_ASV15

CACCGCGGTTATACGAGAGGCCCAAGTTGATAAGCCCGGCGTAAAGAGTGGTTATGGAGA

ATAAAACACTAAAGCTAAAGACCCCCTAGGCTGTTATACGCACCTGGCGGCTCGAACCAC

CTATACGAAAGTAGCTTTACCCCCCTTCCACCAGAATCCACGAAAGCTAAGAAA

>842_Sample_ASV16

CACCGCGGTTATACGAGAGACCCTAGTTGATAAGCCCGGCGTAAAGAGTGGTTATGGAGA

ATAAAACACTAAAGCTAAAGACCCCCTAGGCTGTTATACGCACCTGGCGGCTCGAACCAC

CTATACGAAAGTAGCTTTACCCCCCTTCCACCAGAATCCACGACAGCTGGGGCA

>842_Sample_ASV17

CACCGCGGTTATACGAGAGGCCCAAGTTGACAGACATCGGCGTAAAGAGTGGTTAAGGAA

ACCCCATTACTAAAGCCGAACGCCCTCAGAACTGTCATACGTTCCCGAAGGTAAGAAGCC

CCACTACGAAAGTGGCTTTAACCACACCCGACCCCACGAAAGCTAAGAAA

>842_Sample_ASV18

CACCGCGGTTATACGAGAGGCCCAAGTTGACAGACATCGGCGTAAAGAGTGGTTAAGGAA

ACCCCATTACTAAAGCCGAACGCCCTCAGAACTGTCATACGTTCCCGAAGGTAAGAAGCC

CCACTACGAAAGTGGCTTTAACCACACCCGACCCCACGAAAGCTGTGAAA

>842_Sample_ASV19

CACCGCGGTCACACGATTAACCCAAGTCAATAGAAGCCGGCGTAAAGAGTGTTTTAGATC

ACCCCCTCCCCAATAAAGCTAAAACTCACCTGAGTTGTAAAAAACTCCAGTTGACACAAA

ATAGACTACGAAAGTGGCTTTAACATATCTGAACACACAATAGCTAAGACC

>842_Sample_ASV20

CACCGCGGTTATACGAGAGACCCCAGTTGATAGCTACGGCGTAAAGAGTGGTTAAGGACC

CCATTTAATAAAGCCAAAGACCCCCCAAGCCGTCATACGCACACGAGGGCGCGAAGCTCG

CACACGAAAGTAGCTCTAATTTACCCGACGCCACGAAAGCTAAGGAA

>842_Sample_ASV21

CACCGCGGTTATACGAGAGACCCTAGTTGATAAATACGGCGTAAAGAGTGGTTATGGGAA

AAACAAATAAAGTCAAACAACCTCTTAGCTGTTATACGCATTATGAGAGCATGAAGCCCC

CTCACGAAAGTAACTTTAATACCTCCTGACCCCACGAAAGCTAAGAAA

>842_Sample_ASV22

CACCGCGGTTATACGAGAGACCCTAATTGATAGACACGGCGTAAAGAGTGGTTTGGGAAA

GCCCAAAAATAAAGCTAAAGACCTTCTAAGCCGTCACACGCACGCCGAAGGCACGAAGCC

CTGACACGAAAGTAGCTTTACTATTACCCCCGACGCCACGAAAGCTAAGAAA

>842_Sample_ASV23

CACCGCGGTTATACGAAAGACCCTAGTTTATAGATGCGGCGTAAAGGGTGGTTAAGGAAA

ACCTAAAATAATGCTAAAGACTCTCAAAGCTGTTATACGCTTCATGAGCACACGAAATCC

AAATACGAAAGTTGCCTTAATAAACTTTGCCTGACCCCACGAAAGCTGAAAAA

>842_Sample_ASV24

CACCGCGGTTATACGAAAGACCCTAGTTTATAGATGCGGCGTAAAGGGTGGTTAAGGAAA

ACCTAAAATAATGCTAAAGACTCTCAAAGCTGTTATACGCTTCATGAGCACACGAAATCC

AAATACGAAAGTTGCCTTAATAAACTTTGCCTGACCCCACGAAAGCTAAGAAA

>842_Sample_ASV25

CACCGCGGTTATACGAAAGACCCCAGTTTATAGATGCGGCGTAAAGGGTGGTTAAGAGAA

AATCAAAATAAGGCCAAAGATCCCCTAAGCAGTCATACGCTCCACGAGGACACGAAACCC

AAATACGAAAGTTGCCTTAATAAACTTCACCTGATCCCACGAAAGCTGAAAAA

>842_Sample_ASV26

CACCGCGGTTATACGAGAGACCCTAGTTGATAGCTACGGCGTAAAGCGTGGTTAGGAGAA

CAAGATAAATAAAGTCAAACAATCTCTCGGCCGTTATACGTTATCTGAGAATATGAAGTC

CTACCACGAAAGTAACTTTAATTTTTCTGACCCCACGAAAACTGAGGAA

>842_Sample_ASV27

CACCGCGGTTAAACGAGAGGCCCTAGTTGATAGTACAACGGCGTAAAGGGTGGTTAAGGA

TAGTGAAATAATAAAGTCGAATGGCCCTTTGGCTGTCATACGCTTCTAGGAGTCCGAAGC

CCAATATACGAAAGTAACTTTAAGAGCGCCCACCTGACCCCACGAAAACTGAGAAA

>842_Sample_ASV28

CACCGCGGTTATACGAAAGACCCTAGTTTATAGACACGGCGTAAAGGGTGGTTCGGGACT

AAACAAAATAAAGCCAAAGACCTCCTAAGCCGTCATACGCTCACGGAAGCACGAAGCCCA

AGTACGAAAGTAGCTTTACAAAACATGCCTGACTCCACGAAAGCTAGGAAA

>842_Sample_ASV29

CACCGCGGTTATACGAAAGACCCTAGTTGATAGGCACGGCGTAAAGGGTGGTTAGGGATA

TATTAAAAATAAAGTTAAAGAGCCTCTAAGCTGTCGCACGCATCCCGAGAGCTCGAGACC

CAGACACGAAAGTAACTTTAAAATACCTCACCTGACCCCACGAAAGCTAAGAAA

>842_Sample_ASV30

CACCGCGGTTATACGAGAGACCCCAGTTGATAAACACGGCGTAAAGAGTGGTTAAGGGCA

CAAAACAAATAAAGTCAAACAACCTCTTAGCTGTTATACGCATTATGAGGATATGAAGCC

CCCCTACGAAAGTAACTTTACCAACCCCTGACCCCACGAAAGCTAAGAAA

>842_Sample_ASV31

CACCGCGGTTATACGAGAGACCCTAGTTGATATATACGGCGTAAAGAGTGGTTTGGAATA

ACCAAGTAATATAGCCAAAGACCTCCCAAGCCGTCACACGCACCCCGGAGGCACGAAGCC

CAAACACGAAAGTAGCTTTATTAACTTACCGACGCCACGAAAGCTAAGAAA

>842_Sample_ASV32

CACCGCGGTTATACGAAAGACCCTAGTTTATAGGCACGGCGTAAAGGGTGGTTAGGGGAT

AAACAAAATAAAGCCAAAGACCTTCTAAGTCGTCATACACTCATGAAGGCACGAAGCCCA

AATACGAAAGTAACTTTACAACTTAGACCCGACTCCACGAAAGCTGGGGGA

>842_Sample_ASV33

CACCGCGGTTATACGAGAGACCCTAATTAATAGATACGGCGTAAAGAGTGGTTTAGGACA

AAAATTAATAAAGCCGAAGTTTCTCCTAGCCGTTGTACGCACTCTGAGAACACGAAGCCC

CACTACGAAAGTAGCTTTACCTATTATTTCCTGACCCCACGAAAGCTAAGAAA

>842_Sample_ASV34

CACCGCGGTTATACGAGAGACCCTAGTTGATATATACGGCGTAAAGAGTGGTTAAGGAAG

TCTCAAAACTAAAGCCGAATGCCTTCAAGACTGTTATACGTTTCCGAAGGTAAGAAGCCC

CACTACGAAAGTGGCTTTACTCCACCTGACTCCACGAAAGCTGTGAAA

>842_Sample_ASV35

CACCGCGGTTATACGAGAGGCCCAAGTTGATAGACATCGGCGTAAAGAGTGGTTAAGGAA

GTCTCAAAACTAAAGCCGAATGCCTTCAAGACTGTTATACGTTTCCGAAGGTAAGAAGCC

CCACTACGAAAGTGGCTTTACTCCACCTGACTCCACGAAAGCTGTGAAA

>842_Sample_ASV36

CACCGCGGTTATACGAGAGGCCCAAGTTGATAGACATCGGCGTAAAGAGTGGTTAAGGAA

GTCTCAAAACTAAAGCCGAATGCCTTCAAGACTGTTATACGTTTCCGAAGGTAAGAAGCC

CCACTACGAAAGTGGCTTTACTCCACCTGACTCCACGAAAGCTAAGAAA

>842_Sample_ASV37

CACCGCGGTTATACGAGAGACCCTAATTGATAGACTACGGCGTAAAGAGTGGTTTAGAAC

AAAAATTAATAAAGCCAAAGATCCTCCTGGCCGTCGCACGCATTTCGAGGATACGAAGCC

CCACTACGAAAGTAGCTTTACTACAACTTTTCTGACCCCACGAAAGCTAAGAAA

>842_Sample_ASV38

CACCGCGGTTATACGAAAGACCCTAGTTGATAGATCACGGCGTAAAGGGTGGTTAAGGAG

AACAAAAATAAAGCTAAAAATCCTCTAAGCTGTCATACGCTTTCCGAAGACATGAGACCC

AACCACGAAAGTAACTTTAAACTTTCCTGACACCACGAAAGCCAAGAAA

>842_Sample_ASV39

CACCGCGGTTATACGAAAGACCCTAGTTGATAGATCACGGCGTAAAGGGTGGTTAAGGAG

AACAAAAATAAAGCTAAAAATCTTCTAAGCTGTCATACGCTTTCCGAAGACATGAGACCC

AACCACGAAAGTAGCTTTAAACTTTCCTGACACCACGAAAGCCAAGAAA

>844_Sample_ASV1

CACCGCGGTTATACGAAAGACCCTAGTTGATGGGTGCGGCGTAAAGGGTGGTTAGGGACT

ACAAAAAATAGAGCTAAAGATCCTCTAGGTCGTCATACACATCACGAGGAAACGAAGCCC

AAATACGAAAGTAGCTCTAAATATAATCCTGACGCCACGAAAGCCAAGAAA

>844_Sample_ASV2

CACCGCGGTTATACGAGAGACCCTAGTTGATTAAAGCGGCGTAAAGAGTGGTTATGGAAC

TATTCTTTTAAAGCAGAAAACCTCTCAAACTGTTATACGCACCCAGAGGTCTGAATCCCT

CACACGAAAGTGACTTTATTTATGCCTACCAGAACCCACGAAAGCTGGGACA

>844_Sample_ASV3

CACCGCGGTTATACGAGAGGCCCAAGTTGACAGACACCGGCGTAAAGAGTGGCTAGGGAA

AAATTACTACTAAAGCCGAACACCTTCAGAACTGTCATACGTTCCCGAAGATAAGAAGCC

CCACCACGAAAGTGACTTTATATTACCCGACCCCACGAAAGCTGGGGGA

>844_Sample_ASV4

CACCGCGGTTATACGAGAGGCCCAAGTTGACAGACACCGGCGTAAAGAGTGGCTAGGGAA

AAATTACTACTAAAGCCGAACACCTTCAGAACTGTCATACGTTCCCGAAGATAAGAAGCC

CCACCACGAAAGTGACTTTATATTACCCGACCCCACGAAAGCTGTGAAA

>844_Sample_ASV5

CACCGCGGTTATACGAGAGGCCCAAGTTGACAGACACCGGCGTAAAGAGTGGCTAGGGAA

AAATTACTACTAAAGCCGAACACCTTCAGAACTGTCATACGTTCCCGAAGATAAGAAGCC

CCACCACGAAAGTGGCTTTATATTACCCGACCCCACGAAAGCTGTGAAA

>844_Sample_ASV6

CACCGCGGTTATACGAGAGGCCCAAGTTGACAGACACCGGCGTAAAGAGTGGCTAGGGAA

AAATTACTACTAAAGCCGAACACCCTCAGAACTGTCATACGTTCCCGAAGGTAAGAAGCC

CCACCACGAAAGTGGCTTTATAATACCCGACCCCACGAAAGCTGTGAAA

>844_Sample_ASV7

CACCGCGGTTATACGAGAGGCCCAAGTTGACAGACACCGGCGTAAAGAGTGGCTAAGGAA

AAATTTCTACTAAAGCCGAACACCTTCAGAACTGTCATACGTTCCCGAAGATAAGAAGCC

CCACCACGAAAGTGGCTTTATATTACCCGACACCACGAAAGCTGTGAAA

>844_Sample_ASV8

CACCGCGGTTATACGAGAGGCCCAAGTTGACAGACACCGGCGTAAAGAGTGGCTAAGGAA

AAATTTCTACTAAAGCCGAACACCTTCAGAACTGTCATACGTTCCCGAAGAAAAGAAGCC

CCACCACGAAAGTGGCTTTATATTACCAGACCCCACGAAAGCTGTGAAA

>844_Sample_ASV9

CACCGCGGTTATACGAAAGACCCTAGTTTATAGAAACGGCGTAAAGGGTGGTTAAGGGAC

AAACAAAATAAGGCCAAAGACTTCCTAAGCCGTCATACGCTCCACGAAAGCACGAAGCCC

AAACACGAAAGTAGCCTTAATAACTTCACCTGACCCCACGAAAGCTGGGAGA

>844_Sample_ASV10

CACCGCGGTTAAACGAGAGACCCTAGTTGATCATCACGGCGTAAAGAGTGGTTAGGGGAT

TATCATAAATAAAGCCAAAGACCTCCCAAGCTGTCGCACGCATTCCGGAGGCGCGAAGCC

CACACACGAAAGTAGCTTTAACTATTGACCCTGATGCCACGAAAGCTAAGAAA

>844_Sample_ASV11

CGCCGCGGTTAAACGAGAGGCCCTAGTTGATGGAACAACGGCGTAAAGGGTGGTTAAGGA

TAGTGAGACAATAAAGTCGAATGGCCCTTTGGCTGTCATACGCTTCTGGGAGCCTGAAGC

CCACTATACGAAAGTAGCTTTAACAACGTCCACCTGACCCCACGAAAACTGAGAAA

>844_Sample_ASV12

CACCGCGGTTATACGAAAGACCCTAGTTGATAGATCACGGCGTAAAGGGTGGTTAGGGAA

TACAAAAATAAAGCTAAAAATCCTCTAAGCTGTCATACGCTTCCCGAAGACATGAAACCC

AACCACGAAAGTAGCTTTAAACTCTCCTGACACCACGAAAGCCAAGGAA

>844_Sample_ASV13

CACCGCGGTTATACGAGAGGCCCAAGTTGATAAGCCCGGCGTAAAGAGTGGTTATGGAGA

ATAAAACACTAAAGCTAAAGACCCCCTAGGCTGTTATACGCACCTGGCGGCTCGAACCAC

CTATACGAAAGTAGCTTTACCCCCCTTCCACCAGAATCCACGACAGCTGGGGCA

>844_Sample_ASV14

CACCGCGGTTATACGAGAGGCCCAAGTTGACAGACATCGGCGTAAAGAGTGGTTAAGGAA

ACCCCATTACTAAAGCCGAACGCCCTCAGAACTGTCATACGTTCCCGAAGGTAAGAAGCC

CCACTACGAAAGTGGCTTTAACCACACCCGACCCCACGAAAGCTAAGAAA

>844_Sample_ASV15

CACCGCGGTTATACGAGAGGCCCAAGTTGACAGACATCGGCGTAAAGAGTGGTTAAGGAA

ACCCCATTACTAAAGCCGAACGCCCTCAGAACTGTCATACGTTCCCGAAGGTAAGAAGCC

CCACTACGAAAGTGGCTTTAACCACACCCGACCCCACGAAAGCTGTGAAA

>844_Sample_ASV16

CACCGCGGTTAAACGAGAGGCCCTAGTTGATATTACAACGGCGTAAAGGGTGGTTAAGGA

AAGCAAGCAATAAAGCCGAATGGCCCTTTGGCTGTCATACGCTTCTAGGTGTCCGAAGCC

CAATATACGAAAGTAGCTTTAGAAAAGCCCACCTGACACCACGAAAGCTGAGGAA

>844_Sample_ASV17

CACCGCGGTTATACGAGAGGCCCCAGTTGATATTAGCGGCGTAAAGGGTGGTTAAGGACT

TTAATAACTAAAGCTAAACACTTCCCCGGCTGTTGTACGTAGACCGGAAGCTTGAAGCCC

AGACACGAAAGTAGCTTTATTCCCCCTGACCCCACGAAAGCTGAGAAA

>844_Sample_ASV18

CACCGCGGTCACACGATTAACCCAAGTCAATAGAAGCCGGCGTAAAGAGTGTTTTAGATC

ACCCCCTCCCCAATAAAGCTAAAACTCACCTGAGTTGTAAAAAACTCCAGTTGACACAAA

ATAGACTACGAAAGTGGCTTTAACATATCTGAACACACAATAGCTAAGACC

>844_Sample_ASV19

CACCGCGGTTATACGAGAGGCCCTAGTTGATAAATACGGCGTAAAGAGTGGTTAGAGGAC

TCCCATAATAAAGCCAAAGACCCTCCGGGCCGTCACACGCATACGAGGGCACGAAGTTCA

CACACGAAAGTAGCTTTAAATTACCCGACGCCACGAAAGCTAAGAAA

>844_Sample_ASV20

CACCGCGGTTATACGAAAGACCCTAGTTTATAAGTACGGCGTAAAGGGTGGTTAGGGGAC

AAACAAAATAAAGCCAAAGACCTCCTAAGCCGTCATACGCTTACGGAGGCACGAAGCCCA

AACACGAAAGTAGCTTTACCGAACATGCCCGACTCCACGAAAGCTGGGAAA

>844_Sample_ASV21

CACCGCGGTTATACGAGAGACCCTAGTTGATAAATACGGCGTAAAGAGTGGTTATGGGAA

AAACAAATAAAGTCAAACAACCTCTTAGCTGTTATACGCATTATGAGAGCATGAAGCCCC

CTCACGAAAGTAACTTTAATACCTCCTGACCCCACGAAAGCTAAGAAA

>844_Sample_ASV22

AACCGCGGTTATACGAGAGACCCTAATTGATAGCTCACGGCGTAAAGGGTGGTTTGGGGA

AACCTCAAAATAAAGCTAAAGACCTTCTAAGCCGTTACACGCATACCGAAGGCACGAGGC

CCTAACACGAAAGTAGCTTTACTATTACCCCTGACGCCACGAAAGCTAAGAAA

>844_Sample_ASV23

CACCGCGGTTATACGAAAGACCCTAGTTTATAGGCACGGCGTAAAGGGTGGTTAAGAGAA

AATCAAAATAAGGCCAAAGATCCCCTAAGCAGTCATACGCTCCACGAGGACACGAAACCC

AAATACGAAAGTTGCCTTAATAAACTTCACCTGATCCCACGAAAGCTGAAAAA

>844_Sample_ASV24

CACCGCGGTTATACGAAAGACCCCAGTTTATAGATGCGGCGTAAAGGGTGGTTAAGAGAA

AATCAAAATAAGGCCAAAGATCCCCTAAGCAGTCATACGCTCCACGAGGACACGAAACCC

AAATACGAAAGTTGCCTTAATAAACTTCACCTGATCCCACGAAAGCTGAAAAA

>844_Sample_ASV25

CACCGCGGTTATACGAAAGACCCTAGTTTATAGATGCGGCGTAAAGGGTGGTTAAGAGAA

AATCAAAATAAGGCCAAAGATCCCCTAAGCAGTCATACGCTCCACGAGGACACGAAACCC

AAATACGAAAGTTGCCTTAATAAACTTCACCTGATCCCACGAAAGCTGAAAAA

>844_Sample_ASV26

CACCGCGGTTATACGAAAGACCCCAGTTTATAGATGCGGCGTAAAGGGTGGTTAAGAGAA

AATCAAAATAAGGCCAAAGATCCCCTAAGCAGTCATACGCTCCACGAGGACACGAAACCC

AAATACGAAAGTTGCCTTAATAAACTTCACCTGATCCCACGAAAGCTGGGGGA

>844_Sample_ASV27

CACCGCGGTTAAACGAGAGGCCCTAGTTGATAGTACAACGGCGTAAAGGGTGGTTAAGGA

TAGTGAAATAATAAAGTCGAATGGCCCTTTGGCTGTCATACGCTTCTAGGAGTCCGAAGC

CCAATATACGAAAGTAACTTTAAGAGCGCCCACCTGACCCCACGAAAACTGAGAAA

>844_Sample_ASV28

CACCGCGGTTATACGAAAGACCCTAGTTTATAGACACGGCGTAAAGGGTGGTTCGGGACT

AAACAAAATAAAGCCAAAGACCTCCTAAGCCGTCATACGCTCACGGAAGCACGAAGCCCA

AGTACGAAAGTAGCTTTACAAAACATGCCTGACTCCACGAAAGCTAGGAAA

>844_Sample_ASV29

CACCGCGGTTATACGAAAGACCCTAGTTGATAGGCACGGCGTAAAGGGTGGTTAGGGATA

TATCAAAAATAAAGTTAAAGAGCCTCTAAGCTGTCGCACGCATCCCGAGAGCTCGAGACC

CAGACACGAAAGTAACTTTAAAATACCTCACCTGACCCCACGAAAGCTAAGAAA

>844_Sample_ASV30

CACCGCGGTTATACGAAAGACCCTAGTTGATAGGCACGGCGTAAAGGGTGGTTAGGGATA

CACCAAAAATAAAGTTAAAGAGCCTCTAAGCTGTCGCACGCATCCCGAGAGCTCGAGACC

CAGACACGAAAGTAACTTTAAAATACCTCACCTGACCCCACGAAAGCTAAGAAA

>844_Sample_ASV31

CACCGCGGTTATACGAAAGACCCTAGTTGATAGCCACGGCGTAAAGGGTGGTTAAGGTAT

ACAACAAATAAAGCTAAATAGCCTCTAGGCCGTCGCACGCATTCCGAGAGCTCGAAACCC

AAAAACGAAAGTAGCTTTAAAATAAACAGACCTGACCCCACGAAAGCTAAGAAA

>844_Sample_ASV32

CACCGCGGTTATACGAGAGGCCCAAGTTGATACTCCACGGCGTAAAGAGTGGTTAGAAAA

GACCTACTACTAAAGCCGAACGCCTTCAAAGCTGTTATACGCATCCGAAGGTGAGAAGCC

CACCCACGAAAGTGGCTTTACAACCTTGACTCCACGAAAGCTATGACA

>844_Sample_ASV33

CACCGCGGTTATACGAGAGACCCCAGTTGATAAACACGGCGTAAAGAGTGGTTAAGGGCA

CAAAACAAATAAAGTCAAACAACCTCTTAGCTGTTATACGCATTATGAGGATATGAAGCC

CCCCTACGAAAGTAACTTTACCAACCCCTGACCCCACGAAAGCTAAGAAA

>844_Sample_ASV34

CACCGCGGTTATACGAGAGACCCTAGTTGATATATACGGCGTAAAGAGTGGTTTGGAATA

ACCAAGTAATATAGCCAAAGACCTCCCAAGCCGTCACACGCACCCCGGAGGCACGAAGCC

CAAACACGAAAGTAGCTTTATTAACTTACCGACGCCACGAAAGCTAAGAAA

>844_Sample_ASV35

CACCGCGGTTATACGAAAGACCCTAGTTTATAGGCACGGCGTAAAGGGTGGTTAGGGGAT

AAACAAAATAAAGCCAAAGACCTTCTAAGTCGTCATACACTCATGAAGGCACGAAGCCCA

AATACGAAAGTAACTTTACAACTTAGACCCGACTCCACGAAAGCTGGGGGA

>844_Sample_ASV36

CACCGCGGTTATACGAGAGGCCCAAGTTGATAGACATCGGCGTAAAGAGTGGTTAAGGAA

GTCTCAAAACTAAAGCCGAATGCCTTCAAGACTGTTATACGTTTCCGAAGGTAAGAAGCC

CCACTACGAAAGTGGCTTTACTCCACCTGACTCCACGAAAGCTGTGAAA

>844_Sample_ASV37

CACCGCGGTTATACGAAAGACCCTAGTTGATAGACCACGGCGTAAAGGGTGGTTAAGGAA

CACAAAAATAAAGCTAAAGATCCTCTAAGCTGTCATACGCTTCCCGAAGACATGAAACCC

AATCACGAAAGTAGCTTTAAACTGTCCTGACACCACGAAAGCCAAGAAA

>847_Sample_ASV1

CACCGCGGTTATACGAGAGGCCCAAGTTGACAGACATCGGCGTAAAGAGTGGTTAAGGAA

AACCTTCAACTAAAGTCGAACGCTCTCAAGACTGTCGTACGTTTCCGAGAGTATGAAGCC

CCACCACGAAAGTGACTTTACCTCACCTGACTCCACGAAAGCTGCGAAA

>847_Sample_ASV2

CACCGCGGTTATACGAAAGACCCTAGTTGATGGGTGCGGCGTAAAGGGTGGTTAGGGACT

ACAAAAAATAGAGCTAAAGATCCTCTAGGTCGTCATACACATCACGAGGAAACGAAGCCC

AAATACGAAAGTAGCTCTAAATATAATCCTGACGCCACGAAAGCCAAGAAA

>847_Sample_ASV3

CACCGCGGTTATACGAAAGACCCTAGTTGATAACTACGGCATAAAGGGTGGTTAGGGAGT

TTAAAAAATAAAGCCAAAGCTCTTCTAAGCTGTCATACGCTCTCTGAAAGAACGAGGCCC

AAACACGAAAGTAGCTTTAAATAAATAACCTGATACCACGAAAGCCAAGAAA

>847_Sample_ASV4

CACCGCGGTTATACGAGAGGCCCAAGTTGACAGACACCGGCGTAAAGAGTGGCTAGGGAA

AAATTACTACTAAAGCCGAACACCTTCAGAACTGTCATACGTTCCCGAAGATAAGAAGCC

CCACCACGAAAGTGGCTTTATATTACCCGACCCCACGAAAGCTGTGAAA

>847_Sample_ASV5

CACCGCGGTTATACGAGAGGCCCAAGTTGACAGACACCGGCGTAAAGAGTGGCTAGGGAA

AAATTACTACTAAAGCCGAACACCTTCAGAACTGTCATACGTTCCCGAAGATAAGAAGCC

CCACCACGAAAGTGGCTTTATATTACCCGACCCCACGAAAGCTAAGAAA

>847_Sample_ASV6

CACCGCGGTTATACGAAAGACCCTAGTTTATAGAAACGGCGTAAAGGGTGGTTAAGGGAC

AAACAAAATAAGGCCAAAGACTTCCTAAGCCGTCATACGCTCCACGAAAGCACGAAGCCC

AAACACGAAAGTAGCCTTAATAACTTCACCTGACCCCACGAAAGCTGGGAGA

>847_Sample_ASV7

CACCGCGGTTATACGAGAGACCCTAGTTGATATATACGGCGTAAAGAGTGGTTTGGGATA

TTTCTCCAAAATAAAGCCGAAGATCTCCCAAGCCGTCACACGCATCCCGGAGACACGAAG

CCCTGATACGAAGGTAGCTTTATTAACACCCCGACGCCACGAAAGCTAAGAAA

>847_Sample_ASV8

CACCGCGGTTATACGAGAGGCCCAAGTTGATAAGCCCGGCGTAAAGAGTGGTTATGGAGA

ATAAAACACTAAAGCTAAAGACCCCCTAGGCTGTTATACGCACCTGGCGGCTCGAACCAC

CTATACGAAAGTAGCTTTACCCCCCTTCCACCAGAATCCACGACAGCTGGGGCA

>847_Sample_ASV9

CACCGCGGTTATACGAGAGGCCCAAGTTGACAGACATCGGCGTAAAGAGTGGTTAAGGAA

ACCCCATTACTAAAGCCGAACGCCCTCAGAACTGTCATACGTTCCCGAAGGTAAGAAGCC

CCACTACGAAAGTGGCTTTAACCACACCCGACCCCACGAAAGCTGTGAAA

>847_Sample_ASV10

CACCGCGGTTATACGAGAGGCCCAAGTTGACAGACACCGGCGTAAAGAGTGGTTAAGGAA

ATTCATCAACTAAAGCCGAATGCCCTCAGGACTGTTATACGTTTGCGAAGGTAAGAAGCC

CTACTACGAAAGTGGCTTTATTCCACCTGATTCCACGAAAGCTGCGGAA

>847_Sample_ASV11

CACCGCGGTTATACGAGAGGCCCCAGTTGATATTAGCGGCGTAAAGGGTGGTTAAGGACT

TTAATAACTAAAGCTAAACACTTCCCCGGCTGTTGTACGTAGACCGGAAGCTTGAAGCCC

AGACACGAAAGTAGCTTTATTCCCCCTGACCCCACGAAAGCTGAGAAA

>847_Sample_ASV12

CACCGCGGTTATACGAGAAACCCTAGTTGACAAATACGGCGTAAAGAGTGGTTAAGGGGT

ATCAAAAATAAAGTCAAACATTTTCCTAGTTGTTATAAGCATTTCGAGAACATGAAGCCC

AACCACGAAAGTAACTTTACCTTCACCTGACCCCACGAAAGCTAAGAAA

>847_Sample_ASV13

CACCGCGGTTATACGAGAGACCCTAATTGATAGGTACGGCGTAAAGAGTGGTTTGGGATA

ACCCAAAAATAAAGCTAAAGACCTTCTAAGCCGTCACACGCATACCGAAGGCACGAGGCC

CCAACACGAAAGTAGCTTTACTATTACCCCGACGCCACGAAAGCTAAGAAA

>847_Sample_ASV14

CACCGCGGTTATACGAGAGACCCTAATTGATAGGCACGGCGTAAAGAGTGGTTTAGGGTA

ACCCATAAATAAAGCTAAAGACCTTCTAAGCCGTCACACGCACACCGAAGGCACGAGACC

CCAACACGAAAGTAGCTTTACTTCTACCCCTGACGCCACGAAAGCTAAGAAA

>847_Sample_ASV15

CACCGCGGTTATACGAGAGGCCCTAGTTGATAACTACCGGCGTAAAGAGTGGTTATGGAA

AAATATTTAATAAAGCCGAACACCCCCTCAGCCGTCATACGCACCTGGGAGCACGAAGAC

CTACTGCGAAAGCAGCTTTAATTATGCCTGACCCCACGACAGCTAAGAAA

>847_Sample_ASV16

CACCGCGGTTATACGAGAGACCCCAGTTGATAAACACGGCGTAAAGAGTGGTTAAGGGCA

CAAAACAAATAAAGTCAAACAACCTCTTAGCTGTTATACGCATTATGAGGATATGAAGCC

CCCCTACGAAAGTAACTTTACCAACCCCTGACCCCACGAAAGCTAAGAAA

>847_Sample_ASV17

CACCGCGGTTATACGAAAGACCCTAGTTTATAGGCACGGCGTAAAGGGTGGTTAGGGGAT

AAACAAAATAAAGCCAAAGACCTTCTAAGTCGTCATACACTCATGAAGGCACGAAGCCCA

AATACGAAAGTAACTTTACAACTTAGACCCGACTCCACGAAAGCTGGGGGA

>847_Sample_ASV18

CACCGCGGTTATACGAGAGACTCCAGTTGATAGCTACGGCGTAAAGAGTGGTTTGGGGCC

CGCCCAAAATAAAGCCAAAGACCTCCCAAGCCGTCAAACGCACCCCGGAGGCACGAAGTC

CTAACGCGAAAGCAACTTTACCTTCCCCGACGCCACGAAAGCTAAGAAA

>847_Sample_ASV19

CACCGCGGTTATACGAAAGACCCTAGTTGATAGATCACGGCGTAAAGGGTGGTTAAGGAG

AACAAAAATAAAGCTAAAAATCCTCTAAGCTGTCATACGCTTTCCGAAGACATGAGACCC

AACCACGAAAGTAACTTTAAACTTTCCTGACACCACGAAAGCCAAGAAA

>851_Sample_ASV1

CACCGCGGTTATACGAAAGACCCTAGTTGATGGGTGCGGCGTAAAGGGTGGTTAGGGACT

ACAAAAAATAGAGCTAAAGATCCTCTAGGTCGTCATACACATCACGAGAAAACGAAGCCC

AAATACGAAAGTAGCTCTAAATATAATCCTGACGCCACGAAAGCCAAGAAA

>851_Sample_ASV2

CACCGCGGTTATACGAGAGACCCTAGTTGATTAAAGCGGCGTAAAGAGTGGTTATGGAAC

TATTCTTTTAAAGCAGAAAACCTCTCAAACTGTTATACGCACCCAGAGGTCTGAATCCCT

CACACGAAAGTGACTTTATTTATGCCTACCAGAACCCACGAAAGCTGGGACA

>851_Sample_ASV3

CACCGCGGTTATACGAAAGACCCTAGTTTATAGGTACGGCGTAAAGGGTGGTTAGGGGAC

AAACAAAATAAAGCCAAAGACCTCCTAAGCCGTCATACGCTCACGGAAGCACGAAGCCCA

AACACGAAAGTAGCTTTACCGAACATGCCCGACTCCACGAAAGCTGGGGAA

>851_Sample_ASV4

CACCGCGGTTATACGAGAGGCCCAAGTTGACAGACACCGGCGTAAAGAGTGGCTAGGGAA

AAATTACTACTAAAGCCGAACACCTTCAGAACTGTCATACGTTCCCGAAGATAAGAAGCC

CCACCACGAAAGTGACTTTATATTACCCGACCCCACGAAAGCTGTGAAA

>851_Sample_ASV5

CACCGCGGTTATACGAGAGGCCCAAGTTGACAGACACCGGCGTAAAGAGTGGCTAGGGAA

AAATTACTACTAAAGCCGAACACCTTCAGAACTGTCATACGTTCCCGAAGATAAGAAGCC

CCACCACGAAAGTGACTTTATATTACCCGGCCCCACGAAAGCTGTGAAA

>851_Sample_ASV6

CACCGCGGTTATACGAGAGGCCCAAGTTGACAGACACCGGCGTAAAGAGTGGCTAGGGAA

AAATTACTACTAAAGCCGAACACCTTCAGAACTGTCATACGTTCCCGAAGATAAGAAGCC

CCACCACGAAAGTGGCTTTATATTACCCGACCCCACGAAAGCTAAGAAA

>851_Sample_ASV7

CACCGCGGTTATACGAGAGGCCCAAGTTGACAGACACCGGCGTAAAGAGTGGCTAGGGAA

AAATTACTACTAAAGCCGAACACCTTCAGAACTGTCATACGTTCCCGAAGATAAGAAGCC

CCACCACGAAAGTGGCTTTATATTACCCGACCCCACGAAAGCTGTGAAA

>851_Sample_ASV8

CACCGCGGTTATACGAGAGGCCCAAGTTGACAGACACCGGCGTAAAGAGTGGCTAAGGAA

AAATTTCTACTAAAGCCGAACACCTTCAGAACTGTCATACGTTCCCGAAGATAAGAAGCC

CCACCACGAAAGTGGCTTTATATTACCCGACCCCACGAAAGCTGTGAAA

>851_Sample_ASV9

CACCGCGGTTATACGAGAGGCCCAAGTTGACAGACACCGGCGTAAAGAGTGGCTAAGGAA

AAATTTCTACTAAAGCCGAACACCTTCAGAACTGTCATACGTTCCCGAAGAAAAGAAGCC

CCACCACGAAAGTGGCTTTATATTACCCGACCCCACGAAAGCTGTGAAA

>851_Sample_ASV10

CACCGCGGTTATACGAGAGACCCTAGTTGATATATACGGCGTAAAGAGTGGTTTGGGGTT

ATGCCTCCAAAATAAAGCCGAAGATCTCCCAAGCCGTCACACGCATCCCGGAGACACGAA

GCCCTAATACGAAGGTAGCTTTATTAATTCCCCGACGCCACGAAAGCTAAGAAA

>851_Sample_ASV11

CACCGCGGTTATACGAGAGACCCTAGTTGATATATACGGCGTAAAGAGTGGTTTGGGGTT

ATGCCTCCAAAATAAAGCCGAAGATCTCCCAAGCCGTCACACGCATCCCGGAGACACGAA

GCCCTAATACGAAGGTAGCTTTATTAGTACCCCGACGCCACGAAAGCTAAGAAA

>851_Sample_ASV12

CACCGCGGTTATACGAGAGGCCCAAGTTGATAAGCCCGGCGTAAAGAGTGGTTATGGAGA

ATAAAACACTAAAGCTAAAGACCCCCTAGGCTGTTATACGCACCTGGCGGCTCGAACCAC

CTATACGAAAGTAGCTTTACCCCCCTTCCACCAGAATCCACGACAGCTGGGGCA

>851_Sample_ASV13

CACCGCGGTTATACGAGAGGCCCTAGTTGATAGCCACGGCGCAAAGAGTGGTTAAGGAGC

CCCACTAAATAAAGTCGAACACTTCCTAGGCCGTTATACGTTTTCTAGAAGCACGAAACC

CAATTACACGAAAGCAACTTTATACTAAACAACCTGACCCCACGAAAGCTAAGAAA

>851_Sample_ASV14

CACCGCGGTTATACGAGAGGCCCAAGTTGACAGACATCGGCGTAAAGAGTGGTTAAGGAA

ACCCCATTACTAAAGCCGAACGCCCTCAGAACTGTCATACGTTCCCGAAGGTAAGAAGCC

CCACTACGAAAGTGGCTTTAACCACACCCGACCCCACGAAAGCTGTGAAA

>851_Sample_ASV15

CACCGCGGTTATACGAGAGGCCCAAGTTGACAGACACCGGCGTAAAGAGTGGTTAAGGAA

ATTCATCAACTAAAGCCGAATGCCCTCAGGACTGTTATACGTTTGCGAAGGTAAGAAGCC

CTACTACGAAAGTGGCTTTATTCCACCTGATTCCACGAAAGCTGCGGAA

>851_Sample_ASV16

CACCGCGGTCACACGATTAACCCAAGTCAATAGAAGCCGGCGTAAAGAGTGTTTTAGATC

ACCCCCTCCCCAATAAAGCTAAAACTCACCTGAGTTGTAAAAAACTCCAGTTGACACAAA

ATAGACTACGAAAGTGGCTTTAACATATCTGAACACACAATAGCTAAGACC

>851_Sample_ASV17

CACCGCGGTTATACGAGAAACCCTAGTTGACAAATACGGCGTAAAGAGTGGTTAAGGGGT

ATCAAAAATAAAGTCAAACATTTTCCTAGTTGTTATAAGCATTTCGAGAACATGAAGCCC

AACCACGAAAGTAACTTTACCTTCACCTGACCCCACGAAAGCTAAGAAA

>851_Sample_ASV18

CACCGCGGTTATACGAAAGACCCCAGTTTATAGACACGGCGTAAAGGGTGGTTATAGGAC

AAACTAACATAGAGCCAAAGACCCCCTAAGCCGTCATACGCTCACGAGGGCTCGAAGCCC

CTATACGAAAGTAGCTCTACCAAAAAATGCTCGACTCCACGAAAGCTGGGACA

>851_Sample_ASV19

CACCGCGGTTATACGAGAGGCCCCAGTTGATAAACACGGCGTAAAGGGTGGTTAAGGGAG

CATAAGAATAAAGCCGAAGGGCCCTTTGGCCGTTATACGCTTCTAGGTGTCCGAAGCCCA

AACACGAAAGTAGCTTTAGTTAAGCCCACCTGACTCCACGAAAACTGAGAAA

>851_Sample_ASV20

CACCGCGGTTATACGAGAGACCCTAGTTGATAAATACGGCGTAAAGAGTGGTTATGGGAA

AAACAAATAAAGTCAAACAACCTCTTAGCTGTTATACGCATTATGAGAGCATGAAGCCCC

CTCACGAAAGTAACTTTAATACCTCCTGACCCCACGAAAGCTAAGAAA

>851_Sample_ASV21

CACCGCGGTTATACGAGAGGCTCAAGTTGATGAACCCCGGCGTAAAGAGTGGTTAAGGGA

GATCAAAACTAAAGCCGAATGCTTTCAAAGCTGTTATACGCTTCCGAAAGTAAGAAGCTC

AATCACGAAAGTGGCTTTACTTTACCTGACCCCACGAAAGCTACGACA

>851_Sample_ASV22

CACCGCGGTTATACGAGAGGCTCAAGTTGACAAACCCCGGCGTAAAGAGTGGTTAAGGAA

ACCTAAAGACTAAAGCCGAATGCCTTCAAAGCTGTTATACGCTCCCGAAGGTGAGAAGTT

CAATCACGAAAGTGGCTTTATAACCCCTGACCCCACGAAAGCTATGATA

>851_Sample_ASV23

CACCGCGGTTATACGAGAGACCCTAATTGATAGACACGGCGTAAAGAGTGGTTTGGGAAA

GCCCAAAAATAAAGCTAAAGACCTTCTAAGCCGTCACACGCACGCCGAAGGCACGAAGCC

CTGACACGAAAGTAGCTTTACTATTACCCCCGACGCCACGAAAGCTAAGAAA

>851_Sample_ASV24

AACCGCGGTTATACGAGAGACCCTAATTGATAGCTCACGGCGTAAAGGGTGGTTTGGGGA

AACCTCAAAATAAAGCTAAAGACCTTCTAAGCCGTTACACGCATACCGAAGGCACGAGGC

CCTAACACGAAAGTAGCTTTACTATTACCCCTGACGCCACGAAAGCTAAGAAA

>851_Sample_ASV25

CACCGCGGTTATACGAAAGACCCTAGTTGATGGATCACGGCGTAAAGGGTGGTTAAGGGA

TATAAAAATAAAGCTAAAAATCCTCTTAGCTGTCATACGCTTTCCGAAGACATGAGGCCC

AATTACGAAAGTAGCTTTAAACCACCCTGACACCACGAAAGCCAAGAAA

>851_Sample_ASV26

CACCGCGGTTATACGAAAGACCCCAGTTTATAGATGCGGCGTAAAGGGTGGTTAAGAGAA

AATCAAAATAAGGCCAAAGATCCCCTAAGCAGTCATACGCTCCACGAGGACACGAAACCC

AAATACGAAAGTTGCCTTAATAAACTTCACCTGATCCCACGAAAGCTGAAAAA

>851_Sample_ASV27

CACCGCGGTTATACGAGAGGCCCAAGTTGACAAACCCCGGCGTAAAGAGTGGTTAAGGAA

AATTTAAAACTAAAGCCGAATGCCCCCAAAGCTGTTATACGCACCCGAGGGTAAGAAGCC

CAATCACGAAAGTGGCTTTACACTACCTGAACCCACGAAAGCTATGAAA

>851_Sample_ASV28

CACCGCGGTTATACGAGAGGCCCAAGTTGACAAACCCCGGCGTAAAGAGTGGTTAAGGAA

AATTTAAAACTAAAGCCGAATGCCCCCAAAGCTGTTATACGCACCCGAGGATAAGAAGCC

CAATCACGAAAGTGGCTTTACACTACCTGAACCCACGAAAGCTATGAAA

>851_Sample_ASV29

CACCGCGGTCATACGATTAACCCAAGTTAATAGGCACACGGCGTAAAGCGTGTTTAAGCA

CTACGCCAAATAAAGTTAAACTCCAATTAAGCTGTAAAAAGCCATAATCAAGACGAAAAT

AAATAACGAAAGTAACTTTACAACCGCTGAGACACGATAGCTAAGATC

>851_Sample_ASV30

CACCGCGGTCATACGATTAACCCAAGTTAATAGGCACACGGCGTAAAGCGTGTTTAAGCA

CTATGCCAAATAAAGTTAAACTCCAATTAAGCTGTAAAAAGCCATAATCAAGACGAAAAT

AAATAACGAAAGTAACTTTACAACCGCTGAAACACGATAGCTAAGATC

>851_Sample_ASV31

CACCGCGGTTATACGAGAGGCCCTAGTTGATAACTACCGGCGTAAAGAGTGGTTATGGAA

AAATATTTAATAAAGCCGAACACCCCCTCAGCCGTCATACGCACCTGGGAGCACGAAGAC

CTACTGCGAAAGCAGCTTTAATTATGCCTGACCCCACGACAGCTAAGAAA

>851_Sample_ASV32

CACCGCGGTTATACGAAAGACCCTAGTTTATAGACACGGCGTAAAGGGTGGTTCGGGACT

AAACAAAATAAAGCCAAAGACCTCCTAAGCCGTCATACGCTCACGGAAGCACGAAGCCCA

AGTACGAAAGTAGCTTTACAAAACATGCCTGACTCCACGAAAGCTAGGAAA

>851_Sample_ASV33

CACCGCGGTTATACGAAAGACCCTAGTTGATAGGCACGGCGTAAAGGGTGGTTAGGGATA

TATCAAAAATAAAGTTAAAGAGCCTCTAAGCTGTCGCACGCATCCCGAGAGCTCGAGACC

CAGACACGAAAGTAACTTTAAAATACCTCACCTGACCCCACGAAAGCTAAGAAA

>851_Sample_ASV34

CACCGCGGTTATACGAAAGACCCTAGTTGATAGGCACGGCGTAAAGGGTGGTTAGGGATA

TATTAAAAATAAAGTTAAAGAGCCTCTAAGCTGTCGCACGCATCCCGAGAGCTCGAGACC

CAGACACGAAAGTAACTTTAAAATACCTCACCTGACCCCACGAAAGCTAAGAAA

>851_Sample_ASV35

CACCGCGGTTATACGAGAGGCCCAAGTTGATACTCCACGGCGTAAAGAGTGGTTAGAAAA

GACCTACTACTAAAGCCGAACGCCTTCAAAGCTGTTATACGCATCCGAAGGTGAGAAGCC

CACCCACGAAAGTGGCTTTACAACCTTGACTCCACGAAAGCTATGACA

>851_Sample_ASV36

CACCGCGGTTATACGAGAGACCCCAGTTGATAAACACGGCGTAAAGAGTGGTTAAGGGCA

CAAAACAAATAAAGTCAAACAACCTCTTAGCTGTTATACGCATTATGAGGATATGAAGCC

CCCCTACGAAAGTAACTTTACCAACCCCTGACCCCACGAAAGCTAAGAAA

>851_Sample_ASV37

CACCGCGGTTATACGAGAGACCCTAGTTGATATATACGGCGTAAAGAGTGGTTTGGAATA

ACCAAGTAATATAGCCAAAGACCTCCCAAGCCGTCACACGCACCCCGGAGGCACGAAGCC

CAAACACGAAAGTAGCTTTATTAACTTACCGACGCCACGAAAGCTGGGGAA

>851_Sample_ASV38

CACCGCGGTTATACGAGAGACCCTAGTTGATATATACGGCGTAAAGAGTGGTTTGGAATA

ACCAAGTAATATAGCCAAAGACCTCCCAAGCCGTCACACGCACCCCGGAGGCACGAAGCC

CAAACACGAAAGTAGCTTTATTAACTTACCGACGCCACGAAAGCTAAGAAA

>851_Sample_ASV39

CACCGCGGTTATACGAAAGACCCTAGTTTATAGGCACGGCGTAAAGGGTGGTTAGGGGAT

AAACAAAATAAAGCCAAAGACCTTCTAAGTCGTCATACACTCATGAAGGCACGAAGCCCA

AATACGAAAGTAACTTTACAACTTAGACCCGACTCCACGAAAGCTGGGGGA

>851_Sample_ASV40

CACCGCGGTTATACGAGAGACTCCAGTTGATAGCTACGGCGTAAAGAGTGGTTTGGGGCC

CCACCCAAAATAAAGCCAAAGACCTCCCAAGCCGTCAAACGCACCCCGGAGGCACGAAGT

CCTAACGCGAAAGCAACTTTACCTCCCCCGACGCCACGAAAGCTAAGAAA

>851_Sample_ASV41

CACCGCGGTCATACGATTAACCCAAGTTAATAGGCACACGGCGTAAAGCGTGTTAAAGCA

CTATACTAAATAAAGTTAAATTCCAATTAAGCTGTAAAAAGCCATAATTGCAACAAAAAT

AAACAACGAAAGTAACTTTACAGTCGCTGAAACACGATAGCTAGGACC

>851_Sample_ASV42

CACCGCGGTTATACGAAAGACCCTAGTTGATAGATCACGGCGTAAAGGGTGGTTAAGGAG

AACAAAAATAAAGCTAAAAATCCTCTAAGCTGTCATACGCTTTCCGAAGACATGAGACCC

AACCACGAAAGTAACTTTAAACTTTCCTGACACCACGAAAGCCAAGAAA

>854_Sample_ASV1

CACCGCGGTTATACGAGAGGCCCAAGTTGACAGACATCGGCGTAAAGAGTGGTTAAGGAA

AACCTTCAACTAAAGTCGAACGCTCTCAAGACTGTCGTACGTTTCCGAGAGTATGAAGCC

CCACCACGAAAGTGACTTTACCTCACCTGACTCCACGAAAGCTGCGAAA

>854_Sample_ASV2

CACCGCGGTTATACGAAAGACCCTAGTTGATGGGTGCGGCGTAAAGGGTGGTTAGGGACT

ACAAAAAATAGAGCTAAAGATCCTCTAGGTCGTCATACACATCACGAGGAAACGAAGCCC

AAATACGAAAGTAGCTCTAAATATAATCCTGACGCCACGAAAGCCAAGAAA

>854_Sample_ASV3

CACCGCGGTTATACGAAAGACCCTAGTTGATAACTACGGCATAAAGGGTGGTTAGGGAGT

TTAAAAAATAAAGCCAAAGCTCTTCTAAGCTGTCATACGCTCTCTGAAAGAACGAGGCCC

AAACACGAAAGTAGCTTTAAATAAATAACCTGATACCACGAAAGCCAAGAAA

>854_Sample_ASV4

CACCGCGGTTATACGAGAGACCCTAGTTGATTAAAGCGGCGTAAGGAGTGGTTATGGAAC

TATTCTTTTAAAGCAGAAAACCTCTCAAACTGTTATACGCACCCAGAGGTCTGAATCCCT

CACACGAAAGTGACTTTATTTATGCCTACCAGAACCCACGAAAGCTGGGACA

>854_Sample_ASV5

CACCGCGGTTATACGAAAGACCCTAGTTTATAGGTACGGCGTAAAGGGTGGTTAGGGGAC

AAACAAAATAAAGCCAAAGACCTCCTAAGCCGTCATACGCTCACGGAAGCACGAAGCCCA

AACACGAAAGTAGCTTTACCGAACATGCCCGACTCCACGAAAGCTGGGGAA

>854_Sample_ASV6

CACCGCGGTTATACGAGAGGCCCAAGTTGACAGACACCGGCGTAAAGAGTGGCTAGGGAA

AAATTACTACTAAAGCCGAACACCTTCAGAACTGTCATACGTTCCCGAAGATAAGAAGCC

CCACCACGAAAGTGGCTTTATATTACCCGACCCCACGAAAGCTGTGAAA

>854_Sample_ASV7

CACCGCGGTTATACGAGAGGCCCAAGTTGACAGACACCGGCGTAAAGAGTGGCTAAGGAA

AAATTTCTACTAAAGCCGAACACCTTCAGAACTGTCATACGTTCCCGAAGATAAGAAGCC

CCACCACGAAAGTGGCTTTATATTACCCGACCCCACGAAAGCTGTGAAA

>854_Sample_ASV8

CACCGCGGTTATACGAGAGGCCCAAGTTGACAGACACCGGCGTAAAGAGTGGCTAAGGAA

AAATTTCTACTAAAGCCGAACACCTTCAGAACTGTCATACGTTCCCGAAGAAAAGGAGCC

CCACCACGAAAGTGGCTTTATATTACCCGACCCCACGAAAGCTGTGAAA

>854_Sample_ASV9

CACCGCGGTTATACGAAAGACCCTAGTTTATAGAAACGGCGTAAAGGGTGGTTAAGGGAC

AAACAAAATAAGGCCAAAGACTTCCTAAGCCGTCATACGCTCCACGAAAGCACGAAGCCC

AAACACGAAAGTAGCCTTAATAACTTCACCTGACCCCACGAAAGCTGGGAGA

>854_Sample_ASV10

CACCGCGGTTATACGAGAGACCCTAGTTGATATATACGGCGTAAAGAGTGGTTTGGGATA

TTTCTCCAAAATAAAGTCGAAGATCTCCCAAGCCGTCACACGCATCCCGGAGACACGAAG

CCCTGATACGAAGGTAGCTTTATTAACACCCCGACGCCACGAAAGCTAAGAAA

>854_Sample_ASV11

CACCGCGGTTATACGAGAGGCCCAAGTTGATAAGCCCGGCGTAAAGAGTGGTTATGGAGA

ATAAAACACTAAAGCTAAAGACCCCCTAGGCTGTTATACGCACCTGGCGGCTCGAACCAC

CTATACGAAAGTAGCTTTACCCCCCTTCCACCAGAATCCACGACAGCTGGGGCA

>854_Sample_ASV12

CACCGCGGTTATACGAGAGGCCCTAGTTGATAGCTACGGCGCAAAGAGTGGTTAAGGAAC

TCACCCAAATAAAGTCAAACACTTCCTAGGCCGTTATACGTTTTCTAGAAGCACGAAGCC

CAATTACACGAAAGCAACTTTATTCTACACCTGACCCCACGAAAGCTAAGAAA

>854_Sample_ASV13

CACCGCGGTTATACGAGAGGCCCAAGTTGACAGACATCGGCGTAAAGAGTGGTTAAGGAA

ACCCCATTACTAAAGCCGAACGCCCTCAGAACTGTCATACGTTCCCGAAGGTAAGAAGCC

CCACTACGAAAGTGGCTTTAACCACACCCGACCCCACGAAAGCTGTGAAA

>854_Sample_ASV14

CACCGCGGTTATACGAGAGGCCCAAGTTGACAGACACCGGCGTAAAGAGTGGTTAAGGAA

ATTCATCAACTAAAGCCGAATGCCCTCAGGACTGTTATACGTTTGCGAAGGTAAGAAGCC

CTACTACGAAAGTGGCTTTATTCCACCTGATTCCACGAAAGCTGCGGAA

>854_Sample_ASV15

CACCGCGGTTATACGAGAGGCCCCAGTTGATATTAGCGGCGTAAAGGGTGGTTAAGGACT

TTAATAACTAAAGCTAAACACTTCCCCGGCTGTTGTACGTAGACCGGAAGCTTGAAGCCC

AGACACGAAAGTAGCTTTATTCCCCCTGACCCCACGAAAGCTGAGAAA

>854_Sample_ASV16

CACCGCGGTTATACGAGAAACCCTAGTTGACAAATACGGCGTAAAGAGTGGTTAAGGGGT

ATCAAAAATAAAGTCAAACATTTTCCTAGTTGTTATAAGCATTTCGAGAACATGAAGCCC

AACCACGAAAGTAACTTTACCTTCACCTGACCCCACGAAAGCTAAGAAA

>854_Sample_ASV17

CACCGCGGTTATACGAGAGGCTCAAGTTGATAAACCCCGGCGTAAAGAGTGGTTAAGGAA

AAATAGAAACTAAAGCCGAATGCTTTCAAGACTGTTATACGTTCCCGAGAGTAAGAAGAC

CAATTACGAAAGTGGCTTTACTTTACCTGACCCCACGAAAGCTACGACA

>854_Sample_ASV18

CACCGCGGTTATACGAGAGACCCTAATTGATAGACACGGCGTAAAGAGTGGTTTGGGAAA

GCCCAAAAATAAAGCTAAAGACCTTCTAAGCCGTCACACGCACGCCGAAGGCACGAAGCC

CTGACACGAAAGTAGCTTTACTATTACCCCCGACGCCACGAAAGCTAAGAAA

>854_Sample_ASV19

CACCGCGGTTATACGAGAGACCCTAATTGATAGGTACGGCGTAAAGAGTGGTTTGGGATA

ACCCAAAAATAAAGCTAAAGACCTTCTAAGCCGTCACACGCATACCGAAGGCACGAGGCC

CCAACACGAAAGTAGCTTTACTATTACCCCGACGCCACGAAAGCTAAGAAA

>854_Sample_ASV20

CACCGCGGTTATACGAAAGACCCTAGTTGATGGATCACGGCGTAAAGGGTGGTTAAGGGA

TATAAAAATAAAGCTAAAAATCCTCTTAGCTGTCATACGCTTTCCGAAGACATGAGGCCC

AATTACGAAAGTAGCTTTAAACCACCCTGACACCACGAAAGCCAAGAAA

>854_Sample_ASV21

CACCGCGGTCATACGATTAACCCAAGTTAATAGGCACACGGCGTAAAGCGTGTTTAAGCA

CTACGCCAAATAAAGTTAAACTCCAATTAAGCTGTAAAAAGCCATAATCAAGACGAAAAT

AAATAACGAAAGTAACTTTACAACCGCTGAAACACGATAGCTAAGATC

>854_Sample_ASV22

CACCGCGGTCATACGATTAACCCAAGTTAATAGGCACACGGCGTAAAGCGTGTTTAAGCA

CTATGCCAAATAAAGTTAAACTCCAATTAAGCTGTAAAAAGCCATAATCAAGACGAAAAT

AAATAACGAAAGTAACTTTACAACCGCTGAAACACGATAGCTAAGATC

>854_Sample_ASV23

CACCGCGGTTATACGAGAGGCCCTAGTTGATAACTACCGGCGTAAAGAGTGGTTATGGAA

AAATATTTAATAAAGCCGAACACCCCCTCAGCCGTCATACGCACCTGGGAGCACGAAGAC

CTACTGCGAAAGCAGCTTTAATTATGCCTGACCCCACGACAGCTAAGAAA

>854_Sample_ASV24

CACCGCGGTTATACGAGAGACCCCAGTTGATAAACACGGCGTAAAGAGTGGTTAAGGGCA

CAAAACAAATAAAGTCAAACAACCTCTTAGCTGTTATACGCATTATGAGGATATGAAGCC

CCCCTACGAAAGTAACTTTACCAACCCCTGACCCCACGAAAGCTAAGAAA

>854_Sample_ASV25

CACCGCGGTTATACGAGAGACCCTAGTTGATATATACGGCGTAAAGAGTGGTTTGGAATA

ACCAAGTAATATAGCCAAAGACCTCCCAAGCCGTCACACGCACCCCGGAGGCACGAAGCC

CAAACACGAAAGTAGCTTTATTAACTTACCGACGCCACGAAAGCTAAGAAA

>854_Sample_ASV26

CACCGCGGTCATACGATTAACCCAAGTTAATAGGCACACGGCGTAAAGCGTGTTAAAGCA

CTATACTAAATAAAGTTAAATTCCAATTAAGCTGTAAAAAGCCATAATTGCAACAAAAAT

AAACAACGAAAGTAACTTTACAGTCGCTGAAACACGATAGCTAGGACC

>854_Sample_ASV27

CACCGCGGTTATACGAGAGACTCCAGTTGATAGCTACGGCGTAAAGAGTGGTTTGGGGCC

CGCCCAAAATAAAGCCAAAGACCTCCCAAGCCGTCAAACGCACCCCGGAGGCACGAAGTC

CTAACGCGAAAGCAACTTTACCTTCCCCGACGCCACGAAAGCTAAGAAA

>854_Sample_ASV28

CACCGCGGTTATACGAAAGACCCTAGTTGATAGATCACGGCGTAAAGGGTGGTTAAGGAG

AACAAAAATAAAGCTAAAAATCCTCTAAGCTGTCATACGCTTTCCGAAGACATGAGACCC

AACCACGAAAGTAACTTTAAACTTTCCTGACACCACGAAAGCCAAGAAA

>856_Sample_ASV1

CACCGCGGTTATACGAAAGACCCTAGTTGATGGGTGCGGCGTAAAGGGTGGTTAGGGACT

ACAAAAAATAGAGCTAAAGATCCTCTAGGTCGTCATACACATCACGAGGAAACGAAGCCC

AAATACGAAAGTAGCTCTAAATATAATCCTGACGCCACGAAAGCCAAGAAA

>856_Sample_ASV2

CACCGCGGTTATACGAAAGACCCTAGTTGATAGCCACGGCGTAAAGGGTGGTTAAGGAAC

CTAAAAAATAAAGCTAAAGACCCTCTAGGCCGTCATACGCATTACGAGGCAAACGAAACC

CCAACACGAAAGTAGCTTTAGACAAATATCCTGATGCCACGAAAGCCAAGAAA

>856_Sample_ASV3

CACCGCGGTTATACGAGAGGCCCAAGTTGACAGACACCGGCGTAAAGAGTGGCTAGGGAA

AAATTACTACTAAAGCCGAACACCTTCAGAACTGTCATACGTTCCCGAAGATAAGAAGCC

CCACCACGAAAGTGACTTTATATTACCCGACCCCACGAAAGCTGTGAAA

>856_Sample_ASV4

CACCGCGGTTATACGAGAGGCCCAAGTTGACAGACACCGGCGTAAAGAGTGGCTAGGGAA

AAATTACTACTAAAGCCGAACACCTTCAGAACTGTCATACGTTCCCGAAGATAAGAAGCC

CCACCACGAAAGTGACTTTATATTACCCGACCCCACGAAAGCTAAGAAA

>856_Sample_ASV5

CACCGCGGTTATACGAGAGGCCCAAGTTGACAGACACCGGCGTAAAGAGTGGCTAGGGAA

AAATTACTACTAAAGCCGAACACCTTCAGAACTGTCATACGTTCCCGAAGATAAGAAGCC

CCACCACGAAAGTGGCTTTATATTACCCGACCCCACGAAAGCTGTGAAA

>856_Sample_ASV6

CACCGCGGTTATACGAGAGGCCCAAGTTGATAAGCCCGGCGTAAAGAGTGGTTATGGAGA

ATAAAACACTAAAGCTAAAGACCCCCTAGGCTGTTATACGCACCTGGCGGCTCGAACCAC

CTATACGAAAGTAGCTTTACCCCCCTTCCACCAGAATCCACGACAGCTGGGGCA

>856_Sample_ASV7

CACCGCGGTTATACGAGAGGCCCAAGTTGACAGACATCGGCGTAAAGAGTGGTTAAGGAA

ACCCCATTACTAAAGCCGAACGCCCTCAGAACTGTCATACGTTCCCGAAGGTAAGAAGCC

CCACTACGAAAGTGGCTTTAACCACACCCGACCCCACGAAAGCTGTGAAA

>856_Sample_ASV8

CACCGCGGTTATACGAGAGACCCCAGTTGATAGCTACGGCGTAAAGAGTGGTTAAGGACC

CCATTTAATAAAGCCAAAGACCCCCCAAGCCGTCATACGCACACGAGGGCGCGAAGCTCG

CACACGAAAGTAGCTCTAATTTACCCGACGCCACGAAAGCTAAGGAA

>856_Sample_ASV9

CACCGCGGTTATACGAAAGACCCCAGTTTATAGACACGGCGTAAAGGGTGGTTATAGGAC

AAACTAACATAGAGCCAAAGACCCCCTAAGCCGTCATACGCTCACGAGGGCTCGAAGCCC

CTATACGAAAGTAGCTCTACCAAAAAATGCTCGACTCCACGAAAGCTGGGACA

>856_Sample_ASV10

CACCGCGGTTATACGAGAGACCCTAGTTGATAAATACGGCGTAAAGAGTGGTTATGGGAA

AAACAAATAAAGTCAAACAACCTCTTAGCTGTTATACGCATTATGAGAGCATGAAGCCCC

CTCACGAAAGTAACTTTAATACCTCCTGACCCCACGAAAGCTAAGAAA

>856_Sample_ASV11

CACCGCGGTTATACGAGAGACCCTAATTGATAGACACGGCGTAAAGAGTGGTTTGGGAAA

GCCCAAAAATAAAGCTAAAGACCTTCTAAGCCGTCACACGCACGCCGAAGGCACGAAGCC

CTGACACGAAAGTAGCTTTACTATTACCCCCGACGCCACGAAAGCTAAGAAA

>856_Sample_ASV12

CACCGCGGTTATACGAAAGACCCTAGTTTATAGATGCGGCGTAAAGGGTGGTTAAGGAAA

ACCTAAAATAATGCTAAAGACTCTCAAAGCTGTTATACGCTTCATGAGCACACGAAATCC

AAATACGAAAGTTGCCTTAATAAACTTTGCCTGACCCCACGAAAGCTGAAAAA

>856_Sample_ASV13

CACCGCGGTTATACGAGAGACCCTAGTTGATAGCTACGGCGTAAAGCGTGGTTAGGAGAA

CAAGATAAATAAAGTCAAACAATCTCTCGGCCGTTATACGTTATCTGAGAATATGAAGTC

CTACCACGAAAGTAACTTTAATTTTTCTGACCCCACGAAAACTGAGGAA

>856_Sample_ASV14

CACCGCGGTTAAACGAGAGGCCCTAGTTGATAGTACAACGGCGTAAAGGGTGGTTAAGGA

TAGTGAAATAATAAAGTCGAATGGCCCTTTGGCTGTCATACGCTTCTAGGAGTCCGAAGC

CCAATATACGAAAGTAACTTTAAGAGCGCCCACCTGACCCCACGAAAACTGAGAAA

>856_Sample_ASV15

CACCGCGGTTATACGAAAGACCCTAGTTGATAGGCACGGCGTAAAGGGTGGTTAGGGATA

TATTAAAAATAAAGTTAAAGAGCCTCTAAGCTGTCGCACGCATCCCGAGAGCTCGAGACC

CAGACACGAAAGTAACTTTAAAATACCTCACCTGACCCCACGAAAGCTAAGAAA

>856_Sample_ASV16

CACCGCGGTTATACGAGAGACCCCAGTTGATAAACACGGCGTAAAGAGTGGTTAAGGGCA

CAAAACAAATAAAGTCAAACAACCTCTTAGCTGTTATACGCATTATGAGGATATGAAGCC

CCCCTACGAAAGTAACTTTACCAACCCCTGACCCCACGAAAGCTAAGAAA

>856_Sample_ASV17

CACCGCGGTTATACGAGAGACCCTAGTTGATATATACGGCGTAAAGAGTGGTTTGGAATA

ACCAAGTAATATAGCCAAAGACCTCCCAAGCCGTCACACGCACCCCGGAGGCACGAAGCC

CAAACACGAAAGTAGCTTTATTAACTTACCGACGCCACGAAAGCTAAGAAA

>856_Sample_ASV18

CACCGCGGTTATACGAAAGACCCTAGTTTATAGGCACGGCGTAAAGGGTGGTTAGGGGAT

AAACAAAATAAAGCCAAAGACCTTCTAAGTCGTCATACACTCATGAAGGCACGAAGCCCA

AATACGAAAGTAACTTTACAACTTAGACCCGACTCCACGAAAGCTGGGGGA

>856_Sample_ASV19

CACCGCGGTTATACGAGAGACCCTAATTGATAGACTACGGCGTAAAGAGTGGTTTAGAAC

AAAAATTAATAAAGCCAAAGATCCTCCTGGCCGTCGCACGCATTTCGAGGATACGAAGCC

CCACTACGAAAGTAGCTTTACTACAACTTTTCTGACCCCACGAAAGCTAAGAAA

>856_Sample_ASV20

CACCGCGGTTATACGAAAGACCCTAGTTGATAGATCACGGCGTAAAGGGTGGTTAAGGAG

AACAAAAATAAAGCTAAAAATCCTCTAAGCTGTCATACGCTTTCCGAAGACATGAGACCC

AACCACGAAAGTAACTTTAAACTTTCCTGACACCACGAAAGCCAAGAAA

>860_Sample_ASV1

CACCGCGGTTATACGAAAGACCCTAGTTGATGGGTGCGGCGTAAAGGGTGGTTAGGGACT

ACAAAAAATAGAGCTAAAGATCCTCTAGGTCGTCATACACATCACGAGGAAACGAAGCCC

AAATACGAAAGTAGCTCTAAATATAATCCTGACGCCACGAAAGCCAAGAAA

>860_Sample_ASV2

CACCGCGGTTATACGAGAGACCCTAGTTGATTAAAGCGGCGTAAAGAGTGGTTATGGAAC

TATTCTTTTAAAGCAGAAAACCTCTCAAACTGTTATACGCACCCAGAGGTCTGAATCCCT

CACACGAAAGTGACTTTATTTATGCCTACCAGAACCCACGAAAGCTGGGACA

>860_Sample_ASV3

CACCGCGGTTATACGAAAGACCCTAGTTGATAGCCACGGCGTAAAGGGTGGTTAAGGAAC

CTAAAAAATAAAGCTAAAGACCCTCTAGGCCGTCATACGCATTACGAGGCAAACGAAACC

CCAACACGAAAGTAGCTTTAGACAAATATCCTGATGCCACGAAAGCCAAGAAA

>860_Sample_ASV4

CACCGCGGTTATACGAGAGGCCCAAGTTGACAGACACCGGCGTAAAGAGTGGCTAGGGAA

AAATTACTACTAAAGCCGAACACCTTCAGAACTGTCATACGTTCCCGAAGATAAGAAGCC

CCACCACGAAAGTGACTTTATATTACCCGACCCCACGAAAGCTAAGAAA

>860_Sample_ASV5

CACCGCGGTTATACGAGAGGCCCAAGTTGACAGACACCGGCGTAAAGAGTGGCTAGGGAA

AAATTACTACTAAAGCCGAACACCTTCAGAACTGTCATACGTTCCCGAAGATAAGAAGCC

CCACCACGAAAGTGACTTTATATTACCCGACCCCACGAAAGCTGTGAAA

>860_Sample_ASV6

CACCGCGGTTATACGAGAGGCCCAAGTTGACAGACACCGGCGTAAAGAGTGGCTAGGGAA

AAATTACTACTAAAGCCGAACACCCTCAGAACTGTCATACGTTCCCGAAGGTAAGAAGCC

CCACCACGAAAGTGGCTTTATAATACCCGACCCCACGAAAGCTGTGAAA

>860_Sample_ASV7

CACCGCGGTTATACGAGAGGCCCAAGTTGACAGACACCGGCGTAAAGAGTGGCTAGGGAA

AAATTACTACTAAAGCCGAACACCCTCAGAACTGTCATACGTTCCCGAAGGTAAGAAGCC

CCACCACGAAAGTGGCTTTATAATACCCGACCCCACGAAAGCTAAGAAA

>860_Sample_ASV8

CACCGCGGTTATACGAGAGGCCCAAGTTGACAGACACCGGCGTAAAGAGTGGCTAAGGAA

AAATTTCTACTAAAGCCGAACACCTTCAGAACTGTCATACGTTCCCGAAGATAAGAAGCC

CCACCACGAAAGTGGCTTTATATTACCCGACACCACGAAAGCTAAGAAA

>860_Sample_ASV9

CACCGCGGTTATACGAGAGGCCCAAGTTGACAGACACCGGCGTAAAGAGTGGCTAAGGAA

AAATTTCTACTAAAGCCGAACACCTTCAGAACTGTCATACGTTCCCGAAGATAAGAAGCC

CCACCACGAAAGTGGCTTTATATTACCCGACACCACGAAAGCTGTGAAA

>860_Sample_ASV10

CACCGCGGTTATACGAGAGACCCTAGTTGATATATACGGCGTAAAGAGTGGTTTGGGGTT

ATGCCTCCAAAATAAAGCCGAAGATCTCCCAAGCCGTCACACGCATCCCGGAGACACGAA

GCCCTAATACGAAGGTAGCTTTATTAATTCCCCGACGCCACGAAAGCTAAGAAA

>860_Sample_ASV11

CACCGCGGTTATACGAGAGACCCTAGTTGATATATACGGCGTAAAGAGTGGTTTGGGGTT

ATGCCTCCAAAATAAAGCCGAAGATCTCCCAAGCCGTCACACGCATCCCGGAGACACGAA

GCCCTAATACGAAGGTAGCTTTATTAGTACCCCGACGCCACGAAAGCTAAGAAA

>860_Sample_ASV12

CACCGCGGTTATACGAGAGACCCTAGTTGATATATACGGCGTAAAGAGTGGTTTGGGATA

TTTCTCCAAAATAAAGCCGAAGATCTCCCAAGCCGTCACACGCATCCCGGAGACACGAAG

CCCTAATACGAAGGTAGCTTTATTAGTACCCCGACGCCACGAAAGCTAAGAAA

>860_Sample_ASV13

CACCGCGGTTATACGAGAGACCCTAGTTGATATATACGGCGTAAAGAGTGGTTTGGGGTT

ATGCCTCCAAAATAAAGTCGAAGATCTCCCAAGCCGTCACACGCACCCCGGAGACACGAA

GCCCTAATACGAAGGTAGCTTTATTAATTCCCCGACGCCACGAAAGCTAAGAAA

>860_Sample_ASV14

CACCGCGGTTATACGAGAGACCCTAGTTGATATATACGGCGTAAAGAGTGGTTTGGGGTT

ATGCCTCCAAAATAAAGCCGAAGATCTCCCAAGCCGTCACACGCACCCCGGAGACACGAA

GCCCTAATACGAAGGTAGCTTTATTAATTCCCCGACGCCACGAAAGCTAAGAAA

>860_Sample_ASV15

CACCGCGGTTATACGAGAGGCCCAAGTTGATAAGCCCGGCGTAAAGAGTGGTTATGGAGA

ATAAAACACTAAAGCTAAAGACCCCCTAGGCTGTTATACGCACCTGGCGGCTCGAACCAC

CTATACGAAAGTAGCTTTACCCCCCTTCCACCAGAATCCACGACAGCTGGGGCA

>860_Sample_ASV16

CACCGCGGTTATACGAGAGGCCCAAGTTGACAGACATCGGCGTAAAGAGTGGTTAAGGAA

ACCCCATTACTAAAGCCGAACGCCCTCAGAACTGTCATACGTTCCCGAAGGTAAGAAGCC

CCACTACGAAAGTGGCTTTAACCACACCCGACCCCACGAAAGCTAAGAAA

>860_Sample_ASV17

CACCGCGGTTATACGAGAGGCCCAAGTTGACAGACATCGGCGTAAAGAGTGGTTAAGGAA

ACCCCATTACTAAAGCCGAACGCCCTCAGAACTGTCATACGTTCCCGAAGGTAAGAAGCC

CCACTACGAAAGTGGCTTTAACCACACCCGACCCCACGAAAGCTGTGAAA

>860_Sample_ASV18

CACCGCGGTTATACGAGAGGCCCCAGTTGATATTAGCGGCGTAAAGGGTGGTTAAGGACT

TTAATAACTAAAGCTAAACACTTCCCCGGCTGTTGTACGTAGACCGGAAGCTTGAAGCCC

AGACACGAAAGTAGCTTTATTCCCCCTGACCCCACGAAAGCTGAGAAA

>860_Sample_ASV19

CACCGCGGTCACACGATTAACCCAAGTCAATAGAAGCCGGCGTAAAGAGTGTTTTAGATC

ACCCCCTCCCCAATAAAGCTAAAACTCACCTGAGTTGTAAAAAACTCCAGTTGACACAAA

ATAGACTACGAAAGTGGCTTTAACATATCTGAACACACAATAGCTAAGACC

>860_Sample_ASV20

CACCGCGGTCACACGATTAACCCAAGTCAATAGAAGCCGGCGTAAAGAGTGTTTTAGATC

ACCCCCTCCCCAATAAAGCTAAAACTCACCTGAGTTGTAAAAAACTCCAGTTGACACAAA

ATAGACTACGAAAGTGGCTTTAACATATCTGAACACACAATAGCTAAGAAA

>860_Sample_ASV21

CACCGCGGTTATACGAGAGACCCCAGTTGATAGCTACGGCGTAAAGAGTGGTTAAGGACC

CCATTTAATAAAGCCAAAGACCCCCCAAGCCGTCATACGCACACGAGGGCGCGAAGCTCG

CACACGAAAGTAGCTCTAATTTACCCGACGCCACGAAAGCTAAGGAA

>860_Sample_ASV22

CACCGCGGTTATACGAGAGACCCTAGTTGATAAATACGGCGTAAAGAGTGGTTATGGGAA

AAACAAATAAAGTCAAACAACCTCTTAGCTGTTATACGCATTATGAGAGCATGAAGCCCC

CTCACGAAAGTAACTTTAATACCTCCTGACCCCACGAAAGCTAAGAAA

>860_Sample_ASV23

CACCGCGGTTATACGAGAGACCCTAATTGATAGACACGGCGTAAAGAGTGGTTTGGGAAA

GCCCAAAAATAAAGCTAAAGACCTTCTAAGCCGTCACACGCACGCCGAAGGCACGAAGCC

CTGACACGAAAGTAGCTTTACTATTACCCCCGACGCCACGAAAGCTAAGAAA

>860_Sample_ASV24

CACCGCGGTTATACGAAAGACCCTAGTTGATGGATCACGGCGTAAAGGGTGGTTAAGGGA

TATAAAAATAAAGCTAAAAATCCTCTTAGCTGTCATACGCTTTCCGAAGACATGAGGCCC

AATTACGAAAGTAGCTTTAAACCACCCTGACACCACGAAAGCCAAGAAA

>860_Sample_ASV25

CACCGCGGTTATACGAAAGACCCTAGTTTATAGATGCGGCGTAAAGGGTGGTTAAGGAAA

ACCTAAAATAATGCTAAAGACTCTCAAAGCTGTTATACGCTTCATGAGCACACGAAATCC

AAATACGAAAGTTGCCTTAATAAACTTTGCCTGACCCCACGAAAGCTGAAAAA

>860_Sample_ASV26

CACCGCGGTTATACGAAAGACCCCAGTTTATAGATGCGGCGTAAAGGGTGGTTAAGAGAA

AATCAAAATAAGGCCAAAGATCCCCTAAGCAGTCATACGCTCCACGAGGACACGAAACCC

AAATACGAAAGTTGCCTTAATAAACTCCACCTGACCCCACGAAAGCTAAGAAA

>860_Sample_ASV27

CACCGCGGTTATACGAAAGACCCCAGTTGATAGCCACGGCGTAAAGGGTGGTTAAGAGAA

AATCAAAATAAGGCCAAAGATCCCCTAAGCAGTCATACGCTCCACGAGGACACGAAACCC

AAATACGAAAGTTGCCTTAATAAACTTCACCTGATCCCACGAAAGCTAAGAAA

>860_Sample_ASV28

CACCGCGGTTATACGAAAGACCCCAGTTGATAGCCACGGCGTAAAGGGTGGTTAAGAGAA

AATCAAAATAAGGCCAAAGATCCCCTAAGCAGTCATACGCTCCACGAGGACACGAAACCC

AAATACGAAAGTTGCCTTAATAAACTTCACCTGATCCCACGAAAGCTGAAAAA

>860_Sample_ASV29

CACCGCGGTTATACGAAAGACCCCAGTTTATAGATGCGGCGTAAAGGGTGGTTAAGAGAA

AATCAAAATAAGGCCAAAGATCCCCTAAGCAGTCATACGCTCCACGAGGACACGAAACCC

AAATACGAAAGTTGCCTTAATAAACTTCACCTGATCCCACGAAAGCTGAAAAA

>860_Sample_ASV30

CACCGCGGTTATACGAAAGACCCCAGTTTATAGATGCGGCGTAAAGGGTGGTTAAGAGAA

AATCAAAATAAGGCCAAAGATCCCCTAAGCAGTCATACGCTCCACGAGGACACGAAACCC

AAATACGAAAGTTGCCTTAATAAACTTCACCTGATCCCACGAAAGCTAAGAAA

>860_Sample_ASV31

CACCGCGGTTATACGAGAGACCCTAGTTGATAGCTACGGCGTAAAGCGTGGTTAGGAGAA

CAAGATAAATAAAGTCAAACAATCTCTCGGCCGTTATACGTTATCTGAGAATATGAAGTC

CTACCACGAAAGTAACTTTAATTTTTCTGACCCCACGAAAACTGAGGAA

>860_Sample_ASV32

CACCGCGGTTAAACGAGAGGCCCTAGTTGATAGTACAACGGCGTAAAGGGTGGTTAAGGA

TAGTGAAATAATAAAGTCGAATGGCCCTTTGGCTGTCATACGCTTCTAGGAGTCCGAAGC

CCAATATACGAAAGTAACTTTAAGAGCGCCCACCTGACCCCACGAAAACTGAGAAA

>860_Sample_ASV33

CACCGCGGTTATACGAAAGACCCTAGTTGATAGGCACGGCGTAAAGGGTGGTTAGGGATA

TATTAAAAATAAAGTTAAAGAGCCTCTAAGCTGTCGCACGCATCCCGAGAGCTCGAGACC

CAGACACGAAAGTAACTTTAAAATACCTCACCTGACCCCACGAAAGCTAAGAAA

>860_Sample_ASV34

CACCGCGGTTATACGAAAGACCCTAGTTGATAGCCACGGCGTAAAGGGTGGTTAAGGTAT

ACAACAAATAAAGCTAAATAGCCTCTAGGCCGTCGCACGCATTCCGAGAGCTCGAAACCC

AAAAACGAAAGTAGCTTTAAAATAAACAGACCTGACCCCACGAAAGCTAAGAAA

>860_Sample_ASV35

CACCGCGGTTATACGAGAGGCCCAAGTTGATACTCCACGGCGTAAAGAGTGGTTAGAAAA

GACCTACTACTAAAGCCGAACGCCTTCAAAGCTGTTATACGCATCCGAAGGTGAGAAGCC

CACCCACGAAAGTGGCTTTACAACCTTGACTCCACGAAAGCTATGACA

>860_Sample_ASV36

CACCGCGGTTATACGAGAGGCCCAAGTTGATACTCCACGGCGTAAAGAGTGGTTAGAAAA

GACCTACTACTAAAGCCGAACGCCTTCAAAGCTGTTATACGCATCCGAAGGTGAGAAGCC

CACCCACGAAAGTGGCTTTACAACCTTGACTCCACGAAAGCTAAGAAA

>860_Sample_ASV37

CACCGCGGTTATACGAGAGACCCCAGTTGATAAACACGGCGTAAAGAGTGGTTAAGGGCA

CAAAACAAATAAAGTCAAACAACCTCTTAGCTGTTATACGCATTATGAGGATATGAAGCC

CCCCTACGAAAGTAACTTTACCAACCCCTGACCCCACGAAAGCTAAGAAA

>860_Sample_ASV38

CACCGCGGTTATACGAGAGACCCTAGTTGATATATACGGCGTAAAGAGTGGTTTGGAATA

ACCAAGTAATATAGCCAAAGACCTCCCAAGCCGTCACACGCACCCCGGAGGCACGAAGCC

CAAACACGAAAGTAGCTTTATTAACTTACCGACGCCACGAAAGCTAAGAAA

>860_Sample_ASV39

CACCGCGGTTATACGAAAGACCCTAGTTTATAGGCACGGCGTAAAGGGTGGTTAGGGGAT

AAACAAAATAAAGCCAAAGACCTTCTAAGTCGTCATACACTCATGAAGGCACGAAGCCCA

AATACGAAAGTAACTTTACAACTTAGACCCGACTCCACGAAAGCTGGGGGA

>860_Sample_ASV40

CACCGCGGTTATACGAGAGACCCTAATTGATAGACTACGGCGTAAAGAGTGGTTTAGAAC

AAAAATTAATAAAGCCAAAGATCCTCCTGGCCGTCGCACGCATTTCGAGGATACGAAGCC

CCACTACGAAAGTAGCTTTACTACAACTTTTCTGACCCCACGAAAGCTAAGAAA

>860_Sample_ASV41

CACCGCGGTTATACGAAAGACCCTAGTTGATAGATCACGGCGTAAAGGGTGGTTAAGGAG

AACAAAAATAAAGCTAAAAATCCTCTAAGCTGTCATACGCTTTCCGAAGACATGAGACCC

AACCACGAAAGTAACTTTAAACTTTCCTGACACCACGAAAGCCAAGAAA

>867_Sample_ASV1

CACCGCGGTTATACGAGAGGCCCAAGTTGACAGACATCGGCGTAAAGAGTGGTTAAGGAA

AACCTTCAACTAAAGTCGAACGCTCTCAAGACTGTCGTACGTTTCCGAGAGTATGAAGCC

CCACCACGAAAGTGACTTTACCTCACCTGACTCCACGAAAGCTGCGAAA

>867_Sample_ASV2

CACCGCGGTTATACGAAAGACCCTAGTTGATGGGTGCGGCGTAAAGGGTGGTTAGGGACT

ACAAAAAATAGAGCTAAAGATCCTCTAGGTCGTCATACACATCACGAGGAAACGAAGCCC

AAATACGAAAGTAGCTCTAAATATAATCCTGACGCCACGAAAGCCAAGAAA

>867_Sample_ASV3

CACCGCGGTTATACGAGAGACCCTAGTTGATTAAAGCGGCGTAAAGAGTGGTTATGGAAC

TATTCTTTTAAAGCAGAAAACCTCTCAAACTGTTATACGCACCCAGAGGTCTGAATCCCT

CACACGAAAGTGACTTTATTTATGCCTACCAGAACCCACGAAAGCTATGACA

>867_Sample_ASV4

CACCGCGGTTATACGAGAGACCCTAGTTGATTAAAGCGGCGTAAAGAGTGGTTATGGAAC

TATTCTTTTAAAGCAGAAAACCTCTCAAACTGTTATACGCACCCAGAGGTCTGAATCCCT

CACACGAAAGTGACTTTATTTATGCCTACCAGAACCCACGAAAGCTGGGACA

>867_Sample_ASV5

CACCGCGGTTATACGAAAGACCCTAGTTTATAGGTACGGCGTAAAGGGTGGTTAGGGGAC

AAACAAAATAAAGCCAAAGACCTCCTAAGCCGTCATACGCTCACGGAAGCACGAAGCCCA

AACACGAAAGTAGCTTTACCGAACATGCCCGACTCCACGAAAGCTGGGGAA

>867_Sample_ASV6

CACCGCGGTTATACGAGAGGCCCAAGTTGACAGACACCGGCGTAAAGAGTGGCTAGGGAA

AAATTACTACTAAAGCCGAACACCTTCAGAACTGTCATACGTTCCCGAAGATAAGAAGCC

CCACCACGAAAGTGACTTTATATTACCCGACCCCACGAAAGCTGTGAAA

>867_Sample_ASV7

CACCGCGGTTATACGAGAGGCCCAAGTTGACAGACACCGGCGTAAAGAGTGGCTAGGGAA

AAATTACTACTAAAGCCGAACACCTTCAGAACTGTCATACGTTCCCGAAGATAAGAAGCC

CCACCACGAAAGTGACTTTATATTACCCGACCCCACGAAAGCTAGGAAA

>867_Sample_ASV8

CACCGCGGTTATACGAGAGGCCCAAGTTGACAGACACCGGCGTAAAGAGTGGCTAGGGAA

AAATTACTACTAAAGCCGAACACCCTCAGAACTGTCATACGTTCCCGAAGGTAAGAAGCC

CCACCACGAAAGTGGCTTTATAATACCCGACTCCACGAAAGCTAGGAAA

>867_Sample_ASV9

CACCGCGGTTATACGAGAGGCCCAAGTTGACAGACACCGGCGTAAAGAGTGGCTAGGGAA

AAATTACTACTAAAGCCGAACACCCTCAGAACTGTCATACGTTCCCGAAGGTAAGAAGCC

CCACCACGAAAGTGGCTTTATAATACCCGACCCCACGAAAGCTAGGAAA

>867_Sample_ASV10

CACCGCGGTTATACGAGAGGCCCAAGTTGACAGACACCGGCGTAAAGAGTGGCTAGGGAA

AAATTACTACTAAAGCCGAACACCCTCAGAACTGTCATACGTTCCCGAAGGTAAGAAGCC

CCACCACGAAAGTGGCTTTATAATACCCGACCCCACGAAAGCTGTGAAA

>867_Sample_ASV11

CACCGCGGTTATACGAGAGGCCCAAGTTGACAGACACCGGCGTAAAGAGTGGCTAAGGAA

AAATTTCTACTAAAGCCGAACACCTTCAGAACTGTCATACGTTCCCGAAGATAAGAAGCC

CCACCACGAAAGTGGCTTTATATTACCCGACACCACGAAAGCTAGGAAA

>867_Sample_ASV12

CACCGCGGTTATACGAGAGGCCCAAGTTGACAGACACCGGCGTAAAGAGTGGCTAAGGAA

AAATTTCTACTAAAGCCGAACACCTTCAGAACTGTCATACGTTCCCGAAGATAAGAAGCC

CCACCACGAAAGTGGCTTTATATTACCCGACACCACGAAAGCTGTGAAA

>867_Sample_ASV13

CACCGCGGTTAAACGAGAGACCCTAGTTGATCATCACGGCGTAAAGAGTGGTTAGGGGAT

TATCATAAATAAAGCCAAAGACCTCCCAAGCTGTCGCACGCATTCCGGAGGCGCGAAGCC

CACACACGAAAGTAGCTTTAACTATTGACCCTGATGCCACGAAAGCTAAGAAA

>867_Sample_ASV14

CACCGCGGTTATACGAGAGGCCCAAGTTGATAAGCCCGGCGTAAAGAGTGGTTATGGAGA

ATAAAACACTAAAGCTAAAGACCCCCTAGGCTGTTATACGCACCTGGCGGCTCGAACCAC

CTATACGAAAGTAGCTTTACCCCCCTTCCACCAGAATCCACGACAGCTGGGGCA

>867_Sample_ASV15

CACCGCGGTTATACGAGAGGCCCAAGTTGACAGACATCGGCGTAAAGAGTGGTTAAGGAA

ACCCCATTACTAAAGCCGAACGCCCTCAGAACTGTCATACGTTCCCGAAGGTAAGAAGCC

CCACTACGAAAGTGGCTTTAACCACACCCGACCCCACGAAAGCTGTGAAA

>867_Sample_ASV16

CACCGCGGTTATACGAGAGGCCCAAGTTGATAGATATCGGCGTAAAGAGTGGTTAAGGAA

ACCCCGTTACTAAAGCCGAACGCCCTCAGAACTGTCATACGTTCCCGAAGGTAAGAAGCC

CCACTACGAAAGTGGCTTTAACCACACCCGACCCCACGAAAGCTGTGAAA

>867_Sample_ASV17

CACCGCGGTTATACGAGAGGCCCAAGTTGACAGACACCGGCGTAAAGAGTGGTTAAGGAA

ATTCATCAACTAAAGCCGAATGCCCTCAGGACTGTTATACGTTTGCGAAGGTAAGAAGCC

CTACTACGAAAGTGGCTTTATTCCACCTGATTCCACGAAAGCTGCGGAA

>867_Sample_ASV18

CACCGCGGTTATACGAGAGGCCCTAGTTGATAAATACGGCGTAAAGAGTGGTTAGAGGAC

TCCCATAATAAAGCCAAAGACCCCCCGGGCCGTCACACGCATACGAGGGCACGAAGTTCA

CACACGAAAGTAGCTTTAAATTACCCGACGCCACGAAAGCTAAGAAA

>867_Sample_ASV19

GACCGCGGTTATACGAGAGACTCTAGTTGATAGATACGGCGTAAAGAGTGGTTTGGGATA

AACTATAAATAAAGCCAAAGACCTTCCAAGCTGTTAAACGCATACCGAAGGCACGAAGCC

CCAACACGAAAGTAGCTTTACTACTACCCCCGATGCCACGAAAGCTAAGACA

>867_Sample_ASV20

CACCGCGGTTATACGAAAGACCCTAGTTTATAAGTACGGCGTAAAGGGTGGTTAGGGGAC

AAACAAAATAAAGCCAAAGACCTCCTAAGCCGTCATACGCTTACGGAGGCACGAAGCCCA

AACACGAAAGTAGCTTTACCGAACATGCCCGACTCCACGAAAGCTGGGAAA

>867_Sample_ASV21

CACCGCGGTTATACGAGAGACCCTAGTTGATAAATACGGCGTAAAGAGTGGTTATGGGAA

AAACAAATAAAGTCAAACAACCTCTTAGCTGTTATACGCATTATGAGAGCATGAAGCCCC

CTCACGAAAGTAACTTTAATACCTCCTGACCCCACGAAAGCTAAGAAA

>867_Sample_ASV22

AACCGCGGTTATACGAGAGACCCTAATTGATAGCTCACGGCGTAAAGGGTGGTTTGGGGA

AACCTCAAAATAAAGCTAAAGACCTTCTAAGCCGTTACACGCATACCGAAGGCACGAGGC

CCTAACACGAAAGTAGCTTTACTATTACCCCTGACGCCACGAAAGCTAAGAAA

>867_Sample_ASV23

CACCGCGGTTATACGAAAGACCCTAGTTGATGGATCACGGCGTAAAGGGTGGTTAAGGGA

TATAAAAATAAAGCTAAAAATCCTCTTAGCTGTCATACGCTTTCCGAAGACATGAGGCCC

AATTACGAAAGTAGCTTTAAACCACCCTGACACCACGAAAGCCAAGAAA

>867_Sample_ASV24

CACCGCGGTCATACGATTAACCCAAGTTAATAGGCACACGGCGTAAAGCGTGTTTAAGCA

CTATGCCAAATAAAGTTAAACTCCAATTAAGCTGTAAAAAGCCATAATCAAGACGAAAAT

AAATAACGAAAGTAACTTTACAACCGCTGAAACACGATAGCTAAGATC

>867_Sample_ASV25

CACCGCGGTTATACGAAAGACCCTAGTTTATAGACACGGCGTAAAGGGTGGTTCGGGACT

AAACAAAATAAAGCCAAAGACCTCCTAAGCCGTCATACGCTCACGGAAGCACGAAGCCCA

AGTACGAAAGTAGCTTTACAAAACATGCCTGACTCCACGAAAGCTAGGAAA

>867_Sample_ASV26

CACCGCGGTTATACGAAAGACCCTAGTTGATAGGCACGGCGTAAAGGGTGGTTAGGGATA

TATCAAAAATAAAGTTAAAGAGCCTCTAAGCTGTCGCACGCATCCCGAGAGCTCGAGACC

CAGACACGAAAGTAACTTTAAAATACCTCACCTGACCCCACGAAAGCTAAGAAA

>867_Sample_ASV27

CACCGCGGTTATACGAAAGACCCTAGTTGATAGCCACGGCGTAAAGGGTGGTTAAGGTAT

ACAACAAATAAAGCTAAATAGCCTCTAGGCCGTCGCACGCATTCCGAGAGCTCGAAACCC

AAAAACGAAAGTAGCTTTAAAATAAACAGACCTGACCCCACGAAAGCTAAGAAA

>867_Sample_ASV28

CACCGCGGTTATACGAGAGGCCCAAGTTGATACTCCACGGCGTAAAGAGTGGTTAGAAAA

GACCTACTACTAAAGCCGAACGCCTTCAAAGCTGTTATACGCATCCGAAGGTGAGAAGCC

CACCCACGAAAGTGGCTTTACAACCTTGACTCCACGAAAGCTATGACA

>867_Sample_ASV29

CACCGCGGTTATACGAGAGGCCCAAGTTGATACTCCACGGCGTAAAGAGTGGTTAGAAAA

GACCTACTACTAAAGCCGAACGCCTTCAAAGCTGTTATACGCATCCGAAGGTGAGAAGCC

CACCCACGAAAGTGGCTTTACAACCTTGACTCCACGAAAGCTGGGACA

>867_Sample_ASV30

CACCGCGGTTATACGAGAGACCCCAGTTGATAAACACGGCGTAAAGAGTGGTTAAGGGCA

CAAAACAAATAAAGTCAAACAACCTCTTAGCTGTTATACGCATTATGAGGATATGAAGCC

CCCCTACGAAAGTAACTTTACCAACCCCTGACCCCACGAAAGCTAAGAAA

>867_Sample_ASV31

CACCGCGGTTATACGAAAGACCCTAGTTTATAGGCACGGCGTAAAGGGTGGTTAGGGGAT

AAACAAAATAAAGCCAAAGACCTTCTAAGTCGTCATACACTCATGAAGGCACGAAGCCCA

AATACGAAAGTAACTTTACAACTTAGACCCGACTCCACGAAAGCTGGGGGA

>867_Sample_ASV32

CACCGCGGTTATACGAGAGACCCTAATTGATAGACTACGGCGTAAAGAGTGGTTTAGAAC

AAAAATTAATAAAGCCAAAGATCCTCCTGGCCGTCGCACGCATTTCGAGGATACGAAGCC

CCACTACGAAAGTAGCTTTACTACAACTTTTCTGACCCCACGAAAGCTAAGAAA

>867_Sample_ASV33

CACCGCGGTTATACGAAAGACCCTAGTTGATAGATCACGGCGTAAAGGGTGGTTAAGGAG

AACAAAAATAAAGCTAAAAATCTTCTAAGCTGTCATACGCTTTCCGAAGACATGAGACCC

AACCACGAAAGTAGCTTTAAACTTTCCTGACACCACGAAAGCCAAGAAA

>868_Sample_ASV1

CACCGCGGTTATACGAAAGACCCTAGTTGATGGGTGCGGCGTAAAGGGTGGTTAGGGACT

ACAAAAAATAGAGCTAAAGATCCTCTAGGTCGTCATACACATCACGAGGAAACGAAGCCC

AAATACGAAAGTAGCTCTAAATATAATCCTGACGCCACGAAAGCCAAGAAA

>868_Sample_ASV2

CACCGCGGTTATACGAAAGACCCTAGTTGATGGGTGCGGCGTAAAGGGTGGTTAGGGACT

ACAAAAAATAGAGCTAAAGATCCTCTAGGTCGTCATACACATCACGAGAAAACGAAGCCC

AAATACGAAAGTAGCTCTAAATATAATCCTGACGCCACGAAAGCCAAGAAA

>868_Sample_ASV3

CACCGCGGTTATACGAAAGACCCTAGTTGATAACTACGGCATAAAGGGTGGTTAGGGAGT

TTAAAAAATAAAGCCAAAGCTCTTCTAAGCTGTCATACGCTCTCTGAAAGAACGAGGCCC

AAACACGAAAGTAGCTTTAAATAAATAACCTGATACCACGAAAGCCAAGAAA

>868_2_Sample_ASV1

CACCGCGGTTATACGAAAGACCCTAGTTGATAACTACGGCATAAAGGGTGGTTAGGGAGT

TTAAAAAATAAAGCCAAAGCTCTTCTAAGCTGTCATACGCTCTCTGAAAGAACGAGGCCC

AAACACGAAAGTAGCTTTAAATAAATAACCTGATACCACGAAAGCCAAGAAA

>868_Sample_ASV4

CACCGCGGTTATACGAGAGACCCTAGTTGATTAAAGCGGCGTAAAGAGTGGTTATGGAAC

TATTCTTTTAAAGCAGAAAACCTCTCAAACTGTTATACGCACCCAGAGGTCTGAATCCCT

CACACGAAAGTGACTTTATTTATGCCTACCAGAACCCACGAAAGCTGGGACA

>868_Sample_ASV5

CACCGCGGTTATACGAGAGACCCTAGTTGATTAAAGCGGCGTAAAGAGTGGTTATGGAAC

TATTCTTTTAAAGCAGAAAACCTCTCAAACTGTTATACGCACCCAGAGGTCTGAATCCCT

CACACGAAAGTGACTTTATTTATGCCTACCAGAACCCACGAAAGCTGGGGGA

>868_2_Sample_ASV2

CACCGCGGTTATACGAGAGACCCTAGTTGATTAAAGCGGCGTAAAGAGTGGTTATGGAAC

TATTCTTTTAAAGCAGAAAACCTCTCAAACTGTTATACGCACCCAGAGGTCTGAATCCCT

CACACGAAAGTGACTTTATTTATGCCTACCAGAACCCACGAAAGCTGGGACA

>868_2_Sample_ASV3

CACCGCGGTTATACGAAAGACCCTAGTTTATAGGTACGGCGTAAAGGGTGGTTAGGGGAC

AAACAAAATAAAGCCAAAGACCTCCTAAGCCGTCATACGCTCACGGAAGCACGAAGCCCA

AACACGAAAGTAGCTTTACCGAACATGCCCGACTCCACGAAAGCTGGGGAA

>868_2_Sample_ASV4

CACCGCGGTTATACGAGAGACCCTAATTAATAATTACGGCGTAAAGAGTGGTTAAAAGTT

AATATAAATAAAGCCAAAGATTTTCCAAGCTGTTATACGCACTCGAAAACATGAAGCCTT

ATAACGAAAGTAGCTTTACAAACCTTTTGACCCCACGAAAGCTATGATA

>868_Sample_ASV6

CACCGCGATTATACGAGAGGCCCAAGTTGACAGACATCGGCGTAAAGAGTGGTTAAGGAA

AAATTACTACTAAAGCCGAACACCTTCAGAACTGTCATACGTTCCCGAAGATAAGAAGCC

CCACCACGAAAGTGACTTTATATTACCCGACCCCACGAAAGCTGTGAAA

>868_Sample_ASV7

CACCGCGGTTATACGAGAGGCCCAAGTTGACAGACACCGGCGTAAAGAGTGGCTAGGGAA

AAATTACTACTAAAGCCGAACACCTTCAGAACTGTCATACGTTCCCGAAGATAAGAAGCC

CCACCACGAAAGTGACTTTATATTACCCGACCCCACGAAAGCTGGGGGA

>868_Sample_ASV8

CACCGCGGTTATACGAGAGGCCCAAGTTGACAGACACCGGCGTAAAGAGTGGCTAGGGAA

AAATTACTACTAAAGCCGAACACCTTCAGAACTGTCATACGTTCCCGAAGATAAGAAGCC

CCACCACGAAAGTGACTTTATATTACCCGACCCCACGAAAGCTGTGAAA

>868_2_Sample_ASV5

CACCGCGGTTATACGAGAGGCCCAAGTTGACAGACACCGGCGTAAAGAGTGGCTAGGGAA

AAATTACTACTAAAGCCGAACACCTTCAGAACTGTCATACGTTCCCGAAGATAAGAAGCC

CCACCACGAAAGTGACTTTATATTACCCGACCCCACGAAAGCTAGGAAA

>868_2_Sample_ASV6

CACCGCGGTTATACGAGAGGCCCAAGTTGACAGACACCGGCGTAAAGAGTGGCTAGGGAA

AAATTACTACTAAAGCCGAACACCTTCAGAACTGTCATACGTTCCCGAAGATAAGAAGCC

CCACCACGAAAGTGACTTTATATTACCCGACCCCACGAAAGCTGTGAAA

>868_2_Sample_ASV7

CACCGCGGTTATACGAGAGGCCCAAGTTGACAGACACCGGCGTAAAGAGTGGCTAGGGAA

AAATTACTACTAAAGCCGAACACCTTCAGAACTGTCATACGTTCCCGAAGATAAGAAGCC

CCACCACGAAAGTGACTTTATATTACCCGACTCCACGAAAGCTAGGAAA

>868_Sample_ASV9

CACCGCGGTTATACGAGAGGCCCAAGTTGACAGACACCGGCGTAAAGAGTGGCTAGGGAA

AAATTACTACTAAAGCCGAACACCTTCAGAACTGTCATACGTTCCCGAAGATAAGAAGCC

CCACCACGAAAGTGGCTTTATATTACCCGACCCCACGAAAGCTGTGAAA

>868_Sample_ASV10

CACCGCGGTTATACGAGAGGCCCAAGTTGACAGACACCGGCGTAAAGAGTGGCTAGGGAA

AAATTACTACTAAAGCCGAACACCTTCAGAACTGTCATACGTTCCCGAAGATAAGAAGCC

CCACCACGAAAGTGGCTTTATATTACCTGACCCCACGAAAGCTGTGAAA

>868_Sample_ASV11

CACCGCGGTTATACGAGAGGCCCAAGTTGACAGACACCGGCGTAAAGAGTGGCTAGGGAA

AAATTACTACTAAAGCCGAACACCCTCAGAACTGTCATACGTTCCCGAAGGTAAGAAGCC

CCACCACGAAAGTGGCTTTATAATACCCGACCCCACGAAAGCTGGGGGA

>868_Sample_ASV12

CACCGCGGTTATACGAGAGGCCCAAGTTGACAGACACCGGCGTAAAGAGTGGCTAGGGAA

AAATTACTACTAAAGCCGAACACCTTCAGAACTGTCATACGTTCCCGAAGATAAGAAGCC

CCACCACGAAAGTGGCTTTATATTACCCGACTCCACGAAAGCTGGGGGA

>868_Sample_ASV13

CACCGCGGTTATACGAGAGGCCCAAGTTGACAGACACCGGCGTAAAGAGTGGCTAGGGAA

AAATTACTACTAAAGCCGAACACCTTCAGAACTGTCATACGTTCCCGAAGATAAGAAGCC

CCACCACGAAAGTGGCTTTATATTACCCGACCCCACGAAAGCTGGGGGA

>868_Sample_ASV14

CACCGCGGTTATACGAGAGGCCCAAGTTGACAGACACCGGCGTAAAGAGTGGCTAGGGAA

AAATTACTACTAAAGCCGAACACCCTCAGAACTGTCATACGTTCCCGAAGGTAAGAAGCC

CCACCACGAAAGTGGCTTTATAATACCCGACCCCACGAAAGCTGTGAAA

>868_2_Sample_ASV8

CACCGCGGTTATACGAGAGGCCCAAGTTGACAGACACCGGCGTAAAGAGTGGCTAGGGAA

AAATTACTACTAAAGCCGAACACCTTCAGAACTGTCATACGTTCCCGAAGATAAGAAGCC

CCACCACGAAAGTGGCTTTATATTACCCGACCCCACGAAAGCTGTGAAA

>868_Sample_ASV15

CACCGCGGTTATACGAGAGGCCCAAGTTGACAGACACCGGCGTAAAGAGTGGCTAAGGAA

AAATTTCTACTAAAGCCGAACACCTTCAGAACTGTCATACGTTCCCGAAGAAAAGAAGCC

CCACCACGAAAGTGGCTTTATATTACCCGACCCCACGAAAGCTGTGAAA

>868_Sample_ASV16

CACCGCGGTTATACGAGAGGCCCAAGTTGACAGACACCGGCGTAAAGAGTGGCTAAGGAA

AAATTTCTACTAAAGCCGAACACCTTCAGAACTGTCATACGTTCCCGAAGATAAGAAGCC

CCACCACGAAAGTGGCTTTATATTACCCGACACCACGAAAGCTGTGAAA

>868_2_Sample_ASV9

CACCGCGGTTATACGAGAGACCCTAGTTGATATATACGGCGTAAAGAGTGGTTTGGGGTT

ATGCCTCCAAAATAAAGCCGAAGATCTCCCAAGCCGTCACACGCATCCCGGAGACACGAA

GCCCTAATACGAAGGTAGCTTTATTAATTCCCCGACGCCACGAAAGCTAAGAAA

>868_Sample_ASV17

CACCGCGGTTATACGAGAGGCCCAAGTTGATAAGCCCGGCGTAAAGAGTGGTTATGGAGA

ATAAAACACTAAAGCTAAAGACCCCCTAGGCTGTTATACGCACCTGGCGGCTCGAACCAC

CTATACGAAAGTAGCTTTACCCCCCTTCCACCAGAATCCACGACAGCTGGGGCA

>868_2_Sample_ASV10

CACCGCGGTTATACGAGAGGCCCAAGTTGATAAGCCCGGCGTAAAGAGTGGTTATGGAGA

ATAAAACACTAAAGCTAAAGACCCCCTAGGCTGTTATACGCACCTGGCGGCTCGAACCAC

CTATACGAAAGTAGCTTTACCCCCCTTCCACCAGAATCCACGACAGCTGGGGCA

>868_2_Sample_ASV11

CACCGCGGTTATACGAGAGGCCCTAGTTGATAGCCACGGCGCAAAGAGTGGTTAAGGAGC

CCCACTAAATAAAGTCGAACACTTCCTAGGCCGTTATACGTTTTCTAGAAGCACGAAACC

CAATTACACGAAAGCAACTTTATACTAAACAACCTGACCCCACGAAAGCTAAGAAA

>868_Sample_ASV18

CACCGCGGTTATACGAGAGGCCCTAGTTGATAGCCACGGCGCAAAGAGTGGTTAAGGAGC

CCCACTAAATAAAGTCGAACACTTCCTAGGCCGTTATACGTTTTCTAGAAGCACGAAACC

CAATTACACGAAAGCAACTTTATACTAAACAACCTGACCCCACGAAAGCTAAGAAA

>868_Sample_ASV19

CACCGCGGTTATACGAGAGGCCCTAGTTGATAGCTACGGCGCAAAGAGTGGTTAAGGAAC

TCACCCAAATAAAGTCAAACACTTCCTAGGCCGTTATACGTTTTCTAGAAGCACGAAGCC

CAATTACACGAAAGCAACTTTATTCTACACCTGACCCCACGAAAGCTAAGAAA

>868_2_Sample_ASV12

CACCGCGGTTATACGAGAGGCCCAAGTTGACAGACATCGGCGTAAAGAGTGGTTAAGGAA

ACCCCATTACTAAAGCCGAACGCCCTCAGAACTGTCATACGTTCCCGAAGGTAAGAAGCC

CCACTACGAAAGTGGCTTTAACCACACCCGACCCCACGAAAGCTGTGAAA

>868_Sample_ASV20

CACCGCGGTTATACGAGAGGCCCAAGTTGACAGACATCGGCGTAAAGAGTGGTTAAGGAA

ACCCCATTACTAAAGCCGAACGCCCTCAGAACTGTCATACGTTCCCGAAGGTAAGAAGCC

CCACTACGAAAGTGGCTTTAACCACACCCGACCCCACGAAAGCTGTGAAA

>868_2_Sample_ASV13

CACCGCGGTTATACGAGAGGCCCAAGTTGATAGATATCGGCGTAAAGAGTGGTTAAGGAA

ACCCCGTTACTAAAGCCGAACGCCCTCAGAACTGTCATACGTTCCCGAAGGTAAGAAGCC

CCACTACGAAAGTGGCTTTAACCACACCCGACCCCACGAAAGCTGTGAAA

>868_Sample_ASV21

CACCGCGGTTATACGAGAGGCCCAAGTTGACAGACACCGGCGTAAAGAGTGGTTAAGGAA

ATTCATCAACTAAAGCCGAATGCCCTCAGGACTGTTATACGTTTGCGAAGGTAAGAAGCC

CTACTACGAAAGTGGCTTTATTCCACCTGATTCCACGAAAGCTGGGGGA

>868_Sample_ASV22

CACCGCGGTTATACGAGAGGCCCAAGTTGACAGACACCGGCGTAAAGAGTGGTTAAGGAA

ATTCATCAACTAAAGCCGAATGCCCTCAGGACTGTTATACGTTTGCGAAGGTAAGAAGCC

CTACTACGAAAGTGGCTTTATTCCACCTGATTCCACGAAAGCTGCGGAA

>868_2_Sample_ASV14

CACCGCGGTTATACGAGAGGCCCAAGTTGACAGACACCGGCGTAAAGAGTGGTTAAGGAA

ATTCATCAACTAAAGCCGAATGCCCTCAGGACTGTTATACGTTTGCGAAGGTAAGAAGCC

CTACTACGAAAGTGGCTTTATTCCACCTGATTCCACGAAAGCTGCGGAA

>868_Sample_ASV23

GACCGCGGTTATACGAGAGACTCTAGTTGATAGATACGGCGTAAAGAGTGGTTTGGGATA

AACTATAAATAAAGCCAAAGACCTTCCAAGCTGTTAAACGCATACCGAAGGCACGAAGCC

CCAACACGAAAGTAGCTTTACTACTACCCCCGATGCCACGAAAGCTAAGACA

>868_Sample_ASV24

GACCGCGGTTATACGAGAGACTCTAGTTGATAGATACGGCGTAAAGAGTGGTTTGGGATA

AACTATAAATAAAGCCAAAGACCTTCCAAGCTGTTAAACGCATACCGAAGGCACGAAGCC

CCAACACGAAAGTAGCTTTACTACTACCCCCGATGCCACGAAAGCTGGGGGA

>868_Sample_ASV25

CACCGCGGTTATACGAAAGACCCCAGTTTATAGACACGGCGTAAAGGGTGGTTATAGGAC

AAACTAACATAGAGCCAAAGACCCCCTAAGCCGTCATACGCTCACGAGGGCTCGAAGCCC

CTATACGAAAGTAGCTCTACCAAAAAATGCTCGACTCCACGAAAGCTGGGACA

>868_2_Sample_ASV15

CACCGCGGTTATACGAGAGGCCCCAGTTGATAAACACGGCGTAAAGGGTGGTTAAGGGAG

CATAAGAATAAAGCCGAAGGGCCCTTTGGCCGTTATACGCTTCTAGGTGTCCGAAGCCCA

AACACGAAAGTAGCTTTAGTTAAGCCCACCTGACTCCACGAAAACTGAGAAA

>868_2_Sample_ASV16

CACCGCGGTTATACGAGAGACCCTAGTTGATAAATACGGCGTAAAGAGTGGTTATGGGAA

AAACAAATAAAGTCAAACAACCTCTTAGCTGTTATACGCATTATGAGAGCATGAAGCCCC

CTCACGAAAGTAACTTTAATACCTCCTGACCCCACGAAAGCTAAGAAA

>868_2_Sample_ASV17

CACCGCGGTTATACGAGAGGCCCAAGTTGACAAACCCCGGCGTAAAGAGTGGTTAAGGGA

GATCAAAACTAAAGCCGAATGCTTTCAAAGCTGTTATACGCTTCCGAAAGTAAGAAGCTC

AATCACGAAAGTGGCTTTACTTTACCTGACCCCACGAAAGCTACGACA

>868_2_Sample_ASV18

CACCGCGGTTATACGAGAGGCTCAAGTTGATGAACCCCGGCGTAAAGAGTGGTTAAGGGA

GATCAAAACTAAAGCCGAATGCTTTCAAAGCTGTTATACGCTTCCGAAAGTAAGAAGCTC

AATCACGAAAGTGGCTTTACTTTACCTGACCCCACGAAAGCTACGACA

>868_Sample_ASV26

CACCGCGGTTATACGAGAGACCCTAATTGATAGACACGGCGTAAAGAGTGGTTTGGGAAA

GCCCAAAAATAAAGCTAAAGACCTTCTAAGCCGTCACACGCACGCCGAAGGCACGAAGCC

CTGACACGAAAGTAGCTTTACTATTACCCCCGACGCCACGAAAGCTAAGAAA

>868_Sample_ASV27

AACCGCGGTTATACGAGAGACCCTAATTGATAGCTCACGGCGTAAAGGGTGGTTTGGGGA

AACCTCAAAATAAAGCTAAAGACCTTCTAAGCCGTTACACGCATACCGAAGGCACGAGGC

CCTAACACGAAAGTAGCTTTACTATTACCCCTGACGCCACGAAAGCTAAGAAA

>868_Sample_ASV28

CACCGCGGTTATACGAGAGACCCTAATTGATAGGCACGGCGTAAAGAGTGGTTTAGGGTA

ACCCATAAATAAAGCTAAAGACCTTCTAAGCCGTCACACGCACACCGAAGGCACGAGACC

CCAACACGAAAGTAGCTTTACTTCTACCCCTGACGCCACGAAAGCTAAGAAA

>868_2_Sample_ASV19

CACCGCGGTTATACGAAAGACCCTAGTTGATGGATCACGGCGTAAAGGGTGGTTAAGGGA

TATAAAAATAAAGCTAAAAATCCTCTTAGCTGTCATACGCTTTCCGAAGACATGAGGCCC

AATTACGAAAGTAGCTTTAAACCACCCTGACACCACGAAAGCCAAGAAA

>868_Sample_ASV29

CACCGCGGTTATACGAAAGACCCTAGTTGATGGATCACGGCGTAAAGGGTGGTTAAGGGA

TATAAAAATAAAGCTAAAAATCCTCTTAGCTGTCATACGCTTTCCGAAGACATGAGGCCC

AATTACGAAAGTAGCTTTAAACCACCCTGACACCACGAAAGCCAAGAAA

>868_2_Sample_ASV20

CACCGCGGTTATACGAAAGACCCCAGTTTATAGATGCGGCGTAAAGGGTGGTTAAGAGAA

AATCAAAATAAGGCCAAAGATCCCCTAAGCAGTCATACGCTCCACGAGGACACGAAACCC

AAATACGAAAGTTGCCTTAATAAACTTCACCTGATCCCACGAAAGCTGAAAAA

>868_2_Sample_ASV21

CACCGCGGTTATACGAGAGGCCCAAGTTGACAAACCCCGGCGTAAAGAGTGGTTAAGGAA

AATTTAAAACTAAAGCCGAATGCCCCCAAAGCTGTTATACGCACCCGAGGGTAAGAAGCC

CAATCACGAAAGTGGCTTTACACTACCTGAACCCACGAAAGCTATGAAA

>868_2_Sample_ASV22

CACCGCGGTTATACGAGAGGCCCAAGTTGACAAACCCCGGCGTAAAGAGTGGTTAAGGAA

AATTTAAAACTAAAGCCGAATGCCCCCAAAGCTGTTATACGCACCCGAGGATAAGAAGCC

CAATCACGAAAGTGGCTTTACACTACCTGAACCCACGAAAGCTATGAAA

>868_2_Sample_ASV23

CACCGCGGTTATACGAGAGGCCCTAGTTGATAACTACCGGCGTAAAGAGTGGTTATGGAA

AAATATTTAATAAAGCCGAACACCCCCTCAGCCGTCATACGCACCTGGGAGCACGAAGAC

CTACTGCGAAAGCAGCTTTAATTATGCCTGACCCCACGACAGCTAAGAAA

>868_Sample_ASV30

CACCGCGGTTATACGAGAGGCCCTAGTTGATAACTACCGGCGTAAAGAGTGGTTATGGAA

AAATATTTAATAAAGCCGAACACCCCCTCAGCCGTCATACGCACCTGGGAGCACGAAGAC

CTACTGCGAAAGCAGCTTTAATTATGCCTGACCCCACGACAGCTAAGAAA

>868_Sample_ASV31

CACCGCGGTTATACGAAAGACCCTAGTTTATAGGCACGGCGTAAAGGGTGGTTAGGGGAT

AAACAAAATAAAGCCAAAGACCTCCTAAGCCGTCATACGCTCACGGAAGCACGAAGCCCA

AGTACGAAAGTAGCTTTACAAAACATGCCTGACTCCACGAAAGCTAGGAAA

>868_Sample_ASV32

CACCGCGGTTATACGAAAGACCCTAGTTTATAGACACGGCGTAAAGGGTGGTTCGGGACT

AAACAAAATAAAGCCAAAGACCTCCTAAGCCGTCATACGCTCACGGAAGCACGAAGCCCA

AGTACGAAAGTAGCTTTACAAAACATGCCTGACTCCACGAAAGCTAGGAAA

>868_Sample_ASV33

CACCGCGGTTATACGAAAGACCCTAGTTTATAGACACGGCGTAAAGGGTGGTTCGGGACT

AAACAAAATAAAGCCAAAGACCTCCTAAGCCGTCATACGCTCACGGAAGCACGAAGCCCA

AGTACGAAAGTAGCTTTACAAAACATGCCTGACTCCACGAAAGCTGGGGGA

>868_2_Sample_ASV24

CACCGCGGTTATACGAAAGACCCTAGTTTATAGACACGGCGTAAAGGGTGGTTCGGGACT

AAACAAAATAAAGCCAAAGACCTCCTAAGCCGTCATACGCTCACGGAAGCACGAAGCCCA

AGTACGAAAGTAGCTTTACAAAACATGCCTGACTCCACGAAAGCTAGGAAA

>868_Sample_ASV34

CACCGCGGTTATACGAGAGGCCCAAGTTGATTATTACCGGCGTAAAGCGTGGTTAAGATT

TTTATAGGACTAAAGCCGAACCCCCTCAGAGCTGTTATACGCACCCGAAGGTTAGAAGTT

CAATCACGAAAGTGGCTTTACAACCCCGAACCCACGAAAGCTAAGATA

>868_Sample_ASV35

CACCGCGGTTATACGAGAGACCCTAATTGATAGGCGCGGCGTAAAGAGTGGTTTAGGACT

AAAACTAATAAAGCTAAAGATCCTCCCAGCCGTCGCACGCATTCTGAGGACACGAAACTC

AACCACGAAAGTAGCTTTAACATACTCTCCTGACCCCACGAAAGCTAAGAAA

>868_Sample_ASV36

CACCGCGGTTATACGAAAGACCCTAGTTGATAGGCACGGCGTAAAGGGTGGTTAGGGATA

TATCAAAAATAAAGTTAAAGAGCCTCTAAGCTGTCGCACGCATCCCGAGAGCTCGAGACC

CAGACACGAAAGTAACTTTAAAATACCTCACCTGACCCCACGAAAGCTAAGAAA

>868_2_Sample_ASV25

CACCGCGGTTATACGAGAGGCCCAAGTTGATACTCCACGGCGTAAAGAGTGGTTAGAAAA

GACCTACTACTAAAGCCGAACGCCTTCAAAGCTGTTATACGCATCCGAAGGTGAGAAGCC

CACCCACGAAAGTGGCTTTACAACCTTGACTCCACGAAAGCTATGACA

>868_Sample_ASV37

CACCGCGGTTATACGAGAGGCCCAAGTTGATACTCCACGGCGTAAAGAGTGGTTAGAAAA

GACCTACTACTAAAGCCGAACGCCTTCAAAGCTGTTATACGCATCCGAAGGTGAGAAGCC

CACCCACGAAAGTGGCTTTACAACCTTGACTCCACGAAAGCTATGACA

>868_2_Sample_ASV26

CACCGCGGTTATACGAGAGACCCCAGTTGATAAACACGGCGTAAAGAGTGGTTAAGGGCA

CAAAACAAATAAAGTCAAACAACCTCTTAGCTGTTATACGCATTATGAGGATATGAAGCC

CCCCTACGAAAGTAACTTTACCAACCCCTGACCCCACGAAAGCTAAGAAA

>868_Sample_ASV38

CACCGCGGTTATACGAAAGACCCTAGTTTATAGGCACGGCGTAAAGGGTGGTTAGGGGAT

AAACAAAATAAAGCCAAAGACCTTCTAAGTCGTCATACACTCATGAAGGCACGAAGCCCA

AATACGAAAGTAACTTTACAACTTAGACCCGACCCCACGAAAGCTGTGAAA

>868_Sample_ASV39

CACCGCGGTTATACGAAAGACCCTAGTTTATAGGCACGGCGTAAAGGGTGGTTAGGGGAT

AAACAAAATAAAGCCAAAGACCTTCTAAGTCGTCATACACTCATGAAGGCACGAAGCCCA

AATACGAAAGTAACTTTACAACTTAGACCCGACTCCACGAAAGCTGGGGGA

>868_2_Sample_ASV27

CACCGCGGTTATACGAAAGACCCTAGTTTATAGGCACGGCGTAAAGGGTGGTTAGGGGAT

AAACAAAATAAAGCCAAAGACCTTCTAAGTCGTCATACACTCATGAAGGCACGAAGCCCA

AATACGAAAGTAACTTTACAACTTAGACCCGACTCCACGAAAGCTGGGGGA

>868_Sample_ASV40

CACCGCGGTTATACGAGAGACCCTAATTAATAGATACGGCGTAAAGAGTGGTTTAGGACA

AAAATTAATAAAGCCGAAGTTTCTCCTAGCCGTTGTACGCACTCTGAGAACACGAAGCCC

CACTACGAAAGTAGCTTTACCTATTATTTCCTGACCCCACGAAAGCTAAGAAA

>868_Sample_ASV41

CACCGCGGTTATACGAGAGACCCTAATTAATAGCTACGGCGTAAAGAGTGGTTTAGGGTA

AAAATTAATAAAGCCGAAGTTTCTCCTAGCCGTTGTACGCACTCTGAGAACACGAAGCCC

CACTACGAAAGTAGCTTTACCTATTATTTCCTGACCCCACGAAAGCTAAGAAA

>868_Sample_ASV42

CACCGCGGTTATACGAGAGACCCTAATTAATAGCTACGGCGTAAAGAGTGGTTTAGGACA

AAAATTAATAAAGCCGAAGTTTCTCCTAGCCGTTGTACGCACTCTGAGAACACGAAGCCC

CACTACGAAAGTAGCTTTACCTATTATTTCCTGACCCCACGAAAGCTAAGAAA

>868_Sample_ASV43

CACCGCGGTTATACGAGAGACCCTAATTAATAGATACGGCGTAAAGAGTGGTTTAGGGTA

AAAATTAATAAAGCCGAAGTTTCTCCTAGCCGTTGTACGCACTCTGAGAACACGAAGCCC

CACTACGAAAGTAGCTTTACCTATTATTTCCTGACCCCACGAAAGCTAAGAAA

>868_Sample_ASV44

CACCGCGGTTATACGAGAGACCCTAATTAATAGATACGGCGTAAAGAGTGGTTTAGGACA

AAAATTAATAAAGCCGAAGTTTCTCCTAGCCGTTGTACGCACTCTGAGAACACGAAGCCC

CACTACGAAAGTAGCTTTACCAATAATCTCCTGACCCCACGAAAGCTAAGAAA

>868_Sample_ASV45

CACCGCGGTTATACGAAAGACCCTAGTTGATAGACCACGGCGTAAAGGGTGGTTAAGGAA

CACAAAAATAAAGCTAAAGATCCTCTAAGCTGTCATACGCTTCCCGAAGACATGAAACCC

AATCACGAAAGTAGCTTTAAACTGTCCTGACACCACGAAAGCCAAGAAA

>868_2_Sample_ASV28

CACCGCGGTTATACGAAAGACCCTAGTTGATAGACCACGGCGTAAAGGGTGGTTAAGGAA

CACAAAAATAAAGCTAAAGATCCTCTAAGCTGTCATACGCTTCCCGAAGACATGAAACCC

AATCACGAAAGTAGCTTTAAACTGTCCTGACACCACGAAAGCCAAGAAA

>868_Sample_ASV46

CACCGCGGTTATACGAAAGACCCTAGTTGATAGATCACGGCGTAAAGGGTGGTTAAGGAA

AACAAAAATAAAGCTAAAAATCCTCTAAGCTGTCATACGCTTTCCGAAGACATGAGATCC

AACCACGAAAGTAGCTTTAAAATTTCCTGACACCACGAAAGCCAAGGAA

>872_Sample_ASV1

CACCGCGGTTATACGAGAGACCCTAGTTGATTAAAGCGGCGTAAAGAGTGGTTATGGAAC

TATTCTTTTAAAGCAGAAAACCTCTCAAACTGTTATACGCACCCAGAGGTCTGAATCCCT

CACACGAAAGTGACTTTATTTATGCCTACCAGAACCCACGAAAGCTGGGACA

>872_Sample_ASV2

CACCGCGGTTATACGAAAGACCCTAGTTTATAGGTACGGCGTAAAGGGTGGTTAGGGGAC

AAACAAAATAAAGCCAAAGACCTCCTAAGCCGTCATACGCTCACGGAAGCACGAAGCCCA

AACACGAAAGTAGCTTTACCGAACATGCCCGACTCCACGAAAGCTGGGGAA

>872_Sample_ASV3

CACCGCGGTTATACGAGAGGCCCAAGTTGACAGACACCGGCGTAAAGAGTGGCTAGGGAA

AAATTACTACTAAAGCCGAACACCTTCAGAACTGTCATACGTTCCCGAAGATAAGAAGCC

CCACCACGAAAGTGGCTTTATATTACCCGACCCCACGAAAGCTGTGAAA

>872_Sample_ASV4

CACCGCGATTATACGAGAGGCCCAAGTTGACAGACATCGGCGTAAAGAGTGGTTAAGGAA

AAATTACTACTAAAGCCGAACACCTTCAGAACTGTCATACGTTCCCGAAGATAAGAAGCC

CCACCACGAAAGTGGCTTTATATTACCCGACCCCACGAAAGCTGTGAAA

>872_Sample_ASV5

CATGGCGATTATACGAGAGGCCCAAGTTGACAGACACTGGCGTAAAGAGTGGCTAAGGAA

AAACTACTACTAAAGCCGAACACCTTCAGAACTGTCATACGTTCCCGAAGATAAGAAGCC

CCACCACGAAAGTGGCTTTATATTACCCGACCCCACGAAAGCTGTGAAA

>872_Sample_ASV6

CATGGCGATTATACGAGAGGCCCAAGTTGACAGACACTGGCGTAAAGAGTGGCTAAGGAA

AAATTACTACTAAAGCCGAACACCTTCAGAACTGTCATACGTTCCCGAAGATAAGAAGCC

CCACCACGAAAGTGGCTTTATATTACCCGACCCCACGAAAGCTGTGAAA

>872_Sample_ASV7

CACCGCGGTTATACGAGAGGCCCAAGTTGACAGACACCGGCGTAAAGAGTGGCTAAGGAA

AAATTTCTACTAAAGCCGAACACCTTCAGAACTGTCATACGTTCCCGAAGAAAAGAAGCC

CCACCACGAAAGTGGCTTTATATTACCCGACCCCACGAAAGCTGTGAAA

>872_Sample_ASV8

CACCGCGGTTATACGAGAGACCCTAGTTGATATATACGGCGTAAAGAGTGGTTTGGGATA

TTTCTCCAAAATAAAGCCGAAGATCTCCCAAGCCGTCACACGCATCCCGGAGACACGAAG

CCCTAATACGAAGGTAGCTTTATTAATTCCCCGACGCCACGAAAGCTAAGAAA

>872_Sample_ASV9

CACCGCGGTTATACGAGAGACCCTAGTTGATATATACGGCGTAAAGAGTGGTTTGGGATA

TTTCTCCAAAATAAAGCCGAAGATCTCCCAAGCCGTCACACGCATCCCGGAGACACGAAG

CCCTAATACGAAGGTAGCTTTATTAGTACCCCGACGCCACGAAAGCTAAGAAA

>872_Sample_ASV10

CACCGCGGTTATACGAGAGACCCTAGTTGATATATACGGCGTAAAGAGTGGTTTGGGGTT

ATGCCTCCAAAATAAAGTCGAAGATCTCCCAAGCCGTCACACGCACCCCGGAGACACGAA

GCCCTAATACGAAGGTAGCTTTATTAATTCCCCGACGCCACGAAAGCTAAGAAA

>872_Sample_ASV11

CACCGCGGTTATACGAGAGACCCTAGTTGATATATACGGCGTAAAGAGTGGTTTGGGGTT

ATGCCTCCAAAATAAAGCCGAAGATCTCCCAAGCCGTCACACGCACCCCGGAGACACGAA

GCCCTAATACGAAGGTAGCTTTATTAATTCCCCGACGCCACGAAAGCTAAGAAA

>872_Sample_ASV12

CACCGCGGTTATACGAGAGACCCTAGTTGATATATACGGCGTAAAGAGTGGTTTGGGGTT

ATGCCTCCAAAATAAAGCCGAAGATCTCCCAAGCCGTCACACGCATCCCGGAGACACGAA

GCCCTAATACGAAGGTAGCTTTATTAATTCCCCGACGCCACGAAAGCTAAGAAA

>872_Sample_ASV13

CACCGCGGTTATACGAGAGACCCTAGTTGATATATACGGCGTAAAGAGTGGTTTGGGGTT

ATGCCTCCAAAATAAAGCCGAAGATCTCCCAAGCCGTCACACGCATCCCGGAGACACGAA

GCCCTAATACGAAGGTAGCTTTATTAGTACCCCGACGCCACGAAAGCTAAGAAA

>872_Sample_ASV14

CACCGCGGTTATACGAGAGGCCCAAGTTGATAAGCCCGGCGTAAAGAGTGGTTATGGAGA

ATAAAACACTAAAGCTAAAGACCCCCTAGGCTGTTATACGCACCTGGCGGCTCGAACCAC

CTATACGAAAGTAGCTTTACCCCCCTTCCACCAGAATCCACGACAGCTGGGGCA

>872_Sample_ASV15

CACCGCGGTTATACGAGAGGCCCTAGTTGATAGCCACGGCGCAAAGAGTGGTTAAGGAGC

CCCACTAAATAAAGTCGAACACTTCCTAGGCCGTTATACGTTTTCTAGAAGCACGAAACC

CAATTACACGAAAGCAACTTTATACTAAACAACCTGACCCCACGAAAGCTAAGAAA

>872_Sample_ASV16

CACCGCGGTTATACGAGAGGCCCAAGTTGACAGACATCGGCGTAAAGAGTGGTTAAGGAA

ACCCCATTACTAAAGCCGAACGCCCTCAGAACTGTCATACGTTCCCGAAGGTAAGAAGCC

CCACTACGAAAGTGGCTTTAACCACACCCGACCCCACGAAAGCTGTGAAA

>872_Sample_ASV17

CACCGCGGTTATACGAGAGGCCCAAGTTGATAGATATCGGCGTAAAGAGTGGTTAAGGAA

ACCCCGTTACTAAAGCCGAACGCCCTCAGAACTGTCATACGTTCCCGAAGGTAAGAAGCC

CCACTACGAAAGTGGCTTTAACCACACCCGACCCCACGAAAGCTGTGAAA

>872_Sample_ASV18

CACCGCGGTTAAACGAGAGGCCCTAGTTGATATTACAACGGCGTAAAGGGTGGTTAAGGA

AAGCAAGCAATAAAGCCGAATGGCCCTTTGGCTGTCATACGCTTCTAGGTGTCCGAAGCC

CAATATACGAAAGTAGCTTTAGAAAAGCCCACCTGACACCACGAAAGCTGAGGAA

>872_Sample_ASV19

CACCGCGGTTATACGAGAGGCCCAAGTTGACAGACACCGGCGTAAAGAGTGGTTAAGGAA

ATTCATCAACTAAAGCCGAATGCCCTCAGGACTGTTATACGTTTGCGAAGGTAAGAAGCC

CTACTACGAAAGTGGCTTTATTCCACCTGATTCCACGAAAGCTGCGGAA

>872_Sample_ASV20

CACCGCGGTTATACGAGAGGCCCCAGTTGATATTAGCGGCGTAAAGGGTGGTTAAGGACT

TTAATAACTAAAGCTAAACACTTCCCCGGCTGTTGTACGTAGACCGGAAGCTTGAAGCCC

AGACACGAAAGTAGCTTTATTCCCCCTGACCCCACGAAAGCTGAGAAA

>872_Sample_ASV21

CACCGCGGTTATACGAAAGACCCCAGTTTATAGACACGGCGTAAAGGGTGGTTATAGGAC

AAACTAACATAGAGCCAAAGACCCCCTAAGCCGTCATACGCTCACGAGGGCTCGAAGCCC

CTATACGAAAGTAGCTCTACCAAAAAATGCTCGACTCCACGAAAGCTGGGACA

>872_Sample_ASV22

CACCGCGGTTATACGAGAGGCCCCAGTTGATAAACACGGCGTAAAGGGTGGTTAAGGGAG

CATAAGAATAAAGCCGAAGGGCCCTTTGGCCGTTATACGCTTCTAGGTGTCCGAAGCCCA

AACACGAAAGTAGCTTTAGTTAAGCCCACCTGACTCCACGAAAACTGAGAAA

>872_Sample_ASV23

CACCGCGGTTATACGAGAGACCCTAGTTGATAAATACGGCGTAAAGAGTGGTTATGGGAA

AAACAAATAAAGTCAAACAACCTCTTAGCTGTTATACGCATTATGAGAGCATGAAGCCCC

CTCACGAAAGTAACTTTAATACCTCCTGACCCCACGAAAGCTAAGAAA

>872_Sample_ASV24

CACCGCGGTTATACGAGAGGCTCAAGTTGATGAACCCCGGCGTAAAGAGTGGTTAAGGGA

GATCAAAACTAAAGCCGAATGCTTTCAAAGCTGTTATACGCTTCCGAAAGTAAGAAGCTC

AATCACGAAAGTGGCTTTACTTTACCTGACCCCACGAAAGCTACGACA

>872_Sample_ASV25

CACCGCGGTTATACGAAAGACCCTAGTTGATGGATCACGGCGTAAAGGGTGGTTAAGGGA

TATAAAAATAAAGCTAAAAATCCTCTTAGCTGTCATACGCTTTCCGAAGACATGAGGCCC

AATTACGAAAGTAGCTTTAAACCACCCTGACACCACGAAAGCCAAGAAA

>872_Sample_ASV26

CACCGCGGTTATACGAAAGACCCCAGTTTATAGATGCGGCGTAAAGGGTGGTTAAGAGAA

AATCAAAATAAGGCCAAAGATCCCCTAAGCAGTCATACGCTCCACGAGGACACGAAACCC

AAATACGAAAGTTGCCTTAATAAACTTCACCTGATCCCACGAAAGCTGAAAAA

>872_Sample_ASV27

CACCGCGGTTATACGAGAGGCCCAAGTTGACAAACCCCGGCGTAAAGAGTGGTTAAGGAA

AATTTAAAACTAAAGCCGAATGCCCCCAAAGCTGTTATACGCACCCGAGGATAAGAAGCC

CAATCACGAAAGTGGCTTTACACTACCTGAACCCACGAAAGCTATGAAA

>872_Sample_ASV28

CACCGCGGTTATACGAGAGGCCCAAGTTGACAAACCCCGGCGTAAAGAGTGGTTAAGGAA

AATTTAAAACTAAAGCCGAATGCCCCCAAAGCTGTTATACGCACCCGAGGGTAAGAAGCC

CAATCACGAAAGTGGCTTTACACTACCTGAACCCACGAAAGCTATGAAA

>872_Sample_ASV29

CACCGCGGTTATACGAGAGGCTCAAGTTGATGAACCCCGGCGTAAAGAGTGGTTAAGGAA

AATTTAAAACTAAAGCCGAATGCCCCCAAAGCTGTTATACGCACCCGAGGGTAAGAAGCC

CAATCACGAAAGTGGCTTTACACTACCTGAACCCACGAAAGCTATGAAA

>872_Sample_ASV30

CACCGCGGTTATACGAGAGGCCCTAGTTGATAACTACCGGCGTAAAGAGTGGTTATGGAA

AAATATTTAATAAAGCCGAACACCCCCTCAGCCGTCATACGCACCTGGGAGCACGAAGAC

CTACTGCGAAAGCAGCTTTAATTATGCCTGACCCCACGACAGCTAAGAAA

>872_Sample_ASV31

CACCGCGGTTATACGAAAGACCCTAGTTTATAGACACGGCGTAAAGGGTGGTTCGGGACT

AAACAAAATAAAGCCAAAGACCTCCTAAGCCGTCATACGCTCACGGAAGCACGAAGCCCA

AGTACGAAAGTAGCTTTACAAAACATGCCTGACTCCACGAAAGCTAGGAAA

>872_Sample_ASV32

CACCGCGGTTATACGAAAGACCCTAGTTGATAGCCACGGCGTAAAGGGTGGTTAAGGTAT

ACAACAAATAAAGCTAAATAGCCTCTAGGCCGTCGCACGCATTCCGAGAGCTCGAAACCC

AAAAACGAAAGTAGCTTTAAAATAAACAGACCTGACCCCACGAAAGCTAAGAAA

>872_Sample_ASV33

CACCGCGGTTATACGAGAGGCCCAAGTTGATACTCCACGGCGTAAAGAGTGGTTAGAAAA

GACCTACTACTAAAGCCGAACGCCTTCAAAGCTGTTATACGCATCCGAAGGTGAGAAGCC

CACCCACGAAAGTGGCTTTACAACCTTGACTCCACGAAAGCTATGACA

>872_Sample_ASV34

CACCGCGGTTATACGAGAGACCCCAGTTGATAAACACGGCGTAAAGAGTGGTTAAGGGCA

CAAAACAAATAAAGTCAAACAACCTCTTAGCTGTTATACGCATTATGAGGATATGAAGCC

CCCCTACGAAAGTAACTTTACCAACCCCTGACCCCACGAAAGCTAAGAAA

>872_Sample_ASV35

CACCGCGGTTATACGAAAGACCCTAGTTTATAGGCACGGCGTAAAGGGTGGTTAGGGGAT

AAACAAAATAAAGCCAAAGACCTTCTAAGTCGTCATACACTCATGAAGGCACGAAGCCCA

AATACGAAAGTAACTTTACAACTTAGACCCGACTCCACGAAAGCTGGGGGA

>872_Sample_ASV36

CACCGCGGTTATACGAGAGACCCTAATTGATAGACTACGGCGTAAAGAGTGGTTTAGAAC

AAAAATTAATAAAGCCAAAGATCCTCCTGGCCGTCGCACGCATTTCGAGGATACGAAGCC

CCACTACGAAAGTAGCTTTACTACAACTTTTCTGACCCCACGAAAGCTAAGAAA

>880_Sample_ASV1

CACCGCGGTTATACGAGAGGCCCAAGTTGACAGACATCGGCGTAAAGAGTGGTTAAGGAA

AACCTTCAACTAAAGTCGAACGCTCTCAAGACTGTCGTACGTTTCCGAGAGTATGAAGCC

CCACCACGAAAGTGACTTTACCTCACCTGACTCCACGAAAGCTGCGAAA

>880_Sample_ASV2

CACCGCGGTTATACGAAAGACCCTAGTTGATGGGTGCGGCGTAAAGGGTGGTTAGGGACT

ACAAAAAATAGAGCTAAAGATCCTCTAGGTCGTCATACACATCACGAGGAAACGAAGCCC

AAATACGAAAGTAGCTCTAAATATAATCCTGACGCCACGAAAGCCAAGAAA

>880_Sample_ASV3

CACCGCGGTTATACGAAAGACCCTAGTTGATAACTACGGCATAAAGGGTGGTTAGGGAGT

TTAAAAAATAAAGCCAAAGCTCTTCTAAGCTGTCATACGCTCTCTGAAAGAACGAGGCCC

AAACACGAAAGTAGCTTTAAATAAATAACCTGATACCACGAAAGCCAAGAAA

>880_Sample_ASV4

CACCGCGGTTATACGAGAGACCCTAGTTGATTAAAGCGGCGTAAAGAGTGGTTATGGAAC

TATTCTTTTAAAGCAGAAAACCTCTCAAACTGTTATACGCACCCAGAGGTCTGAATCCCT

CACACGAAAGTGACTTTATTTATGCCTACCAGAACCCACGAAAGCTGGGACA

>880_Sample_ASV5

CACCGCGGTTATACGAGAGGCCCAAGTTGACAGACACCGGCGTAAAGAGTGGCTAGGGAA

AAATTACTACTAAAGCCGAACACCTTCAGAACTGTCATACGTTCCCGAAGATAAGAAGCC

CCACCACGAAAGTGGCTTTATATTACCCGACCCCACGAAAGCTGTGAAA

>880_Sample_ASV6

CACCGCGGTTATACGAGAGACCCAAGTTGACAGACACCGGCGTAAAGAGTGGCTAAGGAA

AAATTTCTACTAAAGCCGAACACCTTCAGAACTGTCATACGTTCCCGAAGAAAATAAGCC

CCACCACGAAAGTGGCTTTATATTACCCGACCCCACGAAAGCTGTGAAA

>880_Sample_ASV7

CACCGCGGTTATACGAGAGGCCCAAGTTGACAGACACCGGCGTAAAGAGTGGCTAAGGAA

AAATTTCTACTAAAGCCGAACACCTTCAGAACTGTCATACGTTCCCGAAGAAAAGAAGCC

CCACCACGAAAGTGGCTTTATATTACCCGACCCCACGAAAGCTGTGAAA

>880_Sample_ASV8

CACCGCGGTTATACGAAAGACCCTAGTTTATAGAAACGGCGTAAAGGGTGGTTAAGGGAC

AAACAAAATAAGGCCAAAGACTTCCTAAGCCGTCATACGCTCCACGAAAGCACGAAGCCC

AAACACGAAAGTAGCCTTAATAACTTCACCTGACCCCACGAAAGCTGGGAGA

>880_Sample_ASV9

CACCGCGGTTAAACGAGAGACCCTAGTTGATCATCACGGCGTAAAGAGTGGTTAGGGGAT

TATCATAAATAAAGCCAAAGACCTCCCAAGCTGTCGCACGCATTCCGGAGGCGCGAAGCC

CACACACGAAAGTAGCTTTAACTATTGACCCTGATGCCACGAAAGCTAAGAAA

>880_Sample_ASV10

CACCGCGGTTATACGAGAGACCCTAGTTGATATATACGGCGTAAAGAGTGGTTTGGGATA

TTTCTCCAAAATAAAGTCGAAGATCTCCCAAGCCGTCACACGCATCCCGGAGACACGAAG

CCCTGATACGAAGGTAGCTTTATTAACACCCCGACGCCACGAAAGCTAAGAAA

>880_Sample_ASV11

CACCGCGGTTATACGAGAGGCCCAAGTTGATAAGCCCGGCGTAAAGAGTGGTTATGGAGA

ATAAAACACTAAAGCTAAAGACCCCCTAGGCTGTTATACGCACCTGGCGGCTCGAACCAC

CTATACGAAAGTAGCTTTACCCCCCTTCCACCAGAATCCACGACAGCTGGGGCA

>880_Sample_ASV12

CACCGCGGTTATACGAGAGGCCCAAGTTGACAGACATCGGCGTAAAGAGTGGTTAAGGAA

ACCCCATTACTAAAGCCGAACGCCCTCAGAACTGTCATACGTTCCCGAAGGTAAGAAGCC

CCACTACGAAAGTGGCTTTAACCACACCCGACCCCACGAAAGCTGTGAAA

>880_Sample_ASV13

CACCGCGGTTATACGAGAGGCCCAAGTTGATAGATATCGGCGTAAAGAGTGGTTAAGGAA

ACCCCGTTACTAAAGCCGAACGCCCTCAGAACTGTCATACGTTCCCGAAGGTAAGAAGCC

CCACTACGAAAGTGGCTTTAACCACACCCGACCCCACGAAAGCTGTGAAA

>880_Sample_ASV14

CACCGCGGTTATACGAGAGGCCCAAGTTGACAGACACCGGCGTAAAGAGTGGTTAAGGAA

ATTCATCAACTAAAGCCGAATGCCCTCAGGACTGTTATACGTTTGCGAAGGTAAGAAGCC

CTACTACGAAAGTGGCTTTATTCCACCTGATTCCACGAAAGCTGCGGAA

>880_Sample_ASV15

CACCGCGGTTATACGAGAGGCCCCAGTTGATATTAGCGGCGTAAAGGGTGGTTAAGGACT

TTAATAACTAAAGCCAAACACTTCCCCGGCTGTTGTACGTAGACCGGAAGCTTGAAGCCC

AGACACGAAAGTAGCTTTATTTCCCCTGACCCCACGAAAGCTGAGAAA

>880_Sample_ASV16

CACCGCGGTTATACGAGAGGCCCCAGTTGATATTAGCGGCGTAAAGGGTGGTTAAGGACT

TTAATAACTAAAGCTAAACACTTCCCCGGCTGTTGTACGTAGACCGGAAGCTTGAAGCCC

AGACACGAAAGTAGCTTTATTTCCCCTGACCCCACGAAAGCTGAGAAA

>880_Sample_ASV17

CACCGCGGTTATACGAGAGGCCCCAGTTGATATTAGCGGCGTAAAGGGTGGTTAAGGACT

TTAATAACTAAAGCTAAACACTTCCCCGGCTGTTGTACGTAGACCGGAAGCTTGAAGCCC

AGACACGAAAGTAGCTTTATTCCCCCTGACCCCACGAAAGCTGAGAAA

>880_Sample_ASV18

CACCGCGGTCACACGATTAACCCAAGTCAATAGAAGCCGGCGTAAAGAGTGTTTTAGATC

ACCCCCTCCCCAATAAAGCTAAAACTCACCTGAGTTGTAAAAAACTCCAGTTGACACAAA

ATAGACTACGAAAGTGGCTTTAACATATCTGAACACACAATAGCTAAGACC

>880_Sample_ASV19

CACCGCGGTTATACGAGAAACCCTAGTTGACAAATACGGCGTAAAGAGTGGTTAAGGGGT

ATCAAAAATAAAGTCAAACATTTTCCTAGTTGTTATAAGCATTTCGAGAACATGAAGCCC

AACCACGAAAGTAACTTTACCTTCACCTGACCCCACGAAAGCTAAGAAA

>880_Sample_ASV20

CACCGCGGTTATACGAGAGGCTCAAGTTGATAAACCCCGGCGTAAAGAGTGGTTAAGGAA

AAATAGAAACTAAAGCCGAATGCTTTCAAGACTGTTATACGTTCCCGAGAGTAAGAAGAC

CAATTACGAAAGTGGCTTTACTTTACCTGACCCCACGAAAGCTACGACA

>880_Sample_ASV21

CACCGCGGTTATACGAGAGACCCTAATTGATAGACACGGCGTAAAGAGTGGTTTGGGAAA

GCCCAAAAATAAAGCTAAAGACCTTCTAAGCCGTCACACGCACGCCGAAGGCACGAAGCC

CTGACACGAAAGTAGCTTTACTATTACCCCCGACGCCACGAAAGCTAAGAAA

>880_Sample_ASV22

AACCGCGGTTATACGAGAGACCCTAATTGATAGCTCACGGCGTAAAGGGTGGTTTGGGGA

AACCTCAAAATAAAGCTAAAGACCTTCTAAGCCGTTACACGCATACCGAAGGCACGAGGC

CCTAACACGAAAGTAGCTTTACTATTACCCCTGACGCCACGAAAGCTAAGAAA

>880_Sample_ASV23

CACCGCGGTTATACGAGAGACCCTAATTGATAGACACGGCGTAAAGAGTGGTTTAGGACA

ACCCAAAAATAAAGCTAAAGACCTTCTAAGCCGTCACACGCACGCCGAAGGCACGAAGCC

CTGACACGAAAGTAGCTTTACTATTACCCCCGACGCCACGAAAGCTAAGAAA

>880_Sample_ASV24

CACCGCGGTTATACGAGAGACCCTAATTGATAGGTACGGCGTAAAGAGTGGTTTGGGATA

ACCCAAAAATAAAGCTAAAGACCTTCTAAGCCGTCACACGCATACCGAAGGCACGAGGCC

CCAACACGAAAGTAGCTTTACTATTACCCCGACGCCACGAAAGCTAAGAAA

>880_Sample_ASV25

CACCGCGGTTATACGAGAGACCCTAATTGATAGGCACGGCGTAAAGAGTGGTTTAGGGTA

ACCCATAAATAAAGCTAAAGACCTTCTAAGCCGTCACACGCACACCGAAGGCACGAGACC

CCAACACGAAAGTAGCTTTACTTCTACCCCTGACGCCACGAAAGCTAAGAAA

>880_Sample_ASV26

CACCGCGGTTATACGAAAGACCCTAGTTGATGGATCACGGCGTAAAGGGTGGTTAAGGGA

TATAAAAATAAAGCTAAAAATCCTCTTAGCTGTCATACGCTTTCCGAAGACATGAGGCCC

AATTACGAAAGTAGCTTTAAACCACCCTGACACCACGAAAGCCAAGAAA

>880_Sample_ASV27

CACCGCGGTTATACGAAAGACCCTAGTTTATAGATGCGGCGTAAAGGGTGGTTAAGGAAA

ACCTAAAATAATGCTAAAGACTCTCAAAGCTGTTATACGCTTCATGAGCACACGAAATCC

AAATACGAAAGTTGCCTTAATAAACTTTGCCTGACCCCACGAAAGCTGAAAAA

>880_Sample_ASV28

CACCGCGGTTATACGAAAGGCTCTAGTTTATAAATACGGCGTAAAGGGTGGTTAAGGAAA

AATTAAAATAATGCTAAAGACCCCCTAAGCAGTTATACGCTTCACGGAGGCACGAAACCC

AAATACGAAAGTTGCCTTAATAAACTTTACCTGACCCCACGAAAGCTGAGAAA

>880_Sample_ASV29

CACCGCGGTTATACGAAAGGCTCTAGTTTATAAATACGGCGTAAAGGGTGGTTAAGGAAA

AATTAAAATAATGCTAAAGACCCCCTAAGCAGTTATACGCTTCACGGAGGCACGAAACCC

AAATACGAAAGTTGCCTTAATAAACTTTACCTGACCCCACGAAAGCTGAAAAA

>880_Sample_ASV30

CACCGCGGTTATACGAGAGGCCCTAGTTGATAACTACCGGCGTAAAGAGTGGTTATGGAA

AAATATTTAATAAAGCCGAACACCCCCTCAGCCGTCATACGCACCTGGGAGCACGAAGAC

CTACTGCGAAAGCAGCTTTAATTATGCCTGACCCCACGACAGCTAAGAAA

>880_Sample_ASV31

CACCGCGGTTATACGAAAGACCCTAGTTTATAGACACGGCGTAAAGGGTGGTTCGGGACT

AAACAAAATAAAGCCAAAGACCTCCTAAGCCGTCATACGCTCACGGAAGCACGAAGCCCA

AGTACGAAAGTAGCTTTACAAAACATGCCTGACTCCACGAAAGCTAGGAAA

>880_Sample_ASV32

CACCGCGGTTATACGAAAGACCCTAGTTGATAGGCACGGCGTAAAGGGTGGTTAGGGATA

TATTAAAAATAAAGTTAAAGAGCCTCTAAGCTGTCGCACGCATCCCGAGAGCTCGAGACC

CAGACACGAAAGTAACTTTAAAATACCTCACCTGACCCCACGAAAGCTAAGAAA

>880_Sample_ASV33

CACCGCGGTTATACGAGAGGCCCAAGTTGATACTCCACGGCGTAAAGAGTGGTTAGAAAA

GACCTACTACTAAAGCCGAACGCCTTCAAAGCTGTTATACGCATCCGAAGGTGAGAAGCC

CACCCACGAAAGTGGCTTTACAACCTTGACTCCACGAAAGCTATGACA

>880_Sample_ASV34

CACCGCGGTTATACGAGAGACCCCAGTTGATAAACACGGCGTAAAGAGTGGTTAAGGGCA

CAAAACAAATAAAGTCAAACAACCTCTTAGCTGTTATACGCATTATGAGGATATGAAGCC

CCCCTACGAAAGTAACTTTACCAACCCCTGACCCCACGAAAGCTAAGAAA

>880_Sample_ASV35

CACCGCGGTTATACGAGAGACCCTAGTTGATATATACGGCGTAAAGAGTGGTTTGGAATA

ACCAAGTAATATAGCCAAAGACCTCCCAAGCCGTCACACGCACCCCGGAGGCACGAAGCC

CAAACACGAAAGTAGCTTTATTAACTTACCGACGCCACGAAAGCTAAGAAA

>880_Sample_ASV36

CACCGCGGTTATACGAAAGACCCTAGTTTATAGGCACGGCGTAAAGGGTGGTTAGGGGAT

AAACAAAATAAAGCCAAAGACCTTCTAAGTCGTCATACACTCATGAAGGCACGAAGCCCA

AATACGAAAGTAACTTTACAACTTAGACCCGACTCCACGAAAGCTGGGGGA

>880_Sample_ASV37

CACCGCGGTTATACGAGAGGCCCAAGTTGATAGACATCGGCGTAAAGAGTGGTTAAGGAA

ATCTTAAAACTAAAGCCGAATGCCTTCAAGACTGTTATACGTTTACGAAGGTAAGAAGCC

CCACTACGAAAGTGGCTTTATTCCACCTGACCCCACGAAAGCTGTGAAA

>880_Sample_ASV38

CACCGCGGTTATACGAGAGGCCCAAGTTGATAGACATCGGCGTAAAGAGTGGTTAAGGAA

GTCTCAAAACTAAAGCCGAATGCCTTCAAGACTGTTATACGTTTCCGAAGGTAAGAAGCC

CCACTACGAAAGTGGCTTTACTCCACCTGACTCCACGAAAGCTGTGAAA

>880_Sample_ASV39

CACCGCGGTTATACGAGAGGCCAAAGTTGACAGATATCGGCGTAAAGAGTGGTTAAGGAA

GTCTCAAAACTAAAGCCGAATGCCTTCAAGACTGTTATACGTTTCCGAAGGTAAGAAGCC

CCACTACGAAAGTGGCTTTACTCCACCTGACTCCACGAAAGCTGTGAAA

>880_Sample_ASV40

CACCGCGGTTATACGAGAGGCCAAAGTTGACAGATATCGGCGTAAAGAGTGGTTAAGGAA

ATCTTAAAACTAAAGCCGAATGCCTTCAAGACTGTTATACGTTTCCGAAGGTAAGAAGCC

CCACTACGAAAGTGGCTTTACTCCACCTGACTCCACGAAAGCTGTGAAA

>880_Sample_ASV41

CACCGCGGTTATACGAGAGGCCCAAGTTGATAGACATCGGCGTAAAGAGTGGTTAAGGAA

GTCTCAAAACTAAAGCCGAATGCCTTCAAGACTGTTATACGTTTACGAAGGTAAGAAGCC

CCACTACGAAAGTGGCTTTATTCCACCTGACCCCACGAAAGCTGTGAAA

>880_Sample_ASV42

CACCGCGGTTATACGAGAGGCCCAAGTTGACAGATATCGGCGTAAAGAGTGGTTAAGGAA

ATCTTAAAACTAAAGCCGAATGCCTTCAAGACTGTTATACGTTTCCGAAGGTAAGAAGCC

CCACTACGAAAGTGGCTTTACTCCACCTGACTCCACGAAAGCTGTGAAA

>880_Sample_ASV43

CACCGCGGTTATACGAGAGGCCCAAGTTGACAGATATCGGCGTAAAGAGTGGTTAAGGAA

ATCTTAAAACTAAAGCCGAATGCCTTCAAGACTGTTATACGTTTACGAAGGTAAGAAGCC

CCACTACGAAAGTGGCTTTACTCCACCTGACTCCACGAAAGCTGTGAAA

>880_Sample_ASV44

CACCGCGGTTATACGAGAGGCCCAAGTTGATAGACATCGGCGTAAAGAGTGGTTAAGGAA

GTCTCAAAACTAAAGCCGAATGCCTTCAAGACTGTTATACGTTTCCGAAGGTAAGAAGCC

CCACTACGAAAGTGGCTTTATTCCACCTGACCCCACGAAAGCTGTGAAA

>880_Sample_ASV45

CACCGCGGTTATACGAGAGACTCCAGTTGATAGCTACGGCGTAAAGAGTGGTTTGGGGCC

CGCCCAAAATAAAGCCAAAGACCTCCCAAGCCGTCAAACGCACCCCGGAGGCACGAAGTC

CTAACGCGAAAGCAACTTTACCTTCCCCGACGCCACGAAAGCTAAGAAA

>880_Sample_ASV46

CACCGCGGTTATACGAGAGACCCTAATTGATAGACTACGGCGTAAAGAGTGGTTTAGAAC

AAAAATTAATAAAGCCAAAGATCCTCCTGGCCGTCGCACGCATTTCGAGGATACGAAGCC

CCACTACGAAAGTAGCTTTACTACAACTTTTCTGACCCCACGAAAGCTAAGAAA

>880_Sample_ASV47

CACCGCGGTTATACGAAAGACCCTAGTTGATAGATCACGGCGTAAAGGGTGGTTAAGGAG

AACAAAAATAAAGCTAAAAATCCTCTAAGCTGTCATACGCTTTCCGAAGACATGAGACCC

AACCACGAAAGTAACTTTAAACTTTCCTGACACCACGAAAGCCAAGAAA

>883_Sample_ASV1

CACCGCGGTTATACGAGAGACCCTAGTTGATTAAAGCGGCGTAAAGAGTGGTTATGGAAC

TATTCTTTTAAAGCAGAAAACCTCTCAAACTGTTATACGCACCCAGAGGTCTGAATCCCT

CACACGAAAGTGACTTTATTTATGCCTACCAGAACCCACGAAAGCTGGGACA

>883_Sample_ASV2

CACCGCGGTTATACGAAAGACCCTAGTTTATAGGTACGGCGTAAAGGGTGGTTAGGGGAC

AAACAAAATAAAGCCAAAGACCTCCTAAGCCGTCATACGCTCACGGAAGCACGAAGCCCA

AACACGAAAGTAGCTTTACCGAACATGCCCGACTCCACGAAAGCTGGGGAA

>883_Sample_ASV3

CACCGCGGTTATACGAGAGGCCCAAGTTGACAGACACCGGCGTAAAGAGTGGCTAGGGAA

AAATTACTACTAAAGCCGAACACCTTCAGAACTGTCATACGTTCCCGAAGATAAGAAGCC

CCACCACGAAAGTGACTTTATATTACCCGACCCCACGAAAGCTGCGGAA

>883_Sample_ASV4

CACCGCGGTTATACGAGAGGCCCAAGTTGACAGACACCGGCGTAAAGAGTGGCTAGGGAA

AAATTACTACTAAAGCCGAACACCTTCAGAACTGTCATACGTTCCCGAAGATAAGAAGCC

CCACCACGAAAGTGACTTTATATTACCCGACCCCACGAAAGCTGTGAAA

>883_Sample_ASV5

CACCGCGGTTATACGAGAGGCCCAAGTTGACAGACACCGGCGTAAAGAGTGGCTAGGGAA

AAATTACTACTAAAGCCGAACACCCTCAGAACTGTCATACGTTCCCGAAGGTAAGAAGCC

CCACCACGAAAGTGGCTTTATAATACCCGACCCCACGAAAGCTGTGAAA

>883_Sample_ASV6

CACCGCGGTTATACGAGAGGCCCAAGTTGACAGACACCGGCGTAAAGAGTGGCTAGGGAA

AAATTACTACTAAAGCCGAACACCTTCAGAACTGTCATACGTTCCCGAAGATAAGAAGCC

CCACCACGAAAGTGGCTTTATATTACCCGACCCCACGAAAGCTGTGAAA

>883_Sample_ASV7

CACCGCGGTTATACGAGAGGCCCAAGTTGACAGACACCGGCGTAAAGAGTGGCTAGGGAA

AAATTACTACTAAAGCCGAACACCTTCAGAACTGTCATACGTTCCCGAAGATAAGAAGCC

CCACCACGAAAGTGGCTTTATATTACCCGACCCCACGAAAGCTAAGAAA

>883_Sample_ASV8

CACCGCGGTTATACGAGAGGCCCAAGTTGACAGACACCGGCGTAAAGAGTGGCTAAGGAA

AAATTTCTACTAAAGCCGAACACCTTCAGAACTGTCATACGTTCCCGAAGATAAGAAGCC

CCACCACGAAAGTGGCTTTATATTACCCGACACCACGAAAGCTGTGAAA

>883_Sample_ASV9

CACCGCGGTTATACGAGAGACCCTAGTTGATATATACGGCGTAAAGAGTGGTTTGGGGTT

ATGCCTCCAAAATAAAGCCGAAGATCTCCCAAGCCGTCACACGCATCCCGGAGACACGAA

GCCCTAATACGAAGGTAGCTTTATTAGTACCCCGACGCCACGAAAGCTAAGAAA

>883_Sample_ASV10

CACCGCGGTTATACGAGAGACCCTAGTTGATATATACGGCGTAAAGAGTGGTTTGGGGTT

ATGCCTCCAAAATAAAGCCGAAGATCTCCCAAGCCGTCACACGCATCCCGGAGACACGAA

GCCCTAATACGAAGGTAGCTTTATTAATTCCCCGACGCCACGAAAGCTAAGAAA

>883_Sample_ASV11

CACCGCGGTTATACGAGAGGCCCAAGTTGACAGACACCGGCGTAAAGAGTGGTTATGGAG

AATAAAACACTAAAGCTAAAGACCCCCTAGGCTGTTATACGCACCTGGCGGCTCGAACCA

CCTATACGAAAGTAGCTTTACCCCCCTTCCACCAGAATCCACGACAGCTGGGGCA

>883_Sample_ASV12

CACCGCGGTTATACGAGAGGCCCAAGTTGATAAGCCCGGCGTAAAGAGTGGTTATGGAGA

ATAAAACACTAAAGCTAAAGACCCCCTAGGCTGTTATACGCACCTGGCGGCTCGAACCAC

CTATACGAAAGTAGCTTTACCCCCCTTCCACCAGAATCCACGACAGCTGGGGCA

>883_Sample_ASV13

CACCGCGGTTATACGAGAGGCCCTAGTTGATAGCCACGGCGCAAAGAGTGGTTAAGGAGC

CCCACTAAATAAAGTCGAACACTTCCTAGGCCGTTATACGTTTTCTAGAAGCACGAAACC

CAATTACACGAAAGCAACTTTATACTAAACAACCTGACCCCACGAAAGCTAAGAAA

>883_Sample_ASV14

CACCGCGGTTATACGAGAGGCCCAAGTTGACAGACATCGGCGTAAAGAGTGGTTAAGGAA

ACCCCATTACTAAAGCCGAACGCCCTCAGAACTGTCATACGTTCCCGAAGGTAAGAAGCC

CCACTACGAAAGTGGCTTTAACCACACCCGACCCCACGAAAGCTGTGAAA

>883_Sample_ASV15

CACCGCGGTTATACGAGAGGCCCAAGTTGATAGATATCGGCGTAAAGAGTGGTTAAGGAA

ACCCCGTTACTAAAGCCGAACGCCCTCAGAACTGTCATACGTTCCCGAAGGTAAGAAGCC

CCACTACGAAAGTGGCTTTAACCACACCCGACCCCACGAAAGCTGTGAAA

>883_Sample_ASV16

CACCGCGGTTATACGAGAGGCCCAAGTTGACAGACACCGGCGTAAAGAGTGGTTAAGGAA

ATTCATCAACTAAAGCCGAATGCCCTCAGGACTGTTATACGTTTGCGAAGGTAAGAAGCC

CTACTACGAAAGTGGCTTTATTCCACCTGATTCCACGAAAGCTGCGGAA

>883_Sample_ASV17

CACCGCGGTTATACGAGAGGCCCCAGTTGATAAACACGGCGTAAAGGGTGGTTAAGGGAG

CATAAGAATAAAGCCGAAGGGCCCTTTGGCCGTTATACGCTTCTAGGTGTCCGAAGCCCA

AACACGAAAGTAGCTTTAGTTAAGCCCACCTGACTCCACGAAAACTGAGAAA

>883_Sample_ASV18

CACCGCGGTTATACGAGAGACCCTAGTTGATAAATACGGCGTAAAGAGTGGTTATGGGAA

AAACAAATAAAGTCAAACAACCTCTTAGCTGTTATACGCATTATGAGAGCATGAAGCCCC

CTCACGAAAGTAACTTTAATACCTCCTGACCCCACGAAAGCTAAGAAA

>883_Sample_ASV19

CACCGCGGTTATACGAGAGGCTCAAGTTGATAAACCCCGGCGTAAAGAGTGGTTAAGGAA

AAATAGAAACTAAAGCCGAATGCTTTCAAGACTGTTATACGTTCCCGAGAGTAAGAAGAC

CAATTACGAAAGTGGCTTTACTTTACCTGACCCCACGAAAGCTACGACA

>883_Sample_ASV20

CACCGCGGTTATACGAGAGGCTCAAGTTGATGAACCCCGGCGTAAAGAGTGGTTAAGGGA

GATCAAAACTAAAGCCGAATGCTTTCAAAGCTGTTATACGCTTCCGAAAGTAAGAAGCTC

AATCACGAAAGTGGCTTTACTTTACCTGACCCCACGAAAGCTACGACA

>883_Sample_ASV21

CACCGCGGTTATACGAGAGACCCTAATTGATAGGTACGGCGTAAAGAGTGGTTTGGGATA

ACCCAAAAATAAAGCTAAAGACCTTCTAAGCCGTCACACGCATACCGAAGGCACGAGGCC

CCAACACGAAAGTAGCTTTACTATTACCCCGACGCCACGAAAGCTAAGAAA

>883_Sample_ASV22

CACCGCGGTTATACGAGAGACCCTAATTGATAGGCACGGCGTAAAGAGTGGTTTAGGGTA

ACCCATAAATAAAGCTAAAGACCTTCTAAGCCGTCACACGCACACCGAAGGCACGAGACC

CCAACACGAAAGTAGCTTTACTTCTACCCCTGACGCCACGAAAGCTAAGAAA

>883_Sample_ASV23

CACCGCGGTTATACGAAAGACCCTAGTTGATGGATCACGGCGTAAAGGGTGGTTAAGGGA

TATAAAAATAAAGCTAAAAATCCTCTTAGCTGTCATACGCTTTCCGAAGACATGAGGCCC

AATTACGAAAGTAGCTTTAAACCACCCTGACACCACGAAAGCCAAGAAA

>883_Sample_ASV24

CACCGCGGTTATACGAAAGACCCCAGTTTATAGATGCGGCGTAAAGGGTGGTTAAGAGAA

AATCAAAATAAGGCCAAAGATCCCCTAAGCAGTCATACGCTCCACGAGGACACGAAACCC

AAATACGAAAGTTGCCTTAATAAACTTCACCTGATCCCACGAAAGCTGAAAAA

>883_Sample_ASV25

CACCGCGGTTATACGAGAGGCCCAAGTTGACAAACCCCGGCGTAAAGAGTGGTTAAGGAA

AATTTAAAACTAAAGCCGAATGCCCCCAAAGCTGTTATACGCACCCGAGGGTAAGAAGCC

CAATCACGAAAGTGGCTTTACACTACCTGAACCCACGAAAGCTATGAAA

>883_Sample_ASV26

CACCGCGGTTATACGAGAGGCCCAAGTTGACAAACCCCGGCGTAAAGAGTGGTTAAGGAA

AATTTAAAACTAAAGCCGAATGCCCCCAAAGCTGTTATACGCACCCGAGGATAAGAAGCC

CAATCACGAAAGTGGCTTTACACTACCTGAACCCACGAAAGCTATGAAA

>883_Sample_ASV27

CACCGCGGTTATACGAAAGACCCAAACTGATAGATGCCGGCGTAAAGAGTGGTTAAGTAT

CTTGATAGAACTAAAGCCGAACGCCCTCAAGACCGTCATACGTTTCCGAAGGTATGAAGC

CCAACTACGAAAGTAACTTTAACTGTATCCGACTCCACGAAAGCTGTGAAA

>883_Sample_ASV28

CACCGCGGTTATACGAGAGGCCCTAGTTGATAACTACCGGCGTAAAGAGTGGTTATGGAA

AAATATTTAATAAAGCCGAACACCCCCTCAGCCGTCATACGCACCTGGGAGCACGAAGAC

CTACTGCGAAAGCAGCTTTAATTATGCCTGACCCCACGACAGCTAAGAAA

>883_Sample_ASV29

CACCGCGGTTATACGAAAGACCCTAGTTTATAGACACGGCGTAAAGGGTGGTTCGGGACT

AAACAAAATAAAGCCAAAGACCTCCTAAGCCGTCATACGCTCACGGAAGCACGAAGCCCA

AGTACGAAAGTAGCTTTACAAAACATGCCTGACTCCACGAAAGCTAGGAAA

>883_Sample_ASV30

CACCGCGGTTATACGAGAGGCCCAAGTTGATACTCCACGGCGTAAAGAGTGGTTAGAAAA

GACCTACTACTAAAGCCGAACGCCTTCAAAGCTGTTATACGCATCCGAAGGTGAGAAGCC

CACCCACGAAAGTGGCTTTACAACCTTGACTCCACGAAAGCTATGACA

>883_Sample_ASV31

CACCGCGGTTATACGAGAGACCCCAGTTGATAAACACGGCGTAAAGAGTGGTTAAGGGCA

CAAAACAAATAAAGTCAAACAACCTCTTAGCTGTTATACGCATTATGAGGATATGAAGCC

CCCCTACGAAAGTAACTTTACCAACCCCTGACCCCACGAAAGCTAAGAAA

>883_Sample_ASV32

CACCGCGGTTATACGAAAGACCCTAGTTTATAGGCACGGCGTAAAGGGTGGTTAGGGGAT

AAACAAAATAAAGCCAAAGACCTTCTAAGTCGTCATACACTCATGAAGGCACGAAGCCCA

AATACGAAAGTAACTTTACAACTTAGACCCGACTCCACGAAAGCTGGGGGA

>889_Sample_ASV1

CACCGCGGTTATACGAGAGGCCCAAGTTGACAGACATCGGCGTAAAGAGTGGTTAAGGAA

AACCTTCAACTAAAGTCGAACGCTCTCAAGACTGTCGTACGTTTCCGAGAGTATGAAGCC

CCACCACGAAAGTGACTTTACCTCACCTGACTCCACGAAAGCTGCGAAA

>889_Sample_ASV2

CACCGCGGTTATACGAAAGACCCTAGTTGATGGGTGCGGCGTAAAGGGTGGTTAGGGACT

ACAAAAAATAGAGCTAAAGATCCTCTAGGTCGTCATACACATCACGAGGAAACGAAGCCC

AAATACGAAAGTAGCTCTAAATATAATCCTGACGCCACGAAAGCCAAGAAA

>889_Sample_ASV3

CACCGCGGTTATACGAAAGACCCTAGTTGATAACTACGGCATAAAGGGTGGTTAGGGAGT

TTAAAAAATAAAGCCAAAGCTCTTCTAAGCTGTCATACGCTCTCTGAAAGAACGAGGCCC

AAACACGAAAGTAGCTTTAAATAAATAACCTGATACCACGAAAGCCAAGAAA

>889_Sample_ASV4

CACCGCGGTTATACGAGAGGCCCAAGTTGACAGACACCGGCGTAAAGAGTGGCTAGGGAA

AAATTACTACTAAAGCCGAACACCTTCAGAACTGTCATACGTTCCCGAAGATAAGAAGCC

CCACCACGAAAGTGACTTTATATTACCCGACCCCACGAAAGCTGTGAAA

>889_Sample_ASV5

CACCGCGGTTATACGAGAGGCCCAAGTTGACAGACACCGGCGTAAAGAGTGGCTAGGGAA

AAATTACTACTAAAGCCGAACACCTTCAGAACTGTCATACGTTCCCGAAGATAAGAAGCC

CCACCACGAAAGTGACTTTATATTACCCGACCCCACGAAAGCTGCGGAA

>889_Sample_ASV6

CACCGCGGTTATACGAGAGGCCCAAGTTGACAGACACCGGCGTAAAGAGTGGCTAGGGAA

AAATTACTACTAAAGCCGAACACCTTCAGAACTGTCATACGTTCCCGAAGATAAGAAGCC

CCACCACGAAAGTGACTTTATATTACCCGACCCCACGAAAGCCAAGAAA

>889_Sample_ASV7

CACCGCGGTTATACGAGAGGCCCAAGTTGACAGACACCGGCGTAAAGAGTGGCTAGGGAA

AAATTACTACTAAAGCCGAACACCTTCAGAACTGTCATACGTTCCCGAAGATAAGAAGCC

CCACCACGAAAGTGGCTTTATATTACCCGACCCCACGAAAGCTGTGAAA

>889_Sample_ASV8

CACCGCGGTTATACGAGAGGCCCAAGTTGACAGACACCGGCGTAAAGAGTGGCTAGGGAA

AAATTACTACTAAAGCCGAACACCCTCAGAACTGTCATACGTTCCCGAAGGTAAGAAGCC

CCACCACGAAAGTGGCTTTATAATACCCGACCCCACGAAAGCTGTGAAA

>889_Sample_ASV9

CACCGCGGTTATACGAAAGACCCTAGTTTATAGAAACGGCGTAAAGGGTGGTTAAGGGAC

AAACAAAATAAGGCCAAAGACTTCCTAAGCCGTCATACGCTCCACGAAAGCACGAAGCCC

AAACACGAAAGTAGCCTTAATAACTTCACCTGACCCCACGAAAGCTGGGAGA

>889_Sample_ASV10

CACCGCGGTTATACGAGAGACCCTAGTTGATATATACGGCGTAAAGAGTGGTTTGGGATA

TTTCTCCAAAATAAAGCCGAAGATCTCCCAAGCCGTCACACGCATCCCGGAGACACGAAG

CCCTGATACGAAGGTAGCTTTATTAACACCCCGACGCCACGAAAGCTAAGAAA

>889_Sample_ASV11

CACCGCGGTTATACGAGAGACCCTAGTTGATATATACGGCGTAAAGAGTGGTTTGGGATA

TTTCTCCAAAATAAAGTCGAAGATCTCCCAAGCCGTCACACGCATCCCGGAGACACGAAG

CCCTGATACGAAGGTAGCTTTATTAACACCCCGACGCCACGAAAGCTAAGAAA

>889_Sample_ASV12

CACCGCGGTTATACGAGAGGCCCAAGTTGATAAGCCCGGCGTAAAGAGTGGTTATGGAGA

ATAAAACACTAAAGCTAAAGACCCCCTAGGCTGTTATACGCACCTGGCGGCTCGAACCAC

CTATACGAAAGTAGCTTTACCCCCCTTCCACCAGAATCCACGACAGCTGGGGCA

>889_Sample_ASV13

CACCGCGGTTATACGAGAGGCCCAAGTTGACAGACATCGGCGTAAAGAGTGGTTAAGGAA

ACCCCATTACTAAAGCCGAACGCCCTCAGAACTGTCATACGTTCCCGAAGGTAAGAAGCC

CCACTACGAAAGTGGCTTTAACCACACCCGACCCCACGAAAGCTGTGAAA

>889_Sample_ASV14

CACCGCGGTTATACGAGAGGCCCAAGTTGACAGACACCGGCGTAAAGAGTGGTTAAGGAA

ATTCATCAACTAAAGCCGAATGCCCTCAGGACTGTTATACGTTTGCGAAGGTAAGAAGCC

CTACTACGAAAGTGGCTTTATTCCACCTGATTCCACGAAAGCTGCGGAA

>889_Sample_ASV15

CACCGCGGTTATACGAGAGGCCCCAGTTGATATTAGCGGCGTAAAGGGTGGTTAAGGACT

TTAATAACTAAAGCTAAACACTTCCCCGGCTGTTGTACGTAGACCGGAAGCTTGAAGCCC

AGACACGAAAGTAGCTTTATTCCCCCTGACCCCACGAAAGCTGAGAAA

>889_Sample_ASV16

CACCGCGGTTATACGAGAAACCCTAGTTGACAAATACGGCGTAAAGAGTGGTTAAGGGGT

ATCAAAAATAAAGTCAAACATTTTCCTAGTTGTTATAAGCATTTCGAGAACATGAAGCCC

AACCACGAAAGTAACTTTACCTTCACCTGACCCCACGAAAGCTAAGAAA

>889_Sample_ASV17

CACCGCGGTTATACGAAAGACCCTAGTTTATAAGTACGGCGTAAAGGGTGGTTAGGGGAC

AAACAAAATAAAGCCAAAGACCTCCTAAGCCGTCATACGCTTACGGAGGCACGAAGCCCA

AACACGAAAGTAGCTTTACCGAACATGCCCGACTCCACGAAAGCTGGGAAA

>889_Sample_ASV18

AACCGCGGTTATACGAGAGACCCTAATTGATAGCTCACGGCGTAAAGGGTGGTTTGGGGA

AACCTCAAAATAAAGCTAAAGACCTTCTAAGCCGTTACACGCATACCGAAGGCACGAGGC

CCTAACACGAAAGTAGCTTTACTATTACCCCTGACGCCACGAAAGCTAAGAAA

>889_Sample_ASV19

CACCGCGGTTATACGAGAGACCCTAATTGATAGGTACGGCGTAAAGAGTGGTTTGGGATA

ACCCAAAAATAAAGCTAAAGACCTTCTAAGCCGTCACACGCATACCGAAGGCACGAGGCC

CCAACACGAAAGTAGCTTTACTATTACCCCGACGCCACGAAAGCTAAGAAA

>889_Sample_ASV20

CACCGCGGTTATACGAAAGACCCTAGTTGATGGATCACGGCGTAAAGGGTGGTTAAGGGA

TATAAAAATAAAGCTAAAAATCCTCTTAGCTGTCATACGCTTTCCGAAGACATGAGGCCC

AATTACGAAAGTAGCTTTAAACCACCCTGACACCACGAAAGCCAAGAAA

>889_Sample_ASV21

CACCGCGGTTATACGAGAGGCCCTAGTTGATAACTACCGGCGTAAAGAGTGGTTATGGAA

AAATATTTAATAAAGCCGAACACCCCCTCAGCCGTCATACGCACCTGGGAGCACGAAGAC

CTACTGCGAAAGCAGCTTTAATTATGCCTGACCCCACGACAGCTAAGAAA

>889_Sample_ASV22

CACCGCGGTTATACGAAAGACCCTAGTTTATAGACACGGCGTAAAGGGTGGTTCGGGACT

AAACAAAATAAAGCCAAAGACCTCCTAAGCCGTCATACGCTCACGGAAGCACGAAGCCCA

AGTACGAAAGTAGCTTTACAAAACATGCCTGACTCCACGAAAGCTAGGAAA

>889_Sample_ASV23

CACCGCGGTTATACGAGAGACCCCAGTTGATAAACACGGCGTAAAGAGTGGTTAAGGGCA

CAAAACAAATAAAGTCAAACAACCTCTTAGCTGTTATACGCATTATGAGGATATGAAGCC

CCCCTACGAAAGTAACTTTACCAACCCCTGACCCCACGAAAGCTAAGAAA

>889_Sample_ASV24

CACCGCGGTTATACGAAAGACCCTAGTTTATAGGCACGGCGTAAAGGGTGGTTAGGGGAT

AAACAAAATAAAGCCAAAGACCTTCTAAGTCGTCATACACTCATGAAGGCACGAAGCCCA

AATACGAAAGTAACTTTACAACTTAGACCCGACTCCACGAAAGCTGGGGGA

>889_Sample_ASV25

CACCGCGGTTATACGAAAGACCCTAGTTGATAGCCACGGCGTAAAGGGTGGTTAAGGTAA

CTAATAAATAAAGCTAAAGAGCCTCTAAGCCGTCGCACGCATTCCGAGAGCTCGAAGCCC

AAACACGAAAGTAGCTTTAAAATAAAGCACACCTGACCCCACGAAAGCTAAGAAA

>889_Sample_ASV26

CACCGCGGTTATACGAGAGGCCCAAGTTGATAGACATCGGCGTAAAGAGTGGTTAAGGAA

GTCTCAAAACTAAAGCCGAATGCCTTCAAGACTGTTATACGTTTCCGAAGGTAAGAAGCC

CCACTACGAAAGTGGCTTTACTCCACCTGACTCCACGAAAGCTGTGAAA

>889_Sample_ASV27

CACCGCGGTTATACGAGAGACTCCAGTTGATAGCTACGGCGTAAAGAGTGGTTTGGGGCC

CGCCCAAAATAAAGCCAAAGACCTCCCAAGCCGTCAAACGCACCCCGGAGGCACGAAGTC

CTAACGCGAAAGCAACTTTACCTTCCCCGACGCCACGAAAGCTAAGAAA

>889_Sample_ASV28

CACCGCGGTTATACGAGAGACTCCAGTTGATAGCTACGGCGTAAAGAGTGGTTTGGGGCC

CGCCCAAAATAAAGCCAAAGACCTCCCAAGCCGTCAAACGCACCCCGGAGGCACGAAGTC

CTAACGCGAAAGCAACTTTACCTTCCCCGACGCCACGAAAGCTGTGAAA

>889_Sample_ASV29

CACCGCGGTTATACGAGAGACCCTAATTGATAGACTACGGCGTAAAGAGTGGTTTAGAAC

AAAAATTAATAAAGCCAAAGATCCTCCTGGCCGTCGCACGCATTTCGAGGATACGAAGCC

CCACTACGAAAGTAGCTTTACTACAACTTTTCTGACCCCACGAAAGCTAAGAAA

>889_Sample_ASV30

CACCGCGGTTATACGAAAGACCCTAGTTGATAGATCACGGCGTAAAGGGTGGTTAAGGAG

AACAAAAATAAAGCTAAAAATCCTCTAAGCTGTCATACGCTTTCCGAAGACATGAGACCC

AACCACGAAAGTAACTTTAAACTTTCCTGACACCACGAAAGCCAAGAAA

>892_Sample_ASV1

TACCGCGGTTATACGAAAGACCCAAGTTAATAAACTACGGCGTAAAGCGTGGTTAGAGAC

CTCCAATAATTAAAGCCAAAGACCTTCTAAGCCGTCGCACGCCCCGCGAAGACACGAAGC

CCAAAAACGAAAGTAGCTTTATTCTTACTCGACCCCACGAAAGCTAGGGCA

>892_Sample_ASV2

CACCGCGGTTATACGAAAGACCCTAGTTGATGGGTGCGGCGTAAAGGGTGGTTAGGGACT

ACAAAAAATAGAGCTAAAGATCCTCTAGGTCGTCATACACATCACGAGGAAACGAAGCCC

AAATACGAAAGTAGCTCTAAATATAATCCTGACGCCACGAAAGCCAAGAAA

>892_Sample_ASV3

CACCGCGGTTATACGAGAGACCCTAGTTGATTAAAGCGGCGTAAAGAGTGGTTATGGAAC

TATTCTTTTAAAGCAGAAAACCTCTCAAACTGTTATACGCACCCAGAGGTCTGAATCCCT

CACACGAAAGTGACTTTATTTATGCCTACCAGAACCCACGAAAGCTGGGACA

>892_Sample_ASV4

CACCGCGGTTATACGAGAGGCCCAAGTTGACAGACACCGGCGTAAAGAGTGGCTAGGGAA

AAATTACTACTAAAGCCGAACACCTTCAGAACTGTCATACGTTCCCGAAGATAAGAAGCC

CCACCACGAAAGTGGCTTTATATTACCCGACCCCACGAAAGCTGTGAAA

>892_Sample_ASV5

CACCGCGGTTATACGAGAGGCCCAAGTTGACAGACACCGGCGTAAAGAGTGGCTAGGGAA

AAATTACTACTAAAGCCGAACACCTTCAGAACTGTCATACGTTCCCGAAGATAAGAAGCC

CCACCACGAAAGTGGCTTTATATTACCCGACCCCACGAAAGCTGTGATA

>892_Sample_ASV6

CACCGCGATTATACGAGAGGCCCAAGTTGACAGACATCGGCGTAAAGAGTGGTTAAGGAA

AAATTACTACTAAAGCCGAACACCTTCAGAACTGTCATACGTTCCCGAAGATAAGAAGCC

CCACCACGAAAGTGGCTTTATATTACCCGACCCCACGAAAGCTGTGAAA

>892_Sample_ASV7

CATGGCGATTATACGAGAGGCCCAAGTTGACAGACACTGGCGTAAAGAGTGGCTAAGGAA

AAACTACTACTAAAGCCGAACACCTTCAGAACTGTCATACGTTCCCGAAGATAAGAAGCC

CCACCACGAAAGTGGCTTTATATTACCCGACCCCACGAAAGCTGTGAAA

>892_Sample_ASV8

CATGGCGATTATACGAGAGGCCCAAGTTGACAGACACTGGCGTAAAGAGTGGCTAAGGAA

AAATTACTACTAAAGCCGAACACCTTCAGAACTGTCATACGTTCCCGAAGATAAGAAGCC

CCACCACGAAAGTGGCTTTATATTACCCGACCCCACGAAAGCTGTGAAA

>892_Sample_ASV9

CACCGCGGTTATACGAGAGGCCCAAGTTGACAGACACCGGCGTAAAGAGTGGCTAGGGAA

AAATTACTACTAAAGCCGAACACCTTCAGAACTGTCATACGTTCCCGAAGATAAGAAGCC

CCACCACGAAAGTGGCTTTACAATGCCTGACCCCACGAAAGCTGTGATA

>892_Sample_ASV10

CACCGCGGTTATACGAAAGACTCTAGTTGATAAACACGGCGTAAAGGGTGGTTAGGACAA

TAATAAAATAAAGTTAAAGACTTACCAAGCCGTCATACGCCCATGAAAGAACGAAAACCA

CAAACGAAAGTAACTTTATTACACATCCGAACCCACGAAAGCTAAGAAA

>892_Sample_ASV11

CACCGCGGTTATACGAAAGACTCTAGTTGACAAACACGGCGTAAAGGGTGGTTAGGATAA

CAACAAAATAAAGCTAAAGACTTACCAAGCCGTCATACGCCCATGTAAGAGCGAAAACCA

CAAACGAAAGTAACTTTATTACACCATCCGAATCCACGAAAGCTAAGAAA

>892_Sample_ASV12

CACCGCGGTTATACGAAAGACCCTAGTTTATAGAAACGGCGTAAAGGGTGGTTAAGGGAC

AAACAAAATAAGGCCAAAGACTTCCTAAGCCGTCATACGCTCCACGAAAGCACGAAGCCC

AAACACGAAAGTAGCCTTAATAACTTCACCTGACCCCACGAAAGCTGGGAGA

>892_Sample_ASV13

CACCGCGGTTATACGAAAGACCCTAGTTGATAGATCACGGCGTAAAGGGTGGTTAGGGAA

TACAAAAATAAAGCTAAAAATCCTCTAAGCTGTCATACGCTTCCCGAAGACATGAAACCC

AACCACGAAAGTAGCTTTAAACTCTCCTGACACCACGAAAGCCAAGGAA

>892_Sample_ASV14

CACCGCGGTTATACGAGAGGCCCAAGTTGATAAGCCCGGCGTAAAGAGTGGTTATGGAGA

ATAAAACACTAAAGCTAAAGACCCCCTAGGCTGTTATACGCACCTGGCGGCTCGAACCAC

CTATACGAAAGTAGCTTTACCCCCCTTCCACCAGAATCCACGACAGCTGGGGCA

>892_Sample_ASV15

CACCGCGGTTATACGAGAGGCCCAAGTTGACAGACATCGGCGTAAAGAGTGGTTAAGGAA

ACCCCATTACTAAAGCCGAACGCCCTCAGAACTGTCATACGTTCCCGAAGGTAAGAAGCC

CCACTACGAAAGTGGCTTTAACCACACCCGACCCCACGAAAGCTAAGAAA

>892_Sample_ASV16

CACCGCGGTTATACGAGAGGCCCAAGTTGACAGACATCGGCGTAAAGAGTGGTTAAGGAA

ACCCCATTACTAAAGCCGAACGCCCTCAGAACTGTCATACGTTCCCGAAGGTAAGAAGCC

CCACTACGAAAGTGGCTTTAACCACACCCGACCCCACGAAAGCTGTGAAA

>892_Sample_ASV17

CACCGCGGTTATACGAGAGGCCCAAGTTGACAGACACCGGCGTAAAGAGTGGTTAAGGAA

ATTCATCAACTAAAGCCGAATGCCCTCAGGACTGTTATACGTTTGCGAAGGTAAGAAGCC

CTACTACGAAAGTGGCTTTATTCCACCTGATTCCACGAAAGCTGTGAAA

>892_Sample_ASV18

CACCGCGGTTATACGAGAGGCCCAAGTTGACAGACACCGGCGTAAAGAGTGGTTAAGGAA

ATTCATCAACTAAAGCCGAATGCCCTCAGGACTGTTATACGTTTGCGAAGGTAAGAAGCC

CTACTACGAAAGTGGCTTTATTCCACCTGATTCCACGAAAGCTGTGATA

>892_Sample_ASV19

CACCGCGGTTATACGAGAGGCCCAAGTTGACAGACACCGGCGTAAAGAGTGGTTAAGGAA

ATTCATCAACTAAAGCCGAATGCCCTCAGGACTGTTATACGTTTGCGAAGGTAAGAAGCC

CTACTACGAAAGTGGCTTTATTCCACCTGATTCCACGAAAGCTGCGGAA

>892_Sample_ASV20

CACCGCGGTTATACGAGAGGCCCCAGTTGATATTAGCGGCGTAAAGGGTGGTTAAGGACT

TTAATAACTAAAGCTAAACACTTCCCCGGCTGTTGTACGTAGACCGGAAGCTTGAAGCCC

AGACACGAAAGTAGCTTTATTTCCCCTGACCCCACGAAAGCTGAGAAA

>892_Sample_ASV21

CACCGCGGTTATACGAGAGGCCCCAGTTGATATTAGCGGCGTAAAGGGTGGTTAAGGACT

TTAATAACTAAAGCCAAACACTTCCCCGGCTGTTGTACGTAGACCGGAAGCTTGAAGCCC

AGACACGAAAGTAGCTTTATTTCCCCTGACCCCACGAAAGCTGAGAAA

>892_Sample_ASV22

CACCGCGGTTATACGAGAGGCCCCAGTTGATATTAGCGGCGTAAAGGGTGGTTAAGGACT

TTAATAACTAAAGCTAAACACTTCCCCGGCTGTTGTACGTAGACCGGAAGCTTGAAGCCC

AGACACGAAAGTAGCTTTATTCCCCCTGACCCCACGAAAGCTGAGAAA

>892_Sample_ASV23

CACCGCGGTTATACGAAAGACCCCAGTTTATAGACACGGCGTAAAGGGTGGTTATAGGAC

AAACTAACATAGAGCCAAAGACCCCCTAAGCCGTCATACGCTCACGAGGGCTCGAAGCCC

CTATACGAAAGTAGCTCTACCAAAAAATGCTCGACTCCACGAAAGCTGGGACA

>892_Sample_ASV24

CACCGCGGTTATACGAGAGACCCTAGTTGATAAATACGGCGTAAAGAGTGGTTATGGGAA

AAACAAATAAAGTCAAACAACCTCTTAGCTGTTATACGCATTATGAGAGCATGAAGCCCC

CTCACGAAAGTAACTTTAATACCTCCTGACCCCACGAAAGCTAAGAAA

>892_Sample_ASV25

CACCGCGGTTATACGAGAGACCCTAATTGATAGGCACGGCGTAAAGAGTGGTTTAGGGTA

ACCCATAAATAAAGCTAAAGACCTTCTAAGCCGTCACACGCACACCGAAGGCACGAGACC

CCAACACGAAAGTAGCTTTACTTCTACCCCTGACGCCACGAAAGCTAAGAAA

>892_Sample_ASV26

CACCGCGGTTATACGAAAGACCCTAGTTGATGGATCACGGCGTAAAGGGTGGTTAAGGGA

TATAAAAATAAAGCTAAAAATCCTCTTAGCTGTCATACGCTTTCCGAAGACATGAGGCCC

AATTACGAAAGTAGCTTTAAACCACCCTGACACCACGAAAGCCAAGAAA

>892_Sample_ASV27

CACCGCGGTTATACGAAAGACCCCAGTTTATAGATGCGGCGTAAAGGGTGGTTAAGAGAA

AATCAAAATAAGGCCAAAGATCCCCTAAGCAGTCATACGCTCCACGAGGACACGAAACCC

AAATACGAAAGTTGCCTTAATAAACTTCACCTGATCCCACGAAAGCTGAAAAA

>892_Sample_ASV28

CACCGCGGTTATACGAAAGGCTCTAGTTTATAAATACGGCGTAAAGGGTGGTTAAGGAAA

AATTAAAATAATGCTAAAGACCCCCTAAGCAGTTATACGCTTCACGGAGGCACGAAACCC

AAATACGAAAGTTGCCTTAATAAACTTTACCTGACCCCACGAAAGCTGAAAAA

>892_Sample_ASV29

CACCGCGGTTATACGAGAGACTCTAGTTGATACCAACGGCGTAAAGAGTGGTTTGGGATG

CCCCTTAAAATAAAGCCAAAGACCTCCCAAGCCGTCAAACGCACCCCGGAGGCACGAAGC

CCCAACGCGAAAGCAACTTTACCTCCCTCCCGACGCCACGAAAGCTAAGAAA

>892_Sample_ASV30

CACCGCGGTTATACGAGAGACCCTAGTTGATAGCTACGGCGTAAAGCGTGGTTAGGAGAA

CAAGATAAATAAAGTCAAACAATCTCTCGGCCGTTATACGTTATCTGAGAATATGAAGTC

CTACCACGAAAGTAACTTTAATTTTTCTGACCCCACGAAAACTGAGGAA

>892_Sample_ASV31

CACCGCGGTTATACGAGAGGCCCTAGTTGATAACTACCGGCGTAAAGAGTGGTTATGGAA

AAATATTTAATAAAGCCGAACACCCCCTCAGCCGTCATACGCACCTGGGAGCACGAAGAC

CTACTGCGAAAGCAGCTTTAATTATGCCTGACCCCACGACAGCTAAGAAA

>892_Sample_ASV32

CACCGCGGTTATACGAGAGGCCCAAGTTGATTATTACCGGCGTAAAGCGTGGTTAAGATT

TTTATAGGACTAAAGCCGAACCCCCTCAGAGCTGTTATACGCACCCGAAGGTTAGAAGTT

CAATCACGAAAGTGGCTTTACAACCCCGAACCCACGAAAGCTAAGATA

>892_Sample_ASV33

CACCGCGGTTATACGAAAGACCCTAGTTGATAGGCACGGCGTAAAGGGTGGTTAGGGATA

TATCAAAAATAAAGTTAAAGAGCCTCTAAGCTGTCGCACGCATCCCGAGAGCTCGAGACC

CAGACACGAAAGTAACTTTAAAATACCTCACCTGACCCCACGAAAGCTAAGAAA

>892_Sample_ASV34

CACCGCGGTTATACGAAAGACCCTAGTTGATAGCCACGGCGTAAAGGGTGGTTAAGGTAT

ACAACAAATAAAGCTAAATAGCCTCTAGGCCGTCGCACGCATTCCGAGAGCTCGAAACCC

AAAAACGAAAGTAGCTTTAAAATAAACAGACCTGACCCCACGAAAGCTAAGAAA

>892_Sample_ASV35

CACCGCGGTTATACGAGAGGCCCAAGTTGATACTCCACGGCGTAAAGAGTGGTTAGAAAA

GACCTACTACTAAAGCCGAACGCCTTCAAAGCTGTTATACGCATCCGAAGGTGAGAAGCC

CACCCACGAAAGTGGCTTTACAACCTTGACTCCACGAAAGCTATGACA

>892_Sample_ASV36

CACCGCGGTTATACGAGAGACCCCAGTTGATAAACACGGCGTAAAGAGTGGTTAAGGGCA

CAAAACAAATAAAGTCAAACAACCTCTTAGCTGTTATACGCATTATGAGGATATGAAGCC

CCCCTACGAAAGTAACTTTACCAACCCCTGACCCCACGAAAGCTAAGAAA

>892_Sample_ASV37

CACCGCGGTTATACGAAAGACCCTAGTTTATAGGCACGGCGTAAAGGGTGGTTAGGGGAT

AAACAAAATAAAGCCAAAGACCTTCTAAGTCGTCATACACTCATGAAGGCACGAAGCCCA

AATACGAAAGTAACTTTACAACTTAGACCCGACTCCACGAAAGCTGGGGGA

>892_Sample_ASV38

CACCGCGGTTATACGAAAGACCCTAGTTGATAGCTACGGCGTAAAGGGTGGTTAAGGTAT

ATAACAAATAAAGCTAAAGAGCCTCCAAGCCGTCGTACGCATTCTGAGAGCTCGAAACCC

TAACACGAAAGTAGCTTTAAAAAATAACACCTGACCCCACGAAAGCTAAGAAA

>892_Sample_ASV39

CACCGCGGTTATACGAAAGACCCTAGTTGATAGATCACGGCGTAAAGGGTGGTTAAGGAG

AACAAAAATAAAGCTAAAAATCTTCTAAGCTGTCATACGCTTTCCGAAGACATGAGACCC

AACCACGAAAGTAGCTTTAAACTTTCCTGACACCACGAAAGCCAAGAAA

>893_Sample_ASV1

CACCGCGGTTATACGAAAGACCCTAGTTGATGGGTGCGGCGTAAAGGGTGGTTAGGGACT

ACAAAAAATAGAGCTAAAGATCCTCTAGGTCGTCATACACATCACGAGGAAACGAAGCCC

AAATACGAAAGTAGCTCTAAATATAATCCTGACGCCACGAAAGCCAAGAAA

>893_Sample_ASV2

CACCGCGGTTATACGAGAGACCCTAGTTGATTAGAGCGGCGTAAAGAGTGGTTATGGAAC

TATTCTTTTAAAGCAGAAAACCTCTCAAACTGTTATACGCACCCAGAGGTCTGAATCCCT

CACACGAAAGTGACTTTATTTATGCCTACCAGAACCCACGAAAGCTGGGACA

>893_Sample_ASV3

CACCGCGGTTATACGAGAGACCCTAGTTGATTAAAGCGGCGTAAGGAGTGGTTATGGAAC

TATTCTTTTAAAGCAGAAAACCTCTCAAACTGTTATACGCACCCAGAGGTCTGAATCCCT

CACACGAAAGTGACTTTATTTATGCCTACCAGAACCCACGAAAGCTGGGACA

>893_Sample_ASV4

CACCGCGGTTATACGAGAGACCCTAGTTGATTAAAGCGGCGTAAAGAGTGGTTATGGAAC

TATTCTTTTAAAGCAGAAAACCTCTCAAACTGTTATACGCACCCAGAGGTCTGAATCCCT

CACACGAAAGTGACTTTATTTATGCCTACCAGAACCCACGAAAGCTGGGACA

>893_2_Sample_ASV1

CACCGCGGTTATACGAGAGACCCTAGTTGATTAAAGCGGCGTAAAGAGTGGTTATGGAAC

TATTCTTTTAAAGCAGAAAACCTCTCAAACTGTTATACGCACCCAGAGGTCTGAATCCCT

CACACGAAAGTGACTTTATTTATGCCTACCAGAACCCACGAAAGCTGGGACA

>893_Sample_ASV5

CACCGCGGTTATACGAGAGACCCTAGTTGATTAAAGCGGCGTAAAGAGTGGTTATGGAAC

TATTCTTTTAAAGCAGAAAACCTCTCAAACTGTTATACACACCCAGAGGTCTGAATCCCT

CACACGAAAGTGACTTTATTTATGCCTACCAGAACCCACGAAAGCTGGGACA

>893_Sample_ASV6

CACCGCGGTTATACGAAAGACCCTAGTTTATAGGTACGGCGTAAAGGGTGGTTAGGGGAC

AAACAAAATAAAGCCAAAGACCTCCTAAGCCGTCATACGCTCACGGAAGCACGAAGCCCA

AACACGAAAGTAGCTTTACCGAACATGCCCGACTCCACGAAAGCTGGGGAA

>893_2_Sample_ASV2

CACCGCGGTTATACGAAAGACCCTAGTTTATAGGTACGGCGTAAAGGGTGGTTAGGGGAC

AAACAAAATAAAGCCAAAGACCTCCTAAGCCGTCATACGCTCACGGAAGCACGAAGCCCA

AACACGAAAGTAGCTTTACCGAACATGCCCGACTCCACGAAAGCTGGGGAA

>893_Sample_ASV7

CACCGCGGTTATACGAAAGACCCTAGTTGATAGCCACGGCGTAAAGGGTGGTTAAGGAAC

CTAAAAAATAAAGCTAAAGACCCTCTAGGCCGTCATACGCATTACGAGGCAAACGAAACC

CCAACACGAAAGTAGCTTTAGACAAATATCCTGATGCCACGAAAGCCAAGAAA

>893_2_Sample_ASV3

CACCGCGGTTATACGAGAGGCCCAAGTTGACAGACACCGGCGTAAAGAGTGGCTAGGGAA

AAATTACTACTAAAGCCGAACACCTTCAGAACTGTCATACGTTCCCGAAGATAAGAAGCC

CCACCACGAAAGTGACTTTATATTACCCGACCCCACGAAAGCTGAAAAA

>893_2_Sample_ASV4

CACCGCGGTTATACGAGAGGCCCAAGTTGACAGACACCGGCGTAAAGAGTGGCTAGGGAA

AAATTACTACTAAAGCCGAACACCTTCAGAACTGTCATACGTTCCCGAAGATAAGAAGCC

CCACCACGAAAGTGACTTTATATTACCCGACCCCACGAAAGCTGTGAAA

>893_Sample_ASV8

CACCGCGGTTATACGAGAGGCCCAAGTTGACAGACACCGGCGTAAAGAGTGGCTAGGGAA

AAATTACTACTAAAGCCGAACACCTTCAGAACTGTCATACGTTCCCGAAGATAAGAAGCC

CCACCACGAAAGTGACTTTATATTACCCGACCCCACGAAAGCTGTGAAA

>893_Sample_ASV9

CACCGCGGTTATACGAGAGGCCCAAGTTGACAGACACCGGCGTAAAGAGTGGCTAGGGAA

AAATTACTACTAAAGCCGAACACCTTCAGAACTGTCATACGTTCCCGAAGATAAGAAGCC

CCACCACGAAAGTGACTTTATATTACCCGACCCCACGAAAGCTAAGAAA

>893_Sample_ASV10

CACCGCGGTTATACGAGAGACCCTAATTGATAGACACGGCGTAAAGAGTGGCTAGGGAAA

AATTACTACTAAAGCCGAACACCTTCAGAACTGTCATACGTTCCCGAAGATAAGAAGCCC

CACCACGAAAGTGACTTTATATTACCCGACCCCACGAAAGCTGTGAAA

>893_2_Sample_ASV5

CACCGCGGTTATACGAGAGGCCCAAGTTGACAGACACCGGCGTAAAGAGTGGCTAGGGAA

AAATTACTACTAAAGCCGAACACCTTCAGAACTGTCATACGTTCCCGAAGATAAGAAGCC

CCACCACGAAAGTGGCTTTATATTACCCGACCCCACGAAAGCTAAGAAA

>893_2_Sample_ASV6

CACCGCGGTTATACGAGAGGCCCAAGTTGACAGACACCGGCGTAAAGAGTGGCTAGGGAA

AAATTACTACTAAAGCCGAACACCTTCAGAACTGTCATACGTTCCCGAAGATAAGAAGCC

CCACCACGAAAGTGGCTTTATATTACCCGACCCCACGAAAGCTGTGAAA

>893_Sample_ASV11

CACCGCGGTTATACGAGAGACCCTAATTGATAGACACGGCGTAAAGAGTGGCTAGGGAAA

AATTACTACTAAAGCCGAACACCTTCAGAACTGTCATACGTTCCCGAAGATAAGAAGCCC

CACCACGAAAGTGGCTTTATATTACCCGACCCCACGAAAGCTGTGAAA

>893_Sample_ASV12

CACCGCGGTTATACGAGAGGCCCAAGTTGACAGACACCGGCGTAAAGAGTGGCTAGGGAA

AAATTACTACTAAAGCCGAACACCCTCAGAACTGTCATACGTTCCCGAAGGTAAGAAGCC

CCACCACGAAAGTGGCTTTATAATACCCGACCCCACGAAAGCTGTGAAA

>893_Sample_ASV13

CACCGCGGTTATACGAGAGGCCCAAGTTGACAGACACCGGCGTAAAGAGTGGCTAGGGAA

AAATTACTACTAAAGCCGAACACCTTCAGAACTGTCATACGTTCCCGAAGATAAGAAGCC

CCACCACGAAAGTGGCTTTATATTACCCGACCCCACGAAAGCTAAGAAA

>893_Sample_ASV14

CACCGCGGTTATACGAGAGGCCCAAGTTGACAGACACCGGCGTAAAGAGTGGCTAGGGAA

AAATTACTACTAAAGCCGAACACCTTCAGAACTGTCATACGTTCCCGAAGATAAGAAGCC

CCACCACGAAAGTGGCTTTATATTACCCGACCCCACGAAAGCTGTGAAA

>893_Sample_ASV15

CACCGCGGTTATACGAGAGGCCCAAGTTGACAGACACCGGCGTAAAGAGTGGCTAAGGAA

AAATTTCTACTAAAGCCGAACACCTTCAGAACTGTCATACGTTCCCGAAGAAAAGAAGCC

CCACCACGAAAGTGGCTTTATATTACCCGACCCCACGAAAGCTGTGAAA

>893_Sample_ASV16

CACCGCGGTTATACGAGAGGCCCAAGTTGACAGACACCGGCGTAAAGAGTGGCTAAGGAA

AAATTTCTACTAAAGCCGAACACCTTCAGAACTGTCATACGTTCCCGAAGATAAGAAGCC

CCACCACGAAAGTGGCTTTATATTACCCGACACCACGAAAGCTGTGAAA

>893_2_Sample_ASV7

CACCGCGGTTATACGAGAGGCCCAAGTTGACAGACACCGGCGTAAAGAGTGGCTAAGGAA

AAATTTCTACTAAAGCCGAACACCTTCAGAACTGTCATACGTTCCCGAAGAAAAGAAGCC

CCACCACGAAAGTGGCTTTATATTACCCGACCCCACGAAAGCTGTGAAA

>893_Sample_ASV17

CACCGCGGTTATACGAGAGGCCCAAGTTGACAGACACCGGCGTAAAGAGTGGCTAAGGAA

AAATTTCTACTAAAGCCGAACACCTTCAGAACTGTCATACGTTCCCGAAGAAAAGGAGCC

CCACCACGAAAGTGGCTTTATATTACCCGACCCCACGAAAGCTGTGAAA

>893_2_Sample_ASV8

CACCGCGGTTATACGAGAGACCCTAGTTGATATATACGGCGTAAAGAGTGGTTTGGGGTT

ATGCCTCCAAAATAAAGCCGAAGATCTCCCAAGCCGTCACACGCATCCCGGAGACACGAA

GCCCTAATACGAAGGTAGCTTTATTAGTACCCCGACGCCACGAAAGCTAAGAAA

>893_2_Sample_ASV9

CACCGCGGTTATACGAGAGACCCTAGTTGATATATACGGCGTAAAGAGTGGTTTGGGGTT

ATGCCTCCAAAATAAAGCCGAAGATCTCCCAAGCCGTCACACGCATCCCGGAGACACGAA

GCCCTAATACGAAGGTAGCTTTATTAATTCCCCGACGCCACGAAAGCTAAGAAA

>893_2_Sample_ASV10

CACCGCGGTTATACGAAAGACCCTAGTTGATAGATCACGGCGTAAAGGGTGGTTAGGGAA

TACAAAAATAAAGCTAAAAATCCTCTAAGCTGTCATACGCTTCCCGAAGACATGAAACCC

AACCACGAAAGTAGCTTTAAACTCTCCTGACACCACGAAAGCCAAGGAA

>893_Sample_ASV18

CACCGCGGTTATACGAAAGACCCTAGTTGATAGATCACGGCGTAAAGGGTGGTTAGGGAA

TACAAAAATAAAGCTAAAAATCCTCTAAGCTGTCATACGCTTCCCGAAGACATGAAACCC

AACCACGAAAGTAGCTTTAAACTCTCCTGACACCACGAAAGCCAAGGAA

>893_2_Sample_ASV11

CACCGCGGTTATACGAGAGGCCCAAGTTGATAAGCCCGGCGTAAAGAGTGGTTATGGAGA

ATAAAACACTAAAGCTAAAGACCCCCTAGGCTGTTATACGCACCTGGCGGCTCGAACCAC

CTATACGAAAGTAGCTTTACCCCCCTTCCACCAGAATCCACGAAAGCTGAAAAA

>893_2_Sample_ASV12

CACCGCGGTTATACGAGAGGCCCAAGTTGATAAGCCCGGCGTAAAGAGTGGTTATGGAGA

ATAAAACACTAAAGCTAAAGACCCCCTAGGCTGTTATACGCACCTGGCGGCTCGAACCAC

CTATACGAAAGTAGCTTTACCCCCCTTCCACCAGAATCCACGACAGCTGGGGCA

>893_2_Sample_ASV13

CACCGCGGTTATACGAGAGGCCCAAGTTGATAAGCCCGGCGTAAAGAGTGGTTATGGAGA

ATAAAACACTAAAGCTAAAGACCCCCTAGGCTGTTATACGCACCTGGCGGCTCGAACCAC

CTATACGAAAGTAGCTTTACCCCCCTTCCACCTGATCCCACGAAAGCTGAAAAA

>893_Sample_ASV19

CACCGCGGTTATACGAGAGGCCCAAGTTGATAAGCCCGGCGTAAAGAGTGGTTATGGAGA

ATAAAACACTAAAGCTAAAGACCCCCTAGGCTGTTATACGCACCTGGCGGCTCGAACCAC

CTATACGAAAGTAGCTTTACCCCCCTTCCACCAGAATCCACGACAGCTGGGGCA

>893_Sample_ASV20

CACCGCGGTTATACGAGAGGCCCAAGTTGATAAGCCCGGCGTAAAGAGTGGTTATGGAGA

ATAAAACACTAAAGCTAAAGACCCCCTAGGCTGTTATACGCACCTGGCGGCTCGAACCAC

CTATACGAAAGTAGCTTTACCCCCCTTCCACCAGAATCCACGAAAGCTAAGAAA

>893_2_Sample_ASV14

CACCGCGGTTATACGAGAGGCCCTAGTTGATAGCCACGGCGCAAAGAGTGGTTAAGGAGC

CCCACTAAATAAAGTCGAACACTTCCTAGGCCGTTATACGTTTTCTAGAAGCACGAAACC

CAATTACACGAAAGCAACTTTATACTAAACAACCTGACCCCACGAAAGCTAAGAAA

>893_2_Sample_ASV15

CACCGCGGTTATACGAGAGGCCCAAGTTGACAGACATCGGCGTAAAGAGTGGTTAAGGAA

ACCCCATTACTAAAGCCGAACGCCCTCAGAACTGTCATACGTTCCCGAAGGTAAGAAGCC

CCACTACGAAAGTGGCTTTAACCACACCCGACCCCACGAAAGCTAAGAAA

>893_2_Sample_ASV16

CACCGCGGTTATACGAGAGGCCCAAGTTGACAGACATCGGCGTAAAGAGTGGTTAAGGAA

ACCCCATTACTAAAGCCGAACGCCCTCAGAACTGTCATACGTTCCCGAAGGTAAGAAGCC

CCACTACGAAAGTGGCTTTAACCACACCCGACCCCACGAAAGCTGTGAAA

>893_Sample_ASV21

CACCGCGGTTATACGAGAGGCCCAAGTTGACAGACATCGGCGTAAAGAGTGGTTAAGGAA

ACCCCATTACTAAAGCCGAACGCCCTCAGAACTGTCATACGTTCCCGAAGGTAAGAAGCC

CCACTACGAAAGTGGCTTTAACCACACCCGACCCCACGAAAGCTGTGAAA

>893_2_Sample_ASV17

CACCGCGGTTATACGAGAGGCCCAAGTTGACAGACACCGGCGTAAAGAGTGGTTAAGGAA

ATTCATCAACTAAAGCCGAATGCCCTCAGGACTGTTATACGTTTGCGAAGGTAAGAAGCC

CTACTACGAAAGTGGCTTTATTCCACCTGATTCCACGAAAGCTGCGGAA

>893_Sample_ASV22

CACCGCGGTTATACGAGAGGCCCAAGTTGACAGACACCGGCGTAAAGAGTGGTTAAGGAA

ATTCATCAACTAAAGCCGAATGCCCTCAGGACTGTTATACGTTTGCGAAGGTAAGAAGCC

CTACTACGAAAGTGGCTTTATTCCACCTGATTCCACGAAAGCTGCGGAA

>893_Sample_ASV23

CACCGCGGTTATACGAGAGGCCCCAGTTGATATTAGCGGCGTAAAGGGTGGTTAAGGACT

TTAATAACTAAAGCTAAACACTTCCCCGGCTGTTGTACGTAGACCGGAAGCTTGAAGCCC

AGACACGAAAGTAGCTTTATTCCCCCTGACCCCACGAAAGCTGAGAAA

>893_Sample_ASV24

CACCGCGGTTATACGAGAGACCCCAGTTGATAGCTACGGCGTAAAGAGTGGTTAAGGACC

CCATTTAATAAAGCCAAAGACCCCCCAAGCCGTCATACGCACACGAGGGCGCGAAGCTCG

CACACGAAAGTAGCTCTAATTTACCCGACGCCACGAAAGCTAAGGAA

>893_2_Sample_ASV18

CACCGCGGTTATACGAGAGGCCCTAGTTGATAAATACGGCGTAAAGAGTGGTTAGAGGAC

TCCCATAATAAAGCCAAAGACCCCCCGGGCCGTCACACGCATACGAGGGCACGAAGTTCA

CACACGAAAGTAGCTTTAAATTACCCGACGCCACGAAAGCTAAGAAA

>893_Sample_ASV25

CACCGCGGTTATACGAGAAACCCTAGTTGACAAATACGGCGTAAAGAGTGGTTAAGGGGT

ATCAAAAATAAAGTCAAACATTTTCCTAGTTGTTATAAGCATTTCGAGAACATGAAGCCC

AACCACGAAAGTAACTTTACCTTCACCTGACCCCACGAAAGCTAAGAAA

>893_2_Sample_ASV19

CACCGCGGTTATACGAGAGGCCCCAGTTGATAAACACGGCGTAAAGGGTGGTTAAGGGAG

CATAAGAATAAAGCCGAAGGGCCCTTTGGCCGTTATACGCTTCTAGGTGTCCGAAGCCCA

AACACGAAAGTAGCTTTAGTTAAGCCCACCTGACTCCACGAAAACTGAGAAA

>893_2_Sample_ASV20

CACCGCGGTTATACGAGAGACCCTAGTTGATAAATACGGCGTAAAGAGTGGTTATGGGAA

AAACAAATAAAGTCAAACAACCTCTTAGCTGTTATACGCATTATGAGAGCATGAAGCCCC

CTCACGAAAGTAACTTTAATACCTCCTGACCCCACGAAAGCTAAGAAA

>893_Sample_ASV26

CACCGCGGTTATACGAGAGACCCTAGTTGATAAATACGGCGTAAAGAGTGGTTATGGGAA

AAACAAATAAAGTCAAACAACCTCTTAGCTGTTATACGCATTATGAGAGCATGAAGCCCC

CTCACGAAAGTAACTTTAATACCTCCTGACCCCACGAAAGCTAAGAAA

>893_Sample_ASV27

CACCGCGGTTATACGAGAGGCTCAAGTTGATAAACCCCGGCGTAAAGAGTGGTTAAGGAA

AAATAGAAACTAAAGCCGAATGCTTTCAAGACTGTTATACGTTCCCGAGAGTAAGAAGAC

CAATTACGAAAGTGGCTTTACTTTACCTGACCCCACGAAAGCTACGACA

>893_2_Sample_ASV21

CACCGCGGTTATACGAGAGGCCCAAGTTGACAAACCCCGGCGTAAAGAGTGGTTAAGGGA

GATCAAAACTAAAGCCGAATGCTTTCAAAGCTGTTATACGCTTCCGAAAGTAAGAAGCTC

AATCACGAAAGTGGCTTTACTTTACCTGACCCCACGAAAGCTACGACA

>893_2_Sample_ASV22

CACCGCGGTTATACGAGAGGCTCAAGTTGATGAACCCCGGCGTAAAGAGTGGTTAAGGGA

GATCAAAACTAAAGCCGAATGCTTTCAAAGCTGTTATACGCTTCCGAAAGTAAGAAGCTC

AATCACGAAAGTGGCTTTACTTTACCTGACCCCACGAAAGCTACGACA

>893_Sample_ASV28

CACCGCGGTTATACGAGAGACCCTAATTGATAGACACGGCGTAAAGAGTGGTTTGGGAAA

GCCCAAAAATAAAGCTAAAGACCTTCTAAGCCGTCACACGCACGCCGAAGGCACGAAGCC

CTGACACGAAAGTAGCTTTACTATTACCCCCGACGCCACGAAAGCTAAGAAA

>893_2_Sample_ASV23

CACCGCGGTTATACGAAAGACCCTAGTTGATGGATCACGGCGTAAAGGGTGGTTAAGGGA

TATAAAAATAAAGCTAAAAATCCTCTTAGCTGTCATACGCTTTCCGAAGACATGAGGCCC

AATTACGAAAGTAGCTTTAAACCACCCTGACACCACGAAAGCCAAGAAA

>893_Sample_ASV29

CACCGCGGTTATACGAAAGACCCTAGTTGATGGATCACGGCGTAAAGGGTGGTTAAGGGA

TATAAAAATAAAGCTAAAAATCCTCTTAGCTGTCATACGCTTTCCGAAGACATGAGGCCC

AATTACGAAAGTAGCTTTAAACCACCCTGACACCACGAAAGCCAAGAAA

>893_Sample_ASV30

CACCGCGGTTATACGAAAGACCCTAGTTTATAGATGCGGCGTAAAGGGTGGTTAAGGAAA

ACCTAAAATAATGCTAAAGACTCTCAAAGCTGTTATACGCTTCATGAGCACACGAAATCC

AAATACGAAAGTTGCCTTAATAAACTTTGCCTGACCCCACGAAAGCTGAAAAA

>893_2_Sample_ASV24

CACCGCGGTTATACGAAAGACCCCAGTTTATAGATGCGGCGTAAAGGGTGGTTAAGAGAA

AATCAAAATAAGGCCAAAGATCCCCTAAGCAGTCATACGCTCCACGAGGACACGAAACCC

AAATACGAAAGTTGCCTTAATAAACTTCACCTGATCCCACGAAAGCTGAAAAA

>893_2_Sample_ASV25

CACCGCGGTTATACGAGAGACTCTAGTTGATACCAACGGCGTAAAGAGTGGTTTGGGATG

CCCCTTAAAATAAAGCCAAAGACCTCCCAAGCCGTCAAACGCACCCCGGAGGCACGAAGC

CCCAACGCGAAAGCAACTTTACCTCCCTCCCGACGCCACGAAAGCTAAGAAA

>893_2_Sample_ASV26

CACCGCGGTTATACGAGAGGCCCAAGTTGACAAACCCCGGCGTAAAGAGTGGTTAAGGAA

AATTTAAAACTAAAGCCGAATGCCCCCAAAGCTGTTATACGCACCCGAGGGTAAGAAGCC

CAATCACGAAAGTGGCTTTACACTACCTGAACCCACGAAAGCTATGAAA

>893_2_Sample_ASV27

CACCGCGGTTATACGAGAGGCCCAAGTTGACAAACCCCGGCGTAAAGAGTGGTTAAGGAA

AATTTAAAACTAAAGCCGAATGCCCCCAAAGCTGTTATACGCACCCGAGGATAAGAAGCC

CAATCACGAAAGTGGCTTTACACTACCTGAACCCACGAAAGCTATGAAA

>893_Sample_ASV31

CACCGCGGTTATACGAGAGACCCTAGTTGATAGCTACGGCGTAAAGCGTGGTTAGGAGAA

CAAGATAAATAAAGTCAAACAATCTCTCGGCCGTTATACGTTATCTGAGAATATGAAGTC

CTACCACGAAAGTAACTTTAATTTTTCTGACCCCACGAAAACTGAGGAA

>893_Sample_ASV32

CACCGCGGTTAAACGAGAGGCCCTAGTTGATAGTACAACGGCGTAAAGGGTGGTTAAGGA

TAGTGAAATAATAAAGTCGAATGGCCCTTTGGCTGTCATACGCTTCTAGGAGTCCGAAGC

CCAATATACGAAAGTAACTTTAAGAGCGCCCACCTGACCCCACGAAAACTGAGAAA

>893_Sample_ASV33

CACCGCGGTCATACGATTAACCCAAGTTAATAGGCACACGGCGTAAAGCGTGTTTAAGCA

CTACGCCAAATAAAGTTAAACTCCAATTAAGCTGTAAAAAGCCATAATCAAGACGAAAAT

AAATAACGAAAGTAACTTTACAACCGCTGAGACACGATAGCTAAGATC

>893_Sample_ASV34

CACCGCGGTCATACGATTAACCCAAGTTAATAGGCACACGGCGTAAAGCGTGTTTAAGCA

CTATGCCAAATAAAGTTAAACTCCAATTAAGCTGTAAAAAGCCATAATCAAGACGAAAAT

AAATAACGAAAGTAACTTTACAACCGCTGAAACACGATAGCTAAGATC

>893_Sample_ASV35

CACCGCGGTCATACGATTAACCCAAGTTAATAGGCACACGGCGTAAAGCGTGTTTAAGCA

CTACGCCAAATAAAGTTAAACTCCAATTAAGCTGTAAAAAGCCATAATCAAGACGAAAAT

AAATAACGAAAGTAACTTTACAACCGCTGAAACACGATAGCTAAGATC

>893_2_Sample_ASV28

CACCGCGGTTATACGAGAGGCCCTAGTTGATAACTACCGGCGTAAAGAGTGGTTATGGAA

AAATATTTAATAAAGCCGAACACCCCCTCAGCCGTCATACGCACCTGGGAGCACGAAGAC

CTACTGCGAAAGCAGCTTTAATTATGCCTGACCCCACGACAGCTAAGAAA

>893_2_Sample_ASV29

CACCGCGGTTATACGAAAGACCCTAGTTTATAGACACGGCGTAAAGGGTGGTTCGGGACT

AAACAAAATAAAGCCAAAGACCTCCTAAGCCGTCATACGCTCACGGAAGCACGAAGCCCA

AGTACGAAAGTAGCTTTACAAAACATGCCTGACTCCACGAAAGCTAGGAAA

>893_Sample_ASV36

CACCGCGGTTATACGAAAGACCCTAGTTGATAGGCACGGCGTAAAGGGTGGTTAGGGATA

TATTAAAAATAAAGTTAAAGAGCCTCTAAGCTGTCGCACGCATCCCGAGAGCTCGAGACC

CAGACACGAAAGTAACTTTAAAATACCTCACCTGACCCCACGAAAGCTAAGAAA

>893_Sample_ASV37

CACCGCGGTTATACGAAAGACCCTAGTTGATAGGCACGGCGTAAAGGGTGGTTAGGGATA

TATCAAAAATAAAGTTAAAGAGCCTCTAAGCTGTCGCACGCATCCCGAGAGCTCGAGACC

CAGACACGAAAGTAACTTTAAAATACCTCACCTGACCCCACGAAAGCTAAGAAA

>893_Sample_ASV38

CACCGCGGTTATACGAGAGACCCTAATTGATAGGCACGGCGTAAAGGGTGGTTAGGGATA

TATCAAAAATAAAGTTAAAGAGCCTCTAAGCTGTCGCACGCATCCCGAGAGCTCGAGACC

CAGACACGAAAGTAACTTTAAAATACCTCACCTGACCCCACGAAAGCTAAGAAA

>893_Sample_ASV39

CACCGCGGTTATACGAGAGACCCTAATTGATAGACACGGCGTAAAGGGTGGTTAGGGATA

TATCAAAAATAAAGTTAAAGAGCCTCTAAGCTGTCGCACGCATCCCGAGAGCTCGAGACC

CAGACACGAAAGTAACTTTAAAATACCTCACCTGACCCCACGAAAGCTAAGAAA

>893_2_Sample_ASV30

CACCGCGGTTATACGAGAGGCCCAAGTTGATACTCCACGGCGTAAAGAGTGGTTAGAAAA

GACCTACTACTAAAGCCGAACGCCTTCAAAGCTGTTATACGCATCCGAAGGTGAGAAGCC

CACCCACGAAAGTGGCTTTACAACCTTGACTCCACGAAAGCTATGACA

>893_Sample_ASV40

CACCGCGGTTATACGAGAGACCCCAGTTGATAAACACGGCGTAAAGAGTGGTTAAGGGCA

CAAAACAAATAAAGTCAAACAACCTCTTAGCTGTTATACGCATTATGAGGATATGAAGCC

CCCCTACGAAAGTAACTTTACCAACCCCTGACCCCACGAAAGCTAAGAAA

>893_2_Sample_ASV31

CACCGCGGTTATACGAGAGACCCCAGTTGATAAACACGGCGTAAAGAGTGGTTAAGGGCA

CAAAACAAATAAAGTCAAACAACCTCTTAGCTGTTATACGCATTATGAGGATATGAAGCC

CCCCTACGAAAGTAACTTTACCAACCCCTGACCCCACGAAAGCTAAGAAA

>893_Sample_ASV41

CACCGCGGTTATACGAGAGACCCTAGTTGATATATACGGCGTAAAGAGTGGTTTGGAATA

ACCAAGTAATATAGCCAAAGACCTCCCAAGCCGTCACACGCACCCCGGAGGCACGAAGCC

CAAACACGAAAGTAGCTTTATTAACTTACCGACGCCACGAAAGCTAAGAAA

>893_Sample_ASV42

CACCGCGGTTATACGAAAGACCCTAGTTTATAGGCACGGCGTAAAGGGTGGTTAGGGGAT

AAACAAAATAAAGCCAAAGACCTTCTAAGTCGTCATACACTCATGAAGGCACGAAGCCCA

AATACGAAAGTAACTTTACAACTTAGACCCGACTCCACGAAAGCTAAGAAA

>893_Sample_ASV43

CACCGCGGTTATACGAAAGACCCTAGTTTATAGGCACGGCGTAAAGGGTGGTTAGGGGAT

AAACAAAATAAAGCCAAAGACCTTCTAAGTCGTCATACACTCATGAAGGCACGAAGCCCA

AATACGAAAGTAACTTTACAACTTAGACCCGACTCCACGAAAGCTGGGGGA

>893_2_Sample_ASV32

CACCGCGGTTATACGAAAGACCCTAGTTTATAGGCACGGCGTAAAGGGTGGTTAGGGGAT

AAACAAAATAAAGCCAAAGACCTTCTAAGTCGTCATACACTCATGAAGGCACGAAGCCCA

AATACGAAAGTAACTTTACAACTTAGACCCGACTCCACGAAAGCTGGGGGA

>893_2_Sample_ASV33

CACCGCGGTTATACGAAAGACCCTAGTTTATAGGCACGGCGTAAAGGGTGGTTAGGGGAT

AAACAAAATAAAGCCAAAGACCTTCTAAGTCGTCATACACTCATGAAGGCACGAAGCCCA

AATACGAAAGTAACTTTACAACTTAGACCCGACTCCACGAAAGCTAAGAAA

>893_Sample_ASV44

CACCGCGGTCATACGATTAACCCAAGTTAATAGGCACACGGCGTAAAGCGTGTTAAAGCA

CTATACTAAATAAAGTTAAATTCCAATTAAGCTGTAAAAAGCCATAATTGCAACAAAAAT

AAACAACGAAAGTAACTTTACAGTCGCTGAAACACGATAGCTAGGACC

>893_2_Sample_ASV34

CACCGCGGTTATACGAGAGGCCCAAGTTGACAAACTCCGGCGTAAAGCGTGGTTAAGGCA

CACTCAAAAACTAAAGCCGAATATCTTCAGAGCAGTCATACGCATCCGAAGACACGAAGC

CCCACCACGAAAGTGGCTTTATGACACCTGACCCCACGAAAGCTAGGACA

>893_Sample_ASV45

CACCGCGGTTATACGAGAGACCCTAATTGATAGACTACGGCGTAAAGAGTGGTTTAGAAC

AAAAATTAATAAAGCCAAAGATCCTCCTGGCCGTCGCACGCATTTCGAGGATACGAAGCC

CCACTACGAAAGTAGCTTTACTACAACTTTTCTGACCCCACGAAAGCTAAGAAA

>893_2_Sample_ASV35

CACCGCGGTTATACGAGAGACCCTAATTGATAGACTACGGCGTAAAGAGTGGTTTAGAAC

AAAAATTAATAAAGCCAAAGATCCTCCTGGCCGTCGCACGCATTTCGAGGATACGAAGCC

CCACTACGAAAGTAGCTTTACTACAACTTTTCTGACCCCACGAAAGCTAAGAAA

>893_Sample_ASV46

CACCGCGGTTATACGAAAGACCCTAGTTGATAGATCACGGCGTAAAGGGTGGTTAAGGAG

AACAAAAATAAAGCTAAAAATCCTCTAAGCTGTCATACGCTTTCCGAAGACATGAGACCC

AACCACGAAAGTAACTTTAAACTTTCCTGACACCACGAAAGCCAAGAAA

>894_Sample_ASV1

CACCGCGGTTATACGAAAGACCCTAGTTGATGGGTGCGGCGTAAAGGGTGGTTAGGGACT

ACAAAAAATAGAGCTAAAGATCCTCTAGGTCGTCATACACATCACGAGGAAACGAAGCCC

AAATACGAAAGTAGCTCTAAATATAATCCTGACGCCACGAAAGCCAAGAAA

>894_Sample_ASV2

CACCGCGGTTATACGAGAGACCCTAGTTGATTAAAGCGGCGTAAAGAGTGGTTATGGAAC

TATTCTTTTAAAGCAGAAAACCTCTCAAACTGTTATACGCACCCAGAGGTCTGAATCCCT

CACACGAAAGTGACTTTATTTATGCCTACCAGAACCCACGAAAGCTGGGACA

>894_Sample_ASV3

CACCGCGGTTATACGAAAGACCCTAGTTTATAGGTACGGCGTAAAGGGTGGTTAGGGGAC

AAACAAAATAAAGCCAAAGACCTCCTAAGCCGTCATACGCTCACGGAAGCACGAAGCCCA

AACACGAAAGTAGCTTTACCGAACATGCCCGACTCCACGAAAGCTGGGGAA

>894_Sample_ASV4

CACCGCGGTTATACGAGAGGCCCAAGTTGACAGACACCGGCGTAAAGAGTGGCTAGGGAA

AAATTACTACTAAAGCCGAACACCTTCAGAACTGTCATACGTTCCCGAAGATAAGAAGCC

CCACCACGAAAGTGACTTTATATTACCCGACCCCACGAAAGCTGTGAAA

>894_Sample_ASV5

CACCGCGGTTATACGAGAGGCCCAAGTTGACAGACACCGGCGTAAAGAGTGGCTAGGGAA

AAATTACTACTAAAGCCGAACACCTTCAGAACTGTCATACGTTCCCGAAGATAAGAAGCC

CCACCACGAAAGTGGCTTTATATTACCCGACCCCACGAAAGCTGTGAAA

>894_Sample_ASV6

CACCGCGGTTATACGAGAGGCCCAAGTTGACAGACACCGGCGTAAAGAGTGGCTAGGGAA

AAATTACTACTAAAGCCGAACACCTTCAGAACTGTCATACGTTCCCGAAGATAAGAAGCC

CCACCACGAAAGTGGCTTTATATTACCCGACCCCACGAAAGCTAAGAAA

>894_Sample_ASV7

CACCGCGGTTATACGAGAGGCCCAAGTTGACAGACACCGGCGTAAAGAGTGGCTAAGGAA

AAATTTCTACTAAAGCCGAACACCTTCAGAACTGTCATACGTTCCCGAAGATAAGAAGCC

CCACCACGAAAGTGGCTTTATATTACCCGACACCACGAAAGCTGTGAAA

>894_Sample_ASV8

CACCGCGGTTAAACGAGAGACCCTAGTTGATCATCACGGCGTAAAGAGTGGTTAGGGGAT

TATCATAAATAAAGCCAAAGACCTCCCAAGCTGTCGCACGCATTCCGGAGGCGCGAAGCC

CACACACGAAAGTAGCTTTAACTATTGACCCTGATGCCACGAAAGCTAAGAAA

>894_Sample_ASV9

CACCGCGGTTATACGAGAGACCCTAGTTGATATATACGGCGTAAAGAGTGGTTTGGGGTT

ATGCCTCCAAAATAAAGCCGAAGATCTCCCAAGCCGTCACACGCACCCCGGAGACACGAA

GCCCTAATACGAAGGTAGCTTTATTAATTCCCCGACGCCACGAAAGCTAAGAAA

>894_Sample_ASV10

CACCGCGGTTATACGAGAGACCCTAGTTGATATATACGGCGTAAAGAGTGGTTTGGGGTT

ATGCCTCCAAAATAAAGCCGAAGATCTCCCAAGCCGTCACACGCATCCCGGAGACACGAA

GCCCTAATACGAAGGTAGCTTTATTAGTACCCCGACGCCACGAAAGCTAAGAAA

>894_Sample_ASV11

CACCGCGGTTATACGAGAGACCCTAGTTGATATATACGGCGTAAAGAGTGGTTTGGGATA

TTTCTCCAAAATAAAGCCGAAGATCTCCCAAGCCGTCACACGCATCCCGGAGACACGAAG

CCCTAATACGAAGGTAGCTTTATTAGTACCCCGACGCCACGAAAGCTAAGAAA

>894_Sample_ASV12

CACCGCGGTTATACGAAAGACCCTAGTTTATAGGCACGGCGTAAAGGGTGGTTAGGGAAT

ACAAAAATAAAGCTAAAAATCCTCTAAGCTGTCATACGCTTCCCGAAGACATGAAACCCA

ACCACGAAAGTAGCTTTAAACTCTCCTGACACCACGAAAGCCAAGGAA

>894_Sample_ASV13

CACCGCGGTTATACGAAAGACCCTAGTTGATAGATCACGGCGTAAAGGGTGGTTAGGGAA

TACAAAAATAAAGCTAAAAATCCTCTAAGCTGTCATACGCTTCCCGAAGACATGAAACCC

AACCACGAAAGTAGCTTTAAACTCTCCTGACACCACGAAAGCCAAGGAA

>894_Sample_ASV14

CACCGCGGTTATACGAGAGGCCCAAGTTGATAAGCCCGGCGTAAAGAGTGGTTATGGAGA

ATAAAACACTAAAGCTAAAGACCCCCTAGGCTGTTATACGCACCTGGCGGCTCGAACCAC

CTATACGAAAGTAGCTTTACCCCCCTTCCACCAGAATCCACGACAGCTGGGGCA

>894_Sample_ASV15

CACCGCGGTTATACGAGAGGCCCTAGTTGATAGCCACGGCGCAAAGAGTGGTTAAGGAGC

CCCACTAAATAAAGTCGAACACTTCCTAGGCCGTTATACGTTTTCTAGAAGCACGAAACC

CAATTACACGAAAGCAACTTTATACTAAACAACCTGACCCCACGAAAGCTAAGAAA

>894_Sample_ASV16

CACCGCGGTTATACGAGAGGCCCAAGTTGACAGACATCGGCGTAAAGAGTGGTTAAGGAA

ACCCCATTACTAAAGCCGAACGCCCTCAGAACTGTCATACGTTCCCGAAGGTAAGAAGCC

CCACTACGAAAGTGGCTTTAACCACACCCGACCCCACGAAAGCTGTGAAA

>894_Sample_ASV17

CACCGCGGTTATACGAGAGGCCCAAGTTGATAGATATCGGCGTAAAGAGTGGTTAAGGAA

ACCCCGTTACTAAAGCCGAACGCCCTCAGAACTGTCATACGTTCCCGAAGGTAAGAAGCC

CCACTACGAAAGTGGCTTTAACCACACCCGACCCCACGAAAGCTGTGAAA

>894_Sample_ASV18

CACCGCGGTTATACGAGAGGCCCAAGTTGACAGACACCGGCGTAAAGAGTGGTTAAGGAA

ATTCATCAACTAAAGCCGAATGCCCTCAGGACTGTTATACGTTTGCGAAGGTAAGAAGCC

CTACTACGAAAGTGGCTTTATTCCACCTGATTCCACGAAAGCTGGGGGA

>894_Sample_ASV19

CACCGCGGTTATACGAGAGGCCCAAGTTGACAGACACCGGCGTAAAGAGTGGTTAAGGAA

ATTCATCAACTAAAGCCGAATGCCCTCAGGACTGTTATACGTTTGCGAAGGTAAGAAGCC

CTACTACGAAAGTGGCTTTATTCCACCTGATTCCACGAAAGCTGCGGAA

>894_Sample_ASV20

CACCGCGGTTATACGAGAGGCCCCAGTTGATAAACACGGCGTAAAGGGTGGTTAAGGGAG

CATAAGAATAAAGCCGAAGGGCCCTTTGGCCGTTATACGCTTCTAGGTGTCCGAAGCCCA

AACACGAAAGTAGCTTTAGTTAAGCCCACCTGACTCCACGAAAACTGAGAAA

>894_Sample_ASV21

CACCGCGGTTATACGAGAGACCCTAGTTGATAAATACGGCGTAAAGAGTGGTTATGGGAA

AAACAAATAAAGTCAAACAACCTCTTAGCTGTTATACGCATTATGAGAGCATGAAGCCCC

CTCACGAAAGTAACTTTAATACCTCCTGACCCCACGAAAGCTAAGAAA

>894_Sample_ASV22

CACCGCGGTTATACGAGAGGCTCAAGTTGATAAACCCCGGCGTAAAGAGTGGTTAAGGAA

AAATAGAAACTAAAGCCGAATGCTTTCAAGACTGTTATACGTTCCCGAGAGTAAGAAGAC

CAATTACGAAAGTGGCTTTACTTTACCTGACCCCACGAAAGCTACGACA

>894_Sample_ASV23

CACCGCGGTTATACGAGAGGCTCAAGTTGATGAACCCCGGCGTAAAGAGTGGTTAAGGGA

GATCAAAACTAAAGCCGAATGCTTTCAAAGCTGTTATACGCTTCCGAAAGTAAGAAGCTC

AATCACGAAAGTGGCTTTACTTTACCTGACCCCACGAAAGCTACGACA

>894_Sample_ASV24

CACCGCGGTTATACGAGAGGCCCAAGTTGACAAACCCCGGCGTAAAGAGTGGTTAAGGGA

GATCAAAACTAAAGCCGAATGCTTTCAAAGCTGTTATACGCTTCCGAAAGTAAGAAGCTC

AATCACGAAAGTGGCTTTACTTTACCTGACCCCACGAAAGCTACGACA

>894_Sample_ASV25

CACCGCGGTTATACGAGAGACCCTAATTGATAGACACGGCGTAAAGAGTGGTTTGGGAAA

GCCCAAAAATAAAGCTAAAGACCTTCTAAGCCGTCACACGCACGCCGAAGGCACGAAGCC

CTGACACGAAAGTAGCTTTACTATTACCCCCGACGCCACGAAAGCTAAGAAA

>894_Sample_ASV26

CACCGCGGTTATACGAAAGACCCTAGTTGATGGATCACGGCGTAAAGGGTGGTTAAGGGA

TATAAAAATAAAGCTAAAAATCCTCTTAGCTGTCATACGCTTTCCGAAGACATGAGGCCC

AATTACGAAAGTAGCTTTAAACCACCCTGACACCACGAAAGCCAAGAAA

>894_Sample_ASV27

CACCGCGGTTATACGAAAGACCCCAGTTTATAGATGCGGCGTAAAGGGTGGTTAAGAGAA

AATCAAAATAAGGCCAAAGATCCCCTAAGCAGTCATACGCTCCACGAGGACACGAAACCC

AAATACGAAAGTTGCCTTAATAAACTTCACCTGATCCCACGAAAGCTGAAAAA

>894_Sample_ASV28

CACCGCGGTTATACGAGAGGCTCAAGTTGATGAACCCCGGCGTAAAGAGTGGTTAAGGAA

AATTTAAAACTAAAGCCGAATGCCCCCAAAGCTGTTATACGCACCCGAGGATAAGAAGCC

CAATCACGAAAGTGGCTTTACACTACCTGAACCCACGAAAGCTATGAAA

>894_Sample_ASV29

CACCGCGGTTATACGAGAGGCCCAAGTTGACAAACCCCGGCGTAAAGAGTGGTTAAGGAA

AATTTAAAACTAAAGCCGAATGCCCCCAAAGCTGTTATACGCACCCGAGGATAAGAAGCC

CAATCACGAAAGTGGCTTTACACTACCTGAACCCACGAAAGCTATGAAA

>894_Sample_ASV30

CACCGCGGTTATACGAGAGGCCCAAGTTGACAAACCCCGGCGTAAAGAGTGGTTAAGGAA

AATTTAAAACTAAAGCCGAATGCCCCCAAAGCTGTTATACGCACCCGAGGGTAAGAAGCC

CAATCACGAAAGTGGCTTTACACTACCTGAACCCACGAAAGCTATGAAA

>894_Sample_ASV31

CACCGCGGTTATACGAGAGGCCCTAGTTGATAACTACCGGCGTAAAGAGTGGTTATGGAA

AAATATTTAATAAAGCCGAACACCCCCTCAGCCGTCATACGCACCTGGGAGCACGAAGAC

CTACTGCGAAAGCAGCTTTAATTATGCCTGACCCCACGACAGCTAAGAAA

>894_Sample_ASV32

CACCGCGGTTATACGAAAGACCCTAGTTTATAGACACGGCGTAAAGGGTGGTTCGGGACT

AAACAAAATAAAGCCAAAGACCTCCTAAGCCGTCATACGCTCACGGAAGCACGAAGCCCA

AGTACGAAAGTAGCTTTACAAAACATGCCTGACTCCACGAAAGCTAGGAAA

>894_Sample_ASV33

CACCGCGGTTATACGAAAGACCCTAGTTGATAGGCACGGCGTAAAGGGTGGTTAGGGATA

TATTAAAAATAAAGTTAAAGAGCCTCTAAGCTGTCGCACGCATCCCGAGAGCTCGAGACC

CAGACACGAAAGTAACTTTAAAATACCTCACCTGACCCCACGAAAGCTAAGAAA

>894_Sample_ASV34

CACCGCGGTTATACGAGAGGCCCAAGTTGATACTCCACGGCGTAAAGAGTGGTTAGAAAA

GACCTACTACTAAAGCCGAACGCCTTCAAAGCTGTTATACGCATCCGAAGGTGAGAAGCC

CACCCACGAAAGTGGCTTTACAACCTTGACTCCACGAAAGCTATGACA

>894_Sample_ASV35

CACCGCGGTTATACGAGAGACCCCAGTTGATAAACACGGCGTAAAGAGTGGTTAAGGGCA

CAAAACAAATAAAGTCAAACAACCTCTTAGCTGTTATACGCATTATGAGGATATGAAGCC

CCCCTACGAAAGTAACTTTACCAACCCCTGACCCCACGAAAGCTAAGAAA

>894_Sample_ASV36

CACCGCGGTTATACGAAAGACCCTAGTTTATAGGCACGGCGTAAAGGGTGGTTAGGGGAT

AAACAAAATAAAGCCAAAGACCTTCTAAGTCGTCATACACTCATGAAGGCACGAAGCCCA

AATACGAAAGTAACTTTACAACTTAGACCCGACTCCACGAAAGCTGGGGGA

>897_Sample_ASV1

CACCGCGGTTATACGAAAGACCCTAGTTGATGGGTGCGGCGTAAAGGGTGGTTAGGGACT

ACAAAAAATAGAGCTAAAGATCCTCTAGGTCGTCATACACATCACGAGGAAACGAAGCCC

AAATACGAAAGTAGCTCTAAATATAATCCTGACGCCACGAAAGCCAAGAAA

>897_Sample_ASV2

CACCGCGGTTATACGAGAGACCCTAGTTGATTAAAGCGGCGTAAAGAGTGGTTATGGAAC

TATTCTTTTAAAGCAGAAAACCTCTCAAACTGTTATACGCACCCAGAGGTCTGAATCCCT

CACACGAAAGTGACTTTATTTATGCCTACCAGAACCCACGAAAGCTAAGAAA

>897_Sample_ASV3

CACCGCGGTTATACGAGAGACCCTAGTTGATTAAAGCGGCGTAAAGAGTGGTTATGGAAC

TATTCTTTTAAAGCAGAAAACCTCTCAAACTGTTATACGCACCCAGAGGTCTGAATCCCT

CACACGAAAGTGACTTTATTTATGCCTACCAGAACCCACGAAAGCTGGGACA

>897_Sample_ASV4

CACCGCGGTTATACGAAAGACCCTAGTTGATAGCCACGGCGTAAAGGGTGGTTAAGGAAC

CTAAAAAATAAAGCTAAAGACCCTCTAGGCCGTCATACGCATTACGAGGCAAACGAAACC

CCAACACGAAAGTAGCTTTAGACAAATATCCTGATGCCACGAAAGCCAAGAAA

>897_Sample_ASV5

CACCGCGGTTATACGAGAGGCCCAAGTTGACAGACACCGGCGTAAAGAGTGGCTAGGGAA

AAATTACTACTAAAGCCGAACACCTTCAGAACTGTCATACGTTCCCGAAGATAAGAAGCC

CCACCACGAAAGTGACTTTATATTACCCGACCCCACGAAAGCTGTGAAA

>897_Sample_ASV6

CACCGCGGTTATACGAGAGGCCCAAGTTGACAGACACCGGCGTAAAGAGTGGCTAGGGAA

AAATTACTACTAAAGCCGAACACCTTCAGAACTGTCATACGTTCCCGAAGATAAGAAGCC

CCACCACGAAAGTGACTTTATATTACCCGACCCCACGAAAGCTAAGAAA

>897_Sample_ASV7

CACCGCGGTTATACGAGAGGCCCAAGTTGACAGACACCGGCGTAAAGAGTGGCTAGGGAA

AAATTACTACTAAAGCCGAACACCTTCAGAACTGTCATACGTTCCCGAAGATAAGAAGCC

CCACCACGAAAGTGGCTTTATATTACCCGACCCCACGAAAGCTAAGAAA

>897_Sample_ASV8

CACCGCGGTTATACGAGAGGCCCAAGTTGACAGACACCGGCGTAAAGAGTGGCTAGGGAA

AAATTACTACTAAAGCCGAACACCTTCAGAACTGTCATACGTTCCCGAAGATAAGAAGCC

CCACCACGAAAGTGGCTTTATATTACCCGACCCCACGAAAGCTGTGAAA

>897_Sample_ASV9

CACCGCGGTTATACGAGAGGCCCAAGTTGACAGACACCGGCGTAAAGAGTGGCTAGGGAA

AAATTACTACTAAAGCCGAACACCCTCAGAACTGTCATACGTTCCCGAAGGTAAGAAGCC

CCACCACGAAAGTGGCTTTATAATACCCGACCCCACGAAAGCTAAGAAA

>897_Sample_ASV10

CACCGCGATTATACGAGAGGCCCAAGTTGACAGACATCGGCGTAAAGAGTGGTTAAGGAA

AAATTACTACTAAAGCCGAACACCTTCAGAACTGTCATACGTTCCCGAAGATAAGAAGCC

CCACCACGAAAGTGGCTTTATATTACCCGACCCCACGAAAGCTGTGAAA

>897_Sample_ASV11

CATGGCGATTATACGAGAGGCCCAAGTTGACAGACACTGGCGTAAAGAGTGGCTAAGGAA

AAACTACTACTAAAGCCGAACACCTTCAGAACTGTCATACGTTCCCGAAGATAAGAAGCC

CCACCACGAAAGTGGCTTTATATTACCCGACCCCACGAAAGCTGTGAAA

>897_Sample_ASV12

CATGGCGATTATACGAGAGGCCCAAGTTGACAGACACTGGCGTAAAGAGTGGCTAAGGAA

AAATTACTACTAAAGCCGAACACCTTCAGAACTGTCATACGTTCCCGAAGATAAGAAGCC

CCACCACGAAAGTGGCTTTATATTACCCGACCCCACGAAAGCTGTGAAA

>897_Sample_ASV13

CACCGCGGTTATACGAGAGGCCCAAGTTGACAGACATCGGCGTAAAGAGTGGTTAAGGAA

AAATTACTGCTAAAGCCGAACACCCTCAGAACTGTCATACGTTCCCGAAGATAAGAAGCC

CCACCACGAAAGTGGCTTTATATTACCCGACCCCACGAAAGCTGTGAAA

>897_Sample_ASV14

CACCGCGGTTATACGAAAGACCCTAGTTGACAGACACCGGCGTAAAGAGTGGCTAGGGAA

AAATTACTACTAAAGCCGAACACCTTCAGAACTGTCATACGTTCCCGAAGATAAGAAGCC

CCACCACGAAAGTGGCTTTATATTACCTGACCCCACGAAAGCTAAGAAA

>897_Sample_ASV15

CACCGCGGTTATACGAGAGGCCCAAGTTGACAGACACCGGCGTAAAGAGTGGCTAGGGAA

AAATTACTACTAAAGCCGAACACCCTCAGAACTGTCATACGTTCCCGAAGGTAAGAAGCC

CCACCACGAAAGTGGCTTTATAATACCCGACCCCACGAAAGCTGTGAAA

>897_Sample_ASV16

CACCGCGGTTATACGAGAGGCCCAAGTTGACAGACACCGGCGTAAAGAGTGGCTAAGGAA

AAATTTCTACTAAAGCCGAACACCTTCAGAACTGTCATACGTTCCCGAAGATAAGAAGCC

CCACCACGAAAGTGGCTTTATATTACCCGACACCACGAAAGCTAAGAAA

>897_Sample_ASV17

CACCGCGGTTATACGAGAGGCCCAAGTTGACAGACACCGGCGTAAAGAGTGGCTAAGGAA

AAATTTCTACTAAAGCCGAACACCTTCAGAACTGTCATACGTTCCCGAAGAAAAGAAGCC

CCACCACGAAAGTGGCTTTATATTACCCGACCCCACGAAAGCTAAGAAA

>897_Sample_ASV18

CACCGCGGTTATACGAGAGGCCCAAGTTGACAGACACCGGCGTAAAGAGTGGCTAAGGAA

AAATTTCTACTAAAGCCGAACACCTTCAGAACTGTCATACGTTCCCGAAGATAAGAAGCC

CCACCACGAAAGTGGCTTTATATTACCCGACACCACGAAAGCTGTGAAA

>897_Sample_ASV19

CACCGCGGTTAAACGAGAGACCCTAGTTGATCATCACGGCGTAAAGAGTGGTTAGGGGAT

TATCATAAATAAAGCCAAAGACCTCCCAAGCTGTCGCACGCATTCCGGAGGCGCGAAGCC

CACACACGAAAGTAGCTTTAACTATTGACCCTGATGCCACGAAAGCTAAGAAA

>897_Sample_ASV20

CACCGCGGTTATACGAAAGACCCTAGTTTATAGGCACGGCGTAAAGGGTGGTTAGGGAAT

ACAAAAATAAAGCTAAAAATCCTCTAAGCTGTCATACGCTTCCCGAAGACATGAAACCCA

ACCACGAAAGTAGCTTTAAACTCTCCTGACACCACGAAAGCCAAGGAA

>897_Sample_ASV21

CACCGCGGTTATACGAAAGACCCTAGTTGATAGATCACGGCGTAAAGGGTGGTTAGGGAA

TACAAAAATAAAGCTAAAAATCCTCTAAGCTGTCATACGCTTCCCGAAGACATGAAACCC

AACCACGAAAGTAGCTTTAAACTCTCCTGACACCACGAAAGCCAAGGAA

>897_Sample_ASV22

CACCGCGGTTATACGAAAGACCCTAGTTGATAGATCACGGCGTAAAGGGTGGTTAGGGAA

TACAAAAATAAAGCTAAAAATCCTCTAAGCTGTCATACGCTTCCCGAAGACATGAAACCC

AACCACGAAAGTAGCTTTAAACTCTCCTGACACCACGAAAGCTGGGGGA

>897_Sample_ASV23

CACCGCGGTTATACGAGAGGCCCAAGTTGATAAGCCCGGCGTAAAGAGTGGTTATGGAGA

ATAAAACACTAAAGCTAAAGACCCCCTAGGCTGTTATACGCACCTGGCGGCTCGAACCAC

CTATACGAAAGTAGCTTTACCCCCCTTCCACCTGACCCCACGAAAGCTAAGAAA

>897_Sample_ASV24

CACCGCGGTTATACGAGAGGCCCAAGTTGATAAGCCCGGCGTAAAGAGTGGTTATGGAGA

ATAAAACACTAAAGCTAAAGACCCCCTAGGCTGTTATACGCACCTGGCGGCTCGAACCAC

CTATACGAAAGTAGCTTTACCCCCCTTCCACCAGAATCCACGACAGCTGGGGCA

>897_Sample_ASV25

CACCGCGGTTATACGAGAGGCCCAAGTTGATAAGCCCGGCGTAAAGAGTGGTTATGGAGA

ATAAAACACTAAAGCTAAAGACCCCCTAGGCTGTTATACGCACCTGGCGGCTCGAACCAC

CTATACGAAAGTAGCTTTACCCCCCTTCCACCAGAATCCACGAAAGCTAAGAAA

>897_Sample_ASV26

CACCGCGGTTATACGAGAGGCCCAAGTTGACAGACATCGGCGTAAAGAGTGGTTAAGGAA

ACCCCATTACTAAAGCCGAACGCCCTCAGAACTGTCATACGTTCCCGAAGGTAAGAAGCC

CCACTACGAAAGTGGCTTTAACCACACCCGACCCCACGAAAGCTGTGAAA

>897_Sample_ASV27

CACCGCGGTTATACGAGAGGCCCAAGTTGACAGACACCGGCGTAAAGAGTGGTTAAGGAA

ATTCATCAACTAAAGCCGAATGCCCTCAGGACTGTTATACGTTTGCGAAGGTAAGAAGCC

CTACTACGAAAGTGGCTTTATTCCACCTGATTCCACGAAAGCTGCGGAA

>897_Sample_ASV28

CACCGCGGTCACACGATTAACCCAAGTCAATAGAAGCCGGCGTAAAGAGTGTTTTAGATC

ACCCCCTCCCCAATAAAGCTAAAACTCACCTGAGTTGTAAAAAACTCCAGTTGACACAAA

ATAGACTACGAAAGTGGCTTTAACATATCTGAACACACAATAGCTAAGACC

>897_Sample_ASV29

CACCGCGGTTATACGAGAGACCCCAGTTGATAGCTACGGCGTAAAGAGTGGTTAAGGACC

CCATTTAATAAAGCCAAAGACCCCCCAAGCCGTCATACGCACACGAGGGCGCGAAGCTCG

CACACGAAAGTAGCTCTAATTTACCCGACGCCACGAAAGCTAAGGAA

>897_Sample_ASV30

CACCGCGGTTATACGAGAAACCCTAGTTGACAAATACGGCGTAAAGAGTGGTTAAGGGGT

ATCAAAAATAAAGTCAAACATTTTCCTAGTTGTTATAAGCATTTCGAGAACATGAAGCCC

AACCACGAAAGTAACTTTACCTTCACCTGACCCCACGAAAGCTAAGAAA

>897_Sample_ASV31

CACCGCGGTTATACGAGAGACCCTAGTTGATAAATACGGCGTAAAGAGTGGTTATGGGAA

AAACAAATAAAGTCAAACAACCTCTTAGCTGTTATACGCATTATGAGAGCATGAAGCCCC

CTCACGAAAGTAACTTTAATACCTCCTGACCCCACGAAAGCTAAGAAA

>897_Sample_ASV32

CACCGCGGTTATACGAGAGACCCTAATTGATAGACACGGCGTAAAGAGTGGTTTGGGAAA

GCCCAAAAATAAAGCTAAAGACCTTCTAAGCCGTCACACGCACGCCGAAGGCACGAAGCC

CTGACACGAAAGTAGCTTTACTATTACCCCCGACGCCACGAAAGCTAAGAAA

>897_Sample_ASV33

AACCGCGGTTATACGAGAGACCCTAATTGATAGCTCACGGCGTAAAGGGTGGTTTGGGGA

AACCTCAAAATAAAGCTAAAGACCTTCTAAGCCGTTACACGCATACCGAAGGCACGAGGC

CCTAACACGAAAGTAGCTTTACTATTACCCCTGACGCCACGAAAGCTAAGAAA

>897_Sample_ASV34

CACCGCGGTTATACGAAAGACCCTAGTTGATAGGCACGGCGTAAAGGGTGGTTTGGGGAA

ACCTCAAAATAAAGCTAAAGACCTTCTAAGCCGTTACACGCATACCGAAGGCACGAGGCC

CTAACACGAAAGTAGCTTTACTATTACCCCTGACGCCACGAAAGCTAAGAAA

>897_Sample_ASV35

CACCGCGGTTATACGAAAGACCCTAGTTTATAGATGCGGCGTAAAGGGTGGTTAAGGAAA

ACCTAAAATAATGCTAAAGACTCTCAAAGCTGTTATACGCTTCATGAGCACACGAAATCC

AAATACGAAAGTTGCCTTAATAAACTTTGCCTGACCCCACGAAAGCTGAAAAA

>897_Sample_ASV36

CACCGCGGTTATACGAGAGACCCTAGTTGATAGCTACGGCGTAAAGCGTGGTTAGGAGAA

CAAGATAAATAAAGTCAAACAATCTCTCGGCCGTTATACGTTATCTGAGAATATGAAGTC

CTACCACGAAAGTAACTTTAATTTTTCTGACCCCACGAAAACTGAGGAA

>897_Sample_ASV37

CACCGCGGTTAAACGAGAGGCCCTAGTTGATAGTACAACGGCGTAAAGGGTGGTTAAGGA

TAGTGAAATAATAAAGTCGAATGGCCCTTTGGCTGTCATACGCTTCTAGGAGTCCGAAGC

CCAATATACGAAAGTAACTTTAAGAGCGCCCACCTGACCCCACGAAAACTGAGAAA

>897_Sample_ASV38

CACCGCGGTTATACGAAAGACCCTAGTTTATAGACACGGCGTAAAGGGTGGTTCGGGACT

AAACAAAATAAAGCCAAAGACCTCCTAAGCCGTCATACGCTCACGGAAGCACGAAGCCCA

AGTACGAAAGTAGCTTTACAAAACATGCCTGACTCCACGAAAGCTAGGAAA

>897_Sample_ASV39

CACCGCGGTTATACGAAAGACCCTAGTTTATAGACACGGCGTAAAGGGTGGTTCGGGACT

AAACAAAATAAAGCCAAAGACCTCCTAAGCCGTCATACGCTCACGGAAGCACGAAGCCCA

AGTACGAAAGTAGCTTTACAAAACATGCCTGACTCCACGAAAGCTAAGAAA

>897_Sample_ASV40

CACCGCGGTTATACGAAAGACCCTAGTTGATAGGCACGGCGTAAAGGGTGGTTAGGGATA

TATTAAAAATAAAGTTAAAGAGCCTCTAAGCTGTCGCACGCATCCCGAGAGCTCGAGACC

CAGACACGAAAGTAACTTTAAAATACCTCACCTGACCCCACGAAAGCTAAGAAA

>897_Sample_ASV41

CACCGCGGTTATACGAGAGGCCCAAGTTGATACTCCACGGCGTAAAGAGTGGTTAGAAAA

GACCTACTACTAAAGCCGAACGCCTTCAAAGCTGTTATACGCATCCGAAGGTGAGAAGCC

CACCCACGAAAGTGGCTTTACAACCTTGACTCCACGAAAGCTATGACA

>897_Sample_ASV42

CACCGCGGTTATACGAGAGGCCCAAGTTGATACTCCACGGCGTAAAGAGTGGTTAGAAAA

GACCTACTACTAAAGCCGAACGCCTTCAAAGCTGTTATACGCATCCGAAGGTGAGAAGCC

CACCCACGAAAGTGGCTTTACAACCTTGACTCCACGAAAGCTAAGAAA

>897_Sample_ASV43

CACCGCGGTTATACGAGAGACCCCAGTTGATAAACACGGCGTAAAGAGTGGTTAAGGGCA

CAAAACAAATAAAGTCAAACAACCTCTTAGCTGTTATACGCATTATGAGGATATGAAGCC

CCCCTACGAAAGTAACTTTACCAACCCCTGACCCCACGAAAGCTAAGAAA

>897_Sample_ASV44

CACCGCGGTTATACGAGAGACCCTAGTTGATATATACGGCGTAAAGAGTGGTTTGGAATA

ACCAAGTAATATAGCCAAAGACCTCCCAAGCCGTCACACGCACCCCGGAGGCACGAAGCC

CAAACACGAAAGTAGCTTTATTAACTTACCGACGCCACGAAAGCTAAGAAA

>897_Sample_ASV45

CACCGCGGTTATACGAAAGACCCTAGTTTATAGGCACGGCGTAAAGGGTGGTTAGGGGAT

AAACAAAATAAAGCCAAAGACCTTCTAAGTCGTCATACACTCATGAAGGCACGAAGCCCA

AATACGAAAGTAACTTTACAACTTAGACCCGACTCCACGAAAGCTAAGAAA

>897_Sample_ASV46

CACCGCGGTTATACGAAAGACCCTAGTTTATAGGCACGGCGTAAAGGGTGGTTAGGGGAT

AAACAAAATAAAGCCAAAGACCTTCTAAGTCGTCATACACTCATGAAGGCACGAAGCCCA

AATACGAAAGTAACTTTACAACTTAGACCCGACTCCACGAAAGCCAAGGAA

>897_Sample_ASV47

CACCGCGGTTATACGAAAGACCCTAGTTTATAGGCACGGCGTAAAGGGTGGTTAGGGGAT

AAACAAAATAAAGCCAAAGACCTTCTAAGTCGTCATACACTCATGAAGGCACGAAGCCCA

AATACGAAAGTAACTTTACAACTTAGACCCGACTCCACGAAAGCTGGGGGA

>897_Sample_ASV48

CACCGCGGTTATACGAGAGACTCCAGTTGATAGCTACGGCGTAAAGAGTGGTTTGGGGCC

CCACCCAAAATAAAGCCAAAGACCTCCCAAGCCGTCAAACGCACCCCGGAGGCACGAAGT

CCTAACGCGAAAGCAACTTTACCTCCCCCGACGCCACGAAAGCTAAGAAA

>897_Sample_ASV49

CACCGCGGTTATACGAGAGACCCTAATTGATAGACTACGGCGTAAAGAGTGGTTTAGAAC

AAAAATTAATAAAGCCAAAGATCCTCCTGGCCGTCGCACGCATTTCGAGGATACGAAGCC

CCACTACGAAAGTAGCTTTACTACAACTTTTCTGACCCCACGAAAGCTAAGAAA

>897_Sample_ASV50

CACCGCGGTTATACGAAAGACCCTAGTTGATAGATCACGGCGTAAAGGGTGGTTAAGGAG

AACAAAAATAAAGCTAAAAATCCTCTAAGCTGTCATACGCTTTCCGAAGACATGAGACCC

AACCACGAAAGTAACTTTAAACTTTCCTGACACCACGAAAGCCAAGAAA

>899_Sample_ASV1

CACCGCGGTTATACGAGAGGCCCAAGTTGACAGACATCGGCGTAAAGAGTGGTTAAGGAA

AACCTTCAACTAAAGTCGAACGCTCTCAAGACTGTCGTACGTTTCCGAGAGTATGAAGCC

CCACCACGAAAGTGACTTTACCTCACCTGACTCCACGAAAGCTGCGAAA

>899_Sample_ASV2

CACCGCGGTTATACGAAAGACCCTAGTTGATGGGTGCGGCGTAAAGGGTGGTTAGGGACT

ACAAAAAATAGAGCTAAAGATCCTCTAGGTCGTCATACACATCACGAGGAAACGAAGCCC

AAATACGAAAGTAGCTCTAAATATAATCCTGACGCCACGAAAGCCAAGAAA

>899_2_Sample_ASV1

CACCGCGGTTATACGAAAGACCCTAGTTGATGGGTGCGGCGTAAAGGGTGGTTAGGGACT

ACAAAAAATAGAGCTAAAGATCCTCTAGGTCGTCATACACATCACGAGGAAACGAAGCCC

AAATACGAAAGTAGCTCTAAATATAATCCTGACGCCACGAAAGCCAAGAAA

>899_2_Sample_ASV2

CACCGCGGTTATACGAAAGACCCTAGTTGATGGGTGCGGCGTAAAGGGTGGTTAGGGACT

ACAAAAAATAGAGCTAAAGATCCTCTAGGTCGTCATACACATCACGAGAAAACGAAGCCC

AAATACGAAAGTAGCTCTAAATATAATCCTGACGCCACGAAAGCCAAGAAA

>899_Sample_ASV3

CACCGCGGTTATACGAGAGACCCTAGTTGATTAAAGCGGCGTAAAGAGTGGTTATGGAAC

TATTCTTTTAAAGCAGAAAACCTCTCAAACTGTTATACGCACCCAGAGGTCTGAATCCCT

CACACGAAAGTGACTTTATTTATGCCTACCAGAACCCACGAAAGCTATGACA

>899_Sample_ASV4

CACCGCGGTTATACGAGAGACCCTAGTTGATTAAAGCGGCGTAAAGAGTGGTTATGGAAC

TATTCTTTTAAAGCAGAAAACCTCTCAAACTGTTATACGCACCCAGAGGTCTGAATCCCT

CACACGAAAGTGACTTTATTTATGCCTACCAGAACCCACGAAAGCTGGGACA

>899_Sample_ASV5

CACCGCGGTTATACGAAAGACCCTAGTTTATAGGTACGGCGTAAAGGGTGGTTAGGGGAC

AAACAAAATAAAGCCAAAGACCTCCTAAGCCGTCATACGCTCACGGAAGCACGAAGCCCA

AACACGAAAGTAGCTTTACCGAACATGCCCGACTCCACGAAAGCTGGGGAA

>899_2_Sample_ASV3

CACCGCGGTTATACGAGAGGCCCAAGTTGACAGACACCGGCGTAAAGAGTGGCTAGGGAA

AAATTACTACTAAAGCCGAACACCTTCAGAACTGTCATACGTTCCCGAAGATAAGAAGCC

CCACCACGAAAGTGACTTTATATTACCCGACCCCACGAAAGCTGTGAAA

>899_2_Sample_ASV4

CACCGCGGTTATACGAGAGGCCCAAGTTGACAGACACCGGCGTAAAGAGTGGCTAGGGAA

AAATTACTACTAAAGCCGAACACCTTCAGAACTGTCATACGTTCCCGAAGATAAGAAGCC

CCACCACGAAAGTGGCTTTATATTACCCGACCCCACGAAAGCTGTGAAA

>899_2_Sample_ASV5

CACCGCGGTTATACGAGAGGCCCAAGTTGACAGACACCGGCGTAAAGAGTGGCTAGGGAA

AAATTACTACTAAAGCCGAACACCCTCAGAACTGTCATACGTTCCCGAAGGTAAGAAGCC

CCACCACGAAAGTGGCTTTATAATACCCGACCCCACGAAAGCTGTGAAA

>899_Sample_ASV6

AACCGCGGTTATACGAGAGACCCAAGTTGACAGACACCGGCGTAAAGAGTGGCTAGGGAA

AAATTACTACTAAAGCCGAACACCTTCAGAACTGTCATACGTTCCCGAAGATAAGAAGCC

CCACCACGAAAGTGGCTTTATATTACCCGACCCCACGAAAGCTAAGGAA

>899_Sample_ASV7

AACCGCGGTTATACGAGAGACCCTAGTTGACAGACACCGGCGTAAAGAGTGGCTAGGGAA

AAATTACTACTAAAGCCGAACACCTTCAGAACTGTCATACGTTCCCGAAGATAAGAAGCC

CCACCACGAAAGTGGCTTTATATTACCCGACCCCACGAAAGCTGTGAAA

>899_Sample_ASV8

AACCGCGGTTATACGAGAGGCCCAAGTTGACAGACACCGGCGTAAAGAGTGGCTAGGGAA

AAATTACTACTAAAGCCGAACACCTTCAGAACTGTCATACGTTCCCGAAGATAAGAAGCC

CCACCACGAAAGTGGCTTTATATTACCCGACCCCACGAAAGCTAAGGAA

>899_Sample_ASV9

CACCGCGGTTATACGAGAGGCCCAAGTTGACAGACACCGGCGTAAAGAGTGGCTAGGGAA

AAATTACTACTAAAGCCGAACACCTTCAGAACTGTCATACGTTCCCGAAGATAAGAAGCC

CCACCACGAAAGTGGCTTTATATTACCCGACCCCACGAAAGCTGTGAAA

>899_Sample_ASV10

CACCGCGGTTATACGAGAGGCCCAAGTTGACAGACACCGGCGTAAAGAGTGGCTAGGGAA

AAATTACTACTAAAGCCGAACACCTTCAGAACTGTCATACGTTCCCGAAGATAAGAAGCC

CCACCACGAAAGTGGCTTTATATTACCCGACCCCACGAAAGCTAAGGAA

>899_2_Sample_ASV6

CACCGCGGTTATACGAGAGGCCCAAGTTGACAGACACCGGCGTAAAGAGTGGCTAAGGAA

AAATTTCTACTAAAGCCGAACACCTTCAGAACTGTCATACGTTCCCGAAGATAAGAAGCC

CCACCACGAAAGTGGCTTTATATTACCCGACACCACGAAAGCTGTGAAA

>899_2_Sample_ASV7

CACCGCGGTTATACGAGAGGCCCAAGTTGACAGACACCGGCGTAAAGAGTGGCTAAGGAA

AAATTTCTACTAAAGCCGAACACCTTCAGAACTGTCATACGTTCCCGAAGAAAAGAAGCC

CCACCACGAAAGTGGCTTTATATTACCCGACCCCACGAAAGCTGTGAAA

>899_2_Sample_ASV8

CACCGCGGTTATACGAAAGACTCTAGTTGACAAACACGGCGTAAAGGGTGGTTAGGATAA

CAACAAAATAAAGCTAAAGACTTACCAAGCCGTCATACGCCCATGTAAGAGCGAAAACCA

CAAACGAAAGTAACTTTATTACACCATCCGAATCCACGAAAGCTAAGAAA

>899_2_Sample_ASV9

CACCGCGGTTATACGAAAGACTCTAGTTGACAAACACGGCGTAAAGGGTGGTTAGGATAA

CAACAAAATAAAGCTAAAGACTTACCAAGCCGTCATACGCCCATGTAAGAGCGAAAACCA

CAAACGAAAGTAACTTTATTACACCATCCGAATCCACGAAAGCCAAGGAA

>899_Sample_ASV11

CACCGCGGTTAAACGAGAGACCCTAGTTGATCATCACGGCGTAAAGAGTGGTTAGGGGAT

TATCATAAATAAAGCCAAAGACCTCCCAAGCTGTCGCACGCATTCCGGAGGCGCGAAGCC

CACACACGAAAGTAGCTTTAACTATTGACCCTGATGCCACGAAAGCTAAGAAA

>899_2_Sample_ASV10

CACCGCGGTTATACGAGAGACCCTAGTTGATATATACGGCGTAAAGAGTGGTTTGGGATA

TTTCTCCAAAATAAAGCCGAAGATCTCCCAAGCCGTCACACGCATCCCGGAGACACGAAG

CCCTGATACGAAGGTAGCTTTATTAACACCCCGACGCCACGAAAGCTAAGAAA

>899_2_Sample_ASV11

CACCGCGGTTATACGAGAGACCCTAGTTGATATATACGGCGTAAAGAGTGGTTTGGGATA

TTTCTCCAAAATAAAGTCGAAGATCTCCCAAGCCGTCACACGCATCCCGGAGACACGAAG

CCCTGATACGAAGGTAGCTTTATTAACACCCCGACGCCACGAAAGCTAAGAAA

>899_2_Sample_ASV12

CACCGCGGTTATACGAAAGACCCTAGTTGATAGATCACGGCGTAAAGGGTGGTTAAGGAA

AACAAAAATAAAGCTAAAAATCCTCTAAGCTGTCATACGCTTCCCGAAGACATGAAACCC

AACCACGAAAGTAGCTTTAAACTCTCCTGACACCACGAAAGCCAAGGAA

>899_2_Sample_ASV13

CACCGCGGTTATACGAAAGACCCTAGTTGATAGATCACGGCGTAAAGAGTGGTTAGGGGC

ACACCAAAATAAAGCTAAAAATCCTCTAAGCTGTCATACGCTTCCCGAAGACATGAAACC

CAACCACGAAAGTAGCTTTAAACTCTCCTGACACCACGAAAGCCAAGGAA

>899_2_Sample_ASV14

CACCGCGGTTATACGAAAGACCCTAGTTGATAGATCACGGCGTAAAGGGTGGTTAGGGAA

TACAAAAATAAAGCTAAAAATCCTCTAAGCTGTCATACGCTTCCCGAAGACATGAAACCC

AACCACGAAAGTAGCTTTAAACTCTCCTGACACCACGAAAGCCAAGGAA

>899_2_Sample_ASV15

CACCGCGGTTATACGAAAGACCCTAGTTGATAGATCACGGCGTAAAGGGTGGTTAAGGAA

AACAAAAATAAAGCTAAAAATCCTCTAAGCTGTCATACGCTTTCCGAAGACATGAAACCC

AACCACGAAAGTAGCTTTAAACTCTCCTGACACCACGAAAGCCAAGGAA

>899_2_Sample_ASV16

CACCGCGGTTATACGAAAGACCCTAGTTGATAGATCACGGCGTAAAGGGTGGTTAGGGAA

TACAAAAATAAAGCTAAAAATCCTCTAAGCTGTCATACGCTTCCCGAAGACATGAAACCC

AACCACGAAAGTAGCTTTAAAATTTCCTGACACCACGAAAGCCAAGGAA

>899_Sample_ASV12

CACCGCGGTTATACGAGAGGCCCAAGTTGATAAGCCCGGCGTAAAGAGTGGTTATGGAGA

ATAAAACACTAAAGCTAAAGACCCCCTAGGCTGTTATACGCACCTGGCGGCTCGAACCAC

CTATACGAAAGTAGCTTTACCCCCCTTCCACCAGAATCCACGACAGCTGGGGCA

>899_2_Sample_ASV17

CACCGCGGTTATACGAGAGGCCCAAGTTGATAAGCCCGGCGTAAAGAGTGGTTATGGAGA

ATAAAACACTAAAGCTAAAGACCCCCTAGGCTGTTATACGCACCTGGCGGCTCGAACCAC

CTATACGAAAGTAGCTTTACCCCCCTTCCACCAGAATCCACGACAGCTGGGGCA

>899_Sample_ASV13

CACCGCGGTTATACGAGAGGCCCTAGTTGATAGCCACGGCGCAAAGAGTGGTTAAGGAGC

CCCACTAAATAAAGTCGAACACTTCCTAGGCCGTTATACGTTTTCTAGAAGCACGAAACC

CAATTACACGAAAGCAACTTTATACTAAACAACCTGACCCCACGAAAGCTAAGAAA

>899_2_Sample_ASV18

CACCGCGGTTATACGAGAGGCCCTAGTTGATAGCCACGGCGCAAAGAGTGGTTAAGGAGC

CCCACTAAATAAAGTCGAACACTTCCTAGGCCGTTATACGTTTTCTAGAAGCACGAAACC

CAATTACACGAAAGCAACTTTATACTAAACAACCTGACCCCACGAAAGCTAAGAAA

>899_2_Sample_ASV19

CACCGCGGTTATACGAGAGGCCCAAGTTGACAGACATCGGCGTAAAGAGTGGTTAAGGAA

ACCCCATTACTAAAGCCGAACGCCCTCAGAACTGTCATACGTTCCCGAAGGTAAGAAGCC

CCACTACGAAAGTGGCTTTAACCACACCCGACCCCACGAAAGCTGTGAAA

>899_Sample_ASV14

CACCGCGGTTATACGAGAGGCCCAAGTTGACAGACATCGGCGTAAAGAGTGGTTAAGGAA

ACCCCATTACTAAAGCCGAACGCCCTCAGAACTGTCATACGTTCCCGAAGGTAAGAAGCC

CCACTACGAAAGTGGCTTTAACCACACCCGACCCCACGAAAGCTGTGAAA

>899_Sample_ASV15

CACCGCGGTTATACGAGAGGCCCAAGTTGATAGATATCGGCGTAAAGAGTGGTTAAGGAA

ACCCCGTTACTAAAGCCGAACGCCCTCAGAACTGTCATACGTTCCCGAAGGTAAGAAGCC

CCACTACGAAAGTGGCTTTAACCACACCCGACCCCACGAAAGCTGTGAAA

>899_2_Sample_ASV20

CACCGCGGTTATACGAGAGACCCCAGTTGATAGCTACGGCGTAAAGAGTGGTTAAGGACC

CCATTTAATAAAGCCAAAGACCCCCCAAGCCGTCATACGCACACGAGGGCGCGAAGCTCG

CACACGAAAGTAGCTCTAATTTACCCGACGCCACGAAAGCTAAGGAA

>899_Sample_ASV16

CACCGCGGTTATACGAGAGGCCCTAGTTGATAAATACGGCGTAAAGAGTGGTTAGAGGAC

TCCCATAATAAAGCCAAAGACCCCCCGGGCCGTCACACGCATACGAGGGCACGAAGTTCA

CACACGAAAGTAGCTTTAAATTACCCGACGCCACGAAAGCTAAGAAA

>899_2_Sample_ASV21

GACCGCGGTTATACGAGAGACTCTAGTTGATAGATACGGCGTAAAGAGTGGTTTGGGATA

AACTATAAATAAAGCCAAAGACCTTCCAAGCTGTTAAACGCATACCGAAGGCACGAAGCC

CCAACACGAAAGTAGCTTTACTACTACCCCCGATGCCACGAAAGCTAAGACA

>899_Sample_ASV17

GACCGCGGTTATACGAGAGACTCTAGTTGATAGATACGGCGTAAAGAGTGGTTTGGGATA

AACTATAAATAAAGCCAAAGACCTTCCAAGCTGTTAAACGCATACCGAAGGCACGAAGCC

CCAACACGAAAGTAGCTTTACTACTACCCCCGATGCCACGAAAGCTAAGACA

>899_2_Sample_ASV22

CACCGCGGTTATACGAAAGACCCCAGTTTATAGACACGGCGTAAAGGGTGGTTATAGGAC

AAACTAACATAGAGCCAAAGACCCCCTAAGCCGTCATACGCTCACGAGGGCTCGAAGCCC

CTATACGAAAGTAGCTCTACCAAAAAATGCTCGACTCCACGAAAGCTGGGACA

>899_Sample_ASV18

CACCGCGGTTATACGAGAGACCCTAGTTGATAAATACGGCGTAAAGAGTGGTTATGGGAA

AAACAAATAAAGTCAAACAACCTCTTAGCTGTTATACGCATTATGAGAGCATGAAGCCCC

CTCACGAAAGTAACTTTAATACCTCCTGACCCCACGAAAGCTAAGAAA

>899_2_Sample_ASV23

AACCGCGGTTATACGAGAGACCCTAATTGATAGCTCACGGCGTAAAGGGTGGTTTGGGGA

AACCTCAAAATAAAGCTAAAGACCTTCTAAGCCGTTACACGCATACCGAAGGCACGAGGC

CCTAACACGAAAGTAGCTTTACTATTACCCCTGACGCCACGAAAGCTAAGAAA

>899_2_Sample_ASV24

CACCGCGGTTATACGAAAGACCCTAGTTGATGGATCACGGCGTAAAGGGTGGTTAAGGGA

TATAAAAATAAAGCTAAAAATCCTCTTAGCTGTCATACGCTTTCCGAAGACATGAGGCCC

AATTACGAAAGTAGCTTTAAACCACCCTGACACCACGAAAGCCAAGAAA

>899_2_Sample_ASV25

CACCGCGGTTATACGAAAGACCCTAGTTTATAGATGCGGCGTAAAGGGTGGTTAAGGAAA

ACCTAAAATAATGCTAAAGACTCTCAAAGCTGTTATACGCTTCATGAGCACACGAAATCC

AAATACGAAAGTTGCCTTAATAAACTTTGCCTGACCCCACGAAAGCTGAAAAA

>899_2_Sample_ASV26

CACCGCGGTTATACGAAAGACCCTAGTTTATAGATGCGGCGTAAAGGGTGGTTAAGGAAA

ACCTAAAATAATGCTAAAGACTCTCAAAGCTGTTATACGCTTCATGAGCACACGAAATCC

AAATACGAAAGTTGCCTTAATAAACTTTGCCTGACCCCACGAAAGCCAAGGAA

>899_2_Sample_ASV27

CACCGCGGTTATACGAAAGACCCTAGTTTATAGATGCGGCGTAAAGGGTGGTTAAGGAAA

ACCTAAAATAATGCTAAAGACTCTCAAAGCTGTTATACGCTTCATGAGCACACGAAATCC

AAATACGAAAGTTGCCTTAATAAACTTTGCCTGACCCCACGAAAGCTAAGAAA

>899_2_Sample_ASV28

CACCGCGGTTATACGAAAGACCCTAGTTGATAGATCACGGCGTAAAGGGTGGTTAAGGAA

AACCTAAAATAATGCTAAAGACTCTCAAAGCTGTTATACGCTTCATGAGCACACGAAATC

CAAATACGAAAGTTGCCTTAATAAACTTTGCCTGACCCCACGAAAGCTGAAAAA

>899_2_Sample_ASV29

CACCGCGGTTATACGAAAGACCCTAGTTGATAGATCACGGCGTAAAGGGTGGTTAAGGAA

AACCTAAAATAATGCTAAAGACTCTCAAAGCTGTTATACGCTTCATGAGCACACGAAATC

CAAATACGAAAGTTGCCTTAATAAACTTTGCCTGACCCCACGAAAGCTAAGAAA

>899_Sample_ASV19

CACCGCGGTCATACGATTAACCCAAGTTAATAGGCACACGGCGTAAAGCGTGTTTAAGCA

CTATGCCAAATAAAGTTAAACTCCAATTAAGCTGTAAAAAGCCATAATCAAGACGAAAAT

AAATAACGAAAGTAACTTTACAACCGCTGAAACACGATAGCTAAGATC

>899_2_Sample_ASV30

CACCGCGGTTATACGAGAGGCCCTAGTTGATAACTACCGGCGTAAAGAGTGGTTATGGAA

AAATATTTAATAAAGCCGAACACCCCCTCAGCCGTCATACGCACCTGGGAGCACGAAGAC

CTACTGCGAAAGCAGCTTTAATTATGCCTGACCCCACGACAGCTAAGAAA

>899_Sample_ASV20

CACCGCGGTTATACGAAAGACCCTAGTTTATAGACACGGCGTAAAGGGTGGTTCGGGACT

AAACAAAATAAAGCCAAAGACCTCCTAAGCCGTCATACGCTCACGGAAGCACGAAGCCCA

AGTACGAAAGTAGCTTTACAAAACATGCCTGACTCCACGAAAGCTAGGAAA

>899_2_Sample_ASV31

CACCGCGGTTATACGAGAGGCCCAAGTTGATTATTACCGGCGTAAAGCGTGGTTAAGATT

TTTATAGGACTAAAGCCGAACCCCCTCAGAGCTGTTATACGCACCCGAAGGTTAGAAGTT

CAATCACGAAAGTGGCTTTACAACCCCGAACCCACGAAAGCTAAGATA

>899_2_Sample_ASV32

CACCGCGGTTATACGAGAGACCCTAATTGATAGGCGCGGCGTAAAGAGTGGTTTAGGACT

AAAACTAATAAAGCTAAAGATCCTCCCAGCCGTCGCACGCATTCTGAGGACACGAAACTC

AACCACGAAAGTAGCTTTAACATACTCTCCTGACCCCACGAAAGCTAAGAAA

>899_2_Sample_ASV33

CACCGCGGTTATACGAAAGACCCTAGTTGATAGGCACGGCGTAAAGGGTGGTTAGGGATA

TATTAAAAATAAAGTTAAAGAGCCTCTAAGCTGTCGCACGCATCCCGAGAGCTCGAGACC

CAGACACGAAAGTAACTTTAAAAATCTTCACCTGACCCCACGAAAGCTAAGAAA

>899_2_Sample_ASV34

CACCGCGGTTATACGAAAGACCCTAGTTGATAGGCACGGCGTAAAGGGTGGTTAGGGATA

TATTAAAAATAAAGTTAAAGAGCCTCTAAGCTGTCGCACGCATCCCGAGAGCTCGAGACC

CAGACACGAAAGTAACTTTAAAATACCTCACCTGACCCCACGAAAGCTAAGAAA

>899_2_Sample_ASV35

CACCGCGGTTATACGAAAGACCCTAGTTGATAGGCACGGCGTAAAGGGTGGTTAGGGATA

TATTAAAAATAAAGTTAAAGAGCCTCTAAGCTGTCGCACGCATCCCGAGAGCTCGAGACC

CAGACACGAAAGTAACTTTAAAATACCTCACCTGACCCCACGAAAGCCAAGGAA

>899_Sample_ASV21

CACCGCGGTTATACGAAAGACCCTAGTTGATAGGCACGGCGTAAAGGGTGGTTAGGGATA

TATCAAAAATAAAGTTAAAGAGCCTCTAAGCTGTCGCACGCATCCCGAGAGCTCGAGACC

CAGACACGAAAGTAACTTTAAAATACCTCACCTGACCCCACGAAAGCTAAGAAA

>899_Sample_ASV22

CACCGCGGTTATACGAAAGACCCTAGTTGATAGCCACGGCGTAAAGGGTGGTTAAGGTAT

ACAACAAATAAAGCTAAATAGCCTCTAGGCCGTCGCACGCATTCCGAGAGCTCGAAACCC

AAAAACGAAAGTAGCTTTAAAATAAACAGACCTGACCCCACGAAAGCTAAGAAA

>899_Sample_ASV23

CACCGCGGTTATACGAGAGGCCCAAGTTGATACTCCACGGCGTAAAGAGTGGTTAGAAAA

GACCTACTACTAAAGCCGAACGCCTTCAAAGCTGTTATACGCATCCGAAGGTGAGAAGCC

CACCCACGAAAGTGGCTTTACAACCTTGACTCCACGAAAGCTATGACA

>899_Sample_ASV24

CACCGCGGTTATACGAGAGACCCCAGTTGATAAACACGGCGTAAAGAGTGGTTAAGGGCA

CAAAACAAATAAAGTCAAACAACCTCTTAGCTGTTATACGCATTATGAGGATATGAAGCC

CCCCTACGAAAGTAACTTTACCAACCCCTGACCCCACGAAAGCTAAGAAA

>899_2_Sample_ASV36

CACCGCGGTTATACGAGAGACCCCAGTTGATAAACACGGCGTAAAGAGTGGTTAAGGGCA

CAAAACAAATAAAGTCAAACAACCTCTTAGCTGTTATACGCATTATGAGGATATGAAGCC

CCCCTACGAAAGTAACTTTACCAACCCCTGACCCCACGAAAGCTAAGAAA

>899_2_Sample_ASV37

CACCGCGGTTATACGAAAGACCCTAGTTTATAGGCACGGCGTAAAGGGTGGTTAGGGGAT

AAACAAAATAAAGCCAAAGACCTTCTAAGTCGTCATACACTCATGAAGGCACGAAGCCCA

AATACGAAAGTAACTTTACAACTTAGACCCGACTCCACGAAAGCTGGGGGA

>899_Sample_ASV25

CACCGCGGTTATACGAAAGACCCTAGTTTATAGGCACGGCGTAAAGGGTGGTTAGGGGAT

AAACAAAATAAAGCCAAAGACCTTCTAAGTCGTCATACACTCATGAAGGCACGAAGCCCA

AATACGAAAGTAACTTTACAACTTAGACCCGACTCCACGAAAGCTGGGGGA

>899_2_Sample_ASV38

CACCGCGGTTATACGAGAGACCCTAATTAATAGATACGGCGTAAAGAGTGGTTTAGGACA

AAAATTAATAAAGCCGAAGTTTCTCCTAGCCGTTGTACGCACTCTGAGAACACGAAGCCC

CACTACGAAAGTAGCTTTACCTATTATTTCCTGACCCCACGAAAGCTAAGAAA

>899_2_Sample_ASV39

CACCGCGGTTATACGAGAGACCCTAATTAATAGCTACGGCGTAAAGAGTGGTTTAGGGTA

AAAATTAATAAAGCCGAAGTTTCTCCTAGCCGTTGTACGCACTCTGAGAACACGAAGCCC

CACTACGAAAGTAGCTTTACCTATTATTTCCTGACCCCACGAAAGCTAAGAAA

>899_2_Sample_ASV40

CACCGCGGTTATACGAGAGACCCTAATTAATAGATACGGCGTAAAGAGTGGTTTAGGACA

AAAATTAATAAAGCCGAAGTTTCTCCTAGCCGTTGTACGCACTCTGAGAACACGAAGCCC

CACTACGAAAGTAGCTTTACCAATAATCTCCTGACCCCACGAAAGCTAAGAAA

>899_2_Sample_ASV41

CACCGCGGTTATACGAAAGACCCTAGTTGATAGATCACGGCGTAAAGGGTGGTTAAGGAA

AACAAAAATAAAGCTAAAAATCCTCTAAGCTGTCATACGCTTTCCGAAGACATGAGATCC

AACCACGAAAGTAGCTTTAAAATTTCCTGACACCACGAAAGCCAAGGAA

>899_Sample_ASV26

CACCGCGGTTATACGAAAGACCCTAGTTGATAGATCACGGCGTAAAGGGTGGTTAAGGAG

AACAAAAATAAAGCTAAAAATCTTCTAAGCTGTCATACGCTTTCCGAAGACATGAGACCC

AACCACGAAAGTAGCTTTAAACTTTCCTGACACCACGAAAGCCAAGAAA

>901_Sample_ASV1

TACCGCGGTTATACGAAAGACCCAAGTTAATAAACTACGGCGTAAAGCGTGGTTAGAGAC

CTCCAATAATTAAAGCCAAAGACCTTCTAAGCCGTCGCACGCCCCGCGAAGACACGAAGC

CCAAAAACGAAAGTAGCTTTATTCTTACTCGACCCCACGAAAGCTAGGGCA

>901_Sample_ASV2

CACCGCGGTTATACGAAAGACCCTAGTTGATGGGTGCGGCGTAAAGGGTGGTTAGGGACT

ACAAAAAATAGAGCTAAAGATCCTCTAGGTCGTCATACACATCACGAGGAAACGAAGCCC

AAATACGAAAGTAGCTCTAAATATAATCCTGACGCCACGAAAGCCAAGAAA

>901_Sample_ASV3

CACCGCGGTTATACGAGAGACCCTAGTTGATTAAAGCGGCGTAAAGAGTGGTTATGGAAC

TATTCTTTTAAAGCAGAAAACCTCTCAAACTGTTATACGCACCCAGAGGTCTGAATCCCT

CACACGAAAGTGACTTTATTTATGCCTACCAGAACCCACGAAAGCTGGGACA

>901_Sample_ASV4

CACCGCGGTTATACGAGAGGCCCAAGTTGACAGACACCGGCGTAAAGAGTGGCTAGGGAA

AAATTACTACTAAAGCCGAACACCTTCAGAACTGTCATACGTTCCCGAAGATAAGAAGCC

CCACCACGAAAGTGACTTTATATTACCCGACCCCACGAAAGCTGTGAAA

>901_Sample_ASV5

CACCGCGGTTATACGAGAGGCCCAAGTTGACAGACACCGGCGTAAAGAGTGGCTAGGGAA

AAATTACTACTAAAGCCGAACACCTTCAGAACTGTCATACGTTCCCGAAGATAAGAAGCC

CCACCACGAAAGTGACTTTATATTACCCGACCCCACGAAAGCTGAGAAA

>901_Sample_ASV6

CACCGCGGTTATACGAGAGGCCCAAGTTGACAGACACCGGCGTAAAGAGTGGCTAGGGAA

AAATTACTACTAAAGCCGAACACCTTCAGAACTGTCATACGTTCCCGAAGATAAGAAGCC

CCACCACGAAAGTGACTTTATTTCCCCTGACCCCACGAAAGCTGAGAAA

>901_Sample_ASV7

CACCGCGATTATACGAGAGGCCCAAGTTGACAGACATCGGCGTAAAGAGTGGTTAAGGAA

AAATTACTACTAAAGCCGAACACCTTCAGAACTGTCATACGTTCCCGAAGATAAGAAGCC

CCACCACGAAAGTGGCTTTATATTACCCGACCCCACGAAAGCTGTGAAA

>901_Sample_ASV8

CACCGCGGTTATACGAGAGGCCCAAGTTGACAGACACCGGCGTAAAGAGTGGCTAGGGAA

AAATTACTACTAAAGCCGAACACCCTCAGAACTGTCATACGTTCCCGAAGGTAAGAAGCC

CCACCACGAAAGTGGCTTTATAATACCCGACCCCACGAAAGCTGAGAAA

>901_Sample_ASV9

CACCGCGGTTATACGAGAGGCCCAAGTTGACAGACACCGGCGTAAAGAGTGGCTAGGGAA

AAATTACTACTAAAGCCGAACACCCTCAGAACTGTCATACGTTCCCGAAGGTAAGAAGCC

CCACCACGAAAGTGGCTTTATAATACCCGACCCCACGAAAGCTGTGAAA

>901_Sample_ASV10

CACCGCGGTTATACGAGAGGCCCAAGTTGACAGACACCGGCGTAAAGAGTGGCTAGGGAA

AAATTACTACTAAAGCCGAACACCTTCAGAACTGTCATACGTTCCCGAAGATAAGAAGCC

CCACCACGAAAGTAGCTTTATTTCCCCTGACCCCACGAAAGCTGAGAAA

>901_Sample_ASV11

CACCGCGGTTATACGAGAGGCCCAAGTTGACAGACACCGGCGTAAAGAGTGGCTAAGGAA

AAATTTCTACTAAAGCCGAACACCTTCAGAACTGTCATACGTTCCCGAAGATAAGAAGCC

CCACCACGAAAGTGGCTTTATATTACCCGACACCACGAAAGCTGAGAAA

>901_Sample_ASV12

CACCGCGGTTATACGAGAGGCCCAAGTTGACAGACACCGGCGTAAAGAGTGGCTAAGGAA

AAATTTCTACTAAAGCCGAACACCTTCAGAACTGTCATACGTTCCCGAAGATAAGAAGCC

CCACCACGAAAGTGGCTTTATATTACCCGACACCACGAAAGCTGTGAAA

>901_Sample_ASV13

CACCGCGGTTATACGAAAGACTCTAGTTGACAAACACGGCGTAAAGGGTGGTTAGGATAA

CAACAAAATAAAGCTAAAGACTTACCAAGCCGTCATACGCCCATGTAAGAGCGAAAACCA

CAAACGAAAGTAACTTTATTACACCATCCGAATCCACGAAAGCTAAGAAA

>901_Sample_ASV14

CACCGCGGTTATACGAAAGACCCTAGTTGATAGATCACGGCGTAAAGGGTGGTTAGGGAA

TACAAAAATAAAGCTAAAAATCCTCTAAGCTGTCATACGCTTCCCGAAGACATGAAACCC

AACCACGAAAGTAGCTTTAAACTCTCCTGACACCACGAAAGCCAAGGAA

>901_Sample_ASV15

CACCGCGGTTATACGAGAGGCCCAAGTTGATAAGCCCGGCGTAAAGAGTGGTTATGGAGA

ATAAAACACTAAAGCTAAAGACCCCCTAGGCTGTTATACGCACCTGGCGGCTCGAACCAC

CTATACGAAAGTAGCTTTACCCCCCTTCCACCAGAATCCACGACAGCTGGGGCA

>901_Sample_ASV16

CACCGCGGTTATACGAGAGGCCCAAGTTGACAGACATCGGCGTAAAGAGTGGTTAAGGAA

ACCCCATTACTAAAGCCGAACGCCCTCAGAACTGTCATACGTTCCCGAAGGTAAGAAGCC

CCACTACGAAAGTGGCTTTAACCACACCCGACCCCACGAAAGCTGTGAAA

>901_Sample_ASV17

CACCGCGGTTATACGAGAGGCCCAAGTTGACAGACACCGGCGTAAAGAGTGGTTAAGGAA

ATTCATCAACTAAAGCCGAATGCCCTCAGGACTGTTATACGTTTGCGAAGGTAAGAAGCC

CTACTACGAAAGTGGCTTTATTCCACCTGATTCCACGAAAGCTGCGGAA

>901_Sample_ASV18

CACCGCGGTTATACGAGAGGCCCCAGTTGATATTAGCGGCGTAAAGGGTGGTTAAGGACT

TTAATAACTAAAGCTAAACACTTCCCCGGCTGTTGTACGTAGACCGGAAGCTTGAAGCCC

AGACACGAAAGTAGCTTTATTTCCCCTGACCCCACGAAAGCTGAGAAA

>901_Sample_ASV19

CACCGCGGTTATACGAGAGGCCCCAGTTGATATTAGCGGCGTAAAGGGTGGTTAAGGACT

TTAATAACTAAAGCTAAACACTTCCCCGGCTGTTGTACGTAGACCGGAAGCTTGAAGCCC

AGACACGAAAGTAGCTTTATTCCCCCTGACCCCACGAAAGCTGAGAAA

>901_Sample_ASV20

CACCGCGGTTATACGAGAGGCCCCAGTTGATATTAGCGGCGTAAAGGGTGGTTAAGGACT

TTAATAACTAAAGCCAAACACTTCCCCGGCTGTTGTACGTAGACCGGAAGCTTGAAGCCC

AGACACGAAAGTAGCTTTATTTCCCCTGACCCCACGAAAGCTGAGAAA

>901_Sample_ASV21

CACCGCGGTCACACGATTAACCCAAGTCAATAGAAGCCGGCGTAAAGAGTGTTTTAGATC

ACCCCCTCCCCAATAAAGCTAAAACTCACCTGAGTTGTAAAAAACTCCAGTTGACACAAA

ATAGACTACGAAAGTGGCTTTAACATATCTGAACACACAATAGCTAAGACC

>901_Sample_ASV22

CACCGCGGTTATACGAAAGACCCCAGTTTATAGACACGGCGTAAAGGGTGGTTATAGGAC

AAACTAACATAGAGCCAAAGACCCCCTAAGCCGTCATACGCTCACGAGGGCTCGAAGCCC

CTATACGAAAGTAGCTCTACCAAAAAATGCTCGACTCCACGAAAGCTGGGACA

>901_Sample_ASV23

CACCGCGGTTATACGAGAGACCCTAATTGATAGACACGGCGTAAAGAGTGGTTTGGGAAA

GCCCAAAAATAAAGCTAAAGACCTTCTAAGCCGTCACACGCACGCCGAAGGCACGAAGCC

CTGACACGAAAGTAGCTTTACTATTACCCCCGACGCCACGAAAGCTAAGAAA

>901_Sample_ASV24

CACCGCGGTTATACGAGAGACCCTAATTGATAGACACGGCGTAAAGAGTGGTTTGGGAAA

GCCCAAAAATAAAGCTAAAGACCTTCTAAGCCGTCACACGCACGCCGAAGGCACGAAGCC

CTGACACGAAAGTAGCTTTACTACTGCCCCTGACGCCACGAAAGCTAAGAAA

>901_Sample_ASV25

AACCGCGGTTATACGAGAGACCCTAATTGATAGCTCACGGCGTAAAGGGTGGTTTGGGGA

AACCTCAAAATAAAGCTAAAGACCTTCTAAGCCGTTACACGCATACCGAAGGCACGAGGC

CCTAACACGAAAGTAGCTTTACTATTACCCCTGACGCCACGAAAGCTAAGAAA

>901_Sample_ASV26

CACCGCGGTTATACGAGAGACCCTAATTGATAGACACGGCGTAAAGAGTGGTTTAGGACA

ACCCAAAAATAAAGCTAAAGACCTTCTAAGCCGTCACACGCATACCGAAGGCACGAAGCC

CTGACACGAAAGTAGCTTTACTATTACCCCCGACGCCACGAAAGCTAAGAAA

>901_Sample_ASV27

CACCGCGGTTATACGAGAGACCCTAATTGATAGACACGGCGTAAAGAGTGGTTTAGGACA

ACCCAAAAATAAAGCTAAAGACCTTCTAAGCCGTCACACGCACGCCGAAGGCACGAAGCC

CTGACACGAAAGTAGCTTTACTATTACCCCCGACGCCACGAAAGCTAAGAAA

>901_Sample_ASV28

CACCGCGGTTATACGAGAGACCCTAATTGATAGGCACGGCGTAAAGAGTGGTTTAGGGTA

ACCCATAAATAAAGCTAAAGACCTTCTAAGCCGTCACACGCACACCGAAGGCACGAGACC

CCAACACGAAAGTAGCTTTACTTCTACCCCTGACGCCACGAAAGCTAAGAAA

>901_Sample_ASV29

CACCGCGGTTATACGAAAGACCCTAGTTGATGGATCACGGCGTAAAGGGTGGTTAAGGGA

TATAAAAATAAAGCTAAAAATCCTCTTAGCTGTCATACGCTTTCCGAAGACATGAGGCCC

AATTACGAAAGTAGCTTTAAACCACCCTGACACCACGAAAGCCAAGAAA

>901_Sample_ASV30

CACCGCGGTTATACGAAAGACCCTAGTTTATAGATGCGGCGTAAAGGGTGGTTAAGGAAA

ACCTAAAATAATGCTAAAGACTCTCAAAGCTGTTATACGCTTCATGAGCACACGAAATCC

AAATACGAAAGTTGCCTTAATAAACTTTGCCTGACCCCACGAAAGCTGAAAAA

>901_Sample_ASV31

CACCGCGGTTATACGAAAGACCCCAGTTTATAGATGCGGCGTAAAGGGTGGTTAAGAGAA

AATCAAAATAAGGCCAAAGATCCCCTAAGCAGTCATACGCTCCACGAGGACACGAAACCC

AAATACGAAAGTTGCCTTAATAAACTTCACCTGATCCCACGAAAGCTGAAAAA

>901_Sample_ASV32

CACCGCGGTTATACGAAAGGCTCTAGTTTATAAATACGGCGTAAAGGGTGGTTAAGGAAA

AATTAAAATAATGCTAAAGACCCCCTAAGCAGTTATACGCTTCACGGAGGCACGAAACCC

AAATACGAAAGTTGCCTTAATAAACTTTACCTGACCCCACGAAAGCTGAAAAA

>901_Sample_ASV33

CACCGCGGTTATACGAAAGGCTCTAGTTTATAAATACGGCGTAAAGGGTGGTTAAGGAAA

AATTAAAATAATGCTAAAGACCCCCTAAGCAGTTATACGCTTCACGGAGGCACGAAACCC

AAATACGAAAGTTGCCTTAATAAACTTTACCTGACCCCACGAAAGCTGAGAAA

>901_Sample_ASV34

CACCGCGGTTATACGAGAGACTCTAGTTGATACCAACGGCGTAAAGAGTGGTTTGGGATG

CCCCTTAAAATAAAGCCAAAGACCTCCCAAGCCGTCAAACGCACCCCGGAGGCACGAAGC

CCCAACGCGAAAGCAACTTTACCTCCCTCCCGACGCCACGAAAGCTAAGAAA

>901_Sample_ASV35

CACCGCGGTTATACGAGAGACCCTAGTTGATAGCTACGGCGTAAAGCGTGGTTAGGAGAA

CAAGATAAATAAAGTCAAACAATCTCTCGGCCGTTATACGTTATCTGAGAATATGAAGTC

CTACCACGAAAGTAACTTTAATTTTTCTGACCCCACGAAAACTGAGGAA

>901_Sample_ASV36

CACCGCGGTTATACGAGAGGCCCTAGTTGATAACTACCGGCGTAAAGAGTGGTTATGGAA

AAATATTTAATAAAGCCGAACACCCCCTCAGCCGTCATACGCACCTGGGAGCACGAAGAC

CTACTGCGAAAGCAGCTTTAATTATGCCTGACCCCACGACAGCTAAGAAA

>901_Sample_ASV37

CACCGCGGTTATACGAGAGGCCCAAGTTGATTATTACCGGCGTAAAGCGTGGTTAAGATT

TTTATAGGACTAAAGCCGAACCCCCTCAGAGCTGTTATACGCACCCGAAGGTTAGAAGTT

CAATCACGAAAGTGGCTTTACAACCCCGAACCCACGAAAGCTAAGATA

>901_Sample_ASV38

CACCGCGGTTATACGAAAGACCCTAGTTGATAGGCACGGCGTAAAGGGTGGTTAGGGATA

TATTAAAAATAAAGTTAAAGAGCCTCTAAGCTGTCGCACGCATCCCGAGAGCTCGAGACC

CAGACACGAAAGTAACTTTAAAATACCTCACCTGACCCCACGAAAGCTAAGAAA

>901_Sample_ASV39

CACCGCGGTTATACGAGAGGCCCAAGTTGATACTCCACGGCGTAAAGAGTGGTTAGAAAA

GACCTACTACTAAAGCCGAACGCCTTCAAAGCTGTTATACGCATCCGAAGGTGAGAAGCC

CACCCACGAAAGTGGCTTTACAACCTTGACTCCACGAAAGCTATGACA

>901_Sample_ASV40

CACCGCGGTTATACGAGAGACCCCAGTTGATAAACACGGCGTAAAGAGTGGTTAAGGGCA

CAAAACAAATAAAGTCAAACAACCTCTTAGCTGTTATACGCATTATGAGGATATGAAGCC

CCCCTACGAAAGTAACTTTACCAACCCCTGACCCCACGAAAGCTAAGAAA

>901_Sample_ASV41

CACCGCGGTTATACGAGAGACCCTAGTTGATATATACGGCGTAAAGAGTGGTTTGGAATA

ACCAAGTAATATAGCCAAAGACCTCCCAAGCCGTCACACGCACCCCGGAGGCACGAAGCC

CAAACACGAAAGTAGCTTTATTAACTTACCGACGCCACGAAAGCTAAGAAA

>901_Sample_ASV42

CACCGCGGTTATACGAAAGACCCTAGTTTATAGGCACGGCGTAAAGGGTGGTTAGGGGAT

AAACAAAATAAAGCCAAAGACCTTCTAAGTCGTCATACACTCATGAAGGCACGAAGCCCA

AATACGAAAGTAACTTTACAACTTAGACCCGACTCCACGAAAGCTGGGGGA

>901_Sample_ASV43

CACCGCGGTTATACGAGAGGCCCAAGTTGACAGATATCGGCGTAAAGAGTGGTTAAGGAA

ATCTTAAAACTAAAGCCGAATGCCTTCAAGACTGTTATACGTTTCCGAAGGTAAGAAGCC

CCACTACGAAAGTGGCTTTACTCCACCTGACTCCACGAAAGCTGTGAAA

>901_Sample_ASV44

CACCGCGGTTATACGAGAGGCCCAAGTTGATAGACATCGGCGTAAAGAGTGGTTAAGGAA

GTCTCAAAACTAAAGCCGAATGCCTTCAAGACTGTTATACGTTTACGAAGGTAAGAAGCC

CCACTACGAAAGTGGCTTTATTCCACCTGACCCCACGAAAGCTGTGAAA

>901_Sample_ASV45

CACCGCGGTTATACGAGAGGCCCAAGTTGATAGACATCGGCGTAAAGAGTGGTTAAGGAA

GTCTCAAAACTAAAGCCGAATGCCTTCAAGACTGTTATACGTTTCCGAAGGTAAGAAGCC

CCACTACGAAAGTGGCTTTACTCCACCTGACTCCACGAAAGCTGTGAAA

>901_Sample_ASV46

CACCGCGGTTATACGAGAGGCCAAAGTTGACAGATATCGGCGTAAAGAGTGGTTAAGGAA

GTCTCAAAACTAAAGCCGAATGCCTTCAAGACTGTTATACGTTTCCGAAGGTAAGAAGCC

CCACTACGAAAGTGGCTTTACTCCACCTGACTCCACGAAAGCTGTGAAA

>901_Sample_ASV47

CACCGCGGTTATACGAGAGGCCAAAGTTGACAGATATCGGCGTAAAGAGTGGTTAAGGAA

ATCTTAAAACTAAAGCCGAATGCCTTCAAGACTGTTATACGTTTCCGAAGGTAAGAAGCC

CCACTACGAAAGTGGCTTTACTCCACCTGACTCCACGAAAGCTGTGAAA

>901_Sample_ASV48

CACCGCGGTTATACGAGAGGCCCAAGTTGACAGATATCGGCGTAAAGAGTGGTTAAGGAA

ATCTTAAAACTAAAGCCGAATGCCTTCAAGACTGTTATACGTTTACGAAGGTAAGAAGCC

CCACTACGAAAGTGGCTTTACTCCACCTGACTCCACGAAAGCTGTGAAA

>901_Sample_ASV49

CACCGCGGTTATACGAAAGACCCTAGTTGATAGCTACGGCGTAAAGGGTGGTTAAGGTAT

ATAACAAATAAAGCTAAAGAGCCTCCAAGCCGTCGTACGCATTCTGAGAGCTCGAAACCC

TAACACGAAAGTAGCTTTAAAAAATAACACCTGACCCCACGAAAGCTAAGAAA

>901_Sample_ASV50

CACCGCGGTTATACGAGAGACCCTAATTGATAGACTACGGCGTAAAGAGTGGTTTAGAAC

AAAAATTAATAAAGCCAAAGATCCTCCTGGCCGTCGCACGCATTTCGAGGATACGAAGCC

CCACTACGAAAGTAGCTTTACTACAACTTTTCTGACCCCACGAAAGCTAAGAAA

>901_Sample_ASV51

CACCGCGGTTATACGAAAGACCCTAGTTGATAGATCACGGCGTAAAGGGTGGTTAAGGAG

AACAAAAATAAAGCTAAAAATCTTCTAAGCTGTCATACGCTTTCCGAAGACATGAGACCC

AACCACGAAAGTAGCTTTAAACTTTCCTGACACCACGAAAGCCAAGAAA

>903_Sample_ASV1

CACCGCGGTTATACGAAAGACCCTAGTTGATGGGTGCGGCGTAAAGGGTGGTTAGGGACT

ACAAAAAATAGAGCTAAAGATCCTCTAGGTCGTCATACACATCACGAGGAAACGAAGCCC

AAATACGAAAGTAGCTCTAAATATAATCCTGACGCCACGAAAGCCAAGAAA

>903_Sample_ASV2

CACCGCGGTTATACGAGAGACCCTAGTTGATTAAAGCGGCGTAAAGAGTGGTTATGGAAC

TATTCTTTTAAAGCAGAAAACCTCTCAAACTGTTATACGCACCCAGAGGTCTGAATCCCT

CACACGAAAGTGACTTTATTTATGCCTACCAGAACCCACGAAAGCTGGGACA

>903_Sample_ASV3

CACCGCGGTTATACGAGAGGCCCAAGTTGACAGACACCGGCGTAAAGAGTGGCTAGGGAA

AAATTACTACTAAAGCCGAACACCTTCAGAACTGTCATACGTTCCCGAAGATAAGAAGCC

CCACCACGAAAGTGACTTTATATTACCCGACCCCACGAAAGCTGGGGGA

>903_Sample_ASV4

CACCGCGGTTATACGAGAGGCCCAAGTTGACAGACACCGGCGTAAAGAGTGGCTAGGGAA

AAATTACTACTAAAGCCGAACACCTTCAGAACTGTCATACGTTCCCGAAGATAAGAAGCC

CCACCACGAAAGTGACTTTATATTACCCGACCCCACGAAAGCTGTGAAA

>903_Sample_ASV5

CACCGCGGTTATACGAGAGGCCCAAGTTGACAGACACCGGCGTAAAGAGTGGCTAGGGAA

AAATTACTACTAAAGCCGAACACCCTCAGAACTGTCATACGTTCCCGAAGGTAAGAAGCC

CCACCACGAAAGTGGCTTTATAATACCCGACCCCACGAAAGCTGTGAAA

>903_Sample_ASV6

CACCGCGGTTATACGAGAGGCCCAAGTTGACAGACACCGGCGTAAAGAGTGGCTAGGGAA

AAATTACTACTAAAGCCGAACACCTTCAGAACTGTCATACGTTCCCGAAGATAAGAAGCC

CCACCACGAAAGTGGCTTTATATTACCCGACCCCACGAAAGCTGTGAAA

>903_Sample_ASV7

CACCGCGGTTATACGAGAGGCCCAAGTTGACAGACACCGGCGTAAAGAGTGGCTAAGGAA

AAATTTCTACTAAAGCCGAACACCTTCAGAACTGTCATACGTTCCCGAAGAAAAGAAGCC

CCACCACGAAAGTGGCTTTATATTACCCGACCCCACGAAAGCTGTGAAA

>903_Sample_ASV8

CACCGCGGTTATACGAGAGGCCCAAGTTGACAGACACCGGCGTAAAGAGTGGCTAAGGAA

AAATTTCTACTAAAGCCGAACACCTTCAGAACTGTCATACGTTCCCGAAGATAAGAAGCC

CCACCACGAAAGTGGCTTTATATTACCCGACACCACGAAAGCTGTGAAA

>903_Sample_ASV9

CACCGCGGTTAAACGAGAGACCCTAGTTGATCATCACGGCGTAAAGAGTGGTTAGGGGAT

TATCATAAATAAAGCCAAAGACCTCCCAAGCTGTCGCACGCATTCCGGAGGCGCGAAGCC

CACACACGAAAGTAGCTTTAACTATTGACCCTGATGCCACGAAAGCTAAGAAA

>903_Sample_ASV10

CACCGCGGTTATACGAGAGACCCTAGTTGATATATACGGCGTAAAGAGTGGTTTGGGATA

TTTCTCCAAAATAAAGCCGAAGATCTCCCAAGCCGTCACACGCATCCCGGAGACACGAAG

CCCTAATACGAAGGTAGCTTTATTAATTCCCCGACGCCACGAAAGCTAAGAAA

>903_Sample_ASV11

CACCGCGGTTATACGAGAGACCCTAGTTGATATATACGGCGTAAAGAGTGGTTTGGGGTT

ATGCCTCCAAAATAAAGCCGAAGATCTCCCAAGCCGTCACACGCATCCCGGAGACACGAA

GCCCTAATACGAAGGTAGCTTTATTAATTCCCCGACGCCACGAAAGCTAAGAAA

>903_Sample_ASV12

CACCGCGGTTATACGAGAGACCCTAGTTGATATATACGGCGTAAAGAGTGGTTTGGGGTT

ATGCCTCCAAAATAAAGCCGAAGATCTCCCAAGCCGTCACACGCACCCCGGAGACACGAA

GCCCTAATACGAAGGTAGCTTTATTAATTCCCCGACGCCACGAAAGCTAAGAAA

>903_Sample_ASV13

CACCGCGGTTATACGAGAGACCCTAGTTGATATATACGGCGTAAAGAGTGGTTTGGGGTT

ATGCCTCCAAAATAAAGCCGAAGATCTCCCAAGCCGTCACACGCATCCCGGAGACACGAA

GCCCTAATACGAAGGTAGCTTTATTAGTACCCCGACGCCACGAAAGCTAAGAAA

>903_Sample_ASV14

CACCGCGGTTATACGAGAGACCCTAGTTGATATATACGGCGTAAAGAGTGGTTTGGGGTT

ATGCCTCCAAAATAAAGTCGAAGATCTCCCAAGCCGTCACACGCACCCCGGAGACACGAA

GCCCTAATACGAAGGTAGCTTTATTAATTCCCCGACGCCACGAAAGCTAAGAAA

>903_Sample_ASV15

CACCGCGGTTATACGAGAGACCCTAGTTGATATATACGGCGTAAAGAGTGGTTTGGGATA

TTTCTCCAAAATAAAGCCGAAGATCTCCCAAGCCGTCACACGCATCCCGGAGACACGAAG

CCCTAATACGAAGGTAGCTTTATTAGTACCCCGACGCCACGAAAGCTAAGAAA

>903_Sample_ASV16

CGCCGCGGTTAAACGAGAGGCCCTAGTTGATGGAACAACGGCGTAAAGGGTGGTTAAGGA

TAGTGAGACAATAAAGTCGAATGGCCCTTTGGCTGTCATACGCTTCTGGGAGCCTGAAGC

CCACTATACGAAAGTAGCTTTAACAATGTCCACCTGACCCCACGAAAACTGAGAAA

>903_Sample_ASV17

CACCGCGGTTATACGAAAGACCCTAGTTGATAGATCACGGCGTAAAGGGTGGTTAGGGAA

TACAAAAATAAAGCTAAAAATCCTCTAAGCTGTCATACGCTTCCCGAAGACATGAAACCC

AACCACGAAAGTAGCTTTAAACTCTCCTGACACCACGAAAGCCAAGGAA

>903_Sample_ASV18

CACCGCGGTTATACGAGAGGCCCAAGTTGATAAGCCCGGCGTAAAGAGTGGTTATGGAGA

ATAAAACACTAAAGCTAAAGACCCCCTAGGCTGTTATACGCACCTGGCGGCTCGAACCAC

CTATACGAAAGTAGCTTTACCCCCCTTCCACCAGAATCCACGACAGCTGGGGCA

>903_Sample_ASV19

CACCGCGGTTATACGAGAGGCCCAAGTTGACAGACATCGGCGTAAAGAGTGGTTAAGGAA

ACCCCATTACTAAAGCCGAACGCCCTCAGAACTGTCATACGTTCCCGAAGGTAAGAAGCC

CCACTACGAAAGTGGCTTTAACCACACCCGACCCCACGAAAGCTAAGAAA

>903_Sample_ASV20

CACCGCGGTTATACGAGAGGCCCAAGTTGACAGACATCGGCGTAAAGAGTGGTTAAGGAA

ACCCCATTACTAAAGCCGAACGCCCTCAGAACTGTCATACGTTCCCGAAGGTAAGAAGCC

CCACTACGAAAGTGGCTTTAACCACACCCGACCCCACGAAAGCTGTGAAA

>903_Sample_ASV21

CACCGCGGTTATACGAGAGGCCCAAGTTGACAGACACCGGCGTAAAGAGTGGTTAAGGAA

ATTCATCAACTAAAGCCGAATGCCCTCAGGACTGTTATACGTTTGCGAAGGTAAGAAGCC

CTACTACGAAAGTGGCTTTATTCCACCTGATTCCACGAAAGCTGCGGAA

>903_Sample_ASV22

CACCGCGGTTATACGAGAGGCCCCAGTTGATATTAGCGGCGTAAAGGGTGGTTAAGGACT

TTAATAACTAAAGCTAAACACTTCCCCGGCTGTTGTACGTAGACCGGAAGCTTGAAGCCC

AGACACGAAAGTAGCTTTATTCCCCCTGACCCCACGAAAGCTGAGAAA

>903_Sample_ASV23

CACCGCGGTCACACGATTAACCCAAGTCAATAGAAGCCGGCGTAAAGAGTGTTTTAGATC

ACCCCCTCCCCAATAAAGCTAAAACTCACCTGAGTTGTAAAAAACTCCAGTTGACACAAA

ATAGACTACGAAAGTGGCTTTAACATATCTGAACACACAATAGCTAAGACC

>903_Sample_ASV24

CACCGCGGTTATACGAGAGGCCCTAGTTGATAAATACGGCGTAAAGAGTGGTTAGAGGAC

TCCCATAATAAAGCCAAAGACCCTCCGGGCCGTCACACGCATACGAGGGCACGAAGTTCA

CACACGAAAGTAGCTTTAAATTACCCGACGCCACGAAAGCTAAGAAA

>903_Sample_ASV25

CACCGCGGTTATACGAAAGACCCTAGTTTATAAGTACGGCGTAAAGGGTGGTTAGGGGAC

AAACAAAATAAAGCCAAAGACCTCCTAAGCCGTCATACGCTTACGGAGGCACGAAGCCCA

AACACGAAAGTAGCTTTACCGAACATGCCCGACTCCACGAAAGCTGGGAAA

>903_Sample_ASV26

CACCGCGGTTATACGAGAGACCCTAGTTGATAAATACGGCGTAAAGAGTGGTTATGGGAA

AAACAAATAAAGTCAAACAACCTCTTAGCTGTTATACGCATTATGAGAGCATGAAGCCCC

CTCACGAAAGTAACTTTAATACCTCCTGACCCCACGAAAGCTAAGAAA

>903_Sample_ASV27

CACCGCGGTTATACGAGAGGCTCAAGTTGATAAACCCCGGCGTAAAGAGTGGTTAAGGAA

AAATAGAAACTAAAGCCGAATGCTTTCAAGACTGTTATACGTTCCCGAGAGTAAGAAGAC

CAATTACGAAAGTGGCTTTACTTTACCTGACCCCACGAAAGCTACGACA

>903_Sample_ASV28

CACCGCGGTTATACGAGAGACCCTAATTGATAGACACGGCGTAAAGAGTGGTTTGGGAAA

GCCCAAAAATAAAGCTAAAGACCTTCTAAGCCGTCACACGCACGCCGAAGGCACGAAGCC

CTGACACGAAAGTAGCTTTACTATTACCCCCGACGCCACGAAAGCTAAGAAA

>903_Sample_ASV29

AACCGCGGTTATACGAGAGACCCTAATTGATAGCTCACGGCGTAAAGGGTGGTTTGGGGA

AACCTCAAAATAAAGCTAAAGACCTTCTAAGCCGTTACACGCATACCGAAGGCACGAGGC

CCTAACACGAAAGTAGCTTTACTATTACCCCTGACGCCACGAAAGCTAAGAAA

>903_Sample_ASV30

CACCGCGGTTATACGAAAGACCCCAGTTTATAGATGCGGCGTAAAGGGTGGTTAAGAGAA

AATCAAAATAAGGCCAAAGATCCCCTAAGCAGTCATACGCTCCACGAGGACACGAAACCC

AAATACGAAAGTTGCCTTAATAAACTTCACCTGATCCCACGAAAGCTGAAAAA

>903_Sample_ASV31

CACCGCGGTTAAACGAGAGGCCCTAGTTGATAGTACAACGGCGTAAAGGGTGGTTAAGGA

TAGTGAAATAATAAAGTCGAATGGCCCTTTGGCTGTCATACGCTTCTAGGAGTCCGAAGC

CCAATATACGAAAGTAACTTTAAGAGCGCCCACCTGACCCCACGAAAACTGAGAAA

>903_Sample_ASV32

CACCGCGGTTATACGAAAGACCCTAGTTGATAGCCACGGCGTAAAGGGTGGTTAAGGTAT

ACAACAAATAAAGCTAAATAGCCTCTAGGCCGTCGCACGCATTCCGAGAGCTCGAAACCC

AAAAACGAAAGTAGCTTTAAAATAAACAGACCTGACCCCACGAAAGCTAAGAAA

>903_Sample_ASV33

CACCGCGGTTATACGAGAGGCCCAAGTTGATACTCCACGGCGTAAAGAGTGGTTAGAAAA

GACCTACTACTAAAGCCGAACGCCTTCAAAGCTGTTATACGCATCCGAAGGTGAGAAGCC

CACCCACGAAAGTGGCTTTACAACCTTGACTCCACGAAAGCTGGGGGA

>903_Sample_ASV34

CACCGCGGTTATACGAGAGGCCCAAGTTGATACTCCACGGCGTAAAGAGTGGTTAGAAAA

GACCTACTACTAAAGCCGAACGCCTTCAAAGCTGTTATACGCATCCGAAGGTGAGAAGCC

CACCCACGAAAGTGGCTTTACAACCTTGACTCCACGAAAGCTATGACA

>903_Sample_ASV35

CACCGCGGTTATACGAGAGACCCCAGTTGATAAACACGGCGTAAAGAGTGGTTAAGGGCA

CAAAACAAATAAAGTCAAACAACCTCTTAGCTGTTATACGCATTATGAGGATATGAAGCC

CCCCTACGAAAGTAACTTTACCAACCCCTGACCCCACGAAAGCTAAGAAA

>903_Sample_ASV36

CACCGCGGTTATACGAGAGACCCTAGTTGATATATACGGCGTAAAGAGTGGTTTGGAATA

ACCAAGTAATATAGCCAAAGACCTCCCAAGCCGTCACACGCACCCCGGAGGCACGAAGCC

CAAACACGAAAGTAGCTTTATTAACTTACCGACGCCACGAAAGCTAAGAAA

>903_Sample_ASV37

CACCGCGGTTATACGAAAGACCCTAGTTTATAGGCACGGCGTAAAGGGTGGTTAGGGGAT

AAACAAAATAAAGCCAAAGACCTTCTAAGTCGTCATACACTCATGAAGGCACGAAGCCCA

AATACGAAAGTAACTTTACAACTTAGACCCGACTCCACGAAAGCTGGGGGA

>903_Sample_ASV38

CACCGCGGTTATACGAGAGGCCCAAGTTGATAGACATCGGCGTAAAGAGTGGTTAAGGAA

GTCTCAAAACTAAAGCCGAATGCCTTCAAGACTGTTATACGTTTCCGAAGGTAAGAAGCC

CCACTACGAAAGTGGCTTTACTCCACCTGACTCCACGAAAGCTGTGAAA

>903_Sample_ASV39

CACCGCGGTTATACGAAAGACCCTAGTTGATAGACCACGGCGTAAAGGGTGGTTAAGGAA

CACAAAAATAAAGCTAAAGATCCTCTAAGCTGTCATACGCTTCCCGAAGACATGAAACCC

AATCACGAAAGTAGCTTTAAACTGTCCTGACACCACGAAAGCCAAGAAA

>903_Sample_ASV40

CACCGCGGTTATACGAGAGACCCTAATTGATAGACTACGGCGTAAAGAGTGGTTTAGAAC

AAAAATTAATAAAGCCAAAGATCCTCCTGGCCGTCGCACGCATTTCGAGGATACGAAGCC

CCACTACGAAAGTAGCTTTACTACAACTTTTCTGACCCCACGAAAGCTAAGAAA

>905_Sample_ASV1

CACCGCGGTTATACGAGAGACCCTAGTTGATTAAAGCGGCGTAAAGAGTGGTTATGGAAC

TATTCTTTTAAAGCAGAAAACCTCTCAAACTGTTATACGCACCCAGAGGTCTGAATCCCT

CACACGAAAGTGACTTTATTTATGCCTACCAGAACCCACGAAAGCTGGGACA

>905_Sample_ASV2

CACCGCGGTTATACGAAAGACCCTAGTTTATAGGTACGGCGTAAAGGGTGGTTAGGGGAC

AAACAAAATAAAGCCAAAGACCTCCTAAGCCGTCATACGCTCACGGAAGCACGAAGCCCA

AACACGAAAGTAGCTTTACCGAACATGCCCGACTCCACGAAAGCTGGGGAA

>905_Sample_ASV3

CACCGCGATTATACGAGAGGCCCAAGTTGACAGACATCGGCGTAAAGAGTGGTTAAGGAA

AAATTACTGCTAAAGCCGAACACCTTCAGAACTGTCATACGTTCCCGAAGATAAGAAGCC

CCACCACGAAAGTGACTTTATATTACCCGACCCCACGAAAGCTGTGAAA

>905_Sample_ASV4

CATGGCGATTATACGAGAGGCCCAAGTTGACAGACACTGGCGTAAAGAGTGGCTAAGGAA

AAACTACTACTAAAGCCGAACACCTTCAGAACTGTCATACGTTCCCGAAGATAAGAAGCC

CCACCACGAAAGTGACTTTATATTACCCGACCCCACGAAAGCTGTGAAA

>905_Sample_ASV5

CATGGCGATTATACGAGAGGCCCAAGTTGACAGACACTGGCGTAAAGAGTGGCTAGGGAA

AAATTACTACTAAAGCCGAACACCTTCAGAACTGTCATACGTTCCCGAAGATAAGAAGCC

CCACCACGAAAGTGACTTTATATTACCCGACCCCACGAAAGCTGTGAAA

>905_Sample_ASV6

CACCGCGGTTATACGAGAGGCCCAAGTTGACAGACACCGGCGTAAAGAGTGGCTAGGGAA

AAATTACTACTAAAGCCGAACACCTTCAGAACTGTCATACGTTCCCGAAGATAAGAAGCC

CCACCACGAAAGTGACTTTATATTACCCGACCCCACGAAAGCCAAGAAA

>905_Sample_ASV7

CACCGCGGTTATACGAGAGGCCCAAGTTGACAGACACCGGCGTAAAGAGTGGCTAGGGAA

AAATTACTACTAAAGCCGAACACCTTCAGAACTGTCATACGTTCCCGAAGATAAGAAGCC

CCACCACGAAAGTGACTTTATATTACCCGACCCCACGAAAGCTGTGAAA

>905_Sample_ASV8

CACCGCGGTTATACGAAAGACCCTAGTTGACAGACACCGGCGTAAAGAGTGGCTAGGGAA

AAATTACTACTAAAGCCGAACACCTTCAGAACTGTCATACGTTCCCGAAGATAAGAAGCC

CCACCACGAAAGTGACTTTATATTACCCGACACCACGAAAGCCAAGAAA

>905_Sample_ASV9

CATGGCGATTATACGAGAGGCCCAAGTTGACAGACACTGGCGTAAAGAGTGGCTAAGGAA

AAATTACTACTAAAGCCGAACACCTTCAGAACTGTCATACGTTCCCGAAGATAAGAAGCC

CCACCACGAAAGTGACTTTATATTACCCGACCCCACGAAAGCTGTGAAA

>905_Sample_ASV10

CACCGCGGTTATACGAGAGGCCCAAGTTGACAGACATCGGCGTAAAGAGTGGTTAAGGAA

AAATTACTGCTAAAGCCGAACACCCTCAGAACTGTCATACGTTCCCGAAGATAAGAAGCC

CCACCACGAAAGTGACTTTATATTACCCGACCCCACGAAAGCTGTGAAA

>905_Sample_ASV11

CACCGCGGTTATACGAAAGACCCTAGTTGACAGACACCGGCGTAAAGAGTGGCTAGGGAA

AAATTACTACTAAAGCCGAACACCTTCAGAACTGTCATACGTTCCCGAAGATAAGAAGCC

CCACCACGAAAGTGACTTTATATTACCCGACCCCACGAAAGCCAAGAAA

>905_Sample_ASV12

CACCGCGGTTATACGAGAGGCCCAAGTTGACAGACACCGGCGTAAAGAGTGGCTAGGGAA

AAATTACTACTAAAGCCGAACACCCTCAGAACTGTCATACGTTCCAGAAGGCAAGAAGCT

CCACCACGAAAGTGACTTTATATTACCCGACCCCACGAAAGCTGTGAAA

>905_Sample_ASV13

CATGGCGATTATACGAGAGGCCCAAGTTGACAGACACTGGCGTAAAGAGTGGCTAAGGAA

AAATTTCTACTAAAGCCGAACACCTTCAGAACTGTCATACGTTCCCGAAGATAAGAAGCC

CCACCACGAAAGTGACTTTATATTACCCGACCCCACGAAAGCTGTGAAA

>905_Sample_ASV14

CATGGCGATTATACGAGAGGCCCAAGTTGACAGACACTGGCGTAAAGAGTGGCTAAGGAA

AAATTACTACTAAAGCCGAACACCTTCAGAACTGTCATACGTTCCCGAAGGTAAGAAGCC

CCACCACGAAAGTGACTTTATATTACCCGACCCCACGAAAGCTGTGAAA

>905_Sample_ASV15

CACCGCGATTATACGAGAGGCCCAAGTTGACAGACATCGGCGTAAAGAGTGGTTAAGGAA

AAATTACTACTAAAGCCGAACACCTTCAGAACTGTCATACGTTCCCGAAGATAAGAAGCC

CCACCACGAAAGTGACTTTATATTACCCGACCCCACGAAAGCTGTGAAA

>905_Sample_ASV16

CACCGCGGTTATACGAGAGGCCCAAGTTGACAGACACCGGCGTAAAGAGTGGCTAGGGAA

AAATTACTACTAAAGCCGAACACCCTCAGAACTGTCATACGTTCCCGAAGGTAAGAAGCC

CCACCACGAAAGTGGCTTTATAATACCCGACCCCACGAAAGCTGTGAAA

>905_Sample_ASV17

CACCGCGGTTATACGAGAGGCCCAAGTTGACAGACACCGGCGTAAAGAGTGGCTAGGGAA

AAATTACTACTAAAGCCGAACACCCTCAGAACTGTCATACGTTCCCGAAGGTAAGAAGCC

CCACCACGAAAGTGGCTTTATAATACCCGACACCACGAAAGCCAAGAAA

>905_Sample_ASV18

CACCGCGGTTATACGAGAGGCCCAAGTTGACAGACACCGGCGTAAAGAGTGGCTAGGGAA

AAATTACTACTAAAGCCGAACACCTTCAGAACTGTCATACGTTCCAGAAGGCAAGAAGCT

CCACCACGAAAGTGGCTTTATATTACCCGACCCCACGAAAGCTGTGAAA

>905_Sample_ASV19

CACCGCGGTTATACGAGAGGCCCAAGTTGACAGACACCGGCGTAAAGAGTGGCTAGGGAA

AAATTACTACTAAAGCCGAACACCTTCAGAACTGTCATACGTTCCCGAAGATAAGAAGCC

CCACCACGAAAGTGGCTTTATATTACCCGACACCACGAAAGCCAAGAAA

>905_Sample_ASV20

CACCGCGGTTATACGAGAGGCCCAAGTTGACAGACACCGGCGTAAAGAGTGGCTAGGGAA

AAATTACTACTAAAGCCGAACACCTTCAGAACTGTCATACGTTCCCGAAGATAAGAAGCC

CCACCACGAAAGTGGCTTTACAATGCCTGACCCCACGAAAGCTGTGAAA

>905_Sample_ASV21

CACCGCGGTTATACGAGAGGCCCAAGTTGACAGACACCGGCGTAAAGAGTGGCTAGGGAA

AAATTACTACTAAAGCCGAACACCCTCAGAACTGTCATACGTTCCAGAAGGCAAGAAGCT

CCACCACGAAAGTGGCTTTATATTACCCGACCCCACGAAAGCTGTGAAA

>905_Sample_ASV22

CACCGCGGTTATACGAGAGGCCCAAGTTGACAGACACCGGCGTAAAGAGTGGCTAGGGAA

AAATTACTACTAAAGCCGAACACCTTCAGAACTGTCATACGTTCCAGAAGGCAAGAAGCT

CCACCACGAAAGTGGCTTTATAATACCCGACCCCACGAAAGCTGTGAAA

>905_Sample_ASV23

CACCGCGGTTATACGAGAGGCCCAAGTTGACAGACACCGGCGTAAAGAGTGGCTAGGGAA

AAATTACTACTAAAGCCGAACACCTTCAGAACTGTCATACGTTCCCGAAGATAAGAAGCT

CCACCACGAAAGTGGCTTTACAATGCCTGACCCCACGAAAGCTGTGAAA

>905_Sample_ASV24

CACCGCGGTTATACGAGAGGCCCAAGTTGACAGACACCGGCGTAAAGAGTGGCTAAGGAA

AAATTTCTACTAAAGCCGAACACCTTCAGAACTGTCATACGTTCCCGAAGATAAGAAGCC

CCACCACGAAAGTGGCTTTATATTACCCGACACCACGAAAGCTGTGAAA

>905_Sample_ASV25

CACCGCGGTTATACGAGAGGCCCAAGTTGACAGACACCGGCGTAAAGAGTGGCTAAGGAA

AAATTTCTACTAAAGCCGAACACCTTCAGAACTGTCATACGTTCCCGAAGATAAGAAGCC

CCACCACGAAAGTGGCTTTATATTACCCGACACCACGAAAGCCAAGAAA

>905_Sample_ASV26

CACCGCGGTTATACGAGAGACCCTAGTTGATATATACGGCGTAAAGAGTGGTTTGGGGTT

ATGCCTCCAAAATAAAGCCGAAGATCTCCCAAGCCGTCACACGCATCCCGGAGACACGAA

GCCCTAATACGAAGGTAGCTTTATTAGTACCCCGACGCCACGAAAGCTAAGAAA

>905_Sample_ASV27

CACCGCGGTTATACGAGAGACCCTAGTTGATATATACGGCGTAAAGAGTGGTTTGGGGTT

ATGCCTCCAAAATAAAGCCGAAGATCTCCCAAGCCGTCACACGCATCCCGGAGACACGAA

GCCCTAATACGAAGGTAGCTTTATTAATTCCCCGACGCCACGAAAGCTAAGAAA

>905_Sample_ASV28

CACCGCGGTTATACGAGAGGCCCAAGTTGATAAGCCCGGCGTAAAGAGTGGTTATGGAGA

ATAAAACACTAAAGCTAAAGACCCCCTAGGCTGTTATACGCACCTGGCGGCTCGAACCAC

CTATACGAAAGTAGCTTTACCCCCCTTCCACCAGAATCCACGACAGCTGGGGCA

>905_Sample_ASV29

CACCGCGGTTATACGAGAGGCCCTAGTTGATAGCCACGGCGCAAAGAGTGGTTAAGGAGC

CCCACTAAATAAAGTCGAACACTTCCTAGGCCGTTATACGTTTTCTAGAAGCACGAAACC

CAATTACACGAAAGCAACTTTATACTAAACAACCTGACCCCACGAAAGCTAAGAAA

>905_Sample_ASV30

CACCGCGGTTATACGAGAGGCCCAAGTTGACAGACATCGGCGTAAAGAGTGGTTAAGGAA

ACCCCATTACTAAAGCCGAACGCCCTCAGAACTGTCATACGTTCCCGAAGGTAAGAAGCC

CCACTACGAAAGTGGCTTTAACCACACCCGACCCCACGAAAGCTGTGAAA

>905_Sample_ASV31

CACCGCGGTTATACGAGAGGCCCAAGTTGATAGATATCGGCGTAAAGAGTGGTTAAGGAA

ACCCCGTTACTAAAGCCGAACGCCCTCAGAACTGTCATACGTTCCCGAAGGTAAGAAGCC

CCACTACGAAAGTGGCTTTAACCACACCCGACCCCACGAAAGCTGTGAAA

>905_Sample_ASV32

CACCGCGGTTATACGAGAGGCCCAAGTTGACAGACACCGGCGTAAAGAGTGGTTAAGGAA

ATTCATCAACTAAAGCCGAATGCCCTCAGGACTGTTATACGTTTGCGAAGGTAAGAAGCC

CTACTACGAAAGTGGCTTTATTCCACCTGATTCCACGAAAGCTGCGGAA

>905_Sample_ASV33

CACCGCGGTTATACGAAAGACCCTAGTTTATAAGTACGGCGTAAAGGGTGGTTAGGGGAC

AAACAAAATAAAGCCAAAGACCTCCTAAGCCGTCATACGCTTACGGAGGCACGAAGCCCA

AACACGAAAGTAGCTTTACCGAACATGCCCGACTCCACGAAAGCTGGGAAA

>905_Sample_ASV34

CACCGCGGTTATACGAGAGGCCCCAGTTGATAAACACGGCGTAAAGGGTGGTTAAGGGAG

CATAAGAATAAAGCCGAAGGGCCCTTTGGCCGTTATACGCTTCTAGGTGTCCGAAGCCCA

AACACGAAAGTAGCTTTAGTTAAGCCCACCTGACTCCACGAAAACTGAGAAA

>905_Sample_ASV35

CACCGCGGTTATACGAGAGACCCTAGTTGATAAATACGGCGTAAAGAGTGGTTATGGGAA

AAACAAATAAAGTCAAACAACCTCTTAGCTGTTATACGCATTATGAGAGCATGAAGCCCC

CTCACGAAAGTAACTTTAATACCTCCTGACCCCACGAAAGCTAAGAAA

>905_Sample_ASV36

CACCGCGGTTATACGAGAGGCTCAAGTTGATAAACCCCGGCGTAAAGAGTGGTTAAGGAA

AAATAGAAACTAAAGCCGAATGCTTTCAAGACTGTTATACGTTCCCGAGAGTAAGAAGAC

CAATTACGAAAGTGGCTTTACTTTACCTGACCCCACGAAAGCTACGACA

>905_Sample_ASV37

CACCGCGGTTATACGAGAGGCTCAAGTTGATGAACCCCGGCGTAAAGAGTGGTTAAGGGA

GATCAAAACTAAAGCCGAATGCTTTCAAAGCTGTTATACGCTTCCGAAAGTAAGAAGCTC

AATCACGAAAGTGGCTTTACTTTACCTGACCCCACGAAAGCTACGACA

>905_Sample_ASV38

AACCGCGGTTATACGAGAGACCCTAATTGATAGCTCACGGCGTAAAGGGTGGTTTGGGGA

AACCTCAAAATAAAGCTAAAGACCTTCTAAGCCGTTACACGCATACCGAAGGCACGAGGC

CCTAACACGAAAGTAGCTTTACTATTACCCCTGACGCCACGAAAGCTAAGAAA

>905_Sample_ASV39

CACCGCGGTTATACGAAAGACCCTAGTTGATGGATCACGGCGTAAAGGGTGGTTAAGGGA

TATAAAAATAAAGCTAAAAATCCTCTTAGCTGTCATACGCTTTCCGAAGACATGAGGCCC

AATTACGAAAGTAGCTTTAAACCACCCTGACACCACGAAAGCCAAGAAA

>905_Sample_ASV40

CACCGCGGTTATACGAAAGACCCCAGTTTATAGATGCGGCGTAAAGGGTGGTTAAGAGAA

AATCAAAATAAGGCCAAAGATCCCCTAAGCAGTCATACGCTCCACGAGGACACGAAACCC

AAATACGAAAGTTGCCTTAATAAACTTCACCTGATCCCACGAAAGCTGAAAAA

>905_Sample_ASV41

CACCGCGGTTATACGAGAGGCCCAAGTTGACAAACCCCGGCGTAAAGAGTGGTTAAGGAA

AATTTAAAACTAAAGCCGAATGCCCCCAAAGCTGTTATACGCACCCGAGGGTAAGAAGCC

CAATCACGAAAGTGGCTTTACACTACCTGAACCCACGAAAGCTATGAAA

>905_Sample_ASV42

CACCGCGGTTATACGAGAGGCCCAAGTTGACAAACCCCGGCGTAAAGAGTGGTTAAGGAA

AATTTAAAACTAAAGCCGAATGCCCCCAAAGCTGTTATACGCACCCGAGGATAAGAAGCC

CAATCACGAAAGTGGCTTTACACTACCTGAACCCACGAAAGCTATGAAA

>905_Sample_ASV43

CACCGCGGTTATACGAGAGGCCCTAGTTGATAACTACCGGCGTAAAGAGTGGTTATGGAA

AAATATTTAATAAAGCCGAACACCCCCTCAGCCGTCATACGCACCTGGGAGCACGAAGAC

CTACTGCGAAAGCAGCTTTAATTATGCCTGACCCCACGACAGCTAAGAAA

>905_Sample_ASV44

CACCGCGGTTATACGAAAGACCCTAGTTTATAGACACGGCGTAAAGGGTGGTTCGGGACT

AAACAAAATAAAGCCAAAGACCTCCTAAGCCGTCATACGCTCACGGAAGCACGAAGCCCA

AGTACGAAAGTAGCTTTACAAAACATGCCTGACTCCACGAAAGCTAGGAAA

>905_Sample_ASV45

CACCGCGGTTATACGAGAGGCCCAAGTTGATACTCCACGGCGTAAAGAGTGGTTAGAAAA

GACCTACTACTAAAGCCGAACGCCTTCAAAGCTGTTATACGCATCCGAAGGTGAGAAGCC

CACCCACGAAAGTGGCTTTACAACCTTGACTCCACGAAAGCTATGACA

>905_Sample_ASV46

CACCGCGGTTATACGAGAGACCCCAGTTGATAAACACGGCGTAAAGAGTGGTTAAGGGCA

CAAAACAAATAAAGTCAAACAACCTCTTAGCTGTTATACGCATTATGAGGATATGAAGCC

CCCCTACGAAAGTAACTTTACCAACCCCTGACCCCACGAAAGCTAAGAAA

>905_Sample_ASV47

CACCGCGGTTATACGAAAGACCCTAGTTTATAGGCACGGCGTAAAGGGTGGTTAGGGGAT

AAACAAAATAAAGCCAAAGACCTTCTAAGTCGTCATACACTCATGAAGGCACGAAGCCCA

AATACGAAAGTAACTTTACAACTTAGACCCGACTCCACGAAAGCTGGGGGA

>905_Sample_ASV48

CACCGCGGTTATACGAAAGACCCTAGTTGATAGCCACGGCGTAAAGGGTGGTTAAGGTAA

CTAATAAATAAAGCTAAAGAGCCTCTAAGCCGTCGCACGCATTCCGAGAGCTCGAAGCCC

AAACACGAAAGTAGCTTTAAAATAAAGCACACCTGACCCCACGAAAGCTAAGAAA

>905_Sample_ASV49

CACCGCGGTTATACGAGAGACCCTAATTGATAGACTACGGCGTAAAGAGTGGTTTAGAAC

AAAAATTAATAAAGCCAAAGATCCTCCTGGCCGTCGCACGCATTTCGAGGATACGAAGCC

CCACTACGAAAGTAGCTTTACTACAACTTTTCTGACCCCACGAAAGCTAAGAAA

>911_Sample_ASV1

CACCGCGGTTATACGAGAGGCCCAAGTTGACAGACATCGGCGTAAAGAGTGGTTAAGGAA

AACCTTCAACTAAAGTCGAACGCTCTCAAGACTGTCGTACGTTTCCGAGAGTATGAAGCC

CCACCACGAAAGTGACTTTACCTCACCTGACTCCACGAAAGCTGCGAAA

>911_Sample_ASV2

CACCGCGGTTATACGAAAGACCCTAGTTGATGGGTGCGGCGTAAAGGGTGGTTAGGGACT

ACAAAAAATAGAGCTAAAGATCCTCTAGGTCGTCATACACATCACGAGGAAACGAAGCCC

AAATACGAAAGTAGCTCTAAATATAATCCTGACGCCACGAAAGCCAAGAAA

>911_Sample_ASV3

CACCGCGGTTATACGAAAGACCCTAGTTGATAACTACGGCATAAAGGGTGGTTAGGGAGT

TTAAAAAATAAAGCCAAAGCTCTTCTAAGCTGTCATACGCTCTCTGAAAGAACGAGGCCC

AAACACGAAAGTAGCTTTAAATAAATAACCTGATACCACGAAAGCCAAGAAA

>911_Sample_ASV4

CACCGCGGTTATACGAGAGACCCTAGTTGATTAAAGCGGCGTAAAGAGTGGTTATGGAAC

TATTCTTTTAAAGCAGAAAACCTCTCAAACTGTTATACGCACCCAGAGGTCTGAATCCCT

CACACGAAAGTGACTTTATTTATGCCTACCAGAACCCACGAAAGCTGGGACA

>911_Sample_ASV5

CACCGCGGTTATACGAAAGACCCTAGTTGACAGACACCGGCGTAAAGAGTGGCTAGGGAA

AAATTACTACTAAAGCCGAACACCTTCAGAACTGTCATACGTTCCCGAAGATAAGAAGCC

CCACCACGAAAGTGACTTTATATTACCCGACCCCACGAAAGCTGGGAGA

>911_Sample_ASV6

CATGGCGATTATACGAGAGGCCCAAGTTGACAGACACTGGCGTAAAGAGTGGCTAAGGAA

AAATTACTACTAAAGCCGAACACCTTCAGAACTGTCATACGTTCCCGAAGATAAGAAGCC

CCACCACGAAAGTGACTTTATATTACCCGACCCCACGAAAGCTGTGAAA

>911_Sample_ASV7

CACCGCGGTTATACGAGAGGCCCAAGTTGACAGACACCGGCGTAAAGAGTGGCTAGGGAA

AAATTACTACTAAAGCCGAACACCTTCAGAACTGTCATACGTTCCCGAAGATAAGAAGCC

CCACCACGAAAGTGACTTTATATTACCCGACCCCACGAAAGCTGTGAAA

>911_Sample_ASV8

CACCGCGATTATACGAGAGGCCCAAGTTGACAGACATCGGCGTAAAGAGTGGTTAAGGAA

AAATTACTACTAAAGCCGAACACCTTCAGAACTGTCATACGTTCCCGAAGATAAGAAGCC

CCACCACGAAAGTGACTTTATATTACCCGACCCCACGAAAGCTGTGAAA

>911_Sample_ASV9

CACCGCGGTTATACGAGAGGCCCAAGTTGACAGACACCGGCGTAAAGAGTGGCTAGGGAA

AAATTACTACTAAAGCCGAACACCTTCAGAACTGTCATACGTTCCCGAAGATAAGAAGCC

CCACCACGAAAGTGACTTTATATTACCCGACCCCACGAAAGCTGGGAGA

>911_Sample_ASV10

CACCGCGGTTATACGAGAGGCCCAAGTTGACAGACACCGGCGTAAAGAGTGGCTAGGGAA

AAATTACTACTAAAGCCGAACACCTTCAGAACTGTCATACGTTCCCGAAGATAAGAAGCC

CCACCACGAAAGTGGCTTTACAATGCCTGACCCCACGAAAGCTGGGAGA

>911_Sample_ASV11

CACCGCGGTTATACGAGAGGCCCAAGTTGACAGACACCGGCGTAAAGAGTGGCTAGGGAA

AAATTACTACTAAAGCCGAACACCTTCAGAACTGTCATACGTTCCCGAAGATAAGAAGCC

CCACCACGAAAGTGGCTTTATATTACCCGACCCCACGAAAGCTGTGAAA

>911_Sample_ASV12

CACCGCGGTTATACGAGAGGCCCAAGTTGACAGACACCGGCGTAAAGAGTGGCTAGGGAA

AAATTACTACTAAAGCCGAACACCCTCAGAACTGTCATACGTTCCCGAAGGTAAGAAGCC

CCACCACGAAAGTGGCTTTATAATACCCGACCCCACGAAAGCTGTGAAA

>911_Sample_ASV13

CACCGCGGTTATACGAGAGGCCCAAGTTGACAGACACCGGCGTAAAGAGTGGCTAGGGAA

AAATTACTACTAAAGCCGAACACCCTCAGAACTGTCATACGTTCCCGAAGGTAAGAAGCC

CCACCACGAAAGTGGCTTTATAATACCCGACCCCACGAAAGCTGGGAGA

>911_Sample_ASV14

CACCGCGGTTATACGAGAGGCCCAAGTTGACAGACACCGGCGTAAAGAGTGGCTAAGGAA

AAATTTCTACTAAAGCCGAACACCTTCAGAACTGTCATACGTTCCCGAAGATAAGAAGCC

CCACCACGAAAGTGGCTTTATATTACCCGACACCACGAAAGCTGGGAGA

>911_Sample_ASV15

CACCGCGGTTATACGAGAGGCCCAAGTTGACAGACACCGGCGTAAAGAGTGGCTAAGGAA

AAATTTCTACTAAAGCCGAACACCTTCAGAACTGTCATACGTTCCCGAAGAAAAGAAGCC

CCACCACGAAAGTGGCTTTATATTACCAGACCCCACGAAAGCTGTGAAA

>911_Sample_ASV16

CACCGCGGTTATACGAGAGGCCCAAGTTGACAGACACCGGCGTAAAGAGTGGCTAAGGAA

AAATTTCTACTAAAGCCGAACACCTTCAGAACTGTCATACGTTCCCGAAGAAAAGAAGCC

CCACCACGAAAGTGGCTTTATATTACCCGACCCCACGAAAGCTGTGAAA

>911_Sample_ASV17

CACCGCGGTTATACGAGAGGCCCAAGTTGACAGACACCGGCGTAAAGAGTGGCTAAGGAA

AAATTTCTACTAAAGCCGAACACCTTCAGAACTGTCATACGTTCCCGAAGATAAGAAGCC

CCACCACGAAAGTGGCTTTATATTACCCGACACCACGAAAGCTGTGAAA

>911_Sample_ASV18

CACCGCGGTTATACGAGAGGCCCAAGTTGACAGACACCGGCGTAAAGAGTGGCTAAGGAA

AAATTTCTACTAAAGCCGAACACCTTCAGAACTGTCATACGTTCCCGAAGAAAAGAAGCC

CCACCACGAAAGTGGCTTTATATTACCCGACCCCACGAAAGCTGGGAGA

>911_Sample_ASV19

CACCGCGGTTATACGAGAGGCCCAAGTTGACAGACACCGGCGTAAAGAGTGGCTAAGGAA

AAATTTCTACTAAAGCCGAACACCTTCAGAACTGTCATACGTTCCCGAAGATAAGAAGCC

CCACCACGAAAGTGGCTTTATATTACCCGACCCCACGAAAGCTGGGAGA

>911_Sample_ASV20

CACCGCGGTTATACGAAAGACCCTAGTTTATAGAAACGGCGTAAAGGGTGGTTAAGGGAC

AAACAAAATAAGGCCAAAGACTTCCTAAGCCGTCATACGCTCCACGAAAGCACGAAGCCC

AAACACGAAAGTAGCCTTAATAACTTCACCTGACCCCACGAAAGCTGGGAGA

>911_Sample_ASV21

CACCGCGGTTATACGAGAGACCCTAGTTGATATATACGGCGTAAAGAGTGGTTTGGGATA

TTTCTCCAAAATAAAGTCGAAGATCTCCCAAGCCGTCACACGCATCCCGGAGACACGAAG

CCCTGATACGAAGGTAGCTTTATTAACACCCCGACGCCACGAAAGCTAAGAAA

>911_Sample_ASV22

CACCGCGGTTATACGAGAGGCCCAAGTTGATAAGCCCGGCGTAAAGAGTGGTTATGGAGA

ATAAAACACTAAAGCTAAAGACCCCCTAGGCTGTTATACGCACCTGGCGGCTCGAACCAC

CTATACGAAAGTAGCTTTACCCCCCTTCCACCAGAATCCACGACAGCTGGGGCA

>911_Sample_ASV23

CACCGCGGTTATACGAGAGGCCCAAGTTGACAGACATCGGCGTAAAGAGTGGTTAAGGAA

ACCCCATTACTAAAGCCGAACGCCCTCAGAACTGTCATACGTTCCCGAAGGTAAGAAGCC

CCACTACGAAAGTGGCTTTAACCACACCCGACCCCACGAAAGCTGTGAAA

>911_Sample_ASV24

CACCGCGGTTATACGAGAGGCCCAAGTTGACAGACATCGGCGTAAAGAGTGGTTAAGGAA

ACCCCATTACTAAAGCCGAACGCCCTCAGAACTGTCATACGTTCCCGAAGGTAAGAAGCC

CCACTACGAAAGTGGCTTTAACCACACCCGACCCCACGAAAGCTAAGAAA

>911_Sample_ASV25

CACCGCGGTTATACGAGAGGCCCAAGTTGACAGACACCGGCGTAAAGAGTGGTTAAGGAA

ATTCATCAACTAAAGCCGAATGCCCTCAGGACTGTTATACGTTTGCGAAGGTAAGAAGCC

CTACTACGAAAGTGGCTTTATTCCACCTGATTCCACGAAAGCTGCGGAA

>911_Sample_ASV26

CACCGCGGTTATACGAGAGGCCCCAGTTGATATTAGCGGCGTAAAGGGTGGTTAAGGACT

TTAATAACTAAAGCTAAACACTTCCCCGGCTGTTGTACGTAGACCGGAAGCTTGAAGCCC

AGACACGAAAGTAGCTTTATTCCCCCTGACCCCACGAAAGCTGAGAAA

>911_Sample_ASV27

CACCGCGGTCACACGATTAACCCAAGTCAATAGAAGCCGGCGTAAAGAGTGTTTTAGATC

ACCCCCTCCCCAATAAAGCTAAAACTCACCTGAGTTGTAAAAAACTCCAGTTGACACAAA

ATAGACTACGAAAGTGGCTTTAACATATCTGAACACACAATAGCTAAGACC

>911_Sample_ASV28

CACCGCGGTTATACGAGAAACCCTAGTTGACAAATACGGCGTAAAGAGTGGTTAAGGGGT

ATCAAAAATAAAGTCAAACATTTTCCTAGTTGTTATAAGCATTTCGAGAACATGAAGCCC

AACCACGAAAGTAACTTTACCTTCACCTGACCCCACGAAAGCTAAGAAA

>911_Sample_ASV29

CACCGCGGTTATACGAGAGACCCTAATTGATAGGTACGGCGTAAAGAGTGGTTTGGGATA

ACCCAAAAATAAAGCTAAAGACCTTCTAAGCCGTCACACGCATACCGAAGGCACGAGGCC

CCAACACGAAAGTAGCTTTACTATTACCCCGACGCCACGAAAGCTAAGAAA

>911_Sample_ASV30

CACCGCGGTTATACGAGAGGCCCTAGTTGATAACTACCGGCGTAAAGAGTGGTTATGGAA

AAATATTTAATAAAGCCGAACACCCCCTCAGCCGTCATACGCACCTGGGAGCACGAAGAC

CTACTGCGAAAGCAGCTTTAATTATGCCTGACCCCACGACAGCTAAGAAA

>911_Sample_ASV31

CACCGCGGTTATACGAAAGACCCTAGTTGATAGGCACGGCGTAAAGGGTGGTTAGGGATA

TATCAAAAATAAAGTTAAAGAGCCTCTAAGCTGTCGCACGCATCCCGAGAGCTCGAGACC

CAGACACGAAAGTAACTTTAAAATACCTCACCTGACCCCACGAAAGCTAAGAAA

>911_Sample_ASV32

CACCGCGGTTATACGAAAGACCCTAGTTGATAGGCACGGCGTAAAGGGTGGTTAGGGATA

TATCAAAAATAAAGTTAAAGAGCCTCTAAGCTGTCGCACGCATCCCGAGAGCTCGAGACC

CAGACACGAAAGTAACTTTAAAACATCTTTACCTGACCCCACGAAAGCTAAGAAA

>911_Sample_ASV33

CACCGCGGTTATACGAAAGACCCTAGTTGATAGCCACGGCGTAAAGGGTGGTTAAGGTAT

ACAACAAATAAAGCTAAATAGCCTCTAGGCCGTCGCACGCATTCCGAGAGCTCGAAACCC

AAAAACGAAAGTAGCTTTAAAATAAACAGACCTGACCCCACGAAAGCTAAGAAA

>911_Sample_ASV34

CACCGCGGTTATACGAGAGACCCCAGTTGATAAACACGGCGTAAAGAGTGGTTAAGGGCA

CAAAACAAATAAAGTCAAACAACCTCTTAGCTGTTATACGCATTATGAGGATATGAAGCC

CCCCTACGAAAGTAACTTTACCAACCCCTGACCCCACGAAAGCTAAGAAA

>911_Sample_ASV35

CACCGCGGTTATACGAAAGACCCTAGTTTATAGGCACGGCGTAAAGGGTGGTTAGGGGAT

AAACAAAATAAAGCCAAAGACCTTCTAAGTCGTCATACACTCATGAAGGCACGAAGCCCA

AATACGAAAGTAACTTTACAACTTAGACCCGACTCCACGAAAGCTGGGGGA

>911_Sample_ASV36

CACCGCGGTTATACGAGAGACTCCAGTTGATAGCTACGGCGTAAAGAGTGGTTTGGGGCC

CCACCCAAAATAAAGCCAAAGACCTCCCAAGCCGTCAAACGCACCCCGGAGGCACGAAGT

CCTAACGCGAAAGCAACTTTACCTCCCCCGACGCCACGAAAGCTAAGAAA

>911_Sample_ASV37

CACCGCGGTTATACGAGAGACTCCAGTTGATAGCTACGGCGTAAAGAGTGGTTTGGGGCC

CGCCCAAAATAAAGCCAAAGACCTCCCAAGCCGTCAAACGCACCCCGGAGGCACGAAGTC

CTAACGCGAAAGCAACTTTACCTTCCCCGACGCCACGAAAGCTAAGAAA

>911_Sample_ASV38

CACCGCGGTTATACGAAAGACCCTAGTTGATAGATCACGGCGTAAAGGGTGGTTAAGGAG

AACAAAAATAAAGCTAAAAATCCTCTAAGCTGTCATACGCTTTCCGAAGACATGAGACCC

AACCACGAAAGTAACTTTAAACTTTCCTGACACCACGAAAGCCAAGAAA

>914_Sample_ASV1

CACCGCGGTTATACGAGAGGCCCAAGTTGACAGACATCGGCGTAAAGAGTGGTTAAGGAA

AACCTTCAACTAAAGTCGAACGCTCTCAAGACTGTCGTACGTTTCCGAGAGTATGAAGCC

CCACCACGAAAGTGACTTTACCTCACCTGACTCCACGAAAGCTGCGAAA

>914_Sample_ASV2

CACCGCGGTTATACGAAAGACCCTAGTTGATGGGTGCGGCGTAAAGGGTGGTTAGGGACT

ACAAAAAATAGAGCTAAAGATCCTCTAGGTCGTCATACACATCACGAGGAAACGAAGCCC

AAATACGAAAGTAGCTCTAAATATAATCCTGACGCCACGAAAGCCAAGAAA

>914_Sample_ASV3

CACCGCGGTTATACGAGATGGCCCAAGTTGATAGACACCGGCGTAAAGAGTGGCTAGGGA

AAAATTACTACTAAAGCCGAACACCTTCAGAACTGTCATACGTTCCCGAAGATAAGAAGC

CCCACCACGAAAGTGACTTTATATTACCCGACCCCACGAAAGCTGTGAAA

>914_Sample_ASV4

CACCGCGGTTATACGAGAGGCCCAAGTTGACAGACACCGGCGTAAAGAGTGGCTAGGGAA

AAATTACTACTAAAGCCGAACACCTTCAGAACTGTCATACGTTCCCGAAGATAAGAAGCC

CCACCACGAAAGTGACTTTATATTACCCGACCCCACGAAAGCTGTGAAA

>914_Sample_ASV5

CACCGCGGTTATACGAGATGGCCCAAGTTGATAGACACCGGCGTAAAGAGTGGCTAGGGA

AAAATTACTACTAAAGCCGAACACCTTCAGAACTGTCATACGTTCCCGAAGATAAGAAGC

CCCACCACGAAAGTGACTTTATATTACCCGACCCCACGAAAGCTGTGATA

>914_Sample_ASV6

CACCGCGGTTATACGAGATGGCCCAAGTTGATAGACACCGGCGTAAAGGGTGGTTAGGGA

AAAATTACTACTAAAGCCGAACACCTTCAGAACTGTCATACGTTCCCGAAGATAAGAAGC

CCCACCACGAAAGTGACTTTATATTACCCGACCCCACGAAAGCTGTGATA

>914_Sample_ASV7

CACCGCGGTTATACGAGAGGCCCAAGTTGACAGACACCGGCGTAAAGAGTGGCTAGGGAA

AAATTACTACTAAAGCCGAACACCTTCAGAACTGTCATACGTTCCCGAAGATAAGAAGCC

CCACCACGAAAGTGACTTTATATTACCCGACCCCACGAAAGCTGTGATA

>914_Sample_ASV8

CACCGCGGTTATACGAGATGGCCCAAGTTGATAGACACCGGCGTAAAGAGTGGCTAGGGA

AAAATTACTACTAAAGCCGAACACCCTCAGAACTGTCATACGTTCCCGAAGGTAAGAAGC

CCCACCACGAAAGTGGCTTTATAATACCCGACCCCACGAAAGCTGTGAAA

>914_Sample_ASV9

CACCGCGGTTATACGAGAGGCCCAAGTTGACAGACACCGGCGTAAAGAGTGGCTAGGGAA

AAATTACTACTAAAGCCGAACACCCTCAGAACTGTCATACGTTCCCGAAGGTAAGAAGCC

CCACCACGAAAGTGGCTTTATAATACCCGACCCCACGAAAGCTGTGATA

>914_Sample_ASV10

CACCGCGGTTATACGAGAGGCCCAAGTTGACAGACACCGGCGTAAAGAGTGGCTAGGGAA

AAATTACTACTAAAGCCGAACACCCTCAGAACTGTCATACGTTCCCGAAGGTAAGAAGCC

CCACCACGAAAGTGGCTTTATAATACCCGACCCCACGAAAGCTGTGAAA

>914_Sample_ASV11

CACCGCGGTTATACGAGAGGCCCAAGTTGACAGACACCGGCGTAAAGAGTGGCTAGGGAA

AAATTACTACTAAAGCCGAACACCTTCAGAACTGTCATACGTTCCCGAAGATAAGAAGCC

CCACCACGAAAGTGGCTTTATATTACCCGACCCCACGAAAGCTGTGAAA

>914_Sample_ASV12

CACCGCGGTTATACGAGAGGCCCAAGTTGACAGACACCGGCGTAAAGAGTGGCTAAGGAA

AAATTTCTACTAAAGCCGAACACCTTCAGAACTGTCATACGTTCCCGAAGATAAGAAGCC

CCACCACGAAAGTGGCTTTATATTACCCGACACCACGAAAGCTGTGAAA

>914_Sample_ASV13

CACCGCGGTTATACGAGAGGCCCAAGTTGACAGACACCGGCGTAAAGAGTGGCTAAGGAA

AAATTTCTACTAAAGCCGAACACCTTCAGAACTGTCATACGTTCCCGAAGATAAGAAGCC

CCACCACGAAAGTGGCTTTATATTACCCGACACCACGAAAGCTGTGATA

>914_Sample_ASV14

CACCGCGGTTATACGAGAGGCCCAAGTTGACAGACACCGGCGTAAAGAGTGGCTAAGGAA

AAATTTCTACTAAAGCCGAACACCTTCAGAACTGTCATACGTTCCCGAAGAAAAGAAGCC

CCACCACGAAAGTGGCTTTATATTACCCGACCCCACGAAAGCTGTGATA

>914_Sample_ASV15

CACCGCGGTTATACGAGAGGCCCAAGTTGACAGACACCGGCGTAAAGAGTGGCTAAGGAA

AAATTTCTACTAAAGCCGAACACCTTCAGAACTGTCATACGTTCCCGAAGAAAAGAAGCC

CCACCACGAAAGTGGCTTTATATTACCCGACCCCACGAAAGCTGTGAAA

>914_Sample_ASV16

CACCGCGGTTATACGAAAGACCCTAGTTTATAGAAACGGCGTAAAGGGTGGTTAAGGGAC

AAACAAAATAAGGCCAAAGACTTCCTAAGCCGTCATACGCTCCACGAAAGCACGAAGCCC

AAACACGAAAGTAGCCTTAATAACTTCACCTGACCCCACGAAAGCTGGGAGA

>914_Sample_ASV17

CACCGCGGTTATACGAGAGACCCTAGTTGATATATACGGCGTAAAGAGTGGTTTGGGATA

TTTCTCCAAAATAAAGTCGAAGATCTCCCAAGCCGTCACACGCATCCCGGAGACACGAAG

CCCTGATACGAAGGTAGCTTTATTAACACCCCGACGCCACGAAAGCTAAGAAA

>914_Sample_ASV18

CACCGCGGTTATACGAGAGGCCCAAGTTGATAAGCCCGGCGTAAAGAGTGGTTATGGAGA

ATAAAACACTAAAGCTAAAGACCCCCTAGGCTGTTATACGCACCTGGCGGCTCGAACCAC

CTATACGAAAGTAGCTTTACCCCCCTTCCACCAGAATCCACGACAGCTGGGGCA

>914_Sample_ASV19

CACCGCGGTTATACGAGAGGCCCAAGTTGACAGACATCGGCGTAAAGAGTGGTTAAGGAA

ACCCCATTACTAAAGCCGAACGCCCTCAGAACTGTCATACGTTCCCGAAGGTAAGAAGCC

CCACTACGAAAGTGGCTTTAACCACACCCGACCCCACGAAAGCTGTGAAA

>914_Sample_ASV20

CACCGCGGTTATACGAGAGGCCCAAGTTGACAGACACCGGCGTAAAGAGTGGTTAAGGAA

ATTCATCAACTAAAGCCGAATGCCCTCAGGACTGTTATACGTTTGCGAAGGTAAGAAGCC

CTACTACGAAAGTGGCTTTATTCCACCTGATTCCACGAAAGCTGCGGAA

>914_Sample_ASV21

CACCGCGGTTATACGAGAGGCCCCAGTTGATATTAGCGGCGTAAAGGGTGGTTAAGGACT

TTAATAACTAAAGCTAAACACTTCCCCGGCTGTTGTACGTAGACCGGAAGCTTGAAGCCC

AGACACGAAAGTAGCTTTATTCCCCCTGACCCCACGAAAGCTGAGAAA

>914_Sample_ASV22

CACCGCGGTTATACGAGAAACCCTAGTTGACAAATACGGCGTAAAGAGTGGTTAAGGGGT

ATCAAAAATAAAGTCAAACATTTTCCTAGTTGTTATAAGCATTTCGAGAACATGAAGCCC

AACCACGAAAGTAACTTTACCTTCACCTGACCCCACGAAAGCTAAGAAA

>914_Sample_ASV23

CACCGCGGTTATACGAAAGACCCCAGTTTATAGACACGGCGTAAAGGGTGGTTATAGGAC

AAACTAACATAGAGCCAAAGACCCCCTAAGCCGTCATACGCTCACGAGGGCTCGAAGCCC

CTATACGAAAGTAGCTCTACCAAAAAATGCTCGACTCCACGAAAGCTGGGACA

>914_Sample_ASV24

CACCGCGGTTATACGAGAGACCCTAATTGATAGGTACGGCGTAAAGAGTGGTTTGGGATA

ACCCAAAAATAAAGCTAAAGACCTTCTAAGCCGTCACACGCATACCGAAGGCACGAGGCC

CCAACACGAAAGTAGCTTTACTATTACCCCGACGCCACGAAAGCTAAGAAA

>914_Sample_ASV25

CACCGCGGTTATACGAGAGACCCTAGTTGATAGCTACGGCGTAAAGCGTGGTTAGGAGAA

CAAGATAAATAAAGTCAAACAATCTCTCGGCCGTTATACGTTATCTGAGAATATGAAGTC

CTACCACGAAAGTAACTTTAATTTTTCTGACCCCACGAAAACTGAGGAA

>914_Sample_ASV26

CACCGCGGTTATACGAGAGGCCCTAGTTGATAACTACCGGCGTAAAGAGTGGTTATGGAA

AAATATTTAATAAAGCCGAACACCCCCTCAGCCGTCATACGCACCTGGGAGCACGAAGAC

CTACTGCGAAAGCAGCTTTAATTATGCCTGACCCCACGACAGCTAAGAAA

>914_Sample_ASV27

CACCGCGGTTATACGAGAGACCCCAGTTGATAAACACGGCGTAAAGAGTGGTTAAGGGCA

CAAAACAAATAAAGTCAAACAACCTCTTAGCTGTTATACGCATTATGAGGATATGAAGCC

CCCCTACGAAAGTAACTTTACCAACCCCTGACCCCACGAAAGCTAAGAAA

>914_Sample_ASV28

CACCGCGGTTATACGAGAGACTCCAGTTGATAGCTACGGCGTAAAGAGTGGTTTGGGGCC

CGCCCAAAATAAAGCCAAAGACCTCCCAAGCCGTCAAACGCACCCCGGAGGCACGAAGTC

CTAACGCGAAAGCAACTTTACCTTCCCCGACGCCACGAAAGCTAAGAAA

>914_Sample_ASV29

CACCGCGGTTATACGAAAGACCCTAGTTGATAGATCACGGCGTAAAGGGTGGTTAAGGAG

AACAAAAATAAAGCTAAAAATCCTCTAAGCTGTCATACGCTTTCCGAAGACATGAGACCC

AACCACGAAAGTAACTTTAAACTTTCCTGACACCACGAAAGCCAAGAAA

>915_Sample_ASV1

CACCGCGGTTATACGAGAGGCCCAAGTTGACAGACATCGGCGTAAAGAGTGGTTAAGGAA

AACCTTCAACTAAAGTCGAACGCTCTCAAGACTGTCGTACGTTTCCGAGAGTATGAAGCC

CCACCACGAAAGTGACTTTACCTCACCTGACTCCACGAAAGCTGCGAAA

>915_Sample_ASV2

CACCGCGGTTATACGAAAGACCCTAGTTGATGGGTGCGGCGTAAAGGGTGGTTAGGGACT

ACAAAAAATAGAGCTAAAGATCCTCTAGGTCGTCATACACATCACGAGGAAACGAAGCCC

AAATACGAAAGTAGCTCTAAATATAATCCTGACGCCACGAAAGCCAAGAAA

>915_Sample_ASV3

CACCGCGGTTATACGAAAGACCCTAGTTGATGGGTGCGGCGTAAAGGGTGGTTAGGGACT

ACAAAAAATAGAGCTAAAGATCCTCTAGGTCGTCATACACATCACGAGGAAACGAAGCCC

AAGTACGAAAGTAGCTCTAAATATAATCCTGACGCCACGAAAGCCAAGAAA

>915_Sample_ASV4

CACCGCGGTTATACGAGAGACCCTAGTTGATTAAAGCGGCGTAAAGAGTGGTTATGGAAC

TATTCTTTTAAAGCAGAAAACCTCTCAAACTGTTATACGCACCCAGAGGTCTGAATCCCT

CACACGAAAGTGACTTTATTTATGCCTACCAGAACCCACGAAAGCTGGGACA

>915_Sample_ASV5

CACCGCGGTTATACGAGAGACCCTAGTTGATTAAAGCGGCGTAAAGAGTGGTTATGGAAC

TATTCTTTTAAAGCAGAAAACCTCTCAAACTGTTATACGCACCCAGAGGTCTGAATCCCT

CACACGAAAGTGACTTTATTTATGCCTACCAGAACCCACGAAAGCTATGACA

>915_Sample_ASV6

CACCGCGGTTATACGAAAGACCCTAGTTTATAGGTACGGCGTAAAGGGTGGTTAGGGGAC

AAACAAAATAAAGCCAAAGACCTCCTAAGCCGTCATACGCTCACGGAAGCACGAAGCCCA

AACACGAAAGTAGCTTTACCGAACATGCCCGACTCCACGAAAGCTGGGGAA

>915_Sample_ASV7

CACCGCGGTTATACGAGAGGCCCAAGTTGACAGACACCGGCGTAAAGAGTGGCTAGGGAA

AAATTACTACTAAAGCCGAATACCTTCAGAACTGTCATACGTTCCCGAAGATAAGAAGCC

CCACCACGAAAGTGGCTTTATATTACCCGACCCCACGAAAGCTGTGAAA

>915_Sample_ASV8

CACCGCGGTTATACGAGAGGCCCAAGTTGACAGACACCGGCGTAAGGAGTGGCTAGGGAA

AAATTACTACTAAAGCCGAACACCTTCAGAACTGTCATACGTTCCCGAAGATAAGAAGCC

CCACCACGAAAGTGGCTTTATATTACCCGACCCCACGAAAGCTGTGAAA

>915_Sample_ASV9

CACCGCGGTTATACGAGAGGCCCAAGTTGACAGACACCGGCGTAAAGAGTGGCTAGGGAA

AAATTACTACTAAAGCCGAACACCTTCAGAACTGTCATACGTTCCCGAAGATAAGAAGCC

CCACCACGAAAGTGGCTTTATATTACCCGACCCCACGAAAGCTGTGAAA

>915_Sample_ASV10

CACCGCGGTTATACGAGAGGCCCAAGTTGACAGACACCGGCGTAAAGAGTGGCTAAGGAA

AAATTTCTACTAAAGCCGAACACCTTCAGAACTGTCATACGTTCCCGAAGAAAAGAAGCC

CCACCACGAAAGTGGCTTTATATTACCAGACCCCACGAAAGCTGTGAAA

>915_Sample_ASV11

CACCGCGGTTATACGAAAGACCCTAGTTTATAGAAACGGCGTAAAGGGTGGTTAAGGGAC

AAACAAAATAAGGCCAAAGACTTCCTAAGCCGTCATACGCTCCACGAAAGCACGAAGCCC

AAACACGAAAGTAGCCTTAATAACTTCACCTGACCCCACGAAAGCTGGGAGA

>915_Sample_ASV12

CACCGCGGTTAAACGAGAGACCCTAGTTGATCATCACGGCGTAAAGAGTGGTTAGGGGAT

TATCATAAATAAAGCCAAAGACCTCCCAAGCTGTCGCACGCATTCCGGAGGCGCGAAGCC

CACACACGAAAGTAGCTTTAACTATTGACCCTGATGCCACGAAAGCTAAGAAA

>915_Sample_ASV13

CACCGCGGTTATACGAGAGGCCCAAGTTGATAAGCCCGGCGTAAAGAGTGGTTATGGAGA

ATAAAACACTAAAGCTAAAGACCCCCTAGGCTGTTATACGCACCTGGCGGCTCGAACCAC

CTATACGAAAGTAGCTTTACCCCCCTTCCACCAGAATCCACGACAGCTGGGGCA

>915_Sample_ASV14

CACCGCGGTTATACGAGAGGCCCAAGTTGACAGACATCGGCGTAAAGAGTGGTTAAGGAA

ACCCCATTACTAAAGCCGAACGCCCTCAGAACTGTCATACGTTCCCGAAGGTAAGAAGCC

CCACTACGAAAGTGGCTTTAACCACACCCGACCCCACGAAAGCTAAGAAA

>915_Sample_ASV15

CACCGCGGTTATACGAGAGGCCCAAGTTGACAGACATCGGCGTAAAGAGTGGTTAAGGAA

ACCCCATTACTAAAGCCGAACGCCCTCAGAACTGTCATACGTTCCCGAAGGTAAGAAGCC

CCACTACGAAAGTGGCTTTAACCACACCCGACCCCACGAAAGCTGTGAAA

>915_Sample_ASV16

CACCGCGGTTATACGAGAGGCCCAAGTTGATAGATATCGGCGTAAAGAGTGGTTAAGGAA

ACCCCGTTACTAAAGCCGAACGCCCTCAGAACTGTCATACGTTCCCGAAGGTAAGAAGCC

CCACTACGAAAGTGGCTTTAACCACACCCGACCCCACGAAAGCTGTGAAA

>915_Sample_ASV17

CACCGCGGTTATACGAGAGGCCCAAGTTGACAGACACCGGCGTAAAGAGTGGTTAAGGAA

ATTCATCAACTAAAGCCGAATGCCCTCAGGACTGTTATACGTTTGCGAAGGTAAGAAGCC

CTACTACGAAAGTGGCTTTATTCCACCTGATTCCACGAAAGCTGCGGAA

>915_Sample_ASV18

CACCGCGGTCACACGATTAACCCAAGTCAATAGAAGCCGGCGTAAAGAGTGTTTTAGATC

ACCCCCTCCCCAATAAAGCTAAAACTCACCTGAGTTGTAAAAAACTCCAGTTGACACAAA

ATAGACTACGAAAGTGGCTTTAACATATCTGAACACACAATAGCTAAGACC

>915_Sample_ASV19

CACCGCGGTCACACGATTAACCCAAGTCAATAGAAGCCGGCGTAAAGAGTGTTTTAGATC

ACCCCCTCCCCAATAAAGCTAAAACTCACCTGAGTTGTAAAAAACTCTAGTTGACACAAA

ATAGACTACGAAAGTGGCTTTAACATATCTGAACACACAATAGCTAAGACC

>915_Sample_ASV20

CACCGCGGTTATACGAGAGGCCCTAGTTGATAAATACGGCGTAAAGAGTGGTTAGAGGAC

TCCCATAATAAAGCCAAAGACCCCCCGGGCCGTCACACGCATACGAGGGCACGAAGTTCA

CACACGAAAGTAGCTTTAAATTACCCGACGCCACGAAAGCTAAGAAA

>915_Sample_ASV21

GACCGCGGTTATACGAGAGACTCTAGTTGATAGATACGGCGTAAAGAGTGGTTTGGGATA

AACTATAAATAAAGCCAAAGACCTTCCAAGCTGTTAAACGCATACCGAAGGCACGAAGCC

CCAACACGAAAGTAGCTTTACTACTACCCCCGATGCCACGAAAGCTAAGACA

>915_Sample_ASV22

CACCGCGGTTATACGAGAGACCCTAGTTGATAAATACGGCGTAAAGAGTGGTTATGGGAA

AAACAAATAAAGTCAAACAACCTCTTAGCTGTTATACGCATTATGAGAGCATGAAGCCCC

CTCACGAAAGTAACTTTAATACCTCCTGACCCCACGAAAGCTAAGAAA

>915_Sample_ASV23

CACCGCGGTCATACGATTAACCCAAGTTAATAGGCACACGGCGTAAAGCGTGTTTAAGCA

CTATGCCAAATAAAGTTAAACTCCAATTAAGCTGTAAAAAGCCATAATCAAGACGAAAAT

AAATAACGAAAGTAACTTTACAACCGCTGAAACACGATAGCTAAGATC

>915_Sample_ASV24

CACCGCGGTTATACGAAAGACCCTAGTTTATAGACACGGCGTAAAGGGTGGTTCGGGACT

AAACAAAATAAAGCCAAAGACCTCCTAAGCCGTCATACGCTCACGGAAGCACGAAGCCCA

AGTACGAAAGTAGCTTTACAAAACATGCCTGACTCCACGAAAGCTAGGAAA

>915_Sample_ASV25

CACCGCGGTTATACGAAAGACCCTAGTTGATAGGCACGGCGTAAAGGGTGGTTAGGGATA

TATCAAAAATAAAGTTAAAGAGCCTCTAAGCTGTCGCACGCATCCCGAGAGCTCGAGACC

CAGACACGAAAGTAACTTTAAAATACCTCACCTGACCCCACGAAAGCTAAGAAA

>915_Sample_ASV26

CACCGCGGTTATACGAAAGACCCTAGTTGATAGCCACGGCGTAAAGGGTGGTTAAGGTAT

ACAACAAATAAAGCTAAATAGCCTCTAGGCCGTCGCACGCATTCCGAGAGCTCGAAACCC

AAAAACGAAAGTAGCTTTAAAATAAACAGACCTGACCCCACGAAAGCTAAGAAA

>915_Sample_ASV27

CACCGCGGTTATACGAGAGGCCCAAGTTGATACTCCACGGCGTAAAGAGTGGTTAGAAAA

GACCTACTACTAAAGCCGAACGCCTTCAAAGCTGTTATACGCATCCGAAGGTGAGAAGCC

CACCCACGAAAGTGGCTTTACAACCTTGACTCCACGAAAGCTATGACA

>915_Sample_ASV28

CACCGCGGTTATACGAGAGGCCCAAGTTGATACTCCACGGCGTAAAGAGTGGTTAGAAAA

GACCTACTACTAAAGCCGAACGCCTTCAAAGCTGTTATACGCATCCGAAGGTGAGAAGCC

CACCCACGAAAGTGGCTTTACAACCTTGACTCCACGAAAGCTGGGACA

>915_Sample_ASV29

CACCGCGGTTATACGAGAGACCCCAGTTGATAAACACGGCGTAAAGAGTGGTTAAGGGCA

CAAAACAAATAAAGTCAAACAACCTCTTAGCTGTTATACGCATTATGAGGATATGAAGCC

CCCCTACGAAAGTAACTTTACCAACCCCTGACCCCACGAAAGCTAAGAAA

>915_Sample_ASV30

CACCGCGGTTATACGAAAGACCCTAGTTTATAGGCACGGCGTAAAGGGTGGTTAGGGGAT

AAACAAAATAAAGCCAAAGACCTTCTAAGTCGTCATACACTCATGAAGGCACGAAGCCCA

AATACGAAAGTAACTTTACAACTTAGACCCGACTCCACGAAAGCTGGGGGA

>915_Sample_ASV31

CACCGCGGTTATACGAAAGACCCTAGTTGATAGATCACGGCGTAAAGGGTGGTTAAGGAG

AACAAAAATAAAGCTAAAAATCTTCTAAGCTGTCATACGCTTTCCGAAGACATGAGACCC

AACCACGAAAGTAGCTTTAAACTTTCCTGACACCACGAAAGCCAAGAAA

>917_Sample_ASV1

CACCGCGGTTATACGAGAGGCCCAAGTTGACAGACATCGGCGTAAAGAGTGGTTAAGGAA

AACCTTCAACTAAAGTCGAACGCTCTCAAGACTGTCGTACGTTTCCGAGAGTATGAAGCC

CCACCACGAAAGTGACTTTACCTCACCTGACTCCACGAAAGCTGCGAAA

>917_Sample_ASV2

CACCGCGGTTATACGAAAGACCCTAGTTGATGGGTGCGGCGTAAAGGGTGGTTAGGGACT

ACAAAAAATAGAGCTAAAGATCCTCTAGGTCGTCATACACATCACGAGGAAACGAAGCCC

AAATACGAAAGTAGCTCTAAATATAATCCTGACGCCACGAAAGCCAAGAAA

>917_2_Sample_ASV1

CACCGCGGTTATACGAAAGACCCTAGTTGATGGGTGCGGCGTAAAGGGTGGTTAGGGACT

ACAAAAAATAGAGCTAAAGATCCTCTAGGTCGTCATACACATCACGAGGAAACGAAGCCC

AAATACGAAAGTAGCTCTAAATATAATCCTGACGCCACGAAAGCCAAGAAA

>917_Sample_ASV3

CACCGCGGTTATACGAAAGACCCTAGTTGATAACTACGGCATAAAGGGTGGTTAGGGAGT

TTAAAAAATAAAGCCAAAGCTCTTCTAAGCTGTCATACGCTCTCTGAAAGAACGAGGCCC

AAACACGAAAGTAGCTTTAAATAAATAACCTGATACCACGAAAGCCAAGAAA

>917_2_Sample_ASV2

CACCGCGGTTATACGAGAGACCCTAGTTGATTAAAGCGGCGTAAAGAGTGGTTATGGAAC

TATTCTTTTAAAGCAGAAAACCTCTCAAACTGTTATACGCACCCAGAGGTCTGAATCCCT

CACACGAAAGTGACTTTATTTATGCCTACCAGAACCCACGAAAGCTGGGACA

>917_2_Sample_ASV3

CACCGCGGTTATACGAGAGACCCTAGTTGATATACACGGCGTAAAGAGTGGTTTGGGGTA

TATAATAAATAAAGCCAAAGACCTTCTAGGCCGTTGAACGCACCACGAAGGAGCGAAGTC

CCAATACGAAAGTAGCTTTATAAAACCTGACGCCACGAAAGCTAAGAAA

>917_2_Sample_ASV4

CACCGCGGTTATACGAGAGGCCCAAGTTGACAGACACCGGCGTAAAGAGTGGCTAGGGAA

AAATTACTACTAAAGCCGAACACCTTCAGAACTGTCATACGTTCCCGAAGATAAGAAGCC

CCACCACGAAAGTGACTTTATATTACCCGACCCCACGAAAGCTGTGAAA

>917_Sample_ASV4

CACCGCGGTTATACGAGAGGCCCAAGTTGACAGACACCGGCGTAAAGAGTGGCTAGGGAA

AAATTACTACTAAAGCCGAACACCTTCAGAACTGTCATACGTTCCCGAAGATAAGAAGCC

CCACCACGAAAGTGGCTTTATATTACCCGACCCCACGAAAGCTAAGAAA

>917_Sample_ASV5

CACCGCGGTTATACGAGAGGCCCAAGTTGACAGACACCGGCGTAAAGAGTGGCTAGGGAA

AAATTACTACTAAAGCCGAACACCTTCAGAACTGTCATACGTTCCCGAAGATAAGAAGCC

CCACCACGAAAGTGGCTTTATATTACCCGACCCCACGAAAGCTGTGAAA

>917_2_Sample_ASV5

CACCGCGGTTATACGAGAGGCCCAAGTTGACAGACACCGGCGTAAAGAGTGGCTAGGGAA

AGATTACTACTAAAGCCGAACACCTTCAGAACTGTCATACGTTCCCGAAGATAAGACGCC

CCACCACGAAAGTGGCTTTATATTACCCGACCCCACGAAAGCTGTGAAA

>917_2_Sample_ASV6

CACCGCGGTTATACGAGAGGCCCAAGTTGACAGACACCGGCGTAAAGAGTGGCTAGGGAA

AAATTACTACTAAAGCCGAACACCTTCAGAACTGTCATACGTTCCCGAAGATAAGAAGCC

CCACCACGAAAGTGGCTTTATATTACCCGACCCCACGAAAGCTGTGAAA

>917_2_Sample_ASV7

CACCGCGGTTATACGAGAGGCCCAAGTTGACAGACACCGGCGTAAAGAGTGGCTAGGGAA

AAATTACTACTAAAGCCGAACACCTTCAGAACTGTCATACGTTCCCGAAGATAAGAAGCC

CCACCACGAAAGTGGCTTTATATTACCCGACCCCACGAAAGCTGAAAAA

>917_2_Sample_ASV8

CACCGCGGTTATACGAGAGGCCCAAGTTGACAGACACCGGCGTAAAGAGTGGCTAGGGAA

AAATTACTACTAAAGCCGAACACCTTCAGAACTGTCATACGTTCCCGAAGATAAGAAGCC

CCACCACGAAAGTGGCTTTATATTACCCGACCCCACGAAAGCCGAAAAA

>917_2_Sample_ASV9

CACCGCGGTTATACGAGAGGCCCAAGTTGACAGACACCGGCGTAAAGAGTGGCTAGGGGA

AAATTACTACTAAAGCCGAACACCTTCAGAACTGTCATACGTTCCCGAAGATACGAAGCC

CCACCACGAAAGTGGCTTTATATTACCCGACCCCACGAAAGCTGTGAAA

>917_Sample_ASV6

CACCGCGGTTATACGAAAGACCCTAGTTTATAGAAACGGCGTAAAGGGTGGTTAAGGGAC

AAACAAAATAAGGCCAAAGACTTCCTAAGCCGTCATACGCTCCACGAAAGCACGAAGCCC

AAACACGAAAGTAGCCTTAATAACTTCACCTGACCCCACGAAAGCTGGGAGA

>917_Sample_ASV7

CACCGCGGTTATACGAGAGACCCTAGTTGATATATACGGCGTAAAGAGTGGTTTGGGATA

TTTCTCCAAAATAAAGTCGAAGATCTCCCAAGCCGTCACACGCATCCCGGAGACACGAAG

CCCTGATACGAAGGTAGCTTTATTAACACCCCGACGCCACGAAAGCTAAGAAA

>917_2_Sample_ASV10

CACCGCGGTTATACGAGAGACCCTAGTTGATATATACGGCGTAAAGAGTGGTTTGGGATA

TTTCTCCAAAATAAAGCCGAAGATCTCCCAAGCCGTCACACGCATCCCGGAGACACGAAG

CCCTAATACGAAGGTAGCTTTATTAATTCCCCGACGCCACGAAAGCTAAGAAA

>917_Sample_ASV8

CACCGCGGTTATACGAGAGACCCTAGTTGATATATACGGCGTAAAGAGTGGTTTGGGATA

TTTCTCCAAAATAAAGCCGAAGATCTCCCAAGCCGTCACACGCATCCCGGAGACACGAAG

CCCTGATACGAAGGTAGCTTTATTAACACCCCGACGCCACGAAAGCTAAGAAA

>917_2_Sample_ASV11

CACCGCGGTTATACGAGAGACCCTAGTTGATATATACGGCGTAAAGAGTGGTTTGGGGTT

ATGCCTCCAAAATAAAGCCGAAGATCTCCCAAGCCGTCACACGCATCCCGGAGACACGAA

GCCCTAATACGAAGGTAGCTTTATTAGTACCCCGACGCCACGAAAGCTAAGAAA

>917_2_Sample_ASV12

CACCGCGGTTATACGAGAGACCCTAGTTGATATATACGGCGTAAAGAGTGGTTTGGGGTT

ATGCCTCCAAAATAAAGCCGAAGATCTCCCAAGCCGTCACACGCATCCCGGAGACACGAA

GCCCTAATACGAAGGTAGCTTTATTAATTCCCCGACGCCACGAAAGCTAAGAAA

>917_2_Sample_ASV13

CACCGCGGTTATACGAGAGACCCTAGTTGATATATACGGCGTAAAGAGTGGTTTGGGGTT

ATGCCTCCAAAATAAAGTCGAAGATCTCCCAAGCCGTCACACGCACCCCGGAGACACGAA

GCCCTAATACGAAGGTAGCTTTATTAATTCCCCGACGCCACGAAAGCTAAGAAA

>917_2_Sample_ASV14

CACCGCGGTTATACGAGAGACCCTAGTTGATATATACGGCGTAAAGAGTGGTTTGGGGTT

ATGCCTCCAAAATAAAGCCGAAGATCTCCCAAGCCGTCACACGCACCCCGGAGACACGAA

GCCCTAATACGAAGGTAGCTTTATTAATTCCCCGACGCCACGAAAGCTAAGAAA

>917_2_Sample_ASV15

CACCGCGGTTATACGAGAGACCCTAGTTGATATATACGGCGTAAAGAGTGGTTTGGGATA

TTTCTCCAAAATAAAGCCGAAGATCTCCCAAGCCGTCACACGCATCCCGGAGACACGAAG

CCCTAATACGAAGGTAGCTTTATTAGTACCCCGACGCCACGAAAGCTAAGAAA

>917_2_Sample_ASV16

CACCGCGGTTATACGAGAGGCCCAAGTTGATAAGCCCGGCGTAAAGAGTGGTTATGGAGA

ATAAAACACTAAAGCTAAAGACCCCCTAGGCTGTTATACGCACCTGGCGGCTCGAACCAC

CTATACGAAAGTAGCTTTACCCCCCTTCCACCAGAATCCACGACAGCTGGGGCA

>917_Sample_ASV9

CACCGCGGTTATACGAGAGGCCCAAGTTGATAAGCCCGGCGTAAAGAGTGGTTATGGAGA

ATAAAACACTAAAGCTAAAGACCCCCTAGGCTGTTATACGCACCTGGCGGCTCGAACCAC

CTATACGAAAGTAGCTTTACCCCCCTTCCACCAGAATCCACGACAGCTGGGGCA

>917_2_Sample_ASV17

CACCGCGGTTATACGAGAGGCCCAAGTTGACAGACATCGGCGTAAAGAGTGGTTAAGGAA

ACCCCATTACTAAAGCCGAACGCCCTCAGAACTGTCATACGTTCCCGAAGGTAAGAAGCC

CCACTACGAAAGTGGCTTTAACCACACCCGACCCCACGAAAGCTGTGAAA

>917_2_Sample_ASV18

CGCCGCGGTTAGACGATGGACCCAAGTTGACAGACATCGGCGTAAAGAGTGGTTAAGGAA

ACCCCATTACTAAAGCCGAACGCCCTCAGAACTGTCATACGTTCCCGAAGGTAAGAAGCC

CCACTAGGAAAGTGGCTTTAACCACACCCGACCCCACGAAAGCTGTGAAA

>917_2_Sample_ASV19

CACCGCGGTTATACGAGAGGCCCAAGTTGACAGACATCGGCGTAAAGAGTGGTTAAGGAA

ACCCCATTACTAAAGCCGAACGCCCTCAGAACTGTCATACGTTCCCGAAGGTAAGAAGCC

CCACTACGAAAGTGGCTTTAACCACACCCGACCCCACGAAAGCTAAGAAA

>917_Sample_ASV10

CACCGCGGTTATACGAGAGGCCCAAGTTGACAGACATCGGCGTAAAGAGTGGTTAAGGAA

ACCCCATTACTAAAGCCGAACGCCCTCAGAACTGTCATACGTTCCCGAAGGTAAGAAGCC

CCACTACGAAAGTGGCTTTAACCACACCCGACCCCACGAAAGCTAAGAAA

>917_Sample_ASV11

CACCGCGGTTATACGAGAGGCCCAAGTTGACAGACATCGGCGTAAAGAGTGGTTAAGGAA

ACCCCATTACTAAAGCCGAACGCCCTCAGAACTGTCATACGTTCCCGAAGGTAAGAAGCC

CCACTACGAAAGTGGCTTTAACCACACCCGACCCCACGAAAGCTGTGAAA

>917_2_Sample_ASV20

CACCGCGGTTATACGAGAGGCCCAAGTTGACAGACATCGGCGTAAAGAGTGGTTAAGGAA

ACCCCATTACTAAAGCCGAACGCCCTCAGAACTGTCATACGTTCCCGAAGGTAAGAAGCC

CCACTACGAAAGTGGCTTTACCCCCTCCTGACCCCACGAAAGCTAAGAAA

>917_2_Sample_ASV21

CACCGCGGTTATACGAGAGGCCCAAGTTGATAGATATCGGCGTAAAGAGTGGTTAAGGAA

ACCCCGTTACTAAAGCCGAACGCCCTCAGAACTGTCATACGTCCCCGAAGGTGAGAAGCC

CCACTACGAAAGTGGCTTTAACCACACCCGACCCCACGAAAGCTGTGAAA

>917_2_Sample_ASV22

CGCCGCGGTTATACGAGAGGCCCAAGTTGATAGATATCGGCGTAAAGAGTGGTTAAGGAA

ACCCCGTTACTAAAGCCGAACGCCCTCAGAACTGTCATACGTTCCCGAAGGTAAGAAGCC

CCACTACGAAAGTGGCTTTAACCACACCCGACCCCACGAAAGCTGTGAAA

>917_2_Sample_ASV23

CACCGCGGTTATACGAGAGGCCCAAGTTGATAGATATCGGCGTAAAGAGTGGTTAAGGAA

ACCCCGTTACTAAAGCCGAACGCCCTCAGAACTGTCATACGTTCCCGAAGGTAAGAAGCC

CCACTACGAAAGTGGCTTTAACCACACCCGACCCCACGAAAACTGTGAAA

>917_2_Sample_ASV24

CACCGCGGTTATACGAGAGGCCCAAGTTGATAGATATCGGCGTAAAGAGTGGTTAAGGAA

ACCCCGTTACTAAAGCCGAACGCCCTCAGAACTGTCATACGTTCCCGAAGGTAAGAAGCC

CCACTACGAAAGTGGCTTTAACCACACCCGACCCCACGAAAGCTGTGAAA

>917_2_Sample_ASV25

CACCGCGGTTATACGAGAGGCCCAAGTTGATAGATATCGGCGTAAAGAGTGGTTAAGGAA

ACCCCGTTACTAAAGCCGAGCGCCCTCAGAACTGTCATACGTTCCCGAAGGTAAGAAGCC

CCACTACGAAAGTGGCTTTAACCACACCCGACCCCACGAAAGCTGTGAAA

>917_2_Sample_ASV26

CACCGCGGTTATACGAGAGGCCCAAGTTGACAGACATCGGCGTAAAGAGTGGTTAAGGAA

ACCCCATTACTAAAGCCGAACGCCCTCAGAACTGTCATACGTTCCCGAAGGTAAGAAGCC

CCACTACGAAAGTGGCTTTACTCCACCTGACTCCACGAAAGCTGTGAAA

>917_Sample_ASV12

CACCGCGGTTATACGAGAGGCCCAAGTTGACAGACACCGGCGTAAAGAGTGGTTAAGGAA

ATTCATCAACTAAAGCCGAATGCCCTCAGGACTGTTATACGTTTGCGAAGGTAAGAAGCC

CTACTACGAAAGTGGCTTTATTCCACCTGATTCCACGAAAGCTGCGGAA

>917_Sample_ASV13

CACCGCGGTTATACGAGAGGCCCCAGTTGATATTAGCGGCGTAAAGGGTGGTTAAGGACT

TTAATAACTAAAGCTAAACACTTCCCCGGCTGTTGTACGTAGACCGGAAGCTTGAAGCCC

AGACACGAAAGTAGCTTTATTCCCCCTGACCCCACGAAAGCTGAGAAA

>917_2_Sample_ASV27

CACCGCGGTTATACGAGAGGCCCCAGTTGATATTAGCGGCGTAAAGGGTGGTTAAGGACT

TTAATAACTAAAGCTAAACACTTCCCCGGCTGTTGTACGTAGACCGGAAGCTTGAAGCCC

AGACACGAAAGTAGCTTTATTCCCCCTGACCCCACGAAAGCTGAGAAA

>917_Sample_ASV14

CACCGCGGTCACACGATTAACCCAAGTCAATAGAAGCCGGCGTAAAGAGTGTTTTAGATC

ACCCCCTCCCCAATAAAGCTAAAACTCACCTGAGTTGTAAAAAACTCCAGTTGACACAAA

ATAGACTACGAAAGTGGCTTTAACATATCTGAACACACAATAGCTAAGACC

>917_Sample_ASV15

CACCGCGGTTATACGAGAAACCCTAGTTGACAAATACGGCGTAAAGAGTGGTTAAGGGGT

ATCAAAAATAAAGTCAAACATTTTCCTAGTTGTTATAAGCATTTCGAGAACATGAAGCCC

AACCACGAAAGTAACTTTACCTTCACCTGACCCCACGAAAGCTAAGAAA

>917_2_Sample_ASV28

CACCGCGGTTATACGAGAAACCCTAGTTGACAAATACGGCGTAAAGAGTGGTTAAGGGGT

ATCAAAAATAAAGTCAAACATTTTCCTAGTTGTTATAAGCATTTCGAGAACATGAAGCCC

AACCACGAAAGTAACTTTACCTTCACCTGACCCCACGAAAGCTAAGAAA

>917_Sample_ASV16

CACCGCGGTTATACGAAAGACCCCAGTTTATAGACACGGCGTAAAGGGTGGTTATAGGAC

AAACTAACATAGAGCCAAAGACCCCCTAAGCCGTCATACGCTCACGAGGGCTCGAAGCCC

CTATACGAAAGTAGCTCTACCAAAAAATGCTCGACTCCACGAAAGCTGGGACA

>917_2_Sample_ASV29

CACCGCGGTTATACGAGAGACCCTAGTTGATAAATACGGCGTAAAGAGTGGTTATGGGAA

AAACAAATAAAGTCAAACAACCTCTTAGCTGTTATACGCATTATGAGAGCATGAAGCCCC

CTCACGAAAGTAACTTTAATACCTCCTGACCCCACGAAAGCTAAGAAA

>917_Sample_ASV17

CACCGCGGTTATACGAGAGACCCTAGTTGATAAATACGGCGTAAAGAGTGGTTATGGGAA

AAACAAATAAAGTCAAACAACCTCTTAGCTGTTATACGCATTATGAGAGCATGAAGCCCC

CTCACGAAAGTAACTTTAATACCTCCTGACCCCACGAAAGCTAAGAAA

>917_2_Sample_ASV30

AACCGCGGTTATACGAGAGACCCTAATTGATAGCTCACGGCGTAAAGGGTGGTTTGGGGA

AACCTCAAAATAAAGCTAAAGACCTTCTAAGCCGTTACACGCATACCGAAGGCACGAGGC

CCTAACACGAAAGTAGCTTTACTATTACCCCTGACGCCACGAAAGCTAAGAAA

>917_Sample_ASV18

CACCGCGGTTATACGAGAGACCCTAATTGATAGGTACGGCGTAAAGAGTGGTTTGGGATA

ACCCAAAAATAAAGCTAAAGACCTTCTAAGCCGTCACACGCATACCGAAGGCACGAGGCC

CCAACACGAAAGTAGCTTTACTATTACCCCGACGCCACGAAAGCTAAGAAA

>917_2_Sample_ASV31

CACCGCGGTTATACGAAAGACCCTAGTTGATGGATCACGGCGTAAAGGGTGGTTAAGGGA

TATAAAAATAAAGCTAAAAATCCTCTTAGCTGTCATACGCTTTCCGAAGACATGAGGCCC

AATTACGAAAGTAGCTTTAAACCACCCTGACACCACGAAAGCCAAGAAA

>917_2_Sample_ASV32

CACCGCGGTTATACGAAAGACCCCAGTTTATAGATGCGGCGTAAAGGGTGGTTAAGAGAA

AATCAAAATAAGGCCAAAGATCCCCTAAGCAGTCATACGCTCCACGAGGACACGAAACCC

AAATACGAAAGTTGCCTTAATAAACTTCACCTGATCCCACGAAAGCTGAAAAA

>917_Sample_ASV19

CACCGCGGTCATACGATTAACCCAAGTTAATAGGCACACGGCGTAAAGCGTGTTTAAGCA

CTACGCCAAATAAAGTTAAACTCCAATTAAGCTGTAAAAAGCCATAATCAAGACGAAAAT

AAATAACGAAAGTAACTTTACAACCGCTGAAACACGATAGCTAAGATC

>917_Sample_ASV20

CACCGCGGTTATACGAGAGGCCCTAGTTGATAACTACCGGCGTAAAGAGTGGTTATGGAA

AAATATTTAATAAAGCCGAACACCCCCTCAGCCGTCATACGCACCTGGGAGCACGAAGAC

CTACTGCGAAAGCAGCTTTAATTATGCCTGACCCCACGACAGCTAAGAAA

>917_Sample_ASV21

CACCGCGGTTATACGAAAGACCCTAGTTGATAGGCACGGCGTAAAGGGTGGTTAGGGATA

TATCAAAAATAAAGTTAAAGAGCCTCTAAGCTGTCGCACGCATCCCGAGAGCTCGAGACC

CAGACACGAAAGTAACTTTAAAATACCTCACCTGACCCCACGAAAGCTAAGAAA

>917_2_Sample_ASV33

CACCGCGGTTATACGAAAGACCCTAGTTGATAGCCACGGCGTAAAGGGTGGTTAAGGTAT

ACAACAAATAAAGCTAAATAGCCTCTAGGCCGTCGCACGCATTCCGAGAGCTCGAAACCC

AAAAACGAAAGTAGCTTTAAAATAAACAGACCTGACCCCACGAAAGCTAAGAAA

>917_Sample_ASV22

CACCGCGGTTATACGAGAGACCCCAGTTGATAAACACGGCGTAAAGAGTGGTTAAGGGCA

CAAAACAAATAAAGTCAAACAACCTCTTAGCTGTTATACGCATTATGAGGATATGAAGCC

CCCCTACGAAAGTAACTTTACCAACCCCTGACCCCACGAAAGCTAAGAAA

>917_2_Sample_ASV34

CACCGCGGTTATACGAGAGGCCCCAGTTGATAAACACGGCGTAAAGAGTGGTTAAGGGCA

CAAAACAAATAAAGTCAAACAACCTCTTAGCTGTTATACGCATTATGAGGATATGAAGCC

CCCCTACGAAAGTAACTTTACCAACCCCTGACCCCACGAAAGCTAAGAAA

>917_2_Sample_ASV35

CACCGCGGTTATACGAGAGACCCCAGTTGATAAACACGTCGTAAAGAGTGGTTAAGGGCA

CAAAACAAATAAAGTCAAACAACCTCTTAGCTGTTATACGCATTATGAGGATATGAAGCC

CCCCTACGAAAGTAACTTTACCAACCCCTGACCCCACGAAAGCTAAGAAA

>917_2_Sample_ASV36

CACCGCGGTTATACGAGAGACCCCAGTTGATAAACACGGCGTAAAGAGTGGTTAAGGGCA

CAAAACAAATAAAGTCAAACAACCTCTTAGCTGTTATACGCATTATGAGGATATGAAGCC

CCCCTACGAAAGTAACTTTACCAACCCCTGACCCCACGAAAGCTAAGAAA

>917_Sample_ASV23

CACCGCGGTTATACGAGAGACCCTAGTTGATATATACGGCGTAAAGAGTGGTTAAGGGCA

CAAAACAAATAAAGTCAAACAACCTCTTAGCTGTTATACGCATTATGAGGATATGAAGCC

CCCCTACGAAAGTAACTTTACCAACCCCTGACCCCACGAAAGCTAAGAAA

>917_2_Sample_ASV37

CACCGCGGTTATACGAAAGACCCTAGTTTATAGGCACGGCGTAAAGGGTGGTTAGGGGAT

AAACAAAATAAAGCCAAAGACCTTCTAAGTCGTCATACACTCATGAAGGCACGAAGCCCA

AATACGAAAGTAACTTTACAACTTAGACCCGACTCCACGAAAGCTGGGGGA

>917_2_Sample_ASV38

CACCGCGGTTATACGAGAGACCCTAATTAATAGATACGGCGTAAAGAGTGGTTTAGGACA

AAAATTAATAAAGCCGAAGTTTCTCCTAGCCGTTGTACGCACTCTGAGAACACGAAGCCC

CACTACGAAAGTAGCTTTACCTATTATTTCCTGACCCCACGAAAGCTAAGAAA

>917_2_Sample_ASV39

CACCGCGGTTATACGAGAGACCCTAATTAATAGATACGGCGTAAAGAGTGGTTTAGGACA

AAAATTAATAAAGCCGAAGTTTCTCCTAGCCGTTGTACGCACTCTGAGAACACGAAGCCC

CACTACGAACGTAGCTTTACCTATTATTTCCTGACCCCACGAAAGCTAAGAAA

>917_2_Sample_ASV40

CACCGCGGTTATACGAGAGGCCCAAGTTGATAGACATCGGCGTAAAGAGTGGTTAAGGAA

GTCTCAAAACTAAAGCCGAATGCCTTCAAGACTGTTATACGTTTCCGAAGGTAAGAAGCC

CCACTACGAAAGTGGCTTTAACCACACCCGACCCCACGAAAGCTGTGAAA

>917_2_Sample_ASV41

CACCGCGGTTATACGAGAGGCCCAAGTTGATAGACATCGGCGTAAAGAGTGGTTAAGGAA

GTCTCAAAACTAAAGCCGAATGCCTTCAAGACTGTTATACGTTTCCGAAGGTAAGAAGCC

CCACTACGAAAGTGGCTTTACTCCACCTGACTCCACGAAAGCTGTGAAA

>917_2_Sample_ASV42

CACCGCGGTTATACGAGAGGCCCAAGTTGATAGACATCGGCGTAAAGAGTGGTTAAGGAA

GTCTCAAAACTAAAGCCGAATGCCTTCAAGACTGTTATACGTTTCCGAAGGTAAGAAGCC

CCACTACGAAAGTGGCTTTATTCCACCTGACCCCACGAAAGCTGTGAAA

>917_Sample_ASV24

CACCGCGGTTATACGAGAGACTCCAGTTGATAGCTACGGCGTAAAGAGTGGTTTGGGGCC

CGCCCAAAATAAAGCCAAAGACCTCCCAAGCCGTCAAACGCACCCCGGAGGCACGAAGTC

CTAACGCGAAAGCAACTTTACCTTCCCCGACGCCACGAAAGCTAAGAAA

>917_2_Sample_ASV43

CACCGCGGTTATACGAGAGACCCTAATTGATAGACTACGGCGTAAAGAGTGGTTTAGAAC

AAAAATTAATAAAGCCAAAGATCCTCCTGGCCGTCGCACGCATTTCGAGGATACGAAGCC

CCACTACGAAAGTAGCTTTACTACAACTTTTCTGACCCCACGAAAGCTAAGAAA

>917_Sample_ASV25

CACCGCGGTTATACGAAAGACCCTAGTTGATAGATCACGGCGTAAAGGGTGGTTAAGGAG

AACAAAAATAAAGCTAAAAATCCTCTAAGCTGTCATACGCTTTCCGAAGACATGAGACCC

AACCACGAAAGTAACTTTAAACTTTCCTGACACCACGAAAGCCAAGAAA

>918_Sample_ASV1

CACCGCGGTTATACGAAAGACCCTAGTTGATGGGTGCGGCGTAAAGGGTGGTTAGGGACT

ACAAAAAATAGAGCTAAAGATCCTCTAGGTCGTCATACACATCACGAGGAAACGAAGCCC

AAATACGAAAGTAGCTCTAAATATAATCCTGACGCCACGAAAGCCAAGAAA

>918_Sample_ASV2

CACCGCGGTTATACGAGAGACCCTAGTTGATTAAAGCGGCGTAAAGAGTGGTTATGGAAC

TATTCTTTTAAAGCAGAAAACCTCTCAAACTGTTATACGCACCCAGAGGTCTGAATCCCT

CACACGAAAGTGACTTTATTTATGCCTACCAGAACCCACGAAAGCTGGGACA

>918_Sample_ASV3

CACCGCGGTTATACGAGAGACCCTAGTTGATATACACGGCGTAAAGAGTGGTTTGGGGTA

TATAATAAATAAAGCCAAAGACCTTCTAGGCCGTTGAACGCACCACGAAGGAGCGAAGTC

CCAATACGAAAGTAGCTTTATAAAACCTGACGCCACGAAAGCTAAGAAA

>918_Sample_ASV4

CACCGCGGTTATACGAGAGGCCCAAGTTGACAGACACCGGCGTAAAGAGTGGCTAGGGAA

AAATTACTACTAAAGCCGAACACCTTCAGAACTGTCATACGTTCCCGAAGATAAGAAGCC

CCACCACGAAAGTGACTTTATATTACCCGACCCCACGAAAGCTAAGAAA

>918_Sample_ASV5

CACCGCGGTTATACGAGAGGCCCAAGTTGACAGACACCGGCGTAAAGAGTGGCTAGGGAA

AAATTACTACTAAAGCCGAACACCTTCAGAACTGTCATACGTTCCCGAAGATAAGAAGCC

CCACCACGAAAGTGACTTTATATTACCCGACCCCACGAAAGCTGTGAAA

>918_Sample_ASV6

AACCGCGGTTATACGAGAGGCCCAAGTTGACAGACACCGGCGTAAAGAGTGGCTAGGGAA

AAATTACTACTAAAGCCGAACACCTTCAGAACTGTCATACGTTCCCGAAGATAAGAAGCC

CCACCACGAAAGTGACTTTATATTACCCGACCCCACGAAAGCTAAGAAA

>918_Sample_ASV7

CACCGCGGTTATACGAGAGGCCCAAGTTGACAGACACCGGCGTAAAGAGTGGCTAGGGAA

AAATTACTACTAAAGCCGAACACCTTCAGAACTGTCATACGTTCCCGAAGATAAGAAGCC

CCACCACGAAAGTGACTTTATATTACCCGACCCCACGAAAGCTGGGGGA

>918_Sample_ASV8

CACCGCGGTTATACGAGAGGCCCAAGTTGACAGACACCGGCGTAAAGAGTGGCTAGGGAA

AAATTACTACTAAAGCCGAACACCTTCAGAACTGTCATACGTTCCCGAAGATAAGAAGCC

CCACCACGAAAGTGACTTTATATTACCCGACCCCACGAAAGCCAAGAAA

>918_Sample_ASV9

CACCGCGGTTATACGAGAGGCCCAAGTTGATAAGCCCGGCGTAAAGAGTGGTTATGGAGA

ATAAAACACTAAAGCTAAAGACCCCCTAGGCTGTTATACGCACCTGGCGGCTCGAACCAC

CTATACGAAAGTAGCTTTACCCCCCTTCCACCAGAATCCACGACAGCTGGGGCA

>918_Sample_ASV10

CACCGCGGTTATACGAGAGGCCCAAGTTGACAGACATCGGCGTAAAGAGTGGTTAAGGAA

ACCCCATTACTAAAGCCGAACGCCCTCAGAACTGTCATACGTTCCCGAAGGTAAGAAGCC

CCACTACGAAAGTGGCTTTAACCACACCCGACCCCACGAAAGCTGTGAAA

>918_Sample_ASV11

CACCGCGGTTATACGAGAGGCCCAAGTTGATAGATATCGGCGTAAAGAGTGGTTAAGGAA

ACCCCGTTACTAAAGCCGAACGCCCTCAGAACTGTCATACGTTCCCGAAGGTAAGAAGCC

CCACTACGAAAGTGGCTTTAACCACACCCGACCCCACGAAAGCTGTGAAA

>918_Sample_ASV12

CACCGCGGTTATACGAGAGGCCCAAGTTGACAGACACCGGCGTAAAGAGTGGTTAAGGAA

ATTCATCAACTAAAGCCGAATGCCCTCAGGACTGTTATACGTTTGCGAAGGTAAGAAGCC

CTACTACGAAAGTGGCTTTATTCCACCTGATTCCACGAAAGCTGCGGAA

>918_Sample_ASV13

CACCGCGGTTATACGAGAAACCCTAGTTGACAAATACGGCGTAAAGAGTGGTTAAGGGGT

ATCAAAAATAAAGTCAAACATTTTCCTAGTTGTTATAAGCATTTCGAGAACATGAAGCCC

AACCACGAAAGTAACTTTACCTTCACCTGACCCCACGAAAGCTAAGAAA

>918_Sample_ASV14

CACCGCGGTTATACGAAAGACCCTAGTTTATAAGTACGGCGTAAAGGGTGGTTAGGGGAC

AAACAAAATAAAGCCAAAGACCTCCTAAGCCGTCATACGCTTACGGAGGCACGAAGCCCA

AACACGAAAGTAGCTTTACCGAACATGCCCGACTCCACGAAAGCTGGGAAA

>918_Sample_ASV15

CACCGCGGTTATACGAGAGACCCTAGTTGATAAATACGGCGTAAAGAGTGGTTATGGGAA

AAACAAATAAAGTCAAACAACCTCTTAGCTGTTATACGCATTATGAGAGCATGAAGCCCC

CTCACGAAAGTAACTTTAATACCTCCTGACCCCACGAAAGCTAAGAAA

>918_Sample_ASV16

AACCGCGGTTATACGAGAGACCCTAATTGATAGCTCACGGCGTAAAGGGTGGTTTGGGGA

AACCTCAAAATAAAGCTAAAGACCTTCTAAGCCGTTACACGCATACCGAAGGCACGAGGC

CCTAACACGAAAGTAGCTTTACTATTACCCCTGACGCCACGAAAGCTAAGAAA

>918_Sample_ASV17

CACCGCGGTTATACGAAAGACCCTAGTTGATGGATCACGGCGTAAAGGGTGGTTAAGGGA

TATAAAAATAAAGCTAAAAATCCTCTTAGCTGTCATACGCTTTCCGAAGACATGAGGCCC

AATTACGAAAGTAGCTTTAAACCACCCTGACACCACGAAAGCCAAGAAA

>918_Sample_ASV18

CACCGCGGTTATACGAGAGGCCCTAGTTGATAACTACCGGCGTAAAGAGTGGTTATGGAA

AAATATTTAATAAAGCCGAACACCCCCTCAGCCGTCATACGCACCTGGGAGCACGAAGAC

CTACTGCGAAAGCAGCTTTAATTATGCCTGACCCCACGACAGCTAAGAAA

>918_Sample_ASV19

CACCGCGGTTATACGAAAGACCCTAGTTTATAGACACGGCGTAAAGGGTGGTTCGGGACT

AAACAAAATAAAGCCAAAGACCTCCTAAGCCGTCATACGCTCACGGAAGCACGAAGCCCA

AGTACGAAAGTAGCTTTACAAAACATGCCTGACTCCACGAAAGCTAGGAAA

>918_Sample_ASV20

CACCGCGGTTATACGAGAGGCCCAAGTTGATACTCCACGGCGTAAAGAGTGGTTAGAAAA

GACCTACTACTAAAGCCGAACGCCTTCAAAGCTGTTATACGCATCCGAAGGTGAGAAGCC

CACCCACGAAAGTGGCTTTACAACCTTGACTCCACGAAAGCTATGACA

>918_Sample_ASV21

CACCGCGGTTATACGAGAGACCCCAGTTGATAAACACGGCGTAAAGAGTGGTTAAGGGCA

CAAAACAAATAAAGTCAAACAACCTCTTAGCTGTTATACGCATTATGAGGATATGAAGCC

CCCCTACGAAAGTAACTTTACCAACCCCTGACCCCACGAAAGCTAAGAAA

>918_Sample_ASV22

CACCGCGGTTATACGAAAGACCCTAGTTTATAGGCACGGCGTAAAGGGTGGTTAGGGGAT

AAACAAAATAAAGCCAAAGACCTTCTAAGTCGTCATACACTCATGAAGGCACGAAGCCCA

AATACGAAAGTAACTTTACAACTTAGACCCGACTCCACGAAAGCTGGGGGA

>918_Sample_ASV23

CACCGCGGTTATACGAAAGACCCTAGTTTATAGGCACGGCGTAAAGGGTGGTTAGGGGAT

AAACAAAATAAAGCCAAAGACCTTCTAAGTCGTCATACACTCATGAAGGCACGAAGCCCA

AATACGAAAGTAACTTTACAACTTAGACCCGACTCCACGAAAGCTAAGAAA

>918_Sample_ASV24

AACCGCGGTTATACGAAAGACCCTAGTTTATAGGCACGGCGTAAAGGGTGGTTAGGGGAT

AAACAAAATAAAGCCAAAGACCTTCTAAGTCGTCATACACTCATGAAGGCACGAAGCCCA

AATACGAAAGTAACTTTACAACTTAGACCCGACTCCACGAAAGCTAAGAAA

>918_Sample_ASV25

AACCGCGGTTATACGAGAGACCCTAGTTTATAGGCACGGCGTAAAGGGTGGTTAGGGGAT

AAACAAAATAAAGCCAAAGACCTTCTAAGTCGTCATACACTCATGAAGGCACGAAGCCCA

AATACGAAAGTAACTTTACAACTTAGACCCGACTCCACGAAAGCTAAGAAA

>918_Sample_ASV26

CACCGCGGTTATACGAGAGACCCTAATTAATAGATACGGCGTAAAGAGTGGTTTAGGACA

AAAATTAATAAAGCCGAAGTTTCTCCTAGCCGTTGTACGCACTCTGAGAACACGAAGCCC

CACTACGAAAGTAGCTTTACCTATTATTTCCTGACCCCACGAAAGCTAAGAAA

>918_Sample_ASV27

CACCGCGGTTATACGAGAGGCCCAAGTTGATAGACATCGGCGTAAAGAGTGGTTAAGGAA

GTCTCAAAACTAAAGCCGAATGCCTTCAAGACTGTTATACGTTTCCGAAGGTAAGAAGCC

CCACTACGAAAGTGGCTTTACTCCACCTGACTCCACGAAAGCTGTGAAA

>918_Sample_ASV28

CACCGCGGTTATACGAGAGGCCCAAGTTGATAGACATCGGCGTAAAGAGTGGTTAAGGAA

GTCTCAAAACTAAAGCCGAATGCCTTCAAGACTGTTATACGTTTCCGAAGGTAAGAAGCC

CCACTACGAAAGTGGCTTTATTCCACCTGACCCCACGAAAGCTGTGAAA

>918_Sample_ASV29

CACCGCGGTTATACGAGAGGCCCAAGTTGACAGATATCGGCGTAAAGAGTGGTTAAGGAA

ATCTTAAAACTAAAGCCGAATGCCTTCAAGACTGTTATACGTTTCCGAAGGTAAGAAGCC

CCACTACGAAAGTGGCTTTACTCCACCTGACTCCACGAAAGCTGTGAAA

>918_Sample_ASV30

CACCGCGGTTATACGAGAGGCCCAAGTTGATAGACATCGGCGTAAAGAGTGGTTAAGGAA

GTCTCAAAACTAAAGCCGAATGCCTTCAAGACTGTTATACGTTTACGAAGGTAAGAAGCC

CCACTACGAAAGTGGCTTTATTCCACCTGACCCCACGAAAGCTGTGAAA

>918_Sample_ASV31

CACCGCGGTTATACGAGAGACCCTAATTGATAGACTACGGCGTAAAGAGTGGTTTAGAAC

AAAAATTAATAAAGCCAAAGATCCTCCTGGCCGTCGCACGCATTTCGAGGATACGAAGCC

CCACTACGAAAGTAGCTTTACTACAACTTTTCTGACCCCACGAAAGCTAAGAAA

>989_Sample_ASV1

TACCGCGGTTATACGAAAGACCCAAGTTAATAAACTACGGCGTAAAGCGTGGTTAGAGAC

CTCCAATAATTAAAGCCAAAGACCTTCTAAGCCGTCGCACGCCCCGCGAAGACACGAAGC

CCAAAAACGAAAGTAGCTTTATTCTTACTCGACCCCACGAAAGCTAGGGCA

>989_Sample_ASV2

CACCGCGGTTATACGAAAGACCCTAGTTGATGGGTGCGGCGTAAAGGGTGGTTAGGGACT

ACAAAAAATAGAGCTAAAGATCCTCTAGGTCGTCATACACATCACGAGGAAACGAAGCCC

AAATACGAAAGTAGCTCTAAATATAATCCTGACGCCACGAAAGCCAAGAAA

>989_Sample_ASV3

CACCGCGGTTATACGAGAGACCCTAGTTGATTAAAGCGGCGTAAAGAGTGGTTATGGAAC

TATTCTTTTAAAGCAGAAAACCTCTCAAACTGTTATACGCACCCAGAGGTCTGAATCCCT

CACACGAAAGTGACTTTATTTATGCCTACCAGAACCCACGAAAGCTGTGAAA

>989_Sample_ASV4

CACCGCGGTTATACGAGAGACCCTAGTTGATTAAAGCGGCGTAAAGAGTGGTTATGGAAC

TATTCTTTTAAAGCAGAAAACCTCTCAAACTGTTATACGCACCCAGAGGTCTGAATCCCT

CACACGAAAGTGACTTTATTTATGCCTACCAGAACCCACGAAAGCTGGGACA

>989_Sample_ASV5

CACCGCGGTTATACGAGAGGCCCAAGTTGACAGACACCGGCGTAAAGAGTGGCTAGGGAA

AAATTACTACTAAAGCCGAACACCCTCAGAACTGTTATACGTTCCCGAAGGCAAGAAGCT

CCACCACGAAAGTGACTTTATATTACCCGACCCCACGAAAGCTGTGAAA

>989_Sample_ASV6

CATGGCGATTATACGAGAGGCCCAAGTTGACAGACACTGGCGTAAAGAGTGGCTAAGGAA

AAATTACTACTAAAGCCGAACACCTTCAGAACTGTCATACGTTCCCGAAGATAAGAAGCC

CCACCACGAAAGTGACTTTATATTACCCGACCCCACGAAAGCTGTGAAA

>989_Sample_ASV7

CACCGCGGTTATACGAGAGGCCCAAGTTGACAGACACCGGCGTAAAGAGTGGCTAGGGAA

AAATTACTACTAAAGCCGAACACCTTCAGAACTGTCATACGTTCCCGAAGATAAGAAGCC

CCACCACGAAAGTGACTTTATATTACCCGACCCCACGAAAGCTGTGAAA

>989_Sample_ASV8

CACCGCGGTTATACGAGAGGCCCAAGTTGACAGACACCGGCGTAAAGAGTGGCTAGGGAA

AAATTACTACTAAAGCCGAACACCTTCAGAACTGTCATACGTTCCCGAAGATAAGAAGCC

CCACCACGAAAGTGACTTTATATTACCCGACCCCACGAAAGCCAAGAAA

>989_Sample_ASV9

CACCGCGATTATACGAGAGGCCCAAGTTGACAGACATCGGCGTAAAGAGTGGTTAAGGAA

AAATTACTGCTAAAGCCGAACACCTTCAGAACTGTCATACGTTCCCGAAGATAAGAAGCC

CCACCACGAAAGTGACTTTATATTACCCGACCCCACGAAAGCTGTGAAA

>989_Sample_ASV10

CATGGCGATTATACGAGAGGCCCAAGTTGACAGACACTGGCGTAAAGAGTGGCTAGGGAA

AAATTACTACTAAAGCCGAACACCTTCAGAACTGTCATACGTTCCCGAAGATAAGAAGCC

CCACCACGAAAGTGACTTTATATTACCCGACCCCACGAAAGCTGTGAAA

>989_Sample_ASV11

CATGGCGATTATACGAGAGGCCCAAGTTGACAGACACTGGCGTAAAGAGTGGCTAAGGAA

AAACTACTACTAAAGCCGAACACCTTCAGAACTGTCATACGTTCCCGAAGATAAGAAGCC

CCACCACGAAAGTGACTTTATATTACCCGACCCCACGAAAGCTGTGAAA

>989_Sample_ASV12

CACCGCGATTATACGAGAGGCCCAAGTTGACAGACATCGGCGTAAAGAGTGGTTAAGGAA

AAATTACTACTAAAGCCGAACACCTTCAGAACTGTCATACGTTCCCGAAGATAAGAAGCC

CCACCACGAAAGTGACTTTATATTACCCGACCCCACGAAAGCTGTGAAA

>989_Sample_ASV13

CACCGCGGTTATACGAGAGGCCCAAGTTGACAGACACCGGCGTAAAGAGTGGCTAGGGAA

AAATTACTACTAAAGCCGAACACCTTCAGAACTGTCATACGTTCCCGAAGATAAGAAGCC

CCACCACGAAAGTGGCTTTACAATGCCTGACCCCACGAAAGCTGTGAAA

>989_Sample_ASV14

CACCGCGGTTATACGAGAGGCCCAAGTTGACAGACACCGGCGTAAAGAGTGGCTAGGGAA

AAATTACTACTAAAGCCGAACACCTTCAGAACTGTCATACGTTCCCGAAGATAAGAAGCC

CCACCACGAAAGTGGCTTTATATTACCCGACACCACGAAAGCCAAGAAA

>989_Sample_ASV15

CACCGCGGTTATACGAGAGGCCCAAGTTGACAGACACCGGCGTAAAGAGTGGCTAGGGAA

AAATTACTACTAAAGCCGAACACCCTCAGAACTGTCATACGTTCCAGAAGGCAAGAAGCT

CCACCACGAAAGTGGCTTTATATTACCCGACCCCACGAAAGCTGTGAAA

>989_Sample_ASV16

CACCGCGGTTATACGAGAGGCCCAAGTTGACAGACACCGGCGTAAAGAGTGGCTAGGGAA

AAATTACTACTAAAGCCGAACACCTTCAGAACTGTCATACGTTCCAGAAGGCAAGAAGCT

CCACCACGAAAGTGGCTTTATAATACCCGACCCCACGAAAGCTGTGAAA

>989_Sample_ASV17

CACCGCGGTTATACGAAAGACCCTAGTTGACAGACACCGGCGTAAAGAGTGGCTAGGGAA

AAATTACTACTAAAGCCGAACACCTTCAGAACTGTCATACGTTCCCGAAGGTAAGAAGCC

CCACCACGAAAGTGGCTTTATAATACCCGACCCCACGAAAGCTGTGAAA

>989_Sample_ASV18

CACCGCGGTTATACGAGAGGCCCAAGTTGACAGACACCGGCGTAAAGAGTGGCTAGGGAA

AAATTACTACTAAAGCCGAACACCCTCAGAACTGTCATACGTTCCCGAAGGTAAGAAGCC

CCACCACGAAAGTGGCTTTATAATACCCGACCCCACGAAAGCTGTGAAA

>989_Sample_ASV19

CACCGCGGTTATACGAGAGGCCCAAGTTGACAGACACCGGCGTAAAGAGTGGCTAGGGAA

AAATTTCTACTAAAGCCGAACACCTTCAGAACTGTCATACGTTCCCGAAGATAAGAAGCC

CCACCACGAAAGTGGCTTTATATTACCCGACACCACGAAAGCCAAGAAA

>989_Sample_ASV20

CACCGCGGTTATACGAGAGGCCCAAGTTGACAGACACCGGCGTAAAGAGTGGCTAAGGAA

AAATTTCTACTAAAGCCGAACACCTTCAGAACTGTCATACGTTCCCGAAGATAAGAAGCC

CCACCACGAAAGTGGCTTTATATTACCCGACACCACGAAAGCTGTGAAA

>989_Sample_ASV21

CACCGCGGTTATACGAAAGACTCTAGTTGACAAACACGGCGTAAAGGGTGGTTAGGATAA

CAACAAAATAAAGCTAAAGACTTACCAAGCCGTCATACGCCCATGTAAGAGCGAAAACCA

CAAACGAAAGTAACTTTATTACACCATCCGAATCCACGAAAGCTAAGAAA

>989_Sample_ASV22

CACCGCGGTTAAACGAGAGACCCTAGTTGATCATCACGGCGTAAAGAGTGGTTAGGGGAT

TATCATAAATAAAGCCAAAGACCTCCCAAGCTGTCGCACGCATTCCGGAGGCGCGAAGCC

CACACACGAAAGTAGCTTTAACTATTGACCCTGATGCCACGAAAGCTAAGAAA

>989_Sample_ASV23

CACCGCGGTTATACGAGAGACCCTAGTTGATATATACGGCGTAAAGAGTGGTTTGGGATA

TTTCTCCAAAATAAAGTCGAAGATCTCCCAAGCCGTCACACGCATCCCGGAGACACGAAG

CCCTGATACGAAGGTAGCTTTATTAACACCCCGACGCCACGAAAGCTAAGAAA

>989_Sample_ASV24

CACCGCGGTTATACGAGAGACCCTAGTTGATATATACGGCGTAAAGAGTGGTTTGGGATA

TTTCTCCAAAATAAAGCCGAAGATCTCCCAAGCCGTCACACGCATCCCGGAGACACGAAG

CCCTGATACGAAGGTAGCTTTATTAACACCCCGACGCCACGAAAGCTAAGAAA

>989_Sample_ASV25

CACCGCGGTTATACGAGAGACCCTAGTTGATATATACGGCGTAAAGAGTGGTTTGGGATA

TTTCTCCAAAATAAAGCCGAAGATCTCCCAAGCCGTCACACGCATCCCGGAGACACGAAG

CCCTGATACGAAGGTAGCTTTATTAATTCCCCGACGCCACGAAAGCTAAGAAA

>989_Sample_ASV26

CACCGCGGTTATACGAGAGACCCTAGTTGATATATACGGCGTAAAGAGTGGTTTGGGATA

TTTCTCCAAAATAAAGTCGAAGATCTCCCAAGCCGTCACACGCATCCCGGAGACACGAAG

CCCTGATACGAAGGTAGCTTTATTAATTCCCCGACGCCACGAAAGCTAAGAAA

>989_Sample_ASV27

CACCGCGGTTATACGAAAGACCCTAGTTGATAGATCACGGCGTAAAGGGTGGTTAGGGAA

TACAAAAATAAAGCTAAAAATCCTCTAAGCTGTCATACGCTTCCCGAAGACATGAAACCC

AACCACGAAAGTAGCTTTAAACTCTCCTGACACCACGAAAGCCAAGGAA

>989_Sample_ASV28

CACCGCGGTTATACGAGAGGCCCAAGTTGATAAGCCCGGCGTAAAGAGTGGTTATGGAGA

ATAAAACACTAAAGCTAAAGACCCCCTAGGCTGTTATACGCACCTGGCGGCTCGAACCAC

CTATACGAAAGTAGCTTTACCCCCCTTCCACCAGAATCCACGACAGCTGGGGCA

>989_Sample_ASV29

CACCGCGGTTATACGAGAGGCCCAAGTTGATAAGCCCGGCGTAAAGAGTGGTTATGGAGA

ATAAAACACTAAAGCTAAAGACCCCCTAGGCTGTTATACGCACCTGGCGGCTCGAACCAC

CTATACGAAAGTAGCTTTACCCCCCTTCCACCAGAATCCACGAAAGCTGTGAAA

>989_Sample_ASV30

CACCGCGGCCATACGATTAACCCAAACTAATAGACCCACGGCGTAAAGCGTGTTACAGAG

AAAAAAATATACTAAAGTTAAATTTTAACTAGGCCGTAGAAAGCTACAGTTAACATAAAA

ATACAGCACGAAAGTAACTTTAACACCTCCGACTACACGACAGCTAAGACC

>989_Sample_ASV31

CACCGCGGTTATACGAGAGGCCCAAGTTGACAGACATCGGCGTAAAGAGTGGTTAAGGAA

ACCCCATTACTAAAGCCGAACGCCCTCAGAACTGTCATACGTTCCCGAAGGTAAGAAGCC

CCACTACGAAAGTGGCTTTAACCACACCCGACCCCACGAAAGCTGTGAAA

>989_Sample_ASV32

CACCGCGGTTATACGAGAGGCCCAAGTTGACAGACACCGGCGTAAAGAGTGGTTAAGGAA

ATTCATCAACTAAAGCCGAATGCCCTCAGGACTGTTATACGTTTGCGAAGGTAAGAAGCC

CTACTACGAAAGTGGCTTTATTCCACCTGATTCCACGAAAGCTGTGAAA

>989_Sample_ASV33

CACCGCGGTTATACGAGAGGCCCAAGTTGACAGACACCGGCGTAAAGAGTGGTTAAGGAA

ATTCATCAACTAAAGCCGAATGCCCTCAGGACTGTTATACGTTTGCGAAGGTAAGAAGCC

CTACTACGAAAGTGGCTTTATTCCACCTGATTCCACGAAAGCTGCGGAA

>989_Sample_ASV34

CACCGCGGTTATACGAGAGGCCCCAGTTGATATTAGCGGCGTAAAGGGTGGTTAAGGACT

TTAATAACTAAAGCTAAACACTTCCCCGGCTGTTGTACGTAGACCGGAAGCTTGAAGCCC

AGACACGAAAGTAGCTTTATTTCCCCTGACCCCACGAAAGCTGAGAAA

>989_Sample_ASV35

CACCGCGGTTATACGAGAGGCCCCAGTTGATATTAGCGGCGTAAAGGGTGGTTAAGGACT

TTAATAACTAAAGCTAAACACTTCCCCGGCTGTTGTACGTAGACCGGAAGCTTGAAGCCC

AGACACGAAAGTAGCTTTATTCCCCCTGACCCCACGAAAGCTGAGAAA

>989_Sample_ASV36

CACCGCGGTTATACGAGAGGCCCCAGTTGATATTAGCGGCGTAAAGGGTGGTTAAGGACT

TTAATAACTAAAGCCAAACACTTCCCCGGCTGTTGTACGTAGACCGGAAGCTTGAAGCCC

AGACACGAAAGTAGCTTTATTTCCCCTGACCCCACGAAAGCTGAGAAA

>989_Sample_ASV37

CACCGCGGTCACACGATTAACCCAAGTCAATAGAAGCCGGCGTAAAGAGTGTTTTAGATC

ACCCCCTCCCCAATAAAGCTAAAACTCACCTGAGTTGTAAAAAACTCCAGTTGACACAAA

ATAGACTACGAAAGTGGCTTTAACATATCTGAACACACAATAGCTAAGACC

>989_Sample_ASV38

CACCGCGGTTATACGAGAGACCCCAGTTGATAGCTACGGCGTAAAGAGTGGTTAAGGACC

CCATTTAATAAAGCCAAAGACCCCCCAAGCCGTCATACGCACACGAGGGCGCGAAGCTCG

CACACGAAAGTAGCTCTAATTTACCCGACGCCACGAAAGCTAAGGAA

>989_Sample_ASV39

CACCGCGGTTATACGAGAAACCCTAGTTGACAAATACGGCGTAAAGAGTGGTTAAGGGGT

ATCAAAAATAAAGTCAAACATTTTCCTAGTTGTTATAAGCATTTCGAGAACATGAAGCCC

AACCACGAAAGTAACTTTACCTTCACCTGACCCCACGAAAGCTAAGAAA

>989_Sample_ASV40

GACCGCGGTTATACGAGAGACTCTAGTTGATAGATACGGCGTAAAGAGTGGTTTGGGATA

AACTATAAATAAAGCCAAAGACCTTCCAAGCTGTTAAACGCATACCGAAGGCACGAAGCC

CCAACACGAAAGTAGCTTTACTACTACCCCCGATGCCACGAAAGCTAAGACA

>989_Sample_ASV41

CACCGCGGTTATACGAAAGACCCCAGTTTATAGACACGGCGTAAAGGGTGGTTATAGGAC

AAACTAACATAGAGCCAAAGACCCCCTAAGCCGTCATACGCTCACGAGGGCTCGAAGCCC

CTATACGAAAGTAGCTCTACCAAAAAATGCTCGACTCCACGAAAGCTGGGACA

>989_Sample_ASV42

CACCGCGGTTATACGAGAGACCCTAATTGATAGACACGGCGTAAAGAGTGGTTTGGGAAA

GCCCAAAAATAAAGCTAAAGACCTTCTAAGCCGTCACACGCACGCCGAAGGCACGAAGCC

CTGACACGAAAGTAGCTTTACTATTACCCCCGACGCCACGAAAGCTAAGAAA

>989_Sample_ASV43

CACCGCGGTTATACGAGAGACCCTAATTGATAGGCACGGCGTAAAGAGTGGTTTAGGGTA

ACCCATAAATAAAGCTAAAGACCTTCTAAGCCGTCACACGCACACCGAAGGCACGAGACC

CCAACACGAAAGTAGCTTTACTTCTACCCCTGACGCCACGAAAGCTAAGAAA

>989_Sample_ASV44

CACCGCGGTTATACGAAAGACCCTAGTTGATGGATCACGGCGTAAAGGGTGGTTAAGGGA

TATAAAAATAAAGCTAAAAATCCTCTTAGCTGTCATACGCTTTCCGAAGACATGAGGCCC

AATTACGAAAGTAGCTTTAAACCACCCTGACACCACGAAAGCCAAGAAA

>989_Sample_ASV45

CACCGCGGTTATACGAAAGACCCTAGTTGATGGATCACGGCGTAAAGGGTGGTTAAGGGA

TATAAAAATAAAGCTAAAAATCCTCTTAGCTGTCATACGCTTTCCGAAGACATGAGGCCC

AATTACGAAAGTAGCTTTAAACCACCCTGACACCACGAAAGCTGTGAAA

>989_Sample_ASV46

CACCGCGGTTATACGAGAGGCCCAAGTTGATGGATCACGGCGTAAAGGGTGGTTAAGGGA

TATAAAAATAAAGCTAAAAATCCTCTTAGCTGTCATACGCTTTCCGAAGACATGAGGCCC

AATTACGAAAGTAGCTTTAAACCACCCTGACACCACGAAAGCTGTGAAA

>989_Sample_ASV47

CACCGCGGTTATACGAAAGACCCTAGTTGATGGATCACGGCGTAAAGGGTGGTTAAGGGA

TATAAAAATAAAGCTAAAAATCCTCTTAGCTGTCATACGCTTTCCGAAGACATGAGGCCC

AATTACGAAAGTAGCTTTAAACCACCCTGACCCCACGAAAGCTGTGAAA

>989_Sample_ASV48

CACCGCGGTTATACGAAAGACCCCAGTTTATAGATGCGGCGTAAAGGGTGGTTAAGAGAA

AATCAAAATAAGGCCAAAGATCCCCTAAGCAGTCATACGCTCCACGAGGACACGAAACCC

AAATACGAAAGTTGCCTTAATAAACTTCACCTGATCCCACGAAAGCTGAAAAA

>989_Sample_ASV49

CACCGCGGTTATACGAAAGGCTCTAGTTTATAAATACGGCGTAAAGGGTGGTTAAGGAAA

AATTAAAATAATGCTAAAGACCCCCTAAGCAGTTATACGCTTCACGGAGGCACGAAACCC

AAATACGAAAGTTGCCTTAATAAACTTTACCTGACCCCACGAAAGCTGAGAAA

>989_Sample_ASV50

CACCGCGGTTATACGAAAGGCTCTAGTTTATAAATACGGCGTAAAGGGTGGTTAAGGAAA

AATTAAAATAATGCTAAAGACCCCCTAAGCAGTTATACGCTTCACGGAGGCACGAAACCC

AAATACGAAAGTTGCCTTAATAAACTTTACCTGACCCCACGAAAGCTGAAAAA

>989_Sample_ASV51

CACCGCGGTTATACGAGAGACTCTAGTTGATACCAACGGCGTAAAGAGTGGTTTGGGATG

CCCCTTAAAATAAAGCCAAAGACCTCCCAAGCCGTCAAACGCACCCCGGAGGCACGAAGC

CCCAACGCGAAAGCAACTTTACCTCCCTCCCGACGCCACGAAAGCTAAGAAA

>989_Sample_ASV52

CACCGCGGTTATACGAGAGACCCTAGTTGATAGCTACGGCGTAAAGCGTGGTTAGGAGAA

CAAGATAAATAAAGTCAAACAATCTCTCGGCCGTTATACGTTATCTGAGAATATGAAGTC

CTACCACGAAAGTAACTTTAATTTTTCTGACCCCACGAAAACTGAGGAA

>989_Sample_ASV53

CACCGCGGTTATACGAGAGACCCTAGTTGATAGCTACGGCGTAAAGAGTGGTTTGGGACC

CTCACTAAAATAAAGCTAAAGACCTCCCAAGCCGTCAAACGCACTCCGGAGACACGAAGC

CCCAACACGAAAGTAGCTTTACCTTTTTCCCCGACGCCACGAAAGCTAAGAAA

>989_Sample_ASV54

CACCGCGGTTATACGAGAGACCCTAGTTGACAGCTACGGCGTAAAGAGTGGTTTGGGGCA

CCCCATAAAAATAAAGCCAAAGACCTCCCAAGCTGTCAAACGCACTCCGGAGGCACGAAA

CCCCAACACGAAAGTAGCTTTACCTTCACCCCGACGCCACGAAAGCTAAGAAA

>989_Sample_ASV55

CACCGCGGTTATACGAGAGGCCCTAGTTGATAACTACCGGCGTAAAGAGTGGTTATGGAA

AAATATTTAATAAAGCCGAACACCCCCTCAGCCGTCATACGCACCTGGGAGCACGAAGAC

CTACTGCGAAAGCAGCTTTAATTATGCCTGACCCCACGACAGCTAAGAAA

>989_Sample_ASV56

CACCGCGGTTATACGAAAGACCCTAGTTTATAGACACGGCGTAAAGGGTGGTTCGGGACT

AAACAAAATAAAGCCAAAGACCTCCTAAGCCGTCATACGCTCACGGAAGCACGAAGCCCA

AGTACGAAAGTAGCTTTACAAAACATGCCTGACTCCACGAAAGCTAGGAAA

>989_Sample_ASV57

CACCGCGGTTATACGAGAGGCCCAAGTTGATTATTACCGGCGTAAAGCGTGGTTAAGATT

TTTATAGGACTAAAGCCGAACCCCCTCAGAGCTGTTATACGCACCCGAAGGTTAGAAGTT

CAATCACGAAAGTGGCTTTACAACCCCGAACCCACGAAAGCTAAGATA

>989_Sample_ASV58

CACCGCGGTTATACGAAAGACCCTAGTTGATAGGCACGGCGTAAAGGGTGGTTAGGGATA

TATTAAAAATAAAGTTAAAGAGCCTCTAAGCTGTCGCACGCATCCCGAGAGCTCGAGACC

CAGACACGAAAGTAACTTTAAAATACCTCACCTGACCCCACGAAAGCTAAGAAA

>989_Sample_ASV59

CACCGCGGTTATACGAGAGGCCCAAGTTGATACTCCACGGCGTAAAGAGTGGTTAGAAAA

GACCTACTACTAAAGCCGAACGCCTTCAAAGCTGTTATACGCATCCGAAGGTGAGAAGCC

CACCCACGAAAGTGGCTTTACAACCTTGACTCCACGAAAGCTATGACA

>989_Sample_ASV60

CACCGCGGTTATACGAGAGACCCCAGTTGATAAACACGGCGTAAAGAGTGGTTAAGGGCA

CAAAACAAATAAAGTCAAACAACCTCTTAGCTGTTATACGCATTATGAGGATATGAAGCC

CCCCTACGAAAGTAACTTTACCAACCCCTGACCCCACGAAAGCTAAGAAA

>989_Sample_ASV61

CACCGCGGTTATACGAGAGACCCTAGTTGATATATACGGCGTAAAGAGTGGTTTGGAATA

ACCAAGTAATATAGCCAAAGACCTCCCAAGCCGTCACACGCACCCCGGAGGCACGAAGCC

CAAACACGAAAGTAGCTTTATTAACTTACCGACGCCACGAAAGCTAAGAAA

>989_Sample_ASV62

CACCGCGGTTATACGAAAGACCCTAGTTTATAGGCACGGCGTAAAGGGTGGTTAGGGGAT

AAACAAAATAAAGCCAAAGACCTTCTAAGTCGTCATACACTCATGAAGGCACGAAGCCCA

AATACGAAAGTAACTTTACAACTTAGACCCGACTCCACGAAAGCTGGGGGA

>989_Sample_ASV63

CACCGCGGTTATACGAGAGACTCCAGTTGATAGCTACGGCGTAAAGAGTGGTTTGGGGCC

CCACCCAAAATAAAGCCAAAGACCTCCCAAGCCGTCAAACGCACCCCGGAGGCACGAAGT

CCTAACGCGAAAGCAACTTTACCTCCCCCGACGCCACGAAAGCTAAGAAA

>989_Sample_ASV64

CACCGCGGTTATACGAGAGGCCCAAGTTGATAGACATCGGCGTAAAGAGTGGTTAAGGAA

GTCTCAAAACTAAAGCCGAATGCCTTCAAGACTGTTATACGTTTCCGAAGGTAAGAAGCC

CCACTACGAAAGTGGCTTTACTCCACCTGACTCCACGAAAGCTGTGAAA

>989_Sample_ASV65

CACCGCGGTTATACGAAAGACCCTAGTTGATAGCTACGGCGTAAAGGGTGGTTAAGGTAT

ATAACAAATAAAGCTAAAGAGCCTCCAAGCCGTCGTACGCATTCTGAGAGCTCGAAACCC

TAACACGAAAGTAGCTTTAAAAAATAACACCTGACCCCACGAAAGCTAAGAAA

>989_Sample_ASV66

CACCGCGGTTATACGAAAGACCCTAGTTGATAGATCACGGCGTAAAGGGTGGTTAAGGAA

AACAAAAATAAAGCTAAAAATCCTCTAAGCTGTCATACGCTTTCCGAAGACATGAGATCC

AACCACGAAAGTAGCTTTAAAATTTCCTGACACCACGAAAGCCAAGGAA

>989_Sample_ASV67

CACCGCGGTTATACGAAAGACCCTAGTTGATAGATCACGGCGTAAAGGGTGGTTAAGGAG

AACAAAAATAAAGCTAAAAATCTTCTAAGCTGTCATACGCTTTCCGAAGACATGAGACCC

AACCACGAAAGTAGCTTTAAACTTTCCTGACACCACGAAAGCCAAGAAA

>1002_Sample_ASV1

CACCGCGGTTATACGAAAGACCCTAGTTGATGGGTGCGGCGTAAAGGGTGGTTAGGGACT

ACAAAAAATAGAGCTAAAGATCCTCTAGGTCGTCATACACATCACGAGGAAACGAAGCCC

AAATACGAAAGTAGCTCTAAATATAATCCTGACGCCACGAAAGCCAAGAAA

>1002_Sample_ASV2

CACCGCGGTTAAACGAGAGGCCCTAGTTGATGGTGCAACGGCGTAAAGGGTGGTTAAGGA

TAGCGAAATAATAAAGTCAAATGGCCCTCTGGCTGTCATACGCTTCTAGGAGTCCGAAGC

CCAGTATACGAAAGTAGCTTTAAGAAAGTCCACCTGACCCCACGAAAACTGAGGAA

>1002_Sample_ASV3

CACCGCGGTTATACGAGAGACCCTAGTTGATTAAAGCGGCGTAAAGAGTGGTTATGGAAC

TATTCTTTTAAAGCAGAAAACCTCTCAAACTGTTATACGCACCCAGAGGTCTGAATCCCT

CACACGAAAGTGACTTTATTTATGCCTACCAGAACCCACGAAAGCTGGGACA

>1002_Sample_ASV4

CACCGCGGTTATACGAGAGACCCTAGTTGATTAAAGCGGCGTAAAGAGTGGTTATGGAAC

TATTCTTTTAAAGCAGAAAACCTCTCAAACTGTTATACGCACCCAGAGGTCTGAATCCCT

CACACGAAAGTGACTTTATTTATGCCTACCAGAACCCACGAAAGCTGGGGGA

>1002_Sample_ASV5

CACCGCGGTTATACGAGAGGCCCAAGTTGACAGACACCGGCGTAAAGAGTGGCTAGGGAA

AAATTACTACTAAAGCCGAACACCTTCAGAACTGTCATACGTTCCCGAAGATAAGAAGCC

CCACCACGAAAGTGACTTTATATTACCCGACCCCACGAAAGCTGTGAAA

>1002_Sample_ASV6

CACCGCGGTTATACGAGAGGCCCAAGTTGACAGACACCGGCGTAAAGAGTGGCTAGGGAA

AAATTACTACTAAAGCCGAACACCTTCAGAACTGTCATACGTTCCCGAAGATAAGAAGCC

CCACCACGAAAGTGACTTTATATTACCCGACCCCACGAAAGCTGGGGGA

>1002_Sample_ASV7

CACCGCGGTTATACGAGAGGCCCAAGTTGACAGACACCTGCGTAAAGAGTGGCTAGGGAA

AAATTACTACTAAAGCCGAACACCTTCAGAACTGTCATACGTTCCCGAAGATAAGAAGCC

CCACCACGAAAGTGGCTTTATATTACCCGACCCCACGAAAGCTATGACA

>1002_Sample_ASV8

CACCGCGGTTATACGAGAGGCCCAAGTTGACAGACACCGGCGTAAAGAGTGGCTAGGGAA

AAATTACTACTAAAGCCGAACACCTTCAGAACTGTCATACGTTCCCGAAGATAAGAAGCC

CCACCACGAAAGTGGCTTTATATTACCCGACCCCACGAAAGCTGGGGGA

>1002_Sample_ASV9

CACCGCGGTTATACGAGAGGCCCAAGTTGACAGACACCGGCGTAAAGAGTGGCTAGGGAA

AAATTACTACTAAAGCCGAACACCTTCAGAACTGTCATACGTTCCCGAAGATAAGAAGCC

CCACCACGAAAGTGGCTTTATATTACCCGACCCCACGAAAGCTAAGAAA

>1002_Sample_ASV10

CACCGCGGTTATACGAGAGGCCCAAGTTGACAGACACCGGCGTAAAGAGTGGCTAGGGAA

AAATTACTACTAAAGCCGAACACCCTCAGAACTGTCATACGTTCCCGAAGGTAAGAAGCC

CCACCACGAAAGTGGCTTTATAATACCCGACCCCACGAAAGCTGTGAAA

>1002_Sample_ASV11

CACCGCGGTTATACGAGAGGCCCAAGTTGACAGACACCGGCGTAAAGAGTGGCTAGGGAA

AAATTACTACTAAAGCCGAACACCTTCAGAACTGTCATACGTTCCCGAAGATAAGAAGCC

CCACCACGAAAGTGGCTTTATATTACCCGACCCCACGAAAGCTGTGAAA

>1002_Sample_ASV12

CACCGCGGTTATACGAGAGGCCCAAGTTGACAGACACCGGCGTAAAGAGTGGCTAAGGAA

AAATTTCTACTAAAGCCGAACACCTTCAGAACTGTCATACGTTCCCGAAGATAAGAAGCC

CCACCACGAAAGTGGCTTTATATTACCCGACACCACGAAAGCTGTGAAA

>1002_Sample_ASV13

CACCGCGGTTATACGAGAGGCCCAAGTTGACAGACACCGGCGTAAAGAGTGGCTAAGGAA

AAATTTCTACTAAAGCCGAACACCTTCAGAACTGTCATACGTTCCCGAAGAAAAGAAGCC

CCACCACGAAAGTGGCTTTATATTACCCGACCCCACGAAAGCTGTGAAA

>1002_Sample_ASV14

CACCGCGGTTAAACGAGAGACCCTAGTTGATCATCACGGCGTAAAGAGTGGTTAGGGGAT

TATCATAAATAAAGCCAAAGACCTCCCAAGCTGTCGCACGCATTCCGGAGGCGCGAAGCC

CACACACGAAAGTAGCTTTAACTATTGACCCTGATGCCACGAAAGCTAAGAAA

>1002_Sample_ASV15

CACCGCGGTTATACGAAAGACCCTAGTTGATAGATCACGGCGTAAAGGGTGGTTAGGGAA

TACAAAAATAAAGCTAAAAATCCTCTAAGCTGTCATACGCTTCCCGAAGACATGAAACCC

AACCACGAAAGTAGCTTTAAACTCTCCTGACACCACGAAAGCCAAGGAA

>1002_Sample_ASV16

CACCGCGGTTATACGAAAGACCCTAGTTTATAGGCACGGCGTAAAGGGTGGTTAGGGAAT

ACAAAAATAAAGCTAAAAATCCTCTAAGCTGTCATACGCTTCCCGAAGACATGAAACCCA

ACCACGAAAGTAGCTTTAAACTCTCCTGACACCACGAAAGCCAAGGAA

>1002_Sample_ASV17

CACCGCGGTTATACGAGAGGCCCAAGTTGATAAGCCCGGCGTAAAGAGTGGTTATGGAGA

ATAAAACACTAAAGCTAAAGACCCCCTAGGCTGTTATACGCACCTGGCGGCTCGAACCAC

CTATACGAAAGTAGCTTTACCCCCCTTCCACCAGAATCCACGACAGCTGGGGCA

>1002_Sample_ASV18

CACCGCGGTTATACGAGAGGCCCAAGTTGACAGACATCGGCGTAAAGAGTGGTTAAGGAA

ACCCCATTACTAAAGCCGAACGCCCTCAGAACTGTCATACGTTCCCGAAGGTAAGAAGCC

CCACTACGAAAGTGGCTTTAACCACACCCGACCCCACGAAAGCTGTGAAA

>1002_Sample_ASV19

CACCGCGGTTAAACGAGAGGCCCTAGTTGATATTACAACGGCGTAAAGGGTGGTTAAGGA

AAGCAAGCAATAAAGCCGAATGGCCCTTTGGCTGTCATACGCTTCTAGGTGTCCGAAGCC

CAATATACGAAAGTAGCTTTAGAAAAGCCCACCTGACACCACGAAAGCTGAGGAA

>1002_Sample_ASV20

CACCGCGGTTATACGAGAGGCCCCAGTTGATATTAGCGGCGTAAAGGGTGGTTAAGGACT

TTAATAACTAAAGCTAAACACTTCCCCGGCTGTTGTACGTAGACCGGAAGCTTGAAGCCC

AGACACGAAAGTAGCTTTATTCCCCCTGACCCCACGAAAGCTGAGAAA

>1002_Sample_ASV21

CACCGCGGTTATACGAGAGGCCCTAGTTGATAAATACGGCGTAAAGAGTGGTTAGAGGAC

TCCCATAATAAAGCCAAAGACCCTCCGGGCCGTCACACGCATACGAGGGCACGAAGTTCA

CACACGAAAGTAGCTTTAAATTACCCGACGCCACGAAAGCTAAGAAA

>1002_Sample_ASV22

CACCGCGGTTATACGAAAGACCCTAGTTTATAAGTACGGCGTAAAGGGTGGTTAGGGGAC

AAACAAAATAAAGCCAAAGACCTCCTAAGCCGTCATACGCTTACGGAGGCACGAAGCCCA

AACACGAAAGTAGCTTTACCGAACATGCCCGACTCCACGAAAGCTGGGAAA

>1002_Sample_ASV23

CACCGCGGTTATACGAAAGACCCTAGTTTATAAGTACGGCGTAAAGGGTGGTTAGGGGAC

AAACAAAATAAAGCCAAAGACCTCCTAAGCCGTCATACGCTTACGGAGGCACGAAGCCCA

AACACGAAAGTAGCTTTACCGAACATGCCCGACTCCACGAAAGCTAAGAAA

>1002_Sample_ASV24

CACCGCGGTTATACGAGAGACCCTAGTTGATAAATACGGCGTAAAGAGTGGTTATGGGAA

AAACAAATAAAGTCAAACAACCTCTTAGCTGTTATACGCATTATGAGAGCATGAAGCCCC

CTCACGAAAGTAACTTTAATACCTCCTGACCCCACGAAAGCTAAGAAA

>1002_Sample_ASV25

CACCGCGGTTATACGAGAGGCTCAAGTTGATAAACCCCGGCGTAAAGAGTGGTTAAGGAA

AAATAGAAACTAAAGCCGAATGCTTTCAAGACTGTTATACGTTCCCGAGAGTAAGAAGAC

CAATTACGAAAGTGGCTTTACTTTACCTGACCCCACGAAAGCTACGACA

>1002_Sample_ASV26

CACCGCGGTTATACGAGAGACCCTAATTGATAGACACGGCGTAAAGAGTGGTTTGGGAAA

GCCCAAAAATAAAGCTAAAGACCTTCTAAGCCGTCACACGCACGCCGAAGGCACGAAGCC

CTGACACGAAAGTAGCTTTACTATTACCCCCGACGCCACGAAAGCTAAGAAA

>1002_Sample_ASV27

AACCGCGGTTATACGAGAGACCCTAATTGATAGCTCACGGCGTAAAGGGTGGTTTGGGGA

AACCTCAAAATAAAGCTAAAGACCTTCTAAGCCGTTACACGCATACCGAAGGCACGAGGC

CCTAACACGAAAGTAGCTTTACTATTACCCCTGACGCCACGAAAGCTAAGAAA

>1002_Sample_ASV28

CACCGCGGTTATACGAAAGACCCTAGTTGATGGATCACGGCGTAAAGGGTGGTTAAGGGA

TATAAAAATAAAGCTAAAAATCCTCTTAGCTGTCATACGCTTTCCGAAGACATGAGGCCC

AATTACGAAAGTAGCTTTAAACCACCCTGACACCACGAAAGCCAAGAAA

>1002_Sample_ASV29

CACCGCGGTTATACGAAAGACCCTAGTTTATAGGCACGGCGTAAAGGGTGGTTAGGGGAT

AAACAAAATAAAGCTAAAGACTTCCAAAGCTGTTATACGCTAACGCAAGTACTAAACCCA

AATACGAAAGTAACTTTATTAAATGCCTGACTCCACGAAAGCTGAGAAA

>1002_Sample_ASV30

CACCGCGGTTATACGAAAGACCCTAGTCTATAGACACGGCGTAAAGGGTGGTTAAGGGAC

AAACAAAATAAAGCTAAAGACTTCCAAAGCTGTTATACGCTAACGCAAGTACTAAACCCA

AATACGAAAGTAACTTTATTAAATGCCTGACTCCACGAAAGCTGAGAAA

>1002_Sample_ASV31

CACCGCGGTTAAACGAGAGGCCCTAGTTGATAGTACAACGGCGTAAAGGGTGGTTAAGGA

TAGTGAAATAATAAAGTCGAATGGCCCTTTGGCTGTCATACGCTTCTAGGAGTCCGAAGC

CCAATATACGAAAGTAACTTTAAGAGCGCCCACCTGACCCCACGAAAACTGAGAAA

>1002_Sample_ASV32

CACCGCGGTTATACGAAAGACCCTAGTTGATAGGCACGGCGTAAAGGGTGGTTAGGGATA

TATTAAAAATAAAGTTAAAGAGCCTCTAAGCTGTCGCACGCATCCCGAGAGCTCGAGACC

CAGACACGAAAGTAACTTTAAAATACCTCACCTGACCCCACGAAAGCTAAGAAA

>1002_Sample_ASV33

CACCGCGGTTATACGAGAGGCCCAAGTTGATACTCCACGGCGTAAAGAGTGGTTAGAAAA

GACCTACTACTAAAGCCGAACGCCTTCAAAGCTGTTATACGCATCCGAAGGTGAGAAGCC

CACCCACGAAAGTGGCTTTACAACCTTGACTCCACGAAAGCTATGACA

>1002_Sample_ASV34

CACCGCGGTTATACGAGAGACCCCAGTTGATAAACACGGCGTAAAGAGTGGTTAAGGGCA

CAAAACAAATAAAGTCAAACAACCTCTTAGCTGTTATACGCATTATGAGGATATGAAGCC

CCCCTACGAAAGTAACTTTACCAACCCCTGACCCCACGAAAGCTAAGAAA

>1002_Sample_ASV35

CACCGCGGTTATACGAGAGACCCTAGTTGATATATACGGCGTAAAGAGTGGTTTGGAATA

ACCAAGTAATATAGCCAAAGACCTCCCAAGCCGTCACACGCACCCCGGAGGCACGAAGCC

CAAACACGAAAGTAGCTTTATTAACTTACCGACGCCACGAAAGCTAAGAAA

>1002_Sample_ASV36

CACCGCGGTTATACGAAAGACCCTAGTTTATAGGCACGGCGTAAAGGGTGGTTAGGGGAT

AAACAAAATAAAGCCAAAGACCTTCTAAGTCGTCATACACTCATGAAGGCACGAAGCCCA

AATACGAAAGTAACTTTACAACTTAGACCCGACTCCACGAAAGCTGGGGGA

>1002_Sample_ASV37

CACCGCGGTTATACGAGAGGCCCAAGTTGATAGACATCGGCGTAAAGAGTGGTTAAGGAA

GTCTCAAAACTAAAGCCGAATGCCTTCAAGACTGTTATACGTTTCCGAAGGTAAGAAGCC

CCACTACGAAAGTGGCTTTACTCCACCTGACTCCACGAAAGCTGTGAAA

>1002_Sample_ASV38

CACCGCGGTTATACGAGAGGCCCAAGTTGACAAACTCCGGCGTAAAGCGTGGTTAAGGCA

CACTCAAAAACTAAAGCCGAATATCTTCAGAGCAGTCATACGCATCCGAAGACACGAAGC

CCCACCACGAAAGTGGCTTTATGACACCTGACCCCACGAAAGCTAGGACA

>1002_Sample_ASV39

CACCGCGGTTATACGAAAGACCCTAGTTGATAGACCACGGCGTAAAGGGTGGTTAAGGAA

CACAAAAATAAAGCTAAAGATCCTCTAAGCTGTCATACGCTTCCCGAAGACATGAAACCC

AATCACGAAAGTAGCTTTAAACTGTCCTGACACCACGAAAGCCAAGAAA

>1002_Sample_ASV40

CACCGCGGTTATACGAGAGACCCTAATTGATAGACTACGGCGTAAAGAGTGGTTTAGAAC

AAAAATTAATAAAGCCAAAGATCCTCCTGGCCGTCGCACGCATTTCGAGGATACGAAGCC

CCACTACGAAAGTAGCTTTACTACAACTTTTCTGACCCCACGAAAGCTAAGAAA

>1005_Sample_ASV1

CACCACGGTTATACGAGAGGCCCAAGTTGAAAGACATCGGCGTAAAGAGTGGTTAAGGAA

AACCTTCTACTAAAGTCGAACGCTCTCAGGACTGTCGTACGTTTCCGAGAGTATGAAGCC

CCACCACGAAAGTGACTTTACCTCACCTGACTCCACGAAAGCTGCGAAA

>1005_Sample_ASV2

CACCGCGGTTATACGAGAGGCCCAAGTTGACAGACATCGGCGTAAAGAGTGGTTAAGGAA

AACCTTCAACTAAAGTCGAACGCTCTCAAGACTGTCGTACGTTTCCGAGAGTATGAAGCC

CCACCACGAAAGTGACTTTACCTCACCTGACTCCACGAAAGCTGCGAAA

>1005_Sample_ASV3

CACCGCGGTTATACGAAAGACCCTAGTTGATGGGTGCGGCGTAAAGGGTGGTTAGGGACT

ACAAAAAATAGAGCTAAAGATCCTCTAGGTCGTCATACACATCACGAGGAAACGAAGCCC

AAATACGAAAGTAGCTCTAAATATAATCCTGACGCCACGAAAGCCAAGAAA

>1005_Sample_ASV4

CACCGCGGTTATACGAGAGACCCTAGTTGATTAAAGCGGCGTAAAGAGTGGTTATGGAAC

TATTCTTTTAAAGCAGAAAACCTCTCAAACTGTTATACGCACCCAGAGGTCTGAATCCCT

CACACGAAAGTGACTTTATTTATGCCTACCAGAACCCACGAAAGCTGTGATA

>1005_Sample_ASV5

CACCGCGGTTATACGAGAGACCCTAGTTGATTAAAGCGGCGTAAAGAGTGGTTATGGAAC

TATTCTTTTAAAGCAGAAAACCTCTCAAACTGTTATACGCACCCAGAGGTCTGAATCCCT

CACACGAAAGTGACTTTATTTATGCCTACCAGAACCCACGAAAGCTGGGACA

>1005_Sample_ASV6

CACCGCGGTTATACGAAAGACCCTAGTTGATAGCCACGGCGTAAAGGGTGGTTAAGGAAC

CTAAAAAATAAAGCTAAAGACCCTCTAGGCCGTCATACGCATTACGAGGCAAACGAAACC

CCAACACGAAAGTAGCTTTAGACAAATATCCTGATGCCACGAAAGCCAAGAAA

>1005_Sample_ASV7

CACCGCGGTTATACGAGAGGCCCAAGTTGACAGACACCGGCGTAAAGAGTGGCTAGGGAA

AAATTACTACTAAAGCCGAACACCTTCAGAACTGTCATACGTTCCCGAAGATAAGAAGCC

CCACCACGAAAGTGACTTTATATTACCCGACCCCACGAAAGCTAAGAAA

>1005_Sample_ASV8

CACCGCGGTTATACGAGAGGCCCAAGTTGACAGACACCGGCGTAAAGAGTGGCTAGGGAA

AAATTACTACTAAAGCCGAACACCTTCAGAACTGTCATACGTTCCCGAAGATAAGAAGCC

CCACCACGAAAGTGGCTTTATATTACCCGACCCCACGAAAGCTGTGAAA

>1005_Sample_ASV9

CACCGCGGTTATACGAGATGGCCCAAGTTGATAGACACCGGCGTAAAGAGTGGCTAGGGA

AAAATTACTACTAAAGCCGAACACCTTCAGAACTGTCATACGTTCCCGAAGATAAGAAGC

CCCACCACGAAAGTGGCTTTATATTACCCGACCCCACGAAAGCTGTGATA

>1005_Sample_ASV10

CACCGCGGTTATACGAGAGGCCCAAGTTGACAGACACCGGCGTAAAGAGTGGCTAGGGAA

AAATTACTACTAAAGCCGAACACCTTCAGAACTGTCATACGTTCCCGAAGATAAGAAGCC

CCACCACGAAAGTGGCTTTATATTACCCGACCCCACGAAAGCTGTGATA

>1005_Sample_ASV11

CACCGCGGTTATACGAGAGGCCCAAGTTGACAGACACCGGCGTAAAGAGTGGCTAAGGAA

AAATTTCTACTAAAGCCGAACACCTTCAGAACTGTCATACGTTCCCGAAGAAAAGAAGCC

CCACCACGAAAGTGGCTTTATATTACCCGACCCCACGAAAGCTGTGATA

>1005_Sample_ASV12

CACCGCGGTTATACGAGAGGCCCAAGTTGACAGACACCGGCGTAAAGAGTGGCTAAGGAA

AAATTTCTACTAAAGCCGAACACCTTCAGAACTGTCATACGTTCCCGAAGAAAAGAAGCC

CCACCACGAAAGTGGCTTTATATTACCCGACCCCACGAAAGCTGTGAAA

>1005_Sample_ASV13

CACCGCGGTTATACGAGAGGCCCAAGTTGATAAGCCCGGCGTAAAGAGTGGTTATGGAGA

ATAAAACACTAAAGCTAAAGACCCCCTAGGCTGTTATACGCACCTGGCGGCTCGAACCAC

CTATACGAAAGTAGCTTTACCCCCCTTCCACCAGAATCCACGAAAGCTGTGATA

>1005_Sample_ASV14

CACCGCGGTTATACGAGAGGCCCAAGTTGATAAGCCCGGCGTAAAGAGTGGTTATGGAGA

ATAAAACACTAAAGCTAAAGACCCCCTAGGCTGTTATACGCACCTGGCGGCTCGAACCAC

CTATACGAAAGTAGCTTTACCCCCCTTCCACCAGAATCCACGACAGCTGTGATA

>1005_Sample_ASV15

CACCGCGGTTATACGAGAGGCCCAAGTTGATAAGCCCGGCGTAAAGAGTGGTTATGGAGA

ATAAAACACTAAAGCTAAAGACCCCCTAGGCTGTTATACGCACCTGGCGGCTCGAACCAC

CTATACGAAAGTAGCTTTACCCCCCTTCCACCAGAATCCACGACAGCTGGGGCA

>1005_Sample_ASV16

CACCGCGGTTATACGAGAGGCCCAAGTTGACAGACATCGGCGTAAAGAGTGGTTAAGGAA

ACCCCATTACTAAAGCCGAACGCCCTCAGAACTGTCATACGTTCCCGAAGGTAAGAAGCC

CCACTACGAAAGTGGCTTTAACCACACCCGACCCCACGAAAGCTGTGAAA

>1005_Sample_ASV17

CACCGCGGTTATACGAGAGACCCCAGTTGATAGCTACGGCGTAAAGAGTGGTTAAGGACC

CCATTTAATAAAGCCAAAGACCCCCCAAGCCGTCATACGCACACGAGGGCGCGAAGCTCG

CACACGAAAGTAGCTCTAATTTACCCGACGCCACGAAAGCTAAGGAA

>1005_Sample_ASV18

CACCGCGGTTATACGAGAAACCCTAGTTGACAAATACGGCGTAAAGAGTGGTTAAGGGGT

ATCAAAAATAAAGTCAAACATTTTCCTAGTTGTTATAAGCATTTCGAGAACATGAAGCCC

AACCACGAAAGTAACTTTACCTTCACCTGACCCCACGAAAGCTAAGAAA

>1005_Sample_ASV19

CACCGCGGTTATACGAGAGACCCTAGTTGATAAATACGGCGTAAAGAGTGGTTATGGGAA

AAACAAATAAAGTCAAACAACCTCTTAGCTGTTATACGCATTATGAGAGCATGAAGCCCC

CTCACGAAAGTAACTTTAATACCTCCTGACCCCACGAAAGCTAAGAAA

>1005_Sample_ASV20

CACCGCGGTTATACGAGAGACCCTAATTGATAGACACGGCGTAAAGAGTGGTTTGGGAAA

GCCCAAAAATAAAGCTAAAGACCTTCTAAGCCGTCACACGCACGCCGAAGGCACGAAGCC

CTGACACGAAAGTAGCTTTACTATTACCCCCGACGCCACGAAAGCTAAGAAA

>1005_Sample_ASV21

AACCGCGGTTATACGAGAGACCCTAATTGATAGCTCACGGCGTAAAGGGTGGTTTGGGGA

AACCTCAAAATAAAGCTAAAGACCTTCTAAGCCGTTACACGCATACCGAAGGCACGAGGC

CCTAACACGAAAGTAGCTTTACTATTACCCCTGACGCCACGAAAGCTAAGAAA

>1005_Sample_ASV22

CACCGCGGTTATACGAAAGACCCTAGTTTATAGATGCGGCGTAAAGGGTGGTTAAGGAAA

ACCTAAAATAATGCTAAAGACTCTCAAAGCTGTTATACGCTTCATGAGCACACGAAATCC

AAATACGAAAGTTGCCTTAATAAACTTTGCCTGACCCCACGAAAGCTGAAAAA

>1005_Sample_ASV23

CACCGCGGTTATACGAGAGACCCTAGTTGATAGCTACGGCGTAAAGCGTGGTTAGGAGAA

CAAGATAAATAAAGTCAAACAATCTCTCGGCCGTTATACGTTATCTGAGAATATGAAGTC

CTACCACGAAAGTAACTTTAATTTTTCTGACCCCACGAAAACTGAGGAA

>1005_Sample_ASV24

CACCGCGGTTAAACGAGAGGCCCTAGTTGATAGTACAACGGCGTAAAGGGTGGTTAAGGA

TAGTGAAATAATAAAGTCGAATGGCCCTTTGGCTGTCATACGCTTCTAGGAGTCCGAAGC

CCAATATACGAAAGTAACTTTAAGAGCGCCCACCTGACCCCACGAAAACTGAGAAA

>1005_Sample_ASV25

CACCGCGGTTATACGAAAGACCCTAGTTGATAGGCACGGCGTAAAGGGTGGTTAGGGATA

TATTAAAAATAAAGTTAAAGAGCCTCTAAGCTGTCGCACGCATCCCGAGAGCTCGAGACC

CAGACACGAAAGTAACTTTAAAATACCTCACCTGACCCCACGAAAGCTAAGAAA

>1005_Sample_ASV26

CACCGCGGTTATACGAGAGGCCCAAGTTGATACTCCACGGCGTAAAGAGTGGTTAGAAAA

GACCTACTACTAAAGCCGAACGCCTTCAAAGCTGTTATACGCATCCGAAGGTGAGAAGCC

CACCCACGAAAGTGGCTTTACAACCTTGACTCCACGAAAGCTGTGATA

>1005_Sample_ASV27

CACCGCGGTTATACGAGAGGCCCAAGTTGATACTCCACGGCGTAAAGAGTGGTTAGAAAA

GACCTACTACTAAAGCCGAACGCCTTCAAAGCTGTTATACGCATCCGAAGGTGAGAAGCC

CACCCACGAAAGTGGCTTTACAACCTTGACTCCACGAAAGCTATGACA

>1005_Sample_ASV28

CACCGCGGTTATACGAGAGACCCCAGTTGATAAACACGGCGTAAAGAGTGGTTAAGGGCA

CAAAACAAATAAAGTCAAACAACCTCTTAGCTGTTATACGCATTATGAGGATATGAAGCC

CCCCTACGAAAGTAACTTTACCAACCCCTGACCCCACGAAAGCTAAGAAA

>1005_Sample_ASV29

CACCGCGGTTATACGAGAGACCCTAGTTGATATATACGGCGTAAAGAGTGGTTTGGAATA

ACCAAGTAATATAGCCAAAGACCTCCCAAGCCGTCACACGCACCCCGGAGGCACGAAGCC

CAAACACGAAAGTAGCTTTATTAACTTACCGACGCCACGAAAGCTAAGAAA

>1005_Sample_ASV30

CACCGCGGTTATACGAAAGACCCTAGTTTATAGGCACGGCGTAAAGGGTGGTTAGGGGAT

AAACAAAATAAAGCCAAAGACCTTCTAAGTCGTCATACACTCATGAAGGCACGAAGCCCA

AATACGAAAGTAACTTTACAACTTAGACCCGACTCCACGAAAGCTGGGGGA

>1005_Sample_ASV31

CACCGCGGTTATACGAGAGACCCTAATTGATAGACTACGGCGTAAAGAGTGGTTTAGAAC

AAAAATTAATAAAGCCAAAGATCCTCCTGGCCGTCGCACGCATTTCGAGGATACGAAGCC

CCACTACGAAAGTAGCTTTACTACAACTTTTCTGACCCCACGAAAGCTAAGAAA

>1005_Sample_ASV32

CACCGCGGTTATACGAAAGACCCTAGTTGATAGATCACGGCGTAAAGGGTGGTTAAGGAG

AACAAAAATAAAGCTAAAAATCCTCTAAGCTGTCATACGCTTTCCGAAGACATGAGACCC

AACCACGAAAGTAACTTTAAACTTTCCTGACACCACGAAAGCCAAGAAA

>1102_Sample_ASV1

CACCGCGGTTATACGAAAGACCCTAGTTGATGGGTGCGGCGTAAAGGGTGGTTAGGGACT

ACAAAAAATAGAGCTAAAGATCCTCTAGGTCGTCATACACATCACGAGGAAACGAAGCCC

AAATACGAAAGTAGCTCTAAATATAATCCTGACGCCACGAAAGCCAAGAAA

>1102_Sample_ASV2

CACCGCGGTTATACGAGAGACCCTAGTTGATTAAAGCGGCGTAAAGAGTGGTTATGGAAC

TATTCTTTTAAAGCAGAAAACCTCTCAAACTGTTATACGCACCCAGAGGTCTGAATCCCT

CACACGAAAGTGACTTTATTTATGCCTACCAGAACCCACGAAAGCTGGGACA

>1102_Sample_ASV3

CACCGCGGTTATACGAAAGACCCTAGTTGATAGCCACGGCGTAAAGGGTGGTTAAGGAAC

CTAAAAAATAAAGCTAAAGACCCTCTAGGCCGTCATACGCATTACGAGGCAAACGAAACC

CCAACACGAAAGTAGCTTTAGACAAATATCCTGATGCCACGAAAGCCAAGAAA

>1102_Sample_ASV4

CACCGCGGTTATACGAGAGGCCCAAGTTGACAGACACCGGCGTAAAGAGTGGCTAGGGAA

AAATTACTACTAAAGCCGAACACCTTCAGAACTGTCATACGTTCCCGAAGATAAGAAGCC

CCACCACGAAAGTGACTTTATATTACCCGACCCCACGAAAGCTAAGGAA

>1102_Sample_ASV5

CACCGCGGTTATACGAGAGGCCCAAGTTGACAGACACCGGCGTAAAGAGTGGCTAGGGAA

AAATTACTACTAAAGCCGAACACCTTCAGAACTGTCATACGTTCCCGAAGATAAGAAGCC

CCACCACGAAAGTGACTTTATATTACCCGACCCCACGAAAGCTGTGAAA

>1102_Sample_ASV6

CACCGCGGTTATACGAGAGGCCCAAGTTGACAGACACCGGCGTAAAGAGTGGCTAGGGAA

AAATTACTACTAAAGCCGAACACCCTCAGAACTGTCATACGTTCCCGAAGGTAAGAAGCC

CCACCACGAAAGTGGCTTTATAATACCCGACCCCACGAAAGCTGTGAAA

>1102_Sample_ASV7

CACCGCGGTTATACGAGAGGCCCAAGTTGACAGACACCGGCGTAAAGAGTGGCTAAGGAA

AAATTTCTACTAAAGCCGAACACCTTCAGAACTGTCATACGTTCCCGAAGATAAGAAGCC

CCACCACGAAAGTGGCTTTATATTACCCGACACCACGAAAGCTGTGAAA

>1102_Sample_ASV8

CACCGCGGTTATACGAGAGGCCCAAGTTGACAGACACCGGCGTAAAGAGTGGCTAAGGAA

AAATTTCTACTAAAGCCGAACACCTTCAGAACTGTCATACGTTCCCGAAGAAAAGAAGCC

CCACCACGAAAGTGGCTTTATATTACCCGACCCCACGAAAGCTGTGAAA

>1102_Sample_ASV9

CACCGCGGTTATACGAAAGACTCTAGTTGACAAACACGGCGTAAAGGGTGGTTAGGATAA

CAACAAAATAAAGCTAAAGACTTACCAAGCCGTCATACGCCCATGTAAGAGCGAAAACCA

CAAACGAAAGTAACTTTATTACACCATCCGAATCCACGAAAGCTAAGAAA

>1102_Sample_ASV10

CACCGCGGTTAAACGAGAGACCCTAGTTGATCATCACGGCGTAAAGAGTGGTTAGGGGAT

TATCATAAATAAAGCCAAAGACCTCCCAAGCTGTCGCACGCATTCCGGAGGCGCGAAGCC

CACACACGAAAGTAGCTTTAACTATTGACCCTGATGCCACGAAAGCTAAGAAA

>1102_Sample_ASV11

CACCGCGGTTATACGAGAGACCCTAGTTGATATATACGGCGTAAAGAGTGGTTTGGGATA

TTTCTCCAAAATAAAGTCGAAGATCTCCCAAGCCGTCACACGCATCCCGGAGACACGAAG

CCCTGATACGAAGGTAGCTTTATTAATTCCCCGACGCCACGAAAGCTAAGAAA

>1102_Sample_ASV12

CACCGCGGTTATACGAGAGACCCTAGTTGATATATACGGCGTAAAGAGTGGTTTGGGATA

TTTCTCCAAAATAAAGCCGAAGATCTCCCAAGCCGTCACACGCATCCCGGAGACACGAAG

CCCTGATACGAAGGTAGCTTTATTAACACCCCGACGCCACGAAAGCTAAGAAA

>1102_Sample_ASV13

CACCGCGGTTATACGAGAGACCCTAGTTGATATATACGGCGTAAAGAGTGGTTTGGGATA

TTTCTCCAAAATAAAGTCGAAGATCTCCCAAGCCGTCACACGCATCCCGGAGACACGAAG

CCCTGATACGAAGGTAGCTTTATTAACACCCCGACGCCACGAAAGCTAAGAAA

>1102_Sample_ASV14

CACCGCGGTTATACGAAAGACCCTAGTTGATAGATCACGGCGTAAAGGGTGGTTAAGGAA

AACAAAAATAAAGCTAAAAATCCTCTAAGCTGTCATACGCTTCCCGAAGACATGAAACCC

AACCACGAAAGTAGCTTTAAACTCTCCTGACACCACGAAAGCCAAGGAA

>1102_Sample_ASV15

CACCGCGGTTATACGAAAGACCCTAGTTGATAGATCACGGCGTAAAGGGTGGTTAGGGAA

TACAAAAATAAAGCTAAAAATCCTCTAAGCTGTCATACGCTTCCCGAAGACATGAAACCC

AACCACGAAAGTAGCTTTAAACTCTCCTGACACCACGAAAGCCAAGGAA

>1102_Sample_ASV16

CACCGCGGTTATACGAGAGGCCCAAGTTGATAAGCCCGGCGTAAAGAGTGGTTATGGAGA

ATAAAACACTAAAGCTAAAGACCCCCTAGGCTGTTATACGCACCTGGCGGCTCGAACCAC

CTATACGAAAGTAGCTTTACCCCCCTTCCACCAGAATCCACGACAGCTGGGGCA

>1102_Sample_ASV17

CACCGCGGTTATACGAGAGACCCCAGTTGATAGCTACGGCGTAAAGAGTGGTTATGGAGA

ATAAAACACTAAAGCTAAAGACCCCCTAGGCTGTTATACGCACCTGGCGGCTCGAACCAC

CTATACGAAAGTAGCTTTACCCCCCTTCCACCAGAATCCACGACAGCTGGGGCA

>1102_Sample_ASV18

CACCGCGGTTATACGAGAGGCCCAAGTTGATAAGCCCGGCGTAAAGAGTGGTTATGGAGA

ATAAAACACTAAAGCTAAAGACCCCCTAGGCTGTTATACGCACCTGGCGGCTCGAACCAC

CTATACGAAAGTAGCTTTACCCCCCTTCCACCAGAATCCACGAAAGCTAAGGAA

>1102_Sample_ASV19

CACCGCGGTTATACGAGAGGCCCAAGTTGACAGACATCGGCGTAAAGAGTGGTTAAGGAA

ACCCCATTACTAAAGCCGAACGCCCTCAGAACTGTCATACGTTCCCGAAGGTAAGAAGCC

CCACTACGAAAGTGGCTTTAACCACACCCGACCCCACGAAAGCTGTGAAA

>1102_Sample_ASV20

CACCGCGGTCACACGATTAACCCAAGTCAATAGAAGCCGGCGTAAAGAGTGTTTTAGATC

ACCCCCTCCCCAATAAAGCTAAAACTCACCTGAGTTGTAAAAAACTCCAGTTGACACAAA

ATAGACTACGAAAGTGGCTTTAACATATCTGAACACACAATAGCTAAGACC

>1102_Sample_ASV21

CACCGCGGTTATACGAGAGACCCCAGTTGATAGCTACGGCGTAAAGAGTGGTTAAGGACC

CCATTTAATAAAGCCAAAGACCCCCCAAGCCGTCATACGCACACGAGGGCGCGAAGCTCG

CACACGAAAGTAGCTCTAATTTACCCGACGCCACGAAAGCTAAGGAA

>1102_Sample_ASV22

GACCGCGGTTATACGAGAGACTCTAGTTGATAGATACGGCGTAAAGAGTGGTTTGGGATA

AACTATAAATAAAGCCAAAGACCTTCCAAGCTGTTAAACGCATACCGAAGGCACGAAGCC

CCAACACGAAAGTAGCTTTACTACTACCCCCGATGCCACGAAAGCTAAGACA

>1102_Sample_ASV23

CACCGCGGTTATACGAAAGACCCCAGTTTATAGACACGGCGTAAAGGGTGGTTATAGGAC

AAACTAACATAGAGCCAAAGACCCCCTAAGCCGTCATACGCTCACGAGGGCTCGAAGCCC

CTATACGAAAGTAGCTCTACCAAAAAATGCTCGACTCCACGAAAGCTGGGACA

>1102_Sample_ASV24

CACCGCGGTTATACGAGAGACCCTAGTTGATAAATACGGCGTAAAGAGTGGTTATGGGAA

AAACAAATAAAGTCAAACAACCTCTTAGCTGTTATACGCATTATGAGAGCATGAAGCCCC

CTCACGAAAGTAACTTTAATACCTCCTGACCCCACGAAAGCTAAGAAA

>1102_Sample_ASV25

CACCGCGGTTATACGAGAGACCCTAATTGATAGACACGGCGTAAAGAGTGGTTTGGGAAA

GCCCAAAAATAAAGCTAAAGACCTTCTAAGCCGTCACACGCACGCCGAAGGCACGAAGCC

CTGACACGAAAGTAGCTTTACTATTACCCCCGACGCCACGAAAGCTAAGAAA

>1102_Sample_ASV26

CACCGCGGTTATACGAAAGACCCTAGTTGATGGATCACGGCGTAAAGGGTGGTTAAGGGA

TATAAAAATAAAGCTAAAAATCCTCTTAGCTGTCATACGCTTTCCGAAGACATGAGGCCC

AATTACGAAAGTAGCTTTAAACCACCCTGACACCACGAAAGCCAAGAAA

>1102_Sample_ASV27

CACCGCGGTTATACGAAAGACCCTAGTTTATAGATGCGGCGTAAAGGGTGGTTAAGGAAA

ACCTAAAATAATGCTAAAGACTCTCAAAGCTGTTATACGCTTCATGAGCACACGAAATCC

AAATACGAAAGTTGCCTTAATAAACTTTGCCTGACCCCACGAAAGCTGAAAAA

>1102_Sample_ASV28

CACCGCGGTTATACGAGAGACCCTAGTTGATAGCTACGGCGTAAAGCGTGGTTAGGAGAA

CAAGATAAATAAAGTCAAACAATCTCTCGGCCGTTATACGTTATCTGAGAATATGAAGTC

CTACCACGAAAGTAACTTTAATTTTTCTGACCCCACGAAAACTGAGGAA

>1102_Sample_ASV29

CACCGCGGTTAAACGAGAGGCCCTAGTTGATAGTACAACGGCGTAAAGGGTGGTTAAGGA

TAGTGAAATAATAAAGTCGAATGGCCCTTTGGCTGTCATACGCTTCTAGGAGTCCGAAGC

CCAATATACGAAAGTAACTTTAAGAGCGCCCACCTGACCCCACGAAAACTGAGAAA

>1102_Sample_ASV30

CACCGCGGTTATACGAAAGACCCTAGTTTATAGACACGGCGTAAAGGGTGGTTCGGGACT

AAACAAAATAAAGCCAAAGACCTCCTAAGCCGTCATACGCTCACGGAAGCACGAAGCCCA

AGTACGAAAGTAGCTTTACAAAACATGCCTGACTCCACGAAAGCTAGGAAA

>1102_Sample_ASV31

CACCGCGGTTATACGAAAGACCCTAGTTGATAGGCACGGCGTAAAGGGTGGTTAGGGATA

TATTAAAAATAAAGTTAAAGAGCCTCTAAGCTGTCGCACGCATCCCGAGAGCTCGAGACC

CAGACACGAAAGTAACTTTAAAATACCTCACCTGACCCCACGAAAGCTAAGAAA

>1102_Sample_ASV32

CACCGCGGTTATACGAGAGGCCCAAGTTGATACTCCACGGCGTAAAGAGTGGTTAGAAAA

GACCTACTACTAAAGCCGAACGCCTTCAAAGCTGTTATACGCATCCGAAGGTGAGAAGCC

CACCCACGAAAGTGGCTTTACAACCTTGACTCCACGAAAGCTATGACA

>1102_Sample_ASV33

CACCGCGGTTATACGAGAGACCCCAGTTGATAAACACGGCGTAAAGAGTGGTTAAGGGCA

CAAAACAAATAAAGTCAAACAACCTCTTAGCTGTTATACGCATTATGAGGATATGAAGCC

CCCCTACGAAAGTAACTTTACCAACCCCTGACCCCACGAAAGCTAAGAAA

>1102_Sample_ASV34

CACCGCGGTTATACGAGAGACCCTAGTTGATATATACGGCGTAAAGAGTGGTTTGGAATA

ACCAAGTAATATAGCCAAAGACCTCCCAAGCCGTCACACGCACCCCGGAGGCACGAAGCC

CAAACACGAAAGTAGCTTTATTAACTTACCGACGCCACGAAAGCTAAGAAA

>1102_Sample_ASV35

CACCGCGGTTATACGAAAGACCCTAGTTTATAGGCACGGCGTAAAGGGTGGTTAGGGGAT

AAACAAAATAAAGCCAAAGACCTTCTAAGTCGTCATACACTCATGAAGGCACGAAGCCCA

AATACGAAAGTAACTTTACAACTTAGACCCGACTCCACGAAAGCTGGGGGA

>1102_Sample_ASV36

CACCGCGGTTATACGAGAGACCCTAATTGATAGACTACGGCGTAAAGAGTGGTTTAGAAC

AAAAATTAATAAAGCCAAAGATCCTCCTGGCCGTCGCACGCATTTCGAGGATACGAAGCC

CCACTACGAAAGTAGCTTTACTACAACTTTTCTGACCCCACGAAAGCTAAGAAA

>1102_Sample_ASV37

CACCGCGGTTATACGAAAGACCCTAGTTGATAGATCACGGCGTAAAGGGTGGTTAAGGAA

AACAAAAATAAAGCTAAAAATCCTCTAAGCTGTCATACGCTTTCCGAAGACATGAGATCC

AACCACGAAAGTAGCTTTAAAATTTCCTGACACCACGAAAGCCAAGGAA

>1102_Sample_ASV38

CACCGCGGTTATACGAAAGACCCTAGTTGATAGATCACGGCGTAAAGGGTGGTTAAGGAG

AACAAAAATAAAGCTAAAAATCCTCTAAGCTGTCATACGCTTTCCGAAGACATGAGACCC

AACCACGAAAGTAACTTTAAACTTTCCTGACACCACGAAAGCCAAGAAA

>1108_Sample_ASV1

TACCGCGGTTATACGAAAGACCCAAGTTAATAAACTACGGCGTAAAGCGTGGTTAGAGAC

CTCCAATAATTAAAGCCAAAGACCTTCTAAGCCGTCGCACGCCCCGCGAAGACACGAAGC

CCAAAAACGAAAGTAGCTTTATTCTTACTCGACCCCACGAAAGCTAGGGCA

>1108_Sample_ASV2

CACCGCGGTTATACGAAAGACCCTAGTTGATGGGTGCGGCGTAAAGGGTGGTTAGGGACT

ACAAAAAATAGAGCTAAAGATCCTCTAGGTCGTCATACACATCACGAGGAAACGAAGCCC

AAATACGAAAGTAGCTCTAAATATAATCCTGACGCCACGAAAGCCAAGAAA

>1108_Sample_ASV3

CACCGCGGTTATACGAGAGACCCTAGTTGATTAAAGCGGCGTAAAGAGTGGTTATGGAAC

TATTCTTTTAAAGCAGAAAACCTCTCAAACTGTTATACGCACCCAGAGGTCTGAATCCCT

CACACGAAAGTGACTTTATTTATGCCTACCAGAACCCACGAAAGCTGGGACA

>1108_Sample_ASV4

CACCGCGGTTATACGAGAGGCCCAAGTTGACAGACACCGGCGTAAAGAGTGGCTAGGGAA

AAATTACTACTAAAGCCGAACACCTTCAGAACTGTCATACGTTCCCGAAGATAAGAAGCC

CCACCACGAAAGTGACTTTATATTACCCGACCCCACGAAAGCTGTGAAA

>1108_Sample_ASV5

CACCGCGGTTATACGAGAGGCCCAAGTTGACAGACACCGGCGTAAAGAGTGGCTAGGGAA

AAATTACTACTAAAGCCGAACACCTTCAGAACTGTCATACGTTCCCGAAGATAAGAAGCC

CCACCACGAAAGTGGCTTTATATTACCCGACCCCACGAAAGCTGTGAAA

>1108_Sample_ASV6

CACCGCGGTTATACGAGAGGCCCAAGTTGACAGACACCGGCGTAAAGAGTGGCTAAGGAA

AAATTTCTACTAAAGCCGAACACCTTCAGAACTGTCATACGTTCCCGAAGAAAAGAAGCC

CCACCACGAAAGTGGCTTTATATTACCCGACCCCACGAAAGCTGTGAAA

>1108_Sample_ASV7

CACCGCGGTTATACGAAAGACTCTAGTTGACAAACACGGCGTAAAGGGTGGTTAGGATAA

CAACAAAATAAAGCTAAAGACTTACCAAGCCGTCATACGCCCATGTAAGAGCGAAAACCA

CAAACGAAAGTAACTTTATTACACCATCCGAATCCACGAAAGCTAAGAAA

>1108_Sample_ASV8

CACCGCGGTTAAACGAGAGACCCTAGTTGATCATCACGGCGTAAAGAGTGGTTAGGGGAT

TATCATAAATAAAGCCAAAGACCTCCCAAGCTGTCGCACGCATTCCGGAGGCGCGAAGCC

CACACACGAAAGTAGCTTTAACTATTGACCCTGATGCCACGAAAGCTAAGAAA

>1108_Sample_ASV9

CACCGCGGTTATACGAAAGACCCTAGTTGATAGATCACGGCGTAAAGGGTGGTTAGGGAA

TACAAAAATAAAGCTAAAAATCCTCTAAGCTGTCATACGCTTCCCGAAGACATGAAACCC

AACCACGAAAGTAGCTTTAAACTCTCCTGACACCACGAAAGCCAAGGAA

>1108_Sample_ASV10

CACCGCGGTTATACGAAAGACCCTAGTTTATAGGCACGGCGTAAAGGGTGGTTAGGGAAT

ACAAAAATAAAGCTAAAAATCCTCTAAGCTGTCATACGCTTCCCGAAGACATGAAACCCA

ACCACGAAAGTAGCTTTAAACTCTCCTGACACCACGAAAGCTGGGGGA

>1108_Sample_ASV11

CACCGCGGTTATACGAAAGACCCTAGTTGATAGATCACGGCGTAAAGGGTGGTTAGGGAA

TACAAAAATAAAGCTAAAAATCCTCTAAGCTGTCATACGCTTCCCGAAGACATGAAACCC

AACCACGAAAGTAGCTTTAAACTCTCCTGACTCCACGAAAGCTGGGGGA

>1108_Sample_ASV12

CACCGCGGTTATACGAAAGACCCTAGTTGATAGATCACGGCGTAAAGGGTGGTTAGGGAA

TACAAAAATAAAGCTAAAAATCCTCTAAGCTGTCATACGCTTCCCGAAGACATGAAACCC

AACCACGAAAGTAGCTTTAAACTCTCCTGACACCACGAAAGCTGGGGGA

>1108_Sample_ASV13

CACCGCGGTTATACGAAAGACCCTAGTTTATAGGCACGGCGTAAAGGGTGGTTAGGGAAT

ACAAAAATAAAGCTAAAAATCCTCTAAGCTGTCATACGCTTCCCGAAGACATGAAACCCA

ACCACGAAAGTAGCTTTAAACTCTCCTGACACCACGAAAGCCAAGGAA

>1108_Sample_ASV14

CACCGCGGTTATACGAGAGGCCCAAGTTGATAAGCCCGGCGTAAAGAGTGGTTATGGAGA

ATAAAACACTAAAGCTAAAGACCCCCTAGGCTGTTATACGCACCTGGCGGCTCGAACCAC

CTATACGAAAGTAGCTTTACCCCCCTTCCACCAGAATCCACGACAGCTGGGGCA

>1108_Sample_ASV15

CACCGCGGTTATACGAGAGGCCCAAGTTGACAGACATCGGCGTAAAGAGTGGTTAAGGAA

ACCCCATTACTAAAGCCGAACGCCCTCAGAACTGTCATACGTTCCCGAAGGTAAGAAGCC

CCACTACGAAAGTGGCTTTAACCACACCCGACCCCACGAAAGCTGTGAAA

>1108_Sample_ASV16

CACCGCGGTTATACGAGAGGCCCCAGTTGATATTAGCGGCGTAAAGGGTGGTTAAGGACT

TTAATAACTAAAGCCAAACACTTCCCCGGCTGTTGTACGTAGACCGGAAGCTTGAAGCCC

AGACACGAAAGTAGCTTTATTTCCCCTGACCCCACGAAAGCTGAGAAA

>1108_Sample_ASV17

CACCGCGGTTATACGAGAGGCCCCAGTTGATATTAGCGGCGTAAAGGGTGGTTAAGGACT

TTAATAACTAAAGCTAAACACTTCCCCGGCTGTTGTACGTAGACCGGAAGCTTGAAGCCC

AGACACGAAAGTAGCTTTATTTCCCCTGACCCCACGAAAGCTGAGAAA

>1108_Sample_ASV18

CACCGCGGTTATACGAGAGGCCCCAGTTGATATTAGCGGCGTAAAGGGTGGTTAAGGACT

TTAATAACTAAAGCTAAACACTTCCCCGGCTGTTGTACGTAGACCGGAAGCTTGAAGCCC

AGACACGAAAGTAGCTTTATTCCCCCTGACCCCACGAAAGCTGAGAAA

>1108_Sample_ASV19

CACCGCGGTTATACGAAAGACCCCAGTTTATAGACACGGCGTAAAGGGTGGTTATAGGAC

AAACTAACATAGAGCCAAAGACCCCCTAAGCCGTCATACGCTCACGAGGGCTCGAAGCCC

CTATACGAAAGTAGCTCTACCAAAAAATGCTCGACTCCACGAAAGCTGGGACA

>1108_Sample_ASV20

CACCGCGGTTATACGAGAGACCCTAATTGATAGACACGGCGTAAAGAGTGGTTTGGGAAA

GCCCAAAAATAAAGCTAAAGACCTTCTAAGCCGTCACACGCACGCCGAAGGCACGAAGCC

CTGACACGAAAGTAGCTTTACTATTACCCCCGACGCCACGAAAGCTAAGAAA

>1108_Sample_ASV21

CACCGCGGTTATACGAGAGACCCTAATTGATAGGCACGGCGTAAAGAGTGGTTTAGGGTA

ACCCATAAATAAAGCTAAAGACCTTCTAAGCCGTCACACGCACACCGAAGGCACGAGACC

CCAACACGAAAGTAGCTTTACTTCTACCCCTGACGCCACGAAAGCTAAGAAA

>1108_Sample_ASV22

CACCGCGGTTATACGAAAGACCCTAGTTGATGGATCACGGCGTAAAGGGTGGTTAAGGGA

TATAAAAATAAAGCTAAAAATCCTCTTAGCTGTCATACGCTTTCCGAAGACATGAGGCCC

AATTACGAAAGTAGCTTTAAACCACCCTGACACCACGAAAGCCAAGAAA

>1108_Sample_ASV23

CACCGCGGTTATACGAAAGACCCCAGTTTATAGATGCGGCGTAAAGGGTGGTTAAGAGAA

AATCAAAATAAGGCCAAAGATCCCCTAAGCAGTCATACGCTCCACGAGGACACGAAACCC

AAATACGAAAGTTGCCTTAATAAACTTCACCTGATCCCACGAAAGCTGAAAAA

>1108_Sample_ASV24

CACCGCGGTTATACGAAAGGCTCTAGTTTATAAATACGGCGTAAAGGGTGGTTAAGGAAA

AATTAAAATAATGCTAAAGACCCCCTAAGCAGTTATACGCTTCACGGAGGCACGAAACCC

AAATACGAAAGTTGCCTTAATAAACTTTACCTGACCCCACGAAAGCTGAGAAA

>1108_Sample_ASV25

CACCGCGGTTATACGAAAGGCTCTAGTTTATAAATACGGCGTAAAGGGTGGTTAAGGAAA

AATTAAAATAATGCTAAAGACCCCCTAAGCAGTTATACGCTTCACGGAGGCACGAAACCC

AAATACGAAAGTTGCCTTAATAAACTTTACCTGACCCCACGAAAGCTGAAAAA

>1108_Sample_ASV26

CACCGCGGTTATACGAGAGACTCTAGTTGATACCAACGGCGTAAAGAGTGGTTTGGGATG

CCCCTTAAAATAAAGCCAAAGACCTCCCAAGCCGTCAAACGCACCCCGGAGGCACGAAGC

CCCAACGCGAAAGCAACTTTACCTCCCTCCCGACGCCACGAAAGCTAAGAAA

>1108_Sample_ASV27

CACCGCGGTTATACGAGAGACCCTAGTTGATAGCTACGGCGTAAAGCGTGGTTAGGAGAA

CAAGATAAATAAAGTCAAACAATCTCTCGGCCGTTATACGTTATCTGAGAATATGAAGTC

CTACCACGAAAGTAACTTTAATTTTTCTGACCCCACGAAAACTGAGGAA

>1108_Sample_ASV28

CACCGCGGTTATACGAGAGGCCCTAGTTGATAACTACCGGCGTAAAGAGTGGTTATGGAA

AAATATTTAATAAAGCCGAACACCCCCTCAGCCGTCATACGCACCTGGGAGCACGAAGAC

CTACTGCGAAAGCAGCTTTAATTATGCCTGACCCCACGACAGCTAAGAAA

>1108_Sample_ASV29

CACCGCGGTTATACGAGAGGCCCAAGTTGATTATTACCGGCGTAAAGCGTGGTTAAGATT

TTTATAGGACTAAAGCCGAACCCCCTCAGAGCTGTTATACGCACCCGAAGGTTAGAAGTT

CAATCACGAAAGTGGCTTTACAACCCCGAACCCACGAAAGCTAAGATA

>1108_Sample_ASV30

CACCGCGGTTATACGAAAGACCCTAGTTGATAGGCACGGCGTAAAGGGTGGTTAGGGATA

TATTAAAAATAAAGTTAAAGAGCCTCTAAGCTGTCGCACGCATCCCGAGAGCTCGAGACC

CAGACACGAAAGTAACTTTAAAATACCTCACCTGACCCCACGAAAGCTAAGAAA

>1108_Sample_ASV31

CACCGCGGTTATACGAGAGGCCCAAGTTGATACTCCACGGCGTAAAGAGTGGTTAGAAAA

GACCTACTACTAAAGCCGAACGCCTTCAAAGCTGTTATACGCATCCGAAGGTGAGAAGCC

CACCCACGAAAGTGGCTTTACAACCTTGACTCCACGAAAGCTATGACA

>1108_Sample_ASV32

CACCGCGGTTATACGAGAGACCCCAGTTGATAAACACGGCGTAAAGAGTGGTTAAGGGCA

CAAAACAAATAAAGTCAAACAACCTCTTAGCTGTTATACGCATTATGAGGATATGAAGCC

CCCCTACGAAAGTAACTTTACCAACCCCTGACCCCACGAAAGCTAAGAAA

>1108_Sample_ASV33

CACCGCGGTTATACGAAAGACCCTAGTTTATAGGCACGGCGTAAAGGGTGGTTAGGGGAT

AAACAAAATAAAGCCAAAGACCTTCTAAGTCGTCATACACTCATGAAGGCACGAAGCCCA

AATACGAAAGTAACTTTACAACTTAGACCCGACTCCACGAAAGCTGGGGGA

>1108_Sample_ASV34

CACCGCGGTTATACGAAAGACCCTAGTTGATAGATCACGGCGTAAAGGGTGGTTAGGGGA

TAAACAAAATAAAGCCAAAGACCTTCTAAGTCGTCATACACTCATGAAGGCACGAAGCCC

AAATACGAAAGTAACTTTACAACTTAGACCCGACTCCACGAAAGCTGGGGGA

>1108_Sample_ASV35

CACCGCGGTTATACGAAAGACCCTAGTTTATAGGCACGGCGTAAAGGGTGGTTAGGGGAT

AAACAAAATAAAGCCAAAGACCTTCTAAGTCGTCATACACTCATGAAGGCACGAAGCCCA

AATACGAAAGTAACTTTACAACTTAGACCCGACTCCACGAAAGCCAAGGAA

>1108_Sample_ASV36

CACCGCGGTTATACGAAAGACCCTAGTTTATAGGCACGGCGTAAAGGGTGGTTAGGGGAT

AAACAAAATAAAGCCAAAGACCTTCTAAGTCGTCATACACTCATGAAGGCACGAAGCCCA

AATACGAAAGTAACTTTACAACTTAGACCCGACACCACGAAAGCCAAGGAA

>1108_Sample_ASV37

CACCGCGGTTATACGAAAGACCCTAGTTGATAGATCACGGCGTAAAGGGTGGTTAGGGGA

TAAACAAAATAAAGCCAAAGACCTTCTAAGTCGTCATACACTCATGAAGGCACGAAGCCC

AAATACGAAAGTAACTTTACAACTTAGACCCGACTCCACGAAAGCCAAGGAA

>1108_Sample_ASV38

CACCGCGGTTATACGAAAGACCCTAGTTGATAGCTACGGCGTAAAGGGTGGTTAAGGTAT

ATAACAAATAAAGCTAAAGAGCCTCCAAGCCGTCGTACGCATTCTGAGAGCTCGAAACCC

TAACACGAAAGTAGCTTTAAAAAATAACACCTGACCCCACGAAAGCTAAGAAA

>1108_Sample_ASV39

CACCGCGGTTATACGAAAGACCCTAGTTGATAGATCACGGCGTAAAGGGTGGTTAAGGAG

AACAAAAATAAAGCTAAAAATCTTCTAAGCTGTCATACGCTTTCCGAAGACATGAGACCC

AACCACGAAAGTAGCTTTAAACTTTCCTGACACCACGAAAGCCAAGAAA

>1109_Sample_ASV1

CACCACGGTTATACGAGAGGCCCAAGTTGAAAGACATCGGCGTAAAGAGTGGTTAAGGAA

AACCTTCTACTAAAGTCGAACGCTCTCAGGACTGTCGTACGTTTCCGAGAGTATGAAGCC

CCACCACGAAAGTGACTTTACCTCACCTGACTCCACGAAAGCTGCGAAA

>1109_Sample_ASV2

CACCGCGGTTATACGAGAGGCCCAAGTTGACAGACATCGGCGTAAAGAGTGGTTAAGGAA

AACCTTCAACTAAAGTCGAACGCTCTCAAGACTGTCGTACGTTTCCGAGAGTATGAAGCC

CCACCACGAAAGTGACTTTACCTCACCTGACTCCACGAAAGCTGCGAAA

>1109_Sample_ASV3

TACCGCGGTTATACGAAAGACCCAAGTTAATAAACTACGGCGTAAAGCGTGGTTAGAGAC

CTCCAATAATTAAAGCCAAAGACCTTCTAAGCCGTCGCACGCCCCGCGAAGACACGAAGC

CCAAAAACGAAAGTAGCTTTATTCTTACTCGACCCCACGAAAGCTAGGGCA

>1109_Sample_ASV4

CACCGCGGTTATACGAAAGACCCTAGTTGATGGGTGCGGCGTAAAGGGTGGTTAGGGACT

ACAAAAAATAGAGCTAAAGATCCTCTAGGTCGTCATACACATCACGAGGAAACGAAGCCC

AAATACGAAAGTAGCTCTAAATATAATCCTGACGCCACGAAAGCCAAGAAA

>1109_Sample_ASV5

CACCGCGGTTATACGAGAGACCCTAGTTGATTAAAGCGGCGTAAAGAGTGGTTATGGAAC

TATTCTTTTAAAGCAGAAAACCTCTCAAACTGTTATACGCACCCAGAGGTCTGAATCCCT

CACACGAAAGTGACTTTATTTATGCCTACCAGAACCCACGAAAGCTGGGACA

>1109_Sample_ASV6

CACCGCGGTTATACGAGAGACCCTAGTTGATTAAAGCGGCGTAAAGAGTGGTTATGGAAC

TATTCTTTTAAAGCAGAAAACCTCTCAAACTGTTATACGCACCCAGAGGTCTGAATCCCT

CACACGAAAGTGACTTTATTTATGCCTACCAGAACCCACGAAAGCTGTGAAA

>1109_Sample_ASV7

CACCGCGGTTATACGAGAGGCCCAAGTTGACAGACACCGGCGTAAAGAGTGGCTAGGGAA

AAATTACTACTAAAGCCGAACACCTTCAGAACTGTCATACGTTCCCGAAGATAAGAAGCC

CCACCACGAAAGTGACTTTATATTACCCGACCCCACGAAAGCTGTGAAA

>1109_Sample_ASV8

CACCGCGGTTATACGAGAGGCCCAAGTTGACAGACACCGGCGTAAAGAGTGGCTAGGGAA

AAATTACTACTAAAGCCGAACACCTTCAGAACTGTCATACGTTCCCGAAGATAAGAAGCC

CCACCACGAAAGTGGCTTTATATTACCCGACCCCACGAAAGCTGTGAAA

>1109_Sample_ASV9

CACCGCGATTATACGAGAGGCCCAAGTTGACAGACATCGGCGTAAAGAGTGGTTAAGGAA

AAATTACTACTAAAGCCGAACACCTTCAGAACTGTCATACGTTCCCGAAGATAAGAAGCC

CCACCACGAAAGTGGCTTTATATTACCCGACCCCACGAAAGCTGTGAAA

>1109_Sample_ASV10

CACCGCGGTTATACGAGAGGCCCAAGTTGACAGACACCGGCGTAAAGAGTGGCTAAGGAA

AAATTTCTACTAAAGCCGAACACCTTCAGAACTGTCATACGTTCCCGAAGAAAAGAAGCC

CCACCACGAAAGTGGCTTTATATTACCCGACCCCACGAAAGCTGTGAAA

>1109_Sample_ASV11

CACCGCGGTTATACGAAAGACTCTAGTTGACAAACACGGCGTAAAGGGTGGTTAGGATAA

CAACAAAATAAAGCTAAAGACTTACCAAGCCGTCATACGCCCATGTAAGAGCGAAAACCA

CAAACGAAAGTAACTTTATTACACCATCCGAATCCACGAAAGCTAAGAAA

>1109_Sample_ASV12

CACCGCGGTTATACGAAAGACCCTAGTTGATAGATCACGGCGTAAAGGGTGGTTAGGGAA

TACAAAAATAAAGCTAAAAATCCTCTAAGCTGTCATACGCTTCCCGAAGACATGAAACCC

AACCACGAAAGTAGCTTTAAACTCTCCTGACACCACGAAAGCCAAGGAA

>1109_Sample_ASV13

CACCGCGGTTATACGAAAGACCCTAGTTTATAGGCACGGCGTAAAGGGTGGTTAGGGAAT

ACAAAAATAAAGCTAAAAATCCTCTAAGCTGTCATACGCTTCCCGAAGACATGAAACCCA

ACCACGAAAGTAGCTTTAAACTCTCCTGACACCACGAAAGCCAAGGAA

>1109_Sample_ASV14

CACCGCGGTTATACGAGAGGCCCAAGTTGATAAGCCCGGCGTAAAGAGTGGTTATGGAGA

ATAAAACACTAAAGCTAAAGACCCCCTAGGCTGTTATACGCACCTGGCGGCTCGAACCAC

CTATACGAAAGTAGCTTTACCCCCCTTCCACCAGAATCCACGACAGCTGGGGCA

>1109_Sample_ASV15

CACCGCGGTTATACGAGAGGCCCAAGTTGATAAGCCCGGCGTAAAGAGTGGTTATGGAGA

ATAAAACACTAAAGCTAAAGACCCCCTAGGCTGTTATACGCACCTGGCGGCTCGAACCAC

CTATACGAAAGTAGCTTTACCCCCCTTCCACCAGAATCCACGACAGCTGTGAAA

>1109_Sample_ASV16

CACCGCGGTTATACGAGAGGCCCAAGTTGACAGACATCGGCGTAAAGAGTGGTTAAGGAA

ACCCCATTACTAAAGCCGAACGCCCTCAGAACTGTCATACGTTCCCGAAGGTAAGAAGCC

CCACTACGAAAGTGGCTTTAACCACACCCGACCCCACGAAAGCTGTGAAA

>1109_Sample_ASV17

CACCGCGGTTATACGAGAGGCCCAAGTTGACAGACATCGGCGTAAAGAGTGGTTAAGGAA

ACCCCATTACTAAAGCCGAACGCCCTCAGAACTGTCATACGTTCCCGAAGGTAAGAAGCC

CCACTACGAAAGTGGCTTTACCCCCTGCCTGACGCCACGAAAGCTGTGAAA

>1109_Sample_ASV18

CACCGCGGTTATACGAGAGGCCCCAGTTGATATTAGCGGCGTAAAGGGTGGTTAAGGACT

TTAATAACTAAAGCCAAACACTTCCCCGGCTGTTGTACGTAGACCGGAAGCTTGAAGCCC

AGACACGAAAGTAGCTTTATTTCCCCTGACCCCACGAAAGCTGAGAAA

>1109_Sample_ASV19

CACCGCGGTTATACGAGAGGCCCCAGTTGATATTAGCGGCGTAAAGGGTGGTTAAGGACT

TTAATAACTAAAGCTAAACACTTCCCCGGCTGTTGTACGTAGACCGGAAGCTTGAAGCCC

AGACACGAAAGTAGCTTTATTTCCCCTGACCCCACGAAAGCTGAGAAA

>1109_Sample_ASV20

CACCGCGGTTATACGAGAGGCCCCAGTTGATATTAGCGGCGTAAAGGGTGGTTAAGGACT

TTAATAACTAAAGCTAAACACTTCCCCGGCTGTTGTACGTAGACCGGAAGCTTGAAGCCC

AGACACGAAAGTAGCTTTATTCCCCCTGACCCCACGAAAGCTGAGAAA

>1109_Sample_ASV21

CACCGCGGTTATACGAAAGACCCCAGTTTATAGACACGGCGTAAAGGGTGGTTATAGGAC

AAACTAACATAGAGCCAAAGACCCCCTAAGCCGTCATACGCTCACGAGGGCTCGAAGCCC

CTATACGAAAGTAGCTCTACCAAAAAATGCTCGACTCCACGAAAGCTGGGACA

>1109_Sample_ASV22

CACCGCGGTTATACGAGAGACCCTAATTGATAGGCACGGCGTAAAGAGTGGTTTAGGGTA

ACCCATAAATAAAGCTAAAGACCTTCTAAGCCGTCACACGCACACCGAAGGCACGAGACC

CCAACACGAAAGTAGCTTTACTTCTACCCCTGACGCCACGAAAGCTAAGAAA

>1109_Sample_ASV23

CACCGCGGTTATACGAAAGACCCTAGTTGATGGATCACGGCGTAAAGGGTGGTTAAGGGA

TATAAAAATAAAGCTAAAAATCCTCTTAGCTGTCATACGCTTTCCGAAGACATGAGGCCC

AATTACGAAAGTAGCTTTAAACCACCCTGACACCACGAAAGCCAAGAAA

>1109_Sample_ASV24

CACCGCGGTTATACGAAAGACCCCAGTTTATAGATGCGGCGTAAAGGGTGGTTAAGAGAA

AATCAAAATAAGGCCAAAGATCCCCTAAGCAGTCATACGCTCCACGAGGACACGAAACCC

AAATACGAAAGTTGCCTTAATAAACTTCACCTGATCCCACGAAAGCTGAAAAA

>1109_Sample_ASV25

CACCGCGGTTATACGAAAGGCTCTAGTTTATAAATACGGCGTAAAGGGTGGTTAAGGAAA

AATTAAAATAATGCTAAAGACCCCCTAAGCAGTTATACGCTTCACGGAGGCACGAAACCC

AAATACGAAAGTTGCCTTAATAAACTTTACCTGACCCCACGAAAGCTGAAAAA

>1109_Sample_ASV26

CACCGCGGTTATACGAGAGACTCTAGTTGATACCAACGGCGTAAAGAGTGGTTTGGGATG

CCCCTTAAAATAAAGCCAAAGACCTCCCAAGCCGTCAAACGCACCCCGGAGGCACGAAGC

CCCAACGCGAAAGCAACTTTACCTCCCTCCCGACGCCACGAAAGCTAAGAAA

>1109_Sample_ASV27

CACCGCGGTTATACGAGAGACCCTAGTTGATAGCTACGGCGTAAAGCGTGGTTAGGAGAA

CAAGATAAATAAAGTCAAACAATCTCTCGGCCGTTATACGTTATCTGAGAATATGAAGTC

CTACCACGAAAGTAACTTTAATTTTTCTGACCCCACGAAAACTGAGGAA

>1109_Sample_ASV28

CACCGCGGTTATACGAGAGGCCCTAGTTGATAACTACCGGCGTAAAGAGTGGTTATGGAA

AAATATTTAATAAAGCCGAACACCCCCTCAGCCGTCATACGCACCTGGGAGCACGAAGAC

CTACTGCGAAAGCAGCTTTAATTATGCCTGACCCCACGACAGCTAAGAAA

>1109_Sample_ASV29

CACCGCGGTTATACGAGAGGCCCAAGTTGATTATTACCGGCGTAAAGCGTGGTTAAGATT

TTTATAGGACTAAAGCCGAACCCCCTCAGAGCTGTTATACGCACCCGAAGGTTAGAAGTT

CAATCACGAAAGTGGCTTTACAACCCCGAACCCACGAAAGCTAAGATA

>1109_Sample_ASV30

CACCGCGGTTATACGAGAGGCCCAAGTTGATACTCCACGGCGTAAAGAGTGGTTAGAAAA

GACCTACTACTAAAGCCGAACGCCTTCAAAGCTGTTATACGCATCCGAAGGTGAGAAGCC

CACCCACGAAAGTGGCTTTACAACCTTGACTCCACGAAAGCTATGACA

>1109_Sample_ASV31

CACCGCGGTTATACGAGAGACCCCAGTTGATAAACACGGCGTAAAGAGTGGTTAAGGGCA

CAAAACAAATAAAGTCAAACAACCTCTTAGCTGTTATACGCATTATGAGGATATGAAGCC

CCCCTACGAAAGTAACTTTACCAACCCCTGACCCCACGAAAGCTAAGAAA

>1109_Sample_ASV32

CACCGCGGTTATACGAGAGACCCTAGTTGATATATACGGCGTAAAGAGTGGTTTGGAATA

ACCAAGTAATATAGCCAAAGACCTCCCAAGCCGTCACACGCACCCCGGAGGCACGAAGCC

CAAACACGAAAGTAGCTTTATTAACTTACCGACGCCACGAAAGCTAAGAAA

>1109_Sample_ASV33

CACCGCGGTTATACGAGAGACCCTAGTTGATATATACGGCGTAAAGAGTGGTTTGGAATA

ACCAAGTAATATAGCCAAAGACCTCCCAAGCCGTCACACGCACCCCGGAGGCACGAAGCC

CAAACACGAAAGTAGCTTTATTAACTTACCGACGCCACGAAAGCTGTGAAA

>1109_Sample_ASV34

CACCGCGGTTATACGAAAGACCCTAGTTTATAGGCACGGCGTAAAGGGTGGTTAGGGGAT

AAACAAAATAAAGCCAAAGACCTTCTAAGTCGTCATACACTCATGAAGGCACGAAGCCCA

AATACGAAAGTAACTTTACAACTTAGACCCGACTCCACGAAAGCTGGGGGA

>1109_Sample_ASV35

CACCGCGGTTATACGAAAGACCCTAGTTTATAGGCACGGCGTAAAGGGTGGTTAGGGGAT

AAACAAAATAAAGCCAAAGACCTTCTAAGTCGTCATACACTCATGAAGGCACGAAGCCCA

AATACGAAAGTAACTTTACAACTTAGACCCGACTCCACGAAAGCTGTGAAA

>1109_Sample_ASV36

CACCGCGGTTATACGAAAGACCCTAGTTTATAGGCACGGCGTAAAGGGTGGTTAGGGGAT

AAACAAAATAAAGCCAAAGACCTTCTAAGTCGTCATACACTCATGAAGGCACGAAGCCCA

AATACGAAAGTAACTTTACAACTTAGACCCGACCCCACGAAAGCTGTGAAA

>1109_Sample_ASV37

CACCGCGGTTATACGAAAGACCCTAGTTTATAGGCACGGCGTAAAGGGTGGTTAGGGGAT

AAACAAAATAAAGCCAAAGACCTTCTAAGTCGTCATACACTCATGAAGGCACGAAGCCCA

AATACGAAAGTAACTTTACAACTTAGACCCGACTCCACGAAAGCCAAGGAA

>1109_Sample_ASV38

CACCGCGGTTATACGAGAGACTCCAGTTGATAGCTACGGCGTAAAGAGTGGTTTGGGGCC

CCACCCAAAATAAAGCCAAAGACCTCCCAAGCCGTCAAACGCACCCCGGAGGCACGAAGT

CCTAACGCGAAAGCAACTTTACCTCCCCCGACGCCACGAAAGCTAAGAAA

>1109_Sample_ASV39

CACCGCGGTTATACGAGAGACTCCAGTTGATAGCTACGGCGTAAAGAGTGGTTTGGGGCC

CCACCCAAAATAAAGCCAAAGACCTCCCAAGCCGTCAAACGCACCCCGGAGGCACGAAGT

CCTAACGCGAAAGCAACTTTACCTCCCCCGACGCCACGAAAGCTGTGAAA

>1109_Sample_ASV40

CACCGCGGTTATACGAAAGACCCTAGTTGATAGCTACGGCGTAAAGGGTGGTTAAGGTAT

ATAACAAATAAAGCTAAAGAGCCTCCAAGCCGTCGTACGCATTCTGAGAGCTCGAAACCC

TAACACGAAAGTAGCTTTAAAAAATAACACCTGACCCCACGAAAGCTAAGAAA

>1109_Sample_ASV41

CACCGCGGTTATACGAGAGACCCTAATTGATAGACTACGGCGTAAAGAGTGGTTTAGAAC

AAAAATTAATAAAGCCAAAGATCCTCCTGGCCGTCGCACGCATTTCGAGGATACGAAGCC

CCACTACGAAAGTAGCTTTACTACAACTTTTCTGACCCCACGAAAGCTAAGAAA

>1109_Sample_ASV42

CACCGCGGTTATACGAAAGACCCTAGTTGATAGATCACGGCGTAAAGGGTGGTTAAGGAG

AACAAAAATAAAGCTAAAAATCTTCTAAGCTGTCATACGCTTTCCGAAGACATGAGACCC

AACCACGAAAGTAGCTTTAAACTTTCCTGACACCACGAAAGCCAAGAAA

>1110_Sample_ASV1

CACCGCGGTTATACGAAAGACCCTAGTTGATGGGTGCGGCGTAAAGGGTGGTTAGGGACT

ACAAAAAATAGAGCTAAAGATCCTCTAGGTCGTCATACACATCACGAGGAAACGAAGCCC

AAATACGAAAGTAGCTCTAAATATAATCCTGACGCCACGAAAGCCAAGAAA

>1110_Sample_ASV2

CACCGCGGTTATACGAGAGACCCTAGTTGATTAAAGCGGCGTAAAGAGTGGTTATGGAAC

TATTCTTTTAAAGCAGAAAACCTCTCAAACTGTTATACGCACCCAGAGGTCTGAATCCCT

CACACGAAAGTGACTTTATTTATGCCTACCAGAACCCACGAAAGCTGGGACA

>1110_Sample_ASV3

CACCGCGGTTATACGAGAGGCCCAAGTTGACAGACACCGGCGTAAAGAGTGGCTAGGGAA

AAATTACTACTAAAGCCGAACACCTTCAGAACTGTCATACGTTCCCGAAGATAAGAAGCC

CCACCACGAAAGTGACTTTATATTACCCGACCCCACGAAAGCTGTGAAA

>1110_Sample_ASV4

CACCGCGGTTATACGAGAGGCCCAAGTTGACAGACACCGGCGTAAAGAGTGGCTAGGGAA

AAATTACTACTAAAGCCGAACACCTTCAGAACTGTCATACGTTCCCGAAGATAAGAAGCC

CCACCACGAAAGTGACTTTATATTACCCGACCCCACGAAAGCTAAGAAA

>1110_Sample_ASV5

CACCGCGGTTATACGAGAGACCCTAGTTGACAGACACCGGCGTAAAGAGTGGCTAGGGAA

AAATTACTACTAAAGCCGAACACCTTCAGAACTGTCATACGTTCCCGAAGATAAGAAGCC

CCACCACGAAAGTGACTTTATATTACCCGACCCCACGAAAGCTAAGAAA

>1110_Sample_ASV6

CACCGCGGTTATACGAGAGACCCTAGTTGACAGACACCGGCGTAAAGAGTGGCTAGGGAA

AAATTACTACTAAAGCCGAACACCTTCAGAACTGTCATACGTTCCCGAAGATAAGAAGCC

CCACCACGAAAGTGACTTTATATTACCCGACCCCACGAAAGCTGTGAAA

>1110_Sample_ASV7

CACCGCGGTTATACGAGAGGCCCAAGTTGACAGACACCGGCGTAAAGAGTGGCTAGGGAA

AAATTACTACTAAAGCCGAACACCCTCAGAACTGTCATACGTTCCCGAAGGTAAGAAGCC

CCACTACGAAAGTGGCTTTATAATACCCGACCCCACGAAAGCTGTGAAA

>1110_Sample_ASV8

CACCGCGGTTATACGAGAGGCCCAAGTTGACAGACACCGGCGTAAAGAGTGGCTAGGGAA

AAATTACTACTAAAGCCGAACACCTTCAGAACTGTCATACGTTCCCGAAGATAAGAAGCC

CCACCACGAAAGTGGCTTTATATTACCTGACCCCACGAAAGCTAAGAAA

>1110_Sample_ASV9

CACCGCGGTTATACGAGAGGCCCAAGTTGACAGACACCGGCGTAAAGAGTGGCTAGGGAA

AAATTACTACTAAAGCCGAACACCTTCAGAACTGTCATACGTTCCCGAAGATAAGAAGCC

CCACCACGAAAGTGGCTTTATATTACCCGACCCCACGAAAGCTGTGAAA

>1110_Sample_ASV10

CACCGCGGTTATACGAGAGGCCCAAGTTGACAGACACCGGCGTAAAGAGTGGCTAGGGAA

AAATTACTACTAAAGCCGAACACCCTCAGAACTGTCATACGTTCCCGAAGGTAAGAAGCC

CCACCACGAAAGTGGCTTTATAATACCCGACCCCACGAAAGCTAAGAAA

>1110_Sample_ASV11

CACCGCGGTTATACGAGAGGCCCAAGTTGACAGACACCGGCGTAAAGAGTGGCTAGGGAA

AAATTACTACTAAAGCCGAACACCCTCAGAACTGTCATACGTTCCCGAAGGTAAGAAGCC

CCACCACGAAAGTGGCTTTATAATACCCGACCCCACGAAAGCTGTGAAA

>1110_Sample_ASV12

CACCGCGGTTATACGAGAGGCCCAAGTTGACAGACACCGGCGTAAAGAGTGGCTAAGGAA

AAATTTCTACTAAAGCCGAACACCTTCAGAACTGTCATACGTTCCCGAAGAAAAGAAGCC

CCACCACGAAAGTGGCTTTATATTACCCGACCCCACGAAAGCTGTGAAA

>1110_Sample_ASV13

CACCGCGGTTATACGAGAGGCCCAAGTTGACAGACACCGGCGTAAAGAGTGGCTAAGGAA

AAATTTCTACTAAAGCCGAACACCTTCAGAACTGTCATACGTTCCCGAAGATAAGAAGCC

CCACCACGAAAGTGGCTTTATATTACCCGACACCACGAAAGCTAAGAAA

>1110_Sample_ASV14

CACCGCGGTTATACGAGAGGCCCAAGTTGACAGACACCGGCGTAAAGAGTGGCTAAGGAA

AAATTTCTACTAAAGCCGAACACCTTCAGAACTGTCATACGTTCCCGAAGATAAGAAGCC

CCACCACGAAAGTGGCTTTATATTACCCGACACCACGAAAGCTGTGAAA

>1110_Sample_ASV15

CACCGCGGTTAAACGAGAGACCCTAGTTGATCATCACGGCGTAAAGAGTGGTTAGGGGAT

TATCATAAATAAAGCCAAAGACCTCCCAAGCTGTCGCACGCATTCCGGAGGCGCGAAGCC

CACACACGAAAGTAGCTTTAACTATTGACCCTGATGCCACGAAAGCTAAGAAA

>1110_Sample_ASV16

CACCGCGGTTATACGAAAGACCCTAGTTGATAGATCACGGCGTAAAGGGTGGTTAGGGAA

TACAAAAATAAAGCTAAAAATCCTCTAAGCTGTCATACGCTTCCCGAAGACATGAAACCC

AACCACGAAAGTAGCTTTAAACTCTCCTGACACCACGAAAGCCAAGGAA

>1110_Sample_ASV17

CACCGCGGTTATACGAGAGGCCCAAGTTGATAAGCCCGGCGTAAAGAGTGGTTATGGAGA

ATAAAACACTAAAGCTAAAGACCCCCTAGGCTGTTATACGCACCTGGCGGCTCGAACCAC

CTATACGAAAGTAGCTTTACCCCCCTTCCACCAGAATCCACGACAGCTGGGGCA

>1110_Sample_ASV18

CACCGCGGTTATACGAGAGGCCCAAGTTGACAGACATCGGCGTAAAGAGTGGTTAAGGAA

ACCCCATTACTAAAGCCGAACGCCCTCAGAACTGTCATACGTTCCCGAAGGTAAGAAGCC

CCACTACGAAAGTGGCTTTAACCACACCCGACCCCACGAAAGCTAAGAAA

>1110_Sample_ASV19

CACCGCGGTTATACGAGAGGCCCAAGTTGACAGACATCGGCGTAAAGAGTGGTTAAGGAA

ACCCCATTACTAAAGCCGAACGCCCTCAGAACTGTCATACGTTCCCGAAGGTAAGAAGCC

CCACTACGAAAGTGGCTTTAACCACACCCGACCCCACGAAAGCTGTGAAA

>1110_Sample_ASV20

CACCGCGGTTATACGAGAGACCCTAGTTGATATATACGGCGTAAAGAGTGGTTAAGGAAA

CCCCATTACTAAAGCCGAACGCCCTCAGAACTGTCATACGTTCCCGAAGGTAAGAAGCCC

CACTACGAAAGTGGCTTTAACCACACCCGACCCCACGAAAGCTGTGAAA

>1110_Sample_ASV21

CACCGCGGTTAAACGAGAGGCCCTAGTTGATATTACAACGGCGTAAAGGGTGGTTAAGGA

AAGCAAGCAATAAAGCCGAATGGCCCTTTGGCTGTCATACGCTTCTAGGTGTCCGAAGCC

CAATATACGAAAGTAGCTTTAGAAAAGCCCACCTGACACCACGAAAGCTGAGGAA

>1110_Sample_ASV22

CACCGCGGTTATACGAGAGGCCCCAGTTGATATTAGCGGCGTAAAGGGTGGTTAAGGACT

TTAATAACTAAAGCTAAACACTTCCCCGGCTGTTGTACGTAGACCGGAAGCTTGAAGCCC

AGACACGAAAGTAGCTTTATTCCCCCTGACCCCACGAAAGCTGAGAAA

>1110_Sample_ASV23

CACCGCGGTTATACGAGAGGCCCTAGTTGATAAATACGGCGTAAAGAGTGGTTAGAGGAC

TCCCATAATAAAGCCAAAGACCCTCCGGGCCGTCACACGCATACGAGGGCACGAAGTTCA

CACACGAAAGTAGCTTTAAATTACCCGACGCCACGAAAGCTAAGAAA

>1110_Sample_ASV24

CACCGCGGTTATACGAAAGACCCTAGTTTATAAGTACGGCGTAAAGGGTGGTTAGGGGAC

AAACAAAATAAAGCCAAAGACCTCCTAAGCCGTCATACGCTTACGGAGGCACGAAGCCCA

AACACGAAAGTAGCTTTACCGAACATGCCCGACTCCACGAAAGCTGGGAAA

>1110_Sample_ASV25

CACCGCGGTTATACGAGAGACCCTAGTTGATATATACGGCGTAAAGAGTGGTTATGGGAA

AAACAAATAAAGTCAAACAACCTCTTAGCTGTTATACGCATTATGAGAGCATGAAGCCCC

CTCACGAAAGTAACTTTAATACCTCCTGACCCCACGAAAGCTAAGAAA

>1110_Sample_ASV26

CACCGCGGTTATACGAGAGACCCTAGTTGATAAATACGGCGTAAAGAGTGGTTATGGGAA

AAACAAATAAAGTCAAACAACCTCTTAGCTGTTATACGCATTATGAGAGCATGAAGCCCC

CTCACGAAAGTAACTTTAATACCTCCTGACCCCACGAAAGCTAAGAAA

>1110_Sample_ASV27

CACCGCGGTTATACGAGAGGCTCAAGTTGATAAACCCCGGCGTAAAGAGTGGTTAAGGAA

AAATAGAAACTAAAGCCGAATGCTTTCAAGACTGTTATACGTTCCCGAGAGTAAGAAGAC

CAATTACGAAAGTGGCTTTACTTTACCTGACCCCACGAAAGCTACGACA

>1110_Sample_ASV28

AACCGCGGTTATACGAGAGACCCTAATTGATAGCTCACGGCGTAAAGGGTGGTTTGGGGA

AACCTCAAAATAAAGCTAAAGACCTTCTAAGCCGTTACACGCATACCGAAGGCACGAGGC

CCTAACACGAAAGTAGCTTTACTATTACCCCTGACGCCACGAAAGCTAAGAAA

>1110_Sample_ASV29

CACCGCGGTTATACGAAAGACCCTAGTTGATGGATCACGGCGTAAAGGGTGGTTAAGGGA

TATAAAAATAAAGCTAAAAATCCTCTTAGCTGTCATACGCTTTCCGAAGACATGAGGCCC

AATTACGAAAGTAGCTTTAAACCACCCTGACACCACGAAAGCCAAGAAA

>1110_Sample_ASV30

CACCGCGGTTAAACGAGAGGCCCTAGTTGATAGTACAACGGCGTAAAGGGTGGTTAAGGA

TAGTGAAATAATAAAGTCGAATGGCCCTTTGGCTGTCATACGCTTCTAGGAGTCCGAAGC

CCAATATACGAAAGTAACTTTAAGAGCGCCCACCTGACCCCACGAAAACTGAGAAA

>1110_Sample_ASV31

CACCGCGGTTATACGAGAGGCCCAAGTTGATACTCCACGGCGTAAAGAGTGGTTAGAAAA

GACCTACTACTAAAGCCGAACGCCTTCAAAGCTGTTATACGCATCCGAAGGTGAGAAGCC

CACCCACGAAAGTGGCTTTACAACCTTGACTCCACGAAAGCTATGACA

>1110_Sample_ASV32

CACCGCGGTTATACGAGAGACCCCAGTTGATAAACACGGCGTAAAGAGTGGTTAAGGGCA

CAAAACAAATAAAGTCAAACAACCTCTTAGCTGTTATACGCATTATGAGGATATGAAGCC

CCCCTACGAAAGTAACTTTACCAACCCCTGACCCCACGAAAGCTAAGAAA

>1110_Sample_ASV33

CACCGCGGTTATACGAGAGACCCTAGTTGATATATACGGCGTAAAGAGTGGTTTGGAATA

ACCAAGTAATATAGCCAAAGACCTCCCAAGCCGTCACACGCACCCCGGAGGCACGAAGCC

CAAACACGAAAGTAGCTTTATTAACTTACCGACGCCACGAAAGCTAAGAAA

>1110_Sample_ASV34

CACCGCGGTTATACGAAAGACCCTAGTTTATAGGCACGGCGTAAAGGGTGGTTAGGGGAT

AAACAAAATAAAGCCAAAGACCTTCTAAGTCGTCATACACTCATGAAGGCACGAAGCCCA

AATACGAAAGTAACTTTACAACTTAGACCCGACTCCACGAAAGCTGGGGGA

>1110_Sample_ASV35

CACCGCGGTTATACGAGAGACCCTAGTTGATATATACGGCGTAAAGAGTGGTTTGGGGCC

CCACCCAAAATAAAGCCAAAGACCTCCCAAGCCGTCAAACGCACCCCGGAGGCACGAAGT

CCTAACGCGAAAGCAACTTTACCTCCCCCGACGCCACGAAAGCTAAGAAA

>1110_Sample_ASV36

CACCGCGGTTATACGAGAGACCCTAGTTGATAGCTACGGCGTAAAGAGTGGTTTGGGGCC

CCACCCAAAATAAAGCCAAAGACCTCCCAAGCCGTCAAACGCACCCCGGAGGCACGAAGT

CCTAACGCGAAAGCAACTTTACCTCCCCCGACGCCACGAAAGCTAAGAAA

>1110_Sample_ASV37

CACCGCGGTTATACGAGAGACTCCAGTTGATAGCTACGGCGTAAAGAGTGGTTTGGGGCC

CCACCCAAAATAAAGCCAAAGACCTCCCAAGCCGTCAAACGCACCCCGGAGGCACGAAGT

CCTAACGCGAAAGCAACTTTACCTCCCCCGACGCCACGAAAGCTAAGAAA

>1110_Sample_ASV38

CACCGCGGTTATACGAGAGACCCTAATTAATAGATACGGCGTAAAGAGTGGTTTAGGACA

AAAATTAATAAAGCCGAAGTTTCTCCTAGCCGTTGTACGCACTCTGAGAACACGAAGCCC

CACTACGAAAGTAGCTTTACCTATTATTTCCTGACCCCACGAAAGCTAAGAAA

>1110_Sample_ASV39

CACCGCGGTTATACGAGAGGCCCAAGTTGATAGACATCGGCGTAAAGAGTGGTTAAGGAA

GTCTCAAAACTAAAGCCGAATGCCTTCAAGACTGTTATACGTTTCCGAAGGTAAGAAGCC

CCACTACGAAAGTGGCTTTACTCCACCTGACTCCACGAAAGCTGTGAAA

>1110_Sample_ASV40

CACCGCGGTTATACGAGAGGCCCAAGTTGACAAACTCCGGCGTAAAGCGTGGTTAAGGCA

CACTCAAAAACTAAAGCCGAATATCTTCAGAGCAGTCATACGCATCCGAAGACACGAAGC

CCCACCACGAAAGTGGCTTTATGACACCTGACCCCACGAAAGCTAGGACA

>1110_Sample_ASV41

CACCGCGGTTATACGAAAGACCCTAGTTGATAGACCACGGCGTAAAGGGTGGTTAAGGAA

CACAAAAATAAAGCTAAAGATCCTCTAAGCTGTCATACGCTTCCCGAAGACATGAAACCC

AATCACGAAAGTAGCTTTAAACTGTCCTGACACCACGAAAGCCAAGAAA
